# Supplementary material for: Experimental evaluation of DPF performance loaded over Pt and sulfur-resisting material for marine diesel engines
Source: PLoS One. 2022 Sep 22;17(9):e0272441. doi: 10.1371/journal.pone.0272441 (PMC9499249; doi:10.1371/journal.pone.0272441)

S1 Fig 1. DPF catalyst module. (A) individual catalyst, (B) Packaged catalyst.

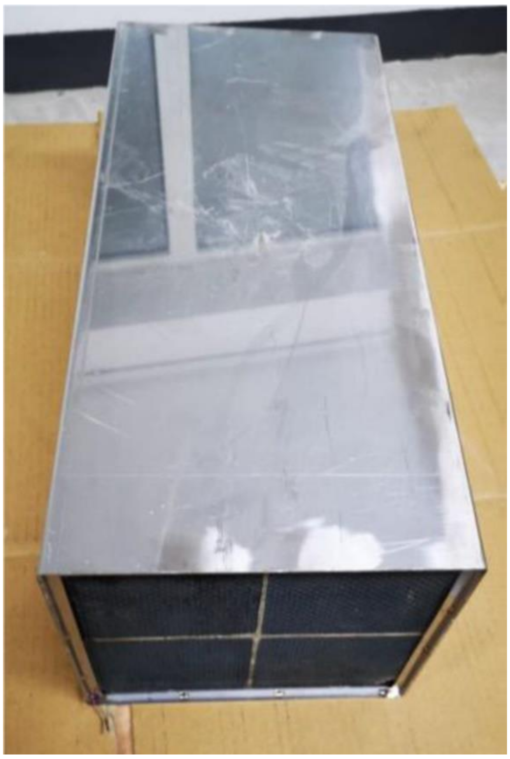

（A）

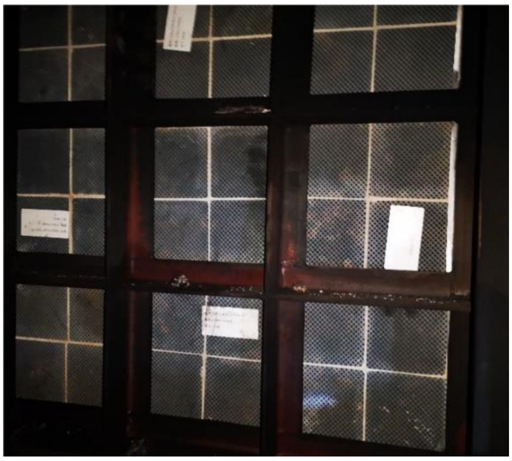

（B）

S1 Table 1. Coating materials and Pt loadings of DPF module

No.	Carriers	Coating	PGM
1	SiC	None	Pt 10g/cft
2	SiC	SR-1	Pt 10g/cft
3	SiC	SR-1	Pt 40g/cft

S1 Fig 2. The simulated-exhaust-gas experiments bench.

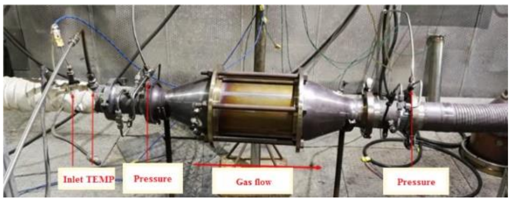

S1 Fig 3. The pilot-scale tests bench. (A)Diesel engine, (B) Bench schematic figure.

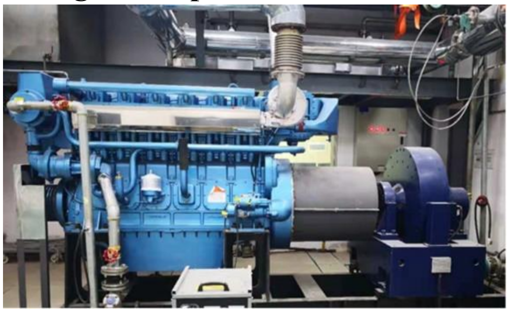

（A）

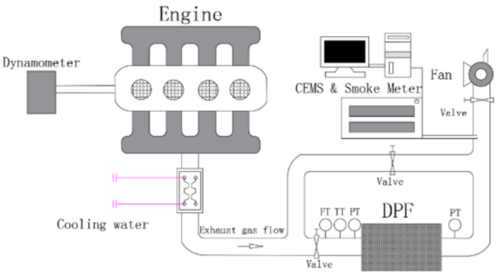

（B）

S1 Fig 4. BPT results for DPF with different coating and loading contents. (A) DPF 1#, (B) DPF 2#, (C) DPF 3#.

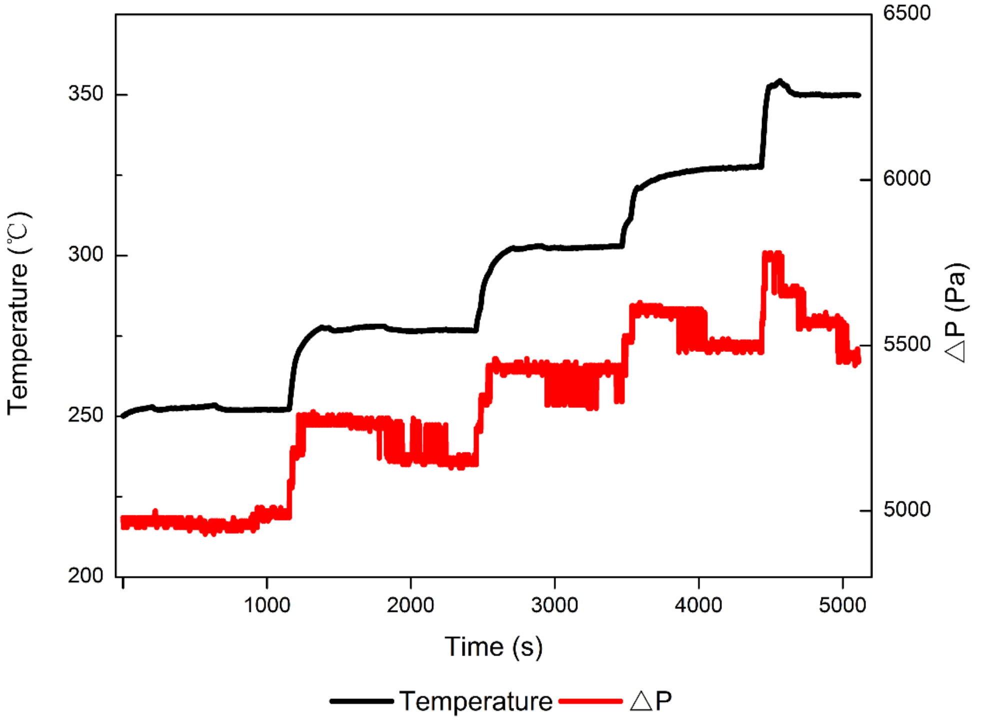

（A）

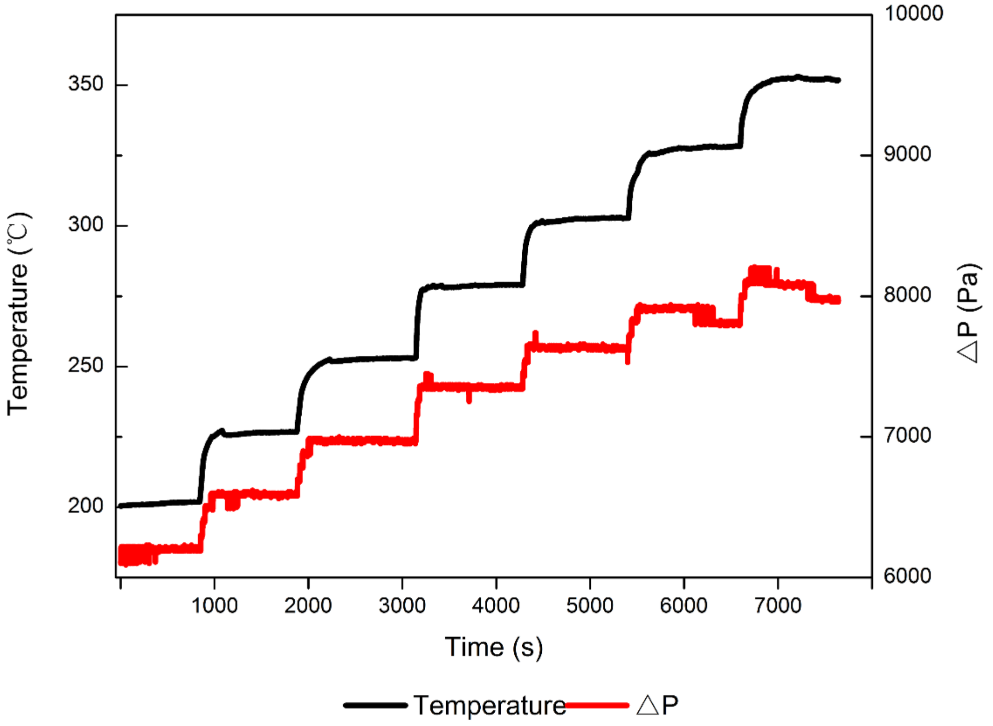

（B）

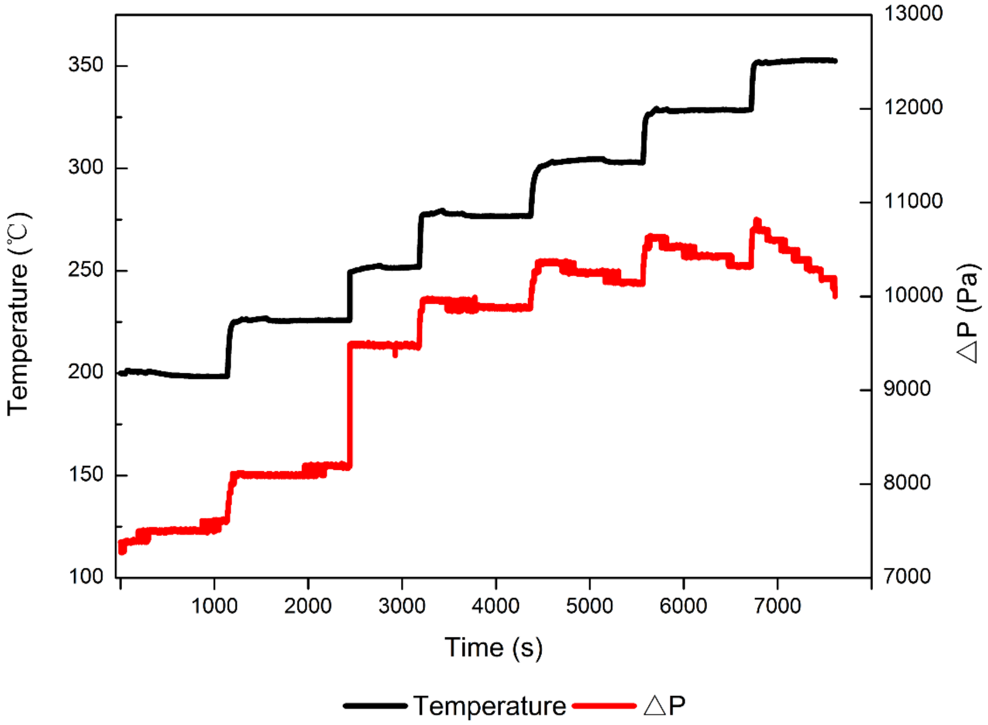
Temperature (℃)
350
300
250
200
150
100
△P (Pa)
13000
12000
11000
10000
9000
8000
7000
1000
2000
3000
4000
5000
6000
7000
Time (s)
Temperature
△P

（C）

S1 Fig 5. PMs deposition and residuals in DPF with different coating and loading contents. (A) DPF 1#, (B) DPF 2#, (C) DPF 3#.

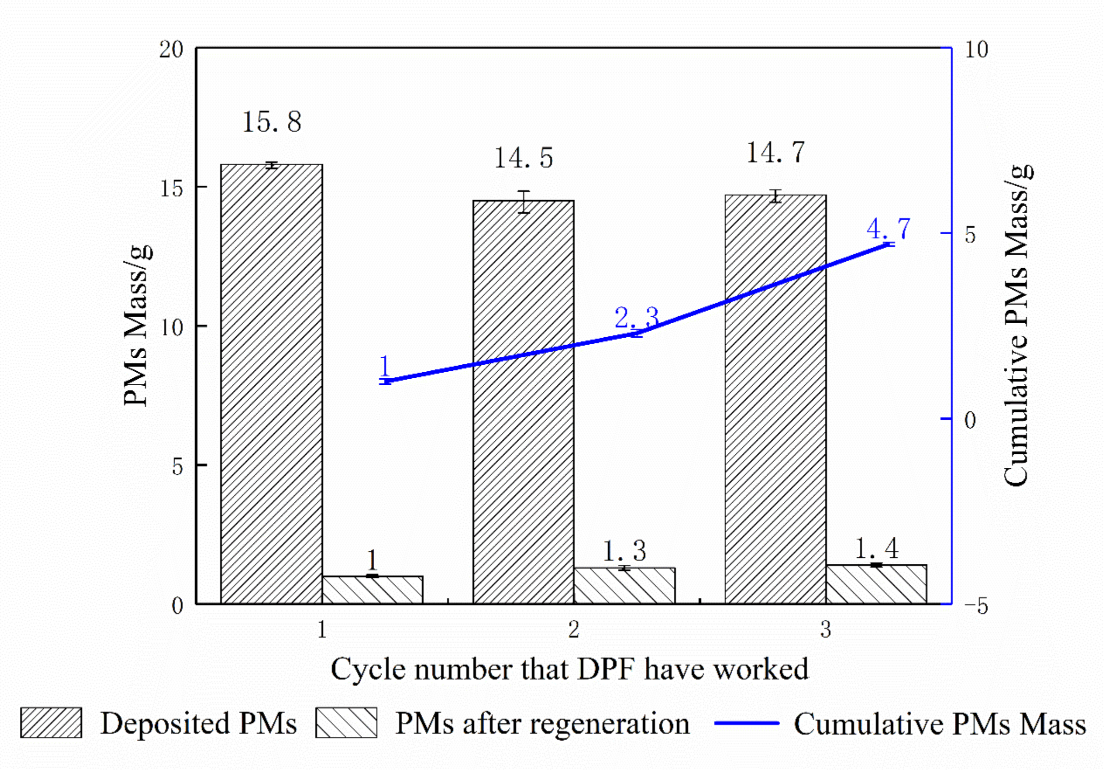

（A）

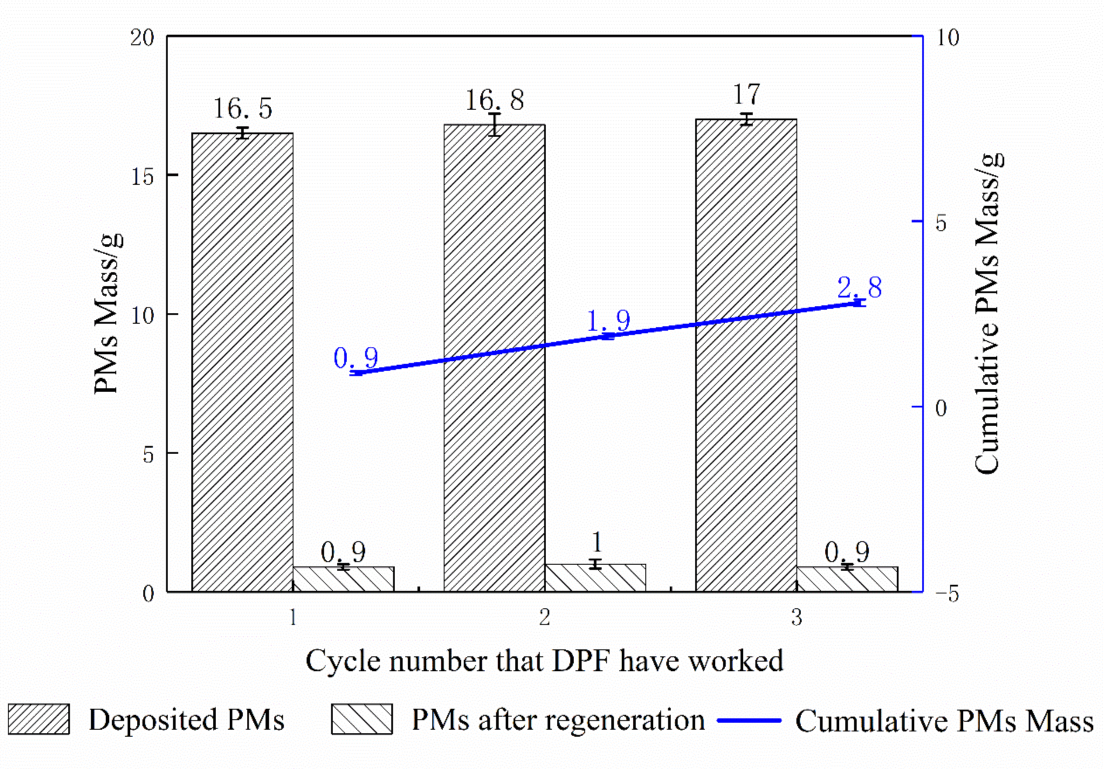

（B）

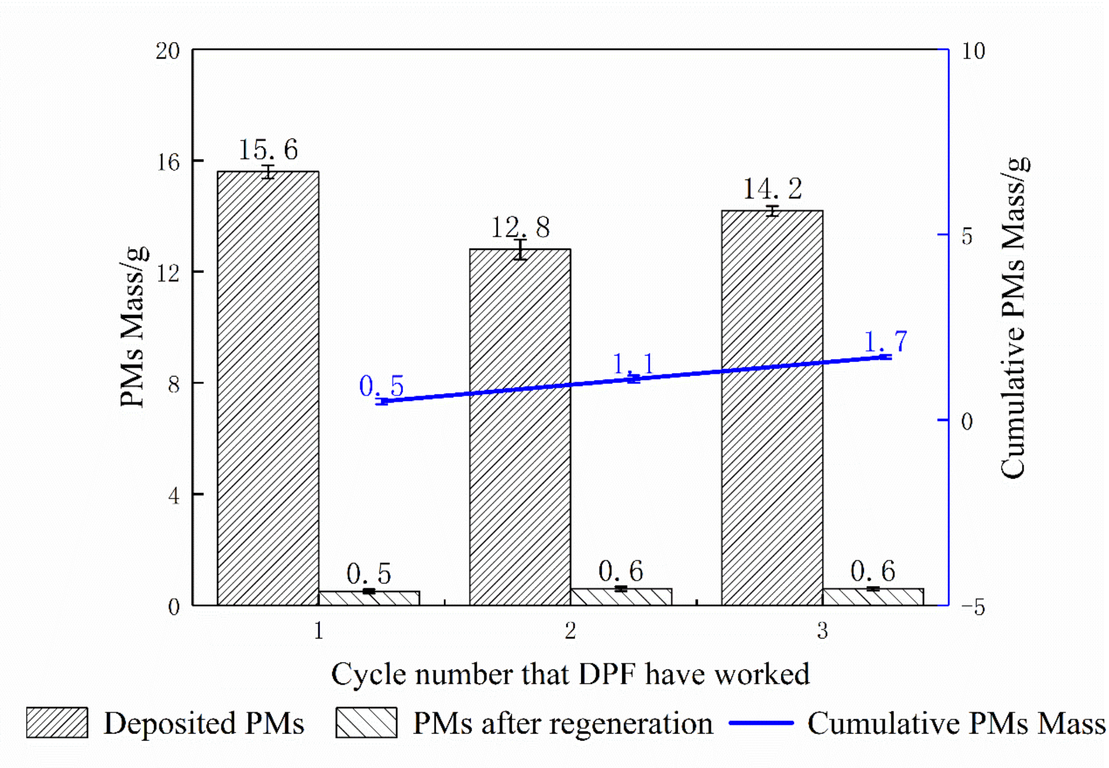
PMs Mass/g
Cumulative PMs Mass/g
Cycle number that DPF have worked
15. 6
12. 8
14. 2
0. 5
0. 6
0. 6
0. 5
1. 1
1. 7
Deposited PMs
PMs after regeneration
Cumulative PMs Mass

（C）

S1 Table 2. Upstream and downstream PMs emissions of unit power (g/kW-h) and PMs filtering efficiency (%) of DPF at different diesel engine loads.

No.	Load	PMs emissions of unit power (g/kW-h)		Filtering efficiency (%)
		Upstream	downstream	
1	10%	1.2370	0.2180	82.4%
2	25%	1.1148	0.2428	78.2%
3	50%	0.7637	0.1878	75.4%
4	65%	0.6417	0.1713	73.3%
5	75%	0.5683	0.1866	67.2%
6	100%	0.4320	0.2765	36.0%

S1 Table 3. Upstream and downstream smoke level (mg/m³) and filtering efficiency (%) of DPF module under different loads.

No.	Load	Smoke level (g/kW-h)		Filtering efficiency (%)
		Upstream	downstream	
1	65%	53.11	0.17	99.7%
2	75%	59.33	0.23	99.6%
3	100%	56.66	3.29	94.2%

S1 Fig 6. DPF BPT tests in pilot-scale bench.

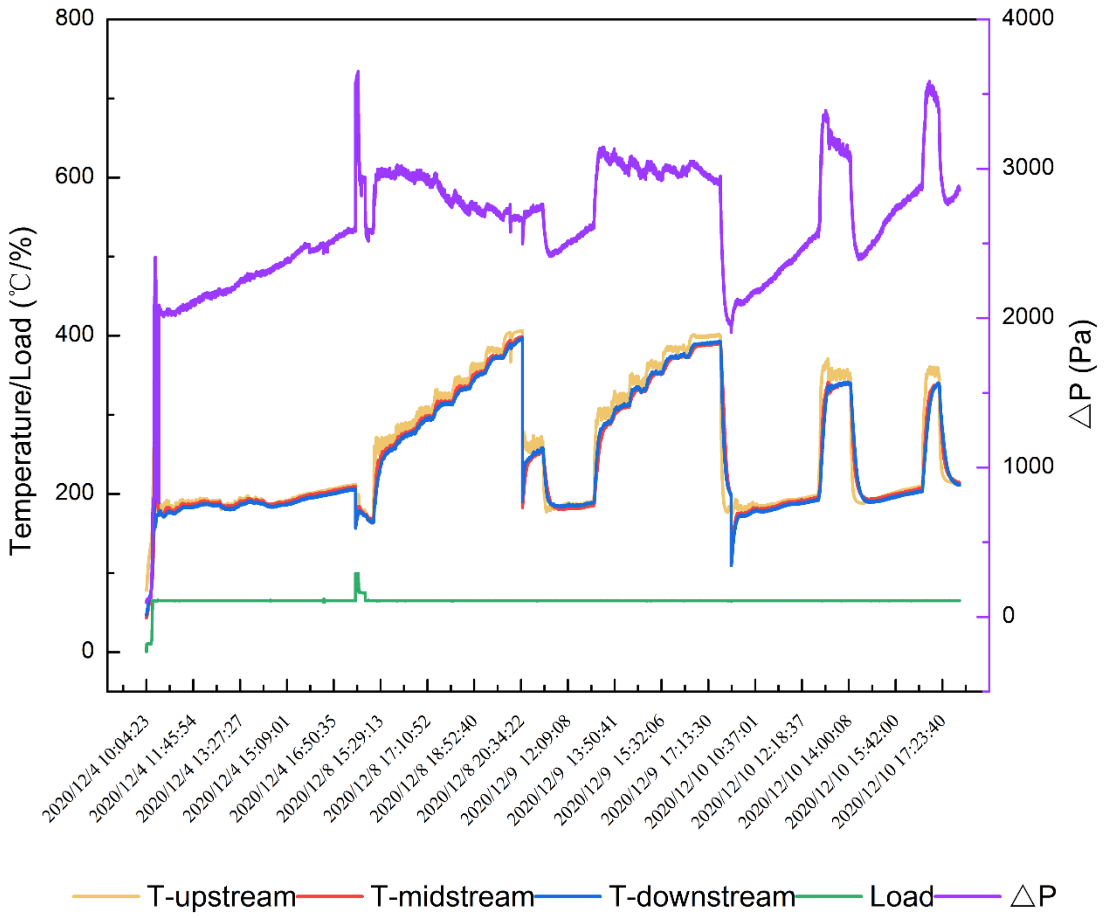

S1 Fig 7. Long-term DPF module regeneration durability test.

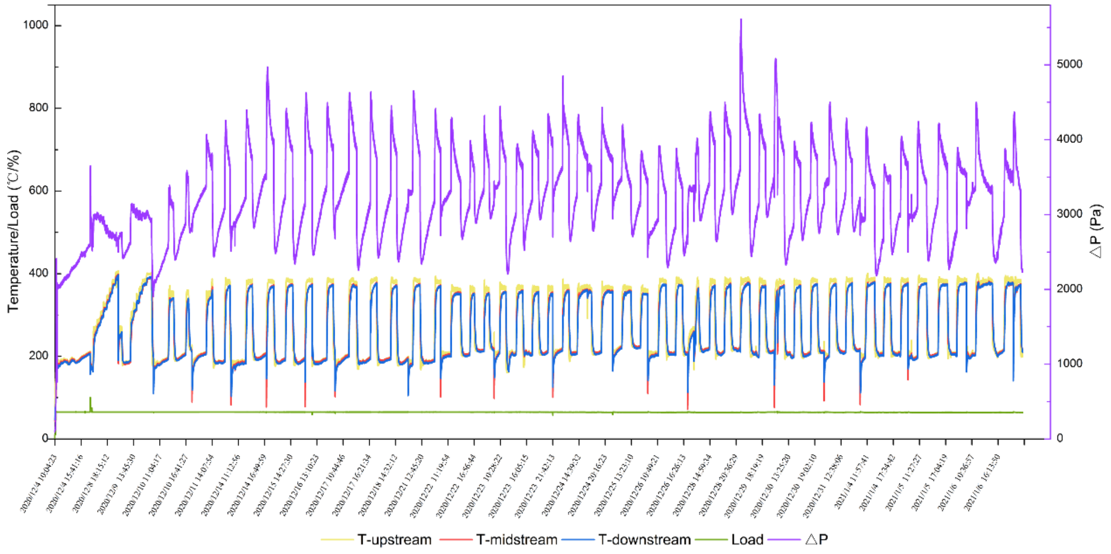

S1 Fig 8. DPF module. (A) Upstream; (B) Inside; (C) Downstream.

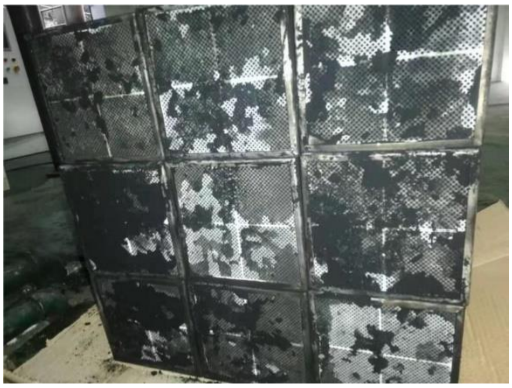

（A）

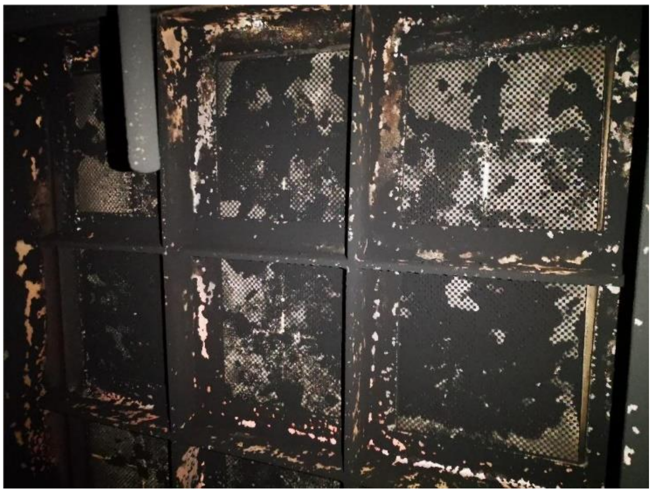

（B）

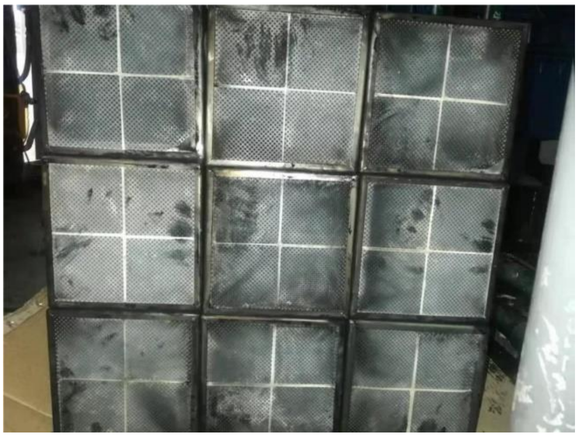

（C）

S1 Fig 9. Upstream and downstream gaseous emissions of DPF.

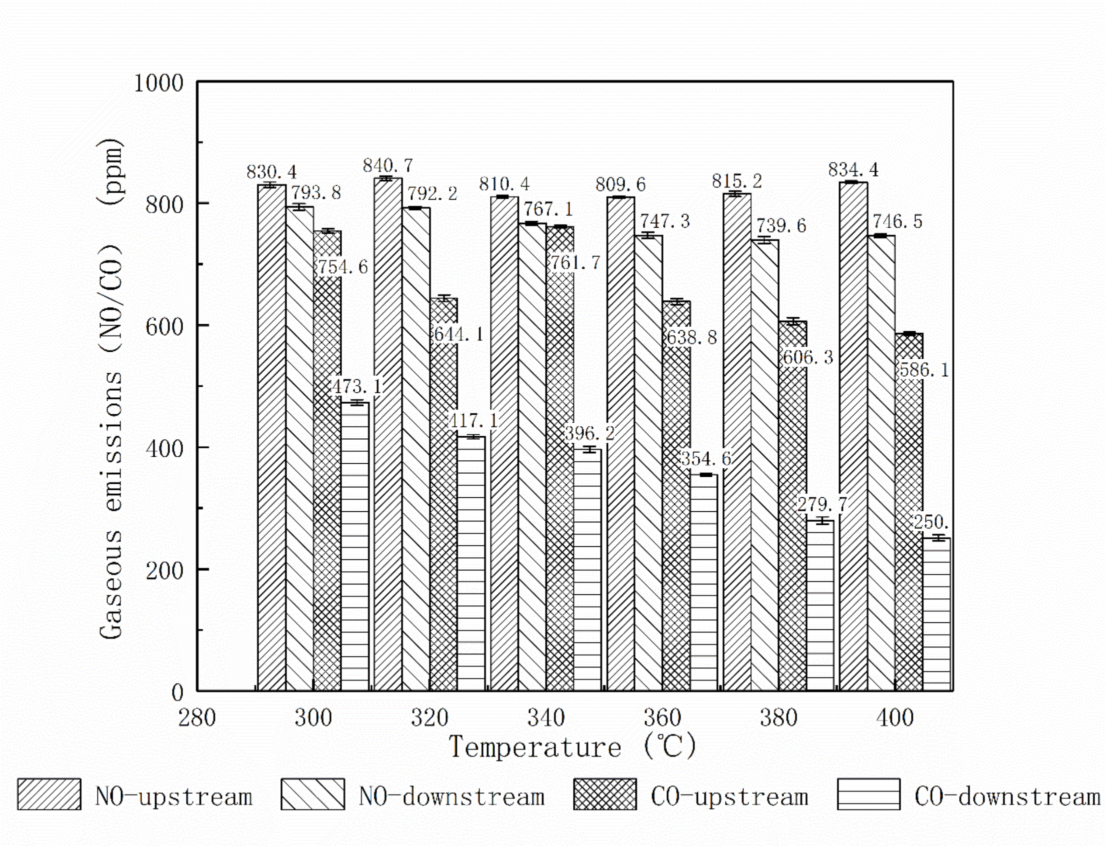

Supplement: S1 File — (PDF) [file pone.0272441.s001.pdf]
