# Supplementary material for: Experimental evaluation of DPF performance loaded over Pt and sulfur-resisting material for marine diesel engines
Source: PLoS One. 2022 Sep 22;17(9):e0272441. doi: 10.1371/journal.pone.0272441 (PMC9499249; doi:10.1371/journal.pone.0272441)
Supplement: S1 Data — (PDF) [file pone.0272441.s002.pdf]

Data: Fig 4. BPT results for DPF with different coating and loading contents. (A) DPF 1#, (B) DPF 2#, (C) DPF 3#.								
DPF1			DPF2			DPF3		
Time(s)	Temp.(℃)	△P(Pa)	Time(s)	Temp.(℃)	△P(Pa)	Time(s)	Temp.(℃)	△P(Pa)
1	250.2	4980	1	200.4	6110	1	200	7380
2	250.2	4980	2	200.4	6100	2	200	7380
3	250.1	4950	3	200.5	6210	3	200	7380
4	250.2	4970	4	200.5	6210	4	200.1	7380
5	250.2	4960	5	200.4	6200	5	200.1	7280
6	250.2	4960	6	200.4	6210	6	200.1	7370
7	250.2	4970	7	200.4	6190	7	200.1	7270
8	250.3	4970	8	200.4	6200	8	200	7380
9	250.3	4970	9	200.4	6220	9	200	7370
10	250.3	4970	10	200.4	6210	10	199.9	7360
11	250.3	4980	11	200.4	6210	11	199.9	7370
12	250.4	4980	12	200.4	6200	12	199.8	7270
13	250.5	4980	13	200.4	6210	13	199.8	7380
14	250.5	4970	14	200.5	6210	14	199.8	7280
15	250.6	4980	15	200.5	6110	15	199.8	7270
16	250.6	4980	16	200.5	6210	16	199.9	7370
17	250.6	4970	17	200.5	6110	17	200	7380
18	250.7	4960	18	200.5	6100	18	200	7370
19	250.7	4970	19	200.5	6220	19	200	7270
20	250.7	4970	20	200.5	6120	20	200.1	7370
21	250.8	4980	21	200.5	6220	21	200.1	7280
22	250.8	4980	22	200.5	6120	22	200.1	7280
23	250.8	4970	23	200.5	6110	23	200.1	7380
24	250.8	4980	24	200.6	6220	24	200.1	7380
25	250.8	4970	25	200.6	6200	25	200.1	7380
26	250.8	4970	26	200.5	6210	26	200	7380
27	250.8	4950	27	200.6	6120	27	200	7370
28	250.9	4970	28	200.6	6200	28	200.1	7370
29	250.9	4960	29	200.6	6220	29	200.1	7370
30	250.9	4970	30	200.6	6210	30	200.2	7380
31	250.9	4970	31	200.5	6210	31	200.2	7370
32	250.9	4970	32	200.6	6110	32	200.2	7370
33	251	4960	33	200.6	6200	33	200.2	7380
34	251	4970	34	200.6	6220	34	200.2	7380
35	251	4960	35	200.6	6210	35	200.2	7370
36	251	4970	36	200.6	6200	36	200.2	7380
37	251	4970	37	200.6	6100	37	200.1	7280
38	251	4960	38	200.6	6190	38	200.1	7390
39	251.1	4970	39	200.6	6210	39	200.1	7390
40	251.1	4970	40	200.6	6200	40	200	7390
41	251.1	4970	41	200.6	6210	41	200	7380
42	251.1	4960	42	200.6	6210	42	200	7390
43	251.2	4980	43	200.6	6210	43	199.9	7370
44	251.3	4980	44	200.6	6210	44	199.9	7380
45	251.3	4980	45	200.6	6210	45	199.8	7380
46	251.3	4970	46	200.6	6200	46	199.7	7390

47	251.3	4970	47	200.6	6210	47	199.7	7390
48	251.4	4970	48	200.6	6200	48	199.6	7390
49	251.4	4980	49	200.6	6210	49	199.6	7390
50	251.3	4980	50	200.6	6210	50	199.7	7370
51	251.4	4970	51	200.6	6200	51	199.7	7380
52	251.4	4960	52	200.6	6210	52	199.8	7380
53	251.4	4960	53	200.6	6210	53	199.9	7370
54	251.5	4960	54	200.6	6200	54	200	7370
55	251.5	4960	55	200.6	6210	55	200.2	7390
56	251.5	4960	56	200.6	6110	56	200.3	7380
57	251.5	4960	57	200.6	6090	57	200.4	7380
58	251.5	4970	58	200.6	6210	58	200.4	7390
59	251.4	4970	59	200.6	6220	59	200.5	7400
60	251.4	4970	60	200.6	6220	60	200.6	7380
61	251.5	4980	61	200.6	6120	61	200.6	7380
62	251.5	4970	62	200.6	6110	62	200.7	7390
63	251.5	4970	63	200.6	6220	63	200.7	7380
64	251.5	4970	64	200.6	6220	64	200.8	7380
65	251.6	4960	65	200.6	6120	65	200.9	7380
66	251.6	4970	66	200.6	6220	66	201	7380
67	251.6	4970	67	200.6	6110	67	201.1	7380
68	251.6	4970	68	200.6	6110	68	201.2	7370
69	251.6	4980	69	200.6	6210	69	201.2	7380
70	251.7	4970	70	200.6	6210	70	201.3	7390
71	251.7	4980	71	200.6	6100	71	201.4	7380
72	251.7	4980	72	200.6	6210	72	201.4	7370
73	251.7	4960	73	200.7	6220	73	201.5	7390
74	251.7	4970	74	200.7	6220	74	201.5	7390
75	251.7	4970	75	200.7	6200	75	201.5	7380
76	251.7	4960	76	200.7	6120	76	201.5	7400
77	251.7	4970	77	200.7	6220	77	201.5	7390
78	251.8	4980	78	200.7	6220	78	201.5	7370
79	251.8	4970	79	200.7	6220	79	201.5	7390
80	251.9	4970	80	200.7	6100	80	201.5	7380
81	251.8	4960	81	200.7	6110	81	201.4	7390
82	251.9	4970	82	200.7	6210	82	201.5	7380
83	251.9	4970	83	200.7	6210	83	201.4	7390
84	251.9	4960	84	200.7	6120	84	201.4	7390
85	251.9	4970	85	200.7	6220	85	201.4	7390
86	251.9	4970	86	200.7	6220	86	201.4	7380
87	252	4960	87	200.7	6210	87	201.4	7390
88	251.9	4960	88	200.7	6210	88	201.4	7380
89	252	4970	89	200.7	6130	89	201.3	7380
90	252	4970	90	200.7	6200	90	201.3	7380
91	252	4970	91	200.7	6210	91	201.2	7390
92	252	4970	92	200.7	6210	92	201.2	7390
93	252	4960	93	200.7	6220	93	201.2	7380
94	252	4950	94	200.7	6210	94	201.1	7380
95	252	4970	95	200.7	6210	95	201.1	7380
96	252	4960	96	200.7	6220	96	201.1	7380

97	252	4960	97	200.7	6100	97	201.1	7380
98	252	4970	98	200.8	6220	98	201.1	7390
99	252	4970	99	200.7	6220	99	201	7380
100	252	4970	100	200.7	6210	100	201	7390
101	252	4970	101	200.7	6220	101	201	7390
102	252	4960	102	200.7	6210	102	200.9	7380
103	252.1	4970	103	200.7	6100	103	200.9	7390
104	252	4980	104	200.7	6110	104	200.8	7390
105	252.1	4980	105	200.7	6200	105	200.8	7380
106	252.1	4970	106	200.7	6220	106	200.7	7370
107	252.1	4980	107	200.7	6200	107	200.8	7370
108	252.1	4980	108	200.7	6200	108	200.8	7390
109	252.1	4980	109	200.7	6210	109	200.9	7390
110	252.1	4970	110	200.7	6210	110	200.9	7370
111	252.1	4960	111	200.7	6120	111	201	7380
112	252.2	4950	112	200.7	6120	112	201	7370
113	252.2	4970	113	200.7	6100	113	201	7380
114	252.2	4960	114	200.7	6110	114	201	7380
115	252.2	4970	115	200.8	6200	115	201	7390
116	252.2	4960	116	200.7	6210	116	201	7390
117	252.2	4970	117	200.8	6210	117	201	7400
118	252.2	4960	118	200.8	6200	118	201	7380
119	252.2	4970	119	200.8	6200	119	201	7390
120	252.2	4970	120	200.8	6110	120	201	7390
121	252.3	4980	121	200.8	6220	121	201	7390
122	252.3	4970	122	200.8	6110	122	201	7390
123	252.3	4970	123	200.8	6210	123	201	7380
124	252.2	4980	124	200.8	6210	124	200.9	7380
125	252.2	4970	125	200.8	6200	125	200.9	7400
126	252.2	4970	126	200.8	6200	126	200.9	7380
127	252.3	4960	127	200.8	6200	127	200.9	7380
128	252.3	4980	128	200.9	6200	128	200.9	7400
129	252.2	4960	129	200.8	6100	129	200.8	7390
130	252.3	4970	130	200.9	6220	130	200.8	7390
131	252.3	4960	131	200.9	6210	131	200.7	7380
132	252.3	4970	132	200.9	6210	132	200.7	7400
133	252.3	4970	133	200.9	6220	133	200.7	7390
134	252.3	4970	134	200.9	6210	134	200.7	7390
135	252.3	4970	135	200.9	6210	135	200.7	7400
136	252.3	4960	136	200.9	6210	136	200.6	7400
137	252.3	4970	137	200.9	6210	137	200.6	7400
138	252.3	4980	138	200.9	6210	138	200.6	7410
139	252.3	4970	139	200.9	6220	139	200.6	7380
140	252.3	4980	140	200.9	6210	140	200.6	7380
141	252.4	4970	141	200.9	6220	141	200.6	7380
142	252.4	4980	142	200.9	6110	142	200.7	7380
143	252.4	4970	143	200.9	6100	143	200.7	7390
144	252.4	4970	144	200.9	6210	144	200.8	7380
145	252.4	4970	145	200.9	6210	145	200.8	7390
146	252.4	4980	146	200.9	6220	146	200.9	7390

147	252.4	4970	147	200.8	6220	147	200.9	7390
148	252.4	4950	148	200.9	6120	148	200.9	7390
149	252.5	4970	149	200.8	6110	149	200.9	7390
150	252.5	4970	150	200.8	6210	150	200.9	7390
151	252.4	4950	151	200.9	6210	151	201	7390
152	252.4	4960	152	200.8	6220	152	201	7380
153	252.5	4980	153	200.8	6220	153	201	7390
154	252.4	4980	154	200.8	6210	154	201	7390
155	252.4	4970	155	200.8	6190	155	201	7400
156	252.4	4970	156	200.8	6200	156	201	7390
157	252.4	4970	157	200.8	6200	157	201	7390
158	252.4	4970	158	200.8	6210	158	200.9	7390
159	252.5	4970	159	200.8	6210	159	200.9	7390
160	252.5	4970	160	200.9	6200	160	200.9	7390
161	252.5	4970	161	200.9	6200	161	200.9	7390
162	252.5	4970	162	200.8	6210	162	200.9	7400
163	252.6	4980	163	200.9	6210	163	200.9	7400
164	252.6	4970	164	200.8	6110	164	200.9	7390
165	252.6	4970	165	200.8	6110	165	200.9	7380
166	252.6	4960	166	200.9	6110	166	200.9	7390
167	252.6	4960	167	200.8	6200	167	200.9	7400
168	252.6	4970	168	200.9	6210	168	200.8	7390
169	252.6	4970	169	200.9	6100	169	200.8	7400
170	252.6	4970	170	200.9	6120	170	200.8	7390
171	252.6	4970	171	200.9	6120	171	200.8	7400
172	252.6	4970	172	200.9	6200	172	200.8	7400
173	252.7	4980	173	200.9	6210	173	200.8	7390
174	252.7	4970	174	200.9	6100	174	200.7	7400
175	252.7	4980	175	200.9	6200	175	200.7	7390
176	252.7	4970	176	200.9	6200	176	200.6	7400
177	252.7	4970	177	200.9	6210	177	200.6	7400
178	252.7	4970	178	200.9	6210	178	200.6	7390
179	252.7	4960	179	200.9	6210	179	200.5	7400
180	252.8	4960	180	200.9	6210	180	200.5	7390
181	252.8	4970	181	200.9	6210	181	200.5	7400
182	252.8	4960	182	200.9	6220	182	200.5	7400
183	252.8	4970	183	200.9	6200	183	200.4	7400
184	252.8	4980	184	200.9	6210	184	200.4	7400
185	252.8	4960	185	200.9	6210	185	200.3	7400
186	252.8	4970	186	200.9	6200	186	200.4	7390
187	252.8	4980	187	200.9	6220	187	200.4	7400
188	252.8	4970	188	200.9	6200	188	200.5	7490
189	252.8	4970	189	200.9	6210	189	200.5	7400
190	252.8	4970	190	200.9	6210	190	200.6	7500
191	252.8	4960	191	200.8	6210	191	200.6	7390
192	252.8	4970	192	200.9	6200	192	200.7	7410
193	252.8	4970	193	200.9	6210	193	200.7	7400
194	252.8	4970	194	200.8	6200	194	200.8	7390
195	252.8	4970	195	200.8	6220	195	200.8	7380
196	252.9	4980	196	200.8	6210	196	200.8	7390

197	252.9	4960	197	200.8	6190	197	200.8	7400
198	252.9	4970	198	200.8	6200	198	200.9	7400
199	252.8	4970	199	200.8	6200	199	200.9	7390
200	252.8	4960	200	200.8	6200	200	200.9	7390
201	252.8	4970	201	200.8	6220	201	200.9	7500
202	252.8	4960	202	200.8	6200	202	200.9	7500
203	252.8	4960	203	200.8	6210	203	200.9	7400
204	252.9	4970	204	200.9	6200	204	200.9	7400
205	252.9	4980	205	200.9	6190	205	200.9	7410
206	252.9	4970	206	200.9	6200	206	200.9	7390
207	252.9	4960	207	200.9	6210	207	200.9	7400
208	252.8	4960	208	200.8	6200	208	200.9	7400
209	252.7	4970	209	200.9	6110	209	200.9	7410
210	252.7	4970	210	200.9	6190	210	200.9	7490
211	252.6	4960	211	200.9	6210	211	200.9	7500
212	252.6	4970	212	200.9	6210	212	200.9	7400
213	252.5	4980	213	200.9	6200	213	200.9	7400
214	252.5	4970	214	200.9	6200	214	200.9	7400
215	252.5	4960	215	200.9	6200	215	200.9	7490
216	252.4	4970	216	200.9	6210	216	200.9	7490
217	252.4	4980	217	200.9	6210	217	200.9	7490
218	252.4	4970	218	200.9	6220	218	200.8	7400
219	252.4	4980	219	200.9	6200	219	200.8	7390
220	252.4	4990	220	200.9	6200	220	200.8	7400
221	252.3	4980	221	201	6210	221	200.8	7490
222	252.3	4970	222	201	6200	222	200.7	7390
223	252.2	4980	223	201	6210	223	200.8	7390
224	252.2	5000	224	201	6210	224	200.7	7390
225	252.2	4980	225	201	6200	225	200.7	7380
226	252.2	4970	226	201	6210	226	200.7	7400
227	252.2	4970	227	201	6200	227	200.7	7400
228	252.2	4960	228	201	6210	228	200.7	7380
229	252.2	4960	229	201	6200	229	200.7	7390
230	252.2	4970	230	201	6200	230	200.7	7390
231	252.2	4970	231	201	6210	231	200.6	7390
232	252.2	4970	232	201	6200	232	200.6	7490
233	252.1	4960	233	201	6220	233	200.6	7390
234	252.1	4970	234	201	6210	234	200.6	7490
235	252.1	4950	235	201	6200	235	200.6	7410
236	252.1	4960	236	201	6200	236	200.6	7410
237	252.1	4960	237	201	6210	237	200.5	7400
238	252.1	4970	238	201	6210	238	200.5	7400
239	252.1	4960	239	200.9	6210	239	200.5	7400
240	252.1	4960	240	200.9	6200	240	200.6	7500
241	252	4960	241	200.9	6200	241	200.5	7400
242	252	4980	242	200.9	6200	242	200.5	7510
243	252	4970	243	200.9	6190	243	200.5	7400
244	252	4960	244	200.9	6200	244	200.5	7400
245	252	4980	245	200.9	6190	245	200.5	7390
246	252	4960	246	200.9	6190	246	200.5	7400

247	252	4960	247	200.9	6200	247	200.4	7400
248	252	4970	248	200.9	6200	248	200.4	7500
249	252	4960	249	200.9	6190	249	200.4	7490
250	252	4960	250	200.9	6200	250	200.4	7490
251	252	4970	251	200.9	6200	251	200.4	7390
252	252	4980	252	200.9	6200	252	200.4	7400
253	252	4970	253	200.9	6200	253	200.3	7390
254	252	4970	254	200.9	6210	254	200.3	7390
255	252	4960	255	200.9	6220	255	200.3	7490
256	252.1	4970	256	200.9	6200	256	200.2	7390
257	252.1	4980	257	200.9	6210	257	200.2	7400
258	252	4980	258	200.9	6200	258	200.2	7390
259	252	4970	259	200.9	6220	259	200.2	7380
260	252	4980	260	200.9	6220	260	200.2	7400
261	252.1	4980	261	201	6210	261	200.1	7380
262	252	4970	262	200.9	6110	262	200.1	7390
263	252.1	4970	263	200.9	6210	263	200.1	7400
264	252.1	4980	264	200.9	6200	264	200.1	7400
265	252.1	4970	265	200.9	6210	265	200	7500
266	252.1	4970	266	200.9	6200	266	200	7490
267	252	4980	267	200.9	6210	267	200.1	7390
268	252	4970	268	200.9	6210	268	200.1	7390
269	252	4980	269	200.9	6210	269	200.1	7390
270	252.1	4970	270	200.9	6200	270	200	7490
271	252.1	4970	271	201	6210	271	200	7490
272	252.1	4970	272	200.9	6210	272	200	7400
273	252.2	4970	273	201	6210	273	200.1	7400
274	252.2	4980	274	201	6220	274	200.2	7400
275	252.1	4970	275	201	6230	275	200.2	7380
276	252.1	4970	276	201	6220	276	200.3	7400
277	252.1	4980	277	200.9	6220	277	200.4	7400
278	252.1	4980	278	200.9	6210	278	200.4	7380
279	252.1	4970	279	200.9	6220	279	200.5	7380
280	252.1	4970	280	200.9	6210	280	200.5	7390
281	252.1	4960	281	200.9	6210	281	200.6	7400
282	252.2	4970	282	200.9	6210	282	200.6	7400
283	252.1	4970	283	200.9	6210	283	200.6	7490
284	252.2	4960	284	200.9	6210	284	200.6	7490
285	252.2	4970	285	200.9	6210	285	200.7	7480
286	252.2	4970	286	201	6210	286	200.7	7510
287	252.2	4960	287	201	6210	287	200.7	7500
288	252.2	4970	288	201	6200	288	200.7	7500
289	252.2	4970	289	201	6210	289	200.8	7500
290	252.1	4960	290	201	6210	290	200.8	7500
291	252.2	4950	291	201	6210	291	200.7	7500
292	252.2	4960	292	201	6200	292	200.8	7500
293	252.2	4950	293	201	6210	293	200.8	7500
294	252.2	4960	294	201	6110	294	200.8	7400
295	252.2	4960	295	201	6220	295	200.8	7490
296	252.2	4970	296	201	6210	296	200.8	7410

297	252.2	4950	297	201	6190	297	200.8	7490
298	252.2	4970	298	201	6210	298	200.8	7500
299	252.3	4970	299	201	6120	299	200.8	7500
300	252.3	4970	300	201	6210	300	200.8	7490
301	252.3	4950	301	201	6210	301	200.8	7500
302	252.3	4970	302	201	6120	302	200.9	7500
303	252.3	4970	303	201	6230	303	200.8	7500
304	252.3	4980	304	201	6220	304	200.8	7500
305	252.3	4950	305	201	6100	305	200.8	7510
306	252.3	4980	306	201	6210	306	200.8	7490
307	252.3	4980	307	201	6210	307	200.8	7490
308	252.3	4980	308	201.1	6210	308	200.8	7510
309	252.3	4970	309	201	6200	309	200.9	7500
310	252.3	4960	310	201	6200	310	200.8	7490
311	252.4	4960	311	201	6190	311	200.8	7500
312	252.4	4960	312	201	6210	312	200.8	7510
313	252.4	4970	313	201	6200	313	200.9	7510
314	252.4	4960	314	201	6210	314	200.9	7510
315	252.5	4970	315	201	6210	315	200.8	7500
316	252.5	4970	316	201	6210	316	200.9	7500
317	252.4	4970	317	201	6200	317	200.9	7510
318	252.4	4960	318	201	6210	318	200.9	7510
319	252.4	4980	319	201	6210	319	200.8	7500
320	252.5	4970	320	201	6210	320	200.8	7500
321	252.5	4970	321	201	6210	321	200.8	7510
322	252.5	4960	322	201	6210	322	200.8	7500
323	252.5	4970	323	201	6200	323	200.7	7510
324	252.5	4970	324	201.1	6190	324	200.7	7500
325	252.5	4970	325	201.1	6200	325	200.8	7510
326	252.5	4970	326	201.1	6200	326	200.8	7510
327	252.5	4970	327	201.1	6190	327	200.7	7510
328	252.5	4970	328	201	6210	328	200.7	7500
329	252.5	4970	329	201	6220	329	200.7	7500
330	252.5	4960	330	201.1	6200	330	200.7	7490
331	252.5	4960	331	201	6200	331	200.7	7490
332	252.5	4970	332	201	6200	332	200.6	7500
333	252.5	4970	333	201	6220	333	200.6	7500
334	252.6	4960	334	201.1	6210	334	200.6	7500
335	252.5	4960	335	201	6220	335	200.6	7500
336	252.5	4960	336	201.1	6220	336	200.6	7510
337	252.6	4970	337	201.1	6210	337	200.6	7510
338	252.5	4970	338	201.1	6210	338	200.6	7510
339	252.5	4980	339	201	6210	339	200.6	7500
340	252.5	4970	340	201	6210	340	200.6	7510
341	252.5	4970	341	201	6210	341	200.6	7510
342	252.5	4960	342	201	6210	342	200.6	7500
343	252.4	4970	343	201	6210	343	200.6	7500
344	252.5	4970	344	201	6200	344	200.5	7510
345	252.4	4970	345	201	6210	345	200.5	7490
346	252.4	4980	346	201	6200	346	200.5	7490

347	252.4	4980	347	201	6190	347	200.5	7490
348	252.4	4970	348	201	6190	348	200.5	7500
349	252.4	4960	349	201	6200	349	200.5	7500
350	252.4	4970	350	201.1	6180	350	200.5	7490
351	252.4	4970	351	201.1	6200	351	200.5	7510
352	252.5	4970	352	201.1	6200	352	200.5	7500
353	252.4	4960	353	201.1	6200	353	200.5	7490
354	252.5	4970	354	201.1	6210	354	200.5	7490
355	252.5	4960	355	201.1	6190	355	200.5	7500
356	252.5	4970	356	201.1	6190	356	200.5	7500
357	252.6	4970	357	201.1	6200	357	200.5	7510
358	252.5	4960	358	201.1	6190	358	200.5	7500
359	252.5	4970	359	201.1	6200	359	200.5	7500
360	252.6	4960	360	201.1	6210	360	200.5	7500
361	252.5	4960	361	201.1	6200	361	200.4	7500
362	252.5	4970	362	201.1	6210	362	200.4	7500
363	252.5	4980	363	201.1	6200	363	200.5	7510
364	252.6	4960	364	201.1	6210	364	200.4	7490
365	252.5	4970	365	201.2	6210	365	200.4	7490
366	252.6	4950	366	201.2	6200	366	200.4	7510
367	252.6	4970	367	201.2	6190	367	200.4	7500
368	252.6	4960	368	201.2	6190	368	200.4	7500
369	252.5	4960	369	201.2	6190	369	200.4	7500
370	252.5	4960	370	201.2	6200	370	200.4	7510
371	252.5	4950	371	201.2	6110	371	200.4	7510
372	252.4	4960	372	201.2	6200	372	200.4	7500
373	252.5	4970	373	201.1	6210	373	200.3	7500
374	252.4	4960	374	201.1	6200	374	200.3	7510
375	252.4	4950	375	201.1	6200	375	200.3	7490
376	252.4	4960	376	201.1	6210	376	200.3	7490
377	252.5	4960	377	201.1	6200	377	200.3	7500
378	252.5	4960	378	201.1	6200	378	200.3	7500
379	252.5	4970	379	201.1	6190	379	200.3	7500
380	252.5	4960	380	201.1	6190	380	200.3	7490
381	252.5	4970	381	201.1	6190	381	200.3	7510
382	252.5	4970	382	201.1	6190	382	200.2	7500
383	252.5	4960	383	201.1	6200	383	200.2	7500
384	252.5	4970	384	201.1	6190	384	200.2	7510
385	252.5	4960	385	201.1	6200	385	200.2	7510
386	252.5	4960	386	201.1	6200	386	200.2	7490
387	252.5	4960	387	201.1	6210	387	200.2	7500
388	252.5	4980	388	201.1	6190	388	200.2	7490
389	252.5	4980	389	201.1	6200	389	200.2	7490
390	252.5	4970	390	201.1	6200	390	200.2	7490
391	252.5	4960	391	201.1	6200	391	200.1	7490
392	252.5	4970	392	201.1	6200	392	200.1	7500
393	252.5	4970	393	201.1	6190	393	200.2	7490
394	252.5	4970	394	201.1	6200	394	200.1	7500
395	252.5	4960	395	201.1	6190	395	200.1	7490
396	252.5	4970	396	201.1	6190	396	200.1	7480

397	252.5	4970	397	201.1	6200	397	200.1	7490
398	252.5	4980	398	201.2	6200	398	200.1	7500
399	252.5	4950	399	201.1	6200	399	200.1	7490
400	252.5	4960	400	201.1	6190	400	200.1	7490
401	252.6	4950	401	201.1	6200	401	200.1	7500
402	252.5	4960	402	201.1	6200	402	200.1	7490
403	252.6	4960	403	201.1	6190	403	200.1	7490
404	252.6	4980	404	201.1	6200	404	200.1	7490
405	252.6	4960	405	201.1	6200	405	200	7490
406	252.6	4960	406	201.1	6180	406	200	7500
407	252.7	4980	407	201.2	6200	407	200	7490
408	252.7	4970	408	201.2	6200	408	200	7500
409	252.7	4960	409	201.2	6210	409	200	7480
410	252.6	4970	410	201.2	6200	410	200	7490
411	252.6	4970	411	201.2	6200	411	200	7490
412	252.6	4960	412	201.2	6200	412	200	7480
413	252.6	4960	413	201.2	6210	413	199.9	7490
414	252.7	4960	414	201.2	6220	414	199.9	7480
415	252.6	4960	415	201.2	6200	415	199.9	7490
416	252.7	4960	416	201.3	6200	416	199.9	7490
417	252.7	4970	417	201.3	6200	417	199.9	7500
418	252.7	4960	418	201.2	6200	418	199.9	7510
419	252.7	4950	419	201.3	6190	419	199.9	7500
420	252.8	4960	420	201.3	6210	420	199.9	7500
421	252.8	4970	421	201.3	6210	421	199.9	7500
422	252.8	4950	422	201.3	6200	422	199.9	7500
423	252.8	4960	423	201.3	6190	423	199.9	7500
424	252.8	4970	424	201.3	6190	424	199.9	7480
425	252.8	4960	425	201.3	6190	425	199.9	7500
426	252.8	4960	426	201.3	6200	426	199.8	7500
427	252.8	4960	427	201.3	6210	427	199.8	7500
428	252.7	4970	428	201.3	6210	428	199.8	7500
429	252.7	4960	429	201.3	6200	429	199.8	7500
430	252.7	4960	430	201.4	6200	430	199.8	7490
431	252.7	4960	431	201.4	6210	431	199.8	7490
432	252.7	4960	432	201.4	6210	432	199.8	7500
433	252.7	4970	433	201.4	6190	433	199.8	7500
434	252.7	4960	434	201.4	6200	434	199.8	7490
435	252.6	4950	435	201.4	6200	435	199.8	7500
436	252.7	4970	436	201.4	6210	436	199.8	7500
437	252.7	4970	437	201.4	6190	437	199.8	7500
438	252.7	4970	438	201.3	6190	438	199.8	7500
439	252.7	4960	439	201.4	6210	439	199.8	7500
440	252.7	4960	440	201.4	6200	440	199.8	7490
441	252.7	4960	441	201.4	6190	441	199.7	7500
442	252.7	4970	442	201.4	6200	442	199.8	7510
443	252.7	4960	443	201.4	6200	443	199.7	7500
444	252.7	4960	444	201.4	6200	444	199.7	7490
445	252.7	4970	445	201.4	6190	445	199.7	7490
446	252.6	4970	446	201.4	6190	446	199.7	7490

447	252.6	4950	447	201.4	6200	447	199.7	7490
448	252.6	4970	448	201.4	6210	448	199.7	7490
449	252.7	4960	449	201.3	6200	449	199.7	7500
450	252.6	4960	450	201.4	6210	450	199.7	7500
451	252.7	4980	451	201.3	6200	451	199.7	7490
452	252.7	4960	452	201.3	6200	452	199.7	7500
453	252.7	4960	453	201.4	6190	453	199.7	7490
454	252.7	4950	454	201.4	6190	454	199.7	7500
455	252.7	4960	455	201.3	6210	455	199.6	7500
456	252.7	4960	456	201.4	6200	456	199.6	7500
457	252.7	4960	457	201.4	6200	457	199.6	7490
458	252.7	4970	458	201.4	6190	458	199.6	7490
459	252.7	4960	459	201.4	6210	459	199.6	7490
460	252.7	4960	460	201.4	6200	460	199.6	7500
461	252.7	4960	461	201.4	6200	461	199.6	7500
462	252.7	4940	462	201.4	6210	462	199.6	7490
463	252.7	4980	463	201.4	6200	463	199.6	7490
464	252.7	4970	464	201.4	6210	464	199.6	7500
465	252.7	4960	465	201.4	6210	465	199.6	7500
466	252.7	4950	466	201.4	6190	466	199.6	7490
467	252.7	4960	467	201.3	6200	467	199.6	7480
468	252.7	4960	468	201.4	6210	468	199.6	7500
469	252.7	4950	469	201.3	6200	469	199.6	7510
470	252.7	4950	470	201.3	6200	470	199.6	7490
471	252.7	4960	471	201.4	6200	471	199.5	7500
472	252.7	4980	472	201.4	6210	472	199.5	7490
473	252.7	4960	473	201.3	6190	473	199.6	7490
474	252.7	4970	474	201.4	6200	474	199.5	7500
475	252.7	4970	475	201.4	6210	475	199.5	7490
476	252.8	4960	476	201.4	6210	476	199.5	7490
477	252.8	4960	477	201.4	6200	477	199.5	7510
478	252.8	4960	478	201.3	6200	478	199.5	7500
479	252.8	4950	479	201.4	6200	479	199.5	7490
480	252.9	4960	480	201.4	6220	480	199.5	7490
481	252.8	4970	481	201.4	6210	481	199.5	7490
482	252.9	4960	482	201.4	6200	482	199.4	7500
483	252.9	4950	483	201.4	6210	483	199.4	7480
484	252.9	4960	484	201.4	6200	484	199.4	7500
485	252.9	4960	485	201.4	6200	485	199.4	7510
486	252.9	4960	486	201.4	6200	486	199.4	7500
487	252.8	4960	487	201.4	6210	487	199.4	7500
488	252.9	4960	488	201.4	6200	488	199.4	7500
489	252.9	4960	489	201.4	6190	489	199.4	7500
490	252.9	4970	490	201.5	6200	490	199.3	7510
491	252.9	4960	491	201.5	6190	491	199.4	7500
492	252.9	4960	492	201.4	6210	492	199.4	7510
493	252.9	4960	493	201.5	6200	493	199.4	7490
494	252.8	4960	494	201.4	6200	494	199.4	7490
495	252.9	4960	495	201.4	6210	495	199.4	7500
496	252.8	4950	496	201.5	6200	496	199.4	7490

497	252.8	4970	497	201.5	6210	497	199.4	7500
498	252.8	4950	498	201.5	6210	498	199.4	7490
499	252.8	4960	499	201.5	6200	499	199.4	7500
500	252.8	4970	500	201.5	6190	500	199.4	7500
501	252.8	4960	501	201.5	6200	501	199.3	7490
502	252.8	4960	502	201.5	6200	502	199.3	7490
503	252.7	4960	503	201.5	6200	503	199.3	7500
504	252.7	4950	504	201.6	6200	504	199.3	7500
505	252.8	4950	505	201.5	6200	505	199.3	7500
506	252.8	4960	506	201.5	6200	506	199.3	7510
507	252.7	4970	507	201.6	6200	507	199.3	7500
508	252.7	4970	508	201.5	6200	508	199.2	7510
509	252.8	4950	509	201.5	6200	509	199.2	7510
510	252.7	4960	510	201.5	6210	510	199.2	7500
511	252.7	4970	511	201.5	6200	511	199.2	7500
512	252.8	4970	512	201.5	6190	512	199.2	7500
513	252.8	4970	513	201.5	6200	513	199.2	7490
514	252.8	4950	514	201.5	6190	514	199.2	7500
515	252.7	4950	515	201.5	6200	515	199.2	7500
516	252.8	4950	516	201.5	6200	516	199.2	7500
517	252.8	4970	517	201.5	6210	517	199.2	7500
518	252.8	4960	518	201.5	6190	518	199.2	7510
519	252.8	4960	519	201.5	6200	519	199.2	7510
520	252.9	4950	520	201.5	6200	520	199.2	7500
521	252.9	4960	521	201.5	6190	521	199.2	7500
522	252.8	4960	522	201.5	6200	522	199.2	7500
523	252.8	4950	523	201.5	6200	523	199.1	7500
524	252.8	4960	524	201.5	6200	524	199.1	7500
525	252.8	4960	525	201.6	6210	525	199.1	7500
526	252.8	4970	526	201.6	6190	526	199.1	7500
527	252.8	4960	527	201.6	6200	527	199.1	7500
528	252.8	4960	528	201.6	6200	528	199.1	7500
529	252.8	4970	529	201.6	6200	529	199.1	7510
530	252.9	4950	530	201.6	6200	530	199.1	7490
531	252.9	4960	531	201.6	6210	531	199.1	7500
532	252.9	4970	532	201.6	6210	532	199.1	7500
533	252.9	4970	533	201.6	6210	533	199.1	7500
534	253	4970	534	201.5	6200	534	199.1	7510
535	253	4980	535	201.5	6210	535	199.1	7500
536	253	4970	536	201.6	6200	536	199.1	7510
537	253	4970	537	201.6	6200	537	199.1	7500
538	253	4960	538	201.6	6210	538	199.1	7510
539	253	4970	539	201.6	6200	539	199.1	7500
540	252.9	4960	540	201.6	6210	540	199.1	7520
541	252.9	4950	541	201.6	6210	541	199.1	7500
542	252.9	4960	542	201.6	6210	542	199	7500
543	252.9	4950	543	201.6	6210	543	199	7500
544	252.9	4960	544	201.6	6200	544	199	7510
545	252.9	4960	545	201.6	6210	545	199	7500
546	252.9	4950	546	201.6	6200	546	199	7500

547	252.9	4940	547	201.6	6200	547	199	7500
548	253	4960	548	201.6	6200	548	199	7500
549	252.9	4970	549	201.6	6220	549	199	7500
550	252.9	4950	550	201.6	6210	550	199	7490
551	252.9	4960	551	201.6	6210	551	199	7490
552	252.9	4970	552	201.6	6210	552	199	7510
553	252.9	4960	553	201.6	6210	553	199	7510
554	252.9	4960	554	201.6	6210	554	199	7510
555	253	4960	555	201.6	6220	555	199	7500
556	252.9	4960	556	201.6	6210	556	199	7510
557	252.9	4950	557	201.6	6200	557	199	7510
558	252.9	4960	558	201.6	6200	558	199	7510
559	252.9	4960	559	201.6	6210	559	199	7500
560	252.9	4970	560	201.6	6220	560	199	7500
561	252.9	4960	561	201.6	6200	561	199	7500
562	252.9	4970	562	201.6	6200	562	198.9	7510
563	253	4960	563	201.6	6210	563	198.9	7510
564	253	4960	564	201.6	6200	564	198.9	7520
565	253	4950	565	201.6	6200	565	199	7510
566	253	4950	566	201.6	6210	566	199	7520
567	253.1	4970	567	201.6	6210	567	199	7510
568	253.1	4960	568	201.7	6190	568	198.9	7520
569	253.1	4960	569	201.6	6210	569	199	7510
570	253.1	4950	570	201.6	6200	570	199	7520
571	253.1	4930	571	201.6	6200	571	199	7500
572	253.1	4950	572	201.6	6210	572	199	7510
573	253.1	4940	573	201.6	6200	573	199	7510
574	253.1	4950	574	201.6	6200	574	199	7510
575	253.1	4950	575	201.6	6200	575	199	7510
576	253.1	4950	576	201.6	6200	576	199	7520
577	253.1	4950	577	201.6	6200	577	199	7500
578	253.1	4950	578	201.6	6200	578	199	7510
579	253	4950	579	201.6	6200	579	199	7520
580	253.1	4950	580	201.6	6200	580	199	7500
581	253.1	4950	581	201.6	6200	581	198.9	7500
582	253.1	4960	582	201.6	6190	582	198.9	7520
583	253	4950	583	201.6	6210	583	198.9	7510
584	253	4960	584	201.6	6210	584	198.9	7510
585	253.1	4950	585	201.6	6220	585	198.9	7500
586	253	4950	586	201.6	6220	586	198.9	7500
587	253.1	4950	587	201.6	6210	587	198.9	7510
588	253.1	4960	588	201.6	6200	588	198.9	7500
589	253.1	4950	589	201.6	6200	589	198.9	7510
590	253.1	4960	590	201.6	6190	590	198.9	7510
591	253.1	4960	591	201.6	6200	591	198.9	7510
592	253.1	4960	592	201.6	6200	592	198.8	7500
593	253.1	4960	593	201.6	6200	593	198.9	7500
594	253.1	4960	594	201.6	6210	594	198.9	7510
595	253.1	4950	595	201.6	6200	595	198.9	7500
596	253.2	4960	596	201.6	6210	596	198.8	7500

597	253.2	4950	597	201.6	6210	597	198.9	7500
598	253.2	4950	598	201.6	6210	598	198.8	7500
599	253.1	4950	599	201.6	6210	599	198.9	7500
600	253.2	4940	600	201.6	6200	600	198.9	7500
601	253.2	4950	601	201.6	6200	601	198.9	7510
602	253.2	4950	602	201.7	6210	602	198.9	7500
603	253.2	4950	603	201.6	6190	603	198.9	7500
604	253.2	4960	604	201.7	6200	604	198.9	7500
605	253.2	4960	605	201.6	6210	605	198.9	7510
606	253.3	4960	606	201.7	6190	606	198.9	7500
607	253.3	4950	607	201.6	6200	607	198.9	7510
608	253.3	4960	608	201.6	6200	608	198.9	7500
609	253.3	4960	609	201.7	6190	609	198.9	7500
610	253.3	4960	610	201.7	6210	610	198.9	7490
611	253.3	4970	611	201.7	6200	611	198.9	7500
612	253.3	4970	612	201.7	6200	612	198.9	7510
613	253.2	4960	613	201.7	6190	613	198.9	7500
614	253.3	4960	614	201.7	6190	614	198.9	7500
615	253.2	4960	615	201.6	6200	615	198.9	7510
616	253.2	4960	616	201.6	6200	616	198.9	7500
617	253.2	4970	617	201.6	6190	617	198.9	7510
618	253.3	4960	618	201.6	6190	618	198.9	7510
619	253.3	4950	619	201.6	6200	619	198.9	7500
620	253.3	4960	620	201.6	6200	620	198.9	7500
621	253.3	4950	621	201.7	6200	621	198.9	7510
622	253.3	4950	622	201.7	6200	622	198.9	7510
623	253.3	4960	623	201.7	6210	623	198.9	7510
624	253.4	4970	624	201.7	6210	624	198.8	7510
625	253.4	4960	625	201.7	6210	625	198.9	7500
626	253.4	4960	626	201.7	6210	626	198.8	7500
627	253.4	4960	627	201.7	6190	627	198.8	7500
628	253.4	4970	628	201.7	6190	628	198.8	7500
629	253.4	4940	629	201.7	6200	629	198.8	7500
630	253.4	4930	630	201.7	6190	630	198.8	7500
631	253.4	4950	631	201.7	6190	631	198.8	7510
632	253.4	4950	632	201.7	6200	632	198.8	7510
633	253.4	4950	633	201.7	6200	633	198.8	7510
634	253.4	4950	634	201.7	6200	634	198.8	7500
635	253.4	4950	635	201.7	6200	635	198.8	7500
636	253.4	4970	636	201.7	6190	636	198.8	7490
637	253.4	4950	637	201.7	6200	637	198.8	7500
638	253.4	4960	638	201.7	6210	638	198.8	7500
639	253.4	4950	639	201.7	6200	639	198.8	7500
640	253.4	4940	640	201.7	6200	640	198.8	7500
641	253.4	4960	641	201.7	6200	641	198.8	7500
642	253.4	4950	642	201.7	6190	642	198.8	7520
643	253.4	4950	643	201.7	6200	643	198.8	7510
644	253.4	4960	644	201.7	6200	644	198.8	7490
645	253.4	4960	645	201.7	6210	645	198.8	7490
646	253.3	4970	646	201.7	6200	646	198.8	7490

647	253.2	4950	647	201.7	6200	647	198.8	7490
648	253.1	4950	648	201.7	6200	648	198.7	7500
649	253.1	4960	649	201.6	6210	649	198.7	7510
650	253	4970	650	201.6	6190	650	198.7	7500
651	253	4960	651	201.6	6190	651	198.7	7500
652	252.9	4950	652	201.6	6200	652	198.7	7500
653	252.9	4960	653	201.6	6190	653	198.8	7510
654	252.9	4950	654	201.7	6190	654	198.8	7490
655	252.8	4950	655	201.7	6200	655	198.7	7500
656	252.7	4950	656	201.7	6190	656	198.7	7500
657	252.7	4970	657	201.6	6200	657	198.8	7500
658	252.7	4960	658	201.6	6200	658	198.7	7500
659	252.7	4950	659	201.7	6200	659	198.7	7500
660	252.7	4950	660	201.7	6220	660	198.7	7500
661	252.7	4950	661	201.6	6200	661	198.7	7490
662	252.6	4960	662	201.6	6200	662	198.7	7510
663	252.6	4960	663	201.7	6200	663	198.7	7500
664	252.6	4950	664	201.6	6200	664	198.7	7510
665	252.6	4960	665	201.7	6210	665	198.7	7500
666	252.5	4950	666	201.7	6200	666	198.7	7510
667	252.5	4970	667	201.6	6210	667	198.7	7490
668	252.5	4950	668	201.7	6210	668	198.7	7500
669	252.5	4970	669	201.7	6200	669	198.7	7500
670	252.5	4950	670	201.7	6210	670	198.7	7500
671	252.5	4960	671	201.7	6190	671	198.7	7500
672	252.6	4940	672	201.7	6190	672	198.7	7510
673	252.5	4940	673	201.7	6200	673	198.7	7500
674	252.6	4960	674	201.7	6200	674	198.7	7510
675	252.5	4960	675	201.7	6200	675	198.7	7490
676	252.5	4960	676	201.7	6200	676	198.7	7500
677	252.5	4950	677	201.7	6210	677	198.7	7500
678	252.5	4950	678	201.6	6200	678	198.7	7500
679	252.5	4950	679	201.6	6210	679	198.7	7510
680	252.4	4960	680	201.6	6200	680	198.7	7510
681	252.4	4960	681	201.6	6230	681	198.6	7500
682	252.4	4950	682	201.6	6210	682	198.7	7500
683	252.4	4960	683	201.7	6200	683	198.7	7490
684	252.3	4960	684	201.7	6200	684	198.6	7490
685	252.3	4950	685	201.7	6190	685	198.7	7500
686	252.3	4950	686	201.8	6210	686	198.7	7500
687	252.3	4960	687	201.7	6200	687	198.6	7500
688	252.3	4970	688	201.7	6210	688	198.6	7510
689	252.3	4960	689	201.8	6200	689	198.7	7510
690	252.3	4960	690	201.7	6200	690	198.7	7510
691	252.3	4960	691	201.7	6200	691	198.6	7500
692	252.2	4960	692	201.7	6200	692	198.6	7520
693	252.3	4960	693	201.7	6220	693	198.6	7510
694	252.2	4960	694	201.7	6200	694	198.6	7520
695	252.2	4950	695	201.7	6210	695	198.6	7510
696	252.2	4960	696	201.7	6200	696	198.6	7500

697	252.2	4970	697	201.7	6210	697	198.6	7520
698	252.2	4960	698	201.7	6200	698	198.6	7500
699	252.2	4960	699	201.7	6220	699	198.6	7510
700	252.2	4960	700	201.7	6210	700	198.6	7500
701	252.2	4960	701	201.7	6200	701	198.6	7500
702	252.2	4950	702	201.7	6200	702	198.6	7490
703	252.1	4950	703	201.7	6200	703	198.6	7500
704	252.2	4950	704	201.7	6200	704	198.6	7500
705	252.1	4950	705	201.8	6210	705	198.7	7500
706	252.1	4950	706	201.8	6210	706	198.6	7500
707	252.1	4960	707	201.8	6220	707	198.6	7500
708	252.1	4950	708	201.7	6210	708	198.6	7500
709	252.1	4960	709	201.8	6200	709	198.6	7500
710	252.1	4960	710	201.8	6210	710	198.6	7510
711	252.1	4960	711	201.8	6210	711	198.6	7510
712	252	4960	712	201.8	6220	712	198.6	7510
713	252	4960	713	201.8	6200	713	198.6	7500
714	252	4960	714	201.8	6190	714	198.6	7510
715	252	4960	715	201.8	6220	715	198.6	7500
716	252	4960	716	201.8	6200	716	198.6	7500
717	252	4960	717	201.8	6190	717	198.6	7500
718	252	4950	718	201.8	6200	718	198.7	7500
719	252	4950	719	201.8	6210	719	198.6	7490
720	252	4950	720	201.7	6200	720	198.6	7510
721	252	4960	721	201.8	6200	721	198.6	7510
722	252	4950	722	201.8	6210	722	198.6	7510
723	252	4950	723	201.8	6210	723	198.6	7510
724	252	4940	724	201.8	6200	724	198.6	7500
725	252	4960	725	201.8	6210	725	198.5	7510
726	252	4950	726	201.8	6210	726	198.6	7510
727	252	4950	727	201.8	6210	727	198.6	7490
728	252	4960	728	201.8	6200	728	198.5	7500
729	252	4950	729	201.8	6210	729	198.6	7500
730	252	4950	730	201.8	6190	730	198.6	7500
731	252	4950	731	201.8	6210	731	198.5	7510
732	252	4950	732	201.8	6210	732	198.5	7510
733	252	4950	733	201.8	6200	733	198.5	7490
734	252	4950	734	201.8	6200	734	198.5	7500
735	252	4950	735	201.8	6190	735	198.5	7510
736	252	4940	736	201.8	6200	736	198.5	7510
737	252	4940	737	201.8	6210	737	198.5	7500
738	252	4950	738	201.8	6210	738	198.5	7500
739	252	4960	739	201.8	6210	739	198.5	7500
740	252	4950	740	201.8	6200	740	198.5	7500
741	252	4940	741	201.8	6200	741	198.5	7500
742	252	4950	742	201.8	6200	742	198.6	7500
743	252	4950	743	201.8	6190	743	198.5	7490
744	252	4940	744	201.8	6200	744	198.6	7520
745	252.1	4950	745	201.9	6210	745	198.5	7510
746	252.1	4940	746	201.9	6210	746	198.5	7500

747	252.1	4950	747	201.8	6210	747	198.5	7490
748	252	4960	748	201.8	6220	748	198.5	7500
749	252.1	4950	749	201.8	6200	749	198.5	7510
750	252	4950	750	201.8	6200	750	198.5	7500
751	252	4950	751	201.8	6190	751	198.5	7500
752	252	4960	752	201.8	6210	752	198.5	7510
753	252	4960	753	201.8	6210	753	198.5	7500
754	252	4960	754	201.8	6200	754	198.5	7510
755	252	4950	755	201.8	6210	755	198.5	7490
756	252	4960	756	201.8	6230	756	198.5	7500
757	252	4960	757	201.7	6210	757	198.5	7500
758	252	4960	758	201.8	6220	758	198.4	7510
759	252	4940	759	201.7	6220	759	198.5	7500
760	252	4950	760	201.8	6220	760	198.5	7500
761	252	4960	761	201.8	6200	761	198.5	7510
762	252	4960	762	201.8	6210	762	198.5	7500
763	252	4960	763	201.8	6200	763	198.4	7500
764	252	4960	764	201.8	6200	764	198.4	7500
765	251.9	4970	765	201.8	6210	765	198.4	7500
766	252	4980	766	201.8	6210	766	198.4	7510
767	252	4960	767	201.8	6200	767	198.5	7500
768	252	4960	768	201.8	6210	768	198.4	7490
769	252	4970	769	201.8	6210	769	198.4	7500
770	252	4960	770	201.8	6210	770	198.4	7490
771	252	4960	771	201.8	6220	771	198.4	7490
772	252	4950	772	201.8	6210	772	198.4	7500
773	252	4950	773	201.8	6200	773	198.5	7510
774	252	4960	774	201.8	6210	774	198.4	7520
775	252	4960	775	201.8	6200	775	198.4	7510
776	252	4960	776	201.8	6210	776	198.4	7510
777	252	4960	777	201.8	6200	777	198.4	7510
778	251.9	4960	778	201.8	6210	778	198.4	7510
779	252	4950	779	201.8	6210	779	198.4	7500
780	251.9	4940	780	201.8	6190	780	198.4	7510
781	251.9	4970	781	201.9	6200	781	198.4	7500
782	251.9	4950	782	201.9	6210	782	198.4	7520
783	252	4950	783	201.8	6200	783	198.4	7500
784	252	4950	784	201.9	6200	784	198.4	7500
785	251.9	4950	785	201.9	6200	785	198.4	7510
786	251.9	4950	786	201.8	6200	786	198.4	7500
787	251.9	4950	787	201.8	6200	787	198.4	7500
788	252	4940	788	201.8	6200	788	198.4	7510
789	251.9	4950	789	201.8	6210	789	198.4	7510
790	252	4950	790	201.8	6200	790	198.4	7500
791	251.9	4960	791	201.8	6200	791	198.4	7520
792	252	4950	792	201.8	6200	792	198.4	7510
793	252	4950	793	201.8	6210	793	198.4	7500
794	252	4940	794	201.8	6210	794	198.4	7510
795	252	4960	795	201.8	6210	795	198.4	7510
796	252	4950	796	201.8	6200	796	198.4	7510

797	252	4960	797	201.8	6200	797	198.4	7520
798	252	4950	798	201.8	6210	798	198.4	7510
799	252	4960	799	201.8	6210	799	198.4	7500
800	252	4960	800	201.8	6210	800	198.4	7500
801	252	4950	801	201.8	6220	801	198.4	7500
802	252	4960	802	201.8	6200	802	198.4	7510
803	252	4960	803	201.8	6200	803	198.4	7500
804	251.9	4960	804	201.8	6190	804	198.5	7500
805	251.9	4950	805	201.8	6200	805	198.4	7500
806	252	4960	806	201.8	6210	806	198.5	7490
807	252	4960	807	201.8	6200	807	198.5	7510
808	252	4950	808	201.8	6210	808	198.5	7500
809	251.9	4950	809	201.8	6200	809	198.5	7510
810	251.9	4960	810	201.8	6190	810	198.5	7510
811	251.9	4960	811	201.8	6200	811	198.5	7500
812	251.9	4950	812	201.8	6200	812	198.5	7500
813	251.9	4960	813	201.8	6200	813	198.5	7490
814	251.9	4960	814	201.8	6210	814	198.5	7500
815	251.9	4960	815	201.8	6210	815	198.5	7490
816	251.9	4950	816	201.8	6200	816	198.5	7510
817	251.9	4970	817	201.8	6210	817	198.5	7500
818	251.9	4960	818	201.8	6190	818	198.5	7500
819	251.9	4960	819	201.8	6210	819	198.5	7500
820	251.9	4960	820	201.8	6200	820	198.5	7500
821	251.9	4970	821	201.8	6200	821	198.5	7490
822	251.9	4960	822	201.8	6200	822	198.5	7510
823	251.9	4950	823	201.8	6200	823	198.5	7500
824	251.9	4940	824	201.8	6210	824	198.5	7500
825	251.9	4960	825	201.8	6200	825	198.5	7500
826	251.9	4960	826	201.8	6220	826	198.5	7500
827	251.9	4960	827	201.8	6200	827	198.5	7500
828	251.9	4970	828	201.8	6200	828	198.5	7510
829	251.9	4970	829	201.8	6210	829	198.5	7500
830	251.9	4970	830	201.8	6200	830	198.5	7500
831	251.9	4960	831	201.8	6210	831	198.5	7500
832	251.9	4950	832	201.8	6210	832	198.5	7500
833	251.9	4950	833	201.8	6190	833	198.5	7500
834	251.9	4950	834	201.8	6220	834	198.5	7500
835	251.9	4960	835	201.8	6210	835	198.4	7500
836	252	4960	836	201.8	6210	836	198.4	7500
837	252	4960	837	201.9	6200	837	198.4	7500
838	252	4960	838	201.9	6200	838	198.4	7490
839	252	4960	839	201.9	6190	839	198.4	7490
840	252	4950	840	201.9	6200	840	198.4	7510
841	252	4950	841	201.9	6210	841	198.4	7500
842	252	4960	842	201.9	6200	842	198.4	7500
843	252	4970	843	201.9	6210	843	198.4	7510
844	252	4960	844	202.2	6200	844	198.4	7510
845	252	4960	845	202.6	6210	845	198.5	7500
846	252	4950	846	203	6200	846	198.5	7500

847	252	4960	847	203.4	6210	847	198.5	7490
848	252	4960	848	203.7	6210	848	198.4	7500
849	252	4960	849	204.1	6210	849	198.4	7490
850	252	4960	850	204.5	6190	850	198.4	7510
851	252	4960	851	204.9	6200	851	198.4	7500
852	252	4960	852	205.3	6300	852	198.4	7500
853	252	4960	853	205.6	6190	853	198.4	7500
854	252	4960	854	205.9	6290	854	198.4	7490
855	251.9	4960	855	206.3	6290	855	198.4	7500
856	251.9	4970	856	206.7	6300	856	198.4	7490
857	252	4960	857	207.1	6290	857	198.4	7510
858	252	4950	858	207.5	6290	858	198.4	7500
859	252	4960	859	207.8	6300	859	198.4	7500
860	252	4950	860	208.2	6290	860	198.4	7490
861	252	4960	861	208.6	6290	861	198.4	7500
862	252	4960	862	209.1	6300	862	198.4	7500
863	252	4960	863	209.6	6280	863	198.4	7490
864	252	4960	864	210	6290	864	198.4	7500
865	252	4960	865	210.5	6300	865	198.4	7500
866	252	4950	866	210.9	6290	866	198.4	7500
867	252	4960	867	211.3	6300	867	198.4	7500
868	252	4950	868	211.7	6300	868	198.4	7610
869	252	4960	869	212.2	6300	869	198.4	7500
870	252	4950	870	212.6	6300	870	198.4	7510
871	252	4960	871	213.1	6390	871	198.4	7500
872	251.9	4960	872	213.5	6300	872	198.4	7490
873	251.9	4960	873	213.9	6400	873	198.3	7500
874	251.9	4960	874	214.3	6400	874	198.3	7500
875	251.9	4960	875	214.6	6400	875	198.3	7500
876	251.9	4960	876	214.9	6400	876	198.3	7500
877	251.9	4960	877	215.3	6390	877	198.3	7500
878	252	4960	878	215.6	6410	878	198.3	7500
879	251.9	4960	879	215.8	6410	879	198.3	7500
880	251.9	4960	880	216.1	6400	880	198.4	7500
881	252	4960	881	216.3	6400	881	198.4	7490
882	252	4960	882	216.6	6410	882	198.4	7500
883	252	4960	883	216.8	6400	883	198.4	7510
884	251.9	4970	884	217.1	6420	884	198.4	7510
885	252	4950	885	217.3	6410	885	198.4	7510
886	252	4960	886	217.5	6410	886	198.4	7510
887	252	4950	887	217.7	6420	887	198.4	7490
888	252	4960	888	217.8	6410	888	198.4	7500
889	252	4960	889	218	6400	889	198.4	7490
890	252	4980	890	218.2	6390	890	198.4	7500
891	252	4960	891	218.4	6400	891	198.4	7500
892	252	4960	892	218.5	6400	892	198.4	7500
893	252	4960	893	218.7	6390	893	198.4	7500
894	252	4970	894	218.8	6400	894	198.4	7500
895	252	4950	895	218.9	6400	895	198.4	7500
896	252	4950	896	219	6400	896	198.4	7500

897	252	4950	897	219.1	6400	897	198.4	7500
898	252	4960	898	219.3	6400	898	198.4	7490
899	252	4960	899	219.4	6400	899	198.4	7500
900	252	4970	900	219.5	6500	900	198.4	7500
901	252	4990	901	219.6	6500	901	198.4	7500
902	252	4960	902	219.7	6510	902	198.4	7510
903	252	4950	903	219.8	6510	903	198.4	7510
904	252	4960	904	219.9	6510	904	198.4	7610
905	252	4940	905	220	6500	905	198.4	7510
906	252	4950	906	220.2	6500	906	198.4	7500
907	252	4950	907	220.2	6500	907	198.4	7490
908	252	4950	908	220.4	6500	908	198.4	7500
909	252	4990	909	220.5	6510	909	198.4	7490
910	252	4960	910	220.6	6510	910	198.4	7490
911	252	4950	911	220.7	6510	911	198.4	7510
912	252	4950	912	220.8	6500	912	198.4	7500
913	252	4960	913	220.9	6500	913	198.4	7500
914	252.1	4960	914	220.9	6500	914	198.4	7590
915	252	4960	915	221	6510	915	198.4	7600
916	252.1	4960	916	221.1	6510	916	198.5	7500
917	252.1	4960	917	221.2	6500	917	198.5	7600
918	252.1	4980	918	221.3	6500	918	198.5	7510
919	252.1	4980	919	221.4	6500	919	198.5	7510
920	252.1	4980	920	221.4	6500	920	198.5	7490
921	252.1	4970	921	221.5	6490	921	198.5	7510
922	252.1	4990	922	221.6	6480	922	198.5	7490
923	252.1	4980	923	221.7	6490	923	198.4	7510
924	252.1	4960	924	221.8	6500	924	198.5	7500
925	252.1	4960	925	221.9	6490	925	198.4	7480
926	252.1	4960	926	222	6490	926	198.5	7510
927	252.1	4980	927	222.1	6490	927	198.4	7490
928	252.1	4980	928	222.2	6490	928	198.4	7500
929	252.1	4980	929	222.3	6490	929	198.4	7600
930	252.1	4990	930	222.4	6500	930	198.4	7580
931	252.1	4980	931	222.4	6490	931	198.4	7490
932	252.1	4950	932	222.5	6490	932	198.5	7500
933	252.1	4970	933	222.6	6500	933	198.4	7490
934	252	5010	934	222.7	6500	934	198.5	7600
935	252.1	4990	935	222.8	6500	935	198.4	7490
936	252	4980	936	222.8	6500	936	198.4	7500
937	252.1	4970	937	222.9	6500	937	198.5	7610
938	252.1	4990	938	223	6500	938	198.5	7590
939	252.1	5000	939	223	6490	939	198.4	7600
940	252.1	4980	940	223.1	6500	940	198.5	7610
941	252.1	4990	941	223.1	6500	941	198.5	7590
942	252.1	4980	942	223.1	6500	942	198.4	7600
943	252.1	4980	943	223.2	6510	943	198.4	7600
944	252.1	4990	944	223.3	6510	944	198.4	7590
945	252.1	4990	945	223.3	6490	945	198.4	7610
946	252.1	4990	946	223.4	6490	946	198.4	7590

947	252.1	5000	947	223.5	6510	947	198.4	7600
948	252.1	5000	948	223.5	6500	948	198.4	7590
949	252.1	5000	949	223.6	6510	949	198.4	7600
950	252.1	4990	950	223.6	6500	950	198.4	7600
951	252.1	5000	951	223.7	6500	951	198.4	7600
952	252.1	5000	952	223.8	6510	952	198.4	7600
953	252	5000	953	223.8	6520	953	198.4	7600
954	252.1	4990	954	223.9	6520	954	198.4	7590
955	252.1	4990	955	223.9	6490	955	198.4	7600
956	252.1	5010	956	224	6510	956	198.4	7600
957	252	5000	957	224.1	6500	957	198.4	7600
958	252.1	4990	958	224.2	6500	958	198.4	7590
959	252.1	4990	959	224.2	6490	959	198.4	7600
960	252.1	5000	960	224.3	6590	960	198.4	7610
961	252.1	4990	961	224.3	6490	961	198.4	7600
962	252.1	5000	962	224.4	6490	962	198.3	7510
963	252.1	4990	963	224.4	6490	963	198.4	7600
964	252.1	4990	964	224.5	6500	964	198.4	7600
965	252.1	5000	965	224.5	6490	965	198.4	7600
966	252.1	5010	966	224.6	6580	966	198.4	7610
967	252.1	4990	967	224.7	6580	967	198.4	7600
968	252.1	4990	968	224.7	6590	968	198.4	7600
969	252.1	4990	969	224.8	6600	969	198.4	7600
970	252.1	4970	970	224.9	6580	970	198.4	7580
971	252.1	4990	971	224.9	6580	971	198.4	7600
972	252.1	4990	972	225	6490	972	198.4	7590
973	252.1	5000	973	225	6590	973	198.4	7600
974	252.1	4990	974	225.1	6590	974	198.4	7610
975	252.1	5000	975	225.1	6590	975	198.4	7600
976	252.1	5000	976	225.2	6490	976	198.4	7610
977	252.1	5000	977	225.1	6600	977	198.4	7610
978	252.1	5000	978	225.1	6580	978	198.4	7600
979	252.1	4990	979	225.1	6580	979	198.4	7600
980	252.1	4990	980	225.1	6480	980	198.4	7600
981	252.1	4990	981	225.1	6490	981	198.4	7600
982	252.1	4990	982	225	6590	982	198.4	7610
983	252.1	4970	983	225	6590	983	198.4	7600
984	252.1	4990	984	225	6490	984	198.4	7600
985	252.1	4980	985	225	6590	985	198.4	7610
986	252.1	5000	986	225	6590	986	198.4	7610
987	252.1	4990	987	225	6600	987	198.4	7590
988	252.2	4980	988	225.1	6590	988	198.4	7600
989	252.2	4980	989	225	6590	989	198.4	7610
990	252.2	4990	990	225.1	6590	990	198.4	7610
991	252.2	4980	991	225.1	6580	991	198.4	7490
992	252.2	4980	992	225.1	6580	992	198.4	7500
993	252.1	4980	993	225.2	6580	993	198.4	7510
994	252.1	4980	994	225.2	6590	994	198.4	7610
995	252.1	4990	995	225.2	6600	995	198.4	7600
996	252.1	4990	996	225.2	6590	996	198.4	7620

997	252.1	4980	997	225.2	6600	997	198.4	7600
998	252.1	4990	998	225.2	6590	998	198.4	7610
999	252.1	4990	999	225.2	6590	999	198.4	7600
1000	252.1	4990	1000	225.3	6590	1000	198.4	7510
1001	252.1	5000	1001	225.3	6600	1001	198.4	7590
1002	252.1	4980	1002	225.4	6590	1002	198.4	7600
1003	252.1	4980	1003	225.4	6600	1003	198.4	7530
1004	252	4990	1004	225.4	6590	1004	198.4	7600
1005	252	4980	1005	225.5	6600	1005	198.4	7610
1006	252	4990	1006	225.5	6600	1006	198.4	7610
1007	252	4970	1007	225.5	6590	1007	198.4	7610
1008	252	4970	1008	225.6	6590	1008	198.4	7610
1009	252	4990	1009	225.6	6590	1009	198.4	7600
1010	252	4980	1010	225.6	6600	1010	198.3	7610
1011	252	4980	1011	225.6	6590	1011	198.4	7610
1012	252	4990	1012	225.7	6600	1012	198.4	7610
1013	252	4990	1013	225.7	6600	1013	198.4	7600
1014	252	4990	1014	225.7	6590	1014	198.4	7500
1015	252	4990	1015	225.7	6590	1015	198.4	7620
1016	252	5000	1016	225.7	6590	1016	198.4	7620
1017	251.9	4990	1017	225.8	6580	1017	198.4	7610
1018	251.9	4980	1018	225.8	6600	1018	198.4	7600
1019	252	4990	1019	225.8	6600	1019	198.4	7590
1020	252	4980	1020	225.9	6600	1020	198.4	7510
1021	252.1	4980	1021	225.9	6590	1021	198.4	7610
1022	252.1	4980	1022	225.9	6580	1022	198.4	7610
1023	252.1	4990	1023	225.9	6580	1023	198.4	7610
1024	252.1	4990	1024	226	6600	1024	198.4	7610
1025	252.1	4980	1025	226	6590	1025	198.4	7620
1026	252.1	4990	1026	226.1	6600	1026	198.4	7610
1027	252.1	4990	1027	226.1	6590	1027	198.4	7610
1028	252.1	4990	1028	226.1	6590	1028	198.3	7610
1029	252.1	4990	1029	226.1	6580	1029	198.4	7600
1030	252.1	4990	1030	226.2	6580	1030	198.3	7610
1031	252.1	4990	1031	226.2	6590	1031	198.4	7610
1032	252.1	5000	1032	226.2	6600	1032	198.4	7600
1033	252.2	4990	1033	226.3	6590	1033	198.4	7600
1034	252.2	5010	1034	226.3	6580	1034	198.4	7590
1035	252.2	4990	1035	226.3	6580	1035	198.4	7610
1036	252.1	5010	1036	226.3	6590	1036	198.4	7600
1037	252.2	5000	1037	226.3	6590	1037	198.4	7600
1038	252.2	5000	1038	226.4	6590	1038	198.4	7610
1039	252.2	5000	1039	226.4	6590	1039	198.4	7610
1040	252.2	4990	1040	226.4	6610	1040	198.4	7610
1041	252.2	5000	1041	226.4	6590	1041	198.4	7600
1042	252.1	5000	1042	226.4	6590	1042	198.4	7610
1043	252.1	5010	1043	226.5	6580	1043	198.4	7590
1044	252.1	5000	1044	226.5	6590	1044	198.4	7600
1045	252.1	5000	1045	226.5	6580	1045	198.4	7600
1046	252.1	5000	1046	226.6	6590	1046	198.4	7600

1047	252	5000	1047	226.5	6600	1047	198.4	7610
1048	252	5000	1048	226.6	6590	1048	198.4	7510
1049	252	4990	1049	226.7	6580	1049	198.4	7600
1050	252	5000	1050	226.7	6590	1050	198.4	7610
1051	252	5000	1051	226.7	6600	1051	198.4	7600
1052	252.1	5010	1052	226.7	6580	1052	198.4	7610
1053	252.1	5000	1053	226.7	6600	1053	198.4	7600
1054	252.1	5000	1054	226.7	6590	1054	198.4	7600
1055	252.1	4990	1055	226.7	6590	1055	198.4	7600
1056	252	4990	1056	226.7	6600	1056	198.4	7610
1057	252	5000	1057	226.8	6600	1057	198.5	7610
1058	252	4990	1058	226.8	6590	1058	198.4	7610
1059	252	4990	1059	226.8	6590	1059	198.4	7600
1060	252	4990	1060	226.9	6600	1060	198.4	7610
1061	252	4980	1061	226.8	6590	1061	198.4	7610
1062	252.1	4990	1062	226.9	6590	1062	198.5	7610
1063	252.1	4990	1063	226.9	6590	1063	198.5	7600
1064	252.1	4990	1064	226.9	6590	1064	198.5	7600
1065	252.1	4990	1065	227	6590	1065	198.5	7610
1066	252.1	4990	1066	226.9	6590	1066	198.5	7620
1067	252.1	5000	1067	227	6590	1067	198.5	7620
1068	252.1	5000	1068	227	6600	1068	198.5	7600
1069	252	5000	1069	227	6600	1069	198.5	7600
1070	252	5000	1070	227	6600	1070	198.4	7610
1071	252	4990	1071	227	6600	1071	198.5	7600
1072	252	4990	1072	227	6590	1072	198.5	7600
1073	252	5000	1073	227.1	6600	1073	198.4	7600
1074	252	5000	1074	227.1	6600	1074	198.5	7600
1075	252	4990	1075	227.1	6580	1075	198.4	7590
1076	252	4980	1076	227.1	6590	1076	198.5	7590
1077	252	4980	1077	227.2	6590	1077	198.5	7590
1078	252	4990	1078	227.2	6590	1078	198.5	7610
1079	252	4980	1079	227.3	6590	1079	198.5	7600
1080	252	4990	1080	227.3	6600	1080	198.5	7600
1081	251.9	4990	1081	227.3	6610	1081	198.5	7610
1082	251.9	5000	1082	227.2	6610	1082	198.5	7600
1083	252	4990	1083	227.2	6600	1083	198.5	7610
1084	251.9	5000	1084	227.1	6600	1084	198.5	7610
1085	251.9	4990	1085	227	6600	1085	198.5	7620
1086	251.9	5000	1086	226.8	6600	1086	198.4	7600
1087	252	5000	1087	226.7	6590	1087	198.4	7600
1088	252	4980	1088	226.6	6600	1088	198.5	7610
1089	252	4980	1089	226.5	6590	1089	198.4	7610
1090	252	4980	1090	226.4	6590	1090	198.4	7610
1091	252	5000	1091	226.4	6600	1091	198.4	7600
1092	252	5000	1092	226.3	6580	1092	198.5	7610
1093	252	5000	1093	226.3	6600	1093	198.4	7610
1094	252.1	5000	1094	226.2	6590	1094	198.5	7610
1095	252.1	5000	1095	226.1	6600	1095	198.5	7630
1096	252.1	5010	1096	226.1	6600	1096	198.4	7610

1097	252.1	4990	1097	226.1	6600	1097	198.4	7610
1098	252.1	4990	1098	226	6580	1098	198.4	7610
1099	252.1	4990	1099	226	6590	1099	198.4	7610
1100	252.1	5000	1100	226	6600	1100	198.4	7610
1101	252.1	4980	1101	225.9	6580	1101	198.4	7600
1102	252.1	4990	1102	225.9	6590	1102	198.4	7610
1103	252.1	4990	1103	225.9	6600	1103	198.4	7610
1104	252.1	4990	1104	225.9	6590	1104	198.4	7610
1105	252.1	4990	1105	225.9	6590	1105	198.4	7610
1106	252.1	4990	1106	225.8	6580	1106	198.4	7610
1107	252.1	5000	1107	225.8	6590	1107	198.5	7610
1108	252.1	4990	1108	225.8	6600	1108	198.5	7610
1109	252.1	4990	1109	225.8	6590	1109	198.5	7610
1110	252	4990	1110	225.8	6590	1110	198.4	7600
1111	252	4980	1111	225.8	6590	1111	198.5	7600
1112	252.1	5000	1112	225.8	6590	1112	198.5	7610
1113	252	4990	1113	225.8	6590	1113	198.5	7610
1114	252.1	4990	1114	225.7	6590	1114	198.5	7600
1115	252.1	5000	1115	225.8	6580	1115	198.5	7620
1116	252.1	5000	1116	225.7	6590	1116	198.5	7610
1117	252.1	4990	1117	225.7	6590	1117	198.5	7610
1118	252.1	4990	1118	225.8	6590	1118	198.5	7600
1119	252.1	5000	1119	225.7	6580	1119	198.5	7600
1120	252.1	5000	1120	225.7	6580	1120	198.5	7610
1121	252.1	4990	1121	225.7	6590	1121	198.5	7600
1122	252.1	5000	1122	225.7	6590	1122	198.4	7600
1123	252.1	4990	1123	225.7	6580	1123	198.5	7600
1124	252.1	4990	1124	225.7	6580	1124	198.4	7600
1125	252.1	4980	1125	225.6	6570	1125	198.4	7590
1126	252.1	4990	1126	225.6	6580	1126	198.4	7600
1127	252.1	4980	1127	225.6	6580	1127	198.4	7620
1128	252.1	4990	1128	225.6	6590	1128	198.4	7600
1129	252.1	4990	1129	225.6	6600	1129	198.4	7610
1130	252.1	4990	1130	225.6	6590	1130	198.4	7610
1131	252.1	4990	1131	225.6	6590	1131	198.4	7610
1132	252.1	4980	1132	225.6	6580	1132	198.4	7610
1133	252.2	4990	1133	225.6	6580	1133	198.4	7610
1134	252.2	4980	1134	225.6	6580	1134	198.4	7600
1135	252.2	4990	1135	225.6	6580	1135	198.4	7610
1136	252.2	4990	1136	225.6	6590	1136	198.4	7610
1137	252.2	4990	1137	225.6	6590	1137	198.4	7610
1138	252.2	4980	1138	225.6	6580	1138	198.5	7610
1139	252.2	5000	1139	225.6	6490	1139	198.9	7610
1140	252.2	4990	1140	225.7	6590	1140	199.6	7710
1141	252.2	4990	1141	225.7	6500	1141	200.4	7710
1142	252.2	4990	1142	225.7	6600	1142	201.3	7700
1143	252.2	5000	1143	225.7	6600	1143	202.3	7700
1144	252.2	4990	1144	225.7	6600	1144	203.4	7700
1145	252.2	5000	1145	225.7	6590	1145	204.4	7700
1146	252.2	4990	1146	225.7	6600	1146	205.5	7700

1147	252.2	5000	1147	225.7	6590	1147	206.4	7790
1148	252.2	4990	1148	225.7	6590	1148	207.3	7810
1149	252.1	5000	1149	225.6	6600	1149	208.3	7810
1150	252.2	4990	1150	225.6	6590	1150	209.2	7800
1151	252.2	5000	1151	225.6	6600	1151	210.2	7810
1152	252.2	4990	1152	225.6	6600	1152	211.1	7810
1153	252.2	4990	1153	225.6	6600	1153	212	7810
1154	252.3	4990	1154	225.6	6600	1154	212.8	7790
1155	252.3	4980	1155	225.6	6590	1155	213.5	7800
1156	252.7	5080	1156	225.6	6600	1156	214.2	7800
1157	253.1	5090	1157	225.6	6600	1157	214.9	7800
1158	253.4	5080	1158	225.6	6590	1158	215.5	7800
1159	253.7	5090	1159	225.6	6590	1159	216.1	7910
1160	254	5080	1160	225.7	6590	1160	216.6	7900
1161	254.5	5070	1161	225.7	6590	1161	217.1	7900
1162	255	5080	1162	225.7	6580	1162	217.6	7910
1163	255.4	5080	1163	225.6	6600	1163	218.1	7900
1164	255.9	5080	1164	225.6	6590	1164	218.5	7910
1165	256.3	5090	1165	225.6	6590	1165	218.9	7900
1166	256.7	5090	1166	225.6	6590	1166	219.2	7900
1167	257	5090	1167	225.6	6490	1167	219.6	7900
1168	257.4	5080	1168	225.6	6590	1168	219.9	7900
1169	257.9	5080	1169	225.6	6600	1169	220.3	7910
1170	258.4	5080	1170	225.6	6590	1170	220.6	7910
1171	258.9	5070	1171	225.6	6600	1171	221	7910
1172	259.3	5080	1172	225.6	6600	1172	221.3	7900
1173	259.6	5070	1173	225.6	6590	1173	221.6	7900
1174	260	5080	1174	225.7	6600	1174	221.8	8000
1175	260.4	5070	1175	225.6	6590	1175	222.1	8000
1176	260.8	5180	1176	225.7	6600	1176	222.3	8000
1177	261.1	5080	1177	225.7	6590	1177	222.5	7990
1178	261.5	5170	1178	225.7	6580	1178	222.7	7990
1179	261.8	5190	1179	225.7	6500	1179	222.7	8010
1180	262.1	5180	1180	225.7	6590	1180	222.8	8000
1181	262.5	5180	1181	225.7	6600	1181	222.9	8000
1182	262.8	5190	1182	225.6	6600	1182	223	8000
1183	263.2	5180	1183	225.6	6500	1183	223.1	7990
1184	263.6	5180	1184	225.7	6600	1184	223.3	8000
1185	263.9	5170	1185	225.7	6600	1185	223.3	8000
1186	264.2	5170	1186	225.7	6590	1186	223.5	8000
1187	264.5	5170	1187	225.6	6600	1187	223.6	7990
1188	264.8	5170	1188	225.7	6590	1188	223.7	8000
1189	265.1	5170	1189	225.6	6600	1189	223.8	7980
1190	265.4	5180	1190	225.7	6600	1190	223.9	7990
1191	265.7	5180	1191	225.7	6600	1191	224	8000
1192	265.9	5180	1192	225.6	6500	1192	224.1	8000
1193	266.1	5170	1193	225.7	6600	1193	224.2	8110
1194	266.4	5180	1194	225.7	6600	1194	224.3	8100
1195	266.6	5180	1195	225.6	6600	1195	224.4	8000
1196	266.8	5190	1196	225.7	6590	1196	224.5	7990

1197	267	5180	1197	225.7	6590	1197	224.6	8100
1198	267.1	5180	1198	225.7	6490	1198	224.5	8000
1199	267.3	5190	1199	225.7	6580	1199	224.6	8110
1200	267.5	5180	1200	225.7	6580	1200	224.7	8080
1201	267.6	5190	1201	225.7	6500	1201	224.7	8100
1202	267.8	5180	1202	225.7	6600	1202	224.8	8010
1203	267.9	5180	1203	225.7	6600	1203	224.9	8110
1204	268.1	5190	1204	225.7	6600	1204	224.9	8110
1205	268.3	5190	1205	225.8	6490	1205	225	8100
1206	268.4	5190	1206	225.8	6600	1206	225	8000
1207	268.5	5170	1207	225.8	6590	1207	225.1	8100
1208	268.7	5160	1208	225.8	6590	1208	225.1	8010
1209	268.8	5170	1209	225.8	6590	1209	225.1	8000
1210	268.9	5180	1210	225.8	6500	1210	225	8010
1211	269	5180	1211	225.8	6600	1211	224.9	8100
1212	269.1	5180	1212	225.8	6610	1212	224.8	8090
1213	269.2	5180	1213	225.8	6580	1213	224.8	8100
1214	269.3	5180	1214	225.8	6600	1214	224.8	8090
1215	269.5	5190	1215	225.8	6590	1215	224.8	8100
1216	269.6	5180	1216	225.8	6610	1216	224.8	8090
1217	269.7	5190	1217	225.8	6600	1217	224.8	8090
1218	269.8	5180	1218	225.8	6600	1218	224.8	8090
1219	270	5180	1219	225.8	6600	1219	224.9	8100
1220	270.1	5180	1220	225.8	6600	1220	224.9	8100
1221	270.2	5290	1221	225.7	6590	1221	224.8	8090
1222	270.3	5180	1222	225.8	6590	1222	224.9	8090
1223	270.4	5180	1223	225.8	6600	1223	224.9	8100
1224	270.5	5180	1224	225.7	6600	1224	224.8	8100
1225	270.6	5270	1225	225.8	6590	1225	224.9	8100
1226	270.7	5280	1226	225.8	6610	1226	224.9	8110
1227	270.7	5170	1227	225.8	6590	1227	224.9	8100
1228	270.9	5280	1228	225.8	6590	1228	224.9	8100
1229	270.9	5180	1229	225.8	6600	1229	224.9	8100
1230	271	5270	1230	225.8	6590	1230	224.9	8100
1231	271	5270	1231	225.8	6600	1231	225	8100
1232	271.1	5180	1232	225.8	6600	1232	225	8100
1233	271.2	5180	1233	225.8	6590	1233	225.1	8100
1234	271.3	5280	1234	225.8	6610	1234	225.1	8100
1235	271.3	5180	1235	225.8	6600	1235	225.2	8110
1236	271.4	5270	1236	225.8	6600	1236	225.2	8090
1237	271.5	5270	1237	225.8	6600	1237	225.2	8090
1238	271.5	5180	1238	225.8	6600	1238	225.2	8090
1239	271.6	5280	1239	225.8	6500	1239	225.2	8110
1240	271.7	5280	1240	225.8	6590	1240	225.1	8100
1241	271.7	5280	1241	225.8	6600	1241	225.1	8100
1242	271.8	5270	1242	225.8	6600	1242	225.1	8110
1243	271.8	5270	1243	225.8	6590	1243	225.1	8100
1244	271.9	5170	1244	225.8	6600	1244	225.1	8080
1245	272	5280	1245	225.8	6510	1245	225.1	8110
1246	272.1	5270	1246	225.8	6600	1246	225.1	8110

1247	272.2	5170	1247	225.8	6590	1247	225.2	8090
1248	272.3	5280	1248	225.8	6600	1248	225.2	8090
1249	272.4	5280	1249	225.8	6600	1249	225.2	8110
1250	272.4	5180	1250	225.9	6600	1250	225.2	8110
1251	272.5	5290	1251	225.9	6590	1251	225.2	8100
1252	272.6	5280	1252	225.9	6600	1252	225.2	8110
1253	272.7	5270	1253	225.9	6590	1253	225.2	8090
1254	272.7	5280	1254	225.9	6590	1254	225.2	8100
1255	272.8	5290	1255	225.9	6600	1255	225.3	8120
1256	272.8	5280	1256	225.9	6590	1256	225.3	8110
1257	272.9	5280	1257	225.9	6580	1257	225.3	8110
1258	273	5280	1258	225.9	6590	1258	225.3	8110
1259	273	5290	1259	225.9	6590	1259	225.3	8100
1260	273.1	5290	1260	226	6590	1260	225.4	8100
1261	273.2	5290	1261	225.9	6590	1261	225.4	8100
1262	273.2	5280	1262	225.9	6590	1262	225.4	8100
1263	273.3	5270	1263	225.9	6580	1263	225.5	8100
1264	273.3	5290	1264	225.9	6600	1264	225.5	8100
1265	273.4	5280	1265	225.9	6590	1265	225.5	8120
1266	273.5	5270	1266	225.9	6600	1266	225.6	8120
1267	273.5	5280	1267	225.9	6580	1267	225.6	8110
1268	273.5	5280	1268	225.9	6600	1268	225.6	8100
1269	273.5	5280	1269	225.9	6600	1269	225.7	8110
1270	273.7	5280	1270	225.9	6600	1270	225.7	8110
1271	273.7	5290	1271	225.9	6600	1271	225.7	8100
1272	273.8	5270	1272	225.9	6580	1272	225.8	8100
1273	273.9	5260	1273	226	6600	1273	225.8	8110
1274	273.9	5280	1274	225.9	6590	1274	225.8	8110
1275	274	5270	1275	226	6590	1275	225.8	8110
1276	274.1	5280	1276	226	6590	1276	225.9	8110
1277	274.1	5280	1277	226	6590	1277	225.9	8110
1278	274.1	5270	1278	226	6580	1278	225.9	8100
1279	274.2	5280	1279	226.1	6590	1279	225.9	8100
1280	274.2	5270	1280	226.1	6600	1280	225.9	8100
1281	274.3	5280	1281	226	6600	1281	225.9	8100
1282	274.4	5280	1282	226.1	6590	1282	226	8100
1283	274.4	5280	1283	226.1	6590	1283	226.1	8100
1284	274.4	5260	1284	226	6590	1284	226.1	8110
1285	274.4	5270	1285	226	6600	1285	226.1	8110
1286	274.5	5280	1286	226	6600	1286	226.2	8110
1287	274.5	5280	1287	226	6590	1287	226.2	8110
1288	274.6	5280	1288	226	6600	1288	226.2	8110
1289	274.6	5270	1289	226	6590	1289	226.3	8110
1290	274.7	5290	1290	226	6600	1290	226.3	8100
1291	274.8	5280	1291	226	6590	1291	226.4	8100
1292	274.8	5270	1292	226	6590	1292	226.4	8110
1293	274.9	5280	1293	226	6600	1293	226.4	8120
1294	274.9	5280	1294	226	6600	1294	226.3	8110
1295	274.9	5280	1295	226	6590	1295	226.3	8100
1296	275	5290	1296	226	6590	1296	226.3	8100

1297	275	5290	1297	226	6590	1297	226.2	8090
1298	275.1	5280	1298	226.1	6590	1298	226.2	8100
1299	275.1	5260	1299	226	6580	1299	226.2	8100
1300	275.1	5260	1300	226	6590	1300	226.2	8090
1301	275.1	5270	1301	226	6600	1301	226.2	8110
1302	275.2	5280	1302	226.1	6600	1302	226.2	8110
1303	275.3	5280	1303	226.1	6580	1303	226.1	8120
1304	275.3	5270	1304	226.1	6590	1304	226.2	8100
1305	275.4	5270	1305	226	6600	1305	226.1	8090
1306	275.4	5280	1306	226	6590	1306	226.1	8100
1307	275.5	5290	1307	226.1	6590	1307	226.1	8090
1308	275.5	5290	1308	226.1	6590	1308	226.2	8090
1309	275.5	5280	1309	226.1	6590	1309	226.1	8100
1310	275.6	5280	1310	226.1	6590	1310	226.1	8110
1311	275.6	5270	1311	226.1	6600	1311	226.1	8100
1312	275.6	5260	1312	226.1	6580	1312	226.1	8090
1313	275.7	5270	1313	226.1	6590	1313	226.1	8100
1314	275.7	5270	1314	226.1	6610	1314	226.2	8100
1315	275.8	5260	1315	226.1	6600	1315	226.2	8090
1316	275.8	5280	1316	226.1	6590	1316	226.2	8090
1317	275.9	5270	1317	226.1	6600	1317	226.2	8100
1318	275.9	5290	1318	226.1	6590	1318	226.2	8100
1319	276	5280	1319	226.1	6590	1319	226.2	8090
1320	276	5290	1320	226.1	6580	1320	226.2	8080
1321	276	5300	1321	226.1	6600	1321	226.2	8100
1322	276	5300	1322	226.1	6600	1322	226.3	8090
1323	276.1	5280	1323	226.2	6580	1323	226.3	8090
1324	276.1	5270	1324	226.2	6590	1324	226.3	8110
1325	276.1	5260	1325	226.2	6590	1325	226.3	8100
1326	276.1	5280	1326	226.2	6590	1326	226.3	8100
1327	276.2	5270	1327	226.2	6600	1327	226.3	8100
1328	276.2	5270	1328	226.2	6590	1328	226.3	8100
1329	276.3	5280	1329	226.2	6590	1329	226.3	8100
1330	276.3	5280	1330	226.1	6600	1330	226.3	8090
1331	276.3	5280	1331	226.1	6600	1331	226.3	8100
1332	276.4	5290	1332	226.1	6600	1332	226.4	8110
1333	276.4	5280	1333	226.2	6590	1333	226.3	8100
1334	276.4	5280	1334	226.2	6590	1334	226.3	8100
1335	276.5	5280	1335	226.2	6600	1335	226.4	8090
1336	276.5	5260	1336	226.2	6590	1336	226.4	8100
1337	276.5	5290	1337	226.2	6590	1337	226.4	8100
1338	276.6	5280	1338	226.2	6610	1338	226.4	8100
1339	276.6	5290	1339	226.2	6610	1339	226.5	8100
1340	276.6	5260	1340	226.2	6600	1340	226.5	8090
1341	276.7	5280	1341	226.2	6600	1341	226.5	8100
1342	276.7	5280	1342	226.2	6600	1342	226.5	8110
1343	276.7	5280	1343	226.2	6590	1343	226.5	8100
1344	276.8	5280	1344	226.2	6590	1344	226.4	8110
1345	276.8	5270	1345	226.2	6600	1345	226.3	8090
1346	276.8	5280	1346	226.2	6600	1346	226.3	8090

1347	276.9	5280	1347	226.2	6590	1347	226.2	8090
1348	276.9	5270	1348	226.2	6590	1348	226.2	8110
1349	276.9	5290	1349	226.2	6590	1349	226.2	8100
1350	276.9	5280	1350	226.2	6590	1350	226.1	8090
1351	276.9	5260	1351	226.2	6590	1351	226.1	8100
1352	277	5270	1352	226.3	6590	1352	226	8090
1353	277	5270	1353	226.3	6590	1353	226	8110
1354	277	5280	1354	226.3	6590	1354	226	8080
1355	277	5270	1355	226.3	6610	1355	226	8090
1356	277.1	5280	1356	226.3	6610	1356	225.9	8080
1357	277.1	5270	1357	226.3	6610	1357	226	8090
1358	277.1	5260	1358	226.3	6610	1358	225.9	8090
1359	277.1	5260	1359	226.2	6600	1359	225.9	8100
1360	277.2	5270	1360	226.2	6610	1360	225.9	8090
1361	277.2	5260	1361	226.2	6610	1361	225.8	8090
1362	277.3	5270	1362	226.2	6590	1362	225.9	8100
1363	277.3	5260	1363	226.3	6600	1363	225.9	8090
1364	277.3	5270	1364	226.3	6600	1364	225.9	8100
1365	277.3	5270	1365	226.2	6600	1365	225.9	8100
1366	277.3	5260	1366	226.3	6600	1366	225.9	8100
1367	277.3	5270	1367	226.3	6600	1367	225.9	8090
1368	277.3	5270	1368	226.3	6600	1368	225.9	8110
1369	277.4	5270	1369	226.3	6590	1369	225.8	8090
1370	277.4	5270	1370	226.3	6600	1370	225.9	8090
1371	277.5	5280	1371	226.3	6600	1371	225.9	8090
1372	277.5	5270	1372	226.3	6590	1372	225.8	8100
1373	277.5	5280	1373	226.2	6590	1373	225.9	8100
1374	277.6	5270	1374	226.2	6600	1374	225.9	8090
1375	277.6	5270	1375	226.3	6590	1375	225.9	8100
1376	277.6	5260	1376	226.2	6600	1376	225.9	8110
1377	277.7	5280	1377	226.2	6590	1377	225.9	8090
1378	277.7	5270	1378	226.2	6600	1378	225.9	8090
1379	277.6	5280	1379	226.2	6590	1379	225.9	8090
1380	277.6	5270	1380	226.2	6590	1380	225.9	8100
1381	277.6	5270	1381	226.2	6600	1381	225.9	8100
1382	277.7	5270	1382	226.2	6590	1382	225.9	8110
1383	277.7	5280	1383	226.2	6600	1383	225.9	8100
1384	277.7	5270	1384	226.3	6600	1384	225.9	8100
1385	277.7	5260	1385	226.3	6600	1385	225.9	8080
1386	277.7	5280	1386	226.3	6590	1386	225.9	8090
1387	277.6	5270	1387	226.3	6590	1387	225.9	8100
1388	277.6	5280	1388	226.3	6590	1388	225.9	8090
1389	277.6	5270	1389	226.2	6600	1389	225.9	8100
1390	277.5	5280	1390	226.3	6590	1390	225.9	8100
1391	277.5	5280	1391	226.3	6590	1391	225.9	8110
1392	277.5	5270	1392	226.3	6600	1392	225.9	8110
1393	277.4	5280	1393	226.3	6600	1393	225.9	8110
1394	277.4	5270	1394	226.3	6600	1394	225.9	8110
1395	277.4	5280	1395	226.3	6600	1395	225.9	8110
1396	277.5	5270	1396	226.3	6600	1396	225.9	8090

1397	277.4	5280	1397	226.3	6580	1397	225.9	8100
1398	277.5	5270	1398	226.3	6590	1398	225.9	8100
1399	277.4	5270	1399	226.3	6600	1399	225.9	8100
1400	277.4	5260	1400	226.3	6590	1400	225.9	8100
1401	277.4	5270	1401	226.3	6600	1401	226	8100
1402	277.4	5260	1402	226.3	6600	1402	226	8100
1403	277.4	5270	1403	226.3	6590	1403	226	8100
1404	277.4	5270	1404	226.3	6580	1404	226	8110
1405	277.5	5270	1405	226.3	6620	1405	226	8100
1406	277.4	5270	1406	226.4	6590	1406	226	8100
1407	277.5	5270	1407	226.4	6590	1407	226	8100
1408	277.4	5270	1408	226.4	6590	1408	226.1	8100
1409	277.4	5270	1409	226.4	6600	1409	226.1	8110
1410	277.4	5270	1410	226.4	6590	1410	226.1	8100
1411	277.4	5270	1411	226.4	6580	1411	226.1	8090
1412	277.4	5270	1412	226.4	6590	1412	226.1	8100
1413	277.4	5280	1413	226.4	6600	1413	226.1	8090
1414	277.5	5270	1414	226.5	6590	1414	226.2	8090
1415	277.4	5260	1415	226.5	6600	1415	226.2	8090
1416	277.5	5260	1416	226.4	6600	1416	226.1	8100
1417	277.5	5260	1417	226.4	6600	1417	226.1	8100
1418	277.5	5260	1418	226.5	6590	1418	226.2	8100
1419	277.5	5280	1419	226.4	6590	1419	226.1	8100
1420	277.5	5270	1420	226.4	6570	1420	226.2	8100
1421	277.5	5270	1421	226.4	6580	1421	226.2	8110
1422	277.5	5270	1422	226.4	6590	1422	226.2	8100
1423	277.5	5290	1423	226.4	6600	1423	226.1	8110
1424	277.5	5280	1424	226.4	6600	1424	226.1	8110
1425	277.6	5280	1425	226.4	6600	1425	226.1	8090
1426	277.6	5260	1426	226.4	6600	1426	226.2	8100
1427	277.6	5280	1427	226.4	6590	1427	226.1	8090
1428	277.6	5270	1428	226.4	6600	1428	226.2	8090
1429	277.6	5280	1429	226.4	6600	1429	226.2	8100
1430	277.6	5270	1430	226.4	6610	1430	226.2	8100
1431	277.6	5270	1431	226.5	6600	1431	226.2	8090
1432	277.6	5280	1432	226.4	6600	1432	226.2	8100
1433	277.6	5270	1433	226.5	6590	1433	226.2	8100
1434	277.5	5270	1434	226.4	6600	1434	226.2	8100
1435	277.5	5280	1435	226.4	6590	1435	226.2	8090
1436	277.4	5270	1436	226.5	6590	1436	226.2	8090
1437	277.3	5280	1437	226.5	6580	1437	226.2	8100
1438	277.3	5260	1438	226.5	6590	1438	226.3	8090
1439	277.2	5260	1439	226.5	6600	1439	226.3	8100
1440	277.1	5270	1440	226.5	6590	1440	226.3	8090
1441	277.1	5270	1441	226.5	6600	1441	226.3	8100
1442	277	5280	1442	226.5	6590	1442	226.3	8110
1443	277	5270	1443	226.5	6600	1443	226.3	8100
1444	277	5280	1444	226.5	6590	1444	226.3	8090
1445	276.9	5260	1445	226.4	6600	1445	226.3	8100
1446	276.9	5270	1446	226.5	6600	1446	226.3	8090

1447	276.9	5280	1447	226.4	6590	1447	226.3	8090
1448	276.9	5280	1448	226.5	6590	1448	226.3	8100
1449	276.9	5270	1449	226.5	6590	1449	226.3	8080
1450	276.9	5270	1450	226.5	6580	1450	226.4	8100
1451	276.9	5280	1451	226.5	6580	1451	226.4	8110
1452	276.9	5290	1452	226.5	6590	1452	226.4	8100
1453	276.9	5280	1453	226.5	6590	1453	226.4	8100
1454	276.8	5290	1454	226.5	6610	1454	226.4	8100
1455	276.8	5280	1455	226.5	6590	1455	226.4	8090
1456	276.8	5270	1456	226.5	6590	1456	226.4	8090
1457	276.8	5280	1457	226.5	6590	1457	226.4	8100
1458	276.8	5280	1458	226.5	6590	1458	226.4	8080
1459	276.7	5270	1459	226.5	6580	1459	226.4	8090
1460	276.7	5280	1460	226.5	6600	1460	226.4	8100
1461	276.7	5280	1461	226.5	6590	1461	226.4	8100
1462	276.6	5280	1462	226.5	6600	1462	226.4	8090
1463	276.6	5280	1463	226.5	6590	1463	226.4	8090
1464	276.6	5280	1464	226.5	6600	1464	226.5	8100
1465	276.6	5270	1465	226.5	6590	1465	226.5	8100
1466	276.6	5260	1466	226.5	6590	1466	226.5	8090
1467	276.6	5280	1467	226.5	6600	1467	226.5	8090
1468	276.6	5270	1468	226.5	6590	1468	226.5	8100
1469	276.6	5280	1469	226.5	6580	1469	226.5	8090
1470	276.7	5280	1470	226.5	6600	1470	226.5	8090
1471	276.6	5280	1471	226.5	6590	1471	226.5	8090
1472	276.6	5270	1472	226.5	6590	1472	226.5	8090
1473	276.6	5280	1473	226.5	6600	1473	226.5	8100
1474	276.6	5270	1474	226.5	6590	1474	226.5	8100
1475	276.6	5270	1475	226.5	6580	1475	226.5	8090
1476	276.6	5270	1476	226.5	6590	1476	226.5	8100
1477	276.7	5270	1477	226.5	6590	1477	226.5	8100
1478	276.7	5270	1478	226.5	6600	1478	226.5	8110
1479	276.7	5270	1479	226.5	6580	1479	226.5	8100
1480	276.6	5270	1480	226.5	6570	1480	226.5	8090
1481	276.6	5260	1481	226.6	6580	1481	226.5	8100
1482	276.6	5260	1482	226.5	6590	1482	226.6	8100
1483	276.6	5260	1483	226.5	6590	1483	226.6	8100
1484	276.7	5270	1484	226.5	6580	1484	226.5	8090
1485	276.7	5270	1485	226.5	6580	1485	226.6	8090
1486	276.7	5280	1486	226.5	6590	1486	226.6	8100
1487	276.7	5270	1487	226.5	6600	1487	226.6	8100
1488	276.7	5280	1488	226.5	6590	1488	226.6	8100
1489	276.8	5280	1489	226.6	6580	1489	226.6	8090
1490	276.8	5280	1490	226.5	6570	1490	226.6	8090
1491	276.8	5260	1491	226.5	6580	1491	226.6	8080
1492	276.8	5270	1492	226.5	6580	1492	226.6	8080
1493	276.9	5270	1493	226.5	6590	1493	226.6	8090
1494	276.8	5270	1494	226.5	6590	1494	226.6	8100
1495	276.9	5280	1495	226.5	6590	1495	226.6	8090
1496	276.8	5270	1496	226.4	6580	1496	226.6	8090

1497	276.8	5270	1497	226.4	6590	1497	226.6	8090
1498	276.8	5270	1498	226.5	6600	1498	226.6	8100
1499	276.9	5270	1499	226.5	6590	1499	226.6	8090
1500	276.8	5270	1500	226.4	6580	1500	226.6	8100
1501	276.8	5280	1501	226.5	6580	1501	226.6	8110
1502	276.8	5270	1502	226.5	6590	1502	226.6	8090
1503	276.8	5270	1503	226.5	6600	1503	226.7	8110
1504	276.8	5290	1504	226.5	6590	1504	226.7	8100
1505	276.8	5260	1505	226.5	6590	1505	226.7	8090
1506	276.8	5270	1506	226.4	6590	1506	226.7	8090
1507	276.8	5260	1507	226.5	6590	1507	226.7	8100
1508	276.8	5270	1508	226.5	6590	1508	226.7	8100
1509	276.8	5270	1509	226.5	6590	1509	226.7	8100
1510	276.8	5270	1510	226.4	6580	1510	226.7	8080
1511	276.8	5280	1511	226.5	6590	1511	226.7	8100
1512	276.8	5280	1512	226.4	6590	1512	226.7	8100
1513	276.9	5280	1513	226.5	6580	1513	226.7	8100
1514	276.9	5260	1514	226.5	6580	1514	226.7	8110
1515	276.8	5270	1515	226.5	6600	1515	226.7	8090
1516	276.8	5270	1516	226.5	6590	1516	226.7	8100
1517	276.8	5270	1517	226.5	6590	1517	226.7	8090
1518	276.8	5270	1518	226.5	6600	1518	226.7	8090
1519	276.8	5280	1519	226.5	6590	1519	226.6	8090
1520	276.8	5280	1520	226.6	6600	1520	226.6	8090
1521	276.8	5270	1521	226.6	6590	1521	226.6	8090
1522	276.9	5270	1522	226.6	6590	1522	226.7	8090
1523	276.9	5260	1523	226.6	6590	1523	226.7	8090
1524	276.9	5280	1524	226.6	6590	1524	226.7	8090
1525	276.8	5280	1525	226.6	6600	1525	226.7	8100
1526	276.8	5280	1526	226.6	6590	1526	226.7	8090
1527	276.9	5290	1527	226.5	6600	1527	226.8	8090
1528	276.9	5270	1528	226.5	6590	1528	226.8	8090
1529	276.9	5270	1529	226.6	6590	1529	226.8	8100
1530	276.9	5270	1530	226.6	6590	1530	226.8	8100
1531	276.9	5270	1531	226.6	6610	1531	226.8	8090
1532	276.9	5280	1532	226.6	6600	1532	226.8	8090
1533	277	5280	1533	226.5	6590	1533	226.8	8090
1534	277	5280	1534	226.6	6600	1534	226.8	8090
1535	276.9	5280	1535	226.5	6600	1535	226.8	8090
1536	277	5270	1536	226.6	6590	1536	226.8	8100
1537	277	5280	1537	226.6	6600	1537	226.8	8080
1538	277	5260	1538	226.6	6580	1538	226.8	8090
1539	277	5270	1539	226.6	6590	1539	226.8	8090
1540	277	5280	1540	226.6	6590	1540	226.8	8090
1541	276.9	5280	1541	226.5	6590	1541	226.9	8090
1542	276.9	5280	1542	226.5	6600	1542	226.9	8090
1543	276.9	5270	1543	226.5	6590	1543	226.9	8100
1544	277	5260	1544	226.5	6580	1544	226.9	8090
1545	277	5280	1545	226.5	6600	1545	226.9	8100
1546	277	5270	1546	226.5	6580	1546	226.9	8100

1547	277	5280	1547	226.5	6590	1547	226.9	8100
1548	277	5280	1548	226.5	6600	1548	226.9	8100
1549	277	5290	1549	226.4	6600	1549	226.9	8100
1550	277	5270	1550	226.5	6590	1550	226.9	8090
1551	276.9	5270	1551	226.5	6590	1551	226.8	8100
1552	277	5270	1552	226.5	6600	1552	226.9	8090
1553	277	5270	1553	226.5	6580	1553	226.9	8090
1554	277	5270	1554	226.5	6590	1554	226.9	8090
1555	277	5270	1555	226.5	6580	1555	226.9	8090
1556	277.1	5270	1556	226.5	6580	1556	226.9	8090
1557	277.1	5280	1557	226.5	6580	1557	226.9	8090
1558	277.1	5290	1558	226.5	6590	1558	226.7	8090
1559	277.1	5280	1559	226.5	6600	1559	226.7	8100
1560	277.1	5280	1560	226.5	6600	1560	226.6	8090
1561	277.1	5280	1561	226.5	6600	1561	226.6	8100
1562	277.1	5280	1562	226.5	6590	1562	226.5	8100
1563	277.1	5270	1563	226.5	6590	1563	226.5	8090
1564	277.1	5270	1564	226.5	6590	1564	226.4	8100
1565	277.1	5270	1565	226.5	6600	1565	226.4	8100
1566	277.1	5280	1566	226.5	6590	1566	226.4	8090
1567	277.1	5260	1567	226.5	6600	1567	226.3	8090
1568	277.1	5270	1568	226.5	6590	1568	226.3	8090
1569	277	5270	1569	226.5	6580	1569	226.2	8090
1570	277	5270	1570	226.5	6580	1570	226.2	8100
1571	277	5270	1571	226.5	6590	1571	226.1	8110
1572	277	5280	1572	226.5	6590	1572	226.1	8090
1573	277	5260	1573	226.5	6580	1573	226.1	8090
1574	277	5260	1574	226.5	6580	1574	226.1	8080
1575	277	5270	1575	226.5	6580	1575	226.1	8090
1576	277	5280	1576	226.5	6590	1576	226	8090
1577	277	5260	1577	226.5	6590	1577	226	8110
1578	277	5280	1578	226.5	6590	1578	226	8090
1579	277.1	5290	1579	226.4	6600	1579	225.9	8090
1580	277.1	5270	1580	226.5	6580	1580	226	8090
1581	277.1	5280	1581	226.5	6600	1581	226	8100
1582	277.1	5270	1582	226.4	6600	1582	226	8090
1583	277.1	5260	1583	226.4	6600	1583	225.9	8090
1584	277.1	5250	1584	226.4	6600	1584	225.9	8080
1585	277.2	5270	1585	226.5	6600	1585	225.8	8100
1586	277.1	5270	1586	226.5	6590	1586	225.8	8100
1587	277.2	5270	1587	226.5	6580	1587	225.9	8100
1588	277.2	5270	1588	226.5	6600	1588	225.9	8110
1589	277.2	5260	1589	226.5	6590	1589	225.8	8090
1590	277.2	5260	1590	226.5	6580	1590	225.8	8100
1591	277.2	5260	1591	226.5	6590	1591	225.9	8090
1592	277.2	5270	1592	226.5	6600	1592	225.9	8100
1593	277.3	5270	1593	226.5	6590	1593	225.9	8080
1594	277.3	5270	1594	226.5	6590	1594	225.9	8080
1595	277.3	5280	1595	226.5	6590	1595	225.8	8080
1596	277.3	5280	1596	226.5	6600	1596	225.9	8080

1597	277.3	5280	1597	226.5	6580	1597	225.8	8090
1598	277.4	5270	1598	226.5	6590	1598	225.8	8090
1599	277.4	5280	1599	226.5	6590	1599	225.8	8110
1600	277.4	5280	1600	226.5	6570	1600	225.7	8100
1601	277.5	5270	1601	226.5	6570	1601	225.7	8100
1602	277.5	5280	1602	226.5	6570	1602	225.7	8090
1603	277.5	5280	1603	226.5	6590	1603	225.7	8080
1604	277.5	5270	1604	226.5	6590	1604	225.7	8090
1605	277.5	5260	1605	226.5	6580	1605	225.7	8090
1606	277.5	5260	1606	226.6	6600	1606	225.7	8090
1607	277.5	5270	1607	226.5	6590	1607	225.7	8090
1608	277.6	5260	1608	226.5	6590	1608	225.7	8100
1609	277.5	5260	1609	226.5	6590	1609	225.7	8090
1610	277.5	5260	1610	226.5	6590	1610	225.7	8090
1611	277.5	5270	1611	226.5	6590	1611	225.7	8100
1612	277.5	5260	1612	226.5	6580	1612	225.7	8090
1613	277.5	5260	1613	226.5	6590	1613	225.7	8090
1614	277.6	5270	1614	226.5	6600	1614	225.7	8090
1615	277.6	5260	1615	226.5	6590	1615	225.7	8100
1616	277.6	5270	1616	226.5	6600	1616	225.7	8090
1617	277.6	5280	1617	226.5	6580	1617	225.7	8090
1618	277.5	5280	1618	226.5	6590	1618	225.7	8090
1619	277.5	5280	1619	226.5	6580	1619	225.7	8090
1620	277.6	5260	1620	226.5	6600	1620	225.7	8090
1621	277.6	5260	1621	226.5	6600	1621	225.6	8100
1622	277.6	5270	1622	226.5	6590	1622	225.6	8100
1623	277.6	5270	1623	226.5	6590	1623	225.6	8100
1624	277.6	5270	1624	226.5	6590	1624	225.7	8100
1625	277.6	5250	1625	226.5	6590	1625	225.6	8100
1626	277.6	5280	1626	226.5	6600	1626	225.7	8100
1627	277.6	5260	1627	226.5	6590	1627	225.7	8100
1628	277.6	5260	1628	226.5	6590	1628	225.7	8090
1629	277.6	5270	1629	226.5	6590	1629	225.7	8100
1630	277.5	5260	1630	226.5	6600	1630	225.7	8090
1631	277.5	5270	1631	226.5	6590	1631	225.7	8080
1632	277.5	5280	1632	226.5	6590	1632	225.7	8090
1633	277.5	5280	1633	226.6	6600	1633	225.7	8090
1634	277.5	5270	1634	226.6	6600	1634	225.7	8100
1635	277.5	5270	1635	226.6	6590	1635	225.7	8080
1636	277.6	5260	1636	226.5	6590	1636	225.7	8090
1637	277.6	5260	1637	226.5	6600	1637	225.7	8100
1638	277.6	5260	1638	226.5	6590	1638	225.7	8090
1639	277.6	5260	1639	226.5	6600	1639	225.7	8090
1640	277.7	5250	1640	226.6	6600	1640	225.7	8080
1641	277.8	5260	1641	226.5	6600	1641	225.7	8080
1642	277.7	5270	1642	226.5	6600	1642	225.6	8100
1643	277.8	5280	1643	226.5	6590	1643	225.6	8090
1644	277.8	5270	1644	226.5	6600	1644	225.6	8090
1645	277.8	5260	1645	226.5	6600	1645	225.6	8090
1646	277.8	5270	1646	226.5	6590	1646	225.6	8090

1647	277.8	5260	1647	226.5	6580	1647	225.6	8080
1648	277.8	5260	1648	226.5	6590	1648	225.6	8100
1649	277.8	5280	1649	226.5	6590	1649	225.6	8090
1650	277.8	5270	1650	226.5	6580	1650	225.6	8090
1651	277.8	5280	1651	226.6	6580	1651	225.6	8090
1652	277.8	5260	1652	226.6	6580	1652	225.6	8100
1653	277.8	5270	1653	226.6	6590	1653	225.6	8110
1654	277.8	5270	1654	226.5	6590	1654	225.6	8110
1655	277.8	5260	1655	226.6	6580	1655	225.5	8100
1656	277.8	5280	1656	226.6	6580	1656	225.5	8100
1657	277.7	5280	1657	226.6	6580	1657	225.5	8100
1658	277.8	5250	1658	226.6	6590	1658	225.5	8090
1659	277.7	5270	1659	226.6	6580	1659	225.5	8080
1660	277.8	5260	1660	226.6	6580	1660	225.5	8090
1661	277.7	5260	1661	226.6	6590	1661	225.6	8100
1662	277.7	5260	1662	226.6	6580	1662	225.6	8090
1663	277.8	5260	1663	226.6	6580	1663	225.6	8100
1664	277.8	5270	1664	226.6	6590	1664	225.6	8100
1665	277.7	5270	1665	226.6	6580	1665	225.6	8100
1666	277.7	5260	1666	226.6	6580	1666	225.6	8110
1667	277.7	5270	1667	226.5	6580	1667	225.6	8100
1668	277.8	5250	1668	226.6	6580	1668	225.6	8100
1669	277.8	5260	1669	226.6	6580	1669	225.6	8100
1670	277.8	5260	1670	226.6	6600	1670	225.6	8100
1671	277.8	5260	1671	226.5	6590	1671	225.6	8100
1672	277.8	5260	1672	226.6	6580	1672	225.6	8090
1673	277.9	5270	1673	226.5	6580	1673	225.6	8100
1674	277.9	5270	1674	226.5	6580	1674	225.6	8100
1675	277.9	5260	1675	226.5	6590	1675	225.6	8100
1676	277.9	5250	1676	226.5	6600	1676	225.6	8090
1677	277.9	5270	1677	226.5	6600	1677	225.6	8090
1678	277.9	5260	1678	226.5	6590	1678	225.5	8100
1679	277.9	5270	1679	226.5	6580	1679	225.5	8090
1680	277.8	5260	1680	226.5	6590	1680	225.5	8090
1681	277.8	5260	1681	226.5	6580	1681	225.5	8100
1682	277.8	5270	1682	226.5	6580	1682	225.5	8090
1683	277.7	5270	1683	226.5	6560	1683	225.5	8100
1684	277.8	5260	1684	226.5	6600	1684	225.5	8090
1685	277.8	5250	1685	226.5	6580	1685	225.5	8080
1686	277.8	5270	1686	226.5	6580	1686	225.5	8090
1687	277.8	5270	1687	226.6	6590	1687	225.5	8090
1688	277.9	5260	1688	226.6	6580	1688	225.5	8100
1689	277.9	5260	1689	226.6	6590	1689	225.5	8100
1690	277.9	5270	1690	226.6	6590	1690	225.5	8080
1691	277.9	5270	1691	226.6	6590	1691	225.5	8100
1692	278	5270	1692	226.6	6590	1692	225.6	8080
1693	277.9	5260	1693	226.6	6590	1693	225.6	8080
1694	277.9	5280	1694	226.6	6580	1694	225.5	8100
1695	277.9	5260	1695	226.6	6590	1695	225.5	8090
1696	277.9	5260	1696	226.6	6580	1696	225.5	8090

1697	277.9	5260	1697	226.6	6580	1697	225.6	8090
1698	277.9	5260	1698	226.6	6590	1698	225.6	8090
1699	277.9	5260	1699	226.6	6600	1699	225.6	8100
1700	277.9	5270	1700	226.6	6600	1700	225.6	8090
1701	277.9	5260	1701	226.7	6580	1701	225.6	8090
1702	277.9	5270	1702	226.7	6590	1702	225.5	8090
1703	277.9	5270	1703	226.6	6600	1703	225.5	8100
1704	277.9	5270	1704	226.7	6590	1704	225.5	8090
1705	277.9	5270	1705	226.7	6600	1705	225.5	8100
1706	277.8	5260	1706	226.7	6600	1706	225.4	8100
1707	277.8	5270	1707	226.7	6600	1707	225.4	8100
1708	277.9	5270	1708	226.7	6600	1708	225.4	8100
1709	277.9	5260	1709	226.7	6600	1709	225.4	8100
1710	277.9	5260	1710	226.7	6590	1710	225.4	8110
1711	277.9	5260	1711	226.7	6600	1711	225.4	8080
1712	277.9	5250	1712	226.7	6600	1712	225.4	8090
1713	277.9	5270	1713	226.7	6600	1713	225.5	8090
1714	277.9	5260	1714	226.8	6590	1714	225.5	8090
1715	277.9	5250	1715	226.7	6590	1715	225.5	8080
1716	277.9	5240	1716	226.7	6590	1716	225.5	8100
1717	278	5260	1717	226.7	6590	1717	225.5	8100
1718	278	5250	1718	226.7	6590	1718	225.5	8090
1719	277.9	5260	1719	226.7	6600	1719	225.5	8090
1720	277.9	5260	1720	226.6	6590	1720	225.5	8090
1721	277.9	5270	1721	226.6	6570	1721	225.5	8080
1722	277.9	5280	1722	226.6	6600	1722	225.5	8090
1723	277.9	5270	1723	226.7	6580	1723	225.5	8080
1724	277.9	5280	1724	226.7	6590	1724	225.5	8090
1725	277.9	5260	1725	226.7	6600	1725	225.5	8080
1726	277.9	5260	1726	226.7	6590	1726	225.5	8090
1727	277.9	5260	1727	226.7	6590	1727	225.5	8090
1728	278	5280	1728	226.7	6580	1728	225.5	8090
1729	278	5260	1729	226.6	6580	1729	225.5	8090
1730	277.9	5270	1730	226.6	6590	1730	225.6	8090
1731	277.9	5260	1731	226.7	6600	1731	225.6	8090
1732	277.9	5270	1732	226.6	6590	1732	225.5	8100
1733	277.9	5260	1733	226.6	6600	1733	225.5	8100
1734	277.9	5270	1734	226.6	6590	1734	225.5	8090
1735	277.9	5270	1735	226.6	6600	1735	225.5	8090
1736	277.9	5260	1736	226.6	6600	1736	225.6	8110
1737	277.9	5260	1737	226.7	6580	1737	225.6	8090
1738	277.9	5270	1738	226.7	6590	1738	225.6	8090
1739	277.9	5270	1739	226.6	6590	1739	225.5	8080
1740	277.9	5270	1740	226.6	6590	1740	225.5	8090
1741	277.9	5270	1741	226.6	6590	1741	225.5	8090
1742	277.9	5270	1742	226.6	6590	1742	225.5	8080
1743	277.9	5260	1743	226.6	6590	1743	225.5	8080
1744	278	5260	1744	226.6	6580	1744	225.5	8090
1745	278	5270	1745	226.6	6590	1745	225.5	8080
1746	277.9	5270	1746	226.6	6580	1746	225.5	8090

1747	277.9	5260	1747	226.6	6580	1747	225.6	8090
1748	278	5260	1748	226.6	6580	1748	225.5	8090
1749	278	5270	1749	226.6	6590	1749	225.6	8090
1750	278	5260	1750	226.6	6580	1750	225.5	8090
1751	278	5270	1751	226.6	6580	1751	225.5	8090
1752	278	5260	1752	226.7	6580	1752	225.5	8090
1753	278	5270	1753	226.6	6580	1753	225.5	8090
1754	278.1	5260	1754	226.6	6590	1754	225.5	8110
1755	278.1	5250	1755	226.7	6580	1755	225.5	8080
1756	278	5260	1756	226.7	6580	1756	225.5	8100
1757	278	5270	1757	226.7	6590	1757	225.5	8100
1758	278	5260	1758	226.7	6580	1758	225.5	8090
1759	278.1	5270	1759	226.7	6590	1759	225.5	8090
1760	278	5250	1760	226.7	6580	1760	225.5	8090
1761	278.1	5260	1761	226.7	6600	1761	225.5	8100
1762	278.1	5260	1762	226.7	6590	1762	225.5	8090
1763	278	5250	1763	226.7	6590	1763	225.5	8100
1764	278	5270	1764	226.7	6590	1764	225.5	8100
1765	278	5270	1765	226.7	6580	1765	225.5	8090
1766	278.1	5250	1766	226.7	6590	1766	225.5	8090
1767	278.1	5250	1767	226.7	6590	1767	225.5	8090
1768	278.1	5260	1768	226.7	6590	1768	225.4	8090
1769	278.1	5260	1769	226.7	6600	1769	225.5	8090
1770	278.1	5270	1770	226.7	6600	1770	225.4	8100
1771	278.1	5250	1771	226.6	6580	1771	225.4	8100
1772	278.1	5270	1772	226.7	6580	1772	225.5	8090
1773	278.1	5270	1773	226.6	6590	1773	225.5	8090
1774	278.1	5270	1774	226.7	6590	1774	225.4	8100
1775	278.1	5270	1775	226.6	6580	1775	225.4	8100
1776	278.1	5270	1776	226.6	6580	1776	225.4	8100
1777	278.1	5280	1777	226.6	6590	1777	225.4	8090
1778	278	5270	1778	226.6	6590	1778	225.4	8090
1779	278	5260	1779	226.6	6570	1779	225.5	8090
1780	278.1	5160	1780	226.7	6580	1780	225.5	8080
1781	278.1	5270	1781	226.7	6580	1781	225.5	8080
1782	278	5280	1782	226.7	6590	1782	225.5	8090
1783	278	5270	1783	226.7	6590	1783	225.5	8090
1784	278	5270	1784	226.7	6590	1784	225.5	8110
1785	278	5260	1785	226.7	6580	1785	225.5	8090
1786	278.1	5260	1786	226.7	6600	1786	225.5	8100
1787	278.1	5270	1787	226.7	6590	1787	225.5	8090
1788	278.1	5270	1788	226.7	6610	1788	225.5	8090
1789	278.1	5260	1789	226.7	6590	1789	225.5	8090
1790	278.1	5260	1790	226.7	6590	1790	225.5	8090
1791	278.1	5270	1791	226.7	6590	1791	225.4	8080
1792	278.1	5260	1792	226.7	6590	1792	225.5	8090
1793	278	5260	1793	226.7	6600	1793	225.4	8080
1794	278.1	5250	1794	226.7	6590	1794	225.4	8090
1795	278.1	5260	1795	226.7	6600	1795	225.4	8090
1796	278.1	5270	1796	226.7	6590	1796	225.4	8090

1797	278.1	5260	1797	226.7	6600	1797	225.4	8090
1798	278.1	5280	1798	226.7	6590	1798	225.5	8100
1799	278.1	5270	1799	226.8	6590	1799	225.5	8080
1800	278.1	5280	1800	226.8	6590	1800	225.6	8090
1801	278.1	5260	1801	226.8	6600	1801	225.5	8080
1802	278.1	5270	1802	226.8	6590	1802	225.5	8090
1803	278.1	5280	1803	226.8	6590	1803	225.5	8080
1804	278.1	5260	1804	226.9	6600	1804	225.6	8090
1805	278.1	5250	1805	226.9	6600	1805	225.6	8090
1806	278	5260	1806	226.8	6580	1806	225.6	8090
1807	278.1	5250	1807	226.8	6600	1807	225.6	8090
1808	278.1	5260	1808	226.8	6610	1808	225.6	8090
1809	278.1	5260	1809	226.8	6600	1809	225.6	8090
1810	278	5260	1810	226.8	6610	1810	225.6	8080
1811	278	5260	1811	226.8	6600	1811	225.6	8080
1812	278	5260	1812	226.8	6610	1812	225.6	8100
1813	278.1	5260	1813	226.7	6600	1813	225.6	8100
1814	278.1	5260	1814	226.7	6580	1814	225.6	8090
1815	278	5260	1815	226.7	6590	1815	225.6	8090
1816	278.1	5260	1816	226.7	6580	1816	225.7	8100
1817	278	5270	1817	226.7	6590	1817	225.7	8080
1818	278	5260	1818	226.7	6590	1818	225.7	8090
1819	278	5260	1819	226.7	6590	1819	225.7	8090
1820	277.9	5270	1820	226.7	6590	1820	225.7	8090
1821	277.9	5260	1821	226.7	6590	1821	225.7	8100
1822	277.9	5270	1822	226.7	6590	1822	225.7	8090
1823	277.8	5270	1823	226.7	6600	1823	225.7	8100
1824	277.7	5260	1824	226.7	6590	1824	225.7	8110
1825	277.7	5250	1825	226.7	6590	1825	225.7	8110
1826	277.7	5280	1826	226.7	6600	1826	225.7	8090
1827	277.6	5270	1827	226.7	6590	1827	225.7	8100
1828	277.6	5260	1828	226.7	6600	1828	225.6	8090
1829	277.5	5270	1829	226.7	6590	1829	225.6	8090
1830	277.5	5170	1830	226.7	6590	1830	225.7	8100
1831	277.5	5270	1831	226.7	6590	1831	225.7	8100
1832	277.4	5260	1832	226.7	6590	1832	225.6	8100
1833	277.4	5260	1833	226.7	6590	1833	225.7	8100
1834	277.4	5260	1834	226.8	6600	1834	225.7	8100
1835	277.4	5270	1835	226.8	6600	1835	225.7	8100
1836	277.4	5270	1836	226.8	6600	1836	225.8	8100
1837	277.4	5260	1837	226.8	6600	1837	225.8	8090
1838	277.4	5160	1838	226.7	6590	1838	225.8	8080
1839	277.3	5170	1839	226.7	6600	1839	225.8	8100
1840	277.3	5260	1840	226.7	6590	1840	225.7	8080
1841	277.3	5270	1841	226.7	6590	1841	225.7	8090
1842	277.3	5270	1842	226.7	6590	1842	225.7	8090
1843	277.3	5270	1843	226.7	6590	1843	225.7	8090
1844	277.2	5280	1844	226.7	6600	1844	225.7	8090
1845	277.2	5170	1845	226.7	6600	1845	225.7	8100
1846	277.2	5260	1846	226.7	6580	1846	225.7	8100

1847	277.2	5270	1847	226.7	6590	1847	225.7	8100
1848	277.2	5260	1848	226.7	6590	1848	225.7	8080
1849	277.2	5160	1849	226.7	6600	1849	225.8	8090
1850	277.2	5160	1850	226.6	6590	1850	225.8	8090
1851	277.2	5260	1851	226.6	6590	1851	225.7	8090
1852	277.1	5150	1852	226.6	6590	1852	225.7	8100
1853	277.1	5260	1853	226.6	6580	1853	225.7	8100
1854	277.1	5260	1854	226.6	6580	1854	225.7	8100
1855	277.1	5260	1855	226.6	6590	1855	225.7	8100
1856	277.1	5260	1856	226.7	6590	1856	225.8	8090
1857	277.1	5160	1857	226.7	6590	1857	225.8	8090
1858	277.1	5180	1858	226.7	6580	1858	225.8	8090
1859	277.1	5160	1859	226.7	6590	1859	225.8	8090
1860	277.1	5160	1860	226.7	6600	1860	225.7	8090
1861	277.1	5270	1861	226.7	6590	1861	225.8	8090
1862	277.1	5270	1862	226.7	6590	1862	225.8	8080
1863	277.1	5160	1863	226.7	6590	1863	225.8	8100
1864	277.1	5160	1864	226.7	6590	1864	225.8	8100
1865	277.1	5260	1865	226.7	6600	1865	225.8	8090
1866	277.1	5170	1866	226.7	6610	1866	225.7	8100
1867	277.1	5160	1867	226.6	6600	1867	225.7	8080
1868	277.1	5160	1868	226.7	6600	1868	225.7	8080
1869	277.1	5170	1869	226.7	6590	1869	225.7	8090
1870	277	5170	1870	226.7	6600	1870	225.7	8090
1871	276.9	5160	1871	226.6	6600	1871	225.7	8110
1872	276.9	5160	1872	226.7	6610	1872	225.7	8080
1873	276.9	5170	1873	226.7	6600	1873	225.7	8090
1874	277	5160	1874	226.7	6590	1874	225.7	8090
1875	277	5160	1875	226.7	6600	1875	225.7	8090
1876	277	5260	1876	226.7	6580	1876	225.7	8080
1877	277	5260	1877	226.9	6590	1877	225.6	8090
1878	277	5150	1878	227	6580	1878	225.7	8080
1879	277	5160	1879	227.3	6580	1879	225.7	8090
1880	277	5160	1880	227.5	6580	1880	225.7	8090
1881	277	5160	1881	227.9	6580	1881	225.7	8100
1882	277	5150	1882	228.2	6600	1882	225.7	8090
1883	277	5150	1883	228.6	6700	1883	225.7	8100
1884	277	5160	1884	229	6690	1884	225.7	8090
1885	277	5170	1885	229.4	6680	1885	225.7	8090
1886	277	5160	1886	229.8	6700	1886	225.8	8090
1887	277	5150	1887	230.1	6680	1887	225.8	8090
1888	277	5240	1888	230.5	6690	1888	225.8	8090
1889	277	5170	1889	230.8	6690	1889	225.8	8090
1890	277	5160	1890	231.1	6680	1890	225.8	8090
1891	277	5250	1891	231.4	6680	1891	225.8	8090
1892	277	5250	1892	231.7	6700	1892	225.8	8090
1893	277	5160	1893	232	6700	1893	225.8	8080
1894	277	5150	1894	232.2	6680	1894	225.8	8080
1895	277	5160	1895	232.4	6690	1895	225.8	8090
1896	277	5160	1896	232.7	6680	1896	225.7	8080

1897	276.9	5160	1897	232.9	6680	1897	225.7	8090
1898	277	5160	1898	233.1	6690	1898	225.8	8080
1899	277	5270	1899	233.4	6680	1899	225.8	8090
1900	277	5270	1900	233.7	6690	1900	225.8	8090
1901	276.9	5170	1901	234.1	6680	1901	225.8	8090
1902	276.9	5160	1902	234.4	6690	1902	225.8	8080
1903	276.9	5170	1903	234.6	6690	1903	225.8	8090
1904	276.9	5170	1904	234.9	6680	1904	225.8	8090
1905	276.9	5170	1905	235.1	6680	1905	225.8	8090
1906	276.9	5150	1906	235.4	6690	1906	225.8	8110
1907	276.9	5160	1907	235.7	6680	1907	225.8	8090
1908	276.9	5270	1908	236	6800	1908	225.8	8090
1909	276.9	5160	1909	236.3	6780	1909	225.8	8090
1910	276.9	5260	1910	236.6	6780	1910	225.8	8090
1911	276.9	5170	1911	237	6770	1911	225.9	8090
1912	276.8	5170	1912	237.4	6780	1912	225.9	8110
1913	276.8	5150	1913	237.7	6780	1913	225.9	8080
1914	276.8	5150	1914	238	6790	1914	225.8	8100
1915	276.8	5160	1915	238.3	6780	1915	225.8	8090
1916	276.8	5160	1916	238.5	6790	1916	225.8	8080
1917	276.8	5160	1917	238.8	6790	1917	225.8	8090
1918	276.8	5170	1918	239.1	6770	1918	225.8	8090
1919	276.8	5170	1919	239.3	6780	1919	225.8	8080
1920	276.8	5160	1920	239.5	6780	1920	225.8	8100
1921	276.8	5170	1921	239.7	6790	1921	225.7	8100
1922	276.8	5260	1922	239.9	6790	1922	225.8	8090
1923	276.8	5150	1923	240	6800	1923	225.8	8090
1924	276.8	5160	1924	240.3	6780	1924	225.8	8100
1925	276.8	5160	1925	240.4	6780	1925	225.8	8100
1926	276.8	5270	1926	240.6	6780	1926	225.7	8100
1927	276.8	5250	1927	240.7	6780	1927	225.7	8090
1928	276.8	5150	1928	240.9	6780	1928	225.7	8090
1929	276.8	5150	1929	241.1	6790	1929	225.7	8100
1930	276.8	5150	1930	241.2	6800	1930	225.7	8080
1931	276.8	5150	1931	241.4	6790	1931	225.7	8110
1932	276.8	5150	1932	241.5	6780	1932	225.7	8100
1933	276.7	5150	1933	241.6	6780	1933	225.7	8080
1934	276.7	5150	1934	241.7	6790	1934	225.7	8100
1935	276.7	5260	1935	241.8	6790	1935	225.7	8100
1936	276.7	5150	1936	241.9	6880	1936	225.7	8100
1937	276.7	5170	1937	242	6780	1937	225.8	8110
1938	276.7	5160	1938	242.1	6880	1938	225.8	8110
1939	276.7	5150	1939	242.2	6880	1939	225.8	8100
1940	276.7	5260	1940	242.3	6880	1940	225.8	8100
1941	276.7	5150	1941	242.4	6880	1941	225.7	8090
1942	276.6	5160	1942	242.6	6780	1942	225.7	8080
1943	276.6	5160	1943	242.7	6890	1943	225.7	8090
1944	276.6	5140	1944	242.7	6900	1944	225.7	8090
1945	276.6	5160	1945	242.9	6880	1945	225.7	8090
1946	276.6	5160	1946	243	6880	1946	225.7	8090

1947	276.6	5160	1947	243.1	6880	1947	225.7	8100
1948	276.7	5170	1948	243.2	6890	1948	225.8	8090
1949	276.7	5160	1949	243.3	6890	1949	225.8	8090
1950	276.7	5160	1950	243.4	6880	1950	225.8	8080
1951	276.7	5150	1951	243.5	6880	1951	225.8	8090
1952	276.7	5160	1952	243.6	6890	1952	225.8	8090
1953	276.6	5160	1953	243.7	6870	1953	225.8	8100
1954	276.7	5170	1954	243.8	6890	1954	225.8	8090
1955	276.6	5160	1955	243.9	6880	1955	225.8	8090
1956	276.6	5160	1956	244	6890	1956	225.8	8090
1957	276.6	5150	1957	244	6880	1957	225.8	8100
1958	276.6	5170	1958	244.1	6880	1958	225.8	8090
1959	276.6	5160	1959	244.2	6880	1959	225.8	8190
1960	276.6	5160	1960	244.3	6880	1960	225.8	8100
1961	276.6	5160	1961	244.4	6880	1961	225.8	8090
1962	276.6	5160	1962	244.4	6870	1962	225.8	8090
1963	276.6	5170	1963	244.5	6890	1963	225.7	8090
1964	276.6	5160	1964	244.5	6870	1964	225.7	8200
1965	276.6	5160	1965	244.6	6880	1965	225.7	8080
1966	276.6	5140	1966	244.6	6880	1966	225.7	8090
1967	276.6	5150	1967	244.7	6880	1967	225.7	8100
1968	276.6	5170	1968	244.8	6870	1968	225.7	8190
1969	276.5	5170	1969	244.8	6880	1969	225.7	8190
1970	276.5	5170	1970	244.9	6870	1970	225.7	8090
1971	276.6	5160	1971	245	6880	1971	225.7	8100
1972	276.5	5160	1972	245.1	6880	1972	225.7	8200
1973	276.5	5160	1973	245.2	6880	1973	225.7	8090
1974	276.5	5160	1974	245.2	6870	1974	225.7	8100
1975	276.5	5170	1975	245.3	6880	1975	225.7	8090
1976	276.6	5170	1976	245.3	6870	1976	225.7	8090
1977	276.6	5160	1977	245.4	6880	1977	225.7	8190
1978	276.6	5160	1978	245.5	6870	1978	225.7	8190
1979	276.6	5160	1979	245.6	6870	1979	225.7	8180
1980	276.6	5160	1980	245.6	6880	1980	225.7	8200
1981	276.6	5160	1981	245.7	6890	1981	225.7	8180
1982	276.6	5160	1982	245.7	6880	1982	225.7	8200
1983	276.5	5140	1983	245.8	6880	1983	225.8	8090
1984	276.5	5160	1984	245.9	6880	1984	225.7	8090
1985	276.5	5160	1985	245.9	6880	1985	225.7	8090
1986	276.5	5160	1986	246	6870	1986	225.7	8090
1987	276.5	5150	1987	246.1	6880	1987	225.7	8190
1988	276.5	5160	1988	246.2	6880	1988	225.7	8090
1989	276.5	5170	1989	246.2	6860	1989	225.6	8190
1990	276.6	5160	1990	246.3	6860	1990	225.7	8090
1991	276.5	5160	1991	246.3	6860	1991	225.7	8100
1992	276.5	5170	1992	246.4	6880	1992	225.7	8080
1993	276.5	5150	1993	246.4	6880	1993	225.7	8090
1994	276.5	5170	1994	246.5	6880	1994	225.7	8190
1995	276.5	5160	1995	246.5	6870	1995	225.7	8180
1996	276.5	5170	1996	246.5	6870	1996	225.7	8090

1997	276.5	5170	1997	246.6	6880	1997	225.7	8100
1998	276.5	5170	1998	246.7	6890	1998	225.7	8090
1999	276.5	5160	1999	246.7	6980	1999	225.7	8090
2000	276.5	5160	2000	246.8	6970	2000	225.7	8090
2001	276.5	5170	2001	246.9	6880	2001	225.7	8190
2002	276.5	5160	2002	246.9	6880	2002	225.7	8080
2003	276.5	5160	2003	247	6880	2003	225.7	8200
2004	276.5	5160	2004	247.1	6980	2004	225.7	8190
2005	276.6	5160	2005	247.1	6880	2005	225.7	8190
2006	276.5	5160	2006	247.1	6880	2006	225.7	8100
2007	276.5	5160	2007	247.2	6890	2007	225.7	8090
2008	276.5	5170	2008	247.2	6880	2008	225.8	8100
2009	276.5	5170	2009	247.3	6990	2009	225.7	8100
2010	276.6	5160	2010	247.3	6880	2010	225.7	8080
2011	276.6	5260	2011	247.4	6870	2011	225.7	8200
2012	276.5	5160	2012	247.4	6890	2012	225.8	8190
2013	276.5	5170	2013	247.4	6970	2013	225.8	8090
2014	276.5	5170	2014	247.5	6990	2014	225.7	8190
2015	276.5	5170	2015	247.5	6890	2015	225.8	8190
2016	276.5	5280	2016	247.5	6900	2016	225.7	8200
2017	276.4	5170	2017	247.6	6990	2017	225.7	8190
2018	276.4	5170	2018	247.6	6890	2018	225.8	8190
2019	276.4	5160	2019	247.7	6980	2019	225.8	8100
2020	276.4	5170	2020	247.7	6980	2020	225.8	8100
2021	276.4	5160	2021	247.8	6890	2021	225.7	8200
2022	276.4	5170	2022	247.8	6980	2022	225.7	8200
2023	276.4	5170	2023	247.8	6880	2023	225.7	8190
2024	276.5	5160	2024	247.8	6980	2024	225.7	8200
2025	276.5	5270	2025	247.9	6980	2025	225.8	8100
2026	276.5	5160	2026	247.9	6980	2026	225.8	8200
2027	276.4	5260	2027	247.9	6970	2027	225.8	8180
2028	276.4	5170	2028	248	6970	2028	225.8	8090
2029	276.4	5160	2029	248.1	6960	2029	225.8	8210
2030	276.4	5270	2030	248.1	6980	2030	225.8	8090
2031	276.5	5160	2031	248.2	6970	2031	225.8	8100
2032	276.5	5170	2032	248.2	6970	2032	225.8	8200
2033	276.4	5170	2033	248.2	6960	2033	225.8	8100
2034	276.5	5150	2034	248.3	6970	2034	225.8	8190
2035	276.4	5170	2035	248.3	6970	2035	225.8	8090
2036	276.5	5160	2036	248.3	6980	2036	225.8	8100
2037	276.5	5170	2037	248.4	6980	2037	225.8	8100
2038	276.5	5170	2038	248.4	6980	2038	225.8	8200
2039	276.5	5170	2039	248.5	6970	2039	225.8	8200
2040	276.5	5180	2040	248.5	6980	2040	225.8	8200
2041	276.5	5170	2041	248.5	6980	2041	225.7	8090
2042	276.5	5160	2042	248.6	6980	2042	225.8	8100
2043	276.5	5150	2043	248.6	6960	2043	225.8	8100
2044	276.6	5160	2044	248.6	6960	2044	225.7	8110
2045	276.6	5160	2045	248.7	6980	2045	225.8	8200
2046	276.6	5170	2046	248.7	6980	2046	225.8	8110

2047	276.6	5170	2047	248.8	6980	2047	225.8	8200
2048	276.6	5170	2048	248.8	6980	2048	225.8	8190
2049	276.6	5180	2049	248.8	6980	2049	225.8	8200
2050	276.6	5170	2050	248.8	6970	2050	225.8	8100
2051	276.6	5160	2051	248.9	6980	2051	225.8	8200
2052	276.6	5160	2052	248.9	6990	2052	225.8	8190
2053	276.6	5260	2053	249	6980	2053	225.8	8080
2054	276.6	5270	2054	249	6970	2054	225.8	8090
2055	276.6	5170	2055	249	6980	2055	225.8	8180
2056	276.6	5180	2056	249	6980	2056	225.8	8080
2057	276.6	5160	2057	249.1	6990	2057	225.8	8190
2058	276.6	5260	2058	249.1	6980	2058	225.8	8190
2059	276.6	5260	2059	249.2	6990	2059	225.8	8200
2060	276.6	5150	2060	249.2	6990	2060	225.8	8100
2061	276.6	5160	2061	249.3	6970	2061	225.8	8080
2062	276.6	5160	2062	249.3	6980	2062	225.8	8190
2063	276.6	5170	2063	249.3	6980	2063	225.8	8190
2064	276.6	5160	2064	249.4	6980	2064	225.8	8190
2065	276.6	5150	2065	249.4	6970	2065	225.8	8190
2066	276.7	5160	2066	249.4	6980	2066	225.8	8190
2067	276.7	5150	2067	249.5	6980	2067	225.8	8190
2068	276.6	5150	2068	249.5	6970	2068	225.8	8200
2069	276.6	5150	2069	249.5	6980	2069	225.8	8180
2070	276.6	5160	2070	249.6	6990	2070	225.8	8090
2071	276.6	5160	2071	249.6	6980	2071	225.8	8100
2072	276.5	5160	2072	249.6	6990	2072	225.8	8090
2073	276.5	5150	2073	249.6	6980	2073	225.8	8180
2074	276.5	5160	2074	249.6	6980	2074	225.9	8100
2075	276.5	5160	2075	249.7	6980	2075	225.8	8100
2076	276.6	5160	2076	249.7	6970	2076	225.8	8190
2077	276.6	5170	2077	249.7	6970	2077	225.8	8190
2078	276.6	5160	2078	249.8	6980	2078	225.9	8200
2079	276.6	5160	2079	249.8	6970	2079	225.9	8180
2080	276.6	5160	2080	249.8	6980	2080	225.9	8190
2081	276.6	5160	2081	249.8	6980	2081	225.9	8080
2082	276.6	5150	2082	249.8	6970	2082	225.9	8100
2083	276.6	5160	2083	249.9	6970	2083	225.8	8090
2084	276.6	5140	2084	249.9	6980	2084	225.9	8190
2085	276.6	5160	2085	249.9	6970	2085	225.9	8190
2086	276.6	5170	2086	250	6980	2086	225.8	8190
2087	276.6	5150	2087	250	6990	2087	225.8	8190
2088	276.7	5160	2088	250.1	6970	2088	225.8	8190
2089	276.7	5170	2089	250.1	6970	2089	225.8	8190
2090	276.7	5160	2090	250.1	6970	2090	225.8	8200
2091	276.7	5150	2091	250.1	6980	2091	225.8	8090
2092	276.7	5150	2092	250.1	6960	2092	225.8	8200
2093	276.7	5140	2093	250.2	6970	2093	225.8	8190
2094	276.6	5140	2094	250.2	6970	2094	225.8	8190
2095	276.6	5160	2095	250.1	6960	2095	225.8	8190
2096	276.6	5150	2096	250.2	6990	2096	225.8	8190

2097	276.6	5150	2097	250.2	6980	2097	225.8	8180
2098	276.6	5160	2098	250.2	6980	2098	225.8	8190
2099	276.7	5160	2099	250.3	6980	2099	225.9	8190
2100	276.7	5160	2100	250.3	6980	2100	225.9	8180
2101	276.7	5150	2101	250.4	6980	2101	225.9	8190
2102	276.7	5250	2102	250.4	6970	2102	225.9	8180
2103	276.7	5160	2103	250.4	6970	2103	225.9	8210
2104	276.7	5150	2104	250.4	6980	2104	225.9	8190
2105	276.7	5150	2105	250.5	6980	2105	226	8190
2106	276.7	5150	2106	250.5	6970	2106	226	8180
2107	276.7	5260	2107	250.6	6980	2107	226	8190
2108	276.7	5170	2108	250.6	6980	2108	226	8190
2109	276.7	5170	2109	250.6	6980	2109	225.9	8190
2110	276.7	5160	2110	250.6	6980	2110	226	8190
2111	276.7	5160	2111	250.6	6980	2111	225.9	8190
2112	276.7	5150	2112	250.7	6980	2112	225.9	8190
2113	276.7	5150	2113	250.7	6980	2113	225.9	8190
2114	276.7	5260	2114	250.7	6980	2114	225.9	8190
2115	276.7	5160	2115	250.8	6970	2115	225.9	8190
2116	276.7	5160	2116	250.8	6990	2116	225.9	8200
2117	276.7	5160	2117	250.8	6980	2117	225.9	8200
2118	276.8	5160	2118	250.9	6980	2118	225.9	8190
2119	276.8	5160	2119	250.9	6970	2119	225.8	8190
2120	276.8	5150	2120	250.9	6980	2120	225.8	8180
2121	276.8	5150	2121	250.9	6980	2121	225.8	8190
2122	276.7	5150	2122	250.9	6980	2122	225.9	8170
2123	276.7	5150	2123	250.9	6960	2123	225.8	8190
2124	276.7	5160	2124	250.9	6980	2124	225.9	8180
2125	276.7	5160	2125	250.9	6990	2125	225.9	8190
2126	276.7	5150	2126	250.9	6980	2126	225.9	8190
2127	276.7	5150	2127	250.9	6980	2127	225.9	8200
2128	276.7	5160	2128	250.9	6970	2128	225.9	8190
2129	276.7	5160	2129	251	6990	2129	225.9	8190
2130	276.7	5170	2130	251	6990	2130	225.9	8190
2131	276.8	5260	2131	251	6960	2131	225.9	8190
2132	276.8	5150	2132	251	6970	2132	225.9	8200
2133	276.8	5150	2133	251	6980	2133	225.8	8200
2134	276.8	5160	2134	251	6980	2134	225.8	8200
2135	276.8	5250	2135	251	6970	2135	225.8	8200
2136	276.8	5160	2136	251.1	6970	2136	225.8	8180
2137	276.8	5160	2137	251.1	6980	2137	225.8	8190
2138	276.8	5170	2138	251.1	6970	2138	225.8	8190
2139	276.8	5150	2139	251.2	6970	2139	225.8	8190
2140	276.8	5170	2140	251.2	6980	2140	225.8	8190
2141	276.8	5160	2141	251.2	6970	2141	225.8	8180
2142	276.7	5160	2142	251.3	6970	2142	225.8	8200
2143	276.7	5170	2143	251.3	6980	2143	225.8	8190
2144	276.8	5160	2144	251.3	6980	2144	225.8	8090
2145	276.8	5160	2145	251.3	6970	2145	225.8	8100
2146	276.8	5170	2146	251.3	6980	2146	225.8	8180

2147	276.8	5160	2147	251.3	6970	2147	225.8	8090
2148	276.8	5260	2148	251.3	6980	2148	225.8	8190
2149	276.8	5250	2149	251.3	6980	2149	225.8	8090
2150	276.8	5160	2150	251.3	6980	2150	225.8	8190
2151	276.8	5160	2151	251.4	6980	2151	225.9	8200
2152	276.8	5160	2152	251.4	6970	2152	225.9	8190
2153	276.9	5150	2153	251.4	6980	2153	225.8	8090
2154	276.8	5160	2154	251.4	6980	2154	225.8	8090
2155	276.8	5160	2155	251.4	6980	2155	225.8	8100
2156	276.8	5150	2156	251.5	6970	2156	225.8	8190
2157	276.8	5160	2157	251.5	6980	2157	225.8	8200
2158	276.8	5150	2158	251.5	6980	2158	225.8	8200
2159	276.8	5150	2159	251.5	6970	2159	225.8	8200
2160	276.8	5150	2160	251.5	6970	2160	225.8	8190
2161	276.9	5160	2161	251.6	6980	2161	225.8	8200
2162	276.9	5160	2162	251.6	6970	2162	225.8	8200
2163	276.9	5150	2163	251.6	6980	2163	225.8	8190
2164	276.8	5170	2164	251.6	6970	2164	225.8	8200
2165	276.9	5160	2165	251.6	6950	2165	225.8	8190
2166	276.9	5160	2166	251.7	6960	2166	225.8	8200
2167	276.9	5170	2167	251.6	6970	2167	225.8	8090
2168	276.9	5170	2168	251.7	6970	2168	225.8	8200
2169	276.9	5150	2169	251.7	6970	2169	225.8	8200
2170	276.9	5170	2170	251.7	6980	2170	225.8	8200
2171	276.9	5260	2171	251.8	6970	2171	225.8	8200
2172	276.9	5170	2172	251.8	6970	2172	225.8	8200
2173	276.9	5160	2173	251.8	6980	2173	225.8	8190
2174	276.9	5170	2174	251.8	6980	2174	225.8	8190
2175	276.9	5150	2175	251.8	6980	2175	225.8	8210
2176	276.9	5150	2176	251.8	6970	2176	225.8	8190
2177	276.9	5150	2177	251.8	6950	2177	225.9	8190
2178	276.9	5150	2178	251.8	6980	2178	225.9	8190
2179	276.9	5160	2179	251.9	6970	2179	225.8	8210
2180	276.9	5150	2180	251.9	6980	2180	225.8	8200
2181	276.9	5240	2181	251.9	6970	2181	225.8	8200
2182	276.9	5150	2182	251.9	6980	2182	225.9	8190
2183	276.9	5160	2183	251.9	6970	2183	225.9	8180
2184	276.9	5160	2184	251.9	6980	2184	225.9	8190
2185	276.9	5150	2185	251.9	6980	2185	225.9	8200
2186	276.9	5150	2186	251.9	6970	2186	225.9	8190
2187	276.9	5160	2187	252	6970	2187	225.9	8200
2188	276.9	5250	2188	252	6960	2188	225.9	8200
2189	276.9	5150	2189	252	6970	2189	225.9	8200
2190	276.9	5260	2190	252	6970	2190	225.9	8200
2191	276.9	5150	2191	252.1	6980	2191	225.8	8190
2192	276.9	5150	2192	252.1	6970	2192	225.9	8210
2193	276.9	5150	2193	252.1	6950	2193	225.8	8210
2194	276.9	5260	2194	252.1	6960	2194	225.8	8200
2195	276.8	5170	2195	252.1	6960	2195	225.9	8200
2196	276.8	5150	2196	252.2	6970	2196	225.9	8180

2197	276.9	5150	2197	252.2	6970	2197	225.9	8200
2198	276.9	5150	2198	252.2	6970	2198	225.9	8200
2199	276.9	5150	2199	252.2	6970	2199	225.9	8190
2200	276.9	5160	2200	252.2	6970	2200	225.9	8200
2201	276.9	5150	2201	252.2	6960	2201	225.9	8200
2202	276.9	5140	2202	252.2	6970	2202	225.9	8190
2203	276.9	5150	2203	252.2	6980	2203	225.9	8190
2204	276.9	5250	2204	252.2	6970	2204	225.9	8200
2205	276.8	5150	2205	252.2	6980	2205	225.9	8190
2206	276.9	5250	2206	252.2	6970	2206	225.9	8200
2207	276.9	5150	2207	252.3	6980	2207	225.9	8210
2208	276.9	5140	2208	252.3	6970	2208	225.9	8190
2209	276.9	5140	2209	252.3	6980	2209	225.9	8210
2210	276.9	5150	2210	252.3	6960	2210	225.9	8200
2211	276.9	5140	2211	252.4	6970	2211	225.9	8190
2212	276.9	5160	2212	252.4	6970	2212	225.9	8210
2213	276.9	5160	2213	252.4	6970	2213	225.9	8190
2214	276.9	5250	2214	252.4	6970	2214	226	8190
2215	276.9	5150	2215	252.4	6970	2215	225.9	8190
2216	276.9	5150	2216	252.4	6980	2216	225.9	8200
2217	276.9	5160	2217	252.5	6960	2217	226	8190
2218	276.9	5160	2218	252.5	6980	2218	226	8190
2219	276.9	5160	2219	252.5	6970	2219	226	8200
2220	276.8	5150	2220	252.5	6980	2220	226	8200
2221	276.8	5150	2221	252.5	6970	2221	226	8200
2222	276.8	5140	2222	252.6	6970	2222	226	8200
2223	276.9	5150	2223	252.6	6980	2223	226	8190
2224	276.9	5160	2224	252.6	6970	2224	226	8190
2225	276.9	5160	2225	252.6	6980	2225	226	8200
2226	277	5150	2226	252.6	6970	2226	226	8200
2227	277	5150	2227	252.6	6970	2227	226	8200
2228	277	5150	2228	252.6	6970	2228	226	8190
2229	276.9	5150	2229	252.6	6980	2229	226	8190
2230	276.9	5150	2230	252.5	6960	2230	226	8190
2231	276.9	5150	2231	252.4	6980	2231	225.9	8200
2232	276.9	5160	2232	252.4	6960	2232	225.9	8190
2233	276.9	5160	2233	252.4	6970	2233	225.9	8200
2234	276.9	5150	2234	252.3	6970	2234	225.9	8190
2235	276.9	5160	2235	252.3	6980	2235	225.9	8190
2236	276.9	5160	2236	252.2	6970	2236	225.9	8190
2237	276.9	5150	2237	252.2	6970	2237	225.9	8190
2238	276.9	5240	2238	252.1	6960	2238	225.9	8200
2239	276.9	5150	2239	252.1	6980	2239	225.9	8200
2240	276.9	5150	2240	252.1	6970	2240	225.9	8190
2241	277	5260	2241	252.1	6980	2241	225.9	8210
2242	277	5260	2242	252	6980	2242	225.9	8200
2243	277.1	5260	2243	252	6970	2243	225.9	8190
2244	277.1	5160	2244	252	6980	2244	225.9	8190
2245	277	5150	2245	251.9	6970	2245	225.9	8200
2246	277	5160	2246	251.9	6990	2246	225.9	8190

2247	277	5160	2247	252	6970	2247	225.9	8190
2248	277	5150	2248	251.9	6980	2248	225.9	8200
2249	277	5140	2249	251.9	6990	2249	225.9	8200
2250	277	5140	2250	251.8	6980	2250	225.8	8210
2251	276.9	5150	2251	251.8	6980	2251	225.8	8190
2252	276.9	5150	2252	251.8	6980	2252	225.8	8200
2253	276.9	5150	2253	251.8	6960	2253	225.9	8190
2254	276.9	5150	2254	251.9	6960	2254	225.9	8200
2255	276.8	5160	2255	251.8	6980	2255	225.9	8200
2256	276.8	5140	2256	251.9	6970	2256	225.9	8210
2257	276.8	5150	2257	251.9	6970	2257	225.9	8200
2258	276.8	5150	2258	251.9	6960	2258	225.9	8200
2259	276.8	5150	2259	251.9	6980	2259	225.9	8200
2260	276.8	5160	2260	251.9	6980	2260	225.9	8190
2261	276.8	5150	2261	251.9	6980	2261	225.8	8190
2262	276.8	5150	2262	251.9	6960	2262	225.8	8190
2263	276.8	5150	2263	251.9	6980	2263	225.8	8200
2264	276.8	5160	2264	251.9	6970	2264	225.8	8190
2265	276.8	5140	2265	251.9	6980	2265	225.8	8200
2266	276.8	5140	2266	251.9	6970	2266	225.8	8190
2267	276.8	5150	2267	251.9	6970	2267	225.8	8200
2268	276.9	5160	2268	251.9	6970	2268	225.8	8200
2269	276.9	5140	2269	251.8	6980	2269	225.8	8200
2270	276.9	5150	2270	251.9	6970	2270	225.8	8200
2271	276.9	5150	2271	251.9	6970	2271	225.8	8200
2272	276.9	5150	2272	251.9	6980	2272	225.8	8190
2273	276.8	5150	2273	251.9	6970	2273	225.8	8200
2274	276.9	5160	2274	251.9	6980	2274	225.7	8190
2275	276.8	5150	2275	251.9	6970	2275	225.7	8190
2276	276.8	5150	2276	251.9	6970	2276	225.7	8200
2277	276.8	5150	2277	251.9	6970	2277	225.7	8200
2278	276.8	5150	2278	251.9	6980	2278	225.8	8190
2279	276.8	5150	2279	251.8	6970	2279	225.8	8180
2280	276.8	5130	2280	251.9	6970	2280	225.8	8190
2281	276.8	5140	2281	251.9	6970	2281	225.8	8200
2282	276.9	5140	2282	251.8	6960	2282	225.8	8180
2283	276.9	5150	2283	251.9	6980	2283	225.8	8190
2284	276.9	5160	2284	251.9	6980	2284	225.8	8200
2285	276.8	5150	2285	251.8	6970	2285	225.8	8190
2286	276.8	5150	2286	251.9	6980	2286	225.8	8180
2287	276.8	5140	2287	251.9	6980	2287	225.8	8190
2288	276.8	5150	2288	251.9	6960	2288	225.8	8190
2289	276.8	5150	2289	251.9	6970	2289	225.8	8190
2290	276.8	5150	2290	251.9	6970	2290	225.8	8190
2291	276.7	5140	2291	251.9	6980	2291	225.8	8190
2292	276.8	5150	2292	251.9	6970	2292	225.8	8190
2293	276.7	5150	2293	251.9	6970	2293	225.8	8200
2294	276.7	5160	2294	251.8	6980	2294	225.8	8210
2295	276.8	5140	2295	251.9	6980	2295	225.9	8200
2296	276.8	5150	2296	251.9	6970	2296	225.8	8180

2297	276.7	5150	2297	251.9	6970	2297	225.9	8180
2298	276.7	5160	2298	251.9	6970	2298	225.9	8190
2299	276.7	5160	2299	251.9	6970	2299	225.9	8190
2300	276.8	5150	2300	251.9	6970	2300	225.9	8190
2301	276.8	5140	2301	251.9	6970	2301	225.9	8180
2302	276.9	5150	2302	251.9	6980	2302	225.9	8180
2303	276.9	5150	2303	251.9	6990	2303	225.9	8180
2304	276.9	5150	2304	252	6970	2304	225.9	8200
2305	276.8	5150	2305	252	6980	2305	225.9	8190
2306	276.8	5150	2306	251.9	6970	2306	225.9	8190
2307	276.8	5150	2307	251.9	6980	2307	225.9	8190
2308	276.8	5150	2308	251.9	6980	2308	225.9	8190
2309	276.8	5150	2309	252	6980	2309	225.9	8200
2310	276.8	5150	2310	252	6960	2310	225.9	8190
2311	276.8	5150	2311	252	6970	2311	225.9	8200
2312	276.8	5150	2312	252	6970	2312	225.9	8190
2313	276.8	5150	2313	251.9	6980	2313	225.9	8190
2314	276.9	5160	2314	252	6970	2314	225.9	8190
2315	276.9	5140	2315	252	6970	2315	225.8	8190
2316	276.9	5140	2316	252	6960	2316	225.8	8190
2317	276.9	5150	2317	252	6970	2317	225.8	8180
2318	276.9	5140	2318	252	6970	2318	225.9	8190
2319	276.9	5150	2319	252	6970	2319	225.9	8190
2320	276.9	5150	2320	252	6970	2320	225.9	8200
2321	276.9	5140	2321	252	6970	2321	225.9	8200
2322	276.9	5140	2322	252.1	6990	2322	225.9	8180
2323	276.9	5140	2323	252.1	6980	2323	225.9	8200
2324	276.9	5140	2324	252	6980	2324	225.9	8190
2325	276.9	5150	2325	252	6980	2325	225.9	8190
2326	276.9	5150	2326	252	6980	2326	225.9	8190
2327	276.9	5150	2327	252	6960	2327	225.9	8180
2328	276.9	5160	2328	252	7000	2328	225.9	8200
2329	276.9	5150	2329	252	6980	2329	225.9	8190
2330	276.8	5140	2330	252	6980	2330	225.9	8200
2331	276.8	5150	2331	252	6970	2331	225.9	8190
2332	276.8	5160	2332	252	6970	2332	225.9	8200
2333	276.8	5160	2333	252	6980	2333	225.9	8200
2334	276.8	5170	2334	252	6970	2334	225.9	8200
2335	276.8	5150	2335	252	6980	2335	225.9	8190
2336	276.8	5150	2336	252	6970	2336	225.9	8200
2337	276.8	5140	2337	252	6970	2337	225.9	8190
2338	276.8	5130	2338	252	6980	2338	225.9	8190
2339	276.8	5140	2339	252	6990	2339	225.9	8200
2340	276.8	5150	2340	252	6970	2340	225.9	8200
2341	276.8	5140	2341	252	6980	2341	225.9	8190
2342	276.8	5140	2342	252	6970	2342	225.8	8190
2343	276.8	5160	2343	252	6980	2343	225.8	8200
2344	276.8	5150	2344	252.1	6980	2344	225.9	8180
2345	276.8	5150	2345	252.1	6970	2345	225.8	8200
2346	276.8	5150	2346	252.1	6990	2346	225.8	8190

2347	276.8	5160	2347	252.2	6970	2347	225.9	8190
2348	276.8	5160	2348	252.1	6970	2348	225.9	8190
2349	276.8	5150	2349	252.1	6980	2349	225.9	8200
2350	276.8	5150	2350	252.1	6980	2350	225.9	8200
2351	276.8	5150	2351	252.1	6970	2351	225.9	8200
2352	276.8	5140	2352	252.1	6970	2352	225.9	8190
2353	276.8	5160	2353	252.1	6970	2353	225.9	8200
2354	276.8	5150	2354	252	6980	2354	225.9	8210
2355	276.8	5150	2355	252	6980	2355	225.9	8210
2356	276.7	5150	2356	252	6980	2356	225.9	8210
2357	276.8	5150	2357	252	6970	2357	225.9	8220
2358	276.8	5140	2358	252	6970	2358	225.8	8210
2359	276.8	5130	2359	252	6970	2359	225.9	8190
2360	276.9	5160	2360	252.1	6970	2360	225.8	8200
2361	276.8	5150	2361	252.1	6980	2361	225.8	8200
2362	276.9	5150	2362	252.1	6970	2362	225.8	8190
2363	276.9	5150	2363	252.1	6960	2363	225.8	8180
2364	276.9	5160	2364	252.1	6970	2364	225.8	8200
2365	276.9	5150	2365	252.1	6970	2365	225.8	8200
2366	276.8	5160	2366	252.1	6960	2366	225.8	8190
2367	276.8	5160	2367	252.1	6980	2367	225.8	8190
2368	276.8	5150	2368	252.1	6990	2368	225.8	8190
2369	276.8	5170	2369	252.1	6980	2369	225.8	8200
2370	276.8	5160	2370	252.1	6980	2370	225.8	8190
2371	276.8	5150	2371	252.1	6970	2371	225.8	8190
2372	276.8	5160	2372	252.1	6980	2372	225.8	8190
2373	276.8	5160	2373	252.1	6970	2373	225.8	8170
2374	276.7	5160	2374	252.2	6980	2374	225.9	8190
2375	276.8	5140	2375	252.2	6980	2375	225.9	8180
2376	276.7	5150	2376	252.2	6960	2376	225.9	8190
2377	276.7	5150	2377	252.2	6970	2377	225.9	8190
2378	276.7	5150	2378	252.2	6960	2378	225.9	8190
2379	276.7	5150	2379	252.2	6970	2379	225.9	8200
2380	276.7	5170	2380	252.2	6960	2380	225.9	8200
2381	276.7	5160	2381	252.2	6960	2381	225.9	8210
2382	276.7	5150	2382	252.3	6980	2382	225.9	8190
2383	276.7	5160	2383	252.3	6970	2383	225.9	8200
2384	276.7	5150	2384	252.2	6970	2384	225.9	8200
2385	276.8	5150	2385	252.2	6970	2385	225.9	8180
2386	276.8	5160	2386	252.2	6970	2386	225.9	8180
2387	276.8	5150	2387	252.3	6980	2387	225.9	8190
2388	276.8	5160	2388	252.3	6980	2388	225.9	8180
2389	276.8	5150	2389	252.2	6970	2389	225.9	8190
2390	276.8	5160	2390	252.3	6980	2390	225.9	8180
2391	276.8	5160	2391	252.3	6960	2391	225.8	8190
2392	276.8	5150	2392	252.3	6970	2392	225.8	8180
2393	276.8	5140	2393	252.3	6960	2393	225.9	8180
2394	276.8	5150	2394	252.3	6970	2394	225.9	8180
2395	276.8	5170	2395	252.3	6960	2395	225.8	8180
2396	276.7	5160	2396	252.3	6970	2396	225.8	8190

2397	276.7	5150	2397	252.3	6960	2397	225.8	8180
2398	276.7	5150	2398	252.3	6960	2398	225.8	8180
2399	276.7	5170	2399	252.2	6970	2399	225.8	8190
2400	276.7	5150	2400	252.3	6970	2400	225.9	8180
2401	276.7	5160	2401	252.2	6980	2401	225.9	8180
2402	276.7	5150	2402	252.3	6970	2402	225.9	8190
2403	276.7	5160	2403	252.3	6970	2403	225.9	8190
2404	276.8	5160	2404	252.2	6960	2404	225.9	8190
2405	276.7	5160	2405	252.2	6970	2405	225.9	8190
2406	276.8	5160	2406	252.2	6980	2406	226	8180
2407	276.8	5150	2407	252.2	6970	2407	225.9	8170
2408	276.8	5150	2408	252.2	6970	2408	225.9	8180
2409	276.7	5160	2409	252.3	6970	2409	225.9	8180
2410	276.7	5150	2410	252.3	6980	2410	225.9	8190
2411	276.8	5150	2411	252.3	6980	2411	225.9	8200
2412	276.8	5160	2412	252.3	6970	2412	225.9	8200
2413	276.7	5150	2413	252.3	6970	2413	225.9	8190
2414	276.7	5150	2414	252.2	6960	2414	225.9	8190
2415	276.7	5150	2415	252.2	6970	2415	225.9	8190
2416	276.7	5150	2416	252.2	6970	2416	225.9	8180
2417	276.7	5160	2417	252.3	6960	2417	225.9	8190
2418	276.7	5150	2418	252.3	6970	2418	225.9	8180
2419	276.8	5150	2419	252.3	6960	2419	225.9	8180
2420	276.8	5150	2420	252.3	6980	2420	225.9	8190
2421	276.7	5140	2421	252.3	6990	2421	225.9	8190
2422	276.7	5140	2422	252.3	6980	2422	225.9	8180
2423	276.7	5140	2423	252.3	6980	2423	225.9	8190
2424	276.7	5160	2424	252.3	6980	2424	225.9	8190
2425	276.7	5150	2425	252.3	6980	2425	225.9	8190
2426	276.7	5160	2426	252.3	6960	2426	225.9	8170
2427	276.7	5160	2427	252.3	6960	2427	225.9	8180
2428	276.7	5150	2428	252.3	6970	2428	225.9	8180
2429	276.7	5150	2429	252.3	6980	2429	225.9	8180
2430	276.7	5150	2430	252.3	6980	2430	225.9	8190
2431	276.7	5140	2431	252.3	6980	2431	225.9	8200
2432	276.7	5150	2432	252.3	6960	2432	225.9	8190
2433	276.7	5160	2433	252.3	6970	2433	225.9	8190
2434	276.7	5150	2434	252.3	6970	2434	225.9	8200
2435	276.7	5150	2435	252.4	6960	2435	225.9	8200
2436	276.7	5140	2436	252.3	6970	2436	225.9	8200
2437	276.6	5150	2437	252.3	6980	2437	225.9	8210
2438	276.7	5160	2438	252.3	6980	2438	225.8	8200
2439	276.6	5150	2439	252.3	6970	2439	225.8	8200
2440	276.6	5160	2440	252.4	6970	2440	225.9	8200
2441	276.7	5150	2441	252.4	6970	2441	225.9	8200
2442	276.7	5170	2442	252.3	6970	2442	225.9	8190
2443	276.7	5150	2443	252.3	6970	2443	225.9	8190
2444	276.7	5150	2444	252.3	6980	2444	249.3	9490
2445	276.7	5160	2445	252.3	6980	2445	249.3	9480
2446	276.7	5150	2446	252.3	6980	2446	249.4	9480

2447	276.7	5160	2447	252.3	6960	2447	249.4	9490
2448	276.7	5150	2448	252.3	6960	2448	249.4	9490
2449	276.6	5160	2449	252.3	6980	2449	249.4	9490
2450	276.7	5140	2450	252.3	6980	2450	249.4	9480
2451	276.7	5150	2451	252.3	6970	2451	249.4	9480
2452	276.8	5140	2452	252.3	6980	2452	249.5	9490
2453	277	5140	2453	252.3	6980	2453	249.4	9480
2454	277.4	5240	2454	252.3	6980	2454	249.4	9480
2455	277.7	5250	2455	252.4	6980	2455	249.5	9480
2456	278.1	5250	2456	252.4	6980	2456	249.5	9490
2457	278.5	5240	2457	252.4	6980	2457	249.5	9490
2458	278.8	5250	2458	252.4	6980	2458	249.5	9490
2459	279.1	5240	2459	252.4	6980	2459	249.5	9490
2460	279.4	5240	2460	252.4	6970	2460	249.5	9490
2461	279.7	5250	2461	252.4	6970	2461	249.5	9500
2462	280	5250	2462	252.4	6980	2462	249.6	9490
2463	280.2	5250	2463	252.4	6980	2463	249.6	9490
2464	280.4	5240	2464	252.5	6980	2464	249.6	9480
2465	280.6	5240	2465	252.4	6960	2465	249.6	9490
2466	280.8	5250	2466	252.5	6970	2466	249.6	9490
2467	281	5250	2467	252.5	6960	2467	249.6	9490
2468	281.1	5250	2468	252.5	6980	2468	249.6	9480
2469	281.3	5260	2469	252.5	6980	2469	249.7	9490
2470	281.4	5260	2470	252.5	6970	2470	249.7	9500
2471	281.7	5250	2471	252.5	6970	2471	249.7	9490
2472	281.9	5250	2472	252.5	6980	2472	249.7	9480
2473	282.1	5250	2473	252.5	6970	2473	249.7	9490
2474	282.3	5250	2474	252.5	6970	2474	249.7	9490
2475	282.5	5250	2475	252.5	6990	2475	249.8	9480
2476	282.7	5240	2476	252.5	6980	2476	249.8	9470
2477	282.8	5250	2477	252.5	6970	2477	249.8	9490
2478	283	5270	2478	252.5	6970	2478	249.8	9490
2479	283	5260	2479	252.5	6980	2479	249.8	9480
2480	283.2	5260	2480	252.5	6980	2480	249.8	9490
2481	283.3	5250	2481	252.5	6980	2481	249.8	9490
2482	283.4	5250	2482	252.5	6950	2482	249.8	9490
2483	283.5	5240	2483	252.5	6970	2483	249.9	9480
2484	283.8	5240	2484	252.5	6980	2484	249.9	9480
2485	284.2	5240	2485	252.5	6960	2485	249.9	9490
2486	284.6	5250	2486	252.5	6970	2486	249.8	9480
2487	285	5340	2487	252.5	6980	2487	249.9	9490
2488	285.5	5340	2488	252.5	6980	2488	249.9	9500
2489	285.9	5350	2489	252.5	6970	2489	249.9	9490
2490	286.4	5340	2490	252.5	6960	2490	249.9	9490
2491	286.8	5340	2491	252.5	6970	2491	250	9480
2492	287.1	5330	2492	252.5	6970	2492	250	9500
2493	287.5	5340	2493	252.5	6960	2493	250	9480
2494	287.8	5340	2494	252.5	6960	2494	250	9490
2495	288.2	5350	2495	252.5	6970	2495	250.1	9480
2496	288.4	5340	2496	252.5	6970	2496	250.1	9480

2497	288.7	5330	2497	252.5	6980	2497	250.1	9480
2498	288.9	5340	2498	252.5	6970	2498	250.1	9480
2499	289.2	5340	2499	252.5	6970	2499	250.1	9480
2500	289.4	5350	2500	252.5	6970	2500	250.2	9480
2501	289.7	5340	2501	252.5	6950	2501	250.2	9490
2502	289.9	5340	2502	252.5	6960	2502	250.2	9490
2503	290.1	5340	2503	252.5	6970	2503	250.2	9480
2504	290.3	5350	2504	252.5	6960	2504	250.2	9490
2505	290.5	5340	2505	252.4	6970	2505	250.2	9470
2506	290.6	5340	2506	252.4	6980	2506	250.3	9490
2507	290.8	5330	2507	252.4	6970	2507	250.3	9490
2508	291	5350	2508	252.4	6960	2508	250.3	9480
2509	291.2	5340	2509	252.5	6960	2509	250.3	9480
2510	291.3	5340	2510	252.5	6970	2510	250.3	9490
2511	291.5	5350	2511	252.5	6960	2511	250.3	9480
2512	291.6	5340	2512	252.6	6970	2512	250.4	9490
2513	291.7	5340	2513	252.6	6970	2513	250.4	9480
2514	291.9	5340	2514	252.6	6970	2514	250.4	9500
2515	292	5340	2515	252.6	6970	2515	250.4	9480
2516	292.1	5330	2516	252.6	6970	2516	250.4	9490
2517	292.3	5340	2517	252.6	6980	2517	250.4	9500
2518	292.4	5330	2518	252.5	6970	2518	250.4	9470
2519	292.5	5340	2519	252.5	6990	2519	250.4	9480
2520	292.6	5340	2520	252.5	6980	2520	250.4	9490
2521	292.8	5320	2521	252.4	6980	2521	250.4	9480
2522	292.9	5340	2522	252.4	6970	2522	250.4	9490
2523	293	5340	2523	252.5	6970	2523	250.4	9490
2524	293.1	5330	2524	252.5	6960	2524	250.4	9490
2525	293.2	5350	2525	252.5	6970	2525	250.4	9490
2526	293.3	5340	2526	252.6	6980	2526	250.4	9490
2527	293.4	5340	2527	252.5	6970	2527	250.5	9490
2528	293.5	5340	2528	252.6	6960	2528	250.5	9480
2529	293.6	5320	2529	252.5	6960	2529	250.5	9490
2530	293.6	5350	2530	252.5	6960	2530	250.5	9480
2531	293.7	5350	2531	252.6	6960	2531	250.5	9490
2532	293.8	5340	2532	252.6	6980	2532	250.5	9490
2533	293.9	5340	2533	252.6	6970	2533	250.5	9490
2534	293.9	5350	2534	252.6	6970	2534	250.6	9480
2535	294	5340	2535	252.6	6970	2535	250.6	9480
2536	294.1	5350	2536	252.6	6970	2536	250.6	9480
2537	294.2	5430	2537	252.6	6970	2537	250.6	9500
2538	294.3	5440	2538	252.7	6980	2538	250.6	9490
2539	294.4	5330	2539	252.7	6980	2539	250.6	9480
2540	294.5	5330	2540	252.7	6970	2540	250.6	9500
2541	294.5	5330	2541	252.7	6970	2541	250.5	9490
2542	294.6	5340	2542	252.7	6970	2542	250.5	9480
2543	294.6	5330	2543	252.7	6960	2543	250.6	9480
2544	294.6	5330	2544	252.7	6970	2544	250.6	9480
2545	294.7	5350	2545	252.7	6970	2545	250.6	9480
2546	294.8	5330	2546	252.6	6970	2546	250.6	9490

2547	294.8	5330	2547	252.7	6980	2547	250.7	9480
2548	295	5340	2548	252.7	6980	2548	250.7	9470
2549	295	5340	2549	252.7	6980	2549	250.7	9480
2550	295.2	5430	2550	252.7	6980	2550	250.7	9480
2551	295.3	5440	2551	252.7	6990	2551	250.7	9480
2552	295.4	5430	2552	252.7	6970	2552	250.7	9480
2553	295.6	5420	2553	252.7	6980	2553	250.7	9490
2554	295.7	5430	2554	252.7	6980	2554	250.7	9480
2555	295.8	5440	2555	252.7	6970	2555	250.8	9480
2556	295.9	5440	2556	252.7	6980	2556	250.8	9480
2557	296.1	5430	2557	252.7	6990	2557	250.8	9480
2558	296.2	5440	2558	252.7	6980	2558	250.8	9480
2559	296.4	5440	2559	252.7	6970	2559	250.8	9480
2560	296.4	5440	2560	252.7	6970	2560	250.9	9490
2561	296.5	5430	2561	252.7	6970	2561	250.9	9480
2562	296.6	5440	2562	252.7	6970	2562	251	9480
2563	296.7	5440	2563	252.6	6970	2563	250.9	9480
2564	296.8	5440	2564	252.6	6950	2564	251	9480
2565	296.9	5430	2565	252.6	6980	2565	251	9480
2566	297	5440	2566	252.6	6980	2566	251	9480
2567	297	5440	2567	252.7	6960	2567	251.1	9480
2568	297.2	5440	2568	252.6	6970	2568	251.1	9500
2569	297.2	5440	2569	252.6	6970	2569	251.1	9490
2570	297.3	5430	2570	252.6	6960	2570	251	9490
2571	297.4	5430	2571	252.7	6970	2571	251.1	9490
2572	297.5	5440	2572	252.7	6980	2572	251.1	9480
2573	297.6	5430	2573	252.7	6980	2573	251.1	9490
2574	297.6	5440	2574	252.6	6970	2574	251.1	9500
2575	297.7	5440	2575	252.6	6980	2575	251.1	9500
2576	297.7	5440	2576	252.6	6970	2576	251.1	9490
2577	297.7	5430	2577	252.7	6960	2577	251.1	9490
2578	297.8	5430	2578	252.7	6970	2578	251.1	9490
2579	297.8	5440	2579	252.7	6960	2579	251.1	9490
2580	297.9	5440	2580	252.7	6980	2580	251.1	9490
2581	298	5450	2581	252.6	6970	2581	251.1	9490
2582	298.1	5430	2582	252.7	6980	2582	251.1	9490
2583	298.1	5460	2583	252.6	6970	2583	251.1	9480
2584	298.2	5440	2584	252.7	6970	2584	251.1	9490
2585	298.2	5440	2585	252.7	6970	2585	251.1	9500
2586	298.3	5440	2586	252.7	6970	2586	251.1	9480
2587	298.3	5440	2587	252.6	6970	2587	251.1	9480
2588	298.3	5440	2588	252.6	6970	2588	251.1	9490
2589	298.4	5430	2589	252.6	6970	2589	251.1	9490
2590	298.5	5450	2590	252.6	6980	2590	251.2	9490
2591	298.5	5460	2591	252.6	6970	2591	251.2	9480
2592	298.6	5440	2592	252.7	6970	2592	251.2	9490
2593	298.6	5450	2593	252.7	6980	2593	251.2	9480
2594	298.7	5440	2594	252.7	6980	2594	251.2	9470
2595	298.7	5420	2595	252.7	6980	2595	251.3	9470
2596	298.7	5440	2596	252.7	6970	2596	251.3	9470

2597	298.8	5430	2597	252.7	6970	2597	251.3	9480
2598	298.9	5440	2598	252.7	6970	2598	251.3	9470
2599	299	5440	2599	252.7	6960	2599	251.3	9480
2600	299.1	5440	2600	252.7	6970	2600	251.3	9480
2601	299.1	5450	2601	252.6	6970	2601	251.3	9480
2602	299.2	5430	2602	252.7	6970	2602	251.4	9490
2603	299.3	5450	2603	252.7	6980	2603	251.4	9480
2604	299.3	5440	2604	252.7	6980	2604	251.3	9480
2605	299.3	5440	2605	252.7	6970	2605	251.3	9490
2606	299.3	5440	2606	252.7	6970	2606	251.4	9480
2607	299.3	5430	2607	252.8	6980	2607	251.4	9480
2608	299.4	5430	2608	252.8	6970	2608	251.4	9490
2609	299.4	5430	2609	252.8	6980	2609	251.4	9480
2610	299.5	5430	2610	252.8	6970	2610	251.4	9490
2611	299.5	5430	2611	252.7	6970	2611	251.4	9480
2612	299.5	5430	2612	252.7	6970	2612	251.4	9480
2613	299.6	5440	2613	252.7	6980	2613	251.4	9480
2614	299.6	5440	2614	252.7	6980	2614	251.4	9490
2615	299.7	5430	2615	252.7	6970	2615	251.4	9480
2616	299.7	5430	2616	252.7	6980	2616	251.5	9480
2617	299.8	5440	2617	252.7	6980	2617	251.5	9480
2618	299.9	5440	2618	252.6	6970	2618	251.5	9480
2619	299.9	5430	2619	252.6	6980	2619	251.5	9480
2620	299.9	5430	2620	252.6	6990	2620	251.6	9480
2621	299.9	5440	2621	252.6	6990	2621	251.6	9490
2622	300	5430	2622	252.6	6980	2622	251.6	9480
2623	300	5430	2623	252.6	6990	2623	251.6	9480
2624	300.1	5430	2624	252.7	6980	2624	251.6	9490
2625	300.1	5430	2625	252.7	6980	2625	251.6	9490
2626	300.1	5430	2626	252.6	6980	2626	251.6	9490
2627	300.2	5430	2627	252.6	6980	2627	251.5	9490
2628	300.3	5440	2628	252.6	6980	2628	251.5	9480
2629	300.3	5430	2629	252.6	6980	2629	251.6	9490
2630	300.3	5430	2630	252.6	6980	2630	251.6	9470
2631	300.4	5430	2631	252.7	6980	2631	251.6	9490
2632	300.4	5430	2632	252.7	6980	2632	251.6	9480
2633	300.4	5430	2633	252.7	6970	2633	251.6	9480
2634	300.4	5420	2634	252.7	6970	2634	251.6	9490
2635	300.5	5440	2635	252.6	6980	2635	251.6	9490
2636	300.5	5430	2636	252.6	6980	2636	251.6	9490
2637	300.5	5420	2637	252.6	6990	2637	251.6	9490
2638	300.6	5410	2638	252.7	6990	2638	251.6	9490
2639	300.6	5420	2639	252.7	6990	2639	251.6	9490
2640	300.7	5410	2640	252.7	6990	2640	251.7	9500
2641	300.7	5420	2641	252.7	6980	2641	251.7	9510
2642	300.7	5420	2642	252.6	6970	2642	251.7	9480
2643	300.8	5420	2643	252.7	6990	2643	251.6	9480
2644	300.8	5410	2644	252.7	6970	2644	251.6	9470
2645	300.9	5420	2645	252.7	6970	2645	251.6	9480
2646	300.9	5410	2646	252.7	6980	2646	251.7	9490

2647	300.9	5430	2647	252.7	6980	2647	251.7	9470
2648	301	5420	2648	252.7	6980	2648	251.7	9480
2649	301	5420	2649	252.7	6980	2649	251.7	9490
2650	301	5420	2650	252.7	6980	2650	251.7	9480
2651	301	5410	2651	252.7	6980	2651	251.7	9490
2652	301	5420	2652	252.7	6980	2652	251.7	9480
2653	301	5420	2653	252.7	6980	2653	251.7	9480
2654	301	5430	2654	252.7	6970	2654	251.7	9490
2655	301.1	5430	2655	252.7	6980	2655	251.7	9480
2656	301.1	5430	2656	252.7	6980	2656	251.7	9490
2657	301.1	5430	2657	252.8	6970	2657	251.7	9470
2658	301.2	5430	2658	252.7	6970	2658	251.7	9480
2659	301.2	5440	2659	252.8	6980	2659	251.8	9480
2660	301.2	5440	2660	252.7	6980	2660	251.8	9480
2661	301.3	5440	2661	252.8	6980	2661	251.8	9480
2662	301.3	5440	2662	252.8	6980	2662	251.8	9490
2663	301.3	5430	2663	252.8	6970	2663	251.8	9480
2664	301.4	5430	2664	252.8	6970	2664	251.8	9490
2665	301.4	5420	2665	252.8	6970	2665	251.8	9490
2666	301.4	5430	2666	252.8	6970	2666	251.8	9480
2667	301.5	5430	2667	252.8	6970	2667	251.8	9470
2668	301.5	5430	2668	252.8	6980	2668	251.8	9470
2669	301.5	5440	2669	252.8	6980	2669	251.8	9470
2670	301.6	5430	2670	252.8	6970	2670	251.8	9480
2671	301.6	5430	2671	252.8	6980	2671	251.8	9470
2672	301.6	5440	2672	252.8	6970	2672	251.8	9480
2673	301.7	5440	2673	252.8	6970	2673	251.8	9490
2674	301.6	5430	2674	252.8	6980	2674	251.8	9480
2675	301.6	5430	2675	252.8	6980	2675	251.8	9480
2676	301.6	5420	2676	252.8	6990	2676	251.8	9480
2677	301.7	5430	2677	252.8	6980	2677	251.8	9490
2678	301.7	5420	2678	252.8	6990	2678	251.8	9480
2679	301.7	5430	2679	252.8	6980	2679	251.8	9480
2680	301.7	5430	2680	252.8	6980	2680	251.8	9480
2681	301.8	5440	2681	252.8	6990	2681	251.8	9480
2682	301.8	5430	2682	252.8	6960	2682	251.8	9480
2683	301.8	5430	2683	252.8	6990	2683	251.9	9490
2684	301.8	5430	2684	252.8	6980	2684	251.9	9480
2685	301.9	5430	2685	252.8	6980	2685	251.9	9490
2686	301.9	5440	2686	252.7	6970	2686	251.9	9480
2687	302	5440	2687	252.7	6980	2687	251.9	9480
2688	302	5440	2688	252.7	6970	2688	251.9	9480
2689	302	5440	2689	252.7	6960	2689	251.9	9470
2690	302	5440	2690	252.6	6970	2690	251.9	9480
2691	302.1	5440	2691	252.7	6970	2691	251.9	9480
2692	302.1	5430	2692	252.7	6970	2692	251.9	9480
2693	302.1	5440	2693	252.7	6980	2693	251.9	9500
2694	302.1	5440	2694	252.7	6980	2694	251.9	9480
2695	302.1	5440	2695	252.7	6990	2695	251.9	9480
2696	302.1	5440	2696	252.7	6980	2696	252	9480

2697	302.2	5430	2697	252.7	6970	2697	252	9480
2698	302.2	5440	2698	252.7	6970	2698	252	9480
2699	302.2	5430	2699	252.8	6980	2699	252.1	9480
2700	302.2	5430	2700	252.8	6970	2700	252.1	9470
2701	302.2	5430	2701	252.8	6970	2701	252.1	9480
2702	302.2	5440	2702	252.8	6960	2702	252.1	9480
2703	302.2	5430	2703	252.8	6980	2703	252.1	9460
2704	302.2	5430	2704	252.8	6980	2704	252.1	9470
2705	302.3	5430	2705	252.8	6990	2705	252.1	9470
2706	302.3	5440	2706	252.8	6980	2706	252.1	9480
2707	302.3	5440	2707	252.8	6970	2707	252.1	9480
2708	302.2	5440	2708	252.8	6980	2708	252.1	9470
2709	302.3	5430	2709	252.8	6980	2709	252.1	9480
2710	302.3	5440	2710	252.8	6970	2710	252.2	9480
2711	302.3	5430	2711	252.8	6980	2711	252.2	9480
2712	302.4	5420	2712	252.8	6980	2712	252.2	9480
2713	302.4	5430	2713	252.8	6980	2713	252.2	9480
2714	302.4	5430	2714	252.8	6980	2714	252.2	9480
2715	302.3	5430	2715	252.8	6980	2715	252.2	9470
2716	302.3	5420	2716	252.8	6980	2716	252.2	9470
2717	302.3	5420	2717	252.8	6970	2717	252.3	9480
2718	302.2	5430	2718	252.8	6970	2718	252.3	9480
2719	302.1	5430	2719	252.8	6970	2719	252.3	9480
2720	302.1	5430	2720	252.8	6970	2720	252.3	9480
2721	302.1	5430	2721	252.8	6970	2721	252.3	9470
2722	302.1	5430	2722	252.9	6970	2722	252.3	9480
2723	302.1	5430	2723	252.9	6970	2723	252.3	9480
2724	302.1	5440	2724	252.9	6980	2724	252.3	9480
2725	302.1	5430	2725	252.9	6990	2725	252.3	9480
2726	302	5420	2726	252.9	6980	2726	252.3	9480
2727	302	5420	2727	252.9	6970	2727	252.3	9490
2728	302	5440	2728	252.9	6970	2728	252.3	9470
2729	302	5430	2729	252.9	6970	2729	252.3	9470
2730	302	5430	2730	252.9	6980	2730	252.3	9470
2731	302	5440	2731	252.9	6970	2731	252.3	9480
2732	302.1	5450	2732	252.9	6970	2732	252.3	9490
2733	302.1	5440	2733	252.9	6970	2733	252.3	9470
2734	302.1	5420	2734	252.9	6970	2734	252.3	9480
2735	302.1	5440	2735	252.9	6980	2735	252.3	9480
2736	302.1	5430	2736	252.9	6970	2736	252.3	9480
2737	302.1	5430	2737	252.8	6970	2737	252.3	9490
2738	302.1	5440	2738	252.8	6970	2738	252.3	9470
2739	302.1	5440	2739	252.9	6980	2739	252.3	9490
2740	302.1	5440	2740	252.8	6990	2740	252.3	9490
2741	302.1	5440	2741	252.9	6980	2741	252.4	9470
2742	302.1	5440	2742	252.8	6980	2742	252.4	9490
2743	302	5430	2743	252.8	6980	2743	252.4	9490
2744	302	5420	2744	252.8	6980	2744	252.4	9480
2745	302	5430	2745	252.8	6990	2745	252.5	9480
2746	302	5450	2746	252.8	6980	2746	252.5	9480

2747	302	5440	2747	252.8	6980	2747	252.5	9480
2748	302	5430	2748	252.8	6970	2748	252.5	9490
2749	302	5430	2749	252.8	6970	2749	252.5	9470
2750	302	5450	2750	252.8	6980	2750	252.5	9480
2751	302.1	5440	2751	252.8	6970	2751	252.5	9490
2752	302.1	5440	2752	252.8	6970	2752	252.5	9490
2753	302.1	5430	2753	252.8	6960	2753	252.5	9480
2754	302.1	5430	2754	252.8	6970	2754	252.5	9470
2755	302.1	5430	2755	252.8	6980	2755	252.6	9470
2756	302.1	5430	2756	252.8	6980	2756	252.5	9470
2757	302.2	5430	2757	252.8	6980	2757	252.6	9470
2758	302.2	5420	2758	252.8	6990	2758	252.5	9480
2759	302.2	5420	2759	252.8	6980	2759	252.5	9480
2760	302.2	5430	2760	252.8	6970	2760	252.5	9470
2761	302.1	5430	2761	252.8	6980	2761	252.5	9480
2762	302.1	5430	2762	252.8	6970	2762	252.5	9460
2763	302.1	5430	2763	252.9	6990	2763	252.4	9460
2764	302.1	5450	2764	252.9	6980	2764	252.4	9460
2765	302.1	5440	2765	252.8	6970	2765	252.4	9470
2766	302.1	5440	2766	252.8	6950	2766	252.5	9480
2767	302.1	5440	2767	252.8	6970	2767	252.5	9470
2768	302.1	5430	2768	252.8	6970	2768	252.5	9480
2769	302.1	5430	2769	252.8	6970	2769	252.5	9480
2770	302.1	5430	2770	252.8	6980	2770	252.5	9480
2771	302.1	5430	2771	252.8	6980	2771	252.4	9480
2772	302.1	5440	2772	252.8	6980	2772	252.4	9480
2773	302.2	5440	2773	252.8	6970	2773	252.3	9480
2774	302.2	5440	2774	252.8	6960	2774	252.3	9470
2775	302.1	5440	2775	252.8	6960	2775	252.3	9480
2776	302.2	5440	2776	252.8	6960	2776	252.2	9490
2777	302.1	5440	2777	252.8	6970	2777	252.2	9470
2778	302.2	5440	2778	252.8	6960	2778	252.1	9480
2779	302.1	5430	2779	252.8	6960	2779	252.1	9480
2780	302.2	5420	2780	252.8	6960	2780	252.1	9490
2781	302.2	5430	2781	252.8	6960	2781	252.1	9480
2782	302.2	5420	2782	252.8	6970	2782	252.1	9480
2783	302.2	5420	2783	252.9	6970	2783	252	9480
2784	302.2	5420	2784	252.9	6970	2784	252	9490
2785	302.2	5430	2785	252.8	6970	2785	252	9480
2786	302.2	5430	2786	252.8	6970	2786	252	9480
2787	302.3	5430	2787	252.8	6970	2787	252	9480
2788	302.3	5420	2788	252.8	6980	2788	251.9	9490
2789	302.3	5440	2789	252.9	6980	2789	251.9	9480
2790	302.3	5430	2790	252.9	6970	2790	251.9	9470
2791	302.3	5420	2791	252.9	6970	2791	251.9	9480
2792	302.3	5440	2792	252.9	6980	2792	251.9	9480
2793	302.3	5430	2793	252.9	6970	2793	251.8	9490
2794	302.3	5430	2794	252.9	6970	2794	251.8	9490
2795	302.3	5430	2795	253	6970	2795	251.8	9470
2796	302.3	5430	2796	253	6970	2796	251.8	9480

2797	302.3	5440	2797	252.9	6970	2797	251.8	9470
2798	302.3	5440	2798	252.9	6980	2798	251.8	9470
2799	302.4	5440	2799	252.9	6970	2799	251.8	9470
2800	302.4	5420	2800	252.9	6970	2800	251.8	9460
2801	302.4	5440	2801	252.9	6970	2801	251.7	9470
2802	302.5	5420	2802	252.9	6980	2802	251.8	9470
2803	302.5	5430	2803	252.9	6970	2803	251.7	9470
2804	302.5	5440	2804	252.9	6980	2804	251.7	9490
2805	302.5	5460	2805	252.9	6980	2805	251.7	9480
2806	302.5	5440	2806	252.9	6970	2806	251.7	9480
2807	302.5	5440	2807	252.9	6960	2807	251.7	9490
2808	302.5	5440	2808	252.9	6980	2808	251.7	9470
2809	302.5	5440	2809	252.9	6980	2809	251.7	9470
2810	302.5	5430	2810	252.9	6990	2810	251.7	9480
2811	302.6	5430	2811	252.9	6990	2811	251.7	9480
2812	302.6	5430	2812	252.8	6980	2812	251.7	9480
2813	302.6	5430	2813	252.8	6970	2813	251.7	9490
2814	302.6	5440	2814	252.9	6960	2814	251.7	9480
2815	302.6	5420	2815	252.9	6970	2815	251.6	9480
2816	302.6	5430	2816	252.9	6960	2816	251.6	9490
2817	302.6	5420	2817	252.9	6970	2817	251.6	9480
2818	302.6	5410	2818	252.8	6970	2818	251.7	9470
2819	302.6	5430	2819	252.8	6970	2819	251.7	9480
2820	302.6	5430	2820	252.9	6970	2820	251.6	9480
2821	302.6	5440	2821	252.9	6960	2821	251.6	9490
2822	302.5	5430	2822	252.9	6970	2822	251.6	9480
2823	302.6	5430	2823	252.9	6980	2823	251.6	9480
2824	302.7	5420	2824	252.9	6990	2824	251.6	9470
2825	302.6	5430	2825	252.9	6970	2825	251.6	9480
2826	302.6	5440	2826	252.8	6990	2826	251.6	9480
2827	302.6	5420	2827	252.8	6970	2827	251.6	9480
2828	302.6	5430	2828	252.9	6990	2828	251.6	9480
2829	302.6	5420	2829	252.9	6990	2829	251.6	9490
2830	302.6	5430	2830	252.9	6960	2830	251.6	9480
2831	302.7	5440	2831	252.9	6990	2831	251.6	9480
2832	302.6	5420	2832	252.9	6980	2832	251.6	9470
2833	302.6	5430	2833	252.9	6980	2833	251.6	9480
2834	302.7	5420	2834	252.9	6980	2834	251.6	9480
2835	302.7	5430	2835	252.9	6970	2835	251.5	9480
2836	302.7	5420	2836	252.9	6980	2836	251.5	9480
2837	302.7	5430	2837	253	6970	2837	251.6	9480
2838	302.7	5430	2838	253	6960	2838	251.6	9480
2839	302.6	5420	2839	252.9	6980	2839	251.6	9480
2840	302.7	5420	2840	252.9	6960	2840	251.5	9480
2841	302.7	5430	2841	252.9	6970	2841	251.5	9480
2842	302.7	5430	2842	252.9	6970	2842	251.5	9480
2843	302.7	5430	2843	252.9	6980	2843	251.5	9470
2844	302.7	5440	2844	252.9	6980	2844	251.5	9490
2845	302.7	5430	2845	252.9	6970	2845	251.5	9490
2846	302.7	5430	2846	252.9	6960	2846	251.5	9490

2847	302.7	5440	2847	252.9	6960	2847	251.5	9490
2848	302.7	5440	2848	252.9	6960	2848	251.4	9490
2849	302.7	5430	2849	252.9	6960	2849	251.5	9490
2850	302.7	5440	2850	252.9	6970	2850	251.5	9490
2851	302.7	5440	2851	252.9	6980	2851	251.5	9500
2852	302.7	5430	2852	252.9	6970	2852	251.4	9490
2853	302.7	5440	2853	252.9	6980	2853	251.4	9480
2854	302.8	5440	2854	252.9	6980	2854	251.4	9480
2855	302.8	5440	2855	252.9	6970	2855	251.4	9490
2856	302.8	5430	2856	252.9	6970	2856	251.4	9480
2857	302.8	5430	2857	252.9	6980	2857	251.4	9480
2858	302.8	5440	2858	253	6980	2858	251.4	9480
2859	302.7	5450	2859	252.9	6970	2859	251.4	9470
2860	302.8	5440	2860	252.9	6970	2860	251.4	9470
2861	302.8	5430	2861	252.9	6970	2861	251.4	9480
2862	302.9	5430	2862	253	6970	2862	251.4	9490
2863	302.9	5430	2863	253	6970	2863	251.4	9480
2864	302.8	5430	2864	253	6970	2864	251.4	9470
2865	302.9	5420	2865	253	6960	2865	251.4	9480
2866	302.9	5440	2866	253	6970	2866	251.4	9480
2867	302.9	5430	2867	253	6970	2867	251.4	9480
2868	303	5430	2868	253	6970	2868	251.4	9470
2869	303	5420	2869	253	6980	2869	251.4	9480
2870	303	5420	2870	253	6960	2870	251.4	9480
2871	303	5420	2871	253	6970	2871	251.4	9480
2872	303	5430	2872	253	6980	2872	251.4	9470
2873	303	5420	2873	253	6960	2873	251.4	9470
2874	303	5430	2874	253	6970	2874	251.4	9470
2875	303	5430	2875	253	6960	2875	251.5	9480
2876	302.9	5440	2876	253	6970	2876	251.5	9470
2877	302.9	5420	2877	253	6970	2877	251.5	9490
2878	302.9	5430	2878	253	6970	2878	251.5	9470
2879	302.9	5440	2879	253	6970	2879	251.5	9480
2880	302.9	5440	2880	253	6970	2880	251.5	9480
2881	303	5430	2881	253	6970	2881	251.5	9480
2882	303	5430	2882	253	6970	2882	251.5	9470
2883	303	5440	2883	253	6970	2883	251.5	9470
2884	303.1	5420	2884	253	6980	2884	251.5	9480
2885	303	5420	2885	253	6980	2885	251.4	9480
2886	303	5430	2886	252.9	6970	2886	251.4	9470
2887	303	5430	2887	253	6970	2887	251.4	9470
2888	303	5420	2888	253	6980	2888	251.4	9470
2889	303	5430	2889	253	6980	2889	251.4	9480
2890	303	5430	2890	253	6970	2890	251.4	9470
2891	303	5420	2891	253	6970	2891	251.4	9470
2892	302.9	5440	2892	253	6970	2892	251.3	9480
2893	302.9	5430	2893	253	6970	2893	251.4	9470
2894	302.9	5440	2894	252.9	6970	2894	251.4	9480
2895	302.9	5440	2895	252.9	6970	2895	251.4	9470
2896	302.9	5430	2896	252.9	6970	2896	251.4	9480

2897	302.9	5430	2897	252.9	6980	2897	251.4	9470
2898	303	5420	2898	252.9	6970	2898	251.4	9470
2899	303	5420	2899	252.9	6980	2899	251.4	9470
2900	303	5420	2900	252.9	6980	2900	251.4	9480
2901	303	5430	2901	252.9	6960	2901	251.4	9460
2902	303	5430	2902	252.9	6970	2902	251.4	9480
2903	303	5430	2903	252.9	6970	2903	251.4	9460
2904	303	5430	2904	252.9	6960	2904	251.4	9480
2905	303	5430	2905	252.9	6970	2905	251.4	9470
2906	303.1	5450	2906	252.9	6970	2906	251.5	9470
2907	303.1	5420	2907	252.9	6980	2907	251.5	9480
2908	303.1	5440	2908	252.9	6970	2908	251.4	9460
2909	303.1	5440	2909	252.9	6960	2909	251.5	9470
2910	303.1	5430	2910	252.9	6950	2910	251.5	9480
2911	303	5440	2911	252.9	6960	2911	251.5	9480
2912	303	5430	2912	253	6970	2912	251.5	9470
2913	302.9	5410	2913	253	6970	2913	251.5	9480
2914	302.9	5430	2914	252.9	6980	2914	251.5	9470
2915	302.8	5420	2915	253	6980	2915	251.5	9460
2916	302.8	5430	2916	252.9	6970	2916	251.5	9470
2917	302.7	5430	2917	252.9	6970	2917	251.5	9470
2918	302.7	5420	2918	253	6970	2918	251.5	9470
2919	302.8	5430	2919	252.9	6970	2919	251.5	9480
2920	302.7	5430	2920	252.9	6980	2920	251.5	9480
2921	302.7	5430	2921	253	6980	2921	251.5	9480
2922	302.7	5430	2922	253	6970	2922	251.5	9480
2923	302.7	5430	2923	252.9	6970	2923	251.5	9470
2924	302.6	5440	2924	252.9	6970	2924	251.4	9470
2925	302.6	5420	2925	252.9	6980	2925	251.4	9470
2926	302.6	5430	2926	252.9	6970	2926	251.4	9370
2927	302.6	5430	2927	252.9	6970	2927	251.4	9490
2928	302.6	5440	2928	252.9	6960	2928	251.4	9480
2929	302.6	5430	2929	253	6970	2929	251.4	9480
2930	302.6	5440	2930	253	6980	2930	251.4	9480
2931	302.5	5430	2931	253	6970	2931	251.4	9480
2932	302.5	5420	2932	253	6960	2932	251.4	9490
2933	302.5	5430	2933	253	6970	2933	251.4	9470
2934	302.5	5420	2934	252.9	6960	2934	251.4	9480
2935	302.4	5420	2935	252.9	6960	2935	251.4	9480
2936	302.5	5420	2936	252.9	6980	2936	251.4	9470
2937	302.5	5420	2937	252.9	6980	2937	251.4	9480
2938	302.4	5430	2938	252.9	6970	2938	251.4	9480
2939	302.4	5430	2939	252.9	6970	2939	251.5	9500
2940	302.5	5440	2940	252.9	6960	2940	251.4	9490
2941	302.4	5420	2941	252.9	6980	2941	251.5	9480
2942	302.4	5430	2942	252.9	6970	2942	251.5	9480
2943	302.4	5440	2943	253	6960	2943	251.4	9480
2944	302.4	5430	2944	253	6970	2944	251.4	9490
2945	302.3	5450	2945	253	6980	2945	251.4	9480
2946	302.3	5320	2946	253	6970	2946	251.4	9470

2947	302.3	5320	2947	253	6970	2947	251.4	9470
2948	302.3	5410	2948	253	6970	2948	251.4	9490
2949	302.3	5320	2949	253	6970	2949	251.4	9480
2950	302.3	5420	2950	252.9	6980	2950	251.4	9480
2951	302.3	5320	2951	252.9	6980	2951	251.4	9490
2952	302.3	5420	2952	252.9	6980	2952	251.4	9480
2953	302.3	5440	2953	253	6990	2953	251.4	9490
2954	302.3	5430	2954	252.9	6980	2954	251.5	9490
2955	302.3	5440	2955	252.9	6970	2955	251.5	9480
2956	302.3	5430	2956	252.9	6970	2956	251.5	9480
2957	302.3	5440	2957	252.9	6970	2957	251.5	9480
2958	302.3	5340	2958	252.9	6960	2958	251.5	9480
2959	302.2	5330	2959	252.9	6980	2959	251.5	9480
2960	302.2	5430	2960	252.9	6960	2960	251.5	9480
2961	302.2	5340	2961	252.9	6970	2961	251.5	9470
2962	302.2	5440	2962	252.9	6970	2962	251.5	9470
2963	302.2	5440	2963	252.9	6970	2963	251.5	9480
2964	302.2	5340	2964	252.9	6970	2964	251.5	9470
2965	302.3	5340	2965	252.9	6980	2965	251.5	9480
2966	302.3	5440	2966	252.9	6960	2966	251.5	9480
2967	302.3	5330	2967	252.9	6970	2967	251.5	9480
2968	302.3	5350	2968	253	6970	2968	251.4	9480
2969	302.3	5440	2969	252.9	6980	2969	251.4	9480
2970	302.3	5350	2970	252.9	6970	2970	251.4	9480
2971	302.3	5330	2971	252.9	6960	2971	251.4	9480
2972	302.3	5340	2972	252.9	6970	2972	251.4	9480
2973	302.4	5440	2973	252.8	6960	2973	251.4	9480
2974	302.4	5440	2974	252.8	6960	2974	251.4	9490
2975	302.4	5350	2975	252.9	6970	2975	251.4	9470
2976	302.4	5340	2976	252.9	6960	2976	251.4	9480
2977	302.4	5330	2977	252.9	6960	2977	251.4	9460
2978	302.4	5340	2978	252.9	6970	2978	251.5	9480
2979	302.4	5430	2979	252.9	6970	2979	251.5	9490
2980	302.4	5420	2980	252.9	6980	2980	251.6	9480
2981	302.4	5340	2981	252.9	6980	2981	251.6	9470
2982	302.5	5430	2982	252.9	6960	2982	251.6	9480
2983	302.4	5340	2983	253	6970	2983	251.6	9480
2984	302.5	5340	2984	253	6970	2984	251.6	9490
2985	302.5	5430	2985	253	6970	2985	251.6	9480
2986	302.4	5340	2986	253	6960	2986	251.6	9480
2987	302.4	5430	2987	253	6960	2987	251.6	9480
2988	302.4	5430	2988	253	6960	2988	251.6	9480
2989	302.4	5430	2989	253	6970	2989	251.6	9470
2990	302.4	5430	2990	253	6960	2990	251.6	9470
2991	302.5	5320	2991	253	6960	2991	251.6	9470
2992	302.5	5330	2992	253	6960	2992	251.6	9470
2993	302.5	5340	2993	253	6960	2993	251.6	9460
2994	302.5	5340	2994	253	6960	2994	251.6	9470
2995	302.6	5440	2995	253	6970	2995	251.6	9480
2996	302.6	5330	2996	253	6980	2996	251.6	9480

2997	302.6	5430	2997	253	6980	2997	251.6	9480
2998	302.5	5440	2998	253	6980	2998	251.6	9480
2999	302.5	5340	2999	253	6970	2999	251.6	9480
3000	302.5	5420	3000	253	6970	3000	251.6	9480
3001	302.5	5430	3001	253	6970	3001	251.6	9480
3002	302.5	5430	3002	253	6970	3002	251.6	9480
3003	302.5	5430	3003	253	6970	3003	251.6	9480
3004	302.5	5430	3004	253	6980	3004	251.6	9480
3005	302.4	5340	3005	253	6970	3005	251.6	9470
3006	302.4	5430	3006	253.1	6980	3006	251.6	9470
3007	302.4	5430	3007	253.1	6970	3007	251.5	9470
3008	302.5	5330	3008	253.1	6980	3008	251.6	9470
3009	302.4	5320	3009	253	6970	3009	251.5	9470
3010	302.4	5340	3010	253.1	6970	3010	251.5	9480
3011	302.4	5340	3011	253.1	6960	3011	251.6	9470
3012	302.4	5340	3012	253.1	6970	3012	251.6	9470
3013	302.4	5430	3013	253.1	6960	3013	251.6	9480
3014	302.4	5440	3014	253.1	6970	3014	251.6	9480
3015	302.4	5430	3015	253.1	6960	3015	251.5	9470
3016	302.4	5340	3016	253.1	6960	3016	251.5	9470
3017	302.4	5420	3017	253.1	6950	3017	251.5	9480
3018	302.4	5420	3018	253.1	6970	3018	251.6	9480
3019	302.4	5430	3019	253.1	6970	3019	251.6	9470
3020	302.4	5340	3020	253.1	6980	3020	251.6	9480
3021	302.4	5320	3021	253.1	6950	3021	251.6	9490
3022	302.4	5320	3022	253.1	6970	3022	251.6	9480
3023	302.4	5430	3023	253.1	6970	3023	251.6	9470
3024	302.4	5330	3024	253.1	6980	3024	251.6	9480
3025	302.3	5420	3025	253.1	6960	3025	251.6	9490
3026	302.3	5430	3026	253.1	6970	3026	251.6	9480
3027	302.3	5320	3027	253.1	6970	3027	251.6	9470
3028	302.4	5330	3028	253.1	6970	3028	251.6	9490
3029	302.4	5430	3029	253.1	6970	3029	251.6	9470
3030	302.4	5430	3030	253.1	6980	3030	251.6	9480
3031	302.4	5410	3031	253.1	6950	3031	251.6	9500
3032	302.4	5320	3032	253.1	6970	3032	251.6	9500
3033	302.5	5430	3033	253.1	6970	3033	251.6	9500
3034	302.5	5420	3034	253.1	6960	3034	251.6	9480
3035	302.5	5430	3035	253.1	6970	3035	251.6	9480
3036	302.5	5430	3036	253.1	6960	3036	251.6	9470
3037	302.5	5420	3037	253.1	6950	3037	251.6	9480
3038	302.5	5430	3038	253.1	6960	3038	251.6	9480
3039	302.5	5430	3039	253.1	6950	3039	251.6	9470
3040	302.6	5430	3040	253.1	6960	3040	251.6	9480
3041	302.5	5420	3041	253.1	6970	3041	251.7	9480
3042	302.5	5420	3042	253.1	6960	3042	251.6	9480
3043	302.5	5430	3043	253.1	6960	3043	251.6	9480
3044	302.5	5340	3044	253.1	6980	3044	251.6	9470
3045	302.4	5420	3045	253.1	6970	3045	251.6	9470
3046	302.4	5420	3046	253.1	6970	3046	251.6	9470

3047	302.3	5330	3047	253.1	6960	3047	251.6	9470
3048	302.4	5330	3048	253.1	6960	3048	251.6	9480
3049	302.3	5440	3049	253.2	6960	3049	251.6	9480
3050	302.3	5430	3050	253.1	6980	3050	251.6	9480
3051	302.3	5440	3051	253.1	6960	3051	251.6	9500
3052	302.3	5430	3052	253.1	6960	3052	251.6	9480
3053	302.3	5420	3053	253.2	6950	3053	251.6	9480
3054	302.4	5430	3054	253.2	6960	3054	251.7	9470
3055	302.4	5420	3055	253.2	6960	3055	251.7	9480
3056	302.4	5430	3056	253.2	6970	3056	251.7	9490
3057	302.3	5430	3057	253.2	6980	3057	251.7	9480
3058	302.3	5430	3058	253.1	6970	3058	251.7	9480
3059	302.3	5430	3059	253.1	6960	3059	251.7	9480
3060	302.3	5440	3060	253.1	6950	3060	251.6	9480
3061	302.3	5440	3061	253.1	6970	3061	251.6	9490
3062	302.3	5430	3062	253.1	6970	3062	251.6	9480
3063	302.2	5430	3063	253.1	6970	3063	251.6	9470
3064	302.2	5430	3064	253.1	6980	3064	251.6	9480
3065	302.2	5440	3065	253.1	6970	3065	251.6	9470
3066	302.2	5320	3066	253	6970	3066	251.6	9480
3067	302.3	5340	3067	253	6960	3067	251.6	9490
3068	302.3	5340	3068	253	6970	3068	251.6	9480
3069	302.2	5440	3069	253.1	6970	3069	251.6	9470
3070	302.2	5440	3070	253.1	6960	3070	251.6	9470
3071	302.2	5440	3071	253.1	6960	3071	251.7	9480
3072	302.2	5440	3072	253.1	6960	3072	251.7	9470
3073	302.2	5450	3073	253.1	6960	3073	251.6	9480
3074	302.3	5350	3074	253.1	6950	3074	251.7	9480
3075	302.3	5440	3075	253.1	6970	3075	251.7	9480
3076	302.3	5340	3076	253.1	6960	3076	251.7	9480
3077	302.3	5330	3077	253.1	6960	3077	251.7	9470
3078	302.3	5330	3078	253.1	6960	3078	251.7	9460
3079	302.2	5430	3079	253	6970	3079	251.7	9480
3080	302.2	5420	3080	253	6970	3080	251.7	9480
3081	302.2	5330	3081	253	6970	3081	251.7	9490
3082	302.2	5330	3082	253	6970	3082	251.7	9470
3083	302.2	5330	3083	253	6950	3083	251.7	9470
3084	302.2	5340	3084	253	6970	3084	251.7	9470
3085	302.2	5340	3085	253	6970	3085	251.7	9470
3086	302.2	5440	3086	253	6980	3086	251.7	9480
3087	302.2	5340	3087	253	6970	3087	251.7	9480
3088	302.3	5440	3088	253	6960	3088	251.7	9470
3089	302.3	5440	3089	253	6960	3089	251.7	9480
3090	302.3	5450	3090	253	6960	3090	251.7	9470
3091	302.3	5340	3091	253	6960	3091	251.7	9490
3092	302.3	5350	3092	252.9	6970	3092	251.7	9480
3093	302.3	5340	3093	252.9	6970	3093	251.7	9480
3094	302.2	5340	3094	252.9	6960	3094	251.7	9470
3095	302.2	5440	3095	252.9	6960	3095	251.8	9480
3096	302.3	5430	3096	253	6970	3096	251.8	9480

3097	302.3	5430	3097	253	6980	3097	251.8	9480
3098	302.3	5440	3098	253	6980	3098	251.8	9490
3099	302.3	5330	3099	253	6960	3099	251.8	9480
3100	302.2	5340	3100	253	6970	3100	251.8	9490
3101	302.2	5340	3101	253	6980	3101	251.8	9480
3102	302.2	5430	3102	253	6970	3102	251.8	9490
3103	302.2	5350	3103	252.9	6960	3103	251.9	9500
3104	302.2	5340	3104	252.9	6960	3104	251.9	9480
3105	302.2	5340	3105	252.9	6970	3105	251.8	9490
3106	302.3	5430	3106	252.9	6970	3106	251.8	9490
3107	302.3	5320	3107	252.9	6960	3107	251.8	9500
3108	302.3	5350	3108	252.9	6970	3108	251.8	9480
3109	302.2	5330	3109	252.9	6980	3109	251.8	9490
3110	302.3	5330	3110	252.9	6970	3110	251.8	9480
3111	302.2	5330	3111	252.9	6980	3111	251.8	9490
3112	302.2	5430	3112	252.9	6970	3112	251.8	9480
3113	302.3	5330	3113	252.9	6980	3113	251.8	9490
3114	302.3	5430	3114	252.9	6960	3114	251.8	9490
3115	302.3	5430	3115	252.9	6980	3115	251.8	9480
3116	302.4	5440	3116	253	6990	3116	251.8	9470
3117	302.4	5330	3117	252.9	6970	3117	251.8	9470
3118	302.4	5420	3118	252.9	6980	3118	251.8	9490
3119	302.4	5320	3119	252.9	6970	3119	251.8	9470
3120	302.4	5320	3120	252.9	6970	3120	251.8	9480
3121	302.4	5320	3121	253	6980	3121	251.8	9480
3122	302.4	5330	3122	253	6980	3122	251.8	9470
3123	302.4	5430	3123	252.9	6960	3123	251.8	9490
3124	302.4	5350	3124	253	6970	3124	251.9	9470
3125	302.3	5330	3125	253	6970	3125	251.9	9460
3126	302.3	5440	3126	253	6960	3126	251.9	9470
3127	302.4	5430	3127	253	6980	3127	251.9	9480
3128	302.4	5320	3128	253	6970	3128	251.9	9470
3129	302.4	5330	3129	253	6970	3129	251.9	9470
3130	302.4	5330	3130	253	6980	3130	251.9	9470
3131	302.4	5330	3131	253	6970	3131	252	9480
3132	302.4	5440	3132	253.1	6980	3132	252	9470
3133	302.4	5330	3133	253	6960	3133	252	9460
3134	302.5	5330	3134	253	6980	3134	252	9470
3135	302.5	5340	3135	253	6970	3135	252	9480
3136	302.4	5430	3136	253	6960	3136	252	9470
3137	302.4	5440	3137	253	6960	3137	252	9480
3138	302.4	5340	3138	253	6960	3138	252	9480
3139	302.4	5440	3139	253	6950	3139	252	9470
3140	302.4	5340	3140	253	6960	3140	252	9470
3141	302.4	5330	3141	253	6960	3141	252	9470
3142	302.4	5340	3142	253.1	6960	3142	252	9470
3143	302.4	5330	3143	253.1	6970	3143	252	9480
3144	302.4	5430	3144	253.1	6970	3144	252	9470
3145	302.4	5330	3145	253.3	7050	3145	252	9470
3146	302.4	5440	3146	254	7060	3146	252	9480

3147	302.4	5450	3147	254.9	7060	3147	252	9470
3148	302.3	5340	3148	255.8	7060	3148	252	9460
3149	302.3	5340	3149	256.7	7160	3149	252	9470
3150	302.3	5320	3150	257.8	7160	3150	252	9470
3151	302.3	5420	3151	258.8	7160	3151	252	9470
3152	302.3	5330	3152	259.8	7150	3152	252	9480
3153	302.4	5320	3153	260.8	7150	3153	252	9470
3154	302.4	5430	3154	261.6	7150	3154	252	9480
3155	302.3	5420	3155	262.4	7150	3155	252	9480
3156	302.4	5420	3156	263.1	7150	3156	252.1	9480
3157	302.4	5330	3157	263.9	7150	3157	252.1	9470
3158	302.3	5330	3158	264.6	7150	3158	252.1	9460
3159	302.3	5330	3159	265.2	7150	3159	252.1	9460
3160	302.4	5330	3160	265.8	7160	3160	252	9470
3161	302.4	5340	3161	266.4	7150	3161	252	9480
3162	302.4	5330	3162	267	7250	3162	252	9460
3163	302.4	5340	3163	267.5	7250	3163	252	9460
3164	302.4	5330	3164	268	7250	3164	252	9470
3165	302.4	5340	3165	268.5	7260	3165	252	9480
3166	302.4	5430	3166	268.9	7240	3166	252	9470
3167	302.4	5330	3167	269.4	7240	3167	252	9480
3168	302.4	5330	3168	269.8	7250	3168	252	9470
3169	302.4	5330	3169	270.1	7250	3169	252	9470
3170	302.4	5340	3170	270.5	7260	3170	252	9450
3171	302.5	5330	3171	270.8	7250	3171	252	9460
3172	302.5	5330	3172	271	7260	3172	252	9470
3173	302.5	5430	3173	271.3	7270	3173	252	9480
3174	302.5	5320	3174	271.6	7250	3174	252	9460
3175	302.5	5340	3175	271.9	7250	3175	252	9460
3176	302.5	5330	3176	272.2	7260	3176	252	9470
3177	302.5	5330	3177	272.5	7250	3177	251.9	9470
3178	302.4	5320	3178	272.7	7270	3178	252	9480
3179	302.4	5320	3179	273	7260	3179	251.9	9480
3180	302.4	5330	3180	273.1	7260	3180	251.9	9470
3181	302.4	5330	3181	273.3	7260	3181	252	9470
3182	302.4	5430	3182	273.6	7260	3182	252.2	9560
3183	302.4	5330	3183	273.8	7350	3183	252.7	9560
3184	302.4	5330	3184	273.9	7360	3184	253.5	9560
3185	302.4	5320	3185	274.1	7350	3185	254.4	9640
3186	302.4	5330	3186	274.3	7360	3186	255.4	9660
3187	302.4	5320	3187	274.5	7350	3187	256.6	9660
3188	302.4	5320	3188	274.6	7360	3188	257.9	9760
3189	302.4	5430	3189	274.8	7360	3189	259.4	9760
3190	302.4	5420	3190	275	7350	3190	260.9	9740
3191	302.5	5320	3191	275.2	7350	3191	262.2	9740
3192	302.4	5320	3192	275.4	7350	3192	263.4	9740
3193	302.5	5340	3193	275.5	7350	3193	264.8	9840
3194	302.5	5320	3194	275.7	7350	3194	266.1	9840
3195	302.5	5340	3195	275.9	7360	3195	267.5	9840
3196	302.5	5330	3196	276	7360	3196	268.7	9850

3197	302.5	5330	3197	276.2	7350	3197	269.8	9850
3198	302.5	5330	3198	276.3	7360	3198	270.9	9850
3199	302.5	5330	3199	276.4	7350	3199	271.8	9830
3200	302.5	5320	3200	276.5	7340	3200	272.7	9830
3201	302.5	5330	3201	276.7	7360	3201	273.6	9850
3202	302.5	5330	3202	276.8	7350	3202	274.3	9840
3203	302.5	5330	3203	276.9	7360	3203	274.9	9830
3204	302.5	5330	3204	277	7350	3204	275.3	9850
3205	302.5	5330	3205	277.1	7360	3205	275.7	9840
3206	302.5	5330	3206	277.1	7340	3206	276	9850
3207	302.5	5330	3207	277.1	7350	3207	276.3	9850
3208	302.5	5320	3208	277.1	7340	3208	276.5	9860
3209	302.5	5330	3209	277.1	7340	3209	276.6	9850
3210	302.5	5340	3210	277.1	7340	3210	276.8	9850
3211	302.5	5330	3211	277.1	7350	3211	276.9	9840
3212	302.5	5330	3212	277.1	7360	3212	277	9850
3213	302.5	5440	3213	277.2	7360	3213	277	9860
3214	302.5	5330	3214	277.2	7350	3214	277.1	9850
3215	302.6	5420	3215	277.2	7370	3215	277.2	9950
3216	302.5	5410	3216	277.3	7350	3216	277.3	9860
3217	302.5	5320	3217	277.3	7350	3217	277.3	9960
3218	302.5	5330	3218	277.4	7350	3218	277.4	9940
3219	302.5	5330	3219	277.4	7350	3219	277.4	9960
3220	302.5	5320	3220	277.5	7360	3220	277.4	9960
3221	302.5	5340	3221	277.5	7350	3221	277.4	9940
3222	302.5	5330	3222	277.4	7350	3222	277.4	9960
3223	302.5	5440	3223	277.4	7340	3223	277.4	9960
3224	302.5	5340	3224	277.4	7340	3224	277.4	9950
3225	302.5	5330	3225	277.4	7350	3225	277.4	9960
3226	302.5	5330	3226	277.3	7350	3226	277.5	9950
3227	302.5	5320	3227	277.3	7350	3227	277.5	9960
3228	302.5	5340	3228	277.4	7350	3228	277.6	9970
3229	302.6	5330	3229	277.4	7350	3229	277.6	9960
3230	302.6	5330	3230	277.4	7350	3230	277.6	9970
3231	302.6	5330	3231	277.4	7350	3231	277.6	9960
3232	302.6	5420	3232	277.4	7350	3232	277.6	9960
3233	302.6	5310	3233	277.3	7350	3233	277.6	9960
3234	302.6	5430	3234	277.3	7350	3234	277.6	9960
3235	302.6	5420	3235	277.3	7360	3235	277.6	9950
3236	302.5	5410	3236	277.3	7350	3236	277.6	9970
3237	302.6	5320	3237	277.3	7350	3237	277.6	9960
3238	302.6	5430	3238	277.2	7370	3238	277.6	9960
3239	302.6	5430	3239	277.2	7360	3239	277.6	9960
3240	302.6	5320	3240	277.2	7360	3240	277.6	9960
3241	302.6	5440	3241	277.2	7350	3241	277.6	9960
3242	302.6	5340	3242	277.3	7350	3242	277.6	9960
3243	302.7	5440	3243	277.3	7360	3243	277.6	9970
3244	302.7	5340	3244	277.3	7370	3244	277.6	9960
3245	302.6	5430	3245	277.4	7360	3245	277.6	9960
3246	302.7	5320	3246	277.4	7360	3246	277.6	9960

3247	302.6	5430	3247	277.4	7350	3247	277.6	9960
3248	302.6	5430	3248	277.5	7360	3248	277.5	9970
3249	302.6	5440	3249	277.5	7360	3249	277.5	9950
3250	302.6	5320	3250	277.5	7350	3250	277.5	9960
3251	302.6	5330	3251	277.5	7360	3251	277.5	9950
3252	302.6	5310	3252	277.6	7450	3252	277.5	9970
3253	302.6	5430	3253	277.6	7360	3253	277.5	9960
3254	302.6	5440	3254	277.6	7360	3254	277.5	9970
3255	302.5	5320	3255	277.7	7360	3255	277.5	9960
3256	302.5	5440	3256	277.7	7350	3256	277.5	9960
3257	302.5	5430	3257	277.7	7340	3257	277.6	9950
3258	302.5	5430	3258	277.7	7350	3258	277.6	9960
3259	302.6	5430	3259	277.8	7350	3259	277.7	9960
3260	302.5	5430	3260	277.8	7350	3260	277.7	9950
3261	302.5	5430	3261	277.9	7360	3261	277.8	9970
3262	302.5	5410	3262	277.9	7360	3262	277.8	9960
3263	302.5	5430	3263	278	7350	3263	277.8	9950
3264	302.5	5430	3264	278	7360	3264	277.8	9950
3265	302.5	5430	3265	278.1	7350	3265	277.8	9960
3266	302.6	5430	3266	278.1	7350	3266	277.8	9970
3267	302.5	5440	3267	278.1	7350	3267	277.8	9980
3268	302.6	5430	3268	278.2	7350	3268	277.8	9960
3269	302.5	5420	3269	278.2	7350	3269	277.7	9960
3270	302.6	5430	3270	278.3	7360	3270	277.6	9960
3271	302.6	5430	3271	278.3	7450	3271	277.5	9960
3272	302.6	5430	3272	278.3	7350	3272	277.5	9970
3273	302.6	5420	3273	278.3	7350	3273	277.5	9970
3274	302.7	5420	3274	278.4	7360	3274	277.5	9950
3275	302.7	5430	3275	278.4	7340	3275	277.5	9960
3276	302.7	5430	3276	278.3	7350	3276	277.5	9980
3277	302.7	5440	3277	278.3	7350	3277	277.5	9970
3278	302.8	5430	3278	278.2	7350	3278	277.5	9960
3279	302.8	5430	3279	278.2	7370	3279	277.4	9960
3280	302.7	5430	3280	278.2	7360	3280	277.4	9960
3281	302.7	5420	3281	278.2	7360	3281	277.4	9950
3282	302.7	5410	3282	278.2	7360	3282	277.5	9950
3283	302.7	5420	3283	278.1	7360	3283	277.5	9970
3284	302.6	5430	3284	278.1	7350	3284	277.5	9960
3285	302.6	5430	3285	278.1	7350	3285	277.5	9960
3286	302.7	5420	3286	278.1	7350	3286	277.5	9970
3287	302.6	5310	3287	278.1	7360	3287	277.5	9970
3288	302.6	5420	3288	278.1	7370	3288	277.6	9960
3289	302.6	5420	3289	278.1	7360	3289	277.6	9960
3290	302.6	5410	3290	278.1	7360	3290	277.6	9960
3291	302.6	5430	3291	278.1	7360	3291	277.7	9960
3292	302.6	5430	3292	278.1	7350	3292	277.6	9950
3293	302.6	5420	3293	278.1	7350	3293	277.7	9960
3294	302.6	5410	3294	278.1	7350	3294	277.7	9960
3295	302.6	5430	3295	278.2	7350	3295	277.8	9950
3296	302.6	5430	3296	278.2	7350	3296	277.8	9960

3297	302.6	5430	3297	278.2	7340	3297	277.8	9960
3298	302.6	5430	3298	278.2	7350	3298	277.9	9950
3299	302.6	5430	3299	278.2	7350	3299	277.9	9960
3300	302.6	5430	3300	278.2	7350	3300	277.9	9950
3301	302.7	5430	3301	278.2	7350	3301	277.9	9960
3302	302.7	5430	3302	278.3	7360	3302	278	9960
3303	302.7	5440	3303	278.3	7360	3303	278	9960
3304	302.7	5430	3304	278.3	7360	3304	278	9950
3305	302.7	5440	3305	278.3	7440	3305	278.1	9960
3306	302.7	5420	3306	278.3	7340	3306	278.1	9970
3307	302.7	5440	3307	278.3	7340	3307	278.1	9960
3308	302.7	5430	3308	278.3	7350	3308	278.1	9970
3309	302.6	5430	3309	278.3	7350	3309	278.2	9970
3310	302.7	5420	3310	278.4	7350	3310	278.2	9950
3311	302.7	5420	3311	278.4	7350	3311	278.1	9960
3312	302.8	5410	3312	278.4	7350	3312	278	9950
3313	302.8	5420	3313	278.4	7350	3313	278	9950
3314	302.8	5420	3314	278.4	7340	3314	278	9950
3315	302.8	5430	3315	278.4	7350	3315	277.9	9960
3316	302.8	5440	3316	278.5	7360	3316	277.9	9960
3317	302.8	5430	3317	278.5	7350	3317	277.8	9960
3318	302.8	5420	3318	278.6	7350	3318	277.8	9960
3319	302.8	5430	3319	278.6	7350	3319	277.8	9960
3320	302.8	5440	3320	278.6	7340	3320	277.8	9980
3321	302.8	5420	3321	278.6	7360	3321	277.8	9970
3322	302.8	5420	3322	278.7	7350	3322	277.7	9960
3323	302.8	5430	3323	278.7	7350	3323	277.8	9950
3324	302.7	5420	3324	278.7	7350	3324	277.8	9950
3325	302.7	5440	3325	278.7	7350	3325	277.7	9950
3326	302.6	5420	3326	278.7	7340	3326	277.7	9960
3327	302.6	5420	3327	278.8	7360	3327	277.7	9960
3328	302.7	5420	3328	278.8	7350	3328	277.7	9970
3329	302.7	5420	3329	278.9	7350	3329	277.7	9960
3330	302.7	5420	3330	278.9	7350	3330	277.7	9960
3331	302.7	5430	3331	278.9	7360	3331	277.8	9970
3332	302.6	5430	3332	278.9	7360	3332	277.8	9960
3333	302.7	5420	3333	278.8	7360	3333	277.8	9960
3334	302.7	5430	3334	278.7	7350	3334	277.8	9970
3335	302.8	5420	3335	278.7	7360	3335	277.9	9970
3336	302.7	5430	3336	278.6	7370	3336	277.9	9970
3337	302.7	5420	3337	278.6	7350	3337	277.9	9950
3338	302.7	5420	3338	278.5	7340	3338	277.9	9950
3339	302.8	5420	3339	278.5	7350	3339	278	9950
3340	302.8	5420	3340	278.5	7340	3340	278	9960
3341	302.7	5420	3341	278.5	7340	3341	278.1	9960
3342	302.8	5440	3342	278.4	7350	3342	278.1	9950
3343	302.7	5430	3343	278.4	7350	3343	278.1	9950
3344	302.8	5440	3344	278.4	7360	3344	278.1	9960
3345	302.7	5420	3345	278.3	7350	3345	278.1	9970
3346	302.8	5430	3346	278.3	7340	3346	278.1	9970

3347	302.7	5430	3347	278.3	7360	3347	278.1	9970
3348	302.7	5420	3348	278.3	7350	3348	278.1	9960
3349	302.7	5430	3349	278.3	7350	3349	278.1	9960
3350	302.7	5430	3350	278.3	7360	3350	278.1	9960
3351	302.7	5430	3351	278.3	7350	3351	278.1	9970
3352	302.7	5420	3352	278.2	7350	3352	278.2	9960
3353	302.7	5420	3353	278.3	7360	3353	278.2	9960
3354	302.7	5430	3354	278.2	7370	3354	278.2	9960
3355	302.7	5430	3355	278.2	7370	3355	278.3	9970
3356	302.7	5440	3356	278.2	7370	3356	278.3	9960
3357	302.7	5440	3357	278.3	7360	3357	278.3	9960
3358	302.7	5440	3358	278.3	7360	3358	278.3	9970
3359	302.7	5420	3359	278.3	7350	3359	278.4	9960
3360	302.7	5440	3360	278.3	7350	3360	278.4	9960
3361	302.7	5440	3361	278.3	7370	3361	278.4	9970
3362	302.7	5430	3362	278.3	7370	3362	278.4	9960
3363	302.7	5450	3363	278.4	7360	3363	278.4	9960
3364	302.7	5450	3364	278.4	7350	3364	278.5	9960
3365	302.7	5440	3365	278.4	7350	3365	278.5	9960
3366	302.7	5440	3366	278.4	7360	3366	278.5	9970
3367	302.7	5430	3367	278.4	7350	3367	278.5	9960
3368	302.7	5450	3368	278.3	7350	3368	278.5	9970
3369	302.7	5440	3369	278.3	7360	3369	278.6	9960
3370	302.7	5430	3370	278.4	7360	3370	278.6	9950
3371	302.8	5410	3371	278.4	7350	3371	278.6	9950
3372	302.8	5430	3372	278.4	7370	3372	278.7	9950
3373	302.8	5430	3373	278.5	7350	3373	278.7	9960
3374	302.8	5430	3374	278.4	7360	3374	278.6	9960
3375	302.8	5420	3375	278.5	7360	3375	278.7	9970
3376	302.9	5440	3376	278.5	7360	3376	278.7	9970
3377	302.9	5440	3377	278.5	7350	3377	278.7	9960
3378	302.9	5430	3378	278.5	7360	3378	278.7	9960
3379	302.9	5430	3379	278.5	7350	3379	278.8	9970
3380	302.9	5430	3380	278.5	7360	3380	278.8	9960
3381	302.8	5420	3381	278.5	7350	3381	278.8	9970
3382	302.8	5430	3382	278.6	7340	3382	278.8	9960
3383	302.9	5420	3383	278.6	7360	3383	278.8	9960
3384	302.8	5420	3384	278.6	7350	3384	278.8	9970
3385	302.9	5420	3385	278.6	7360	3385	278.9	9960
3386	302.9	5420	3386	278.6	7350	3386	278.8	9960
3387	302.9	5430	3387	278.6	7360	3387	278.9	9970
3388	302.9	5420	3388	278.6	7350	3388	278.9	9960
3389	302.9	5430	3389	278.6	7360	3389	278.9	9960
3390	302.9	5420	3390	278.6	7360	3390	278.9	9970
3391	302.9	5420	3391	278.6	7340	3391	279	9960
3392	302.9	5420	3392	278.6	7350	3392	279	9950
3393	303	5420	3393	278.6	7350	3393	279	9950
3394	302.9	5420	3394	278.6	7350	3394	279.1	9960
3395	303	5420	3395	278.6	7360	3395	279	9970
3396	303	5440	3396	278.6	7360	3396	279.1	9970

3397	303	5420	3397	278.7	7360	3397	279.1	9970
3398	302.9	5420	3398	278.7	7350	3398	279.1	9960
3399	303	5430	3399	278.7	7360	3399	279.1	9960
3400	303	5430	3400	278.7	7350	3400	279.1	9960
3401	303	5430	3401	278.7	7350	3401	279.1	9960
3402	303	5440	3402	278.7	7340	3402	279.1	9950
3403	303	5420	3403	278.7	7350	3403	279.1	9950
3404	303	5430	3404	278.7	7350	3404	279.2	9940
3405	303	5430	3405	278.7	7340	3405	279.2	9940
3406	303	5420	3406	278.7	7360	3406	279.2	9950
3407	302.9	5420	3407	278.7	7350	3407	279.2	9960
3408	302.9	5430	3408	278.8	7350	3408	279.2	9960
3409	302.9	5430	3409	278.8	7360	3409	279.2	9960
3410	302.9	5430	3410	278.7	7350	3410	279.2	9970
3411	302.9	5430	3411	278.8	7350	3411	279.2	9950
3412	302.9	5430	3412	278.8	7350	3412	279.3	9950
3413	302.9	5430	3413	278.8	7340	3413	279.3	9960
3414	303	5430	3414	278.8	7360	3414	279.3	9960
3415	303	5430	3415	278.8	7350	3415	279.3	9970
3416	303	5440	3416	278.9	7370	3416	279.3	9970
3417	302.9	5420	3417	278.9	7360	3417	279.3	9970
3418	302.9	5430	3418	278.9	7370	3418	279.4	9960
3419	302.9	5430	3419	278.9	7370	3419	279.4	9950
3420	302.9	5430	3420	278.8	7360	3420	279.4	9960
3421	302.9	5430	3421	278.7	7370	3421	279.3	9960
3422	302.9	5420	3422	278.6	7360	3422	279.3	9970
3423	302.9	5440	3423	278.5	7350	3423	279.3	9960
3424	303	5430	3424	278.5	7360	3424	279.4	9950
3425	302.9	5430	3425	278.5	7360	3425	279.4	9950
3426	302.9	5430	3426	278.4	7360	3426	279.4	9940
3427	302.9	5430	3427	278.4	7360	3427	279.4	9950
3428	302.9	5420	3428	278.3	7350	3428	279.4	9940
3429	302.9	5330	3429	278.3	7350	3429	279.4	9960
3430	302.9	5430	3430	278.3	7360	3430	279.4	9960
3431	302.9	5430	3431	278.2	7360	3431	279.5	9970
3432	302.9	5420	3432	278.2	7370	3432	279.5	9960
3433	302.9	5430	3433	278.2	7350	3433	279.5	9960
3434	302.9	5430	3434	278.2	7370	3434	279.5	9960
3435	302.9	5420	3435	278.2	7360	3435	279.5	9970
3436	302.9	5420	3436	278.2	7360	3436	279.4	9940
3437	302.9	5430	3437	278.2	7350	3437	279.2	9960
3438	302.9	5430	3438	278.1	7350	3438	279.1	9950
3439	302.9	5430	3439	278.1	7350	3439	279	9960
3440	302.9	5420	3440	278.1	7370	3440	278.9	9960
3441	302.9	5440	3441	278.1	7360	3441	278.8	9960
3442	302.9	5430	3442	278.1	7350	3442	278.7	9940
3443	302.9	5420	3443	278.1	7350	3443	278.6	9950
3444	302.9	5430	3444	278.1	7350	3444	278.6	9940
3445	302.9	5430	3445	278.1	7360	3445	278.5	9960
3446	302.9	5430	3446	278.1	7350	3446	278.4	9960

3447	302.9	5420	3447	278.1	7360	3447	278.4	9960
3448	302.9	5430	3448	278	7360	3448	278.3	9940
3449	302.9	5440	3449	278.1	7360	3449	278.3	9950
3450	302.9	5440	3450	278.1	7360	3450	278.3	9960
3451	303	5430	3451	278.1	7360	3451	278.2	9950
3452	303	5340	3452	278.1	7360	3452	278.2	9950
3453	303	5420	3453	278.1	7360	3453	278.2	9950
3454	303	5430	3454	278.1	7350	3454	278.2	9950
3455	303	5440	3455	278.1	7360	3455	278.2	9940
3456	303	5430	3456	278.1	7360	3456	278.2	9950
3457	303	5430	3457	278.1	7360	3457	278.2	9960
3458	303	5440	3458	278.1	7360	3458	278.2	9970
3459	303	5430	3459	278.1	7360	3459	278.2	9950
3460	303	5440	3460	278.1	7370	3460	278.2	9970
3461	303	5440	3461	278.1	7360	3461	278.1	9960
3462	302.9	5340	3462	278.1	7370	3462	278.1	9950
3463	302.9	5440	3463	278.1	7360	3463	278.1	9950
3464	302.9	5440	3464	278.1	7360	3464	278	9950
3465	302.9	5330	3465	278.2	7360	3465	278.1	9960
3466	302.9	5440	3466	278.2	7360	3466	278.1	9950
3467	302.9	5430	3467	278.2	7350	3467	278	9960
3468	303.3	5430	3468	278.1	7350	3468	278	9950
3469	303.7	5430	3469	278.1	7360	3469	278	9960
3470	304.3	5420	3470	278.1	7350	3470	278	9960
3471	304.8	5420	3471	278.1	7360	3471	278	9960
3472	305.2	5410	3472	278.1	7350	3472	278	9950
3473	305.6	5420	3473	278.2	7350	3473	278	9950
3474	305.9	5410	3474	278.2	7360	3474	278	9970
3475	306.3	5420	3475	278.1	7350	3475	278	9960
3476	306.6	5420	3476	278.1	7350	3476	278	9960
3477	306.9	5440	3477	278.1	7340	3477	278	9970
3478	307.1	5420	3478	278.1	7350	3478	278	9950
3479	307.3	5420	3479	278.1	7350	3479	277.9	9860
3480	307.6	5410	3480	278.1	7360	3480	277.9	9960
3481	307.8	5520	3481	278.1	7360	3481	277.9	9950
3482	308.1	5410	3482	278.1	7360	3482	277.9	9960
3483	308.3	5420	3483	278.1	7360	3483	277.9	9960
3484	308.4	5430	3484	278.1	7360	3484	278	9960
3485	308.6	5530	3485	278.1	7370	3485	277.9	9960
3486	308.7	5420	3486	278.1	7360	3486	277.9	9850
3487	308.8	5520	3487	278.1	7360	3487	278	9960
3488	308.9	5510	3488	278.1	7350	3488	278	9960
3489	309	5520	3489	278.1	7370	3489	278	9940
3490	309.1	5520	3490	278.2	7370	3490	277.9	9950
3491	309.2	5510	3491	278.2	7360	3491	278	9850
3492	309.3	5410	3492	278.2	7360	3492	278	9970
3493	309.5	5520	3493	278.2	7360	3493	277.9	9960
3494	309.5	5520	3494	278.2	7350	3494	277.9	9960
3495	309.7	5510	3495	278.2	7350	3495	277.8	9950
3496	309.7	5520	3496	278.2	7360	3496	277.8	9950

3497	309.9	5520	3497	278.2	7360	3497	277.8	9860
3498	309.9	5520	3498	278.2	7360	3498	277.8	9870
3499	310	5530	3499	278.3	7350	3499	277.8	9870
3500	310.1	5510	3500	278.3	7360	3500	277.8	9970
3501	310.1	5520	3501	278.2	7370	3501	277.8	9860
3502	310.2	5520	3502	278.3	7350	3502	277.8	9970
3503	310.2	5520	3503	278.2	7340	3503	277.8	9860
3504	310.3	5530	3504	278.2	7350	3504	277.8	9960
3505	310.4	5520	3505	278.2	7360	3505	277.8	9970
3506	310.4	5520	3506	278.3	7360	3506	277.8	9860
3507	310.4	5530	3507	278.3	7360	3507	277.8	9850
3508	310.4	5530	3508	278.3	7350	3508	277.8	9860
3509	310.5	5530	3509	278.3	7370	3509	277.7	9850
3510	310.6	5520	3510	278.3	7350	3510	277.8	9950
3511	310.7	5520	3511	278.3	7350	3511	277.8	9860
3512	310.8	5510	3512	278.3	7340	3512	277.8	9950
3513	310.8	5520	3513	278.2	7340	3513	277.8	9860
3514	310.9	5530	3514	278.2	7360	3514	277.8	9860
3515	311	5530	3515	278.2	7360	3515	277.8	9860
3516	311	5530	3516	278.3	7370	3516	277.8	9860
3517	311.1	5520	3517	278.3	7370	3517	277.8	9860
3518	311.1	5520	3518	278.3	7370	3518	277.8	9860
3519	311.2	5520	3519	278.3	7370	3519	277.8	9860
3520	311.3	5520	3520	278.3	7350	3520	277.8	9860
3521	311.4	5510	3521	278.3	7350	3521	277.8	9870
3522	311.4	5520	3522	278.2	7360	3522	277.8	9850
3523	311.4	5510	3523	278.2	7360	3523	277.8	9850
3524	311.4	5510	3524	278.1	7350	3524	277.8	9850
3525	311.5	5520	3525	278.2	7350	3525	277.9	9850
3526	311.5	5520	3526	278.2	7350	3526	277.9	9860
3527	311.6	5510	3527	278.2	7350	3527	277.8	9860
3528	311.8	5510	3528	278.2	7350	3528	277.7	9850
3529	312.1	5520	3529	278.2	7340	3529	277.7	9860
3530	312.5	5510	3530	278.2	7360	3530	277.7	9840
3531	312.9	5510	3531	278.2	7360	3531	277.7	9850
3532	313.3	5510	3532	278.2	7350	3532	277.7	9960
3533	313.7	5510	3533	278.2	7360	3533	277.7	9850
3534	314.2	5610	3534	278.2	7370	3534	277.7	9860
3535	314.5	5520	3535	278.2	7350	3535	277.7	9850
3536	314.9	5620	3536	278.2	7350	3536	277.7	9850
3537	315.3	5610	3537	278.2	7360	3537	277.7	9880
3538	315.6	5520	3538	278.2	7360	3538	277.7	9860
3539	315.9	5600	3539	278.3	7350	3539	277.8	9860
3540	316.3	5610	3540	278.2	7350	3540	277.8	9860
3541	316.6	5610	3541	278.2	7350	3541	277.8	9960
3542	316.9	5610	3542	278.2	7360	3542	277.8	9860
3543	317.1	5610	3543	278.2	7350	3543	277.8	9950
3544	317.4	5620	3544	278.2	7340	3544	277.8	9860
3545	317.7	5610	3545	278.2	7350	3545	277.8	9860
3546	317.9	5610	3546	278.1	7360	3546	277.8	9940

3547	318.1	5600	3547	278.1	7350	3547	277.8	9950
3548	318.3	5610	3548	278.2	7360	3548	277.8	9940
3549	318.5	5610	3549	278.2	7350	3549	277.8	9960
3550	318.6	5610	3550	278.2	7360	3550	277.8	9950
3551	318.8	5600	3551	278.2	7360	3551	277.8	9950
3552	319	5600	3552	278.2	7360	3552	277.8	9950
3553	319.1	5610	3553	278.2	7350	3553	277.8	9870
3554	319.2	5600	3554	278.3	7360	3554	277.8	9860
3555	319.4	5610	3555	278.3	7360	3555	277.8	9960
3556	319.6	5600	3556	278.3	7360	3556	277.8	9860
3557	319.7	5610	3557	278.3	7350	3557	277.8	9870
3558	319.8	5610	3558	278.3	7350	3558	277.8	9870
3559	319.9	5610	3559	278.3	7350	3559	277.7	9860
3560	320.1	5610	3560	278.3	7350	3560	277.8	9950
3561	320.2	5610	3561	278.3	7360	3561	277.8	9960
3562	320.3	5610	3562	278.3	7350	3562	277.8	9860
3563	320.4	5610	3563	278.3	7360	3563	277.8	9860
3564	320.4	5590	3564	278.3	7350	3564	277.8	9960
3565	320.5	5600	3565	278.3	7370	3565	277.7	9860
3566	320.5	5600	3566	278.2	7360	3566	277.8	9870
3567	320.6	5610	3567	278.2	7350	3567	277.8	9860
3568	320.8	5620	3568	278.2	7360	3568	277.8	9860
3569	320.9	5610	3569	278.2	7350	3569	277.8	9860
3570	321	5610	3570	278.2	7360	3570	277.8	9860
3571	321.1	5610	3571	278.2	7350	3571	277.8	9850
3572	321.2	5600	3572	278.2	7360	3572	277.8	9850
3573	321.2	5610	3573	278.2	7360	3573	277.9	9850
3574	321.2	5620	3574	278.2	7360	3574	277.8	9850
3575	321.1	5610	3575	278.2	7360	3575	277.8	9860
3576	321	5620	3576	278.2	7360	3576	277.8	9860
3577	320.9	5600	3577	278.2	7360	3577	277.8	9850
3578	320.9	5610	3578	278.3	7350	3578	277.9	9860
3579	320.9	5610	3579	278.3	7360	3579	277.8	9860
3580	320.9	5600	3580	278.3	7350	3580	277.9	9950
3581	320.9	5590	3581	278.3	7350	3581	277.9	9860
3582	320.9	5600	3582	278.3	7360	3582	277.9	9860
3583	320.9	5620	3583	278.4	7370	3583	277.9	9860
3584	321	5610	3584	278.3	7360	3584	277.9	9850
3585	320.9	5620	3585	278.3	7350	3585	277.9	9960
3586	320.9	5610	3586	278.4	7350	3586	278	9960
3587	320.9	5600	3587	278.4	7360	3587	278	9960
3588	320.9	5610	3588	278.3	7360	3588	277.9	9860
3589	320.9	5610	3589	278.3	7350	3589	277.9	9840
3590	321	5620	3590	278.3	7360	3590	277.9	9860
3591	321	5630	3591	278.3	7360	3591	277.9	9860
3592	321	5620	3592	278.3	7350	3592	277.9	9850
3593	321	5610	3593	278.3	7360	3593	277.9	9860
3594	321	5610	3594	278.3	7350	3594	277.9	9850
3595	321	5600	3595	278.3	7350	3595	277.9	9960
3596	321	5610	3596	278.2	7330	3596	277.9	9850

3597	321	5610	3597	278.2	7350	3597	277.9	9860
3598	321.1	5620	3598	278.2	7370	3598	277.8	9860
3599	321	5600	3599	278.2	7360	3599	277.8	9850
3600	321.1	5600	3600	278.3	7360	3600	277.8	9860
3601	321.1	5610	3601	278.3	7350	3601	277.8	9860
3602	321.2	5620	3602	278.3	7360	3602	277.8	9840
3603	321.2	5600	3603	278.3	7350	3603	277.8	9850
3604	321.3	5610	3604	278.3	7350	3604	277.8	9860
3605	321.4	5600	3605	278.3	7350	3605	277.8	9860
3606	321.4	5600	3606	278.3	7350	3606	277.7	9860
3607	321.5	5600	3607	278.3	7350	3607	277.7	9960
3608	321.5	5600	3608	278.3	7350	3608	277.8	9860
3609	321.5	5600	3609	278.3	7350	3609	277.8	9860
3610	321.6	5610	3610	278.3	7370	3610	277.8	9840
3611	321.6	5600	3611	278.3	7360	3611	277.9	9840
3612	321.7	5610	3612	278.3	7350	3612	277.8	9840
3613	321.7	5600	3613	278.3	7350	3613	277.8	9860
3614	321.7	5610	3614	278.4	7350	3614	277.8	9850
3615	321.8	5610	3615	278.4	7360	3615	277.8	9850
3616	321.8	5610	3616	278.4	7350	3616	277.8	9840
3617	321.8	5620	3617	278.4	7370	3617	277.8	9950
3618	321.9	5610	3618	278.4	7360	3618	277.8	9960
3619	321.9	5600	3619	278.4	7360	3619	277.8	9950
3620	321.9	5600	3620	278.4	7350	3620	277.8	9850
3621	321.9	5610	3621	278.4	7340	3621	277.8	9940
3622	322	5610	3622	278.5	7340	3622	277.9	9850
3623	322	5610	3623	278.5	7350	3623	277.9	9850
3624	322	5600	3624	278.5	7360	3624	277.9	9850
3625	322	5610	3625	278.5	7350	3625	277.9	9960
3626	322	5610	3626	278.5	7350	3626	277.9	9970
3627	322	5610	3627	278.5	7350	3627	277.9	9950
3628	322	5610	3628	278.5	7360	3628	277.9	9940
3629	322	5610	3629	278.5	7360	3629	277.9	9950
3630	322.1	5610	3630	278.5	7360	3630	278	9950
3631	322.2	5620	3631	278.5	7340	3631	278	9950
3632	322.1	5610	3632	278.5	7360	3632	278	9950
3633	322.2	5610	3633	278.5	7360	3633	278	9940
3634	322.3	5610	3634	278.5	7360	3634	278	9950
3635	322.3	5610	3635	278.5	7360	3635	278	9950
3636	322.4	5620	3636	278.5	7350	3636	277.9	9960
3637	322.4	5610	3637	278.6	7350	3637	277.9	9850
3638	322.4	5610	3638	278.6	7350	3638	277.9	9950
3639	322.4	5590	3639	278.6	7350	3639	278	9960
3640	322.4	5600	3640	278.6	7360	3640	278	9950
3641	322.5	5610	3641	278.6	7360	3641	278	9950
3642	322.5	5610	3642	278.6	7350	3642	278	9940
3643	322.5	5600	3643	278.6	7350	3643	278	9940
3644	322.5	5610	3644	278.6	7350	3644	278	9940
3645	322.6	5600	3645	278.6	7360	3645	278	9950
3646	322.6	5610	3646	278.6	7350	3646	278	9960

3647	322.7	5600	3647	278.6	7340	3647	278	9950
3648	322.7	5610	3648	278.6	7360	3648	277.9	9970
3649	322.7	5620	3649	278.6	7350	3649	277.8	9970
3650	322.7	5600	3650	278.6	7350	3650	277.8	9960
3651	322.8	5610	3651	278.6	7350	3651	277.7	9960
3652	322.8	5610	3652	278.6	7360	3652	277.6	9960
3653	322.8	5610	3653	278.5	7350	3653	277.6	9970
3654	322.8	5610	3654	278.5	7350	3654	277.5	9960
3655	322.9	5610	3655	278.5	7370	3655	277.4	9950
3656	322.8	5620	3656	278.5	7350	3656	277.4	9960
3657	322.9	5610	3657	278.5	7350	3657	277.4	9960
3658	323	5620	3658	278.5	7350	3658	277.4	9960
3659	323	5610	3659	278.5	7360	3659	277.4	9960
3660	323	5620	3660	278.6	7340	3660	277.4	9950
3661	323	5620	3661	278.5	7350	3661	277.4	9940
3662	323	5600	3662	278.6	7340	3662	277.4	9950
3663	323	5610	3663	278.5	7350	3663	277.4	9950
3664	323.1	5620	3664	278.6	7350	3664	277.4	9950
3665	323.1	5610	3665	278.6	7360	3665	277.4	9960
3666	323.1	5600	3666	278.6	7350	3666	277.4	9960
3667	323.2	5610	3667	278.5	7360	3667	277.3	9960
3668	323.2	5610	3668	278.5	7360	3668	277.3	9960
3669	323.2	5620	3669	278.5	7350	3669	277.2	9960
3670	323.2	5610	3670	278.5	7360	3670	277.2	9950
3671	323.3	5610	3671	278.6	7350	3671	277.2	9970
3672	323.4	5610	3672	278.5	7360	3672	277.2	9960
3673	323.4	5610	3673	278.5	7350	3673	277.1	9960
3674	323.4	5600	3674	278.6	7360	3674	277.2	9950
3675	323.4	5610	3675	278.5	7350	3675	277.1	9960
3676	323.4	5600	3676	278.5	7350	3676	277.1	9960
3677	323.3	5600	3677	278.5	7360	3677	277.1	9960
3678	323.3	5600	3678	278.5	7360	3678	277.1	9950
3679	323.3	5620	3679	278.5	7360	3679	277.1	9960
3680	323.4	5600	3680	278.5	7360	3680	277	9950
3681	323.4	5610	3681	278.5	7360	3681	277	9950
3682	323.4	5610	3682	278.5	7350	3682	277.1	9960
3683	323.4	5620	3683	278.5	7360	3683	277.1	9940
3684	323.4	5610	3684	278.5	7350	3684	277	9950
3685	323.4	5620	3685	278.5	7350	3685	277	9950
3686	323.4	5610	3686	278.5	7340	3686	277	9960
3687	323.4	5610	3687	278.5	7340	3687	277	9950
3688	323.4	5600	3688	278.6	7350	3688	277	9960
3689	323.4	5610	3689	278.6	7340	3689	277	9950
3690	323.4	5610	3690	278.6	7350	3690	277	9960
3691	323.5	5610	3691	278.6	7350	3691	277	9850
3692	323.5	5610	3692	278.6	7350	3692	277	9850
3693	323.5	5600	3693	278.5	7360	3693	277	9940
3694	323.5	5620	3694	278.6	7350	3694	277	9960
3695	323.6	5610	3695	278.6	7350	3695	277	9950
3696	323.7	5600	3696	278.6	7350	3696	277	9960

3697	323.7	5610	3697	278.6	7360	3697	277	9950
3698	323.7	5600	3698	278.6	7370	3698	277	9850
3699	323.7	5600	3699	278.6	7350	3699	277	9860
3700	323.7	5620	3700	278.6	7360	3700	277	9950
3701	323.8	5610	3701	278.6	7360	3701	276.9	9870
3702	323.8	5590	3702	278.6	7350	3702	277	9860
3703	323.8	5610	3703	278.6	7370	3703	277	9860
3704	323.8	5620	3704	278.6	7360	3704	276.9	9950
3705	323.8	5610	3705	278.6	7350	3705	276.9	9850
3706	323.8	5610	3706	278.7	7350	3706	276.9	9850
3707	323.8	5610	3707	278.7	7360	3707	276.9	9950
3708	323.9	5610	3708	278.7	7350	3708	276.9	9970
3709	323.9	5600	3709	278.7	7350	3709	276.9	9860
3710	323.8	5610	3710	278.7	7250	3710	276.9	9860
3711	323.9	5610	3711	278.6	7350	3711	277	9860
3712	323.9	5610	3712	278.6	7360	3712	277	9860
3713	324	5610	3713	278.6	7350	3713	276.9	9960
3714	324	5610	3714	278.7	7350	3714	276.9	9860
3715	324	5610	3715	278.6	7350	3715	277	9950
3716	324	5610	3716	278.6	7360	3716	276.9	9960
3717	324	5610	3717	278.6	7360	3717	277	9950
3718	324.1	5610	3718	278.6	7350	3718	277	9950
3719	324.2	5610	3719	278.6	7350	3719	277	9860
3720	324.3	5610	3720	278.7	7350	3720	277	9950
3721	324.3	5600	3721	278.7	7350	3721	277	9850
3722	324.4	5610	3722	278.7	7350	3722	277	9860
3723	324.3	5600	3723	278.7	7350	3723	277	9860
3724	324.3	5620	3724	278.7	7340	3724	277	9850
3725	324.4	5610	3725	278.7	7350	3725	276.9	9850
3726	324.4	5610	3726	278.7	7340	3726	276.9	9850
3727	324.4	5600	3727	278.7	7350	3727	276.9	9860
3728	324.4	5610	3728	278.8	7350	3728	276.9	9840
3729	324.4	5600	3729	278.7	7350	3729	276.8	9860
3730	324.4	5600	3730	278.8	7350	3730	276.8	9840
3731	324.4	5610	3731	278.8	7350	3731	276.8	9860
3732	324.4	5620	3732	278.8	7350	3732	276.9	9950
3733	324.4	5620	3733	278.8	7360	3733	276.9	9950
3734	324.4	5600	3734	278.9	7360	3734	276.8	9860
3735	324.5	5610	3735	278.8	7360	3735	276.8	9850
3736	324.5	5590	3736	278.8	7360	3736	276.8	9860
3737	324.4	5600	3737	278.8	7350	3737	276.8	9960
3738	324.5	5600	3738	278.8	7360	3738	276.8	9860
3739	324.5	5610	3739	278.8	7340	3739	276.8	9960
3740	324.5	5600	3740	278.8	7350	3740	276.8	9950
3741	324.5	5610	3741	278.8	7350	3741	276.7	9850
3742	324.5	5610	3742	278.7	7340	3742	276.7	9850
3743	324.5	5610	3743	278.8	7340	3743	276.7	9850
3744	324.6	5600	3744	278.7	7350	3744	276.7	9850
3745	324.6	5600	3745	278.7	7350	3745	276.7	9850
3746	324.6	5600	3746	278.8	7350	3746	276.7	9840

3747	324.6	5590	3747	278.8	7350	3747	276.8	9840
3748	324.6	5600	3748	278.8	7360	3748	276.8	9850
3749	324.6	5600	3749	278.9	7360	3749	276.8	9860
3750	324.6	5600	3750	278.9	7350	3750	276.8	9860
3751	324.6	5600	3751	278.8	7340	3751	276.8	9850
3752	324.6	5600	3752	278.9	7360	3752	276.8	9850
3753	324.6	5600	3753	278.8	7340	3753	276.8	9850
3754	324.6	5600	3754	278.9	7350	3754	276.8	9850
3755	324.6	5600	3755	278.9	7350	3755	276.7	9850
3756	324.6	5600	3756	278.9	7340	3756	276.7	9860
3757	324.5	5610	3757	278.9	7340	3757	276.7	9840
3758	324.6	5590	3758	278.9	7350	3758	276.7	9850
3759	324.6	5600	3759	278.9	7350	3759	276.7	9850
3760	324.6	5600	3760	278.9	7360	3760	276.7	9860
3761	324.6	5610	3761	278.9	7350	3761	276.7	9850
3762	324.7	5610	3762	278.9	7360	3762	276.8	9860
3763	324.7	5610	3763	278.9	7360	3763	276.7	9860
3764	324.8	5610	3764	278.9	7350	3764	276.8	9880
3765	324.7	5610	3765	278.9	7350	3765	276.8	9860
3766	324.7	5600	3766	278.9	7350	3766	276.8	9860
3767	324.7	5590	3767	278.9	7360	3767	276.7	9860
3768	324.7	5600	3768	278.9	7350	3768	276.8	9860
3769	324.8	5610	3769	278.9	7360	3769	276.7	9880
3770	324.8	5610	3770	278.9	7350	3770	276.7	9870
3771	324.8	5590	3771	278.9	7340	3771	276.7	9870
3772	324.9	5610	3772	279	7350	3772	276.7	9850
3773	325	5610	3773	279	7350	3773	276.7	9870
3774	325	5600	3774	279	7340	3774	276.7	9990
3775	325	5600	3775	279	7350	3775	276.7	9870
3776	325	5600	3776	279	7340	3776	276.7	9850
3777	325	5600	3777	279	7350	3777	276.8	9880
3778	325	5610	3778	278.9	7340	3778	276.7	9870
3779	325	5610	3779	279	7350	3779	276.8	9860
3780	325	5600	3780	278.9	7360	3780	276.7	9860
3781	325.1	5600	3781	278.9	7340	3781	276.7	9860
3782	325.1	5600	3782	278.9	7340	3782	276.7	9870
3783	325.1	5600	3783	278.9	7360	3783	276.7	9880
3784	325	5600	3784	278.9	7340	3784	276.7	9880
3785	325.1	5600	3785	278.9	7350	3785	276.7	9890
3786	325.1	5600	3786	278.9	7340	3786	276.7	9880
3787	325.1	5600	3787	278.9	7350	3787	276.7	9890
3788	325.1	5590	3788	278.9	7340	3788	276.7	9890
3789	325.1	5600	3789	278.9	7350	3789	276.7	9890
3790	325.1	5600	3790	278.9	7340	3790	276.7	9850
3791	325.1	5600	3791	278.8	7350	3791	276.7	9880
3792	325.1	5600	3792	278.9	7350	3792	276.7	9890
3793	325.1	5600	3793	278.8	7350	3793	276.7	9900
3794	325.1	5610	3794	278.8	7350	3794	276.7	9890
3795	325.1	5610	3795	278.8	7350	3795	276.7	9890
3796	325.1	5610	3796	278.8	7350	3796	276.7	9890

3797	325.1	5600	3797	278.8	7350	3797	276.7	9880
3798	325.2	5600	3798	278.8	7360	3798	276.7	9890
3799	325.1	5590	3799	278.8	7340	3799	276.7	9900
3800	325.1	5600	3800	278.8	7340	3800	276.6	9890
3801	325.2	5600	3801	278.8	7350	3801	276.7	9900
3802	325.1	5600	3802	278.8	7340	3802	276.7	9880
3803	325.2	5590	3803	278.7	7360	3803	276.7	9890
3804	325.2	5610	3804	278.8	7340	3804	276.6	9890
3805	325.2	5600	3805	278.8	7360	3805	276.6	9890
3806	325.2	5600	3806	278.8	7350	3806	276.7	9890
3807	325.3	5580	3807	278.8	7350	3807	276.6	9880
3808	325.3	5600	3808	278.8	7360	3808	276.7	9890
3809	325.3	5600	3809	278.7	7360	3809	276.6	9880
3810	325.3	5610	3810	278.7	7370	3810	276.6	9890
3811	325.2	5590	3811	278.7	7350	3811	276.7	9890
3812	325.2	5600	3812	278.7	7360	3812	276.6	9900
3813	325.2	5590	3813	278.7	7350	3813	276.7	9910
3814	325.2	5610	3814	278.7	7350	3814	276.7	9890
3815	325.2	5590	3815	278.8	7350	3815	276.7	9900
3816	325.2	5600	3816	278.8	7360	3816	276.7	9890
3817	325.3	5600	3817	278.8	7360	3817	276.7	9890
3818	325.3	5600	3818	278.8	7350	3818	276.7	9890
3819	325.3	5590	3819	278.8	7350	3819	276.7	9900
3820	325.3	5600	3820	278.8	7360	3820	276.7	9900
3821	325.3	5600	3821	278.8	7360	3821	276.7	9890
3822	325.3	5590	3822	278.8	7360	3822	276.6	9890
3823	325.3	5610	3823	278.8	7360	3823	276.6	9890
3824	325.4	5600	3824	278.8	7360	3824	276.6	9890
3825	325.4	5610	3825	278.8	7360	3825	276.6	9880
3826	325.4	5600	3826	278.8	7350	3826	276.6	9890
3827	325.4	5610	3827	278.8	7350	3827	276.6	9890
3828	325.4	5600	3828	278.8	7350	3828	276.6	9890
3829	325.4	5610	3829	278.8	7360	3829	276.6	9900
3830	325.4	5600	3830	278.8	7360	3830	276.6	9900
3831	325.5	5590	3831	278.8	7360	3831	276.6	9890
3832	325.5	5590	3832	278.8	7360	3832	276.6	9900
3833	325.5	5600	3833	278.8	7360	3833	276.6	9880
3834	325.5	5590	3834	278.8	7360	3834	276.6	9880
3835	325.5	5600	3835	278.8	7360	3835	276.6	9890
3836	325.6	5600	3836	278.8	7350	3836	276.6	9900
3837	325.6	5600	3837	278.8	7350	3837	276.6	9890
3838	325.6	5590	3838	278.8	7360	3838	276.6	9890
3839	325.6	5610	3839	278.8	7370	3839	276.6	9900
3840	325.5	5600	3840	278.8	7350	3840	276.6	9880
3841	325.5	5610	3841	278.8	7360	3841	276.6	9890
3842	325.6	5600	3842	278.9	7360	3842	276.6	9890
3843	325.5	5610	3843	278.8	7340	3843	276.6	9880
3844	325.6	5590	3844	278.8	7350	3844	276.6	9880
3845	325.5	5610	3845	278.8	7360	3845	276.6	9890
3846	325.6	5600	3846	278.8	7360	3846	276.6	9900

3847	325.6	5600	3847	278.8	7350	3847	276.6	9890
3848	325.5	5600	3848	278.8	7350	3848	276.6	9900
3849	325.5	5600	3849	278.8	7340	3849	276.6	9880
3850	325.6	5590	3850	278.8	7350	3850	276.7	9890
3851	325.6	5590	3851	278.8	7340	3851	276.7	9890
3852	325.6	5600	3852	278.9	7350	3852	276.7	9890
3853	325.6	5590	3853	278.8	7350	3853	276.7	9890
3854	325.6	5600	3854	278.8	7340	3854	276.7	9890
3855	325.6	5600	3855	278.8	7350	3855	276.7	9890
3856	325.6	5590	3856	278.8	7350	3856	276.7	9900
3857	325.6	5600	3857	278.8	7340	3857	276.8	9880
3858	325.6	5600	3858	278.8	7340	3858	276.8	9880
3859	325.6	5610	3859	278.8	7350	3859	276.8	9880
3860	325.6	5590	3860	278.8	7350	3860	276.8	9880
3861	325.6	5590	3861	278.8	7350	3861	276.8	9880
3862	325.6	5480	3862	278.8	7340	3862	276.7	9900
3863	325.6	5580	3863	278.8	7350	3863	276.7	9890
3864	325.5	5600	3864	278.8	7340	3864	276.7	9890
3865	325.5	5600	3865	278.8	7360	3865	276.7	9870
3866	325.6	5610	3866	278.9	7340	3866	276.7	9890
3867	325.7	5600	3867	278.9	7360	3867	276.7	9880
3868	325.7	5600	3868	278.9	7340	3868	276.7	9890
3869	325.7	5590	3869	278.9	7360	3869	276.7	9890
3870	325.7	5590	3870	278.9	7350	3870	276.7	9890
3871	325.7	5610	3871	278.9	7360	3871	276.7	9900
3872	325.8	5580	3872	278.9	7350	3872	276.7	9870
3873	325.8	5590	3873	278.9	7360	3873	276.7	9880
3874	325.8	5590	3874	279	7360	3874	276.7	9880
3875	325.9	5590	3875	278.9	7350	3875	276.7	9890
3876	325.8	5580	3876	278.9	7360	3876	276.7	9880
3877	325.8	5580	3877	278.9	7350	3877	276.8	9880
3878	325.8	5590	3878	278.9	7360	3878	276.8	9880
3879	325.9	5610	3879	278.9	7350	3879	276.8	9880
3880	325.9	5600	3880	278.9	7350	3880	276.8	9890
3881	325.9	5610	3881	278.9	7360	3881	276.8	9890
3882	325.9	5600	3882	278.8	7350	3882	276.8	9900
3883	325.9	5600	3883	278.9	7350	3883	276.8	9890
3884	325.9	5600	3884	278.9	7350	3884	276.7	9890
3885	325.9	5590	3885	278.9	7350	3885	276.8	9880
3886	325.9	5600	3886	278.9	7350	3886	276.8	9880
3887	325.9	5500	3887	278.9	7360	3887	276.7	9890
3888	325.9	5600	3888	278.8	7350	3888	276.8	9880
3889	326	5600	3889	278.8	7360	3889	276.7	9890
3890	326	5600	3890	278.8	7360	3890	276.7	9890
3891	326	5590	3891	278.8	7350	3891	276.8	9890
3892	325.9	5610	3892	278.8	7360	3892	276.7	9890
3893	325.9	5600	3893	278.9	7360	3893	276.8	9890
3894	325.9	5590	3894	278.8	7350	3894	276.8	9890
3895	326	5590	3895	278.8	7350	3895	276.8	9890
3896	326	5590	3896	278.8	7340	3896	276.8	9890

3897	326	5490	3897	278.8	7350	3897	276.8	9890
3898	326	5500	3898	278.8	7340	3898	276.7	9890
3899	326	5600	3899	278.8	7350	3899	276.7	9890
3900	326	5500	3900	278.8	7350	3900	276.7	9890
3901	326	5600	3901	278.8	7340	3901	276.7	9880
3902	326	5600	3902	278.8	7350	3902	276.7	9890
3903	326	5590	3903	278.8	7350	3903	276.7	9880
3904	326	5580	3904	278.8	7350	3904	276.7	9890
3905	326	5590	3905	278.8	7350	3905	276.8	9890
3906	326.1	5600	3906	278.8	7360	3906	276.7	9880
3907	326.1	5590	3907	278.8	7350	3907	276.7	9890
3908	326.2	5600	3908	278.8	7350	3908	276.7	9880
3909	326.1	5590	3909	278.8	7350	3909	276.7	9900
3910	326.1	5600	3910	278.8	7350	3910	276.7	9890
3911	326.2	5500	3911	278.8	7350	3911	276.7	9880
3912	326.2	5490	3912	278.9	7350	3912	276.7	9890
3913	326.2	5480	3913	278.9	7350	3913	276.8	9880
3914	326.3	5590	3914	278.9	7350	3914	276.8	9890
3915	326.2	5590	3915	278.9	7360	3915	276.8	9880
3916	326.3	5600	3916	278.9	7350	3916	276.7	9880
3917	326.3	5600	3917	278.9	7350	3917	276.8	9890
3918	326.3	5610	3918	278.9	7350	3918	276.7	9890
3919	326.3	5590	3919	278.9	7360	3919	276.8	9870
3920	326.3	5590	3920	278.9	7340	3920	276.8	9880
3921	326.2	5600	3921	278.9	7340	3921	276.8	9890
3922	326.2	5600	3922	278.8	7350	3922	276.7	9880
3923	326.2	5600	3923	278.8	7350	3923	276.8	9880
3924	326.2	5610	3924	278.8	7350	3924	276.8	9900
3925	326.1	5600	3925	278.9	7340	3925	276.8	9890
3926	326.2	5600	3926	278.9	7350	3926	276.8	9890
3927	326.2	5610	3927	278.9	7340	3927	276.8	9890
3928	326.2	5610	3928	278.9	7350	3928	276.8	9890
3929	326.2	5510	3929	278.9	7360	3929	276.8	9880
3930	326.2	5500	3930	278.9	7340	3930	276.8	9880
3931	326.2	5610	3931	278.9	7360	3931	276.7	9880
3932	326.2	5610	3932	278.9	7340	3932	276.8	9900
3933	326.2	5600	3933	278.9	7340	3933	276.8	9890
3934	326.2	5600	3934	278.9	7360	3934	276.8	9880
3935	326.2	5580	3935	278.9	7350	3935	276.8	9890
3936	326.2	5590	3936	278.9	7340	3936	276.8	9870
3937	326.2	5600	3937	278.8	7330	3937	276.8	9880
3938	326.2	5600	3938	278.8	7340	3938	276.8	9900
3939	326.3	5600	3939	278.8	7330	3939	276.8	9890
3940	326.3	5610	3940	278.8	7340	3940	276.8	9900
3941	326.3	5600	3941	278.8	7340	3941	276.9	9890
3942	326.3	5610	3942	278.8	7350	3942	276.9	9880
3943	326.3	5580	3943	278.8	7350	3943	276.8	9880
3944	326.3	5590	3944	278.8	7350	3944	276.8	9890
3945	326.3	5590	3945	278.8	7350	3945	276.8	9890
3946	326.3	5600	3946	278.8	7340	3946	276.8	9890

3947	326.3	5600	3947	278.8	7330	3947	276.8	9900
3948	326.3	5600	3948	278.8	7350	3948	276.8	9880
3949	326.3	5600	3949	278.8	7350	3949	276.8	9880
3950	326.3	5500	3950	278.8	7350	3950	276.8	9890
3951	326.4	5600	3951	278.8	7360	3951	276.8	9890
3952	326.3	5490	3952	278.8	7350	3952	276.8	9890
3953	326.4	5600	3953	278.8	7340	3953	276.8	9890
3954	326.4	5590	3954	278.8	7340	3954	276.8	9900
3955	326.5	5590	3955	278.8	7350	3955	276.8	9880
3956	326.4	5610	3956	278.8	7350	3956	276.8	9880
3957	326.4	5590	3957	278.9	7340	3957	276.8	9880
3958	326.4	5600	3958	278.9	7340	3958	276.8	9890
3959	326.3	5600	3959	278.9	7340	3959	276.8	9870
3960	326.3	5600	3960	278.9	7340	3960	276.8	9880
3961	326.3	5610	3961	278.9	7350	3961	276.8	9880
3962	326.4	5600	3962	278.8	7350	3962	276.8	9890
3963	326.3	5610	3963	278.9	7340	3963	276.8	9880
3964	326.3	5600	3964	278.9	7350	3964	276.8	9880
3965	326.4	5510	3965	278.9	7340	3965	276.8	9880
3966	326.4	5590	3966	278.9	7350	3966	276.8	9880
3967	326.4	5590	3967	278.9	7340	3967	276.8	9890
3968	326.4	5500	3968	278.9	7360	3968	276.8	9890
3969	326.5	5600	3969	278.9	7340	3969	276.8	9890
3970	326.4	5500	3970	278.9	7340	3970	276.8	9880
3971	326.4	5600	3971	278.9	7350	3971	276.8	9900
3972	326.5	5500	3972	278.9	7350	3972	276.8	9890
3973	326.5	5600	3973	278.9	7350	3973	276.8	9890
3974	326.4	5600	3974	278.9	7340	3974	276.7	9890
3975	326.4	5600	3975	278.9	7340	3975	276.8	9880
3976	326.4	5500	3976	278.9	7330	3976	276.8	9880
3977	326.4	5500	3977	278.9	7350	3977	276.8	9890
3978	326.4	5590	3978	278.9	7350	3978	276.7	9880
3979	326.5	5500	3979	278.9	7350	3979	276.8	9880
3980	326.5	5500	3980	278.9	7350	3980	276.7	9880
3981	326.5	5610	3981	278.9	7340	3981	276.7	9880
3982	326.5	5610	3982	278.9	7340	3982	276.7	9880
3983	326.5	5500	3983	279	7350	3983	276.7	9880
3984	326.5	5600	3984	279	7330	3984	276.7	9870
3985	326.6	5500	3985	279	7340	3985	276.7	9880
3986	326.6	5500	3986	279	7330	3986	276.7	9880
3987	326.6	5500	3987	279	7340	3987	276.7	9890
3988	326.6	5610	3988	279	7340	3988	276.6	9880
3989	326.6	5490	3989	279	7340	3989	276.7	9880
3990	326.6	5490	3990	279	7340	3990	276.6	9870
3991	326.6	5610	3991	279	7350	3991	276.7	9870
3992	326.6	5510	3992	279.1	7340	3992	276.7	9880
3993	326.7	5500	3993	279	7340	3993	276.7	9880
3994	326.6	5510	3994	279	7350	3994	276.7	9890
3995	326.6	5500	3995	279	7360	3995	276.7	9880
3996	326.6	5490	3996	279	7340	3996	276.6	9890

3997	326.6	5500	3997	278.9	7350	3997	276.6	9880
3998	326.6	5500	3998	278.9	7350	3998	276.6	9880
3999	326.6	5600	3999	278.9	7350	3999	276.6	9890
4000	326.6	5610	4000	279	7350	4000	276.6	9890
4001	326.6	5610	4001	279	7340	4001	276.6	9880
4002	326.6	5500	4002	278.9	7350	4002	276.6	9880
4003	326.6	5590	4003	279	7360	4003	276.6	9880
4004	326.6	5600	4004	279	7340	4004	276.6	9890
4005	326.6	5600	4005	279	7340	4005	276.6	9880
4006	326.6	5600	4006	279	7350	4006	276.7	9880
4007	326.7	5500	4007	279	7360	4007	276.6	9870
4008	326.7	5590	4008	279.1	7360	4008	276.6	9880
4009	326.8	5490	4009	279.1	7360	4009	276.7	9890
4010	326.8	5600	4010	279.1	7350	4010	276.7	9870
4011	326.7	5490	4011	279.1	7350	4011	276.7	9880
4012	326.7	5600	4012	279	7360	4012	276.7	9880
4013	326.7	5490	4013	279	7360	4013	276.7	9880
4014	326.8	5600	4014	279	7360	4014	276.7	9880
4015	326.8	5500	4015	279.1	7350	4015	276.7	9880
4016	326.8	5500	4016	279.1	7340	4016	276.7	9880
4017	326.8	5610	4017	279.1	7350	4017	276.7	9880
4018	326.8	5510	4018	279.1	7340	4018	276.7	9880
4019	326.8	5600	4019	279.1	7360	4019	276.6	9870
4020	326.8	5600	4020	279.1	7350	4020	276.6	9870
4021	326.8	5590	4021	279.1	7360	4021	276.6	9880
4022	326.8	5500	4022	279.2	7350	4022	276.6	9880
4023	326.8	5590	4023	279.2	7350	4023	276.6	9880
4024	326.8	5490	4024	279.2	7350	4024	276.6	9880
4025	326.8	5500	4025	279.2	7340	4025	276.6	9880
4026	326.8	5600	4026	279.2	7360	4026	276.7	9880
4027	326.8	5500	4027	279.2	7340	4027	276.7	9890
4028	326.8	5520	4028	279.2	7340	4028	276.7	9880
4029	326.9	5500	4029	279.2	7350	4029	276.7	9880
4030	326.9	5510	4030	279.1	7350	4030	276.7	9890
4031	326.9	5510	4031	279.1	7350	4031	276.7	9890
4032	326.9	5490	4032	279.1	7340	4032	276.7	9880
4033	326.9	5500	4033	279.1	7340	4033	276.7	9890
4034	326.9	5500	4034	279	7330	4034	276.7	9870
4035	326.9	5500	4035	279	7340	4035	276.7	9880
4036	326.9	5500	4036	279.1	7350	4036	276.7	9880
4037	326.9	5490	4037	279.1	7350	4037	276.7	9880
4038	326.9	5500	4038	279.1	7350	4038	276.7	9870
4039	326.9	5500	4039	279.1	7360	4039	276.7	9890
4040	326.9	5600	4040	279.1	7340	4040	276.7	9890
4041	326.9	5500	4041	279.1	7330	4041	276.7	9880
4042	326.9	5590	4042	279.1	7340	4042	276.7	9880
4043	326.9	5500	4043	279.1	7360	4043	276.7	9880
4044	326.9	5500	4044	279.1	7350	4044	276.7	9880
4045	326.9	5500	4045	279.1	7340	4045	276.7	9880
4046	326.9	5500	4046	279.1	7350	4046	276.7	9880

4047	326.9	5510	4047	279.1	7350	4047	276.7	9880
4048	326.9	5500	4048	279.1	7360	4048	276.7	9890
4049	327	5490	4049	279.1	7340	4049	276.7	9870
4050	326.9	5490	4050	279.1	7350	4050	276.7	9890
4051	326.9	5500	4051	279.1	7360	4051	276.7	9890
4052	326.9	5500	4052	279.1	7360	4052	276.7	9890
4053	326.9	5500	4053	279	7360	4053	276.7	9880
4054	326.9	5510	4054	279	7370	4054	276.6	9880
4055	326.9	5490	4055	279	7340	4055	276.6	9890
4056	326.9	5490	4056	279	7340	4056	276.6	9880
4057	326.9	5490	4057	279	7360	4057	276.7	9880
4058	326.9	5490	4058	279	7360	4058	276.6	9870
4059	326.9	5480	4059	279	7350	4059	276.7	9870
4060	326.9	5490	4060	279	7360	4060	276.7	9870
4061	326.9	5490	4061	279	7350	4061	276.7	9880
4062	327	5500	4062	279	7350	4062	276.7	9890
4063	326.9	5490	4063	279.1	7350	4063	276.6	9880
4064	326.9	5500	4064	279.1	7360	4064	276.6	9870
4065	327	5500	4065	279.1	7360	4065	276.6	9880
4066	327	5500	4066	279.1	7350	4066	276.6	9890
4067	327	5500	4067	279.1	7350	4067	276.6	9870
4068	327	5500	4068	279.1	7350	4068	276.6	9880
4069	327	5500	4069	279.1	7350	4069	276.6	9870
4070	327	5500	4070	279.1	7360	4070	276.6	9880
4071	326.9	5500	4071	279.1	7350	4071	276.6	9880
4072	326.9	5500	4072	279.1	7350	4072	276.7	9880
4073	327	5500	4073	279.1	7360	4073	276.7	9880
4074	327	5500	4074	279.1	7360	4074	276.7	9880
4075	327	5480	4075	279.1	7350	4075	276.7	9870
4076	327	5500	4076	279.2	7360	4076	276.7	9890
4077	327.1	5490	4077	279.1	7360	4077	276.7	9880
4078	327.1	5490	4078	279.1	7350	4078	276.7	9870
4079	327.1	5480	4079	279.1	7340	4079	276.7	9870
4080	327.1	5500	4080	279.1	7350	4080	276.7	9870
4081	327.1	5500	4081	279.1	7350	4081	276.7	9870
4082	327.1	5500	4082	279.1	7350	4082	276.7	9880
4083	327.1	5500	4083	279.1	7360	4083	276.7	9880
4084	327.1	5500	4084	279.1	7350	4084	276.7	9870
4085	327.1	5500	4085	279.1	7360	4085	276.7	9870
4086	327.1	5510	4086	279.1	7360	4086	276.7	9870
4087	327.1	5500	4087	279	7350	4087	276.6	9880
4088	327	5500	4088	279.1	7360	4088	276.6	9880
4089	327	5490	4089	279	7350	4089	276.7	9870
4090	327	5500	4090	279.1	7350	4090	276.7	9880
4091	327	5510	4091	279	7350	4091	276.7	9880
4092	327.1	5510	4092	279	7360	4092	276.7	9880
4093	327.1	5520	4093	279	7360	4093	276.7	9870
4094	327.1	5500	4094	279	7340	4094	276.7	9880
4095	327.1	5500	4095	279	7350	4095	276.7	9870
4096	327.1	5510	4096	279	7350	4096	276.7	9880

4097	327.1	5500	4097	279	7340	4097	276.7	9890
4098	327.1	5490	4098	279	7350	4098	276.7	9890
4099	327.1	5500	4099	279	7350	4099	276.7	9880
4100	327.1	5500	4100	279.1	7350	4100	276.8	9880
4101	327	5490	4101	279.1	7350	4101	276.8	9890
4102	327.1	5490	4102	279.1	7350	4102	276.7	9890
4103	327.1	5490	4103	279.1	7360	4103	276.7	9880
4104	327.1	5490	4104	279.1	7360	4104	276.7	9870
4105	327.1	5500	4105	279.1	7360	4105	276.7	9870
4106	327.1	5500	4106	279.1	7360	4106	276.7	9880
4107	327.1	5500	4107	279	7370	4107	276.6	9880
4108	327.1	5500	4108	279	7350	4108	276.6	9880
4109	327.1	5510	4109	279.1	7360	4109	276.7	9890
4110	327.1	5510	4110	279.1	7370	4110	276.6	9890
4111	327.1	5510	4111	279.1	7350	4111	276.6	9890
4112	327.1	5510	4112	279.1	7360	4112	276.7	9880
4113	327.1	5500	4113	279.1	7360	4113	276.7	9880
4114	327.1	5500	4114	279.1	7350	4114	276.7	9870
4115	327.1	5510	4115	279.1	7360	4115	276.7	9870
4116	327.1	5500	4116	279.1	7360	4116	276.7	9880
4117	327.1	5510	4117	279.1	7360	4117	276.7	9880
4118	327.1	5500	4118	279.1	7360	4118	276.7	9900
4119	327.1	5500	4119	279.1	7370	4119	276.7	9880
4120	327.1	5510	4120	279.1	7350	4120	276.7	9870
4121	327.1	5510	4121	279.1	7350	4121	276.7	9880
4122	327.1	5510	4122	279.1	7340	4122	276.7	9880
4123	327.2	5510	4123	279.1	7340	4123	276.7	9880
4124	327.2	5500	4124	279	7360	4124	276.7	9890
4125	327.2	5500	4125	279.1	7360	4125	276.7	9880
4126	327.2	5490	4126	279.1	7350	4126	276.7	9880
4127	327.2	5500	4127	279.1	7360	4127	276.7	9890
4128	327.2	5500	4128	279.2	7360	4128	276.6	9890
4129	327.2	5500	4129	279.2	7360	4129	276.6	9890
4130	327.1	5510	4130	279.1	7350	4130	276.6	9880
4131	327.2	5500	4131	279.1	7350	4131	276.6	9890
4132	327.2	5480	4132	279.1	7350	4132	276.6	9880
4133	327.2	5490	4133	279.1	7350	4133	276.6	9880
4134	327.2	5500	4134	279.1	7340	4134	276.6	9880
4135	327.2	5500	4135	279.1	7340	4135	276.6	9880
4136	327.2	5500	4136	279.1	7350	4136	276.6	9890
4137	327.2	5490	4137	279.1	7350	4137	276.6	9890
4138	327.2	5510	4138	279	7350	4138	276.6	9880
4139	327.1	5500	4139	279	7360	4139	276.6	9870
4140	327.1	5500	4140	279.1	7350	4140	276.6	9890
4141	327.2	5500	4141	279.1	7360	4141	276.6	9890
4142	327.2	5500	4142	279.1	7360	4142	276.6	9880
4143	327.2	5500	4143	279.1	7350	4143	276.6	9870
4144	327.2	5510	4144	279.1	7350	4144	276.6	9880
4145	327.2	5510	4145	279.1	7350	4145	276.6	9880
4146	327.1	5510	4146	279.1	7350	4146	276.6	9880

4147	327.1	5510	4147	279.1	7360	4147	276.6	9880
4148	327.2	5510	4148	279	7350	4148	276.6	9880
4149	327.1	5500	4149	279.1	7370	4149	276.6	9870
4150	327.1	5500	4150	279.1	7370	4150	276.6	9870
4151	327.1	5510	4151	279.1	7350	4151	276.6	9880
4152	327.1	5500	4152	279	7350	4152	276.6	9880
4153	327.1	5510	4153	279.1	7360	4153	276.7	9880
4154	327.1	5500	4154	279.1	7360	4154	276.7	9880
4155	327.1	5500	4155	279.1	7350	4155	276.7	9890
4156	327.1	5510	4156	279.1	7360	4156	276.7	9890
4157	327.2	5510	4157	279.1	7360	4157	276.7	9880
4158	327.1	5510	4158	279.1	7350	4158	276.7	9880
4159	327.2	5510	4159	279.1	7340	4159	276.7	9880
4160	327.2	5500	4160	279.1	7350	4160	276.7	9880
4161	327.2	5490	4161	279.1	7340	4161	276.7	9870
4162	327.2	5500	4162	279.1	7350	4162	276.8	9880
4163	327.2	5490	4163	279.1	7350	4163	276.8	9890
4164	327.2	5510	4164	279.1	7350	4164	276.8	9870
4165	327.3	5520	4165	279.1	7350	4165	276.8	9870
4166	327.3	5510	4166	279.1	7350	4166	276.8	9880
4167	327.3	5520	4167	279.1	7350	4167	276.8	9880
4168	327.4	5510	4168	279.1	7350	4168	276.8	9880
4169	327.4	5510	4169	279.1	7370	4169	276.8	9870
4170	327.3	5500	4170	279.1	7350	4170	276.8	9870
4171	327.3	5510	4171	279.1	7350	4171	276.8	9870
4172	327.3	5510	4172	279.1	7350	4172	276.8	9870
4173	327.2	5500	4173	279.1	7350	4173	276.8	9870
4174	327.3	5510	4174	279.1	7350	4174	276.8	9890
4175	327.3	5500	4175	279.1	7360	4175	276.9	9870
4176	327.2	5500	4176	279.1	7350	4176	276.8	9870
4177	327.2	5510	4177	279	7350	4177	276.8	9880
4178	327.2	5500	4178	279	7360	4178	276.8	9870
4179	327.2	5510	4179	279	7360	4179	276.8	9870
4180	327.1	5510	4180	279	7350	4180	276.8	9880
4181	327.2	5500	4181	279	7350	4181	276.8	9870
4182	327.2	5500	4182	279	7360	4182	276.8	9870
4183	327.2	5500	4183	279.1	7360	4183	276.8	9860
4184	327.2	5500	4184	279.1	7360	4184	276.8	9870
4185	327.2	5500	4185	279	7360	4185	276.8	9870
4186	327.1	5500	4186	279	7360	4186	276.8	9880
4187	327.2	5500	4187	279.1	7350	4187	276.8	9880
4188	327.1	5500	4188	279.1	7360	4188	276.8	9880
4189	327.2	5490	4189	279.1	7350	4189	276.8	9870
4190	327.2	5500	4190	279.1	7350	4190	276.8	9880
4191	327.1	5490	4191	279.1	7360	4191	276.8	9870
4192	327.1	5490	4192	279.1	7360	4192	276.8	9870
4193	327.2	5500	4193	279.1	7350	4193	276.8	9870
4194	327.2	5500	4194	279.1	7360	4194	276.8	9880
4195	327.2	5510	4195	279.1	7360	4195	276.8	9890
4196	327.2	5510	4196	279.1	7350	4196	276.9	9890

4197	327.2	5500	4197	279.1	7340	4197	276.8	9870
4198	327.2	5490	4198	279.1	7360	4198	276.8	9880
4199	327.2	5500	4199	279.1	7350	4199	276.8	9890
4200	327.2	5500	4200	279.1	7340	4200	276.8	9890
4201	327.2	5500	4201	279.1	7350	4201	276.8	9890
4202	327.2	5510	4202	279.1	7350	4202	276.8	9880
4203	327.2	5500	4203	279.1	7360	4203	276.8	9880
4204	327.3	5510	4204	279.1	7360	4204	276.8	9890
4205	327.3	5520	4205	279.1	7350	4205	276.8	9890
4206	327.3	5500	4206	279.1	7350	4206	276.8	9890
4207	327.4	5510	4207	279.1	7350	4207	276.8	9880
4208	327.4	5510	4208	279.1	7350	4208	276.8	9870
4209	327.4	5510	4209	279.1	7350	4209	276.8	9860
4210	327.4	5510	4210	279.1	7350	4210	276.8	9870
4211	327.4	5500	4211	279	7360	4211	276.8	9880
4212	327.5	5510	4212	279	7360	4212	276.8	9870
4213	327.4	5510	4213	279	7360	4213	276.8	9870
4214	327.5	5500	4214	279	7360	4214	276.8	9870
4215	327.4	5510	4215	279	7340	4215	276.8	9870
4216	327.4	5510	4216	279	7360	4216	276.8	9880
4217	327.5	5500	4217	279	7350	4217	276.9	9880
4218	327.4	5490	4218	279.1	7350	4218	276.9	9880
4219	327.4	5500	4219	279.1	7350	4219	276.9	9880
4220	327.4	5490	4220	279.1	7350	4220	276.8	9880
4221	327.4	5500	4221	279.1	7360	4221	276.9	9880
4222	327.3	5490	4222	279	7350	4222	276.8	9880
4223	327.3	5500	4223	279	7360	4223	276.8	9890
4224	327.4	5510	4224	279	7350	4224	276.8	9900
4225	327.3	5500	4225	279	7340	4225	276.8	9880
4226	327.3	5510	4226	279	7340	4226	276.8	9890
4227	327.3	5510	4227	279	7350	4227	276.8	9890
4228	327.3	5510	4228	279	7340	4228	276.8	9890
4229	327.3	5510	4229	279	7350	4229	276.8	9880
4230	327.3	5500	4230	279.1	7350	4230	276.8	9880
4231	327.3	5490	4231	279.1	7350	4231	276.8	9880
4232	327.3	5490	4232	279.1	7360	4232	276.8	9890
4233	327.3	5490	4233	279.1	7360	4233	276.8	9880
4234	327.2	5490	4234	279.1	7350	4234	276.8	9880
4235	327.2	5500	4235	279.1	7360	4235	276.8	9890
4236	327.1	5500	4236	279.1	7350	4236	276.8	9890
4237	327.2	5500	4237	279.1	7350	4237	276.8	9890
4238	327.1	5500	4238	279.1	7350	4238	276.7	9880
4239	327.2	5500	4239	279.1	7350	4239	276.8	9870
4240	327.2	5510	4240	279.1	7340	4240	276.8	9880
4241	327.2	5500	4241	279.2	7350	4241	276.8	9880
4242	327.2	5510	4242	279.2	7350	4242	276.8	9880
4243	327.2	5500	4243	279.2	7360	4243	276.8	9880
4244	327.2	5500	4244	279.2	7350	4244	276.8	9880
4245	327.3	5510	4245	279.1	7350	4245	276.9	9890
4246	327.3	5500	4246	279.2	7350	4246	276.9	9880

4247	327.3	5500	4247	279.1	7350	4247	276.9	9900
4248	327.3	5490	4248	279.1	7350	4248	276.9	9890
4249	327.3	5490	4249	279.1	7360	4249	276.9	9890
4250	327.2	5480	4250	279.1	7360	4250	276.9	9890
4251	327.2	5490	4251	279.1	7350	4251	276.9	9880
4252	327.3	5500	4252	279.1	7340	4252	276.9	9880
4253	327.3	5500	4253	279.1	7360	4253	276.9	9880
4254	327.2	5490	4254	279.1	7350	4254	276.9	9880
4255	327.2	5490	4255	279	7350	4255	276.9	9880
4256	327.2	5490	4256	279	7350	4256	276.9	9880
4257	327.1	5500	4257	279	7350	4257	276.9	9890
4258	327.1	5490	4258	279	7350	4258	276.9	9900
4259	327.2	5500	4259	278.9	7360	4259	276.9	9890
4260	327.2	5490	4260	279	7340	4260	276.9	9890
4261	327.3	5490	4261	279	7350	4261	276.9	9890
4262	327.2	5500	4262	278.9	7350	4262	276.9	9890
4263	327.2	5510	4263	278.9	7350	4263	276.9	9890
4264	327.3	5480	4264	278.9	7350	4264	277	9860
4265	327.3	5490	4265	278.9	7350	4265	276.9	9890
4266	327.3	5510	4266	279	7370	4266	277	9880
4267	327.3	5500	4267	279	7360	4267	277	9880
4268	327.4	5490	4268	279	7350	4268	277	9880
4269	327.4	5490	4269	279	7340	4269	277	9880
4270	327.4	5500	4270	279	7360	4270	277	9890
4271	327.4	5500	4271	279	7360	4271	276.9	9880
4272	327.5	5500	4272	279	7360	4272	276.9	9880
4273	327.4	5500	4273	279	7350	4273	276.9	9890
4274	327.5	5490	4274	278.9	7350	4274	276.9	9880
4275	327.5	5500	4275	279	7340	4275	276.9	9880
4276	327.5	5500	4276	279	7350	4276	276.8	9880
4277	327.5	5490	4277	278.9	7350	4277	276.8	9880
4278	327.5	5500	4278	279	7350	4278	276.8	9880
4279	327.5	5500	4279	279	7340	4279	276.8	9890
4280	327.5	5490	4280	279	7350	4280	276.8	9880
4281	327.5	5490	4281	279.2	7350	4281	276.8	9890
4282	327.5	5500	4282	279.5	7450	4282	276.8	9890
4283	327.5	5490	4283	280.1	7450	4283	276.8	9880
4284	327.6	5490	4284	280.6	7460	4284	276.8	9890
4285	327.5	5490	4285	281.2	7450	4285	276.9	9880
4286	327.5	5490	4286	281.8	7440	4286	276.9	9890
4287	327.4	5490	4287	282.4	7440	4287	276.9	9880
4288	327.4	5500	4288	282.8	7440	4288	276.9	9880
4289	327.4	5500	4289	283.3	7440	4289	276.9	9880
4290	327.4	5500	4290	283.7	7440	4290	276.9	9890
4291	327.4	5500	4291	284.1	7450	4291	276.9	9880
4292	327.5	5490	4292	284.5	7440	4292	276.9	9890
4293	327.5	5500	4293	285	7450	4293	276.9	9890
4294	327.5	5510	4294	285.4	7450	4294	276.9	9870
4295	327.5	5500	4295	285.9	7440	4295	276.9	9880
4296	327.5	5500	4296	286.3	7440	4296	276.9	9870

4297	327.5	5500	4297	286.8	7440	4297	276.9	9880
4298	327.5	5490	4298	287.2	7520	4298	276.9	9880
4299	327.5	5510	4299	287.8	7540	4299	276.8	9870
4300	327.6	5500	4300	288.3	7430	4300	276.9	9890
4301	327.5	5480	4301	288.8	7530	4301	276.8	9900
4302	327.6	5490	4302	289.3	7520	4302	276.8	9890
4303	327.6	5490	4303	289.7	7530	4303	276.8	9890
4304	327.5	5500	4304	290.1	7530	4304	276.8	9890
4305	327.5	5500	4305	290.5	7530	4305	276.8	9890
4306	327.5	5500	4306	290.8	7540	4306	276.8	9880
4307	327.5	5490	4307	291.2	7550	4307	276.8	9880
4308	327.5	5500	4308	291.5	7540	4308	276.8	9880
4309	327.5	5490	4309	291.7	7530	4309	276.8	9880
4310	327.4	5500	4310	292	7530	4310	276.8	9880
4311	327.5	5480	4311	292.3	7540	4311	276.8	9900
4312	327.5	5490	4312	292.6	7520	4312	276.8	9890
4313	327.5	5510	4313	292.9	7530	4313	276.8	9890
4314	327.5	5500	4314	293.2	7540	4314	276.8	9890
4315	327.6	5500	4315	293.4	7540	4315	276.8	9890
4316	327.6	5500	4316	293.5	7530	4316	276.8	9880
4317	327.6	5500	4317	293.7	7530	4317	276.8	9880
4318	327.6	5500	4318	293.9	7540	4318	276.8	9870
4319	327.6	5500	4319	294.1	7540	4319	276.8	9880
4320	327.5	5500	4320	294.2	7550	4320	276.8	9870
4321	327.5	5500	4321	294.3	7550	4321	276.8	9870
4322	327.6	5490	4322	294.5	7530	4322	276.8	9880
4323	327.6	5510	4323	294.7	7530	4323	276.8	9880
4324	327.6	5490	4324	294.9	7550	4324	276.8	9870
4325	327.5	5490	4325	295	7540	4325	276.8	9870
4326	327.5	5490	4326	295.1	7550	4326	276.8	9880
4327	327.5	5490	4327	295.2	7650	4327	276.8	9890
4328	327.4	5500	4328	295.3	7640	4328	276.9	9870
4329	327.5	5490	4329	295.4	7550	4329	276.9	9880
4330	327.5	5500	4330	295.6	7630	4330	276.9	9880
4331	327.5	5490	4331	295.7	7640	4331	276.9	9880
4332	327.5	5490	4332	295.8	7620	4332	276.8	9870
4333	327.5	5500	4333	295.9	7630	4333	276.8	9890
4334	327.4	5490	4334	296	7630	4334	276.8	9880
4335	327.4	5490	4335	296.2	7630	4335	276.8	9890
4336	327.5	5490	4336	296.3	7640	4336	276.9	9880
4337	327.5	5490	4337	296.5	7630	4337	276.8	9880
4338	327.4	5490	4338	296.6	7530	4338	276.8	9890
4339	327.4	5510	4339	296.7	7640	4339	276.8	9880
4340	327.5	5500	4340	296.8	7640	4340	276.8	9880
4341	327.4	5500	4341	296.9	7650	4341	276.8	9870
4342	327.4	5500	4342	297.1	7640	4342	276.8	9880
4343	327.5	5490	4343	297.1	7630	4343	276.8	9890
4344	327.5	5500	4344	297.2	7640	4344	276.8	9880
4345	327.5	5490	4345	297.3	7640	4345	276.8	9880
4346	327.5	5500	4346	297.4	7640	4346	276.8	9890

4347	327.5	5490	4347	297.4	7640	4347	276.8	9880
4348	327.5	5500	4348	297.5	7640	4348	276.8	9870
4349	327.6	5490	4349	297.6	7640	4349	276.8	9880
4350	327.6	5500	4350	297.7	7640	4350	276.8	9880
4351	327.6	5490	4351	297.8	7650	4351	276.9	9870
4352	327.5	5500	4352	297.9	7650	4352	276.9	9870
4353	327.6	5490	4353	298	7640	4353	276.9	9870
4354	327.5	5500	4354	298	7650	4354	276.9	9880
4355	327.6	5510	4355	298	7660	4355	276.9	9880
4356	327.6	5510	4356	298.1	7650	4356	276.9	9870
4357	327.6	5500	4357	298.3	7640	4357	276.9	9870
4358	327.6	5500	4358	298.4	7640	4358	276.9	9880
4359	327.6	5500	4359	298.4	7640	4359	276.8	9880
4360	327.7	5480	4360	298.5	7650	4360	276.8	9870
4361	327.6	5490	4361	298.6	7650	4361	276.8	9880
4362	327.6	5490	4362	298.7	7640	4362	276.8	9880
4363	327.6	5490	4363	298.7	7640	4363	276.8	9890
4364	327.5	5490	4364	298.8	7640	4364	276.8	9890
4365	327.6	5500	4365	298.8	7630	4365	276.9	9870
4366	327.5	5490	4366	299	7640	4366	276.8	9870
4367	327.5	5490	4367	299	7640	4367	276.8	9880
4368	327.6	5490	4368	299.1	7630	4368	276.8	9880
4369	327.6	5490	4369	299.1	7640	4369	276.9	9970
4370	327.6	5500	4370	299.2	7640	4370	277.2	9960
4371	327.6	5490	4371	299.3	7640	4371	277.8	9970
4372	327.6	5500	4372	299.4	7630	4372	278.6	9970
4373	327.6	5490	4373	299.5	7630	4373	279.2	9970
4374	327.6	5500	4374	299.6	7630	4374	279.9	9970
4375	327.6	5490	4375	299.6	7650	4375	280.5	9980
4376	327.6	5490	4376	299.6	7640	4376	281	9970
4377	327.5	5500	4377	299.6	7660	4377	281.6	10070
4378	327.5	5500	4378	299.6	7640	4378	282.2	10080
4379	327.6	5500	4379	299.6	7640	4379	282.9	10070
4380	327.6	5500	4380	299.7	7660	4380	283.7	10070
4381	327.6	5510	4381	299.7	7640	4381	284.4	10060
4382	327.6	5490	4382	299.7	7650	4382	285	10070
4383	327.6	5500	4383	299.7	7640	4383	285.6	10070
4384	327.6	5490	4384	299.7	7640	4384	286.3	10070
4385	327.6	5490	4385	299.7	7650	4385	286.9	10060
4386	327.6	5490	4386	299.7	7650	4386	287.4	10050
4387	327.6	5490	4387	299.8	7640	4387	287.9	10070
4388	327.5	5500	4388	299.8	7640	4388	288.5	10160
4389	327.6	5510	4389	299.8	7640	4389	289.1	10150
4390	327.5	5510	4390	299.8	7640	4390	289.6	10150
4391	327.5	5510	4391	299.8	7640	4391	290.1	10170
4392	327.6	5500	4392	299.8	7640	4392	290.6	10150
4393	327.6	5500	4393	299.8	7650	4393	291	10160
4394	327.6	5500	4394	299.9	7640	4394	291.4	10160
4395	327.6	5500	4395	299.9	7650	4395	291.8	10170
4396	327.7	5500	4396	300	7650	4396	292.1	10170

4397	327.7	5500	4397	300	7640	4397	292.4	10150
4398	327.7	5510	4398	300.1	7630	4398	292.7	10160
4399	327.7	5510	4399	300.1	7620	4399	293	10160
4400	327.7	5490	4400	300.1	7640	4400	293.3	10150
4401	327.8	5490	4401	300.2	7640	4401	293.5	10160
4402	327.7	5490	4402	300.2	7630	4402	293.7	10170
4403	327.8	5500	4403	300.2	7640	4403	294	10180
4404	327.8	5490	4404	300.3	7630	4404	294.2	10160
4405	327.7	5490	4405	300.4	7640	4405	294.4	10170
4406	327.7	5500	4406	300.5	7650	4406	294.6	10250
4407	327.7	5500	4407	300.5	7630	4407	294.8	10170
4408	327.8	5500	4408	300.5	7640	4408	295	10270
4409	327.8	5500	4409	300.5	7650	4409	295.3	10260
4410	327.8	5500	4410	300.6	7640	4410	295.4	10260
4411	327.8	5510	4411	300.6	7640	4411	295.6	10270
4412	327.9	5500	4412	300.6	7740	4412	295.8	10270
4413	327.8	5510	4413	300.7	7640	4413	296	10260
4414	327.9	5510	4414	300.8	7650	4414	296.1	10260
4415	327.8	5500	4415	300.8	7630	4415	296.3	10270
4416	327.7	5500	4416	300.8	7650	4416	296.4	10260
4417	327.7	5500	4417	300.8	7630	4417	296.6	10260
4418	327.7	5500	4418	300.8	7630	4418	296.8	10270
4419	327.7	5500	4419	300.9	7630	4419	296.9	10250
4420	327.7	5510	4420	300.8	7630	4420	297.1	10260
4421	327.6	5510	4421	300.8	7640	4421	297.2	10270
4422	327.7	5510	4422	300.9	7640	4422	297.4	10270
4423	327.7	5510	4423	300.9	7740	4423	297.5	10250
4424	327.7	5480	4424	301	7630	4424	297.6	10260
4425	327.7	5500	4425	301	7640	4425	297.8	10250
4426	327.7	5490	4426	301.1	7630	4426	298	10260
4427	327.7	5500	4427	301.1	7650	4427	298.1	10260
4428	327.7	5490	4428	301.1	7640	4428	298.2	10260
4429	327.7	5480	4429	301.1	7650	4429	298.3	10260
4430	327.7	5490	4430	301.1	7640	4430	298.3	10260
4431	327.7	5490	4431	301.1	7640	4431	298.3	10260
4432	327.7	5500	4432	301	7650	4432	298.4	10280
4433	327.7	5500	4433	301	7650	4433	298.4	10260
4434	327.6	5500	4434	300.9	7650	4434	298.5	10270
4435	327.6	5500	4435	300.9	7650	4435	298.6	10260
4436	327.9	5490	4436	300.9	7640	4436	298.7	10250
4437	328.5	5480	4437	300.9	7640	4437	298.7	10260
4438	329.2	5570	4438	300.8	7630	4438	298.7	10260
4439	329.9	5570	4439	300.8	7650	4439	298.7	10350
4440	330.5	5570	4440	300.9	7630	4440	298.8	10260
4441	331.1	5560	4441	300.9	7640	4441	298.9	10260
4442	331.6	5580	4442	300.9	7630	4442	298.9	10260
4443	332.1	5580	4443	300.8	7630	4443	299	10270
4444	332.7	5600	4444	300.9	7640	4444	299.1	10370
4445	333.3	5580	4445	300.9	7640	4445	299.1	10370
4446	333.9	5580	4446	300.9	7650	4446	299.2	10370

4447	334.5	5580	4447	300.9	7640	4447	299.2	10370
4448	335.1	5580	4448	300.9	7640	4448	299.3	10340
4449	335.8	5670	4449	300.9	7630	4449	299.4	10360
4450	336.5	5670	4450	300.9	7630	4450	299.5	10370
4451	337.1	5660	4451	300.9	7630	4451	299.6	10370
4452	337.7	5670	4452	300.9	7620	4452	299.7	10260
4453	338.4	5680	4453	300.9	7630	4453	299.8	10360
4454	339.2	5670	4454	300.9	7650	4454	299.8	10260
4455	339.9	5680	4455	300.9	7640	4455	299.9	10360
4456	340.8	5690	4456	300.9	7650	4456	300	10360
4457	341.6	5670	4457	301	7640	4457	300.1	10350
4458	342.3	5760	4458	301	7640	4458	300.2	10360
4459	343	5780	4459	301.1	7650	4459	300.2	10360
4460	343.6	5670	4460	301.1	7640	4460	300.2	10360
4461	344.2	5760	4461	301.2	7630	4461	300.3	10360
4462	344.7	5770	4462	301.2	7640	4462	300.3	10360
4463	345.3	5770	4463	301.2	7640	4463	300.5	10360
4464	345.7	5770	4464	301.2	7650	4464	300.6	10360
4465	346.2	5770	4465	301.2	7640	4465	300.7	10360
4466	346.6	5770	4466	301.2	7640	4466	300.7	10350
4467	347	5770	4467	301.2	7630	4467	300.8	10360
4468	347.4	5770	4468	301.2	7630	4468	300.7	10360
4469	347.7	5760	4469	301.3	7630	4469	300.7	10360
4470	348.1	5760	4470	301.3	7620	4470	300.8	10360
4471	348.4	5760	4471	301.3	7640	4471	300.7	10370
4472	348.7	5760	4472	301.2	7630	4472	300.7	10360
4473	349	5760	4473	301.3	7630	4473	300.7	10370
4474	349.2	5770	4474	301.3	7640	4474	300.7	10380
4475	349.5	5770	4475	301.3	7640	4475	300.7	10370
4476	349.9	5760	4476	301.3	7650	4476	300.8	10350
4477	350.1	5760	4477	301.4	7640	4477	300.8	10360
4478	350.4	5760	4478	301.4	7650	4478	300.9	10360
4479	350.7	5760	4479	301.4	7640	4479	300.9	10340
4480	350.9	5770	4480	301.5	7650	4480	300.9	10360
4481	351.2	5770	4481	301.5	7640	4481	300.9	10360
4482	351.4	5760	4482	301.5	7640	4482	300.9	10360
4483	351.6	5760	4483	301.6	7640	4483	300.9	10360
4484	351.8	5770	4484	301.6	7640	4484	300.9	10350
4485	352	5760	4485	301.7	7640	4485	301	10350
4486	352.2	5760	4486	301.7	7640	4486	300.9	10350
4487	352.3	5760	4487	301.7	7640	4487	301	10340
4488	352.5	5760	4488	301.8	7630	4488	301	10350
4489	352.6	5770	4489	301.8	7640	4489	301.1	10350
4490	352.6	5770	4490	301.7	7640	4490	301.1	10350
4491	352.5	5770	4491	301.7	7640	4491	301.2	10350
4492	352.4	5770	4492	301.6	7630	4492	301.2	10340
4493	352.4	5780	4493	301.5	7640	4493	301.3	10350
4494	352.4	5780	4494	301.4	7650	4494	301.3	10350
4495	352.4	5770	4495	301.4	7640	4495	301.3	10340
4496	352.4	5760	4496	301.4	7640	4496	301.2	10360

4497	352.4	5770	4497	301.4	7630	4497	301.2	10360
4498	352.4	5770	4498	301.3	7630	4498	301.2	10360
4499	352.5	5770	4499	301.3	7640	4499	301.2	10360
4500	352.5	5780	4500	301.3	7640	4500	301.2	10370
4501	352.5	5770	4501	301.3	7630	4501	301.2	10360
4502	352.6	5770	4502	301.2	7630	4502	301.2	10370
4503	352.7	5770	4503	301.2	7630	4503	301.2	10350
4504	352.7	5770	4504	301.2	7640	4504	301.2	10360
4505	352.8	5770	4505	301.2	7640	4505	301.2	10360
4506	352.9	5770	4506	301.2	7650	4506	301.2	10360
4507	353	5770	4507	301.2	7640	4507	301.3	10360
4508	353	5770	4508	301.1	7630	4508	301.3	10370
4509	353.1	5770	4509	301.2	7640	4509	301.3	10370
4510	353.1	5770	4510	301.2	7630	4510	301.3	10360
4511	353	5760	4511	301.2	7630	4511	301.3	10360
4512	352.9	5760	4512	301.2	7630	4512	301.3	10360
4513	352.9	5770	4513	301.2	7640	4513	301.3	10360
4514	352.9	5770	4514	301.1	7640	4514	301.3	10350
4515	352.9	5770	4515	301.1	7630	4515	301.3	10370
4516	352.9	5770	4516	301.1	7630	4516	301.3	10360
4517	352.9	5770	4517	301.1	7640	4517	301.3	10360
4518	352.8	5770	4518	301	7640	4518	301.3	10350
4519	352.8	5760	4519	301.1	7640	4519	301.4	10350
4520	352.7	5760	4520	301.1	7630	4520	301.4	10360
4521	352.7	5770	4521	301.1	7630	4521	301.4	10380
4522	352.7	5760	4522	301.1	7630	4522	301.4	10350
4523	352.7	5770	4523	301.1	7640	4523	301.5	10350
4524	352.8	5760	4524	301.1	7630	4524	301.5	10360
4525	352.8	5660	4525	301.1	7620	4525	301.6	10360
4526	352.9	5770	4526	301.2	7630	4526	301.6	10350
4527	352.9	5760	4527	301.2	7640	4527	301.6	10350
4528	352.9	5760	4528	301.2	7640	4528	301.6	10360
4529	352.9	5750	4529	301.2	7630	4529	301.7	10370
4530	352.9	5760	4530	301.2	7630	4530	301.7	10360
4531	352.9	5770	4531	301.2	7620	4531	301.7	10360
4532	352.9	5760	4532	301.2	7620	4532	301.7	10370
4533	353	5770	4533	301.2	7630	4533	301.8	10360
4534	353	5760	4534	301.2	7630	4534	301.8	10370
4535	353	5770	4535	301.3	7620	4535	301.8	10360
4536	353.1	5760	4536	301.3	7620	4536	301.8	10370
4537	353.1	5770	4537	301.3	7630	4537	301.8	10370
4538	353.2	5760	4538	301.3	7630	4538	301.8	10360
4539	353.3	5760	4539	301.3	7630	4539	301.8	10360
4540	353.4	5760	4540	301.4	7620	4540	301.8	10360
4541	353.5	5770	4541	301.4	7620	4541	301.8	10370
4542	353.6	5770	4542	301.3	7630	4542	301.8	10370
4543	353.6	5770	4543	301.3	7640	4543	301.8	10370
4544	353.6	5780	4544	301.3	7630	4544	301.9	10360
4545	353.6	5770	4545	301.3	7620	4545	301.9	10370
4546	353.7	5770	4546	301.4	7640	4546	301.9	10360

4547	353.8	5770	4547	301.4	7640	4547	301.9	10370
4548	353.8	5760	4548	301.3	7640	4548	302	10370
4549	353.8	5760	4549	301.3	7640	4549	302	10360
4550	353.8	5770	4550	301.3	7640	4550	302	10350
4551	353.8	5760	4551	301.3	7640	4551	302.1	10370
4552	353.9	5770	4552	301.3	7640	4552	302.1	10360
4553	354	5770	4553	301.3	7630	4553	302.2	10370
4554	354	5770	4554	301.4	7630	4554	302.2	10370
4555	354	5770	4555	301.4	7640	4555	302.2	10350
4556	354.1	5770	4556	301.4	7630	4556	302.2	10370
4557	354.1	5770	4557	301.4	7640	4557	302.3	10370
4558	354.1	5780	4558	301.4	7630	4558	302.3	10370
4559	354.2	5780	4559	301.4	7630	4559	302.3	10370
4560	354.2	5770	4560	301.4	7630	4560	302.3	10370
4561	354.2	5770	4561	301.4	7630	4561	302.3	10360
4562	354.3	5770	4562	301.3	7640	4562	302.4	10370
4563	354.3	5760	4563	301.4	7640	4563	302.4	10370
4564	354.3	5760	4564	301.4	7650	4564	302.4	10370
4565	354.4	5770	4565	301.4	7630	4565	302.4	10360
4566	354.3	5670	4566	301.4	7640	4566	302.5	10370
4567	354.1	5660	4567	301.4	7630	4567	302.5	10370
4568	354.1	5670	4568	301.4	7650	4568	302.5	10380
4569	354	5670	4569	301.4	7630	4569	302.5	10360
4570	353.9	5770	4570	301.4	7630	4570	302.6	10360
4571	353.8	5760	4571	301.4	7630	4571	302.6	10360
4572	353.8	5670	4572	301.4	7630	4572	302.7	10360
4573	353.8	5650	4573	301.4	7630	4573	302.7	10370
4574	353.8	5660	4574	301.4	7630	4574	302.7	10370
4575	353.7	5660	4575	301.4	7640	4575	302.8	10350
4576	353.6	5770	4576	301.4	7640	4576	302.8	10360
4577	353.6	5680	4577	301.4	7640	4577	302.8	10360
4578	353.6	5680	4578	301.4	7620	4578	302.8	10360
4579	353.6	5670	4579	301.4	7640	4579	302.8	10370
4580	353.6	5660	4580	301.4	7640	4580	302.9	10380
4581	353.5	5670	4581	301.4	7630	4581	302.9	10380
4582	353.5	5660	4582	301.4	7640	4582	302.9	10360
4583	353.5	5680	4583	301.4	7640	4583	302.9	10360
4584	353.5	5670	4584	301.4	7640	4584	303	10360
4585	353.4	5680	4585	301.4	7640	4585	302.9	10360
4586	353.3	5670	4586	301.4	7650	4586	303	10360
4587	353.3	5670	4587	301.4	7630	4587	303	10360
4588	353.2	5670	4588	301.4	7650	4588	303	10360
4589	353.1	5660	4589	301.4	7620	4589	303.1	10370
4590	353.1	5660	4590	301.4	7620	4590	303	10370
4591	353	5670	4591	301.4	7640	4591	303.1	10370
4592	353	5680	4592	301.4	7630	4592	303.1	10360
4593	352.9	5660	4593	301.4	7640	4593	303.1	10360
4594	352.9	5660	4594	301.4	7640	4594	303.2	10370
4595	352.9	5660	4595	301.5	7640	4595	303.2	10370
4596	352.9	5660	4596	301.5	7640	4596	303.2	10370

4597	352.9	5670	4597	301.5	7640	4597	303.2	10370
4598	352.8	5660	4598	301.5	7640	4598	303.3	10350
4599	352.8	5650	4599	301.5	7640	4599	303.3	10360
4600	352.9	5670	4600	301.5	7630	4600	303.2	10360
4601	352.9	5670	4601	301.5	7640	4601	303.3	10370
4602	352.8	5680	4602	301.4	7630	4602	303.3	10380
4603	352.9	5670	4603	301.4	7630	4603	303.3	10360
4604	352.9	5660	4604	301.4	7640	4604	303.2	10370
4605	352.9	5670	4605	301.4	7630	4605	303.2	10380
4606	352.9	5660	4606	301.5	7630	4606	303.1	10350
4607	352.9	5660	4607	301.5	7630	4607	303.1	10350
4608	352.9	5660	4608	301.5	7620	4608	303.1	10360
4609	352.8	5670	4609	301.5	7640	4609	303.1	10360
4610	352.8	5650	4610	301.5	7630	4610	303	10370
4611	352.8	5670	4611	301.5	7650	4611	303	10360
4612	352.7	5650	4612	301.5	7640	4612	302.9	10370
4613	352.7	5660	4613	301.5	7640	4613	302.9	10360
4614	352.7	5650	4614	301.5	7640	4614	302.9	10360
4615	352.6	5650	4615	301.5	7640	4615	302.8	10380
4616	352.4	5650	4616	301.5	7640	4616	302.9	10370
4617	352.3	5660	4617	301.5	7640	4617	302.8	10360
4618	352.1	5670	4618	301.5	7640	4618	302.8	10370
4619	352	5660	4619	301.6	7630	4619	302.8	10360
4620	351.9	5660	4620	301.6	7630	4620	302.8	10360
4621	351.8	5650	4621	301.5	7630	4621	302.8	10370
4622	351.8	5670	4622	301.5	7630	4622	302.7	10360
4623	351.7	5670	4623	301.5	7640	4623	302.8	10370
4624	351.6	5650	4624	301.6	7630	4624	302.8	10360
4625	351.5	5660	4625	301.6	7620	4625	302.8	10360
4626	351.4	5660	4626	301.6	7620	4626	302.8	10370
4627	351.3	5670	4627	301.5	7630	4627	302.9	10360
4628	351.3	5660	4628	301.5	7630	4628	302.8	10370
4629	351.2	5670	4629	301.6	7630	4629	302.8	10360
4630	351.2	5670	4630	301.6	7630	4630	302.9	10370
4631	351.1	5670	4631	301.6	7640	4631	302.9	10350
4632	351.1	5660	4632	301.7	7630	4632	302.9	10350
4633	351	5660	4633	301.6	7630	4633	302.9	10360
4634	350.9	5660	4634	301.6	7620	4634	302.9	10360
4635	351	5660	4635	301.6	7630	4635	302.9	10360
4636	351	5660	4636	301.6	7630	4636	302.9	10360
4637	350.9	5650	4637	301.7	7640	4637	302.9	10370
4638	350.9	5660	4638	301.6	7640	4638	302.9	10360
4639	350.9	5670	4639	301.6	7630	4639	302.9	10370
4640	350.9	5660	4640	301.7	7630	4640	302.9	10370
4641	350.9	5670	4641	301.7	7630	4641	302.9	10350
4642	350.9	5660	4642	301.6	7640	4642	302.9	10380
4643	350.9	5660	4643	301.6	7640	4643	302.9	10360
4644	350.8	5660	4644	301.6	7640	4644	303	10360
4645	350.8	5660	4645	301.7	7650	4645	302.9	10370
4646	350.7	5650	4646	301.6	7640	4646	302.9	10360

4647	350.7	5660	4647	301.6	7640	4647	302.9	10360
4648	350.7	5660	4648	301.6	7630	4648	303	10360
4649	350.6	5670	4649	301.6	7640	4649	303	10360
4650	350.6	5660	4650	301.6	7630	4650	303	10350
4651	350.6	5670	4651	301.7	7630	4651	303	10360
4652	350.5	5670	4652	301.7	7630	4652	303	10360
4653	350.5	5680	4653	301.7	7630	4653	303	10360
4654	350.5	5670	4654	301.7	7630	4654	303	10350
4655	350.5	5680	4655	301.7	7630	4655	303	10350
4656	350.5	5670	4656	301.7	7620	4656	303	10360
4657	350.5	5680	4657	301.6	7630	4657	303	10360
4658	350.5	5680	4658	301.7	7630	4658	303	10350
4659	350.4	5670	4659	301.7	7640	4659	303	10350
4660	350.4	5660	4660	301.6	7640	4660	302.9	10360
4661	350.4	5670	4661	301.6	7630	4661	303	10350
4662	350.3	5660	4662	301.6	7620	4662	303	10360
4663	350.3	5670	4663	301.5	7640	4663	303	10370
4664	350.3	5660	4664	301.6	7640	4664	303	10360
4665	350.2	5680	4665	301.6	7630	4665	303	10350
4666	350.2	5680	4666	301.6	7630	4666	303	10350
4667	350.3	5660	4667	301.7	7640	4667	303	10370
4668	350.2	5670	4668	301.7	7630	4668	303.1	10370
4669	350.1	5670	4669	301.8	7620	4669	303.1	10360
4670	350.1	5670	4670	301.8	7620	4670	303.1	10360
4671	350.2	5670	4671	301.8	7630	4671	303.1	10360
4672	350.1	5670	4672	301.8	7630	4672	303.1	10370
4673	350.1	5680	4673	301.8	7630	4673	303.1	10360
4674	350.1	5660	4674	301.7	7630	4674	303.1	10350
4675	350.1	5670	4675	301.7	7620	4675	303.1	10370
4676	350.1	5670	4676	301.7	7630	4676	303.1	10360
4677	350.2	5650	4677	301.7	7630	4677	303.1	10360
4678	350.2	5660	4678	301.8	7640	4678	303.1	10340
4679	350.2	5660	4679	301.8	7630	4679	303.1	10350
4680	350.2	5670	4680	301.8	7630	4680	303.1	10350
4681	350.2	5670	4681	301.8	7620	4681	303.1	10360
4682	350.2	5680	4682	301.8	7610	4682	303.1	10350
4683	350.2	5670	4683	301.8	7640	4683	303.1	10360
4684	350.2	5660	4684	301.8	7650	4684	303.1	10350
4685	350.2	5670	4685	301.8	7630	4685	303	10350
4686	350.2	5670	4686	301.7	7640	4686	303.1	10350
4687	350.2	5670	4687	301.7	7640	4687	303.1	10350
4688	350.2	5670	4688	301.8	7640	4688	303	10350
4689	350.2	5660	4689	301.8	7640	4689	303	10350
4690	350.2	5660	4690	301.8	7630	4690	303.1	10350
4691	350.2	5660	4691	301.8	7640	4691	303	10360
4692	350.1	5570	4692	301.8	7630	4692	303	10350
4693	350.1	5550	4693	301.8	7630	4693	303.1	10350
4694	350.1	5660	4694	301.8	7630	4694	303.1	10360
4695	350.1	5670	4695	301.8	7630	4695	303.1	10350
4696	350.1	5570	4696	301.8	7630	4696	303.1	10360

4697	350	5560	4697	301.8	7620	4697	303.2	10360
4698	350	5560	4698	301.8	7640	4698	303.2	10350
4699	350	5560	4699	301.8	7640	4699	303.2	10360
4700	350	5650	4700	301.8	7630	4700	303.2	10350
4701	350	5660	4701	301.8	7630	4701	303.2	10360
4702	350	5560	4702	301.8	7630	4702	303.2	10270
4703	350	5570	4703	301.8	7640	4703	303.2	10360
4704	349.9	5670	4704	301.8	7640	4704	303.2	10370
4705	349.9	5550	4705	301.8	7640	4705	303.3	10350
4706	350	5560	4706	301.9	7640	4706	303.3	10360
4707	350	5560	4707	301.9	7630	4707	303.3	10360
4708	350	5650	4708	301.9	7630	4708	303.3	10360
4709	350	5660	4709	301.9	7630	4709	303.3	10360
4710	350.1	5670	4710	301.9	7630	4710	303.3	10270
4711	350.1	5560	4711	301.9	7630	4711	303.3	10350
4712	350.1	5560	4712	302	7630	4712	303.4	10350
4713	350	5570	4713	302	7620	4713	303.4	10260
4714	350.1	5670	4714	302	7640	4714	303.4	10350
4715	350.1	5670	4715	302	7630	4715	303.4	10360
4716	350.1	5580	4716	302.1	7630	4716	303.4	10360
4717	350.1	5580	4717	302	7640	4717	303.4	10360
4718	350.1	5560	4718	302.1	7630	4718	303.4	10260
4719	350.1	5570	4719	302.1	7640	4719	303.3	10370
4720	350.1	5570	4720	302.2	7640	4720	303.3	10360
4721	350.1	5580	4721	302.1	7640	4721	303.4	10370
4722	350.1	5570	4722	302.1	7630	4722	303.4	10350
4723	350.1	5580	4723	302.2	7640	4723	303.4	10370
4724	350.1	5580	4724	302.2	7640	4724	303.4	10370
4725	350	5580	4725	302.2	7640	4725	303.4	10370
4726	350	5560	4726	302.2	7640	4726	303.4	10370
4727	350	5580	4727	302.2	7630	4727	303.4	10370
4728	350	5570	4728	302.2	7640	4728	303.4	10360
4729	350	5660	4729	302.2	7640	4729	303.4	10270
4730	350.1	5570	4730	302.2	7640	4730	303.4	10360
4731	350.1	5580	4731	302.2	7640	4731	303.4	10370
4732	350	5570	4732	302.2	7640	4732	303.4	10260
4733	350.1	5570	4733	302.1	7640	4733	303.4	10350
4734	350.1	5570	4734	302.1	7630	4734	303.4	10360
4735	350.1	5560	4735	302	7630	4735	303.5	10360
4736	350	5570	4736	302	7630	4736	303.5	10260
4737	350	5580	4737	302.1	7630	4737	303.5	10360
4738	350	5570	4738	302.1	7620	4738	303.6	10260
4739	350	5570	4739	302.1	7640	4739	303.6	10260
4740	350	5570	4740	302.1	7630	4740	303.6	10360
4741	349.9	5570	4741	302.1	7640	4741	303.6	10370
4742	350	5560	4742	302.1	7640	4742	303.6	10360
4743	350	5570	4743	302.1	7630	4743	303.6	10260
4744	350	5560	4744	302.1	7630	4744	303.6	10380
4745	350	5570	4745	302.1	7640	4745	303.6	10260
4746	350	5570	4746	302.1	7640	4746	303.6	10360

4747	350.1	5570	4747	302.1	7630	4747	303.6	10350
4748	350.1	5560	4748	302.1	7630	4748	303.6	10350
4749	350	5560	4749	302.1	7630	4749	303.6	10360
4750	350	5570	4750	302.1	7630	4750	303.6	10360
4751	350	5560	4751	302.1	7620	4751	303.6	10360
4752	350	5570	4752	302.1	7630	4752	303.6	10260
4753	350	5570	4753	302.1	7630	4753	303.6	10270
4754	350	5570	4754	302.1	7640	4754	303.6	10260
4755	350	5580	4755	302.1	7630	4755	303.6	10260
4756	350.1	5570	4756	302.1	7640	4756	303.6	10360
4757	350.1	5570	4757	302.2	7640	4757	303.7	10260
4758	350.1	5570	4758	302.2	7650	4758	303.6	10360
4759	350.1	5580	4759	302.2	7650	4759	303.6	10370
4760	350.1	5570	4760	302.2	7650	4760	303.6	10360
4761	350.1	5570	4761	302.2	7640	4761	303.6	10360
4762	350.1	5570	4762	302.2	7660	4762	303.6	10370
4763	350.1	5560	4763	302.2	7640	4763	303.6	10350
4764	350.1	5570	4764	302.2	7640	4764	303.6	10350
4765	350.1	5570	4765	302.2	7640	4765	303.7	10350
4766	350.1	5550	4766	302.2	7640	4766	303.7	10260
4767	350	5560	4767	302.2	7640	4767	303.6	10260
4768	350	5570	4768	302.2	7640	4768	303.6	10370
4769	350	5570	4769	302.2	7640	4769	303.6	10260
4770	350	5570	4770	302.2	7640	4770	303.6	10360
4771	350	5560	4771	302.2	7650	4771	303.7	10360
4772	350	5560	4772	302.2	7630	4772	303.7	10250
4773	350.1	5560	4773	302.2	7640	4773	303.7	10260
4774	350.1	5560	4774	302.3	7640	4774	303.7	10260
4775	350.1	5560	4775	302.3	7630	4775	303.6	10250
4776	350.1	5560	4776	302.3	7620	4776	303.6	10240
4777	350.2	5570	4777	302.3	7640	4777	303.6	10250
4778	350.1	5560	4778	302.3	7640	4778	303.6	10250
4779	350.1	5570	4779	302.3	7650	4779	303.6	10250
4780	350.1	5570	4780	302.3	7640	4780	303.6	10350
4781	350.1	5560	4781	302.3	7630	4781	303.6	10350
4782	350.1	5570	4782	302.2	7630	4782	303.6	10360
4783	350.1	5570	4783	302.2	7630	4783	303.6	10260
4784	350.1	5570	4784	302.2	7630	4784	303.6	10370
4785	350.1	5570	4785	302.2	7630	4785	303.6	10250
4786	350.1	5570	4786	302.2	7630	4786	303.6	10360
4787	350.1	5570	4787	302.2	7630	4787	303.6	10360
4788	350.1	5580	4788	302.2	7620	4788	303.7	10280
4789	350.1	5560	4789	302.2	7640	4789	303.7	10260
4790	350.1	5560	4790	302.2	7640	4790	303.7	10260
4791	350.1	5560	4791	302.2	7650	4791	303.7	10260
4792	350.2	5570	4792	302.2	7640	4792	303.6	10260
4793	350.2	5560	4793	302.2	7640	4793	303.7	10350
4794	350.1	5570	4794	302.2	7640	4794	303.7	10270
4795	350.1	5570	4795	302.2	7640	4795	303.7	10270
4796	350.1	5560	4796	302.3	7630	4796	303.7	10250

4797	350.1	5570	4797	302.3	7640	4797	303.7	10250
4798	350.1	5580	4798	302.3	7640	4798	303.7	10260
4799	350.1	5570	4799	302.3	7640	4799	303.7	10250
4800	350.1	5570	4800	302.2	7640	4800	303.7	10360
4801	350.1	5570	4801	302.3	7650	4801	303.7	10260
4802	350.1	5590	4802	302.2	7640	4802	303.8	10270
4803	350.1	5580	4803	302.3	7630	4803	303.8	10260
4804	350.1	5580	4804	302.3	7650	4804	303.8	10260
4805	350.1	5580	4805	302.3	7640	4805	303.8	10260
4806	350.1	5570	4806	302.3	7640	4806	303.8	10360
4807	350.1	5580	4807	302.3	7640	4807	303.8	10260
4808	350.1	5580	4808	302.3	7640	4808	303.8	10250
4809	350.1	5560	4809	302.3	7650	4809	303.8	10260
4810	350.1	5580	4810	302.2	7640	4810	303.8	10260
4811	350.2	5580	4811	302.3	7640	4811	303.8	10270
4812	350.2	5570	4812	302.3	7640	4812	303.8	10260
4813	350.2	5570	4813	302.3	7640	4813	303.8	10360
4814	350.2	5580	4814	302.3	7650	4814	303.8	10260
4815	350.2	5570	4815	302.4	7640	4815	303.8	10260
4816	350.2	5580	4816	302.3	7640	4816	303.8	10260
4817	350.1	5570	4817	302.3	7640	4817	303.9	10270
4818	350.2	5570	4818	302.4	7630	4818	303.9	10260
4819	350.2	5560	4819	302.4	7640	4819	303.8	10260
4820	350.1	5570	4820	302.4	7640	4820	303.9	10250
4821	350.1	5570	4821	302.3	7640	4821	303.9	10260
4822	350.1	5570	4822	302.4	7640	4822	303.8	10270
4823	350.1	5570	4823	302.4	7640	4823	303.9	10270
4824	350.1	5570	4824	302.4	7640	4824	303.9	10260
4825	350.1	5580	4825	302.4	7640	4825	303.9	10260
4826	350.1	5580	4826	302.4	7630	4826	303.9	10260
4827	350.1	5580	4827	302.4	7630	4827	303.9	10360
4828	350.2	5560	4828	302.4	7630	4828	303.9	10260
4829	350.2	5570	4829	302.4	7640	4829	303.9	10260
4830	350.2	5570	4830	302.5	7630	4830	303.9	10360
4831	350.2	5580	4831	302.5	7640	4831	303.9	10250
4832	350.2	5570	4832	302.5	7640	4832	303.9	10250
4833	350.1	5570	4833	302.5	7640	4833	303.9	10250
4834	350.1	5580	4834	302.5	7630	4834	303.8	10270
4835	350.1	5570	4835	302.6	7630	4835	303.8	10270
4836	350.1	5580	4836	302.6	7630	4836	303.8	10260
4837	350.1	5580	4837	302.6	7630	4837	303.8	10270
4838	350.1	5590	4838	302.6	7630	4838	303.8	10260
4839	350.2	5580	4839	302.5	7640	4839	303.8	10260
4840	350.2	5580	4840	302.5	7640	4840	303.8	10250
4841	350.2	5570	4841	302.5	7640	4841	303.9	10250
4842	350.2	5570	4842	302.6	7630	4842	303.9	10260
4843	350.2	5580	4843	302.6	7640	4843	303.9	10270
4844	350.1	5570	4844	302.5	7630	4844	303.9	10250
4845	350.1	5570	4845	302.5	7640	4845	303.9	10250
4846	350.1	5580	4846	302.5	7640	4846	303.9	10260

4847	350	5580	4847	302.5	7650	4847	303.9	10260
4848	350	5560	4848	302.5	7630	4848	303.9	10250
4849	350	5570	4849	302.6	7650	4849	303.9	10260
4850	350	5570	4850	302.6	7650	4850	303.9	10270
4851	350	5580	4851	302.5	7640	4851	303.9	10270
4852	350	5570	4852	302.6	7640	4852	303.9	10270
4853	349.9	5570	4853	302.6	7630	4853	303.9	10280
4854	349.9	5580	4854	302.6	7640	4854	303.9	10260
4855	349.9	5570	4855	302.6	7650	4855	303.9	10250
4856	349.9	5570	4856	302.6	7630	4856	303.9	10250
4857	349.9	5580	4857	302.6	7640	4857	304	10260
4858	349.8	5580	4858	302.6	7640	4858	304	10260
4859	349.8	5570	4859	302.6	7640	4859	304	10260
4860	349.8	5570	4860	302.6	7640	4860	304	10260
4861	349.9	5570	4861	302.6	7650	4861	303.9	10260
4862	349.8	5570	4862	302.6	7640	4862	303.9	10260
4863	349.8	5570	4863	302.6	7640	4863	303.9	10250
4864	349.8	5560	4864	302.6	7630	4864	304	10250
4865	349.7	5580	4865	302.6	7640	4865	303.9	10250
4866	349.8	5570	4866	302.6	7640	4866	303.9	10250
4867	349.8	5560	4867	302.6	7630	4867	303.9	10250
4868	349.8	5580	4868	302.6	7630	4868	303.9	10240
4869	349.8	5580	4869	302.6	7630	4869	303.9	10260
4870	349.9	5570	4870	302.6	7640	4870	303.9	10260
4871	349.9	5570	4871	302.6	7630	4871	304	10250
4872	349.8	5580	4872	302.5	7620	4872	304	10260
4873	349.8	5570	4873	302.5	7620	4873	304	10260
4874	349.8	5590	4874	302.5	7630	4874	304	10250
4875	349.9	5580	4875	302.6	7640	4875	304	10250
4876	349.9	5570	4876	302.6	7640	4876	304	10260
4877	349.9	5570	4877	302.6	7650	4877	304	10250
4878	350	5580	4878	302.6	7640	4878	304	10250
4879	349.9	5580	4879	302.5	7640	4879	304.1	10250
4880	349.9	5580	4880	302.5	7630	4880	304.1	10250
4881	349.9	5570	4881	302.5	7640	4881	304.1	10260
4882	349.9	5600	4882	302.5	7640	4882	304.2	10250
4883	349.8	5570	4883	302.5	7620	4883	304.2	10250
4884	349.8	5570	4884	302.5	7620	4884	304.2	10250
4885	349.8	5580	4885	302.5	7630	4885	304.3	10260
4886	349.8	5570	4886	302.5	7640	4886	304.2	10260
4887	349.9	5590	4887	302.5	7640	4887	304.2	10250
4888	349.9	5570	4888	302.5	7640	4888	304.2	10240
4889	349.9	5580	4889	302.5	7640	4889	304.2	10250
4890	349.9	5560	4890	302.5	7630	4890	304.3	10260
4891	349.9	5570	4891	302.5	7640	4891	304.3	10250
4892	349.8	5580	4892	302.5	7640	4892	304.3	10260
4893	349.8	5560	4893	302.5	7660	4893	304.3	10270
4894	349.8	5570	4894	302.5	7660	4894	304.3	10270
4895	349.8	5570	4895	302.5	7640	4895	304.2	10270
4896	349.8	5580	4896	302.5	7640	4896	304.2	10260

4897	349.8	5570	4897	302.5	7640	4897	304.2	10250
4898	349.9	5580	4898	302.6	7640	4898	304.2	10240
4899	349.8	5570	4899	302.6	7640	4899	304.2	10250
4900	349.9	5570	4900	302.5	7630	4900	304.2	10260
4901	349.8	5570	4901	302.5	7640	4901	304.3	10250
4902	349.8	5560	4902	302.5	7630	4902	304.3	10260
4903	349.8	5560	4903	302.5	7640	4903	304.3	10250
4904	349.8	5570	4904	302.5	7650	4904	304.3	10250
4905	349.8	5570	4905	302.5	7650	4905	304.3	10260
4906	349.9	5560	4906	302.6	7640	4906	304.3	10250
4907	349.9	5550	4907	302.5	7640	4907	304.2	10250
4908	349.9	5560	4908	302.5	7650	4908	304.2	10240
4909	349.9	5570	4909	302.5	7650	4909	304.3	10240
4910	349.9	5580	4910	302.5	7630	4910	304.3	10260
4911	349.9	5570	4911	302.5	7640	4911	304.3	10260
4912	349.8	5560	4912	302.5	7640	4912	304.3	10250
4913	349.9	5580	4913	302.5	7640	4913	304.3	10270
4914	349.9	5560	4914	302.6	7640	4914	304.3	10260
4915	349.9	5570	4915	302.5	7640	4915	304.3	10250
4916	349.8	5570	4916	302.5	7640	4916	304.3	10250
4917	349.8	5560	4917	302.5	7650	4917	304.3	10250
4918	349.7	5560	4918	302.6	7640	4918	304.3	10240
4919	349.7	5570	4919	302.5	7650	4919	304.3	10250
4920	349.7	5560	4920	302.5	7640	4920	304.3	10250
4921	349.7	5560	4921	302.6	7640	4921	304.3	10250
4922	349.7	5560	4922	302.5	7650	4922	304.3	10250
4923	349.7	5570	4923	302.5	7640	4923	304.3	10250
4924	349.7	5580	4924	302.6	7640	4924	304.2	10250
4925	349.7	5570	4925	302.5	7640	4925	304.2	10270
4926	349.7	5560	4926	302.6	7660	4926	304.2	10270
4927	349.8	5570	4927	302.6	7640	4927	304.2	10260
4928	349.8	5580	4928	302.6	7640	4928	304.2	10250
4929	349.8	5570	4929	302.6	7650	4929	304.2	10250
4930	349.8	5580	4930	302.6	7650	4930	304.2	10250
4931	349.8	5560	4931	302.5	7650	4931	304.2	10270
4932	349.8	5570	4932	302.5	7660	4932	304.2	10260
4933	349.8	5560	4933	302.5	7660	4933	304.3	10270
4934	349.8	5560	4934	302.6	7650	4934	304.3	10250
4935	349.8	5570	4935	302.5	7650	4935	304.3	10250
4936	349.8	5580	4936	302.5	7640	4936	304.3	10260
4937	349.9	5570	4937	302.5	7660	4937	304.3	10250
4938	349.9	5560	4938	302.5	7640	4938	304.3	10260
4939	349.9	5570	4939	302.5	7630	4939	304.3	10250
4940	349.9	5570	4940	302.5	7650	4940	304.2	10240
4941	349.8	5570	4941	302.4	7650	4941	304.2	10250
4942	349.8	5560	4942	302.5	7650	4942	304.2	10250
4943	349.8	5560	4943	302.5	7640	4943	304.2	10250
4944	349.9	5570	4944	302.5	7650	4944	304.2	10260
4945	349.9	5560	4945	302.6	7650	4945	304.2	10250
4946	349.9	5560	4946	302.6	7640	4946	304.2	10260

4947	349.9	5570	4947	302.6	7640	4947	304.2	10250
4948	349.9	5570	4948	302.5	7640	4948	304.2	10260
4949	349.9	5560	4949	302.5	7640	4949	304.2	10250
4950	349.9	5570	4950	302.5	7640	4950	304.2	10250
4951	349.9	5570	4951	302.4	7650	4951	304.2	10250
4952	349.9	5570	4952	302.4	7650	4952	304.2	10260
4953	349.9	5570	4953	302.4	7650	4953	304.2	10260
4954	349.9	5560	4954	302.4	7650	4954	304.2	10270
4955	349.9	5570	4955	302.4	7650	4955	304.3	10260
4956	349.9	5570	4956	302.4	7640	4956	304.2	10260
4957	349.9	5580	4957	302.4	7650	4957	304.3	10260
4958	349.9	5580	4958	302.4	7640	4958	304.2	10260
4959	349.9	5580	4959	302.5	7640	4959	304.3	10230
4960	349.9	5570	4960	302.5	7650	4960	304.3	10260
4961	349.9	5570	4961	302.5	7650	4961	304.3	10260
4962	349.9	5580	4962	302.5	7630	4962	304.3	10260
4963	349.9	5590	4963	302.5	7630	4963	304.3	10260
4964	349.9	5580	4964	302.5	7630	4964	304.3	10250
4965	349.9	5570	4965	302.5	7640	4965	304.3	10260
4966	349.9	5560	4966	302.5	7650	4966	304.3	10250
4967	349.8	5480	4967	302.5	7630	4967	304.4	10240
4968	349.8	5560	4968	302.5	7640	4968	304.4	10250
4969	349.8	5560	4969	302.5	7650	4969	304.4	10250
4970	349.7	5560	4970	302.4	7640	4970	304.5	10250
4971	349.7	5450	4971	302.5	7630	4971	304.5	10240
4972	349.8	5470	4972	302.5	7630	4972	304.4	10260
4973	349.8	5570	4973	302.5	7640	4973	304.4	10250
4974	349.8	5470	4974	302.5	7650	4974	304.4	10240
4975	349.8	5470	4975	302.5	7620	4975	304.4	10250
4976	349.8	5550	4976	302.5	7630	4976	304.4	10240
4977	349.8	5550	4977	302.5	7630	4977	304.4	10230
4978	349.8	5450	4978	302.5	7640	4978	304.4	10250
4979	349.8	5560	4979	302.6	7640	4979	304.4	10240
4980	349.8	5570	4980	302.6	7640	4980	304.4	10250
4981	349.9	5460	4981	302.6	7650	4981	304.4	10250
4982	349.8	5460	4982	302.5	7640	4982	304.4	10260
4983	349.8	5460	4983	302.6	7640	4983	304.4	10260
4984	349.9	5470	4984	302.6	7640	4984	304.4	10240
4985	349.9	5460	4985	302.6	7630	4985	304.5	10240
4986	349.9	5460	4986	302.6	7630	4986	304.5	10250
4987	350	5580	4987	302.6	7640	4987	304.6	10250
4988	350	5470	4988	302.6	7650	4988	304.6	10250
4989	350	5560	4989	302.5	7650	4989	304.6	10260
4990	350	5560	4990	302.5	7630	4990	304.6	10260
4991	350	5560	4991	302.6	7640	4991	304.6	10260
4992	350	5470	4992	302.6	7640	4992	304.5	10260
4993	350	5460	4993	302.6	7650	4993	304.5	10260
4994	350	5560	4994	302.6	7640	4994	304.5	10240
4995	350	5470	4995	302.6	7630	4995	304.5	10260
4996	350	5460	4996	302.5	7640	4996	304.6	10250

4997	350.1	5470	4997	302.5	7640	4997	304.6	10250
4998	350.1	5470	4998	302.5	7640	4998	304.5	10260
4999	350.1	5460	4999	302.6	7640	4999	304.5	10260
5000	350.1	5560	5000	302.6	7640	5000	304.5	10250
5001	350.1	5460	5001	302.6	7630	5001	304.5	10260
5002	350.1	5460	5002	302.6	7640	5002	304.5	10250
5003	350.1	5470	5003	302.6	7630	5003	304.5	10270
5004	350.1	5470	5004	302.6	7640	5004	304.6	10250
5005	350.1	5460	5005	302.6	7630	5005	304.6	10250
5006	350.1	5570	5006	302.6	7620	5006	304.6	10260
5007	350.1	5460	5007	302.6	7640	5007	304.6	10240
5008	350.1	5570	5008	302.6	7650	5008	304.5	10250
5009	350.1	5560	5009	302.6	7640	5009	304.5	10250
5010	350.1	5460	5010	302.6	7640	5010	304.5	10260
5011	350.1	5470	5011	302.6	7640	5011	304.5	10250
5012	350	5460	5012	302.6	7630	5012	304.5	10250
5013	350	5460	5013	302.5	7640	5013	304.5	10250
5014	350.1	5460	5014	302.6	7640	5014	304.4	10250
5015	350.1	5460	5015	302.5	7650	5015	304.5	10260
5016	350	5450	5016	302.5	7640	5016	304.5	10240
5017	350.1	5450	5017	302.6	7640	5017	304.5	10250
5018	350.1	5460	5018	302.6	7620	5018	304.5	10240
5019	350.1	5470	5019	302.6	7640	5019	304.5	10260
5020	350	5460	5020	302.6	7630	5020	304.5	10250
5021	350	5470	5021	302.6	7640	5021	304.5	10250
5022	350	5460	5022	302.6	7630	5022	304.6	10240
5023	350	5560	5023	302.6	7630	5023	304.6	10250
5024	350	5460	5024	302.6	7620	5024	304.6	10250
5025	350	5460	5025	302.6	7630	5025	304.6	10240
5026	349.9	5470	5026	302.6	7630	5026	304.6	10250
5027	350	5560	5027	302.6	7630	5027	304.7	10260
5028	350	5560	5028	302.6	7640	5028	304.6	10240
5029	350	5470	5029	302.6	7630	5029	304.7	10250
5030	350	5460	5030	302.6	7640	5030	304.7	10250
5031	350	5470	5031	302.6	7650	5031	304.7	10250
5032	350	5460	5032	302.6	7640	5032	304.6	10250
5033	350	5460	5033	302.6	7630	5033	304.6	10240
5034	350	5470	5034	302.6	7620	5034	304.6	10250
5035	350	5460	5035	302.6	7630	5035	304.7	10250
5036	350	5460	5036	302.6	7640	5036	304.6	10240
5037	350	5450	5037	302.6	7650	5037	304.6	10260
5038	350	5460	5038	302.5	7630	5038	304.6	10250
5039	350	5470	5039	302.6	7640	5039	304.6	10240
5040	350	5460	5040	302.5	7640	5040	304.6	10250
5041	350	5470	5041	302.5	7630	5041	304.6	10240
5042	350	5470	5042	302.6	7640	5042	304.5	10240
5043	350	5460	5043	302.6	7640	5043	304.5	10240
5044	350	5470	5044	302.6	7630	5044	304.5	10240
5045	350	5470	5045	302.6	7640	5045	304.5	10240
5046	350	5470	5046	302.6	7640	5046	304.5	10240

5047	350	5480	5047	302.6	7640	5047	304.6	10240
5048	350	5480	5048	302.6	7640	5048	304.5	10250
5049	350	5480	5049	302.6	7640	5049	304.5	10240
5050	350	5470	5050	302.6	7640	5050	304.5	10260
5051	350	5480	5051	302.6	7640	5051	304.6	10250
5052	350.1	5470	5052	302.7	7640	5052	304.6	10260
5053	350.1	5460	5053	302.7	7640	5053	304.6	10240
5054	350.1	5470	5054	302.6	7640	5054	304.5	10250
5055	350	5460	5055	302.6	7640	5055	304.5	10250
5056	350.1	5460	5056	302.6	7640	5056	304.5	10240
5057	350.1	5460	5057	302.6	7640	5057	304.5	10240
5058	350.1	5460	5058	302.6	7640	5058	304.6	10240
5059	350.1	5460	5059	302.6	7640	5059	304.6	10250
5060	350.1	5460	5060	302.7	7650	5060	304.6	10250
5061	350.1	5460	5061	302.7	7630	5061	304.6	10250
5062	350.1	5460	5062	302.7	7640	5062	304.6	10250
5063	350.1	5470	5063	302.6	7620	5063	304.6	10260
5064	350.1	5470	5064	302.7	7630	5064	304.6	10250
5065	350.1	5470	5065	302.6	7640	5065	304.6	10250
5066	350.1	5470	5066	302.7	7640	5066	304.6	10250
5067	350.1	5460	5067	302.6	7640	5067	304.7	10250
5068	350.1	5460	5068	302.7	7630	5068	304.6	10250
5069	350	5470	5069	302.7	7630	5069	304.7	10240
5070	350	5460	5070	302.7	7640	5070	304.7	10250
5071	350	5470	5071	302.6	7630	5071	304.7	10250
5072	350	5470	5072	302.7	7640	5072	304.7	10260
5073	350	5470	5073	302.7	7640	5073	304.6	10250
5074	350	5460	5074	302.7	7630	5074	304.6	10270
5075	350	5470	5075	302.7	7640	5075	304.6	10250
5076	349.9	5470	5076	302.7	7650	5076	304.6	10240
5077	350	5460	5077	302.7	7630	5077	304.6	10250
5078	350	5460	5078	302.7	7630	5078	304.6	10250
5079	350	5480	5079	302.7	7640	5079	304.6	10250
5080	350	5470	5080	302.7	7640	5080	304.6	10250
5081	350	5470	5081	302.7	7640	5081	304.6	10250
5082	350.1	5470	5082	302.6	7630	5082	304.6	10260
5083	350.1	5470	5083	302.6	7640	5083	304.6	10240
5084	350	5460	5084	302.7	7630	5084	304.6	10260
5085	350.1	5440	5085	302.6	7620	5085	304.6	10250
5086	350.1	5460	5086	302.6	7640	5086	304.6	10240
5087	350.1	5460	5087	302.6	7630	5087	304.5	10250
5088	350.1	5460	5088	302.6	7640	5088	304.5	10250
5089	350	5460	5089	302.6	7630	5089	304.6	10240
5090	350	5460	5090	302.6	7640	5090	304.5	10240
5091	350	5460	5091	302.6	7640	5091	304.6	10240
5092	350	5470	5092	302.7	7640	5092	304.6	10240
5093	350	5460	5093	302.7	7620	5093	304.6	10240
5094	349.9	5460	5094	302.7	7630	5094	304.6	10250
5095	350	5460	5095	302.6	7620	5095	304.6	10250
5096	349.9	5470	5096	302.7	7640	5096	304.6	10250

5097	349.9	5470	5097	302.7	7630	5097	304.6	10250
5098	349.9	5470	5098	302.7	7630	5098	304.6	10260
5099	349.9	5460	5099	302.7	7620	5099	304.6	10250
5100	349.9	5470	5100	302.7	7620	5100	304.6	10250
5101	349.9	5470	5101	302.7	7630	5101	304.6	10250
5102	349.9	5490	5102	302.8	7640	5102	304.6	10250
5103	349.9	5460	5103	302.8	7630	5103	304.7	10250
5104	349.9	5470	5104	302.7	7640	5104	304.7	10260
5105	349.9	5460	5105	302.7	7620	5105	304.7	10250
5106	349.9	5470	5106	302.7	7630	5106	304.7	10250
5107	349.9	5470	5107	302.7	7620	5107	304.7	10250
5108	349.9	5460	5108	302.7	7630	5108	304.7	10270
5109	349.9	5450	5109	302.7	7640	5109	304.7	10250
			5110	302.8	7640	5110	304.7	10250
			5111	302.8	7640	5111	304.7	10240
			5112	302.7	7640	5112	304.6	10250
			5113	302.7	7630	5113	304.6	10250
			5114	302.8	7640	5114	304.6	10250
			5115	302.8	7640	5115	304.6	10250
			5116	302.8	7620	5116	304.6	10240
			5117	302.8	7640	5117	304.6	10240
			5118	302.8	7630	5118	304.6	10250
			5119	302.8	7630	5119	304.6	10240
			5120	302.8	7640	5120	304.7	10250
			5121	302.8	7630	5121	304.7	10250
			5122	302.9	7640	5122	304.7	10250
			5123	302.9	7640	5123	304.7	10240
			5124	302.8	7640	5124	304.7	10260
			5125	302.8	7630	5125	304.7	10260
			5126	302.8	7640	5126	304.6	10260
			5127	302.8	7640	5127	304.6	10260
			5128	302.8	7630	5128	304.7	10250
			5129	302.8	7650	5129	304.7	10250
			5130	302.8	7640	5130	304.6	10250
			5131	302.8	7650	5131	304.6	10260
			5132	302.8	7640	5132	304.6	10260
			5133	302.8	7630	5133	304.6	10280
			5134	302.8	7660	5134	304.6	10270
			5135	302.8	7640	5135	304.6	10250
			5136	302.8	7640	5136	304.6	10250
			5137	302.8	7640	5137	304.6	10250
			5138	302.8	7650	5138	304.6	10250
			5139	302.9	7640	5139	304.6	10250
			5140	302.9	7640	5140	304.7	10250
			5141	302.9	7640	5141	304.6	10270
			5142	302.9	7630	5142	304.5	10250
			5143	302.9	7630	5143	304.5	10250
			5144	302.9	7630	5144	304.4	10250
			5145	302.9	7630	5145	304.3	10240
			5146	302.9	7640	5146	304.3	10250

			5147	302.9	7630	5147	304.2	10250
			5148	302.9	7640	5148	304.2	10260
			5149	302.9	7640	5149	304.2	10250
			5150	302.9	7620	5150	304.1	10250
			5151	302.9	7630	5151	304	10260
			5152	302.9	7630	5152	304	10250
			5153	302.9	7630	5153	304	10250
			5154	302.9	7630	5154	303.9	10260
			5155	302.8	7630	5155	303.9	10260
			5156	302.8	7620	5156	303.8	10250
			5157	302.8	7640	5157	303.8	10260
			5158	302.8	7650	5158	303.8	10250
			5159	302.8	7630	5159	303.8	10260
			5160	302.8	7630	5160	303.8	10270
			5161	302.8	7630	5161	303.8	10270
			5162	302.8	7630	5162	303.8	10260
			5163	302.8	7630	5163	303.8	10150
			5164	302.8	7640	5164	303.8	10260
			5165	302.8	7630	5165	303.7	10260
			5166	302.7	7620	5166	303.7	10250
			5167	302.8	7620	5167	303.7	10260
			5168	302.8	7630	5168	303.7	10250
			5169	302.8	7610	5169	303.7	10240
			5170	302.8	7630	5170	303.6	10250
			5171	302.8	7610	5171	303.6	10250
			5172	302.8	7620	5172	303.6	10260
			5173	302.8	7630	5173	303.6	10260
			5174	302.8	7620	5174	303.6	10250
			5175	302.8	7630	5175	303.5	10150
			5176	302.8	7620	5176	303.5	10270
			5177	302.8	7620	5177	303.6	10260
			5178	302.8	7620	5178	303.6	10250
			5179	302.8	7630	5179	303.5	10250
			5180	302.8	7620	5180	303.5	10160
			5181	302.8	7630	5181	303.5	10260
			5182	302.8	7620	5182	303.5	10260
			5183	302.8	7630	5183	303.4	10260
			5184	302.8	7630	5184	303.4	10170
			5185	302.8	7630	5185	303.4	10270
			5186	302.8	7630	5186	303.3	10250
			5187	302.8	7640	5187	303.3	10170
			5188	302.8	7620	5188	303.3	10150
			5189	302.8	7630	5189	303.4	10160
			5190	302.8	7630	5190	303.4	10170
			5191	302.8	7620	5191	303.4	10160
			5192	302.8	7630	5192	303.4	10170
			5193	302.7	7630	5193	303.4	10270
			5194	302.7	7630	5194	303.4	10260
			5195	302.7	7630	5195	303.4	10170
			5196	302.7	7630	5196	303.4	10150

			5197	302.7	7630	5197	303.4	10170
			5198	302.7	7630	5198	303.4	10160
			5199	302.7	7620	5199	303.4	10160
			5200	302.7	7630	5200	303.4	10150
			5201	302.7	7630	5201	303.3	10150
			5202	302.7	7640	5202	303.3	10250
			5203	302.7	7640	5203	303.3	10260
			5204	302.7	7640	5204	303.3	10250
			5205	302.7	7630	5205	303.3	10260
			5206	302.7	7630	5206	303.2	10260
			5207	302.7	7630	5207	303.2	10150
			5208	302.7	7620	5208	303.2	10160
			5209	302.8	7630	5209	303.2	10150
			5210	302.8	7620	5210	303.1	10150
			5211	302.8	7630	5211	303.2	10150
			5212	302.9	7620	5212	303.2	10250
			5213	302.9	7630	5213	303.2	10260
			5214	302.9	7630	5214	303.2	10160
			5215	302.9	7630	5215	303.1	10250
			5216	302.8	7630	5216	303.1	10250
			5217	302.8	7620	5217	303.1	10150
			5218	302.8	7630	5218	303.1	10160
			5219	302.8	7630	5219	303.1	10150
			5220	302.8	7630	5220	303.1	10160
			5221	302.8	7620	5221	303.1	10150
			5222	302.7	7630	5222	303.1	10250
			5223	302.7	7630	5223	303.1	10170
			5224	302.8	7630	5224	303.1	10250
			5225	302.8	7630	5225	303.1	10150
			5226	302.8	7620	5226	303.1	10240
			5227	302.9	7630	5227	303.1	10240
			5228	302.9	7630	5228	303.1	10160
			5229	303	7620	5229	303.1	10160
			5230	303	7630	5230	303.1	10150
			5231	303	7610	5231	303.1	10150
			5232	303	7620	5232	303	10150
			5233	303	7620	5233	303.1	10160
			5234	303	7630	5234	303.1	10160
			5235	303	7630	5235	303.1	10160
			5236	302.9	7630	5236	303.1	10160
			5237	302.9	7620	5237	303	10160
			5238	302.9	7640	5238	303	10170
			5239	302.9	7630	5239	303	10160
			5240	303	7630	5240	303	10160
			5241	303	7620	5241	303	10160
			5242	303	7630	5242	303	10160
			5243	303	7650	5243	303	10260
			5244	303	7630	5244	303	10170
			5245	303	7640	5245	303	10170
			5246	303	7640	5246	303	10270

			5247	302.9	7630	5247	303	10160
			5248	302.9	7620	5248	303	10160
			5249	302.9	7630	5249	303	10160
			5250	302.9	7610	5250	303	10150
			5251	302.8	7630	5251	303	10160
			5252	302.8	7630	5252	303	10150
			5253	302.8	7630	5253	303	10160
			5254	302.8	7630	5254	303	10160
			5255	302.9	7630	5255	303	10140
			5256	302.8	7630	5256	303	10140
			5257	302.8	7630	5257	303	10150
			5258	302.8	7640	5258	303	10160
			5259	302.8	7630	5259	303	10160
			5260	302.7	7620	5260	303	10150
			5261	302.7	7640	5261	303	10160
			5262	302.8	7620	5262	303	10160
			5263	302.8	7630	5263	303	10160
			5264	302.9	7630	5264	302.9	10150
			5265	302.8	7630	5265	302.9	10150
			5266	302.9	7630	5266	302.9	10260
			5267	302.8	7630	5267	303	10160
			5268	302.9	7620	5268	303	10150
			5269	302.8	7630	5269	303	10150
			5270	302.8	7630	5270	303	10150
			5271	302.9	7620	5271	303	10160
			5272	302.9	7620	5272	303.1	10260
			5273	302.9	7620	5273	303.1	10150
			5274	302.9	7630	5274	303.1	10140
			5275	302.9	7640	5275	303.1	10150
			5276	302.9	7640	5276	303.1	10250
			5277	303	7630	5277	303.1	10150
			5278	302.9	7640	5278	303.1	10170
			5279	303	7640	5279	303	10150
			5280	302.9	7630	5280	303	10150
			5281	302.9	7640	5281	303	10150
			5282	303	7650	5282	303	10150
			5283	303	7640	5283	303	10150
			5284	303	7620	5284	303.1	10160
			5285	302.9	7640	5285	303.1	10160
			5286	302.9	7640	5286	303	10150
			5287	302.9	7640	5287	303.1	10150
			5288	302.9	7640	5288	303.1	10160
			5289	302.9	7640	5289	303.1	10160
			5290	302.9	7640	5290	303.1	10160
			5291	302.8	7640	5291	303.1	10160
			5292	302.8	7640	5292	303.1	10160
			5293	302.9	7640	5293	303	10150
			5294	303	7630	5294	303.1	10150
			5295	302.9	7640	5295	303	10160
			5296	302.9	7640	5296	303	10150

			5297	302.9	7640	5297	303	10150
			5298	302.9	7630	5298	303	10260
			5299	302.9	7640	5299	303	10160
			5300	302.9	7640	5300	303	10150
			5301	302.9	7630	5301	303	10160
			5302	302.9	7630	5302	303.1	10160
			5303	302.8	7630	5303	303.1	10150
			5304	302.8	7640	5304	303	10270
			5305	302.9	7620	5305	303	10150
			5306	302.9	7620	5306	303	10160
			5307	302.9	7630	5307	303	10160
			5308	302.9	7630	5308	302.9	10160
			5309	302.9	7640	5309	303	10160
			5310	303	7640	5310	302.9	10160
			5311	303	7640	5311	302.9	10160
			5312	303	7630	5312	302.9	10160
			5313	303	7630	5313	302.9	10150
			5314	302.9	7620	5314	303	10150
			5315	302.9	7620	5315	303	10160
			5316	303	7640	5316	303	10160
			5317	303	7630	5317	303	10150
			5318	303	7630	5318	303	10140
			5319	302.9	7630	5319	303	10150
			5320	302.9	7630	5320	303	10150
			5321	302.9	7630	5321	302.9	10150
			5322	302.9	7630	5322	303	10150
			5323	302.9	7620	5323	303	10170
			5324	302.9	7630	5324	303	10140
			5325	303	7630	5325	302.9	10140
			5326	303	7640	5326	302.9	10140
			5327	303	7630	5327	302.9	10140
			5328	303	7640	5328	302.9	10140
			5329	303	7640	5329	302.9	10150
			5330	302.9	7630	5330	302.9	10150
			5331	302.9	7630	5331	303	10150
			5332	302.8	7630	5332	302.9	10140
			5333	302.8	7640	5333	302.9	10150
			5334	302.8	7630	5334	302.9	10140
			5335	302.8	7630	5335	302.8	10140
			5336	302.8	7620	5336	302.9	10160
			5337	302.8	7620	5337	302.8	10150
			5338	302.8	7620	5338	302.9	10140
			5339	302.8	7640	5339	302.9	10130
			5340	302.8	7620	5340	302.9	10150
			5341	302.8	7630	5341	302.9	10140
			5342	302.8	7630	5342	302.9	10160
			5343	302.8	7630	5343	302.9	10160
			5344	302.8	7620	5344	302.9	10150
			5345	302.7	7630	5345	303	10150
			5346	302.7	7620	5346	302.9	10160

			5347	302.7	7620	5347	302.9	10140
			5348	302.6	7620	5348	302.9	10150
			5349	302.6	7640	5349	302.9	10150
			5350	302.6	7620	5350	302.9	10140
			5351	302.6	7640	5351	302.9	10160
			5352	302.7	7630	5352	302.9	10160
			5353	302.7	7620	5353	303	10150
			5354	302.7	7630	5354	303	10160
			5355	302.7	7630	5355	303	10150
			5356	302.7	7620	5356	303	10140
			5357	302.7	7620	5357	303	10130
			5358	302.7	7640	5358	303	10140
			5359	302.8	7630	5359	303	10150
			5360	302.7	7620	5360	302.9	10150
			5361	302.7	7630	5361	303	10140
			5362	302.7	7630	5362	303	10140
			5363	302.7	7630	5363	303.1	10150
			5364	302.7	7630	5364	303.1	10150
			5365	302.7	7620	5365	303.1	10160
			5366	302.7	7620	5366	303.1	10140
			5367	302.7	7620	5367	303.1	10140
			5368	302.7	7630	5368	303.1	10150
			5369	302.7	7630	5369	303.1	10160
			5370	302.8	7620	5370	303.1	10140
			5371	302.8	7630	5371	303.1	10150
			5372	302.8	7620	5372	303.1	10150
			5373	302.8	7630	5373	303	10140
			5374	302.8	7630	5374	303	10140
			5375	302.7	7620	5375	303	10150
			5376	302.7	7630	5376	303	10150
			5377	302.7	7620	5377	303	10150
			5378	302.7	7620	5378	303	10150
			5379	302.7	7620	5379	303	10150
			5380	302.7	7630	5380	303	10160
			5381	302.7	7630	5381	303	10150
			5382	302.7	7630	5382	303	10160
			5383	302.8	7640	5383	303.1	10160
			5384	302.8	7620	5384	303.1	10160
			5385	302.7	7630	5385	303.1	10140
			5386	302.7	7620	5386	303.1	10150
			5387	302.7	7620	5387	303.1	10150
			5388	302.7	7620	5388	303.1	10160
			5389	302.7	7630	5389	303	10160
			5390	302.8	7630	5390	303.1	10150
			5391	302.8	7630	5391	303	10160
			5392	302.8	7640	5392	303	10150
			5393	302.8	7610	5393	303	10160
			5394	302.8	7630	5394	303	10140
			5395	302.8	7630	5395	303	10150
			5396	302.8	7630	5396	303	10160

			5397	302.8	7630	5397	303	10160
			5398	302.8	7530	5398	303	10160
			5399	302.8	7640	5399	303	10150
			5400	302.8	7630	5400	303	10150
			5401	302.8	7630	5401	303	10160
			5402	302.8	7630	5402	303.1	10150
			5403	302.7	7630	5403	303.1	10160
			5404	302.7	7620	5404	303.1	10150
			5405	302.7	7640	5405	303.1	10150
			5406	302.7	7630	5406	303.1	10160
			5407	302.7	7630	5407	303.2	10160
			5408	303	7620	5408	303.2	10150
			5409	303.4	7630	5409	303.1	10150
			5410	304	7620	5410	303.1	10160
			5411	304.6	7610	5411	303.1	10160
			5412	305.1	7720	5412	303.1	10160
			5413	305.6	7720	5413	303	10150
			5414	306.1	7710	5414	303.1	10160
			5415	306.7	7710	5415	303.1	10160
			5416	307.3	7710	5416	303.1	10150
			5417	307.8	7710	5417	303.1	10160
			5418	308.4	7710	5418	303.1	10160
			5419	308.9	7700	5419	303.1	10140
			5420	309.3	7730	5420	303.2	10160
			5421	309.8	7720	5421	303.2	10150
			5422	310.2	7720	5422	303.1	10150
			5423	310.4	7720	5423	303.1	10150
			5424	310.8	7720	5424	303.2	10150
			5425	311	7720	5425	303.2	10160
			5426	311.4	7720	5426	303.2	10150
			5427	311.7	7720	5427	303.2	10150
			5428	312	7720	5428	303.2	10150
			5429	312.2	7730	5429	303.2	10150
			5430	312.5	7720	5430	303.2	10150
			5431	312.6	7720	5431	303.2	10160
			5432	312.8	7720	5432	303.2	10140
			5433	313	7730	5433	303.2	10150
			5434	313.3	7730	5434	303.2	10150
			5435	313.4	7730	5435	303.2	10150
			5436	313.6	7730	5436	303.2	10150
			5437	313.7	7730	5437	303.2	10160
			5438	313.9	7720	5438	303.2	10150
			5439	314	7730	5439	303.2	10150
			5440	314.2	7720	5440	303.2	10140
			5441	314.3	7830	5441	303.1	10150
			5442	314.5	7820	5442	303.1	10150
			5443	314.6	7820	5443	303	10140
			5444	314.7	7830	5444	303	10140
			5445	314.8	7830	5445	303	10160
			5446	314.9	7820	5446	303	10150

			5447	315.1	7840	5447	303	10150
			5448	315.1	7830	5448	303	10150
			5449	315.2	7810	5449	303	10150
			5450	315.3	7820	5450	303	10140
			5451	315.5	7830	5451	303	10140
			5452	315.6	7820	5452	303	10140
			5453	315.7	7820	5453	303.1	10150
			5454	315.8	7820	5454	303.1	10140
			5455	315.8	7810	5455	303.1	10150
			5456	316	7820	5456	303.1	10150
			5457	316	7820	5457	303	10140
			5458	316.1	7820	5458	303	10150
			5459	316.2	7830	5459	303	10140
			5460	316.2	7820	5460	303	10130
			5461	316.3	7820	5461	303	10150
			5462	316.4	7820	5462	303	10150
			5463	316.5	7830	5463	303	10150
			5464	316.6	7820	5464	303	10160
			5465	316.7	7820	5465	303	10150
			5466	316.7	7820	5466	303	10140
			5467	316.8	7830	5467	303	10150
			5468	316.9	7810	5468	303.1	10130
			5469	316.9	7820	5469	303	10140
			5470	317	7830	5470	303	10150
			5471	317.1	7820	5471	303	10140
			5472	317.2	7820	5472	303	10150
			5473	317.2	7840	5473	303	10150
			5474	317.4	7830	5474	303	10150
			5475	317.4	7830	5475	303	10150
			5476	317.5	7830	5476	303	10150
			5477	317.5	7820	5477	303	10160
			5478	317.6	7830	5478	303	10140
			5479	317.6	7820	5479	303	10140
			5480	317.7	7830	5480	303	10160
			5481	317.7	7820	5481	303	10150
			5482	317.8	7830	5482	303.1	10160
			5483	317.9	7820	5483	303.1	10150
			5484	317.9	7840	5484	303.2	10140
			5485	318	7810	5485	303.2	10150
			5486	318	7810	5486	303.2	10150
			5487	318.1	7820	5487	303.2	10140
			5488	318.2	7820	5488	303.1	10140
			5489	318.3	7810	5489	303.1	10160
			5490	318.3	7820	5490	303.1	10140
			5491	318.4	7820	5491	303.1	10160
			5492	318.4	7830	5492	303.1	10140
			5493	318.4	7830	5493	303.1	10150
			5494	318.5	7820	5494	303.1	10140
			5495	318.5	7820	5495	303.1	10140
			5496	318.6	7910	5496	303.1	10140

			5497	318.6	7820	5497	303.1	10140
			5498	318.7	7820	5498	303.1	10140
			5499	318.8	7820	5499	303.1	10140
			5500	318.8	7830	5500	303.1	10140
			5501	318.9	7820	5501	303.1	10140
			5502	319	7830	5502	303.1	10140
			5503	319	7840	5503	303.1	10140
			5504	319.1	7820	5504	303.1	10130
			5505	319.2	7920	5505	303.1	10150
			5506	319.3	7830	5506	303.1	10140
			5507	319.3	7920	5507	303.1	10140
			5508	319.4	7930	5508	303.1	10150
			5509	319.6	7930	5509	303.1	10140
			5510	319.7	7910	5510	303.1	10150
			5511	319.9	7930	5511	303	10140
			5512	320.1	7930	5512	303.1	10150
			5513	320.3	7920	5513	303	10150
			5514	320.4	7920	5514	303	10140
			5515	320.6	7920	5515	303	10130
			5516	320.7	7930	5516	303	10140
			5517	320.9	7920	5517	302.9	10140
			5518	321	7920	5518	302.9	10130
			5519	321.1	7920	5519	302.9	10140
			5520	321.2	7920	5520	303	10140
			5521	321.3	7890	5521	303	10140
			5522	321.3	7910	5522	303	10140
			5523	321.4	7920	5523	302.9	10140
			5524	321.5	7930	5524	302.9	10150
			5525	321.6	7930	5525	302.9	10140
			5526	321.8	7920	5526	302.9	10140
			5527	321.8	7920	5527	302.9	10140
			5528	321.9	7930	5528	302.9	10140
			5529	322	7940	5529	302.9	10130
			5530	322.1	7920	5530	302.9	10140
			5531	322.2	7910	5531	302.9	10150
			5532	322.3	7920	5532	302.9	10140
			5533	322.3	7920	5533	303	10140
			5534	322.3	7920	5534	303	10130
			5535	322.4	7920	5535	303	10140
			5536	322.4	7930	5536	303	10130
			5537	322.5	7920	5537	303	10140
			5538	322.5	7920	5538	302.9	10130
			5539	322.6	7910	5539	302.9	10130
			5540	322.7	7910	5540	302.9	10130
			5541	322.7	7900	5541	302.9	10150
			5542	322.8	7910	5542	302.9	10130
			5543	322.8	7910	5543	302.9	10140
			5544	322.8	7910	5544	302.9	10140
			5545	322.9	7890	5545	302.9	10140
			5546	323	7910	5546	302.9	10150

			5547	323.1	7910	5547	302.9	10140
			5548	323.1	7900	5548	302.9	10150
			5549	323.1	7910	5549	303	10140
			5550	323.1	7910	5550	303	10150
			5551	323.2	7910	5551	302.9	10160
			5552	323.2	7910	5552	302.9	10150
			5553	323.2	7920	5553	302.9	10140
			5554	323.3	7910	5554	302.9	10140
			5555	323.4	7910	5555	302.9	10150
			5556	323.4	7920	5556	302.9	10140
			5557	323.5	7930	5557	302.9	10140
			5558	323.5	7920	5558	302.9	10140
			5559	323.5	7910	5559	302.9	10140
			5560	323.6	7920	5560	302.8	10140
			5561	323.6	7920	5561	302.9	10140
			5562	323.7	7920	5562	302.9	10130
			5563	323.8	7920	5563	302.9	10140
			5564	323.8	7920	5564	302.9	10140
			5565	323.8	7930	5565	303	10140
			5566	323.9	7920	5566	303.1	10230
			5567	324	7920	5567	303.7	10240
			5568	324.1	7930	5568	304.5	10240
			5569	324.1	7930	5569	305.3	10340
			5570	324.1	7920	5570	306.1	10330
			5571	324.2	7920	5571	307	10340
			5572	324.3	7910	5572	308	10320
			5573	324.4	7920	5573	309.1	10330
			5574	324.4	7920	5574	310.1	10440
			5575	324.4	7920	5575	311.2	10430
			5576	324.4	7910	5576	312.4	10420
			5577	324.5	7920	5577	313.5	10420
			5578	324.5	7910	5578	314.6	10420
			5579	324.5	7930	5579	315.6	10430
			5580	324.6	7910	5580	316.5	10420
			5581	324.6	7920	5581	317.4	10430
			5582	324.6	7920	5582	318.1	10410
			5583	324.7	7900	5583	318.8	10520
			5584	324.7	7910	5584	319.4	10420
			5585	324.7	7900	5585	320.1	10520
			5586	324.7	7910	5586	320.7	10530
			5587	324.8	7900	5587	321.2	10520
			5588	324.8	7910	5588	321.7	10520
			5589	324.8	7900	5589	322.2	10520
			5590	324.8	7910	5590	322.7	10530
			5591	324.9	7920	5591	323.1	10520
			5592	324.9	7910	5592	323.4	10530
			5593	324.9	7910	5593	323.6	10520
			5594	325	7910	5594	323.7	10520
			5595	325	7910	5595	323.9	10530
			5596	324.9	7900	5596	324.1	10540

			5597	325	7900	5597	324.2	10530
			5598	325	7900	5598	324.4	10540
			5599	325.1	7910	5599	324.6	10520
			5600	325.1	7910	5600	324.7	10530
			5601	325.2	7890	5601	324.8	10530
			5602	325.2	7900	5602	324.9	10530
			5603	325.2	7910	5603	325	10530
			5604	325.3	7910	5604	325	10520
			5605	325.4	7920	5605	325.1	10540
			5606	325.4	7910	5606	325.2	10530
			5607	325.3	7920	5607	325.3	10540
			5608	325.4	7900	5608	325.4	10540
			5609	325.4	7920	5609	325.5	10540
			5610	325.4	7900	5610	325.7	10630
			5611	325.5	7910	5611	325.8	10520
			5612	325.5	7920	5612	325.9	10540
			5613	325.6	7910	5613	326	10530
			5614	325.6	7910	5614	326.1	10520
			5615	325.6	7910	5615	326	10530
			5616	325.7	7920	5616	326.1	10520
			5617	325.7	7910	5617	326.2	10530
			5618	325.7	7910	5618	326.3	10630
			5619	325.7	7900	5619	326.4	10520
			5620	325.7	7910	5620	326.4	10530
			5621	325.8	7920	5621	326.4	10530
			5622	325.8	7920	5622	326.4	10530
			5623	325.8	7920	5623	326.5	10610
			5624	325.8	7920	5624	326.4	10620
			5625	325.8	7900	5625	326.4	10610
			5626	325.9	7920	5626	326.4	10630
			5627	325.9	7910	5627	326.4	10540
			5628	325.9	7910	5628	326.5	10620
			5629	325.9	7900	5629	326.5	10510
			5630	325.9	7920	5630	326.5	10630
			5631	325.9	7910	5631	326.5	10520
			5632	325.9	7910	5632	326.5	10530
			5633	325.9	7910	5633	326.5	10640
			5634	325.8	7900	5634	326.4	10620
			5635	325.7	7910	5635	326.4	10530
			5636	325.7	7910	5636	326.4	10630
			5637	325.6	7910	5637	326.4	10530
			5638	325.6	7910	5638	326.4	10640
			5639	325.6	7910	5639	326.4	10540
			5640	325.6	7910	5640	326.3	10630
			5641	325.6	7920	5641	326.4	10640
			5642	325.5	7910	5642	326.4	10640
			5643	325.5	7910	5643	326.4	10640
			5644	325.5	7910	5644	326.4	10630
			5645	325.5	7910	5645	326.4	10630
			5646	325.5	7910	5646	326.4	10650

			5647	325.5	7910	5647	326.5	10630
			5648	325.4	7910	5648	326.5	10630
			5649	325.4	7900	5649	326.6	10640
			5650	325.5	7910	5650	326.6	10640
			5651	325.5	7910	5651	326.6	10640
			5652	325.4	7910	5652	326.5	10630
			5653	325.4	7910	5653	326.6	10620
			5654	325.4	7910	5654	326.7	10630
			5655	325.4	7900	5655	326.7	10640
			5656	325.4	7910	5656	326.8	10630
			5657	325.5	7910	5657	326.9	10630
			5658	325.5	7900	5658	326.9	10640
			5659	325.6	7920	5659	326.9	10640
			5660	325.6	7910	5660	327	10630
			5661	325.6	7910	5661	327	10640
			5662	325.7	7910	5662	327.2	10630
			5663	325.6	7900	5663	327.2	10630
			5664	325.6	7900	5664	327.2	10620
			5665	325.6	7920	5665	327.2	10630
			5666	325.6	7900	5666	327.2	10630
			5667	325.7	7910	5667	327.2	10630
			5668	325.6	7900	5668	327.3	10630
			5669	325.6	7900	5669	327.4	10630
			5670	325.6	7920	5670	327.4	10630
			5671	325.6	7910	5671	327.5	10620
			5672	325.7	7910	5672	327.5	10630
			5673	325.7	7900	5673	327.6	10630
			5674	325.7	7910	5674	327.7	10630
			5675	325.7	7900	5675	327.8	10630
			5676	325.6	7910	5676	327.8	10620
			5677	325.6	7910	5677	327.9	10630
			5678	325.6	7910	5678	327.9	10630
			5679	325.6	7890	5679	328	10640
			5680	325.5	7900	5680	328	10630
			5681	325.6	7890	5681	328.1	10630
			5682	325.6	7910	5682	328.1	10630
			5683	325.6	7910	5683	328.1	10620
			5684	325.6	7910	5684	328.2	10610
			5685	325.6	7910	5685	328.2	10620
			5686	325.7	7920	5686	328.3	10630
			5687	325.7	7920	5687	328.3	10630
			5688	325.7	7910	5688	328.3	10630
			5689	325.7	7910	5689	328.4	10630
			5690	325.6	7910	5690	328.5	10620
			5691	325.6	7910	5691	328.5	10620
			5692	325.6	7930	5692	328.6	10620
			5693	325.7	7930	5693	328.6	10620
			5694	325.6	7910	5694	328.6	10630
			5695	325.7	7910	5695	328.7	10630
			5696	325.6	7910	5696	328.7	10620

			5697	325.6	7920	5697	328.8	10620
			5698	325.6	7900	5698	328.8	10630
			5699	325.7	7910	5699	328.9	10620
			5700	325.6	7920	5700	328.9	10630
			5701	325.6	7920	5701	329	10610
			5702	325.7	7910	5702	329	10630
			5703	325.6	7910	5703	329	10630
			5704	325.7	7910	5704	329.1	10620
			5705	325.7	7920	5705	329.1	10630
			5706	325.7	7910	5706	329.2	10630
			5707	325.8	7920	5707	329.2	10630
			5708	325.8	7920	5708	329.2	10620
			5709	325.7	7920	5709	329.3	10620
			5710	325.7	7910	5710	329.2	10630
			5711	325.8	7920	5711	329.3	10640
			5712	325.7	7920	5712	329.2	10630
			5713	325.7	7910	5713	329.1	10620
			5714	325.8	7910	5714	328.9	10630
			5715	325.8	7910	5715	328.9	10630
			5716	325.8	7910	5716	328.8	10640
			5717	325.8	7910	5717	328.7	10630
			5718	325.8	7910	5718	328.6	10630
			5719	325.8	7920	5719	328.5	10630
			5720	325.8	7920	5720	328.4	10630
			5721	325.8	7910	5721	328.4	10630
			5722	325.7	7910	5722	328.4	10640
			5723	325.8	7910	5723	328.3	10640
			5724	325.8	7910	5724	328.3	10630
			5725	325.8	7910	5725	328.3	10630
			5726	325.8	7910	5726	328.2	10620
			5727	325.8	7910	5727	328.2	10630
			5728	325.8	7920	5728	328.2	10630
			5729	325.8	7910	5729	328.2	10630
			5730	325.9	7910	5730	328.2	10630
			5731	325.9	7900	5731	328.2	10630
			5732	325.9	7900	5732	328.2	10630
			5733	325.9	7910	5733	328.2	10620
			5734	326	7900	5734	328.2	10630
			5735	326	7920	5735	328.2	10630
			5736	326	7920	5736	328.2	10620
			5737	326	7920	5737	328.2	10630
			5738	326	7910	5738	328.1	10620
			5739	326	7920	5739	328.2	10610
			5740	326.1	7910	5740	328.2	10620
			5741	326.1	7910	5741	328.2	10640
			5742	326.1	7900	5742	328.2	10630
			5743	326.1	7910	5743	328.3	10630
			5744	326.2	7900	5744	328.2	10630
			5745	326.2	7910	5745	328.2	10630
			5746	326.2	7920	5746	328.2	10630

			5747	326.2	7910	5747	328.3	10630
			5748	326.2	7920	5748	328.3	10620
			5749	326.2	7920	5749	328.3	10630
			5750	326.1	7910	5750	328.3	10620
			5751	326.1	7910	5751	328.3	10620
			5752	326.2	7900	5752	328.3	10620
			5753	326.2	7910	5753	328.3	10620
			5754	326.2	7920	5754	328.3	10620
			5755	326.2	7920	5755	328.3	10620
			5756	326.2	7910	5756	328.3	10610
			5757	326.3	7900	5757	328.3	10630
			5758	326.3	7920	5758	328.3	10620
			5759	326.3	7910	5759	328.3	10610
			5760	326.3	7910	5760	328.3	10520
			5761	326.4	7920	5761	328.3	10630
			5762	326.4	7920	5762	328.3	10620
			5763	326.3	7910	5763	328.3	10610
			5764	326.3	7910	5764	328.3	10640
			5765	326.3	7920	5765	328.3	10630
			5766	326.3	7910	5766	328.3	10520
			5767	326.3	7910	5767	328.3	10630
			5768	326.3	7920	5768	328.3	10640
			5769	326.3	7920	5769	328.3	10630
			5770	326.3	7910	5770	328.4	10620
			5771	326.3	7910	5771	328.4	10620
			5772	326.3	7910	5772	328.4	10620
			5773	326.3	7920	5773	328.4	10520
			5774	326.2	7910	5774	328.4	10630
			5775	326.2	7910	5775	328.4	10620
			5776	326.2	7910	5776	328.4	10620
			5777	326.2	7910	5777	328.4	10620
			5778	326.3	7900	5778	328.4	10520
			5779	326.3	7920	5779	328.4	10630
			5780	326.3	7910	5780	328.4	10530
			5781	326.3	7920	5781	328.4	10610
			5782	326.3	7920	5782	328.4	10630
			5783	326.4	7910	5783	328.4	10620
			5784	326.4	7920	5784	328.4	10620
			5785	326.3	7930	5785	328.5	10620
			5786	326.4	7920	5786	328.5	10520
			5787	326.4	7910	5787	328.6	10530
			5788	326.4	7910	5788	328.6	10620
			5789	326.4	7920	5789	328.5	10620
			5790	326.5	7910	5790	328.6	10620
			5791	326.5	7910	5791	328.7	10640
			5792	326.5	7910	5792	328.7	10520
			5793	326.5	7910	5793	328.7	10630
			5794	326.5	7910	5794	328.7	10630
			5795	326.5	7920	5795	328.7	10620
			5796	326.6	7920	5796	328.7	10610

			5797	326.6	7930	5797	328.7	10610
			5798	326.6	7910	5798	328.7	10520
			5799	326.6	7920	5799	328.7	10620
			5800	326.6	7910	5800	328.6	10530
			5801	326.6	7920	5801	328.7	10520
			5802	326.6	7920	5802	328.6	10520
			5803	326.6	7930	5803	328.6	10510
			5804	326.6	7910	5804	328.7	10520
			5805	326.6	7910	5805	328.7	10530
			5806	326.7	7920	5806	328.7	10530
			5807	326.7	7910	5807	328.7	10520
			5808	326.7	7910	5808	328.7	10520
			5809	326.7	7910	5809	328.7	10520
			5810	326.7	7910	5810	328.8	10530
			5811	326.7	7920	5811	328.7	10520
			5812	326.7	7910	5812	328.8	10630
			5813	326.7	7910	5813	328.8	10520
			5814	326.7	7910	5814	328.8	10630
			5815	326.7	7910	5815	328.9	10540
			5816	326.7	7910	5816	328.9	10520
			5817	326.7	7910	5817	328.9	10520
			5818	326.7	7910	5818	328.9	10530
			5819	326.7	7900	5819	329	10530
			5820	326.7	7920	5820	329	10520
			5821	326.8	7910	5821	328.9	10510
			5822	326.8	7910	5822	328.9	10520
			5823	326.8	7910	5823	329	10520
			5824	326.7	7910	5824	329	10520
			5825	326.7	7910	5825	328.9	10520
			5826	326.7	7910	5826	328.9	10540
			5827	326.7	7920	5827	328.8	10530
			5828	326.7	7920	5828	328.7	10520
			5829	326.8	7910	5829	328.6	10520
			5830	326.8	7910	5830	328.5	10530
			5831	326.8	7920	5831	328.5	10540
			5832	326.8	7920	5832	328.4	10530
			5833	326.8	7910	5833	328.4	10530
			5834	326.9	7920	5834	328.3	10520
			5835	326.9	7920	5835	328.3	10520
			5836	327	7910	5836	328.3	10540
			5837	327	7920	5837	328.2	10530
			5838	327	7920	5838	328.1	10520
			5839	327	7920	5839	328.1	10530
			5840	327.1	7910	5840	328.1	10540
			5841	327.1	7910	5841	328.1	10530
			5842	327.1	7900	5842	328	10530
			5843	327.1	7920	5843	328.1	10540
			5844	327	7910	5844	328	10520
			5845	327	7920	5845	328.1	10530
			5846	327	7930	5846	328.1	10530

			5847	327	7920	5847	328	10530
			5848	327	7920	5848	328	10540
			5849	327	7920	5849	328.1	10530
			5850	327	7920	5850	328.1	10530
			5851	327.1	7920	5851	328.1	10530
			5852	327	7920	5852	328	10520
			5853	327	7910	5853	328	10510
			5854	327.1	7910	5854	328	10530
			5855	327.1	7920	5855	328	10520
			5856	327.1	7930	5856	328	10520
			5857	327.1	7940	5857	327.9	10530
			5858	327.1	7920	5858	328	10520
			5859	327.1	7910	5859	328	10520
			5860	327.1	7910	5860	328	10520
			5861	327.1	7910	5861	328	10520
			5862	327.1	7910	5862	328	10520
			5863	327.1	7900	5863	328	10520
			5864	327.1	7910	5864	328	10520
			5865	327.1	7920	5865	328.1	10520
			5866	327.1	7920	5866	328	10530
			5867	327.1	7900	5867	328	10540
			5868	327.1	7910	5868	328	10540
			5869	327.2	7910	5869	328	10530
			5870	327.2	7910	5870	328	10540
			5871	327.2	7910	5871	328	10520
			5872	327.2	7920	5872	328.1	10530
			5873	327.2	7920	5873	328.1	10510
			5874	327.2	7910	5874	328.1	10520
			5875	327.2	7920	5875	328	10530
			5876	327.2	7920	5876	328	10530
			5877	327.3	7910	5877	328.1	10530
			5878	327.3	7910	5878	328.1	10520
			5879	327.2	7910	5879	328	10540
			5880	327.2	7920	5880	328	10540
			5881	327.2	7910	5881	328	10520
			5882	327.2	7910	5882	327.9	10530
			5883	327.2	7910	5883	327.9	10540
			5884	327.2	7910	5884	328	10540
			5885	327.2	7910	5885	327.9	10530
			5886	327.3	7900	5886	327.9	10530
			5887	327.3	7900	5887	327.8	10530
			5888	327.4	7910	5888	327.9	10540
			5889	327.4	7920	5889	327.9	10540
			5890	327.4	7910	5890	327.9	10530
			5891	327.4	7930	5891	327.9	10520
			5892	327.4	7920	5892	327.9	10540
			5893	327.4	7920	5893	328	10540
			5894	327.4	7920	5894	328	10530
			5895	327.4	7910	5895	328	10510
			5896	327.4	7920	5896	328.1	10530

			5897	327.4	7920	5897	328	10540
			5898	327.3	7930	5898	328	10520
			5899	327.3	7920	5899	328	10510
			5900	327.3	7910	5900	327.9	10520
			5901	327.4	7910	5901	327.9	10530
			5902	327.3	7910	5902	327.9	10540
			5903	327.3	7910	5903	327.9	10540
			5904	327.3	7920	5904	327.9	10540
			5905	327.3	7920	5905	327.9	10530
			5906	327.3	7910	5906	328	10540
			5907	327.4	7930	5907	328.1	10520
			5908	327.4	7910	5908	328.1	10530
			5909	327.4	7920	5909	328	10520
			5910	327.4	7910	5910	328	10540
			5911	327.4	7920	5911	328	10530
			5912	327.4	7920	5912	328	10530
			5913	327.4	7910	5913	328	10530
			5914	327.4	7920	5914	328.1	10540
			5915	327.4	7910	5915	328	10540
			5916	327.4	7920	5916	328	10540
			5917	327.4	7920	5917	328	10540
			5918	327.4	7930	5918	328	10540
			5919	327.4	7910	5919	328	10540
			5920	327.3	7920	5920	328	10540
			5921	327.3	7910	5921	328.1	10540
			5922	327.3	7920	5922	328.1	10540
			5923	327.4	7920	5923	328.1	10550
			5924	327.4	7920	5924	328.1	10540
			5925	327.4	7920	5925	328.1	10530
			5926	327.4	7920	5926	328.1	10540
			5927	327.5	7920	5927	328	10520
			5928	327.5	7910	5928	328.1	10540
			5929	327.5	7930	5929	328.1	10540
			5930	327.5	7920	5930	328.1	10540
			5931	327.5	7910	5931	328	10540
			5932	327.5	7920	5932	328.1	10530
			5933	327.5	7910	5933	328	10530
			5934	327.5	7910	5934	328	10530
			5935	327.5	7920	5935	328	10540
			5936	327.6	7920	5936	328	10530
			5937	327.6	7910	5937	328.1	10540
			5938	327.6	7910	5938	328	10530
			5939	327.5	7920	5939	328	10530
			5940	327.6	7920	5940	328.1	10530
			5941	327.7	7920	5941	328.1	10530
			5942	327.7	7920	5942	328.1	10520
			5943	327.7	7930	5943	328	10530
			5944	327.7	7920	5944	328.1	10520
			5945	327.8	7910	5945	328.1	10520
			5946	327.7	7910	5946	328.1	10530

			5947	327.7	7910	5947	328.1	10530
			5948	327.7	7920	5948	328.1	10530
			5949	327.7	7920	5949	328.1	10520
			5950	327.7	7920	5950	328.1	10530
			5951	327.6	7930	5951	328.1	10540
			5952	327.6	7930	5952	328.1	10540
			5953	327.7	7910	5953	328.1	10540
			5954	327.6	7920	5954	328.1	10540
			5955	327.6	7920	5955	328.1	10530
			5956	327.6	7930	5956	328.1	10540
			5957	327.6	7920	5957	328.2	10540
			5958	327.6	7920	5958	328.2	10530
			5959	327.5	7920	5959	328.2	10530
			5960	327.6	7910	5960	328.2	10530
			5961	327.6	7910	5961	328.2	10550
			5962	327.6	7930	5962	328.3	10530
			5963	327.7	7930	5963	328.3	10540
			5964	327.7	7930	5964	328.3	10540
			5965	327.7	7900	5965	328.3	10520
			5966	327.7	7910	5966	328.3	10530
			5967	327.7	7910	5967	328.2	10520
			5968	327.7	7910	5968	328.2	10540
			5969	327.6	7910	5969	328.1	10530
			5970	327.6	7910	5970	328.1	10520
			5971	327.6	7930	5971	328.2	10530
			5972	327.5	7920	5972	328.2	10520
			5973	327.5	7910	5973	328.2	10530
			5974	327.5	7920	5974	328.3	10530
			5975	327.6	7910	5975	328.2	10530
			5976	327.6	7920	5976	328.3	10530
			5977	327.6	7920	5977	328.3	10520
			5978	327.7	7920	5978	328.3	10530
			5979	327.6	7910	5979	328.3	10520
			5980	327.6	7910	5980	328.3	10520
			5981	327.6	7920	5981	328.3	10530
			5982	327.6	7920	5982	328.3	10520
			5983	327.6	7910	5983	328.3	10530
			5984	327.6	7910	5984	328.2	10520
			5985	327.6	7920	5985	328.2	10540
			5986	327.6	7900	5986	328.2	10520
			5987	327.6	7910	5987	328.2	10520
			5988	327.6	7910	5988	328.2	10530
			5989	327.6	7910	5989	328.2	10520
			5990	327.6	7900	5990	328.2	10530
			5991	327.6	7920	5991	328.2	10530
			5992	327.6	7900	5992	328.2	10530
			5993	327.6	7910	5993	328.2	10530
			5994	327.6	7910	5994	328.2	10530
			5995	327.6	7910	5995	328.2	10530
			5996	327.6	7910	5996	328.2	10530

			5997	327.7	7910	5997	328.2	10520
			5998	327.7	7920	5998	328.2	10520
			5999	327.6	7910	5999	328.3	10420
			6000	327.6	7920	6000	328.2	10530
			6001	327.6	7920	6001	328.2	10520
			6002	327.6	7900	6002	328.2	10530
			6003	327.6	7910	6003	328.2	10520
			6004	327.6	7910	6004	328.2	10520
			6005	327.5	7920	6005	328	10530
			6006	327.6	7920	6006	328.1	10420
			6007	327.6	7910	6007	328.1	10520
			6008	327.7	7920	6008	328.1	10440
			6009	327.6	7920	6009	328.1	10530
			6010	327.6	7920	6010	328.1	10430
			6011	327.7	7910	6011	328.2	10430
			6012	327.6	7900	6012	328.2	10520
			6013	327.6	7920	6013	328.2	10520
			6014	327.7	7910	6014	328.2	10540
			6015	327.6	7910	6015	328.2	10530
			6016	327.6	7910	6016	328.3	10530
			6017	327.5	7910	6017	328.3	10530
			6018	327.5	7910	6018	328.3	10430
			6019	327.5	7910	6019	328.3	10530
			6020	327.5	7920	6020	328.3	10520
			6021	327.5	7920	6021	328.3	10530
			6022	327.5	7920	6022	328.3	10420
			6023	327.5	7910	6023	328.3	10520
			6024	327.5	7910	6024	328.3	10430
			6025	327.5	7920	6025	328.2	10430
			6026	327.5	7910	6026	328.2	10430
			6027	327.5	7910	6027	328.2	10430
			6028	327.5	7910	6028	328.1	10410
			6029	327.5	7910	6029	328.2	10420
			6030	327.5	7910	6030	328.2	10510
			6031	327.5	7910	6031	328.2	10520
			6032	327.5	7920	6032	328.2	10520
			6033	327.5	7910	6033	328.2	10520
			6034	327.5	7920	6034	328.2	10520
			6035	327.5	7910	6035	328.3	10530
			6036	327.5	7910	6036	328.3	10430
			6037	327.4	7910	6037	328.3	10420
			6038	327.4	7920	6038	328.3	10430
			6039	327.4	7920	6039	328.3	10440
			6040	327.4	7920	6040	328.3	10520
			6041	327.5	7920	6041	328.3	10520
			6042	327.5	7920	6042	328.4	10530
			6043	327.5	7910	6043	328.4	10530
			6044	327.5	7910	6044	328.4	10530
			6045	327.5	7920	6045	328.4	10430
			6046	327.5	7930	6046	328.3	10420

			6047	327.4	7920	6047	328.4	10530
			6048	327.4	7920	6048	328.4	10510
			6049	327.4	7910	6049	328.4	10420
			6050	327.4	7920	6050	328.4	10430
			6051	327.4	7920	6051	328.4	10520
			6052	327.4	7910	6052	328.3	10420
			6053	327.4	7920	6053	328.3	10420
			6054	327.4	7910	6054	328.4	10430
			6055	327.4	7920	6055	328.4	10420
			6056	327.4	7920	6056	328.4	10430
			6057	327.3	7920	6057	328.4	10420
			6058	327.4	7930	6058	328.4	10420
			6059	327.4	7920	6059	328.4	10430
			6060	327.4	7910	6060	328.4	10530
			6061	327.4	7910	6061	328.4	10420
			6062	327.4	7910	6062	328.4	10440
			6063	327.4	7920	6063	328.4	10440
			6064	327.4	7920	6064	328.4	10440
			6065	327.4	7910	6065	328.4	10440
			6066	327.4	7910	6066	328.4	10530
			6067	327.5	7910	6067	328.4	10520
			6068	327.4	7900	6068	328.4	10540
			6069	327.4	7910	6069	328.5	10520
			6070	327.4	7930	6070	328.5	10520
			6071	327.4	7930	6071	328.5	10430
			6072	327.4	7920	6072	328.5	10420
			6073	327.5	7910	6073	328.5	10520
			6074	327.5	7920	6074	328.5	10450
			6075	327.4	7910	6075	328.5	10430
			6076	327.4	7910	6076	328.5	10420
			6077	327.4	7920	6077	328.5	10410
			6078	327.5	7920	6078	328.5	10420
			6079	327.5	7920	6079	328.5	10430
			6080	327.5	7920	6080	328.5	10440
			6081	327.5	7910	6081	328.5	10420
			6082	327.5	7930	6082	328.6	10420
			6083	327.6	7920	6083	328.6	10420
			6084	327.6	7930	6084	328.6	10430
			6085	327.6	7910	6085	328.6	10420
			6086	327.6	7910	6086	328.6	10430
			6087	327.6	7910	6087	328.6	10430
			6088	327.6	7910	6088	328.5	10420
			6089	327.6	7910	6089	328.6	10420
			6090	327.6	7920	6090	328.6	10420
			6091	327.6	7920	6091	328.6	10440
			6092	327.6	7900	6092	328.6	10440
			6093	327.7	7910	6093	328.6	10420
			6094	327.6	7920	6094	328.6	10430
			6095	327.6	7910	6095	328.6	10420
			6096	327.6	7920	6096	328.5	10420

			6097	327.6	7930	6097	328.5	10420
			6098	327.6	7910	6098	328.5	10430
			6099	327.6	7910	6099	328.5	10430
			6100	327.6	7920	6100	328.5	10440
			6101	327.7	7930	6101	328.5	10440
			6102	327.7	7930	6102	328.5	10530
			6103	327.6	7920	6103	328.5	10520
			6104	327.6	7920	6104	328.4	10430
			6105	327.6	7920	6105	328.4	10430
			6106	327.6	7920	6106	328.4	10420
			6107	327.6	7910	6107	328.4	10440
			6108	327.6	7920	6108	328.4	10430
			6109	327.6	7910	6109	328.4	10430
			6110	327.6	7900	6110	328.4	10430
			6111	327.6	7910	6111	328.5	10430
			6112	327.6	7920	6112	328.5	10430
			6113	327.6	7920	6113	328.5	10420
			6114	327.6	7920	6114	328.5	10420
			6115	327.6	7920	6115	328.5	10430
			6116	327.6	7910	6116	328.4	10440
			6117	327.6	7910	6117	328.5	10530
			6118	327.6	7800	6118	328.5	10430
			6119	327.6	7910	6119	328.5	10440
			6120	327.6	7910	6120	328.6	10430
			6121	327.6	7910	6121	328.6	10440
			6122	327.6	7930	6122	328.6	10430
			6123	327.6	7920	6123	328.6	10440
			6124	327.6	7920	6124	328.6	10430
			6125	327.6	7910	6125	328.6	10430
			6126	327.6	7900	6126	328.6	10430
			6127	327.6	7920	6127	328.6	10440
			6128	327.6	7920	6128	328.6	10430
			6129	327.6	7910	6129	328.6	10440
			6130	327.6	7910	6130	328.6	10430
			6131	327.6	7910	6131	328.6	10420
			6132	327.6	7910	6132	328.6	10420
			6133	327.6	7910	6133	328.6	10430
			6134	327.6	7910	6134	328.6	10440
			6135	327.6	7920	6135	328.6	10430
			6136	327.6	7910	6136	328.6	10440
			6137	327.6	7920	6137	328.6	10440
			6138	327.6	7910	6138	328.6	10430
			6139	327.7	7900	6139	328.6	10430
			6140	327.7	7900	6140	328.7	10430
			6141	327.7	7920	6141	328.6	10430
			6142	327.7	7900	6142	328.6	10440
			6143	327.8	7920	6143	328.6	10430
			6144	327.8	7900	6144	328.7	10440
			6145	327.8	7910	6145	328.7	10420
			6146	327.7	7940	6146	328.7	10430

			6147	327.7	7930	6147	328.6	10430
			6148	327.7	7820	6148	328.6	10430
			6149	327.7	7920	6149	328.5	10430
			6150	327.7	7920	6150	328.5	10440
			6151	327.7	7920	6151	328.5	10430
			6152	327.7	7810	6152	328.5	10440
			6153	327.7	7920	6153	328.5	10430
			6154	327.7	7910	6154	328.4	10420
			6155	327.7	7910	6155	328.4	10430
			6156	327.7	7920	6156	328.4	10430
			6157	327.7	7920	6157	328.4	10420
			6158	327.8	7910	6158	328.4	10430
			6159	327.8	7910	6159	328.4	10430
			6160	327.8	7910	6160	328.4	10410
			6161	327.8	7920	6161	328.4	10420
			6162	327.8	7910	6162	328.3	10440
			6163	327.8	7910	6163	328.3	10430
			6164	327.8	7900	6164	328.3	10430
			6165	327.8	7890	6165	328.4	10430
			6166	327.8	7900	6166	328.4	10430
			6167	327.8	7890	6167	328.5	10430
			6168	327.8	7910	6168	328.5	10430
			6169	327.8	7900	6169	328.5	10440
			6170	327.7	7920	6170	328.5	10430
			6171	327.8	7920	6171	328.6	10430
			6172	327.7	7910	6172	328.6	10430
			6173	327.7	7810	6173	328.6	10440
			6174	327.8	7820	6174	328.6	10430
			6175	327.9	7900	6175	328.6	10430
			6176	327.9	7910	6176	328.6	10420
			6177	327.9	7800	6177	328.6	10430
			6178	327.8	7810	6178	328.6	10440
			6179	327.8	7900	6179	328.6	10440
			6180	327.8	7920	6180	328.6	10420
			6181	327.8	7910	6181	328.6	10440
			6182	327.9	7910	6182	328.6	10430
			6183	327.9	7810	6183	328.6	10440
			6184	327.9	7920	6184	328.5	10430
			6185	327.8	7920	6185	328.5	10420
			6186	327.8	7810	6186	328.5	10430
			6187	327.9	7910	6187	328.5	10440
			6188	327.9	7820	6188	328.5	10430
			6189	327.9	7810	6189	328.5	10430
			6190	327.8	7820	6190	328.5	10440
			6191	327.8	7800	6191	328.5	10440
			6192	327.8	7920	6192	328.5	10440
			6193	327.8	7920	6193	328.6	10450
			6194	327.8	7920	6194	328.5	10440
			6195	327.8	7920	6195	328.5	10440
			6196	327.9	7930	6196	328.5	10440

			6197	328	7920	6197	328.5	10430
			6198	328	7910	6198	328.6	10420
			6199	328	7910	6199	328.6	10440
			6200	328	7910	6200	328.5	10440
			6201	328	7910	6201	328.5	10430
			6202	327.9	7900	6202	328.5	10420
			6203	327.9	7920	6203	328.5	10440
			6204	327.9	7810	6204	328.5	10430
			6205	327.9	7910	6205	328.5	10420
			6206	328	7810	6206	328.5	10430
			6207	328	7820	6207	328.5	10420
			6208	328	7920	6208	328.5	10420
			6209	328.1	7910	6209	328.5	10430
			6210	328.2	7920	6210	328.6	10430
			6211	328.2	7920	6211	328.5	10430
			6212	328.2	7820	6212	328.5	10430
			6213	328.2	7920	6213	328.5	10430
			6214	328.2	7810	6214	328.5	10430
			6215	328.2	7910	6215	328.5	10430
			6216	328.2	7910	6216	328.5	10420
			6217	328.1	7800	6217	328.5	10440
			6218	328.1	7910	6218	328.5	10420
			6219	328.1	7920	6219	328.5	10420
			6220	328.1	7810	6220	328.5	10420
			6221	328	7810	6221	328.5	10420
			6222	328	7810	6222	328.5	10420
			6223	328	7810	6223	328.5	10420
			6224	328	7920	6224	328.5	10430
			6225	328	7810	6225	328.5	10430
			6226	328	7820	6226	328.5	10420
			6227	328	7820	6227	328.5	10430
			6228	328	7800	6228	328.5	10440
			6229	328	7810	6229	328.4	10440
			6230	328	7830	6230	328.4	10430
			6231	328.1	7810	6231	328.4	10430
			6232	328.1	7810	6232	328.3	10440
			6233	328.1	7810	6233	328.3	10430
			6234	328.1	7810	6234	328.4	10420
			6235	328.1	7820	6235	328.4	10420
			6236	328.1	7810	6236	328.4	10430
			6237	328.1	7900	6237	328.4	10430
			6238	328.1	7810	6238	328.4	10440
			6239	328	7810	6239	328.3	10440
			6240	328	7810	6240	328.3	10440
			6241	328	7820	6241	328.3	10440
			6242	328	7810	6242	328.3	10430
			6243	328	7920	6243	328.3	10440
			6244	327.9	7920	6244	328.3	10430
			6245	328	7810	6245	328.3	10430
			6246	328	7820	6246	328.3	10430

			6247	328	7920	6247	328.4	10440
			6248	327.9	7930	6248	328.4	10430
			6249	328	7910	6249	328.3	10430
			6250	328	7810	6250	328.3	10450
			6251	327.9	7910	6251	328.3	10440
			6252	327.9	7820	6252	328.4	10420
			6253	327.9	7820	6253	328.3	10430
			6254	327.9	7820	6254	328.4	10430
			6255	327.9	7820	6255	328.4	10430
			6256	328	7820	6256	328.4	10430
			6257	328	7820	6257	328.4	10440
			6258	328	7810	6258	328.4	10430
			6259	328	7810	6259	328.4	10430
			6260	328	7810	6260	328.4	10430
			6261	328.1	7920	6261	328.4	10420
			6262	328.1	7920	6262	328.4	10430
			6263	328.2	7810	6263	328.4	10430
			6264	328.2	7910	6264	328.4	10430
			6265	328.2	7900	6265	328.4	10430
			6266	328.2	7910	6266	328.4	10430
			6267	328.3	7910	6267	328.4	10430
			6268	328.3	7820	6268	328.4	10430
			6269	328.3	7810	6269	328.4	10430
			6270	328.3	7800	6270	328.4	10430
			6271	328.3	7810	6271	328.4	10430
			6272	328.2	7910	6272	328.4	10430
			6273	328.3	7800	6273	328.4	10440
			6274	328.3	7820	6274	328.4	10430
			6275	328.3	7810	6275	328.5	10430
			6276	328.3	7810	6276	328.4	10420
			6277	328.3	7910	6277	328.5	10420
			6278	328.3	7910	6278	328.5	10430
			6279	328.3	7800	6279	328.5	10430
			6280	328.3	7810	6280	328.5	10430
			6281	328.3	7810	6281	328.5	10430
			6282	328.3	7810	6282	328.5	10440
			6283	328.3	7810	6283	328.5	10430
			6284	328.3	7820	6284	328.5	10440
			6285	328.3	7900	6285	328.4	10430
			6286	328.3	7810	6286	328.5	10430
			6287	328.3	7810	6287	328.5	10440
			6288	328.2	7810	6288	328.4	10430
			6289	328.3	7820	6289	328.4	10440
			6290	328.3	7810	6290	328.4	10430
			6291	328.3	7810	6291	328.4	10430
			6292	328.2	7810	6292	328.4	10440
			6293	328.2	7810	6293	328.5	10440
			6294	328.2	7900	6294	328.5	10430
			6295	328.2	7810	6295	328.5	10430
			6296	328.2	7920	6296	328.5	10430

			6297	328.2	7810	6297	328.5	10420
			6298	328.1	7900	6298	328.5	10420
			6299	328.1	7820	6299	328.5	10420
			6300	328.1	7820	6300	328.5	10430
			6301	328.1	7810	6301	328.4	10430
			6302	328.1	7810	6302	328.4	10420
			6303	328	7800	6303	328.4	10410
			6304	328	7810	6304	328.4	10420
			6305	328	7920	6305	328.4	10420
			6306	328.1	7800	6306	328.4	10430
			6307	328.1	7910	6307	328.4	10420
			6308	328	7810	6308	328.4	10430
			6309	328	7810	6309	328.4	10420
			6310	328	7800	6310	328.5	10420
			6311	328	7810	6311	328.4	10430
			6312	328.1	7820	6312	328.4	10410
			6313	328.1	7820	6313	328.4	10420
			6314	328.1	7820	6314	328.4	10420
			6315	328.1	7810	6315	328.4	10420
			6316	328.1	7810	6316	328.4	10420
			6317	328.1	7820	6317	328.4	10430
			6318	328.1	7820	6318	328.4	10430
			6319	328.1	7810	6319	328.5	10420
			6320	328.1	7820	6320	328.5	10430
			6321	328.2	7800	6321	328.5	10420
			6322	328.2	7810	6322	328.4	10430
			6323	328.2	7810	6323	328.5	10430
			6324	328.2	7810	6324	328.5	10430
			6325	328.2	7810	6325	328.5	10430
			6326	328.3	7810	6326	328.5	10430
			6327	328.3	7800	6327	328.5	10420
			6328	328.2	7800	6328	328.5	10420
			6329	328.2	7800	6329	328.5	10420
			6330	328.3	7800	6330	328.5	10430
			6331	328.2	7810	6331	328.5	10430
			6332	328.3	7800	6332	328.6	10420
			6333	328.3	7800	6333	328.6	10430
			6334	328.2	7800	6334	328.6	10420
			6335	328.3	7810	6335	328.6	10430
			6336	328.3	7810	6336	328.6	10410
			6337	328.3	7800	6337	328.6	10430
			6338	328.3	7810	6338	328.6	10420
			6339	328.2	7810	6339	328.6	10430
			6340	328.2	7810	6340	328.5	10420
			6341	328.2	7800	6341	328.5	10430
			6342	328.2	7800	6342	328.6	10430
			6343	328.2	7800	6343	328.6	10430
			6344	328.2	7800	6344	328.6	10420
			6345	328.1	7800	6345	328.6	10440
			6346	328.1	7810	6346	328.6	10440

			6347	328.1	7810	6347	328.6	10430
			6348	328.1	7820	6348	328.6	10430
			6349	328	7810	6349	328.6	10420
			6350	328	7810	6350	328.6	10410
			6351	328	7810	6351	328.6	10420
			6352	327.9	7810	6352	328.6	10430
			6353	328	7800	6353	328.6	10430
			6354	328	7800	6354	328.6	10440
			6355	327.9	7810	6355	328.6	10430
			6356	327.9	7810	6356	328.6	10430
			6357	328	7810	6357	328.6	10420
			6358	328	7810	6358	328.6	10440
			6359	328	7800	6359	328.6	10440
			6360	328	7810	6360	328.6	10440
			6361	328	7810	6361	328.6	10420
			6362	328	7810	6362	328.6	10410
			6363	327.9	7810	6363	328.5	10420
			6364	328	7800	6364	328.5	10410
			6365	328	7800	6365	328.5	10430
			6366	328	7810	6366	328.6	10420
			6367	327.9	7810	6367	328.5	10420
			6368	328	7800	6368	328.6	10420
			6369	328	7810	6369	328.6	10430
			6370	328	7800	6370	328.7	10420
			6371	328	7800	6371	328.6	10430
			6372	328	7810	6372	328.6	10420
			6373	327.9	7810	6373	328.6	10420
			6374	328	7820	6374	328.6	10430
			6375	328	7810	6375	328.6	10430
			6376	328	7800	6376	328.6	10430
			6377	328	7800	6377	328.6	10430
			6378	328	7800	6378	328.6	10430
			6379	328.1	7810	6379	328.6	10430
			6380	328	7800	6380	328.6	10440
			6381	328	7800	6381	328.6	10440
			6382	328	7790	6382	328.6	10440
			6383	328	7810	6383	328.6	10420
			6384	328.1	7810	6384	328.6	10430
			6385	328	7810	6385	328.6	10430
			6386	328	7810	6386	328.6	10420
			6387	328	7820	6387	328.6	10430
			6388	328	7830	6388	328.6	10430
			6389	328	7810	6389	328.6	10430
			6390	328	7810	6390	328.6	10440
			6391	328	7810	6391	328.5	10430
			6392	327.9	7810	6392	328.5	10430
			6393	327.9	7810	6393	328.5	10440
			6394	328	7800	6394	328.6	10430
			6395	328	7810	6395	328.6	10430
			6396	328	7820	6396	328.6	10440

			6397	328	7810	6397	328.6	10420
			6398	328	7820	6398	328.6	10430
			6399	327.9	7810	6399	328.7	10430
			6400	327.9	7810	6400	328.7	10430
			6401	327.9	7810	6401	328.8	10420
			6402	328	7810	6402	328.7	10430
			6403	328	7800	6403	328.7	10440
			6404	327.9	7800	6404	328.7	10430
			6405	327.9	7810	6405	328.7	10430
			6406	327.9	7800	6406	328.7	10430
			6407	328	7810	6407	328.7	10440
			6408	328	7800	6408	328.7	10430
			6409	328.1	7810	6409	328.7	10440
			6410	328	7800	6410	328.7	10430
			6411	328	7810	6411	328.7	10430
			6412	328	7810	6412	328.7	10430
			6413	328	7810	6413	328.7	10440
			6414	328	7800	6414	328.8	10430
			6415	328.1	7810	6415	328.8	10440
			6416	328.1	7810	6416	328.8	10430
			6417	328	7800	6417	328.7	10430
			6418	328	7810	6418	328.8	10420
			6419	328	7800	6419	328.8	10430
			6420	328	7810	6420	328.8	10420
			6421	328	7800	6421	328.8	10430
			6422	328	7810	6422	328.7	10430
			6423	328	7820	6423	328.6	10430
			6424	328.1	7810	6424	328.6	10430
			6425	328.1	7810	6425	328.6	10430
			6426	328	7820	6426	328.7	10430
			6427	328	7810	6427	328.7	10430
			6428	328	7800	6428	328.6	10430
			6429	328	7800	6429	328.6	10430
			6430	328	7810	6430	328.6	10420
			6431	327.9	7810	6431	328.6	10440
			6432	328	7790	6432	328.6	10430
			6433	327.9	7800	6433	328.6	10430
			6434	327.9	7810	6434	328.5	10420
			6435	327.9	7800	6435	328.6	10420
			6436	327.9	7800	6436	328.6	10420
			6437	327.9	7800	6437	328.6	10430
			6438	327.9	7810	6438	328.7	10440
			6439	327.9	7790	6439	328.6	10430
			6440	327.9	7810	6440	328.6	10430
			6441	327.9	7810	6441	328.6	10430
			6442	327.9	7810	6442	328.6	10430
			6443	327.9	7800	6443	328.6	10420
			6444	327.9	7810	6444	328.7	10430
			6445	327.9	7800	6445	328.7	10430
			6446	327.9	7800	6446	328.7	10420

			6447	327.9	7810	6447	328.7	10420
			6448	328	7790	6448	328.7	10430
			6449	327.9	7800	6449	328.8	10420
			6450	327.9	7800	6450	328.8	10420
			6451	327.9	7820	6451	328.8	10430
			6452	328	7810	6452	328.7	10430
			6453	328	7810	6453	328.6	10430
			6454	328	7790	6454	328.6	10440
			6455	328	7800	6455	328.6	10430
			6456	328.1	7800	6456	328.7	10430
			6457	328.1	7810	6457	328.6	10420
			6458	328	7810	6458	328.6	10420
			6459	328	7820	6459	328.6	10420
			6460	328	7800	6460	328.7	10430
			6461	328.1	7810	6461	328.7	10430
			6462	328.1	7800	6462	328.6	10440
			6463	328.1	7790	6463	328.6	10420
			6464	328.1	7810	6464	328.6	10430
			6465	328.1	7810	6465	328.7	10440
			6466	328.1	7810	6466	328.7	10420
			6467	328	7810	6467	328.6	10440
			6468	328	7810	6468	328.6	10440
			6469	328	7820	6469	328.6	10420
			6470	328	7800	6470	328.7	10430
			6471	328	7800	6471	328.6	10430
			6472	328	7800	6472	328.7	10420
			6473	328.1	7810	6473	328.7	10440
			6474	328.1	7810	6474	328.6	10340
			6475	328.1	7810	6475	328.7	10330
			6476	328.1	7800	6476	328.6	10430
			6477	328.1	7820	6477	328.7	10340
			6478	328.1	7810	6478	328.7	10330
			6479	328.1	7810	6479	328.7	10430
			6480	328.1	7820	6480	328.6	10440
			6481	328.1	7810	6481	328.6	10330
			6482	328.1	7820	6482	328.6	10320
			6483	328.1	7820	6483	328.7	10330
			6484	328.1	7810	6484	328.7	10330
			6485	328.1	7810	6485	328.7	10320
			6486	328.1	7820	6486	328.7	10340
			6487	328.2	7820	6487	328.6	10430
			6488	328.1	7820	6488	328.6	10440
			6489	328.2	7810	6489	328.6	10430
			6490	328.1	7810	6490	328.6	10420
			6491	328.1	7810	6491	328.5	10320
			6492	328.1	7800	6492	328.5	10330
			6493	328.1	7820	6493	328.5	10430
			6494	328	7820	6494	328.6	10430
			6495	328	7800	6495	328.7	10340
			6496	328	7800	6496	328.6	10440

			6497	328	7800	6497	328.6	10440
			6498	328	7810	6498	328.6	10340
			6499	328.1	7810	6499	328.6	10330
			6500	328.1	7810	6500	328.6	10320
			6501	328.1	7810	6501	328.6	10330
			6502	328.1	7800	6502	328.6	10330
			6503	328.2	7820	6503	328.6	10340
			6504	328.2	7810	6504	328.7	10330
			6505	328.2	7810	6505	328.7	10330
			6506	328.2	7810	6506	328.7	10340
			6507	328.2	7820	6507	328.6	10330
			6508	328.2	7810	6508	328.6	10340
			6509	328.2	7810	6509	328.5	10330
			6510	328.2	7800	6510	328.5	10430
			6511	328.2	7800	6511	328.6	10320
			6512	328.1	7800	6512	328.6	10320
			6513	328.2	7800	6513	328.5	10330
			6514	328.2	7800	6514	328.5	10320
			6515	328.2	7810	6515	328.5	10340
			6516	328.2	7820	6516	328.5	10330
			6517	328.2	7810	6517	328.5	10340
			6518	328.1	7800	6518	328.5	10330
			6519	328.1	7800	6519	328.5	10340
			6520	328.1	7810	6520	328.4	10340
			6521	328.1	7810	6521	328.4	10320
			6522	328.2	7800	6522	328.4	10330
			6523	328.2	7820	6523	328.4	10320
			6524	328.2	7790	6524	328.4	10320
			6525	328.3	7800	6525	328.4	10330
			6526	328.3	7800	6526	328.4	10330
			6527	328.3	7810	6527	328.4	10330
			6528	328.3	7810	6528	328.5	10320
			6529	328.2	7820	6529	328.4	10330
			6530	328.3	7820	6530	328.4	10330
			6531	328.2	7810	6531	328.4	10330
			6532	328.2	7800	6532	328.4	10330
			6533	328.2	7800	6533	328.4	10330
			6534	328.2	7810	6534	328.5	10340
			6535	328.3	7810	6535	328.5	10340
			6536	328.3	7810	6536	328.5	10330
			6537	328.3	7810	6537	328.5	10330
			6538	328.2	7800	6538	328.6	10320
			6539	328.3	7810	6539	328.5	10340
			6540	328.3	7810	6540	328.5	10340
			6541	328.3	7820	6541	328.5	10330
			6542	328.3	7800	6542	328.5	10330
			6543	328.3	7820	6543	328.4	10330
			6544	328.4	7810	6544	328.4	10320
			6545	328.4	7820	6545	328.5	10330
			6546	328.4	7810	6546	328.4	10330

			6547	328.4	7810	6547	328.4	10320
			6548	328.4	7820	6548	328.4	10330
			6549	328.4	7820	6549	328.4	10330
			6550	328.4	7810	6550	328.4	10320
			6551	328.4	7800	6551	328.3	10320
			6552	328.4	7820	6552	328.3	10330
			6553	328.4	7810	6553	328.2	10320
			6554	328.4	7800	6554	328.3	10330
			6555	328.4	7810	6555	328.2	10330
			6556	328.4	7810	6556	328.3	10340
			6557	328.4	7820	6557	328.2	10330
			6558	328.4	7820	6558	328.3	10330
			6559	328.4	7820	6559	328.3	10340
			6560	328.3	7820	6560	328.3	10330
			6561	328.4	7810	6561	328.4	10320
			6562	328.4	7810	6562	328.4	10330
			6563	328.3	7820	6563	328.3	10330
			6564	328.3	7810	6564	328.3	10330
			6565	328.3	7810	6565	328.3	10320
			6566	328.3	7810	6566	328.3	10340
			6567	328.3	7800	6567	328.3	10340
			6568	328.4	7810	6568	328.3	10330
			6569	328.3	7800	6569	328.3	10320
			6570	328.4	7820	6570	328.3	10330
			6571	328.4	7800	6571	328.3	10330
			6572	328.4	7810	6572	328.2	10310
			6573	328.3	7820	6573	328.2	10320
			6574	328.3	7810	6574	328.3	10320
			6575	328.3	7800	6575	328.3	10330
			6576	328.3	7810	6576	328.2	10320
			6577	328.3	7810	6577	328.2	10330
			6578	328.3	7810	6578	328.2	10320
			6579	328.3	7810	6579	328.3	10320
			6580	328.3	7810	6580	328.3	10310
			6581	328.3	7800	6581	328.3	10330
			6582	328.4	7810	6582	328.3	10330
			6583	328.4	7810	6583	328.3	10330
			6584	328.3	7820	6584	328.3	10330
			6585	328.3	7810	6585	328.3	10320
			6586	328.3	7810	6586	328.3	10320
			6587	328.3	7790	6587	328.3	10320
			6588	328.3	7820	6588	328.3	10330
			6589	328.2	7820	6589	328.3	10320
			6590	328.2	7810	6590	328.3	10320
			6591	328.3	7820	6591	328.3	10320
			6592	328.3	7820	6592	328.3	10310
			6593	328.2	7820	6593	328.3	10320
			6594	328.3	7820	6594	328.3	10320
			6595	328.2	7810	6595	328.4	10320
			6596	328.3	7810	6596	328.4	10320

			6597	328.3	7820	6597	328.4	10330
			6598	328.3	7800	6598	328.5	10330
			6599	328.3	7910	6599	328.4	10330
			6600	328.8	7900	6600	328.4	10320
			6601	329.6	7900	6601	328.4	10340
			6602	330.4	7900	6602	328.4	10340
			6603	331.2	7900	6603	328.4	10340
			6604	331.9	7900	6604	328.3	10330
			6605	332.5	7990	6605	328.3	10320
			6606	333.1	7910	6606	328.4	10340
			6607	333.6	7990	6607	328.4	10330
			6608	334.2	7900	6608	328.3	10330
			6609	334.7	8010	6609	328.3	10330
			6610	335.2	8010	6610	328.3	10320
			6611	335.5	8010	6611	328.3	10330
			6612	335.9	8000	6612	328.3	10330
			6613	336.2	8000	6613	328.4	10330
			6614	336.5	8010	6614	328.3	10320
			6615	336.8	7990	6615	328.4	10320
			6616	337	7990	6616	328.3	10330
			6617	337.2	7990	6617	328.4	10320
			6618	337.5	8000	6618	328.4	10330
			6619	337.7	7990	6619	328.4	10330
			6620	337.9	8000	6620	328.4	10330
			6621	338.1	8010	6621	328.4	10330
			6622	338.3	8000	6622	328.4	10320
			6623	338.5	7990	6623	328.3	10330
			6624	338.6	8000	6624	328.3	10330
			6625	338.9	8000	6625	328.3	10320
			6626	339	7990	6626	328.3	10320
			6627	339.1	7990	6627	328.3	10340
			6628	339.2	7990	6628	328.3	10340
			6629	339.4	7990	6629	328.4	10330
			6630	339.4	8000	6630	328.3	10340
			6631	339.6	8010	6631	328.3	10330
			6632	339.7	8000	6632	328.3	10320
			6633	339.8	7990	6633	328.3	10330
			6634	339.9	8000	6634	328.3	10340
			6635	340	7990	6635	328.3	10330
			6636	340.1	8010	6636	328.4	10320
			6637	340.2	7990	6637	328.4	10330
			6638	340.3	8000	6638	328.3	10330
			6639	340.4	8000	6639	328.3	10320
			6640	340.6	8000	6640	328.3	10330
			6641	340.7	7990	6641	328.3	10320
			6642	340.9	8000	6642	328.3	10320
			6643	341.1	8000	6643	328.4	10330
			6644	341.2	7990	6644	328.4	10330
			6645	341.4	8000	6645	328.4	10340
			6646	341.6	8010	6646	328.4	10330

			6647	341.7	8000	6647	328.4	10330
			6648	341.8	8090	6648	328.4	10340
			6649	342	8100	6649	328.5	10340
			6650	342.2	8110	6650	328.5	10340
			6651	342.4	8110	6651	328.4	10320
			6652	342.6	8100	6652	328.5	10320
			6653	342.7	8100	6653	328.5	10320
			6654	342.9	8090	6654	328.4	10340
			6655	343	8110	6655	328.4	10330
			6656	343.2	8100	6656	328.4	10320
			6657	343.4	8090	6657	328.4	10310
			6658	343.4	8100	6658	328.4	10320
			6659	343.6	8100	6659	328.5	10320
			6660	343.7	8100	6660	328.5	10320
			6661	343.9	8100	6661	328.4	10330
			6662	344.1	8090	6662	328.5	10320
			6663	344.2	8100	6663	328.4	10340
			6664	344.3	8100	6664	328.5	10330
			6665	344.4	8090	6665	328.5	10330
			6666	344.4	8090	6666	328.5	10320
			6667	344.4	8080	6667	328.5	10330
			6668	344.5	8100	6668	328.5	10320
			6669	344.6	8100	6669	328.5	10330
			6670	344.7	8100	6670	328.5	10330
			6671	344.8	8100	6671	328.5	10330
			6672	344.9	8100	6672	328.6	10320
			6673	344.9	8100	6673	328.5	10330
			6674	345	8090	6674	328.5	10320
			6675	345	8110	6675	328.5	10330
			6676	345.1	8120	6676	328.5	10320
			6677	345.2	8090	6677	328.5	10320
			6678	345.2	8090	6678	328.5	10330
			6679	345.4	8090	6679	328.5	10320
			6680	345.4	8100	6680	328.5	10320
			6681	345.5	8100	6681	328.5	10340
			6682	345.5	8090	6682	328.5	10330
			6683	345.6	8080	6683	328.5	10330
			6684	345.7	8110	6684	328.5	10330
			6685	345.8	8100	6685	328.5	10340
			6686	345.8	8100	6686	328.6	10330
			6687	345.9	8100	6687	328.6	10330
			6688	345.9	8090	6688	328.6	10320
			6689	345.9	8100	6689	328.6	10320
			6690	346	8100	6690	328.6	10330
			6691	346.1	8100	6691	328.6	10330
			6692	346.1	8090	6692	328.6	10330
			6693	346.2	8090	6693	328.6	10330
			6694	346.3	8090	6694	328.6	10330
			6695	346.4	8100	6695	328.6	10330
			6696	346.4	8100	6696	328.6	10310

			6697	346.4	8110	6697	328.7	10330
			6698	346.5	8100	6698	328.6	10330
			6699	346.5	8100	6699	328.7	10310
			6700	346.6	8100	6700	328.7	10320
			6701	346.6	8100	6701	328.7	10320
			6702	346.7	8090	6702	328.7	10330
			6703	346.8	8100	6703	328.7	10320
			6704	346.8	8090	6704	328.8	10330
			6705	346.8	8090	6705	328.7	10320
			6706	346.9	8090	6706	328.7	10330
			6707	347	8190	6707	328.7	10320
			6708	347	8090	6708	328.7	10330
			6709	347.1	8090	6709	328.7	10330
			6710	347.2	8100	6710	328.8	10330
			6711	347.2	8090	6711	328.8	10340
			6712	347.3	8200	6712	328.8	10330
			6713	347.3	8090	6713	328.8	10320
			6714	347.4	8100	6714	328.8	10330
			6715	347.4	8090	6715	328.8	10330
			6716	347.4	8100	6716	328.8	10320
			6717	347.4	8090	6717	328.8	10320
			6718	347.4	8190	6718	329.1	10420
			6719	347.5	8090	6719	329.6	10410
			6720	347.5	8090	6720	330.3	10510
			6721	347.5	8190	6721	331.1	10520
			6722	347.5	8200	6722	332.1	10510
			6723	347.6	8200	6723	333.2	10520
			6724	347.6	8200	6724	334.3	10510
			6725	347.6	8110	6725	335.2	10610
			6726	347.7	8200	6726	336.4	10610
			6727	347.8	8190	6727	337.5	10620
			6728	347.8	8110	6728	338.7	10610
			6729	347.8	8100	6729	339.8	10610
			6730	347.8	8090	6730	340.8	10600
			6731	347.9	8200	6731	341.8	10710
			6732	347.9	8100	6732	342.6	10710
			6733	347.9	8100	6733	343.4	10710
			6734	347.9	8100	6734	344.2	10690
			6735	347.9	8200	6735	344.9	10700
			6736	347.9	8110	6736	345.6	10700
			6737	347.9	8110	6737	346.2	10710
			6738	347.9	8100	6738	346.7	10700
			6739	347.9	8190	6739	347.2	10710
			6740	348	8110	6740	347.7	10710
			6741	348	8100	6741	348.1	10710
			6742	348	8100	6742	348.6	10700
			6743	348	8100	6743	349	10720
			6744	348	8200	6744	349.3	10710
			6745	348.1	8190	6745	349.6	10710
			6746	348.1	8180	6746	349.9	10720

			6747	348.1	8200	6747	350.1	10710
			6748	348.2	8200	6748	350.2	10720
			6749	348.2	8200	6749	350.3	10710
			6750	348.2	8210	6750	350.4	10720
			6751	348.3	8090	6751	350.5	10710
			6752	348.3	8200	6752	350.6	10720
			6753	348.4	8200	6753	350.7	10710
			6754	348.5	8190	6754	350.8	10720
			6755	348.5	8200	6755	350.8	10710
			6756	348.5	8200	6756	350.8	10720
			6757	348.5	8190	6757	350.9	10720
			6758	348.6	8190	6758	350.9	10710
			6759	348.6	8190	6759	351	10720
			6760	348.7	8180	6760	351	10710
			6761	348.7	8190	6761	351.1	10710
			6762	348.8	8190	6762	351.2	10710
			6763	348.8	8180	6763	351.3	10710
			6764	348.8	8180	6764	351.3	10720
			6765	348.9	8190	6765	351.3	10710
			6766	348.9	8190	6766	351.3	10710
			6767	349	8190	6767	351.3	10700
			6768	348.9	8200	6768	351.3	10710
			6769	349	8180	6769	351.4	10800
			6770	349	8190	6770	351.4	10810
			6771	349	8200	6771	351.5	10810
			6772	349	8200	6772	351.6	10820
			6773	349.1	8190	6773	351.6	10720
			6774	349.1	8190	6774	351.7	10810
			6775	349.1	8200	6775	351.7	10710
			6776	349.2	8190	6776	351.7	10810
			6777	349.2	8200	6777	351.7	10820
			6778	349.2	8190	6778	351.7	10810
			6779	349.2	8190	6779	351.8	10700
			6780	349.2	8190	6780	351.7	10710
			6781	349.2	8200	6781	351.7	10710
			6782	349.3	8190	6782	351.7	10810
			6783	349.3	8180	6783	351.8	10710
			6784	349.4	8190	6784	351.8	10700
			6785	349.4	8090	6785	351.9	10710
			6786	349.4	8190	6786	351.9	10710
			6787	349.4	8200	6787	351.8	10710
			6788	349.4	8200	6788	351.9	10710
			6789	349.5	8190	6789	351.8	10720
			6790	349.5	8090	6790	351.8	10710
			6791	349.5	8090	6791	351.8	10710
			6792	349.5	8190	6792	351.7	10710
			6793	349.5	8200	6793	351.7	10720
			6794	349.5	8100	6794	351.7	10800
			6795	349.5	8100	6795	351.7	10700
			6796	349.6	8090	6796	351.8	10710

			6797	349.6	8090	6797	351.8	10710
			6798	349.6	8200	6798	351.9	10720
			6799	349.6	8190	6799	351.9	10710
			6800	349.7	8200	6800	351.9	10710
			6801	349.7	8100	6801	352	10810
			6802	349.7	8200	6802	352	10710
			6803	349.6	8100	6803	352	10700
			6804	349.6	8100	6804	352	10690
			6805	349.6	8190	6805	351.9	10710
			6806	349.6	8090	6806	351.9	10700
			6807	349.6	8100	6807	351.9	10700
			6808	349.6	8100	6808	351.9	10700
			6809	349.6	8180	6809	351.9	10710
			6810	349.7	8200	6810	351.8	10720
			6811	349.7	8200	6811	351.7	10700
			6812	349.8	8090	6812	351.6	10710
			6813	349.8	8090	6813	351.6	10720
			6814	349.9	8200	6814	351.5	10730
			6815	350	8100	6815	351.5	10710
			6816	350	8090	6816	351.4	10720
			6817	350	8200	6817	351.4	10720
			6818	350	8200	6818	351.4	10700
			6819	350	8180	6819	351.4	10710
			6820	350	8190	6820	351.4	10710
			6821	350	8100	6821	351.3	10710
			6822	349.9	8200	6822	351.3	10710
			6823	350	8190	6823	351.3	10720
			6824	350	8200	6824	351.3	10720
			6825	350.1	8190	6825	351.3	10700
			6826	350.1	8190	6826	351.4	10710
			6827	350.2	8180	6827	351.4	10710
			6828	350.2	8190	6828	351.4	10710
			6829	350.2	8090	6829	351.3	10700
			6830	350.2	8200	6830	351.3	10700
			6831	350.3	8090	6831	351.3	10700
			6832	350.3	8190	6832	351.2	10710
			6833	350.3	8190	6833	351.2	10710
			6834	350.4	8200	6834	351.2	10710
			6835	350.4	8110	6835	351.2	10700
			6836	350.4	8190	6836	351.3	10700
			6837	350.5	8100	6837	351.3	10690
			6838	350.5	8100	6838	351.4	10710
			6839	350.5	8100	6839	351.4	10710
			6840	350.5	8100	6840	351.4	10710
			6841	350.5	8200	6841	351.4	10710
			6842	350.4	8190	6842	351.4	10710
			6843	350.5	8100	6843	351.4	10710
			6844	350.5	8090	6844	351.4	10710
			6845	350.5	8190	6845	351.4	10700
			6846	350.5	8100	6846	351.4	10700

			6847	350.5	8110	6847	351.5	10710
			6848	350.5	8200	6848	351.5	10710
			6849	350.5	8100	6849	351.5	10710
			6850	350.5	8090	6850	351.5	10690
			6851	350.6	8090	6851	351.5	10700
			6852	350.5	8200	6852	351.5	10700
			6853	350.5	8200	6853	351.5	10710
			6854	350.5	8200	6854	351.5	10710
			6855	350.6	8090	6855	351.6	10710
			6856	350.5	8190	6856	351.6	10710
			6857	350.6	8090	6857	351.7	10710
			6858	350.6	8090	6858	351.7	10720
			6859	350.6	8180	6859	351.8	10700
			6860	350.6	8190	6860	351.8	10700
			6861	350.5	8100	6861	351.8	10700
			6862	350.5	8080	6862	351.8	10700
			6863	350.5	8100	6863	351.8	10700
			6864	350.6	8100	6864	351.8	10700
			6865	350.6	8100	6865	351.8	10700
			6866	350.6	8190	6866	351.8	10700
			6867	350.7	8100	6867	351.7	10710
			6868	350.7	8190	6868	351.8	10700
			6869	350.7	8110	6869	351.8	10710
			6870	350.7	8200	6870	351.8	10710
			6871	350.7	8180	6871	351.8	10710
			6872	350.8	8090	6872	351.9	10710
			6873	350.8	8080	6873	351.9	10700
			6874	350.8	8090	6874	352	10700
			6875	350.8	8190	6875	352.1	10710
			6876	350.9	8090	6876	352.1	10700
			6877	350.9	8110	6877	352.1	10690
			6878	350.9	8100	6878	352.1	10710
			6879	350.9	8090	6879	352	10700
			6880	350.9	8190	6880	351.9	10700
			6881	350.9	8090	6881	351.9	10700
			6882	350.9	8090	6882	351.8	10710
			6883	350.9	8090	6883	351.7	10690
			6884	350.9	8080	6884	351.7	10710
			6885	350.9	8090	6885	351.7	10700
			6886	350.9	8100	6886	351.6	10600
			6887	350.9	8190	6887	351.6	10700
			6888	350.9	8090	6888	351.6	10710
			6889	350.9	8200	6889	351.6	10710
			6890	351	8090	6890	351.6	10710
			6891	351	8100	6891	351.5	10620
			6892	351.1	8090	6892	351.5	10610
			6893	351.1	8090	6893	351.4	10710
			6894	351.1	8080	6894	351.5	10600
			6895	351.1	8100	6895	351.5	10600
			6896	351.1	8090	6896	351.5	10600

			6897	351.1	8090	6897	351.5	10600
			6898	351.1	8080	6898	351.5	10600
			6899	351.2	8080	6899	351.6	10610
			6900	351.2	8100	6900	351.6	10710
			6901	351.3	8100	6901	351.6	10600
			6902	351.3	8100	6902	351.5	10600
			6903	351.3	8080	6903	351.5	10600
			6904	351.2	8080	6904	351.5	10610
			6905	351.2	8100	6905	351.5	10600
			6906	351.2	8190	6906	351.4	10600
			6907	351.2	8200	6907	351.5	10610
			6908	351.2	8100	6908	351.4	10600
			6909	351.2	8080	6909	351.5	10600
			6910	351.2	8080	6910	351.5	10600
			6911	351.2	8100	6911	351.5	10610
			6912	351.2	8090	6912	351.5	10600
			6913	351.2	8080	6913	351.5	10610
			6914	351.1	8090	6914	351.5	10610
			6915	351.1	8100	6915	351.5	10600
			6916	351.2	8080	6916	351.5	10610
			6917	351.3	8100	6917	351.5	10600
			6918	351.3	8090	6918	351.5	10610
			6919	351.4	8090	6919	351.5	10590
			6920	351.4	8080	6920	351.6	10600
			6921	351.5	8080	6921	351.5	10610
			6922	351.5	8090	6922	351.6	10590
			6923	351.5	8080	6923	351.6	10610
			6924	351.6	8080	6924	351.6	10600
			6925	351.6	8080	6925	351.7	10620
			6926	351.6	8100	6926	351.6	10610
			6927	351.7	8100	6927	351.6	10610
			6928	351.7	8090	6928	351.6	10600
			6929	351.7	8100	6929	351.6	10610
			6930	351.7	8090	6930	351.6	10610
			6931	351.7	8090	6931	351.5	10610
			6932	351.7	8090	6932	351.6	10590
			6933	351.7	8090	6933	351.6	10600
			6934	351.8	8100	6934	351.6	10590
			6935	351.8	8100	6935	351.6	10600
			6936	351.8	8100	6936	351.6	10590
			6937	351.8	8100	6937	351.7	10600
			6938	351.8	8100	6938	351.8	10600
			6939	351.8	8100	6939	351.7	10600
			6940	351.8	8090	6940	351.7	10590
			6941	351.8	8080	6941	351.8	10600
			6942	351.7	8100	6942	351.7	10590
			6943	351.8	8090	6943	351.7	10610
			6944	351.8	8080	6944	351.7	10610
			6945	351.8	8090	6945	351.6	10610
			6946	351.8	8100	6946	351.7	10600

			6947	351.8	8090	6947	351.7	10590
			6948	351.8	8100	6948	351.6	10600
			6949	351.9	8090	6949	351.6	10590
			6950	351.8	8090	6950	351.6	10590
			6951	351.8	8090	6951	351.6	10600
			6952	351.8	8100	6952	351.7	10600
			6953	351.8	8100	6953	351.6	10590
			6954	351.9	8090	6954	351.7	10600
			6955	351.9	8090	6955	351.7	10610
			6956	351.8	8090	6956	351.7	10610
			6957	351.8	8080	6957	351.7	10610
			6958	351.8	8090	6958	351.7	10600
			6959	351.8	8100	6959	351.7	10590
			6960	351.8	8090	6960	351.7	10590
			6961	351.8	8090	6961	351.7	10600
			6962	351.8	8090	6962	351.8	10610
			6963	351.8	8100	6963	351.8	10610
			6964	351.8	8090	6964	351.7	10580
			6965	351.8	8100	6965	351.7	10590
			6966	351.8	8080	6966	351.7	10600
			6967	351.8	8110	6967	351.8	10600
			6968	351.8	8080	6968	351.7	10610
			6969	351.8	8090	6969	351.8	10590
			6970	351.9	8100	6970	351.8	10590
			6971	351.9	8090	6971	351.8	10590
			6972	351.9	8090	6972	351.8	10590
			6973	351.9	8090	6973	351.8	10600
			6974	351.9	8080	6974	351.8	10590
			6975	351.9	8090	6975	351.8	10600
			6976	351.9	8090	6976	351.8	10590
			6977	351.9	8090	6977	351.8	10590
			6978	352	8090	6978	351.8	10610
			6979	352	8100	6979	351.8	10600
			6980	352	8090	6980	351.8	10600
			6981	352	8090	6981	351.9	10600
			6982	352	8080	6982	351.9	10600
			6983	352	8180	6983	351.9	10600
			6984	352.1	8090	6984	351.9	10590
			6985	352	8090	6985	351.9	10590
			6986	352.1	8190	6986	351.9	10590
			6987	352.1	8090	6987	352	10590
			6988	352.1	8190	6988	352	10590
			6989	352	8100	6989	352	10600
			6990	352.1	8090	6990	352	10590
			6991	352.1	8090	6991	352	10590
			6992	352.1	8090	6992	352.1	10600
			6993	352	8100	6993	352.1	10600
			6994	352.1	8090	6994	352	10600
			6995	352	8100	6995	352	10610
			6996	352	8080	6996	352	10600

			6997	352	8090	6997	352	10600
			6998	352.1	8080	6998	352	10590
			6999	352	8080	6999	352	10600
			7000	352	8090	7000	351.9	10610
			7001	352	8090	7001	351.9	10590
			7002	352	8090	7002	351.9	10600
			7003	352	8080	7003	351.9	10600
			7004	352	8080	7004	351.9	10590
			7005	352	8080	7005	351.9	10600
			7006	352	8080	7006	351.9	10600
			7007	352.1	8080	7007	351.9	10590
			7008	352.1	8090	7008	352	10600
			7009	352	8090	7009	352	10600
			7010	352.1	8100	7010	352	10600
			7011	352.1	8090	7011	352	10600
			7012	352.1	8090	7012	352	10600
			7013	352.1	8080	7013	352	10600
			7014	352.1	8070	7014	352	10600
			7015	352.1	8080	7015	352	10600
			7016	352.1	8080	7016	352.1	10610
			7017	352.2	8080	7017	352	10610
			7018	352.2	8080	7018	352	10600
			7019	352.2	8090	7019	352	10610
			7020	352.2	8080	7020	352	10610
			7021	352.2	8080	7021	352	10600
			7022	352.2	8080	7022	351.9	10610
			7023	352.2	8080	7023	352	10600
			7024	352.2	8090	7024	352	10600
			7025	352.2	8070	7025	352	10610
			7026	352.3	8080	7026	352	10610
			7027	352.2	8070	7027	352	10610
			7028	352.3	8070	7028	352	10590
			7029	352.3	8080	7029	352	10600
			7030	352.3	8070	7030	352	10590
			7031	352.3	8080	7031	352.1	10600
			7032	352.3	8080	7032	352.1	10590
			7033	352.3	8100	7033	352.1	10600
			7034	352.3	8090	7034	352.1	10490
			7035	352.3	8080	7035	352.2	10600
			7036	352.3	8090	7036	352.2	10600
			7037	352.3	8090	7037	352.2	10610
			7038	352.3	8090	7038	352.2	10600
			7039	352.3	8100	7039	352.3	10600
			7040	352.3	8080	7040	352.3	10610
			7041	352.3	8080	7041	352.4	10600
			7042	352.3	8080	7042	352.4	10590
			7043	352.3	8080	7043	352.4	10600
			7044	352.3	8080	7044	352.4	10600
			7045	352.3	8090	7045	352.4	10610
			7046	352.3	8090	7046	352.4	10600

			7047	352.4	8080	7047	352.4	10500
			7048	352.4	8090	7048	352.3	10490
			7049	352.4	8080	7049	352.3	10490
			7050	352.4	8090	7050	352.3	10500
			7051	352.4	8080	7051	352.2	10500
			7052	352.3	8080	7052	352.2	10610
			7053	352.3	8090	7053	352.2	10600
			7054	352.3	8070	7054	352.2	10490
			7055	352.3	8090	7055	352.2	10490
			7056	352.3	8090	7056	352.2	10500
			7057	352.3	8090	7057	352.2	10490
			7058	352.3	8080	7058	352.2	10510
			7059	352.2	8080	7059	352.2	10510
			7060	352.2	8070	7060	352.2	10510
			7061	352.3	8080	7061	352.2	10510
			7062	352.3	8090	7062	352.1	10480
			7063	352.3	8070	7063	352.1	10480
			7064	352.2	8080	7064	352.1	10490
			7065	352.3	8080	7065	352.1	10490
			7066	352.3	8080	7066	352.1	10500
			7067	352.2	8080	7067	352.1	10490
			7068	352.3	8070	7068	352.2	10510
			7069	352.3	8080	7069	352.2	10500
			7070	352.2	8090	7070	352.2	10500
			7071	352.3	8060	7071	352.2	10500
			7072	352.3	8060	7072	352.2	10510
			7073	352.2	8080	7073	352.2	10500
			7074	352.2	8080	7074	352.2	10500
			7075	352.2	8070	7075	352.3	10510
			7076	352.2	8080	7076	352.3	10490
			7077	352.2	8080	7077	352.2	10500
			7078	352.2	8070	7078	352.2	10500
			7079	352.3	8080	7079	352.2	10500
			7080	352.3	8090	7080	352.2	10500
			7081	352.3	8080	7081	352.2	10510
			7082	352.4	8080	7082	352.2	10600
			7083	352.3	8070	7083	352.1	10500
			7084	352.4	8080	7084	352.1	10490
			7085	352.3	8070	7085	352.1	10500
			7086	352.4	8080	7086	352.2	10490
			7087	352.4	8090	7087	352.2	10490
			7088	352.4	8090	7088	352.2	10500
			7089	352.3	8080	7089	352.2	10500
			7090	352.3	8090	7090	352.2	10500
			7091	352.3	8080	7091	352.2	10490
			7092	352.3	8080	7092	352.1	10490
			7093	352.3	8090	7093	352.2	10500
			7094	352.4	8080	7094	352.1	10500
			7095	352.4	8080	7095	352.1	10510
			7096	352.4	8080	7096	352.1	10500

			7097	352.4	8070	7097	352.2	10490
			7098	352.4	8080	7098	352.2	10510
			7099	352.4	8090	7099	352.3	10500
			7100	352.4	8090	7100	352.2	10480
			7101	352.4	8080	7101	352.2	10500
			7102	352.5	8090	7102	352.3	10500
			7103	352.5	8090	7103	352.3	10500
			7104	352.5	8090	7104	352.3	10510
			7105	352.6	8080	7105	352.2	10500
			7106	352.6	8090	7106	352.3	10510
			7107	352.6	8100	7107	352.3	10510
			7108	352.5	8080	7108	352.3	10510
			7109	352.6	8080	7109	352.3	10500
			7110	352.6	8070	7110	352.3	10490
			7111	352.5	8080	7111	352.4	10490
			7112	352.5	8070	7112	352.4	10500
			7113	352.5	8070	7113	352.4	10500
			7114	352.5	8070	7114	352.4	10490
			7115	352.5	8070	7115	352.4	10490
			7116	352.5	8080	7116	352.4	10480
			7117	352.5	8070	7117	352.4	10500
			7118	352.5	8080	7118	352.4	10500
			7119	352.5	8080	7119	352.4	10500
			7120	352.5	8070	7120	352.4	10500
			7121	352.5	8080	7121	352.3	10490
			7122	352.4	8070	7122	352.3	10490
			7123	352.4	8080	7123	352.3	10500
			7124	352.4	8080	7124	352.3	10500
			7125	352.4	8080	7125	352.3	10500
			7126	352.4	8070	7126	352.3	10490
			7127	352.4	8070	7127	352.3	10490
			7128	352.4	8080	7128	352.4	10500
			7129	352.4	8070	7129	352.3	10490
			7130	352.4	8070	7130	352.4	10500
			7131	352.4	8070	7131	352.4	10490
			7132	352.4	8080	7132	352.4	10490
			7133	352.3	8090	7133	352.4	10490
			7134	352.3	8080	7134	352.4	10500
			7135	352.3	8090	7135	352.4	10480
			7136	352.3	8090	7136	352.4	10490
			7137	352.3	8080	7137	352.4	10480
			7138	352.3	8080	7138	352.3	10490
			7139	352.3	8090	7139	352.4	10500
			7140	352.3	8080	7140	352.4	10500
			7141	352.3	8090	7141	352.4	10500
			7142	352.3	8090	7142	352.4	10490
			7143	352.3	8070	7143	352.4	10490
			7144	352.3	8070	7144	352.4	10480
			7145	352.3	8090	7145	352.4	10490
			7146	352.4	8070	7146	352.5	10490

			7147	352.4	8080	7147	352.4	10490
			7148	352.4	8080	7148	352.4	10480
			7149	352.4	8070	7149	352.5	10490
			7150	352.3	8080	7150	352.4	10490
			7151	352.3	8080	7151	352.5	10490
			7152	352.3	8080	7152	352.5	10480
			7153	352.4	8070	7153	352.5	10480
			7154	352.4	8070	7154	352.5	10490
			7155	352.4	8080	7155	352.5	10480
			7156	352.4	8080	7156	352.5	10480
			7157	352.4	8090	7157	352.6	10500
			7158	352.4	8080	7158	352.6	10490
			7159	352.3	8090	7159	352.6	10490
			7160	352.3	8070	7160	352.6	10490
			7161	352.4	8060	7161	352.6	10490
			7162	352.4	8060	7162	352.6	10480
			7163	352.4	8080	7163	352.6	10490
			7164	352.5	8080	7164	352.7	10500
			7165	352.4	8080	7165	352.6	10490
			7166	352.4	8090	7166	352.6	10490
			7167	352.5	8090	7167	352.6	10390
			7168	352.5	8090	7168	352.5	10400
			7169	352.5	8080	7169	352.5	10490
			7170	352.5	8090	7170	352.5	10490
			7171	352.5	8080	7171	352.5	10490
			7172	352.5	8080	7172	352.4	10490
			7173	352.5	8080	7173	352.4	10490
			7174	352.5	8080	7174	352.4	10490
			7175	352.5	8080	7175	352.4	10500
			7176	352.5	8080	7176	352.5	10380
			7177	352.5	8080	7177	352.5	10490
			7178	352.5	8090	7178	352.5	10380
			7179	352.5	8080	7179	352.5	10500
			7180	352.6	8080	7180	352.6	10500
			7181	352.6	8080	7181	352.6	10490
			7182	352.6	8080	7182	352.6	10480
			7183	352.6	8090	7183	352.6	10400
			7184	352.7	8080	7184	352.6	10480
			7185	352.7	8070	7185	352.7	10390
			7186	352.7	8070	7186	352.6	10490
			7187	352.6	8080	7187	352.6	10480
			7188	352.7	8070	7188	352.6	10500
			7189	352.7	8070	7189	352.6	10380
			7190	352.7	8070	7190	352.6	10380
			7191	352.7	8080	7191	352.6	10390
			7192	352.7	8080	7192	352.6	10390
			7193	352.7	8090	7193	352.6	10390
			7194	352.8	8080	7194	352.6	10400
			7195	352.8	8080	7195	352.6	10390
			7196	352.9	8090	7196	352.6	10390

			7197	352.9	8090	7197	352.6	10390
			7198	352.9	8070	7198	352.6	10400
			7199	352.9	8090	7199	352.5	10390
			7200	353	8080	7200	352.6	10400
			7201	353	8080	7201	352.5	10390
			7202	353	8070	7202	352.6	10390
			7203	353	8080	7203	352.6	10390
			7204	353	8080	7204	352.6	10390
			7205	352.9	8090	7205	352.6	10390
			7206	352.9	8100	7206	352.6	10400
			7207	352.9	8080	7207	352.6	10390
			7208	352.9	8090	7208	352.6	10400
			7209	352.9	8070	7209	352.6	10380
			7210	352.9	8070	7210	352.5	10390
			7211	352.9	8090	7211	352.5	10410
			7212	352.9	8090	7212	352.5	10390
			7213	352.9	8090	7213	352.5	10390
			7214	352.9	8090	7214	352.5	10500
			7215	353	8080	7215	352.5	10410
			7216	353	8090	7216	352.6	10400
			7217	353	8070	7217	352.6	10390
			7218	353	8080	7218	352.6	10400
			7219	353	8080	7219	352.6	10390
			7220	353	8090	7220	352.6	10400
			7221	353	8080	7221	352.6	10400
			7222	353	8100	7222	352.6	10390
			7223	353	8080	7223	352.6	10390
			7224	353	8080	7224	352.6	10390
			7225	353	8070	7225	352.6	10390
			7226	352.9	8080	7226	352.6	10390
			7227	352.9	8070	7227	352.6	10390
			7228	352.8	8080	7228	352.6	10390
			7229	352.9	8080	7229	352.6	10390
			7230	352.9	8090	7230	352.6	10380
			7231	352.8	8070	7231	352.6	10390
			7232	352.8	8080	7232	352.6	10400
			7233	352.8	8080	7233	352.6	10400
			7234	352.8	8080	7234	352.7	10390
			7235	352.8	8080	7235	352.7	10400
			7236	352.8	8070	7236	352.7	10390
			7237	352.7	8070	7237	352.7	10380
			7238	352.8	8060	7238	352.7	10400
			7239	352.8	8080	7239	352.7	10380
			7240	352.8	8070	7240	352.7	10380
			7241	352.8	8070	7241	352.7	10380
			7242	352.8	8070	7242	352.7	10390
			7243	352.8	8090	7243	352.7	10400
			7244	352.8	8070	7244	352.7	10390
			7245	352.7	8080	7245	352.7	10390
			7246	352.7	8080	7246	352.7	10390

			7247	352.7	8080	7247	352.8	10400
			7248	352.7	8080	7248	352.8	10390
			7249	352.7	8070	7249	352.7	10390
			7250	352.7	8090	7250	352.7	10390
			7251	352.6	8080	7251	352.6	10380
			7252	352.5	8060	7252	352.6	10400
			7253	352.5	8080	7253	352.6	10380
			7254	352.6	8070	7254	352.7	10390
			7255	352.5	8080	7255	352.7	10400
			7256	352.5	8080	7256	352.7	10390
			7257	352.5	8080	7257	352.8	10380
			7258	352.5	8080	7258	352.8	10390
			7259	352.5	8070	7259	352.8	10390
			7260	352.5	8080	7260	352.7	10390
			7261	352.5	8070	7261	352.8	10390
			7262	352.5	8070	7262	352.8	10400
			7263	352.5	8080	7263	352.8	10400
			7264	352.5	8090	7264	352.8	10390
			7265	352.6	8080	7265	352.8	10400
			7266	352.5	8080	7266	352.8	10390
			7267	352.5	8080	7267	352.7	10390
			7268	352.5	8080	7268	352.8	10390
			7269	352.5	8080	7269	352.8	10390
			7270	352.4	8090	7270	352.7	10390
			7271	352.4	8090	7271	352.7	10400
			7272	352.4	8100	7272	352.7	10380
			7273	352.4	8080	7273	352.7	10400
			7274	352.4	8090	7274	352.7	10390
			7275	352.4	8080	7275	352.8	10400
			7276	352.4	8090	7276	352.8	10390
			7277	352.3	8070	7277	352.8	10390
			7278	352.3	8080	7278	352.9	10380
			7279	352.3	8080	7279	352.8	10390
			7280	352.2	8080	7280	352.8	10390
			7281	352.3	8080	7281	352.8	10400
			7282	352.3	8080	7282	352.8	10380
			7283	352.3	8080	7283	352.9	10380
			7284	352.3	8090	7284	352.9	10380
			7285	352.3	8080	7285	352.9	10400
			7286	352.3	8070	7286	352.9	10390
			7287	352.4	8080	7287	352.9	10400
			7288	352.4	8090	7288	352.9	10400
			7289	352.4	8080	7289	353	10380
			7290	352.4	8070	7290	353	10390
			7291	352.4	8080	7291	352.9	10390
			7292	352.3	8070	7292	352.9	10390
			7293	352.3	8090	7293	352.9	10390
			7294	352.3	8080	7294	353	10400
			7295	352.3	8080	7295	352.9	10400
			7296	352.2	8080	7296	352.9	10400

			7297	352.2	8080	7297	352.9	10390
			7298	352.1	8090	7298	352.9	10400
			7299	352.1	8080	7299	352.9	10400
			7300	352.1	8080	7300	352.9	10390
			7301	352.2	8080	7301	352.9	10390
			7302	352.3	8090	7302	352.9	10390
			7303	352.4	8080	7303	352.9	10390
			7304	352.4	8090	7304	352.9	10390
			7305	352.4	8080	7305	352.9	10400
			7306	352.4	8080	7306	352.9	10400
			7307	352.4	8070	7307	352.9	10300
			7308	352.3	8080	7308	352.9	10290
			7309	352.3	8080	7309	352.9	10390
			7310	352.2	8080	7310	352.9	10400
			7311	352.2	8080	7311	352.8	10300
			7312	352.1	8070	7312	352.8	10290
			7313	352.2	8070	7313	352.8	10390
			7314	352.2	8080	7314	352.8	10290
			7315	352.1	8080	7315	352.8	10400
			7316	352.2	8070	7316	352.8	10400
			7317	352.2	8070	7317	352.9	10390
			7318	352.2	8070	7318	352.9	10390
			7319	352.2	7980	7319	352.9	10290
			7320	352.2	8070	7320	352.9	10290
			7321	352.2	8080	7321	352.9	10300
			7322	352.2	8090	7322	352.8	10390
			7323	352.2	8090	7323	352.8	10290
			7324	352.2	8090	7324	352.8	10390
			7325	352.2	8080	7325	352.9	10290
			7326	352.2	8080	7326	352.9	10400
			7327	352.2	8080	7327	352.8	10290
			7328	352.2	8080	7328	352.8	10390
			7329	352.2	8070	7329	352.8	10290
			7330	352.2	8070	7330	352.8	10390
			7331	352.2	8080	7331	352.9	10400
			7332	352.1	8080	7332	352.9	10280
			7333	352.1	8080	7333	352.9	10290
			7334	352.1	8070	7334	352.9	10290
			7335	352.1	8070	7335	352.9	10390
			7336	352.1	8090	7336	352.9	10400
			7337	352.1	8080	7337	352.9	10400
			7338	352.1	8080	7338	352.9	10290
			7339	352.1	8070	7339	352.9	10300
			7340	352.1	7980	7340	352.9	10290
			7341	352.1	8080	7341	352.9	10280
			7342	352.1	7980	7342	352.9	10270
			7343	352	7990	7343	352.9	10280
			7344	352	7980	7344	352.8	10290
			7345	352	7980	7345	352.8	10290
			7346	352.1	7980	7346	352.9	10300

			7347	352	7980	7347	352.8	10290
			7348	352.1	7980	7348	352.9	10280
			7349	352.1	7980	7349	352.8	10290
			7350	352.1	8080	7350	352.8	10290
			7351	352.1	7980	7351	352.8	10280
			7352	352.1	8070	7352	352.7	10280
			7353	352.2	8080	7353	352.8	10280
			7354	352.2	8070	7354	352.8	10290
			7355	352.2	8070	7355	352.8	10290
			7356	352.2	8090	7356	352.8	10280
			7357	352.1	7970	7357	352.8	10280
			7358	352.1	8080	7358	352.8	10280
			7359	352.1	8090	7359	352.8	10290
			7360	352.2	8080	7360	352.8	10290
			7361	352.2	7980	7361	352.8	10290
			7362	352.1	8070	7362	352.8	10280
			7363	352.2	8080	7363	352.8	10300
			7364	352.2	8070	7364	352.8	10290
			7365	352.1	7990	7365	352.8	10290
			7366	352.1	7980	7366	352.8	10290
			7367	352.1	7980	7367	352.8	10290
			7368	352	7980	7368	352.8	10290
			7369	352.1	7980	7369	352.7	10300
			7370	352.1	7970	7370	352.8	10290
			7371	352	7970	7371	352.8	10290
			7372	352.1	7980	7372	352.8	10290
			7373	352	7980	7373	352.9	10290
			7374	352	7980	7374	352.9	10300
			7375	352	7990	7375	352.9	10280
			7376	352	8080	7376	352.9	10280
			7377	352	7980	7377	352.9	10300
			7378	352	7980	7378	352.9	10290
			7379	352	7980	7379	352.9	10280
			7380	351.9	7970	7380	353	10280
			7381	351.9	7980	7381	353	10270
			7382	351.9	7970	7382	352.9	10280
			7383	352	7990	7383	352.9	10290
			7384	352	7980	7384	353	10290
			7385	352	7980	7385	352.9	10280
			7386	352	7980	7386	352.9	10300
			7387	352	7980	7387	352.9	10290
			7388	352	7980	7388	353	10300
			7389	352.1	7980	7389	352.9	10300
			7390	352.1	7980	7390	352.9	10300
			7391	352	7990	7391	352.9	10300
			7392	352	7980	7392	352.9	10290
			7393	352.1	7980	7393	352.9	10280
			7394	352.1	7970	7394	352.9	10290
			7395	352.1	7980	7395	352.9	10280
			7396	352.2	7980	7396	352.9	10290

			7397	352.2	7980	7397	352.8	10290
			7398	352.2	7970	7398	352.8	10290
			7399	352.2	7980	7399	352.7	10280
			7400	352.2	7970	7400	352.7	10290
			7401	352.1	7970	7401	352.7	10280
			7402	352.1	7980	7402	352.7	10290
			7403	352.1	8000	7403	352.7	10280
			7404	352.1	7970	7404	352.8	10290
			7405	352.1	7980	7405	352.8	10300
			7406	352.1	7980	7406	352.8	10290
			7407	352.1	7980	7407	352.8	10290
			7408	352.1	7980	7408	352.7	10300
			7409	352.1	8000	7409	352.8	10280
			7410	352.1	7980	7410	352.8	10290
			7411	352.1	7980	7411	352.8	10290
			7412	352	7970	7412	352.8	10280
			7413	352	7980	7413	352.8	10280
			7414	352	7970	7414	352.8	10280
			7415	352	7990	7415	352.8	10300
			7416	352	7980	7416	352.8	10280
			7417	351.9	7980	7417	352.7	10300
			7418	352	7990	7418	352.7	10290
			7419	352	7980	7419	352.7	10300
			7420	352	7980	7420	352.7	10290
			7421	352	7990	7421	352.8	10280
			7422	352	7970	7422	352.8	10280
			7423	352	7980	7423	352.8	10280
			7424	352	7980	7424	352.8	10280
			7425	352	7980	7425	352.8	10280
			7426	352	7980	7426	352.9	10290
			7427	352	7970	7427	352.9	10290
			7428	352	7970	7428	352.9	10280
			7429	352	7970	7429	352.9	10290
			7430	352.1	7980	7430	352.9	10290
			7431	352.1	7980	7431	352.9	10290
			7432	352.1	7980	7432	352.9	10280
			7433	352.1	7980	7433	352.9	10280
			7434	352.1	7980	7434	353	10290
			7435	352.1	7970	7435	353	10270
			7436	352.1	7970	7436	353	10290
			7437	352.1	7980	7437	353	10290
			7438	352.1	7990	7438	353	10280
			7439	352.1	7990	7439	353	10290
			7440	352	7990	7440	353	10290
			7441	352	7990	7441	353	10280
			7442	352	7980	7442	353	10290
			7443	352.1	7980	7443	353	10290
			7444	352	7980	7444	353	10270
			7445	352	7970	7445	353	10290
			7446	352	7970	7446	353	10290

			7447	352	7970	7447	352.9	10280
			7448	352	7980	7448	352.9	10290
			7449	352	7970	7449	352.9	10280
			7450	352	7980	7450	352.9	10280
			7451	352	7980	7451	352.9	10290
			7452	352	7970	7452	352.9	10280
			7453	352	7970	7453	352.9	10280
			7454	352	7970	7454	352.9	10290
			7455	352	7970	7455	352.9	10280
			7456	352	7980	7456	352.9	10280
			7457	352.1	7980	7457	352.9	10280
			7458	352	7990	7458	352.9	10290
			7459	352	7980	7459	352.9	10280
			7460	352	7970	7460	352.8	10290
			7461	352.1	7980	7461	352.8	10190
			7462	352.1	7970	7462	352.8	10290
			7463	352.1	7980	7463	352.8	10180
			7464	352.1	7980	7464	352.8	10190
			7465	352.1	7990	7465	352.8	10180
			7466	352	7980	7466	352.7	10190
			7467	352.1	7980	7467	352.8	10180
			7468	352.1	7970	7468	352.8	10190
			7469	352	7970	7469	352.8	10170
			7470	352	7970	7470	352.8	10180
			7471	352.1	7960	7471	352.8	10190
			7472	352.1	7970	7472	352.8	10190
			7473	352.1	7980	7473	352.8	10180
			7474	352.1	7970	7474	352.8	10180
			7475	352	7970	7475	352.8	10180
			7476	352.1	7980	7476	352.9	10190
			7477	352.1	7980	7477	352.8	10190
			7478	352.2	7980	7478	352.8	10190
			7479	352.2	7980	7479	352.7	10200
			7480	352.1	7980	7480	352.7	10190
			7481	352.2	7990	7481	352.7	10190
			7482	352.2	7990	7482	352.8	10190
			7483	352.1	7980	7483	352.8	10190
			7484	352.1	7980	7484	352.8	10190
			7485	352.1	7980	7485	352.7	10200
			7486	352.1	7970	7486	352.7	10190
			7487	352	7990	7487	352.7	10190
			7488	352	7990	7488	352.7	10190
			7489	352	7990	7489	352.7	10180
			7490	352.1	7990	7490	352.7	10190
			7491	352.1	7980	7491	352.7	10190
			7492	352.1	7990	7492	352.7	10190
			7493	352.1	7990	7493	352.7	10180
			7494	352.2	7980	7494	352.8	10190
			7495	352.2	7980	7495	352.8	10190
			7496	352.2	7980	7496	352.8	10180

			7497	352.2	7990	7497	352.8	10180
			7498	352.3	7980	7498	352.8	10190
			7499	352.3	7990	7499	352.9	10180
			7500	352.3	7980	7500	352.8	10180
			7501	352.3	7980	7501	352.8	10200
			7502	352.3	7990	7502	352.8	10190
			7503	352.3	7980	7503	352.8	10190
			7504	352.4	7970	7504	352.9	10190
			7505	352.4	7970	7505	352.8	10190
			7506	352.4	7980	7506	352.8	10200
			7507	352.4	7990	7507	352.8	10190
			7508	352.3	7970	7508	352.8	10190
			7509	352.3	7980	7509	352.8	10190
			7510	352.3	7980	7510	352.8	10190
			7511	352.4	7980	7511	352.8	10200
			7512	352.4	7970	7512	352.8	10190
			7513	352.3	7980	7513	352.8	10190
			7514	352.3	7990	7514	352.8	10180
			7515	352.3	7980	7515	352.8	10180
			7516	352.3	7970	7516	352.9	10190
			7517	352.4	7990	7517	352.9	10190
			7518	352.3	7980	7518	353	10190
			7519	352.4	7970	7519	352.9	10190
			7520	352.4	7980	7520	352.9	10190
			7521	352.4	7970	7521	352.9	10200
			7522	352.4	7980	7522	352.9	10190
			7523	352.4	7970	7523	352.9	10190
			7524	352.4	7970	7524	352.9	10190
			7525	352.4	7990	7525	352.9	10180
			7526	352.4	7990	7526	352.9	10190
			7527	352.4	7980	7527	352.9	10200
			7528	352.4	7990	7528	352.9	10190
			7529	352.4	7990	7529	352.9	10190
			7530	352.4	7980	7530	352.9	10180
			7531	352.4	7980	7531	352.9	10180
			7532	352.3	7990	7532	352.9	10180
			7533	352.3	7990	7533	352.9	10200
			7534	352.3	7980	7534	352.9	10190
			7535	352.3	7970	7535	352.9	10200
			7536	352.3	7980	7536	352.9	10180
			7537	352.3	7990	7537	352.9	10180
			7538	352.2	7970	7538	352.9	10180
			7539	352.2	7970	7539	352.9	10190
			7540	352.3	7970	7540	352.9	10180
			7541	352.3	7980	7541	352.9	10190
			7542	352.2	7980	7542	352.9	10190
			7543	352.3	7980	7543	352.9	10200
			7544	352.3	7990	7544	352.8	10180
			7545	352.3	7980	7545	352.8	10180
			7546	352.3	8000	7546	352.8	10180

			7547	352.3	7990	7547	352.7	10190
			7548	352.3	8000	7548	352.7	10180
			7549	352.3	8000	7549	352.7	10180
			7550	352.3	7980	7550	352.8	10180
			7551	352.3	7990	7551	352.8	10180
			7552	352.2	8000	7552	352.7	10180
			7553	352.3	7980	7553	352.7	10180
			7554	352.3	7980	7554	352.6	10180
			7555	352.3	8000	7555	352.7	10190
			7556	352.3	7990	7556	352.6	10190
			7557	352.3	7990	7557	352.6	10190
			7558	352.3	7980	7558	352.6	10190
			7559	352.3	7980	7559	352.7	10190
			7560	352.2	7990	7560	352.7	10180
			7561	352.1	7990	7561	352.7	10180
			7562	352.1	7980	7562	352.7	10180
			7563	352.1	7980	7563	352.7	10200
			7564	352.1	7980	7564	352.7	10190
			7565	352	7990	7565	352.7	10190
			7566	352	7980	7566	352.7	10200
			7567	352	7990	7567	352.7	10180
			7568	352	7960	7568	352.7	10180
			7569	352	7960	7569	352.7	10200
			7570	352	7970	7570	352.7	10190
			7571	352	7980	7571	352.7	10190
			7572	352	7970	7572	352.7	10190
			7573	352	7960	7573	352.7	10190
			7574	352	7970	7574	352.7	10180
			7575	352	7980	7575	352.6	10190
			7576	352	7960	7576	352.7	10190
			7577	351.9	7970	7577	352.7	10200
			7578	352	7970	7578	352.7	10190
			7579	352	7970	7579	352.7	10200
			7580	352	7970	7580	352.7	10200
			7581	351.9	7970	7581	352.8	10190
			7582	351.9	7980	7582	352.8	10200
			7583	351.9	7980	7583	352.8	10190
			7584	352	7980	7584	352.7	10180
			7585	351.9	7980	7585	352.6	10190
			7586	352	7970	7586	352.6	10090
			7587	352	7980	7587	352.6	10190
			7588	352	7980	7588	352.7	10190
			7589	352	7970	7589	352.7	10180
			7590	351.9	7970	7590	352.7	10100
			7591	352	7970	7591	352.7	10100
			7592	352	7980	7592	352.7	10190
			7593	352	7970	7593	352.7	10080
			7594	352	7980	7594	352.6	10090
			7595	351.9	7970	7595	352.6	10180
			7596	351.9	7960	7596	352.6	10080

			7597	351.9	7970	7597	352.6	10180
			7598	351.9	7980	7598	352.6	10090
			7599	351.9	7970	7599	352.6	10190
			7600	351.9	7980	7600	352.5	10090
			7601	351.9	7980	7601	352.5	10080
			7602	351.9	7970	7602	352.5	10100
			7603	351.9	7970	7603	352.6	10080
			7604	351.9	7970	7604	352.6	10100
			7605	351.9	7970	7605	352.6	10080
			7606	351.9	7990	7606	352.6	10200
			7607	351.9	7980	7607	352.6	10100
			7608	351.8	7980	7608	352.6	10090
			7609	351.8	7970	7609	352.6	10100
			7610	351.8	7970	7610	352.5	10090
			7611	351.8	7970	7611	352.4	10000
			7612	351.7	7980			
			7613	351.7	7980			
			7614	351.7	7970			
			7615	351.7	7970			
			7616	351.8	7970			
			7617	351.8	7980			
			7618	351.8	7960			
			7619	351.8	7980			
			7620	351.8	7980			
			7621	351.7	7970			
			7622	351.7	7970			
			7623	351.7	7970			
			7624	351.7	7960			
			7625	351.8	7980			
			7626	351.8	7980			
			7627	351.7	7970			
			7628	351.7	7970			
			7629	351.7	7980			
			7630	351.7	7970			
			7631	351.7	7980			
			7632	351.7	7990			
			7633	351.7	7980			
			7634	351.7	7990			
			7635	351.6	7980			
			7636	351.7	7990			
			7637	351.7	7970			
			7638	351.8	7980			
			7639	351.8	7990			
			7640	351.8	7980			
			7641	351.8	7980			
			7642	351.8	7960			
			7643	351.8	7970			

Data: Fig 5. PMs deposition and residuals in DPF with different coating and loading contents. (A) DPF 1#, (B) DPF 2#, (C) DPF 3#.							
			1	2	3	Mean	SD
DPF1	PM after regeneration	1	1.06	0.98	1.06	1.03	0.05
		2	1.37	1.26	1.27	1.30	0.06
		3	1.4	1.48	1.37	1.42	0.06
	Deposited PMs	1	15.8	15.75	15.95	15.83	0.10
		2	14.01	14.6	14.9	14.50	0.45
		3	14.5	14.8	14.8	14.70	0.17
	Cumulative PMs Mass	1	0.96	1.03	1.05	1.01	0.05
		2	2.23	2.39	2.41	2.34	0.10
		3	4.72	4.68	4.69	4.70	0.02
DPF2	PM after regeneration	1	0.85	0.91	0.95	0.90	0.05
		2	0.95	1.07	0.99	1.00	0.06
		3	0.88	0.88	0.96	0.91	0.05
	Deposited PMs	1	16.4	16.48	16.58	16.49	0.09
		2	16.7	16.6	17	16.77	0.21
		3	17.05	17	16.89	16.98	0.08
	Cumulative PMs Mass	1	0.92	0.85	0.92	0.90	0.04
		2	1.87	1.82	1.98	1.89	0.08
		3	2.79	2.82	2.86	2.82	0.04
DPF3	PM after regeneration	1	0.54	0.45	0.5	0.50	0.05
		2	0.52	0.63	0.63	0.59	0.06
		3	0.64	0.56	0.61	0.60	0.04
	Deposited PMs	1	15.65	15.42	15.68	15.58	0.14
		2	12.96	12.59	12.95	12.83	0.21
		3	14.2	14.11	14.31	14.21	0.10
	Cumulative PMs Mass	1	0.48	0.54	0.53	0.52	0.03
		2	1.06	1.23	1.08	1.12	0.09
		3	1.71	1.68	1.68	1.69	0.02

Data: Fig 6. DPF BPT tests in pilot-scale bench.					
Time(s)	Up-Temp.(℃)	Mid-Temp.(℃)	Down-Temp.(℃)	△P(Pa)	Load(%)
12/4/2020 10:04	78.1	43.3	47	96	0.58
12/4/2020 10:04	78.1	43.3	46.8	97	0.75
12/4/2020 10:04	78.2	43.3	46.8	97	0.97
12/4/2020 10:04	78.2	43.3	47.7	97	1.19
12/4/2020 10:04	78.2	43.4	47.5	97	1.44
12/4/2020 10:04	78.3	43.3	47.3	97	1.64
12/4/2020 10:04	78.2	43.4	47.3	97	1.96
12/4/2020 10:04	78.3	43.3	47.3	97	2.17
12/4/2020 10:04	78.4	43.3	47.1	97	2.52
12/4/2020 10:04	78.3	43.2	46.9	97	2.78
12/4/2020 10:04	78.4	43.3	47.2	97	3.02
12/4/2020 10:04	78.5	43.1	47.9	98	3.29
12/4/2020 10:04	78.5	43.3	47.6	97	3.56
12/4/2020 10:04	78.5	43.2	47.4	98	3.86
12/4/2020 10:04	78.5	43.3	47.3	98	4.14
12/4/2020 10:04	78.6	43.4	47.4	98	4.37
12/4/2020 10:04	78.7	43.3	47.5	99	4.61
12/4/2020 10:04	78.7	43.3	47.3	99	4.91
12/4/2020 10:04	78.7	43.4	47.3	100	5.19
12/4/2020 10:04	78.8	43.3	47.7	98	5.44
12/4/2020 10:04	78.8	43.3	48.1	100	5.66
12/4/2020 10:04	78.8	44.5	47.5	101	5.91
12/4/2020 10:04	78.9	44.6	47.5	101	6.14
12/4/2020 10:04	78.9	44.1	47.6	101	6.38
12/4/2020 10:04	79	44.4	47.7	102	6.59
12/4/2020 10:04	79.1	44.5	47.5	102	6.81
12/4/2020 10:04	79.1	44.3	47.4	103	7.04
12/4/2020 10:04	79.2	44.1	48	102	7.28
12/4/2020 10:04	79.3	44.5	48.3	103	7.49
12/4/2020 10:04	79.3	44.1	47.9	104	7.71
12/4/2020 10:04	79.4	44.5	47.8	105	7.92
12/4/2020 10:04	79.5	44.2	47.8	106	8.13
12/4/2020 10:04	79.5	44.2	47.9	107	8.35
12/4/2020 10:04	79.7	44.5	48.1	107	8.56
12/4/2020 10:04	79.7	44.2	47.8	108	8.78
12/4/2020 10:04	79.8	44.3	47.8	108	8.98
12/4/2020 10:04	79.9	44.2	48.5	108	9.17
12/4/2020 10:05	79.9	44.2	48.4	109	9.35
12/4/2020 10:05	80.1	44.4	47.9	109	9.5
12/4/2020 10:05	80	44.2	48	109	9.64
12/4/2020 10:05	80.2	44.1	48.1	110	9.73
12/4/2020 10:05	80.4	44.2	48.2	111	9.85
12/4/2020 10:05	80.5	44.2	48.1	111	9.92
12/4/2020 10:05	80.6	44.3	48	111	10.01
12/4/2020 10:05	80.6	44.6	48.2	111	10.06

12/4/2020 10:05	80.8	44.1	48.9	112	10.09
12/4/2020 10:05	80.9	44.4	48.8	113	10.15
12/4/2020 10:05	81	44.3	48.4	113	10.16
12/4/2020 10:05	81.2	44.1	48.5	112	10.19
12/4/2020 10:05	81.2	45.4	48.5	112	10.19
12/4/2020 10:05	81.5	45.6	48.7	114	10.25
12/4/2020 10:05	81.6	45	48.8	113	10.3
12/4/2020 10:05	81.7	45.7	48.7	115	10.29
12/4/2020 10:05	81.8	45.1	48.4	115	10.29
12/4/2020 10:05	81.9	45.2	48.8	114	10.31
12/4/2020 10:05	82.1	45.7	49.5	114	10.3
12/4/2020 10:05	82.3	45.1	49.2	115	10.33
12/4/2020 10:05	82.2	45.4	48.9	114	10.36
12/4/2020 10:05	82.5	45	49.1	114	10.33
12/4/2020 10:05	82.6	45.4	49	116	10.33
12/4/2020 10:05	82.7	45.7	49	115	10.34
12/4/2020 10:05	83	45.4	48.8	115	10.32
12/4/2020 10:05	83.1	45.7	49	115	10.32
12/4/2020 10:05	83.2	45.7	49.7	115	10.35
12/4/2020 10:05	83.4	45.2	49.9	115	10.34
12/4/2020 10:05	83.4	45.3	49.5	116	10.34
12/4/2020 10:05	83.8	45.5	49.4	115	10.31
12/4/2020 10:05	83.7	45	49.4	116	10.33
12/4/2020 10:05	84	45.9	49.6	116	10.37
12/4/2020 10:05	84.2	45.2	49.5	115	10.34
12/4/2020 10:05	84.7	45.2	49.7	116	10.31
12/4/2020 10:05	84.7	45.8	49.5	116	10.34
12/4/2020 10:05	84.8	45.2	49.5	115	10.34
12/4/2020 10:05	84.9	46.1	50.2	115	10.37
12/4/2020 10:05	85	46.8	50.6	116	10.33
12/4/2020 10:05	85.5	46.2	50.1	116	10.32
12/4/2020 10:05	85.5	46.5	50	116	10.34
12/4/2020 10:05	85.6	46.2	49.9	116	10.35
12/4/2020 10:05	85.8	46.4	50.3	116	10.34
12/4/2020 10:05	85.9	46.8	50.1	116	10.33
12/4/2020 10:05	86	46.6	50.4	116	10.34
12/4/2020 10:05	86.2	46.7	50.4	116	10.34
12/4/2020 10:05	86.2	46.7	50.5	116	10.34
12/4/2020 10:05	86.4	46	50.3	116	10.34
12/4/2020 10:05	86.5	46.2	50.4	116	10.37
12/4/2020 10:05	87	46.8	50.4	116	10.32
12/4/2020 10:05	87.1	46.5	50.4	118	10.32
12/4/2020 10:05	87.1	46.6	50.7	116	10.33
12/4/2020 10:05	87.2	46.4	50.7	116	10.34
12/4/2020 10:05	87.5	46.1	50.9	117	10.33
12/4/2020 10:05	87.5	46.7	51.3	116	10.34
12/4/2020 10:05	88	46.2	51.5	118	10.32

12/4/2020 10:05	88	46.4	51.5	117	10.35
12/4/2020 10:05	88.2	46.6	51.8	118	10.35
12/4/2020 10:05	88.4	46.3	51.8	116	10.33
12/4/2020 10:05	88.3	46.7	52	118	10.33
12/4/2020 10:06	88.8	47.4	52.1	117	10.32
12/4/2020 10:06	88.9	47.4	51.8	118	10.32
12/4/2020 10:06	88.9	47.3	51.9	118	10.32
12/4/2020 10:06	89.2	47.5	51.8	118	10.34
12/4/2020 10:06	89.1	47.3	51.3	116	10.34
12/4/2020 10:06	89.7	47.5	51.5	118	10.34
12/4/2020 10:06	89.7	47.3	51.5	118	10.32
12/4/2020 10:06	89.8	47.3	51.6	118	10.32
12/4/2020 10:06	90	47.6	52	116	10.34
12/4/2020 10:06	90	47.3	51.8	117	10.32
12/4/2020 10:06	90.5	47.4	52	117	10.34
12/4/2020 10:06	90.6	47.5	52.1	116	10.32
12/4/2020 10:06	90.6	47.3	52.3	118	10.32
12/4/2020 10:06	90.8	47.6	53	118	10.33
12/4/2020 10:06	90.8	47.3	51.9	117	10.32
12/4/2020 10:06	91.2	47.4	52	118	10.35
12/4/2020 10:06	91.3	47.5	52.3	118	10.35
12/4/2020 10:06	91.7	47.3	52.5	119	10.32
12/4/2020 10:06	91.8	47.3	52.3	118	10.35
12/4/2020 10:06	91.8	47.3	53.1	118	10.34
12/4/2020 10:06	91.8	47.4	52.7	118	10.34
12/4/2020 10:06	92.1	48.5	52.3	119	10.33
12/4/2020 10:06	92.1	48.7	53	118	10.33
12/4/2020 10:06	92.3	48.7	53	118	10.34
12/4/2020 10:06	92.5	48.4	52.9	118	10.33
12/4/2020 10:06	92.9	48.7	53	118	10.35
12/4/2020 10:06	93.1	48.5	53.2	119	10.32
12/4/2020 10:06	93	48.5	53.6	119	10.32
12/4/2020 10:06	93.1	48.7	53.3	118	10.34
12/4/2020 10:06	93.4	48.5	52.9	119	10.35
12/4/2020 10:06	93.4	48.8	53.5	119	10.35
12/4/2020 10:06	93.6	48.6	53.4	118	10.35
12/4/2020 10:06	93.6	48.5	53.4	119	10.34
12/4/2020 10:06	93.8	48.5	53.5	119	10.35
12/4/2020 10:06	94	48.8	53.6	119	10.32
12/4/2020 10:06	94.5	48.7	53.8	119	10.35
12/4/2020 10:06	94.7	48.6	53.8	118	10.35
12/4/2020 10:06	94.6	48.7	54	119	10.33
12/4/2020 10:06	94.7	48.6	53.6	119	10.33
12/4/2020 10:06	94.8	48.7	53.9	119	10.34
12/4/2020 10:06	95	48.6	53.9	119	10.35
12/4/2020 10:06	95.1	49.2	53.9	120	10.33
12/4/2020 10:06	95.3	49.4	54.1	119	10.33

12/4/2020 10:06	95.7	49.2	54.1	119	10.32
12/4/2020 10:06	95.7	49.4	54.2	119	10.33
12/4/2020 10:06	95.8	49.8	54.4	118	10.32
12/4/2020 10:06	95.9	49.4	54.3	119	10.35
12/4/2020 10:06	96	49.4	54.4	119	10.33
12/4/2020 10:06	96.5	49.6	54.7	118	10.31
12/4/2020 10:06	96.6	49.6	54.4	119	10.34
12/4/2020 10:06	96.6	49.8	54.4	119	10.33
12/4/2020 10:06	96.7	49.7	54.6	118	10.32
12/4/2020 10:06	96.8	49.9	55.5	119	10.34
12/4/2020 10:06	96.9	49.2	55.2	119	10.34
12/4/2020 10:06	97.2	49.2	54.9	119	10.33
12/4/2020 10:06	97.1	49.4	54.8	118	10.33
12/4/2020 10:06	97.5	49.7	55.3	119	10.35
12/4/2020 10:06	97.5	49.1	55.3	119	10.32
12/4/2020 10:06	97.7	49.5	55.1	118	10.33
12/4/2020 10:06	97.9	50.8	55	119	10.35
12/4/2020 10:07	98	50.5	55.2	119	10.32
12/4/2020 10:07	98.5	50.6	55.9	119	10.32
12/4/2020 10:07	98.5	50.1	56	120	10.35
12/4/2020 10:07	98.5	50.8	55.6	119	10.32
12/4/2020 10:07	98.8	50.6	55.7	119	10.32
12/4/2020 10:07	98.8	50.4	55.8	119	10.32
12/4/2020 10:07	99	50.2	55.6	120	10.35
12/4/2020 10:07	99.1	50.9	55.9	119	10.34
12/4/2020 10:07	99.2	50.3	55.8	119	10.35
12/4/2020 10:07	99.3	50.9	55.3	119	10.32
12/4/2020 10:07	99.4	50.6	56.2	119	10.32
12/4/2020 10:07	99.6	50.2	56.6	119	10.32
12/4/2020 10:07	99.8	50.5	56.3	119	10.35
12/4/2020 10:07	99.9	50.2	56.1	119	10.33
12/4/2020 10:07	99.9	50.4	56.4	120	10.33
12/4/2020 10:07	100.1	50.8	56.3	119	10.34
12/4/2020 10:07	100.2	50.2	56.3	119	10.35
12/4/2020 10:07	100.4	51.7	56.2	119	10.31
12/4/2020 10:07	100.5	51.8	56.3	119	10.34
12/4/2020 10:07	100.6	51.3	57	120	10.33
12/4/2020 10:07	100.7	51.4	56.8	119	10.32
12/4/2020 10:07	100.8	51.3	56.6	120	10.33
12/4/2020 10:07	101	51.6	56.8	119	10.33
12/4/2020 10:07	101.1	51.8	56.6	119	10.33
12/4/2020 10:07	101.4	51.5	56.8	120	10.34
12/4/2020 10:07	101.5	51.8	56.7	119	10.35
12/4/2020 10:07	101.6	51.8	56.8	120	10.29
12/4/2020 10:07	101.8	51.2	57.7	119	10.33
12/4/2020 10:07	101.8	51.4	57.3	119	10.32
12/4/2020 10:07	102.1	51.2	57.1	119	10.32

12/4/2020 10:07	102.1	51.3	57.2	119	10.32
12/4/2020 10:07	102.3	51.6	57.4	120	10.33
12/4/2020 10:07	102.5	51.5	57.4	120	10.29
12/4/2020 10:07	102.5	51.4	57.2	119	10.35
12/4/2020 10:07	102.8	51.7	57.1	120	10.35
12/4/2020 10:07	102.9	52.6	57.5	119	10.32
12/4/2020 10:07	102.9	52.6	58.3	120	10.33
12/4/2020 10:07	103.2	52.5	57.9	119	10.34
12/4/2020 10:07	103.2	52.5	57.5	119	10.34
12/4/2020 10:07	103.4	52.6	57.7	120	10.33
12/4/2020 10:07	103.5	52.7	57.8	119	10.34
12/4/2020 10:07	103.7	52.5	57.9	119	10.35
12/4/2020 10:07	103.7	52.6	57.9	119	10.33
12/4/2020 10:07	103.8	52.6	57.8	119	10.32
12/4/2020 10:07	104	52.5	57.9	120	10.33
12/4/2020 10:07	104.1	52.6	58.6	119	10.35
12/4/2020 10:07	104.2	52.5	58.7	120	10.32
12/4/2020 10:07	104.4	52.6	58.2	119	10.35
12/4/2020 10:07	104.4	52.7	58.2	120	10.36
12/4/2020 10:07	104.6	52.6	58.4	120	10.33
12/4/2020 10:07	104.8	52.5	58.4	122	10.35
12/4/2020 10:07	104.9	52.5	58.5	120	10.33
12/4/2020 10:07	105.1	53.8	58.4	120	10.34
12/4/2020 10:07	105.2	53.4	58.5	120	10.32
12/4/2020 10:07	105.3	53.6	58.4	119	10.32
12/4/2020 10:07	105.5	53.7	58.5	119	10.35
12/4/2020 10:07	105.5	53.4	58.6	119	10.36
12/4/2020 10:07	105.7	53.6	58.8	120	10.33
12/4/2020 10:07	105.8	53.7	59.2	119	10.34
12/4/2020 10:08	105.9	53.8	59.3	119	10.33
12/4/2020 10:08	106.2	53.8	59.4	119	10.35
12/4/2020 10:08	106.2	53.6	59.5	119	10.33
12/4/2020 10:08	106.4	53.6	59.6	120	10.32
12/4/2020 10:08	106.4	53.9	59.5	120	10.35
12/4/2020 10:08	106.6	53.5	59.2	120	10.33
12/4/2020 10:08	106.7	53.6	59.1	120	10.32
12/4/2020 10:08	106.8	53.4	59	120	10.32
12/4/2020 10:08	107	53.4	59.1	120	10.33
12/4/2020 10:08	107.1	53.6	59.2	120	10.34
12/4/2020 10:08	107.2	54.7	59.5	119	10.34
12/4/2020 10:08	107.4	54.3	59.3	120	10.33
12/4/2020 10:08	107.4	54.9	59.8	120	10.36
12/4/2020 10:08	107.6	54.9	59.6	120	10.35
12/4/2020 10:08	107.7	54.1	59.5	120	10.33
12/4/2020 10:08	107.8	55	60	121	10.33
12/4/2020 10:08	107.9	54.7	60.3	120	10.33
12/4/2020 10:08	108	54.7	59.8	119	10.33

12/4/2020 10:08	108.1	54.9	59.8	119	10.33
12/4/2020 10:08	108.4	54.4	60	120	10.33
12/4/2020 10:08	108.4	54.8	60.2	120	10.32
12/4/2020 10:08	108.6	55	60.3	121	10.36
12/4/2020 10:08	108.7	54.7	60.5	121	10.34
12/4/2020 10:08	108.8	54.7	60.6	120	10.35
12/4/2020 10:08	109	54.4	60	121	10.29
12/4/2020 10:08	109	54.3	60	121	10.33
12/4/2020 10:08	109.2	55.9	60.5	121	10.32
12/4/2020 10:08	109.3	55.4	60.3	120	10.32
12/4/2020 10:08	109.4	56	60.7	120	10.34
12/4/2020 10:08	109.6	55.8	61	122	10.35
12/4/2020 10:08	109.6	55.2	61.4	122	10.34
12/4/2020 10:08	109.9	55.5	60.4	122	10.32
12/4/2020 10:08	109.9	55.2	60.3	121	10.32
12/4/2020 10:08	110.1	55.8	60.5	120	10.38
12/4/2020 10:08	110.2	55.9	60.8	120	10.29
12/4/2020 10:08	110.2	55.5	61.1	122	10.33
12/4/2020 10:08	110.5	55.8	61.2	122	10.32
12/4/2020 10:08	110.5	55.3	61.1	122	10.31
12/4/2020 10:08	110.6	55.3	61.1	122	10.32
12/4/2020 10:08	110.7	55.3	60.8	119	10.32
12/4/2020 10:08	110.8	55.1	61	122	10.34
12/4/2020 10:08	111	56.5	61.1	122	10.33
12/4/2020 10:08	111.1	56.7	61.4	122	10.33
12/4/2020 10:08	111.2	56.6	61.8	122	10.34
12/4/2020 10:08	111.3	56.6	61.8	122	10.35
12/4/2020 10:08	111.3	56.8	62	122	10.35
12/4/2020 10:08	111.4	56.4	62.3	122	10.33
12/4/2020 10:08	111.6	56.3	62.3	122	10.32
12/4/2020 10:08	111.7	56.8	62.2	122	10.33
12/4/2020 10:08	111.9	56.6	62.4	120	10.35
12/4/2020 10:08	111.9	56.4	62.3	122	10.33
12/4/2020 10:08	112.1	56.8	62.4	122	10.32
12/4/2020 10:08	112.3	56.5	62.3	120	10.32
12/4/2020 10:08	112.3	56.8	62.3	122	10.35
12/4/2020 10:08	112.4	56.3	62.4	122	10.33
12/4/2020 10:08	112.5	56.3	62.4	122	10.38
12/4/2020 10:08	112.7	56.5	62.2	121	10.34
12/4/2020 10:08	112.8	56.3	62.3	122	10.34
12/4/2020 10:08	112.9	57.6	62.1	122	10.33
12/4/2020 10:08	113.1	57.5	62.1	122	10.32
12/4/2020 10:09	113.1	57.6	62.5	123	10.33
12/4/2020 10:09	113.1	57.6	62.3	122	10.33
12/4/2020 10:09	113.4	57.5	62.3	123	10.33
12/4/2020 10:09	113.4	57.5	62.4	122	10.32
12/4/2020 10:09	113.6	57.6	62.2	122	10.33

12/4/2020 10:09	113.7	57.5	62.3	122	10.33
12/4/2020 10:09	113.7	57.6	62.4	122	10.33
12/4/2020 10:09	113.8	57.5	62.4	122	10.32
12/4/2020 10:09	113.9	57.6	63.1	125	10.33
12/4/2020 10:09	114	57.5	63.3	122	10.32
12/4/2020 10:09	114.2	57.5	63	122	10.35
12/4/2020 10:09	114.2	57.5	62.9	122	10.35
12/4/2020 10:09	114.3	57.6	62.9	122	10.31
12/4/2020 10:09	114.4	58.9	63.1	122	10.35
12/4/2020 10:09	114.5	58.6	63.2	122	10.36
12/4/2020 10:09	114.6	58.8	62.9	123	10.33
12/4/2020 10:09	114.6	58.4	63.1	122	10.34
12/4/2020 10:09	114.8	58.8	63.2	122	10.32
12/4/2020 10:09	114.8	58.4	63	122	10.33
12/4/2020 10:09	115	58.9	63.4	122	10.34
12/4/2020 10:09	115	58.9	63.3	123	10.34
12/4/2020 10:09	115.1	58.4	63	122	10.32
12/4/2020 10:09	115.3	58.6	63.4	122	10.33
12/4/2020 10:09	115.4	58.8	63.3	123	10.33
12/4/2020 10:09	115.5	58.5	63.1	123	10.28
12/4/2020 10:09	115.7	58.9	63.3	123	10.32
12/4/2020 10:09	115.7	58.6	63.5	122	10.32
12/4/2020 10:09	115.8	58.4	63.5	122	10.31
12/4/2020 10:09	115.9	59.8	63.9	122	10.34
12/4/2020 10:09	116	59.3	64	123	10.31
12/4/2020 10:09	116.1	59.5	64.3	123	10.33
12/4/2020 10:09	116.2	59.2	64.3	122	10.29
12/4/2020 10:09	116.4	59.6	64.4	122	10.29
12/4/2020 10:09	116.5	59.8	64.7	122	10.31
12/4/2020 10:09	116.6	59.7	64.7	122	10.33
12/4/2020 10:09	116.7	59.3	64.5	123	10.32
12/4/2020 10:09	116.8	60	64.5	122	10.31
12/4/2020 10:09	116.9	59.3	64.5	123	10.32
12/4/2020 10:09	117	59.5	64.3	122	10.31
12/4/2020 10:09	117	60.2	64.2	122	10.34
12/4/2020 10:09	117.2	59.8	64	122	10.32
12/4/2020 10:09	117.2	59.8	63.9	123	10.32
12/4/2020 10:09	117.3	59.5	64.3	122	10.33
12/4/2020 10:09	117.4	60	64.2	123	10.31
12/4/2020 10:09	117.5	60.7	64.3	123	10.34
12/4/2020 10:09	117.6	60.7	64.5	122	10.31
12/4/2020 10:09	117.7	60.4	64.7	122	10.28
12/4/2020 10:09	117.8	61.1	64.5	123	10.33
12/4/2020 10:09	117.9	60.7	64.9	122	10.34
12/4/2020 10:09	118	61	64.7	123	10.31
12/4/2020 10:09	118.1	60.9	64.7	124	10.31
12/4/2020 10:09	118.1	60.5	64.8	123	10.31

12/4/2020 10:09	118.2	60.9	64.7	123	10.33
12/4/2020 10:09	118.3	60.4	65.5	123	10.31
12/4/2020 10:09	118.4	60.7	65.6	123	10.31
12/4/2020 10:09	118.5	61	64.9	123	10.32
12/4/2020 10:09	118.6	60.9	64.7	122	10.38
12/4/2020 10:09	118.7	60.5	64.9	124	10.31
12/4/2020 10:09	118.8	60.3	65.3	123	10.32
12/4/2020 10:10	118.8	61.5	65.2	124	10.28
12/4/2020 10:10	118.9	61.5	65.4	124	10.34
12/4/2020 10:10	119.1	61.5	65.4	123	10.3
12/4/2020 10:10	119.2	61.5	65.4	122	10.34
12/4/2020 10:10	119.4	61.5	65.9	122	10.31
12/4/2020 10:10	119.4	61.4	66.1	123	10.29
12/4/2020 10:10	119.5	61.6	65.5	123	10.34
12/4/2020 10:10	119.6	61.6	65.2	123	10.31
12/4/2020 10:10	119.6	61.5	65.5	123	10.31
12/4/2020 10:10	119.8	61.7	65.4	124	10.31
12/4/2020 10:10	119.8	61.7	65.7	123	10.32
12/4/2020 10:10	120	61.3	65.8	124	10.33
12/4/2020 10:10	120	61.5	65.7	125	10.31
12/4/2020 10:10	120	61.6	65.6	123	10.33
12/4/2020 10:10	120.2	61.7	65.9	124	10.31
12/4/2020 10:10	120.3	61.6	65.8	124	10.3
12/4/2020 10:10	120.4	62.6	65.7	123	10.31
12/4/2020 10:10	120.4	62.6	65.9	125	10.32
12/4/2020 10:10	120.5	62.4	66	123	10.3
12/4/2020 10:10	120.6	62.6	66	123	10.31
12/4/2020 10:10	120.7	62.5	66.3	123	10.3
12/4/2020 10:10	120.8	62.4	66.1	123	10.33
12/4/2020 10:10	120.9	62.4	66.2	125	10.31
12/4/2020 10:10	120.9	62.6	66.2	124	10.3
12/4/2020 10:10	121.1	62.6	66.3	125	10.27
12/4/2020 10:10	121.1	62.6	66.3	125	10.34
12/4/2020 10:10	121.1	62.6	66.2	125	10.34
12/4/2020 10:10	121.4	62.7	66.3	125	10.33
12/4/2020 10:10	121.3	62.6	66.4	124	10.31
12/4/2020 10:10	121.4	62.5	66.4	124	10.31
12/4/2020 10:10	121.5	62.7	66.4	125	10.33
12/4/2020 10:10	121.6	63.3	66.4	125	10.31
12/4/2020 10:10	121.7	63.6	66.3	125	10.3
12/4/2020 10:10	121.7	63.8	66.3	124	10.3
12/4/2020 10:10	121.8	63.3	66.3	125	10.32
12/4/2020 10:10	121.9	63.3	66.4	125	10.31
12/4/2020 10:10	122	63.9	66.5	125	10.3
12/4/2020 10:10	122.2	63.2	66.8	124	10.33
12/4/2020 10:10	122.1	63.6	67.1	125	10.32
12/4/2020 10:10	122.2	63.6	67.2	124	10.32

12/4/2020 10:10	122.3	63.2	67.3	125	10.31
12/4/2020 10:10	122.4	63.6	67.2	126	10.28
12/4/2020 10:10	122.5	63.6	67.1	125	10.31
12/4/2020 10:10	122.5	63.4	67.1	124	10.29
12/4/2020 10:10	122.6	63.9	67	125	10.3
12/4/2020 10:10	122.8	63.5	66.9	124	10.3
12/4/2020 10:10	122.8	64.9	67	126	10.32
12/4/2020 10:10	122.9	65	67.3	125	10.32
12/4/2020 10:10	123	64.3	67.1	126	10.33
12/4/2020 10:10	123.1	64.5	67.1	126	10.31
12/4/2020 10:10	123.3	64.7	67.4	125	10.31
12/4/2020 10:10	123.3	64.3	67.3	126	10.33
12/4/2020 10:10	123.3	64.6	67.3	124	10.28
12/4/2020 10:10	123.5	64.3	67.3	125	10.31
12/4/2020 10:10	123.5	64.2	67.5	124	10.33
12/4/2020 10:10	123.6	64.8	67.3	125	10.34
12/4/2020 10:10	123.6	64.5	67.3	124	10.33
12/4/2020 10:10	123.7	64.9	67.5	126	10.31
12/4/2020 10:10	123.9	64.2	67.3	126	10.31
12/4/2020 10:11	123.9	64.4	67.3	125	10.33
12/4/2020 10:11	124	64.7	67.6	126	10.3
12/4/2020 10:11	124	64.9	67.5	125	10.31
12/4/2020 10:11	124.1	65.3	67.6	126	10.31
12/4/2020 10:11	124.2	65.6	67.6	125	10.31
12/4/2020 10:11	124.3	65.1	67.7	125	10.31
12/4/2020 10:11	124.3	65.4	67.7	126	10.28
12/4/2020 10:11	124.4	65.6	67.6	125	10.33
12/4/2020 10:11	124.5	65.5	67.8	125	10.31
12/4/2020 10:11	124.6	65.4	67.8	126	10.31
12/4/2020 10:11	124.6	65.7	67.8	126	10.32
12/4/2020 10:11	124.7	65.3	67.8	126	10.32
12/4/2020 10:11	124.8	65.7	68.3	126	10.31
12/4/2020 10:11	124.8	65.8	68.3	126	10.32
12/4/2020 10:11	125	65.4	68.2	126	10.31
12/4/2020 10:11	125	65.7	68.3	125	10.3
12/4/2020 10:11	125.1	65.3	68.3	126	10.31
12/4/2020 10:11	125.1	65.7	68.3	126	10.29
12/4/2020 10:11	125.2	66.6	68.5	126	10.31
12/4/2020 10:11	125.2	66.6	69.1	126	10.33
12/4/2020 10:11	125.3	66.4	68.5	126	10.32
12/4/2020 10:11	125.4	66.6	68.2	126	10.32
12/4/2020 10:11	125.5	66.4	68.5	126	10.31
12/4/2020 10:11	125.5	66.6	68.6	126	10.34
12/4/2020 10:11	125.7	66.3	68.8	126	10.32
12/4/2020 10:11	125.7	66.4	68.6	127	10.32
12/4/2020 10:11	125.8	66.7	68.7	126	10.31
12/4/2020 10:11	125.9	66.5	69	126	10.32

12/4/2020 10:11	125.9	66.4	69.2	127	10.33
12/4/2020 10:11	126	66.4	68.7	126	10.31
12/4/2020 10:11	126.1	66.6	68.8	126	10.31
12/4/2020 10:11	126.1	66.6	68.7	126	10.32
12/4/2020 10:11	126.2	66.4	68.9	126	10.33
12/4/2020 10:11	126.3	67.5	69	126	10.34
12/4/2020 10:11	126.3	67.3	69.1	126	10.32
12/4/2020 10:11	126.5	67.3	69.1	126	10.34
12/4/2020 10:11	126.5	67.6	69.2	126	10.37
12/4/2020 10:11	126.6	67.4	69.4	127	10.33
12/4/2020 10:11	126.7	67.5	69.7	126	10.34
12/4/2020 10:11	126.7	67.3	69.7	127	10.34
12/4/2020 10:11	126.9	67.2	69.9	126	10.32
12/4/2020 10:11	127	67.5	69.9	126	10.32
12/4/2020 10:11	127	67.5	69.7	126	10.34
12/4/2020 10:11	127	67.6	69.9	126	10.31
12/4/2020 10:11	127.1	67.3	70	127	10.33
12/4/2020 10:11	127.2	67.2	69.8	126	10.33
12/4/2020 10:11	127.2	67.5	70.2	127	10.32
12/4/2020 10:11	127.3	67.5	70.3	127	10.31
12/4/2020 10:11	127.3	68.6	69.9	127	10.31
12/4/2020 10:11	127.4	68.6	69.9	127	10.33
12/4/2020 10:11	127.5	68.4	69.5	127	10.32
12/4/2020 10:11	127.6	68.2	69.6	127	10.34
12/4/2020 10:11	127.6	68.7	69.9	127	10.32
12/4/2020 10:11	127.6	68.2	69.9	127	10.32
12/4/2020 10:11	127.7	68.7	69.9	126	10.32
12/4/2020 10:11	127.8	68.8	69.7	128	10.34
12/4/2020 10:11	127.9	68.2	69.9	127	10.28
12/4/2020 10:11	127.9	68.6	70.8	127	10.32
12/4/2020 10:11	128	68.2	69.9	126	10.32
12/4/2020 10:11	128.1	68.2	69.8	127	10.31
12/4/2020 10:12	128.1	68.7	70.3	127	10.34
12/4/2020 10:12	128.3	68.6	70.2	126	10.32
12/4/2020 10:12	128.3	68.1	70	127	10.32
12/4/2020 10:12	128.3	68.3	70	127	10.32
12/4/2020 10:12	128.5	69.4	70.4	127	10.32
12/4/2020 10:12	128.5	69.1	70.8	127	10.32
12/4/2020 10:12	128.5	69.3	70.4	127	10.32
12/4/2020 10:12	128.5	69.4	70.6	127	10.31
12/4/2020 10:12	128.6	69.6	70.4	127	10.31
12/4/2020 10:12	128.7	69.9	70.6	127	10.32
12/4/2020 10:12	128.8	69.1	70.6	128	10.34
12/4/2020 10:12	128.9	69.6	70.5	127	10.32
12/4/2020 10:12	128.9	69.3	70.3	128	10.31
12/4/2020 10:12	128.9	69.6	70.9	127	10.32
12/4/2020 10:12	129	69.9	71.5	127	10.34

12/4/2020 10:12	129.1	69.2	70.9	128	10.32
12/4/2020 10:12	129.1	69.7	70.8	127	10.34
12/4/2020 10:12	129.2	69.3	71.1	127	10.32
12/4/2020 10:12	129.3	69.1	70.8	127	10.32
12/4/2020 10:12	129.4	70.3	70.8	127	10.28
12/4/2020 10:12	129.4	70.8	71.1	127	10.35
12/4/2020 10:12	129.6	70.4	70.7	127	10.32
12/4/2020 10:12	129.6	70.7	70.8	127	10.31
12/4/2020 10:12	129.6	70.6	70.9	128	10.34
12/4/2020 10:12	129.8	70.6	71.1	127	10.31
12/4/2020 10:12	129.8	70.6	71.5	127	10.34
12/4/2020 10:12	129.8	70.4	72	127	10.32
12/4/2020 10:12	129.9	70.6	71.8	128	10.32
12/4/2020 10:12	130	71	71.6	127	10.33
12/4/2020 10:12	130.1	70.6	71.3	127	10.33
12/4/2020 10:12	130.2	70.8	71.4	127	10.31
12/4/2020 10:12	130.2	70.6	71.6	127	10.31
12/4/2020 10:12	130.3	70.4	71.5	128	10.31
12/4/2020 10:12	130.4	70.9	71.5	127	10.29
12/4/2020 10:12	130.4	70.3	71.7	127	10.35
12/4/2020 10:12	130.4	71.6	71.6	128	10.32
12/4/2020 10:12	130.6	71.6	71.6	128	10.32
12/4/2020 10:12	130.6	71.6	71.8	129	10.35
12/4/2020 10:12	130.7	71.5	71.6	128	10.32
12/4/2020 10:12	130.7	71.5	71.4	128	10.34
12/4/2020 10:12	130.9	71.7	71.6	129	10.32
12/4/2020 10:12	130.9	71.5	72.1	129	10.32
12/4/2020 10:12	130.9	71.6	72.7	128	10.34
12/4/2020 10:12	131	71.6	72.2	129	10.31
12/4/2020 10:12	131	71.6	72	129	10.32
12/4/2020 10:12	131.1	71.5	72.1	128	10.34
12/4/2020 10:12	131.1	71.6	72.2	128	10.32
12/4/2020 10:12	131.3	71.5	72.2	129	10.32
12/4/2020 10:12	131.3	71.4	72.2	128	10.31
12/4/2020 10:12	131.3	71.6	72	129	10.34
12/4/2020 10:12	131.4	71.5	72.2	129	10.32
12/4/2020 10:12	131.5	72.5	72.4	128	10.34
12/4/2020 10:12	131.6	72.1	72.3	129	10.32
12/4/2020 10:12	131.7	72.5	72.3	129	10.32
12/4/2020 10:12	131.7	72.7	72.7	127	10.34
12/4/2020 10:12	131.9	72.5	72.6	129	10.33
12/4/2020 10:12	131.9	72.7	72.6	129	10.3
12/4/2020 10:12	131.9	72.6	72.7	129	10.31
12/4/2020 10:12	132	72.1	72.6	128	10.32
12/4/2020 10:13	132	72.6	72.7	129	10.34
12/4/2020 10:13	132.1	72.5	72.7	128	10.32
12/4/2020 10:13	132.2	72.3	72.7	128	10.31

12/4/2020 10:13	132.2	72.6	72.9	129	10.32
12/4/2020 10:13	132.3	72.3	72.6	129	10.34
12/4/2020 10:13	132.3	72.3	72.7	128	10.31
12/4/2020 10:13	132.4	72.7	73.2	128	10.32
12/4/2020 10:13	132.4	72.3	73.4	128	10.34
12/4/2020 10:13	132.5	73.4	73.2	128	10.31
12/4/2020 10:13	132.5	73.6	72.8	128	10.32
12/4/2020 10:13	132.6	73.6	72.9	129	10.33
12/4/2020 10:13	132.6	73.8	73.4	129	10.32
12/4/2020 10:13	132.7	73.6	73.4	128	10.34
12/4/2020 10:13	132.7	73.2	73.1	129	10.33
12/4/2020 10:13	132.9	73.8	73.4	128	10.31
12/4/2020 10:13	132.9	73.2	74	128	10.35
12/4/2020 10:13	132.9	73.4	73	129	10.34
12/4/2020 10:13	133	73.7	73.2	128	10.34
12/4/2020 10:13	133	73.2	73.4	127	10.31
12/4/2020 10:13	133.2	73.9	73.7	129	10.34
12/4/2020 10:13	133.2	73.4	73.6	129	10.34
12/4/2020 10:13	133.2	73.1	73.7	128	10.31
12/4/2020 10:13	133.4	73.8	74.1	129	10.32
12/4/2020 10:13	133.4	73.6	74.1	127	10.32
12/4/2020 10:13	133.5	73.6	73.4	127	10.31
12/4/2020 10:13	133.5	74.8	73.6	128	10.33
12/4/2020 10:13	133.6	74.6	73.7	127	10.31
12/4/2020 10:13	133.6	74.4	73.7	127	10.34
12/4/2020 10:13	133.7	74.4	74.1	128	10.35
12/4/2020 10:13	133.8	74.1	74	128	10.31
12/4/2020 10:13	133.9	74.5	74	128	10.31
12/4/2020 10:13	133.9	74.9	74.2	130	10.34
12/4/2020 10:13	134	74.5	73.9	129	10.35
12/4/2020 10:13	134	74.9	74.3	129	10.29
12/4/2020 10:13	134.1	74.3	74.4	128	10.32
12/4/2020 10:13	134.1	74.4	74.7	128	10.33
12/4/2020 10:13	134.2	74.8	74.9	128	10.29
12/4/2020 10:13	134.2	74.4	74.9	129	10.32
12/4/2020 10:13	134.3	74.8	74.8	129	10.34
12/4/2020 10:13	134.4	74.3	75.1	127	10.32
12/4/2020 10:13	134.4	74.4	75	128	10.33
12/4/2020 10:13	134.5	75.5	74.7	129	10.35
12/4/2020 10:13	134.5	75.4	74.7	129	10.32
12/4/2020 10:13	134.6	75.4	74.5	128	10.34
12/4/2020 10:13	134.7	75.5	74.4	128	10.34
12/4/2020 10:13	134.8	75.7	74.8	129	10.32
12/4/2020 10:13	134.8	75.5	74.6	129	10.32
12/4/2020 10:13	134.8	75.7	74.4	129	10.34
12/4/2020 10:13	134.9	75.6	74.6	129	10.32
12/4/2020 10:13	135	75.5	75.3	129	10.32

12/4/2020 10:13	135	75.6	74.8	128	10.32
12/4/2020 10:13	135	75.5	74.7	129	10.34
12/4/2020 10:13	135.2	75.5	74.9	129	10.33
12/4/2020 10:13	135.2	75.6	75	128	10.33
12/4/2020 10:13	135.2	75.3	74.7	129	10.33
12/4/2020 10:13	135.4	75.6	74.7	129	10.33
12/4/2020 10:13	135.4	75.4	75.7	129	10.32
12/4/2020 10:13	135.5	76.7	75.1	129	10.35
12/4/2020 10:13	135.5	76.5	75.1	129	10.32
12/4/2020 10:13	135.6	76.5	75.2	129	10.32
12/4/2020 10:14	135.6	76.5	75.1	129	10.33
12/4/2020 10:14	135.7	76.6	75	129	10.32
12/4/2020 10:14	135.8	76.5	75	129	10.33
12/4/2020 10:14	135.8	76.4	75.1	129	10.34
12/4/2020 10:14	135.9	76.4	75.8	129	10.32
12/4/2020 10:14	136	76.5	75.7	128	10.32
12/4/2020 10:14	135.9	76.4	75.2	129	10.34
12/4/2020 10:14	136.1	76.5	75.5	129	10.33
12/4/2020 10:14	136.1	76.5	75.5	129	10.31
12/4/2020 10:14	136.2	76.5	75.6	129	10.32
12/4/2020 10:14	136.2	76.5	75.3	129	10.34
12/4/2020 10:14	136.2	76.6	75.5	129	10.34
12/4/2020 10:14	136.3	76.4	76.3	129	10.34
12/4/2020 10:14	136.3	76.6	75.8	129	10.32
12/4/2020 10:14	136.4	77.7	75.6	129	10.32
12/4/2020 10:14	136.5	77.4	75.6	129	10.32
12/4/2020 10:14	136.5	77.5	76	129	10.33
12/4/2020 10:14	136.6	77.3	75.9	129	10.32
12/4/2020 10:14	136.7	77.2	75.8	130	10.29
12/4/2020 10:14	136.7	77.9	75.7	129	10.32
12/4/2020 10:14	136.8	77.6	75.8	129	10.31
12/4/2020 10:14	136.8	77.7	76.6	129	10.35
12/4/2020 10:14	136.9	77.8	76.2	129	10.32
12/4/2020 10:14	137	77.4	76	129	10.33
12/4/2020 10:14	137	77.3	76	129	10.32
12/4/2020 10:14	137.1	77.3	76.2	130	10.36
12/4/2020 10:14	137.2	77.2	76.2	129	10.34
12/4/2020 10:14	137.2	77.9	76.3	129	10.34
12/4/2020 10:14	137.3	77.6	76	129	10.34
12/4/2020 10:14	137.3	77.2	76.2	129	10.34
12/4/2020 10:14	137.4	78.6	76.3	129	10.32
12/4/2020 10:14	137.5	78.1	76.7	131	10.31
12/4/2020 10:14	137.5	78.2	77.1	129	10.32
12/4/2020 10:14	137.5	78.7	77.2	129	10.35
12/4/2020 10:14	137.6	78.3	77.2	129	10.32
12/4/2020 10:14	137.6	78.8	77.2	129	10.29
12/4/2020 10:14	137.7	78.7	77.4	129	10.32

12/4/2020 10:14	137.8	78.4	77.2	131	10.28
12/4/2020 10:14	137.8	78.6	76.7	129	10.34
12/4/2020 10:14	137.9	78	76.5	130	10.37
12/4/2020 10:14	137.9	78	76.5	131	10.34
12/4/2020 10:14	138	78.8	76.6	131	10.46
12/4/2020 10:14	138	78.5	76.9	132	10.51
12/4/2020 10:14	138.1	78.9	77	134	10.58
12/4/2020 10:14	138.1	78.8	76.9	134	10.62
12/4/2020 10:14	138.2	78.1	77.1	136	10.72
12/4/2020 10:14	138.2	78.1	77.8	137	10.77
12/4/2020 10:14	138.2	79.7	76.7	139	10.83
12/4/2020 10:14	138.4	79.5	77.2	140	10.91
12/4/2020 10:14	138.4	79.7	77.3	140	10.98
12/4/2020 10:14	138.5	79.3	77.3	143	11.02
12/4/2020 10:14	138.6	79	77.2	146	11.14
12/4/2020 10:14	138.5	79.7	77.9	147	11.2
12/4/2020 10:14	138.6	79.7	77.6	148	11.24
12/4/2020 10:14	138.7	79.3	77.3	151	11.39
12/4/2020 10:14	138.7	79.5	77.6	154	11.43
12/4/2020 10:14	138.8	79.5	77.6	156	11.49
12/4/2020 10:14	138.8	79.4	77.7	158	11.56
12/4/2020 10:14	138.9	79.8	77.6	160	11.69
12/4/2020 10:15	139	79.6	78.4	164	11.78
12/4/2020 10:15	139	79.8	77.9	166	11.87
12/4/2020 10:15	139	79.8	77.5	168	11.9
12/4/2020 10:15	139.1	79.6	77.9	169	11.96
12/4/2020 10:15	139.1	79.7	78.2	170	12.04
12/4/2020 10:15	139.2	80.5	78.1	173	12.12
12/4/2020 10:15	139.3	80.4	78.3	175	12.14
12/4/2020 10:15	139.3	80.5	78.2	180	12.32
12/4/2020 10:15	139.4	80.6	79	182	12.43
12/4/2020 10:15	139.5	80.3	78.9	187	12.56
12/4/2020 10:15	139.6	80.9	78.4	190	12.57
12/4/2020 10:15	139.6	80.4	78.6	194	12.66
12/4/2020 10:15	139.6	80.5	79	197	12.78
12/4/2020 10:15	139.7	80.5	78.8	201	12.81
12/4/2020 10:15	139.8	80.5	78.9	205	13.03
12/4/2020 10:15	139.9	80.5	79.1	208	13.05
12/4/2020 10:15	139.9	80.3	79.6	211	13.05
12/4/2020 10:15	140	80.6	79.8	213	13.08
12/4/2020 10:15	140	80.5	79.5	216	13.28
12/4/2020 10:15	140.1	80.5	78.9	219	13.4
12/4/2020 10:15	140.3	80.3	79.3	222	13.35
12/4/2020 10:15	140.3	81.4	79.6	225	13.52
12/4/2020 10:15	140.4	81.6	79.8	227	13.66
12/4/2020 10:15	140.5	81.3	79.7	231	13.67
12/4/2020 10:15	140.6	81.4	79.7	237	13.63

12/4/2020 10:15	140.7	81.6	79.8	239	13.75
12/4/2020 10:15	140.8	81.5	80.5	245	13.93
12/4/2020 10:15	141	81.4	80.5	248	14.21
12/4/2020 10:15	141.1	81.7	80.1	250	14.04
12/4/2020 10:15	141.1	81.5	80.3	252	14.15
12/4/2020 10:15	141.2	81.4	80.4	254	14.44
12/4/2020 10:15	141.3	81.4	80.9	253	14.34
12/4/2020 10:15	141.5	81.3	80.7	254	14.39
12/4/2020 10:15	141.5	81.4	80.6	254	14.62
12/4/2020 10:15	141.6	81.6	81.1	258	14.49
12/4/2020 10:15	141.6	81.5	81.7	260	14.68
12/4/2020 10:15	141.8	81.6	81.4	263	14.67
12/4/2020 10:15	141.9	82.6	81.3	265	14.84
12/4/2020 10:15	142.1	82.8	81	267	15.01
12/4/2020 10:15	142.2	83	81.3	270	15.05
12/4/2020 10:15	142.2	82.4	81.8	271	15.02
12/4/2020 10:15	142.4	82.3	81.8	273	15.06
12/4/2020 10:15	142.5	82.1	81.9	276	15.04
12/4/2020 10:15	142.7	82.4	82.1	277	15.13
12/4/2020 10:15	142.8	83	82.8	277	15.2
12/4/2020 10:15	142.8	82.6	82	278	15.04
12/4/2020 10:15	143	82.3	82.3	280	15.15
12/4/2020 10:15	143.1	82.5	82.7	281	15.15
12/4/2020 10:15	143.3	82.3	82.8	282	15.15
12/4/2020 10:15	143.4	82.7	82.8	284	15.32
12/4/2020 10:15	143.5	82.8	82.8	286	15.28
12/4/2020 10:15	143.7	83.5	83	286	15.46
12/4/2020 10:15	143.8	83.4	83.6	288	15.27
12/4/2020 10:15	144.1	83.3	84	286	15.18
12/4/2020 10:15	144.1	83.3	83.7	288	15.47
12/4/2020 10:15	144.3	83.4	83.3	289	15.48
12/4/2020 10:15	144.5	83.7	83.5	289	15.34
12/4/2020 10:15	144.6	83.3	83.6	289	15.23
12/4/2020 10:15	144.8	83.7	83.8	292	15.48
12/4/2020 10:16	144.7	83.3	84.1	292	15.49
12/4/2020 10:16	145	83.5	84.3	292	15.29
12/4/2020 10:16	145.2	83.8	84.3	292	15.43
12/4/2020 10:16	145.6	83.5	84.4	296	15.43
12/4/2020 10:16	145.6	83.3	84.8	297	15.46
12/4/2020 10:16	145.7	84.6	84.7	296	15.38
12/4/2020 10:16	145.8	84.4	84.9	296	15.32
12/4/2020 10:16	146	84.7	84.9	297	15.38
12/4/2020 10:16	146.1	84.7	85.2	297	15.45
12/4/2020 10:16	146.3	84.5	86.1	297	15.38
12/4/2020 10:16	146.3	84.6	85.7	296	15.51
12/4/2020 10:16	146.8	84.5	85.8	298	15.47
12/4/2020 10:16	146.9	84.3	85.7	297	15.55

12/4/2020 10:16	146.9	84.7	86.3	296	15.46
12/4/2020 10:16	147.1	84.4	86.4	297	15.47
12/4/2020 10:16	147.2	84.4	86.1	299	15.49
12/4/2020 10:16	147.6	84.8	87	298	15.55
12/4/2020 10:16	147.8	85.4	87	297	15.61
12/4/2020 10:16	147.8	85.4	86.9	299	15.76
12/4/2020 10:16	148	85.4	87.1	299	16.09
12/4/2020 10:16	148	85.6	87.1	299	16.21
12/4/2020 10:16	148.5	85.4	87.3	299	16.46
12/4/2020 10:16	148.6	85.5	87.3	301	17.06
12/4/2020 10:16	148.6	85.5	87.3	301	17.2
12/4/2020 10:16	148.8	85.4	88.3	302	17.74
12/4/2020 10:16	148.9	85.5	88.3	304	18.12
12/4/2020 10:16	149.4	85.7	88	306	18.59
12/4/2020 10:16	149.5	85.4	88.3	306	18.93
12/4/2020 10:16	149.5	86.8	88.4	310	19.58
12/4/2020 10:16	149.7	86.4	88.5	310	20.22
12/4/2020 10:16	149.8	86.3	88.6	313	20.75
12/4/2020 10:16	150.2	86.7	88.8	316	21.26
12/4/2020 10:16	150.3	86.8	89.4	317	21.9
12/4/2020 10:16	150.3	86.4	89.8	321	22.78
12/4/2020 10:16	150.5	86.8	89.3	323	23.34
12/4/2020 10:16	150.6	86.5	89.3	325	23.74
12/4/2020 10:16	151.3	86.2	89.7	329	24.31
12/4/2020 10:16	151.3	86.8	89.9	332	25.01
12/4/2020 10:16	151.4	86.4	89.9	335	25.62
12/4/2020 10:16	151.5	87.7	90.2	339	26.06
12/4/2020 10:16	152	88	90.8	344	26.7
12/4/2020 10:16	152.1	87.5	91.1	347	27.37
12/4/2020 10:16	152.2	87.8	91	350	27.78
12/4/2020 10:16	152.4	87.9	91.2	355	28.45
12/4/2020 10:16	153	87.5	91.3	359	29.12
12/4/2020 10:16	153.1	87.7	91.6	364	29.74
12/4/2020 10:16	153.1	87.5	91.7	366	30.28
12/4/2020 10:16	153.6	87.2	91.6	370	30.74
12/4/2020 10:16	153.7	87.8	92.1	375	31.38
12/4/2020 10:16	154.3	88.7	92.2	378	32.08
12/4/2020 10:16	154.4	88.5	93.1	382	32.57
12/4/2020 10:16	154.4	88.4	92.7	386	33.17
12/4/2020 10:16	155.1	88.3	92.9	390	33.83
12/4/2020 10:16	155.2	88.9	93.1	393	34.39
12/4/2020 10:16	155.7	89	93.4	398	35.03
12/4/2020 10:16	155.8	88.8	93.5	404	35.55
12/4/2020 10:16	155.8	88.6	93.9	407	36.08
12/4/2020 10:16	156.5	88.4	94	411	36.68
12/4/2020 10:16	156.6	89.5	94.3	414	37.07
12/4/2020 10:16	157.2	89.8	94.4	419	37.76

12/4/2020 10:17	157.3	89.7	94.7	425	38.27
12/4/2020 10:17	157.4	89.6	95.1	428	38.94
12/4/2020 10:17	158.2	89.8	95.1	433	39.57
12/4/2020 10:17	158.4	89.4	95.3	438	39.99
12/4/2020 10:17	159.1	89.7	95.6	442	40.57
12/4/2020 10:17	159.2	89.5	95.8	447	41.25
12/4/2020 10:17	159.2	90.5	96	451	41.87
12/4/2020 10:17	160	90.5	96.1	456	42.39
12/4/2020 10:17	160.3	90.4	96.2	460	42.97
12/4/2020 10:17	160.5	90.7	96.4	465	43.54
12/4/2020 10:17	161.1	90.5	96.5	469	43.91
12/4/2020 10:17	161.2	90.7	97	473	44.61
12/4/2020 10:17	162.1	90.6	97.4	478	45.2
12/4/2020 10:17	162.3	90.3	97.3	483	45.67
12/4/2020 10:17	163.2	91.7	97.7	488	46.38
12/4/2020 10:17	163.3	92	97.9	491	46.66
12/4/2020 10:17	163.5	91.3	98.3	497	47.61
12/4/2020 10:17	164.3	91.7	98.3	501	47.96
12/4/2020 10:17	164.5	91.2	98.7	505	48.72
12/4/2020 10:17	164.5	91.1	98.8	510	49.24
12/4/2020 10:17	165.6	91.7	99.2	514	49.71
12/4/2020 10:17	165.7	92.7	99.7	520	50.25
12/4/2020 10:17	166.7	92.2	99.8	523	50.79
12/4/2020 10:17	167.3	92.9	100	528	51.37
12/4/2020 10:17	167.3	92.5	100.5	534	51.87
12/4/2020 10:17	168	92.9	100.7	540	52.48
12/4/2020 10:17	168.4	92.9	101.1	543	52.99
12/4/2020 10:17	169.2	92.4	101.4	548	53.65
12/4/2020 10:17	169.4	93.9	101.4	554	54.19
12/4/2020 10:17	169.7	93.5	101.9	559	54.65
12/4/2020 10:17	170.5	93.4	102.2	563	55.35
12/4/2020 10:17	171	93.5	102.4	570	55.85
12/4/2020 10:17	171.7	93.4	102.7	577	56.43
12/4/2020 10:17	172.3	93.7	103	580	57.09
12/4/2020 10:17	172.4	94.6	103.5	587	57.59
12/4/2020 10:17	173.2	94.5	103.6	592	58.13
12/4/2020 10:17	173.8	94.4	104.1	597	58.72
12/4/2020 10:17	174.5	94.5	104.7	603	59.26
12/4/2020 10:17	174.6	94.4	104.7	610	59.88
12/4/2020 10:17	175.1	95.7	105.2	615	60.38
12/4/2020 10:17	175.9	95.3	105.6	620	60.88
12/4/2020 10:17	176.6	95.3	105.8	625	61.47
12/4/2020 10:17	176.6	95.2	106.3	632	61.88
12/4/2020 10:17	177.5	95.8	106.5	637	62.47
12/4/2020 10:17	178.1	95.6	106.6	642	63.13
12/4/2020 10:17	178.9	96.7	107.3	646	63.39
12/4/2020 10:17	179.6	96.7	107.3	651	63.74

12/4/2020 10:17	179.8	96.5	107.9	657	64.26
12/4/2020 10:17	180.6	96.3	108.1	661	64.28
12/4/2020 10:17	181.2	96.6	108.5	664	64.52
12/4/2020 10:17	181.9	97.7	108.5	669	64.72
12/4/2020 10:17	182.7	97.5	109.2	672	64.71
12/4/2020 10:17	182.7	97	109.8	675	64.95
12/4/2020 10:17	183.9	97.7	110.2	677	64.95
12/4/2020 10:17	184.1	97.1	110.8	678	65.07
12/4/2020 10:17	185.1	98.3	111.5	680	64.99
12/4/2020 10:17	185.9	98.3	111.8	682	65.11
12/4/2020 10:17	185.9	98.4	112.1	684	65.11
12/4/2020 10:17	187	98.6	112.5	686	65.11
12/4/2020 10:18	187.6	98.4	113	686	65.03
12/4/2020 10:18	188.4	99.4	113.2	688	65.07
12/4/2020 10:18	189	99.4	113.6	691	65.15
12/4/2020 10:18	189.1	99.5	114	693	65.12
12/4/2020 10:18	190.1	99.7	114.4	693	65.07
12/4/2020 10:18	190.8	99.5	114.6	696	65.24
12/4/2020 10:18	191.7	100.4	115.1	695	65.07
12/4/2020 10:18	191.9	100.9	115.6	696	65.12
12/4/2020 10:18	192.4	100.3	115.7	696	65.24
12/4/2020 10:18	193.3	100.4	115.9	697	65.07
12/4/2020 10:18	194	100.7	116	697	65.08
12/4/2020 10:18	194.2	101.4	116.1	699	65.09
12/4/2020 10:18	195.1	101.5	117	700	65.08
12/4/2020 10:18	195.7	101.8	117.1	704	65.04
12/4/2020 10:18	196.5	101	117.2	703	65.28
12/4/2020 10:18	197.3	102.1	117.9	704	65.13
12/4/2020 10:18	197.9	102.6	118.3	706	65.2
12/4/2020 10:18	198.3	102.3	118.6	708	65
12/4/2020 10:18	198.9	102.5	119.2	709	65.04
12/4/2020 10:18	199.7	103.5	119.6	711	65.2
12/4/2020 10:18	200.5	103.4	120.1	711	65.04
12/4/2020 10:18	200.4	103.3	120.6	711	65.03
12/4/2020 10:18	201.5	103.3	120.9	712	65.2
12/4/2020 10:18	202.1	103.1	121.4	711	65.04
12/4/2020 10:18	202.9	104.4	121.8	712	65.08
12/4/2020 10:18	203.6	104.5	122.1	714	65.08
12/4/2020 10:18	203.5	104.5	122.5	714	65
12/4/2020 10:18	204.7	104.2	123.1	715	65.08
12/4/2020 10:18	205.2	105.2	123.3	718	65.05
12/4/2020 10:18	206.1	105.8	123.7	719	65.04
12/4/2020 10:18	206.3	105.4	124	720	64.96
12/4/2020 10:18	206.7	105.7	124.4	720	64.96
12/4/2020 10:18	207.6	106.7	125.1	721	65.12
12/4/2020 10:18	208.3	106.4	125.6	723	65.2
12/4/2020 10:18	209	106.8	126.6	724	65.16

12/4/2020 10:18	209.5	106.9	126.9	724	65
12/4/2020 10:18	209.8	107.6	126.7	726	65.13
12/4/2020 10:18	210.5	107.8	127.2	726	65.12
12/4/2020 10:18	211.1	107.6	127.5	726	65.08
12/4/2020 10:18	211.9	107	127.9	726	65.05
12/4/2020 10:18	212.2	108.6	128.4	726	65
12/4/2020 10:18	212.6	108.3	128.4	727	65.12
12/4/2020 10:18	213.3	108.5	129.1	729	65.12
12/4/2020 10:18	213.9	108.5	130.3	729	65.12
12/4/2020 10:18	214.7	109.2	130.2	730	65.08
12/4/2020 10:18	214.9	109.4	130.3	731	65.03
12/4/2020 10:18	215.5	109.5	130.9	732	65.04
12/4/2020 10:18	216.2	111.1	131.2	734	65.2
12/4/2020 10:18	216.9	110.8	131.7	735	65
12/4/2020 10:18	216.9	110.4	131.8	734	64.96
12/4/2020 10:18	217.7	110.8	132.1	734	65.05
12/4/2020 10:18	218.2	111.3	133.1	735	65.04
12/4/2020 10:18	219	111.2	133.9	735	65.04
12/4/2020 10:18	219.7	111.5	133.7	736	65.04
12/4/2020 10:18	219.6	112	134.1	738	65.05
12/4/2020 10:18	220.5	112.5	134.5	737	65.05
12/4/2020 10:18	220.9	112.8	135.1	739	64.96
12/4/2020 10:18	221.7	112.4	135.3	740	65.12
12/4/2020 10:18	222.3	113.4	135.7	740	65.25
12/4/2020 10:18	222.3	113.3	136.1	742	65.25
12/4/2020 10:19	223.2	113.5	136.7	740	65.04
12/4/2020 10:19	223.4	113.3	136.9	741	65
12/4/2020 10:19	224.3	114.5	137.4	744	65.09
12/4/2020 10:19	224.8	114.3	138	743	65.04
12/4/2020 10:19	224.8	114.3	138.3	745	65.04
12/4/2020 10:19	225.6	114.3	138.6	747	65.05
12/4/2020 10:19	225.8	115.2	139.2	748	65.17
12/4/2020 10:19	226.8	115.7	139.2	748	65.12
12/4/2020 10:19	226.9	115.3	139.7	749	65.04
12/4/2020 10:19	227.4	116.8	140.2	751	65.05
12/4/2020 10:19	228	116.6	140.6	751	65.21
12/4/2020 10:19	228.3	116	141.2	749	64.92
12/4/2020 10:19	228.3	116.5	142.3	751	65.09
12/4/2020 10:19	229.4	117.7	142.3	749	65.12
12/4/2020 10:19	229.9	117.5	142.3	751	65.04
12/4/2020 10:19	230.5	117.4	142.3	752	65
12/4/2020 10:19	231.1	118.5	143.1	752	64.96
12/4/2020 10:19	231.6	118.6	143.6	754	64.96
12/4/2020 10:19	231.8	118.5	144.2	755	65
12/4/2020 10:19	231.9	119.2	144.5	755	64.96
12/4/2020 10:19	232.9	119.6	145	756	64.96
12/4/2020 10:19	233.1	119.3	145.2	757	64.96

12/4/2020 10:19	233.1	119.6	145.9	757	64.91
12/4/2020 10:19	234.1	120.8	146.5	758	65.04
12/4/2020 10:19	234.2	120.7	146.3	760	65.04
12/4/2020 10:19	235	120.4	146.6	758	64.96
12/4/2020 10:19	235.3	121.8	146.9	758	64.96
12/4/2020 10:19	235.3	121.2	147.5	760	64.96
12/4/2020 10:19	236.3	121.6	148.1	762	64.92
12/4/2020 10:19	236.8	121.6	148.4	762	64.99
12/4/2020 10:19	237.3	122.5	149.1	766	64.92
12/4/2020 10:19	237.5	122.3	149.3	765	64.92
12/4/2020 10:19	237.6	122.7	149.4	765	65.03
12/4/2020 10:19	238.6	123.6	150	765	64.83
12/4/2020 10:19	238.6	123.8	150.5	767	65.12
12/4/2020 10:19	239.4	123.7	150.9	765	64.96
12/4/2020 10:19	239.6	124.9	151.2	765	64.95
12/4/2020 10:19	239.6	124.7	151.5	766	64.8
12/4/2020 10:19	240.6	124.4	152.2	767	64.96
12/4/2020 10:19	240.7	124.6	152.4	768	65.03
12/4/2020 10:19	241.5	125.7	152.6	770	65.03
12/4/2020 10:19	241.7	125	153.3	769	65.08
12/4/2020 10:19	241.8	125.6	153.3	769	65.03
12/4/2020 10:19	242.5	126.5	153.7	773	65.08
12/4/2020 10:19	242.8	126.6	154.5	772	64.84
12/4/2020 10:19	243.6	126.5	155.3	773	64.93
12/4/2020 10:19	243.8	126.9	155.1	776	64.95
12/4/2020 10:19	243.9	127.4	155.7	773	64.83
12/4/2020 10:19	244.6	127.4	155.9	774	64.96
12/4/2020 10:19	244.9	127.5	156.2	773	64.96
12/4/2020 10:19	244.9	128.6	156.5	775	64.95
12/4/2020 10:19	245.7	128.4	156.6	779	65.12
12/4/2020 10:19	245.8	128.4	157.1	780	65.08
12/4/2020 10:19	246.5	129.2	158.4	781	64.92
12/4/2020 10:19	246.8	129.2	158.6	780	64.99
12/4/2020 10:19	246.8	129.4	158.5	782	64.88
12/4/2020 10:19	247.7	130.7	158.7	783	65.08
12/4/2020 10:19	247.8	130.1	159.1	785	65
12/4/2020 10:19	248.6	130.6	159.4	784	64.96
12/4/2020 10:20	248.8	130	160.1	784	65.05
12/4/2020 10:20	248.7	131.2	160.3	788	65.05
12/4/2020 10:20	249.6	131.5	160.7	787	64.92
12/4/2020 10:20	249.7	131.6	160.7	789	65.05
12/4/2020 10:20	250.4	132.4	161.2	790	64.92
12/4/2020 10:20	250.7	132.2	161.5	790	64.92
12/4/2020 10:20	250.7	132.6	161.8	791	64.96
12/4/2020 10:20	251.4	133.6	162.4	792	64.96
12/4/2020 10:20	251.6	133.4	163	791	64.99
12/4/2020 10:20	252.3	133.2	163.3	792	64.88

12/4/2020 10:20	252.4	133.6	164	791	65.04
12/4/2020 10:20	252.5	134.1	164.2	791	64.92
12/4/2020 10:20	253.1	134.1	164.9	794	64.84
12/4/2020 10:20	253.4	134.8	165.4	791	64.99
12/4/2020 10:20	253.4	135.4	165.9	793	65.04
12/4/2020 10:20	254.2	135.7	166.4	794	64.91
12/4/2020 10:20	254.3	135.2	166.7	795	64.99
12/4/2020 10:20	255	136.3	167.1	796	64.8
12/4/2020 10:20	255.2	136.5	167.5	798	64.99
12/4/2020 10:20	255.7	136.5	167.5	799	64.92
12/4/2020 10:20	256	136.3	167.9	798	64.99
12/4/2020 10:20	256	137.3	167.9	801	64.91
12/4/2020 10:20	256.8	137.5	168.2	799	64.88
12/4/2020 10:20	257	137.7	168.7	801	65.04
12/4/2020 10:20	257	138.6	168.9	800	64.83
12/4/2020 10:20	257.8	138	169.5	798	64.84
12/4/2020 10:20	257.9	138.5	169.9	801	64.99
12/4/2020 10:20	258.5	139.5	170.3	802	64.91
12/4/2020 10:20	258.7	139.2	170.5	800	64.91
12/4/2020 10:20	258.8	139.9	171.2	802	64.84
12/4/2020 10:20	259.6	139.5	171.4	804	64.99
12/4/2020 10:20	259.6	140.4	171.7	805	64.92
12/4/2020 10:20	260.3	140.7	172.7	806	64.96
12/4/2020 10:20	260.4	140.4	173.1	806	65.12
12/4/2020 10:20	260.5	141.5	172.8	807	64.84
12/4/2020 10:20	261.3	141.6	173.3	808	64.96
12/4/2020 10:20	261.4	141.5	173.5	809	65.03
12/4/2020 10:20	262.2	142.5	174.1	809	65.04
12/4/2020 10:20	262.3	142.1	174.5	812	64.8
12/4/2020 10:20	262.3	142.6	174.8	812	65.04
12/4/2020 10:20	263	143.7	174.9	813	64.76
12/4/2020 10:20	263.1	143.1	176.4	812	64.88
12/4/2020 10:20	263.8	143.7	176	815	65
12/4/2020 10:20	263.8	143.7	175.9	814	64.99
12/4/2020 10:20	264	144	176.6	816	65.03
12/4/2020 10:20	264.6	144.6	177.1	816	64.88
12/4/2020 10:20	264.9	145.5	177.4	816	64.95
12/4/2020 10:20	264.9	145.3	177.8	816	64.88
12/4/2020 10:20	265.6	145.6	178.8	819	65.04
12/4/2020 10:20	265.8	145.3	178.5	819	65.04
12/4/2020 10:20	266.3	146.5	178.7	820	64.92
12/4/2020 10:20	266.4	146.6	179.2	820	64.88
12/4/2020 10:20	266.4	146.6	179.5	822	65
12/4/2020 10:20	267.1	147.7	180	822	64.92
12/4/2020 10:20	267.3	147.5	180.2	822	65.07
12/4/2020 10:20	267.9	147.5	180.9	823	65.01
12/4/2020 10:20	268.1	148.4	181.7	824	64.92

12/4/2020 10:20	268.1	148.4	181.7	825	64.96
12/4/2020 10:20	268.8	148.9	181.4	825	64.84
12/4/2020 10:21	268.9	148.9	181.7	826	64.87
12/4/2020 10:21	269.6	149.2	182.5	827	64.88
12/4/2020 10:21	269.7	149.5	183	826	65.05
12/4/2020 10:21	269.7	150.4	183.1	829	64.88
12/4/2020 10:21	270.4	150.5	183.5	828	64.96
12/4/2020 10:21	270.5	150.7	183.7	829	64.92
12/4/2020 10:21	271.1	150.6	184.3	832	64.96
12/4/2020 10:21	271.2	151.5	184.7	832	65.04
12/4/2020 10:21	271.3	151.6	185.1	832	64.91
12/4/2020 10:21	272	151.7	185.5	832	65.05
12/4/2020 10:21	272.2	152.7	185.8	834	65.13
12/4/2020 10:21	272.7	152.6	186.1	836	64.88
12/4/2020 10:21	272.8	152.2	186.2	836	65.05
12/4/2020 10:21	272.9	153.7	186.5	835	65.01
12/4/2020 10:21	273.5	153.9	186.7	836	65.04
12/4/2020 10:21	273.7	153.9	187.4	833	65.03
12/4/2020 10:21	273.7	154.6	188.4	835	64.93
12/4/2020 10:21	274.2	154.2	188.3	836	65.08
12/4/2020 10:21	274.3	154.2	188.2	837	65
12/4/2020 10:21	274.9	154.6	188.8	836	64.99
12/4/2020 10:21	275	155.3	189	837	65.09
12/4/2020 10:21	275.1	155.3	189.4	837	65.04
12/4/2020 10:21	275.7	155.4	189.3	835	64.96
12/4/2020 10:21	275.8	156.3	190	836	65.09
12/4/2020 10:21	276.3	156	191	837	64.92
12/4/2020 10:21	276.5	156.2	191	834	65
12/4/2020 10:21	276.5	157.6	191.3	836	64.96
12/4/2020 10:21	277.1	157.2	191.5	836	64.92
12/4/2020 10:21	277.1	157.1	191.9	837	65.05
12/4/2020 10:21	277.6	157.5	192.3	838	64.96
12/4/2020 10:21	277.8	158.1	192.1	838	64.95
12/4/2020 10:21	277.8	158.7	192.7	839	65.12
12/4/2020 10:21	278.4	158.8	193.9	838	65.12
12/4/2020 10:21	278.4	159.3	194.3	842	64.96
12/4/2020 10:21	279	159.3	193.8	978	65.16
12/4/2020 10:21	279.1	159.3	194.5	1331	64.99
12/4/2020 10:21	279.2	160.3	194.7	1590	64.88
12/4/2020 10:21	279.8	160.2	195	1760	65.08
12/4/2020 10:21	279.9	160.4	195.1	1863	65.04
12/4/2020 10:21	280.3	160.4	195.2	1929	65.05
12/4/2020 10:21	280.4	161.2	196.3	1969	65.13
12/4/2020 10:21	280.4	161.1	196.2	1995	64.87
12/4/2020 10:21	281	161.4	195.7	2012	65
12/4/2020 10:21	281.1	162.5	195.7	2027	65.04
12/4/2020 10:21	281.6	162.6	195.9	2033	65

12/4/2020 10:21	281.7	162.6	195.8	2040	65
12/4/2020 10:21	281.8	163.7	195.5	2044	64.88
12/4/2020 10:21	282.4	163.2	195.4	2048	64.96
12/4/2020 10:21	282.6	163.7	195	2054	65
12/4/2020 10:21	282.6	164.6	195.6	2057	64.95
12/4/2020 10:21	283.2	164.3	195.5	2061	64.88
12/4/2020 10:21	283.3	164.5	194.5	2062	64.92
12/4/2020 10:21	283.9	165.1	194.3	2065	64.83
12/4/2020 10:21	284	165.4	194.1	2066	64.88
12/4/2020 10:21	284.1	165.1	193.8	2069	65
12/4/2020 10:21	284.6	166	193.5	2073	64.99
12/4/2020 10:21	284.7	166.7	192.6	2074	64.99
12/4/2020 10:21	285.3	166.1	192.4	2080	64.88
12/4/2020 10:21	285.4	167.7	191.7	2091	64.88
12/4/2020 10:21	285.4	167.5	192.1	2092	64.99
12/4/2020 10:22	286.2	167.4	192.1	2093	64.99
12/4/2020 10:22	286.2	168.1	191.3	2094	65.09
12/4/2020 10:22	286.8	168.7	190.3	2097	65.08
12/4/2020 10:22	287	169.5	189.9	2099	64.87
12/4/2020 10:22	287	169.6	189.3	2099	64.93
12/4/2020 10:22	287.6	169.6	188.8	2102	64.88
12/4/2020 10:22	287.7	170.4	188.4	2103	64.8
12/4/2020 10:22	288.2	170.5	187.9	2103	64.99
12/4/2020 10:22	288.3	170.4	187.6	2110	65
12/4/2020 10:22	288.4	171.4	187	2116	65.08
12/4/2020 10:22	289	171.6	186.5	2122	64.84
12/4/2020 10:22	289.1	171	186.2	2122	64.88
12/4/2020 10:22	289.7	172.8	185.4	2123	64.88
12/4/2020 10:22	289.8	172	185.1	2127	64.84
12/4/2020 10:22	289.9	172.5	184.5	2130	64.79
12/4/2020 10:22	290.5	173.5	183.8	2139	65.08
12/4/2020 10:22	290.6	173.3	183.4	2138	64.88
12/4/2020 10:22	290.7	174.5	182.7	2136	64.99
12/4/2020 10:22	291.3	174.5	182.4	2138	64.88
12/4/2020 10:22	291.4	174.4	182.8	2142	64.83
12/4/2020 10:22	291.8	175.5	181.8	2146	65.08
12/4/2020 10:22	291.9	175.2	180.8	2151	64.92
12/4/2020 10:22	292	175.6	180.2	2152	64.8
12/4/2020 10:22	292.6	176.8	179.5	2158	64.92
12/4/2020 10:22	292.7	176.9	179.2	2163	65.04
12/4/2020 10:22	293.2	177.1	178.8	2168	64.83
12/4/2020 10:22	293.4	177.4	178.3	2172	64.84
12/4/2020 10:22	293.4	177.1	177.5	2174	64.83
12/4/2020 10:22	294	178.6	177.2	2174	64.96
12/4/2020 10:22	294.1	178.5	177.3	2180	65.08
12/4/2020 10:22	294.5	178.8	176.8	2182	64.91
12/4/2020 10:22	294.7	179.4	175.4	2183	64.83

12/4/2020 10:22	294.6	179.5	175	2184	65.04
12/4/2020 10:22	295.1	180.4	174.8	2185	64.99
12/4/2020 10:22	295.3	180.8	174.5	2190	64.91
12/4/2020 10:22	295.7	180.2	173.8	2193	64.8
12/4/2020 10:22	295.8	181.9	173.4	2197	64.84
12/4/2020 10:22	295.8	181.6	172.8	2200	65.03
12/4/2020 10:22	296.3	182.7	173.3	2200	64.72
12/4/2020 10:22	296.4	182.9	172.1	2203	64.96
12/4/2020 10:22	296.9	182.8	171.3	2216	64.88
12/4/2020 10:22	297.1	183.4	171.1	2221	65.04
12/4/2020 10:22	297.1	183.3	170.8	2228	64.92
12/4/2020 10:22	297.6	183.4	170.3	2232	64.99
12/4/2020 10:22	297.7	184.2	170	2229	64.88
12/4/2020 10:22	297.7	184.4	169.3	2228	64.92
12/4/2020 10:22	298.3	185.1	169.1	2229	64.84
12/4/2020 10:22	298.3	185.2	169.4	2236	65.12
12/4/2020 10:22	298.4	185.6	169.1	2238	64.92
12/4/2020 10:22	298.6	186.1	167.9	2236	64.83
12/4/2020 10:22	298.6	186.3	167.3	2235	64.99
12/4/2020 10:22	299.3	186.7	167.2	2238	65.05
12/4/2020 10:22	299.3	187.2	166.7	2244	64.92
12/4/2020 10:22	299.4	187.5	166.5	2249	64.96
12/4/2020 10:22	299.9	188.4	166.3	2259	64.99
12/4/2020 10:22	299.9	188.4	165.8	2262	65
12/4/2020 10:22	300.1	189.3	165.5	2268	65.05
12/4/2020 10:22	300.3	189.1	164.9	2267	64.87
12/4/2020 10:22	300.7	189.4	164.6	2270	64.96
12/4/2020 10:23	300.9	190.1	164.5	2275	64.92
12/4/2020 10:23	300.9	190.1	164.7	2285	65
12/4/2020 10:23	301.1	190.5	164.7	2288	65
12/4/2020 10:23	301.2	191	164.1	2284	64.99
12/4/2020 10:23	301.6	191	163.5	2292	65.04
12/4/2020 10:23	301.8	192.5	163	2296	64.96
12/4/2020 10:23	301.8	192.2	162.5	2301	64.88
12/4/2020 10:23	301.9	192.2	162.2	2298	65.08
12/4/2020 10:23	302.1	193.5	162.3	2298	64.96
12/4/2020 10:23	302.4	193.4	162	2296	64.88
12/4/2020 10:23	302.6	193.6	161.8	2300	65.08
12/4/2020 10:23	302.6	194.3	161.6	2301	65.03
12/4/2020 10:23	302.8	194.2	161.1	2304	64.96
12/4/2020 10:23	303	195.2	161.1	2305	65.01
12/4/2020 10:23	303	195.5	160.9	2310	64.96
12/4/2020 10:23	303.1	196	160.7	2317	65.01
12/4/2020 10:23	303.3	196.7	160.4	2321	65.08
12/4/2020 10:23	303.8	196.5	160.2	2317	65.08
12/4/2020 10:23	303.9	196.1	160	2317	64.96
12/4/2020 10:23	303.9	197.5	159.9	2319	64.96

12/4/2020 10:23	304	197.3	159.8	2317	64.96
12/4/2020 10:23	304.2	198.3	159.5	2322	65.09
12/4/2020 10:23	304.3	198.4	159.5	2326	64.91
12/4/2020 10:23	304.5	198.4	159.4	2327	65.04
12/4/2020 10:23	304.5	199.6	159.7	2330	65
12/4/2020 10:23	304.7	199.3	159.7	2331	64.96
12/4/2020 10:23	304.8	199.7	159.9	2335	64.92
12/4/2020 10:23	305	200.7	159.9	2339	64.92
12/4/2020 10:23	305.3	200.4	159.8	2345	64.92
12/4/2020 10:23	305.3	200.7	159.7	2346	64.92
12/4/2020 10:23	305.5	201.2	159.2	2348	65.03
12/4/2020 10:23	305.6	201.1	158.8	2356	65.12
12/4/2020 10:23	305.6	202.5	158.7	2358	65.08
12/4/2020 10:23	305.8	202.5	158.8	2359	64.8
12/4/2020 10:23	305.9	202.3	159	2359	64.92
12/4/2020 10:23	306.1	202.5	158.9	2362	65.04
12/4/2020 10:23	306.1	203.4	158.7	2359	64.96
12/4/2020 10:23	306.4	203.8	158.4	2364	64.84
12/4/2020 10:23	306.4	204.7	158.3	2368	64.99
12/4/2020 10:23	306.5	204.7	159	2357	64.88
12/4/2020 10:23	306.6	204.8	159.3	2409	65.21
12/4/2020 10:23	306.7	205.6	158.9	2394	64.95
12/4/2020 10:23	306.8	205.3	158.4	2377	64.97
12/4/2020 10:23	306.3	205.6	158.2	2369	65.12
12/4/2020 10:23	306.1	206.2	158.6	2355	64.91
12/4/2020 10:23	305	206.5	158.6	2344	64.92
12/4/2020 10:23	304.3	206.7	158.6	2339	64.92
12/4/2020 10:23	303.7	207.5	158.4	2337	65
12/4/2020 10:23	302.5	207.4	158.1	2331	65.05
12/4/2020 10:23	301.9	207.5	158.3	2326	64.96
12/4/2020 10:23	300.6	207.4	159	2327	64.96
12/4/2020 10:23	299.2	208.8	159.2	2327	65.12
12/4/2020 10:23	299.2	208.3	158.5	2322	64.96
12/4/2020 10:23	296.9	208.4	158.3	2325	65.21
12/4/2020 10:23	296.1	208.5	158.6	2328	65.25
12/4/2020 10:23	294.4	209.1	158.5	2326	65.08
12/4/2020 10:23	292.9	209.3	158.5	2325	65.12
12/4/2020 10:23	292.9	209.3	158.4	2326	65.12
12/4/2020 10:23	290.2	209	158.1	2317	65.08
12/4/2020 10:23	289.3	209.5	158.2	2317	65.08
12/4/2020 10:24	287.6	209.2	158.7	2315	64.99
12/4/2020 10:24	285.7	210.7	159.4	2320	65.05
12/4/2020 10:24	282.9	210.5	158.8	2314	64.92
12/4/2020 10:24	282	210.2	158.5	2315	65
12/4/2020 10:24	280.2	210.3	158.6	2315	65.04
12/4/2020 10:24	279.2	210.3	158.6	2312	64.96
12/4/2020 10:24	278.4	210.4	158.8	2316	65.12

12/4/2020 10:24	276.2	211.6	158.8	2315	65.04
12/4/2020 10:24	274.6	211.6	158.8	2316	64.96
12/4/2020 10:24	272.8	211.6	158.9	2314	64.97
12/4/2020 10:24	271.9	211.5	159	2309	64.99
12/4/2020 10:24	271	211.4	159	2308	65.12
12/4/2020 10:24	268.4	211.4	159	2305	64.96
12/4/2020 10:24	267.4	211.4	159.1	2302	64.84
12/4/2020 10:24	265.7	211.5	159.1	2303	64.88
12/4/2020 10:24	264.8	211.6	159.1	2298	64.96
12/4/2020 10:24	263.9	211.4	159.1	2295	64.88
12/4/2020 10:24	262.1	211.4	159.2	2296	64.99
12/4/2020 10:24	260.4	211.5	159.2	2300	64.83
12/4/2020 10:24	260.3	211.4	159.3	2297	65
12/4/2020 10:24	257.7	211.5	159.4	2296	65.05
12/4/2020 10:24	256.9	211.4	159.4	2291	64.91
12/4/2020 10:24	255.2	211.5	159.2	2289	65.04
12/4/2020 10:24	253.5	211.6	159.4	2290	64.96
12/4/2020 10:24	253.5	211.5	159.9	2291	64.96
12/4/2020 10:24	251.2	211.4	160.5	2290	65
12/4/2020 10:24	250.4	211.4	159.5	2289	64.92
12/4/2020 10:24	248.7	211.6	159.5	2292	64.96
12/4/2020 10:24	247.1	211.4	160	2296	65
12/4/2020 10:24	247.1	211.7	159.9	2290	64.96
12/4/2020 10:24	244.7	211.4	160.1	2292	65.08
12/4/2020 10:24	244	211.6	159.9	2293	65.05
12/4/2020 10:24	242.3	211.4	160.8	2286	64.88
12/4/2020 10:24	241.1	211.4	160.8	2286	65.04
12/4/2020 10:24	241	211.5	160.1	2281	64.88
12/4/2020 10:24	238.7	211.6	160.5	2279	64.88
12/4/2020 10:24	238.1	211.4	160.9	2278	64.92
12/4/2020 10:24	236.7	211.5	160.9	2279	64.83
12/4/2020 10:24	235.9	211.4	160.6	2276	65.04
12/4/2020 10:24	235.1	211.6	161.4	2276	65.12
12/4/2020 10:24	233.2	211.5	161.6	2275	64.95
12/4/2020 10:24	232.5	211.4	160.9	2272	65.01
12/4/2020 10:24	231.1	211.6	160.9	2270	65.08
12/4/2020 10:24	230.5	211.7	161.3	2274	65.08
12/4/2020 10:24	229.9	211.6	161.6	2271	64.84
12/4/2020 10:24	228.4	211.4	161.4	2271	65.08
12/4/2020 10:24	227.3	211.4	161.3	2269	65.08
12/4/2020 10:24	227	211.5	161.7	2265	65.08
12/4/2020 10:24	225.4	211.4	162.4	2265	64.71
12/4/2020 10:24	224.8	210.7	162.8	2265	65.12
12/4/2020 10:24	223.7	210.6	162.7	2264	64.96
12/4/2020 10:24	222.6	210.2	162.2	2261	64.95
12/4/2020 10:24	222.5	210.5	161.9	2259	64.84
12/4/2020 10:24	220.8	210.8	162.3	2257	64.92

12/4/2020 10:24	220.2	210.2	162.1	2257	64.99
12/4/2020 10:24	219.1	210.5	162.5	2259	65.12
12/4/2020 10:24	218	210.6	162.8	2257	64.92
12/4/2020 10:24	218	210.2	162.8	2257	64.96
12/4/2020 10:25	216.5	210.6	163	2257	65.04
12/4/2020 10:25	216	210.3	163.3	2257	65.04
12/4/2020 10:25	214.9	209.5	163.2	2255	64.96
12/4/2020 10:25	213.9	209.7	163.2	2254	65
12/4/2020 10:25	213.9	209	163.6	2254	65
12/4/2020 10:25	212.6	209.4	163.8	2252	65
12/4/2020 10:25	212.3	209	164.6	2247	64.87
12/4/2020 10:25	211.1	209.2	164.9	2246	65
12/4/2020 10:25	210.9	209.8	164.6	2242	65
12/4/2020 10:25	210.3	209.3	164.4	2239	64.87
12/4/2020 10:25	209.3	208.6	164.5	2242	65.04
12/4/2020 10:25	208.8	208.4	164.5	2245	64.96
12/4/2020 10:25	207.7	208.1	164.7	1956	64.96
12/4/2020 10:25	207	208.6	164.9	1534	65.04
12/4/2020 10:25	206.9	208.6	164.9	1243	64.83
12/4/2020 10:25	205.9	208.8	165.1	1072	65.08
12/4/2020 10:25	205.4	208.5	164.7	972	64.96
12/4/2020 10:25	204.6	208.3	165.8	910	64.92
12/4/2020 10:25	204.3	208.3	166.4	870	64.92
12/4/2020 10:25	203.8	207.4	165.6	849	64.84
12/4/2020 10:25	203.1	207.5	166	836	64.92
12/4/2020 10:25	202.5	207.4	166.1	828	65.08
12/4/2020 10:25	202.4	207.5	165.7	822	64.83
12/4/2020 10:25	201.5	207.5	166	819	64.88
12/4/2020 10:25	201	207.4	166.1	815	65.04
12/4/2020 10:25	200.3	207.5	166.1	814	64.75
12/4/2020 10:25	199.7	206.4	167	813	64.93
12/4/2020 10:25	199.7	206.6	167.2	811	64.84
12/4/2020 10:25	198.9	206.4	166.6	811	64.91
12/4/2020 10:25	198.5	206.3	166.3	810	64.88
12/4/2020 10:25	197.8	206.3	166.5	811	64.88
12/4/2020 10:25	197.2	206.2	166.7	809	64.99
12/4/2020 10:25	197.2	206.3	166.9	811	65.12
12/4/2020 10:25	196.5	206.7	167	812	64.96
12/4/2020 10:25	196	205.8	167	809	64.84
12/4/2020 10:25	195.4	205.6	167	809	65.08
12/4/2020 10:25	195.3	205.3	166.8	808	64.91
12/4/2020 10:25	195.2	205.1	166.8	809	64.92
12/4/2020 10:25	194.3	205.4	166.7	809	64.88
12/4/2020 10:25	194.2	205.3	167	808	64.8
12/4/2020 10:25	193.3	205.8	167.4	809	64.84
12/4/2020 10:25	193.1	205.3	167.5	809	64.88
12/4/2020 10:25	193	204.1	167.6	807	64.88

12/4/2020 10:25	192.1	204.8	167.6	808	64.84
12/4/2020 10:25	192	204.3	167.6	805	64.79
12/4/2020 10:25	191.2	204.7	167.8	805	64.95
12/4/2020 10:25	191.1	204.8	167.9	805	65.12
12/4/2020 10:25	191	204.2	167.7	805	64.91
12/4/2020 10:25	190.1	204.6	167.7	805	64.83
12/4/2020 10:25	189.9	203.4	167.2	805	64.84
12/4/2020 10:25	189.5	203.6	167.2	803	64.99
12/4/2020 10:25	189	203.6	167.1	807	64.84
12/4/2020 10:25	188.9	203.5	167.2	804	64.99
12/4/2020 10:25	188.2	203.6	167.5	809	65.03
12/4/2020 10:25	187.9	203.6	167.4	825	65.08
12/4/2020 10:25	187.2	203.3	167.5	825	64.86
12/4/2020 10:25	187.2	202.7	167.3	820	64.92
12/4/2020 10:25	187	202.3	167.2	819	65.03
12/4/2020 10:25	186.5	202.5	167.8	817	64.91
12/4/2020 10:26	186.3	202.4	168	818	64.88
12/4/2020 10:26	186.3	202.3	167.2	814	64.99
12/4/2020 10:26	186.2	202.5	167.4	812	65.03
12/4/2020 10:26	186.1	202.5	167.6	814	64.95
12/4/2020 10:26	186	202.3	167.8	812	64.95
12/4/2020 10:26	185.9	201.1	167.7	809	65.04
12/4/2020 10:26	185.9	201.3	168.6	811	65.04
12/4/2020 10:26	185.9	201.3	168.1	810	64.95
12/4/2020 10:26	186	201.1	167.7	809	64.92
12/4/2020 10:26	186	201.7	168	809	64.95
12/4/2020 10:26	186.1	201.2	168.6	809	65.08
12/4/2020 10:26	186.2	201.4	168.7	809	65.04
12/4/2020 10:26	186.3	201.5	168.6	808	64.99
12/4/2020 10:26	186.6	200.9	169.3	809	64.88
12/4/2020 10:26	186.7	200.3	169.2	809	64.84
12/4/2020 10:26	187	200.2	168.7	809	64.76
12/4/2020 10:26	187	200.6	169	800	65.01
12/4/2020 10:26	187.5	200.8	169.4	796	65.08
12/4/2020 10:26	187.6	200.2	169.6	796	64.88
12/4/2020 10:26	187.6	200.2	169.7	798	64.88
12/4/2020 10:26	187.7	200.5	169.6	800	65.01
12/4/2020 10:26	187.9	199.9	170.3	798	64.96
12/4/2020 10:26	188	200.6	170.8	800	64.88
12/4/2020 10:26	188.1	199.7	170.4	802	65
12/4/2020 10:26	188.1	199.9	170	801	65
12/4/2020 10:26	188.3	199.7	170.4	800	65.04
12/4/2020 10:26	188.3	199.4	170.7	801	64.87
12/4/2020 10:26	188.4	199.6	170.8	801	65.09
12/4/2020 10:26	188.5	199.1	170.8	802	65
12/4/2020 10:26	188.5	199.4	170.9	800	64.92
12/4/2020 10:26	188.5	199.8	170.9	800	64.88

12/4/2020 10:26	188.5	199.4	171.2	800	64.96
12/4/2020 10:26	188.5	199.2	171.2	801	65.08
12/4/2020 10:26	188.5	198.2	171.2	802	65.04
12/4/2020 10:26	188.5	198.3	171.9	800	64.92
12/4/2020 10:26	188.4	198.6	172.2	801	64.88
12/4/2020 10:26	188.4	198.4	172.3	802	65.16
12/4/2020 10:26	188.3	198.4	172	799	64.99
12/4/2020 10:26	188.3	198.5	171.5	798	64.96
12/4/2020 10:26	188.3	198.3	171.5	800	65.01
12/4/2020 10:26	188.1	198.3	171.6	798	64.88
12/4/2020 10:26	188.1	198.3	171.4	797	65.04
12/4/2020 10:26	188.1	198.2	171.6	799	65.16
12/4/2020 10:26	187.9	197.3	171.9	799	64.88
12/4/2020 10:26	187.8	197.4	171.7	798	65.04
12/4/2020 10:26	187.7	197.3	171.9	798	64.92
12/4/2020 10:26	187.6	197.4	172	798	64.88
12/4/2020 10:26	187.6	197.3	172.2	796	64.92
12/4/2020 10:26	187.5	197.3	172.7	796	64.88
12/4/2020 10:26	187.5	197.5	172.7	798	64.92
12/4/2020 10:26	187.3	197.5	173.2	796	64.96
12/4/2020 10:26	187.2	197.3	173.2	796	64.87
12/4/2020 10:26	187.2	197.3	172.6	796	65
12/4/2020 10:26	187.1	196	172.6	796	65.09
12/4/2020 10:26	186.9	196.6	172.7	798	64.92
12/4/2020 10:26	186.9	196.6	172.6	796	65
12/4/2020 10:26	186.6	196.1	172.7	796	65
12/4/2020 10:26	186.6	196.4	172.9	798	64.91
12/4/2020 10:26	186.5	196.6	172.6	798	64.96
12/4/2020 10:26	186.4	196.4	172.6	796	65.05
12/4/2020 10:27	186.3	196.6	173.2	795	65
12/4/2020 10:27	186.2	196.4	173.3	796	65.04
12/4/2020 10:27	186.2	196.2	173	794	65.04
12/4/2020 10:27	186	195.5	172.8	792	65.04
12/4/2020 10:27	185.9	195.5	173.2	793	64.97
12/4/2020 10:27	185.8	195.7	173.1	793	64.8
12/4/2020 10:27	185.6	195.8	172.8	791	65.08
12/4/2020 10:27	185.5	195.8	173	791	64.84
12/4/2020 10:27	185.4	195.6	172.9	791	65.08
12/4/2020 10:27	185.3	195.3	172.9	791	64.96
12/4/2020 10:27	185.2	195.8	173.7	791	64.88
12/4/2020 10:27	185.2	195.5	173.8	791	65.08
12/4/2020 10:27	185	195	172.9	791	64.84
12/4/2020 10:27	184.9	194.3	173	791	65.04
12/4/2020 10:27	184.8	194.1	173	790	64.88
12/4/2020 10:27	184.6	194.4	172.9	790	64.99
12/4/2020 10:27	184.6	194.7	173.2	791	64.88
12/4/2020 10:27	184.4	194.5	172.7	791	65.08

12/4/2020 10:27	184.2	194.5	173.1	791	64.83
12/4/2020 10:27	184.1	194.1	173.1	788	65.04
12/4/2020 10:27	184	194.1	173.6	791	64.92
12/4/2020 10:27	183.9	194.5	173.5	788	64.91
12/4/2020 10:27	183.7	194.2	172.9	791	65
12/4/2020 10:27	183.7	193.6	173	791	64.96
12/4/2020 10:27	183.4	193.5	173.1	792	65
12/4/2020 10:27	183.5	193.3	173.3	792	65.04
12/4/2020 10:27	183.3	193.5	173.2	788	64.87
12/4/2020 10:27	183.1	193.4	173.3	790	64.92
12/4/2020 10:27	183.1	193.5	173	791	65
12/4/2020 10:27	182.9	193.5	173.1	789	64.87
12/4/2020 10:27	182.9	193.5	173.4	789	65
12/4/2020 10:27	182.7	193.4	173.1	788	65
12/4/2020 10:27	182.5	193.5	173.1	788	65
12/4/2020 10:27	182.4	192.5	173.4	787	65
12/4/2020 10:27	182.3	192.2	173.1	787	64.83
12/4/2020 10:27	182.2	192.4	173.1	785	64.92
12/4/2020 10:27	182	192.5	173.2	787	65.08
12/4/2020 10:27	181.9	192.6	172.9	787	64.83
12/4/2020 10:27	181.8	192.5	173.2	787	64.99
12/4/2020 10:27	181.6	192.4	173.6	787	65.04
12/4/2020 10:27	181.6	192.3	173.3	788	65.08
12/4/2020 10:27	181.5	192.6	172.9	790	65.04
12/4/2020 10:27	181.3	192.4	172.5	788	64.84
12/4/2020 10:27	181.2	192.6	172.8	787	64.92
12/4/2020 10:27	181.1	191.2	173.1	787	64.93
12/4/2020 10:27	181	191.2	172.8	787	64.84
12/4/2020 10:27	180.9	191.6	172.7	787	65.08
12/4/2020 10:27	180.7	191.4	172.7	786	64.84
12/4/2020 10:27	180.6	191.3	173.4	786	64.88
12/4/2020 10:27	180.5	191.7	173.1	787	64.93
12/4/2020 10:27	180.4	191.5	172.3	788	64.8
12/4/2020 10:27	180.3	191.7	172.6	793	65.08
12/4/2020 10:27	180.1	191.2	172.8	793	64.99
12/4/2020 10:27	180.1	191.2	172.7	791	64.83
12/4/2020 10:27	179.9	190.6	172.4	789	65.04
12/4/2020 10:27	179.9	190.7	173.5	787	64.92
12/4/2020 10:27	179.8	190.6	172.6	789	64.88
12/4/2020 10:27	179.6	190.3	172.4	787	64.8
12/4/2020 10:27	179.6	190	172.7	787	64.95
12/4/2020 10:28	179.6	190.2	172.7	788	65.08
12/4/2020 10:28	179.6	189.9	172.8	790	65
12/4/2020 10:28	179.5	190.6	172.6	788	64.95
12/4/2020 10:28	179.5	190	173.1	787	64.84
12/4/2020 10:28	179.5	190.2	173.4	787	64.84
12/4/2020 10:28	179.6	189.3	172.6	788	65.04

12/4/2020 10:28	179.5	189.3	172.4	787	64.88
12/4/2020 10:28	179.5	189.5	172.7	788	64.99
12/4/2020 10:28	179.6	189.3	172.5	786	64.84
12/4/2020 10:28	179.6	189.1	172.6	785	64.88
12/4/2020 10:28	179.6	189.3	172.7	784	64.75
12/4/2020 10:28	179.7	189.3	172.6	784	65.04
12/4/2020 10:28	179.8	189	172.8	783	64.99
12/4/2020 10:28	179.8	189.4	173.5	781	64.91
12/4/2020 10:28	179.8	189.2	173.4	782	64.8
12/4/2020 10:28	179.9	189.2	172.8	781	64.99
12/4/2020 10:28	180	189.3	172.4	781	64.96
12/4/2020 10:28	180	188.5	172.6	781	64.75
12/4/2020 10:28	180.1	188.3	172.9	782	64.88
12/4/2020 10:28	180.1	188.4	173.1	781	64.99
12/4/2020 10:28	180.2	188.4	173.1	783	64.99
12/4/2020 10:28	180.2	188.3	173.1	781	64.91
12/4/2020 10:28	180.3	188.3	172.8	780	64.95
12/4/2020 10:28	180.3	188.3	172.9	782	64.8
12/4/2020 10:28	180.4	188.2	173.1	781	64.83
12/4/2020 10:28	180.4	188.3	172.9	780	64.8
12/4/2020 10:28	180.4	188.2	172.9	782	64.95
12/4/2020 10:28	180.5	188.3	173	782	64.91
12/4/2020 10:28	180.5	188.4	173.3	781	64.92
12/4/2020 10:28	180.6	187.4	173.4	781	64.87
12/4/2020 10:28	180.6	187.5	173.3	781	64.92
12/4/2020 10:28	180.7	187.2	173.1	781	64.92
12/4/2020 10:28	180.7	187	173.3	780	64.99
12/4/2020 10:28	180.7	187.7	173.4	780	64.88
12/4/2020 10:28	180.7	187.2	173.3	779	64.91
12/4/2020 10:28	180.7	187.7	173.6	780	64.91
12/4/2020 10:28	180.7	187.5	173.6	779	64.96
12/4/2020 10:28	180.6	187.1	173.4	781	64.87
12/4/2020 10:28	180.7	187.4	173.4	779	64.92
12/4/2020 10:28	180.7	187.6	173.2	781	64.96
12/4/2020 10:28	180.7	187.6	173.5	780	64.91
12/4/2020 10:28	180.6	187.7	174	780	64.92
12/4/2020 10:28	180.6	185.9	173.8	780	64.92
12/4/2020 10:28	180.5	186.5	173.3	779	65
12/4/2020 10:28	180.5	186	173.4	779	64.96
12/4/2020 10:28	180.5	186.3	173.3	778	64.91
12/4/2020 10:28	180.5	186.7	173.5	781	65
12/4/2020 10:28	180.5	186.2	173.4	781	65
12/4/2020 10:28	180.4	186.1	173.4	780	64.87
12/4/2020 10:28	180.3	186.1	173.4	780	65
12/4/2020 10:28	180.3	186.1	172.9	778	65.09
12/4/2020 10:28	180.3	186.3	173.1	777	64.96
12/4/2020 10:28	180.2	186.6	173.9	778	64.95

12/4/2020 10:28	180.2	186.3	173.9	778	64.99
12/4/2020 10:28	180.1	186.4	173.8	778	64.96
12/4/2020 10:28	180.1	185	173.5	779	64.88
12/4/2020 10:28	180.1	185.6	173.3	779	64.83
12/4/2020 10:28	180	184.9	173.6	776	65.08
12/4/2020 10:28	179.9	185.6	173.3	777	65.08
12/4/2020 10:28	179.8	185.4	173.4	776	64.91
12/4/2020 10:29	179.8	185.3	173.5	776	65.04
12/4/2020 10:29	179.6	185.5	173.2	778	65.04
12/4/2020 10:29	179.6	185.1	173.3	779	65.08
12/4/2020 10:29	179.5	185.4	173.1	780	64.88
12/4/2020 10:29	179.5	185.2	173.1	780	65.08
12/4/2020 10:29	179.3	185.1	173	779	65.08
12/4/2020 10:29	179.4	185.4	173.1	779	64.91
12/4/2020 10:29	179.3	185.5	172.8	779	65.08
12/4/2020 10:29	179.2	184.4	173	776	65.12
12/4/2020 10:29	179.2	184.4	173	778	65.08
12/4/2020 10:29	179.1	184.2	173	777	64.99
12/4/2020 10:29	179.1	184.4	173.1	779	64.88
12/4/2020 10:29	179	184.4	172.9	779	65.08
12/4/2020 10:29	179	184.2	173.1	777	65.04
12/4/2020 10:29	178.9	184.4	173.2	775	65.08
12/4/2020 10:29	178.9	184.4	173	777	64.88
12/4/2020 10:29	178.8	184.4	173.1	778	64.83
12/4/2020 10:29	178.8	184.4	173.1	777	64.92
12/4/2020 10:29	178.7	184.3	173.1	780	65.01
12/4/2020 10:29	178.6	184.4	173.2	780	64.91
12/4/2020 10:29	178.6	184.5	173.1	778	65.08
12/4/2020 10:29	178.6	183.3	173.4	777	65.03
12/4/2020 10:29	178.6	183.1	173.5	776	65.12
12/4/2020 10:29	178.6	183.2	173.2	773	65.11
12/4/2020 10:29	178.6	183.4	172.9	776	65.49
12/4/2020 10:29	178.6	183.3	173.1	776	65.61
12/4/2020 10:29	178.6	183.4	173.1	779	65.69
12/4/2020 10:29	178.6	183.4	173.6	779	65.69
12/4/2020 10:29	178.7	183.3	174	779	65.65
12/4/2020 10:29	178.6	183.5	173.8	780	65.65
12/4/2020 10:29	178.6	183.3	173.9	779	65.5
12/4/2020 10:29	178.6	183.4	173.5	781	65.51
12/4/2020 10:29	178.7	183.5	173.3	780	65.26
12/4/2020 10:29	178.7	183.2	173.6	779	65.4
12/4/2020 10:29	178.7	183.4	173.4	777	65.27
12/4/2020 10:29	178.8	182.2	173.6	777	65.11
12/4/2020 10:29	178.8	182.6	173.5	777	65.07
12/4/2020 10:29	178.8	182.4	173.5	777	65.12
12/4/2020 10:29	178.7	182.5	173.4	776	65.08
12/4/2020 10:29	178.8	182.7	173.5	777	65.04

12/4/2020 10:29	178.8	182.7	173.6	777	64.96
12/4/2020 10:29	178.9	182.3	173.9	778	65.05
12/4/2020 10:29	178.8	182.5	174	777	65.12
12/4/2020 10:29	178.9	182.3	173.6	775	65.04
12/4/2020 10:29	179	182.2	174	778	65.09
12/4/2020 10:29	178.9	182.8	174.1	778	65.05
12/4/2020 10:29	178.9	182.3	173.8	778	65.09
12/4/2020 10:29	178.9	182.3	174.1	779	65.13
12/4/2020 10:29	179	182.7	173.8	778	64.92
12/4/2020 10:29	178.9	182.2	173.7	779	65.09
12/4/2020 10:29	178.9	182.6	173.8	776	65.05
12/4/2020 10:29	179	182.6	173.8	777	64.92
12/4/2020 10:29	179	181.1	174	776	65.04
12/4/2020 10:29	178.9	181.4	174.1	777	64.84
12/4/2020 10:29	178.9	181.2	173.9	777	65.17
12/4/2020 10:29	178.9	181.6	174	776	64.84
12/4/2020 10:29	179	181.7	173.7	777	64.84
12/4/2020 10:29	179	181.2	173.2	775	64.88
12/4/2020 10:30	179	181.7	173.3	774	65.12
12/4/2020 10:30	179	181.6	173.3	775	65.04
12/4/2020 10:30	179	181.1	173.1	777	64.8
12/4/2020 10:30	179	181.6	173.6	775	65
12/4/2020 10:30	178.9	181.4	173.6	773	65
12/4/2020 10:30	179	181.5	173.4	775	65.04
12/4/2020 10:30	178.9	181.1	173.5	774	64.96
12/4/2020 10:30	179	181.3	173.6	773	64.96
12/4/2020 10:30	178.9	181.6	173.4	772	64.84
12/4/2020 10:30	178.9	181.6	173.8	773	64.92
12/4/2020 10:30	178.8	180.2	174.2	773	65
12/4/2020 10:30	178.8	180.7	173.7	773	64.96
12/4/2020 10:30	178.8	180.3	173.4	773	64.96
12/4/2020 10:30	178.8	180.6	173.1	771	65
12/4/2020 10:30	178.8	180.6	173.4	771	64.92
12/4/2020 10:30	178.8	180.1	173.5	773	65
12/4/2020 10:30	178.7	180.6	173.5	773	65
12/4/2020 10:30	178.8	180.7	173.5	771	64.92
12/4/2020 10:30	178.7	180.2	173.3	773	64.92
12/4/2020 10:30	178.7	180.6	173.3	773	65.08
12/4/2020 10:30	178.8	180.4	173.4	773	65.04
12/4/2020 10:30	178.7	180.2	174	773	65.01
12/4/2020 10:30	178.7	180.2	174	773	64.84
12/4/2020 10:30	178.6	180.2	173.4	772	64.97
12/4/2020 10:30	178.6	180.6	173	771	64.93
12/4/2020 10:30	178.6	180.6	173.1	773	65
12/4/2020 10:30	178.6	180.5	173.3	773	65.12
12/4/2020 10:30	178.5	180.6	173.4	770	64.96
12/4/2020 10:30	178.5	179.5	173.5	773	65.08

12/4/2020 10:30	178.5	179.5	173.3	773	65
12/4/2020 10:30	178.5	179.4	173.1	772	64.96
12/4/2020 10:30	178.5	179.3	173.3	771	65.04
12/4/2020 10:30	178.5	179.5	173.5	770	65.01
12/4/2020 10:30	178.5	179.5	173.8	769	64.96
12/4/2020 10:30	178.4	179.4	174.3	769	65
12/4/2020 10:30	178.5	180.2	173.9	769	64.92
12/4/2020 10:30	178.3	179.4	173.6	770	65
12/4/2020 10:30	178.3	179.2	173.6	770	64.96
12/4/2020 10:30	178.3	179.3	173.4	769	64.88
12/4/2020 10:30	178.3	179.4	173.4	768	64.92
12/4/2020 10:30	178.3	179.5	173.1	769	65.08
12/4/2020 10:30	178.3	179.4	172.9	769	64.92
12/4/2020 10:30	178.3	179.2	173.3	768	64.84
12/4/2020 10:30	178.3	179.2	172.9	767	65.04
12/4/2020 10:30	178.2	179.4	173.1	768	65.08
12/4/2020 10:30	178.3	179.4	173.3	769	65
12/4/2020 10:30	178.3	179.4	173.1	767	64.84
12/4/2020 10:30	178.2	178.6	173.4	766	64.99
12/4/2020 10:30	178.2	178.3	174	767	65.04
12/4/2020 10:30	178.1	178.5	173.6	767	64.92
12/4/2020 10:30	178.1	178.4	173.3	769	65.04
12/4/2020 10:30	178.1	178.3	173.4	770	65.03
12/4/2020 10:30	178.1	178.3	173.1	770	65.04
12/4/2020 10:30	178.1	178.4	173.4	768	65.08
12/4/2020 10:30	178.1	178.3	173.5	769	64.88
12/4/2020 10:30	178.1	178.5	173.2	768	64.88
12/4/2020 10:30	178.1	178.4	173.1	767	64.88
12/4/2020 10:30	178.1	178.3	173.1	769	65.08
12/4/2020 10:30	178.1	178.6	173.8	769	64.99
12/4/2020 10:31	178.1	178.3	173.6	769	65
12/4/2020 10:31	178.1	178.3	173.3	767	65
12/4/2020 10:31	178.1	178.3	173.2	767	65
12/4/2020 10:31	178.1	178.3	173.5	769	65.04
12/4/2020 10:31	178.1	178.4	173.3	768	65
12/4/2020 10:31	178.1	178.3	173.2	767	65
12/4/2020 10:31	178.2	178.5	172.9	767	64.92
12/4/2020 10:31	178.3	178.5	173.2	770	64.92
12/4/2020 10:31	178.2	177.1	173.9	769	65.04
12/4/2020 10:31	178.2	177.4	173.4	768	65.04
12/4/2020 10:31	178.3	177.1	173.2	769	64.96
12/4/2020 10:31	178.3	177.5	173.4	767	64.92
12/4/2020 10:31	178.3	177.8	173.4	768	65.04
12/4/2020 10:31	178.3	177.3	173.5	769	64.96
12/4/2020 10:31	178.3	177.5	173.5	769	64.96
12/4/2020 10:31	178.4	177.3	173.1	768	64.88
12/4/2020 10:31	178.5	177.1	173.1	768	64.88

12/4/2020 10:31	178.6	177.4	173.3	765	65
12/4/2020 10:31	178.5	177.1	173	766	64.99
12/4/2020 10:31	178.5	177.5	173.3	766	65.04
12/4/2020 10:31	178.6	177.7	173.5	769	65.04
12/4/2020 10:31	178.6	177.4	173.6	769	65.08
12/4/2020 10:31	178.6	176.9	173.9	769	64.96
12/4/2020 10:31	178.7	177.4	174	769	64.99
12/4/2020 10:31	178.6	176.9	174	765	64.99
12/4/2020 10:31	178.8	177.7	173.9	765	64.88
12/4/2020 10:31	178.8	177.6	173.7	766	64.93
12/4/2020 10:31	178.7	177.1	173.4	765	64.99
12/4/2020 10:31	178.8	177.6	173.4	766	64.88
12/4/2020 10:31	178.8	177.4	173.3	765	64.84
12/4/2020 10:31	178.8	176.2	173.3	767	64.92
12/4/2020 10:31	178.8	176.2	173.3	766	65.04
12/4/2020 10:31	178.8	176.5	173.3	768	64.92
12/4/2020 10:31	178.7	176.7	173.3	768	65.04
12/4/2020 10:31	178.7	176.6	173.5	769	65.04
12/4/2020 10:31	178.7	176.2	173.2	769	64.99
12/4/2020 10:31	178.7	176.6	173.4	767	65.04
12/4/2020 10:31	178.7	176.1	173.3	768	64.83
12/4/2020 10:31	178.6	176.8	173.4	768	65
12/4/2020 10:31	178.6	176.8	173.6	769	65
12/4/2020 10:31	178.5	176.1	173.3	769	64.88
12/4/2020 10:31	178.5	176.4	173.4	769	64.96
12/4/2020 10:31	178.4	176.2	173.4	769	64.96
12/4/2020 10:31	178.4	176.4	173.2	768	65
12/4/2020 10:31	178.3	176.7	173.2	768	65.04
12/4/2020 10:31	178.3	176.5	173.5	762	65
12/4/2020 10:31	178.3	176.8	173.4	843	64.87
12/4/2020 10:31	178.2	176.2	174	1212	64.96
12/4/2020 10:31	178.2	176.2	174.1	1553	64.96
12/4/2020 10:31	178.1	176.2	174.1	1767	65
12/4/2020 10:31	178.1	176.3	173.8	1893	64.96
12/4/2020 10:31	178.1	176.2	173.5	1968	64.96
12/4/2020 10:31	178	176.8	172.9	2016	65
12/4/2020 10:31	178	176.4	173	2039	64.88
12/4/2020 10:31	177.9	176.2	173.1	2055	64.92
12/4/2020 10:31	177.9	175.2	173.5	2067	65.08
12/4/2020 10:31	177.9	175.4	173.4	2073	64.88
12/4/2020 10:31	177.9	175.6	173.1	2080	65.04
12/4/2020 10:31	177.9	175.5	173.3	2080	65.03
12/4/2020 10:31	177.8	175.2	173.7	2084	64.93
12/4/2020 10:32	177.8	175.6	173.4	2086	65.08
12/4/2020 10:32	177.8	175.3	173.3	2082	64.92
12/4/2020 10:32	177.8	175.6	173.6	2081	64.95
12/4/2020 10:32	177.9	175.4	174.1	2083	64.88

12/4/2020 10:32	177.8	175.4	174.1	2088	65.04
12/4/2020 10:32	177.8	175.4	173.3	2085	64.93
12/4/2020 10:32	177.8	175.2	173.5	2084	64.99
12/4/2020 10:32	178	175.4	173.8	2082	64.96
12/4/2020 10:32	177.8	175.6	173.8	2080	65.08
12/4/2020 10:32	177.9	175.5	173.8	2081	64.88
12/4/2020 10:32	177.9	175.2	174.2	2082	65.08
12/4/2020 10:32	177.9	175.4	173.6	2081	65.08
12/4/2020 10:32	177.9	175.4	174.3	2080	64.92
12/4/2020 10:32	178	175.5	174.8	2082	64.96
12/4/2020 10:32	178	175.7	174.1	2075	64.95
12/4/2020 10:32	178	175.6	174.1	2070	64.95
12/4/2020 10:32	178.1	175.6	174.3	2073	65.08
12/4/2020 10:32	178.1	175.6	174.4	2075	64.92
12/4/2020 10:32	178.1	175.6	174.7	2075	64.92
12/4/2020 10:32	178.2	175.6	174.6	2073	64.88
12/4/2020 10:32	178.1	175.2	174.4	2075	64.96
12/4/2020 10:32	178.1	175.2	175.4	2074	65.04
12/4/2020 10:32	178.2	175.7	175	2072	64.92
12/4/2020 10:32	178.2	175.2	174.3	2073	64.92
12/4/2020 10:32	178.3	174.3	174.7	2075	65.04
12/4/2020 10:32	178.2	174.3	174.8	2074	64.92
12/4/2020 10:32	178.2	174.4	174.8	2075	65
12/4/2020 10:32	178.2	174.5	175	2075	64.92
12/4/2020 10:32	178.2	174.3	175.1	2076	64.96
12/4/2020 10:32	178.2	174.3	176.1	2074	64.92
12/4/2020 10:32	178.3	174.3	175.4	2076	64.96
12/4/2020 10:32	178.3	174.5	175	2076	64.96
12/4/2020 10:32	178.2	174.3	175.4	2080	64.96
12/4/2020 10:32	178.2	174.3	175.4	2080	64.96
12/4/2020 10:32	178.2	174.3	175.4	2072	64.96
12/4/2020 10:32	178.1	174.5	175.2	2069	65
12/4/2020 10:32	178.1	174.4	175.4	2066	64.95
12/4/2020 10:32	178.1	174.3	175.9	2065	64.96
12/4/2020 10:32	178.1	174.4	176.5	2063	64.96
12/4/2020 10:32	178.1	174.4	175.9	2066	65.08
12/4/2020 10:32	178.1	174.3	175.5	2070	65
12/4/2020 10:32	178.1	174.4	175.6	2063	64.92
12/4/2020 10:32	178.1	174.3	175.7	2061	64.99
12/4/2020 10:32	178.1	174.3	175.7	2062	65
12/4/2020 10:32	178.1	174.3	176.1	2058	65.04
12/4/2020 10:32	178.1	174.3	176.1	2060	64.92
12/4/2020 10:32	178.1	174.3	176.1	2057	65.08
12/4/2020 10:32	178.1	174.4	176.4	2060	65.12
12/4/2020 10:32	178.1	174.4	176.3	2059	64.99
12/4/2020 10:32	178.2	174.3	176.1	2058	65.04
12/4/2020 10:32	178.2	174.3	176.5	2057	64.88

12/4/2020 10:32	178.3	174.3	176.5	2056	65.04
12/4/2020 10:32	178.3	174.5	176.5	2054	64.99
12/4/2020 10:32	178.3	174.3	176.6	2055	64.96
12/4/2020 10:32	178.3	174.3	176.5	2055	65.04
12/4/2020 10:32	178.5	174.3	176.8	2053	65
12/4/2020 10:32	178.5	174.3	176.6	2054	64.91
12/4/2020 10:32	178.5	174.3	176.5	2057	64.99
12/4/2020 10:32	178.6	174.3	176.5	2055	64.88
12/4/2020 10:33	178.7	174.3	176.6	2054	64.99
12/4/2020 10:33	178.8	174.4	176.9	2053	64.83
12/4/2020 10:33	178.8	174.3	177.5	2051	64.88
12/4/2020 10:33	178.9	174.3	177.4	2047	64.88
12/4/2020 10:33	179	174.4	176.8	2046	65.08
12/4/2020 10:33	179.1	174.4	177	2050	64.91
12/4/2020 10:33	179.1	174.5	177.1	2051	64.95
12/4/2020 10:33	179.2	174.4	177.4	2053	64.96
12/4/2020 10:33	179.3	174.3	177	2051	65.08
12/4/2020 10:33	179.5	174.3	176.8	2048	64.99
12/4/2020 10:33	179.6	174.4	177.1	2046	64.87
12/4/2020 10:33	179.6	174.4	177.8	2048	64.99
12/4/2020 10:33	179.8	174.3	177.6	2045	65.04
12/4/2020 10:33	179.9	174.3	177.2	2051	64.95
12/4/2020 10:33	180.1	173.3	177.4	2055	64.92
12/4/2020 10:33	180.2	173.4	177.5	2054	64.99
12/4/2020 10:33	180.3	174.3	177.5	2054	65.12
12/4/2020 10:33	180.4	173.2	177.3	2053	65.08
12/4/2020 10:33	180.5	173.5	177.3	2048	65.08
12/4/2020 10:33	180.8	173.4	177.4	2050	65.04
12/4/2020 10:33	180.7	173.5	177.3	2049	65.04
12/4/2020 10:33	181	173.1	177.3	2057	65.01
12/4/2020 10:33	181.1	173	177.4	2058	64.96
12/4/2020 10:33	181.5	173.3	177.5	2054	65.04
12/4/2020 10:33	181.6	173.1	177.8	2055	64.92
12/4/2020 10:33	181.6	173.4	178	2055	65.12
12/4/2020 10:33	181.8	174.4	178	2053	64.92
12/4/2020 10:33	181.9	173.3	178.2	2051	65
12/4/2020 10:33	182.3	174.4	178	2053	65.04
12/4/2020 10:33	182.4	174.5	177.8	2048	65.05
12/4/2020 10:33	182.4	174.3	177.6	2052	65.04
12/4/2020 10:33	182.6	174.4	177.6	2053	65.04
12/4/2020 10:33	182.7	174.3	177.8	2053	65
12/4/2020 10:33	183.2	174.3	177.7	2057	65
12/4/2020 10:33	183.2	174.3	177.9	2052	65.08
12/4/2020 10:33	183.3	174.3	178	2053	65.08
12/4/2020 10:33	183.4	174.4	178.3	2058	65.04
12/4/2020 10:33	183.5	174.5	178.2	2058	65.04
12/4/2020 10:33	183.7	174.3	178.1	2061	64.96

12/4/2020 10:33	183.8	174.3	178.1	2057	64.93
12/4/2020 10:33	184	174.3	178.3	2062	64.93
12/4/2020 10:33	184.1	174.3	179	2064	64.88
12/4/2020 10:33	184.4	174.3	178.3	2060	64.92
12/4/2020 10:33	184.5	174.3	178	2062	65
12/4/2020 10:33	184.6	174.3	178.4	2064	65.04
12/4/2020 10:33	184.6	174.3	178.3	2059	65.08
12/4/2020 10:33	184.7	174.3	178.1	2058	64.96
12/4/2020 10:33	185	174.3	178.6	2057	64.96
12/4/2020 10:33	184.9	174.3	178.8	2054	65.08
12/4/2020 10:33	185	174.3	178	2057	65
12/4/2020 10:33	185.2	174.4	178.3	2058	64.91
12/4/2020 10:33	185.3	174.3	178.6	2057	64.96
12/4/2020 10:33	185.4	174.4	178.4	2058	65.09
12/4/2020 10:33	185.5	174.5	178.3	2055	65
12/4/2020 10:33	185.6	174.3	178.6	2055	64.92
12/4/2020 10:33	185.6	174.3	179	2058	64.96
12/4/2020 10:33	185.7	174.3	178.3	2057	65
12/4/2020 10:33	185.8	174.4	178.2	2058	65.04
12/4/2020 10:33	185.9	174.4	178.2	2060	64.92
12/4/2020 10:33	185.9	174.3	178.5	2058	64.96
12/4/2020 10:34	186	174.3	178.5	2057	65.08
12/4/2020 10:34	186.1	174.3	178.6	2058	64.96
12/4/2020 10:34	186.2	174.4	178.7	2058	64.92
12/4/2020 10:34	186.2	174.3	178.7	2057	64.84
12/4/2020 10:34	186.3	174.4	178.5	2057	64.99
12/4/2020 10:34	186.3	174.4	178.5	2057	65.08
12/4/2020 10:34	186.5	174.5	178.5	2057	64.96
12/4/2020 10:34	186.5	174.3	178.4	2059	64.88
12/4/2020 10:34	186.5	174.4	178.5	2057	64.92
12/4/2020 10:34	186.6	174.3	178.6	2057	64.88
12/4/2020 10:34	186.6	174.3	178.6	2060	64.92
12/4/2020 10:34	186.6	174.4	178.7	2060	64.92
12/4/2020 10:34	186.7	174.3	178.7	2058	64.83
12/4/2020 10:34	186.8	174.3	178.7	2062	64.99
12/4/2020 10:34	186.8	174.3	178.5	2059	65.04
12/4/2020 10:34	186.9	174.3	178.3	2055	65.04
12/4/2020 10:34	186.8	174.3	178.3	2055	64.92
12/4/2020 10:34	186.9	174.4	178.5	2056	65.08
12/4/2020 10:34	187	174.4	179.3	2058	65.08
12/4/2020 10:34	187	174.5	179	2054	65.04
12/4/2020 10:34	187	174.3	178.5	2054	64.96
12/4/2020 10:34	187	174.3	178.6	2056	64.96
12/4/2020 10:34	187.1	174.3	178.6	2055	65
12/4/2020 10:34	187.1	174.3	178.7	2057	65
12/4/2020 10:34	187.1	174.4	178.6	2059	64.96
12/4/2020 10:34	187.2	174.3	178.3	2055	64.92

12/4/2020 10:34	187.1	174.3	178.5	2055	64.96
12/4/2020 10:34	187.2	174.3	178.8	2060	65.08
12/4/2020 10:34	187.1	174.4	179.1	2058	64.96
12/4/2020 10:34	187.3	175.7	178.6	2062	64.96
12/4/2020 10:34	187.2	175.5	178.2	2062	64.88
12/4/2020 10:34	187.2	175.4	178.4	2059	64.96
12/4/2020 10:34	187.2	175.2	178.4	2057	65.12
12/4/2020 10:34	187.2	175.5	178.7	2048	65.04
12/4/2020 10:34	187.1	175.6	178.3	2048	64.88
12/4/2020 10:34	187.2	175.5	178.3	2051	65.03
12/4/2020 10:34	187.2	175.4	178.4	2055	65.12
12/4/2020 10:34	187.2	175.1	179	2055	64.96
12/4/2020 10:34	187.2	175.4	179.1	2055	64.92
12/4/2020 10:34	187.2	175.4	178.3	2055	64.88
12/4/2020 10:34	187.3	175.7	178.3	2051	65.04
12/4/2020 10:34	187.2	175.4	178.5	2051	64.92
12/4/2020 10:34	187.3	175.5	178.5	2049	64.96
12/4/2020 10:34	187.2	175.4	178.5	2053	64.88
12/4/2020 10:34	187.2	175.6	178.1	2057	65.08
12/4/2020 10:34	187.2	175.6	178.1	2057	65
12/4/2020 10:34	187.3	175.4	178.1	2057	64.96
12/4/2020 10:34	187.2	175.4	178.6	2052	64.96
12/4/2020 10:34	187.2	175.5	179.1	2052	64.96
12/4/2020 10:34	187.2	175.7	178.8	2050	65
12/4/2020 10:34	187.2	175.3	178.3	2048	65
12/4/2020 10:34	187.2	175.7	178.1	2048	64.87
12/4/2020 10:34	187.2	175.7	178.1	2048	65
12/4/2020 10:34	187.2	175.7	178.2	2045	65.04
12/4/2020 10:34	187.2	175.5	178.2	2044	65
12/4/2020 10:34	187.2	175.4	178.2	2052	64.96
12/4/2020 10:34	187.1	175.2	178.3	2054	65.04
12/4/2020 10:34	187.1	175.6	178.1	2055	64.92
12/4/2020 10:34	187.1	175.3	178.1	2057	65.05
12/4/2020 10:35	187.1	175.7	178.1	2055	64.99
12/4/2020 10:35	187	175.4	178	2055	64.96
12/4/2020 10:35	187.1	175.1	178	2050	65.04
12/4/2020 10:35	187	175.6	178.1	2051	65.01
12/4/2020 10:35	187.1	175.4	178	2052	65.04
12/4/2020 10:35	187	175.3	177.6	2053	64.96
12/4/2020 10:35	187	175.3	177.8	2052	64.92
12/4/2020 10:35	187	175.2	177.9	2053	65.04
12/4/2020 10:35	187	175.5	178.1	2056	64.96
12/4/2020 10:35	187	175.5	178.1	2054	64.92
12/4/2020 10:35	187	175.5	178.3	2049	64.88
12/4/2020 10:35	187	175.6	178.1	2051	65.12
12/4/2020 10:35	187	175.2	178.1	2049	65.08
12/4/2020 10:35	187	175.2	178.2	2050	64.84

12/4/2020 10:35	187	175.6	178.1	2055	64.88
12/4/2020 10:35	186.9	175.2	177.9	2057	64.88
12/4/2020 10:35	186.9	175.7	178	2054	65.08
12/4/2020 10:35	186.8	175.5	177.6	2055	64.92
12/4/2020 10:35	186.8	175.2	177.6	2057	65.04
12/4/2020 10:35	186.8	175.6	177.5	2060	64.96
12/4/2020 10:35	186.7	175.2	177.5	2055	65.12
12/4/2020 10:35	186.7	175.1	177.6	2055	65
12/4/2020 10:35	186.6	175.7	177.5	2050	65
12/4/2020 10:35	186.6	175.1	177.6	2048	64.92
12/4/2020 10:35	186.5	175.5	177.5	2048	65
12/4/2020 10:35	186.5	175.5	177.5	2048	65
12/4/2020 10:35	186.4	175.3	177.6	2052	65
12/4/2020 10:35	186.3	175.5	177.6	2051	65
12/4/2020 10:35	186.3	175.4	177.3	2051	64.96
12/4/2020 10:35	186.3	175.2	177.5	2051	64.92
12/4/2020 10:35	186.2	175.7	177.5	2056	65
12/4/2020 10:35	186.1	175.4	177.2	2054	64.88
12/4/2020 10:35	186.2	175.5	177.3	2051	64.96
12/4/2020 10:35	186	175.6	177	2050	64.92
12/4/2020 10:35	186	175.1	177.1	2050	65.04
12/4/2020 10:35	185.9	175.7	176.9	2049	65.12
12/4/2020 10:35	185.9	175.2	176.9	2051	65.03
12/4/2020 10:35	185.8	175.1	177.1	2049	64.83
12/4/2020 10:35	185.8	175.5	177	2049	64.84
12/4/2020 10:35	185.7	175.4	176.8	2050	65.12
12/4/2020 10:35	185.7	175.2	176.9	2048	65.08
12/4/2020 10:35	185.6	175.5	176.9	2052	65.03
12/4/2020 10:35	185.6	175.2	176.9	2055	65.04
12/4/2020 10:35	185.5	175.4	177	2051	64.96
12/4/2020 10:35	185.4	175.2	176.9	2055	64.92
12/4/2020 10:35	185.4	175.2	176.9	2052	65.08
12/4/2020 10:35	185.4	175.5	176.9	2049	64.92
12/4/2020 10:35	185.2	176.6	177	2055	65.04
12/4/2020 10:35	185.2	176.2	176.8	2054	65
12/4/2020 10:35	185.2	176.6	176.7	2055	64.92
12/4/2020 10:35	185.1	176.1	177	2053	64.92
12/4/2020 10:35	185.1	176.4	177.5	2052	64.96
12/4/2020 10:35	185	176.6	177.4	2048	65.04
12/4/2020 10:35	184.9	176.5	176.6	2048	65.04
12/4/2020 10:35	184.9	176.8	176.4	2044	64.96
12/4/2020 10:35	184.8	176.7	176.4	2042	64.92
12/4/2020 10:35	184.8	176.6	176.6	2044	65
12/4/2020 10:35	184.7	176.7	176.7	2043	65.04
12/4/2020 10:35	184.6	176.6	176.6	2046	64.96
12/4/2020 10:35	184.6	176.1	176.4	2045	64.88
12/4/2020 10:36	184.5	176.9	176.6	2048	64.96

12/4/2020 10:36	184.6	176.2	176.4	2047	65.08
12/4/2020 10:36	184.4	176.2	176.7	2048	64.96
12/4/2020 10:36	184.4	176.7	177.1	2046	64.83
12/4/2020 10:36	184.4	176	176.3	2042	64.99
12/4/2020 10:36	184.2	176.5	176	2048	65.08
12/4/2020 10:36	184.2	176.6	175.9	2046	64.96
12/4/2020 10:36	184.2	176.6	176.2	2043	64.96
12/4/2020 10:36	184.2	176.6	176.4	2037	64.96
12/4/2020 10:36	184	176.4	176.3	2038	64.88
12/4/2020 10:36	184	176.3	175.9	2041	64.96
12/4/2020 10:36	184	176.9	176.2	2047	64.84
12/4/2020 10:36	183.9	176.6	176.8	2044	65.03
12/4/2020 10:36	183.8	176.7	176.2	2040	64.91
12/4/2020 10:36	183.7	176.8	175.9	2046	65.12
12/4/2020 10:36	183.8	176.2	175.8	2043	65.03
12/4/2020 10:36	183.7	176.8	175.9	2043	64.88
12/4/2020 10:36	183.7	176.2	175.9	2044	65.03
12/4/2020 10:36	183.6	176.8	175.9	2047	65.04
12/4/2020 10:36	183.6	176.8	176.8	2042	65.04
12/4/2020 10:36	183.5	176.1	175.9	2044	65.08
12/4/2020 10:36	183.4	176.2	175.4	2047	64.96
12/4/2020 10:36	183.4	176.6	175.9	2047	65.04
12/4/2020 10:36	183.3	176.4	175.6	2044	65.08
12/4/2020 10:36	183.3	176.8	175.7	2045	64.92
12/4/2020 10:36	183.2	176.7	175.7	2041	64.91
12/4/2020 10:36	183.1	176.8	176.3	2041	64.96
12/4/2020 10:36	183.1	176.8	175.4	2037	64.99
12/4/2020 10:36	183.1	176.2	175.4	2039	65.08
12/4/2020 10:36	183.1	176.2	175.5	2041	64.99
12/4/2020 10:36	183	176.6	175.7	2042	65.04
12/4/2020 10:36	183	176.1	175.5	2041	65.04
12/4/2020 10:36	182.9	176.7	175.6	2035	65.04
12/4/2020 10:36	182.9	176.2	176.1	2039	64.88
12/4/2020 10:36	182.9	176.2	175	2037	64.88
12/4/2020 10:36	182.8	176.7	175	2043	64.99
12/4/2020 10:36	182.7	176.4	175.4	2044	65
12/4/2020 10:36	182.7	175.6	175.1	2046	64.96
12/4/2020 10:36	182.6	175.4	175.1	2041	64.88
12/4/2020 10:36	182.6	175.7	175	2040	64.96
12/4/2020 10:36	182.5	175.6	175.9	2041	65
12/4/2020 10:36	182.5	175.2	175.4	2037	64.96
12/4/2020 10:36	182.4	175.3	175	2044	64.96
12/4/2020 10:36	182.4	175.7	174.8	2039	64.96
12/4/2020 10:36	182.3	175.4	175.1	2040	65
12/4/2020 10:36	182.3	175.7	175.3	2037	65
12/4/2020 10:36	182.2	175.5	175.1	2036	64.92
12/4/2020 10:36	182.2	175.2	175	2038	64.96

12/4/2020 10:36	182.1	175.7	174.9	2037	64.92
12/4/2020 10:36	182.1	175.1	175.6	2035	65
12/4/2020 10:36	182	175.4	175.5	2033	64.96
12/4/2020 10:36	182	175.5	174.8	2035	64.96
12/4/2020 10:36	182	175.5	174.7	2031	64.96
12/4/2020 10:36	182	175.6	174.7	2031	65.04
12/4/2020 10:36	181.9	175.5	174.7	2035	65
12/4/2020 10:36	181.9	175.1	174.7	2031	64.92
12/4/2020 10:36	181.8	175.4	175	2032	64.95
12/4/2020 10:36	181.9	175.2	174.6	2037	65.04
12/4/2020 10:36	181.8	175.7	174.9	2036	65
12/4/2020 10:37	181.7	175.6	175.4	2039	65
12/4/2020 10:37	181.7	175.1	175.1	2036	64.88
12/4/2020 10:37	181.7	175.6	174.4	2035	64.92
12/4/2020 10:37	181.7	175.1	174.3	2039	65.04
12/4/2020 10:37	181.6	175.6	174.3	2036	64.96
12/4/2020 10:37	181.6	175.5	174.7	2032	64.92
12/4/2020 10:37	181.6	175.4	174.5	2029	65.04
12/4/2020 10:37	181.6	175.3	174.4	2033	65.04
12/4/2020 10:37	181.5	175.3	174.5	2035	64.96
12/4/2020 10:37	181.5	175.1	174.2	2028	64.99
12/4/2020 10:37	181.5	175.4	174	2030	65
12/4/2020 10:37	181.5	175.5	174.5	2031	64.92
12/4/2020 10:37	181.5	175.2	174.7	2033	65.16
12/4/2020 10:37	181.5	175.6	175.2	2035	64.92
12/4/2020 10:37	181.5	175.4	175.3	2036	64.88
12/4/2020 10:37	181.5	175.1	174.3	2036	64.96
12/4/2020 10:37	181.5	175.6	174.3	2034	65.05
12/4/2020 10:37	181.5	175.2	173.7	2033	65.04
12/4/2020 10:37	181.6	175.3	173.8	2030	64.92
12/4/2020 10:37	181.5	175.4	174.1	2029	65.04
12/4/2020 10:37	181.6	175.1	174.3	2029	65.04
12/4/2020 10:37	181.5	175.5	174	2028	65.04
12/4/2020 10:37	181.6	175.2	174	2033	64.96
12/4/2020 10:37	181.6	175.5	173.8	2033	64.96
12/4/2020 10:37	181.6	175.7	174.1	2033	65.04
12/4/2020 10:37	181.6	175.4	174.2	2037	65.05
12/4/2020 10:37	181.6	175.4	174.1	2033	65.08
12/4/2020 10:37	181.6	175.3	174.2	2033	65.04
12/4/2020 10:37	181.7	175.1	174	2030	64.88
12/4/2020 10:37	181.7	175.4	173.8	2037	65.05
12/4/2020 10:37	181.7	175.1	174	2037	64.96
12/4/2020 10:37	181.7	175.2	174	2036	64.88
12/4/2020 10:37	181.7	175.6	174	2037	64.96
12/4/2020 10:37	181.8	175.2	174.5	2034	65.01
12/4/2020 10:37	181.8	175.2	174.6	2035	64.93
12/4/2020 10:37	181.8	175.4	174.7	2033	65.04

12/4/2020 10:37	181.8	175.2	174.7	2027	64.88
12/4/2020 10:37	181.9	175.4	174.5	2028	65.01
12/4/2020 10:37	181.8	175.2	174.5	2028	64.93
12/4/2020 10:37	181.9	175.3	173.9	2029	64.92
12/4/2020 10:37	181.9	175.5	173.5	2032	65.04
12/4/2020 10:37	181.9	175.5	173.5	2026	65.12
12/4/2020 10:37	182	175.2	173.7	2024	65
12/4/2020 10:37	182	175.7	173.5	2030	65.04
12/4/2020 10:37	182	175.1	173.7	2033	65.05
12/4/2020 10:37	182	175.5	173.9	2033	64.96
12/4/2020 10:37	182.1	175.6	173.4	2036	65.04
12/4/2020 10:37	182.1	175.2	173.6	2030	64.96
12/4/2020 10:37	182.2	175.3	173.8	2035	65.04
12/4/2020 10:37	182.2	175.2	173.8	2033	64.96
12/4/2020 10:37	182.2	175.1	173.7	2035	64.96
12/4/2020 10:37	182.2	175.6	173.4	2032	65.08
12/4/2020 10:37	182.2	175.5	173.4	2030	65
12/4/2020 10:37	182.2	175.4	174.2	2032	65.04
12/4/2020 10:37	182.2	175.6	174.3	2029	65.08
12/4/2020 10:37	182.3	175.1	174.3	2029	64.88
12/4/2020 10:37	182.3	175.5	174.1	2028	65.12
12/4/2020 10:37	182.4	175.6	173.4	2031	65
12/4/2020 10:37	182.4	175.5	173.3	2032	64.88
12/4/2020 10:38	182.4	175.4	173.1	2030	65
12/4/2020 10:38	182.4	175.4	173.1	2033	65.09
12/4/2020 10:38	182.5	175.5	173.5	2035	65
12/4/2020 10:38	182.5	175.6	173.4	2031	64.92
12/4/2020 10:38	182.4	175.6	173.7	2035	64.92
12/4/2020 10:38	182.4	175.5	173.3	2035	65.04
12/4/2020 10:38	182.6	175.3	173	2037	65.09
12/4/2020 10:38	182.6	175.2	173.4	2033	64.96
12/4/2020 10:38	182.5	175.5	173.6	2030	65
12/4/2020 10:38	182.6	175.2	173.5	2033	65
12/4/2020 10:38	182.6	175.5	173.6	2033	65.08
12/4/2020 10:38	182.6	175.5	173.4	2031	64.88
12/4/2020 10:38	182.6	175.1	173.3	2027	64.92
12/4/2020 10:38	182.6	175.4	173.4	2034	64.99
12/4/2020 10:38	182.7	175.7	173.5	2031	65.08
12/4/2020 10:38	182.6	175.2	173.2	2028	64.92
12/4/2020 10:38	182.7	175.6	173.3	2025	64.99
12/4/2020 10:38	182.6	175.4	173.2	2026	64.96
12/4/2020 10:38	182.7	175.1	173.1	2025	65.08
12/4/2020 10:38	182.6	175.6	173.3	2029	64.96
12/4/2020 10:38	182.6	175.2	173.1	2030	65.04
12/4/2020 10:38	182.7	175.4	173.1	2034	65.04
12/4/2020 10:38	182.6	175.1	172.8	2033	64.96
12/4/2020 10:38	182.6	175.4	173.1	2037	64.96

12/4/2020 10:38	182.5	175.5	173.1	2031	64.96
12/4/2020 10:38	182.5	175.4	173.1	2030	65
12/4/2020 10:38	182.6	175.5	173.4	2033	65.04
12/4/2020 10:38	182.4	175.4	173.7	2035	65.05
12/4/2020 10:38	182.5	175.2	173.7	2033	64.96
12/4/2020 10:38	182.4	175.2	173.2	2036	64.96
12/4/2020 10:38	182.4	175.5	173.2	2031	65
12/4/2020 10:38	182.4	175.2	172.9	2031	65.04
12/4/2020 10:38	182.4	175.6	173.3	2032	64.96
12/4/2020 10:38	182.3	175.5	173.1	2027	64.88
12/4/2020 10:38	182.3	175.1	172.7	2023	65.04
12/4/2020 10:38	182.3	175.4	173	2024	65.05
12/4/2020 10:38	182.3	175.3	172.9	2025	65.03
12/4/2020 10:38	182.2	175.7	173.6	2030	64.84
12/4/2020 10:38	182.2	175.7	173.6	2028	65.08
12/4/2020 10:38	182.1	175.3	172.8	2026	65.04
12/4/2020 10:38	182.1	175.4	172.8	2024	64.88
12/4/2020 10:38	182.1	175.7	173.1	2024	64.92
12/4/2020 10:38	182.1	175.3	172.9	2026	64.88
12/4/2020 10:38	182	175.7	173.1	2028	64.88
12/4/2020 10:38	182	175.3	172.9	2020	65.03
12/4/2020 10:38	181.9	175.6	172.6	2020	65.12
12/4/2020 10:38	182	175.4	173.1	2022	64.83
12/4/2020 10:38	181.9	175.1	173.3	2024	64.99
12/4/2020 10:38	181.9	175.2	173.4	2028	64.96
12/4/2020 10:38	181.9	175.6	173.1	2026	64.92
12/4/2020 10:38	181.8	175.2	172.7	2029	64.95
12/4/2020 10:38	181.8	175.5	172.6	2030	64.96
12/4/2020 10:38	181.8	175.5	173.1	2027	65.04
12/4/2020 10:38	181.7	175	172.9	2023	64.96
12/4/2020 10:38	181.7	175.5	172.9	2023	64.99
12/4/2020 10:38	181.7	175.2	172.8	2024	64.88
12/4/2020 10:38	181.7	175.5	172.6	2023	64.92
12/4/2020 10:38	181.6	175.7	172.9	2022	65.12
12/4/2020 10:38	181.6	175.3	172.6	2026	64.96
12/4/2020 10:38	181.6	175.7	172.7	2025	64.95
12/4/2020 10:39	181.5	175.4	172.9	2027	64.96
12/4/2020 10:39	181.5	175.4	172.4	2027	64.96
12/4/2020 10:39	181.5	175.4	172.5	2024	64.96
12/4/2020 10:39	181.5	175.1	172.7	2023	64.96
12/4/2020 10:39	181.4	175.2	172.7	2026	64.92
12/4/2020 10:39	181.4	175.3	172.8	2027	64.96
12/4/2020 10:39	181.4	175.4	173.1	2033	65.04
12/4/2020 10:39	181.3	175.5	173.4	2033	64.92
12/4/2020 10:39	181.3	175.6	173.1	2028	64.96
12/4/2020 10:39	181.3	175.2	172.4	2025	65
12/4/2020 10:39	181.3	175.5	172.3	2023	64.96

12/4/2020 10:39	181.3	175.6	172.4	2024	64.96
12/4/2020 10:39	181.2	175.2	172.7	2023	64.96
12/4/2020 10:39	181.2	175.6	172.6	2023	64.96
12/4/2020 10:39	181.2	175.4	172.4	2029	65
12/4/2020 10:39	181.1	175.3	172.6	2027	64.92
12/4/2020 10:39	181.1	175.4	173.4	2026	64.92
12/4/2020 10:39	181.1	175.3	172.4	2026	65
12/4/2020 10:39	181	175.5	172.2	2027	65
12/4/2020 10:39	181.1	175.5	172.4	2028	64.92
12/4/2020 10:39	181	175.2	172.6	2032	64.96
12/4/2020 10:39	181	175.6	172.2	2029	64.99
12/4/2020 10:39	180.9	175.2	173.1	2028	64.92
12/4/2020 10:39	180.9	175.2	172.7	2028	64.92
12/4/2020 10:39	180.9	175.5	172	2024	65.04
12/4/2020 10:39	180.9	175.4	172.5	2027	64.88
12/4/2020 10:39	180.9	175.8	172.4	2027	65
12/4/2020 10:39	180.9	175.7	172.2	2026	65.03
12/4/2020 10:39	180.9	175.4	172.3	2024	64.88
12/4/2020 10:39	180.9	175.4	172.9	2028	64.83
12/4/2020 10:39	180.9	175.2	172.8	2026	64.93
12/4/2020 10:39	180.9	175.5	172.2	2025	64.92
12/4/2020 10:39	180.8	175.6	172.3	2022	65.04
12/4/2020 10:39	180.8	175.4	172.1	2023	64.95
12/4/2020 10:39	180.8	175.6	172.2	2021	65.08
12/4/2020 10:39	180.8	175.4	172.2	2019	64.92
12/4/2020 10:39	180.7	175.2	172.9	2023	65.04
12/4/2020 10:39	180.8	175.2	172.1	2026	64.99
12/4/2020 10:39	180.7	175	172	2022	64.99
12/4/2020 10:39	180.8	175.2	172.2	2024	64.99
12/4/2020 10:39	180.7	175.4	172.3	2024	64.88
12/4/2020 10:39	180.7	175.2	172	2028	64.99
12/4/2020 10:39	180.7	175.1	172.4	2029	64.88
12/4/2020 10:39	180.7	175.7	173.1	2026	65.04
12/4/2020 10:39	180.7	175.4	172.1	2025	64.88
12/4/2020 10:39	180.7	175.6	171.7	2025	64.84
12/4/2020 10:39	180.6	175.7	172.1	2024	64.99
12/4/2020 10:39	180.7	175.1	172.1	2026	65.04
12/4/2020 10:39	180.7	175.5	172	2025	65.04
12/4/2020 10:39	180.6	175.2	172.2	2025	65.12
12/4/2020 10:39	180.7	175.2	172.6	2022	64.88
12/4/2020 10:39	180.6	175.7	171.9	2025	64.99
12/4/2020 10:39	180.6	175.5	172	2019	64.95
12/4/2020 10:39	180.6	175.7	172.2	2022	65.08
12/4/2020 10:39	180.6	175.4	172.1	2024	64.99
12/4/2020 10:39	180.5	175.1	172	2025	64.83
12/4/2020 10:39	180.6	175.5	172.3	2026	64.84
12/4/2020 10:39	180.6	175.4	172.8	2031	65.08

12/4/2020 10:39	180.5	175.5	172.5	2032	65.01
12/4/2020 10:40	180.6	175.5	171.9	2029	64.96
12/4/2020 10:40	180.5	175.2	171.7	2033	64.88
12/4/2020 10:40	180.6	175.7	172	2033	65
12/4/2020 10:40	180.5	175.7	172.2	2031	65.05
12/4/2020 10:40	180.5	175.4	172	2030	65
12/4/2020 10:40	180.5	175.6	172	2030	64.92
12/4/2020 10:40	180.5	175.1	172	2033	65
12/4/2020 10:40	180.5	175.1	172	2037	65
12/4/2020 10:40	180.3	175.5	172.5	2033	64.96
12/4/2020 10:40	180.4	175.5	172.8	2028	65
12/4/2020 10:40	180.3	175.2	172.5	2025	65
12/4/2020 10:40	180.3	175.8	172.4	2030	65.04
12/4/2020 10:40	180.2	175.4	172.6	2034	65.08
12/4/2020 10:40	180.3	175.7	172.5	2034	64.99
12/4/2020 10:40	180.2	175.5	172.6	2035	64.88
12/4/2020 10:40	180.1	175.4	172.6	2030	64.92
12/4/2020 10:40	180.1	175.5	172.4	2033	65.08
12/4/2020 10:40	180.1	175.5	172.3	2033	64.84
12/4/2020 10:40	179.9	175.7	171.7	2037	64.99
12/4/2020 10:40	179.9	175.4	171.9	2038	64.96
12/4/2020 10:40	179.8	175.4	171.9	2038	65.08
12/4/2020 10:40	179.9	175.1	171.9	2038	64.92
12/4/2020 10:40	179.8	175.1	171.9	2033	65.04
12/4/2020 10:40	179.6	175.1	171.5	2033	65.04
12/4/2020 10:40	179.6	175.5	171.9	2031	64.96
12/4/2020 10:40	179.6	175.6	172.7	2035	65.08
12/4/2020 10:40	179.5	175.7	171.9	2032	65
12/4/2020 10:40	179.4	175.6	171.6	2037	64.96
12/4/2020 10:40	179.4	175.5	171.8	2036	65.09
12/4/2020 10:40	179.4	175.1	171.9	2033	64.96
12/4/2020 10:40	179.2	175.7	171.5	2037	64.92
12/4/2020 10:40	179.2	175.1	171.7	2039	64.96
12/4/2020 10:40	179.1	175.8	171.4	2037	65.04
12/4/2020 10:40	179	175.3	171.3	2036	65
12/4/2020 10:40	178.9	175.3	172.1	2033	64.96
12/4/2020 10:40	178.9	175.5	172.4	2032	65.04
12/4/2020 10:40	178.9	175.6	172.2	2032	64.96
12/4/2020 10:40	178.8	175.2	172.1	2037	64.96
12/4/2020 10:40	178.7	175.5	171.5	2033	64.92
12/4/2020 10:40	178.7	175.5	171.4	2031	64.96
12/4/2020 10:40	178.7	175.6	171.3	2029	64.93
12/4/2020 10:40	178.7	175.4	171.6	2029	64.93
12/4/2020 10:40	178.6	175.2	171.7	2027	65
12/4/2020 10:40	178.6	175.4	171.6	2025	64.84
12/4/2020 10:40	178.6	175.5	171.4	2028	64.88
12/4/2020 10:40	178.7	175.2	171.5	2031	65.12

12/4/2020 10:40	178.6	175.4	171.5	2030	65.04
12/4/2020 10:40	178.5	175.4	171.4	2031	64.88
12/4/2020 10:40	178.5	175	172.1	2028	64.92
12/4/2020 10:40	178.6	175.7	172.3	2033	64.99
12/4/2020 10:40	178.6	175.5	171.5	2035	64.92
12/4/2020 10:40	178.6	175.1	171.4	2037	64.96
12/4/2020 10:40	178.6	175.4	171.4	2035	65
12/4/2020 10:40	178.6	175.3	171.5	2033	64.96
12/4/2020 10:40	178.6	175.6	171.4	2033	64.96
12/4/2020 10:40	178.6	175.4	171.2	2030	65
12/4/2020 10:40	178.6	175.2	172.1	2032	65
12/4/2020 10:40	178.7	175.3	171.5	2030	65
12/4/2020 10:40	178.7	175.1	171.2	2034	64.96
12/4/2020 10:40	178.6	175.2	171.5	2031	65.09
12/4/2020 10:41	178.7	175.7	171.7	2029	64.88
12/4/2020 10:41	178.8	175.4	171.2	2028	64.92
12/4/2020 10:41	178.8	175.2	171.4	2032	65.08
12/4/2020 10:41	178.8	175.6	172.2	2031	65
12/4/2020 10:41	178.8	175.2	171.5	2026	65
12/4/2020 10:41	178.8	175.6	171.2	2028	65
12/4/2020 10:41	178.9	175.5	171.4	2028	64.88
12/4/2020 10:41	178.8	175.1	171.2	2024	65.12
12/4/2020 10:41	178.9	175.5	171.2	2026	64.99
12/4/2020 10:41	178.9	175.3	171.1	2021	64.84
12/4/2020 10:41	178.9	175.2	172	2024	64.88
12/4/2020 10:41	178.9	175.7	171.5	2025	64.96
12/4/2020 10:41	178.9	175.1	170.8	2029	64.92
12/4/2020 10:41	179	174.3	171.2	2027	65
12/4/2020 10:41	179	174.4	171.2	2026	64.88
12/4/2020 10:41	179	174.3	171.2	2025	64.93
12/4/2020 10:41	179.1	174.2	171.3	2024	64.88
12/4/2020 10:41	179	174.2	172	2023	64.92
12/4/2020 10:41	179	174.3	171.6	2027	65
12/4/2020 10:41	179	174.3	171	2031	64.96
12/4/2020 10:41	179	174.3	171.3	2030	65.08
12/4/2020 10:41	178.9	174.5	171.4	2027	64.92
12/4/2020 10:41	178.9	174.5	171.1	2027	64.92
12/4/2020 10:41	178.9	174.3	171.3	2031	64.92
12/4/2020 10:41	178.9	174.4	172	2034	65.04
12/4/2020 10:41	178.9	174.3	171.2	2032	64.92
12/4/2020 10:41	178.8	174.3	170.9	2030	65
12/4/2020 10:41	178.8	174.3	171.2	2027	64.92
12/4/2020 10:41	178.8	174.3	171.2	2026	65
12/4/2020 10:41	178.8	174.3	171.1	2024	64.92
12/4/2020 10:41	178.7	174.4	171.1	2023	64.92
12/4/2020 10:41	178.8	174.3	171.7	2018	65
12/4/2020 10:41	178.7	174.4	171.2	2019	64.96

12/4/2020 10:41	178.7	174.3	171	2020	64.92
12/4/2020 10:41	178.7	174.3	171.1	2021	64.96
12/4/2020 10:41	178.8	174.3	171.2	2021	65
12/4/2020 10:41	178.7	174.3	170.9	2019	65
12/4/2020 10:41	178.8	174.3	171.4	2020	64.96
12/4/2020 10:41	178.8	174.5	171.8	2023	64.96
12/4/2020 10:41	178.8	174.4	171	2021	65.04
12/4/2020 10:41	178.8	174.3	171	2019	65.08
12/4/2020 10:41	178.8	174.2	171.2	2021	64.88
12/4/2020 10:41	178.9	174.3	171	2019	65.04
12/4/2020 10:41	178.9	174.3	170.8	2019	65.08
12/4/2020 10:41	179	174.1	171.1	2016	65.04
12/4/2020 10:41	178.9	174.3	171.7	2014	64.99
12/4/2020 10:41	179	174.5	171.2	2013	64.92
12/4/2020 10:41	179.2	174.3	170.7	2012	65.04
12/4/2020 10:41	179.2	174.3	170.8	2013	64.96
12/4/2020 10:41	179.4	174.4	171	2015	64.92
12/4/2020 10:41	179.3	174.4	171.1	2016	64.8
12/4/2020 10:41	179.4	174.3	171.1	2013	65.08
12/4/2020 10:41	179.4	174.3	171	2017	65.08
12/4/2020 10:41	179.6	174.3	171.4	2015	64.92
12/4/2020 10:41	179.8	174.3	171.5	2011	64.88
12/4/2020 10:41	179.7	174.4	171.2	2010	65.12
12/4/2020 10:41	179.9	174.3	170.9	2010	64.99
12/4/2020 10:41	180	174.3	170.7	2011	64.99
12/4/2020 10:41	180.1	174.3	171.2	2008	64.96
12/4/2020 10:42	180.2	174.3	171.2	2010	65.08
12/4/2020 10:42	180.3	174.3	170.8	2014	64.92
12/4/2020 10:42	180.4	174.3	171	2010	65.08
12/4/2020 10:42	180.6	174.3	171	2010	65
12/4/2020 10:42	180.7	174.3	171	2011	64.92
12/4/2020 10:42	180.8	174.3	171.2	2014	64.92
12/4/2020 10:42	180.9	174.2	171.3	2015	64.96
12/4/2020 10:42	181	174.3	171.7	2014	65.04
12/4/2020 10:42	181.2	174.3	171.9	2018	65
12/4/2020 10:42	181.2	174.3	171.7	2018	65
12/4/2020 10:42	181.5	174.5	171.9	2018	64.96
12/4/2020 10:42	181.6	174.3	171.7	2017	65.04
12/4/2020 10:42	181.8	174.3	171.8	2015	64.87
12/4/2020 10:42	182	174.3	171.7	2015	65
12/4/2020 10:42	181.9	175.6	171.7	2014	65
12/4/2020 10:42	182.2	175.4	171.9	2015	65
12/4/2020 10:42	182.3	175.1	171.9	2018	64.96
12/4/2020 10:42	182.8	175.2	171.6	2017	64.99
12/4/2020 10:42	182.8	175.1	171.4	2015	64.96
12/4/2020 10:42	182.9	175.2	171.2	2020	65
12/4/2020 10:42	183.1	175.2	171.1	2020	64.92

12/4/2020 10:42	183.1	175.2	171.2	2017	64.95
12/4/2020 10:42	183.6	175.5	171.2	2018	64.92
12/4/2020 10:42	183.7	175.3	171.2	2015	65.04
12/4/2020 10:42	183.7	175.2	171.2	2018	64.96
12/4/2020 10:42	183.9	175.5	171.2	2018	64.99
12/4/2020 10:42	184	175.3	170.8	2025	65.04
12/4/2020 10:42	184.5	175.3	171.3	2021	64.92
12/4/2020 10:42	184.6	175.5	171.7	2022	64.84
12/4/2020 10:42	184.6	175.2	172	2019	64.92
12/4/2020 10:42	184.7	175.3	171.9	2022	64.92
12/4/2020 10:42	184.9	175.2	171.3	2018	64.92
12/4/2020 10:42	185	175.6	171.2	2020	64.92
12/4/2020 10:42	185.1	175.5	171.4	2021	64.99
12/4/2020 10:42	185.3	175.2	171.3	2021	65
12/4/2020 10:42	185.4	175.1	171.4	2022	65.12
12/4/2020 10:42	185.5	175.2	171.5	2023	64.95
12/4/2020 10:42	185.7	175.4	171.4	2026	64.8
12/4/2020 10:42	185.8	175.2	171.7	2026	64.92
12/4/2020 10:42	185.9	175.5	171.3	2024	64.96
12/4/2020 10:42	186.1	175.2	171.2	2021	64.92
12/4/2020 10:42	186.2	175.3	171.4	2020	64.92
12/4/2020 10:42	186.2	175.1	171.1	2019	65.04
12/4/2020 10:42	186.5	175.2	171.4	2019	65
12/4/2020 10:42	186.5	175.5	171.8	2021	64.99
12/4/2020 10:42	186.6	175.4	171.7	2021	64.96
12/4/2020 10:42	186.8	175.5	172.1	2027	65.09
12/4/2020 10:42	186.8	175.6	172	2024	64.96
12/4/2020 10:42	187	175.3	171.8	2024	64.92
12/4/2020 10:42	187	175.3	171.5	2027	64.92
12/4/2020 10:42	187.1	175.6	171.3	2026	65
12/4/2020 10:42	187.1	175.3	171.3	2026	65.04
12/4/2020 10:42	187.2	175.4	171.6	2028	64.96
12/4/2020 10:42	187.3	176.7	171.7	2028	65.04
12/4/2020 10:42	187.4	176.4	171.8	2029	65
12/4/2020 10:42	187.4	176.4	171.7	2030	65
12/4/2020 10:42	187.5	176.4	171.5	2028	65.04
12/4/2020 10:42	187.5	175.9	171.6	2028	64.96
12/4/2020 10:42	187.6	176.7	172.4	2027	65.08
12/4/2020 10:43	187.7	176.6	171.9	2029	64.96
12/4/2020 10:43	187.7	176.6	171.5	2031	64.96
12/4/2020 10:43	187.7	176	171.5	2026	64.92
12/4/2020 10:43	187.9	176.2	171.7	2025	65.08
12/4/2020 10:43	187.8	176.2	171.9	2026	64.92
12/4/2020 10:43	187.9	176.1	171.7	2030	64.92
12/4/2020 10:43	187.9	176.2	172.6	2030	64.88
12/4/2020 10:43	188	176.8	171.8	2033	64.92
12/4/2020 10:43	188	176.4	171.5	2029	64.88

12/4/2020 10:43	188.1	176	171.6	2024	64.92
12/4/2020 10:43	188.2	177.1	171.9	2027	64.92
12/4/2020 10:43	188.2	175.9	171.8	2027	64.96
12/4/2020 10:43	188.2	176.1	171.4	2029	64.96
12/4/2020 10:43	188.2	176.7	172.4	2025	65.08
12/4/2020 10:43	188.3	176.2	172.3	2023	64.83
12/4/2020 10:43	188.3	176.4	171.5	2024	65.08
12/4/2020 10:43	188.3	176.6	172	2027	64.96
12/4/2020 10:43	188.4	176	171.9	2026	64.92
12/4/2020 10:43	188.3	176.6	172.1	2025	64.92
12/4/2020 10:43	188.4	176	171.8	2028	65
12/4/2020 10:43	188.4	176.1	171.8	2027	65
12/4/2020 10:43	188.5	176.7	172.4	2027	65
12/4/2020 10:43	188.4	176.2	172.5	2028	64.96
12/4/2020 10:43	188.4	176.8	172.2	2029	64.96
12/4/2020 10:43	188.5	176.4	171.7	2029	65.09
12/4/2020 10:43	188.5	176.1	171.7	2029	64.92
12/4/2020 10:43	188.4	176.4	171.7	2028	64.96
12/4/2020 10:43	188.5	175.9	172	2031	65.04
12/4/2020 10:43	188.6	177	171.9	2033	65.04
12/4/2020 10:43	188.6	177.4	172	2032	64.92
12/4/2020 10:43	188.6	177.6	172.2	2036	64.99
12/4/2020 10:43	188.7	176.9	171.9	2036	64.92
12/4/2020 10:43	188.7	177.3	172	2031	65.09
12/4/2020 10:43	188.7	176.9	172	2031	64.84
12/4/2020 10:43	188.7	177.2	172.5	2028	65.04
12/4/2020 10:43	188.7	177.5	172.9	2026	64.88
12/4/2020 10:43	188.7	177.3	172.5	2029	65.16
12/4/2020 10:43	188.7	177.1	172	2030	64.92
12/4/2020 10:43	188.7	177.6	171.9	2026	65.04
12/4/2020 10:43	188.7	176.8	171.8	2026	65.04
12/4/2020 10:43	188.7	177.1	171.7	2029	65.12
12/4/2020 10:43	188.8	176.9	171.8	2029	64.88
12/4/2020 10:43	188.7	176.9	171.8	2029	64.99
12/4/2020 10:43	188.8	177.6	171.9	2027	65.04
12/4/2020 10:43	188.7	177.3	171.7	2030	65.05
12/4/2020 10:43	188.8	177.1	171.9	2029	65.04
12/4/2020 10:43	188.8	177.6	171.9	2032	64.96
12/4/2020 10:43	188.7	177.1	171.9	2029	64.96
12/4/2020 10:43	188.8	177.5	171.7	2030	65.09
12/4/2020 10:43	188.8	177.6	172.3	2030	65
12/4/2020 10:43	188.8	177.3	172.7	2028	64.96
12/4/2020 10:43	188.8	177.4	173	2029	64.92
12/4/2020 10:43	188.9	176.8	172.4	2033	65.04
12/4/2020 10:43	188.8	176.8	172.2	2035	65.08
12/4/2020 10:43	188.8	177.4	172.1	2032	64.88
12/4/2020 10:43	188.8	177	172.2	2029	65.04

12/4/2020 10:43	188.8	177.4	172.1	2028	65.08
12/4/2020 10:43	188.8	177.5	172.1	2026	64.92
12/4/2020 10:43	188.8	177.1	172.1	2027	65.08
12/4/2020 10:43	188.8	177.4	172.9	2032	64.88
12/4/2020 10:44	188.8	177.5	172.6	2033	65.04
12/4/2020 10:44	188.8	177.3	172	2033	64.93
12/4/2020 10:44	188.7	177.4	172.4	2032	64.84
12/4/2020 10:44	188.8	177.2	172.5	2033	64.88
12/4/2020 10:44	188.8	177.1	172.2	2033	65.04
12/4/2020 10:44	188.8	177.1	172.1	2034	65
12/4/2020 10:44	188.8	177.3	172	2035	64.96
12/4/2020 10:44	188.8	177.2	172.5	2034	64.96
12/4/2020 10:44	188.8	177.5	172.9	2029	64.92
12/4/2020 10:44	188.8	178.3	172.8	2029	64.96
12/4/2020 10:44	188.8	178.4	172.1	2033	65.04
12/4/2020 10:44	188.8	178	172.5	2033	65
12/4/2020 10:44	188.8	178.1	172.6	2032	64.96
12/4/2020 10:44	188.8	178.3	172.4	2033	64.96
12/4/2020 10:44	188.8	178.3	172.4	2033	65.05
12/4/2020 10:44	188.8	178.3	172.1	2028	64.99
12/4/2020 10:44	188.8	178.5	172.1	2026	64.88
12/4/2020 10:44	188.8	178.3	172.2	2028	65.08
12/4/2020 10:44	188.8	178.4	172.6	2028	65.04
12/4/2020 10:44	188.8	178.5	173	2026	65
12/4/2020 10:44	188.8	178.2	173.2	2027	64.8
12/4/2020 10:44	188.8	178.5	172.9	2026	64.88
12/4/2020 10:44	188.7	178.2	172.7	2030	64.96
12/4/2020 10:44	188.8	178.1	172.4	2029	65.04
12/4/2020 10:44	188.7	178.3	172.2	2031	64.99
12/4/2020 10:44	188.8	178.1	172.2	2030	64.92
12/4/2020 10:44	188.8	178.4	172.2	2033	65.08
12/4/2020 10:44	188.7	178.5	172.2	2036	65.08
12/4/2020 10:44	188.8	178.1	172.4	2037	64.99
12/4/2020 10:44	188.7	178.1	172.5	2032	65.04
12/4/2020 10:44	188.7	178.5	172.4	2035	65.08
12/4/2020 10:44	188.7	178.2	172.6	2035	64.99
12/4/2020 10:44	188.7	178.5	172.3	2035	64.96
12/4/2020 10:44	188.8	178.5	172.4	2037	65.04
12/4/2020 10:44	188.7	178.1	172.6	2036	65
12/4/2020 10:44	188.8	178.3	172.3	2040	65.04
12/4/2020 10:44	188.7	178.3	172.6	2033	64.92
12/4/2020 10:44	188.7	178.5	172.7	2036	64.96
12/4/2020 10:44	188.7	178.4	172.7	2036	65.09
12/4/2020 10:44	188.7	178.1	173.3	2035	64.96
12/4/2020 10:44	188.7	178.3	173.5	2030	65
12/4/2020 10:44	188.7	178.5	172.8	2028	64.92
12/4/2020 10:44	188.7	178.3	172.5	2029	65.12

12/4/2020 10:44	188.7	178.5	172.4	2030	65.01
12/4/2020 10:44	188.7	178.4	172.1	2031	64.88
12/4/2020 10:44	188.7	178.6	172.5	2032	64.96
12/4/2020 10:44	188.7	178.3	172.4	2030	65.05
12/4/2020 10:44	188.7	178.1	172.4	2030	64.92
12/4/2020 10:44	188.7	178.2	172.4	2029	64.92
12/4/2020 10:44	188.6	178.1	173.1	2029	64.92
12/4/2020 10:44	188.6	178.1	173	2033	65.16
12/4/2020 10:44	188.7	178.5	172.1	2033	65.08
12/4/2020 10:44	188.6	178.3	172.6	2035	65.03
12/4/2020 10:44	188.6	178.1	172.8	2033	64.92
12/4/2020 10:44	188.6	178.3	172.6	2036	65.12
12/4/2020 10:44	188.5	178.3	172.7	2034	64.96
12/4/2020 10:44	188.6	178.3	173.1	2039	64.99
12/4/2020 10:44	188.6	179.4	173.5	2040	65.04
12/4/2020 10:44	188.5	179.4	172.6	2038	65
12/4/2020 10:45	188.5	179.4	172.4	2040	65
12/4/2020 10:45	188.5	179.4	172.6	2034	64.87
12/4/2020 10:45	188.5	179.3	172.9	2037	65
12/4/2020 10:45	188.5	179.4	172.7	2036	64.96
12/4/2020 10:45	188.5	179.4	172.4	2037	65
12/4/2020 10:45	188.5	179.4	172.8	2036	64.96
12/4/2020 10:45	188.5	179.2	173.4	2034	64.87
12/4/2020 10:45	188.4	179.2	173.3	2033	64.96
12/4/2020 10:45	188.5	179.4	173	2033	65
12/4/2020 10:45	188.4	179.4	172.5	2033	64.91
12/4/2020 10:45	188.5	179.4	172.4	2033	64.96
12/4/2020 10:45	188.5	179.4	172.6	2032	64.92
12/4/2020 10:45	188.4	179.3	172.7	2030	65.08
12/4/2020 10:45	188.5	179.3	172.8	2027	64.99
12/4/2020 10:45	188.4	179.3	172.9	2027	64.96
12/4/2020 10:45	188.4	179.2	173.1	2028	64.92
12/4/2020 10:45	188.3	179.4	172.8	2028	65.04
12/4/2020 10:45	188.4	179.2	173.1	2028	64.8
12/4/2020 10:45	188.4	179.5	172.8	2029	65.04
12/4/2020 10:45	188.3	179.3	173.1	2031	65.08
12/4/2020 10:45	188.4	179.2	173.1	2033	64.92
12/4/2020 10:45	188.3	179.3	173.1	2031	64.88
12/4/2020 10:45	188.3	179.5	173.1	2031	64.99
12/4/2020 10:45	188.3	179.4	172.8	2032	65.04
12/4/2020 10:45	188.3	179.4	172.9	2031	64.92
12/4/2020 10:45	188.3	179.4	173.1	2033	64.92
12/4/2020 10:45	188.3	179.4	172.8	2036	65.04
12/4/2020 10:45	188.3	179.4	172.9	2034	65.01
12/4/2020 10:45	188.3	179.3	173.1	2036	65
12/4/2020 10:45	188.3	179.5	172.8	2037	64.96
12/4/2020 10:45	188.3	179.4	173.1	2038	64.96

12/4/2020 10:45	188.3	179.4	172.8	2042	65
12/4/2020 10:45	188.3	179.5	172.4	2042	64.96
12/4/2020 10:45	188.2	179.4	172.7	2037	64.92
12/4/2020 10:45	188.2	179.3	173.1	2037	64.96
12/4/2020 10:45	188.2	179.3	173.5	2039	65.04
12/4/2020 10:45	188.2	179.4	173.8	2037	64.87
12/4/2020 10:45	188.2	179.3	173.4	2036	65
12/4/2020 10:45	188.3	179.5	172.8	2038	65.04
12/4/2020 10:45	188.3	179.5	172.9	2036	65.04
12/4/2020 10:45	188.3	179.5	172.8	2036	64.92
12/4/2020 10:45	188.2	179.3	173.2	2036	64.88
12/4/2020 10:45	188.2	179.3	173.3	2036	64.93
12/4/2020 10:45	188.2	179.4	172.9	2034	64.88
12/4/2020 10:45	188.2	179.3	173.3	2035	64.83
12/4/2020 10:45	188.2	179.4	172.8	2032	64.92
12/4/2020 10:45	188.2	179.5	172.6	2035	65.01
12/4/2020 10:45	188.2	179.2	172.7	2035	64.99
12/4/2020 10:45	188.2	179.4	172.7	2033	65.03
12/4/2020 10:45	188.1	179.1	173.4	2036	65.04
12/4/2020 10:45	188.2	179.3	173.8	2037	65.12
12/4/2020 10:45	188.1	179.5	173.8	2033	65.08
12/4/2020 10:45	188.2	179.2	173.1	2030	64.93
12/4/2020 10:45	188.2	179.4	173.1	2027	65.04
12/4/2020 10:45	188.2	179.4	173.1	2025	65.12
12/4/2020 10:45	188.1	179.3	173	2027	64.8
12/4/2020 10:45	188.1	179.3	173.4	2032	64.96
12/4/2020 10:45	188.1	179.5	173.3	2029	65
12/4/2020 10:45	188.1	179.3	173.1	2035	64.92
12/4/2020 10:45	188.2	179.4	172.9	2037	65.04
12/4/2020 10:46	188.1	179.4	172.9	2038	64.96
12/4/2020 10:46	188.1	179.4	173.8	2032	64.96
12/4/2020 10:46	188.1	179.3	173.9	2036	65
12/4/2020 10:46	188.2	179.4	173	2038	64.96
12/4/2020 10:46	188.1	179.2	173.3	2033	64.92
12/4/2020 10:46	188.1	179.5	173.2	2036	65
12/4/2020 10:46	188.1	180	173.4	2037	64.92
12/4/2020 10:46	188.1	180.6	173.3	2037	65
12/4/2020 10:46	188.1	180.6	172.8	2032	65
12/4/2020 10:46	188	180.2	173	2032	65.08
12/4/2020 10:46	188	180.2	173.3	2039	65.08
12/4/2020 10:46	188.1	180.4	173.7	2037	64.92
12/4/2020 10:46	188	180.4	174	2037	65.08
12/4/2020 10:46	188	180.8	173.5	2038	64.88
12/4/2020 10:46	188	180.4	173	2036	64.96
12/4/2020 10:46	188.1	180.6	173.4	2032	65.04
12/4/2020 10:46	188	180.2	173.1	2036	65.04
12/4/2020 10:46	188.1	180.1	173.5	2035	64.99

12/4/2020 10:46	188.1	180.6	173.4	2036	65.08
12/4/2020 10:46	188	180.7	173.2	2036	65.04
12/4/2020 10:46	188	180.1	172.9	2035	64.88
12/4/2020 10:46	188	180.5	173.1	2033	64.96
12/4/2020 10:46	188.1	180	173.1	2035	64.93
12/4/2020 10:46	188	180.1	173.8	2037	64.96
12/4/2020 10:46	188	180.6	174.3	2035	64.84
12/4/2020 10:46	188	180.2	174.2	2033	64.95
12/4/2020 10:46	188	180.3	173.8	2037	64.96
12/4/2020 10:46	188	180.1	173.5	2037	65.03
12/4/2020 10:46	187.9	180.3	173.5	2040	64.92
12/4/2020 10:46	188	180.4	173.5	2036	65
12/4/2020 10:46	188	180.5	173.3	2034	64.92
12/4/2020 10:46	188	180.1	173.1	2033	65
12/4/2020 10:46	188	180.4	173.4	2033	65.04
12/4/2020 10:46	187.9	180.2	173.3	2031	64.92
12/4/2020 10:46	188	180	173.2	2035	65.08
12/4/2020 10:46	188	180.2	173.4	2035	65
12/4/2020 10:46	188	180.1	173.4	2034	65
12/4/2020 10:46	187.9	180.4	173.5	2040	65
12/4/2020 10:46	187.9	180.6	173.6	2040	64.96
12/4/2020 10:46	187.9	180.2	173.4	2040	64.96
12/4/2020 10:46	187.9	180.6	173.6	2034	64.92
12/4/2020 10:46	187.9	180.2	173.4	2035	64.87
12/4/2020 10:46	187.9	180.1	173.4	2037	65.04
12/4/2020 10:46	187.9	180.7	173.8	2032	64.96
12/4/2020 10:46	187.9	180.4	173.8	2030	64.96
12/4/2020 10:46	187.9	180.1	173.9	2026	64.88
12/4/2020 10:46	187.9	180.6	174.4	2030	64.92
12/4/2020 10:46	187.9	180	174.3	2029	65.04
12/4/2020 10:46	187.9	180.2	174.2	2027	64.88
12/4/2020 10:46	187.8	180.1	173.6	2028	64.88
12/4/2020 10:46	187.8	180.1	173.2	2030	64.96
12/4/2020 10:46	187.8	180.6	173.7	2026	64.88
12/4/2020 10:46	187.9	180.4	173.6	2026	64.88
12/4/2020 10:46	187.9	180.1	173.4	2028	64.92
12/4/2020 10:46	187.9	180.2	173.9	2028	65.01
12/4/2020 10:46	187.8	180.1	173.6	2033	65.08
12/4/2020 10:46	187.8	180.3	173.7	2037	64.88
12/4/2020 10:46	187.8	180.6	174	2041	65.04
12/4/2020 10:46	187.8	180.1	173.9	2040	65.12
12/4/2020 10:47	187.9	180.6	174	2037	64.95
12/4/2020 10:47	187.8	180.2	173.9	2033	64.99
12/4/2020 10:47	187.8	180.3	173.6	2035	64.93
12/4/2020 10:47	187.8	180.5	173.8	2035	64.96
12/4/2020 10:47	187.8	180.2	173.7	2035	65.12
12/4/2020 10:47	187.8	180.1	173.9	2035	64.88

12/4/2020 10:47	187.8	180.6	173.9	2033	64.93
12/4/2020 10:47	187.9	180.2	174.1	2032	65.04
12/4/2020 10:47	187.9	180.7	174	2032	64.88
12/4/2020 10:47	187.9	180.1	174	2029	64.88
12/4/2020 10:47	187.9	180.4	173.6	2036	65.17
12/4/2020 10:47	188	180.6	173.4	2036	64.96
12/4/2020 10:47	187.9	180.1	173.7	2036	64.88
12/4/2020 10:47	188.1	180	173.6	2035	64.96
12/4/2020 10:47	188.1	180.6	174	2034	64.96
12/4/2020 10:47	188.1	180.2	174.7	2033	65
12/4/2020 10:47	188.1	180.2	174.5	2030	64.96
12/4/2020 10:47	188.2	180.1	174.1	2030	64.92
12/4/2020 10:47	188.2	180.3	174	2030	65
12/4/2020 10:47	188.3	180.5	174.1	2033	65
12/4/2020 10:47	188.3	180.5	174.1	2031	64.92
12/4/2020 10:47	188.3	180.3	174	2038	64.92
12/4/2020 10:47	188.4	180.5	173.8	2038	64.96
12/4/2020 10:47	188.4	180.1	173.8	2040	65
12/4/2020 10:47	188.5	180.2	174.7	2034	64.92
12/4/2020 10:47	188.6	180.6	174.5	2036	64.96
12/4/2020 10:47	188.6	180.2	174.1	2037	65.04
12/4/2020 10:47	188.7	180.7	174.1	2035	64.96
12/4/2020 10:47	188.7	180.6	174.1	2037	64.96
12/4/2020 10:47	188.8	180.1	174.5	2041	65.12
12/4/2020 10:47	188.6	180.4	174.1	2040	65.04
12/4/2020 10:47	188.9	180.2	174.1	2037	64.83
12/4/2020 10:47	188.9	180.6	173.7	2037	64.95
12/4/2020 10:47	189	181.5	174	2039	65.08
12/4/2020 10:47	189	181.6	174.7	2037	64.92
12/4/2020 10:47	189	181	175.1	2039	64.91
12/4/2020 10:47	189.1	181.1	175	2040	64.99
12/4/2020 10:47	189.2	181.1	174.7	2041	64.93
12/4/2020 10:47	189.3	181.6	174.3	2042	65.04
12/4/2020 10:47	189.3	181.6	174.2	2039	64.96
12/4/2020 10:47	189.3	181.2	174.4	2037	65.04
12/4/2020 10:47	189.4	181.7	174.3	2039	64.88
12/4/2020 10:47	189.5	181.6	174.3	2040	64.92
12/4/2020 10:47	189.5	181	174.7	2037	64.99
12/4/2020 10:47	189.6	180.9	174.9	2033	65
12/4/2020 10:47	189.6	181.5	174.4	2036	65.08
12/4/2020 10:47	189.7	181.7	174.5	2036	64.99
12/4/2020 10:47	189.8	181.3	174.5	2040	64.92
12/4/2020 10:47	189.8	181	174.6	2045	65.08
12/4/2020 10:47	189.9	181.2	174.7	2040	64.99
12/4/2020 10:47	189.9	181.1	174.7	2042	64.92
12/4/2020 10:47	190	181.1	174.4	2040	65
12/4/2020 10:47	190	181.7	174.6	2038	65

12/4/2020 10:47	190.1	181.5	174.6	2038	65
12/4/2020 10:47	190.1	181	175	2038	64.92
12/4/2020 10:47	190.2	181.5	175	2037	64.96
12/4/2020 10:47	190.3	181.4	175.4	2040	64.96
12/4/2020 10:47	190.3	181.8	174.9	2035	65
12/4/2020 10:47	190.4	181.5	174.3	2036	64.96
12/4/2020 10:48	190.5	181.3	174.7	2038	64.96
12/4/2020 10:48	190.5	181.5	174.7	2039	64.96
12/4/2020 10:48	190.6	181.3	175.1	2041	64.96
12/4/2020 10:48	190.7	181	174.9	2040	64.96
12/4/2020 10:48	190.7	181.7	174.8	2039	65.04
12/4/2020 10:48	190.8	181.6	174.9	2038	64.92
12/4/2020 10:48	190.8	181.8	175.7	2037	64.87
12/4/2020 10:48	190.9	181.8	174.7	2037	64.96
12/4/2020 10:48	190.9	181.1	174.6	2033	65.04
12/4/2020 10:48	190.9	181.5	175.2	2031	64.87
12/4/2020 10:48	191	181.8	175.1	2036	64.99
12/4/2020 10:48	191	181.1	174.7	2036	64.92
12/4/2020 10:48	191.1	181.7	175.6	2037	64.96
12/4/2020 10:48	191.1	181.5	175.4	2038	64.96
12/4/2020 10:48	191.1	181.3	174.7	2037	64.83
12/4/2020 10:48	191.3	181.7	175.1	2039	64.92
12/4/2020 10:48	191.4	181	175	2037	64.96
12/4/2020 10:48	191.4	181.4	175.2	2033	65
12/4/2020 10:48	191.4	181.6	175.1	2034	64.88
12/4/2020 10:48	191.6	181.4	175	2031	64.95
12/4/2020 10:48	191.6	181.5	175.9	2035	65
12/4/2020 10:48	191.6	181.4	175.5	2036	65
12/4/2020 10:48	191.6	181.1	174.8	2033	64.91
12/4/2020 10:48	191.7	181.8	175.2	2033	64.84
12/4/2020 10:48	191.8	182.1	175.2	2032	64.88
12/4/2020 10:48	191.8	182.1	175.4	2035	65.04
12/4/2020 10:48	191.9	182.5	175.5	2034	65.08
12/4/2020 10:48	191.9	182.3	175	2039	65.04
12/4/2020 10:48	191.9	182.7	175.6	2039	64.88
12/4/2020 10:48	192	182.7	176.2	2041	64.99
12/4/2020 10:48	192.1	182.1	176.1	2039	64.95
12/4/2020 10:48	192.1	182.3	175.6	2042	64.83
12/4/2020 10:48	192.1	182.1	175.1	2045	65.08
12/4/2020 10:48	192.2	182.3	175.1	2046	64.8
12/4/2020 10:48	192.1	182.6	175.4	2044	64.84
12/4/2020 10:48	192.2	182.5	175.5	2043	64.88
12/4/2020 10:48	192.2	182.7	175.5	2040	65.08
12/4/2020 10:48	192.2	182.2	175.5	2046	64.92
12/4/2020 10:48	192.2	182.1	175.5	2048	64.95
12/4/2020 10:48	192.2	182.3	175.5	2048	64.99
12/4/2020 10:48	192.1	182.1	175.6	2049	64.96

12/4/2020 10:48	192.1	182.4	175.5	2047	64.99
12/4/2020 10:48	192	182.3	175.6	2048	64.92
12/4/2020 10:48	192	182.6	175.8	2047	64.92
12/4/2020 10:48	192	182.2	175.3	2051	65.08
12/4/2020 10:48	192	182.3	175.5	2047	65.04
12/4/2020 10:48	191.9	182.1	175.6	2048	64.92
12/4/2020 10:48	191.9	182.3	175.7	2049	65.08
12/4/2020 10:48	191.8	182.5	176.6	2051	65.08
12/4/2020 10:48	191.6	182.2	176.2	2049	64.83
12/4/2020 10:48	191.6	182.7	175.7	2048	65.04
12/4/2020 10:48	191.6	182.7	175.9	2048	64.92
12/4/2020 10:48	191.4	182.1	175.8	2051	65
12/4/2020 10:48	191.3	182.5	175.9	2050	64.96
12/4/2020 10:48	191.3	182.1	175.5	2046	64.96
12/4/2020 10:48	191.2	182.2	175.4	2055	64.96
12/4/2020 10:48	191.1	182.7	176.5	2053	65.09
12/4/2020 10:48	191	182.2	176.2	2053	64.96
12/4/2020 10:48	190.9	182.7	175.9	2051	64.96
12/4/2020 10:48	190.8	182.5	175.9	2052	65.04
12/4/2020 10:49	190.8	182.1	176	2053	65
12/4/2020 10:49	190.6	182.5	175.9	2055	65.08
12/4/2020 10:49	190.5	182.4	176.1	2054	64.88
12/4/2020 10:49	190.5	182.2	175.9	2055	65.08
12/4/2020 10:49	190.2	182.3	175.9	2051	65.01
12/4/2020 10:49	190.2	182.1	175.7	2053	64.99
12/4/2020 10:49	190	182.3	176.4	2055	65.12
12/4/2020 10:49	189.9	182.7	176.4	2056	65.01
12/4/2020 10:49	189.8	182.1	176.1	2057	64.99
12/4/2020 10:49	189.6	182.4	175.9	2053	65.12
12/4/2020 10:49	189.6	182.1	176.1	2057	64.96
12/4/2020 10:49	189.4	182.2	175.8	2059	65.12
12/4/2020 10:49	189.2	182.5	175.9	2057	64.92
12/4/2020 10:49	189.2	182.2	175.7	2056	64.88
12/4/2020 10:49	189	182.2	175.6	2053	65
12/4/2020 10:49	188.9	182.7	175.5	2057	65.09
12/4/2020 10:49	188.8	182.1	176.4	2057	64.87
12/4/2020 10:49	188.7	182.4	176.4	2053	65.04
12/4/2020 10:49	188.5	182.7	176.4	2057	65.09
12/4/2020 10:49	188.5	182.1	176.2	2055	64.96
12/4/2020 10:49	188.4	182.4	175.9	2053	65.04
12/4/2020 10:49	188.1	182.2	175.7	2057	65.04
12/4/2020 10:49	188.1	182.7	175.7	2061	65
12/4/2020 10:49	188	182.6	175.8	2060	65.04
12/4/2020 10:49	187.8	182.2	175.9	2058	65.04
12/4/2020 10:49	187.7	182.3	176.1	2055	65.08
12/4/2020 10:49	187.7	182	176	2055	64.97
12/4/2020 10:49	187.4	182.1	175.9	2056	65.08

12/4/2020 10:49	187.4	182.4	175.9	2054	64.92
12/4/2020 10:49	187.2	182.6	175.9	2055	65.01
12/4/2020 10:49	187.1	182.7	175.8	2053	65.12
12/4/2020 10:49	187	182.5	175.9	2055	64.99
12/4/2020 10:49	186.8	182.1	175.8	2055	65.12
12/4/2020 10:49	186.7	182.3	176.1	2055	65.04
12/4/2020 10:49	186.6	182.7	176.5	2055	64.96
12/4/2020 10:49	186.4	182.2	176.4	2053	65.04
12/4/2020 10:49	186.3	182.3	175.9	2053	65.05
12/4/2020 10:49	186.1	182.5	175.5	2052	65.12
12/4/2020 10:49	186.1	182.2	176.1	2052	65
12/4/2020 10:49	185.9	182.4	175.9	2055	65
12/4/2020 10:49	185.9	182.2	176	2052	65
12/4/2020 10:49	185.8	182.5	175.9	2058	65.04
12/4/2020 10:49	185.6	182.6	176.3	2059	65
12/4/2020 10:49	185.5	182.4	176.2	2060	65
12/4/2020 10:49	185.5	182.7	175.5	2057	64.96
12/4/2020 10:49	185.4	182.7	175.9	2055	65.12
12/4/2020 10:49	185.3	182	176	2057	64.88
12/4/2020 10:49	185.2	182.5	175.9	2054	64.96
12/4/2020 10:49	185.1	182.6	175.7	2052	64.92
12/4/2020 10:49	185	182.6	176.8	2052	64.92
12/4/2020 10:49	184.9	182.3	175.7	2050	65.08
12/4/2020 10:49	184.9	182.3	175.7	2055	65.01
12/4/2020 10:49	184.8	182.2	176	2054	64.96
12/4/2020 10:49	184.7	182.7	176.1	2057	64.92
12/4/2020 10:49	184.6	182.1	175.8	2057	65.08
12/4/2020 10:49	184.5	182.7	175.8	2052	64.96
12/4/2020 10:49	184.5	182.1	176.5	2055	64.96
12/4/2020 10:49	184.4	182.1	176.1	2054	65
12/4/2020 10:49	184.3	182.4	175.7	2051	64.96
12/4/2020 10:50	184.2	182.2	175.8	2050	65.04
12/4/2020 10:50	184.1	182.5	176.2	2050	64.92
12/4/2020 10:50	184	182.2	175.9	2052	64.96
12/4/2020 10:50	184	182.3	175.9	2055	65.09
12/4/2020 10:50	183.9	182.7	176.6	2053	64.96
12/4/2020 10:50	183.8	182.5	176.5	2046	64.92
12/4/2020 10:50	183.8	182.1	175.5	2046	65
12/4/2020 10:50	183.7	182.3	175.7	2044	65.08
12/4/2020 10:50	183.6	182.1	175.9	2049	65.04
12/4/2020 10:50	183.6	182.5	176	2048	64.88
12/4/2020 10:50	183.5	182.7	176.1	2046	65.12
12/4/2020 10:50	183.6	182.3	175.8	2044	65.04
12/4/2020 10:50	183.6	182.3	176.1	2044	64.92
12/4/2020 10:50	183.5	182.7	176.8	2044	64.99
12/4/2020 10:50	183.4	181	175.7	2044	64.96
12/4/2020 10:50	183.3	181.4	175.5	2044	65.04

12/4/2020 10:50	183.3	181.5	175.9	2044	65.03
12/4/2020 10:50	183.4	181.2	175.8	2048	64.88
12/4/2020 10:50	183.3	181.5	176.1	2046	65.12
12/4/2020 10:50	183.3	181	175.9	2048	64.96
12/4/2020 10:50	183.3	181.4	175.9	2048	65.04
12/4/2020 10:50	183.3	181.7	175.9	2048	65.04
12/4/2020 10:50	183.3	181.7	175.8	2048	65.12
12/4/2020 10:50	183.2	181.5	176.1	2048	64.96
12/4/2020 10:50	183.2	181.4	176.1	2051	64.96
12/4/2020 10:50	183.2	180.9	176.2	2047	65.04
12/4/2020 10:50	183.3	181.3	176.1	2045	64.92
12/4/2020 10:50	183.2	181	176	2046	65.04
12/4/2020 10:50	183.2	181.6	175.9	2044	65
12/4/2020 10:50	183.2	181.3	176	2046	64.99
12/4/2020 10:50	183.2	181	176	2049	65
12/4/2020 10:50	183.3	181.5	175.9	2052	65.08
12/4/2020 10:50	183.2	181.1	176.1	2053	65.04
12/4/2020 10:50	183.2	181.5	175.7	2055	64.96
12/4/2020 10:50	183.2	181.6	175.8	2052	64.96
12/4/2020 10:50	183.3	181.5	175.9	2051	64.96
12/4/2020 10:50	183.2	181.7	175.5	2049	64.99
12/4/2020 10:50	183.3	181.3	175.8	2046	65.04
12/4/2020 10:50	183.3	181.1	176.6	2043	64.93
12/4/2020 10:50	183.3	181.4	176.7	2047	64.99
12/4/2020 10:50	183.2	181.8	176.1	2041	64.99
12/4/2020 10:50	183.3	181	176	2049	65
12/4/2020 10:50	183.3	181.6	175.9	2043	64.96
12/4/2020 10:50	183.3	181.3	176.1	2044	64.92
12/4/2020 10:50	183.3	180.9	176.1	2044	65
12/4/2020 10:50	183.3	181.3	176	2040	65.08
12/4/2020 10:50	183.3	181.2	176.1	2040	64.99
12/4/2020 10:50	183.4	181.2	175.9	2039	64.92
12/4/2020 10:50	183.3	181.8	175.7	2039	65
12/4/2020 10:50	183.4	181.1	175.9	2042	65.12
12/4/2020 10:50	183.4	181.6	175.5	2042	64.96
12/4/2020 10:50	183.4	180.9	175.8	2043	65
12/4/2020 10:50	183.4	181.3	175.9	2042	65.04
12/4/2020 10:50	183.4	181.4	175.9	2044	65.04
12/4/2020 10:50	183.4	181.1	176.5	2040	65.04
12/4/2020 10:50	183.4	181.6	176.2	2046	64.88
12/4/2020 10:50	183.3	181.8	176.6	2042	65.08
12/4/2020 10:50	183.4	181.5	176.6	2037	64.92
12/4/2020 10:50	183.3	181.6	176.6	2033	64.96
12/4/2020 10:50	183.3	181.7	176.6	2034	64.88
12/4/2020 10:51	183.3	181.1	176.9	2033	65.12
12/4/2020 10:51	183.4	181.4	177	2038	64.99
12/4/2020 10:51	183.3	181.1	176.8	2031	64.99

12/4/2020 10:51	183.3	181.1	176.9	2033	64.96
12/4/2020 10:51	183.4	181.5	176.8	2029	65.08
12/4/2020 10:51	183.4	181.1	176.7	2028	65
12/4/2020 10:51	183.5	181.2	176.4	2029	64.88
12/4/2020 10:51	183.5	181.8	175.9	2030	65.08
12/4/2020 10:51	183.5	181.2	175.9	2033	65.12
12/4/2020 10:51	183.6	182	175.7	2028	64.96
12/4/2020 10:51	183.6	181.7	175.9	2025	65
12/4/2020 10:51	183.7	181.2	176.1	2028	65.04
12/4/2020 10:51	183.9	181.6	176.1	2030	65.04
12/4/2020 10:51	183.9	181.8	175.9	2026	64.88
12/4/2020 10:51	184.1	181.1	176.4	2024	65.04
12/4/2020 10:51	184.1	181.7	176.2	2023	64.88
12/4/2020 10:51	184.2	181.3	175.8	2025	64.92
12/4/2020 10:51	184.3	181	176.2	2024	65
12/4/2020 10:51	184.4	181.7	176.7	2020	65
12/4/2020 10:51	184.5	181.1	176.6	2020	64.96
12/4/2020 10:51	184.7	181.7	175.8	2019	64.96
12/4/2020 10:51	184.8	180.9	176	2023	65
12/4/2020 10:51	184.9	181.1	176.3	2024	65
12/4/2020 10:51	185	181.8	176.4	2023	65
12/4/2020 10:51	185.5	181.4	176.2	2024	65
12/4/2020 10:51	185.5	180.9	176.2	2024	65.04
12/4/2020 10:51	185.6	181.7	177.3	2024	65
12/4/2020 10:51	185.8	181.1	176	2020	65
12/4/2020 10:51	185.9	181.2	176.1	2022	65
12/4/2020 10:51	186.3	181	176.2	2022	64.88
12/4/2020 10:51	186.5	181.4	176.4	2026	65.08
12/4/2020 10:51	186.5	181.6	176.3	2025	64.99
12/4/2020 10:51	186.7	181.6	176.5	2026	64.92
12/4/2020 10:51	186.8	181.2	177.1	2026	64.92
12/4/2020 10:51	187.3	181.7	176.4	2026	65.04
12/4/2020 10:51	187.4	181.1	176.3	2028	65.04
12/4/2020 10:51	187.5	181.8	176.7	2025	65.04
12/4/2020 10:51	187.7	181.7	176.7	2024	65.04
12/4/2020 10:51	187.8	181.1	176.6	2023	64.96
12/4/2020 10:51	188.3	181.3	176.6	2022	64.99
12/4/2020 10:51	188.4	181.7	177.1	2023	65.04
12/4/2020 10:51	188.3	181.1	177.1	2026	64.96
12/4/2020 10:51	188.5	181.5	176.4	2024	64.96
12/4/2020 10:51	188.7	181.3	176.5	2022	64.92
12/4/2020 10:51	188.8	181.8	176.8	2026	64.96
12/4/2020 10:51	189	181.4	176.7	2030	64.96
12/4/2020 10:51	189	181.3	176.8	2037	65.04
12/4/2020 10:51	189.2	181.3	176.7	2037	65
12/4/2020 10:51	189.3	181	176.8	2029	64.92
12/4/2020 10:51	189.5	181.1	177.4	2029	64.96

12/4/2020 10:51	189.6	181.6	177.5	2029	64.96
12/4/2020 10:51	189.7	181.6	176.9	2028	65
12/4/2020 10:51	189.8	180.9	176.6	2029	65.04
12/4/2020 10:51	189.9	181.7	176.7	2032	64.92
12/4/2020 10:51	190	181.1	176.9	2030	64.91
12/4/2020 10:51	190	181.6	176.9	2027	65
12/4/2020 10:51	190.2	182.7	177.1	2032	65.04
12/4/2020 10:51	190.3	182.2	177	2039	65
12/4/2020 10:51	190.4	182.7	177.2	2042	65.04
12/4/2020 10:52	190.5	182.8	177.3	2038	64.96
12/4/2020 10:52	190.5	182.1	176.9	2037	64.96
12/4/2020 10:52	190.6	182.4	177.1	2038	64.88
12/4/2020 10:52	190.7	182.1	177.2	2040	65.08
12/4/2020 10:52	190.7	182.1	177.3	2041	64.92
12/4/2020 10:52	190.7	182.5	177.1	2041	64.99
12/4/2020 10:52	190.9	182.2	177.2	2037	65.04
12/4/2020 10:52	190.9	182.2	177.2	2040	64.96
12/4/2020 10:52	190.9	182.7	177.3	2046	64.96
12/4/2020 10:52	190.9	182.1	177.4	2046	64.92
12/4/2020 10:52	190.9	182.5	177.1	2044	65.08
12/4/2020 10:52	190.9	182.7	177.3	2047	65.12
12/4/2020 10:52	190.9	182.2	177.3	2048	65.04
12/4/2020 10:52	190.9	182.5	177	2050	64.88
12/4/2020 10:52	190.9	182.5	177.2	2046	64.96
12/4/2020 10:52	190.9	182.2	177.3	2046	65.04
12/4/2020 10:52	190.8	182.3	177.5	2045	64.96
12/4/2020 10:52	190.9	182.1	177.5	2049	64.96
12/4/2020 10:52	190.9	182.4	177.3	2052	64.99
12/4/2020 10:52	190.9	182.1	177.3	2052	64.96
12/4/2020 10:52	190.8	182.4	177.3	2048	65.04
12/4/2020 10:52	190.7	182.5	177.4	2044	64.96
12/4/2020 10:52	190.7	182.4	177.4	2048	64.96
12/4/2020 10:52	190.6	182.2	177.6	2050	64.96
12/4/2020 10:52	190.6	182.3	177.5	2045	65
12/4/2020 10:52	190.6	182.3	177.6	2048	65.05
12/4/2020 10:52	190.6	182.6	177.5	2045	64.96
12/4/2020 10:52	190.5	182.5	177.4	2048	64.88
12/4/2020 10:52	190.5	182.1	177.6	2048	65.09
12/4/2020 10:52	190.4	182.3	177.5	2044	64.84
12/4/2020 10:52	190.3	182.3	177.4	2046	65
12/4/2020 10:52	190.3	182.4	177.5	2048	64.92
12/4/2020 10:52	190.2	182.7	177.5	2041	64.88
12/4/2020 10:52	190.3	182.1	177.6	2045	64.8
12/4/2020 10:52	190.1	182.2	177.4	2048	64.92
12/4/2020 10:52	190.1	182.2	177.5	2048	64.92
12/4/2020 10:52	190	182.1	177.5	2044	64.92
12/4/2020 10:52	189.9	182.2	177.5	2047	65.04

12/4/2020 10:52	189.9	182.1	177.5	2048	65.04
12/4/2020 10:52	189.8	182.4	177.5	2047	65.04
12/4/2020 10:52	189.8	182.3	177.4	2046	64.99
12/4/2020 10:52	189.7	182	177.5	2042	64.84
12/4/2020 10:52	189.6	182	178	2044	65.04
12/4/2020 10:52	189.5	182.1	178.3	2045	64.99
12/4/2020 10:52	189.5	182.1	177.8	2049	64.88
12/4/2020 10:52	189.4	182.2	177.5	2054	65
12/4/2020 10:52	189.4	182.5	177.3	2050	64.88
12/4/2020 10:52	189.3	182.7	177.7	2049	64.88
12/4/2020 10:52	189.2	182.6	177.6	2048	64.92
12/4/2020 10:52	189.2	182.3	177.5	2051	64.96
12/4/2020 10:52	189.1	182.1	177.5	2051	65
12/4/2020 10:52	189	182.1	178.1	2052	65
12/4/2020 10:52	189	182.1	177.7	2053	65
12/4/2020 10:52	188.9	182.1	177.3	2048	65
12/4/2020 10:52	188.8	182.6	177.6	2046	64.92
12/4/2020 10:52	188.7	182.1	177.6	2051	65
12/4/2020 10:52	188.7	182.3	177.6	2052	65
12/4/2020 10:52	188.7	182.2	178	2054	65
12/4/2020 10:52	188.5	182.3	178.1	2054	64.96
12/4/2020 10:53	188.5	182.7	177.4	2051	65
12/4/2020 10:53	188.3	182.1	177.4	2048	64.96
12/4/2020 10:53	188.4	182.7	177.8	2048	65
12/4/2020 10:53	188.3	182.6	177.6	2049	65.04
12/4/2020 10:53	188.2	182.3	177.7	2051	64.88
12/4/2020 10:53	188.3	182.7	178.5	2053	65.04
12/4/2020 10:53	188.1	182.5	177.4	2052	64.93
12/4/2020 10:53	188.1	182.5	177.4	2051	64.96
12/4/2020 10:53	187.9	182.7	177.5	2054	64.96
12/4/2020 10:53	187.9	182.3	177.6	2051	64.8
12/4/2020 10:53	187.8	182.3	177.6	2053	64.95
12/4/2020 10:53	187.7	182.6	177.8	2053	64.92
12/4/2020 10:53	187.7	182.1	178.3	2051	64.84
12/4/2020 10:53	187.4	182.6	177.5	2048	64.88
12/4/2020 10:53	187.4	182.1	177.3	2048	64.84
12/4/2020 10:53	187.4	182.1	177.5	2048	65.08
12/4/2020 10:53	187.3	182.7	177.8	2055	64.96
12/4/2020 10:53	187.2	182.3	177.5	2053	65.04
12/4/2020 10:53	187.2	182.5	178.1	2052	65.04
12/4/2020 10:53	187	182.6	178.2	2051	64.92
12/4/2020 10:53	187.1	182.1	177.3	2051	65.08
12/4/2020 10:53	187	182.5	177.5	2048	64.88
12/4/2020 10:53	186.9	182.1	177.4	2051	65.08
12/4/2020 10:53	186.8	182.1	177.6	2048	64.83
12/4/2020 10:53	186.7	182.6	177.5	2048	64.92
12/4/2020 10:53	186.7	182.2	177.9	2048	65

12/4/2020 10:53	186.6	182.7	178.2	2048	64.92
12/4/2020 10:53	186.5	182.3	177.3	2049	64.99
12/4/2020 10:53	186.5	182.1	177.3	2054	65
12/4/2020 10:53	186.3	182.3	177.6	2057	65
12/4/2020 10:53	186.4	182.1	177.6	2048	64.92
12/4/2020 10:53	186.3	182.5	177.5	2046	64.96
12/4/2020 10:53	186.2	182.5	177.6	2046	65
12/4/2020 10:53	186.2	182.2	178.3	2045	65.04
12/4/2020 10:53	186.1	182.1	177.6	2044	64.87
12/4/2020 10:53	186.1	182.7	177.2	2042	64.92
12/4/2020 10:53	185.9	182.2	177.4	2047	64.96
12/4/2020 10:53	185.9	182.5	177.4	2054	64.92
12/4/2020 10:53	185.8	182.6	177.5	2055	64.96
12/4/2020 10:53	185.7	182.1	177.5	2051	65
12/4/2020 10:53	185.7	182.4	177.4	2050	64.96
12/4/2020 10:53	185.6	182.5	178	2052	65.04
12/4/2020 10:53	185.5	182.3	178.1	2053	64.96
12/4/2020 10:53	185.5	182.7	177.2	2050	65.04
12/4/2020 10:53	185.4	182.3	177.1	2054	65.04
12/4/2020 10:53	185.4	182.2	177.4	2054	64.99
12/4/2020 10:53	185.2	182.6	177.5	2058	64.96
12/4/2020 10:53	185.2	182.2	177.5	2058	65.01
12/4/2020 10:53	185.2	182.6	177.5	2059	64.83
12/4/2020 10:53	185.1	182.3	177.4	2055	64.88
12/4/2020 10:53	185	182.5	177.2	2058	65.04
12/4/2020 10:53	185	182.2	177.4	2058	64.99
12/4/2020 10:53	184.9	182.2	177.8	2062	65.03
12/4/2020 10:53	184.9	182.2	178.3	2058	65.08
12/4/2020 10:53	184.8	182.1	178.1	2058	64.96
12/4/2020 10:53	184.8	182.3	178	2055	64.92
12/4/2020 10:53	184.8	182.7	177.6	2055	65.04
12/4/2020 10:53	184.6	182.5	177.4	2055	65.08
12/4/2020 10:53	184.6	182.6	177.3	2049	64.88
12/4/2020 10:53	184.5	182.2	177.1	2048	64.83
12/4/2020 10:54	184.6	182.4	177.2	2051	64.95
12/4/2020 10:54	184.4	182.5	177.3	2053	64.96
12/4/2020 10:54	184.4	182.5	177.3	2054	64.83
12/4/2020 10:54	184.4	182	177.3	2053	64.95
12/4/2020 10:54	184.2	182.3	177.6	2051	65.04
12/4/2020 10:54	184.2	182.4	177.7	2054	65.04
12/4/2020 10:54	184.1	182.3	178.2	2055	65.04
12/4/2020 10:54	184	182.3	178.1	2050	64.99
12/4/2020 10:54	184	182.2	177.9	2050	65
12/4/2020 10:54	183.9	182.3	177.5	2054	65
12/4/2020 10:54	183.8	182.7	177.3	2055	64.92
12/4/2020 10:54	183.8	182.3	177.4	2053	65
12/4/2020 10:54	183.7	182.4	177.5	2054	65.05

12/4/2020 10:54	183.7	182.2	177.4	2053	64.91
12/4/2020 10:54	183.6	182.1	177.3	2055	65
12/4/2020 10:54	183.5	182.7	177.3	2056	65.04
12/4/2020 10:54	183.5	182.1	177.1	2055	64.92
12/4/2020 10:54	183.4	182.4	177.1	2057	64.96
12/4/2020 10:54	183.4	182.8	178	2055	65
12/4/2020 10:54	183.3	182.1	177.8	2052	64.96
12/4/2020 10:54	183.2	182.7	177.5	2053	64.92
12/4/2020 10:54	183.1	182.2	177.3	2055	64.96
12/4/2020 10:54	183.1	182.1	177.4	2054	64.88
12/4/2020 10:54	183.1	182.1	177.3	2054	65.04
12/4/2020 10:54	183.1	182.3	177.4	2051	64.99
12/4/2020 10:54	182.9	182.5	177.2	2049	65.08
12/4/2020 10:54	182.9	182.7	177.1	2047	65
12/4/2020 10:54	182.8	182.5	176.8	2048	64.88
12/4/2020 10:54	182.8	182.6	177.3	2048	64.88
12/4/2020 10:54	182.7	182.1	177.5	2045	65.12
12/4/2020 10:54	182.6	182.1	177.9	2044	64.92
12/4/2020 10:54	182.6	182.5	177.8	2044	64.84
12/4/2020 10:54	182.6	181.4	177.7	2048	64.93
12/4/2020 10:54	182.5	181.6	177.6	2048	65.04
12/4/2020 10:54	182.4	181.1	177.6	2051	64.96
12/4/2020 10:54	182.3	181.1	177.4	2048	64.84
12/4/2020 10:54	182.3	181.7	177.3	2045	65.05
12/4/2020 10:54	182.2	181.3	177.3	2049	65.12
12/4/2020 10:54	182.2	181.6	177.1	2050	64.88
12/4/2020 10:54	182.1	181.5	177.3	2045	64.88
12/4/2020 10:54	182	181.1	177.6	2048	64.99
12/4/2020 10:54	182	181.7	177.6	2049	65.21
12/4/2020 10:54	181.9	181.4	177.8	2051	65.04
12/4/2020 10:54	181.9	180.9	178.1	2048	64.99
12/4/2020 10:54	181.9	181.4	177.9	2048	64.88
12/4/2020 10:54	181.8	181	177.8	2047	65
12/4/2020 10:54	181.7	181.5	177.6	2046	64.92
12/4/2020 10:54	181.7	181.8	177	2044	64.92
12/4/2020 10:54	181.6	181.2	176.8	2048	64.96
12/4/2020 10:54	181.5	181.5	176.7	2045	64.92
12/4/2020 10:54	181.6	181	176.9	2045	64.99
12/4/2020 10:54	181.5	181.2	177.3	2046	64.99
12/4/2020 10:54	181.4	181.4	177.2	2046	65
12/4/2020 10:54	181.4	181.3	177.3	2044	64.96
12/4/2020 10:54	181.4	181.6	177.1	2038	64.96
12/4/2020 10:54	181.3	181.6	177.2	2044	64.87
12/4/2020 10:54	181.3	180.9	177.8	2050	65.04
12/4/2020 10:54	181.2	181.3	176.9	2044	64.92
12/4/2020 10:54	181.1	181	176.9	2051	64.96
12/4/2020 10:55	181	181.5	177.1	2055	64.96

12/4/2020 10:55	180.9	181.6	177.2	2054	64.87
12/4/2020 10:55	180.9	181.1	176.8	2049	64.96
12/4/2020 10:55	180.9	181.3	177.6	2047	65
12/4/2020 10:55	180.7	181.1	176.9	2048	65.04
12/4/2020 10:55	180.8	181.3	176.7	2055	64.96
12/4/2020 10:55	180.6	181.6	177.1	2051	64.96
12/4/2020 10:55	180.6	181.3	177	2049	65.04
12/4/2020 10:55	180.6	180.9	176.8	2044	64.92
12/4/2020 10:55	180.4	181.6	176.9	2046	64.92
12/4/2020 10:55	180.5	181.2	177.4	2044	64.96
12/4/2020 10:55	180.4	181.7	177.2	2044	64.96
12/4/2020 10:55	180.4	181.7	176.8	2046	64.95
12/4/2020 10:55	180.3	181	176.8	2046	65.08
12/4/2020 10:55	180.3	181.3	176.8	2044	64.96
12/4/2020 10:55	180.3	181.1	176.9	2040	64.99
12/4/2020 10:55	180.1	181	176.5	2042	64.88
12/4/2020 10:55	180.1	181.5	177.3	2044	64.96
12/4/2020 10:55	180	181.2	177.2	2044	64.99
12/4/2020 10:55	180	181.6	176.6	2045	64.88
12/4/2020 10:55	179.9	181.5	176.6	2044	64.88
12/4/2020 10:55	179.9	181	176.9	2044	64.96
12/4/2020 10:55	179.8	181.6	176.8	2045	64.99
12/4/2020 10:55	179.8	180.1	176.9	2045	64.99
12/4/2020 10:55	179.7	180.1	176.7	2042	65
12/4/2020 10:55	179.7	180.5	177.2	2043	65.08
12/4/2020 10:55	179.6	179.9	177.5	2045	64.92
12/4/2020 10:55	179.6	180.6	176.5	2046	65.03
12/4/2020 10:55	179.6	180.2	176.5	2047	65.04
12/4/2020 10:55	179.4	180	176.8	2046	65.04
12/4/2020 10:55	179.4	180.6	176.6	2044	65.04
12/4/2020 10:55	179.4	180.2	176.8	2045	65.08
12/4/2020 10:55	179.4	180.6	176.5	2044	64.84
12/4/2020 10:55	179.4	180.6	176.7	2045	65.08
12/4/2020 10:55	179.4	179.9	176.8	2046	65
12/4/2020 10:55	179.3	180.2	177.3	2047	65.04
12/4/2020 10:55	179.2	180.4	176.9	2048	64.92
12/4/2020 10:55	179.2	180	176.6	2048	64.99
12/4/2020 10:55	179.1	180.4	176.4	2048	65
12/4/2020 10:55	179	180.1	176.6	2049	65.05
12/4/2020 10:55	179	180.1	177.2	2045	64.87
12/4/2020 10:55	179	180.2	176.6	2049	65
12/4/2020 10:55	178.9	180.4	176.7	2043	64.96
12/4/2020 10:55	178.9	180.6	176.7	2039	64.91
12/4/2020 10:55	178.8	180.6	177.4	2042	64.91
12/4/2020 10:55	178.8	180	176.5	2037	65.04
12/4/2020 10:55	178.8	180.6	176.4	2040	64.92
12/4/2020 10:55	178.7	180.3	176.7	2044	64.96

12/4/2020 10:55	178.6	180.2	176.7	2048	65.04
12/4/2020 10:55	178.6	180.6	176.5	2042	64.87
12/4/2020 10:55	178.6	180.2	176.6	2045	64.92
12/4/2020 10:55	178.5	180.6	177.3	2048	64.96
12/4/2020 10:55	178.5	180.5	176.4	2047	64.96
12/4/2020 10:55	178.5	180.1	176.1	2046	64.92
12/4/2020 10:55	178.5	180.2	176.6	2044	64.95
12/4/2020 10:55	178.3	180	176.4	2044	65.08
12/4/2020 10:55	178.4	180.6	176.7	2046	64.99
12/4/2020 10:55	178.3	180.6	176.6	2046	64.88
12/4/2020 10:55	178.3	180.2	176.9	2046	65.04
12/4/2020 10:55	178.3	180.5	177.1	2048	65.09
12/4/2020 10:56	178.2	180.1	176.3	2045	64.88
12/4/2020 10:56	178.2	180.6	176.2	2046	64.88
12/4/2020 10:56	178.2	180.3	176.6	2048	65.04
12/4/2020 10:56	178.1	180.2	176.4	2046	64.95
12/4/2020 10:56	178.1	180.1	176.4	2048	64.88
12/4/2020 10:56	178.1	180.1	176.2	2044	64.93
12/4/2020 10:56	178.1	179.3	176.4	2040	65.03
12/4/2020 10:56	178.1	179.4	177.1	2039	64.95
12/4/2020 10:56	178	179.1	177	2041	64.93
12/4/2020 10:56	178	179.3	176.3	2036	65.04
12/4/2020 10:56	178	179.3	176.1	2037	64.92
12/4/2020 10:56	178	179.2	176.1	2038	64.8
12/4/2020 10:56	178	179.3	176	2039	65.04
12/4/2020 10:56	178	179.2	176.1	2039	65.04
12/4/2020 10:56	178	179.4	176	2039	64.88
12/4/2020 10:56	178	179.3	176.2	2038	64.92
12/4/2020 10:56	178	179.2	176.3	2040	65.12
12/4/2020 10:56	178.1	179.3	176	2036	64.8
12/4/2020 10:56	178	179.4	176	2035	65.04
12/4/2020 10:56	178	179.2	176.2	2042	65.08
12/4/2020 10:56	178.1	179.4	176	2043	64.99
12/4/2020 10:56	178.1	179.2	175.9	2039	64.84
12/4/2020 10:56	178.1	179.1	175.9	2039	64.96
12/4/2020 10:56	178.1	179.4	175.9	2038	65
12/4/2020 10:56	178.1	179.4	176.3	2039	65.04
12/4/2020 10:56	178.2	179.2	176.6	2042	65
12/4/2020 10:56	178.2	179.1	176.8	2042	65
12/4/2020 10:56	178.2	179.2	177.1	2042	65.04
12/4/2020 10:56	178.2	179.4	176.7	2038	65
12/4/2020 10:56	178.3	179.2	176.2	2037	65.04
12/4/2020 10:56	178.2	179.3	176.1	2038	65
12/4/2020 10:56	178.3	179.5	176.1	2035	64.92
12/4/2020 10:56	178.3	179.4	176.4	2034	64.96
12/4/2020 10:56	178.3	179.2	176.1	2034	65.12
12/4/2020 10:56	178.3	179	176.1	2033	65.04

12/4/2020 10:56	178.3	179.2	176.1	2037	64.83
12/4/2020 10:56	178.4	179.4	176.1	2040	65.12
12/4/2020 10:56	178.4	179.3	175.9	2042	64.96
12/4/2020 10:56	178.5	179.3	176.4	2040	64.93
12/4/2020 10:56	178.4	179.2	176.9	2033	65.04
12/4/2020 10:56	178.4	179.2	176.2	2040	65.12
12/4/2020 10:56	178.5	179.4	176.2	2044	65.08
12/4/2020 10:56	178.5	179.2	176.2	2039	64.88
12/4/2020 10:56	178.6	179.4	176.4	2034	64.88
12/4/2020 10:56	178.6	179.4	176.4	2035	64.96
12/4/2020 10:56	178.6	179.2	175.9	2036	64.92
12/4/2020 10:56	178.6	179.4	176.3	2039	65
12/4/2020 10:56	178.7	179.2	176.9	2040	64.96
12/4/2020 10:56	178.7	179.2	176.5	2044	65.04
12/4/2020 10:56	178.7	179.4	175.9	2044	65
12/4/2020 10:56	178.8	179.3	176.2	2044	65
12/4/2020 10:56	178.7	179.3	176.2	2043	64.96
12/4/2020 10:56	178.8	179.4	176.2	2040	65.04
12/4/2020 10:56	178.8	179.2	176.1	2040	64.92
12/4/2020 10:56	178.8	179.4	175.9	2042	64.92
12/4/2020 10:56	178.9	179.2	176.8	2040	65.04
12/4/2020 10:56	178.9	179.2	176.7	2046	64.96
12/4/2020 10:56	178.8	179.4	175.9	2046	64.92
12/4/2020 10:56	178.9	179.3	176.2	2048	65
12/4/2020 10:57	178.9	179.4	176.1	2042	65
12/4/2020 10:57	179	179.5	176.1	2044	64.96
12/4/2020 10:57	179	179.3	176.2	2040	64.99
12/4/2020 10:57	179	179.5	175.9	2040	65.12
12/4/2020 10:57	179.1	179.4	175.8	2040	65.08
12/4/2020 10:57	179.1	179.2	176.6	2046	64.88
12/4/2020 10:57	179.2	179.4	176.8	2045	64.96
12/4/2020 10:57	179.2	179.3	176.6	2046	65.08
12/4/2020 10:57	179.2	179.3	175.9	2046	65.04
12/4/2020 10:57	179.3	179.3	175.9	2048	64.88
12/4/2020 10:57	179.3	179.2	176.2	2047	65.08
12/4/2020 10:57	179.4	179.2	176.1	2045	65.04
12/4/2020 10:57	179.4	178.5	176.4	2043	64.84
12/4/2020 10:57	179.4	178.3	176.2	2044	64.92
12/4/2020 10:57	179.4	178.1	176	2040	64.96
12/4/2020 10:57	179.5	178.1	176	2038	65
12/4/2020 10:57	179.5	178.1	175.9	2037	64.96
12/4/2020 10:57	179.5	178.3	175.9	2040	64.96
12/4/2020 10:57	179.6	178.3	176.1	2042	65.12
12/4/2020 10:57	179.6	178.2	176.1	2042	64.96
12/4/2020 10:57	179.7	178.1	175.7	2036	64.96
12/4/2020 10:57	179.7	178.3	175.9	2037	65.04
12/4/2020 10:57	179.8	178.2	175.9	2035	65

12/4/2020 10:57	179.8	178.4	176.2	2033	64.96
12/4/2020 10:57	179.8	178.1	176	2032	65.04
12/4/2020 10:57	179.9	178.1	175.9	2040	65
12/4/2020 10:57	179.9	178.3	176	2041	65
12/4/2020 10:57	179.9	178	176.1	2040	65
12/4/2020 10:57	179.9	178.1	176.2	2037	65.08
12/4/2020 10:57	179.9	178.3	176.1	2038	64.96
12/4/2020 10:57	180	178.2	176.3	2042	65.04
12/4/2020 10:57	180	178.3	176.2	2040	64.96
12/4/2020 10:57	180	178	176	2044	64.97
12/4/2020 10:57	180	178.1	176.1	2040	65.03
12/4/2020 10:57	180	178.3	176.8	2037	64.92
12/4/2020 10:57	179.9	178.3	176.8	2038	64.96
12/4/2020 10:57	180	178.1	176.2	2039	65.04
12/4/2020 10:57	179.9	178.3	175.7	2038	65.03
12/4/2020 10:57	179.9	178.3	175.9	2036	64.92
12/4/2020 10:57	179.9	178.5	176	2040	65.04
12/4/2020 10:57	179.9	178.3	176.1	2042	64.92
12/4/2020 10:57	179.9	178.1	176.5	2040	64.96
12/4/2020 10:57	179.8	178.3	176.1	2039	64.96
12/4/2020 10:57	179.8	178.1	176.1	2039	65.08
12/4/2020 10:57	179.8	178.3	176	2040	64.92
12/4/2020 10:57	179.7	178.5	175.7	2040	65
12/4/2020 10:57	179.8	178.3	175.9	2035	64.96
12/4/2020 10:57	179.7	178.4	175.7	2036	64.92
12/4/2020 10:57	179.8	178.3	175.9	2033	64.96
12/4/2020 10:57	179.8	178.1	176	2036	65.05
12/4/2020 10:57	179.7	178.3	176	2038	65
12/4/2020 10:57	179.7	178	176.3	2037	64.96
12/4/2020 10:57	179.7	178.3	176.2	2039	65
12/4/2020 10:57	179.8	178.4	176.1	2032	64.96
12/4/2020 10:57	179.8	178.1	176.2	2036	65.04
12/4/2020 10:57	179.7	178.3	175.9	2037	64.96
12/4/2020 10:57	179.8	178.1	175.9	2037	65.08
12/4/2020 10:57	179.8	178.1	175.9	2035	65
12/4/2020 10:57	179.8	178.3	175.9	2032	65.04
12/4/2020 10:58	179.8	178	176	2030	64.99
12/4/2020 10:58	179.9	178.3	176.1	2030	65.04
12/4/2020 10:58	179.9	178.3	175.9	2026	64.88
12/4/2020 10:58	179.9	178.2	175.8	2026	65.08
12/4/2020 10:58	180	178.3	176.2	2024	65.12
12/4/2020 10:58	180	178.4	175.9	2026	64.84
12/4/2020 10:58	180.1	178.3	176.1	2027	65.04
12/4/2020 10:58	180.1	178.3	175.9	2027	65.04
12/4/2020 10:58	180.2	178.3	175.7	2027	65.04
12/4/2020 10:58	180.2	178.1	175.5	2024	65.08
12/4/2020 10:58	180.3	178.3	175.5	2026	65.08

12/4/2020 10:58	180.3	178.2	175.7	2027	65
12/4/2020 10:58	180.4	178.4	176.4	2025	64.96
12/4/2020 10:58	180.4	178.3	176.6	2025	64.88
12/4/2020 10:58	180.5	177.9	176.4	2026	65
12/4/2020 10:58	180.5	178.4	176.7	2027	64.96
12/4/2020 10:58	180.7	178.1	176.3	2023	64.83
12/4/2020 10:58	180.7	178.3	175.9	2025	64.96
12/4/2020 10:58	180.8	178.5	175.9	2023	65.12
12/4/2020 10:58	180.9	178.2	175.9	2025	64.96
12/4/2020 10:58	180.9	178.4	175.9	2024	65
12/4/2020 10:58	181	178.2	175.9	2025	65
12/4/2020 10:58	181.1	178.2	175.8	2025	64.96
12/4/2020 10:58	181.1	178.3	175.9	2023	64.92
12/4/2020 10:58	181.3	178.1	176.1	2019	65
12/4/2020 10:58	181.3	178.4	176	2023	65
12/4/2020 10:58	181.4	178.1	176.2	2025	64.92
12/4/2020 10:58	181.6	178	175.9	2026	64.92
12/4/2020 10:58	181.6	178.1	175.9	2024	64.96
12/4/2020 10:58	181.7	178	176.1	2023	64.92
12/4/2020 10:58	181.8	178	176.1	2021	64.99
12/4/2020 10:58	181.8	178.4	175.9	2022	65.04
12/4/2020 10:58	182	178.1	176.1	2026	65.08
12/4/2020 10:58	182.1	178.3	175.9	2024	64.99
12/4/2020 10:58	182.1	178.5	175.7	2022	65.08
12/4/2020 10:58	182.2	178.3	175.9	2023	64.93
12/4/2020 10:58	182.3	178.3	176.2	2028	64.84
12/4/2020 10:58	182.6	178.3	176.7	2025	64.88
12/4/2020 10:58	182.6	178.1	176.6	2027	65.05
12/4/2020 10:58	182.7	178.3	175.7	2029	64.88
12/4/2020 10:58	182.9	178	175.8	2028	64.88
12/4/2020 10:58	183	178.3	175.9	2026	65.08
12/4/2020 10:58	183.1	178.5	175.9	2029	64.88
12/4/2020 10:58	183.1	178.4	176.1	2029	64.96
12/4/2020 10:58	183.4	178.3	175.9	2024	64.88
12/4/2020 10:58	183.5	178.3	175.9	2023	65.08
12/4/2020 10:58	183.6	178.2	176	2023	65.04
12/4/2020 10:58	183.7	178.4	176.6	2019	65
12/4/2020 10:58	183.7	178	176.5	2020	64.96
12/4/2020 10:58	183.9	178.5	175.6	2019	65
12/4/2020 10:58	184.2	178.2	175.7	2021	64.87
12/4/2020 10:58	184.2	178.1	176	2021	65
12/4/2020 10:58	184.3	178.3	176.2	2027	65
12/4/2020 10:58	184.4	178.3	176.2	2029	64.92
12/4/2020 10:58	184.5	178	176.1	2031	65
12/4/2020 10:58	184.6	178.5	176.1	2029	64.96
12/4/2020 10:58	184.6	178.1	176.4	2031	65
12/4/2020 10:58	184.8	178.3	177	2034	64.96

12/4/2020 10:58	184.9	178.4	176.4	2033	65
12/4/2020 10:58	184.9	178.2	175.9	2034	64.88
12/4/2020 10:59	185	179.2	175.9	2036	64.96
12/4/2020 10:59	185	179.2	176	2037	65.04
12/4/2020 10:59	185	179.3	176.1	2037	64.92
12/4/2020 10:59	185.1	179.2	176.2	2040	64.88
12/4/2020 10:59	185.2	179.2	176.2	2036	64.84
12/4/2020 10:59	185.2	179.1	176.4	2038	64.96
12/4/2020 10:59	185.2	179.2	176.1	2034	64.99
12/4/2020 10:59	185.3	179.3	175.9	2032	64.88
12/4/2020 10:59	185.3	179.3	176.2	2037	64.96
12/4/2020 10:59	185.3	179.3	176	2031	64.95
12/4/2020 10:59	185.3	179.2	176.1	2034	64.99
12/4/2020 10:59	185.2	179.1	176.4	2036	65.01
12/4/2020 10:59	185.3	179.3	176.2	2035	64.92
12/4/2020 10:59	185.3	179.2	176.3	2035	65.12
12/4/2020 10:59	185.3	179.2	176.4	2036	64.92
12/4/2020 10:59	185.2	179.2	176.1	2037	64.88
12/4/2020 10:59	185.2	179.2	176.2	2039	64.92
12/4/2020 10:59	185.2	179.4	175.9	2040	64.87
12/4/2020 10:59	185.1	179.2	175.8	2037	64.92
12/4/2020 10:59	185.1	179.2	176.1	2040	65
12/4/2020 10:59	185.2	179.4	177.1	2038	64.95
12/4/2020 10:59	185.1	179.3	176.2	2033	64.99
12/4/2020 10:59	185.1	179.3	176.1	2031	64.96
12/4/2020 10:59	185	179.3	176	2033	64.99
12/4/2020 10:59	185	179.2	176	2035	64.96
12/4/2020 10:59	185	179.2	176.1	2033	64.96
12/4/2020 10:59	184.9	179.2	175.7	2031	64.92
12/4/2020 10:59	184.9	179.2	176.6	2035	65.04
12/4/2020 10:59	184.8	179.4	176.8	2032	64.91
12/4/2020 10:59	184.8	179.2	176.1	2034	65.05
12/4/2020 10:59	184.8	179.1	176	2035	65
12/4/2020 10:59	184.8	179.1	176.2	2033	64.96
12/4/2020 10:59	184.6	179.4	176.1	2033	64.96
12/4/2020 10:59	184.6	179.3	176	2035	65
12/4/2020 10:59	184.6	179.4	175.7	2035	64.92
12/4/2020 10:59	184.5	179.4	176	2036	64.92
12/4/2020 10:59	184.4	179.4	176.7	2033	65
12/4/2020 10:59	184.5	179.2	176.9	2036	65
12/4/2020 10:59	184.4	179.5	176.4	2033	64.96
12/4/2020 10:59	184.4	179.3	176.2	2037	64.88
12/4/2020 10:59	184.2	179.2	176.2	2040	65
12/4/2020 10:59	184.2	179.3	176.2	2041	64.92
12/4/2020 10:59	184.2	179.2	176.3	2041	65.04
12/4/2020 10:59	184.2	179.2	175.9	2039	64.88
12/4/2020 10:59	184.2	179.3	175.9	2038	65.04

12/4/2020 10:59	184	179.2	176.9	2033	64.88
12/4/2020 10:59	184	179.2	176.4	2038	64.99
12/4/2020 10:59	184	179.1	176.1	2040	64.92
12/4/2020 10:59	183.9	179.2	176	2037	64.92
12/4/2020 10:59	183.9	179.3	175.9	2036	64.92
12/4/2020 10:59	183.9	179.3	175.9	2033	64.88
12/4/2020 10:59	183.7	179.1	176	2031	64.96
12/4/2020 10:59	183.8	179.4	175.7	2033	64.96
12/4/2020 10:59	183.7	179.2	175.9	2036	64.92
12/4/2020 10:59	183.6	179.4	176.6	2038	65.08
12/4/2020 10:59	183.7	179.3	176.4	2037	64.88
12/4/2020 10:59	183.6	179.3	176.1	2037	64.88
12/4/2020 10:59	183.6	179.3	175.9	2038	64.92
12/4/2020 10:59	183.5	179.2	176.2	2037	65
12/4/2020 11:00	183.5	179.2	176.2	2036	64.87
12/4/2020 11:00	183.4	179.3	176	2035	65.03
12/4/2020 11:00	183.4	179.2	176.1	2038	64.96
12/4/2020 11:00	183.4	179.3	175.9	2037	64.99
12/4/2020 11:00	183.3	179.2	175.5	2037	64.96
12/4/2020 11:00	183.3	179.2	175.7	2034	65
12/4/2020 11:00	183.2	179.3	176.4	2034	64.99
12/4/2020 11:00	183.1	179.2	176.6	2035	64.88
12/4/2020 11:00	183.1	179.2	176.6	2037	64.96
12/4/2020 11:00	183.1	179.4	176.1	2035	65.04
12/4/2020 11:00	183.1	179.3	175.6	2039	65
12/4/2020 11:00	183.1	179.5	175.8	2036	64.87
12/4/2020 11:00	183.1	179.1	175.8	2041	65
12/4/2020 11:00	183	179.1	175.9	2038	65.04
12/4/2020 11:00	182.9	179.3	176.1	2039	64.92
12/4/2020 11:00	182.9	179.2	175.8	2034	64.92
12/4/2020 11:00	182.8	179.2	175.7	2033	65.09
12/4/2020 11:00	182.8	179.3	175.7	2036	64.96
12/4/2020 11:00	182.7	179.2	175.4	2036	65
12/4/2020 11:00	182.7	179.3	176.1	2037	65.05
12/4/2020 11:00	182.6	179.3	176.8	2037	64.92
12/4/2020 11:00	182.6	179.2	175.9	2040	64.83
12/4/2020 11:00	182.6	179.4	175.8	2036	65.08
12/4/2020 11:00	182.5	179.1	175.9	2037	65.04
12/4/2020 11:00	182.4	179.2	176.1	2040	64.92
12/4/2020 11:00	182.4	179.2	176	2040	64.96
12/4/2020 11:00	182.4	179.3	175.5	2038	65.04
12/4/2020 11:00	182.3	179.2	175.3	2039	65.08
12/4/2020 11:00	182.3	179.2	175.9	2037	64.99
12/4/2020 11:00	182.2	179.3	176.5	2032	64.96
12/4/2020 11:00	182.2	179.2	176	2034	65.04
12/4/2020 11:00	182.2	179.2	175.7	2037	65.03
12/4/2020 11:00	182.1	179.2	175.7	2034	64.92

12/4/2020 11:00	182.1	179.2	175.9	2033	65.04
12/4/2020 11:00	182	179.1	175.5	2036	64.95
12/4/2020 11:00	182	179.2	175.4	2036	65.08
12/4/2020 11:00	182	179.2	175.4	2036	65.05
12/4/2020 11:00	181.9	179.2	176.4	2037	64.88
12/4/2020 11:00	181.9	179.4	175.8	2035	64.96
12/4/2020 11:00	181.8	179.2	175.9	2035	65
12/4/2020 11:00	181.8	179.1	175.8	2035	64.96
12/4/2020 11:00	181.7	179.2	175.7	2037	64.96
12/4/2020 11:00	181.7	179.2	175.7	2037	65.04
12/4/2020 11:00	181.7	179.4	175.1	2041	65
12/4/2020 11:00	181.7	179.2	175.2	2039	64.87
12/4/2020 11:00	181.6	179.3	175.8	2038	64.96
12/4/2020 11:00	181.6	179.4	176.1	2039	65
12/4/2020 11:00	181.5	179.4	175.6	2037	65.05
12/4/2020 11:00	181.6	179.3	175.5	2040	65.08
12/4/2020 11:00	181.5	179.1	175.8	2037	65
12/4/2020 11:00	181.4	179.3	175.6	2040	65.12
12/4/2020 11:00	181.4	179.2	175.5	2039	65.04
12/4/2020 11:00	181.3	179.2	175.1	2043	64.88
12/4/2020 11:00	181.3	179.2	175.2	2037	65.16
12/4/2020 11:00	181.2	179.2	176.1	2037	65.08
12/4/2020 11:00	181.1	179.2	176.1	2041	64.99
12/4/2020 11:00	181.2	179.4	175.6	2043	65.08
12/4/2020 11:00	181.1	179.4	175.3	2040	64.96
12/4/2020 11:00	181.1	179.1	175.6	2040	64.92
12/4/2020 11:00	181.1	179.4	175.5	2044	65.04
12/4/2020 11:01	180.9	179.3	175.6	2044	65
12/4/2020 11:01	180.9	179.2	175.6	2044	64.92
12/4/2020 11:01	181	179.2	175.1	2042	65
12/4/2020 11:01	180.9	179.2	175.1	2042	64.96
12/4/2020 11:01	180.9	179.4	175.8	2040	64.92
12/4/2020 11:01	180.8	179.2	176.1	2040	65.04
12/4/2020 11:01	180.8	179.3	175.5	2039	65
12/4/2020 11:01	180.7	179.4	175.3	2038	64.96
12/4/2020 11:01	180.7	179.2	175.3	2040	65
12/4/2020 11:01	180.7	179.2	175.7	2038	65.04
12/4/2020 11:01	180.6	179.4	175.7	2036	64.99
12/4/2020 11:01	180.7	179.2	175.5	2036	64.96
12/4/2020 11:01	180.7	179.2	175.2	2037	65.01
12/4/2020 11:01	180.6	179.2	175	2035	65.03
12/4/2020 11:01	180.5	179.4	175.1	2040	64.84
12/4/2020 11:01	180.5	179.2	175.2	2039	65.04
12/4/2020 11:01	180.5	179.1	175.4	2039	64.92
12/4/2020 11:01	180.5	179.3	175.7	2038	64.88
12/4/2020 11:01	180.4	179.2	176	2032	64.92
12/4/2020 11:01	180.4	179.2	175.6	2035	65.04

12/4/2020 11:01	180.4	179.4	175.4	2032	64.96
12/4/2020 11:01	180.3	179.1	175.1	2035	64.92
12/4/2020 11:01	180.3	179.3	175.2	2040	64.99
12/4/2020 11:01	180.3	179.2	175.4	2040	65.04
12/4/2020 11:01	180.2	179.1	175.5	2040	65
12/4/2020 11:01	180.2	179.2	175.3	2040	65
12/4/2020 11:01	180.1	179.3	175.4	2034	65.04
12/4/2020 11:01	180.1	179.1	175.2	2032	64.92
12/4/2020 11:01	180.1	179.3	175.1	2034	65
12/4/2020 11:01	180.1	179.3	175.2	2032	65.09
12/4/2020 11:01	180.1	179.2	175.2	2032	65
12/4/2020 11:01	180.1	179.2	175	2027	64.96
12/4/2020 11:01	180.1	179.2	175.1	2030	65
12/4/2020 11:01	180.1	179.4	175.1	2032	64.96
12/4/2020 11:01	180.1	179.2	175.2	2030	64.96
12/4/2020 11:01	180.1	179.4	175.5	2029	64.99
12/4/2020 11:01	180.1	179.4	175.9	2031	65
12/4/2020 11:01	180.1	179.1	175.4	2033	64.92
12/4/2020 11:01	180.1	178.1	174.9	2029	64.96
12/4/2020 11:01	180.1	178.1	175	2032	64.92
12/4/2020 11:01	180.1	178	175.2	2029	64.96
12/4/2020 11:01	180.1	178.2	175.1	2033	65.03
12/4/2020 11:01	180.1	178.3	175	2033	64.88
12/4/2020 11:01	180.1	179.3	175.7	2030	64.96
12/4/2020 11:01	180.2	178.1	175.7	2030	65.04
12/4/2020 11:01	180.2	178.1	175	2032	64.95
12/4/2020 11:01	180.1	178.3	175.5	2033	64.96
12/4/2020 11:01	180.2	178.2	175.3	2036	65.04
12/4/2020 11:01	180.2	178	175.3	2037	64.96
12/4/2020 11:01	180.3	178	175.4	2030	65
12/4/2020 11:01	180.3	178.3	175.9	2026	65
12/4/2020 11:01	180.2	178.1	175.1	2029	65.08
12/4/2020 11:01	180.3	178.1	174.9	2026	64.92
12/4/2020 11:01	180.3	178.4	174.9	2032	65
12/4/2020 11:01	180.4	178.2	175.4	2032	65.04
12/4/2020 11:01	180.3	178.3	175.2	2032	64.92
12/4/2020 11:01	180.4	178.3	175	2036	64.92
12/4/2020 11:01	180.4	178.1	176.1	2035	65.08
12/4/2020 11:01	180.5	178.3	175.1	2033	64.96
12/4/2020 11:02	180.5	178.1	174.8	2028	64.92
12/4/2020 11:02	180.5	178.3	175.3	2028	64.96
12/4/2020 11:02	180.5	178.3	175.3	2031	65.03
12/4/2020 11:02	180.4	178.1	175.1	2029	65.08
12/4/2020 11:02	180.5	178.3	175.3	2031	64.88
12/4/2020 11:02	180.5	178.3	175.5	2030	65.12
12/4/2020 11:02	180.6	178	175.4	2028	65.08
12/4/2020 11:02	180.6	178.1	174.7	2029	64.88

12/4/2020 11:02	180.7	178.1	175	2028	65.05
12/4/2020 11:02	180.6	178.6	175.1	2026	64.93
12/4/2020 11:02	180.7	178.1	174.9	2025	64.88
12/4/2020 11:02	180.6	178.1	174.8	2024	64.92
12/4/2020 11:02	180.7	178.3	174.9	2026	64.96
12/4/2020 11:02	180.7	178.1	175.3	2029	65.04
12/4/2020 11:02	180.7	178.3	175.9	2029	64.92
12/4/2020 11:02	180.7	178.4	175.3	2028	65
12/4/2020 11:02	180.8	178	174.8	2030	64.92
12/4/2020 11:02	180.8	178.2	174.7	2028	64.96
12/4/2020 11:02	180.8	178	175	2030	65.04
12/4/2020 11:02	180.8	178.1	174.9	2032	64.92
12/4/2020 11:02	180.9	178.3	174.9	2028	64.96
12/4/2020 11:02	180.9	178.1	175.1	2031	65.05
12/4/2020 11:02	180.9	178	175.4	2030	65.08
12/4/2020 11:02	180.9	178.1	175.8	2033	64.92
12/4/2020 11:02	181	178.2	174.7	2033	65.04
12/4/2020 11:02	181	178.2	174.6	2035	65.04
12/4/2020 11:02	181	178.2	175	2033	64.96
12/4/2020 11:02	181	178.1	175.1	2031	64.88
12/4/2020 11:02	181.1	178.5	175	2029	64.97
12/4/2020 11:02	181.1	178.3	175.1	2033	64.88
12/4/2020 11:02	181.1	178.1	174.9	2029	65.03
12/4/2020 11:02	181	178.3	175.2	2026	64.96
12/4/2020 11:02	181.2	178.1	176	2029	65.08
12/4/2020 11:02	181.1	178.3	174.8	2027	64.99
12/4/2020 11:02	181.1	178.3	174.7	2025	65.08
12/4/2020 11:02	181.2	178.3	174.8	2029	65.12
12/4/2020 11:02	181.2	178.1	175.2	2029	65.04
12/4/2020 11:02	181.2	178.1	175.1	2029	64.96
12/4/2020 11:02	181.2	178.2	175.2	2033	65.13
12/4/2020 11:02	181.2	178.1	175	2035	64.87
12/4/2020 11:02	181.3	178	175.7	2030	64.96
12/4/2020 11:02	181.3	178.3	175.4	2035	65.09
12/4/2020 11:02	181.3	178.1	174.6	2031	64.96
12/4/2020 11:02	181.3	178.1	174.9	2033	65
12/4/2020 11:02	181.4	178	174.9	2032	65.04
12/4/2020 11:02	181.3	178.2	175.2	2029	65
12/4/2020 11:02	181.4	178.5	175	2028	64.92
12/4/2020 11:02	181.4	178.3	174.8	2028	65.09
12/4/2020 11:02	181.4	178.1	175.3	2028	64.88
12/4/2020 11:02	181.4	178.4	175.9	2025	64.92
12/4/2020 11:02	181.4	178.3	175.2	2027	65.12
12/4/2020 11:02	181.4	178.5	174.9	2027	65.01
12/4/2020 11:02	181.4	178.1	174.7	2030	64.96
12/4/2020 11:02	181.5	178.1	174.6	2031	64.84
12/4/2020 11:02	181.5	178.3	175.1	2033	64.96

12/4/2020 11:02	181.5	178.1	175	2029	64.92
12/4/2020 11:02	181.5	178.3	175.2	2030	64.92
12/4/2020 11:02	181.6	178.3	175.2	2029	64.88
12/4/2020 11:02	181.6	178	175	2032	64.97
12/4/2020 11:02	181.6	178.3	174.9	2030	64.92
12/4/2020 11:03	181.6	178.3	175.2	2030	64.96
12/4/2020 11:03	181.6	178.5	175	2035	65.05
12/4/2020 11:03	181.5	178.4	175.1	2036	64.87
12/4/2020 11:03	181.6	178.1	175.1	2037	64.96
12/4/2020 11:03	181.6	178.3	175	2029	64.96
12/4/2020 11:03	181.6	178.3	175	2029	65
12/4/2020 11:03	181.7	178.1	174.7	2029	65
12/4/2020 11:03	181.7	178.4	174.6	2029	65.01
12/4/2020 11:03	181.7	178.1	174.8	2025	64.96
12/4/2020 11:03	181.6	178.3	174.7	2031	64.92
12/4/2020 11:03	181.7	178.1	175.3	2032	64.88
12/4/2020 11:03	181.8	178.2	175.6	2029	64.92
12/4/2020 11:03	181.7	178.3	175.4	2029	64.96
12/4/2020 11:03	181.8	178	175.4	2030	64.92
12/4/2020 11:03	181.8	178.4	174.8	2029	65
12/4/2020 11:03	181.9	178.5	174.8	2029	65.12
12/4/2020 11:03	181.9	178.1	175.1	2032	65
12/4/2020 11:03	181.9	178.3	175	2029	65.04
12/4/2020 11:03	181.9	178.1	175.2	2033	64.96
12/4/2020 11:03	181.9	178.2	175.1	2031	65.04
12/4/2020 11:03	181.9	178.4	174.7	2029	64.99
12/4/2020 11:03	181.9	178.3	174.7	2032	65.08
12/4/2020 11:03	182	178.3	175.4	2030	64.99
12/4/2020 11:03	182	178.6	175.6	2031	64.99
12/4/2020 11:03	182	178.1	175.1	2037	64.96
12/4/2020 11:03	182	178.3	175.4	2039	64.96
12/4/2020 11:03	182	178.3	175.2	2040	64.96
12/4/2020 11:03	182.1	178	175.3	2040	64.96
12/4/2020 11:03	182.1	178.3	174.9	2042	65
12/4/2020 11:03	182.2	178.2	174.8	2040	64.96
12/4/2020 11:03	182.2	178.3	175.1	2037	64.92
12/4/2020 11:03	182.2	178.5	175.7	2033	64.96
12/4/2020 11:03	182.2	178.1	175.7	2032	64.96
12/4/2020 11:03	182.2	178.5	175.4	2029	65
12/4/2020 11:03	182.2	178.1	175	2031	64.91
12/4/2020 11:03	182.2	178.1	175.2	2035	65
12/4/2020 11:03	182.3	178.5	175.4	2032	64.88
12/4/2020 11:03	182.4	178.2	174.9	2029	64.92
12/4/2020 11:03	182.4	178.3	174.9	2027	64.96
12/4/2020 11:03	182.4	178.2	175.6	2023	64.96
12/4/2020 11:03	182.4	178.1	175.9	2022	64.95
12/4/2020 11:03	182.5	179.2	175.1	2024	64.96

12/4/2020 11:03	182.6	179.4	175.1	2024	65.04
12/4/2020 11:03	182.5	179.2	175.1	2023	65.08
12/4/2020 11:03	182.6	179.1	175.1	2029	64.88
12/4/2020 11:03	182.6	179.3	175	2029	64.96
12/4/2020 11:03	182.6	179.2	174.7	2033	65.12
12/4/2020 11:03	182.6	179.4	175.7	2031	64.88
12/4/2020 11:03	182.6	179.2	175.9	2030	65.05
12/4/2020 11:03	182.7	179.1	175.1	2029	65.04
12/4/2020 11:03	182.7	179.3	175.1	2034	64.96
12/4/2020 11:03	182.8	179.2	175.1	2030	64.92
12/4/2020 11:03	182.8	179.4	175.3	2030	65.08
12/4/2020 11:03	182.7	179.3	175.1	2030	64.99
12/4/2020 11:03	182.9	179.1	174.8	2036	64.96
12/4/2020 11:03	182.9	179.3	175.2	2031	65.08
12/4/2020 11:03	182.9	179.5	175.9	2026	64.95
12/4/2020 11:03	182.9	179.2	175.3	2026	64.88
12/4/2020 11:03	182.9	179.2	175.2	2026	65.04
12/4/2020 11:04	182.9	179.2	175.2	2029	64.88
12/4/2020 11:04	182.9	179.3	175.2	2029	64.88
12/4/2020 11:04	182.9	179.2	175.3	2030	64.92
12/4/2020 11:04	182.9	179.2	175.3	2030	64.96
12/4/2020 11:04	182.9	179.4	175.1	2036	64.88
12/4/2020 11:04	182.9	179.1	175	2033	64.91
12/4/2020 11:04	183	179.3	174.9	2036	64.92
12/4/2020 11:04	183.1	179.3	174.9	2037	65
12/4/2020 11:04	183	179.2	175.1	2038	64.92
12/4/2020 11:04	183.1	179.4	175.2	2034	65.04
12/4/2020 11:04	183.1	179.4	175.7	2033	65.04
12/4/2020 11:04	183.1	179.2	175.7	2032	64.96
12/4/2020 11:04	183.1	179.2	175.9	2033	64.96
12/4/2020 11:04	183.1	179.3	176.1	2032	65.04
12/4/2020 11:04	183.1	179.2	176.1	2033	64.91
12/4/2020 11:04	183.1	179.2	176	2038	65
12/4/2020 11:04	183.1	179.2	175.8	2033	65
12/4/2020 11:04	183.1	179.2	175.5	2035	65
12/4/2020 11:04	183.1	179.2	175.4	2037	64.96
12/4/2020 11:04	183.1	179.2	175.6	2040	65.04
12/4/2020 11:04	183.1	179.1	175.4	2044	65.04
12/4/2020 11:04	183.1	179.2	175.6	2037	64.88
12/4/2020 11:04	183.1	179.2	175.6	2038	65.04
12/4/2020 11:04	183	179.3	175.4	2036	65.04
12/4/2020 11:04	183.1	179.3	175.7	2034	64.95
12/4/2020 11:04	183	179.1	175.7	2030	64.92
12/4/2020 11:04	183	179.4	175.7	2028	65
12/4/2020 11:04	182.9	179.2	175.8	2032	65.04
12/4/2020 11:04	182.9	179.2	175.7	2030	64.99
12/4/2020 11:04	182.9	179.3	175.5	2033	65.08

12/4/2020 11:04	182.9	179.2	175.9	2033	65.08
12/4/2020 11:04	182.9	179.3	175.5	2035	64.99
12/4/2020 11:04	182.9	179.2	175.2	2035	64.92
12/4/2020 11:04	182.8	179.3	175.1	2032	65
12/4/2020 11:04	182.8	179.3	175.3	2033	64.83
12/4/2020 11:04	182.8	179.2	175.5	2033	64.92
12/4/2020 11:04	182.8	179.2	175.7	2038	65.08
12/4/2020 11:04	182.7	179.2	175.3	2034	64.96
12/4/2020 11:04	182.7	179.2	175.3	2033	64.88
12/4/2020 11:04	182.6	179.1	175	2034	64.84
12/4/2020 11:04	182.6	179.2	175.1	2036	64.88
12/4/2020 11:04	182.7	179.4	175.9	2037	64.99
12/4/2020 11:04	182.7	179.3	175.9	2037	64.96
12/4/2020 11:04	182.6	179.2	175.2	2033	64.96
12/4/2020 11:04	182.6	179.2	175.4	2032	64.99
12/4/2020 11:04	182.6	179.2	175.2	2034	64.92
12/4/2020 11:04	182.5	179.2	175.4	2038	65.12
12/4/2020 11:04	182.5	179.2	175.2	2041	65
12/4/2020 11:04	182.4	179.5	175.6	2035	65
12/4/2020 11:04	182.4	179.2	176.1	2028	65
12/4/2020 11:04	182.4	179.1	175.5	2028	65
12/4/2020 11:04	182.2	179.2	175.3	2030	64.87
12/4/2020 11:04	182.4	179.2	175.5	2028	64.92
12/4/2020 11:04	182.2	179.3	175.2	2033	65.04
12/4/2020 11:04	182.2	179.2	175.2	2033	64.91
12/4/2020 11:04	182.3	179.2	175.1	2032	64.96
12/4/2020 11:04	182.2	179	175.1	2033	64.96
12/4/2020 11:04	182.2	179.2	175.6	2036	64.87
12/4/2020 11:04	182.2	179.1	176.1	2040	64.96
12/4/2020 11:05	182.2	179.2	175.6	2035	65
12/4/2020 11:05	182.2	179.3	175.4	2033	64.96
12/4/2020 11:05	182.2	179.2	175.7	2032	64.92
12/4/2020 11:05	182.1	179.4	175.5	2033	65
12/4/2020 11:05	182.1	179.2	175.4	2029	64.91
12/4/2020 11:05	182	179.4	175.2	2036	64.92
12/4/2020 11:05	182.1	179.2	175	2037	64.96
12/4/2020 11:05	182	179.1	175.1	2033	64.96
12/4/2020 11:05	182	179.2	174.9	2036	64.92
12/4/2020 11:05	182	179.1	175.1	2033	65
12/4/2020 11:05	182	179.4	175.2	2032	64.96
12/4/2020 11:05	182	179.3	175.6	2031	64.88
12/4/2020 11:05	182	179.2	175.5	2032	64.92
12/4/2020 11:05	182	179.2	175.7	2028	64.96
12/4/2020 11:05	182	179.3	175.5	2033	65
12/4/2020 11:05	181.9	179.1	175.1	2029	64.96
12/4/2020 11:05	182	179.4	175.3	2031	64.92
12/4/2020 11:05	182	179.3	175.2	2037	64.92

12/4/2020 11:05	182	179.3	175.5	2040	65.03
12/4/2020 11:05	182	179.3	175.5	2035	64.88
12/4/2020 11:05	182	179.2	175.2	2036	65.08
12/4/2020 11:05	181.9	179.2	175.3	2033	64.88
12/4/2020 11:05	182	179.2	175.1	2033	64.96
12/4/2020 11:05	182	179.2	175.1	2033	64.96
12/4/2020 11:05	182	179.3	175.4	2035	64.96
12/4/2020 11:05	182.1	179.2	175.2	2030	65.03
12/4/2020 11:05	182.1	179.2	175.1	2032	64.88
12/4/2020 11:05	182	179.2	175.4	2029	64.84
12/4/2020 11:05	182.1	179.1	175.2	2032	65.08
12/4/2020 11:05	182.1	179.2	175.5	2031	64.8
12/4/2020 11:05	182.1	179.2	175.5	2029	64.96
12/4/2020 11:05	182.1	179.2	175.4	2032	64.88
12/4/2020 11:05	182.2	179.2	175.5	2033	64.75
12/4/2020 11:05	182.2	179.1	175.6	2032	64.96
12/4/2020 11:05	182.2	179.2	175.3	2033	64.92
12/4/2020 11:05	182.2	179.2	175.5	2035	64.92
12/4/2020 11:05	182.2	179.3	175.2	2033	64.84
12/4/2020 11:05	182.2	179.4	175.5	2037	65
12/4/2020 11:05	182.2	179.1	176.1	2036	64.92
12/4/2020 11:05	182.2	179.2	176	2040	64.99
12/4/2020 11:05	182.2	179.1	175.4	2041	65.12
12/4/2020 11:05	182.2	179.1	175	2041	65
12/4/2020 11:05	182.2	179.1	175.2	2041	64.92
12/4/2020 11:05	182.2	179.2	175.2	2039	64.92
12/4/2020 11:05	182.1	179.3	175.4	2042	65.04
12/4/2020 11:05	182.1	179.2	175.2	2040	64.92
12/4/2020 11:05	182.1	179.3	175.8	2039	64.96
12/4/2020 11:05	182.1	179.4	175.7	2040	64.96
12/4/2020 11:05	182.1	179.2	174.8	2043	64.92
12/4/2020 11:05	182	179.2	175.4	2043	64.92
12/4/2020 11:05	182	179.1	175.4	2044	65
12/4/2020 11:05	182	179.2	175.5	2045	65
12/4/2020 11:05	182	179.2	175.3	2040	64.92
12/4/2020 11:05	181.9	179.2	175.9	2039	64.96
12/4/2020 11:05	181.9	179.4	175	2040	64.96
12/4/2020 11:05	181.9	179.2	175.1	2040	64.96
12/4/2020 11:05	181.8	179.2	175.2	2042	64.96
12/4/2020 11:05	181.9	179.2	175.4	2038	65.05
12/4/2020 11:05	181.9	179.1	175.3	2035	64.96
12/4/2020 11:05	181.8	179.1	175.2	2032	64.92
12/4/2020 11:06	181.8	179.4	176.1	2033	64.96
12/4/2020 11:06	181.8	179.2	175.3	2028	64.99
12/4/2020 11:06	181.8	179.1	174.9	2030	64.96
12/4/2020 11:06	181.8	179.2	175.4	2028	65.04
12/4/2020 11:06	181.8	179.2	175.3	2033	64.96

12/4/2020 11:06	181.9	179.2	175.4	2035	64.83
12/4/2020 11:06	181.8	179.1	175.2	2037	65.12
12/4/2020 11:06	181.8	179	175.2	2033	64.92
12/4/2020 11:06	181.9	179.3	175.9	2033	64.88
12/4/2020 11:06	181.8	179.2	175.7	2037	64.96
12/4/2020 11:06	181.9	179.3	174.8	2037	65.08
12/4/2020 11:06	182	179.2	175	2033	64.96
12/4/2020 11:06	181.9	179.2	175.4	2030	65.03
12/4/2020 11:06	182	179.5	175.2	2035	64.92
12/4/2020 11:06	182	179.2	175.4	2035	64.84
12/4/2020 11:06	182	179.2	175.3	2037	64.75
12/4/2020 11:06	182.1	179.2	175.1	2036	64.92
12/4/2020 11:06	182	179.1	175.4	2036	65.16
12/4/2020 11:06	182.1	179.3	175.1	2031	64.84
12/4/2020 11:06	182.2	179.1	175.6	2038	65.04
12/4/2020 11:06	182.1	179.2	175.9	2041	64.97
12/4/2020 11:06	182.2	179.4	176.1	2037	64.99
12/4/2020 11:06	182.2	179.4	176	2035	64.92
12/4/2020 11:06	182.2	179.2	175.5	2037	64.92
12/4/2020 11:06	182.3	179.1	175	2033	65.03
12/4/2020 11:06	182.3	179.3	175.1	2029	64.88
12/4/2020 11:06	182.4	179.4	175.3	2030	65.04
12/4/2020 11:06	182.4	179.1	175.4	2026	64.92
12/4/2020 11:06	182.4	179.3	175.5	2030	64.92
12/4/2020 11:06	182.4	179.2	175.4	2033	65
12/4/2020 11:06	182.4	179.1	175.4	2030	65.04
12/4/2020 11:06	182.5	179.2	175.7	2029	64.96
12/4/2020 11:06	182.5	179.2	175.3	2033	64.96
12/4/2020 11:06	182.5	179.2	175.3	2035	64.96
12/4/2020 11:06	182.6	179.2	175.7	2037	65
12/4/2020 11:06	182.5	179.3	175.5	2041	65.04
12/4/2020 11:06	182.5	179.3	175.5	2044	64.96
12/4/2020 11:06	182.5	179.2	175.4	2041	64.96
12/4/2020 11:06	182.5	179.2	175.3	2042	64.92
12/4/2020 11:06	182.6	179.4	175.5	2042	65
12/4/2020 11:06	182.5	179.1	175.1	2045	65.17
12/4/2020 11:06	182.5	179.4	175.3	2043	64.83
12/4/2020 11:06	182.5	179.2	175.5	2040	64.96
12/4/2020 11:06	182.5	179.1	175.2	2040	64.96
12/4/2020 11:06	182.4	179.2	175	2041	64.95
12/4/2020 11:06	182.4	179.2	175	2037	65
12/4/2020 11:06	182.4	179.1	175.1	2039	65.16
12/4/2020 11:06	182.4	179.4	175.2	2035	64.95
12/4/2020 11:06	182.4	179.2	176.1	2035	65.04
12/4/2020 11:06	182.4	179.3	176.2	2032	64.93
12/4/2020 11:06	182.4	179.2	175.5	2030	64.99
12/4/2020 11:06	182.4	179.2	175.5	2030	65.04

12/4/2020 11:06	182.4	179.4	175.3	2028	64.96
12/4/2020 11:06	182.4	179.1	175.6	2028	64.88
12/4/2020 11:06	182.5	179.2	175.3	2027	64.92
12/4/2020 11:06	182.5	179.3	175.1	2026	65.01
12/4/2020 11:06	182.5	179.1	175.1	2028	64.88
12/4/2020 11:06	182.5	179.3	175.3	2022	64.99
12/4/2020 11:06	182.4	179.2	175.9	2025	64.96
12/4/2020 11:07	182.6	179.2	176.1	2021	64.92
12/4/2020 11:07	182.6	179.2	175.3	2020	64.99
12/4/2020 11:07	182.7	179.2	175.6	2025	64.96
12/4/2020 11:07	182.7	179.4	175.5	2024	64.96
12/4/2020 11:07	182.7	179.2	175.5	2019	64.96
12/4/2020 11:07	182.8	179.3	175.6	2018	65.05
12/4/2020 11:07	182.9	179.2	175.4	2022	64.92
12/4/2020 11:07	182.9	179.2	175	2025	64.96
12/4/2020 11:07	182.9	179.2	175.1	2025	64.96
12/4/2020 11:07	183	179.2	175.5	2024	65
12/4/2020 11:07	183.1	179.1	176.1	2022	65
12/4/2020 11:07	183.2	179.3	176.1	2027	65
12/4/2020 11:07	183.2	179.1	175.9	2025	65
12/4/2020 11:07	183.3	179.3	175.7	2028	64.96
12/4/2020 11:07	183.4	179.2	175.9	2024	64.96
12/4/2020 11:07	183.4	179.2	175.6	2021	64.92
12/4/2020 11:07	183.6	179.2	175.9	2020	65.04
12/4/2020 11:07	183.5	179.1	175.8	2024	65
12/4/2020 11:07	183.7	179.2	175.7	2022	64.88
12/4/2020 11:07	183.8	179.1	176.1	2023	64.96
12/4/2020 11:07	183.9	179.2	176.1	2024	64.96
12/4/2020 11:07	184.1	179.2	176.2	2021	64.99
12/4/2020 11:07	184.2	179.4	175.9	2023	65.04
12/4/2020 11:07	184.3	179.2	175.6	2026	65.08
12/4/2020 11:07	184.3	179.3	175.3	2026	65.08
12/4/2020 11:07	184.4	179.1	175.3	2026	64.99
12/4/2020 11:07	184.6	179.3	175.5	2024	65.04
12/4/2020 11:07	184.6	179.2	175.7	2028	65.08
12/4/2020 11:07	184.8	179.3	175.7	2030	64.96
12/4/2020 11:07	185	179.3	175.5	2028	64.88
12/4/2020 11:07	185.1	179.2	176.2	2032	65.01
12/4/2020 11:07	185.1	179.2	175.9	2026	64.96
12/4/2020 11:07	185.2	179.3	175.4	2021	65
12/4/2020 11:07	185.5	179.2	175.7	2029	65
12/4/2020 11:07	185.6	179.4	176	2028	64.92
12/4/2020 11:07	185.7	179.1	175.5	2032	64.92
12/4/2020 11:07	185.8	179.1	175.9	2035	65
12/4/2020 11:07	185.9	179.3	176.5	2030	65
12/4/2020 11:07	186.1	180.2	175.4	2030	64.96
12/4/2020 11:07	186.3	180.6	175.9	2033	65.04

12/4/2020 11:07	186.4	180.4	175.9	2026	65
12/4/2020 11:07	186.6	180.6	176	2023	64.99
12/4/2020 11:07	186.7	180.6	175.9	2025	64.96
12/4/2020 11:07	186.8	180.2	176.3	2026	65.08
12/4/2020 11:07	187	180.6	176.6	2024	65.12
12/4/2020 11:07	187.1	180.1	175.7	2025	64.83
12/4/2020 11:07	187.2	179.9	175.7	2030	65.08
12/4/2020 11:07	187.3	180.6	176.1	2027	65.08
12/4/2020 11:07	187.4	180.1	176	2027	64.99
12/4/2020 11:07	187.6	180.1	175.7	2029	65.04
12/4/2020 11:07	187.6	180.5	176.6	2028	64.96
12/4/2020 11:07	187.7	180	176	2030	64.99
12/4/2020 11:07	187.8	180.3	175.8	2037	64.88
12/4/2020 11:07	187.9	180.2	176.3	2042	65.01
12/4/2020 11:07	188	180.5	176.1	2038	65.04
12/4/2020 11:07	188	180.2	176.1	2036	64.92
12/4/2020 11:07	188.1	180.2	176.4	2031	65
12/4/2020 11:07	188.1	180.4	176.7	2034	65.04
12/4/2020 11:07	188.2	180	176	2040	65
12/4/2020 11:08	188.3	180.4	175.9	2037	65
12/4/2020 11:08	188.3	180.4	176.1	2037	65
12/4/2020 11:08	188.4	180.2	176.1	2037	64.96
12/4/2020 11:08	188.5	180.6	176.4	2039	65
12/4/2020 11:08	188.5	180.1	177.1	2039	65.12
12/4/2020 11:08	188.5	180.3	175.9	2040	64.99
12/4/2020 11:08	188.6	180.3	175.9	2040	64.96
12/4/2020 11:08	188.5	180.4	176.3	2041	64.88
12/4/2020 11:08	188.7	180.6	176.4	2037	64.92
12/4/2020 11:08	188.7	180	175.9	2037	65.08
12/4/2020 11:08	188.8	180.2	176.7	2033	64.88
12/4/2020 11:08	188.7	180.1	176.9	2034	64.84
12/4/2020 11:08	188.8	180.1	176.1	2037	64.92
12/4/2020 11:08	188.8	180.6	176.3	2037	64.93
12/4/2020 11:08	188.9	180	176.3	2039	65.04
12/4/2020 11:08	188.8	180.4	176.5	2040	64.99
12/4/2020 11:08	189	180.1	176.6	2039	65.08
12/4/2020 11:08	189	179.9	176.6	2038	64.92
12/4/2020 11:08	189	181.2	177.2	2037	64.99
12/4/2020 11:08	189	181	176.9	2042	65.08
12/4/2020 11:08	188.9	181.6	176.1	2037	65.04
12/4/2020 11:08	188.9	181.6	176.4	2037	64.92
12/4/2020 11:08	189	181.4	176.4	2035	64.92
12/4/2020 11:08	189	181.5	176.6	2034	64.96
12/4/2020 11:08	189	181.3	176.7	2033	65.04
12/4/2020 11:08	189	181.1	176.4	2037	65
12/4/2020 11:08	189	181.6	176.4	2037	65.04
12/4/2020 11:08	189.1	180.9	177.2	2037	65

12/4/2020 11:08	189	181.5	177.3	2036	65
12/4/2020 11:08	189	181.4	176.6	2037	65
12/4/2020 11:08	189	180.9	176.5	2041	65
12/4/2020 11:08	189	181.3	176.4	2045	65.04
12/4/2020 11:08	189	181.1	176.7	2042	65.04
12/4/2020 11:08	189	181.4	176.7	2043	65.12
12/4/2020 11:08	189	181.6	176.6	2039	65.08
12/4/2020 11:08	189	181.4	176.8	2038	64.96
12/4/2020 11:08	189	181.8	176.7	2044	64.96
12/4/2020 11:08	189	181	176.7	2040	65.04
12/4/2020 11:08	189	181.3	176.9	2037	64.99
12/4/2020 11:08	188.9	180.9	176.7	2040	65.01
12/4/2020 11:08	189	181.3	177	2043	65.04
12/4/2020 11:08	189	181.6	177.1	2040	64.99
12/4/2020 11:08	189	181.2	176.7	2041	64.96
12/4/2020 11:08	188.9	181	176.7	2041	64.88
12/4/2020 11:08	188.9	181.6	176.6	2042	64.96
12/4/2020 11:08	188.9	181.3	176.3	2042	65
12/4/2020 11:08	188.9	181.6	176.9	2041	65
12/4/2020 11:08	189	181.4	177.7	2041	64.96
12/4/2020 11:08	189	181	176.6	2039	65.05
12/4/2020 11:08	188.9	181.3	176.8	2036	65.09
12/4/2020 11:08	188.9	180.9	176.7	2039	64.92
12/4/2020 11:08	189	181.5	176.7	2038	64.96
12/4/2020 11:08	189	181.3	176.5	2037	65.04
12/4/2020 11:08	188.9	180.9	176.9	2038	65
12/4/2020 11:08	189	181.8	177.6	2041	65
12/4/2020 11:08	188.9	180.9	177.2	2042	65.12
12/4/2020 11:08	189	181.4	176.8	2042	64.96
12/4/2020 11:08	188.8	180.8	177.2	2040	65.03
12/4/2020 11:08	188.9	181.4	176.9	2043	65.08
12/4/2020 11:09	188.9	181.6	176.7	2040	65
12/4/2020 11:09	188.8	181.1	176.5	2040	64.84
12/4/2020 11:09	188.9	181.4	177.2	2039	64.96
12/4/2020 11:09	188.8	181.6	177.6	2044	65.12
12/4/2020 11:09	188.8	181.5	177.2	2046	64.88
12/4/2020 11:09	188.8	181.6	176.6	2044	65.08
12/4/2020 11:09	188.8	181.2	176.8	2041	64.96
12/4/2020 11:09	188.8	180.9	177	2044	64.99
12/4/2020 11:09	188.8	181.5	177.1	2043	64.88
12/4/2020 11:09	188.7	181.3	176.9	2045	65.12
12/4/2020 11:09	188.7	181.6	176.9	2044	64.96
12/4/2020 11:09	188.7	182.6	176.5	2043	64.96
12/4/2020 11:09	188.7	182.1	177.1	2044	65.05
12/4/2020 11:09	188.7	182.5	177.8	2046	64.96
12/4/2020 11:09	188.7	182.1	177.7	2048	65
12/4/2020 11:09	188.6	182.1	177.6	2046	65.09

12/4/2020 11:09	188.7	182.6	177.3	2048	64.92
12/4/2020 11:09	188.6	182.1	176.8	2048	65
12/4/2020 11:09	188.6	182.3	176.9	2047	64.99
12/4/2020 11:09	188.5	182.1	177	2045	64.96
12/4/2020 11:09	188.6	182.1	177.2	2046	65.04
12/4/2020 11:09	188.6	182.5	177	2048	64.92
12/4/2020 11:09	188.5	182.3	177.1	2047	64.92
12/4/2020 11:09	188.6	182.5	177.2	2053	65.08
12/4/2020 11:09	188.5	182.1	177.1	2050	64.92
12/4/2020 11:09	188.5	182.1	177.2	2051	65.12
12/4/2020 11:09	188.5	182.6	177.1	2052	65.12
12/4/2020 11:09	188.5	182.1	177.1	2051	65.12
12/4/2020 11:09	188.5	182.3	176.9	2046	65.03
12/4/2020 11:09	188.5	182.1	176.9	2045	65.08
12/4/2020 11:09	188.5	182.2	176.9	2040	65.04
12/4/2020 11:09	188.5	182.5	176.8	2039	65.04
12/4/2020 11:09	188.5	182.1	176.8	2040	65.04
12/4/2020 11:09	188.5	182.6	176.9	2047	65.04
12/4/2020 11:09	188.4	182.5	176.8	2047	65.04
12/4/2020 11:09	188.4	182.1	177	2049	65
12/4/2020 11:09	188.4	182.4	177	2048	65
12/4/2020 11:09	188.4	182.3	177.1	2047	65
12/4/2020 11:09	188.4	182.2	177.8	2044	64.96
12/4/2020 11:09	188.4	182.5	177.8	2048	65.09
12/4/2020 11:09	188.4	182.2	178	2048	64.92
12/4/2020 11:09	188.3	182.2	177.8	2046	64.92
12/4/2020 11:09	188.4	182.1	178	2048	65.04
12/4/2020 11:09	188.4	182.1	177.8	2044	64.92
12/4/2020 11:09	188.3	182.5	178	2048	64.95
12/4/2020 11:09	188.3	182	177.8	2048	64.84
12/4/2020 11:09	188.3	182.5	178	2048	64.97
12/4/2020 11:09	188.3	182	178	2046	64.99
12/4/2020 11:09	188.3	182.2	178	2046	65.08
12/4/2020 11:09	188.3	182.6	178.1	2049	64.93
12/4/2020 11:09	188.3	182.2	177.8	2051	64.88
12/4/2020 11:09	188.3	182.2	177.4	2046	64.92
12/4/2020 11:09	188.3	182.1	177.3	2047	65.04
12/4/2020 11:09	188.2	182.1	176.9	2048	64.96
12/4/2020 11:09	188.3	182.5	177	2050	65.04
12/4/2020 11:09	188.2	182.1	177.3	2049	65.05
12/4/2020 11:09	188	182.5	177.4	2047	64.96
12/4/2020 11:09	188.1	182.6	177.3	2047	64.96
12/4/2020 11:09	188.2	182.1	177.5	2050	64.96
12/4/2020 11:10	188.1	182.4	177.5	2051	65.04
12/4/2020 11:10	188.1	182.1	177.1	2046	65.05
12/4/2020 11:10	188.1	182.7	177.5	2047	64.92
12/4/2020 11:10	188.1	182	178.1	2042	65

12/4/2020 11:10	188.1	181.9	177.4	2044	65
12/4/2020 11:10	188.2	182.2	177.2	2048	65.04
12/4/2020 11:10	188.1	182.4	177.4	2050	65.04
12/4/2020 11:10	188.1	182.1	177.4	2051	65.08
12/4/2020 11:10	188.1	182.4	177.3	2050	64.99
12/4/2020 11:10	188.1	182.1	177.8	2048	65.12
12/4/2020 11:10	188.1	182.3	177.8	2048	65.08
12/4/2020 11:10	188	182.1	177.2	2046	64.99
12/4/2020 11:10	188	182.1	177.1	2044	64.88
12/4/2020 11:10	188	182.6	177.5	2049	65.12
12/4/2020 11:10	188	182.1	177.4	2048	65.04
12/4/2020 11:10	188	182.3	177.3	2044	64.83
12/4/2020 11:10	187.9	182.1	177.8	2048	65.12
12/4/2020 11:10	188	182.5	178.3	2044	64.92
12/4/2020 11:10	187.9	182.6	177.3	2044	64.88
12/4/2020 11:10	187.9	182.1	177.3	2048	64.92
12/4/2020 11:10	187.9	182.3	177.4	2046	64.96
12/4/2020 11:10	187.9	182.5	177.6	2045	64.92
12/4/2020 11:10	187.9	182	177.6	2048	65.04
12/4/2020 11:10	187.8	182.5	177.4	2053	64.96
12/4/2020 11:10	187.8	182.3	178	2055	64.92
12/4/2020 11:10	187.8	182.2	178.2	2053	64.92
12/4/2020 11:10	187.9	182.6	177.2	2054	65.04
12/4/2020 11:10	187.8	182.1	177.5	2050	64.87
12/4/2020 11:10	187.7	182.5	177.6	2050	64.87
12/4/2020 11:10	187.7	182.2	177.6	2050	65
12/4/2020 11:10	187.7	182	177.5	2048	65.09
12/4/2020 11:10	187.7	182.6	177.9	2048	64.96
12/4/2020 11:10	187.7	182.2	178	2049	65.08
12/4/2020 11:10	187.7	182.2	177.3	2040	64.92
12/4/2020 11:10	187.7	182.5	177.4	2042	64.92
12/4/2020 11:10	187.6	182	177.6	2047	64.96
12/4/2020 11:10	187.7	182.6	177.5	2044	64.92
12/4/2020 11:10	187.6	183	177.6	2046	64.92
12/4/2020 11:10	187.7	183.1	178.3	2041	65.04
12/4/2020 11:10	187.6	183.3	178.3	2044	65
12/4/2020 11:10	187.5	183.1	177.6	2044	64.92
12/4/2020 11:10	187.5	183.4	177.3	2048	65.04
12/4/2020 11:10	187.5	183.2	177.6	2048	65.08
12/4/2020 11:10	187.5	183	177.6	2044	64.99
12/4/2020 11:10	187.4	183.2	177.8	2045	64.99
12/4/2020 11:10	187.4	183.1	177.6	2051	65.01
12/4/2020 11:10	187.4	183.4	177.8	2049	64.99
12/4/2020 11:10	187.4	183.2	177.5	2051	64.99
12/4/2020 11:10	187.4	183	177.5	2053	65.12
12/4/2020 11:10	187.4	183.2	177.6	2052	64.88
12/4/2020 11:10	187.4	183.1	177.9	2048	65.03

12/4/2020 11:10	187.3	183.2	177.8	2046	64.93
12/4/2020 11:10	187.3	183.1	177.9	2044	65.03
12/4/2020 11:10	187.3	183.1	177.9	2044	64.99
12/4/2020 11:10	187.3	183.3	177.6	2045	64.96
12/4/2020 11:10	187.3	183.1	177.5	2048	64.88
12/4/2020 11:10	187.3	183.4	177.4	2052	64.83
12/4/2020 11:10	187.2	183.4	177.6	2051	64.93
12/4/2020 11:10	187.2	183	178	2049	64.92
12/4/2020 11:10	187.2	183.2	177.6	2050	64.96
12/4/2020 11:11	187.2	183	177.9	2048	65
12/4/2020 11:11	187.2	183.2	177.7	2050	65.04
12/4/2020 11:11	187.1	183.2	177.4	2051	65.04
12/4/2020 11:11	187.2	183.2	177.5	2054	64.99
12/4/2020 11:11	187.2	183.4	177.4	2054	64.96
12/4/2020 11:11	187.1	183.2	178.3	2051	64.91
12/4/2020 11:11	187.1	183	178.6	2051	65
12/4/2020 11:11	187.1	183.3	177.8	2050	64.92
12/4/2020 11:11	187.1	183	177.8	2049	65.04
12/4/2020 11:11	187.1	183.2	177.9	2051	64.92
12/4/2020 11:11	187	183	177.8	2050	65
12/4/2020 11:11	187	183	177.6	2047	65
12/4/2020 11:11	187	183.2	177.6	2046	65.04
12/4/2020 11:11	187	183	178.6	2044	65.04
12/4/2020 11:11	187	183.3	178.3	2041	64.92
12/4/2020 11:11	187	183.2	177.7	2046	64.96
12/4/2020 11:11	187	183.2	178	2048	64.96
12/4/2020 11:11	186.9	183.3	177.9	2050	64.96
12/4/2020 11:11	186.9	183.1	177.9	2050	65
12/4/2020 11:11	186.8	183.2	177.5	2049	65
12/4/2020 11:11	186.9	183.1	177.8	2048	65
12/4/2020 11:11	186.8	183.2	178.5	2049	64.88
12/4/2020 11:11	186.8	183.2	178.3	2048	64.99
12/4/2020 11:11	186.8	183	177.9	2046	64.99
12/4/2020 11:11	186.8	183	178	2045	64.92
12/4/2020 11:11	186.8	183.2	178.1	2046	64.83
12/4/2020 11:11	186.8	183	178.1	2049	65.08
12/4/2020 11:11	186.7	183.2	177.8	2051	64.83
12/4/2020 11:11	186.7	183.1	177.8	2051	65
12/4/2020 11:11	186.7	183	178.3	2046	65.01
12/4/2020 11:11	186.7	183.2	178.8	2047	64.99
12/4/2020 11:11	186.6	183	178.4	2048	65.04
12/4/2020 11:11	186.6	183.3	177.8	2049	64.95
12/4/2020 11:11	186.7	183.3	177.9	2048	65.08
12/4/2020 11:11	186.6	183	178.1	2049	64.92
12/4/2020 11:11	186.6	183.3	178	2049	64.99
12/4/2020 11:11	186.6	183.2	178.1	2052	65.04
12/4/2020 11:11	186.5	183.4	177.8	2051	65.08

12/4/2020 11:11	186.5	183.4	177.6	2048	64.88
12/4/2020 11:11	186.5	183	177.8	2044	65.04
12/4/2020 11:11	186.4	183	177.6	2048	64.92
12/4/2020 11:11	186.4	183	178	2048	64.99
12/4/2020 11:11	186.4	183	178.1	2049	64.92
12/4/2020 11:11	186.4	183.2	178	2048	65.08
12/4/2020 11:11	186.3	183	178	2048	64.95
12/4/2020 11:11	186.3	183.3	177.8	2049	64.92
12/4/2020 11:11	186.3	183	177.7	2050	65.04
12/4/2020 11:11	186.4	183	177.6	2048	64.88
12/4/2020 11:11	186.3	183.2	177.8	2049	64.99
12/4/2020 11:11	186.4	183	177.7	2047	65.04
12/4/2020 11:11	186.3	183.2	177.8	2045	64.83
12/4/2020 11:11	186.3	183.3	178.1	2042	64.88
12/4/2020 11:11	186.3	183.2	178	2048	64.96
12/4/2020 11:11	186.3	183.1	178	2050	64.83
12/4/2020 11:11	186.2	183	178	2050	64.88
12/4/2020 11:11	186.3	183.3	178.3	2051	64.96
12/4/2020 11:11	186.2	183.3	178.1	2049	64.88
12/4/2020 11:11	186.2	183	178.3	2048	64.96
12/4/2020 11:11	186.2	183.2	178.8	2049	65
12/4/2020 11:12	186.2	183.1	179	2050	64.92
12/4/2020 11:12	186.2	183.2	178.1	2048	64.96
12/4/2020 11:12	186.2	183.4	178	2049	64.99
12/4/2020 11:12	186.1	183.1	178.3	2051	65
12/4/2020 11:12	186.1	183	178.1	2053	64.96
12/4/2020 11:12	186.1	183.1	178.1	2055	64.96
12/4/2020 11:12	186.1	183	178.3	2057	64.96
12/4/2020 11:12	186.1	183.4	179.1	2057	64.96
12/4/2020 11:12	186.1	183.2	178.2	2057	64.96
12/4/2020 11:12	186.1	183.3	177.8	2054	65
12/4/2020 11:12	186.1	183.4	178.1	2055	64.87
12/4/2020 11:12	186.1	183.2	178.3	2051	64.92
12/4/2020 11:12	186.1	183.2	178.3	2047	65
12/4/2020 11:12	186.1	183.4	178	2051	65.04
12/4/2020 11:12	186.1	183.2	178.6	2050	64.92
12/4/2020 11:12	186.1	183.1	178.9	2054	65.04
12/4/2020 11:12	186.1	183	177.8	2051	64.92
12/4/2020 11:12	186	183.4	177.9	2051	65
12/4/2020 11:12	186	183.2	178.1	2048	65
12/4/2020 11:12	186	183.4	178.5	2051	64.96
12/4/2020 11:12	186	183	178.3	2051	64.91
12/4/2020 11:12	185.9	183.2	178.3	2052	64.96
12/4/2020 11:12	186	183.3	179.2	2054	65
12/4/2020 11:12	186	183	178.2	2052	64.91
12/4/2020 11:12	185.9	183.1	178	2053	64.92
12/4/2020 11:12	186	183.5	178.3	2054	64.96

12/4/2020 11:12	185.9	183	178.4	2051	64.87
12/4/2020 11:12	185.9	183	178.4	2048	64.91
12/4/2020 11:12	185.9	183.2	178.4	2050	64.96
12/4/2020 11:12	185.9	183.1	178.2	2051	64.92
12/4/2020 11:12	185.9	183.4	179.1	2051	64.92
12/4/2020 11:12	185.9	183	178.7	2055	65.04
12/4/2020 11:12	185.9	183	178.1	2057	65
12/4/2020 11:12	185.9	183.2	178.1	2055	64.92
12/4/2020 11:12	185.9	183	178.5	2050	65.08
12/4/2020 11:12	185.8	183.2	178.3	2054	65
12/4/2020 11:12	185.9	183.1	178.3	2050	65.04
12/4/2020 11:12	185.8	183.2	178.3	2054	65.04
12/4/2020 11:12	185.8	183.4	178.3	2054	65
12/4/2020 11:12	185.8	183	178.6	2054	65.04
12/4/2020 11:12	185.8	183.2	179	2056	64.96
12/4/2020 11:12	185.8	183.2	179.2	2057	65.08
12/4/2020 11:12	185.8	183.3	179.2	2057	64.99
12/4/2020 11:12	185.7	183.2	178.8	2053	64.88
12/4/2020 11:12	185.8	183.1	178.5	2055	65
12/4/2020 11:12	185.8	183.1	178.6	2061	65.08
12/4/2020 11:12	185.7	183	178.7	2059	65.04
12/4/2020 11:12	185.8	183.1	178.9	2060	65.04
12/4/2020 11:12	185.8	183.1	179.2	2060	64.92
12/4/2020 11:12	185.7	183.2	179.2	2058	64.93
12/4/2020 11:12	185.7	183.3	178.7	2061	65.04
12/4/2020 11:12	185.7	183.2	178.5	2060	64.88
12/4/2020 11:12	185.7	183	178.6	2058	64.96
12/4/2020 11:12	185.7	183.2	178.8	2055	65
12/4/2020 11:12	185.7	183.2	178.3	2060	65.04
12/4/2020 11:12	185.7	183.1	178.1	2066	64.96
12/4/2020 11:12	185.6	183.3	179	2066	65.04
12/4/2020 11:12	185.7	183	179.1	2066	64.96
12/4/2020 11:12	185.7	183.3	178.4	2059	65.05
12/4/2020 11:12	185.7	183.1	178.5	2065	64.96
12/4/2020 11:13	185.6	183.2	178.3	2057	65
12/4/2020 11:13	185.7	183.4	178.6	2055	65
12/4/2020 11:13	185.6	183.1	178.2	2057	65
12/4/2020 11:13	185.6	183.2	178.1	2062	65.08
12/4/2020 11:13	185.6	183.1	179.3	2062	65.04
12/4/2020 11:13	185.5	183.2	179	2061	65.08
12/4/2020 11:13	185.5	183.2	178.4	2057	65.04
12/4/2020 11:13	185.5	182.9	178.7	2054	64.93
12/4/2020 11:13	185.5	183.2	178.7	2055	65.04
12/4/2020 11:13	185.5	183	178.6	2055	64.88
12/4/2020 11:13	185.5	183.2	178.3	2060	64.88
12/4/2020 11:13	185.5	183.3	178.7	2056	65.08
12/4/2020 11:13	185.5	183	179.4	2057	65.12

12/4/2020 11:13	185.5	183.2	178.9	2054	64.96
12/4/2020 11:13	185.5	183	178.5	2055	65
12/4/2020 11:13	185.5	183.1	178.7	2059	65
12/4/2020 11:13	185.5	183.2	178.8	2057	65
12/4/2020 11:13	185.4	183.2	178.7	2054	65
12/4/2020 11:13	185.5	183.3	178.5	2055	64.96
12/4/2020 11:13	185.4	183.3	178.5	2055	64.96
12/4/2020 11:13	185.3	183.1	179.1	2062	65.04
12/4/2020 11:13	185.4	183.2	179.4	2061	65.04
12/4/2020 11:13	185.5	183	178.5	2059	64.96
12/4/2020 11:13	185.4	183.2	178.6	2057	65.09
12/4/2020 11:13	185.4	183.2	178.7	2057	64.96
12/4/2020 11:13	185.3	183.2	178.7	2056	64.96
12/4/2020 11:13	185.4	183.1	178.3	2054	65.08
12/4/2020 11:13	185.4	183	178.3	2058	65.08
12/4/2020 11:13	185.4	183.1	179	2055	64.88
12/4/2020 11:13	185.4	183.4	179.4	2055	64.84
12/4/2020 11:13	185.4	183	178.9	2055	65.01
12/4/2020 11:13	185.4	183.3	178.6	2053	65.04
12/4/2020 11:13	185.4	183	178.5	2058	65.12
12/4/2020 11:13	185.4	183	178.8	2062	64.93
12/4/2020 11:13	185.4	183.2	178.5	2057	64.96
12/4/2020 11:13	185.3	183.1	178.7	2049	65
12/4/2020 11:13	185.4	183.3	178.4	2051	65.04
12/4/2020 11:13	185.3	183.4	178.4	2051	64.88
12/4/2020 11:13	185.4	183	178.5	2052	64.92
12/4/2020 11:13	185.3	183.1	178.7	2052	65.05
12/4/2020 11:13	185.3	183	179.2	2051	65
12/4/2020 11:13	185.4	183.3	179.6	2049	65
12/4/2020 11:13	185.3	183.2	179.4	2055	65
12/4/2020 11:13	185.3	183.1	178.9	2056	65
12/4/2020 11:13	185.3	183.3	178.6	2054	64.91
12/4/2020 11:13	185.3	183	178.7	2057	65.08
12/4/2020 11:13	185.3	183.2	178.8	2057	64.92
12/4/2020 11:13	185.3	183.3	178.8	2060	65.17
12/4/2020 11:13	185.3	183.1	179	2062	65.08
12/4/2020 11:13	185.3	183.4	178.9	2060	65.08
12/4/2020 11:13	185.2	183.2	178.8	2062	64.92
12/4/2020 11:13	185.3	183	178.7	2059	64.93
12/4/2020 11:13	185.3	183.2	178.4	2059	65.04
12/4/2020 11:13	185.2	183	178.3	2052	64.84
12/4/2020 11:13	185.3	183.2	178.5	2054	64.93
12/4/2020 11:13	185.3	183	179	2048	64.92
12/4/2020 11:13	185.3	183.1	179.3	2053	65.12
12/4/2020 11:13	185.3	183.2	179.6	2052	64.88
12/4/2020 11:13	185.3	183.1	179.2	2049	64.92
12/4/2020 11:14	185.3	183.1	178.8	2051	64.96

12/4/2020 11:14	185.3	183.2	178.8	2051	64.96
12/4/2020 11:14	185.2	183	178.7	2059	65
12/4/2020 11:14	185.3	183	178.9	2058	65.04
12/4/2020 11:14	185.3	183.1	178.9	2057	65.04
12/4/2020 11:14	185.2	183.2	178.8	2055	64.96
12/4/2020 11:14	185.3	183.2	178.7	2054	65
12/4/2020 11:14	185.2	183.1	178.6	2055	65.04
12/4/2020 11:14	185.2	183.2	178.6	2054	65.04
12/4/2020 11:14	185.2	183.2	179.2	2058	64.96
12/4/2020 11:14	185.1	183.1	179.8	2061	64.96
12/4/2020 11:14	185.2	183.2	179.1	2062	65
12/4/2020 11:14	185.1	183.1	178.8	2062	65.04
12/4/2020 11:14	185.1	183.2	179.1	2065	65.08
12/4/2020 11:14	185.1	183	179	2063	64.92
12/4/2020 11:14	184.9	183.2	179	2058	64.88
12/4/2020 11:14	185	183.4	178.7	2061	65.05
12/4/2020 11:14	185	183.1	178.8	2058	64.92
12/4/2020 11:14	185	183.2	179.6	2061	64.92
12/4/2020 11:14	184.9	183.1	179.3	2061	65
12/4/2020 11:14	184.9	183.2	178.9	2059	65.12
12/4/2020 11:14	184.9	183.4	179	2058	64.96
12/4/2020 11:14	184.8	183.1	179	2063	65
12/4/2020 11:14	184.8	183.4	179	2059	64.96
12/4/2020 11:14	184.7	183.4	178.8	2062	65
12/4/2020 11:14	184.7	183.1	178.7	2061	65.01
12/4/2020 11:14	184.6	183.2	179.2	2063	64.96
12/4/2020 11:14	184.6	183.2	179.7	2062	65
12/4/2020 11:14	184.6	183.1	179.1	2063	65
12/4/2020 11:14	184.5	183	178.8	2062	65.04
12/4/2020 11:14	184.4	183	179	2061	65.08
12/4/2020 11:14	184.4	183.3	179	2059	64.92
12/4/2020 11:14	184.4	183.2	179	2066	64.92
12/4/2020 11:14	184.4	183.2	178.7	2066	65.08
12/4/2020 11:14	184.4	183.3	179	2062	65.12
12/4/2020 11:14	184.2	183	179.9	2063	65.04
12/4/2020 11:14	184.2	183	179.1	2063	64.96
12/4/2020 11:14	184.2	183.3	178.8	2061	65.04
12/4/2020 11:14	184.1	183.2	179	2062	65.04
12/4/2020 11:14	184	183.3	179	2063	64.92
12/4/2020 11:14	184	183.2	179	2063	64.96
12/4/2020 11:14	184	183	178.6	2061	65.04
12/4/2020 11:14	183.9	183.2	178.6	2061	64.96
12/4/2020 11:14	183.8	183	179.2	2060	65
12/4/2020 11:14	183.8	183.4	179.8	2057	64.92
12/4/2020 11:14	183.8	183	179.1	2055	64.92
12/4/2020 11:14	183.7	183.1	178.8	2055	65
12/4/2020 11:14	183.7	183.2	179	2049	64.96

12/4/2020 11:14	183.7	183.2	179	2053	64.96
12/4/2020 11:14	183.7	183	179	2054	65.04
12/4/2020 11:14	183.7	183.2	179.2	2057	64.91
12/4/2020 11:14	183.6	183.2	179	2053	64.96
12/4/2020 11:14	183.6	183.2	178.9	2054	65.01
12/4/2020 11:14	183.6	183.2	179	2055	64.92
12/4/2020 11:14	183.7	183.3	178.8	2049	65.04
12/4/2020 11:14	183.6	183	179	2054	65.05
12/4/2020 11:14	183.6	183	178.9	2055	64.99
12/4/2020 11:14	183.6	183.1	178.8	2057	65
12/4/2020 11:14	183.6	183.2	178.9	2062	65.12
12/4/2020 11:15	183.6	182.7	178.9	2060	64.83
12/4/2020 11:15	183.7	182.1	178.9	2057	64.88
12/4/2020 11:15	183.6	182.1	178.9	2061	65.12
12/4/2020 11:15	183.7	182.3	178.8	2064	64.88
12/4/2020 11:15	183.6	182	179	2062	64.84
12/4/2020 11:15	183.7	182.3	179	2066	65.09
12/4/2020 11:15	183.7	182.7	179	2062	64.92
12/4/2020 11:15	183.7	182.6	179	2058	64.96
12/4/2020 11:15	183.8	182.7	179.1	2061	65.05
12/4/2020 11:15	183.9	182.2	179	2057	64.83
12/4/2020 11:15	183.8	182.1	179	2051	65
12/4/2020 11:15	183.9	182.6	179.4	2058	65
12/4/2020 11:15	183.9	182.2	179	2050	64.96
12/4/2020 11:15	183.9	182.7	179	2049	64.91
12/4/2020 11:15	184	182.1	179	2051	64.96
12/4/2020 11:15	184	182.5	179	2051	64.92
12/4/2020 11:15	184	182.4	179.1	2051	64.96
12/4/2020 11:15	184	181.9	178.9	2049	65.04
12/4/2020 11:15	184	182.5	178.8	2046	64.87
12/4/2020 11:15	184	182.1	179.1	2055	65
12/4/2020 11:15	184	182.7	179.2	2059	65
12/4/2020 11:15	184	182.1	179.9	2062	64.92
12/4/2020 11:15	184	182.3	179.2	2058	64.96
12/4/2020 11:15	184	182.5	178.5	2060	64.92
12/4/2020 11:15	184	182.3	178.7	2062	64.92
12/4/2020 11:15	184	182.2	179	2062	64.96
12/4/2020 11:15	184	182.1	179	2065	65.04
12/4/2020 11:15	184	182.2	178.8	2065	64.99
12/4/2020 11:15	184	182.6	179.2	2065	64.96
12/4/2020 11:15	183.9	182.4	179.8	2067	64.96
12/4/2020 11:15	184	182.1	178.6	2059	64.96
12/4/2020 11:15	183.8	182.3	178.9	2057	64.96
12/4/2020 11:15	183.8	182.2	179	2055	65.04
12/4/2020 11:15	183.8	182.2	179	2051	64.88
12/4/2020 11:15	183.8	182	179.1	2049	64.99
12/4/2020 11:15	183.8	182.5	179.6	2051	65.04

12/4/2020 11:15	183.7	182.5	179	2048	64.92
12/4/2020 11:15	183.7	182.2	178.7	2048	64.96
12/4/2020 11:15	183.7	182.7	178.8	2048	65.12
12/4/2020 11:15	183.8	182	179	2046	64.96
12/4/2020 11:15	183.7	182	178.8	2048	65
12/4/2020 11:15	183.7	182.5	179.1	2047	64.8
12/4/2020 11:15	183.7	182.1	179.7	2044	65.04
12/4/2020 11:15	183.7	182.1	179	2048	64.92
12/4/2020 11:15	183.7	182.1	178.6	2044	64.88
12/4/2020 11:15	183.7	182.5	179	2042	64.99
12/4/2020 11:15	183.7	182.7	178.8	2044	64.95
12/4/2020 11:15	183.7	182.3	179	2044	64.92
12/4/2020 11:15	183.8	182.1	179	2047	65.04
12/4/2020 11:15	183.8	182.7	178.8	2046	64.92
12/4/2020 11:15	183.9	182.3	179.3	2049	64.96
12/4/2020 11:15	183.9	182.4	179.7	2047	64.99
12/4/2020 11:15	183.9	182.3	179.8	2048	64.88
12/4/2020 11:15	183.9	182.1	179.5	2045	64.99
12/4/2020 11:15	184	182.5	178.8	2045	64.75
12/4/2020 11:15	184	182.1	178.7	2042	65.03
12/4/2020 11:15	184	182.6	178.8	2043	64.83
12/4/2020 11:15	184.1	182.5	178.8	2042	64.99
12/4/2020 11:15	184.1	182.1	178.9	2040	64.8
12/4/2020 11:15	184.2	182.5	178.9	2041	65
12/4/2020 11:16	184.3	182.1	178.8	2037	64.95
12/4/2020 11:16	184.3	182.7	178.9	2035	65.12
12/4/2020 11:16	184.4	182	178.8	2039	64.84
12/4/2020 11:16	184.4	182.1	178.9	2043	64.79
12/4/2020 11:16	184.5	182.5	178.8	2040	64.88
12/4/2020 11:16	184.6	182	178.8	2037	64.92
12/4/2020 11:16	184.6	182.6	178.9	2040	65.08
12/4/2020 11:16	184.7	182.1	178.9	2037	64.99
12/4/2020 11:16	184.8	182	178.9	2036	64.92
12/4/2020 11:16	184.8	182.3	179	2041	64.83
12/4/2020 11:16	185	182.2	179.5	2037	64.99
12/4/2020 11:16	185	182.1	179.6	2038	64.93
12/4/2020 11:16	185.2	182.5	180	2037	64.95
12/4/2020 11:16	185.2	182.1	179.9	2032	65.08
12/4/2020 11:16	185.4	182.3	179.4	2037	64.8
12/4/2020 11:16	185.5	182	179.1	2035	64.99
12/4/2020 11:16	185.5	182.3	179	2034	64.88
12/4/2020 11:16	185.7	182.7	179.2	2035	64.91
12/4/2020 11:16	185.8	182.1	179.1	2037	64.99
12/4/2020 11:16	185.9	182.3	179.2	2039	64.88
12/4/2020 11:16	186.1	182.1	179.2	2037	64.88
12/4/2020 11:16	186.2	182.5	179.2	2038	64.88
12/4/2020 11:16	186.4	182.4	179.1	2042	64.92

12/4/2020 11:16	186.5	182.2	178.8	2044	65.03
12/4/2020 11:16	186.6	182.2	179.7	2044	64.95
12/4/2020 11:16	186.7	182.1	179.9	2046	65.03
12/4/2020 11:16	186.8	182.5	179.4	2044	64.88
12/4/2020 11:16	187	182.3	179.2	2044	65.03
12/4/2020 11:16	187.2	182.2	179.5	2044	64.99
12/4/2020 11:16	187.3	183.2	179.3	2042	64.84
12/4/2020 11:16	187.4	183.4	179.5	2045	65.04
12/4/2020 11:16	187.5	183.1	179.1	2048	64.93
12/4/2020 11:16	187.7	183.2	179.1	2045	65.04
12/4/2020 11:16	187.9	183.3	179.7	2045	65.04
12/4/2020 11:16	187.9	183.1	180.2	2046	65.12
12/4/2020 11:16	188.1	183.2	179.9	2044	64.92
12/4/2020 11:16	188.3	183	179.2	2046	64.92
12/4/2020 11:16	188.3	183.2	179.4	2047	64.96
12/4/2020 11:16	188.6	183.2	179.6	2048	64.96
12/4/2020 11:16	188.5	183.3	179.6	2046	65.04
12/4/2020 11:16	188.8	183.2	179.7	2044	64.92
12/4/2020 11:16	188.9	183	179.7	2043	64.92
12/4/2020 11:16	188.9	183	179.2	2050	65.04
12/4/2020 11:16	189.1	183.4	179.1	2052	64.92
12/4/2020 11:16	189.2	183.1	179.9	2051	65.04
12/4/2020 11:16	189.3	183.2	180.2	2049	64.96
12/4/2020 11:16	189.5	183.2	180.4	2050	64.92
12/4/2020 11:16	189.6	183.2	179.9	2052	65.04
12/4/2020 11:16	189.8	183	179.4	2053	65.16
12/4/2020 11:16	189.8	183.1	179.5	2054	64.84
12/4/2020 11:16	190	183.1	179.8	2048	65.04
12/4/2020 11:16	190.1	183.4	179.8	2050	65.12
12/4/2020 11:16	190.2	183.1	179.7	2050	64.84
12/4/2020 11:16	190.3	183.1	179.7	2046	64.96
12/4/2020 11:16	190.4	183.2	179.7	2048	64.88
12/4/2020 11:16	190.6	183.2	179.8	2055	65.03
12/4/2020 11:16	190.7	183.2	179.7	2053	65.08
12/4/2020 11:16	190.7	183	179.4	2053	65.08
12/4/2020 11:16	190.8	183.2	179.5	2054	64.96
12/4/2020 11:17	190.9	183	179.5	2055	64.83
12/4/2020 11:17	190.9	183.2	179.7	2056	65.12
12/4/2020 11:17	191	183.2	180.1	2053	64.88
12/4/2020 11:17	191	183	180.3	2057	65.01
12/4/2020 11:17	191.1	183.1	180.5	2054	64.92
12/4/2020 11:17	191.1	183.2	180.4	2055	64.88
12/4/2020 11:17	191.2	183.1	180.6	2057	65
12/4/2020 11:17	191.2	183.2	180.6	2055	65
12/4/2020 11:17	191.2	183.1	180.6	2055	64.92
12/4/2020 11:17	191.3	183.1	180.3	2055	65
12/4/2020 11:17	191.4	183.1	179.9	2058	64.96

12/4/2020 11:17	191.4	183.2	179.7	2058	64.96
12/4/2020 11:17	191.4	183.4	179.8	2059	65.04
12/4/2020 11:17	191.5	183.1	180.1	2059	64.96
12/4/2020 11:17	191.6	184.2	180.1	2057	65
12/4/2020 11:17	191.5	184.4	180.2	2062	65
12/4/2020 11:17	191.5	184.1	180	2059	64.99
12/4/2020 11:17	191.6	184	180.2	2060	64.96
12/4/2020 11:17	191.6	184.2	180.8	2061	64.99
12/4/2020 11:17	191.6	183.9	179.9	2062	64.92
12/4/2020 11:17	191.7	184.1	179.9	2062	64.96
12/4/2020 11:17	191.7	184.4	180.2	2060	64.96
12/4/2020 11:17	191.7	184.2	180.1	2056	64.92
12/4/2020 11:17	191.8	184.2	179.9	2055	65.04
12/4/2020 11:17	191.8	184.1	180.6	2059	65.04
12/4/2020 11:17	191.8	184.1	180.8	2062	64.99
12/4/2020 11:17	191.8	184.4	179.9	2059	65.01
12/4/2020 11:17	191.8	184.1	179.9	2060	64.88
12/4/2020 11:17	191.8	184	180.2	2059	64.96
12/4/2020 11:17	191.8	184.3	180.4	2060	64.92
12/4/2020 11:17	191.9	184.2	180.2	2064	64.88
12/4/2020 11:17	191.9	184.1	180.2	2062	64.88
12/4/2020 11:17	191.9	184.2	180.4	2064	65.04
12/4/2020 11:17	191.9	184.3	180.9	2067	65.03
12/4/2020 11:17	191.9	184.3	180.6	2063	64.96
12/4/2020 11:17	191.9	184.1	180	2061	65.08
12/4/2020 11:17	191.9	184.2	179.9	2064	65
12/4/2020 11:17	191.9	183.9	180.6	2062	65
12/4/2020 11:17	191.9	184.1	180.2	2065	65.04
12/4/2020 11:17	191.8	184.3	180.3	2065	64.92
12/4/2020 11:17	191.9	184.1	180.4	2062	64.92
12/4/2020 11:17	191.9	184.1	180.3	2058	65.04
12/4/2020 11:17	191.9	184	180.6	2057	64.96
12/4/2020 11:17	191.9	184.2	181.3	2060	65
12/4/2020 11:17	191.9	184.2	181	2062	64.96
12/4/2020 11:17	191.8	184.1	180.6	2065	65.04
12/4/2020 11:17	191.9	184.1	180.3	2067	64.95
12/4/2020 11:17	191.8	184.2	180	2062	65.12
12/4/2020 11:17	191.9	184.1	180.2	2062	65.04
12/4/2020 11:17	191.8	184.2	180.1	2063	65.04
12/4/2020 11:17	191.9	184.1	180.2	2061	65.12
12/4/2020 11:17	191.8	184.3	180.2	2062	65.04
12/4/2020 11:17	191.8	184.1	180.3	2060	65.08
12/4/2020 11:17	191.8	184.1	180.2	2058	65.04
12/4/2020 11:17	191.8	184.2	180.6	2057	64.99
12/4/2020 11:17	191.8	184.1	180.4	2058	65.04
12/4/2020 11:17	191.8	184.2	180.4	2056	65.04
12/4/2020 11:17	191.8	184.1	180.2	2055	64.93

12/4/2020 11:17	191.8	184	180.2	2062	64.99
12/4/2020 11:17	191.8	184.2	180.2	2066	64.88
12/4/2020 11:18	191.8	184	180.4	2066	64.92
12/4/2020 11:18	191.7	184.2	180.8	2068	65
12/4/2020 11:18	191.8	184	181.1	2074	65.08
12/4/2020 11:18	191.6	184.8	181.4	2073	64.96
12/4/2020 11:18	191.7	184.8	181.1	2074	65
12/4/2020 11:18	191.8	184.6	180.8	2073	65
12/4/2020 11:18	191.6	185	180.4	2068	64.96
12/4/2020 11:18	191.8	185.4	180.4	2065	64.96
12/4/2020 11:18	191.6	185.1	180.6	2064	65
12/4/2020 11:18	191.6	185.3	180.6	2066	64.96
12/4/2020 11:18	191.6	185.3	180.5	2064	64.88
12/4/2020 11:18	191.6	185.3	180.2	2067	65.08
12/4/2020 11:18	191.6	185.2	180.2	2066	65
12/4/2020 11:18	191.5	184.9	180.7	2066	64.96
12/4/2020 11:18	191.6	185.1	181.3	2068	65.12
12/4/2020 11:18	191.5	184.8	181.3	2063	65.12
12/4/2020 11:18	191.5	185.1	180.7	2060	65.04
12/4/2020 11:18	191.5	185.4	180.6	2057	65.08
12/4/2020 11:18	191.5	185.4	180.6	2058	64.96
12/4/2020 11:18	191.4	184.7	180.8	2058	64.99
12/4/2020 11:18	191.4	185.4	180.8	2062	65.12
12/4/2020 11:18	191.3	185.1	180.8	2064	65.04
12/4/2020 11:18	191.3	185.3	180.6	2063	64.96
12/4/2020 11:18	191.3	185.1	180.4	2065	65.01
12/4/2020 11:18	191.3	185.4	180.4	2070	64.96
12/4/2020 11:18	191.3	185.2	180.6	2071	64.92
12/4/2020 11:18	191.3	184.8	181.3	2068	64.92
12/4/2020 11:18	191.3	185	181.4	2069	65.04
12/4/2020 11:18	191.3	184.8	180.9	2070	65
12/4/2020 11:18	191.2	184.8	180.6	2069	65.09
12/4/2020 11:18	191.3	185.2	180.6	2068	65
12/4/2020 11:18	191.1	184.9	180.6	2073	65
12/4/2020 11:18	191.1	185.1	180.6	2073	65.09
12/4/2020 11:18	191.2	184.8	180.8	2071	64.92
12/4/2020 11:18	191.1	184.8	180.8	2071	65
12/4/2020 11:18	191.1	185.3	180.6	2069	64.96
12/4/2020 11:18	191.1	184.9	180.8	2069	64.99
12/4/2020 11:18	191	184.9	180.8	2069	65.08
12/4/2020 11:18	191.1	184.8	180.9	2067	65.12
12/4/2020 11:18	191.1	184.7	180.9	2070	65.04
12/4/2020 11:18	191.1	185.4	180.6	2064	64.99
12/4/2020 11:18	191.1	184.9	180.8	2064	65.12
12/4/2020 11:18	191.1	184.9	180.8	2066	65.12
12/4/2020 11:18	191	184.9	180.9	2065	64.96
12/4/2020 11:18	190.9	184.9	180.8	2061	65.01

12/4/2020 11:18	191	185.3	180.9	2060	64.95
12/4/2020 11:18	191	185.1	180.8	2062	64.92
12/4/2020 11:18	190.9	184.8	181	2062	64.96
12/4/2020 11:18	190.9	184.9	180.9	2061	64.99
12/4/2020 11:18	190.9	184.8	181	2059	65.12
12/4/2020 11:18	190.9	185.2	181.1	2058	64.96
12/4/2020 11:18	190.9	185.1	180.7	2062	64.99
12/4/2020 11:18	190.9	185.4	180.9	2065	64.92
12/4/2020 11:18	190.9	184.9	181.2	2065	65.04
12/4/2020 11:18	190.9	184.8	181.6	2070	65.04
12/4/2020 11:18	190.8	185	181.4	2074	65.09
12/4/2020 11:18	190.7	185	180.8	2072	65
12/4/2020 11:18	190.8	184.7	180.5	2076	64.96
12/4/2020 11:18	190.8	185.4	180.6	2073	65.04
12/4/2020 11:19	190.7	185	180.7	2073	64.96
12/4/2020 11:19	190.7	184.9	180.9	2076	65
12/4/2020 11:19	190.8	185.2	180.9	2075	65
12/4/2020 11:19	190.7	184.8	180.8	2075	64.87
12/4/2020 11:19	190.7	185.3	180.9	2075	64.96
12/4/2020 11:19	190.6	185.1	180.9	2075	64.96
12/4/2020 11:19	190.7	184.8	180.7	2077	64.96
12/4/2020 11:19	190.7	184.8	180.6	2074	65.04
12/4/2020 11:19	190.6	184.8	180.8	2073	65
12/4/2020 11:19	190.6	185	181.3	2070	64.83
12/4/2020 11:19	190.6	185	181.6	2071	64.96
12/4/2020 11:19	190.6	185.3	181.6	2079	65.08
12/4/2020 11:19	190.6	185.3	181.4	2080	65
12/4/2020 11:19	190.5	184.8	181.3	2076	65.08
12/4/2020 11:19	190.6	185.3	180.8	2070	64.92
12/4/2020 11:19	190.5	185.2	180.8	2066	64.99
12/4/2020 11:19	190.5	184.9	180.8	2064	64.96
12/4/2020 11:19	190.5	185.4	180.6	2063	65.04
12/4/2020 11:19	190.5	185	180.7	2062	65.04
12/4/2020 11:19	190.4	185.3	180.7	2066	65.12
12/4/2020 11:19	190.5	184.9	180.8	2068	64.92
12/4/2020 11:19	190.5	184.7	181	2069	64.96
12/4/2020 11:19	190.4	185.4	180.7	2074	64.96
12/4/2020 11:19	190.3	184.9	180.9	2075	64.95
12/4/2020 11:19	190.3	184.9	180.8	2080	64.92
12/4/2020 11:19	190.3	185.3	180.6	2076	65.09
12/4/2020 11:19	190.3	184.8	180.8	2073	64.96
12/4/2020 11:19	190.3	185.4	180.8	2069	64.96
12/4/2020 11:19	190.2	186.2	181.1	2069	65
12/4/2020 11:19	190.2	185.6	181.7	2069	64.92
12/4/2020 11:19	190.2	186.3	181.6	2070	65.08
12/4/2020 11:19	190.1	185.8	181.3	2071	65.09
12/4/2020 11:19	190.1	186.5	180.9	2072	64.99

12/4/2020 11:19	190	186.2	181	2072	65
12/4/2020 11:19	190.1	185.8	181.2	2071	65.04
12/4/2020 11:19	190.1	186	181	2067	64.96
12/4/2020 11:19	190.1	185.7	180.8	2066	64.96
12/4/2020 11:19	190	186.3	180.7	2069	64.88
12/4/2020 11:19	190	186.4	181.5	2072	64.88
12/4/2020 11:19	190	186.3	181.8	2070	64.88
12/4/2020 11:19	189.9	186.2	181.1	2066	64.96
12/4/2020 11:19	189.9	185.9	180.8	2063	64.84
12/4/2020 11:19	189.9	186	180.9	2066	64.92
12/4/2020 11:19	189.9	186.4	181.1	2069	65.08
12/4/2020 11:19	189.9	186.4	181.1	2072	64.99
12/4/2020 11:19	189.8	186.2	180.8	2070	65.08
12/4/2020 11:19	189.8	186.2	180.8	2069	65.12
12/4/2020 11:19	189.8	185.8	180.9	2067	64.8
12/4/2020 11:19	189.8	186	182	2066	65.04
12/4/2020 11:19	189.7	186.2	181.3	2068	65.08
12/4/2020 11:19	189.7	186	180.7	2070	64.92
12/4/2020 11:19	189.7	186.4	181.2	2066	64.99
12/4/2020 11:19	189.7	185.6	181.1	2066	64.92
12/4/2020 11:19	189.7	186.3	181.1	2069	64.88
12/4/2020 11:19	189.7	185.8	180.9	2068	64.96
12/4/2020 11:19	189.6	186	180.8	2064	64.96
12/4/2020 11:19	189.6	186.5	181.4	2065	64.96
12/4/2020 11:19	189.6	186.2	181.9	2065	64.87
12/4/2020 11:19	189.6	186.2	181.4	2073	65.04
12/4/2020 11:20	189.6	185.7	181.1	2070	64.92
12/4/2020 11:20	189.6	186.1	181.3	2068	64.96
12/4/2020 11:20	189.5	185.9	181.3	2069	65
12/4/2020 11:20	189.6	185.6	181.3	2069	64.96
12/4/2020 11:20	189.5	186.2	181.1	2065	64.87
12/4/2020 11:20	189.5	186.2	180.9	2066	65
12/4/2020 11:20	189.5	185.9	181.1	2065	64.92
12/4/2020 11:20	189.4	186.4	180.9	2065	64.96
12/4/2020 11:20	189.4	186	181.1	2064	64.96
12/4/2020 11:20	189.4	186.3	181.3	2070	64.88
12/4/2020 11:20	189.4	186.3	181.5	2067	65.04
12/4/2020 11:20	189.4	185.6	181.8	2070	65.04
12/4/2020 11:20	189.5	186.1	181.6	2066	65.03
12/4/2020 11:20	189.4	185.8	181.6	2060	64.91
12/4/2020 11:20	189.5	186	181.6	2066	65.04
12/4/2020 11:20	189.5	186.2	181.1	2063	65.04
12/4/2020 11:20	189.5	185.6	180.9	2062	64.88
12/4/2020 11:20	189.5	186.2	181	2063	65.04
12/4/2020 11:20	189.5	185.7	180.9	2055	64.96
12/4/2020 11:20	189.6	185.8	181.1	2058	65
12/4/2020 11:20	189.6	186.4	180.9	2061	65.04

12/4/2020 11:20	189.6	185.8	181.4	2065	64.88
12/4/2020 11:20	189.6	186.5	181.3	2062	64.99
12/4/2020 11:20	189.6	186.1	181.6	2065	64.96
12/4/2020 11:20	189.6	186.1	181.8	2060	64.88
12/4/2020 11:20	189.6	186.5	181	2062	64.84
12/4/2020 11:20	189.7	185.8	181.2	2062	64.96
12/4/2020 11:20	189.8	186.1	181.2	2062	64.99
12/4/2020 11:20	189.8	186.1	181.3	2058	65.04
12/4/2020 11:20	189.8	185.7	181.2	2062	64.96
12/4/2020 11:20	189.8	186.5	182	2062	64.88
12/4/2020 11:20	189.8	186	181.6	2065	64.92
12/4/2020 11:20	189.9	186	181	2059	65.04
12/4/2020 11:20	189.9	186.2	181.3	2058	64.88
12/4/2020 11:20	190	186.5	181.3	2058	64.92
12/4/2020 11:20	190	186.2	181.4	2056	64.96
12/4/2020 11:20	190.1	185.9	181.3	2054	64.99
12/4/2020 11:20	190.1	186.1	181.8	2052	64.88
12/4/2020 11:20	190.2	186.2	182	2055	65
12/4/2020 11:20	190.2	186.3	181.6	2059	64.88
12/4/2020 11:20	190.3	186.3	181.3	2064	65
12/4/2020 11:20	190.3	185.8	181.3	2066	64.96
12/4/2020 11:20	190.4	186	181.5	2064	64.96
12/4/2020 11:20	190.4	186.3	181.4	2062	64.96
12/4/2020 11:20	190.5	185.6	181.5	2062	65
12/4/2020 11:20	190.5	186.5	181.6	2062	64.92
12/4/2020 11:20	190.5	185.8	181.6	2062	64.92
12/4/2020 11:20	190.6	186	181.5	2058	65
12/4/2020 11:20	190.6	186.3	181.6	2058	64.96
12/4/2020 11:20	190.7	185.9	181.3	2059	64.96
12/4/2020 11:20	190.7	186.4	181.4	2061	65.04
12/4/2020 11:20	190.8	186.1	181.4	2062	64.88
12/4/2020 11:20	190.8	185.7	181.3	2063	65.08
12/4/2020 11:20	190.9	186.5	181.6	2064	64.96
12/4/2020 11:20	190.9	186	181.5	2062	64.99
12/4/2020 11:20	190.9	186.1	181.7	2060	65.08
12/4/2020 11:20	190.9	186.4	181.7	2066	65
12/4/2020 11:20	191	185.7	181.5	2064	64.92
12/4/2020 11:20	191	186.4	181.5	2062	64.96
12/4/2020 11:20	191.1	185.9	181.5	2063	65.04
12/4/2020 11:21	191.1	186.3	181.3	2062	64.96
12/4/2020 11:21	191.1	185.7	181.6	2064	65.04
12/4/2020 11:21	191.2	185.7	181.5	2065	64.93
12/4/2020 11:21	191.3	186.3	181.5	2061	64.84
12/4/2020 11:21	191.2	185.9	181.6	2059	64.88
12/4/2020 11:21	191.3	186.5	181.7	2061	64.92
12/4/2020 11:21	191.3	186.5	181.5	2062	65.08
12/4/2020 11:21	191.3	185.8	181.8	2061	64.95

12/4/2020 11:21	191.4	186.1	182	2064	64.91
12/4/2020 11:21	191.4	185.7	181.9	2059	64.92
12/4/2020 11:21	191.5	186.2	181.8	2055	64.99
12/4/2020 11:21	191.5	186.5	181.6	2061	64.99
12/4/2020 11:21	191.5	185.8	181.8	2061	65.04
12/4/2020 11:21	191.6	186	182	2064	64.96
12/4/2020 11:21	191.6	185.7	181.8	2063	64.99
12/4/2020 11:21	191.6	185.7	182	2060	65.08
12/4/2020 11:21	191.6	186.3	182	2061	65
12/4/2020 11:21	191.7	185.9	181.9	2062	64.91
12/4/2020 11:21	191.7	186.1	181.9	2063	65.09
12/4/2020 11:21	191.8	186.3	181.9	2060	64.87
12/4/2020 11:21	191.8	185.7	181.5	2058	64.96
12/4/2020 11:21	191.8	186.4	182.2	2058	65.04
12/4/2020 11:21	191.8	185.8	182.4	2057	64.96
12/4/2020 11:21	191.8	185.9	182.7	2058	64.96
12/4/2020 11:21	191.9	186.7	181.8	2058	65.04
12/4/2020 11:21	191.9	185.8	181.5	2060	65
12/4/2020 11:21	192	186.3	182	2058	64.92
12/4/2020 11:21	191.9	185.9	182.1	2062	65
12/4/2020 11:21	192	186	181.5	2062	64.87
12/4/2020 11:21	192.1	185.8	182.8	2059	64.96
12/4/2020 11:21	192	186.1	181.8	2059	64.96
12/4/2020 11:21	192.1	187.1	181.5	2059	64.83
12/4/2020 11:21	192.1	187	182	2054	64.92
12/4/2020 11:21	192.1	186.8	182	2055	65.12
12/4/2020 11:21	192.2	187.4	181.9	2055	65.04
12/4/2020 11:21	192.2	186.8	182.1	2052	64.99
12/4/2020 11:21	192.2	187.3	182.7	2053	64.99
12/4/2020 11:21	192.2	187.4	182.3	2053	64.95
12/4/2020 11:21	192.2	186.8	181.8	2055	64.96
12/4/2020 11:21	192.3	187.1	182	2055	64.96
12/4/2020 11:21	192.2	187.2	182.2	2057	64.84
12/4/2020 11:21	192.3	187	182.2	2057	65.12
12/4/2020 11:21	192.3	187.5	181.9	2058	65.08
12/4/2020 11:21	192.3	187	182.1	2062	64.92
12/4/2020 11:21	192.3	187.2	182.8	2058	64.96
12/4/2020 11:21	192.3	187.2	182.8	2058	64.96
12/4/2020 11:21	192.3	186.8	182.3	2058	65.08
12/4/2020 11:21	192.4	187	181.8	2058	64.99
12/4/2020 11:21	192.4	187	182	2059	65.08
12/4/2020 11:21	192.4	187.2	182.3	2062	65.04
12/4/2020 11:21	192.4	187.3	182.3	2062	65.04
12/4/2020 11:21	192.4	187.2	182.3	2063	64.96
12/4/2020 11:21	192.5	187.5	182.1	2060	64.96
12/4/2020 11:21	192.5	186.8	182.8	2058	65.04
12/4/2020 11:21	192.6	186.9	182.5	2061	64.96

12/4/2020 11:21	192.6	186.8	181.9	2062	65
12/4/2020 11:21	192.6	187.1	182.4	2066	65
12/4/2020 11:21	192.6	187.2	182.5	2064	64.87
12/4/2020 11:21	192.6	187.4	182.3	2062	65
12/4/2020 11:22	192.6	187.4	182.8	2058	64.96
12/4/2020 11:22	192.7	186.9	183	2063	64.92
12/4/2020 11:22	192.7	187	181.9	2062	64.92
12/4/2020 11:22	192.7	187.2	182.3	2058	64.88
12/4/2020 11:22	192.7	186.9	182.3	2063	64.92
12/4/2020 11:22	192.8	187	182.3	2062	65
12/4/2020 11:22	192.7	187.1	182.2	2062	65.12
12/4/2020 11:22	192.8	187.2	182.7	2063	64.92
12/4/2020 11:22	192.8	187.4	182.8	2062	65.03
12/4/2020 11:22	192.9	187.1	182.3	2062	64.92
12/4/2020 11:22	192.9	187	182.4	2059	64.99
12/4/2020 11:22	192.9	186.9	182.8	2059	64.88
12/4/2020 11:22	192.9	187	182.6	2059	64.99
12/4/2020 11:22	192.9	187.1	182.6	2058	65.04
12/4/2020 11:22	192.9	187.3	183.2	2058	64.92
12/4/2020 11:22	192.9	186.8	182.7	2062	65.12
12/4/2020 11:22	193	187.1	182.3	2066	64.84
12/4/2020 11:22	193.1	187	182.5	2063	64.88
12/4/2020 11:22	193.1	187.4	182.1	2062	65.03
12/4/2020 11:22	193.1	187.4	182.4	2062	64.99
12/4/2020 11:22	193.1	187	182.3	2062	64.96
12/4/2020 11:22	193.1	187.1	183.2	2062	64.88
12/4/2020 11:22	193.1	186.9	183.5	2062	64.99
12/4/2020 11:22	193.2	187.4	182.6	2062	64.88
12/4/2020 11:22	193.1	187.4	182.4	2062	64.92
12/4/2020 11:22	193.1	186.8	182.5	2065	64.84
12/4/2020 11:22	193.2	187.2	182.6	2065	65.03
12/4/2020 11:22	193.1	186.8	182.5	2069	65.08
12/4/2020 11:22	193.2	186.9	182.5	2071	64.99
12/4/2020 11:22	193.1	187.3	182.7	2075	65.08
12/4/2020 11:22	193.2	187	183.4	2069	65
12/4/2020 11:22	193.2	187.2	183.5	2075	65.03
12/4/2020 11:22	193.2	186.9	182.6	2075	64.92
12/4/2020 11:22	193.1	187.3	182.2	2076	64.99
12/4/2020 11:22	193.1	187.3	182.7	2074	65.04
12/4/2020 11:22	193.1	187	182.5	2069	64.96
12/4/2020 11:22	193.1	187.4	182.9	2071	64.96
12/4/2020 11:22	193.2	187.3	183	2073	64.96
12/4/2020 11:22	193	187	182.7	2066	65
12/4/2020 11:22	193.1	187.2	182.5	2069	65
12/4/2020 11:22	193	187.2	183.4	2071	64.96
12/4/2020 11:22	193	187	183.7	2073	65.04
12/4/2020 11:22	192.9	187.5	183.4	2072	64.92

12/4/2020 11:22	192.9	186.8	183	2081	64.96
12/4/2020 11:22	192.9	187.1	182.6	2081	65.04
12/4/2020 11:22	192.8	187.2	182.6	2083	65
12/4/2020 11:22	192.8	186.9	182.6	2077	65
12/4/2020 11:22	192.8	187.2	182.5	2079	64.96
12/4/2020 11:22	192.6	187.1	182.6	2076	65
12/4/2020 11:22	192.6	187.1	182.7	2078	64.96
12/4/2020 11:22	192.6	187	182.8	2082	65.08
12/4/2020 11:22	192.5	186.8	182.8	2077	64.88
12/4/2020 11:22	192.5	187.2	183.1	2076	64.84
12/4/2020 11:22	192.4	188	182.8	2076	65
12/4/2020 11:22	192.3	188.1	182.8	2082	64.88
12/4/2020 11:22	192.2	188.1	183	2082	65.08
12/4/2020 11:22	192.2	188.1	183	2087	64.97
12/4/2020 11:22	192.2	188	183	2088	64.96
12/4/2020 11:22	192.1	188.1	183.1	2084	64.99
12/4/2020 11:22	192	188.1	183.2	2084	65.05
12/4/2020 11:23	191.9	188.1	182.9	2084	65.04
12/4/2020 11:23	191.9	188.2	182.9	2082	65.04
12/4/2020 11:23	191.8	188.1	183.1	2082	65.05
12/4/2020 11:23	191.6	188.1	183.1	2076	65
12/4/2020 11:23	191.5	188.1	183	2078	64.96
12/4/2020 11:23	191.5	188.1	182.8	2082	65.05
12/4/2020 11:23	191.3	188.1	182.5	2084	64.96
12/4/2020 11:23	191.3	188.1	182.7	2082	64.96
12/4/2020 11:23	191.2	188.1	183.2	2080	65
12/4/2020 11:23	191.1	188.1	183.7	2078	64.99
12/4/2020 11:23	191.1	188.3	183.4	2076	65.03
12/4/2020 11:23	191	188.1	182.8	2081	65.21
12/4/2020 11:23	190.9	188	182.8	2083	65.03
12/4/2020 11:23	190.8	188.1	183	2081	64.92
12/4/2020 11:23	190.7	188.1	182.7	2084	65.17
12/4/2020 11:23	190.7	188.1	183	2080	64.88
12/4/2020 11:23	190.5	187.3	182.8	2080	64.88
12/4/2020 11:23	190.5	186.9	183.1	2084	64.96
12/4/2020 11:23	190.2	187	183.9	2087	64.88
12/4/2020 11:23	190.2	187	183.4	2084	64.92
12/4/2020 11:23	190.1	187.2	182.7	2084	65.08
12/4/2020 11:23	190	187.2	183	2086	65
12/4/2020 11:23	190	186.8	183.2	2083	65.09
12/4/2020 11:23	189.8	187.4	182.8	2087	65.05
12/4/2020 11:23	189.7	187.1	182.8	2087	65
12/4/2020 11:23	189.6	187	183.2	2085	65.04
12/4/2020 11:23	189.5	186.9	183	2084	64.96
12/4/2020 11:23	189.4	187.2	183	2080	65
12/4/2020 11:23	189.3	187.4	182.9	2083	65.04
12/4/2020 11:23	189.2	187.2	182.9	2084	64.92

12/4/2020 11:23	189.1	187.4	182.7	2083	64.92
12/4/2020 11:23	189	187.2	183	2086	64.96
12/4/2020 11:23	189	186.8	183	2086	65.01
12/4/2020 11:23	188.9	187.1	182.7	2087	64.96
12/4/2020 11:23	188.7	187	183.1	2086	65.12
12/4/2020 11:23	188.7	187	183.7	2087	64.96
12/4/2020 11:23	188.6	187.2	182.7	2086	65.08
12/4/2020 11:23	188.8	187.1	182.8	2084	65.04
12/4/2020 11:23	188.5	187.3	182.8	2086	65.08
12/4/2020 11:23	188.3	187	182.5	2082	65
12/4/2020 11:23	188.3	187	183.2	2080	64.96
12/4/2020 11:23	188.2	187.4	183.5	2086	65
12/4/2020 11:23	188.1	187	182.5	2089	65
12/4/2020 11:23	188	187.5	183	2093	64.96
12/4/2020 11:23	187.9	187	182.8	2087	65.04
12/4/2020 11:23	187.9	186.9	182.7	2084	64.96
12/4/2020 11:23	187.6	187.4	182.8	2084	64.96
12/4/2020 11:23	187.7	187.4	183.8	2078	64.92
12/4/2020 11:23	187.6	187.3	183.3	2073	65.08
12/4/2020 11:23	187.5	187	182.7	2075	65.12
12/4/2020 11:23	187.4	187	182.8	2075	65.08
12/4/2020 11:23	187.4	187.2	182.7	2077	65.08
12/4/2020 11:23	187.3	187	182.8	2080	64.88
12/4/2020 11:23	187.3	187.2	182.7	2079	65.08
12/4/2020 11:23	187.2	187.3	183.5	2076	65.04
12/4/2020 11:23	187.2	186.9	183.2	2080	65
12/4/2020 11:23	187.1	187.2	182.4	2076	65.08
12/4/2020 11:23	187.1	186.8	182.9	2076	64.96
12/4/2020 11:23	187	187	182.8	2075	65
12/4/2020 11:24	187	187.3	182.7	2072	65
12/4/2020 11:24	187	187	182.8	2070	65.04
12/4/2020 11:24	187	187	182.7	2071	64.96
12/4/2020 11:24	186.9	187.4	183	2071	64.92
12/4/2020 11:24	186.8	186.8	183.6	2069	65
12/4/2020 11:24	187	187.2	183.4	2074	65.04
12/4/2020 11:24	186.8	187	182.6	2074	65
12/4/2020 11:24	186.9	187.3	182.5	2072	64.96
12/4/2020 11:24	186.8	187	182.4	2075	64.96
12/4/2020 11:24	186.8	187	182.7	2076	64.84
12/4/2020 11:24	186.8	187.4	183	2072	65.01
12/4/2020 11:24	186.8	187	183	2074	64.88
12/4/2020 11:24	186.8	187.2	183	2076	65.04
12/4/2020 11:24	186.7	187.3	183	2076	64.93
12/4/2020 11:24	186.7	186.8	182.7	2073	65.08
12/4/2020 11:24	186.7	187.1	183	2074	64.93
12/4/2020 11:24	186.8	187.2	182.8	2072	64.88
12/4/2020 11:24	186.7	187	182.6	2072	65.12

12/4/2020 11:24	186.7	187.3	182.8	2072	65.05
12/4/2020 11:24	186.7	187.1	182.7	2073	65
12/4/2020 11:24	186.7	187.4	183.4	2071	65.04
12/4/2020 11:24	186.7	187.3	183.3	2073	65
12/4/2020 11:24	186.7	186.8	183.5	2076	64.96
12/4/2020 11:24	186.7	187.3	183.6	2073	65
12/4/2020 11:24	186.7	187	183.2	2072	65
12/4/2020 11:24	186.8	187.3	183.4	2070	64.92
12/4/2020 11:24	186.7	187.4	183.2	2072	65
12/4/2020 11:24	186.7	187	182.8	2072	65.09
12/4/2020 11:24	186.7	187.3	182.4	2076	64.96
12/4/2020 11:24	186.8	187.1	182.5	2074	65.08
12/4/2020 11:24	186.7	187	182.5	2071	65
12/4/2020 11:24	186.7	187.5	182.5	2072	65.04
12/4/2020 11:24	186.7	186.9	182.7	2071	64.92
12/4/2020 11:24	186.7	187.3	182.7	2073	64.88
12/4/2020 11:24	186.7	187	182.8	2079	64.84
12/4/2020 11:24	186.6	187	183	2075	64.92
12/4/2020 11:24	186.6	187.5	182.8	2073	64.96
12/4/2020 11:24	186.6	187	183.2	2077	64.8
12/4/2020 11:24	186.6	187.3	182.7	2075	64.96
12/4/2020 11:24	186.5	187.1	182.5	2076	65
12/4/2020 11:24	186.5	186.8	182.7	2069	64.99
12/4/2020 11:24	186.5	187.3	182.7	2066	65.08
12/4/2020 11:24	186.5	187	182.8	2069	65.04
12/4/2020 11:24	186.5	187.4	182.9	2066	64.83
12/4/2020 11:24	186.4	187.3	182.8	2066	64.84
12/4/2020 11:24	186.4	186.9	182.6	2064	64.99
12/4/2020 11:24	186.4	186.5	182.8	2066	64.96
12/4/2020 11:24	186.4	186	182.7	2060	65.04
12/4/2020 11:24	186.4	186.3	182.9	2065	65.12
12/4/2020 11:24	186.3	185.6	182.5	2066	64.99
12/4/2020 11:24	186.4	185.9	182.5	2067	65.01
12/4/2020 11:24	186.3	186.3	182.8	2064	64.88
12/4/2020 11:24	186.3	186	182.8	2066	64.96
12/4/2020 11:24	186.4	186.4	183	2069	65.04
12/4/2020 11:24	186.4	186.2	182.9	2066	64.83
12/4/2020 11:24	186.3	185.7	182.7	2062	64.92
12/4/2020 11:24	186.5	185.9	182.8	2064	65
12/4/2020 11:24	186.5	185.7	182.5	2065	65
12/4/2020 11:24	186.4	186.1	182.4	2063	65
12/4/2020 11:25	186.5	186.5	183.1	2061	65.04
12/4/2020 11:25	186.5	185.6	183.7	2058	65
12/4/2020 11:25	186.6	186.3	183.2	2054	64.92
12/4/2020 11:25	186.6	186.2	182.6	2056	65.04
12/4/2020 11:25	186.7	185.8	182.8	2057	64.91
12/4/2020 11:25	186.8	186.5	182.6	2060	64.96

12/4/2020 11:25	186.7	186.1	182.8	2059	64.96
12/4/2020 11:25	186.8	186.2	182.5	2057	64.91
12/4/2020 11:25	186.9	186.2	183.6	2058	64.91
12/4/2020 11:25	187	185.8	183.1	2055	65
12/4/2020 11:25	187.1	186.3	182.5	2062	64.96
12/4/2020 11:25	187.1	186	182.8	2060	64.95
12/4/2020 11:25	187.2	186.2	183	2062	65.05
12/4/2020 11:25	187.3	186.4	182.7	2062	64.96
12/4/2020 11:25	187.4	185.8	182.5	2061	64.88
12/4/2020 11:25	187.4	186.3	183.2	2062	65.04
12/4/2020 11:25	187.5	186	183.4	2059	64.92
12/4/2020 11:25	187.6	185.8	182.7	2064	64.99
12/4/2020 11:25	187.9	185.7	182.3	2062	64.95
12/4/2020 11:25	188	186	182.9	2065	65.04
12/4/2020 11:25	188	186.2	183.1	2058	64.88
12/4/2020 11:25	188.1	186.2	182.8	2060	65.08
12/4/2020 11:25	188.2	186.4	183.1	2058	64.88
12/4/2020 11:25	188.4	186.5	182.8	2062	65.12
12/4/2020 11:25	188.5	186.2	183.1	2065	65.04
12/4/2020 11:25	188.6	186.4	183.7	2062	64.92
12/4/2020 11:25	188.7	186.3	183.8	2059	65.08
12/4/2020 11:25	188.8	185.6	183.4	2058	64.95
12/4/2020 11:25	188.9	185.9	183	2057	64.88
12/4/2020 11:25	188.9	185.9	182.5	2058	64.96
12/4/2020 11:25	189.1	186.1	182.5	2062	64.88
12/4/2020 11:25	189.3	186.5	182.8	2063	64.99
12/4/2020 11:25	189.3	186.5	182.9	2064	64.92
12/4/2020 11:25	189.5	186	183.2	2062	65.03
12/4/2020 11:25	189.5	185.7	183.2	2066	64.88
12/4/2020 11:25	189.7	186.1	183	2068	64.96
12/4/2020 11:25	189.8	186.3	183.2	2065	64.95
12/4/2020 11:25	189.9	185.8	183.4	2066	64.88
12/4/2020 11:25	190	186	183.3	2065	65
12/4/2020 11:25	190	185.7	183.3	2068	65.03
12/4/2020 11:25	190.1	186	183.2	2070	64.88
12/4/2020 11:25	190.3	186.5	183	2071	65.12
12/4/2020 11:25	190.3	187.1	183	2071	64.99
12/4/2020 11:25	190.3	187.4	183	2070	64.99
12/4/2020 11:25	190.4	187	183.2	2071	64.96
12/4/2020 11:25	190.5	187	183.4	2071	64.99
12/4/2020 11:25	190.5	187.2	183.3	2073	64.92
12/4/2020 11:25	190.5	187.3	183.2	2071	64.92
12/4/2020 11:25	190.6	187.4	183.4	2066	64.88
12/4/2020 11:25	190.7	187.2	183.2	2066	64.92
12/4/2020 11:25	190.7	186.8	183.4	2066	65.04
12/4/2020 11:25	190.7	187	183.2	2068	64.83
12/4/2020 11:25	190.8	187	183.1	2069	64.91

12/4/2020 11:25	190.8	187.4	183.4	2069	65.04
12/4/2020 11:25	190.8	187.4	183.2	2070	64.88
12/4/2020 11:25	190.8	187	183.4	2066	64.92
12/4/2020 11:25	190.9	187.2	183.4	2071	65
12/4/2020 11:25	190.9	187.2	183.2	2072	64.83
12/4/2020 11:25	190.9	187	183.2	2075	65
12/4/2020 11:25	190.9	187.4	183.5	2077	65
12/4/2020 11:26	190.9	186.9	183.2	2076	64.92
12/4/2020 11:26	190.9	187	183.4	2071	64.96
12/4/2020 11:26	190.9	186.9	183.2	2072	65.09
12/4/2020 11:26	190.9	187	183.3	2072	64.92
12/4/2020 11:26	191	187.2	183.5	2076	64.92
12/4/2020 11:26	190.9	187.1	183.3	2076	65
12/4/2020 11:26	190.9	187.2	183.2	2072	64.87
12/4/2020 11:26	190.9	186.9	183.5	2074	64.91
12/4/2020 11:26	190.9	187	183.5	2076	65.04
12/4/2020 11:26	190.9	187.2	183.2	2077	64.83
12/4/2020 11:26	190.9	187.1	183.4	2072	64.96
12/4/2020 11:26	190.9	187	183.4	2070	64.96
12/4/2020 11:26	190.9	187.3	183.5	2070	64.96
12/4/2020 11:26	190.9	186.9	183.8	2063	64.83
12/4/2020 11:26	190.8	187.2	183.4	2066	65.04
12/4/2020 11:26	190.8	186.9	183.5	2067	64.96
12/4/2020 11:26	190.8	187.2	183.5	2070	64.88
12/4/2020 11:26	190.7	187.4	183.4	2073	65
12/4/2020 11:26	190.7	187	183.7	2070	64.99
12/4/2020 11:26	190.7	187.2	183.2	2073	65
12/4/2020 11:26	190.7	186.8	183.5	2075	64.93
12/4/2020 11:26	190.7	187.3	183.5	2078	64.95
12/4/2020 11:26	190.6	187.4	183.2	2080	65.04
12/4/2020 11:26	190.6	186.9	183.3	2079	64.96
12/4/2020 11:26	190.6	187.1	183.4	2079	64.88
12/4/2020 11:26	190.5	187.2	183.3	2080	64.83
12/4/2020 11:26	190.5	186.8	183.7	2078	65.04
12/4/2020 11:26	190.5	187.4	183.9	2080	64.91
12/4/2020 11:26	190.5	187.2	183.9	2079	64.88
12/4/2020 11:26	190.4	187.4	184.4	2080	64.93
12/4/2020 11:26	190.4	187.3	184.1	2082	64.88
12/4/2020 11:26	190.3	186.8	184.1	2082	64.95
12/4/2020 11:26	190.3	187.2	184.1	2082	64.88
12/4/2020 11:26	190.2	187	184.1	2086	64.88
12/4/2020 11:26	190.2	187.1	184.1	2078	64.88
12/4/2020 11:26	190.2	187	184.1	2081	64.96
12/4/2020 11:26	190.1	187	183.7	2081	64.88
12/4/2020 11:26	190	186.8	183.5	2080	64.8
12/4/2020 11:26	189.9	186.9	183.2	2082	64.96
12/4/2020 11:26	189.9	187	183.2	2083	65.03

12/4/2020 11:26	189.8	187.2	183.4	2082	64.92
12/4/2020 11:26	189.8	187.2	183.5	2087	65
12/4/2020 11:26	189.8	187.3	183.7	2080	64.88
12/4/2020 11:26	189.7	186.9	183.7	2079	64.88
12/4/2020 11:26	189.7	187	183.5	2076	64.88
12/4/2020 11:26	189.6	187	183.6	2079	64.99
12/4/2020 11:26	189.6	187	183.4	2080	64.92
12/4/2020 11:26	189.6	187.4	184	2078	64.96
12/4/2020 11:26	189.6	186.9	184.6	2079	64.96
12/4/2020 11:26	189.4	186.9	183.9	2076	64.92
12/4/2020 11:26	189.5	187.3	183.2	2077	65
12/4/2020 11:26	189.4	187	183.4	2076	64.91
12/4/2020 11:26	189.3	187.7	183.3	2075	64.96
12/4/2020 11:26	189.4	187.2	183.4	2079	65
12/4/2020 11:26	189.3	186.8	183.8	2077	64.91
12/4/2020 11:26	189.2	187.3	183.5	2075	64.96
12/4/2020 11:26	189.2	187	184.3	2079	65.04
12/4/2020 11:26	189.1	187	184.3	2078	64.96
12/4/2020 11:26	189	187.4	183.2	2079	64.99
12/4/2020 11:27	189	186.7	183.4	2076	65.09
12/4/2020 11:27	188.9	187.4	183.5	2073	64.88
12/4/2020 11:27	189	187.4	183.7	2076	65.08
12/4/2020 11:27	188.8	187.2	183.8	2077	64.96
12/4/2020 11:27	188.8	187.4	183.6	2082	64.88
12/4/2020 11:27	188.7	187	183.4	2081	65
12/4/2020 11:27	188.7	187.3	184	2080	64.96
12/4/2020 11:27	188.7	187.2	184.4	2080	64.99
12/4/2020 11:27	188.5	187.2	184.4	2079	64.92
12/4/2020 11:27	188.5	187.4	183.7	2083	65.12
12/4/2020 11:27	188.5	187	183.1	2083	64.92
12/4/2020 11:27	188.5	187.3	183.4	2078	64.88
12/4/2020 11:27	188.4	186.8	183	2083	65.16
12/4/2020 11:27	188.3	187.1	183.1	2082	65.04
12/4/2020 11:27	188.3	187.4	183.4	2086	65.08
12/4/2020 11:27	188.2	187.1	183.5	2086	65.04
12/4/2020 11:27	188.2	187.3	183.3	2083	64.95
12/4/2020 11:27	188.1	186.9	183.4	2080	65.04
12/4/2020 11:27	188.1	187	183	2082	65.05
12/4/2020 11:27	188	187.4	183	2080	64.92
12/4/2020 11:27	188	186.8	183.2	2080	64.96
12/4/2020 11:27	187.9	187.3	183	2079	65.09
12/4/2020 11:27	187.9	187.3	183.2	2070	64.96
12/4/2020 11:27	187.8	186.9	184.1	2069	64.96
12/4/2020 11:27	187.7	187.1	184.1	2073	65
12/4/2020 11:27	187.7	186.9	184.4	2077	64.96
12/4/2020 11:27	187.6	187	183.7	2082	65.04
12/4/2020 11:27	187.6	187.5	183	2080	64.96

12/4/2020 11:27	187.4	187	183.5	2080	65
12/4/2020 11:27	187.4	187	183.5	2082	64.95
12/4/2020 11:27	187.4	187.2	183.4	2077	64.91
12/4/2020 11:27	187.3	187	183.4	2076	64.96
12/4/2020 11:27	187.3	187	183	2075	64.96
12/4/2020 11:27	187.2	187	183.8	2077	65.04
12/4/2020 11:27	187.1	187.2	184.4	2078	64.99
12/4/2020 11:27	187.1	187.2	183.4	2077	65.04
12/4/2020 11:27	187	187.1	183.5	2078	65.04
12/4/2020 11:27	187	187.4	183.7	2082	64.99
12/4/2020 11:27	187	185.8	183.4	2079	64.96
12/4/2020 11:27	186.9	186.4	183.5	2077	64.84
12/4/2020 11:27	186.9	186.4	183.4	2078	65.04
12/4/2020 11:27	186.9	185.6	183	2075	65.03
12/4/2020 11:27	186.8	186.4	183	2076	64.99
12/4/2020 11:27	186.7	185.8	183.4	2075	65.04
12/4/2020 11:27	186.6	186.2	183.8	2076	65.08
12/4/2020 11:27	186.6	185.8	183.9	2078	64.99
12/4/2020 11:27	186.5	186.3	183.4	2082	64.96
12/4/2020 11:27	186.5	186.2	183	2080	64.96
12/4/2020 11:27	186.4	185.8	183.4	2078	64.88
12/4/2020 11:27	186.3	186.1	183.1	2079	65.04
12/4/2020 11:27	186.3	185.8	183.4	2082	64.83
12/4/2020 11:27	186.3	186	183.4	2080	64.92
12/4/2020 11:27	186.3	186.5	183.1	2080	65.04
12/4/2020 11:27	186.2	185.6	183.3	2076	65.04
12/4/2020 11:27	186.1	185.9	183.2	2080	65.04
12/4/2020 11:27	186.1	185.7	183.1	2079	65.04
12/4/2020 11:27	186	186.1	183.7	2079	64.96
12/4/2020 11:27	186	186.5	183.8	2078	64.96
12/4/2020 11:27	185.9	186	183.4	2081	65.09
12/4/2020 11:27	185.9	185.8	183.3	2080	64.96
12/4/2020 11:28	185.9	185.8	183	2077	64.92
12/4/2020 11:28	185.8	186.1	183.3	2082	65
12/4/2020 11:28	185.8	186.3	183.1	2082	64.96
12/4/2020 11:28	185.8	186.2	183	2082	64.92
12/4/2020 11:28	185.7	186.4	183.3	2078	65.04
12/4/2020 11:28	185.7	185.9	182.9	2081	64.96
12/4/2020 11:28	185.6	186.2	183	2080	64.84
12/4/2020 11:28	185.5	186.2	183	2080	64.88
12/4/2020 11:28	185.5	186.4	183.4	2081	65.04
12/4/2020 11:28	185.5	186.5	184.1	2080	64.92
12/4/2020 11:28	185.5	185.6	183.5	2077	64.93
12/4/2020 11:28	185.3	185.8	183	2079	64.99
12/4/2020 11:28	185.4	186	183	2080	64.92
12/4/2020 11:28	185.3	185.8	183	2080	64.93
12/4/2020 11:28	185.2	186.2	183.3	2075	64.88

12/4/2020 11:28	185.2	186.1	183.2	2075	64.92
12/4/2020 11:28	185.2	186.2	182.7	2075	64.96
12/4/2020 11:28	185.1	186	182.7	2079	64.99
12/4/2020 11:28	185.1	186.4	183.7	2078	64.99
12/4/2020 11:28	185.1	186.5	183.7	2078	65.08
12/4/2020 11:28	185	186.5	183.1	2082	64.88
12/4/2020 11:28	185	186.2	182.9	2083	64.92
12/4/2020 11:28	184.9	186.1	182.9	2083	65
12/4/2020 11:28	184.9	186.5	183	2076	64.95
12/4/2020 11:28	184.8	186.3	183	2075	65.04
12/4/2020 11:28	184.8	186	183	2074	65.04
12/4/2020 11:28	184.8	185.7	183	2075	65
12/4/2020 11:28	184.7	185.6	183	2078	64.96
12/4/2020 11:28	184.7	186.3	182.5	2078	64.92
12/4/2020 11:28	184.7	186	182.5	2080	65
12/4/2020 11:28	184.6	186	182.6	2079	64.96
12/4/2020 11:28	184.6	186.5	182.5	2076	64.92
12/4/2020 11:28	184.6	186.3	183.1	2077	64.96
12/4/2020 11:28	184.6	186.2	183.1	2079	65
12/4/2020 11:28	184.5	185.8	183.5	2075	64.96
12/4/2020 11:28	184.5	186.4	183.8	2077	65.04
12/4/2020 11:28	184.5	185.8	183.7	2075	65
12/4/2020 11:28	184.4	186.1	183.7	2072	64.88
12/4/2020 11:28	184.5	186.5	183.4	2076	64.96
12/4/2020 11:28	184.4	186	183.1	2076	65.04
12/4/2020 11:28	184.4	186.3	183	2076	64.92
12/4/2020 11:28	184.4	186.4	183	2073	64.92
12/4/2020 11:28	184.4	185.8	182.7	2073	64.88
12/4/2020 11:28	184.4	186.3	183	2071	64.92
12/4/2020 11:28	184.4	186.1	182.7	2075	64.88
12/4/2020 11:28	184.4	185.8	182.6	2075	65.04
12/4/2020 11:28	184.5	186.2	182.8	2075	64.84
12/4/2020 11:28	184.4	185.8	182.8	2072	65.21
12/4/2020 11:28	184.4	186.2	182.8	2073	65.04
12/4/2020 11:28	184.5	185.6	182.8	2072	64.84
12/4/2020 11:28	184.5	186.2	182.7	2073	65.08
12/4/2020 11:28	184.5	186.5	182.9	2073	64.92
12/4/2020 11:28	184.4	184.8	182.8	2076	64.84
12/4/2020 11:28	184.5	185.1	182.8	2076	64.99
12/4/2020 11:28	184.5	184.9	183	2077	64.99
12/4/2020 11:28	184.5	185	183	2073	65
12/4/2020 11:28	184.5	185.4	182.7	2080	65.12
12/4/2020 11:28	184.5	184.8	182.8	2076	65.04
12/4/2020 11:28	184.5	185.4	182.5	2080	64.92
12/4/2020 11:29	184.5	184.9	182.4	2078	65
12/4/2020 11:29	184.6	184.6	182.6	2080	65.08
12/4/2020 11:29	184.6	185.4	183.2	2077	65

12/4/2020 11:29	184.6	184.7	183.5	2078	65.04
12/4/2020 11:29	184.6	185.2	182.5	2072	64.96
12/4/2020 11:29	184.6	185.3	182.5	2071	65
12/4/2020 11:29	184.6	184.8	182.8	2071	65
12/4/2020 11:29	184.7	185.3	182.9	2075	65.04
12/4/2020 11:29	184.8	184.8	182.5	2079	64.92
12/4/2020 11:29	184.7	185.2	182.5	2079	65.04
12/4/2020 11:29	184.7	185.4	183.5	2079	64.96
12/4/2020 11:29	184.8	184.7	183	2082	64.88
12/4/2020 11:29	184.7	185	182.5	2079	64.96
12/4/2020 11:29	184.7	184.9	182.7	2076	65.08
12/4/2020 11:29	184.8	185	182.8	2076	64.96
12/4/2020 11:29	184.9	185.4	182.9	2080	64.92
12/4/2020 11:29	184.9	185.1	182.4	2079	64.88
12/4/2020 11:29	184.9	185.4	182.9	2080	64.88
12/4/2020 11:29	184.9	185.2	183.7	2077	64.97
12/4/2020 11:29	184.9	185.4	182.8	2082	64.93
12/4/2020 11:29	185	185.2	182.7	2080	64.92
12/4/2020 11:29	185	184.6	182.7	2077	65.04
12/4/2020 11:29	185	185.3	182.7	2078	65.08
12/4/2020 11:29	185	184.9	182.5	2084	65
12/4/2020 11:29	185.1	185.4	182.1	2081	65.04
12/4/2020 11:29	185	185.1	183	2079	64.96
12/4/2020 11:29	185.1	184.7	183.4	2077	65.08
12/4/2020 11:29	185.1	185.1	182.3	2073	64.96
12/4/2020 11:29	185.2	185.2	182.7	2074	64.96
12/4/2020 11:29	185.2	184.8	182.7	2079	65
12/4/2020 11:29	185.2	185.4	182.4	2078	65.04
12/4/2020 11:29	185.2	184.8	182.5	2077	64.96
12/4/2020 11:29	185.3	185	182.3	2083	65.05
12/4/2020 11:29	185.3	184.9	183.5	2080	65.04
12/4/2020 11:29	185.4	185.1	182.7	2080	64.92
12/4/2020 11:29	185.4	185.4	182.3	2080	65.08
12/4/2020 11:29	185.4	184.9	182.5	2079	65
12/4/2020 11:29	185.4	184.8	182.6	2082	64.99
12/4/2020 11:29	185.4	185.4	182.4	2080	64.92
12/4/2020 11:29	185.5	184.7	182.4	2077	64.92
12/4/2020 11:29	185.5	185.4	182.7	2076	64.92
12/4/2020 11:29	185.5	184.8	183.5	2073	65.08
12/4/2020 11:29	185.6	185	182.8	2076	64.92
12/4/2020 11:29	185.6	185.2	182.2	2072	64.92
12/4/2020 11:29	185.6	184.9	182.5	2079	65.16
12/4/2020 11:29	185.6	185.2	182.4	2080	64.84
12/4/2020 11:29	185.7	184.7	182.3	2075	65.08
12/4/2020 11:29	185.8	184.8	182.2	2076	64.96
12/4/2020 11:29	185.8	185.5	183.2	2071	64.95
12/4/2020 11:29	185.8	184.8	182.9	2072	64.96

12/4/2020 11:29	185.8	185.3	182.3	2073	65.12
12/4/2020 11:29	185.8	184.8	182.5	2076	64.96
12/4/2020 11:29	185.9	184.9	182.5	2076	64.96
12/4/2020 11:29	185.9	185.3	182.7	2076	65
12/4/2020 11:29	185.8	185.1	182.2	2076	64.96
12/4/2020 11:29	185.9	185.3	183.2	2083	65
12/4/2020 11:29	185.9	184.6	183	2084	65.04
12/4/2020 11:29	186	184.7	182.3	2082	64.92
12/4/2020 11:29	185.9	185	182.8	2078	64.96
12/4/2020 11:30	186	185	182.7	2076	65
12/4/2020 11:30	186	185.4	182.7	2075	64.96
12/4/2020 11:30	185.9	185.1	182.5	2077	64.92
12/4/2020 11:30	185.9	184.8	182.3	2080	65
12/4/2020 11:30	185.9	185.4	183	2078	65
12/4/2020 11:30	185.9	185.3	183.3	2075	64.92
12/4/2020 11:30	185.9	184.7	182.6	2076	65.09
12/4/2020 11:30	185.9	185.1	182.8	2073	64.92
12/4/2020 11:30	185.9	184.8	182.8	2070	65.08
12/4/2020 11:30	186	185	182.7	2071	65
12/4/2020 11:30	185.9	185.1	182.3	2066	64.88
12/4/2020 11:30	185.9	184.9	182.4	2070	64.88
12/4/2020 11:30	185.9	185.1	183.3	2066	65.12
12/4/2020 11:30	185.9	184.8	183.2	2068	64.92
12/4/2020 11:30	185.9	185.2	182.5	2067	64.92
12/4/2020 11:30	185.9	185.2	182.7	2073	65.16
12/4/2020 11:30	185.9	184.7	182.7	2070	64.95
12/4/2020 11:30	185.9	185.3	182.4	2070	65.04
12/4/2020 11:30	186	184.7	182.4	2066	64.92
12/4/2020 11:30	186	185.1	182.5	2070	64.88
12/4/2020 11:30	186	184.8	183.4	2069	65.04
12/4/2020 11:30	186.1	184.7	183	2066	64.92
12/4/2020 11:30	186.1	185.5	182.5	2059	64.99
12/4/2020 11:30	186.1	184.7	182.5	2055	64.99
12/4/2020 11:30	186.2	185.1	182.7	2061	65.04
12/4/2020 11:30	186.3	185.4	182.5	2060	65.04
12/4/2020 11:30	186.3	185.4	182.2	2061	65
12/4/2020 11:30	186.3	185.1	182.9	2061	65
12/4/2020 11:30	186.5	185.1	183.2	2058	64.99
12/4/2020 11:30	186.5	185	182.2	2059	65.04
12/4/2020 11:30	186.6	185.3	182.7	2058	64.92
12/4/2020 11:30	186.8	184.9	182.6	2058	64.92
12/4/2020 11:30	186.8	185.4	182.6	2055	65.04
12/4/2020 11:30	187	185.1	182.4	2058	65.04
12/4/2020 11:30	187.1	184.8	182.3	2060	64.96
12/4/2020 11:30	187.1	185.4	183.3	2062	65
12/4/2020 11:30	187.2	184.8	182.8	2062	65.04
12/4/2020 11:30	187.2	185.3	182.3	2062	64.96

12/4/2020 11:30	187.5	185.1	182.7	2063	64.91
12/4/2020 11:30	187.6	184.6	182.7	2059	65.05
12/4/2020 11:30	187.7	184.8	182.7	2058	65
12/4/2020 11:30	187.8	184.8	182.3	2061	65.04
12/4/2020 11:30	187.9	185.4	182.3	2059	65.04
12/4/2020 11:30	188.1	185.2	183	2060	65.04
12/4/2020 11:30	188.2	184.7	183.2	2060	65.12
12/4/2020 11:30	188.3	185.2	182.4	2058	64.96
12/4/2020 11:30	188.4	184.7	182.8	2054	65.04
12/4/2020 11:30	188.5	184.8	183	2057	64.84
12/4/2020 11:30	188.7	184.7	182.8	2056	64.96
12/4/2020 11:30	188.8	185.2	182.4	2057	64.92
12/4/2020 11:30	188.8	185.5	182.6	2058	65
12/4/2020 11:30	189.1	185.1	183.4	2060	64.92
12/4/2020 11:30	189.1	185	183.1	2059	65.03
12/4/2020 11:30	189.3	185.3	182.6	2062	64.92
12/4/2020 11:30	189.5	184.7	182.8	2066	65.04
12/4/2020 11:30	189.5	185.4	182.9	2067	64.96
12/4/2020 11:30	189.7	185.1	182.5	2063	64.88
12/4/2020 11:30	189.7	185.4	182.6	2066	64.92
12/4/2020 11:30	189.8	185.4	182.9	2067	64.88
12/4/2020 11:30	190	184.6	183.4	2070	64.88
12/4/2020 11:31	190	185.3	183.2	2066	65
12/4/2020 11:31	190.1	184.8	182.5	2067	64.88
12/4/2020 11:31	190.3	185	182.8	2068	65.12
12/4/2020 11:31	190.3	185.4	183	2068	65
12/4/2020 11:31	190.5	184.9	182.9	2070	64.96
12/4/2020 11:31	190.6	185.3	182.7	2071	65.04
12/4/2020 11:31	190.6	184.9	182.9	2071	64.96
12/4/2020 11:31	190.7	185.1	183.4	2074	64.96
12/4/2020 11:31	190.8	185.3	183	2075	64.96
12/4/2020 11:31	190.7	185.1	182.5	2076	65
12/4/2020 11:31	190.8	185.1	182.7	2077	64.96
12/4/2020 11:31	190.9	185.3	183	2078	65.05
12/4/2020 11:31	190.9	184.7	183	2076	65.04
12/4/2020 11:31	190.9	186.1	182.7	2079	64.92
12/4/2020 11:31	191	185.7	182.8	2080	65.08
12/4/2020 11:31	190.9	186.4	183.4	2077	65.04
12/4/2020 11:31	191	186.3	183.6	2083	65.08
12/4/2020 11:31	191.1	185.9	183.4	2080	65.04
12/4/2020 11:31	191.1	186.2	182.8	2078	65
12/4/2020 11:31	191	185.8	183.2	2078	64.93
12/4/2020 11:31	191	185.6	183.1	2080	64.92
12/4/2020 11:31	191	186.4	183	2080	64.93
12/4/2020 11:31	191.1	185.8	183	2080	65.04
12/4/2020 11:31	191.1	185.8	182.9	2079	65.16
12/4/2020 11:31	191	186	182.7	2080	64.92

12/4/2020 11:31	191	186.1	183.3	2079	65.08
12/4/2020 11:31	191	186.3	183.7	2080	65.04
12/4/2020 11:31	190.9	186	183.5	2078	65.04
12/4/2020 11:31	191	185.9	183	2078	65
12/4/2020 11:31	190.9	185.8	183	2076	64.96
12/4/2020 11:31	190.9	185.8	182.8	2075	64.88
12/4/2020 11:31	190.9	186.1	183.3	2079	65.04
12/4/2020 11:31	190.8	186.3	183	2080	65
12/4/2020 11:31	190.8	185.8	182.7	2084	64.96
12/4/2020 11:31	190.7	186	182.8	2088	65
12/4/2020 11:31	190.7	185.8	183.8	2090	64.96
12/4/2020 11:31	190.7	186	183.3	2083	64.96
12/4/2020 11:31	190.5	186.3	182.9	2085	65
12/4/2020 11:31	190.5	186.5	183.3	2087	64.96
12/4/2020 11:31	190.5	186.2	183.4	2089	64.87
12/4/2020 11:31	190.3	185.7	183	2089	65
12/4/2020 11:31	190.3	186.1	182.7	2089	64.88
12/4/2020 11:31	190.3	186.3	183	2089	65.04
12/4/2020 11:31	190.3	186.4	183.7	2088	65.04
12/4/2020 11:31	190.1	186.1	183.7	2086	64.88
12/4/2020 11:31	190	186	182.9	2087	64.84
12/4/2020 11:31	189.9	186	183	2087	65.08
12/4/2020 11:31	189.9	185.9	183.2	2091	64.88
12/4/2020 11:31	189.8	185.8	183.1	2091	64.88
12/4/2020 11:31	189.8	186.2	182.6	2094	64.97
12/4/2020 11:31	189.7	186.3	182.6	2090	64.84
12/4/2020 11:31	189.6	186.4	183.4	2090	65.04
12/4/2020 11:31	189.6	186.3	183.4	2091	65.08
12/4/2020 11:31	189.5	185.6	183	2088	64.99
12/4/2020 11:31	189.4	186.1	182.8	2086	65.04
12/4/2020 11:31	189.3	186.3	183	2084	65.12
12/4/2020 11:31	189.4	185.9	183.2	2082	64.92
12/4/2020 11:31	189.2	186	182.9	2079	64.91
12/4/2020 11:31	189.1	185.7	182.7	2079	64.96
12/4/2020 11:32	189.1	186.2	183	2080	64.88
12/4/2020 11:32	189	186.3	183.6	2083	64.96
12/4/2020 11:32	189	186.3	183.2	2083	64.96
12/4/2020 11:32	188.9	186.3	182.8	2082	64.92
12/4/2020 11:32	188.8	185.8	183.1	2080	64.92
12/4/2020 11:32	188.8	186.5	182.9	2080	65
12/4/2020 11:32	188.7	186.2	182.8	2084	65
12/4/2020 11:32	188.7	185.6	182.9	2080	64.92
12/4/2020 11:32	188.7	186.4	182.9	2084	65.09
12/4/2020 11:32	188.7	185.8	182.7	2089	64.96
12/4/2020 11:32	188.7	186.2	182.8	2083	64.96
12/4/2020 11:32	188.7	186.4	183	2084	65.05
12/4/2020 11:32	188.6	185.7	183.4	2082	64.99

12/4/2020 11:32	188.6	186.2	183.6	2083	64.88
12/4/2020 11:32	188.5	185.7	183.3	2088	64.96
12/4/2020 11:32	188.5	185.9	183	2086	64.88
12/4/2020 11:32	188.5	186.5	182.9	2083	65.03
12/4/2020 11:32	188.5	185.7	182.6	2083	65.16
12/4/2020 11:32	188.5	186.3	183	2084	64.92
12/4/2020 11:32	188.4	185.8	182.7	2086	65.03
12/4/2020 11:32	188.4	186.2	182.9	2088	65.16
12/4/2020 11:32	188.4	186.4	183	2086	65.08
12/4/2020 11:32	188.4	185.8	183	2087	64.92
12/4/2020 11:32	188.4	186.4	183	2084	64.92
12/4/2020 11:32	188.3	185.8	183	2080	64.88
12/4/2020 11:32	188.3	185.7	182.8	2080	64.99
12/4/2020 11:32	188.2	186.2	182.5	2084	65.04
12/4/2020 11:32	188.3	185.9	183	2084	65.08
12/4/2020 11:32	188.2	186	182.5	2080	65.04
12/4/2020 11:32	188.2	185.9	182.8	2081	65.05
12/4/2020 11:32	188.2	185.8	182.9	2082	64.96
12/4/2020 11:32	188.1	186.2	182.3	2082	65
12/4/2020 11:32	188.2	186	182.7	2082	65.04
12/4/2020 11:32	188.2	186.3	182.8	2086	64.92
12/4/2020 11:32	188.1	186.4	182.8	2082	65
12/4/2020 11:32	188.1	185.6	183.4	2080	65.04
12/4/2020 11:32	188.1	185.8	183.4	2076	64.96
12/4/2020 11:32	188	186.3	183.3	2079	64.88
12/4/2020 11:32	188	185.8	183.5	2085	65.08
12/4/2020 11:32	187.9	186.5	183.2	2087	65
12/4/2020 11:32	187.9	185.8	182.9	2086	65
12/4/2020 11:32	187.9	186	182.7	2089	64.96
12/4/2020 11:32	187.8	185.6	182.6	2084	65.03
12/4/2020 11:32	187.8	185.7	182.6	2084	64.88
12/4/2020 11:32	187.7	186.5	182.7	2087	65.2
12/4/2020 11:32	187.7	185.8	182.7	2088	65.08
12/4/2020 11:32	187.7	186.2	182.7	2089	64.99
12/4/2020 11:32	187.6	186.5	182.8	2086	64.96
12/4/2020 11:32	187.5	185.8	182.7	2084	64.88
12/4/2020 11:32	187.5	186.1	182.8	2084	64.88
12/4/2020 11:32	187.4	186	182.5	2087	65.17
12/4/2020 11:32	187.4	185.8	182.3	2084	64.83
12/4/2020 11:32	187.4	185.9	182.3	2083	65.04
12/4/2020 11:32	187.3	185.7	182.3	2082	65.08
12/4/2020 11:32	187.2	186.2	182.8	2081	64.99
12/4/2020 11:32	187.2	186.2	183.2	2082	64.96
12/4/2020 11:32	187.2	185.8	183.2	2082	65.04
12/4/2020 11:32	187.1	186	183.4	2080	64.96
12/4/2020 11:32	187.1	185.6	182.7	2083	65
12/4/2020 11:32	187.1	186.2	182.5	2082	65

12/4/2020 11:33	187.1	186.3	182.6	2080	64.96
12/4/2020 11:33	187.1	185.8	182.3	2080	64.96
12/4/2020 11:33	187	186.1	182.8	2077	65.09
12/4/2020 11:33	187	185.7	182.7	2072	64.83
12/4/2020 11:33	187	185.8	182.3	2073	64.92
12/4/2020 11:33	186.9	185.6	182.7	2072	65.12
12/4/2020 11:33	186.7	186.1	182.4	2070	64.88
12/4/2020 11:33	186.8	186.3	182.3	2077	64.88
12/4/2020 11:33	186.9	186	182.3	2071	64.92
12/4/2020 11:33	186.8	186.5	182.1	2070	64.99
12/4/2020 11:33	186.9	185.7	182.7	2070	64.99
12/4/2020 11:33	186.8	185.7	183.1	2073	65.16
12/4/2020 11:33	186.8	186.2	183.3	2071	64.95
12/4/2020 11:33	186.8	186.2	183.2	2071	64.88
12/4/2020 11:33	186.8	186.1	182.9	2074	65.01
12/4/2020 11:33	186.8	186	182.7	2076	64.92
12/4/2020 11:33	187	185.7	182.6	2074	65.04
12/4/2020 11:33	186.9	186.2	182.4	2075	65.04
12/4/2020 11:33	186.8	186.2	182.5	2067	64.99
12/4/2020 11:33	186.9	186.4	182.7	2073	64.99
12/4/2020 11:33	187	186	182.6	2073	65.08
12/4/2020 11:33	187	185.8	182.5	2073	65.08
12/4/2020 11:33	187	185.9	182.7	2073	64.99
12/4/2020 11:33	187.1	185.8	182.4	2069	65.09
12/4/2020 11:33	187	185.8	182.5	2068	64.92
12/4/2020 11:33	187.1	186.2	182.7	2066	65
12/4/2020 11:33	187.2	185.6	182.5	2068	65
12/4/2020 11:33	187.2	186.1	182.7	2068	64.96
12/4/2020 11:33	187.2	186.4	182.7	2062	65
12/4/2020 11:33	187.3	185.7	182.6	2058	65.05
12/4/2020 11:33	187.5	186.4	182.8	2059	64.96
12/4/2020 11:33	187.4	185.6	182.4	2058	64.96
12/4/2020 11:33	187.6	185.8	182.6	2062	64.96
12/4/2020 11:33	187.6	186.3	182.8	2066	64.96
12/4/2020 11:33	187.6	186	182.5	2060	64.92
12/4/2020 11:33	187.7	186.1	182.7	2059	65
12/4/2020 11:33	187.8	185.8	182.7	2058	64.83
12/4/2020 11:33	188.1	186	182.6	2061	65.04
12/4/2020 11:33	188	186.4	182.7	2061	64.92
12/4/2020 11:33	188.1	186	182.7	2062	64.84
12/4/2020 11:33	188.3	186.5	182.5	2062	65.08
12/4/2020 11:33	188.3	186.1	182.7	2062	65.08
12/4/2020 11:33	188.4	185.9	182.7	2061	65.08
12/4/2020 11:33	188.6	186.3	182.2	2060	65.04
12/4/2020 11:33	188.7	185.7	182.8	2066	65
12/4/2020 11:33	188.8	186.3	183	2065	64.84
12/4/2020 11:33	188.9	185.8	182.8	2066	64.96

12/4/2020 11:33	189	186.1	182.5	2064	64.99
12/4/2020 11:33	189.2	186.4	182.6	2062	64.95
12/4/2020 11:33	189.2	186.1	182.8	2064	64.92
12/4/2020 11:33	189.5	185.9	182.8	2062	64.88
12/4/2020 11:33	189.6	186.2	182.7	2066	64.88
12/4/2020 11:33	189.7	185.6	182.8	2068	64.88
12/4/2020 11:33	189.9	185.9	182.5	2069	64.96
12/4/2020 11:33	189.8	185.8	182.7	2069	64.92
12/4/2020 11:33	190.1	186.2	182.6	2066	65.12
12/4/2020 11:33	190.3	186.5	182.6	2075	64.92
12/4/2020 11:33	190.4	185.8	183	2073	64.92
12/4/2020 11:33	190.5	186.2	183.6	2075	64.96
12/4/2020 11:34	190.5	185.8	183.3	2074	64.96
12/4/2020 11:34	190.7	185.8	182.7	2075	64.92
12/4/2020 11:34	190.8	186.5	182.8	2077	64.96
12/4/2020 11:34	190.9	186.1	182.9	2077	64.96
12/4/2020 11:34	190.9	186.2	183	2073	64.96
12/4/2020 11:34	191	186.2	182.8	2075	64.96
12/4/2020 11:34	191.1	186	182.5	2077	65.04
12/4/2020 11:34	191.2	186.1	182.6	2080	65
12/4/2020 11:34	191.3	185.8	182.6	2084	65
12/4/2020 11:34	191.4	186.1	183.5	2082	65
12/4/2020 11:34	191.4	186.4	183.8	2079	64.95
12/4/2020 11:34	191.5	186.3	183	2079	64.92
12/4/2020 11:34	191.6	186.1	183	2082	65
12/4/2020 11:34	191.6	185.7	183	2083	64.92
12/4/2020 11:34	191.6	186.1	182.8	2082	64.96
12/4/2020 11:34	191.7	186.1	183	2080	65.08
12/4/2020 11:34	191.8	186.2	182.8	2083	65.04
12/4/2020 11:34	191.8	186.4	182.8	2080	65.08
12/4/2020 11:34	191.8	185.9	183	2082	64.92
12/4/2020 11:34	191.9	186.1	182.6	2082	64.88
12/4/2020 11:34	191.9	186.4	182.5	2086	64.96
12/4/2020 11:34	192	186	182.9	2079	65.08
12/4/2020 11:34	192	186.3	183.4	2080	65.08
12/4/2020 11:34	192	185.9	183.6	2083	64.92
12/4/2020 11:34	192.1	185.7	184	2082	65.04
12/4/2020 11:34	192.1	186.4	183.6	2082	65.04
12/4/2020 11:34	192.1	185.7	183.7	2084	65.08
12/4/2020 11:34	192.2	186.3	183.3	2085	65.08
12/4/2020 11:34	192.2	186.3	182.9	2082	64.92
12/4/2020 11:34	192.2	186.5	182.8	2085	64.92
12/4/2020 11:34	192.2	187	182.7	2087	65
12/4/2020 11:34	192.2	187	182.8	2084	64.92
12/4/2020 11:34	192.2	186.9	183	2086	64.99
12/4/2020 11:34	192.2	187.3	182.8	2086	65
12/4/2020 11:34	192.2	187	182.9	2083	64.96

12/4/2020 11:34	192.2	187.1	183	2086	65.04
12/4/2020 11:34	192.2	187.3	182.8	2084	65.04
12/4/2020 11:34	192.3	187.5	183.1	2084	64.96
12/4/2020 11:34	192.2	187	183	2085	65.04
12/4/2020 11:34	192.2	187	182.9	2087	65
12/4/2020 11:34	192.2	187.5	183.2	2088	65.08
12/4/2020 11:34	192.2	187	182.8	2086	65
12/4/2020 11:34	192.2	187	183	2084	64.96
12/4/2020 11:34	192.2	187.1	183	2087	64.96
12/4/2020 11:34	192.2	187	182.8	2082	65.08
12/4/2020 11:34	192.2	187	183.1	2084	65.04
12/4/2020 11:34	192.1	187	183.2	2086	64.92
12/4/2020 11:34	192.2	187.4	183.8	2087	65
12/4/2020 11:34	192.1	187.4	183.8	2090	64.99
12/4/2020 11:34	192.1	187	184	2089	64.99
12/4/2020 11:34	192.1	187	183.7	2088	65.08
12/4/2020 11:34	192.1	187.2	183.5	2094	64.88
12/4/2020 11:34	192	186.8	182.8	2093	64.83
12/4/2020 11:34	192	187.2	182.7	2094	64.99
12/4/2020 11:34	191.9	187	182.7	2093	65.08
12/4/2020 11:34	192	187.4	183.1	2095	64.88
12/4/2020 11:34	191.8	187.2	183.4	2090	65.04
12/4/2020 11:34	191.8	186.9	183.2	2089	64.96
12/4/2020 11:34	191.8	187.2	183.2	2086	64.99
12/4/2020 11:35	191.7	186.9	183.2	2088	64.88
12/4/2020 11:35	191.8	187.3	183	2086	64.92
12/4/2020 11:35	191.7	187.4	183.8	2087	64.88
12/4/2020 11:35	191.7	186.8	184.2	2088	64.8
12/4/2020 11:35	191.6	187.1	183.2	2086	65.04
12/4/2020 11:35	191.6	187.2	182.8	2089	64.95
12/4/2020 11:35	191.6	187.3	183	2087	64.88
12/4/2020 11:35	191.5	187.1	183	2091	64.96
12/4/2020 11:35	191.4	186.8	183.4	2087	64.84
12/4/2020 11:35	191.4	186.9	183.4	2088	64.99
12/4/2020 11:35	191.4	187.1	183	2086	64.93
12/4/2020 11:35	191.3	187	183.1	2088	64.88
12/4/2020 11:35	191.3	187.1	183.1	2084	64.84
12/4/2020 11:35	191.3	187	183.5	2090	64.88
12/4/2020 11:35	191.1	187	184.4	2091	64.88
12/4/2020 11:35	191.1	187.1	183.9	2090	64.99
12/4/2020 11:35	191.1	187.2	183.6	2088	65.01
12/4/2020 11:35	191.1	187.3	183	2087	64.95
12/4/2020 11:35	191.1	187.2	183	2086	64.88
12/4/2020 11:35	191	187	182.9	2088	65
12/4/2020 11:35	190.9	187.4	183.2	2089	65.04
12/4/2020 11:35	190.9	187	183	2084	64.92
12/4/2020 11:35	190.9	187.4	183.2	2087	64.96

12/4/2020 11:35	190.8	187.2	183	2090	64.96
12/4/2020 11:35	190.8	186.9	183	2090	64.92
12/4/2020 11:35	190.8	187.4	183.4	2092	65
12/4/2020 11:35	190.7	187	183.1	2095	64.92
12/4/2020 11:35	190.6	187.2	183	2091	64.92
12/4/2020 11:35	190.6	187.1	183.2	2093	65.04
12/4/2020 11:35	190.5	187.4	183	2091	64.92
12/4/2020 11:35	190.5	187.4	183.1	2095	65
12/4/2020 11:35	190.5	186.9	183.2	2097	64.92
12/4/2020 11:35	190.4	187	183	2098	64.96
12/4/2020 11:35	190.3	187.2	183.9	2095	65
12/4/2020 11:35	190.3	187.1	183.7	2093	64.92
12/4/2020 11:35	190.3	187.4	183.3	2093	64.92
12/4/2020 11:35	190.2	187	183.2	2094	65.04
12/4/2020 11:35	190.1	187.3	183	2090	64.96
12/4/2020 11:35	190.1	186.9	183.3	2089	64.99
12/4/2020 11:35	190	187.2	183.2	2090	64.99
12/4/2020 11:35	190	187.4	182.9	2091	64.96
12/4/2020 11:35	190	187	183	2095	64.92
12/4/2020 11:35	189.8	187	183.8	2095	64.96
12/4/2020 11:35	189.8	186.8	183.9	2092	64.83
12/4/2020 11:35	189.7	187	183.2	2093	65.08
12/4/2020 11:35	189.7	187.3	183	2091	64.88
12/4/2020 11:35	189.6	187.2	183	2090	65.01
12/4/2020 11:35	189.6	187	183.4	2095	64.99
12/4/2020 11:35	189.7	187	183	2095	64.83
12/4/2020 11:35	189.6	187	183.1	2091	65.04
12/4/2020 11:35	189.6	187.1	183	2089	64.92
12/4/2020 11:35	189.4	186.9	183.2	2088	64.92
12/4/2020 11:35	189.4	187	183.8	2089	65
12/4/2020 11:35	189.4	187.4	183.9	2088	64.92
12/4/2020 11:35	189.3	187	183.2	2093	64.92
12/4/2020 11:35	189.3	187	183.1	2095	65
12/4/2020 11:35	189.3	187.2	183.1	2095	64.96
12/4/2020 11:35	189.2	186.9	183.2	2094	64.87
12/4/2020 11:35	189.2	187.4	183.2	2093	65
12/4/2020 11:35	189.1	187.1	183	2091	65
12/4/2020 11:36	189	187	182.9	2091	64.96
12/4/2020 11:36	188.9	187.4	183	2095	65
12/4/2020 11:36	188.9	187.1	183.9	2098	65.12
12/4/2020 11:36	188.9	187.2	184	2099	65.04
12/4/2020 11:36	188.8	187	183.2	2096	64.91
12/4/2020 11:36	188.8	187	183	2097	64.96
12/4/2020 11:36	188.8	187.4	183.4	2091	64.92
12/4/2020 11:36	188.7	187.3	183	2091	65.08
12/4/2020 11:36	188.7	187.2	183	2091	64.99
12/4/2020 11:36	188.7	187	183	2091	65.04

12/4/2020 11:36	188.6	187	183	2095	64.88
12/4/2020 11:36	188.6	187	183	2093	65.08
12/4/2020 11:36	188.5	187	183	2096	64.96
12/4/2020 11:36	188.5	187.4	183	2095	64.99
12/4/2020 11:36	188.4	187	183.8	2097	65.04
12/4/2020 11:36	188.4	186.8	184.1	2098	65.04
12/4/2020 11:36	188.4	187	183.9	2095	65
12/4/2020 11:36	188.3	186.8	183.7	2093	64.99
12/4/2020 11:36	188.3	187	183.5	2093	65.04
12/4/2020 11:36	188.3	187.1	183	2097	65.03
12/4/2020 11:36	188.1	187	183.1	2095	65.08
12/4/2020 11:36	188.2	187.2	182.7	2092	64.96
12/4/2020 11:36	188.1	187	182.8	2095	64.99
12/4/2020 11:36	188.1	187	182.9	2095	65
12/4/2020 11:36	188.1	187.2	182.8	2092	65
12/4/2020 11:36	188	187	183	2095	65
12/4/2020 11:36	188	187.4	183	2100	64.96
12/4/2020 11:36	187.9	186.8	183	2097	64.88
12/4/2020 11:36	187.9	186.9	183.2	2090	64.92
12/4/2020 11:36	187.8	187.4	183.1	2091	65.08
12/4/2020 11:36	187.8	187.2	183.2	2093	65
12/4/2020 11:36	187.8	187.3	183	2095	64.96
12/4/2020 11:36	187.7	186.9	182.9	2094	65.04
12/4/2020 11:36	187.6	187	182.8	2095	64.96
12/4/2020 11:36	187.6	187.4	183	2095	65
12/4/2020 11:36	187.6	187.2	183.1	2097	65.04
12/4/2020 11:36	187.5	187.3	183.5	2096	64.91
12/4/2020 11:36	187.4	187	183.7	2089	64.92
12/4/2020 11:36	187.4	187.2	183.8	2090	64.88
12/4/2020 11:36	187.3	187.4	183.2	2093	64.96
12/4/2020 11:36	187.4	186.8	182.8	2094	64.92
12/4/2020 11:36	187.3	187.2	182.7	2095	64.96
12/4/2020 11:36	187.3	186.8	182.9	2098	65.04
12/4/2020 11:36	187.2	187.3	183	2098	64.93
12/4/2020 11:36	187.2	187.1	183	2093	64.96
12/4/2020 11:36	187.1	187	183.2	2093	64.88
12/4/2020 11:36	187.1	187.2	182.8	2094	64.96
12/4/2020 11:36	187.1	186.9	183	2093	64.88
12/4/2020 11:36	187	186.7	183.3	2093	64.99
12/4/2020 11:36	187.1	187.4	183.5	2096	65.04
12/4/2020 11:36	187	186.9	183.2	2095	64.92
12/4/2020 11:36	186.9	187.3	182.5	2095	64.84
12/4/2020 11:36	186.9	187	183	2093	64.92
12/4/2020 11:36	186.8	187	183	2092	64.92
12/4/2020 11:36	186.8	187.4	183	2091	64.92
12/4/2020 11:36	186.7	187	183	2089	64.88
12/4/2020 11:36	186.7	187	183.7	2094	64.88

12/4/2020 11:36	186.7	187.2	183	2100	64.91
12/4/2020 11:36	186.6	187	182.7	2095	65
12/4/2020 11:37	186.6	187.5	182.8	2098	65
12/4/2020 11:37	186.5	187	183.4	2093	65
12/4/2020 11:37	186.5	187	183	2091	64.96
12/4/2020 11:37	186.4	186.9	182.5	2095	65.04
12/4/2020 11:37	186.4	187	183.1	2095	65
12/4/2020 11:37	186.3	187.3	183.5	2092	64.92
12/4/2020 11:37	186.3	187.1	183.5	2095	64.96
12/4/2020 11:37	186.3	186.8	183.1	2095	65.04
12/4/2020 11:37	186.2	186.8	182.5	2091	64.91
12/4/2020 11:37	186.2	187	182.5	2091	64.88
12/4/2020 11:37	186.2	187.2	182.7	2089	64.96
12/4/2020 11:37	186.2	187	182.7	2091	64.92
12/4/2020 11:37	186.1	187	182.5	2095	64.99
12/4/2020 11:37	186.1	187	182.7	2095	65.08
12/4/2020 11:37	186	186.9	182.8	2093	64.99
12/4/2020 11:37	186	186.4	182.6	2091	65.08
12/4/2020 11:37	186	185.9	182.8	2096	64.96
12/4/2020 11:37	185.9	185.9	182.4	2099	64.99
12/4/2020 11:37	185.9	186.4	182.8	2099	65.04
12/4/2020 11:37	185.9	185.8	182.8	2096	64.92
12/4/2020 11:37	185.9	186.5	183.1	2096	64.84
12/4/2020 11:37	185.8	186.2	183.7	2096	64.84
12/4/2020 11:37	185.8	185.6	183	2096	65.16
12/4/2020 11:37	185.8	186.5	182.5	2090	64.99
12/4/2020 11:37	185.7	185.6	183	2095	65.16
12/4/2020 11:37	185.7	186.5	182.7	2092	65.04
12/4/2020 11:37	185.7	186.2	182.6	2095	64.99
12/4/2020 11:37	185.6	185.7	182.5	2097	65.04
12/4/2020 11:37	185.7	186.1	183.3	2096	65
12/4/2020 11:37	185.6	185.7	183.6	2095	64.91
12/4/2020 11:37	185.5	186.1	182.9	2099	64.84
12/4/2020 11:37	185.6	186.1	182.4	2096	64.96
12/4/2020 11:37	185.6	186.1	182.8	2095	64.99
12/4/2020 11:37	185.5	186.5	182.8	2091	65
12/4/2020 11:37	185.5	186	182.4	2092	65.04
12/4/2020 11:37	185.5	186.4	182.8	2092	65.04
12/4/2020 11:37	185.5	185.7	183.4	2092	64.92
12/4/2020 11:37	185.5	185.7	183.1	2087	64.99
12/4/2020 11:37	185.5	186.1	182.8	2091	64.96
12/4/2020 11:37	185.5	185.7	182.9	2092	64.96
12/4/2020 11:37	185.5	186.4	182.9	2093	65.04
12/4/2020 11:37	185.7	186.5	182.8	2093	64.96
12/4/2020 11:37	185.5	185.6	182.4	2095	64.96
12/4/2020 11:37	185.5	186.2	182.6	2094	65.05
12/4/2020 11:37	185.5	186	183.5	2089	65

12/4/2020 11:37	185.5	185.7	183	2087	65.04
12/4/2020 11:37	185.4	186.4	182.7	2086	64.96
12/4/2020 11:37	185.5	185.8	182.8	2089	64.88
12/4/2020 11:37	185.5	186.1	182.7	2088	64.92
12/4/2020 11:37	185.5	186.3	182.8	2091	64.92
12/4/2020 11:37	185.6	185.9	182.3	2092	64.96
12/4/2020 11:37	185.6	186.5	182.8	2094	64.88
12/4/2020 11:37	185.5	186	183.8	2095	65
12/4/2020 11:37	185.6	186.1	182.6	2094	64.92
12/4/2020 11:37	185.6	186.2	183	2091	65.12
12/4/2020 11:37	185.6	185.6	182.8	2099	64.96
12/4/2020 11:37	185.7	186.5	182.5	2097	64.99
12/4/2020 11:37	185.7	186.1	182.7	2093	64.96
12/4/2020 11:37	185.6	186.4	182.9	2089	65
12/4/2020 11:37	185.7	186.2	183.6	2093	65.01
12/4/2020 11:38	185.7	185.6	183	2091	64.99
12/4/2020 11:38	185.8	186.3	182.5	2089	65
12/4/2020 11:38	185.7	185.9	182.8	2089	64.92
12/4/2020 11:38	185.7	186	182.9	2093	64.96
12/4/2020 11:38	185.7	186.2	182.5	2098	65.05
12/4/2020 11:38	185.8	185.7	182.5	2088	64.96
12/4/2020 11:38	185.8	186.3	183	2093	65.05
12/4/2020 11:38	185.8	186.2	183.4	2093	65
12/4/2020 11:38	185.9	186.4	182.7	2093	64.87
12/4/2020 11:38	185.9	186	182.7	2091	64.92
12/4/2020 11:38	185.9	185.8	182.7	2092	65.04
12/4/2020 11:38	185.9	186.4	182.9	2093	64.92
12/4/2020 11:38	185.9	186.2	182.3	2089	64.88
12/4/2020 11:38	185.9	185.8	182.7	2090	65.12
12/4/2020 11:38	186	186.1	183.3	2089	64.88
12/4/2020 11:38	186	185.6	183	2086	65.08
12/4/2020 11:38	186	186.1	182.7	2084	65.08
12/4/2020 11:38	186.1	185.7	182.7	2087	64.88
12/4/2020 11:38	186.1	186.5	182.9	2092	64.96
12/4/2020 11:38	186	186	182.6	2091	65.12
12/4/2020 11:38	186.1	185.8	182.2	2091	64.96
12/4/2020 11:38	186.1	186.3	183.2	2086	65.04
12/4/2020 11:38	186.2	186.4	183.5	2091	65.04
12/4/2020 11:38	186.1	186.2	182.5	2091	65.12
12/4/2020 11:38	186.1	186.1	182.8	2089	65.04
12/4/2020 11:38	186.2	185.9	182.9	2092	65.12
12/4/2020 11:38	186.2	186.1	182.6	2089	64.92
12/4/2020 11:38	186.2	186.3	182.7	2091	65.05
12/4/2020 11:38	186.2	185.8	182.7	2093	64.96
12/4/2020 11:38	186.3	186.4	183.7	2089	64.96
12/4/2020 11:38	186.3	185.8	183.2	2084	65
12/4/2020 11:38	186.3	186.4	182.8	2089	64.96

12/4/2020 11:38	186.3	185.7	183	2089	65
12/4/2020 11:38	186.3	185.8	182.8	2089	65
12/4/2020 11:38	186.3	186.3	182.5	2086	65.04
12/4/2020 11:38	186.3	185.8	182.7	2090	65
12/4/2020 11:38	186.4	186.3	183.2	2087	65.04
12/4/2020 11:38	186.4	186.3	183.3	2091	65.04
12/4/2020 11:38	186.5	185.8	182.9	2088	65.04
12/4/2020 11:38	186.5	186.2	182.8	2091	65.04
12/4/2020 11:38	186.5	186.1	182.7	2086	64.83
12/4/2020 11:38	186.6	186	182.8	2088	64.99
12/4/2020 11:38	186.6	186.4	182.4	2090	65.08
12/4/2020 11:38	186.7	186	182.5	2091	65.01
12/4/2020 11:38	186.7	186.2	183.4	2093	65.08
12/4/2020 11:38	186.7	186.6	183.3	2091	65.12
12/4/2020 11:38	186.8	186.4	182.7	2092	64.92
12/4/2020 11:38	186.8	186.3	182.5	2093	64.88
12/4/2020 11:38	186.8	185.8	182.7	2093	65.12
12/4/2020 11:38	186.8	185.9	183	2089	64.92
12/4/2020 11:38	186.8	185.7	182.5	2091	64.96
12/4/2020 11:38	187	186.2	182.5	2093	64.96
12/4/2020 11:38	186.9	186.4	182.7	2095	65
12/4/2020 11:38	187	186	183.4	2097	65.04
12/4/2020 11:38	187.1	185.9	183.4	2099	65.04
12/4/2020 11:38	187.1	185.8	182.5	2095	64.92
12/4/2020 11:38	187.2	185.7	182.6	2091	65
12/4/2020 11:38	187.1	186.4	183	2093	65.04
12/4/2020 11:38	187.2	185.8	182.7	2093	64.96
12/4/2020 11:39	187.2	185.9	182.5	2089	65.09
12/4/2020 11:39	187.2	186.3	182.7	2089	65.04
12/4/2020 11:39	187.2	186.1	182.6	2091	64.92
12/4/2020 11:39	187.2	186.2	183.4	2092	65.04
12/4/2020 11:39	187.2	186.1	183.3	2089	65.04
12/4/2020 11:39	187.2	185.8	182.5	2091	64.92
12/4/2020 11:39	187.2	185.8	182.8	2087	65.04
12/4/2020 11:39	187.3	185.8	182.9	2090	65.01
12/4/2020 11:39	187.3	186.4	182.6	2089	65.08
12/4/2020 11:39	187.3	186.2	182.9	2089	64.92
12/4/2020 11:39	187.3	185.8	182.7	2088	64.92
12/4/2020 11:39	187.3	185.8	182.8	2086	65.08
12/4/2020 11:39	187.3	186.2	182.5	2089	65.04
12/4/2020 11:39	187.3	186.4	183	2086	65.08
12/4/2020 11:39	187.3	186	183.5	2089	65.12
12/4/2020 11:39	187.3	186.4	183.2	2092	64.96
12/4/2020 11:39	187.3	186.4	183.2	2091	64.92
12/4/2020 11:39	187.3	185.7	183.3	2093	65.04
12/4/2020 11:39	187.2	185.9	183.2	2088	64.88
12/4/2020 11:39	187.3	185.7	183	2086	64.92

12/4/2020 11:39	187.2	185.7	183	2085	65
12/4/2020 11:39	187.2	186.5	183	2087	64.96
12/4/2020 11:39	187.2	186	183	2085	64.87
12/4/2020 11:39	187.2	186.2	183.6	2085	65.09
12/4/2020 11:39	187.2	186.3	183.5	2086	65
12/4/2020 11:39	187.1	185.7	183.6	2084	64.96
12/4/2020 11:39	187.1	186.5	183.4	2087	65.09
12/4/2020 11:39	187.1	186	182.7	2091	65.04
12/4/2020 11:39	187.1	185.6	182.5	2089	64.92
12/4/2020 11:39	187.1	185.8	182.2	2085	64.96
12/4/2020 11:39	187	185.8	182.4	2083	65.04
12/4/2020 11:39	187	186.2	182.7	2082	65.04
12/4/2020 11:39	187	186.1	182.7	2083	65.04
12/4/2020 11:39	187.1	186.4	183.1	2082	64.92
12/4/2020 11:39	187	186.2	182.8	2083	64.99
12/4/2020 11:39	187.1	185.7	182.7	2086	64.93
12/4/2020 11:39	187	186.3	182.5	2083	64.88
12/4/2020 11:39	187.1	185.8	183.2	2084	65
12/4/2020 11:39	187.1	186.1	183.3	2086	65
12/4/2020 11:39	187	186	182.4	2084	64.95
12/4/2020 11:39	187.1	185.5	182.7	2087	64.84
12/4/2020 11:39	187.1	186.3	182.7	2087	65
12/4/2020 11:39	187.1	186.4	182.9	2084	65.03
12/4/2020 11:39	187.2	185.7	182.7	2084	64.99
12/4/2020 11:39	187.2	186	183.7	2087	65.08
12/4/2020 11:39	187.3	185.8	183	2087	64.96
12/4/2020 11:39	187.2	186.2	182.5	2084	64.96
12/4/2020 11:39	187.3	186.4	182.5	2084	65
12/4/2020 11:39	187.3	186.5	183	2084	64.87
12/4/2020 11:39	187.4	186.3	182.7	2084	64.92
12/4/2020 11:39	187.4	185.7	182.8	2085	65
12/4/2020 11:39	187.5	186	183.3	2086	64.96
12/4/2020 11:39	187.5	185.6	183.6	2085	64.96
12/4/2020 11:39	187.6	185.6	182.7	2083	65
12/4/2020 11:39	187.6	186.3	182.6	2083	65
12/4/2020 11:39	187.7	186	182.8	2082	64.92
12/4/2020 11:39	187.8	186.5	182.9	2082	65.05
12/4/2020 11:39	187.8	185.8	183	2084	64.92
12/4/2020 11:39	187.8	185.8	183	2082	65.04
12/4/2020 11:39	187.9	186.3	183	2081	65.01
12/4/2020 11:40	188	185.7	182.9	2080	64.8
12/4/2020 11:40	188.1	186.2	183.7	2082	64.92
12/4/2020 11:40	188.1	186.4	183.7	2080	64.96
12/4/2020 11:40	188.2	185.7	183.4	2075	65.04
12/4/2020 11:40	188.3	186.2	183	2078	64.88
12/4/2020 11:40	188.3	185.7	182.5	2079	65.04
12/4/2020 11:40	188.4	185.8	182.5	2075	65.04

12/4/2020 11:40	188.5	186.4	182.7	2075	64.95
12/4/2020 11:40	188.6	186.1	182.5	2074	64.92
12/4/2020 11:40	188.7	186.2	182.8	2073	65.08
12/4/2020 11:40	188.7	185.8	182.7	2072	65.03
12/4/2020 11:40	188.8	185.6	182.5	2071	64.92
12/4/2020 11:40	188.8	186.5	182.8	2069	64.88
12/4/2020 11:40	188.9	186	182.8	2074	64.83
12/4/2020 11:40	189	186.2	182.7	2075	65.12
12/4/2020 11:40	189.1	186.2	182.9	2075	65.04
12/4/2020 11:40	189.3	185.6	183.2	2073	64.88
12/4/2020 11:40	189.3	186.1	183.5	2075	65.04
12/4/2020 11:40	189.4	185.8	183.8	2073	64.88
12/4/2020 11:40	189.5	186.2	183.7	2076	64.92
12/4/2020 11:40	189.6	186.2	183.5	2075	64.92
12/4/2020 11:40	189.7	185.6	183	2077	64.96
12/4/2020 11:40	189.8	186.5	182.7	2076	64.96
12/4/2020 11:40	189.8	186	183.2	2075	65.09
12/4/2020 11:40	190	185.7	183.2	2069	65
12/4/2020 11:40	190.1	185.9	183.1	2069	64.96
12/4/2020 11:40	190.2	186.1	183	2073	65.04
12/4/2020 11:40	190.3	186.5	183	2076	65.04
12/4/2020 11:40	190.4	186.2	183.8	2075	64.96
12/4/2020 11:40	190.5	185.9	183.6	2079	64.96
12/4/2020 11:40	190.6	185.8	183	2083	64.92
12/4/2020 11:40	190.7	186	183.4	2084	65.04
12/4/2020 11:40	190.7	186.4	183.2	2084	64.96
12/4/2020 11:40	190.7	186.1	183	2090	65.08
12/4/2020 11:40	190.8	185.8	182.9	2092	64.88
12/4/2020 11:40	190.9	186.4	183.2	2094	65.12
12/4/2020 11:40	190.9	185.5	183.9	2089	65.08
12/4/2020 11:40	190.9	186.1	183.7	2088	64.84
12/4/2020 11:40	191	186.4	183.2	2093	65.01
12/4/2020 11:40	191	186.5	183.5	2093	64.95
12/4/2020 11:40	191	185.9	183.3	2095	64.88
12/4/2020 11:40	190.9	185.6	183.2	2098	64.96
12/4/2020 11:40	191	186.1	183	2096	64.92
12/4/2020 11:40	191.1	186.2	183.2	2093	64.92
12/4/2020 11:40	191.1	186.4	183.9	2091	65.16
12/4/2020 11:40	191.1	186.3	184.2	2091	64.99
12/4/2020 11:40	191.1	186.1	184	2095	65.04
12/4/2020 11:40	191.1	186.2	183.2	2097	65.04
12/4/2020 11:40	191.1	185.7	183.2	2095	65.04
12/4/2020 11:40	191	185.8	183.5	2091	64.96
12/4/2020 11:40	191	186.5	183.3	2098	65
12/4/2020 11:40	191	186	183.2	2098	64.96
12/4/2020 11:40	190.9	186.3	183.2	2099	64.96
12/4/2020 11:40	190.9	186.1	183.2	2099	65.09

12/4/2020 11:40	190.8	185.6	183.3	2100	64.96
12/4/2020 11:40	190.9	186.2	183	2100	65.04
12/4/2020 11:40	190.8	185.8	183.5	2099	65.04
12/4/2020 11:40	190.8	186.3	183.7	2102	64.96
12/4/2020 11:40	190.7	186	184.2	2104	64.99
12/4/2020 11:41	190.6	185.6	184.4	2106	65.04
12/4/2020 11:41	190.6	186.2	183.9	2107	64.96
12/4/2020 11:41	190.6	186.1	183.3	2108	64.8
12/4/2020 11:41	190.5	186.5	183.3	2106	64.97
12/4/2020 11:41	190.5	186.3	183.2	2109	64.88
12/4/2020 11:41	190.4	185.8	183.2	2109	64.92
12/4/2020 11:41	190.3	186.1	183.4	2109	64.97
12/4/2020 11:41	190.2	186.2	183.3	2114	64.88
12/4/2020 11:41	190	186.6	183.5	2109	65.04
12/4/2020 11:41	190	186.3	183.5	2113	65.08
12/4/2020 11:41	189.9	185.7	183.4	2110	64.83
12/4/2020 11:41	189.8	186.2	183.4	2110	64.88
12/4/2020 11:41	189.8	186.2	183	2110	64.96
12/4/2020 11:41	189.7	185.8	183	2112	64.96
12/4/2020 11:41	189.7	186.3	183.2	2108	64.92
12/4/2020 11:41	189.6	186	183	2107	65
12/4/2020 11:41	189.5	186.3	183	2100	65.04
12/4/2020 11:41	189.5	186	183	2095	64.92
12/4/2020 11:41	189.4	186	183	2096	65.04
12/4/2020 11:41	189.3	186.5	183.4	2094	64.96
12/4/2020 11:41	189.2	185.7	183.5	2096	64.92
12/4/2020 11:41	189.1	186.2	183.6	2098	64.96
12/4/2020 11:41	189.1	186.3	184	2099	65
12/4/2020 11:41	189.1	185.6	184.1	2098	65
12/4/2020 11:41	189	186.2	184	2095	64.84
12/4/2020 11:41	189	185.9	184.1	2095	65
12/4/2020 11:41	188.9	186.5	184.1	2088	64.99
12/4/2020 11:41	188.9	186.4	184.1	2091	65.08
12/4/2020 11:41	189	185.7	184	2091	64.88
12/4/2020 11:41	188.9	186.2	184	2093	65.03
12/4/2020 11:41	188.8	185.8	183.9	2097	65.04
12/4/2020 11:41	188.8	186.2	183.5	2098	65.04
12/4/2020 11:41	188.8	186.4	183.2	2101	64.96
12/4/2020 11:41	188.7	185.8	183.1	2101	64.88
12/4/2020 11:41	188.8	186	182.8	2106	64.93
12/4/2020 11:41	188.8	186	183	2109	64.88
12/4/2020 11:41	188.7	186	183.1	2112	64.99
12/4/2020 11:41	188.8	186.4	183	2109	65.12
12/4/2020 11:41	188.7	186.3	183.4	2109	64.99
12/4/2020 11:41	188.6	186.3	183.4	2105	64.91
12/4/2020 11:41	188.6	185.8	183.4	2105	64.92
12/4/2020 11:41	188.5	185.8	183.4	2106	65.08

12/4/2020 11:41	188.5	186	183	2102	64.83
12/4/2020 11:41	188.5	186.1	183.6	2107	65.08
12/4/2020 11:41	188.5	186.5	184.1	2102	65.04
12/4/2020 11:41	188.4	186	183.8	2102	64.92
12/4/2020 11:41	188.3	185.8	183.1	2105	65
12/4/2020 11:41	188.4	185.8	182.7	2109	65.04
12/4/2020 11:41	188.3	185.9	183	2105	64.96
12/4/2020 11:41	188.3	186.4	183.2	2100	65
12/4/2020 11:41	188.3	185.9	183.4	2098	65.05
12/4/2020 11:41	188.3	185.7	183	2097	65
12/4/2020 11:41	188.2	186.3	183.4	2099	64.96
12/4/2020 11:41	188.2	185.9	183.2	2101	65.17
12/4/2020 11:41	188.1	186.3	183.1	2099	64.88
12/4/2020 11:41	188.1	186.2	183.3	2097	65.01
12/4/2020 11:41	188.1	186.3	183.1	2100	65.04
12/4/2020 11:41	188	186.1	183.4	2098	64.88
12/4/2020 11:41	188	185.6	183.2	2099	64.88
12/4/2020 11:42	188	186.1	182.8	2095	64.88
12/4/2020 11:42	188	186.3	183.2	2091	64.88
12/4/2020 11:42	187.9	185.8	183.1	2093	64.92
12/4/2020 11:42	187.9	186.3	182.9	2096	65.12
12/4/2020 11:42	187.9	186	183.1	2098	64.99
12/4/2020 11:42	187.9	186.2	183	2097	65.12
12/4/2020 11:42	187.9	186.3	183.1	2094	65.04
12/4/2020 11:42	187.9	185.7	183.2	2094	64.96
12/4/2020 11:42	187.9	186.1	183	2091	65.04
12/4/2020 11:42	187.8	186	183.4	2091	64.88
12/4/2020 11:42	187.7	186.4	183.2	2089	65.08
12/4/2020 11:42	187.8	186.3	183	2089	65.04
12/4/2020 11:42	187.8	185.6	183	2093	65.01
12/4/2020 11:42	187.8	186.3	182.9	2088	64.92
12/4/2020 11:42	187.7	186.1	182.9	2084	65
12/4/2020 11:42	187.7	186.3	184	2088	65.04
12/4/2020 11:42	187.7	186.4	183.5	2084	64.92
12/4/2020 11:42	187.7	185.7	182.8	2084	64.96
12/4/2020 11:42	187.7	186.2	183.1	2088	65.04
12/4/2020 11:42	187.7	185.6	182.8	2087	64.96
12/4/2020 11:42	187.7	185.9	182.9	2087	64.92
12/4/2020 11:42	187.7	186.5	183	2084	65
12/4/2020 11:42	187.8	185.8	183	2085	64.88
12/4/2020 11:42	187.7	186.1	183.9	2086	65.04
12/4/2020 11:42	187.7	186.1	183.5	2089	65.09
12/4/2020 11:42	187.7	185.8	182.8	2086	65.04
12/4/2020 11:42	187.9	186.5	183.2	2088	65.04
12/4/2020 11:42	187.8	185.8	183.2	2086	65.04
12/4/2020 11:42	187.9	186.3	183.2	2086	65.08
12/4/2020 11:42	187.9	185.8	182.8	2082	64.92

12/4/2020 11:42	188	185.9	182.6	2082	64.92
12/4/2020 11:42	188	186.4	183.5	2082	65
12/4/2020 11:42	188.1	186	184.1	2084	65.04
12/4/2020 11:42	188.2	186.2	183.2	2084	65.12
12/4/2020 11:42	188.3	185.6	183.1	2087	64.88
12/4/2020 11:42	188.3	185.8	183.3	2085	65.04
12/4/2020 11:42	188.3	186.4	183	2083	64.92
12/4/2020 11:42	188.4	186.1	183.1	2082	64.92
12/4/2020 11:42	188.5	186.4	182.9	2080	64.96
12/4/2020 11:42	188.5	185.7	183	2084	64.96
12/4/2020 11:42	188.7	186.1	183.7	2082	65.04
12/4/2020 11:42	188.7	186.3	183.8	2080	64.96
12/4/2020 11:42	188.8	185.9	183.7	2082	64.96
12/4/2020 11:42	189	186	183.2	2082	64.96
12/4/2020 11:42	189	186.4	183	2086	65.09
12/4/2020 11:42	189.1	186.4	183.4	2087	65.04
12/4/2020 11:42	189.2	186.3	183.2	2088	65
12/4/2020 11:42	189.3	187	183	2087	65.04
12/4/2020 11:42	189.4	187	183.2	2083	64.92
12/4/2020 11:42	189.5	187.1	182.8	2080	64.92
12/4/2020 11:42	189.6	187	183	2084	65.04
12/4/2020 11:42	189.8	187.4	183.5	2087	64.88
12/4/2020 11:42	189.8	187.3	183.8	2089	64.96
12/4/2020 11:42	189.9	186.9	184	2089	64.96
12/4/2020 11:42	190	187	183.5	2088	64.96
12/4/2020 11:42	190.1	187	183.4	2090	64.84
12/4/2020 11:42	190.2	187.4	183.3	2093	64.92
12/4/2020 11:42	190.3	186.9	183	2096	65.04
12/4/2020 11:42	190.5	186.9	183	2092	64.93
12/4/2020 11:42	190.5	187.3	183.4	2096	64.84
12/4/2020 11:43	190.6	187.2	183.2	2094	64.99
12/4/2020 11:43	190.7	187.4	183.7	2091	65.08
12/4/2020 11:43	190.7	187.4	183.6	2091	64.92
12/4/2020 11:43	190.8	186.8	183.2	2089	64.99
12/4/2020 11:43	190.8	187.1	183.5	2091	65.08
12/4/2020 11:43	190.9	187.2	183.5	2093	64.92
12/4/2020 11:43	190.9	186.8	183.4	2092	64.92
12/4/2020 11:43	190.9	186.9	183.6	2093	65.12
12/4/2020 11:43	191.1	186.9	183.3	2092	64.88
12/4/2020 11:43	191.1	187.2	183.2	2095	64.96
12/4/2020 11:43	191.1	187	183.6	2095	64.96
12/4/2020 11:43	191.2	187	183.4	2101	64.92
12/4/2020 11:43	191.2	187.4	183	2106	65
12/4/2020 11:43	191.3	186.8	183.4	2103	65.04
12/4/2020 11:43	191.3	187.2	183.2	2104	65
12/4/2020 11:43	191.2	187	183.2	2104	64.96
12/4/2020 11:43	191.4	187	183.4	2100	65.09

12/4/2020 11:43	191.3	187.4	183	2100	64.96
12/4/2020 11:43	191.4	186.9	183.5	2102	65
12/4/2020 11:43	191.4	187.1	183.2	2098	65.04
12/4/2020 11:43	191.3	186.9	183.2	2099	65.08
12/4/2020 11:43	191.4	187.2	183.2	2095	65.08
12/4/2020 11:43	191.4	187.5	183.2	2104	64.88
12/4/2020 11:43	191.4	187	183.4	2106	64.88
12/4/2020 11:43	191.4	187.3	183.2	2103	64.93
12/4/2020 11:43	191.5	186.8	183.4	2106	65.16
12/4/2020 11:43	191.4	187	183.7	2106	64.96
12/4/2020 11:43	191.4	186.8	183.2	2105	65.17
12/4/2020 11:43	191.4	187.2	183.4	2105	65.04
12/4/2020 11:43	191.4	187.3	183.6	2101	65.01
12/4/2020 11:43	191.4	187.2	183.2	2102	65
12/4/2020 11:43	191.4	186.9	183.2	2104	64.92
12/4/2020 11:43	191.4	187.2	184	2105	64.96
12/4/2020 11:43	191.3	187.1	184.5	2097	64.92
12/4/2020 11:43	191.4	187.4	184.3	2102	65
12/4/2020 11:43	191.3	187.2	183.5	2103	65.04
12/4/2020 11:43	191.4	187	183.2	2106	65.09
12/4/2020 11:43	191.3	187.2	183.6	2105	64.96
12/4/2020 11:43	191.3	186.8	183.8	2101	65
12/4/2020 11:43	191.3	187.1	183.5	2098	65
12/4/2020 11:43	191.3	187.2	183.7	2104	65.04
12/4/2020 11:43	191.3	186.9	183.6	2103	65
12/4/2020 11:43	191.1	187.2	184.2	2108	64.96
12/4/2020 11:43	191.3	187	184.3	2109	64.92
12/4/2020 11:43	191.1	187.2	183.4	2113	65.04
12/4/2020 11:43	191.1	187.4	183.2	2109	65.01
12/4/2020 11:43	191.1	186.9	183.8	2102	64.84
12/4/2020 11:43	191.1	187.2	183.8	2102	65.08
12/4/2020 11:43	191.1	187	183.8	2101	65.08
12/4/2020 11:43	191	187.2	183.7	2102	64.99
12/4/2020 11:43	191	187.3	183.4	2101	65.04
12/4/2020 11:43	191	187	184.4	2103	64.99
12/4/2020 11:43	190.9	187.2	184.2	2102	65.04
12/4/2020 11:43	190.9	187	183.5	2098	65.04
12/4/2020 11:43	190.9	187	183.5	2103	65.01
12/4/2020 11:43	190.9	187.4	183.5	2104	64.99
12/4/2020 11:43	190.8	187.1	183.8	2104	65.04
12/4/2020 11:43	190.7	187.2	183.7	2108	65.12
12/4/2020 11:43	190.8	187.3	183.7	2105	64.96
12/4/2020 11:44	190.7	187.1	183.7	2108	65
12/4/2020 11:44	190.7	187.4	183.6	2104	65.04
12/4/2020 11:44	190.6	187	183.6	2103	64.96
12/4/2020 11:44	190.6	187.2	183.9	2103	64.96
12/4/2020 11:44	190.5	187.1	184.2	2106	65.05

12/4/2020 11:44	190.5	187	184.4	2101	65
12/4/2020 11:44	190.5	187	184.4	2104	64.92
12/4/2020 11:44	190.5	187	184.1	2106	65.05
12/4/2020 11:44	190.4	187.2	184.1	2107	64.92
12/4/2020 11:44	190.4	187.4	184.2	2104	65
12/4/2020 11:44	190.3	187.3	184.1	2108	64.96
12/4/2020 11:44	190.3	187.2	184.3	2108	64.83
12/4/2020 11:44	190.1	186.8	184.2	2105	64.96
12/4/2020 11:44	190.2	187	184.3	2111	65.09
12/4/2020 11:44	190.2	187.4	184.6	2108	64.96
12/4/2020 11:44	190.1	186.8	184.4	2106	64.99
12/4/2020 11:44	190.1	187.4	184.1	2112	65.04
12/4/2020 11:44	190	187	183.9	2116	65.04
12/4/2020 11:44	190	187	183.4	2112	64.92
12/4/2020 11:44	190	187	183.7	2110	65.04
12/4/2020 11:44	189.8	187.4	183.5	2109	65.04
12/4/2020 11:44	189.8	187.4	183.6	2109	64.84
12/4/2020 11:44	189.8	187	183.7	2109	64.88
12/4/2020 11:44	189.8	187.2	183.5	2111	64.88
12/4/2020 11:44	189.7	187.4	183.4	2107	64.84
12/4/2020 11:44	189.6	186.7	183.5	2107	65.01
12/4/2020 11:44	189.6	187.2	184.2	2107	64.96
12/4/2020 11:44	189.6	186.9	184.2	2102	64.95
12/4/2020 11:44	189.6	187.5	183.5	2105	65.08
12/4/2020 11:44	189.5	187	183.6	2109	64.88
12/4/2020 11:44	189.5	186.8	183.5	2109	64.99
12/4/2020 11:44	189.5	187.1	183.8	2108	64.96
12/4/2020 11:44	189.4	187	183.6	2112	64.92
12/4/2020 11:44	189.4	187.4	183.5	2113	64.96
12/4/2020 11:44	189.3	187.4	184.2	2112	65.04
12/4/2020 11:44	189.3	186.9	184.3	2110	64.88
12/4/2020 11:44	189.2	187.2	183.8	2113	64.87
12/4/2020 11:44	189.2	186.9	183.5	2113	65.04
12/4/2020 11:44	189.1	187.4	183.9	2111	64.96
12/4/2020 11:44	189.1	187.4	183.9	2113	64.96
12/4/2020 11:44	189.1	186.8	183.7	2112	65
12/4/2020 11:44	189	187.2	183.5	2113	64.92
12/4/2020 11:44	189	187	183.4	2114	64.91
12/4/2020 11:44	189	187	184.1	2119	65.04
12/4/2020 11:44	188.9	187.2	184.6	2116	64.88
12/4/2020 11:44	188.8	187	183.5	2118	64.99
12/4/2020 11:44	188.8	187.4	183.7	2115	65.12
12/4/2020 11:44	188.8	187.2	183.9	2115	64.96
12/4/2020 11:44	188.7	186.9	183.8	2114	65.04
12/4/2020 11:44	188.7	187.4	183.7	2116	65.09
12/4/2020 11:44	188.7	187	183.4	2115	64.99
12/4/2020 11:44	188.6	187	183.4	2112	64.91

12/4/2020 11:44	188.6	187.2	183.5	2116	65.04
12/4/2020 11:44	188.5	186.9	184.1	2115	64.95
12/4/2020 11:44	188.5	187	184.6	2118	64.99
12/4/2020 11:44	188.5	187	184.3	2118	65.16
12/4/2020 11:44	188.4	187	183.6	2117	64.83
12/4/2020 11:44	188.4	187.4	183.2	2117	64.99
12/4/2020 11:44	188.4	187.3	183.4	2117	65.12
12/4/2020 11:44	188.3	187.3	183.4	2115	64.8
12/4/2020 11:45	188.3	187	183.5	2112	64.99
12/4/2020 11:45	188.3	187.1	183.4	2111	65.09
12/4/2020 11:45	188.2	187.4	183	2113	65.04
12/4/2020 11:45	188.2	186.9	184.1	2112	64.99
12/4/2020 11:45	188.2	187.2	184.3	2113	65.09
12/4/2020 11:45	188.1	187	183.2	2112	64.83
12/4/2020 11:45	188	187.1	183.5	2113	64.92
12/4/2020 11:45	188	187.3	183.5	2113	64.96
12/4/2020 11:45	188	187.2	183.7	2115	65
12/4/2020 11:45	187.9	187.3	183.3	2120	64.92
12/4/2020 11:45	187.8	187	183	2120	65.12
12/4/2020 11:45	187.8	187.1	183.8	2121	64.96
12/4/2020 11:45	187.8	187.4	184.4	2116	64.96
12/4/2020 11:45	187.8	186.9	183.8	2113	65.05
12/4/2020 11:45	187.7	187.2	183.3	2116	64.92
12/4/2020 11:45	187.7	187.2	183.5	2116	64.96
12/4/2020 11:45	187.6	187	183.5	2120	65.05
12/4/2020 11:45	187.5	186.9	183.7	2120	64.83
12/4/2020 11:45	187.5	187.1	183.4	2118	64.96
12/4/2020 11:45	187.5	187.3	183.4	2118	65.12
12/4/2020 11:45	187.4	187.2	183.4	2118	64.84
12/4/2020 11:45	187.4	187	183.2	2118	64.88
12/4/2020 11:45	187.4	187	183.8	2116	64.97
12/4/2020 11:45	187.3	186.9	184.4	2114	64.88
12/4/2020 11:45	187.2	187.1	183.8	2116	65.12
12/4/2020 11:45	187.3	187.2	183.6	2116	64.97
12/4/2020 11:45	187.2	187.5	183.2	2115	64.99
12/4/2020 11:45	187.2	187	183.6	2112	64.88
12/4/2020 11:45	187.1	186.8	183.8	2116	65
12/4/2020 11:45	187.1	187.1	183.4	2113	64.88
12/4/2020 11:45	187	187.4	182.9	2113	65.03
12/4/2020 11:45	187	187	183.3	2113	64.96
12/4/2020 11:45	187	187.3	183.4	2111	65
12/4/2020 11:45	187	186.9	184.1	2109	64.92
12/4/2020 11:45	186.9	187	184.1	2112	65
12/4/2020 11:45	186.8	187	183.5	2113	65.04
12/4/2020 11:45	186.8	187	183.4	2109	64.96
12/4/2020 11:45	186.8	187.3	183.2	2120	65.04
12/4/2020 11:45	186.8	186.9	183.4	2118	65

12/4/2020 11:45	186.6	186.8	183.6	2114	64.96
12/4/2020 11:45	186.7	186.9	183.6	2116	65
12/4/2020 11:45	186.6	186.8	183.2	2113	64.96
12/4/2020 11:45	186.6	187.2	183.4	2111	64.96
12/4/2020 11:45	186.5	186.8	183.4	2116	65
12/4/2020 11:45	186.5	187	183.2	2118	65
12/4/2020 11:45	186.5	187.4	183.3	2111	65
12/4/2020 11:45	186.5	187.2	183.2	2115	65
12/4/2020 11:45	186.4	187.4	183.2	2113	64.92
12/4/2020 11:45	186.3	187.3	183	2111	64.99
12/4/2020 11:45	186.3	187	183	2114	64.92
12/4/2020 11:45	186.3	187	183	2114	65.04
12/4/2020 11:45	186.3	187	183.1	2116	65.04
12/4/2020 11:45	186.2	187.2	183	2114	64.88
12/4/2020 11:45	186.2	187.2	183	2111	64.99
12/4/2020 11:45	186.2	186.9	183	2110	65.03
12/4/2020 11:45	186.1	187.4	183	2109	64.84
12/4/2020 11:45	186.1	187.1	183	2107	65.03
12/4/2020 11:45	186.1	187.3	183.4	2105	65.04
12/4/2020 11:45	186	187	183.4	2103	64.92
12/4/2020 11:46	186.1	186.9	183.4	2108	65.04
12/4/2020 11:46	186	187.2	183.4	2105	64.92
12/4/2020 11:46	186	187.2	183.2	2102	65.04
12/4/2020 11:46	186	187.3	183.6	2103	65.08
12/4/2020 11:46	186	186.8	184.1	2105	65.04
12/4/2020 11:46	186	186.9	183.2	2104	64.88
12/4/2020 11:46	185.9	187.1	183	2104	64.96
12/4/2020 11:46	185.9	187	183.5	2109	65.04
12/4/2020 11:46	186	187	183.3	2108	65.04
12/4/2020 11:46	185.9	186.9	183.1	2109	64.96
12/4/2020 11:46	185.9	187	184.1	2107	64.95
12/4/2020 11:46	186	187.2	183.7	2106	64.92
12/4/2020 11:46	185.9	187.1	183	2104	64.96
12/4/2020 11:46	185.9	187.4	183.2	2105	64.96
12/4/2020 11:46	185.9	187.4	182.7	2105	64.91
12/4/2020 11:46	185.9	186.8	183.3	2105	65
12/4/2020 11:46	186	187.3	182.9	2108	64.96
12/4/2020 11:46	186	187	183.2	2113	64.96
12/4/2020 11:46	186	187.2	183.8	2108	64.96
12/4/2020 11:46	186	187.3	184.2	2105	64.87
12/4/2020 11:46	186.1	186.8	183.9	2109	64.96
12/4/2020 11:46	186	187.2	183.9	2107	64.96
12/4/2020 11:46	186.1	186.8	183.6	2106	65
12/4/2020 11:46	186.1	187.2	183.7	2108	64.96
12/4/2020 11:46	186.1	187.4	183.7	2110	65.04
12/4/2020 11:46	186.1	187	183.7	2109	65
12/4/2020 11:46	186.1	186.3	183.7	2109	64.88

12/4/2020 11:46	186.1	186	183.9	2108	65.04
12/4/2020 11:46	186.2	186.5	184.2	2110	65.04
12/4/2020 11:46	186.2	185.9	184.1	2110	65.04
12/4/2020 11:46	186.2	185.7	183.8	2116	65.08
12/4/2020 11:46	186.2	186.5	183.5	2115	65.04
12/4/2020 11:46	186.2	185.9	183	2111	65.03
12/4/2020 11:46	186.2	186.5	183	2110	65.04
12/4/2020 11:46	186.3	186.2	183.2	2106	64.99
12/4/2020 11:46	186.3	185.6	183.4	2104	64.88
12/4/2020 11:46	186.3	186	183.5	2106	64.92
12/4/2020 11:46	186.3	185.8	183.3	2107	65.04
12/4/2020 11:46	186.3	185.8	183.2	2109	65.12
12/4/2020 11:46	186.3	186.3	182.9	2105	64.99
12/4/2020 11:46	186.3	186.2	183.6	2105	64.99
12/4/2020 11:46	186.4	186.2	183.8	2107	65
12/4/2020 11:46	186.4	185.8	183.5	2107	64.93
12/4/2020 11:46	186.3	186.1	183.3	2112	64.88
12/4/2020 11:46	186.4	186.4	183.2	2111	65
12/4/2020 11:46	186.5	186.2	183.6	2109	64.88
12/4/2020 11:46	186.5	186.4	183	2106	65.04
12/4/2020 11:46	186.5	186	182.9	2108	65
12/4/2020 11:46	186.6	185.7	183.9	2113	65.04
12/4/2020 11:46	186.6	186.4	184.1	2109	64.96
12/4/2020 11:46	186.6	186.1	183.5	2109	65
12/4/2020 11:46	186.6	186.5	183.2	2104	64.92
12/4/2020 11:46	186.7	186.1	183.1	2107	64.96
12/4/2020 11:46	186.7	185.7	182.9	2109	64.92
12/4/2020 11:46	186.7	185.9	183.1	2111	64.96
12/4/2020 11:46	186.8	186.1	182.9	2108	64.92
12/4/2020 11:46	186.7	186.2	183	2104	64.96
12/4/2020 11:46	186.8	186.2	183.9	2102	64.99
12/4/2020 11:46	186.8	186.2	184	2104	64.92
12/4/2020 11:47	186.8	186.3	183.4	2103	65
12/4/2020 11:47	186.9	185.8	183	2102	64.96
12/4/2020 11:47	186.9	186.6	183.4	2102	64.92
12/4/2020 11:47	187	186.4	183.5	2107	64.96
12/4/2020 11:47	187	185.6	183.1	2113	65.04
12/4/2020 11:47	187	186.2	183.2	2113	64.79
12/4/2020 11:47	187	185.9	183	2112	65.01
12/4/2020 11:47	187	186.1	183	2109	65.04
12/4/2020 11:47	187.1	185.7	183.8	2109	64.96
12/4/2020 11:47	187.1	186	183.9	2113	64.96
12/4/2020 11:47	187.1	186.2	183.7	2113	64.88
12/4/2020 11:47	187.2	186	183.3	2107	64.88
12/4/2020 11:47	187.2	185.8	183.1	2107	64.92
12/4/2020 11:47	187.2	185.6	183.5	2108	64.84
12/4/2020 11:47	187.2	185.9	183.4	2107	64.88

12/4/2020 11:47	187.3	186.1	183.4	2109	64.96
12/4/2020 11:47	187.3	186.2	183	2113	65.04
12/4/2020 11:47	187.3	186	183	2113	64.99
12/4/2020 11:47	187.4	186.3	182.7	2115	65.12
12/4/2020 11:47	187.3	185.6	183.4	2111	64.95
12/4/2020 11:47	187.3	186.3	183.9	2115	64.96
12/4/2020 11:47	187.4	186.1	184.1	2118	65.08
12/4/2020 11:47	187.4	186.2	183.4	2120	64.8
12/4/2020 11:47	187.4	186.5	182.9	2114	65.04
12/4/2020 11:47	187.4	185.9	183.1	2111	64.96
12/4/2020 11:47	187.4	186.4	183.3	2115	64.88
12/4/2020 11:47	187.4	185.7	183.1	2111	64.96
12/4/2020 11:47	187.4	185.8	183	2110	65
12/4/2020 11:47	187.4	186.3	183.2	2109	65
12/4/2020 11:47	187.4	185.8	183	2107	64.96
12/4/2020 11:47	187.5	185.9	184.1	2109	65.01
12/4/2020 11:47	187.4	185.9	184	2109	64.92
12/4/2020 11:47	187.4	185.7	183.6	2108	64.96
12/4/2020 11:47	187.4	186.2	183.2	2108	64.96
12/4/2020 11:47	187.4	185.9	183.2	2109	65.04
12/4/2020 11:47	187.4	186.3	183.4	2112	65
12/4/2020 11:47	187.4	186.2	183.3	2108	64.96
12/4/2020 11:47	187.4	185.7	183.1	2110	64.96
12/4/2020 11:47	187.4	186	183.2	2104	64.88
12/4/2020 11:47	187.4	185.8	183.2	2102	65.04
12/4/2020 11:47	187.4	186.1	183.1	2099	64.96
12/4/2020 11:47	187.5	186.4	183.2	2103	64.99
12/4/2020 11:47	187.4	185.8	183	2097	64.92
12/4/2020 11:47	187.5	186.3	183.2	2097	64.99
12/4/2020 11:47	187.5	185.8	183.1	2098	64.99
12/4/2020 11:47	187.5	185.8	183.3	2100	64.92
12/4/2020 11:47	187.6	186.3	183.8	2100	65
12/4/2020 11:47	187.6	186.1	184.1	2099	64.88
12/4/2020 11:47	187.7	186.3	184	2100	64.93
12/4/2020 11:47	187.7	186	184.1	2100	64.92
12/4/2020 11:47	187.8	185.6	183.8	2100	64.88
12/4/2020 11:47	187.9	186.5	183.2	2098	65.12
12/4/2020 11:47	187.9	185.9	183.5	2096	64.88
12/4/2020 11:47	187.9	186.3	183	2097	64.99
12/4/2020 11:47	188.1	186.1	183	2097	65.12
12/4/2020 11:47	188.1	185.8	183.4	2091	64.99
12/4/2020 11:47	188.2	185.9	183.2	2088	64.83
12/4/2020 11:47	188.3	185.8	183.5	2083	64.99
12/4/2020 11:47	188.3	185.9	183.4	2087	64.88
12/4/2020 11:47	188.5	186.3	183.2	2089	64.8
12/4/2020 11:48	188.5	186.2	183.3	2095	65.01
12/4/2020 11:48	188.6	186.5	183.5	2096	65.08

12/4/2020 11:48	188.7	185.8	183.4	2094	64.96
12/4/2020 11:48	188.7	186.3	183.6	2095	64.88
12/4/2020 11:48	188.9	186	183.4	2095	64.96
12/4/2020 11:48	188.9	185.6	183.4	2095	64.92
12/4/2020 11:48	189	186.2	183.8	2095	64.96
12/4/2020 11:48	189.1	186.2	183.6	2093	64.99
12/4/2020 11:48	189.2	186.3	183.7	2093	64.92
12/4/2020 11:48	189.4	186.3	183.6	2094	65.08
12/4/2020 11:48	189.4	185.6	183.4	2093	64.99
12/4/2020 11:48	189.6	186.4	183.6	2093	64.92
12/4/2020 11:48	189.7	186.2	183.8	2098	65.04
12/4/2020 11:48	189.8	185.6	183.6	2094	64.96
12/4/2020 11:48	190	186.3	183.8	2091	64.92
12/4/2020 11:48	190	186	183.5	2099	65
12/4/2020 11:48	190.1	187	183.5	2095	64.92
12/4/2020 11:48	190.2	187.4	183.8	2095	64.92
12/4/2020 11:48	190.3	187.1	183.7	2099	64.96
12/4/2020 11:48	190.5	187.1	183.7	2102	65
12/4/2020 11:48	190.5	187	183.9	2099	64.92
12/4/2020 11:48	190.7	186.8	183.6	2107	65.04
12/4/2020 11:48	190.7	186.9	183.8	2110	64.99
12/4/2020 11:48	190.8	187.4	183.5	2109	64.92
12/4/2020 11:48	190.8	187.2	183.5	2109	65
12/4/2020 11:48	190.8	187.1	183.7	2108	64.88
12/4/2020 11:48	190.9	186.8	183.4	2104	64.99
12/4/2020 11:48	190.9	187.3	183.4	2102	65
12/4/2020 11:48	191	186.8	184.2	2101	64.92
12/4/2020 11:48	191	187.2	184.6	2103	64.99
12/4/2020 11:48	191.1	187.5	184.4	2108	65.08
12/4/2020 11:48	191.1	187.2	183.9	2103	64.88
12/4/2020 11:48	191.1	187.4	183.5	2102	64.88
12/4/2020 11:48	191.1	186.9	183.9	2101	64.96
12/4/2020 11:48	191.1	187	183.9	2102	64.92
12/4/2020 11:48	191.1	187.2	183.8	2103	64.88
12/4/2020 11:48	191.1	187	184	2105	65.04
12/4/2020 11:48	191.1	187.3	183.8	2101	64.88
12/4/2020 11:48	191.1	186.8	184.2	2105	64.92
12/4/2020 11:48	191.2	187.3	184.6	2104	65.04
12/4/2020 11:48	191.2	187.2	184.1	2106	64.88
12/4/2020 11:48	191.1	187.2	183.8	2108	64.88
12/4/2020 11:48	191.1	187.2	183.8	2110	64.96
12/4/2020 11:48	191.1	186.9	183.7	2107	64.87
12/4/2020 11:48	191	187	183.9	2109	64.92
12/4/2020 11:48	191	187.3	183.8	2110	65.12
12/4/2020 11:48	191	187	183.4	2111	64.99
12/4/2020 11:48	191	187.3	184.4	2110	65.03
12/4/2020 11:48	191	187	184.6	2111	64.96

12/4/2020 11:48	191	187	184.1	2110	64.88
12/4/2020 11:48	190.9	186.8	183.5	2116	64.92
12/4/2020 11:48	190.9	186.9	183.8	2118	65.05
12/4/2020 11:48	190.8	187.2	184.1	2120	64.96
12/4/2020 11:48	190.9	187.4	184.1	2116	64.92
12/4/2020 11:48	190.7	187.5	183.8	2120	65.04
12/4/2020 11:48	190.7	187.2	183.8	2120	64.96
12/4/2020 11:48	190.6	186.9	183.9	2116	64.96
12/4/2020 11:48	190.6	187.1	184.4	2118	65.04
12/4/2020 11:48	190.6	187	184.8	2117	65.04
12/4/2020 11:49	190.5	187.2	184.1	2120	64.91
12/4/2020 11:49	190.5	187.4	183.8	2121	65
12/4/2020 11:49	190.5	187.2	184.1	2123	64.96
12/4/2020 11:49	190.3	187.3	184	2123	64.88
12/4/2020 11:49	190.3	186.8	184.2	2123	65.04
12/4/2020 11:49	190.3	186.9	184.2	2120	64.88
12/4/2020 11:49	190.1	187.3	183.7	2120	64.92
12/4/2020 11:49	190.1	187.1	183.7	2126	65.04
12/4/2020 11:49	190	186.9	183.7	2122	64.88
12/4/2020 11:49	189.9	187.4	184.1	2119	64.99
12/4/2020 11:49	190	186.9	184.7	2117	65.08
12/4/2020 11:49	189.8	187.1	184.6	2120	64.96
12/4/2020 11:49	189.8	186.9	184.5	2120	64.91
12/4/2020 11:49	189.8	186.9	184	2120	65.08
12/4/2020 11:49	189.7	187.4	183.8	2121	64.99
12/4/2020 11:49	189.7	186.8	184	2120	65.08
12/4/2020 11:49	189.7	187.4	184.1	2117	65.04
12/4/2020 11:49	189.6	187	183.7	2113	64.95
12/4/2020 11:49	189.6	187	183.9	2110	65.04
12/4/2020 11:49	189.6	187.4	184	2113	64.96
12/4/2020 11:49	189.6	187.1	183.7	2113	64.84
12/4/2020 11:49	189.6	187.3	183.5	2113	64.88
12/4/2020 11:49	189.6	187	183.5	2113	65.08
12/4/2020 11:49	189.5	186.9	183.8	2113	65.08
12/4/2020 11:49	189.5	187.5	184.2	2112	65.12
12/4/2020 11:49	189.5	187	184.5	2113	65.08
12/4/2020 11:49	189.5	187.4	184.8	2110	64.88
12/4/2020 11:49	189.5	186.9	184.6	2113	65.08
12/4/2020 11:49	189.5	187	184.6	2109	64.88
12/4/2020 11:49	189.4	187.3	184.8	2112	65.01
12/4/2020 11:49	189.4	187.1	184.8	2115	64.88
12/4/2020 11:49	189.4	187.4	184.8	2113	64.96
12/4/2020 11:49	189.4	187	184.8	2110	64.96
12/4/2020 11:49	189.4	187	184.7	2109	65.09
12/4/2020 11:49	189.3	187.3	184.6	2113	65.12
12/4/2020 11:49	189.3	187	184.8	2112	64.92
12/4/2020 11:49	189.3	186.9	184.4	2108	65

12/4/2020 11:49	189.3	187	184.6	2110	64.96
12/4/2020 11:49	189.3	186.9	184.3	2110	65.04
12/4/2020 11:49	189.2	187	183.8	2109	64.96
12/4/2020 11:49	189.2	186.9	183.9	2111	64.87
12/4/2020 11:49	189.2	187.3	183.4	2114	64.87
12/4/2020 11:49	189.2	187.4	183.4	2116	65
12/4/2020 11:49	189.1	186.7	183.7	2116	65
12/4/2020 11:49	189.1	187.1	183.5	2117	64.96
12/4/2020 11:49	189.1	187.2	183.5	2114	65.04
12/4/2020 11:49	189.1	187	183.8	2113	65
12/4/2020 11:49	189	187.4	183.7	2113	64.96
12/4/2020 11:49	189	187	184	2115	64.88
12/4/2020 11:49	189	187.1	183.8	2115	65.04
12/4/2020 11:49	189	186.7	184.1	2118	64.88
12/4/2020 11:49	188.9	187.2	184	2115	64.88
12/4/2020 11:49	188.9	187.4	183.8	2113	64.96
12/4/2020 11:49	188.9	187.2	183.7	2112	65.04
12/4/2020 11:49	189	187.3	183.9	2112	65.04
12/4/2020 11:49	188.9	187.3	183.9	2114	65.12
12/4/2020 11:49	188.9	187	183.9	2117	64.88
12/4/2020 11:49	188.8	187	184	2117	64.88
12/4/2020 11:49	188.8	187	183.9	2118	65
12/4/2020 11:49	188.8	187.3	183.9	2116	65.07
12/4/2020 11:50	188.7	187.3	183.8	2113	64.84
12/4/2020 11:50	188.7	186.9	183.8	2113	64.92
12/4/2020 11:50	188.7	187.4	184.1	2113	64.88
12/4/2020 11:50	188.7	187.1	183.8	2109	64.84
12/4/2020 11:50	188.7	187.1	183.9	2113	65
12/4/2020 11:50	188.6	187	184	2111	64.95
12/4/2020 11:50	188.5	187	183.9	2115	65.03
12/4/2020 11:50	188.5	187.4	183.9	2116	64.92
12/4/2020 11:50	188.5	187.2	184.1	2113	65.03
12/4/2020 11:50	188.5	187	183.8	2109	64.84
12/4/2020 11:50	188.4	187	184.1	2109	64.92
12/4/2020 11:50	188.4	186.9	183.9	2105	64.99
12/4/2020 11:50	188.3	187.1	183.5	2105	64.99
12/4/2020 11:50	188.4	186.9	183.9	2109	64.96
12/4/2020 11:50	188.3	187.5	183.5	2109	64.99
12/4/2020 11:50	188.3	187.2	183.4	2107	64.88
12/4/2020 11:50	188.3	186.8	183.7	2111	65.09
12/4/2020 11:50	188.3	187.2	183.4	2105	64.92
12/4/2020 11:50	188.3	186.9	183.7	2105	64.96
12/4/2020 11:50	188.2	187	183.7	2102	64.96
12/4/2020 11:50	188.3	187.3	183.4	2100	64.87
12/4/2020 11:50	188.3	187	183.5	2102	64.92
12/4/2020 11:50	188.2	187	183.6	2099	65
12/4/2020 11:50	188.2	187.2	183.3	2105	64.96

12/4/2020 11:50	188.2	187	183.7	2106	64.96
12/4/2020 11:50	188.3	187.2	183.6	2109	65.05
12/4/2020 11:50	188.2	187	183.7	2106	64.92
12/4/2020 11:50	188.3	186.9	183.7	2109	64.96
12/4/2020 11:50	188.3	187.3	183.4	2108	65.04
12/4/2020 11:50	188.3	186.8	183.5	2105	64.88
12/4/2020 11:50	188.3	187.2	183.8	2099	65.04
12/4/2020 11:50	188.3	186.8	183.6	2101	64.92
12/4/2020 11:50	188.3	187.3	183.8	2102	64.95
12/4/2020 11:50	188.3	187.3	183.7	2102	64.99
12/4/2020 11:50	188.4	186.9	183.5	2103	65.08
12/4/2020 11:50	188.5	187.4	183.9	2102	65.04
12/4/2020 11:50	188.4	187	183.5	2095	65.04
12/4/2020 11:50	188.5	186.8	183.5	2096	65.01
12/4/2020 11:50	188.6	187.4	183.8	2094	64.83
12/4/2020 11:50	188.5	187	183.5	2095	64.92
12/4/2020 11:50	188.7	187.2	183.8	2098	65.12
12/4/2020 11:50	188.6	186.8	183.5	2095	64.84
12/4/2020 11:50	188.7	186.7	183.2	2093	64.88
12/4/2020 11:50	188.8	187.4	183.7	2097	65.08
12/4/2020 11:50	188.9	186.9	183.5	2093	64.84
12/4/2020 11:50	188.9	187.4	183.9	2094	64.92
12/4/2020 11:50	188.9	187.3	184.4	2097	64.96
12/4/2020 11:50	189.1	187.2	184.4	2097	64.92
12/4/2020 11:50	189.2	187.3	184.7	2098	64.96
12/4/2020 11:50	189.2	187.3	184.8	2097	65.05
12/4/2020 11:50	189.2	187.4	184.2	2091	65.04
12/4/2020 11:50	189.4	187.4	183.8	2089	64.96
12/4/2020 11:50	189.4	186.9	183.7	2091	64.96
12/4/2020 11:50	189.7	187.2	183.6	2091	64.92
12/4/2020 11:50	189.8	187	184.1	2091	64.96
12/4/2020 11:50	189.9	187	184.2	2091	64.92
12/4/2020 11:50	190	187.2	184.1	2091	64.96
12/4/2020 11:50	190.1	187.1	184	2093	64.96
12/4/2020 11:50	190.3	187.4	183.8	2100	65.09
12/4/2020 11:51	190.5	187	183.7	2096	64.92
12/4/2020 11:51	190.5	186.8	184	2095	65
12/4/2020 11:51	190.6	187.5	183.8	2098	65
12/4/2020 11:51	190.7	187	184.7	2100	64.99
12/4/2020 11:51	190.9	187.4	184.6	2096	65
12/4/2020 11:51	190.9	186.8	184.2	2097	64.96
12/4/2020 11:51	191.1	187	184.1	2099	64.92
12/4/2020 11:51	191.2	187.3	184.1	2102	65.08
12/4/2020 11:51	191.3	187	184.4	2099	64.92
12/4/2020 11:51	191.5	187.4	184	2104	65.04
12/4/2020 11:51	191.5	187.2	184	2103	65.16
12/4/2020 11:51	191.6	187.1	184.2	2109	65.16

12/4/2020 11:51	191.7	187.1	184.1	2109	65
12/4/2020 11:51	191.8	187	184	2109	65.12
12/4/2020 11:51	191.9	187	184	2109	65.04
12/4/2020 11:51	192	187.2	184	2110	65.08
12/4/2020 11:51	192.2	186.8	183.6	2111	64.92
12/4/2020 11:51	192.2	187.2	184.1	2106	64.96
12/4/2020 11:51	192.2	186.9	183.9	2103	65
12/4/2020 11:51	192.2	186.9	184.1	2103	65
12/4/2020 11:51	192.3	187.2	184.5	2104	64.96
12/4/2020 11:51	192.4	186.8	184.2	2099	65
12/4/2020 11:51	192.4	187.4	184.5	2104	64.92
12/4/2020 11:51	192.4	188.2	184.5	2102	65.04
12/4/2020 11:51	192.4	188.1	184.1	2102	65.04
12/4/2020 11:51	192.4	188	184.2	2107	65.08
12/4/2020 11:51	192.5	188.1	184.9	2110	64.96
12/4/2020 11:51	192.6	188.2	184.9	2112	65.12
12/4/2020 11:51	192.6	188.1	184.2	2103	65
12/4/2020 11:51	192.6	188	184	2108	64.88
12/4/2020 11:51	192.6	188.3	184.6	2111	64.96
12/4/2020 11:51	192.7	188.2	184.7	2111	65.04
12/4/2020 11:51	192.7	188.1	184.1	2117	64.88
12/4/2020 11:51	192.7	188.1	184.6	2119	65.16
12/4/2020 11:51	192.8	188.2	185	2116	64.92
12/4/2020 11:51	192.8	188	184.1	2113	65
12/4/2020 11:51	192.8	188.1	184.2	2112	65.04
12/4/2020 11:51	192.9	188.1	184.6	2112	65.04
12/4/2020 11:51	192.9	188.1	184.5	2110	65
12/4/2020 11:51	192.9	188.1	184.4	2112	64.96
12/4/2020 11:51	192.9	188.1	184.4	2112	64.96
12/4/2020 11:51	192.9	188.2	185.2	2109	64.92
12/4/2020 11:51	192.8	188.1	184.8	2111	65.12
12/4/2020 11:51	192.9	188.1	184.1	2109	65
12/4/2020 11:51	192.9	188.1	184.2	2112	64.96
12/4/2020 11:51	192.9	188.3	184.4	2116	65.12
12/4/2020 11:51	192.9	188.2	184.2	2114	64.92
12/4/2020 11:51	192.9	188.1	184.6	2113	64.99
12/4/2020 11:51	192.9	188	184.6	2112	65.08
12/4/2020 11:51	192.9	188.2	185.4	2113	64.88
12/4/2020 11:51	192.9	188.1	185.4	2113	65.08
12/4/2020 11:51	192.9	188.3	184.5	2114	65
12/4/2020 11:51	192.9	188	184.2	2116	64.96
12/4/2020 11:51	192.9	188.1	184.4	2117	65
12/4/2020 11:51	192.8	188	184.4	2114	64.99
12/4/2020 11:51	192.8	188.1	184.8	2117	65.08
12/4/2020 11:51	192.7	188.1	184.6	2113	64.88
12/4/2020 11:51	192.8	188.1	184.6	2115	64.96
12/4/2020 11:51	192.8	188.1	184.5	2118	64.96

12/4/2020 11:52	192.8	188.1	184.5	2116	65.04
12/4/2020 11:52	192.7	188.2	184.8	2116	65.04
12/4/2020 11:52	192.7	188.2	185.3	2112	65
12/4/2020 11:52	192.6	188.1	185.2	2115	65.05
12/4/2020 11:52	192.6	188.2	184.6	2120	65
12/4/2020 11:52	192.6	188.2	184.4	2123	65.04
12/4/2020 11:52	192.5	188.2	184.1	2118	65.04
12/4/2020 11:52	192.6	188.1	184.4	2116	64.88
12/4/2020 11:52	192.5	188.2	184.6	2116	65
12/4/2020 11:52	192.5	188.1	184.6	2120	64.92
12/4/2020 11:52	192.5	188.2	184.6	2123	64.99
12/4/2020 11:52	192.4	188.1	184.4	2120	64.88
12/4/2020 11:52	192.4	188.1	184.8	2123	65.08
12/4/2020 11:52	192.3	188.2	184.6	2124	64.92
12/4/2020 11:52	192.4	188.1	184.6	2127	65.01
12/4/2020 11:52	192.3	188.1	184.7	2127	64.92
12/4/2020 11:52	192.3	188.3	184.5	2125	65.08
12/4/2020 11:52	192.3	188.1	184.5	2122	64.88
12/4/2020 11:52	192.3	188.1	184.9	2120	65
12/4/2020 11:52	192.3	188.2	184.8	2117	64.92
12/4/2020 11:52	192.2	188.2	184.9	2118	64.92
12/4/2020 11:52	192.1	188.1	184.8	2116	64.96
12/4/2020 11:52	192.1	188.2	184.6	2118	65.04
12/4/2020 11:52	192.1	188.1	184.6	2122	65
12/4/2020 11:52	192	188.2	185.4	2123	65
12/4/2020 11:52	192	188.1	184.6	2122	64.92
12/4/2020 11:52	192	188.1	184.7	2122	65.09
12/4/2020 11:52	192	188.1	184.8	2124	65
12/4/2020 11:52	192	188.1	184.4	2123	64.96
12/4/2020 11:52	191.9	188.2	184.6	2122	65.08
12/4/2020 11:52	191.9	188.2	184.3	2121	65
12/4/2020 11:52	191.9	188.1	184.9	2122	64.96
12/4/2020 11:52	191.8	188.1	185.5	2122	64.92
12/4/2020 11:52	191.8	188.2	185.1	2120	65.04
12/4/2020 11:52	191.8	188.1	184.6	2121	65.04
12/4/2020 11:52	191.8	188.1	184.9	2127	64.96
12/4/2020 11:52	191.6	188	184.6	2125	64.99
12/4/2020 11:52	191.7	188.1	184.9	2123	64.88
12/4/2020 11:52	191.7	188	184.4	2120	64.88
12/4/2020 11:52	191.6	188.2	184.2	2122	65.08
12/4/2020 11:52	191.6	188.1	185.1	2118	65.01
12/4/2020 11:52	191.6	188.1	185.5	2116	65.08
12/4/2020 11:52	191.6	188.2	184.9	2117	64.91
12/4/2020 11:52	191.5	188.1	184.7	2123	64.92
12/4/2020 11:52	191.4	188	184.5	2124	64.92
12/4/2020 11:52	191.5	188.1	184.7	2122	65
12/4/2020 11:52	191.4	188.1	184.6	2123	64.96

12/4/2020 11:52	191.4	188.1	185	2122	65.09
12/4/2020 11:52	191.4	188	184.9	2125	65
12/4/2020 11:52	191.3	188.1	184.8	2124	65
12/4/2020 11:52	191.3	188	184.5	2122	64.96
12/4/2020 11:52	191.3	188.2	184.6	2127	65
12/4/2020 11:52	191.3	188.1	184.2	2126	64.96
12/4/2020 11:52	191.1	188.1	184.6	2122	65.08
12/4/2020 11:52	191.2	187.9	184.7	2122	65.04
12/4/2020 11:52	191.1	188.1	184.3	2123	64.88
12/4/2020 11:52	191.1	188.2	184.6	2123	65.01
12/4/2020 11:52	191	188.1	184.4	2120	65.04
12/4/2020 11:52	191	188.1	184.6	2117	65.12
12/4/2020 11:52	191	188.1	184.9	2120	64.96
12/4/2020 11:53	191	188.1	184.7	2117	65.08
12/4/2020 11:53	190.9	188.2	184.6	2121	65.12
12/4/2020 11:53	190.9	188.1	184.8	2122	65.04
12/4/2020 11:53	190.9	188.2	184.6	2120	64.88
12/4/2020 11:53	190.8	188.1	185.1	2123	64.96
12/4/2020 11:53	190.8	188.2	185.8	2124	64.96
12/4/2020 11:53	190.8	188.2	184.6	2124	64.92
12/4/2020 11:53	190.7	187.9	184.4	2124	64.96
12/4/2020 11:53	190.7	188.1	184.6	2127	65
12/4/2020 11:53	190.7	188.2	184.6	2127	64.96
12/4/2020 11:53	190.6	188.1	184.8	2127	65.09
12/4/2020 11:53	190.5	188	184.6	2127	65.04
12/4/2020 11:53	190.5	188.2	185.6	2124	64.96
12/4/2020 11:53	190.5	188.2	184.9	2120	65.08
12/4/2020 11:53	190.5	188.1	184.4	2121	65.04
12/4/2020 11:53	190.3	188.1	184.8	2129	64.96
12/4/2020 11:53	190.5	187.9	185.1	2127	64.99
12/4/2020 11:53	190.4	188.1	184.6	2125	65.04
12/4/2020 11:53	190.3	188.2	184.6	2127	65.03
12/4/2020 11:53	190.3	188.1	185.5	2123	64.97
12/4/2020 11:53	190.2	188.2	184.6	2124	65.04
12/4/2020 11:53	190.2	188.1	184.6	2124	65.04
12/4/2020 11:53	190.2	188.1	184.8	2125	65.01
12/4/2020 11:53	190.1	188.2	184.7	2124	65
12/4/2020 11:53	190.1	188.1	184.9	2128	65.08
12/4/2020 11:53	190.1	188.1	184.7	2127	65.08
12/4/2020 11:53	190	188.1	185.3	2126	65.08
12/4/2020 11:53	189.9	188.2	185.1	2121	64.99
12/4/2020 11:53	189.9	188.1	184.2	2127	65
12/4/2020 11:53	189.9	188.2	184.6	2124	65
12/4/2020 11:53	189.8	188	184.6	2127	64.91
12/4/2020 11:53	189.9	188.2	184.8	2124	65.04
12/4/2020 11:53	189.8	188.2	184.6	2124	64.96
12/4/2020 11:53	189.7	188.1	184.8	2116	65

12/4/2020 11:53	189.7	188.1	185.4	2119	65
12/4/2020 11:53	189.7	188.1	184.9	2117	64.96
12/4/2020 11:53	189.6	188.2	184.2	2120	64.87
12/4/2020 11:53	189.6	188.1	184.4	2117	65.05
12/4/2020 11:53	189.6	188.2	184.8	2121	64.88
12/4/2020 11:53	189.5	188.1	184.8	2123	64.92
12/4/2020 11:53	189.4	188.1	184.9	2126	64.96
12/4/2020 11:53	189.4	188.1	184.8	2127	64.88
12/4/2020 11:53	189.4	188	184.4	2122	64.91
12/4/2020 11:53	189.4	188.1	185	2123	64.88
12/4/2020 11:53	189.3	188.1	185.1	2119	64.92
12/4/2020 11:53	189.2	188.2	185.4	2119	64.99
12/4/2020 11:53	189.2	188.2	185.2	2117	65.08
12/4/2020 11:53	189.2	188.2	184.4	2120	65
12/4/2020 11:53	189.1	188.1	184.4	2122	64.99
12/4/2020 11:53	189.1	188	184.5	2120	64.97
12/4/2020 11:53	189.1	188.1	184.5	2116	64.88
12/4/2020 11:53	189.1	188	184.8	2113	65.04
12/4/2020 11:53	189	188.1	184.9	2119	65.08
12/4/2020 11:53	189	188	184.6	2115	64.8
12/4/2020 11:53	188.9	188.1	184.6	2116	64.88
12/4/2020 11:53	188.9	188.1	184.2	2120	64.88
12/4/2020 11:53	188.8	188.2	184.2	2120	64.88
12/4/2020 11:53	188.9	188.1	184.6	2120	64.96
12/4/2020 11:53	188.8	188	184.6	2117	64.88
12/4/2020 11:54	188.7	188.1	185.2	2116	64.92
12/4/2020 11:54	188.8	188.1	185.5	2120	65.03
12/4/2020 11:54	188.7	188.1	185.3	2119	64.8
12/4/2020 11:54	188.7	188.1	185.5	2123	64.92
12/4/2020 11:54	188.6	188.1	185.3	2126	65.08
12/4/2020 11:54	188.6	188.2	184.9	2125	64.99
12/4/2020 11:54	188.6	188.2	184.8	2124	64.99
12/4/2020 11:54	188.5	188.2	184.7	2124	64.92
12/4/2020 11:54	188.5	188.2	184.5	2127	64.99
12/4/2020 11:54	188.5	188.1	184.6	2121	65.08
12/4/2020 11:54	188.3	188.1	184	2122	65.08
12/4/2020 11:54	188.4	188.2	184.4	2116	64.99
12/4/2020 11:54	188.3	188.1	184.3	2120	65.04
12/4/2020 11:54	188.3	188.2	184.2	2120	65.04
12/4/2020 11:54	188.2	188.2	184.5	2120	64.83
12/4/2020 11:54	188.3	188.2	184.5	2117	64.88
12/4/2020 11:54	188.2	188.2	184.4	2123	64.96
12/4/2020 11:54	188.2	188.2	184.7	2116	64.92
12/4/2020 11:54	188.2	188.1	184.7	2116	64.96
12/4/2020 11:54	188.1	188.1	185.4	2117	65.08
12/4/2020 11:54	188	188.2	185.4	2116	64.91
12/4/2020 11:54	188	188.1	185.1	2118	65

12/4/2020 11:54	188	188.1	185.4	2118	64.99
12/4/2020 11:54	187.9	187.9	184.6	2120	64.96
12/4/2020 11:54	187.9	188.1	184.4	2121	64.95
12/4/2020 11:54	187.8	188.2	184.4	2126	64.96
12/4/2020 11:54	187.9	188.2	184.5	2124	64.92
12/4/2020 11:54	187.8	188.1	184.4	2124	64.96
12/4/2020 11:54	187.9	188.1	184.2	2126	65
12/4/2020 11:54	187.7	188	184.4	2123	64.96
12/4/2020 11:54	187.7	188.1	184.4	2122	64.96
12/4/2020 11:54	187.7	188.2	184.3	2124	65
12/4/2020 11:54	187.7	188.2	184.2	2123	64.96
12/4/2020 11:54	187.6	188.1	185.1	2127	64.96
12/4/2020 11:54	187.6	188.1	185	2122	64.92
12/4/2020 11:54	187.6	188.1	184.7	2120	64.96
12/4/2020 11:54	187.6	188.1	184.3	2120	64.96
12/4/2020 11:54	187.5	188.2	184.5	2122	65.05
12/4/2020 11:54	187.5	188.2	184.6	2121	64.92
12/4/2020 11:54	187.5	188.1	184.6	2127	65
12/4/2020 11:54	187.5	188.2	184	2127	64.96
12/4/2020 11:54	187.5	188.2	184.3	2123	64.83
12/4/2020 11:54	187.4	188.1	185.2	2120	64.92
12/4/2020 11:54	187.4	188.1	185.1	2120	65
12/4/2020 11:54	187.4	188.1	184.4	2119	64.88
12/4/2020 11:54	187.4	188.1	184.2	2118	64.88
12/4/2020 11:54	187.3	188.1	184.4	2114	64.92
12/4/2020 11:54	187.4	188.1	184.8	2115	64.92
12/4/2020 11:54	187.4	188	184.4	2113	64.83
12/4/2020 11:54	187.3	188.1	184.7	2118	65.09
12/4/2020 11:54	187.4	188.1	184.4	2113	64.92
12/4/2020 11:54	187.3	188.1	184.1	2114	64.92
12/4/2020 11:54	187.3	188.1	184	2112	64.88
12/4/2020 11:54	187.3	188.2	184	2112	64.92
12/4/2020 11:54	187.3	188.1	184.1	2114	64.88
12/4/2020 11:54	187.3	188.2	184.4	2118	64.96
12/4/2020 11:54	187.3	188.2	184.2	2118	64.88
12/4/2020 11:54	187.4	188.2	184.2	2122	64.84
12/4/2020 11:54	187.5	188.2	184.4	2120	65.12
12/4/2020 11:54	187.4	188.1	184.1	2120	65.03
12/4/2020 11:55	187.4	188.1	184.2	2116	65.12
12/4/2020 11:55	187.4	188.1	184.3	2114	65.12
12/4/2020 11:55	187.4	188.1	184.2	2112	64.99
12/4/2020 11:55	187.5	188.1	184.6	2114	64.88
12/4/2020 11:55	187.4	188.2	184.6	2116	64.99
12/4/2020 11:55	187.5	188.1	184.3	2116	65.04
12/4/2020 11:55	187.5	188.1	184.6	2113	64.99
12/4/2020 11:55	187.5	188	185.3	2113	64.96
12/4/2020 11:55	187.6	188.2	184.6	2114	65.04

12/4/2020 11:55	187.6	188.2	184.2	2113	65.12
12/4/2020 11:55	187.6	188.1	184.4	2116	64.99
12/4/2020 11:55	187.6	188	184.6	2122	64.99
12/4/2020 11:55	187.7	188.2	183.9	2120	64.92
12/4/2020 11:55	187.7	188.1	185.2	2120	64.96
12/4/2020 11:55	187.7	188.1	185.1	2121	65
12/4/2020 11:55	187.7	188.1	184.1	2122	64.96
12/4/2020 11:55	187.7	188.2	184.2	2121	65
12/4/2020 11:55	187.8	188.1	184.6	2119	65
12/4/2020 11:55	187.8	188.2	184.4	2120	64.96
12/4/2020 11:55	187.8	188.1	184.2	2125	65
12/4/2020 11:55	187.8	188.1	184.4	2123	64.92
12/4/2020 11:55	187.8	188.1	185.4	2123	64.91
12/4/2020 11:55	187.8	188.1	184.9	2123	64.96
12/4/2020 11:55	187.8	188.2	184.1	2121	64.96
12/4/2020 11:55	188	188.1	184.4	2124	64.96
12/4/2020 11:55	188	188.1	184.6	2126	64.92
12/4/2020 11:55	188	188.2	184.4	2122	64.96
12/4/2020 11:55	188	188.1	184.4	2119	64.92
12/4/2020 11:55	188	188.1	184.4	2117	64.96
12/4/2020 11:55	188.1	188.1	184.9	2121	64.92
12/4/2020 11:55	188.1	188	185.3	2124	64.92
12/4/2020 11:55	188.1	188.3	184.6	2122	64.91
12/4/2020 11:55	188.2	188.2	184.2	2116	64.96
12/4/2020 11:55	188.2	188.2	184.4	2119	64.92
12/4/2020 11:55	188.2	188	184.6	2120	64.91
12/4/2020 11:55	188.2	187.9	184.6	2121	64.92
12/4/2020 11:55	188.3	188.1	184.6	2119	64.96
12/4/2020 11:55	188.3	188.2	184.4	2118	64.99
12/4/2020 11:55	188.3	188.1	184.5	2118	64.92
12/4/2020 11:55	188.3	188.1	184.4	2120	64.87
12/4/2020 11:55	188.4	188.2	184.1	2127	64.87
12/4/2020 11:55	188.5	188.1	184.2	2126	65.04
12/4/2020 11:55	188.5	188.1	184.4	2122	64.96
12/4/2020 11:55	188.5	188.1	184.4	2118	64.92
12/4/2020 11:55	188.5	188.2	184.4	2123	64.96
12/4/2020 11:55	188.6	188.1	184.4	2120	64.88
12/4/2020 11:55	188.5	188.2	184.4	2121	64.96
12/4/2020 11:55	188.6	188.1	184.6	2120	65.04
12/4/2020 11:55	188.6	188.1	184.5	2118	64.92
12/4/2020 11:55	188.6	188.1	184.6	2119	64.83
12/4/2020 11:55	188.7	188.2	184.4	2120	64.96
12/4/2020 11:55	188.7	188	184.2	2123	64.99
12/4/2020 11:55	188.7	188.2	184.4	2123	64.99
12/4/2020 11:55	188.8	188.1	184.3	2122	65.01
12/4/2020 11:55	188.7	188.2	185.3	2122	65.03
12/4/2020 11:55	188.8	188	185.3	2123	64.96

12/4/2020 11:55	188.8	188.2	184.6	2124	65.08
12/4/2020 11:55	188.7	188	184.8	2127	64.99
12/4/2020 11:55	188.9	188.1	184.6	2129	64.88
12/4/2020 11:56	188.9	188.2	184.6	2127	65.01
12/4/2020 11:56	188.9	188.2	184.4	2129	65
12/4/2020 11:56	189	188.2	184.2	2127	65
12/4/2020 11:56	189	188.1	185.3	2127	64.96
12/4/2020 11:56	189	188.1	185.6	2127	65.04
12/4/2020 11:56	189	188.1	184.5	2127	64.96
12/4/2020 11:56	189	188.2	184.3	2131	65.09
12/4/2020 11:56	189.1	188.2	184.8	2126	64.96
12/4/2020 11:56	189	188.1	184.6	2125	65
12/4/2020 11:56	189.1	188.2	184.8	2126	65.04
12/4/2020 11:56	189.1	188	184.5	2127	65.04
12/4/2020 11:56	189.1	188.1	184.1	2130	64.96
12/4/2020 11:56	189.2	188.1	184.2	2131	64.88
12/4/2020 11:56	189.2	188.1	184.9	2130	65.08
12/4/2020 11:56	189.2	188.1	185.5	2128	65.04
12/4/2020 11:56	189.3	188.2	184.9	2130	64.99
12/4/2020 11:56	189.3	188.1	184.5	2131	65.12
12/4/2020 11:56	189.3	188.1	184.6	2133	65.08
12/4/2020 11:56	189.3	188.1	184.6	2133	65.08
12/4/2020 11:56	189.3	188.1	184.4	2131	64.96
12/4/2020 11:56	189.3	188.2	184.4	2131	65.12
12/4/2020 11:56	189.3	188.2	184.4	2131	64.96
12/4/2020 11:56	189.4	188.2	185.4	2131	65.12
12/4/2020 11:56	189.3	188	185.4	2127	64.92
12/4/2020 11:56	189.3	188.2	184.3	2127	64.96
12/4/2020 11:56	189.3	187.9	184.4	2127	65
12/4/2020 11:56	189.3	188.1	184.8	2129	65
12/4/2020 11:56	189.3	188.1	184.6	2127	65.04
12/4/2020 11:56	189.3	188.2	184.6	2127	64.96
12/4/2020 11:56	189.3	188.2	184.4	2120	65
12/4/2020 11:56	189.3	188.2	184.3	2120	64.96
12/4/2020 11:56	189.2	188.1	185.2	2124	65
12/4/2020 11:56	189.2	188.1	185.3	2124	65.01
12/4/2020 11:56	189.2	188.1	184.9	2125	65.04
12/4/2020 11:56	189.2	188.2	184.5	2124	65.12
12/4/2020 11:56	189.3	188.1	184.8	2120	65.08
12/4/2020 11:56	189.2	188.1	184.9	2124	64.83
12/4/2020 11:56	189.2	188.1	184.8	2120	64.92
12/4/2020 11:56	189.2	188.1	184.6	2116	65.04
12/4/2020 11:56	189.2	188.1	184.4	2116	65.08
12/4/2020 11:56	189.3	188.1	184.4	2113	65.04
12/4/2020 11:56	189.3	188.2	184.2	2113	65.16
12/4/2020 11:56	189.4	188.2	184.7	2113	65.04
12/4/2020 11:56	189.4	188.2	185.3	2116	65.12

12/4/2020 11:56	189.4	188.1	185.5	2116	65
12/4/2020 11:56	189.4	188	185.5	2118	65.04
12/4/2020 11:56	189.4	188.1	185.4	2115	64.96
12/4/2020 11:56	189.5	188.1	185.1	2113	65.04
12/4/2020 11:56	189.5	188.1	184.7	2114	64.96
12/4/2020 11:56	189.6	188.2	184.7	2111	65
12/4/2020 11:56	189.6	188.1	184.7	2113	65.04
12/4/2020 11:56	189.6	188.1	184.7	2116	65
12/4/2020 11:56	189.7	188.1	184.5	2117	65
12/4/2020 11:56	189.8	188.2	184.8	2116	64.96
12/4/2020 11:56	189.8	188.1	184.6	2113	64.88
12/4/2020 11:56	189.8	188.1	184.7	2121	64.96
12/4/2020 11:56	189.9	188.1	184.8	2117	65.04
12/4/2020 11:56	189.9	188.1	184.7	2114	64.88
12/4/2020 11:56	190	188.1	184.9	2116	65.12
12/4/2020 11:57	190	188.1	184.9	2116	64.93
12/4/2020 11:57	190	188.2	184.6	2120	64.92
12/4/2020 11:57	190.1	188.1	185	2119	64.92
12/4/2020 11:57	190.2	188.1	184.9	2121	64.96
12/4/2020 11:57	190.3	188.1	184.6	2118	65.08
12/4/2020 11:57	190.3	188.1	185.1	2123	65.12
12/4/2020 11:57	190.5	188.1	185.1	2123	65.08
12/4/2020 11:57	190.5	188.1	184.8	2124	64.92
12/4/2020 11:57	190.6	188.1	185.1	2120	65.12
12/4/2020 11:57	190.7	188.2	184.9	2118	64.92
12/4/2020 11:57	190.7	188.2	185.1	2120	65.04
12/4/2020 11:57	190.8	188.2	185.2	2120	64.92
12/4/2020 11:57	190.9	188.2	185.1	2116	64.96
12/4/2020 11:57	190.9	188.2	184.9	2109	64.92
12/4/2020 11:57	191.1	188.1	185.1	2108	65
12/4/2020 11:57	191.1	188.1	184.8	2112	64.96
12/4/2020 11:57	191.3	188.2	184.8	2109	64.96
12/4/2020 11:57	191.3	188.2	184.7	2111	65
12/4/2020 11:57	191.3	188.2	184.9	2109	65
12/4/2020 11:57	191.5	188.1	185	2118	65
12/4/2020 11:57	191.5	188.2	184.8	2113	64.92
12/4/2020 11:57	191.6	188.1	184.9	2114	65
12/4/2020 11:57	191.8	188.1	184.6	2116	65
12/4/2020 11:57	191.7	188.1	184.6	2117	65.01
12/4/2020 11:57	192	188.1	184.9	2114	64.92
12/4/2020 11:57	192	188.3	184.8	2116	65.04
12/4/2020 11:57	192.1	188.1	184.9	2118	64.88
12/4/2020 11:57	192.2	188.1	185.3	2120	65.04
12/4/2020 11:57	192.2	188.1	185.4	2121	64.93
12/4/2020 11:57	192.4	188.1	185.4	2121	65.08
12/4/2020 11:57	192.5	188.1	185.8	2123	64.96
12/4/2020 11:57	192.4	188.1	185.8	2122	64.96

12/4/2020 11:57	192.6	188.1	186	2122	65.12
12/4/2020 11:57	192.6	188.2	185.8	2130	64.88
12/4/2020 11:57	192.6	188.2	185.4	2128	65.08
12/4/2020 11:57	192.8	188.2	185.1	2131	65.12
12/4/2020 11:57	192.8	188.2	185.2	2134	64.88
12/4/2020 11:57	192.9	188.1	184.9	2130	65.04
12/4/2020 11:57	192.9	188.2	185.3	2127	64.92
12/4/2020 11:57	192.9	188	185.2	2128	64.91
12/4/2020 11:57	193	188.1	185.3	2131	65.04
12/4/2020 11:57	193.1	187.9	185.1	2131	65
12/4/2020 11:57	193	188.1	185.1	2131	65
12/4/2020 11:57	193.1	188.2	185.2	2134	64.96
12/4/2020 11:57	193	188.1	185.3	2134	65.05
12/4/2020 11:57	193.1	188.1	185.1	2134	65
12/4/2020 11:57	193.1	188.1	185.4	2134	65
12/4/2020 11:57	193	188.1	185.1	2137	65.04
12/4/2020 11:57	193.1	188.2	185.3	2133	64.88
12/4/2020 11:57	193.1	188.2	185.1	2133	65
12/4/2020 11:57	193	188.1	185	2131	65.08
12/4/2020 11:57	193.1	188	184.9	2131	64.92
12/4/2020 11:57	193	188.1	184.9	2131	64.96
12/4/2020 11:57	192.9	188.1	184.8	2133	65.08
12/4/2020 11:57	192.9	188.2	185	2134	65.03
12/4/2020 11:57	192.9	188.2	185.3	2136	64.99
12/4/2020 11:57	192.9	188.2	184.9	2139	65.12
12/4/2020 11:57	192.9	188.1	185.3	2138	64.99
12/4/2020 11:57	192.9	188.1	185.3	2134	64.92
12/4/2020 11:57	192.9	188.2	185.1	2130	64.92
12/4/2020 11:58	192.9	188.1	185.3	2133	64.96
12/4/2020 11:58	192.8	188.1	185.3	2135	65.04
12/4/2020 11:58	192.8	188	185.4	2132	65.12
12/4/2020 11:58	192.7	188.1	185.5	2136	65.08
12/4/2020 11:58	192.7	188.1	185.3	2134	65
12/4/2020 11:58	192.5	188.2	185.5	2136	65
12/4/2020 11:58	192.6	188.2	185.2	2133	64.92
12/4/2020 11:58	192.4	188.1	185.6	2133	64.96
12/4/2020 11:58	192.5	188	186.2	2131	65
12/4/2020 11:58	192.4	188.2	185.5	2134	65.09
12/4/2020 11:58	192.3	188.2	185.2	2137	65.04
12/4/2020 11:58	192.3	188.2	185.3	2138	65.04
12/4/2020 11:58	192.3	188	185.2	2142	65.08
12/4/2020 11:58	192.2	188.1	185.6	2140	65.04
12/4/2020 11:58	192.2	188.1	185.6	2142	64.96
12/4/2020 11:58	192.1	189.2	185.2	2138	65.01
12/4/2020 11:58	192.1	188.2	185.5	2138	65.04
12/4/2020 11:58	192	188.1	186.1	2134	65.12
12/4/2020 11:58	191.9	188.2	185.5	2133	64.92

12/4/2020 11:58	191.9	188.8	185.1	2135	65.08
12/4/2020 11:58	191.9	189.3	184.9	2131	64.92
12/4/2020 11:58	191.8	188.8	185.6	2131	64.96
12/4/2020 11:58	191.8	188.8	185.5	2130	64.88
12/4/2020 11:58	191.7	189.2	185.4	2133	64.96
12/4/2020 11:58	191.7	188.9	185.4	2136	65.08
12/4/2020 11:58	191.7	188.9	185.6	2134	64.96
12/4/2020 11:58	191.6	188.8	185.9	2131	64.92
12/4/2020 11:58	191.7	188.8	186.1	2134	65.04
12/4/2020 11:58	191.6	189.1	185.9	2129	64.96
12/4/2020 11:58	191.6	188.9	185.3	2131	64.92
12/4/2020 11:58	191.6	189.1	185.1	2127	65.04
12/4/2020 11:58	191.5	188.8	184.9	2131	64.92
12/4/2020 11:58	191.5	188.9	185.1	2137	64.96
12/4/2020 11:58	191.5	189.1	185.2	2137	65.09
12/4/2020 11:58	191.5	189.1	185.3	2135	65.04
12/4/2020 11:58	191.4	189.2	185.5	2135	64.99
12/4/2020 11:58	191.4	188.8	185.7	2137	65.05
12/4/2020 11:58	191.4	188.8	186.1	2133	64.99
12/4/2020 11:58	191.4	189.3	185.9	2131	65.04
12/4/2020 11:58	191.4	189.3	185.1	2133	64.92
12/4/2020 11:58	191.4	188.9	185.3	2131	64.99
12/4/2020 11:58	191.4	188.7	185.4	2130	65
12/4/2020 11:58	191.3	188.7	185.1	2130	65.01
12/4/2020 11:58	191.3	189.2	185.1	2133	65.04
12/4/2020 11:58	191.3	188.9	185.8	2132	64.99
12/4/2020 11:58	191.3	189.3	186	2134	65.09
12/4/2020 11:58	191.2	189.1	185.3	2133	64.88
12/4/2020 11:58	191.3	188.8	185.3	2132	64.88
12/4/2020 11:58	191.2	189.1	185.4	2133	65
12/4/2020 11:58	191.2	188.6	185.3	2133	64.92
12/4/2020 11:58	191.1	189.3	185.1	2131	64.96
12/4/2020 11:58	191.1	189.2	185.3	2127	65
12/4/2020 11:58	191.1	188.9	186.2	2127	65.04
12/4/2020 11:58	191.1	189.1	186	2125	65
12/4/2020 11:58	191.1	189.2	185.2	2127	65.04
12/4/2020 11:58	191.1	188.8	185.4	2127	65
12/4/2020 11:58	191	189.2	185.4	2127	65
12/4/2020 11:58	191	189.1	185.4	2131	65
12/4/2020 11:58	191	189.3	185.1	2123	64.91
12/4/2020 11:59	191	188.7	185.1	2127	65.04
12/4/2020 11:59	191	188.7	185.6	2129	64.92
12/4/2020 11:59	191	189.1	186	2126	64.88
12/4/2020 11:59	191	188.9	185.4	2124	64.96
12/4/2020 11:59	190.9	189.2	185.2	2123	64.88
12/4/2020 11:59	190.9	189.1	185.3	2125	64.88
12/4/2020 11:59	190.9	188.8	185.5	2127	64.99

12/4/2020 11:59	190.9	189.3	185.2	2131	65.12
12/4/2020 11:59	190.9	189	184.9	2129	64.84
12/4/2020 11:59	190.9	189.1	185.6	2127	65.04
12/4/2020 11:59	190.8	188.8	186	2124	64.84
12/4/2020 11:59	190.9	188.8	185.4	2127	64.95
12/4/2020 11:59	190.8	189.1	185.3	2125	64.8
12/4/2020 11:59	190.8	188.9	185.3	2127	65.01
12/4/2020 11:59	190.8	188.8	185.4	2123	64.92
12/4/2020 11:59	190.8	189.2	185.4	2122	64.99
12/4/2020 11:59	190.7	188.9	185.1	2124	65.08
12/4/2020 11:59	190.7	189.2	185.1	2125	64.88
12/4/2020 11:59	190.7	189.1	185.2	2120	64.96
12/4/2020 11:59	190.7	188.9	185.9	2117	64.88
12/4/2020 11:59	190.7	189.3	186.2	2120	64.91
12/4/2020 11:59	190.8	188.9	185.5	2116	64.96
12/4/2020 11:59	190.7	189	185.1	2116	64.92
12/4/2020 11:59	190.7	188.7	185.1	2109	64.99
12/4/2020 11:59	190.7	189	185.2	2114	64.99
12/4/2020 11:59	190.8	189.1	185.5	2113	65.12
12/4/2020 11:59	190.7	189.1	185.4	2113	64.96
12/4/2020 11:59	190.8	189.1	184.9	2113	64.92
12/4/2020 11:59	190.8	188.9	185.1	2111	64.92
12/4/2020 11:59	190.9	188.8	185.7	2110	64.79
12/4/2020 11:59	190.9	188.9	186.2	2109	64.92
12/4/2020 11:59	190.9	188.8	185.4	2112	64.92
12/4/2020 11:59	191	189.1	185	2112	64.92
12/4/2020 11:59	191	188.9	185.5	2109	64.96
12/4/2020 11:59	191	189.3	185.3	2108	64.88
12/4/2020 11:59	191.2	189.3	185.4	2107	64.88
12/4/2020 11:59	191.1	188.9	185.6	2107	65
12/4/2020 11:59	191.3	189.1	185.3	2109	65
12/4/2020 11:59	191.3	188.8	185.4	2108	65
12/4/2020 11:59	191.4	189.2	186	2107	64.96
12/4/2020 11:59	191.5	189.2	186.1	2109	64.92
12/4/2020 11:59	191.4	188.9	185.5	2109	64.96
12/4/2020 11:59	191.6	188.8	185.4	2106	64.96
12/4/2020 11:59	191.7	189.1	185.5	2107	64.87
12/4/2020 11:59	191.8	188.8	185.7	2108	64.92
12/4/2020 11:59	191.9	189.2	185.4	2105	64.92
12/4/2020 11:59	191.9	188.9	185.4	2109	64.92
12/4/2020 11:59	192.1	188.8	185.6	2104	64.96
12/4/2020 11:59	192.3	189	186.2	2104	65
12/4/2020 11:59	192.2	189.1	186	2104	64.91
12/4/2020 11:59	192.4	189.3	185.4	2106	64.96
12/4/2020 11:59	192.4	189	185.4	2109	64.96
12/4/2020 11:59	192.6	189.1	185.8	2110	64.92
12/4/2020 11:59	192.7	189.1	185.6	2107	65

12/4/2020 11:59	192.8	188.8	185.4	2110	64.96
12/4/2020 11:59	192.9	189.2	185.5	2113	64.87
12/4/2020 11:59	192.9	188.7	186.1	2116	65
12/4/2020 11:59	193	188.8	186.1	2112	64.92
12/4/2020 11:59	193.3	189.1	185.6	2113	64.91
12/4/2020 11:59	193.2	188.8	185.6	2113	64.96
12/4/2020 12:00	193.4	189	185.8	2120	64.96
12/4/2020 12:00	193.5	189	185.6	2118	64.92
12/4/2020 12:00	193.6	188.8	185.4	2116	65.08
12/4/2020 12:00	193.8	189.2	185.5	2118	64.92
12/4/2020 12:00	193.8	189.1	186.3	2118	64.99
12/4/2020 12:00	193.9	189.3	186.2	2117	64.96
12/4/2020 12:00	194	189.1	185.8	2114	64.96
12/4/2020 12:00	194.1	188.8	185.8	2118	64.96
12/4/2020 12:00	194.1	188.8	185.9	2119	65
12/4/2020 12:00	194.2	188.9	185.6	2120	64.88
12/4/2020 12:00	194.3	189.2	185.5	2124	64.99
12/4/2020 12:00	194.3	188.9	185.5	2127	65
12/4/2020 12:00	194.4	188.8	185.6	2123	64.99
12/4/2020 12:00	194.4	188.7	186.5	2120	65.04
12/4/2020 12:00	194.4	188.9	186.6	2120	64.96
12/4/2020 12:00	194.6	189.2	186.1	2119	64.83
12/4/2020 12:00	194.6	189	185.7	2116	64.88
12/4/2020 12:00	194.6	189	186.1	2117	64.92
12/4/2020 12:00	194.6	189.1	185.9	2117	64.99
12/4/2020 12:00	194.6	188.8	186	2118	64.96
12/4/2020 12:00	194.7	189.3	185.8	2123	65.12
12/4/2020 12:00	194.7	188.9	185.8	2125	65.08
12/4/2020 12:00	194.7	188.8	185.7	2124	64.99
12/4/2020 12:00	194.7	189.1	185.4	2123	64.84
12/4/2020 12:00	194.8	188.8	185.5	2119	64.99
12/4/2020 12:00	194.8	189.1	185.8	2124	64.92
12/4/2020 12:00	194.8	188.7	186.2	2125	64.84
12/4/2020 12:00	194.8	189.3	186.6	2124	64.88
12/4/2020 12:00	194.8	189.1	186.6	2122	64.92
12/4/2020 12:00	194.8	188.8	186.7	2127	65.04
12/4/2020 12:00	194.8	189.1	186.8	2127	65.04
12/4/2020 12:00	194.8	189.7	186.5	2123	65.04
12/4/2020 12:00	194.8	189.7	186.5	2123	64.99
12/4/2020 12:00	194.8	190.2	186.5	2124	64.92
12/4/2020 12:00	194.8	189.7	186.3	2127	64.92
12/4/2020 12:00	194.8	190.3	186.3	2127	64.92
12/4/2020 12:00	194.8	190.1	186.4	2125	64.99
12/4/2020 12:00	194.8	189.6	186.4	2127	64.99
12/4/2020 12:00	194.8	190.3	186.6	2127	64.84
12/4/2020 12:00	194.8	189.7	186.6	2127	65.08
12/4/2020 12:00	194.8	190.4	186.8	2127	64.92

12/4/2020 12:00	194.8	190.3	186.5	2127	65.03
12/4/2020 12:00	194.8	189.6	185.9	2130	65
12/4/2020 12:00	194.8	190.1	186	2131	64.92
12/4/2020 12:00	194.7	189.8	185.7	2131	64.99
12/4/2020 12:00	194.7	190.1	186	2128	64.88
12/4/2020 12:00	194.7	190.4	186.1	2129	64.92
12/4/2020 12:00	194.6	189.8	186.2	2134	65.04
12/4/2020 12:00	194.7	190.2	185.8	2137	64.92
12/4/2020 12:00	194.7	189.8	186.4	2139	65.12
12/4/2020 12:00	194.6	189.9	186.9	2137	64.92
12/4/2020 12:00	194.6	190.3	186.1	2135	65.05
12/4/2020 12:00	194.6	189.8	186	2131	64.96
12/4/2020 12:00	194.6	190.3	186.2	2130	64.87
12/4/2020 12:00	194.5	189.6	186.4	2126	65
12/4/2020 12:00	194.5	189.9	186.2	2127	65
12/4/2020 12:00	194.4	190.3	186	2129	64.99
12/4/2020 12:00	194.4	189.8	186.7	2127	64.96
12/4/2020 12:00	194.4	190.2	186.7	2127	65
12/4/2020 12:01	194.4	190	186	2127	64.96
12/4/2020 12:01	194.4	189.8	186	2130	64.96
12/4/2020 12:01	194.3	190.3	186	2133	64.96
12/4/2020 12:01	194.4	189.7	186.1	2131	64.96
12/4/2020 12:01	194.3	190.2	186.3	2129	64.88
12/4/2020 12:01	194.2	189.7	186.3	2131	64.88
12/4/2020 12:01	194.2	190.3	186.3	2131	65.12
12/4/2020 12:01	194.2	190.3	186.2	2135	64.88
12/4/2020 12:01	194.1	189.6	186	2132	64.95
12/4/2020 12:01	194.1	190	186.1	2135	64.84
12/4/2020 12:01	194.1	190.3	186.2	2134	64.99
12/4/2020 12:01	194	190.2	186.6	2131	65.08
12/4/2020 12:01	194	190.3	187	2131	64.92
12/4/2020 12:01	193.9	189.8	186.8	2133	64.96
12/4/2020 12:01	193.9	190	186.3	2134	64.92
12/4/2020 12:01	193.8	189.7	186	2137	65
12/4/2020 12:01	193.8	190.2	185.7	2133	64.96
12/4/2020 12:01	193.7	190.4	185.9	2131	64.96
12/4/2020 12:01	193.7	189.7	186.2	2130	65.04
12/4/2020 12:01	193.7	189.9	186.1	2127	65
12/4/2020 12:01	193.6	189.8	186.3	2130	64.96
12/4/2020 12:01	193.6	190.2	186.1	2129	65
12/4/2020 12:01	193.6	190.3	186.3	2127	65
12/4/2020 12:01	193.5	190	186.4	2126	65
12/4/2020 12:01	193.5	190.1	186.2	2122	64.88
12/4/2020 12:01	193.5	189.8	186.3	2128	64.96
12/4/2020 12:01	193.4	190	186.2	2127	64.96
12/4/2020 12:01	193.4	190.3	186.2	2131	64.92
12/4/2020 12:01	193.3	189.8	186.2	2129	64.96

12/4/2020 12:01	193.4	190.4	186	2129	64.95
12/4/2020 12:01	193.2	190.2	185.9	2128	64.96
12/4/2020 12:01	193.2	189.7	186	2128	64.88
12/4/2020 12:01	193.1	189.8	186.3	2127	64.88
12/4/2020 12:01	193.1	189.6	187	2130	65.08
12/4/2020 12:01	193.1	190.2	186.6	2130	65.08
12/4/2020 12:01	193	190.2	185.9	2130	64.92
12/4/2020 12:01	193	189.6	186.2	2133	65
12/4/2020 12:01	193	190.1	186.1	2136	64.99
12/4/2020 12:01	192.9	189.8	186.2	2135	64.84
12/4/2020 12:01	192.9	190.1	186.1	2138	65.12
12/4/2020 12:01	192.9	190.3	186	2140	64.88
12/4/2020 12:01	192.9	190.3	187	2133	64.88
12/4/2020 12:01	192.8	190.3	187	2133	65.08
12/4/2020 12:01	192.8	190	186.3	2131	64.99
12/4/2020 12:01	192.7	189.7	186.2	2134	64.88
12/4/2020 12:01	192.7	190.3	186.2	2135	65.09
12/4/2020 12:01	192.6	189.9	186.2	2136	64.92
12/4/2020 12:01	192.6	190.5	186.4	2131	64.96
12/4/2020 12:01	192.6	190.3	186.5	2134	65.04
12/4/2020 12:01	192.5	190.3	186	2130	64.96
12/4/2020 12:01	192.5	189.8	186.1	2133	64.96
12/4/2020 12:01	192.5	189.7	186.2	2131	65
12/4/2020 12:01	192.5	190.2	186.7	2135	64.92
12/4/2020 12:01	192.4	189.8	186.7	2133	64.92
12/4/2020 12:01	192.3	190.2	187	2134	65.04
12/4/2020 12:01	192.4	190.5	187	2138	64.96
12/4/2020 12:01	192.3	189.6	186.9	2138	64.92
12/4/2020 12:01	192.3	190.3	187	2137	65.05
12/4/2020 12:01	192.2	189.6	187	2139	64.92
12/4/2020 12:02	192.2	190.2	187	2135	64.88
12/4/2020 12:02	192.2	190.4	187	2137	65.16
12/4/2020 12:02	192.1	190.2	186.5	2136	64.92
12/4/2020 12:02	192.2	190.3	186.2	2134	64.88
12/4/2020 12:02	192.1	190	186	2139	65.12
12/4/2020 12:02	192	189.6	186	2142	64.92
12/4/2020 12:02	192	189.9	186.1	2142	64.88
12/4/2020 12:02	191.9	189.8	186.2	2140	64.93
12/4/2020 12:02	191.9	190.3	186.1	2134	64.92
12/4/2020 12:02	191.9	190.2	185.9	2135	65
12/4/2020 12:02	191.8	189.7	186.8	2135	65.01
12/4/2020 12:02	191.8	189.8	186.6	2134	65
12/4/2020 12:02	191.7	189.6	186.1	2131	65
12/4/2020 12:02	191.7	190	186.2	2131	64.96
12/4/2020 12:02	191.7	190.3	186.1	2134	65
12/4/2020 12:02	191.6	190.3	186.2	2133	65
12/4/2020 12:02	191.6	190.1	186.2	2133	64.96

12/4/2020 12:02	191.5	190	186.8	2130	64.96
12/4/2020 12:02	191.5	189.6	186.1	2130	64.96
12/4/2020 12:02	191.5	189.7	185.8	2134	65.05
12/4/2020 12:02	191.4	190.2	186.3	2133	64.88
12/4/2020 12:02	191.3	190.5	186.3	2133	65
12/4/2020 12:02	191.3	189.8	186	2137	65.03
12/4/2020 12:02	191.3	190	186.3	2133	64.96
12/4/2020 12:02	191.2	190.4	187	2136	64.99
12/4/2020 12:02	191.2	189.6	185.8	2139	65.08
12/4/2020 12:02	191.1	190.1	185.9	2140	64.92
12/4/2020 12:02	191	190	186.2	2142	64.93
12/4/2020 12:02	191	189.7	186.2	2141	64.96
12/4/2020 12:02	191	190.3	186.1	2141	65.03
12/4/2020 12:02	190.9	189.8	186.3	2140	64.96
12/4/2020 12:02	191	190.2	186.8	2142	65.12
12/4/2020 12:02	190.9	189.6	186.2	2140	65.08
12/4/2020 12:02	190.8	190.5	185.8	2138	64.99
12/4/2020 12:02	190.8	190.3	186	2141	65.12
12/4/2020 12:02	190.8	189.8	186.1	2138	64.95
12/4/2020 12:02	190.7	189.9	186.1	2135	64.96
12/4/2020 12:02	190.7	190.2	186.3	2133	65.04
12/4/2020 12:02	190.6	189.8	186.2	2138	64.91
12/4/2020 12:02	190.6	190.2	186	2138	64.92
12/4/2020 12:02	190.5	189.8	186.2	2139	65
12/4/2020 12:02	190.6	190.3	186	2140	64.96
12/4/2020 12:02	190.5	190.3	186.1	2138	64.92
12/4/2020 12:02	190.5	189.7	186.4	2140	65.09
12/4/2020 12:02	190.5	190.5	186.3	2143	65
12/4/2020 12:02	190.4	190.1	186.5	2144	65
12/4/2020 12:02	190.4	190.4	186.8	2142	64.96
12/4/2020 12:02	190.3	190.2	186.7	2140	64.88
12/4/2020 12:02	190.2	189.7	187	2138	65
12/4/2020 12:02	190.3	190	186.9	2135	65.08
12/4/2020 12:02	190.2	189.7	187	2136	65.04
12/4/2020 12:02	190.1	190.1	187	2137	64.88
12/4/2020 12:02	190.2	190.1	186.5	2140	65.12
12/4/2020 12:02	190	189.7	186.5	2135	64.88
12/4/2020 12:02	190	190.2	186.5	2139	65.04
12/4/2020 12:02	189.9	189.9	186.3	2138	65.01
12/4/2020 12:02	189.8	189.8	186.3	2139	64.96
12/4/2020 12:02	189.9	189.6	186.3	2135	64.92
12/4/2020 12:02	189.8	190.1	186.6	2140	64.96
12/4/2020 12:02	189.8	190.5	186.7	2141	64.99
12/4/2020 12:03	189.7	190.2	186.5	2140	65.04
12/4/2020 12:03	189.7	190.4	186.8	2140	64.96
12/4/2020 12:03	189.6	189.6	186.6	2139	65
12/4/2020 12:03	189.6	189.6	186.6	2141	64.96

12/4/2020 12:03	189.6	190.4	186.9	2141	65.01
12/4/2020 12:03	189.6	190.1	186.8	2138	64.96
12/4/2020 12:03	189.5	189.7	186.7	2136	65
12/4/2020 12:03	189.5	189.8	186.8	2136	65
12/4/2020 12:03	189.5	190.1	186.7	2130	65
12/4/2020 12:03	189.4	190.3	186.8	2133	65
12/4/2020 12:03	189.4	190.2	186.8	2131	65
12/4/2020 12:03	189.4	189.6	186.4	2132	64.96
12/4/2020 12:03	189.4	190.6	186.4	2128	64.92
12/4/2020 12:03	189.3	189.6	186.4	2133	65
12/4/2020 12:03	189.3	190.1	186.2	2133	64.92
12/4/2020 12:03	189.3	190.3	186.1	2131	65
12/4/2020 12:03	189.3	189.6	186.1	2131	65.05
12/4/2020 12:03	189.3	190	186	2127	65.04
12/4/2020 12:03	189.3	189.8	185.9	2129	65.04
12/4/2020 12:03	189.3	190.1	185.8	2127	65.04
12/4/2020 12:03	189.3	190.2	186	2128	65.04
12/4/2020 12:03	189.3	190.3	185.8	2127	65.04
12/4/2020 12:03	189.3	189.9	186.1	2128	64.96
12/4/2020 12:03	189.3	189.7	186.1	2128	64.84
12/4/2020 12:03	189.3	190.2	186.1	2127	64.84
12/4/2020 12:03	189.3	190.2	186.1	2130	65.12
12/4/2020 12:03	189.4	189.7	186.2	2136	64.92
12/4/2020 12:03	189.3	190.4	186.2	2134	64.92
12/4/2020 12:03	189.3	189.7	186.2	2133	64.92
12/4/2020 12:03	189.3	190.1	186.3	2131	64.96
12/4/2020 12:03	189.3	190.5	186.1	2133	65.04
12/4/2020 12:03	189.4	189.9	186.1	2133	64.92
12/4/2020 12:03	189.3	190.3	186.1	2130	64.96
12/4/2020 12:03	189.4	189.6	186.1	2129	64.96
12/4/2020 12:03	189.4	190.1	186.2	2133	64.96
12/4/2020 12:03	189.4	190.3	186	2133	65.04
12/4/2020 12:03	189.5	190.1	186.1	2135	65.04
12/4/2020 12:03	189.5	190.1	186.2	2134	64.96
12/4/2020 12:03	189.4	189.8	186	2131	64.92
12/4/2020 12:03	189.5	190	186	2135	64.92
12/4/2020 12:03	189.4	190.5	186.2	2134	64.96
12/4/2020 12:03	189.5	189	186	2136	65.08
12/4/2020 12:03	189.5	189.2	186.3	2132	65.03
12/4/2020 12:03	189.5	188.8	186.1	2134	64.99
12/4/2020 12:03	189.6	188.9	186	2134	65.04
12/4/2020 12:03	189.5	189.3	186.2	2136	64.96
12/4/2020 12:03	189.6	189.1	186	2131	65.03
12/4/2020 12:03	189.5	189.1	186	2131	65.08
12/4/2020 12:03	189.6	188.8	186.1	2133	65.04
12/4/2020 12:03	189.6	188.9	186.2	2134	65.04
12/4/2020 12:03	189.6	189.3	186.7	2140	64.97

12/4/2020 12:03	189.6	189.2	186.7	2137	64.95
12/4/2020 12:03	189.7	189.1	186.5	2138	64.92
12/4/2020 12:03	189.6	188.9	186	2138	65.12
12/4/2020 12:03	189.7	188.8	185.7	2135	64.99
12/4/2020 12:03	189.8	188.7	185.9	2138	64.92
12/4/2020 12:03	189.7	188.9	186.1	2140	65.04
12/4/2020 12:03	189.8	189.3	186	2138	65.04
12/4/2020 12:03	189.8	189.1	186.1	2135	65
12/4/2020 12:04	189.7	188.9	186.1	2135	65
12/4/2020 12:04	189.8	189.3	186.1	2137	64.96
12/4/2020 12:04	189.8	188.8	186.8	2138	64.96
12/4/2020 12:04	189.8	189.1	186.6	2140	65
12/4/2020 12:04	189.8	189	185.6	2134	64.96
12/4/2020 12:04	189.9	188.8	186	2134	64.96
12/4/2020 12:04	189.9	189.3	186.3	2134	65
12/4/2020 12:04	190	189	186.1	2130	64.96
12/4/2020 12:04	190	189.1	186.1	2129	64.92
12/4/2020 12:04	190	189	186.7	2133	65.05
12/4/2020 12:04	190	188.9	185.9	2136	64.96
12/4/2020 12:04	190.1	189.1	186	2136	65.04
12/4/2020 12:04	190.1	189	186.1	2138	64.96
12/4/2020 12:04	190.1	189.3	186.2	2138	64.99
12/4/2020 12:04	190.1	189	185.9	2138	65.04
12/4/2020 12:04	190.2	188.7	186.3	2140	65.08
12/4/2020 12:04	190.2	189.3	186.9	2138	64.84
12/4/2020 12:04	190.2	188.9	186	2138	65.03
12/4/2020 12:04	190.3	189.3	186	2135	65.05
12/4/2020 12:04	190.3	189.1	186.3	2137	65
12/4/2020 12:04	190.3	188.8	186.2	2134	64.88
12/4/2020 12:04	190.3	189.1	186.1	2133	65.05
12/4/2020 12:04	190.3	188.8	186.5	2133	64.99
12/4/2020 12:04	190.5	189.3	186.8	2133	64.92
12/4/2020 12:04	190.5	189.1	186	2136	64.92
12/4/2020 12:04	190.5	188.7	186	2138	64.87
12/4/2020 12:04	190.5	189.2	186.2	2140	65.04
12/4/2020 12:04	190.5	188.9	186.1	2139	65.16
12/4/2020 12:04	190.5	188.8	186	2138	64.96
12/4/2020 12:04	190.5	189	186.6	2140	64.92
12/4/2020 12:04	190.5	188.8	186.9	2142	65
12/4/2020 12:04	190.5	188.9	186.1	2139	64.96
12/4/2020 12:04	190.6	188.9	186.1	2138	65.04
12/4/2020 12:04	190.5	189.2	186.2	2137	64.96
12/4/2020 12:04	190.5	189.3	186.2	2134	64.88
12/4/2020 12:04	190.5	188.8	186.3	2138	65.04
12/4/2020 12:04	190.6	189.1	186.1	2134	64.88
12/4/2020 12:04	190.6	188.8	186.8	2139	65.08
12/4/2020 12:04	190.5	189.1	186.5	2140	65.01

12/4/2020 12:04	190.6	189.1	185.7	2140	64.88
12/4/2020 12:04	190.5	188.8	185.9	2138	64.92
12/4/2020 12:04	190.5	189.3	186.1	2134	64.92
12/4/2020 12:04	190.5	189	186.2	2133	64.83
12/4/2020 12:04	190.5	188.9	186.4	2127	64.88
12/4/2020 12:04	190.5	189.2	186	2127	65.05
12/4/2020 12:04	190.5	188.7	186	2127	64.96
12/4/2020 12:04	190.5	189.2	186.3	2131	64.92
12/4/2020 12:04	190.5	189.1	186.7	2134	65.01
12/4/2020 12:04	190.5	188.9	187	2131	64.88
12/4/2020 12:04	190.4	188.8	186.7	2133	65
12/4/2020 12:04	190.4	188.8	186.5	2130	64.96
12/4/2020 12:04	190.5	189.2	186.5	2129	64.96
12/4/2020 12:04	190.4	188.9	186.3	2131	64.92
12/4/2020 12:04	190.4	189.3	186.3	2130	65
12/4/2020 12:04	190.4	189	186.2	2135	65
12/4/2020 12:04	190.5	188.8	186.1	2134	64.92
12/4/2020 12:04	190.5	189.2	186.2	2139	65.09
12/4/2020 12:04	190.5	188.9	186.5	2134	64.87
12/4/2020 12:04	190.5	188.7	186.6	2134	64.92
12/4/2020 12:04	190.5	189.3	187	2135	65.05
12/4/2020 12:05	190.5	188.9	187.1	2131	64.99
12/4/2020 12:05	190.6	188.9	186.7	2124	64.96
12/4/2020 12:05	190.6	189.1	186.3	2123	64.96
12/4/2020 12:05	190.7	189.3	186	2124	64.87
12/4/2020 12:05	190.7	188.9	186.2	2127	64.88
12/4/2020 12:05	190.8	188.9	186.1	2129	65.05
12/4/2020 12:05	190.9	189	186.1	2129	64.83
12/4/2020 12:05	190.9	188.8	186.3	2127	64.99
12/4/2020 12:05	190.9	189.1	186.1	2126	65.08
12/4/2020 12:05	191	189.1	186.2	2126	64.99
12/4/2020 12:05	191.1	188.9	186.2	2122	65.04
12/4/2020 12:05	191.1	188.9	186	2122	65.04
12/4/2020 12:05	191.1	188.8	186.3	2115	64.83
12/4/2020 12:05	191.2	189	186.8	2118	64.84
12/4/2020 12:05	191.3	189.2	186.9	2120	64.96
12/4/2020 12:05	191.4	188.9	186.5	2119	64.88
12/4/2020 12:05	191.6	188.8	186.2	2118	64.84
12/4/2020 12:05	191.6	188.6	186.3	2117	65.08
12/4/2020 12:05	191.7	188.9	186.3	2119	64.92
12/4/2020 12:05	191.8	189.1	186.3	2118	65
12/4/2020 12:05	191.9	188.6	186.4	2120	65
12/4/2020 12:05	191.9	189.3	186.2	2121	64.88
12/4/2020 12:05	192	188.8	186.1	2124	65.04
12/4/2020 12:05	192.2	188.8	186.3	2123	65
12/4/2020 12:05	192.3	189.2	186.1	2127	65
12/4/2020 12:05	192.3	188.7	186.3	2127	65

12/4/2020 12:05	192.4	188.7	186.3	2129	65.09
12/4/2020 12:05	192.4	188.8	186.4	2131	64.91
12/4/2020 12:05	192.6	189.1	186.9	2137	64.96
12/4/2020 12:05	192.7	189.2	187	2137	65.04
12/4/2020 12:05	192.7	190.2	187.1	2134	64.96
12/4/2020 12:05	192.8	190.3	187.1	2131	64.96
12/4/2020 12:05	192.9	190.4	187	2133	65
12/4/2020 12:05	192.9	189.8	186.7	2139	65
12/4/2020 12:05	193	190	186.7	2137	65
12/4/2020 12:05	193.1	190.2	186.5	2143	64.96
12/4/2020 12:05	193.1	190.4	186.3	2144	64.96
12/4/2020 12:05	193.2	190	186.3	2142	64.95
12/4/2020 12:05	193.3	190	186.2	2140	64.96
12/4/2020 12:05	193.2	190	186.5	2138	65.04
12/4/2020 12:05	193.3	190.3	186.8	2138	64.92
12/4/2020 12:05	193.3	189.6	186.5	2138	65.08
12/4/2020 12:05	193.3	190.2	186.7	2136	64.84
12/4/2020 12:05	193.3	189.7	186.3	2134	64.95
12/4/2020 12:05	193.3	190	186.7	2138	65.16
12/4/2020 12:05	193.3	190.3	187.3	2142	64.96
12/4/2020 12:05	193.3	189.7	186.6	2144	64.99
12/4/2020 12:05	193.3	190.3	186.3	2144	65.01
12/4/2020 12:05	193.4	189.9	186.7	2140	64.95
12/4/2020 12:05	193.3	189.8	186.7	2142	64.92
12/4/2020 12:05	193.3	190.5	186.7	2144	64.96
12/4/2020 12:05	193.3	189.7	186.5	2142	64.99
12/4/2020 12:05	193.3	190.2	186.7	2143	64.96
12/4/2020 12:05	193.3	190	187.4	2145	64.92
12/4/2020 12:05	193.2	189.6	187	2148	64.96
12/4/2020 12:05	193.2	190.2	186.3	2151	64.96
12/4/2020 12:05	193.3	189.6	186.7	2156	65.09
12/4/2020 12:05	193.2	189.9	186.7	2152	64.96
12/4/2020 12:05	193.2	189.9	186.8	2145	64.92
12/4/2020 12:06	193.1	190.3	186.5	2149	65
12/4/2020 12:06	193.1	190	186.5	2145	64.92
12/4/2020 12:06	193	189.8	187.2	2143	64.92
12/4/2020 12:06	193.1	190.3	187.6	2144	65.04
12/4/2020 12:06	192.9	190.2	186.8	2147	65
12/4/2020 12:06	192.9	189.6	186.4	2147	64.96
12/4/2020 12:06	192.9	189.9	186.3	2152	65.09
12/4/2020 12:06	192.9	190	186.6	2151	64.88
12/4/2020 12:06	192.8	190.2	186.8	2148	65.08
12/4/2020 12:06	192.8	190.1	186.9	2152	65.08
12/4/2020 12:06	192.7	189.6	186.8	2151	64.88
12/4/2020 12:06	192.6	190.2	186.7	2149	64.92
12/4/2020 12:06	192.6	189.7	186.5	2153	64.96
12/4/2020 12:06	192.6	190.1	186.7	2156	64.88

12/4/2020 12:06	192.5	190.2	186.9	2154	64.88
12/4/2020 12:06	192.4	190.2	187	2160	65
12/4/2020 12:06	192.3	190.3	187.6	2157	65.12
12/4/2020 12:06	192.4	189.9	187.5	2156	64.92
12/4/2020 12:06	192.3	190.1	187.2	2160	65.08
12/4/2020 12:06	192.2	189.7	186.8	2156	64.8
12/4/2020 12:06	192.1	190.3	186.5	2152	65.03
12/4/2020 12:06	192.1	190.5	186.5	2148	65.16
12/4/2020 12:06	192	189.7	186.4	2150	65.04
12/4/2020 12:06	192	189.9	186.3	2155	64.99
12/4/2020 12:06	192	189.8	186.4	2150	65.12
12/4/2020 12:06	191.9	190.2	186.5	2152	64.92
12/4/2020 12:06	191.8	190.3	186.5	2149	64.96
12/4/2020 12:06	191.8	189.9	186.7	2151	64.96
12/4/2020 12:06	191.8	190.3	186.6	2146	64.88
12/4/2020 12:06	191.8	190.2	186.6	2146	65.04
12/4/2020 12:06	191.8	189.8	186.7	2146	64.96
12/4/2020 12:06	191.7	190.3	186.5	2146	64.96
12/4/2020 12:06	191.7	189.6	186.8	2142	64.87
12/4/2020 12:06	191.6	190.3	186.7	2148	65.08
12/4/2020 12:06	191.6	190	186.6	2151	64.88
12/4/2020 12:06	191.6	189.6	186.8	2151	64.92
12/4/2020 12:06	191.6	190.1	186.4	2152	64.96
12/4/2020 12:06	191.6	189.8	186.7	2152	65
12/4/2020 12:06	191.5	190	186.5	2152	65
12/4/2020 12:06	191.6	190.3	186.3	2154	65
12/4/2020 12:06	191.5	189.6	186.3	2153	64.92
12/4/2020 12:06	191.6	190.3	186.1	2153	65
12/4/2020 12:06	191.5	189.9	186.1	2153	65
12/4/2020 12:06	191.4	190.1	186.3	2151	65
12/4/2020 12:06	191.5	190.4	186.4	2153	64.96
12/4/2020 12:06	191.4	189.5	186.3	2151	64.96
12/4/2020 12:06	191.5	190.2	186.3	2151	65
12/4/2020 12:06	191.5	190.3	186.4	2148	65
12/4/2020 12:06	191.4	189.7	186.8	2146	64.96
12/4/2020 12:06	191.3	190.4	187	2146	65
12/4/2020 12:06	191.3	190.1	187.1	2149	65.04
12/4/2020 12:06	191.4	190.3	187.2	2150	64.99
12/4/2020 12:06	191.3	190.1	187.1	2147	65.08
12/4/2020 12:06	191.3	189.8	187	2145	65.08
12/4/2020 12:06	191.3	190.3	186.8	2147	65
12/4/2020 12:06	191.3	189.6	186.5	2148	65.04
12/4/2020 12:06	191.3	191.1	186.4	2152	64.96
12/4/2020 12:06	191.2	190.2	186.3	2154	65.04
12/4/2020 12:06	191.2	190.5	186.3	2152	65.03
12/4/2020 12:07	191.1	189.8	186.5	2154	65.08
12/4/2020 12:07	191.1	189.6	186.6	2154	64.88

12/4/2020 12:07	191.2	190.5	186.8	2155	64.99
12/4/2020 12:07	191.1	190	186.6	2156	65.12
12/4/2020 12:07	191.1	189.7	186.3	2159	64.92
12/4/2020 12:07	191.1	189.7	187	2156	64.96
12/4/2020 12:07	191	189.8	187.3	2152	65
12/4/2020 12:07	190.9	190.3	186.3	2152	64.96
12/4/2020 12:07	190.9	190.2	186.5	2151	65
12/4/2020 12:07	190.9	190.3	186.6	2152	64.96
12/4/2020 12:07	190.9	190.2	186.7	2152	65
12/4/2020 12:07	190.8	189.6	186.4	2151	64.96
12/4/2020 12:07	190.8	190.3	186.8	2156	65.05
12/4/2020 12:07	190.7	190	187.2	2155	64.96
12/4/2020 12:07	190.6	189.7	186.3	2152	64.96
12/4/2020 12:07	190.6	189.7	186.6	2151	65.08
12/4/2020 12:07	190.6	189.6	186.7	2152	64.88
12/4/2020 12:07	190.5	190.3	186.5	2148	64.96
12/4/2020 12:07	190.5	190.1	186.3	2145	65.08
12/4/2020 12:07	190.5	189.5	187	2140	64.88
12/4/2020 12:07	190.5	189.8	187	2138	64.96
12/4/2020 12:07	190.4	189.6	186.3	2138	64.96
12/4/2020 12:07	190.4	190.2	186.5	2135	64.99
12/4/2020 12:07	190.3	190.2	186.6	2140	65.04
12/4/2020 12:07	190.3	190.5	186.6	2142	65.08
12/4/2020 12:07	190.3	190.2	186.5	2144	64.83
12/4/2020 12:07	190.3	189.8	187.1	2141	64.92
12/4/2020 12:07	190.2	190.2	186.8	2139	65.08
12/4/2020 12:07	190.3	189.8	186.2	2134	64.88
12/4/2020 12:07	190.3	190.2	186.7	2131	65.03
12/4/2020 12:07	190.2	190.5	186.7	2137	65.01
12/4/2020 12:07	190.2	189.8	186.2	2135	64.84
12/4/2020 12:07	190.3	190.2	186.5	2134	65.08
12/4/2020 12:07	190.2	189.6	187.1	2131	65.16
12/4/2020 12:07	190.2	190.2	186.3	2133	64.96
12/4/2020 12:07	190.2	190.4	186.2	2131	65.04
12/4/2020 12:07	190.2	190	186.3	2134	65.05
12/4/2020 12:07	190.2	189.9	186.3	2129	64.96
12/4/2020 12:07	190.3	189.7	186.4	2131	65.04
12/4/2020 12:07	190.2	190.2	186.2	2132	65.09
12/4/2020 12:07	190.2	190.2	186.8	2133	64.92
12/4/2020 12:07	190.4	189.8	187	2131	64.96
12/4/2020 12:07	190.3	190.1	186	2134	65.04
12/4/2020 12:07	190.4	190.1	186.4	2134	65
12/4/2020 12:07	190.4	190.2	186.5	2134	64.96
12/4/2020 12:07	190.5	190.2	186.3	2137	65.04
12/4/2020 12:07	190.5	190	186.3	2135	64.87
12/4/2020 12:07	190.5	190.3	186.6	2134	64.92
12/4/2020 12:07	190.6	189.8	187.2	2134	65.08

12/4/2020 12:07	190.7	189.6	186.5	2130	65.04
12/4/2020 12:07	190.8	190	186.3	2127	64.88
12/4/2020 12:07	190.8	190.1	186.4	2126	65
12/4/2020 12:07	190.8	190.2	186.5	2123	64.96
12/4/2020 12:07	190.9	189.8	186.7	2123	65.04
12/4/2020 12:07	190.9	189.8	186.7	2127	65
12/4/2020 12:07	191.1	190.4	186.5	2131	64.99
12/4/2020 12:07	191.1	190.3	186.5	2130	64.92
12/4/2020 12:07	191.1	189.7	186.8	2128	64.92
12/4/2020 12:07	191.3	189.8	187.2	2127	64.88
12/4/2020 12:07	191.4	189.6	187.2	2130	64.88
12/4/2020 12:08	191.5	190.2	187.2	2128	64.92
12/4/2020 12:08	191.5	190	187	2127	64.96
12/4/2020 12:08	191.6	189.7	187	2126	64.88
12/4/2020 12:08	191.9	189.5	186.8	2129	65.01
12/4/2020 12:08	191.9	189.8	187	2127	64.92
12/4/2020 12:08	192.1	190.2	187.1	2127	65.03
12/4/2020 12:08	192.1	190.1	186.9	2130	64.88
12/4/2020 12:08	192.1	190.3	187.3	2130	65.04
12/4/2020 12:08	192.2	190.3	187.3	2130	64.84
12/4/2020 12:08	192.4	190.2	187.4	2129	65.04
12/4/2020 12:08	192.5	190.2	187.4	2131	64.84
12/4/2020 12:08	192.6	190.1	187.2	2131	64.93
12/4/2020 12:08	192.6	189.6	187.2	2133	65.12
12/4/2020 12:08	192.8	190.3	187.1	2135	65
12/4/2020 12:08	192.9	189.8	186.9	2134	64.96
12/4/2020 12:08	192.9	190.3	186.7	2139	64.96
12/4/2020 12:08	193.1	190	186.7	2145	64.92
12/4/2020 12:08	193.1	189.8	186.5	2142	65
12/4/2020 12:08	193.2	190.3	186.6	2145	64.91
12/4/2020 12:08	193.3	189.9	186.5	2146	64.96
12/4/2020 12:08	193.3	190.5	186.6	2148	64.92
12/4/2020 12:08	193.5	190.1	186.6	2149	65.04
12/4/2020 12:08	193.5	189.9	186.6	2146	64.96
12/4/2020 12:08	193.6	190.5	186.5	2140	64.96
12/4/2020 12:08	193.6	189.8	186.6	2147	64.96
12/4/2020 12:08	193.6	190.3	186.7	2144	64.96
12/4/2020 12:08	193.8	190.3	186.7	2144	65
12/4/2020 12:08	193.8	190.4	186.7	2145	65
12/4/2020 12:08	193.9	190.1	186.8	2144	65
12/4/2020 12:08	193.9	189.8	186.8	2146	64.96
12/4/2020 12:08	193.9	190.2	186.9	2149	65.05
12/4/2020 12:08	194	189.6	186.7	2153	65.04
12/4/2020 12:08	194.1	190.3	186.7	2156	64.96
12/4/2020 12:08	194.1	190.5	186.7	2160	65.04
12/4/2020 12:08	194.1	190	186.6	2150	64.99
12/4/2020 12:08	194.1	190.2	186.7	2147	65.04

12/4/2020 12:08	194.2	190.3	186.7	2152	64.92
12/4/2020 12:08	194.2	189.8	186.8	2155	64.88
12/4/2020 12:08	194.2	190.5	187	2153	65.08
12/4/2020 12:08	194.2	190	186.8	2154	65.08
12/4/2020 12:08	194.3	190.4	187	2149	64.96
12/4/2020 12:08	194.3	189.7	187	2145	64.96
12/4/2020 12:08	194.3	189.6	187.1	2149	65.12
12/4/2020 12:08	194.3	190.4	187.2	2145	65.04
12/4/2020 12:08	194.3	190	187	2145	65.04
12/4/2020 12:08	194.3	190.3	187	2152	65.05
12/4/2020 12:08	194.4	189.6	186.8	2150	64.96
12/4/2020 12:08	194.4	189.7	186.7	2149	65
12/4/2020 12:08	194.4	189.9	187.2	2148	65.04
12/4/2020 12:08	194.4	189.7	187.6	2147	64.92
12/4/2020 12:08	194.4	190	187.4	2150	64.96
12/4/2020 12:08	194.4	190.3	187	2148	65.09
12/4/2020 12:08	194.4	189.7	186.9	2151	64.96
12/4/2020 12:08	194.4	190.2	187	2151	64.88
12/4/2020 12:08	194.4	189.8	187.2	2154	65.05
12/4/2020 12:08	194.4	189.8	186.9	2152	64.99
12/4/2020 12:08	194.4	189.7	186.8	2151	64.92
12/4/2020 12:08	194.4	190.3	186.7	2152	65.04
12/4/2020 12:08	194.4	190.4	187	2152	64.95
12/4/2020 12:09	194.4	189.7	187.7	2147	64.99
12/4/2020 12:09	194.3	189.7	187.7	2149	65.12
12/4/2020 12:09	194.4	190.5	186.9	2153	65.08
12/4/2020 12:09	194.3	189.8	186.9	2149	64.92
12/4/2020 12:09	194.3	190.1	186.8	2148	65.12
12/4/2020 12:09	194.4	189.5	187.2	2145	64.99
12/4/2020 12:09	194.3	190	187.2	2143	65.12
12/4/2020 12:09	194.3	190.3	187	2144	64.96
12/4/2020 12:09	194.2	189.7	187.2	2146	64.88
12/4/2020 12:09	194.3	190.3	186.9	2149	65.03
12/4/2020 12:09	194.1	189.6	186.9	2147	65
12/4/2020 12:09	194.2	190	187.1	2148	64.96
12/4/2020 12:09	194.1	190.5	187.5	2145	64.99
12/4/2020 12:09	194.1	189.6	188	2150	65
12/4/2020 12:09	194.1	190.3	187.8	2152	65
12/4/2020 12:09	194.1	190.2	187	2150	64.92
12/4/2020 12:09	194	189.9	187	2151	65.05
12/4/2020 12:09	194.1	190.5	187.2	2151	65.04
12/4/2020 12:09	193.9	189.8	187.1	2152	65
12/4/2020 12:09	194	190.5	187.4	2153	65.04
12/4/2020 12:09	194	190.1	187.2	2151	65
12/4/2020 12:09	193.9	189.8	186.8	2153	64.95
12/4/2020 12:09	193.9	190.3	187	2155	65.12
12/4/2020 12:09	193.8	189.6	187.3	2159	64.88

12/4/2020 12:09	193.9	191.4	187.9	2157	64.96
12/4/2020 12:09	193.8	191.5	187.5	2160	65.12
12/4/2020 12:09	193.8	190.8	187	2159	64.92
12/4/2020 12:09	193.8	191	187	2158	64.99
12/4/2020 12:09	193.7	191	187	2159	65.01
12/4/2020 12:09	193.7	191.3	187	2153	64.92
12/4/2020 12:09	193.6	191.4	187.2	2154	64.99
12/4/2020 12:09	193.6	190.8	187	2158	65.08
12/4/2020 12:09	193.6	191.2	187	2155	65.08
12/4/2020 12:09	193.5	190.6	186.9	2150	64.88
12/4/2020 12:09	193.5	190.9	187.2	2152	65
12/4/2020 12:09	193.5	191.5	187.9	2148	64.96
12/4/2020 12:09	193.5	190.8	187.9	2145	64.92
12/4/2020 12:09	193.5	191.4	187.4	2147	65.08
12/4/2020 12:09	193.5	191.4	187	2151	64.92
12/4/2020 12:09	193.4	191.3	186.8	2156	65
12/4/2020 12:09	193.4	191.4	187.2	2155	64.96
12/4/2020 12:09	193.3	190.7	187.3	2156	65
12/4/2020 12:09	193.3	191.2	187.1	2160	64.92
12/4/2020 12:09	193.3	190.7	187.3	2161	65.04
12/4/2020 12:09	193.3	190.9	187.3	2161	65.08
12/4/2020 12:09	193.2	191.4	187.2	2158	64.92
12/4/2020 12:09	193.1	191	187.1	2161	65
12/4/2020 12:09	193.2	191.4	187.1	2159	64.88
12/4/2020 12:09	193.1	191.3	187	2156	64.92
12/4/2020 12:09	193.1	190.8	187.3	2156	64.96
12/4/2020 12:09	193.1	191.4	187.2	2161	65
12/4/2020 12:09	193	190.7	187.2	2160	64.92
12/4/2020 12:09	193	191.2	187	2159	65.08
12/4/2020 12:09	192.9	191	187.2	2159	64.92
12/4/2020 12:09	192.9	190.8	187.3	2159	64.96
12/4/2020 12:09	192.9	191.4	187.1	2157	65.08
12/4/2020 12:09	192.9	190.9	187.2	2160	65.08
12/4/2020 12:09	192.8	191.4	187.2	2162	64.88
12/4/2020 12:09	192.9	191.2	187	2160	65.04
12/4/2020 12:09	192.8	190.7	186.9	2159	64.92
12/4/2020 12:10	192.8	191.4	187	2154	64.92
12/4/2020 12:10	192.8	191	187	2149	65.04
12/4/2020 12:10	192.6	191.5	187.6	2153	64.92
12/4/2020 12:10	192.7	191	187.7	2156	64.92
12/4/2020 12:10	192.7	190.8	187.7	2160	65.04
12/4/2020 12:10	192.6	191.5	187.8	2160	64.92
12/4/2020 12:10	192.6	191.2	187.4	2152	64.92
12/4/2020 12:10	192.6	191.2	186.8	2153	65.05
12/4/2020 12:10	192.6	191.4	186.9	2159	65
12/4/2020 12:10	192.5	190.8	186.8	2161	65
12/4/2020 12:10	192.4	191.1	187	2163	65.08

12/4/2020 12:10	192.5	190.8	187.2	2162	64.96
12/4/2020 12:10	192.4	190.9	187.2	2163	64.96
12/4/2020 12:10	192.4	191.4	187.1	2160	64.96
12/4/2020 12:10	192.4	191.2	186.8	2161	65.03
12/4/2020 12:10	192.4	191.6	187	2161	64.83
12/4/2020 12:10	192.3	190.8	187.7	2160	64.93
12/4/2020 12:10	192.3	190.8	187.9	2157	64.99
12/4/2020 12:10	192.3	191.5	187.6	2160	64.99
12/4/2020 12:10	192.2	191	186.8	2160	65.08
12/4/2020 12:10	192.2	191.3	186.8	2160	65.08
12/4/2020 12:10	192.2	191	187	2162	64.88
12/4/2020 12:10	192.2	191.4	187	2160	64.96
12/4/2020 12:10	192.2	190.9	187.4	2164	64.92
12/4/2020 12:10	192.1	190.7	187.3	2163	64.99
12/4/2020 12:10	192	191.4	187	2163	65.08
12/4/2020 12:10	192	190.9	187	2160	64.96
12/4/2020 12:10	192	191.4	187.2	2156	64.92
12/4/2020 12:10	191.9	191	187.4	2159	65
12/4/2020 12:10	191.9	190.9	187.9	2157	65
12/4/2020 12:10	191.8	191.3	187.7	2156	64.96
12/4/2020 12:10	191.9	191.2	187	2161	65.04
12/4/2020 12:10	191.8	191.2	186.9	2159	64.92
12/4/2020 12:10	191.8	191.2	186.8	2160	64.92
12/4/2020 12:10	191.8	190.7	186.8	2160	65.05
12/4/2020 12:10	191.7	191	187.2	2156	65.04
12/4/2020 12:10	191.6	190.7	187	2153	64.96
12/4/2020 12:10	191.6	191.1	187.2	2156	65
12/4/2020 12:10	191.6	191.1	186.9	2157	64.92
12/4/2020 12:10	191.5	190.9	186.7	2161	64.96
12/4/2020 12:10	191.5	190.9	186.9	2159	65.01
12/4/2020 12:10	191.4	191.3	187.5	2161	64.88
12/4/2020 12:10	191.5	191.4	187.9	2160	64.83
12/4/2020 12:10	191.4	191.2	187.6	2155	65.05
12/4/2020 12:10	191.3	190.8	187	2152	65.04
12/4/2020 12:10	191.3	190.9	186.7	2152	64.99
12/4/2020 12:10	191.3	190.8	186.5	2158	64.96
12/4/2020 12:10	191.2	191.1	186.8	2155	65.04
12/4/2020 12:10	191.2	191.5	187	2157	65.08
12/4/2020 12:10	191.1	190.9	187.1	2156	64.92
12/4/2020 12:10	191.1	190.8	187	2160	64.96
12/4/2020 12:10	191.1	190.7	187	2163	65.09
12/4/2020 12:10	191	190.7	187.2	2160	64.96
12/4/2020 12:10	191	190.4	187	2156	64.96
12/4/2020 12:10	190.9	189.7	187.2	2152	65
12/4/2020 12:10	190.9	190.2	187	2153	64.92
12/4/2020 12:10	190.9	189.8	187.1	2156	64.96
12/4/2020 12:10	190.9	189.7	187	2152	65

12/4/2020 12:10	190.8	190.2	187	2156	64.96
12/4/2020 12:11	190.8	189.9	187.1	2157	65.12
12/4/2020 12:11	190.7	190.4	186.9	2159	65.05
12/4/2020 12:11	190.7	190.3	186.8	2158	64.88
12/4/2020 12:11	190.7	189.6	186.8	2156	64.92
12/4/2020 12:11	190.6	190.2	186.7	2160	65.08
12/4/2020 12:11	190.6	190	187.3	2159	64.92
12/4/2020 12:11	190.6	190.3	187.9	2160	64.92
12/4/2020 12:11	190.6	190.2	187.4	2161	64.88
12/4/2020 12:11	190.5	189.8	186.8	2160	64.99
12/4/2020 12:11	190.5	190.3	187	2161	65.04
12/4/2020 12:11	190.5	189.8	186.6	2167	65.01
12/4/2020 12:11	190.4	190	187	2168	65.04
12/4/2020 12:11	190.4	190.3	186.7	2164	64.88
12/4/2020 12:11	190.3	189.6	186.7	2167	64.96
12/4/2020 12:11	190.3	189.8	187.5	2163	64.99
12/4/2020 12:11	190.3	189.7	187	2164	64.96
12/4/2020 12:11	190.2	190.1	186.7	2165	65.12
12/4/2020 12:11	190.1	190.3	186.9	2164	64.99
12/4/2020 12:11	190.1	190.2	186.8	2160	64.96
12/4/2020 12:11	190.1	190.2	186.8	2157	64.96
12/4/2020 12:11	190.1	189.8	186.6	2155	64.88
12/4/2020 12:11	190	190.1	186.7	2157	64.96
12/4/2020 12:11	190	190.3	187.3	2159	65.12
12/4/2020 12:11	190	189.6	187.6	2160	64.91
12/4/2020 12:11	189.9	190.5	187.1	2160	64.96
12/4/2020 12:11	189.9	190	186.7	2156	65.04
12/4/2020 12:11	189.8	189.6	186.8	2154	64.91
12/4/2020 12:11	189.8	189.8	186.8	2152	64.96
12/4/2020 12:11	189.8	190.1	186.8	2151	65.04
12/4/2020 12:11	189.8	190.4	186.6	2152	64.87
12/4/2020 12:11	189.7	189.8	186.7	2151	65
12/4/2020 12:11	189.7	189.6	187.3	2149	65.04
12/4/2020 12:11	189.7	190.3	187.5	2151	64.92
12/4/2020 12:11	189.6	189.9	187	2152	64.96
12/4/2020 12:11	189.6	190.1	186.7	2158	65.12
12/4/2020 12:11	189.6	190.2	186.7	2160	64.92
12/4/2020 12:11	189.6	189.8	187	2158	64.96
12/4/2020 12:11	189.5	190.3	186.7	2160	65.08
12/4/2020 12:11	189.5	189.8	186.8	2163	64.88
12/4/2020 12:11	189.5	190.1	186.6	2164	64.99
12/4/2020 12:11	189.4	190.2	186.6	2163	64.96
12/4/2020 12:11	189.4	189.8	186.7	2162	64.99
12/4/2020 12:11	189.3	189.8	186.5	2163	64.99
12/4/2020 12:11	189.4	189.6	186.5	2156	64.96
12/4/2020 12:11	189.3	190	186.6	2152	64.99
12/4/2020 12:11	189.3	189.7	186.4	2152	64.96

12/4/2020 12:11	189.3	190.2	186.6	2156	64.96
12/4/2020 12:11	189.2	190.2	186.5	2151	64.99
12/4/2020 12:11	189.2	189.8	186.3	2149	64.88
12/4/2020 12:11	189.2	190	186.5	2147	65.12
12/4/2020 12:11	189.2	189.7	186.4	2151	64.99
12/4/2020 12:11	189.2	189.7	186.5	2148	64.92
12/4/2020 12:11	189.1	190.3	186.6	2149	65.12
12/4/2020 12:11	189.1	190	186.5	2151	64.99
12/4/2020 12:11	189.1	190.1	186.5	2146	64.92
12/4/2020 12:11	189.1	189.8	186.3	2147	64.92
12/4/2020 12:11	189.2	190.2	186.3	2152	65.12
12/4/2020 12:11	189.1	190.4	186.6	2148	64.95
12/4/2020 12:11	189.1	190.1	186.5	2148	65.08
12/4/2020 12:12	189.2	190.1	186.5	2144	65.08
12/4/2020 12:12	189.2	190.2	186.7	2147	64.99
12/4/2020 12:12	189.2	189.8	186.4	2153	65.08
12/4/2020 12:12	189.2	190.2	186.6	2150	65.04
12/4/2020 12:12	189.2	190.2	186.8	2152	64.96
12/4/2020 12:12	189.2	190.3	186.9	2156	65
12/4/2020 12:12	189.3	189.6	186.8	2156	64.87
12/4/2020 12:12	189.2	189.8	186.8	2153	64.92
12/4/2020 12:12	189.2	190.5	186.8	2152	64.96
12/4/2020 12:12	189.3	189.6	186.8	2150	65.04
12/4/2020 12:12	189.3	190.3	186.7	2150	65
12/4/2020 12:12	189.3	190.3	186.7	2152	65.05
12/4/2020 12:12	189.3	189.8	186.7	2147	65
12/4/2020 12:12	189.4	190.1	186.5	2145	65
12/4/2020 12:12	189.4	189.8	187.1	2147	65
12/4/2020 12:12	189.4	189.8	187.4	2143	64.87
12/4/2020 12:12	189.4	189.8	187.4	2145	64.96
12/4/2020 12:12	189.4	189.6	187.2	2144	65.04
12/4/2020 12:12	189.4	190.2	186.5	2142	64.92
12/4/2020 12:12	189.5	189.8	186.5	2140	64.92
12/4/2020 12:12	189.5	189.7	186.7	2143	65
12/4/2020 12:12	189.6	190.3	186.7	2143	64.96
12/4/2020 12:12	189.6	189.7	186.7	2144	65
12/4/2020 12:12	189.6	190.2	186.8	2148	64.92
12/4/2020 12:12	189.6	189.6	186.7	2151	64.87
12/4/2020 12:12	189.6	190.3	186.8	2149	64.96
12/4/2020 12:12	189.6	190.4	186.7	2151	64.96
12/4/2020 12:12	189.6	189.8	186.4	2150	64.92
12/4/2020 12:12	189.7	190.2	186.7	2152	64.87
12/4/2020 12:12	189.7	190.4	187	2155	65.04
12/4/2020 12:12	189.7	189.8	187.5	2157	65.08
12/4/2020 12:12	189.8	190.2	187.3	2155	65.08
12/4/2020 12:12	189.8	190	186.4	2155	64.92
12/4/2020 12:12	189.8	190.4	186.7	2154	64.88

12/4/2020 12:12	189.8	190.2	186.7	2150	65
12/4/2020 12:12	189.8	189.9	186.7	2149	64.92
12/4/2020 12:12	189.9	190.4	186.9	2148	65.03
12/4/2020 12:12	189.9	189.8	186.5	2143	64.99
12/4/2020 12:12	189.9	190.3	186.6	2144	65.04
12/4/2020 12:12	190	190.2	187.2	2149	65.04
12/4/2020 12:12	190	189.7	187.3	2148	65.08
12/4/2020 12:12	190	190.4	186.7	2152	65.12
12/4/2020 12:12	190	190.1	186.6	2151	64.84
12/4/2020 12:12	190	190.4	186.5	2153	65.04
12/4/2020 12:12	190.1	190.1	186.8	2151	65.04
12/4/2020 12:12	190.1	189.6	186.7	2149	64.88
12/4/2020 12:12	190.2	190.3	186.6	2153	64.88
12/4/2020 12:12	190.3	190.1	186.7	2153	65.08
12/4/2020 12:12	190.2	190.3	186.5	2151	64.88
12/4/2020 12:12	190.3	190.1	186.6	2148	64.84
12/4/2020 12:12	190.3	189.6	186.8	2145	65.05
12/4/2020 12:12	190.4	190.3	186.9	2151	64.88
12/4/2020 12:12	190.5	190.1	187.2	2150	64.88
12/4/2020 12:12	190.5	190.5	187.5	2152	65.08
12/4/2020 12:12	190.4	190.3	187.4	2151	64.91
12/4/2020 12:12	190.4	189.6	187.4	2158	64.92
12/4/2020 12:12	190.5	190.3	186.9	2156	64.96
12/4/2020 12:12	190.6	189.9	187	2159	64.91
12/4/2020 12:12	190.6	190.4	186.5	2155	64.87
12/4/2020 12:12	190.7	190.1	186.3	2160	65.08
12/4/2020 12:13	190.6	189.6	186.4	2164	64.92
12/4/2020 12:13	190.7	190.3	186.3	2159	64.96
12/4/2020 12:13	190.7	190	186.2	2159	65.04
12/4/2020 12:13	190.7	190.3	186.5	2155	64.91
12/4/2020 12:13	190.7	189.8	186.5	2158	64.91
12/4/2020 12:13	190.7	189.6	186.5	2158	65.12
12/4/2020 12:13	190.8	190.3	186.5	2159	64.96
12/4/2020 12:13	190.9	190.2	186.4	2152	64.91
12/4/2020 12:13	190.8	189.6	186.5	2156	65.04
12/4/2020 12:13	190.8	190.4	186.5	2156	64.92
12/4/2020 12:13	190.8	189.6	186.3	2156	64.96
12/4/2020 12:13	190.9	190.3	186.7	2159	65.09
12/4/2020 12:13	190.9	190.1	187.1	2160	64.96
12/4/2020 12:13	190.9	189.8	187.4	2158	64.92
12/4/2020 12:13	191	189.9	187.3	2158	65.12
12/4/2020 12:13	190.9	189.8	186.8	2155	64.88
12/4/2020 12:13	191	190.2	186.5	2152	64.96
12/4/2020 12:13	191	189.9	186.5	2153	64.96
12/4/2020 12:13	191.1	190.5	186.8	2147	64.99
12/4/2020 12:13	191.1	189.9	187	2144	64.95
12/4/2020 12:13	191	189.6	186.8	2153	64.92

12/4/2020 12:13	191.1	190.1	186.5	2154	65.08
12/4/2020 12:13	191.1	189.7	186.6	2156	65.04
12/4/2020 12:13	191.1	190.3	187.2	2155	64.96
12/4/2020 12:13	191.2	190.5	187	2152	64.92
12/4/2020 12:13	191.1	189.9	186.6	2154	65.04
12/4/2020 12:13	191.2	190.2	186.7	2157	64.99
12/4/2020 12:13	191.3	190.1	186.9	2161	65.08
12/4/2020 12:13	191.2	189.9	186.7	2161	64.93
12/4/2020 12:13	191.1	190.4	186.3	2163	65.12
12/4/2020 12:13	191.2	189.6	186.7	2158	65.03
12/4/2020 12:13	191.1	190.5	187.5	2155	64.99
12/4/2020 12:13	191.1	190	186.9	2156	64.96
12/4/2020 12:13	191.1	189.6	186.6	2159	64.83
12/4/2020 12:13	191.1	189.8	186.8	2156	64.88
12/4/2020 12:13	191.1	190	187	2160	65.08
12/4/2020 12:13	191.1	190.2	186.9	2157	65.04
12/4/2020 12:13	191.1	190.2	186.5	2151	64.88
12/4/2020 12:13	191	190.3	186.5	2149	65
12/4/2020 12:13	191.1	190	186.7	2151	64.96
12/4/2020 12:13	191	189.7	187.4	2149	64.96
12/4/2020 12:13	191	190.1	187.2	2149	64.96
12/4/2020 12:13	191	190.2	186.6	2149	64.88
12/4/2020 12:13	190.9	190.4	186.6	2147	64.99
12/4/2020 12:13	190.9	190.1	186.8	2151	65.04
12/4/2020 12:13	190.9	189.8	186.7	2151	65
12/4/2020 12:13	190.9	190	186.7	2145	64.96
12/4/2020 12:13	190.9	189.6	186.8	2143	65
12/4/2020 12:13	191	189.8	186.7	2142	64.87
12/4/2020 12:13	190.9	190.3	186.9	2142	65
12/4/2020 12:13	190.9	189.8	186.5	2148	65.04
12/4/2020 12:13	190.9	190.1	186.7	2146	64.96
12/4/2020 12:13	190.9	189.6	186.4	2144	64.96
12/4/2020 12:13	191	189.6	186.3	2149	65.09
12/4/2020 12:13	191	190.4	186.5	2148	64.96
12/4/2020 12:13	191	189.9	186.2	2148	64.92
12/4/2020 12:13	191	189.8	186.3	2147	64.96
12/4/2020 12:13	191	190	186.5	2144	64.92
12/4/2020 12:13	191	189.6	186.5	2138	64.88
12/4/2020 12:14	191.1	190.3	186.6	2139	65.08
12/4/2020 12:14	191.1	189.9	186.9	2139	64.92
12/4/2020 12:14	191.1	190	186.8	2138	64.79
12/4/2020 12:14	191.1	190.3	187.1	2144	65.05
12/4/2020 12:14	191.1	190.3	187.3	2144	64.8
12/4/2020 12:14	191.2	190.3	187.5	2145	64.88
12/4/2020 12:14	191.2	189.8	187.5	2147	65.12
12/4/2020 12:14	191.3	190.5	187.4	2147	64.84
12/4/2020 12:14	191.3	189.7	187.4	2142	64.92

12/4/2020 12:14	191.3	190	187	2138	64.88
12/4/2020 12:14	191.4	190.3	186.8	2138	64.83
12/4/2020 12:14	191.4	189.7	186.6	2139	64.88
12/4/2020 12:14	191.6	190.3	186.8	2138	65.01
12/4/2020 12:14	191.6	189.7	186.7	2139	64.84
12/4/2020 12:14	191.7	189.6	186.8	2140	65.04
12/4/2020 12:14	191.6	190.3	187	2142	64.96
12/4/2020 12:14	191.8	190	186.9	2142	64.99
12/4/2020 12:14	191.8	190.3	186.8	2144	64.88
12/4/2020 12:14	191.8	190.1	186.5	2144	64.88
12/4/2020 12:14	191.9	189.6	186.7	2145	64.88
12/4/2020 12:14	191.9	190.1	186.8	2143	64.83
12/4/2020 12:14	192	189.9	187.4	2143	65
12/4/2020 12:14	192.1	190.3	187.6	2143	65.04
12/4/2020 12:14	192	190.3	187.4	2145	64.99
12/4/2020 12:14	192.1	189.6	187	2149	65.08
12/4/2020 12:14	192.2	190.2	186.7	2143	64.96
12/4/2020 12:14	192.2	189.6	186.7	2149	65
12/4/2020 12:14	192.2	190	186.8	2145	64.96
12/4/2020 12:14	192.3	190.3	186.9	2145	64.92
12/4/2020 12:14	192.3	190.3	186.9	2145	65
12/4/2020 12:14	192.4	190.2	187	2148	65.04
12/4/2020 12:14	192.4	189.6	187	2147	65
12/4/2020 12:14	192.6	189.9	187	2147	64.92
12/4/2020 12:14	192.6	190.3	187	2152	65.04
12/4/2020 12:14	192.6	190.2	187	2150	65
12/4/2020 12:14	192.8	190	186.8	2151	65
12/4/2020 12:14	192.9	189.7	187	2155	64.96
12/4/2020 12:14	192.9	190	187	2152	65
12/4/2020 12:14	192.9	190.5	187	2158	65.08
12/4/2020 12:14	193.1	189.5	187	2152	64.92
12/4/2020 12:14	193.1	190.1	186.8	2150	64.96
12/4/2020 12:14	193.2	190.2	186.8	2151	65.04
12/4/2020 12:14	193.3	189.8	187	2152	64.95
12/4/2020 12:14	193.3	190.3	186.9	2149	64.96
12/4/2020 12:14	193.5	189.9	187.1	2150	65.04
12/4/2020 12:14	193.5	190.3	187.1	2144	64.99
12/4/2020 12:14	193.5	190	187	2146	65.12
12/4/2020 12:14	193.7	190.1	187.2	2147	65.08
12/4/2020 12:14	193.7	190.1	187.2	2149	64.92
12/4/2020 12:14	193.7	190	187.2	2152	65.04
12/4/2020 12:14	193.8	190.3	187.3	2152	64.96
12/4/2020 12:14	193.8	190	187.1	2152	65
12/4/2020 12:14	193.9	189.6	186.8	2156	65.04
12/4/2020 12:14	193.9	190.1	187	2156	65.03
12/4/2020 12:14	193.9	190.1	187.2	2159	64.84
12/4/2020 12:14	194	190.3	187.6	2159	64.93

12/4/2020 12:14	194	189.7	187.6	2157	64.96
12/4/2020 12:14	193.9	189.6	187.9	2158	64.96
12/4/2020 12:14	194	190.2	188.1	2163	64.92
12/4/2020 12:14	194	189.8	187.7	2162	65
12/4/2020 12:15	194	190.3	187.4	2163	64.96
12/4/2020 12:15	194	189.8	187	2165	64.96
12/4/2020 12:15	193.9	190.3	186.9	2162	64.96
12/4/2020 12:15	194	189.8	187	2165	64.96
12/4/2020 12:15	193.9	189.8	186.9	2164	64.92
12/4/2020 12:15	193.9	190.4	186.8	2166	64.92
12/4/2020 12:15	193.8	189.7	186.9	2167	65.04
12/4/2020 12:15	193.8	190.2	186.9	2165	64.92
12/4/2020 12:15	193.8	190	187	2165	65.08
12/4/2020 12:15	193.8	189.6	187.2	2163	65.08
12/4/2020 12:15	193.7	189.8	187.8	2161	64.88
12/4/2020 12:15	193.8	189.7	188.1	2161	65.08
12/4/2020 12:15	193.6	190.3	187.4	2162	64.99
12/4/2020 12:15	193.6	189.9	186.9	2165	64.99
12/4/2020 12:15	193.6	190.3	187.1	2164	64.96
12/4/2020 12:15	193.4	190.3	187.1	2167	64.88
12/4/2020 12:15	193.5	189.5	187	2170	64.88
12/4/2020 12:15	193.4	190	186.8	2167	64.92
12/4/2020 12:15	193.4	189.6	187	2168	64.96
12/4/2020 12:15	193.3	189.6	187.7	2164	64.99
12/4/2020 12:15	193.2	190.4	187.7	2163	65.03
12/4/2020 12:15	193.1	189.6	186.9	2168	64.92
12/4/2020 12:15	193.1	190.2	187.1	2167	65.04
12/4/2020 12:15	193.1	190.3	187	2167	64.96
12/4/2020 12:15	192.9	190.1	187.3	2171	64.92
12/4/2020 12:15	192.8	190.3	186.9	2171	64.88
12/4/2020 12:15	192.8	190	186.8	2163	64.92
12/4/2020 12:15	192.7	190.3	187.5	2162	65.04
12/4/2020 12:15	192.7	189.6	187.9	2160	64.88
12/4/2020 12:15	192.5	190.3	187.6	2167	64.88
12/4/2020 12:15	192.4	190	187	2162	64.92
12/4/2020 12:15	192.4	189.8	187	2161	64.88
12/4/2020 12:15	192.4	190.1	187.2	2163	65.04
12/4/2020 12:15	192.3	189.8	187.1	2160	64.96
12/4/2020 12:15	192.3	190	187.2	2160	64.96
12/4/2020 12:15	192.2	189.9	187	2160	64.96
12/4/2020 12:15	192.1	189.6	186.8	2164	64.96
12/4/2020 12:15	192.1	189.9	186.7	2163	64.96
12/4/2020 12:15	192	190.3	187.4	2156	64.96
12/4/2020 12:15	192	189.6	187.7	2160	64.96
12/4/2020 12:15	191.9	190.2	187.9	2163	64.96
12/4/2020 12:15	191.8	189.5	187.5	2161	65
12/4/2020 12:15	191.9	190.2	187.1	2161	64.91

12/4/2020 12:15	191.8	190	187	2159	64.99
12/4/2020 12:15	191.8	189.5	187.2	2160	65.04
12/4/2020 12:15	191.7	190.3	187	2153	64.96
12/4/2020 12:15	191.6	189.8	187.2	2158	64.99
12/4/2020 12:15	191.7	190	187.3	2159	64.96
12/4/2020 12:15	191.6	189.6	187.2	2157	64.92
12/4/2020 12:15	191.6	190.1	187.3	2159	64.92
12/4/2020 12:15	191.5	190.4	187.2	2160	64.96
12/4/2020 12:15	191.4	189.8	187	2160	65.03
12/4/2020 12:15	191.4	189.8	187.3	2161	64.99
12/4/2020 12:15	191.4	190.4	187	2158	64.92
12/4/2020 12:15	191.4	189.6	187.2	2156	64.88
12/4/2020 12:15	191.4	190.2	187	2153	64.84
12/4/2020 12:15	191.4	190	187.2	2152	64.95
12/4/2020 12:15	191.3	190	187	2157	65.04
12/4/2020 12:15	191.2	190.2	187	2163	64.84
12/4/2020 12:16	191.3	189.7	187	2164	64.84
12/4/2020 12:16	191.3	190.1	187	2167	65.08
12/4/2020 12:16	191.3	189.9	187	2167	65.08
12/4/2020 12:16	191.3	190.4	187	2167	64.88
12/4/2020 12:16	191.2	190.3	187	2161	64.88
12/4/2020 12:16	191.1	189.8	186.7	2164	64.84
12/4/2020 12:16	191.2	190.1	187.1	2160	64.88
12/4/2020 12:16	191.2	189.8	187.7	2160	65.08
12/4/2020 12:16	191.2	190	187.2	2159	64.99
12/4/2020 12:16	191.1	190.1	186.7	2156	64.88
12/4/2020 12:16	191.1	189.6	187	2156	65
12/4/2020 12:16	191.1	190.3	187	2157	64.88
12/4/2020 12:16	191.1	190.1	187	2156	64.96
12/4/2020 12:16	191.1	189.8	187	2156	64.92
12/4/2020 12:16	191.1	190.3	187.7	2156	64.99
12/4/2020 12:16	191	190.3	186.7	2159	65
12/4/2020 12:16	191	190.1	187	2157	64.96
12/4/2020 12:16	190.9	189.6	187	2156	64.96
12/4/2020 12:16	190.9	189.8	187	2157	65
12/4/2020 12:16	190.8	190.4	186.8	2159	65
12/4/2020 12:16	190.8	190.2	187.1	2157	65.04
12/4/2020 12:16	190.7	190.3	187.8	2156	65.04
12/4/2020 12:16	190.7	190	186.9	2160	65
12/4/2020 12:16	190.7	189.7	186.9	2156	65
12/4/2020 12:16	190.6	189.8	187.2	2151	65.04
12/4/2020 12:16	190.5	190.1	187	2148	64.96
12/4/2020 12:16	190.5	190.1	186.8	2151	65.04
12/4/2020 12:16	190.5	190.1	186.7	2153	65.04
12/4/2020 12:16	190.5	190.4	187.1	2149	65.04
12/4/2020 12:16	190.3	189.8	187.6	2150	64.96
12/4/2020 12:16	190.3	189.8	187.4	2149	65.08

12/4/2020 12:16	190.3	190.3	186.7	2151	64.96
12/4/2020 12:16	190.3	190.1	186.5	2152	64.99
12/4/2020 12:16	190.3	189.7	186.7	2152	65.16
12/4/2020 12:16	190.3	190	186.7	2145	65.08
12/4/2020 12:16	190	189.6	187	2140	65.12
12/4/2020 12:16	190.3	190.1	187	2140	64.92
12/4/2020 12:16	190.3	189.8	186.9	2139	64.92
12/4/2020 12:16	190.2	189.6	186.9	2136	64.96
12/4/2020 12:16	190.2	190.3	187	2136	65
12/4/2020 12:16	190.2	189.8	186.9	2140	65
12/4/2020 12:16	190.3	190.1	187	2136	65
12/4/2020 12:16	190.3	189.6	186.9	2138	65
12/4/2020 12:16	190.3	190.3	186.8	2139	65
12/4/2020 12:16	190.3	190.4	186.9	2138	64.92
12/4/2020 12:16	190.2	189.8	186.5	2142	65.08
12/4/2020 12:16	190.3	190	187	2142	65.08
12/4/2020 12:16	190.3	190.2	187.5	2145	64.96
12/4/2020 12:16	190.3	189.6	187.3	2144	65.04
12/4/2020 12:16	190.4	190.5	186.8	2142	64.92
12/4/2020 12:16	190.5	189.9	186.7	2139	65.08
12/4/2020 12:16	190.5	189.9	186.8	2138	65.12
12/4/2020 12:16	190.6	190.1	187	2138	65.16
12/4/2020 12:16	190.5	189.7	186.6	2139	65.04
12/4/2020 12:16	190.6	189.8	186.7	2142	65.04
12/4/2020 12:16	190.7	189.9	187.2	2138	64.96
12/4/2020 12:16	190.7	190	187.6	2142	64.96
12/4/2020 12:16	190.8	190.3	186.9	2145	64.96
12/4/2020 12:16	190.8	189.7	186.7	2142	65
12/4/2020 12:17	190.9	190	187	2144	65
12/4/2020 12:17	190.9	189.7	186.7	2147	65.04
12/4/2020 12:17	190.9	190.4	186.9	2150	65.04
12/4/2020 12:17	191	190.2	186.8	2149	64.92
12/4/2020 12:17	191.1	189.7	186.8	2145	65.04
12/4/2020 12:17	191.1	190.4	186.8	2142	65.08
12/4/2020 12:17	191.1	190.1	187.6	2145	64.96
12/4/2020 12:17	191.2	190.1	187.6	2142	65.04
12/4/2020 12:17	191.3	190.1	186.8	2145	64.99
12/4/2020 12:17	191.4	189.6	186.8	2149	65.04
12/4/2020 12:17	191.4	190.4	186.8	2147	65.08
12/4/2020 12:17	191.5	189.6	186.8	2143	64.96
12/4/2020 12:17	191.5	189.8	187	2142	65.04
12/4/2020 12:17	191.5	189.7	186.8	2141	64.96
12/4/2020 12:17	191.6	190.1	186.9	2145	65.12
12/4/2020 12:17	191.6	190.3	186.7	2144	64.92
12/4/2020 12:17	191.8	189.7	187.3	2145	65
12/4/2020 12:17	191.8	190	187.7	2147	65
12/4/2020 12:17	191.7	190.5	187.8	2150	64.96

12/4/2020 12:17	191.8	189.7	187.2	2148	65
12/4/2020 12:17	191.8	190.2	186.8	2149	65
12/4/2020 12:17	191.8	190.2	186.7	2151	64.96
12/4/2020 12:17	191.9	190.2	186.9	2155	64.92
12/4/2020 12:17	191.9	190.3	187	2154	65.05
12/4/2020 12:17	191.9	190.2	187	2158	64.92
12/4/2020 12:17	192	190.4	187.3	2157	65.08
12/4/2020 12:17	192	190	187	2159	65.08
12/4/2020 12:17	192	189.7	186.3	2157	64.92
12/4/2020 12:17	192	190.3	187.2	2158	64.96
12/4/2020 12:17	192	190.1	187	2154	64.96
12/4/2020 12:17	192.1	190.2	187.2	2160	65.03
12/4/2020 12:17	192.1	190.1	187.2	2159	64.92
12/4/2020 12:17	192	189.7	187	2164	65.05
12/4/2020 12:17	192.1	190	187	2165	64.92
12/4/2020 12:17	192.1	189.8	186.8	2161	65
12/4/2020 12:17	192.1	190.3	186.8	2158	65.05
12/4/2020 12:17	192.2	190.2	186.7	2156	64.92
12/4/2020 12:17	192.1	189.6	186.7	2158	65.09
12/4/2020 12:17	192.2	189.9	186.9	2155	65.04
12/4/2020 12:17	192.2	190	186.8	2152	64.99
12/4/2020 12:17	192	190.2	187	2156	65.08
12/4/2020 12:17	192.1	190.2	187.7	2152	65.08
12/4/2020 12:17	192.1	190.3	187.7	2157	64.96
12/4/2020 12:17	192.1	190.3	187.6	2156	65.04
12/4/2020 12:17	192.1	189.8	187.1	2159	65.01
12/4/2020 12:17	192.1	190.4	186.8	2160	65.03
12/4/2020 12:17	192.2	190.2	186.8	2162	65.12
12/4/2020 12:17	192.1	189.7	187.2	2160	65.12
12/4/2020 12:17	192.1	190	187	2156	65
12/4/2020 12:17	192.1	189.6	187	2157	65.08
12/4/2020 12:17	192.1	190.1	186.8	2156	65.04
12/4/2020 12:17	192.1	190.3	187.1	2159	65
12/4/2020 12:17	192.1	189.7	187.8	2163	64.99
12/4/2020 12:17	192	190.2	187	2163	65.12
12/4/2020 12:17	192.1	189.7	186.9	2163	64.96
12/4/2020 12:17	192	190	187.2	2163	65
12/4/2020 12:17	192	189.7	187.1	2161	65.09
12/4/2020 12:17	192	189.8	186.8	2158	64.96
12/4/2020 12:17	192	190.3	186.7	2160	65.04
12/4/2020 12:17	192	190	186.9	2157	65.04
12/4/2020 12:18	192	189.8	187.7	2162	65
12/4/2020 12:18	191.9	189.7	187.4	2165	65.04
12/4/2020 12:18	191.9	189.5	186.8	2164	65.12
12/4/2020 12:18	191.9	190.2	187	2159	64.99
12/4/2020 12:18	191.9	190.1	186.9	2159	65.04
12/4/2020 12:18	191.9	190.3	187.2	2160	64.96

12/4/2020 12:18	191.8	190	187	2160	65.03
12/4/2020 12:18	191.8	189.6	186.6	2163	64.96
12/4/2020 12:18	191.8	190.2	186.8	2167	64.92
12/4/2020 12:18	191.8	189.8	187.6	2163	64.84
12/4/2020 12:18	191.8	189.7	187.5	2163	64.92
12/4/2020 12:18	191.8	190.2	187	2164	65.12
12/4/2020 12:18	191.7	189.7	187.1	2163	64.88
12/4/2020 12:18	191.6	189.9	187	2164	64.96
12/4/2020 12:18	191.6	190.1	187.1	2169	65.12
12/4/2020 12:18	191.6	189.9	186.8	2164	64.96
12/4/2020 12:18	191.6	190.4	186.7	2163	65.04
12/4/2020 12:18	191.6	190.2	187.2	2163	64.88
12/4/2020 12:18	191.5	190	187.7	2163	64.83
12/4/2020 12:18	191.5	189.5	187.4	2163	64.88
12/4/2020 12:18	191.4	189.7	186.8	2164	65.08
12/4/2020 12:18	191.4	189.8	187	2160	64.92
12/4/2020 12:18	191.4	189.8	187	2159	64.92
12/4/2020 12:18	191.3	190.3	187	2161	65.08
12/4/2020 12:18	191.3	190.1	186.9	2162	64.92
12/4/2020 12:18	191.3	189.8	186.8	2161	64.96
12/4/2020 12:18	191.3	190	186.7	2163	65
12/4/2020 12:18	191.3	190	186.8	2162	64.96
12/4/2020 12:18	191.2	190.1	187.4	2159	64.96
12/4/2020 12:18	191.1	190.1	187.6	2160	65
12/4/2020 12:18	191.1	189.5	187.7	2160	64.96
12/4/2020 12:18	191.1	189.8	187.3	2160	64.99
12/4/2020 12:18	191	189.9	187	2160	65.17
12/4/2020 12:18	191	190	186.9	2157	64.96
12/4/2020 12:18	191.1	190.3	187	2156	65.12
12/4/2020 12:18	190.9	190.3	187	2164	65.08
12/4/2020 12:18	190.9	190.1	187	2162	64.83
12/4/2020 12:18	190.9	189.6	187	2161	65.04
12/4/2020 12:18	190.8	189.8	187.2	2161	65.12
12/4/2020 12:18	190.8	190.3	187.2	2157	65.08
12/4/2020 12:18	190.8	190.2	187	2161	65.03
12/4/2020 12:18	190.8	190.2	187	2159	65.2
12/4/2020 12:18	190.6	189.7	186.8	2161	65.04
12/4/2020 12:18	190.6	189.8	186.8	2166	65.04
12/4/2020 12:18	190.6	190.3	186.8	2170	65.12
12/4/2020 12:18	190.6	190.2	186.7	2163	64.83
12/4/2020 12:18	190.5	190.4	186.7	2163	65.04
12/4/2020 12:18	190.5	189.6	186.6	2165	64.88
12/4/2020 12:18	190.4	189.8	186.6	2163	64.88
12/4/2020 12:18	190.5	190.3	186.8	2164	64.88
12/4/2020 12:18	190.3	189.8	186.7	2160	65
12/4/2020 12:18	190.3	190.2	186.7	2163	64.92
12/4/2020 12:18	190.3	189.6	186.7	2165	64.92

12/4/2020 12:18	190.3	190	186.6	2166	65.05
12/4/2020 12:18	190.3	190.4	186.7	2163	64.96
12/4/2020 12:18	190.1	190.2	186.7	2163	65.04
12/4/2020 12:18	190	189.8	186.8	2167	64.96
12/4/2020 12:18	189.9	189.6	186.8	2167	65
12/4/2020 12:18	190	189.8	186.9	2164	65.04
12/4/2020 12:19	189.9	190.4	186.8	2164	65.12
12/4/2020 12:19	189.8	190.2	187	2163	65
12/4/2020 12:19	189.8	190	187	2163	64.99
12/4/2020 12:19	189.8	190	186.8	2161	65.04
12/4/2020 12:19	189.7	189.7	187	2160	64.99
12/4/2020 12:19	189.7	190.3	186.7	2154	65.08
12/4/2020 12:19	189.7	190.2	186.8	2156	64.96
12/4/2020 12:19	189.6	190.5	186.7	2157	64.96
12/4/2020 12:19	189.6	190.3	186.8	2161	64.96
12/4/2020 12:19	189.5	190.3	187	2165	64.88
12/4/2020 12:19	189.5	190.2	187.2	2162	64.92
12/4/2020 12:19	189.4	189.7	187.2	2162	65.04
12/4/2020 12:19	189.3	189.9	187.7	2165	64.88
12/4/2020 12:19	189.3	189.5	187.4	2163	65
12/4/2020 12:19	189.2	189.6	187.5	2166	65.12
12/4/2020 12:19	189.2	190.3	187.2	2166	64.96
12/4/2020 12:19	189.2	189.8	187.2	2163	65.04
12/4/2020 12:19	189.1	189.9	186.8	2164	65
12/4/2020 12:19	189.1	190.1	186.5	2167	65
12/4/2020 12:19	189	189.6	186.6	2167	64.96
12/4/2020 12:19	189	190.3	186.8	2165	64.92
12/4/2020 12:19	189	189.8	186.6	2164	64.96
12/4/2020 12:19	189	190	186.8	2165	65
12/4/2020 12:19	188.9	190.3	186.8	2166	64.96
12/4/2020 12:19	188.9	189.8	186.4	2165	65
12/4/2020 12:19	188.8	190.3	186.5	2166	64.96
12/4/2020 12:19	188.8	189.8	186.8	2164	64.96
12/4/2020 12:19	188.8	189.9	187.5	2161	65.12
12/4/2020 12:19	188.7	189.6	186.8	2160	64.99
12/4/2020 12:19	188.7	190.2	186.5	2165	64.92
12/4/2020 12:19	188.7	190.3	187	2163	65.04
12/4/2020 12:19	188.6	189.9	186.8	2165	64.96
12/4/2020 12:19	188.5	190	186.7	2160	64.88
12/4/2020 12:19	188.5	190.3	186.6	2162	65
12/4/2020 12:19	188.5	190.2	186.2	2161	64.99
12/4/2020 12:19	188.4	190.2	187	2164	64.96
12/4/2020 12:19	188.3	189.7	187.3	2169	65.12
12/4/2020 12:19	188.3	189.9	186.3	2166	65.03
12/4/2020 12:19	188.3	190.3	186.5	2167	65.04
12/4/2020 12:19	188.2	190.1	186.6	2165	65.12
12/4/2020 12:19	188.1	190.3	186.9	2160	64.88

12/4/2020 12:19	188.2	189.8	186.8	2160	65.03
12/4/2020 12:19	188.1	189.6	186.6	2160	64.96
12/4/2020 12:19	188	190.4	186.2	2160	64.96
12/4/2020 12:19	188	190.2	186.2	2160	64.92
12/4/2020 12:19	187.9	190.3	186.5	2160	65
12/4/2020 12:19	187.9	189.9	187.2	2161	64.96
12/4/2020 12:19	187.9	189.6	187.4	2162	64.96
12/4/2020 12:19	187.8	189.1	187.2	2160	65.12
12/4/2020 12:19	187.8	188.8	186.8	2157	64.96
12/4/2020 12:19	187.8	189.2	186.6	2156	64.92
12/4/2020 12:19	187.7	189.1	186.2	2160	64.96
12/4/2020 12:19	187.7	188.9	186.4	2162	65
12/4/2020 12:19	187.7	189.2	186.4	2161	64.96
12/4/2020 12:19	187.6	189.1	186.5	2167	65.04
12/4/2020 12:19	187.6	189.3	186.7	2167	64.91
12/4/2020 12:19	187.5	189.1	186.5	2163	64.91
12/4/2020 12:19	187.4	189.3	186.7	2166	65
12/4/2020 12:19	187.4	188.9	186.5	2163	64.96
12/4/2020 12:19	187.4	188.8	186.5	2163	65.04
12/4/2020 12:20	187.4	189.2	186.5	2160	65.04
12/4/2020 12:20	187.4	188.9	186.7	2158	64.88
12/4/2020 12:20	187.3	188.9	186.5	2156	64.96
12/4/2020 12:20	187.2	189.1	186.7	2156	65.04
12/4/2020 12:20	187.2	188.9	186.2	2158	64.99
12/4/2020 12:20	187.2	189	186.7	2155	64.92
12/4/2020 12:20	187.1	188.9	186.7	2158	64.96
12/4/2020 12:20	187.1	188.9	186.2	2157	64.99
12/4/2020 12:20	187	189.3	186.5	2157	64.84
12/4/2020 12:20	187.1	188.9	186.5	2160	64.88
12/4/2020 12:20	187	189.1	186.5	2155	64.93
12/4/2020 12:20	187	188.8	186.4	2159	64.99
12/4/2020 12:20	186.9	188.8	186.2	2160	65.03
12/4/2020 12:20	187	189.3	186.3	2158	65.08
12/4/2020 12:20	186.9	188.9	186.1	2159	64.96
12/4/2020 12:20	186.8	189.2	186.3	2160	65
12/4/2020 12:20	186.7	189.3	186.3	2157	65.04
12/4/2020 12:20	186.8	188.8	186.3	2160	64.99
12/4/2020 12:20	186.6	188.8	186.2	2160	65.08
12/4/2020 12:20	186.6	188.9	186.4	2159	65.04
12/4/2020 12:20	186.6	188.8	186.2	2156	64.88
12/4/2020 12:20	186.6	189.6	186.1	2153	64.96
12/4/2020 12:20	186.6	189.1	186	2153	65
12/4/2020 12:20	186.5	189.2	186.2	2151	64.92
12/4/2020 12:20	186.5	188.9	186.3	2149	65
12/4/2020 12:20	186.6	188.9	186.3	2145	64.96
12/4/2020 12:20	186.5	188.6	186.4	2145	64.87
12/4/2020 12:20	186.5	188.9	186.2	2152	65.04

12/4/2020 12:20	186.5	189.1	186.3	2155	64.92
12/4/2020 12:20	186.5	188.8	186.2	2152	65
12/4/2020 12:20	186.4	188.8	186.3	2152	64.96
12/4/2020 12:20	186.4	189.2	186.2	2151	64.96
12/4/2020 12:20	186.5	188.8	186.1	2151	64.92
12/4/2020 12:20	186.5	189.2	186	2153	65.05
12/4/2020 12:20	186.5	189.1	185.7	2154	64.88
12/4/2020 12:20	186.5	189.3	185.9	2158	64.88
12/4/2020 12:20	186.4	188.8	186.2	2160	65
12/4/2020 12:20	186.4	188.9	186.7	2159	64.83
12/4/2020 12:20	186.5	189	187	2153	65
12/4/2020 12:20	186.5	189.1	186.3	2158	65.04
12/4/2020 12:20	186.5	189.1	186.3	2161	64.96
12/4/2020 12:20	186.5	189.1	186.1	2160	64.88
12/4/2020 12:20	186.5	188.8	186.1	2158	65.12
12/4/2020 12:20	186.5	188.9	186.5	2154	65.04
12/4/2020 12:20	186.6	188.8	186	2148	64.88
12/4/2020 12:20	186.6	189.3	186.1	2152	65.01
12/4/2020 12:20	186.6	189.3	186	2151	64.92
12/4/2020 12:20	186.6	188.9	185.9	2154	64.88
12/4/2020 12:20	186.7	189.1	186.6	2153	65.01
12/4/2020 12:20	186.7	188.7	186.7	2152	64.84
12/4/2020 12:20	186.7	188.9	186.2	2153	64.92
12/4/2020 12:20	186.7	189.3	186	2156	65.12
12/4/2020 12:20	186.7	188.9	186.1	2152	65
12/4/2020 12:20	186.7	189.1	186.1	2157	64.83
12/4/2020 12:20	186.8	188.7	186.1	2159	65.09
12/4/2020 12:20	186.8	188.8	186	2159	64.96
12/4/2020 12:20	186.8	189.3	186	2160	64.96
12/4/2020 12:20	186.8	188.9	186.1	2159	65.05
12/4/2020 12:20	186.8	189.3	185.8	2155	64.92
12/4/2020 12:21	186.8	189.1	185.9	2155	64.92
12/4/2020 12:21	186.8	188.9	186.5	2159	65
12/4/2020 12:21	186.8	189.2	186.7	2156	64.96
12/4/2020 12:21	186.9	189.1	186.3	2156	64.96
12/4/2020 12:21	186.9	189.3	186	2158	65
12/4/2020 12:21	187	188	185.8	2160	64.88
12/4/2020 12:21	187	188.2	186.3	2160	64.88
12/4/2020 12:21	187	188	186	2164	65
12/4/2020 12:21	187	188.2	186.3	2166	64.96
12/4/2020 12:21	187.1	188.1	185.8	2163	65
12/4/2020 12:21	187	188.2	185.7	2163	65.08
12/4/2020 12:21	187	188.1	185.8	2161	64.83
12/4/2020 12:21	187	188.1	186.5	2162	65.04
12/4/2020 12:21	187.2	188.1	186.9	2161	65.12
12/4/2020 12:21	187.2	188.1	186.5	2161	64.96
12/4/2020 12:21	187.1	188.1	185.9	2158	64.99

12/4/2020 12:21	187.2	188.2	185.6	2164	65.12
12/4/2020 12:21	187.3	188.1	186	2161	64.99
12/4/2020 12:21	187.2	188.2	186	2160	64.92
12/4/2020 12:21	187.2	188	186.1	2157	64.92
12/4/2020 12:21	187.3	188.1	185.8	2156	64.8
12/4/2020 12:21	187.3	188.1	185.8	2155	64.88
12/4/2020 12:21	187.4	188.3	185.8	2156	64.92
12/4/2020 12:21	187.4	188.1	186	2156	65.08
12/4/2020 12:21	187.4	188	186.4	2152	65.04
12/4/2020 12:21	187.4	188	186.7	2151	65.04
12/4/2020 12:21	187.5	188.2	186	2152	64.88
12/4/2020 12:21	187.5	188	186.2	2152	65.12
12/4/2020 12:21	187.5	188.1	186.1	2147	65.16
12/4/2020 12:21	187.6	188.1	185.9	2146	64.88
12/4/2020 12:21	187.6	188.2	185.8	2148	64.88
12/4/2020 12:21	187.6	188.2	185.5	2150	65.04
12/4/2020 12:21	187.6	188.1	186.3	2151	64.92
12/4/2020 12:21	187.7	188.2	186.8	2148	64.88
12/4/2020 12:21	187.7	188.1	186.1	2149	65.04
12/4/2020 12:21	187.7	188.2	185.8	2152	65
12/4/2020 12:21	187.7	188.1	185.8	2150	64.92
12/4/2020 12:21	187.7	188.1	186	2153	65
12/4/2020 12:21	187.8	187.9	186.1	2154	64.96
12/4/2020 12:21	187.7	188.2	185.9	2153	64.92
12/4/2020 12:21	187.7	188.1	185.7	2155	65
12/4/2020 12:21	187.7	188.1	185.7	2158	64.96
12/4/2020 12:21	187.8	188.1	185.8	2156	64.96
12/4/2020 12:21	187.7	188.1	186.4	2150	64.96
12/4/2020 12:21	187.7	188.1	186.8	2154	65
12/4/2020 12:21	187.7	188.1	186.3	2155	64.96
12/4/2020 12:21	187.8	188.2	186.2	2155	64.99
12/4/2020 12:21	187.7	188.2	185.6	2154	64.96
12/4/2020 12:21	187.7	188.1	186	2154	64.96
12/4/2020 12:21	187.7	188.1	185.9	2152	65.04
12/4/2020 12:21	187.7	188.1	185.9	2147	64.88
12/4/2020 12:21	187.7	188.1	185.9	2142	64.99
12/4/2020 12:21	187.7	188.1	185.8	2141	64.92
12/4/2020 12:21	187.8	188.2	185.6	2138	65
12/4/2020 12:21	187.7	188.2	185.7	2138	65.12
12/4/2020 12:21	187.8	188.1	185.8	2142	64.88
12/4/2020 12:21	187.8	188.1	186.6	2147	64.92
12/4/2020 12:21	187.8	188.2	186.7	2142	65.03
12/4/2020 12:21	187.8	188.2	185.9	2143	64.92
12/4/2020 12:21	187.8	188.1	185.8	2139	64.99
12/4/2020 12:22	187.9	188.2	186.1	2133	64.8
12/4/2020 12:22	187.9	188	186.1	2135	64.92
12/4/2020 12:22	187.9	188.1	185.9	2133	64.92

12/4/2020 12:22	188	188.4	185.5	2133	65.08
12/4/2020 12:22	187.9	188.1	185.8	2135	64.88
12/4/2020 12:22	188	188.1	186.7	2138	65.08
12/4/2020 12:22	188.1	188.1	186.3	2137	64.88
12/4/2020 12:22	188.1	188.1	185.8	2137	64.96
12/4/2020 12:22	188.2	188.2	186.1	2137	64.84
12/4/2020 12:22	188.2	188.2	186	2134	65.04
12/4/2020 12:22	188.3	187.9	186	2140	65
12/4/2020 12:22	188.3	188.1	186	2138	64.84
12/4/2020 12:22	188.3	188.2	185.7	2138	64.96
12/4/2020 12:22	188.5	188.2	185.8	2138	64.92
12/4/2020 12:22	188.5	188.1	185.7	2135	65.04
12/4/2020 12:22	188.6	188.1	186.3	2133	65
12/4/2020 12:22	188.6	188.1	186.8	2138	64.92
12/4/2020 12:22	188.7	188.1	186.1	2135	64.96
12/4/2020 12:22	188.7	188	185.8	2137	64.92
12/4/2020 12:22	188.8	188.1	186.1	2138	65
12/4/2020 12:22	189	188.2	186	2138	64.92
12/4/2020 12:22	189	188.1	186	2135	64.92
12/4/2020 12:22	189	188.1	186	2132	65.04
12/4/2020 12:22	189.2	188.1	186	2131	64.96
12/4/2020 12:22	189.2	188.2	185.8	2136	64.96
12/4/2020 12:22	189.3	188.1	185.7	2138	64.96
12/4/2020 12:22	189.4	188.1	185.8	2134	64.96
12/4/2020 12:22	189.5	188	185.6	2136	64.91
12/4/2020 12:22	189.6	188.1	185.9	2143	65.09
12/4/2020 12:22	189.6	188.1	186.6	2147	64.96
12/4/2020 12:22	189.7	188.1	186.6	2150	64.96
12/4/2020 12:22	189.8	188.2	186.3	2150	65.04
12/4/2020 12:22	189.8	188.1	186.2	2147	64.96
12/4/2020 12:22	189.9	188.1	185.8	2152	64.96
12/4/2020 12:22	190	188.1	185.6	2149	64.92
12/4/2020 12:22	190	188.1	185.8	2150	64.99
12/4/2020 12:22	190.1	188.1	185.8	2151	64.92
12/4/2020 12:22	190.2	188.1	186	2152	65.01
12/4/2020 12:22	190.3	188.1	186.3	2152	65.03
12/4/2020 12:22	190.3	188.1	186	2152	64.99
12/4/2020 12:22	190.3	188.2	186.2	2154	64.96
12/4/2020 12:22	190.4	188.1	186.3	2151	64.88
12/4/2020 12:22	190.5	188.1	186	2150	64.95
12/4/2020 12:22	190.5	188.2	186.2	2151	64.92
12/4/2020 12:22	190.6	188.1	185.9	2148	64.88
12/4/2020 12:22	190.5	188.2	185.8	2151	64.88
12/4/2020 12:22	190.5	188.3	185.6	2153	64.97
12/4/2020 12:22	190.6	188.1	185.6	2155	64.92
12/4/2020 12:22	190.6	188	185.7	2152	65.04
12/4/2020 12:22	190.6	188	186	2156	65.12

12/4/2020 12:22	190.6	188.1	186.7	2155	64.88
12/4/2020 12:22	190.6	187.9	186.7	2155	64.99
12/4/2020 12:22	190.6	188.2	186.9	2156	65.08
12/4/2020 12:22	190.7	188.1	187	2159	64.99
12/4/2020 12:22	190.6	188.1	186.7	2160	64.99
12/4/2020 12:22	190.6	188.1	186.2	2161	64.96
12/4/2020 12:22	190.6	188	186.1	2160	64.96
12/4/2020 12:22	190.6	188.1	186.1	2159	64.96
12/4/2020 12:22	190.6	188.1	185.9	2156	65.04
12/4/2020 12:23	190.5	188.1	186.2	2156	64.96
12/4/2020 12:23	190.6	188	186.3	2160	64.96
12/4/2020 12:23	190.6	188.1	186.1	2160	65.05
12/4/2020 12:23	190.5	188.1	186.3	2164	64.92
12/4/2020 12:23	190.5	188.1	186.2	2161	65.04
12/4/2020 12:23	190.4	188.3	186.2	2161	65.04
12/4/2020 12:23	190.4	188.1	186.2	2159	64.99
12/4/2020 12:23	190.5	188	186	2155	65
12/4/2020 12:23	190.2	188.2	186.1	2153	65.08
12/4/2020 12:23	190.2	188.1	186.1	2158	65.04
12/4/2020 12:23	190.2	188.2	186.1	2163	64.88
12/4/2020 12:23	190.1	188.2	186	2163	64.92
12/4/2020 12:23	190.1	188.2	185.9	2164	65.04
12/4/2020 12:23	190	188.2	186	2163	65.04
12/4/2020 12:23	190	188.3	186.1	2167	65.04
12/4/2020 12:23	190	188.2	186	2165	64.99
12/4/2020 12:23	189.8	188	186.1	2165	65.08
12/4/2020 12:23	189.8	188.1	186.2	2163	65.08
12/4/2020 12:23	189.8	188.1	186	2160	64.99
12/4/2020 12:23	189.7	188.1	186	2165	64.84
12/4/2020 12:23	189.7	188.1	186.1	2164	65
12/4/2020 12:23	189.6	188.1	186	2162	64.88
12/4/2020 12:23	189.6	188.1	186	2160	65.05
12/4/2020 12:23	189.5	188.2	185.9	2158	65.04
12/4/2020 12:23	189.5	188.1	186.1	2156	64.83
12/4/2020 12:23	189.4	188.2	186.1	2155	64.92
12/4/2020 12:23	189.3	188.1	186	2153	64.84
12/4/2020 12:23	189.3	188.1	186.1	2152	64.88
12/4/2020 12:23	189.3	188.1	186.1	2153	65.04
12/4/2020 12:23	189.3	188.1	186	2155	65.08
12/4/2020 12:23	189.3	188.1	186.1	2152	64.99
12/4/2020 12:23	189.2	188.2	186	2155	65.08
12/4/2020 12:23	189.2	188.2	185.8	2155	65.08
12/4/2020 12:23	189.2	188.2	185.6	2155	64.99
12/4/2020 12:23	189.1	188	185.5	2155	65.04
12/4/2020 12:23	189.1	188.1	185.7	2157	65
12/4/2020 12:23	189.1	188.1	186.4	2158	64.92
12/4/2020 12:23	189.2	188.1	186.5	2160	64.96

12/4/2020 12:23	189.2	188	186	2160	64.96
12/4/2020 12:23	189.1	188.1	185.8	2163	64.88
12/4/2020 12:23	189.1	188.1	186	2162	64.92
12/4/2020 12:23	189.1	188.2	186.1	2163	65.01
12/4/2020 12:23	189.1	188.2	185.9	2160	64.91
12/4/2020 12:23	189.1	188.1	185.8	2159	64.96
12/4/2020 12:23	189.1	188.1	186.5	2161	65.12
12/4/2020 12:23	189.1	188.1	186.4	2165	64.92
12/4/2020 12:23	189	188	185.9	2163	64.96
12/4/2020 12:23	189	188.2	185.9	2165	65.09
12/4/2020 12:23	189.1	188.1	186	2167	64.96
12/4/2020 12:23	189	188.1	186	2164	65
12/4/2020 12:23	189.1	188.2	185.6	2162	65.09
12/4/2020 12:23	189	188	185.4	2162	64.92
12/4/2020 12:23	189	188.2	186.2	2160	64.88
12/4/2020 12:23	189	188.1	186.5	2158	65.16
12/4/2020 12:23	189	188.1	185.7	2162	64.99
12/4/2020 12:23	189	188.1	185.8	2159	65.04
12/4/2020 12:23	188.9	188.1	185.8	2163	65.05
12/4/2020 12:23	189	188.1	185.9	2159	65.03
12/4/2020 12:23	188.9	188.2	185.6	2156	65.04
12/4/2020 12:24	188.9	188	185.5	2157	64.96
12/4/2020 12:24	189	188.1	185.6	2161	64.99
12/4/2020 12:24	189	188.1	185.6	2161	65.04
12/4/2020 12:24	188.9	188.1	186.5	2163	64.96
12/4/2020 12:24	188.9	188.1	186.5	2161	65.03
12/4/2020 12:24	188.9	188.1	186	2163	64.84
12/4/2020 12:24	188.8	188.1	185.9	2165	65.16
12/4/2020 12:24	188.8	188.1	185.9	2161	64.99
12/4/2020 12:24	188.7	188.1	185.7	2160	64.99
12/4/2020 12:24	188.7	188.1	186	2159	64.96
12/4/2020 12:24	188.7	188.1	185.9	2157	64.83
12/4/2020 12:24	188.7	188.1	185.5	2159	65
12/4/2020 12:24	188.7	188.2	186	2159	65.08
12/4/2020 12:24	188.7	188.1	186.5	2160	65.04
12/4/2020 12:24	188.7	188.1	186.5	2164	64.92
12/4/2020 12:24	188.6	188.1	186	2167	65.09
12/4/2020 12:24	188.6	188.1	185.6	2163	64.96
12/4/2020 12:24	188.5	188.1	186	2165	65
12/4/2020 12:24	188.5	188.1	185.8	2158	65.05
12/4/2020 12:24	188.5	188.2	185.9	2152	64.91
12/4/2020 12:24	188.5	188.1	185.8	2153	64.96
12/4/2020 12:24	188.5	188.1	185.4	2155	65
12/4/2020 12:24	188.4	188.1	185.6	2150	64.91
12/4/2020 12:24	188.4	188.1	185.8	2149	65.04
12/4/2020 12:24	188.3	188.1	186	2152	65.08
12/4/2020 12:24	188.3	188.2	186.3	2150	64.88

12/4/2020 12:24	188.3	187.9	186.3	2153	64.96
12/4/2020 12:24	188.3	188.1	185.6	2156	65.08
12/4/2020 12:24	188.2	188.1	185.8	2156	64.99
12/4/2020 12:24	188.2	188	185.6	2156	65.04
12/4/2020 12:24	188.2	187.9	185.4	2153	64.92
12/4/2020 12:24	188.2	188.1	185.8	2149	64.96
12/4/2020 12:24	188.2	188.2	185.7	2146	65.04
12/4/2020 12:24	188.2	188	185.6	2144	64.96
12/4/2020 12:24	188.1	188	185.6	2147	65.08
12/4/2020 12:24	188.1	188.2	185.8	2140	65
12/4/2020 12:24	188.1	188.1	185.7	2142	65.04
12/4/2020 12:24	188.2	188.1	186	2144	64.84
12/4/2020 12:24	188.2	188.2	185.6	2147	64.92
12/4/2020 12:24	188.2	188	185.8	2149	65.08
12/4/2020 12:24	188.1	188	185.5	2147	64.99
12/4/2020 12:24	188.1	188.1	185.6	2147	64.92
12/4/2020 12:24	188.2	188.1	185.8	2144	65.04
12/4/2020 12:24	188.2	187.9	185.6	2139	64.88
12/4/2020 12:24	188.2	188	185.6	2140	65.04
12/4/2020 12:24	188.3	188.1	185.6	2140	64.92
12/4/2020 12:24	188.3	188.1	185.6	2136	65.04
12/4/2020 12:24	188.3	188.1	185.8	2136	65
12/4/2020 12:24	188.3	187.9	185.7	2134	64.96
12/4/2020 12:24	188.3	188	185.3	2132	64.96
12/4/2020 12:24	188.4	188.1	185.6	2133	65
12/4/2020 12:24	188.5	188.1	185.2	2133	64.96
12/4/2020 12:24	188.5	187.9	185.4	2133	65.04
12/4/2020 12:24	188.5	188.1	185.7	2134	65
12/4/2020 12:24	188.5	188.1	186	2129	64.96
12/4/2020 12:24	188.6	188.1	186.3	2127	65.04
12/4/2020 12:24	188.7	188.1	186.2	2131	64.92
12/4/2020 12:24	188.7	188	186.3	2136	64.95
12/4/2020 12:24	188.8	188.1	186.4	2141	65.04
12/4/2020 12:24	188.9	188	186.4	2142	65.04
12/4/2020 12:25	188.8	188	186.1	2135	64.99
12/4/2020 12:25	188.9	188.1	186.5	2137	65.01
12/4/2020 12:25	189	188.1	186.3	2139	64.96
12/4/2020 12:25	189.1	188.1	186.1	2139	64.96
12/4/2020 12:25	189.2	188.1	185.9	2140	65.08
12/4/2020 12:25	189.3	188.1	185.3	2139	64.96
12/4/2020 12:25	189.4	188.1	185.4	2138	64.88
12/4/2020 12:25	189.4	187.9	185.8	2139	65.04
12/4/2020 12:25	189.5	188.1	185.7	2142	64.88
12/4/2020 12:25	189.5	188.1	185.8	2138	64.92
12/4/2020 12:25	189.6	188.2	185.5	2142	65.12
12/4/2020 12:25	189.8	187.9	185.5	2142	64.99
12/4/2020 12:25	189.8	188.1	186.5	2139	65.03

12/4/2020 12:25	190	188	185.8	2145	64.93
12/4/2020 12:25	190	188.1	185.4	2143	64.8
12/4/2020 12:25	190.1	187.9	185.9	2140	65.04
12/4/2020 12:25	190.3	188.1	185.6	2146	64.97
12/4/2020 12:25	190.3	188.2	185.5	2146	64.83
12/4/2020 12:25	190.4	188.1	185.3	2146	65.04
12/4/2020 12:25	190.5	188.2	185.6	2154	65.08
12/4/2020 12:25	190.5	188.1	186.5	2148	64.83
12/4/2020 12:25	190.7	188.1	186	2150	64.92
12/4/2020 12:25	190.7	188	185.6	2149	64.96
12/4/2020 12:25	190.8	188.1	185.6	2147	64.88
12/4/2020 12:25	190.9	188	185.8	2150	64.92
12/4/2020 12:25	190.9	188.1	185.5	2154	65.09
12/4/2020 12:25	191	188.1	185.8	2154	64.96
12/4/2020 12:25	191.1	188	185.4	2152	65.04
12/4/2020 12:25	191.1	188.1	185.7	2156	65.04
12/4/2020 12:25	191.3	188	186.4	2153	64.87
12/4/2020 12:25	191.2	188.1	186.2	2152	65
12/4/2020 12:25	191.3	188.1	185.9	2155	65.04
12/4/2020 12:25	191.4	187.9	185.5	2152	65
12/4/2020 12:25	191.4	188	185.8	2151	65.04
12/4/2020 12:25	191.5	188.1	185.8	2152	65.08
12/4/2020 12:25	191.4	188.1	185.8	2151	65.04
12/4/2020 12:25	191.6	188.1	185.8	2152	65
12/4/2020 12:25	191.5	188.1	185.6	2152	65.12
12/4/2020 12:25	191.6	188.1	185.4	2151	64.99
12/4/2020 12:25	191.6	188	185.5	2151	64.88
12/4/2020 12:25	191.6	188.1	186	2155	65.08
12/4/2020 12:25	191.7	188.9	186.2	2155	64.96
12/4/2020 12:25	191.7	189.1	186.5	2153	64.96
12/4/2020 12:25	191.7	189	186.6	2152	65.12
12/4/2020 12:25	191.8	188.8	186.5	2149	65.03
12/4/2020 12:25	191.8	188.9	186.6	2151	64.99
12/4/2020 12:25	191.8	188.9	186.3	2159	65.05
12/4/2020 12:25	191.8	189.1	186.3	2157	64.88
12/4/2020 12:25	191.8	188.9	186.5	2156	64.92
12/4/2020 12:25	191.8	188.9	186.3	2158	65.12
12/4/2020 12:25	191.8	189.1	186.6	2153	64.92
12/4/2020 12:25	192	188.6	186.7	2152	64.96
12/4/2020 12:25	191.9	189.1	186.5	2155	65
12/4/2020 12:25	191.9	189.1	186.1	2152	64.96
12/4/2020 12:25	192	189.3	185.8	2153	64.96
12/4/2020 12:25	191.9	188.9	185.6	2156	65.12
12/4/2020 12:25	191.9	188.7	185.7	2155	64.96
12/4/2020 12:25	191.9	188.9	185.6	2156	64.92
12/4/2020 12:25	191.9	188.7	185.9	2158	65
12/4/2020 12:26	191.9	189.3	185.8	2159	64.92

12/4/2020 12:26	191.9	189.2	185.6	2158	65
12/4/2020 12:26	191.9	188.6	185.9	2156	65.05
12/4/2020 12:26	191.9	189.1	185.9	2153	64.87
12/4/2020 12:26	191.9	188.9	186.5	2151	65
12/4/2020 12:26	192	188.7	186.4	2148	64.92
12/4/2020 12:26	191.9	188.8	185.4	2150	64.92
12/4/2020 12:26	191.9	188.7	185.5	2153	64.83
12/4/2020 12:26	191.9	189.3	185.9	2156	65.04
12/4/2020 12:26	191.9	188.8	185.8	2159	64.88
12/4/2020 12:26	191.9	189.1	186	2156	64.92
12/4/2020 12:26	191.9	189.2	185.8	2156	65.08
12/4/2020 12:26	191.8	188.9	185.8	2156	64.88
12/4/2020 12:26	191.8	189.1	186.6	2156	65.12
12/4/2020 12:26	191.8	188.6	186.5	2152	65.08
12/4/2020 12:26	191.8	188.9	186.2	2151	64.8
12/4/2020 12:26	191.8	189.2	185.9	2152	64.84
12/4/2020 12:26	191.8	189	185.5	2149	64.88
12/4/2020 12:26	191.8	188.9	185.8	2153	64.84
12/4/2020 12:26	191.8	189.2	186.1	2149	65.04
12/4/2020 12:26	191.8	188.9	186.2	2153	64.96
12/4/2020 12:26	191.8	189.3	186	2152	64.99
12/4/2020 12:26	191.8	188.9	186	2156	64.92
12/4/2020 12:26	191.8	188.8	185.7	2158	64.96
12/4/2020 12:26	191.8	188.8	185.9	2156	64.83
12/4/2020 12:26	191.8	189	186.1	2154	64.99
12/4/2020 12:26	191.8	189.1	186.3	2158	65.12
12/4/2020 12:26	191.7	189.3	186.9	2160	64.92
12/4/2020 12:26	191.7	188.9	186.6	2160	64.88
12/4/2020 12:26	191.6	188.8	186.7	2161	65.08
12/4/2020 12:26	191.7	188.9	186.2	2160	64.83
12/4/2020 12:26	191.6	189.2	186	2160	64.96
12/4/2020 12:26	191.6	188.9	186.1	2159	64.96
12/4/2020 12:26	191.6	189.3	185.8	2161	64.92
12/4/2020 12:26	191.6	189.3	185.8	2163	64.96
12/4/2020 12:26	191.6	189.1	186	2166	65
12/4/2020 12:26	191.4	189.1	186.2	2164	64.96
12/4/2020 12:26	191.5	188.9	186.2	2159	64.92
12/4/2020 12:26	191.5	189.2	186.6	2160	65.04
12/4/2020 12:26	191.4	188.8	186.6	2157	64.92
12/4/2020 12:26	191.4	188.8	186.5	2163	65
12/4/2020 12:26	191.3	189	186.1	2163	65
12/4/2020 12:26	191.4	188.8	186	2160	64.92
12/4/2020 12:26	191.3	189.2	186.1	2160	64.92
12/4/2020 12:26	191.3	189.3	186	2160	64.96
12/4/2020 12:26	191.3	189.1	185.9	2158	64.92
12/4/2020 12:26	191.5	188.9	185.8	2157	64.99
12/4/2020 12:26	191.3	188.7	185.4	2160	64.96

12/4/2020 12:26	191.2	189.2	185.8	2158	64.92
12/4/2020 12:26	191.1	189.1	186.5	2158	64.99
12/4/2020 12:26	191.1	188.8	186.2	2160	65.12
12/4/2020 12:26	191.1	189	185.8	2159	64.99
12/4/2020 12:26	191.1	189.2	186.1	2157	65.08
12/4/2020 12:26	191	188.9	185.8	2167	65.08
12/4/2020 12:26	191.1	189.3	185.8	2167	65.03
12/4/2020 12:26	191	188.7	185.5	2165	65.04
12/4/2020 12:26	191	189.1	186	2163	64.92
12/4/2020 12:26	190.9	188.9	186.7	2166	65.04
12/4/2020 12:26	190.9	188.9	186.1	2162	64.88
12/4/2020 12:26	190.9	189.3	185.5	2163	65.21
12/4/2020 12:27	190.9	189	186.1	2164	64.88
12/4/2020 12:27	190.8	189.2	185.8	2160	65
12/4/2020 12:27	190.9	188.9	185.6	2163	65.01
12/4/2020 12:27	190.8	189.1	185.4	2160	64.99
12/4/2020 12:27	190.7	189.3	186.2	2160	65
12/4/2020 12:27	190.8	188.8	186.4	2163	65.08
12/4/2020 12:27	190.6	189.1	185.6	2161	64.92
12/4/2020 12:27	190.6	188.7	185.8	2163	64.92
12/4/2020 12:27	190.6	189.3	185.8	2166	65.05
12/4/2020 12:27	190.5	189.3	185.7	2162	64.92
12/4/2020 12:27	190.5	188.7	185.5	2169	65
12/4/2020 12:27	190.5	188.8	185.6	2169	65.04
12/4/2020 12:27	190.5	189	186.2	2165	64.91
12/4/2020 12:27	190.5	189.1	186.5	2165	65
12/4/2020 12:27	190.4	189.1	185.9	2164	65
12/4/2020 12:27	190.4	188.8	185.6	2164	64.96
12/4/2020 12:27	190.3	189.1	185.7	2160	64.88
12/4/2020 12:27	190.3	188.9	185.4	2165	65.04
12/4/2020 12:27	190.3	188.9	185.9	2167	64.92
12/4/2020 12:27	190.2	189.1	185.8	2163	64.92
12/4/2020 12:27	190.2	188.8	185.4	2166	65.17
12/4/2020 12:27	190.1	189.1	185.6	2165	64.88
12/4/2020 12:27	190.1	188.8	185.7	2167	65.12
12/4/2020 12:27	190.1	189.2	186.3	2167	64.93
12/4/2020 12:27	190	189.3	186.6	2166	64.88
12/4/2020 12:27	190	188.9	186.4	2168	64.99
12/4/2020 12:27	190	188.7	186.1	2162	65.08
12/4/2020 12:27	189.9	189	186	2167	65.04
12/4/2020 12:27	189.8	189.2	185.6	2163	65.08
12/4/2020 12:27	189.8	189.2	185.6	2165	65.05
12/4/2020 12:27	189.8	188.8	185.9	2163	64.99
12/4/2020 12:27	189.7	188.7	185.6	2163	65
12/4/2020 12:27	189.7	189.2	185.8	2167	64.96
12/4/2020 12:27	189.6	188.8	185.8	2171	64.99
12/4/2020 12:27	189.6	189.3	185.5	2169	65.04

12/4/2020 12:27	189.7	189.1	185.6	2169	65.05
12/4/2020 12:27	189.6	188.9	185.8	2173	65
12/4/2020 12:27	189.5	188.9	185.7	2170	64.91
12/4/2020 12:27	189.5	188.8	186	2168	65.09
12/4/2020 12:27	189.5	189.1	185.5	2167	64.96
12/4/2020 12:27	189.5	188.8	185.8	2167	64.96
12/4/2020 12:27	189.4	189.3	185.5	2170	65.09
12/4/2020 12:27	189.4	189.3	185.5	2168	64.96
12/4/2020 12:27	189.3	188.9	185.8	2167	64.88
12/4/2020 12:27	189.3	189.1	185.6	2168	65.09
12/4/2020 12:27	189.3	188.8	185.5	2171	65.04
12/4/2020 12:27	189.2	189.1	185.8	2171	65.04
12/4/2020 12:27	189.1	189	186.2	2169	65.04
12/4/2020 12:27	189.2	188.8	186.1	2167	65.12
12/4/2020 12:27	189.1	189.1	185.5	2162	65.08
12/4/2020 12:27	189.1	189	185.4	2163	65.08
12/4/2020 12:27	189.1	189.3	185.8	2164	64.92
12/4/2020 12:27	189	189.3	185.8	2165	64.92
12/4/2020 12:27	189	188.8	185.6	2165	64.96
12/4/2020 12:27	189	189.2	186.4	2164	65.04
12/4/2020 12:27	188.9	188.7	185.3	2163	64.92
12/4/2020 12:27	189	188.8	185.4	2165	65.04
12/4/2020 12:27	188.9	189.2	185.8	2163	64.92
12/4/2020 12:27	188.9	188.8	185.4	2164	64.96
12/4/2020 12:28	188.8	189.1	185.4	2163	65.04
12/4/2020 12:28	188.7	189	186.3	2166	64.92
12/4/2020 12:28	188.8	189.2	186.2	2164	64.96
12/4/2020 12:28	188.7	189.3	185.4	2163	65
12/4/2020 12:28	188.7	188.9	185.1	2163	65
12/4/2020 12:28	188.7	189	185.5	2167	64.96
12/4/2020 12:28	188.7	189.2	185.8	2167	64.96
12/4/2020 12:28	188.7	189.1	185.2	2168	65.04
12/4/2020 12:28	188.5	189.1	185.4	2167	64.92
12/4/2020 12:28	188.5	188.9	186.2	2163	64.96
12/4/2020 12:28	188.5	189.1	185.8	2160	64.92
12/4/2020 12:28	188.5	188.9	185.2	2164	65.04
12/4/2020 12:28	188.5	188.6	185.6	2167	64.92
12/4/2020 12:28	188.4	188.9	185.7	2165	64.88
12/4/2020 12:28	188.5	189	185.6	2160	65.08
12/4/2020 12:28	188.3	189.1	185.3	2160	64.92
12/4/2020 12:28	188.3	189.1	185.8	2160	64.96
12/4/2020 12:28	188.3	188.6	186.3	2158	64.96
12/4/2020 12:28	188.2	188.8	185.3	2160	64.88
12/4/2020 12:28	188.3	188.9	185	2160	65
12/4/2020 12:28	188.1	189.1	185.7	2161	64.92
12/4/2020 12:28	188.2	189.3	185.5	2159	64.92
12/4/2020 12:28	188.2	189.1	185.6	2160	64.92

12/4/2020 12:28	188.1	189	185.4	2159	65
12/4/2020 12:28	188.1	188.9	185.9	2160	65
12/4/2020 12:28	188	188.8	186.1	2163	65.04
12/4/2020 12:28	187.9	189.3	185.6	2163	65
12/4/2020 12:28	188	189.3	185.4	2163	64.92
12/4/2020 12:28	187.9	189.1	185.3	2163	65.04
12/4/2020 12:28	187.9	188.9	185.3	2161	65.08
12/4/2020 12:28	187.9	188.8	185.6	2162	64.92
12/4/2020 12:28	187.8	189.3	185.5	2161	64.92
12/4/2020 12:28	187.8	189.1	185.3	2165	65
12/4/2020 12:28	187.7	189.2	185.3	2164	64.99
12/4/2020 12:28	187.7	188.9	185.5	2164	65
12/4/2020 12:28	187.7	189.1	185.9	2165	64.93
12/4/2020 12:28	187.7	189	186.1	2167	65.08
12/4/2020 12:28	187.6	188.7	185.8	2163	64.96
12/4/2020 12:28	187.6	189.2	185.5	2163	65.08
12/4/2020 12:28	187.5	189.1	185.2	2163	65.03
12/4/2020 12:28	187.5	189.1	185.1	2163	64.92
12/4/2020 12:28	187.5	189	185.2	2159	65.04
12/4/2020 12:28	187.5	188.9	185.1	2160	64.84
12/4/2020 12:28	187.4	189.1	185.1	2159	64.88
12/4/2020 12:28	187.4	189	185.5	2160	65.08
12/4/2020 12:28	187.4	189.2	185.1	2159	64.96
12/4/2020 12:28	187.4	189.1	185.1	2160	65.08
12/4/2020 12:28	187.3	188.1	185.2	2162	64.96
12/4/2020 12:28	187.3	188.1	185	2162	64.91
12/4/2020 12:28	187.3	188.1	185.4	2156	64.92
12/4/2020 12:28	187.2	188.2	185.7	2155	65
12/4/2020 12:28	187.2	188.1	185.8	2157	65
12/4/2020 12:28	187.2	188	185.5	2157	64.87
12/4/2020 12:28	187.2	188	185.2	2160	64.96
12/4/2020 12:28	187.2	188.1	185.4	2156	65
12/4/2020 12:28	187.2	188.1	185.2	2157	65.04
12/4/2020 12:28	187.2	188.1	184.9	2160	65.04
12/4/2020 12:28	187.2	188	185.1	2156	64.99
12/4/2020 12:28	187.1	188.1	186.1	2159	65
12/4/2020 12:29	187.2	188.1	185.5	2156	65.08
12/4/2020 12:29	187.1	188	185	2156	65
12/4/2020 12:29	187.2	188.2	185.4	2159	64.99
12/4/2020 12:29	187.2	188.1	185.5	2162	65.04
12/4/2020 12:29	187.1	188.1	185.1	2156	64.99
12/4/2020 12:29	187.2	188.2	184.9	2150	64.84
12/4/2020 12:29	187.2	188.3	185.5	2149	65.21
12/4/2020 12:29	187.1	188.1	185.8	2153	65.03
12/4/2020 12:29	187.1	187.9	185.1	2159	65.08
12/4/2020 12:29	187.2	188.1	185.1	2156	65.08
12/4/2020 12:29	187.2	188.2	185.4	2156	64.91

12/4/2020 12:29	187.2	188.1	185.3	2155	65.04
12/4/2020 12:29	187.2	188.1	185.2	2156	65.13
12/4/2020 12:29	187.2	188.2	184.9	2153	65
12/4/2020 12:29	187.2	188.3	184.8	2155	64.96
12/4/2020 12:29	187.3	188.2	185.9	2155	65.09
12/4/2020 12:29	187.3	188.1	185.6	2153	65
12/4/2020 12:29	187.2	188.1	185.3	2156	65.04
12/4/2020 12:29	187.3	188.2	185.4	2155	65.04
12/4/2020 12:29	187.3	188.2	185.1	2157	64.87
12/4/2020 12:29	187.3	188.1	185.4	2157	65
12/4/2020 12:29	187.4	188.2	184.8	2160	65.09
12/4/2020 12:29	187.4	188.1	184.7	2156	64.96
12/4/2020 12:29	187.4	188.2	185.2	2156	64.88
12/4/2020 12:29	187.4	188.1	185.7	2159	65.17
12/4/2020 12:29	187.4	188.1	185.6	2156	65.08
12/4/2020 12:29	187.5	188.2	185.4	2154	65.04
12/4/2020 12:29	187.5	188.1	184.9	2159	65.08
12/4/2020 12:29	187.5	188.1	184.9	2153	64.99
12/4/2020 12:29	187.5	188.2	185.3	2157	64.88
12/4/2020 12:29	187.5	188.2	185.3	2156	65.12
12/4/2020 12:29	187.6	187.9	185.3	2156	64.92
12/4/2020 12:29	187.6	188.1	185.1	2157	64.88
12/4/2020 12:29	187.7	188.1	184.6	2163	65.08
12/4/2020 12:29	187.7	188.1	185	2165	64.88
12/4/2020 12:29	187.7	188.1	185.6	2161	65.04
12/4/2020 12:29	187.8	188.1	185.8	2161	65.12
12/4/2020 12:29	187.8	188.1	185.3	2163	64.83
12/4/2020 12:29	187.9	188.1	184.8	2165	64.92
12/4/2020 12:29	187.9	188.1	185.1	2163	65
12/4/2020 12:29	187.9	188.1	185.3	2156	64.96
12/4/2020 12:29	187.9	188.1	185.1	2156	64.96
12/4/2020 12:29	188.1	188.1	185.2	2160	65.04
12/4/2020 12:29	188	188.1	184.8	2164	64.96
12/4/2020 12:29	188	188.1	185	2163	65
12/4/2020 12:29	188.1	188	184.8	2163	65.04
12/4/2020 12:29	188.1	188.1	184.6	2163	65
12/4/2020 12:29	188.1	187.9	184.6	2159	64.96
12/4/2020 12:29	188.2	188.1	185	2162	65
12/4/2020 12:29	188.1	188.2	184.8	2160	64.88
12/4/2020 12:29	188.2	188.1	185	2158	65.04
12/4/2020 12:29	188.2	188.1	185.1	2162	64.92
12/4/2020 12:29	188.3	188.1	185	2160	65
12/4/2020 12:29	188.3	188.1	185.1	2160	64.92
12/4/2020 12:29	188.3	188.1	184.9	2165	65.04
12/4/2020 12:29	188.4	188.1	184.9	2161	65.04
12/4/2020 12:29	188.4	188.1	184.9	2160	64.84
12/4/2020 12:29	188.3	188.1	184.8	2160	65.08

12/4/2020 12:29	188.4	188.3	185.4	2161	64.99
12/4/2020 12:29	188.4	188.1	185.2	2157	64.99
12/4/2020 12:30	188.5	188	185.4	2162	65.04
12/4/2020 12:30	188.5	188.1	185.7	2163	65
12/4/2020 12:30	188.5	188.1	185.6	2160	64.83
12/4/2020 12:30	188.5	188	185.9	2164	65.04
12/4/2020 12:30	188.6	188.1	186.1	2163	64.92
12/4/2020 12:30	188.5	188.1	185.8	2167	64.92
12/4/2020 12:30	188.6	188.2	185.9	2168	64.96
12/4/2020 12:30	188.6	188.1	185.6	2164	64.96
12/4/2020 12:30	188.7	188.2	185.3	2166	65
12/4/2020 12:30	188.7	188.2	185.1	2167	64.96
12/4/2020 12:30	188.7	188.1	185.3	2166	65
12/4/2020 12:30	188.8	188.1	185.3	2163	65
12/4/2020 12:30	188.7	188.1	185.4	2161	64.92
12/4/2020 12:30	188.7	188.1	185.3	2160	64.96
12/4/2020 12:30	188.8	188.1	185.4	2160	65
12/4/2020 12:30	188.8	188.1	185.4	2163	64.96
12/4/2020 12:30	189.1	188.1	185	2162	64.96
12/4/2020 12:30	188.9	188.1	185.1	2159	64.88
12/4/2020 12:30	188.9	187.9	185.2	2156	65.04
12/4/2020 12:30	188.9	188.2	185.3	2158	64.96
12/4/2020 12:30	188.9	188	186.1	2160	65.12
12/4/2020 12:30	188.9	188.2	185.4	2158	64.84
12/4/2020 12:30	189	188	185.3	2162	65.08
12/4/2020 12:30	189	188.1	185.5	2161	64.88
12/4/2020 12:30	189.1	188.2	185.2	2160	65.08
12/4/2020 12:30	189	188.1	184.9	2163	64.92
12/4/2020 12:30	189.1	188.2	184.8	2162	64.92
12/4/2020 12:30	189.1	188	185.8	2160	64.92
12/4/2020 12:30	189.1	188.1	185.8	2157	64.92
12/4/2020 12:30	189.1	188.1	185.1	2156	64.96
12/4/2020 12:30	189.2	188.2	185.3	2158	65.04
12/4/2020 12:30	189.2	188.1	185.3	2159	65.08
12/4/2020 12:30	189.3	188.1	185.2	2156	65
12/4/2020 12:30	189.2	188.2	185	2154	64.92
12/4/2020 12:30	189.2	188.1	185.3	2147	65
12/4/2020 12:30	189.3	188.1	185.8	2152	65.04
12/4/2020 12:30	189.3	187.9	185.4	2156	64.96
12/4/2020 12:30	189.2	188.1	184.9	2155	64.96
12/4/2020 12:30	189.3	188.2	185.2	2158	65
12/4/2020 12:30	189.3	188.2	185.5	2161	65
12/4/2020 12:30	189.3	188.1	185.4	2160	65.04
12/4/2020 12:30	189.3	188.1	185	2165	65
12/4/2020 12:30	189.3	188.1	185.1	2166	65
12/4/2020 12:30	189.3	188.1	185.5	2167	65.08
12/4/2020 12:30	189.3	188.1	186	2167	65.08

12/4/2020 12:30	189.3	188.1	185.6	2163	65.03
12/4/2020 12:30	189.2	188.2	184.9	2154	64.88
12/4/2020 12:30	189.2	188.1	185.4	2156	64.92
12/4/2020 12:30	189.3	188.1	185.4	2155	65.08
12/4/2020 12:30	189.3	188.2	185.3	2156	65.08
12/4/2020 12:30	189.3	188.2	185.1	2153	65.08
12/4/2020 12:30	189.3	188.1	185.2	2151	64.88
12/4/2020 12:30	189.3	188.1	185.1	2148	65.12
12/4/2020 12:30	189.3	188.2	186	2145	65.04
12/4/2020 12:30	189.4	188.2	185.8	2146	64.96
12/4/2020 12:30	189.4	188.1	185.4	2144	64.88
12/4/2020 12:30	189.4	188.1	185.3	2145	64.96
12/4/2020 12:30	189.3	188.2	185.2	2146	65.04
12/4/2020 12:30	189.4	188.2	185.5	2147	65.04
12/4/2020 12:31	189.4	188.1	185.3	2149	64.92
12/4/2020 12:31	189.4	188.1	185.3	2150	65
12/4/2020 12:31	189.5	188.1	185.1	2148	64.96
12/4/2020 12:31	189.5	188.1	185	2145	64.96
12/4/2020 12:31	189.6	188.1	185	2146	64.96
12/4/2020 12:31	189.6	188.1	185.8	2142	65
12/4/2020 12:31	189.6	188.1	186	2146	65.09
12/4/2020 12:31	189.6	188.1	185.9	2142	65
12/4/2020 12:31	189.6	188.1	185.5	2141	64.96
12/4/2020 12:31	189.7	188.1	185.3	2140	65
12/4/2020 12:31	189.8	188.1	185.5	2142	64.91
12/4/2020 12:31	189.8	188.1	185.7	2144	64.92
12/4/2020 12:31	189.8	188.1	185.4	2147	65.04
12/4/2020 12:31	189.8	188.1	185.4	2149	64.96
12/4/2020 12:31	189.9	188.1	185.2	2150	65.08
12/4/2020 12:31	189.9	188.2	185.2	2145	64.99
12/4/2020 12:31	190.1	187.8	186.1	2148	64.88
12/4/2020 12:31	190	187.9	185.8	2146	65
12/4/2020 12:31	190.1	188.2	185.1	2146	65.08
12/4/2020 12:31	190.2	188.1	185.3	2147	65.08
12/4/2020 12:31	190.2	188.1	185.5	2146	65.04
12/4/2020 12:31	190.3	188.1	185.5	2142	65.08
12/4/2020 12:31	190.4	187.9	185.1	2143	64.99
12/4/2020 12:31	190.3	188.3	185	2147	65.04
12/4/2020 12:31	190.5	188.1	185.8	2149	65.04
12/4/2020 12:31	190.5	188.2	186	2150	64.84
12/4/2020 12:31	190.6	188.1	185.2	2151	64.99
12/4/2020 12:31	190.6	188.2	185.5	2149	64.88
12/4/2020 12:31	190.7	188.2	185.4	2149	65.08
12/4/2020 12:31	190.8	188.2	185.5	2147	64.88
12/4/2020 12:31	190.8	188.2	185.1	2150	65.08
12/4/2020 12:31	190.9	188.2	185.2	2145	64.88
12/4/2020 12:31	190.9	188.1	185.8	2144	64.96

12/4/2020 12:31	191	188.1	186.3	2142	64.99
12/4/2020 12:31	191.1	188.1	186.1	2144	64.96
12/4/2020 12:31	191.1	188.1	185.4	2144	64.96
12/4/2020 12:31	191.2	188.4	185.5	2148	65.04
12/4/2020 12:31	191.3	188.1	185.5	2151	65
12/4/2020 12:31	191.3	188	185.8	2148	64.96
12/4/2020 12:31	191.5	188.1	185.4	2149	65.09
12/4/2020 12:31	191.5	188.1	185.3	2145	64.96
12/4/2020 12:31	191.6	188.1	185.4	2146	65
12/4/2020 12:31	191.7	188.1	185.7	2151	64.96
12/4/2020 12:31	191.7	188.1	186.3	2154	64.96
12/4/2020 12:31	191.8	188.1	186	2156	65
12/4/2020 12:31	191.8	188.3	185.2	2156	64.96
12/4/2020 12:31	191.8	188.2	185.8	2158	65
12/4/2020 12:31	192	188	185.6	2157	64.83
12/4/2020 12:31	192	188.6	185.7	2160	64.96
12/4/2020 12:31	192	189.3	185.6	2160	64.92
12/4/2020 12:31	192.1	189	185.3	2160	64.96
12/4/2020 12:31	192.1	189.1	186	2165	64.96
12/4/2020 12:31	192.2	188.7	186.4	2167	64.84
12/4/2020 12:31	192.2	188.8	185.5	2169	64.92
12/4/2020 12:31	192.3	189.2	185.5	2171	64.96
12/4/2020 12:31	192.2	188.8	185.8	2170	64.88
12/4/2020 12:31	192.2	188.7	185.6	2163	64.99
12/4/2020 12:31	192.2	189.1	185.7	2165	64.96
12/4/2020 12:31	192.2	188.8	185.4	2167	64.96
12/4/2020 12:31	192.2	188.8	186	2165	64.92
12/4/2020 12:32	192.3	189	186.5	2168	64.96
12/4/2020 12:32	192.3	188.8	186.3	2165	64.99
12/4/2020 12:32	192.3	188.9	185.9	2162	65.04
12/4/2020 12:32	192.3	188.8	185.6	2166	64.96
12/4/2020 12:32	192.3	189.1	185.5	2163	64.99
12/4/2020 12:32	192.3	188.9	185.9	2163	64.96
12/4/2020 12:32	192.2	189.1	185.8	2160	65
12/4/2020 12:32	192.2	188.9	185.7	2160	64.92
12/4/2020 12:32	192.2	188.9	185.4	2163	65
12/4/2020 12:32	192.2	189.3	185.4	2169	65.09
12/4/2020 12:32	192.2	188.9	186.2	2167	64.96
12/4/2020 12:32	192.1	189	186.4	2165	64.92
12/4/2020 12:32	192.1	189.1	185.5	2166	65.04
12/4/2020 12:32	192	189	185.6	2164	64.96
12/4/2020 12:32	192	188.9	185.6	2165	65.04
12/4/2020 12:32	191.9	188.8	185.8	2163	65
12/4/2020 12:32	191.6	188.9	185.9	2165	65
12/4/2020 12:32	191.8	189.3	185.5	2167	64.88
12/4/2020 12:32	191.8	189.3	185.5	2176	64.92
12/4/2020 12:32	191.8	189.1	185.7	2174	64.92

12/4/2020 12:32	191.7	188.9	186.1	2173	64.92
12/4/2020 12:32	191.7	189	186.5	2174	64.96
12/4/2020 12:32	191.6	189.1	186.3	2172	64.88
12/4/2020 12:32	191.5	188.8	185.7	2174	64.88
12/4/2020 12:32	191.4	189.3	185.5	2175	64.92
12/4/2020 12:32	191.4	188.8	185.3	2176	64.92
12/4/2020 12:32	191.3	188.7	185.3	2176	64.88
12/4/2020 12:32	191.3	188.9	185.5	2176	64.96
12/4/2020 12:32	191.1	188.9	185.5	2178	65.04
12/4/2020 12:32	191.1	189.2	185.5	2174	65.08
12/4/2020 12:32	190.9	189.1	185.8	2172	65.08
12/4/2020 12:32	191	189.3	185.6	2168	64.92
12/4/2020 12:32	190.9	188.9	185.6	2171	64.88
12/4/2020 12:32	190.8	188.8	185.6	2175	64.92
12/4/2020 12:32	190.9	189.3	185.4	2170	64.96
12/4/2020 12:32	190.7	189.1	185.5	2167	64.99
12/4/2020 12:32	190.7	188.9	185.4	2162	65
12/4/2020 12:32	190.7	189.3	185.5	2165	65.04
12/4/2020 12:32	190.6	189	185.6	2163	64.92
12/4/2020 12:32	190.6	188.9	186	2167	65.04
12/4/2020 12:32	190.5	188.8	186.2	2170	64.96
12/4/2020 12:32	190.5	189.1	186.3	2167	64.92
12/4/2020 12:32	190.4	189.1	186.1	2169	64.92
12/4/2020 12:32	190.4	188.9	185.6	2167	65
12/4/2020 12:32	190.4	189.1	185.5	2168	64.96
12/4/2020 12:32	190.3	188.7	185.6	2167	65
12/4/2020 12:32	190.3	189.2	185.6	2166	64.96
12/4/2020 12:32	190.3	189.1	185.6	2165	64.92
12/4/2020 12:32	190.3	188.9	185.6	2166	64.84
12/4/2020 12:32	190.3	189.2	185.8	2167	64.96
12/4/2020 12:32	190.3	188.8	185.6	2169	64.92
12/4/2020 12:32	190.2	188.9	185.5	2167	64.88
12/4/2020 12:32	190.1	189.4	185.8	2170	64.88
12/4/2020 12:32	190.1	188.9	186.1	2169	64.96
12/4/2020 12:32	190.1	189.3	186.4	2169	64.99
12/4/2020 12:32	190	189	186.2	2169	65.08
12/4/2020 12:32	190	188.8	186.2	2171	64.92
12/4/2020 12:32	190	189.1	186.1	2173	64.96
12/4/2020 12:32	189.9	188.8	185.2	2168	64.88
12/4/2020 12:33	189.9	188.8	185.3	2167	64.88
12/4/2020 12:33	189.9	189.1	185.3	2168	64.84
12/4/2020 12:33	189.8	188.8	185	2169	64.92
12/4/2020 12:33	189.8	189.2	185.4	2167	64.96
12/4/2020 12:33	189.8	188.8	185.3	2167	64.96
12/4/2020 12:33	189.8	189.1	185.7	2167	65.12
12/4/2020 12:33	189.7	189	185.4	2171	65.08
12/4/2020 12:33	189.7	188.8	185.4	2171	64.96

12/4/2020 12:33	189.6	189.1	185.4	2169	65
12/4/2020 12:33	189.6	188.9	185.5	2167	65
12/4/2020 12:33	189.6	188.8	185.5	2169	64.92
12/4/2020 12:33	189.6	189	185.8	2168	65
12/4/2020 12:33	189.5	188.8	185.7	2170	65
12/4/2020 12:33	189.5	188.9	185.8	2172	65
12/4/2020 12:33	189.4	188.7	185.7	2173	65.04
12/4/2020 12:33	189.4	188.8	185.8	2171	65.09
12/4/2020 12:33	189.4	189.3	185.6	2164	65.04
12/4/2020 12:33	189.3	188.7	185.8	2167	65.04
12/4/2020 12:33	189.3	188.9	185.5	2168	65.04
12/4/2020 12:33	189.3	188.9	185.6	2167	64.99
12/4/2020 12:33	189.2	188.6	185.8	2166	64.99
12/4/2020 12:33	189.1	189.1	185.5	2167	65
12/4/2020 12:33	189.1	189.1	185.7	2163	64.92
12/4/2020 12:33	189.1	189.1	185.5	2165	64.83
12/4/2020 12:33	189	189.1	185.5	2164	65.01
12/4/2020 12:33	189	188.8	185.9	2159	65.03
12/4/2020 12:33	189	189.3	185.6	2158	65.03
12/4/2020 12:33	188.9	188.9	185.7	2153	65.08
12/4/2020 12:33	188.9	189.1	185.4	2151	64.99
12/4/2020 12:33	189	189	185.3	2150	64.95
12/4/2020 12:33	188.9	188.8	185.3	2156	65.01
12/4/2020 12:33	188.9	189.3	184.9	2156	64.99
12/4/2020 12:33	188.8	188.8	185.1	2152	64.88
12/4/2020 12:33	188.8	189.1	185.8	2155	65.12
12/4/2020 12:33	188.8	189	186	2150	64.99
12/4/2020 12:33	188.8	188.9	186.1	2149	64.96
12/4/2020 12:33	188.8	189.3	185.4	2154	64.92
12/4/2020 12:33	188.8	188.9	185.1	2152	64.99
12/4/2020 12:33	188.8	188.8	185.5	2151	65.04
12/4/2020 12:33	188.9	189.1	185.5	2152	65.08
12/4/2020 12:33	188.9	188.7	185.4	2152	64.92
12/4/2020 12:33	188.9	188.9	185.2	2152	65.04
12/4/2020 12:33	189	188.8	184.9	2152	65.05
12/4/2020 12:33	188.9	188.9	185.8	2148	64.96
12/4/2020 12:33	189	189.2	186.2	2145	64.96
12/4/2020 12:33	189.1	188.9	185.5	2148	64.96
12/4/2020 12:33	189.1	189.1	185.4	2147	64.87
12/4/2020 12:33	189.1	188.8	185.3	2151	64.92
12/4/2020 12:33	189.1	188.9	185.4	2152	64.96
12/4/2020 12:33	189.1	189.1	185.5	2148	64.87
12/4/2020 12:33	189.3	189.3	185.1	2144	64.96
12/4/2020 12:33	189.3	189.1	185.2	2144	65.05
12/4/2020 12:33	189.4	188.9	186	2141	64.83
12/4/2020 12:33	189.4	188.9	186.1	2140	64.91
12/4/2020 12:33	189.5	189	185.8	2138	64.92

12/4/2020 12:33	189.6	189	185.4	2142	64.88
12/4/2020 12:33	189.6	189.1	185.4	2143	65
12/4/2020 12:33	189.7	188.8	185.6	2144	65.08
12/4/2020 12:33	189.8	188.9	185.5	2142	64.88
12/4/2020 12:34	189.8	188.7	185.4	2139	64.88
12/4/2020 12:34	190	189.1	185.3	2142	64.96
12/4/2020 12:34	190	188.8	186.2	2144	64.99
12/4/2020 12:34	190.1	188.9	186.3	2146	64.92
12/4/2020 12:34	190.3	189.3	185.7	2146	65.01
12/4/2020 12:34	190.2	189.3	185.3	2144	64.8
12/4/2020 12:34	190.4	188.7	185.8	2140	64.84
12/4/2020 12:34	190.4	189.1	185.5	2140	64.84
12/4/2020 12:34	190.5	188.9	185.6	2145	64.8
12/4/2020 12:34	190.6	189.1	185.6	2146	65.04
12/4/2020 12:34	190.7	188.8	185.3	2149	64.96
12/4/2020 12:34	190.7	188.9	185.1	2150	65
12/4/2020 12:34	190.8	189.1	185.3	2154	64.96
12/4/2020 12:34	190.8	189.3	186	2157	65
12/4/2020 12:34	190.9	188.6	186.3	2156	65
12/4/2020 12:34	191	189.1	186	2159	64.88
12/4/2020 12:34	191.1	188.8	185.4	2152	64.96
12/4/2020 12:34	191.1	188.9	185.3	2156	64.96
12/4/2020 12:34	191.2	189.1	185.5	2156	65
12/4/2020 12:34	191.3	189.2	185.6	2156	64.96
12/4/2020 12:34	191.3	188.9	185.6	2155	65.05
12/4/2020 12:34	191.3	188.8	185.7	2153	64.96
12/4/2020 12:34	191.4	188.7	185.6	2154	64.92
12/4/2020 12:34	191.4	188.8	185.6	2159	64.92
12/4/2020 12:34	191.5	189.1	185.8	2162	64.96
12/4/2020 12:34	191.4	189	186	2159	64.96
12/4/2020 12:34	191.5	188.8	185.8	2156	64.96
12/4/2020 12:34	191.6	189.1	185.8	2158	64.96
12/4/2020 12:34	191.6	188.7	185.6	2159	65.04
12/4/2020 12:34	191.6	188.7	185.5	2157	65.04
12/4/2020 12:34	191.7	189.2	185.4	2156	64.96
12/4/2020 12:34	191.7	189	185.5	2160	65.08
12/4/2020 12:34	191.8	189.2	185.5	2156	65.04
12/4/2020 12:34	191.8	189	186.1	2158	64.92
12/4/2020 12:34	191.8	188.8	186.5	2160	65.08
12/4/2020 12:34	191.8	189	186.4	2156	65.08
12/4/2020 12:34	191.8	188.9	186.1	2158	64.99
12/4/2020 12:34	191.9	188.9	185.5	2164	64.96
12/4/2020 12:34	191.9	188.9	185.3	2163	64.84
12/4/2020 12:34	191.9	188.8	185.8	2163	64.99
12/4/2020 12:34	191.9	189.1	185.6	2164	64.88
12/4/2020 12:34	191.9	188.9	185.8	2167	64.88
12/4/2020 12:34	192	188.8	185.5	2165	65.03

12/4/2020 12:34	191.9	189.3	185.9	2167	65.08
12/4/2020 12:34	192	188.8	186.5	2165	64.99
12/4/2020 12:34	192	189.1	185.6	2161	64.88
12/4/2020 12:34	192	188.7	185.6	2164	64.99
12/4/2020 12:34	192	189.2	185.8	2158	64.88
12/4/2020 12:34	192	189	185.6	2155	65
12/4/2020 12:34	192	188.7	185.5	2157	64.92
12/4/2020 12:34	192	189.2	186.2	2157	65.04
12/4/2020 12:34	192.1	188.9	186.3	2163	65.04
12/4/2020 12:34	192	188.9	185.8	2165	64.91
12/4/2020 12:34	192	189.1	185.6	2167	64.96
12/4/2020 12:34	192	188.7	185.7	2173	65
12/4/2020 12:34	192	189	185.8	2167	64.92
12/4/2020 12:34	192	188.9	185.7	2162	64.96
12/4/2020 12:34	192	188.9	185.6	2161	64.88
12/4/2020 12:34	192	189.3	186.7	2164	64.96
12/4/2020 12:34	192	188.8	186.4	2161	64.96
12/4/2020 12:35	191.9	189.1	185.9	2160	65
12/4/2020 12:35	191.9	188.8	185.6	2163	64.92
12/4/2020 12:35	191.9	189.1	185.5	2162	65.04
12/4/2020 12:35	191.9	189.3	185.8	2160	65
12/4/2020 12:35	191.9	189.1	185.9	2163	65.04
12/4/2020 12:35	191.9	189.1	186	2163	64.84
12/4/2020 12:35	191.8	188.7	185.9	2167	64.8
12/4/2020 12:35	191.8	188.9	185.6	2167	64.92
12/4/2020 12:35	191.8	189.2	185.6	2163	64.88
12/4/2020 12:35	191.8	188.9	185.8	2167	64.99
12/4/2020 12:35	191.8	189.3	185.6	2163	64.88
12/4/2020 12:35	191.8	188.9	186	2165	64.99
12/4/2020 12:35	191.8	188.9	186.2	2170	64.99
12/4/2020 12:35	191.8	189.3	186.3	2169	64.93
12/4/2020 12:35	191.7	189	186.5	2167	64.88
12/4/2020 12:35	191.6	189.3	186.5	2167	64.88
12/4/2020 12:35	191.6	188.8	186.8	2172	64.93
12/4/2020 12:35	191.7	188.8	186.7	2174	64.88
12/4/2020 12:35	191.6	189.3	186.4	2174	65.03
12/4/2020 12:35	191.6	188.9	186.6	2174	64.96
12/4/2020 12:35	191.6	189.2	186.3	2173	65
12/4/2020 12:35	191.6	189.2	186.1	2168	65.08
12/4/2020 12:35	191.5	188.8	185.9	2169	64.92
12/4/2020 12:35	191.5	189.2	185.5	2171	65.03
12/4/2020 12:35	191.4	189.1	185.8	2173	65.04
12/4/2020 12:35	191.4	189.3	185.8	2177	65.08
12/4/2020 12:35	191.4	189.1	185.8	2171	64.95
12/4/2020 12:35	191.4	188.8	185.8	2171	64.88
12/4/2020 12:35	191.4	188.8	185.5	2170	64.96
12/4/2020 12:35	191.3	188.9	185.4	2167	64.96

12/4/2020 12:35	191.3	189	186.3	2169	64.96
12/4/2020 12:35	191.3	189.3	186.7	2174	64.96
12/4/2020 12:35	191.2	189.3	185.7	2170	64.96
12/4/2020 12:35	191.2	189	185.8	2169	64.99
12/4/2020 12:35	191.2	188.9	185.8	2173	65.04
12/4/2020 12:35	191.1	188.9	185.7	2169	64.96
12/4/2020 12:35	191.1	189.1	185.7	2171	64.96
12/4/2020 12:35	191.1	189.1	185.8	2173	65.04
12/4/2020 12:35	191	189.1	186.6	2174	64.96
12/4/2020 12:35	191	188.9	186.7	2176	64.96
12/4/2020 12:35	191	188.9	185.5	2174	64.92
12/4/2020 12:35	190.9	189.1	185.7	2172	64.88
12/4/2020 12:35	190.9	188.8	186	2175	64.88
12/4/2020 12:35	190.9	189	186	2169	64.96
12/4/2020 12:35	190.9	188.8	186	2169	64.96
12/4/2020 12:35	190.8	188.8	185.5	2168	65
12/4/2020 12:35	190.8	188.7	185.6	2169	65.08
12/4/2020 12:35	190.8	188.9	185.5	2169	64.88
12/4/2020 12:35	190.6	189.2	186.1	2167	65.08
12/4/2020 12:35	190.7	189.1	186.5	2171	64.96
12/4/2020 12:35	190.6	189.1	186.2	2171	65.08
12/4/2020 12:35	190.6	189.2	185.8	2169	64.99
12/4/2020 12:35	190.5	188.6	185.8	2171	65.08
12/4/2020 12:35	190.5	189	185.8	2169	64.92
12/4/2020 12:35	190.5	188.8	185.9	2168	64.99
12/4/2020 12:35	190.5	189.2	185.8	2170	65.08
12/4/2020 12:35	190.5	189.2	185.6	2171	64.99
12/4/2020 12:35	190.3	188.8	185.4	2173	65.04
12/4/2020 12:35	190.2	189.1	185.6	2176	65.04
12/4/2020 12:36	190.3	188.7	186.4	2173	64.88
12/4/2020 12:36	190.2	189.1	186.7	2169	64.88
12/4/2020 12:36	190.3	189.3	186.2	2172	65.01
12/4/2020 12:36	190.1	188.9	185.8	2167	64.92
12/4/2020 12:36	190	189	185.8	2169	64.96
12/4/2020 12:36	190.1	188.6	185.6	2167	65.05
12/4/2020 12:36	190	189.1	186.1	2165	64.87
12/4/2020 12:36	190	189	185.8	2164	64.96
12/4/2020 12:36	189.9	188.9	185.6	2167	65
12/4/2020 12:36	189.8	188.9	185.8	2167	64.96
12/4/2020 12:36	189.9	189.1	185.8	2171	64.92
12/4/2020 12:36	189.8	189.3	185.8	2173	65.09
12/4/2020 12:36	189.8	188.8	185.5	2176	64.96
12/4/2020 12:36	189.7	188.8	185.3	2178	64.96
12/4/2020 12:36	189.7	189	185.6	2178	65
12/4/2020 12:36	189.6	189.2	185.4	2177	65
12/4/2020 12:36	189.6	188.7	185.4	2174	65
12/4/2020 12:36	189.6	189.3	186.1	2171	65.04

12/4/2020 12:36	189.5	188.9	186.3	2171	64.92
12/4/2020 12:36	189.5	188.8	186.4	2173	64.88
12/4/2020 12:36	189.5	188.8	186	2171	65.08
12/4/2020 12:36	189.5	189.1	185.4	2174	64.99
12/4/2020 12:36	189.4	189	185.5	2170	64.92
12/4/2020 12:36	189.4	188.9	186	2178	65.04
12/4/2020 12:36	189.4	188.9	185.6	2182	65
12/4/2020 12:36	189.3	188.8	185.7	2181	65.16
12/4/2020 12:36	189.3	188.9	185.4	2178	64.88
12/4/2020 12:36	189.2	189.3	185.7	2176	65.04
12/4/2020 12:36	189.2	189	186.5	2171	64.92
12/4/2020 12:36	189.1	189.1	185.8	2174	64.92
12/4/2020 12:36	189	188.7	185.6	2165	64.92
12/4/2020 12:36	189.1	188.6	185.8	2165	64.96
12/4/2020 12:36	189	189.2	185.8	2169	65.04
12/4/2020 12:36	189	188.9	185.8	2170	64.96
12/4/2020 12:36	189	189.1	185.1	2171	64.96
12/4/2020 12:36	188.8	188.8	185.7	2173	64.96
12/4/2020 12:36	188.8	188.8	186.4	2171	65
12/4/2020 12:36	188.8	189.3	186.1	2173	65
12/4/2020 12:36	188.8	188.8	185.6	2172	64.96
12/4/2020 12:36	188.7	188.7	185.7	2171	64.92
12/4/2020 12:36	188.7	189.2	185.6	2168	64.96
12/4/2020 12:36	188.6	188.9	185.6	2168	65
12/4/2020 12:36	188.6	188.9	185.3	2169	65.04
12/4/2020 12:36	188.6	189	185.5	2167	64.96
12/4/2020 12:36	188.6	188.8	186.3	2167	64.96
12/4/2020 12:36	188.5	189.3	186.1	2167	65
12/4/2020 12:36	188.5	188.8	185.2	2163	64.87
12/4/2020 12:36	188.5	189	185.5	2168	65.08
12/4/2020 12:36	188.4	189.1	185.5	2171	64.88
12/4/2020 12:36	188.4	188.8	185.6	2167	64.91
12/4/2020 12:36	188.3	188.9	185.3	2167	64.92
12/4/2020 12:36	188.3	188.8	185	2164	65.04
12/4/2020 12:36	188.3	189.2	186	2163	65.04
12/4/2020 12:36	188.2	189.1	186.5	2165	64.92
12/4/2020 12:36	188.2	188.8	185.8	2171	65.08
12/4/2020 12:36	188.1	189.3	185.3	2168	64.92
12/4/2020 12:36	188.1	188.8	185.4	2173	64.92
12/4/2020 12:36	188.1	188.9	185.5	2169	64.88
12/4/2020 12:36	188.1	189.1	185.5	2166	64.92
12/4/2020 12:36	188.1	189.3	185.3	2169	64.96
12/4/2020 12:37	188	189.1	186	2166	64.83
12/4/2020 12:37	188	188.9	186.5	2168	65.04
12/4/2020 12:37	187.9	188.9	185.4	2167	64.88
12/4/2020 12:37	187.9	188.6	185.6	2171	65.08
12/4/2020 12:37	187.9	188.8	185.6	2171	64.8

12/4/2020 12:37	187.8	189.3	185.5	2173	64.92
12/4/2020 12:37	187.8	188.9	185.6	2173	64.88
12/4/2020 12:37	187.7	189.2	185.2	2176	65.04
12/4/2020 12:37	187.8	189.1	186	2178	64.88
12/4/2020 12:37	187.7	188.7	186.1	2177	64.92
12/4/2020 12:37	187.7	188.9	185.3	2173	64.83
12/4/2020 12:37	187.7	188.8	185.4	2171	65.08
12/4/2020 12:37	187.7	188.9	185.6	2173	64.96
12/4/2020 12:37	187.6	189.1	185.3	2174	64.92
12/4/2020 12:37	187.5	188.7	185.3	2174	64.99
12/4/2020 12:37	187.6	189.2	185	2171	64.88
12/4/2020 12:37	187.5	188.7	185.6	2171	64.84
12/4/2020 12:37	187.5	189.2	186.2	2171	65.04
12/4/2020 12:37	187.4	189.3	185.4	2174	64.88
12/4/2020 12:37	187.5	188.7	185.1	2171	64.96
12/4/2020 12:37	187.5	188.9	185.3	2171	64.96
12/4/2020 12:37	187.4	188.9	185.4	2169	64.92
12/4/2020 12:37	187.4	189.3	185.3	2167	65
12/4/2020 12:37	187.4	188.9	185	2167	65
12/4/2020 12:37	187.4	188.8	185.7	2163	64.92
12/4/2020 12:37	187.5	189.2	186.3	2167	64.96
12/4/2020 12:37	187.4	188.8	185.3	2165	65.04
12/4/2020 12:37	187.4	188.9	185.1	2168	64.92
12/4/2020 12:37	187.4	188.9	185.5	2165	64.96
12/4/2020 12:37	187.4	188.8	185.4	2167	65
12/4/2020 12:37	187.4	189.2	185.4	2169	64.92
12/4/2020 12:37	187.4	188.9	185.1	2166	64.92
12/4/2020 12:37	187.5	189.1	186	2170	65
12/4/2020 12:37	187.5	188.9	186	2166	64.96
12/4/2020 12:37	187.5	188.7	185.3	2165	64.92
12/4/2020 12:37	187.5	189.1	185.3	2171	65
12/4/2020 12:37	187.5	188.8	185.6	2173	64.88
12/4/2020 12:37	187.5	188.8	185.4	2171	64.92
12/4/2020 12:37	187.5	188.8	185.2	2171	64.96
12/4/2020 12:37	187.5	188.9	185.1	2172	64.83
12/4/2020 12:37	187.6	189	185.8	2175	64.88
12/4/2020 12:37	187.6	188.8	186.1	2175	65
12/4/2020 12:37	187.7	188.8	185.4	2174	64.92
12/4/2020 12:37	187.6	189.1	185.1	2170	64.96
12/4/2020 12:37	187.6	188.7	185.6	2170	65.12
12/4/2020 12:37	187.7	189.1	185.4	2167	64.92
12/4/2020 12:37	187.7	189	185.1	2171	64.99
12/4/2020 12:37	187.7	188.8	185.2	2167	65.01
12/4/2020 12:37	187.7	188.8	185.7	2171	64.88
12/4/2020 12:37	187.8	188.8	186.2	2171	64.95
12/4/2020 12:37	187.8	189.1	185.8	2172	65.08
12/4/2020 12:37	187.8	188.1	185.1	2167	64.88

12/4/2020 12:37	187.8	188	185.4	2171	64.88
12/4/2020 12:37	187.8	188.1	185.3	2172	64.92
12/4/2020 12:37	187.8	187.9	185.5	2171	64.83
12/4/2020 12:37	187.9	187.9	185.3	2171	64.83
12/4/2020 12:37	187.9	188.1	185	2167	65
12/4/2020 12:37	187.9	188.1	186.1	2169	64.99
12/4/2020 12:37	188	188.1	185.9	2169	64.8
12/4/2020 12:38	188	188	185.4	2169	64.96
12/4/2020 12:38	188	187.9	185.1	2171	64.8
12/4/2020 12:38	188	188.1	185.4	2167	64.99
12/4/2020 12:38	188	188.1	185.9	2168	65
12/4/2020 12:38	188.1	188.4	185.6	2168	64.95
12/4/2020 12:38	188.1	188	185.3	2169	65.04
12/4/2020 12:38	188.1	188.1	185	2172	65.12
12/4/2020 12:38	188.1	187.9	185.2	2171	65.03
12/4/2020 12:38	188.2	188.1	185.2	2169	64.99
12/4/2020 12:38	188.3	188.1	186	2174	65.16
12/4/2020 12:38	188.2	188.1	186.1	2171	64.95
12/4/2020 12:38	188.2	188.1	186	2172	65.04
12/4/2020 12:38	188.3	188.1	185.7	2176	65.05
12/4/2020 12:38	188.3	188.1	185.2	2178	64.87
12/4/2020 12:38	188.3	187.9	185.1	2174	64.96
12/4/2020 12:38	188.3	188.1	185.5	2174	65.09
12/4/2020 12:38	188.3	188.2	185.3	2180	65.04
12/4/2020 12:38	188.4	188.1	185.5	2179	64.92
12/4/2020 12:38	188.4	188.1	185.6	2174	65
12/4/2020 12:38	188.3	188	185.3	2178	64.96
12/4/2020 12:38	188.5	188.2	185.5	2174	65.04
12/4/2020 12:38	188.5	188.1	185.3	2173	65
12/4/2020 12:38	188.5	188.1	185.2	2176	64.99
12/4/2020 12:38	188.5	188	185.4	2171	64.99
12/4/2020 12:38	188.6	188.1	185.5	2173	65.08
12/4/2020 12:38	188.6	188.1	185.3	2173	65.04
12/4/2020 12:38	188.6	188.1	185.6	2174	64.96
12/4/2020 12:38	188.6	188.1	185.4	2177	65.12
12/4/2020 12:38	188.6	188.1	185.6	2178	64.92
12/4/2020 12:38	188.7	188.1	185.3	2177	64.99
12/4/2020 12:38	188.7	188.1	185.3	2178	65.08
12/4/2020 12:38	188.7	188	185.5	2179	64.88
12/4/2020 12:38	188.7	188.2	185.3	2174	64.96
12/4/2020 12:38	188.8	188.1	185.4	2176	64.96
12/4/2020 12:38	188.8	188.1	185.2	2173	64.92
12/4/2020 12:38	188.8	188	185.4	2169	65.04
12/4/2020 12:38	188.8	188.1	186.1	2167	65.04
12/4/2020 12:38	188.8	188.2	186.3	2166	65.04
12/4/2020 12:38	188.9	188.2	185.8	2168	64.92
12/4/2020 12:38	188.9	188.1	185.3	2173	64.99

12/4/2020 12:38	189	188	184.9	2171	64.96
12/4/2020 12:38	188.9	188	185.1	2171	65
12/4/2020 12:38	189	188	185.4	2178	64.92
12/4/2020 12:38	189	188	185.4	2178	64.96
12/4/2020 12:38	189	187.9	185.5	2176	64.92
12/4/2020 12:38	189	188.1	185.6	2178	64.96
12/4/2020 12:38	189.1	188.2	185.5	2176	65
12/4/2020 12:38	189.1	188.1	185.5	2176	65
12/4/2020 12:38	189	188.1	185.3	2180	64.96
12/4/2020 12:38	189	188.1	185.1	2181	64.96
12/4/2020 12:38	189	188.1	185.4	2177	64.96
12/4/2020 12:38	189	188.1	185.3	2174	65.09
12/4/2020 12:38	189.1	188.1	185.3	2174	64.92
12/4/2020 12:38	189.1	188.1	185.5	2171	64.96
12/4/2020 12:38	189.1	188.1	185.5	2173	64.93
12/4/2020 12:38	189	188.1	185.5	2174	64.88
12/4/2020 12:38	189	188.2	185.6	2174	64.84
12/4/2020 12:38	189.1	188.1	185.3	2171	65.04
12/4/2020 12:38	189	188.1	185.1	2169	64.88
12/4/2020 12:39	189	188.1	185.3	2167	65.04
12/4/2020 12:39	189	188.1	186.3	2167	64.95
12/4/2020 12:39	189	188.1	185.8	2167	65.08
12/4/2020 12:39	189	188	185.1	2167	65.04
12/4/2020 12:39	189	188.1	185.7	2166	65
12/4/2020 12:39	189	188	185.5	2167	64.92
12/4/2020 12:39	189	188.2	185.1	2167	65.04
12/4/2020 12:39	189	188	185.3	2166	64.92
12/4/2020 12:39	189	188.1	185.9	2168	64.93
12/4/2020 12:39	189.1	188	186	2163	65
12/4/2020 12:39	189.1	188.1	185.6	2160	64.96
12/4/2020 12:39	189.1	188.1	185.3	2162	65
12/4/2020 12:39	189.2	188.1	185.6	2156	65
12/4/2020 12:39	189.2	188.1	185.4	2156	65
12/4/2020 12:39	189.2	188.1	185.3	2156	64.92
12/4/2020 12:39	189.2	188.1	185.3	2160	64.92
12/4/2020 12:39	189.2	188.1	185.5	2160	65.04
12/4/2020 12:39	189.3	188.1	186.1	2160	65
12/4/2020 12:39	189.3	188.1	185.5	2160	65
12/4/2020 12:39	189.5	188.1	185.1	2162	65
12/4/2020 12:39	189.5	188.1	185.4	2155	65
12/4/2020 12:39	189.5	188.1	185.5	2160	65.08
12/4/2020 12:39	189.6	188.1	185.6	2160	64.88
12/4/2020 12:39	189.6	187.9	185.4	2157	64.92
12/4/2020 12:39	189.7	188	185.3	2156	64.89
12/4/2020 12:39	189.8	188.1	186	2154	64.84
12/4/2020 12:39	189.8	188.2	186.3	2155	64.96
12/4/2020 12:39	189.9	188.1	185.5	2156	65.04

12/4/2020 12:39	189.9	188	185.4	2157	64.88
12/4/2020 12:39	190.1	188.1	185.8	2152	65.04
12/4/2020 12:39	190.1	188.1	185.7	2158	65.05
12/4/2020 12:39	190.2	188.1	185.6	2157	64.96
12/4/2020 12:39	190.3	188.1	185.4	2154	65
12/4/2020 12:39	190.3	188.1	185.1	2159	65
12/4/2020 12:39	190.5	188.1	186.1	2158	64.96
12/4/2020 12:39	190.6	188.2	186.2	2158	64.96
12/4/2020 12:39	190.7	188.2	185.7	2160	64.88
12/4/2020 12:39	190.7	188.1	185.3	2160	64.92
12/4/2020 12:39	190.8	188	185.3	2160	65.05
12/4/2020 12:39	190.8	188.6	185.7	2163	64.96
12/4/2020 12:39	191	189	185.6	2165	65
12/4/2020 12:39	191	189.3	185.8	2167	64.92
12/4/2020 12:39	191.1	188.9	185.7	2168	65.04
12/4/2020 12:39	191.1	188.8	185.6	2172	65.09
12/4/2020 12:39	191.2	189.1	185.3	2170	64.96
12/4/2020 12:39	191.3	188.7	185.6	2171	64.87
12/4/2020 12:39	191.4	189.3	185.8	2167	64.96
12/4/2020 12:39	191.5	188.9	186.5	2169	64.92
12/4/2020 12:39	191.6	188.7	186.5	2171	65
12/4/2020 12:39	191.5	189.2	186	2176	64.81
12/4/2020 12:39	191.6	188.8	185.6	2176	64.84
12/4/2020 12:39	191.6	189	185.3	2174	64.84
12/4/2020 12:39	191.6	188.9	185.6	2175	64.93
12/4/2020 12:39	191.8	189.1	185.8	2176	64.88
12/4/2020 12:39	191.8	189	185.6	2174	64.81
12/4/2020 12:39	191.8	188.6	185.7	2177	65.01
12/4/2020 12:39	191.8	189.2	185.9	2174	65
12/4/2020 12:39	191.8	188.7	185.4	2172	64.92
12/4/2020 12:39	191.8	188.8	185.3	2174	64.93
12/4/2020 12:39	191.9	189.1	186	2176	64.81
12/4/2020 12:40	191.9	188.9	186.3	2181	64.88
12/4/2020 12:40	191.9	188.9	186.3	2181	64.96
12/4/2020 12:40	191.9	189.1	185.8	2180	65
12/4/2020 12:40	191.9	189.3	185.6	2182	64.96
12/4/2020 12:40	191.9	189.1	185.8	2185	65.05
12/4/2020 12:40	191.9	189	186	2186	64.96
12/4/2020 12:40	191.9	189.2	186	2185	65
12/4/2020 12:40	192	188.9	185.9	2183	65
12/4/2020 12:40	191.9	189.1	185.5	2184	64.92
12/4/2020 12:40	191.8	188.9	185.7	2185	64.92
12/4/2020 12:40	191.8	188.8	186.5	2185	65.01
12/4/2020 12:40	191.8	188.8	186.3	2185	65
12/4/2020 12:40	191.8	188.8	186	2185	64.92
12/4/2020 12:40	191.8	189.3	185.4	2187	65.05
12/4/2020 12:40	191.8	189	185.9	2185	64.81

12/4/2020 12:40	191.8	188.9	186.6	2185	64.81
12/4/2020 12:40	191.8	189	185.7	2187	64.93
12/4/2020 12:40	191.6	188.8	185.7	2184	64.92
12/4/2020 12:40	191.6	188.9	185.4	2182	65
12/4/2020 12:40	191.6	189.2	186.3	2187	65.05
12/4/2020 12:40	191.5	188.9	186.5	2189	64.92
12/4/2020 12:40	191.5	189.2	185.5	2188	65
12/4/2020 12:40	191.4	189.1	185.7	2190	65.01
12/4/2020 12:40	191.3	188.9	185.8	2189	64.96
12/4/2020 12:40	191.4	189.3	185.8	2191	64.92
12/4/2020 12:40	191.3	188.8	185.5	2192	65.05
12/4/2020 12:40	191.3	189.1	185.8	2192	64.96
12/4/2020 12:40	191.2	188.8	186.2	2185	64.92
12/4/2020 12:40	191.1	188.8	186.6	2184	65
12/4/2020 12:40	191.2	189.3	186	2186	64.96
12/4/2020 12:40	191.1	188.8	185.7	2185	65.04
12/4/2020 12:40	191.1	189.1	185.8	2183	64.92
12/4/2020 12:40	191	188.9	185.8	2185	65.08
12/4/2020 12:40	190.9	188.7	186	2183	65.04
12/4/2020 12:40	190.9	189.1	186	2182	65.04
12/4/2020 12:40	190.8	188.8	185.7	2182	65.04
12/4/2020 12:40	190.8	189	185.5	2183	64.96
12/4/2020 12:40	190.8	189	185.8	2179	64.93
12/4/2020 12:40	190.8	188.8	186.3	2180	64.92
12/4/2020 12:40	190.7	189	186.7	2177	64.88
12/4/2020 12:40	190.7	188.9	186	2181	65
12/4/2020 12:40	190.7	188.8	185.4	2185	65.05
12/4/2020 12:40	190.7	189.3	186	2185	65.08
12/4/2020 12:40	190.7	188.6	185.9	2181	64.92
12/4/2020 12:40	190.7	189	185.9	2181	65
12/4/2020 12:40	190.7	188.8	185.7	2183	65
12/4/2020 12:40	190.7	189.2	185.7	2184	65.04
12/4/2020 12:40	190.7	189.1	185.7	2184	64.96
12/4/2020 12:40	190.6	188.8	186	2178	65
12/4/2020 12:40	190.6	189.2	186.3	2177	64.96
12/4/2020 12:40	190.7	188.7	186.6	2178	65.08
12/4/2020 12:40	190.6	188.8	186.2	2181	65
12/4/2020 12:40	190.6	189.2	185.8	2185	64.92
12/4/2020 12:40	190.6	188.8	185.8	2186	64.88
12/4/2020 12:40	190.6	188.9	185.7	2188	64.96
12/4/2020 12:40	190.6	189.1	186.1	2189	64.93
12/4/2020 12:40	190.6	188.8	186	2185	64.96
12/4/2020 12:40	190.6	189.1	185.8	2187	64.92
12/4/2020 12:40	190.6	188.7	185.8	2185	64.84
12/4/2020 12:41	190.5	189.2	185.5	2187	65.12
12/4/2020 12:41	190.5	189.1	185.3	2187	65.08
12/4/2020 12:41	190.5	188.8	186.1	2187	64.96

12/4/2020 12:41	190.5	189.2	186.3	2186	65.05
12/4/2020 12:41	190.5	188.7	186.7	2185	64.92
12/4/2020 12:41	190.5	189	186.2	2185	64.92
12/4/2020 12:41	190.5	188.9	185.6	2185	65
12/4/2020 12:41	190.5	188.7	185.8	2182	64.96
12/4/2020 12:41	190.5	189.2	185.8	2178	64.96
12/4/2020 12:41	190.5	188.8	185.6	2181	64.92
12/4/2020 12:41	190.5	188.9	186	2182	65
12/4/2020 12:41	190.5	189.1	185.8	2188	64.92
12/4/2020 12:41	190.5	188.9	186	2189	65.04
12/4/2020 12:41	190.4	189.1	186	2191	65
12/4/2020 12:41	190.4	188.8	185.7	2192	64.88
12/4/2020 12:41	190.3	189.2	185.9	2194	64.96
12/4/2020 12:41	190.3	189	185.8	2196	64.96
12/4/2020 12:41	190.3	188.8	185.8	2192	64.96
12/4/2020 12:41	190.2	189.3	185.8	2192	64.92
12/4/2020 12:41	190.2	188.9	185.5	2189	65.08
12/4/2020 12:41	190.1	189.2	185.6	2192	65.04
12/4/2020 12:41	190.1	189.1	186.2	2188	64.96
12/4/2020 12:41	190	189.1	186.5	2189	64.96
12/4/2020 12:41	190	189	186.5	2191	64.88
12/4/2020 12:41	190.1	188.9	185.5	2186	64.92
12/4/2020 12:41	190	189.2	185.4	2189	65.08
12/4/2020 12:41	189.9	189.1	185.9	2189	64.92
12/4/2020 12:41	189.9	188.9	185.7	2188	65.08
12/4/2020 12:41	189.8	189.2	185.8	2190	64.88
12/4/2020 12:41	189.8	189	186	2185	64.92
12/4/2020 12:41	189.8	188.9	186.4	2186	64.92
12/4/2020 12:41	189.7	189.3	185.6	2187	65.01
12/4/2020 12:41	189.7	188.8	185.5	2182	64.99
12/4/2020 12:41	189.7	189.1	185.6	2184	65.05
12/4/2020 12:41	189.6	188.9	185.8	2182	65.04
12/4/2020 12:41	189.7	188.7	185.5	2184	64.87
12/4/2020 12:41	189.6	189	186	2184	64.92
12/4/2020 12:41	189.6	188.8	186.5	2183	65.09
12/4/2020 12:41	189.6	189.3	185.3	2187	65
12/4/2020 12:41	189.6	189	185.4	2182	65
12/4/2020 12:41	189.6	189.2	185.6	2188	65
12/4/2020 12:41	189.6	189.2	185.7	2185	65.04
12/4/2020 12:41	189.6	188.8	185.7	2184	65.16
12/4/2020 12:41	189.6	188.9	185.9	2180	64.96
12/4/2020 12:41	189.6	188.8	186.3	2182	64.88
12/4/2020 12:41	189.6	189.3	185.8	2180	65.08
12/4/2020 12:41	189.6	189.3	185.3	2178	65.12
12/4/2020 12:41	189.6	188.8	185.6	2178	64.84
12/4/2020 12:41	189.6	188.8	186.3	2178	65.04
12/4/2020 12:41	189.6	189	185.6	2177	65.08

12/4/2020 12:41	189.6	189.1	185.8	2174	65.08
12/4/2020 12:41	189.6	189.1	186.2	2174	65.08
12/4/2020 12:41	189.7	188.8	186.1	2174	65.08
12/4/2020 12:41	189.7	189	185.6	2175	64.96
12/4/2020 12:41	189.7	189.1	185.6	2172	64.96
12/4/2020 12:41	189.8	188.6	185.5	2171	64.96
12/4/2020 12:41	189.8	189.2	186	2174	64.92
12/4/2020 12:41	189.8	188.8	185.4	2174	65.04
12/4/2020 12:41	190	188.8	185.7	2170	64.96
12/4/2020 12:41	190	189	185.7	2169	64.96
12/4/2020 12:42	190	188.6	185.6	2172	64.96
12/4/2020 12:42	190.1	189.1	185.7	2173	65
12/4/2020 12:42	190	189	185.6	2177	65.04
12/4/2020 12:42	190.2	189	185.5	2178	65
12/4/2020 12:42	190.3	188.8	185.6	2174	64.88
12/4/2020 12:42	190.3	189.1	185.6	2178	64.99
12/4/2020 12:42	190.4	189.1	185.8	2174	64.92
12/4/2020 12:42	190.4	189	186	2178	64.92
12/4/2020 12:42	190.5	188.8	185.9	2180	65.03
12/4/2020 12:42	190.6	188.9	185.8	2177	64.88
12/4/2020 12:42	190.6	188.8	185.6	2173	64.92
12/4/2020 12:42	190.8	189.1	185.6	2174	65
12/4/2020 12:42	190.8	188.9	186.8	2175	65.12
12/4/2020 12:42	190.9	189.1	185.8	2174	64.92
12/4/2020 12:42	191	189	185.8	2175	65.08
12/4/2020 12:42	191.1	188.6	186.2	2168	64.88
12/4/2020 12:42	191.2	189	185.8	2166	65.08
12/4/2020 12:42	191.1	188.8	185.7	2162	65.04
12/4/2020 12:42	191.4	189.3	185.5	2166	64.99
12/4/2020 12:42	191.5	189.1	186.5	2172	64.92
12/4/2020 12:42	191.4	189	186.1	2172	65.04
12/4/2020 12:42	191.6	189	185.5	2173	65
12/4/2020 12:42	191.6	189.1	185.8	2176	64.96
12/4/2020 12:42	191.7	188.7	186.1	2174	64.96
12/4/2020 12:42	191.8	189.3	185.6	2173	64.92
12/4/2020 12:42	191.8	189.1	185.5	2178	65
12/4/2020 12:42	192	189.1	186.4	2179	65.04
12/4/2020 12:42	192	188.8	186.7	2180	64.92
12/4/2020 12:42	192.1	188.8	186.1	2180	64.92
12/4/2020 12:42	192.1	189.1	185.8	2185	65.08
12/4/2020 12:42	192.1	188.9	186	2182	64.96
12/4/2020 12:42	192.2	188.9	186.1	2182	64.99
12/4/2020 12:42	192.2	188.9	185.6	2183	65
12/4/2020 12:42	192.3	189.3	185.8	2182	65.08
12/4/2020 12:42	192.3	189.2	186.7	2185	64.96
12/4/2020 12:42	192.4	189	186.3	2184	64.84
12/4/2020 12:42	192.4	189.2	185.8	2184	65.04

12/4/2020 12:42	192.4	189.2	186	2183	64.96
12/4/2020 12:42	192.4	188.8	185.9	2181	64.83
12/4/2020 12:42	192.4	189	186	2185	65
12/4/2020 12:42	192.5	188.9	185.7	2188	64.93
12/4/2020 12:42	192.5	189.1	185.6	2185	65.08
12/4/2020 12:42	192.5	189.1	186.7	2192	64.92
12/4/2020 12:42	192.5	189.1	186.8	2194	65
12/4/2020 12:42	192.6	189.2	186.1	2192	64.84
12/4/2020 12:42	192.6	188.8	185.8	2186	64.99
12/4/2020 12:42	192.6	189.2	186	2185	65.08
12/4/2020 12:42	192.6	188.9	186.1	2183	64.99
12/4/2020 12:42	192.6	188.6	186.1	2182	64.84
12/4/2020 12:42	192.6	189.2	185.9	2188	64.96
12/4/2020 12:42	192.6	189	186	2189	65.04
12/4/2020 12:42	192.6	189	186	2185	64.99
12/4/2020 12:42	192.6	189.1	186.8	2186	65.01
12/4/2020 12:42	192.6	188.8	186.8	2182	64.92
12/4/2020 12:42	192.6	189.1	186	2183	65
12/4/2020 12:42	192.6	188.9	186.2	2183	65
12/4/2020 12:42	192.6	189.1	186.2	2186	64.92
12/4/2020 12:42	192.6	189.1	186.1	2185	65
12/4/2020 12:42	192.6	188.8	186.1	2182	65
12/4/2020 12:43	192.5	189.1	185.9	2183	64.92
12/4/2020 12:43	192.6	188.8	186.2	2185	64.92
12/4/2020 12:43	192.6	189.1	186.6	2188	65
12/4/2020 12:43	192.5	189.1	186.5	2189	64.92
12/4/2020 12:43	192.5	188.8	186.1	2189	65
12/4/2020 12:43	192.4	189.1	186	2189	65.08
12/4/2020 12:43	192.4	188.8	186.3	2185	64.95
12/4/2020 12:43	192.4	189.1	186.3	2185	64.88
12/4/2020 12:43	192.4	189.1	186.1	2189	64.92
12/4/2020 12:43	192.4	188.9	185.6	2189	65.04
12/4/2020 12:43	192.4	189.3	186	2190	64.96
12/4/2020 12:43	192.3	189.1	186.3	2192	65
12/4/2020 12:43	192.3	190.4	186.7	2190	65.03
12/4/2020 12:43	192.2	190.1	186.7	2190	64.8
12/4/2020 12:43	192.2	189.8	186.6	2192	65.01
12/4/2020 12:43	192.1	190.1	186.5	2193	64.92
12/4/2020 12:43	192.1	190.4	186.3	2195	65.04
12/4/2020 12:43	192	190.3	186.2	2192	64.96
12/4/2020 12:43	191.9	190.1	186.2	2193	65.03
12/4/2020 12:43	191.9	189.8	186	2193	65.04
12/4/2020 12:43	191.8	190	186.1	2185	65.01
12/4/2020 12:43	191.7	190.1	186.2	2186	64.92
12/4/2020 12:43	191.7	189.7	186.2	2185	64.92
12/4/2020 12:43	191.6	190.1	186.3	2182	65.05
12/4/2020 12:43	191.5	189.6	186.5	2181	64.87

12/4/2020 12:43	191.4	190	186.7	2185	65.08
12/4/2020 12:43	191.3	190.2	187	2184	65.04
12/4/2020 12:43	191.2	189.6	186.8	2185	64.96
12/4/2020 12:43	191.1	190	186.5	2182	64.96
12/4/2020 12:43	191	189.6	185.9	2181	65
12/4/2020 12:43	190.9	190	185.6	2180	64.92
12/4/2020 12:43	190.8	190.4	186	2180	64.96
12/4/2020 12:43	190.8	190.2	186	2185	65.05
12/4/2020 12:43	190.6	190.1	186.2	2185	64.87
12/4/2020 12:43	190.6	188.8	186.2	2184	65
12/4/2020 12:43	190.5	188.7	186	2182	65.04
12/4/2020 12:43	190.3	189	186.2	2181	64.99
12/4/2020 12:43	190.3	188.6	187	2180	64.99
12/4/2020 12:43	190.1	189	186.1	2181	64.96
12/4/2020 12:43	190.1	188.8	185.8	2180	64.91
12/4/2020 12:43	190	189.1	186	2179	65.04
12/4/2020 12:43	189.8	189	185.9	2179	64.96
12/4/2020 12:43	189.7	188.8	186.1	2177	64.92
12/4/2020 12:43	189.6	189.1	185.9	2176	64.99
12/4/2020 12:43	189.6	188.9	185.8	2177	64.97
12/4/2020 12:43	189.4	189	186.2	2178	64.84
12/4/2020 12:43	189.3	189.2	186.5	2176	64.92
12/4/2020 12:43	189.3	189.1	186.8	2181	64.96
12/4/2020 12:43	189.1	189.1	186.3	2180	64.88
12/4/2020 12:43	189	188.8	185.6	2181	65.08
12/4/2020 12:43	188.9	189.3	185.5	2181	64.96
12/4/2020 12:43	188.9	189.1	185.6	2182	64.99
12/4/2020 12:43	188.8	188.9	185.5	2178	64.99
12/4/2020 12:43	188.7	188.9	185.7	2180	65.04
12/4/2020 12:43	188.5	188.7	185.8	2178	64.88
12/4/2020 12:43	188.5	189	185.7	2180	64.92
12/4/2020 12:43	188.3	189.1	186.1	2178	64.96
12/4/2020 12:43	188.3	189.2	185.8	2178	64.91
12/4/2020 12:43	188.1	188.9	185.8	2174	64.92
12/4/2020 12:44	188.1	188.9	185.7	2177	64.96
12/4/2020 12:44	188	189	185.5	2174	65.04
12/4/2020 12:44	187.9	189	185.6	2176	64.92
12/4/2020 12:44	187.8	188.8	185.4	2176	65
12/4/2020 12:44	187.8	189.3	185.4	2174	64.83
12/4/2020 12:44	187.5	189	186	2174	65.04
12/4/2020 12:44	187.5	188.9	186.2	2182	65.05
12/4/2020 12:44	187.3	189.1	186.4	2180	64.92
12/4/2020 12:44	187.3	188.8	185.9	2180	65
12/4/2020 12:44	187.1	189.3	185.4	2178	65
12/4/2020 12:44	187	188.9	185.8	2177	65
12/4/2020 12:44	186.8	189.1	185.7	2176	65
12/4/2020 12:44	186.9	189.1	185.7	2181	65.04

12/4/2020 12:44	186.8	188.8	185.4	2182	64.96
12/4/2020 12:44	186.6	188.9	186	2177	64.92
12/4/2020 12:44	186.4	189.3	186.3	2181	65.04
12/4/2020 12:44	186.4	189.2	185.4	2181	65.04
12/4/2020 12:44	186.3	189.2	185.3	2180	65.08
12/4/2020 12:44	186.2	188.8	185.6	2181	65.08
12/4/2020 12:44	186.1	188.9	185.6	2178	65.03
12/4/2020 12:44	185.9	189.1	185.4	2180	65.04
12/4/2020 12:44	185.9	188.8	185.6	2178	65.08
12/4/2020 12:44	185.7	189	185.1	2175	65.03
12/4/2020 12:44	185.6	188.6	186.1	2174	64.88
12/4/2020 12:44	185.5	188.9	186.3	2180	65.01
12/4/2020 12:44	185.5	189.1	185.5	2179	64.92
12/4/2020 12:44	185.4	188.8	185.1	2178	64.96
12/4/2020 12:44	185.3	189	185.2	2175	64.88
12/4/2020 12:44	185.2	188.7	185.3	2173	64.92
12/4/2020 12:44	185.2	188.8	185.5	2174	64.96
12/4/2020 12:44	185	189.1	185.5	2176	65
12/4/2020 12:44	185	188.9	185.1	2177	65
12/4/2020 12:44	184.9	189.3	185.3	2178	64.96
12/4/2020 12:44	184.7	189	185.3	2178	65.04
12/4/2020 12:44	184.7	188.8	186	2174	65
12/4/2020 12:44	184.5	188.8	185.8	2177	65
12/4/2020 12:44	184.5	188.9	185.1	2174	65.04
12/4/2020 12:44	184.4	189.1	185.1	2174	65
12/4/2020 12:44	184.2	188.9	185.3	2178	64.96
12/4/2020 12:44	184.2	188	185.1	2174	65.04
12/4/2020 12:44	184	187.9	185.3	2169	64.96
12/4/2020 12:44	184	188.1	185.1	2168	65.08
12/4/2020 12:44	183.9	188.1	184.9	2168	65.04
12/4/2020 12:44	183.7	188	185	2164	65.04
12/4/2020 12:44	183.7	188	185.5	2166	64.92
12/4/2020 12:44	183.5	188.1	186	2170	65.16
12/4/2020 12:44	183.4	188.1	185.8	2171	64.99
12/4/2020 12:44	183.4	188.2	184.9	2169	65.08
12/4/2020 12:44	183.3	187.9	185	2171	65.16
12/4/2020 12:44	183.3	188.1	184.9	2169	64.99
12/4/2020 12:44	183.2	188.1	185.3	2167	64.96
12/4/2020 12:44	183.1	188.1	184.9	2169	64.92
12/4/2020 12:44	183.1	188.1	184.7	2167	64.95
12/4/2020 12:44	182.9	188.2	184.8	2170	64.92
12/4/2020 12:44	182.9	188.2	184.6	2168	65.12
12/4/2020 12:44	182.7	188.1	184.5	2165	65
12/4/2020 12:44	182.7	188.1	185.3	2168	64.88
12/4/2020 12:44	182.6	188.1	185.6	2168	64.96
12/4/2020 12:44	182.5	188.1	185.4	2165	64.96
12/4/2020 12:44	182.5	188.1	185.4	2164	65.04

12/4/2020 12:45	182.5	188.2	184.8	2166	65.09
12/4/2020 12:45	182.4	188.1	184.5	2164	64.87
12/4/2020 12:45	182.2	188.2	184.7	2162	64.92
12/4/2020 12:45	182.2	188.1	184.8	2167	65.05
12/4/2020 12:45	182.3	188.2	184.8	2171	64.96
12/4/2020 12:45	182.2	188.2	184.9	2167	64.96
12/4/2020 12:45	182.1	188.1	184.6	2169	65.05
12/4/2020 12:45	182.1	188	184.7	2167	64.96
12/4/2020 12:45	182	188.2	184.6	2166	65
12/4/2020 12:45	182	187.9	184.6	2167	65.04
12/4/2020 12:45	182	188.1	184.6	2166	64.99
12/4/2020 12:45	181.9	188.1	184.7	2169	65
12/4/2020 12:45	181.9	188	185	2168	65.08
12/4/2020 12:45	181.9	188.1	185.5	2167	64.84
12/4/2020 12:45	181.8	187.9	185.3	2168	64.93
12/4/2020 12:45	181.8	188	184.8	2163	64.93
12/4/2020 12:45	181.8	188	184.1	2164	65.04
12/4/2020 12:45	181.8	187	184.5	2163	65.04
12/4/2020 12:45	181.8	187.1	184.6	2168	65.01
12/4/2020 12:45	181.7	187	184.5	2168	64.88
12/4/2020 12:45	181.7	186.8	184.2	2166	65.01
12/4/2020 12:45	181.7	186.9	184.9	2165	64.92
12/4/2020 12:45	181.7	187	185	2170	64.92
12/4/2020 12:45	181.7	187.4	184.2	2171	64.88
12/4/2020 12:45	181.6	187.2	184.4	2171	65.08
12/4/2020 12:45	181.6	186.7	184.4	2171	64.96
12/4/2020 12:45	181.6	186.9	184.1	2169	64.92
12/4/2020 12:45	181.6	187	184.5	2168	64.96
12/4/2020 12:45	181.6	187.1	185.3	2161	64.96
12/4/2020 12:45	181.6	187	184.3	2163	64.96
12/4/2020 12:45	181.5	187.2	184.2	2163	65.09
12/4/2020 12:45	181.6	187	184.5	2165	64.92
12/4/2020 12:45	181.5	186.9	184.4	2168	64.96
12/4/2020 12:45	181.4	186.9	183.9	2169	64.96
12/4/2020 12:45	181.4	187.2	184.8	2169	64.96
12/4/2020 12:45	181.4	187.2	184.9	2167	65
12/4/2020 12:45	181.4	187.4	183.9	2171	64.96
12/4/2020 12:45	181.3	187	184.3	2169	65
12/4/2020 12:45	181.3	187.1	184.4	2171	64.96
12/4/2020 12:45	181.2	186.7	184.5	2169	64.96
12/4/2020 12:45	181.2	186.8	183.9	2170	64.96
12/4/2020 12:45	181.1	187.2	184.6	2167	64.96
12/4/2020 12:45	181.1	186.9	184.8	2167	65.12
12/4/2020 12:45	181.1	186.8	184.1	2169	64.99
12/4/2020 12:45	181	187.1	184.1	2167	64.99
12/4/2020 12:45	180.9	186.8	184.3	2171	65.04
12/4/2020 12:45	180.9	187.2	184	2169	64.88

12/4/2020 12:45	180.9	187	183.8	2167	64.99
12/4/2020 12:45	180.8	186.8	184.5	2167	64.92
12/4/2020 12:45	180.9	187.3	184.7	2172	64.96
12/4/2020 12:45	180.8	186.8	184.1	2174	65
12/4/2020 12:45	180.7	187.1	183.9	2171	65.08
12/4/2020 12:45	180.7	186.9	184.1	2167	64.84
12/4/2020 12:45	180.7	186.8	184.1	2171	65.04
12/4/2020 12:45	180.7	186.7	183.7	2165	64.92
12/4/2020 12:45	180.6	186.1	184.4	2165	64.92
12/4/2020 12:45	180.5	186.3	184.8	2161	64.84
12/4/2020 12:45	180.5	185.7	183.6	2165	64.88
12/4/2020 12:45	180.5	185.9	183.8	2163	64.92
12/4/2020 12:46	180.4	185.5	183.9	2163	64.92
12/4/2020 12:46	180.4	186.1	184.1	2166	65.08
12/4/2020 12:46	180.3	186.3	183.8	2165	64.88
12/4/2020 12:46	180.3	186.1	184.2	2167	64.92
12/4/2020 12:46	180.3	185.9	184.6	2164	64.88
12/4/2020 12:46	180.2	186.1	183.9	2165	65.08
12/4/2020 12:46	180.3	185.8	183.5	2167	64.99
12/4/2020 12:46	180.2	186.4	183.8	2169	64.87
12/4/2020 12:46	180.3	186.1	183.9	2169	65
12/4/2020 12:46	180.3	186.4	183.8	2168	65.04
12/4/2020 12:46	180.3	185.8	183.7	2169	64.92
12/4/2020 12:46	180.3	185.7	184.5	2173	65.04
12/4/2020 12:46	180.3	186.3	184.1	2176	65
12/4/2020 12:46	180.3	186.1	183.7	2174	64.96
12/4/2020 12:46	180.2	186.5	183.6	2174	65.04
12/4/2020 12:46	180.3	185.8	183.9	2175	64.96
12/4/2020 12:46	180.3	185.6	183.9	2169	65
12/4/2020 12:46	180.3	186	183.4	2171	65
12/4/2020 12:46	180.3	185.7	183.5	2168	65.04
12/4/2020 12:46	180.3	185.7	184.1	2171	64.92
12/4/2020 12:46	180.4	185.8	184.5	2168	65.12
12/4/2020 12:46	180.4	185.8	184.2	2168	64.88
12/4/2020 12:46	180.4	186.2	183.3	2164	65.08
12/4/2020 12:46	180.5	186.3	183.2	2167	65.01
12/4/2020 12:46	180.5	185.5	183.6	2163	64.88
12/4/2020 12:46	180.5	186	183.5	2169	64.96
12/4/2020 12:46	180.5	185.8	183.9	2172	65.12
12/4/2020 12:46	180.6	186	183.5	2175	64.99
12/4/2020 12:46	180.6	186.2	183.5	2177	64.88
12/4/2020 12:46	180.7	185.6	183.5	2175	65.08
12/4/2020 12:46	180.7	186.3	183.5	2177	64.8
12/4/2020 12:46	180.7	186	183.9	2174	64.92
12/4/2020 12:46	180.7	186.1	184.5	2178	64.93
12/4/2020 12:46	180.7	186.3	184.1	2174	64.99
12/4/2020 12:46	180.8	185.6	183.5	2174	64.99

12/4/2020 12:46	180.8	186.1	183	2171	65.08
12/4/2020 12:46	180.9	185.6	183.4	2169	64.88
12/4/2020 12:46	180.9	186.1	183.4	2169	64.96
12/4/2020 12:46	180.9	186.3	183.6	2167	65.05
12/4/2020 12:46	180.9	185.3	183.7	2168	64.96
12/4/2020 12:46	180.9	185.3	183.7	2169	64.96
12/4/2020 12:46	180.9	184.8	183.5	2168	64.96
12/4/2020 12:46	181	185.2	183.5	2170	64.96
12/4/2020 12:46	181.2	185.1	183.5	2169	65
12/4/2020 12:46	181.1	184.9	183.4	2167	65.05
12/4/2020 12:46	181.2	185.4	184.1	2167	64.96
12/4/2020 12:46	181.2	185.2	184.2	2165	64.87
12/4/2020 12:46	181.3	184.9	183.8	2169	65.04
12/4/2020 12:46	181.3	185	183.5	2171	65
12/4/2020 12:46	181.3	184.6	183.2	2172	65
12/4/2020 12:46	181.4	185.4	183	2174	65.04
12/4/2020 12:46	181.5	184.8	183	2169	65.04
12/4/2020 12:46	181.4	185.5	183.3	2167	65
12/4/2020 12:46	181.5	185.2	183.7	2168	65.09
12/4/2020 12:46	181.5	184.7	183.7	2169	64.88
12/4/2020 12:46	181.6	185.1	183.7	2170	64.96
12/4/2020 12:46	181.6	184.6	183.7	2171	65.04
12/4/2020 12:46	181.7	184.9	183.5	2169	64.99
12/4/2020 12:46	181.7	185.2	183.4	2167	64.92
12/4/2020 12:46	181.8	184.7	183.7	2167	65.08
12/4/2020 12:47	181.8	185	183.4	2162	64.88
12/4/2020 12:47	181.8	184.8	183.5	2164	64.88
12/4/2020 12:47	181.8	185.5	183.4	2170	64.88
12/4/2020 12:47	181.9	185.4	183.1	2174	65.04
12/4/2020 12:47	181.9	184.8	183.3	2173	65.04
12/4/2020 12:47	181.9	185.1	183.1	2169	64.96
12/4/2020 12:47	181.9	184.7	183.2	2171	65.04
12/4/2020 12:47	182.1	185.4	183.4	2171	64.88
12/4/2020 12:47	182.1	185.3	183.9	2171	65.08
12/4/2020 12:47	182.1	185.2	184.3	2171	64.99
12/4/2020 12:47	182.1	185.1	183.8	2171	64.84
12/4/2020 12:47	182.1	184.8	183.2	2174	64.92
12/4/2020 12:47	182.2	185.4	183.4	2170	64.88
12/4/2020 12:47	182.2	185.3	183.5	2167	64.92
12/4/2020 12:47	182.2	184.7	183.4	2174	64.96
12/4/2020 12:47	182.2	185.2	183.2	2170	64.92
12/4/2020 12:47	182.2	184.6	183	2167	64.87
12/4/2020 12:47	182.3	184.8	183.4	2171	65.09
12/4/2020 12:47	182.4	185.3	184.4	2169	64.92
12/4/2020 12:47	182.4	184.9	183.5	2167	64.96
12/4/2020 12:47	182.4	185.5	183.2	2167	65.09
12/4/2020 12:47	182.5	185.1	183.4	2165	64.96

12/4/2020 12:47	182.6	184.8	183.5	2160	64.92
12/4/2020 12:47	182.6	185.5	183.5	2159	65.08
12/4/2020 12:47	182.6	184.9	183.2	2162	64.96
12/4/2020 12:47	182.6	185	183	2167	64.96
12/4/2020 12:47	182.6	185.3	184.2	2170	65
12/4/2020 12:47	182.7	184.7	183.7	2171	65.04
12/4/2020 12:47	182.7	185.2	183.2	2171	64.92
12/4/2020 12:47	182.7	184.8	183.2	2176	64.96
12/4/2020 12:47	182.7	185.3	183.4	2174	64.92
12/4/2020 12:47	182.7	185.4	183.4	2174	64.88
12/4/2020 12:47	182.8	185.4	182.9	2178	65.08
12/4/2020 12:47	182.7	185	183.1	2178	65.04
12/4/2020 12:47	182.7	184.8	183.7	2178	65.04
12/4/2020 12:47	182.7	185.1	183.9	2175	65.04
12/4/2020 12:47	182.6	185.2	183.6	2177	65.08
12/4/2020 12:47	182.6	185.3	182.8	2171	64.99
12/4/2020 12:47	182.7	185	183.2	2169	64.97
12/4/2020 12:47	182.7	184.8	183.7	2171	65.03
12/4/2020 12:47	182.6	185	183.1	2167	64.88
12/4/2020 12:47	182.6	184.6	183	2166	65.08
12/4/2020 12:47	182.6	185.3	183	2167	64.92
12/4/2020 12:47	182.6	185.3	183.6	2167	64.95
12/4/2020 12:47	182.6	185.1	183.9	2166	65.08
12/4/2020 12:47	182.6	185.2	183.6	2166	65.03
12/4/2020 12:47	182.6	184.6	183	2165	65.03
12/4/2020 12:47	182.6	184.7	183.1	2167	64.88
12/4/2020 12:47	182.6	185.3	183.1	2167	65.04
12/4/2020 12:47	182.6	184.8	183.4	2167	64.92
12/4/2020 12:47	182.7	184.8	183.4	2171	64.92
12/4/2020 12:47	182.6	185.3	183.2	2168	64.92
12/4/2020 12:47	182.7	184.8	183	2167	64.95
12/4/2020 12:47	182.7	185.3	183.2	2166	65.04
12/4/2020 12:47	182.7	184.7	183	2167	64.99
12/4/2020 12:47	182.8	184.9	183.1	2167	64.96
12/4/2020 12:47	182.8	185	182.9	2167	64.96
12/4/2020 12:47	182.9	184.6	183.3	2167	64.92
12/4/2020 12:47	182.9	185.3	184.1	2166	64.92
12/4/2020 12:48	182.9	184.9	183.8	2165	65
12/4/2020 12:48	183	185.4	184	2162	64.96
12/4/2020 12:48	183	184.9	183.7	2163	64.88
12/4/2020 12:48	183.1	184.9	183.3	2165	65
12/4/2020 12:48	183.1	185.3	183.2	2167	64.96
12/4/2020 12:48	183.1	184.9	183.2	2167	64.92
12/4/2020 12:48	183.2	185.4	183.1	2169	65
12/4/2020 12:48	183.2	184.8	183	2167	64.96
12/4/2020 12:48	183.3	184.9	183	2164	64.92
12/4/2020 12:48	183.4	185.3	183	2167	65.04

12/4/2020 12:48	183.4	185.3	183.3	2166	64.96
12/4/2020 12:48	183.6	185.3	183.4	2163	64.83
12/4/2020 12:48	183.5	184.8	183.8	2164	65.04
12/4/2020 12:48	183.7	184.9	183.8	2162	64.96
12/4/2020 12:48	183.7	185.2	183.7	2160	64.99
12/4/2020 12:48	183.7	185.1	183.8	2163	65.08
12/4/2020 12:48	183.8	184.6	183.5	2163	64.83
12/4/2020 12:48	183.9	184.7	183.2	2156	65.04
12/4/2020 12:48	184	184.9	183	2163	65.08
12/4/2020 12:48	184.1	185.3	183	2164	64.92
12/4/2020 12:48	184.2	185.2	183	2160	64.88
12/4/2020 12:48	184.2	185.4	183.2	2167	65.08
12/4/2020 12:48	184.3	184.8	183.4	2167	64.95
12/4/2020 12:48	184.4	185.1	183.5	2168	64.88
12/4/2020 12:48	184.4	185.4	183.3	2168	64.96
12/4/2020 12:48	184.5	185.2	183.1	2164	64.91
12/4/2020 12:48	184.6	184.6	183.1	2167	65.04
12/4/2020 12:48	184.6	184.8	182.9	2168	65.04
12/4/2020 12:48	184.6	184.8	183	2171	64.84
12/4/2020 12:48	184.8	184.9	183.2	2171	64.84
12/4/2020 12:48	184.8	184.6	183.2	2171	65.08
12/4/2020 12:48	184.9	184.6	183.3	2167	64.99
12/4/2020 12:48	185	185.1	183.4	2167	64.92
12/4/2020 12:48	185	184.6	183.2	2166	65.08
12/4/2020 12:48	185.2	184.9	183.5	2167	64.99
12/4/2020 12:48	185.2	184.7	183.2	2172	65.04
12/4/2020 12:48	185.3	185.3	183.5	2171	64.92
12/4/2020 12:48	185.4	185.2	183.5	2167	64.99
12/4/2020 12:48	185.4	184.8	183.2	2165	65.04
12/4/2020 12:48	185.5	185.1	183.5	2172	65.04
12/4/2020 12:48	185.6	184.8	183.4	2172	64.99
12/4/2020 12:48	185.6	184.6	183.1	2171	64.96
12/4/2020 12:48	185.7	185.4	183.6	2178	65.04
12/4/2020 12:48	185.8	184.9	183.5	2178	65.04
12/4/2020 12:48	185.8	185.2	183.6	2178	65
12/4/2020 12:48	185.8	185.2	183.5	2180	64.88
12/4/2020 12:48	185.9	184.7	183.4	2177	64.96
12/4/2020 12:48	185.9	185.4	183.2	2178	64.92
12/4/2020 12:48	185.9	184.9	183.5	2178	64.96
12/4/2020 12:48	186	184.9	183.2	2176	64.99
12/4/2020 12:48	186	185	183.1	2177	64.96
12/4/2020 12:48	186	184.6	183.1	2177	64.96
12/4/2020 12:48	186	184.8	183	2176	65
12/4/2020 12:48	186.1	184.9	183	2175	64.96
12/4/2020 12:48	186	185.1	183	2174	64.99
12/4/2020 12:48	186.1	185.3	183	2174	64.92
12/4/2020 12:48	186.1	184.6	183.4	2173	64.96

12/4/2020 12:48	186.1	185.1	183.3	2168	64.96
12/4/2020 12:48	186.1	184.8	183.4	2169	64.92
12/4/2020 12:48	186.1	185.1	183.4	2169	64.96
12/4/2020 12:49	186.1	185.4	183	2173	65.04
12/4/2020 12:49	186.1	184.6	183	2171	64.96
12/4/2020 12:49	186	185.3	182.9	2171	64.83
12/4/2020 12:49	186	184.7	183.1	2175	65
12/4/2020 12:49	185.9	185.3	183	2169	64.96
12/4/2020 12:49	185.9	185.2	182.7	2175	64.96
12/4/2020 12:49	185.9	184.6	183.2	2178	65.04
12/4/2020 12:49	185.8	185.1	183.4	2176	64.96
12/4/2020 12:49	185.9	184.8	183.4	2174	64.99
12/4/2020 12:49	185.7	185.3	183.5	2174	65.08
12/4/2020 12:49	185.7	185.1	183.5	2174	64.91
12/4/2020 12:49	185.7	184.6	183.3	2177	64.96
12/4/2020 12:49	185.7	184.9	183.5	2182	65.05
12/4/2020 12:49	185.5	184.7	183.2	2181	64.96
12/4/2020 12:49	185.5	185.4	183.5	2180	64.96
12/4/2020 12:49	185.3	185.2	183.2	2183	64.96
12/4/2020 12:49	185.4	184.8	182.9	2181	64.88
12/4/2020 12:49	185.2	185	182.8	2182	64.99
12/4/2020 12:49	185.2	184.8	182.9	2178	65.08
12/4/2020 12:49	185.1	185.3	182.9	2180	64.88
12/4/2020 12:49	185	184.7	182.8	2178	64.92
12/4/2020 12:49	185	184.6	183	2178	65.01
12/4/2020 12:49	184.9	185.4	183	2178	64.84
12/4/2020 12:49	184.8	185.2	183.4	2179	64.88
12/4/2020 12:49	184.8	185.3	183.1	2177	65.16
12/4/2020 12:49	184.6	185.3	183.2	2176	65.08
12/4/2020 12:49	184.7	184.6	183	2182	65.08
12/4/2020 12:49	184.5	185.2	183	2185	64.96
12/4/2020 12:49	184.5	184.6	183.2	2181	65.04
12/4/2020 12:49	184.5	184.6	183.8	2182	65.08
12/4/2020 12:49	184.4	185.3	183.5	2179	64.93
12/4/2020 12:49	184.4	185.1	182.5	2184	64.84
12/4/2020 12:49	184.2	185.3	182.5	2180	64.99
12/4/2020 12:49	184.2	184.9	183	2181	65.12
12/4/2020 12:49	184.2	184.7	183.1	2181	65.04
12/4/2020 12:49	184.2	185.4	183	2178	65
12/4/2020 12:49	184.2	184.9	183	2183	65.04
12/4/2020 12:49	184.1	185.3	182.7	2178	64.96
12/4/2020 12:49	184.1	185.1	182.7	2176	64.92
12/4/2020 12:49	184	184.6	183.5	2182	64.92
12/4/2020 12:49	184	185.3	183.7	2183	64.92
12/4/2020 12:49	184	184.8	183.7	2184	64.96
12/4/2020 12:49	184	185.1	182.8	2184	65.09
12/4/2020 12:49	183.9	185	182.3	2187	64.96

12/4/2020 12:49	184	184.6	182.7	2185	64.92
12/4/2020 12:49	183.9	184.9	182.8	2185	65
12/4/2020 12:49	184	184.8	182.9	2181	65
12/4/2020 12:49	184	185.1	183	2182	65.04
12/4/2020 12:49	183.9	185.1	182.5	2185	65
12/4/2020 12:49	183.9	184.8	182.6	2180	64.91
12/4/2020 12:49	183.9	185.1	183	2180	65.04
12/4/2020 12:49	183.9	184.9	183.5	2180	65.08
12/4/2020 12:49	183.8	184.9	183.7	2180	65.04
12/4/2020 12:49	183.9	185.1	183.2	2174	64.92
12/4/2020 12:49	183.9	184.6	182.5	2173	64.96
12/4/2020 12:49	183.9	184.9	182.4	2169	64.92
12/4/2020 12:49	183.9	184.8	182.3	2171	65.04
12/4/2020 12:49	183.9	184.9	182.5	2172	65.16
12/4/2020 12:49	183.9	185.1	182.8	2170	65.03
12/4/2020 12:50	183.9	184.7	182.6	2169	65.04
12/4/2020 12:50	183.9	185.3	182.7	2170	64.93
12/4/2020 12:50	183.9	184.9	182.5	2166	64.92
12/4/2020 12:50	183.7	184.9	182.7	2168	65.04
12/4/2020 12:50	183.8	185.3	183	2168	65.01
12/4/2020 12:50	183.8	184.6	182.7	2169	65.04
12/4/2020 12:50	183.7	185.3	182.5	2169	64.92
12/4/2020 12:50	183.7	184.9	182.5	2168	65.08
12/4/2020 12:50	183.7	185.3	182.7	2172	65.04
12/4/2020 12:50	183.6	184.1	182.8	2174	65.04
12/4/2020 12:50	183.6	183.9	182.7	2174	65.04
12/4/2020 12:50	183.5	184.2	182.6	2167	64.91
12/4/2020 12:50	183.5	184.1	182.6	2166	65
12/4/2020 12:50	183.4	184.2	182.4	2166	65
12/4/2020 12:50	183.3	184.2	182.5	2164	64.92
12/4/2020 12:50	183.3	184	182.7	2165	64.96
12/4/2020 12:50	183.3	184.2	182.5	2164	65
12/4/2020 12:50	183.2	184.2	182.5	2165	64.92
12/4/2020 12:50	183.1	184.2	182.7	2172	64.96
12/4/2020 12:50	183.1	184	182.5	2172	65.12
12/4/2020 12:50	183.1	183.9	182.7	2170	64.99
12/4/2020 12:50	183	184.2	182.6	2171	64.92
12/4/2020 12:50	182.9	184	182.2	2171	65.08
12/4/2020 12:50	182.9	184.1	182.2	2173	65.08
12/4/2020 12:50	182.9	183.9	182.1	2171	65.04
12/4/2020 12:50	182.8	183.9	182.3	2171	64.92
12/4/2020 12:50	182.8	184.2	183.2	2174	65.04
12/4/2020 12:50	182.7	184	183	2171	65.08
12/4/2020 12:50	182.7	183.9	182.4	2174	65
12/4/2020 12:50	182.7	184.1	182	2177	65.04
12/4/2020 12:50	182.7	184	182.2	2172	65.04
12/4/2020 12:50	182.7	184.1	182.4	2170	64.96

12/4/2020 12:50	182.7	184.1	182.5	2164	65.04
12/4/2020 12:50	182.6	184.1	182.3	2166	64.96
12/4/2020 12:50	182.6	184.1	182.1	2167	64.92
12/4/2020 12:50	182.7	183.8	182.2	2162	64.96
12/4/2020 12:50	182.7	184	183.1	2162	64.96
12/4/2020 12:50	182.7	183.9	183	2164	65
12/4/2020 12:50	182.6	184.1	182.1	2163	65.04
12/4/2020 12:50	182.7	184.1	182.1	2164	65.04
12/4/2020 12:50	182.7	184.3	182.1	2163	64.96
12/4/2020 12:50	182.8	184.2	182.3	2156	65.04
12/4/2020 12:50	182.8	184.1	182.3	2158	64.92
12/4/2020 12:50	182.8	184.1	182	2160	65
12/4/2020 12:50	182.9	183.9	182.1	2160	64.88
12/4/2020 12:50	182.9	184.2	182.8	2159	64.84
12/4/2020 12:50	182.9	184.2	182.8	2160	65.08
12/4/2020 12:50	183	183.9	182.1	2160	65.04
12/4/2020 12:50	183.1	183.9	182.1	2163	65.04
12/4/2020 12:50	183.1	184	182.3	2163	65.08
12/4/2020 12:50	183.1	184.1	182.2	2161	64.96
12/4/2020 12:50	183.2	184.1	182	2156	65.04
12/4/2020 12:50	183.2	184.1	181.6	2159	64.92
12/4/2020 12:50	183.3	183.9	182.8	2154	64.96
12/4/2020 12:50	183.4	184.1	182.8	2160	64.88
12/4/2020 12:50	183.5	183.8	182.3	2163	64.92
12/4/2020 12:50	183.5	184.2	182	2165	65
12/4/2020 12:50	183.7	184	182.2	2160	64.96
12/4/2020 12:50	183.7	184.2	182.3	2163	65
12/4/2020 12:51	183.9	184.2	182.2	2165	65
12/4/2020 12:51	184	183.9	181.9	2163	65
12/4/2020 12:51	184	184.2	182	2163	65
12/4/2020 12:51	184.1	183.9	181.8	2159	64.88
12/4/2020 12:51	184.2	183.9	182.5	2159	64.96
12/4/2020 12:51	184.3	184.1	183.3	2165	65.08
12/4/2020 12:51	184.4	184	182.7	2164	65
12/4/2020 12:51	184.4	184.1	182.2	2159	64.96
12/4/2020 12:51	184.6	183.9	181.8	2162	64.96
12/4/2020 12:51	184.6	184.1	182.1	2167	65.04
12/4/2020 12:51	184.7	184.2	182.5	2167	64.99
12/4/2020 12:51	184.9	183.9	182.2	2169	65.12
12/4/2020 12:51	184.8	184.2	182	2169	65.04
12/4/2020 12:51	185	183.9	182	2168	65.08
12/4/2020 12:51	185.1	184.1	181.9	2171	64.88
12/4/2020 12:51	185.1	184.2	182.8	2173	64.96
12/4/2020 12:51	185.2	184.1	183.1	2174	65.12
12/4/2020 12:51	185.3	184.1	182.6	2176	64.93
12/4/2020 12:51	185.3	184.1	182	2176	65.03
12/4/2020 12:51	185.4	184	182.1	2176	64.84

12/4/2020 12:51	185.5	184.4	182.2	2178	65.01
12/4/2020 12:51	185.5	184.2	182.4	2179	64.84
12/4/2020 12:51	185.6	183.9	182.1	2181	65.08
12/4/2020 12:51	185.6	183.9	182	2176	65.01
12/4/2020 12:51	185.7	184.1	182	2172	64.99
12/4/2020 12:51	185.7	184.1	181.9	2169	65.04
12/4/2020 12:51	185.8	184.1	182.5	2173	65.05
12/4/2020 12:51	185.8	184.2	182.7	2170	64.96
12/4/2020 12:51	185.9	184.2	182.8	2171	64.96
12/4/2020 12:51	185.9	183.9	182.6	2171	65
12/4/2020 12:51	185.9	184.1	182.2	2171	64.96
12/4/2020 12:51	186	184	182.1	2171	65.04
12/4/2020 12:51	186	184.2	182	2174	65
12/4/2020 12:51	186	184	182.1	2171	64.99
12/4/2020 12:51	186.1	184.1	182.1	2171	65
12/4/2020 12:51	186	184.2	182	2171	65
12/4/2020 12:51	186.1	184.1	182.2	2171	64.92
12/4/2020 12:51	186.2	184	182.2	2173	64.99
12/4/2020 12:51	186.2	184.2	182.1	2174	65.12
12/4/2020 12:51	186.2	184.1	182	2174	65.04
12/4/2020 12:51	186.2	184.1	181.8	2171	64.96
12/4/2020 12:51	186.3	183.9	181.8	2173	65.01
12/4/2020 12:51	186.3	184	182.3	2169	64.88
12/4/2020 12:51	186.3	184.2	182.9	2170	64.88
12/4/2020 12:51	186.3	184.2	183	2170	65.08
12/4/2020 12:51	186.4	185.3	182.5	2170	64.88
12/4/2020 12:51	186.4	185.1	181.8	2174	64.88
12/4/2020 12:51	186.3	184.6	181.9	2174	65.12
12/4/2020 12:51	186.3	184.8	182.1	2175	65.04
12/4/2020 12:51	186.4	184.9	182.2	2178	64.99
12/4/2020 12:51	186.3	185.4	182.3	2180	65.08
12/4/2020 12:51	186.3	184.8	182.1	2181	64.88
12/4/2020 12:51	186.4	184.7	182.8	2181	64.96
12/4/2020 12:51	186.4	184.8	182.7	2177	65.08
12/4/2020 12:51	186.4	184.8	182	2174	64.96
12/4/2020 12:51	186.4	184.9	182.1	2176	64.96
12/4/2020 12:51	186.3	185.1	182.4	2179	65.09
12/4/2020 12:51	186.3	184.6	182	2178	64.96
12/4/2020 12:51	186.4	184.9	182.3	2174	64.87
12/4/2020 12:51	186.4	184.6	183	2178	65
12/4/2020 12:52	186.4	185	182	2177	64.91
12/4/2020 12:52	186.3	185.3	182	2176	64.96
12/4/2020 12:52	186.3	184.6	182	2180	65.04
12/4/2020 12:52	186.3	185.4	182.2	2181	65.04
12/4/2020 12:52	186.3	184.9	182.2	2180	64.96
12/4/2020 12:52	186.3	185.3	181.9	2178	65.08
12/4/2020 12:52	186.3	184.8	183	2181	65.04

12/4/2020 12:52	186.3	184.8	182.9	2184	64.83
12/4/2020 12:52	186.2	185.3	181.8	2187	65.21
12/4/2020 12:52	186.3	184.7	182.1	2184	64.88
12/4/2020 12:52	186.2	185.3	182.2	2178	64.99
12/4/2020 12:52	186.3	185.3	182	2182	64.96
12/4/2020 12:52	186.2	184.7	181.9	2184	64.88
12/4/2020 12:52	186.2	184.9	182.6	2184	64.88
12/4/2020 12:52	186.3	184.6	183	2184	65.08
12/4/2020 12:52	186.2	184.8	182	2185	64.96
12/4/2020 12:52	186.2	185.3	181.9	2180	64.99
12/4/2020 12:52	186.1	184.7	182.3	2182	65.12
12/4/2020 12:52	186.2	185	182	2181	65
12/4/2020 12:52	186.1	184.6	182	2185	64.99
12/4/2020 12:52	186	184.8	182.7	2184	65.09
12/4/2020 12:52	186	185.3	183	2185	65
12/4/2020 12:52	186	184.6	182	2182	65
12/4/2020 12:52	186	184.9	182.1	2189	65.05
12/4/2020 12:52	185.9	184.9	182.1	2185	64.96
12/4/2020 12:52	185.9	184.7	182.3	2185	64.96
12/4/2020 12:52	185.9	185.4	182.2	2185	65
12/4/2020 12:52	185.9	184.9	181.8	2181	64.92
12/4/2020 12:52	185.8	185.3	182.5	2178	64.92
12/4/2020 12:52	185.8	185.2	183	2181	65.05
12/4/2020 12:52	185.8	185.4	182.3	2181	65.04
12/4/2020 12:52	185.7	184.8	181.8	2178	64.92
12/4/2020 12:52	185.7	184.6	182.1	2176	64.96
12/4/2020 12:52	185.6	184.9	182	2181	64.96
12/4/2020 12:52	185.6	184.9	182.1	2180	64.96
12/4/2020 12:52	185.5	184.8	181.9	2178	64.93
12/4/2020 12:52	185.5	185.3	182.1	2178	64.88
12/4/2020 12:52	185.5	184.7	182.8	2178	65.04
12/4/2020 12:52	185.4	184.9	182.6	2175	64.96
12/4/2020 12:52	185.4	184.6	181.6	2175	64.84
12/4/2020 12:52	185.3	185.2	181.6	2174	65.04
12/4/2020 12:52	185.4	185	182.1	2175	65.01
12/4/2020 12:52	185.3	184.8	182.2	2176	64.84
12/4/2020 12:52	185.2	185.4	182.1	2171	64.88
12/4/2020 12:52	185.3	184.9	182	2174	65.04
12/4/2020 12:52	185.2	185.1	181.6	2171	64.99
12/4/2020 12:52	185.2	185.4	181.8	2175	64.99
12/4/2020 12:52	185.2	184.8	182.5	2174	65.01
12/4/2020 12:52	185	184.6	182.8	2174	64.99
12/4/2020 12:52	185	185.1	182.8	2174	65.04
12/4/2020 12:52	185	184.6	182.9	2176	65.08
12/4/2020 12:52	184.9	185.1	182.6	2174	64.88
12/4/2020 12:52	184.9	184.9	183	2171	64.99
12/4/2020 12:52	184.8	184.8	182.8	2175	65.04

12/4/2020 12:52	184.8	185.1	182.7	2173	64.96
12/4/2020 12:52	184.7	184.6	182.7	2174	64.96
12/4/2020 12:52	184.8	185.1	182.2	2180	65.09
12/4/2020 12:52	184.7	184.6	181.8	2176	64.92
12/4/2020 12:52	184.6	185.4	181.8	2172	64.96
12/4/2020 12:53	184.6	185	181.9	2175	65
12/4/2020 12:53	184.4	184.6	182	2175	64.92
12/4/2020 12:53	184.5	185.3	181.7	2175	65
12/4/2020 12:53	184.5	184.9	181.6	2174	64.92
12/4/2020 12:53	184.3	184.8	182.7	2178	64.92
12/4/2020 12:53	184.3	185.4	182.3	2177	64.96
12/4/2020 12:53	184.3	184.9	181.7	2172	65.12
12/4/2020 12:53	184.2	185.4	181.8	2177	64.91
12/4/2020 12:53	184.1	185.2	182	2178	64.92
12/4/2020 12:53	184.1	184.6	181.6	2181	65.05
12/4/2020 12:53	184.1	185.3	181.6	2178	64.96
12/4/2020 12:53	184	184.9	181.7	2184	64.92
12/4/2020 12:53	183.9	185	182.6	2183	64.84
12/4/2020 12:53	183.9	185.3	182.1	2180	65.04
12/4/2020 12:53	183.9	185.4	181.6	2182	65.01
12/4/2020 12:53	183.8	185	181.8	2182	64.88
12/4/2020 12:53	183.7	184.7	181.8	2181	65.12
12/4/2020 12:53	183.7	184.8	181.6	2183	64.92
12/4/2020 12:53	183.6	184.7	181.5	2178	64.92
12/4/2020 12:53	183.5	184.6	182.3	2178	64.99
12/4/2020 12:53	183.5	185.2	182.6	2183	65.08
12/4/2020 12:53	183.5	184.6	181.6	2178	64.92
12/4/2020 12:53	183.4	185.3	181.6	2174	64.96
12/4/2020 12:53	183.3	184.7	181.9	2180	65.05
12/4/2020 12:53	183.3	184	181.9	2177	64.95
12/4/2020 12:53	183.2	184.3	181.5	2175	65
12/4/2020 12:53	183.2	184.1	181.3	2178	65
12/4/2020 12:53	183.1	184.2	182	2178	64.96
12/4/2020 12:53	183.1	184	182.4	2176	65
12/4/2020 12:53	183	183.9	182.1	2181	65.04
12/4/2020 12:53	182.9	184.2	181.4	2181	65
12/4/2020 12:53	182.9	184.2	181.5	2178	64.92
12/4/2020 12:53	182.8	184.2	181.7	2178	65.09
12/4/2020 12:53	182.8	184.2	181.6	2174	64.96
12/4/2020 12:53	182.7	184.2	181.7	2174	65.04
12/4/2020 12:53	182.6	184.1	181.3	2173	65.05
12/4/2020 12:53	182.6	183.9	181.4	2174	64.92
12/4/2020 12:53	182.4	184.2	181.4	2174	65.04
12/4/2020 12:53	182.5	184.1	182	2171	64.88
12/4/2020 12:53	182.3	184.1	182.2	2172	64.99
12/4/2020 12:53	182.3	184.2	181.7	2177	64.96
12/4/2020 12:53	182.2	183.9	181.3	2177	65.08

12/4/2020 12:53	182.2	184.1	181.4	2175	64.99
12/4/2020 12:53	182.2	184.1	181.3	2177	65.08
12/4/2020 12:53	182.2	184.1	181.5	2177	64.97
12/4/2020 12:53	182	184.1	181.4	2176	64.92
12/4/2020 12:53	182	184.1	181.4	2179	65.08
12/4/2020 12:53	182	184	181.4	2179	64.96
12/4/2020 12:53	182	184.3	181.2	2181	64.99
12/4/2020 12:53	181.9	184.1	181	2183	64.88
12/4/2020 12:53	181.8	184.1	181.2	2187	65.04
12/4/2020 12:53	181.8	183.9	181.6	2183	64.92
12/4/2020 12:53	181.8	184.3	182.1	2180	64.91
12/4/2020 12:53	181.7	183.9	182	2181	65.08
12/4/2020 12:53	181.7	184	181.5	2178	64.96
12/4/2020 12:53	181.7	184.2	181.2	2181	64.92
12/4/2020 12:53	181.6	184.2	181.1	2178	65.09
12/4/2020 12:53	181.6	184.1	181.1	2171	64.87
12/4/2020 12:53	181.5	184.1	181.4	2171	65
12/4/2020 12:53	181.4	184.1	181.3	2171	65.04
12/4/2020 12:54	181.4	184.1	181.4	2169	65
12/4/2020 12:54	181.4	184.1	181.4	2169	64.92
12/4/2020 12:54	181.3	184.1	181.2	2174	64.96
12/4/2020 12:54	181.3	184.1	181.3	2174	64.83
12/4/2020 12:54	181.2	184.1	181.5	2174	64.91
12/4/2020 12:54	181.2	184.1	181.1	2174	65.04
12/4/2020 12:54	181.2	184	181.3	2171	64.96
12/4/2020 12:54	181.1	184	181.2	2174	65
12/4/2020 12:54	181.1	184.2	180.9	2174	65.08
12/4/2020 12:54	181.1	184.1	180.9	2176	65
12/4/2020 12:54	180.9	184.3	180.9	2177	64.96
12/4/2020 12:54	180.9	184.1	180.8	2178	65.04
12/4/2020 12:54	180.9	184.1	181	2178	64.88
12/4/2020 12:54	180.9	184.2	180.8	2183	64.92
12/4/2020 12:54	180.8	183.9	180.8	2185	64.92
12/4/2020 12:54	180.8	184.1	180.8	2185	65.03
12/4/2020 12:54	180.8	184	180.9	2178	64.83
12/4/2020 12:54	180.7	183.9	180.8	2180	64.92
12/4/2020 12:54	180.7	184.2	180.8	2178	65.04
12/4/2020 12:54	180.7	184.1	180.8	2178	64.99
12/4/2020 12:54	180.7	184.2	180.9	2178	65.2
12/4/2020 12:54	180.6	184	180.6	2178	64.99
12/4/2020 12:54	180.7	183.8	180.6	2171	64.92
12/4/2020 12:54	180.6	184.2	180.9	2174	64.92
12/4/2020 12:54	180.6	184.1	180.8	2169	65.03
12/4/2020 12:54	180.7	184.2	180.7	2167	65.03
12/4/2020 12:54	180.7	183.9	180.5	2167	64.96
12/4/2020 12:54	180.7	183.9	180.5	2167	64.79
12/4/2020 12:54	180.7	184.2	180.6	2167	65.03

12/4/2020 12:54	180.7	184.1	180.7	2169	64.88
12/4/2020 12:54	180.6	184.1	180.9	2171	64.95
12/4/2020 12:54	180.7	184.2	181.1	2176	64.83
12/4/2020 12:54	180.7	183.9	181.2	2174	65.12
12/4/2020 12:54	180.7	184.1	181.3	2176	64.83
12/4/2020 12:54	180.7	183.9	181.4	2178	65.03
12/4/2020 12:54	180.7	184.2	181.3	2182	64.92
12/4/2020 12:54	180.7	184	181.5	2179	64.83
12/4/2020 12:54	180.8	184.1	181.5	2177	64.92
12/4/2020 12:54	180.8	184.1	181.5	2178	64.92
12/4/2020 12:54	180.8	184	181.4	2176	64.99
12/4/2020 12:54	180.9	184.2	181.1	2178	64.99
12/4/2020 12:54	180.8	184.2	180.9	2181	65.04
12/4/2020 12:54	180.9	184.2	180.6	2178	64.95
12/4/2020 12:54	180.9	183	180.5	2181	64.91
12/4/2020 12:54	180.9	183.2	180.6	2180	65
12/4/2020 12:54	180.9	183	180.8	2180	64.88
12/4/2020 12:54	180.9	183	180.6	2177	64.92
12/4/2020 12:54	181	182.8	180.8	2181	65
12/4/2020 12:54	181	183	180.8	2180	64.99
12/4/2020 12:54	181	183.1	180.8	2180	64.87
12/4/2020 12:54	181.1	183.1	180.4	2181	65.04
12/4/2020 12:54	181.1	183.2	180.8	2181	64.91
12/4/2020 12:54	181.1	183	181.4	2185	64.92
12/4/2020 12:54	181.1	183	181.4	2181	65.04
12/4/2020 12:54	181.1	183.2	180.6	2178	64.92
12/4/2020 12:54	181.2	183.1	180.3	2180	65
12/4/2020 12:54	181.3	183	180.8	2182	64.96
12/4/2020 12:54	181.3	183.3	180.8	2178	64.96
12/4/2020 12:54	181.3	183	180.8	2174	64.96
12/4/2020 12:55	181.3	183.1	180.4	2176	65.04
12/4/2020 12:55	181.4	183.2	180.7	2178	64.96
12/4/2020 12:55	181.5	183	181.6	2174	64.96
12/4/2020 12:55	181.4	183.1	181.1	2178	65.05
12/4/2020 12:55	181.5	183.4	180.5	2178	64.92
12/4/2020 12:55	181.6	183.1	180.4	2181	64.88
12/4/2020 12:55	181.6	183.2	180.8	2182	64.92
12/4/2020 12:55	181.6	183	180.9	2183	64.96
12/4/2020 12:55	181.6	183.1	180.7	2181	64.99
12/4/2020 12:55	181.6	183	180.6	2182	65.01
12/4/2020 12:55	181.6	183.2	180.5	2181	64.88
12/4/2020 12:55	181.7	183.2	180.9	2180	64.99
12/4/2020 12:55	181.8	183	181.4	2180	64.97
12/4/2020 12:55	181.8	183	181.4	2178	64.84
12/4/2020 12:55	181.8	183	181.1	2180	65.04
12/4/2020 12:55	181.9	183	180.6	2184	65.08
12/4/2020 12:55	181.9	183.2	180.5	2180	64.99

12/4/2020 12:55	181.9	183	180.2	2177	64.84
12/4/2020 12:55	182	183.1	180.2	2177	65.04
12/4/2020 12:55	182	183	180.4	2179	64.84
12/4/2020 12:55	182.1	183.2	180.5	2182	65.04
12/4/2020 12:55	182.1	183.2	180.5	2182	65.17
12/4/2020 12:55	182.2	182.8	180.3	2181	65.08
12/4/2020 12:55	182.2	183.1	180.4	2180	64.92
12/4/2020 12:55	182.2	183	180.5	2178	64.88
12/4/2020 12:55	182.4	183	180.9	2178	64.99
12/4/2020 12:55	182.3	183.1	181.5	2176	65.08
12/4/2020 12:55	182.4	183.2	181.4	2174	64.8
12/4/2020 12:55	182.4	183.3	180.9	2176	64.99
12/4/2020 12:55	182.5	183.2	180.6	2179	65
12/4/2020 12:55	182.6	183.2	180.8	2180	65.04
12/4/2020 12:55	182.6	183.3	180.8	2179	64.92
12/4/2020 12:55	182.7	183	180.4	2179	64.92
12/4/2020 12:55	182.7	183.1	180.6	2181	64.92
12/4/2020 12:55	182.7	183	181.4	2185	65.08
12/4/2020 12:55	182.8	183	180.9	2178	65.04
12/4/2020 12:55	182.8	183.2	180.6	2177	65.04
12/4/2020 12:55	182.9	183	180.7	2178	65
12/4/2020 12:55	182.9	183	180.6	2174	65
12/4/2020 12:55	182.9	183	180.7	2178	64.96
12/4/2020 12:55	182.9	183	180.4	2180	65
12/4/2020 12:55	183	183.1	180.6	2176	65.04
12/4/2020 12:55	183	183	181.3	2176	65.04
12/4/2020 12:55	183	183	181.4	2173	64.96
12/4/2020 12:55	183.1	183	180.8	2172	64.99
12/4/2020 12:55	183	183	180.6	2178	65.04
12/4/2020 12:55	183.1	183.3	180.5	2178	65
12/4/2020 12:55	183	183	180.8	2180	64.96
12/4/2020 12:55	183	183	180.9	2183	64.96
12/4/2020 12:55	183.1	183	180.6	2181	65.08
12/4/2020 12:55	183.1	183	180.4	2184	65.12
12/4/2020 12:55	183	183.1	180.5	2182	65.08
12/4/2020 12:55	183	183	180.7	2180	64.92
12/4/2020 12:55	183	183.1	181.5	2174	65.04
12/4/2020 12:55	183.1	183.1	181.5	2178	64.88
12/4/2020 12:55	183	183	181	2177	65.04
12/4/2020 12:55	183	183.3	180.6	2178	65.08
12/4/2020 12:55	183	183	180.4	2176	64.88
12/4/2020 12:55	183	183	180.8	2175	65.03
12/4/2020 12:56	183	183	180.6	2174	64.96
12/4/2020 12:56	183	183	180.7	2176	65.08
12/4/2020 12:56	183.1	183.2	180.4	2174	65
12/4/2020 12:56	183.1	183	180.4	2171	65
12/4/2020 12:56	183	183	180.5	2171	65

12/4/2020 12:56	183.1	183.1	180.6	2168	64.96
12/4/2020 12:56	183.1	183	181.1	2167	65.04
12/4/2020 12:56	183.1	183.2	181.5	2169	64.96
12/4/2020 12:56	183.1	183.1	181.6	2168	64.96
12/4/2020 12:56	183.1	183	181.3	2165	65.04
12/4/2020 12:56	183.1	183	181.3	2167	65
12/4/2020 12:56	183.2	183.2	180.8	2167	64.92
12/4/2020 12:56	183.3	183.2	180.8	2168	64.96
12/4/2020 12:56	183.3	183.2	180.7	2163	64.92
12/4/2020 12:56	183.3	183	180.5	2162	64.92
12/4/2020 12:56	183.4	183	180.6	2157	65.04
12/4/2020 12:56	183.4	183.2	180.6	2160	64.96
12/4/2020 12:56	183.6	183.2	180.7	2161	65.04
12/4/2020 12:56	183.7	183.2	181.3	2165	64.96
12/4/2020 12:56	183.7	183.1	181.4	2169	64.84
12/4/2020 12:56	183.7	183	181.6	2167	65.04
12/4/2020 12:56	183.7	183	181.3	2171	65.08
12/4/2020 12:56	183.9	183	180.6	2167	64.88
12/4/2020 12:56	184	183.2	180.6	2169	65.08
12/4/2020 12:56	184	183	180.6	2164	64.96
12/4/2020 12:56	184.1	183.3	180.8	2163	64.99
12/4/2020 12:56	184.2	183	181	2163	65.04
12/4/2020 12:56	184.4	183	180.8	2160	64.96
12/4/2020 12:56	184.3	183.2	180.6	2167	64.88
12/4/2020 12:56	184.5	183.2	180.9	2171	64.88
12/4/2020 12:56	184.5	183.2	181.5	2172	65.04
12/4/2020 12:56	184.6	183	180.8	2174	64.96
12/4/2020 12:56	184.8	183	180.6	2174	64.92
12/4/2020 12:56	184.8	183	181	2173	64.96
12/4/2020 12:56	184.9	183	181	2167	65
12/4/2020 12:56	185	183.2	180.6	2174	64.96
12/4/2020 12:56	185	183.2	181	2174	65
12/4/2020 12:56	185.2	183.2	181.7	2175	64.96
12/4/2020 12:56	185.3	183	180.6	2174	64.96
12/4/2020 12:56	185.4	183	180.6	2180	65.04
12/4/2020 12:56	185.5	183.2	181	2178	65.04
12/4/2020 12:56	185.5	183.2	180.9	2178	64.96
12/4/2020 12:56	185.5	183.2	180.9	2175	65
12/4/2020 12:56	185.7	183.2	180.8	2171	65
12/4/2020 12:56	185.7	183	181.3	2171	65.04
12/4/2020 12:56	185.9	183	181.8	2174	65.08
12/4/2020 12:56	185.9	182.9	181.3	2175	64.92
12/4/2020 12:56	185.9	183	180.6	2177	65.04
12/4/2020 12:56	186	183	180.7	2178	64.96
12/4/2020 12:56	186	183	180.8	2178	64.92
12/4/2020 12:56	186.1	183.2	181	2177	65.08
12/4/2020 12:56	186.1	183.9	180.9	2181	65.12

12/4/2020 12:56	186.2	184.2	180.7	2177	65.04
12/4/2020 12:56	186.2	183.9	181.1	2174	65.08
12/4/2020 12:56	186.3	184.1	181.1	2178	64.88
12/4/2020 12:56	186.3	184.1	181	2177	64.92
12/4/2020 12:56	186.4	184.2	181.1	2181	64.92
12/4/2020 12:56	186.4	184.1	180.9	2180	64.88
12/4/2020 12:56	186.4	183.9	181	2185	64.92
12/4/2020 12:56	186.3	184.2	181	2185	64.92
12/4/2020 12:57	186.4	184	181.1	2181	65
12/4/2020 12:57	186.4	184.1	180.9	2180	65
12/4/2020 12:57	186.3	184.1	180.6	2180	64.92
12/4/2020 12:57	186.4	183.9	180.8	2186	64.96
12/4/2020 12:57	186.4	183.9	181.5	2188	65.08
12/4/2020 12:57	186.4	184	181.8	2190	64.92
12/4/2020 12:57	186.4	184	181.1	2189	64.96
12/4/2020 12:57	186.4	184.1	181.1	2184	64.96
12/4/2020 12:57	186.4	184.2	181.2	2185	65
12/4/2020 12:57	186.3	184.2	181.2	2186	65.04
12/4/2020 12:57	186.3	183.9	180.7	2188	65
12/4/2020 12:57	186.3	184.2	180.8	2189	65.04
12/4/2020 12:57	186.3	184.2	181.8	2193	64.96
12/4/2020 12:57	186.3	184	181.9	2193	64.96
12/4/2020 12:57	186.2	184.2	181.2	2195	64.92
12/4/2020 12:57	186.1	183.9	181	2198	65
12/4/2020 12:57	186.1	184.2	181.3	2192	65
12/4/2020 12:57	186	184.2	181.2	2192	64.92
12/4/2020 12:57	185.9	183.9	180.9	2190	65.04
12/4/2020 12:57	185.9	184.1	180.8	2185	65.08
12/4/2020 12:57	185.9	184	181.5	2185	64.96
12/4/2020 12:57	185.8	184.1	181.8	2181	65.03
12/4/2020 12:57	185.8	184.2	181.1	2180	64.96
12/4/2020 12:57	185.7	184.1	181.1	2177	64.92
12/4/2020 12:57	185.7	184.1	181.1	2178	64.96
12/4/2020 12:57	185.5	184.2	181.1	2179	65.04
12/4/2020 12:57	185.5	183.9	180.8	2180	64.92
12/4/2020 12:57	185.5	184.3	180.6	2182	65.08
12/4/2020 12:57	185.3	183.8	181.5	2185	65.04
12/4/2020 12:57	185.3	184.2	181.6	2185	64.96
12/4/2020 12:57	185.3	184.2	181	2189	65.09
12/4/2020 12:57	185.2	184	180.9	2191	64.92
12/4/2020 12:57	185.1	184.1	181	2196	64.96
12/4/2020 12:57	185.1	184.1	181.1	2196	65.04
12/4/2020 12:57	185.1	183.9	180.9	2191	64.96
12/4/2020 12:57	185	184.2	180.6	2187	64.96
12/4/2020 12:57	185	184.1	181.5	2185	65.04
12/4/2020 12:57	185	184.2	181.9	2178	64.96
12/4/2020 12:57	185	184.1	180.8	2178	64.99

12/4/2020 12:57	185	183.9	180.9	2183	65.09
12/4/2020 12:57	184.9	184.2	181.1	2184	64.88
12/4/2020 12:57	184.8	184	181.1	2184	65.03
12/4/2020 12:57	184.9	184.2	181.1	2188	65.05
12/4/2020 12:57	184.8	184.1	180.7	2187	64.99
12/4/2020 12:57	184.8	184.1	180.7	2190	64.84
12/4/2020 12:57	184.8	184.2	180.9	2194	65.04
12/4/2020 12:57	184.8	183.8	181.5	2196	64.88
12/4/2020 12:57	184.8	184.2	181.8	2195	64.92
12/4/2020 12:57	184.8	184.2	181.4	2194	65.16
12/4/2020 12:57	184.8	183.9	180.8	2190	64.96
12/4/2020 12:57	184.8	183.9	180.8	2194	64.99
12/4/2020 12:57	184.8	183.9	180.9	2189	65.05
12/4/2020 12:57	184.8	184.2	181.2	2189	64.88
12/4/2020 12:57	184.8	184.1	181	2187	65.03
12/4/2020 12:57	184.8	184.3	181.1	2189	65.09
12/4/2020 12:57	184.8	184.2	180.9	2187	64.92
12/4/2020 12:57	184.8	184.1	180.6	2188	64.96
12/4/2020 12:57	184.8	184.1	180.6	2196	65
12/4/2020 12:57	184.8	184.2	181.5	2195	64.96
12/4/2020 12:58	184.8	183.9	181.7	2190	65
12/4/2020 12:58	184.8	184.3	181.6	2193	65.04
12/4/2020 12:58	184.8	184.1	181.1	2188	64.92
12/4/2020 12:58	184.7	184	180.7	2186	64.96
12/4/2020 12:58	184.8	184.1	181	2187	65
12/4/2020 12:58	184.7	183.9	181	2185	64.96
12/4/2020 12:58	184.8	184.2	180.9	2182	65
12/4/2020 12:58	184.7	183.9	181	2186	64.96
12/4/2020 12:58	184.6	184.1	181.1	2185	65
12/4/2020 12:58	184.6	184.1	181.1	2185	64.92
12/4/2020 12:58	184.6	183.9	181.1	2190	65.12
12/4/2020 12:58	184.6	184.2	181.1	2185	65.03
12/4/2020 12:58	184.6	183.9	181	2185	65.03
12/4/2020 12:58	184.6	184.2	181.1	2181	65.12
12/4/2020 12:58	184.6	184.2	181	2181	64.88
12/4/2020 12:58	184.5	184.2	181.1	2185	64.84
12/4/2020 12:58	184.5	184.2	181.1	2184	65.08
12/4/2020 12:58	184.5	184	181.1	2178	64.88
12/4/2020 12:58	184.4	183.9	181.1	2176	65.03
12/4/2020 12:58	184.4	184.1	181.1	2176	64.88
12/4/2020 12:58	184.2	184	181.1	2176	64.96
12/4/2020 12:58	184.2	184.1	180.8	2180	65
12/4/2020 12:58	184.2	184.1	180.7	2182	65.08
12/4/2020 12:58	184.2	184	180.8	2178	64.96
12/4/2020 12:58	184	184	180.6	2182	65.04
12/4/2020 12:58	184	184.1	180.8	2187	65.04
12/4/2020 12:58	184	184.2	180.8	2189	65.09

12/4/2020 12:58	183.9	184.2	180.8	2187	65.05
12/4/2020 12:58	183.9	184.1	181.1	2189	64.96
12/4/2020 12:58	183.8	184.2	181.5	2185	64.96
12/4/2020 12:58	183.7	184.1	181.6	2189	65.04
12/4/2020 12:58	183.7	184.1	181.6	2188	64.92
12/4/2020 12:58	183.6	184.1	181.1	2184	64.92
12/4/2020 12:58	183.6	184.2	180.6	2185	64.92
12/4/2020 12:58	183.6	184.2	180.5	2185	65.08
12/4/2020 12:58	183.5	183.9	180.7	2186	65.08
12/4/2020 12:58	183.4	184.2	180.9	2185	65.12
12/4/2020 12:58	183.5	184.2	181.1	2181	65.04
12/4/2020 12:58	183.4	184.1	180.9	2185	65.08
12/4/2020 12:58	183.4	184.1	180.7	2178	64.97
12/4/2020 12:58	183.4	184.1	180.5	2181	65.04
12/4/2020 12:58	183.3	184.2	180.8	2180	65
12/4/2020 12:58	183.3	184.1	181.5	2183	64.92
12/4/2020 12:58	183.3	183.8	181.4	2183	64.88
12/4/2020 12:58	183.4	184.2	180.6	2182	65.04
12/4/2020 12:58	183.3	184.1	180.4	2183	65.09
12/4/2020 12:58	183.3	184.2	180.5	2180	64.96
12/4/2020 12:58	183.3	184.2	180.6	2178	65
12/4/2020 12:58	183.3	184	180.8	2175	64.96
12/4/2020 12:58	183.3	184.2	180.7	2176	65
12/4/2020 12:58	183.4	184	180.8	2174	65.04
12/4/2020 12:58	183.3	184.2	180.7	2176	64.96
12/4/2020 12:58	183.4	184.1	180.8	2176	65.12
12/4/2020 12:58	183.4	183.8	180.9	2178	65
12/4/2020 12:58	183.4	183.9	180.8	2178	65.08
12/4/2020 12:58	183.4	183.9	180.7	2174	64.93
12/4/2020 12:58	183.6	184.1	180.6	2178	64.92
12/4/2020 12:58	183.5	184.1	180.8	2180	64.88
12/4/2020 12:58	183.6	184.2	180.8	2181	64.99
12/4/2020 12:58	183.6	184.1	180.6	2174	64.92
12/4/2020 12:59	183.6	183.9	181	2174	64.92
12/4/2020 12:59	183.7	184.2	180.7	2177	65.03
12/4/2020 12:59	183.8	184.2	180.7	2177	65.08
12/4/2020 12:59	183.8	183.9	180.6	2177	64.92
12/4/2020 12:59	183.9	184.2	180.6	2176	65.08
12/4/2020 12:59	183.9	184.1	180.4	2169	64.99
12/4/2020 12:59	184	184.2	180.6	2172	64.96
12/4/2020 12:59	184	184.1	181.1	2170	64.92
12/4/2020 12:59	184.2	184.1	181.4	2177	64.96
12/4/2020 12:59	184.2	184.1	181	2178	64.92
12/4/2020 12:59	184.3	184.1	180.5	2176	64.96
12/4/2020 12:59	184.4	184.1	180.4	2171	64.92
12/4/2020 12:59	184.4	183.9	180.7	2169	65.04
12/4/2020 12:59	184.6	184.2	181	2171	65.04

12/4/2020 12:59	184.6	184.2	180.6	2169	65.04
12/4/2020 12:59	184.7	184.1	180.5	2178	65.04
12/4/2020 12:59	184.7	184.1	180.8	2177	64.96
12/4/2020 12:59	184.8	184.2	181.8	2176	64.96
12/4/2020 12:59	185	184.1	181.3	2181	65
12/4/2020 12:59	185	184.2	180.7	2183	65
12/4/2020 12:59	185.2	184.1	180.8	2185	65.04
12/4/2020 12:59	185.3	184.1	181.2	2178	64.99
12/4/2020 12:59	185.3	184.1	181	2181	64.96
12/4/2020 12:59	185.4	184.2	180.6	2178	64.83
12/4/2020 12:59	185.5	184.1	180.7	2185	64.96
12/4/2020 12:59	185.5	184	181	2185	64.92
12/4/2020 12:59	185.7	183.9	181.4	2185	64.92
12/4/2020 12:59	185.7	184.1	181.8	2187	64.96
12/4/2020 12:59	185.9	184.2	181.1	2187	65.08
12/4/2020 12:59	185.9	184.1	180.6	2185	64.99
12/4/2020 12:59	186	184.1	180.6	2181	65.04
12/4/2020 12:59	186.1	184.1	180.8	2181	64.97
12/4/2020 12:59	186.2	184.2	180.9	2184	64.92
12/4/2020 12:59	186.4	184.1	181.1	2190	65.01
12/4/2020 12:59	186.5	184.2	181.1	2188	64.92
12/4/2020 12:59	186.6	183.9	181.1	2185	64.92
12/4/2020 12:59	186.6	183.9	180.9	2185	65.04
12/4/2020 12:59	186.8	184.1	180.8	2185	64.92
12/4/2020 12:59	186.8	183.9	180.9	2184	65.04
12/4/2020 12:59	186.9	184.1	180.8	2185	65.05
12/4/2020 12:59	187	184.1	180.6	2178	64.96
12/4/2020 12:59	187	183.9	180.6	2178	65
12/4/2020 12:59	187.1	184.2	180.7	2180	65.12
12/4/2020 12:59	187.1	183.9	180.8	2178	64.96
12/4/2020 12:59	187.2	184.2	180.8	2177	64.96
12/4/2020 12:59	187.2	184.1	180.7	2182	64.96
12/4/2020 12:59	187.3	184	180.8	2181	64.88
12/4/2020 12:59	187.4	184.2	180.9	2184	64.88
12/4/2020 12:59	187.4	184	180.8	2183	64.97
12/4/2020 12:59	187.5	184.1	180.8	2187	64.88
12/4/2020 12:59	187.5	183.9	180.9	2191	65.04
12/4/2020 12:59	187.6	183.9	180.9	2189	65.05
12/4/2020 12:59	187.7	184.1	180.9	2185	64.99
12/4/2020 12:59	187.6	184	180.9	2185	65.03
12/4/2020 12:59	187.7	184.2	181.1	2189	65.12
12/4/2020 12:59	187.7	184.1	181.1	2183	64.99
12/4/2020 12:59	187.7	183.9	181.2	2180	64.92
12/4/2020 12:59	187.8	184.2	181.2	2185	65.08
12/4/2020 12:59	187.8	184.1	180.9	2181	64.99
12/4/2020 13:00	187.8	184.1	181.4	2178	64.88
12/4/2020 13:00	187.8	184.1	181.8	2187	65.04

12/4/2020 13:00	187.8	184	180.9	2191	64.96
12/4/2020 13:00	187.9	184.2	180.8	2192	65
12/4/2020 13:00	187.9	183.9	181.1	2189	64.96
12/4/2020 13:00	188	184.1	181.3	2190	65
12/4/2020 13:00	187.9	184.1	181.3	2191	65
12/4/2020 13:00	187.9	184.1	182.1	2190	65
12/4/2020 13:00	188	184.2	181.4	2188	64.83
12/4/2020 13:00	188.1	183.8	181.1	2192	65
12/4/2020 13:00	188.1	184.2	181.2	2194	65.01
12/4/2020 13:00	188	184.1	181.4	2194	64.96
12/4/2020 13:00	188.1	183.9	181.3	2194	65.12
12/4/2020 13:00	188.1	184.2	180.9	2196	65.04
12/4/2020 13:00	188.1	184.1	182.2	2192	64.92
12/4/2020 13:00	188.1	184.1	181.7	2194	64.88
12/4/2020 13:00	188.1	185.4	181.1	2192	64.88
12/4/2020 13:00	188.1	184.7	181.5	2192	64.88
12/4/2020 13:00	188.1	185.2	181.3	2194	65.01
12/4/2020 13:00	188.2	185	180.9	2194	64.96
12/4/2020 13:00	188.1	184.7	182.1	2195	65.12
12/4/2020 13:00	188.1	185.1	181.8	2195	65.04
12/4/2020 13:00	188.1	184.7	181.1	2189	64.92
12/4/2020 13:00	188.1	185.1	181.3	2191	65.04
12/4/2020 13:00	188.1	185.1	181.3	2191	65.08
12/4/2020 13:00	188.1	184.8	181.1	2192	64.96
12/4/2020 13:00	188.1	184.8	181.4	2196	64.96
12/4/2020 13:00	188.1	184.9	182.2	2196	65.08
12/4/2020 13:00	188	185.1	181.3	2197	64.96
12/4/2020 13:00	188.1	184.9	181.1	2197	65.09
12/4/2020 13:00	188.1	185.3	181.5	2195	65.04
12/4/2020 13:00	188.1	184.9	181.5	2194	64.96
12/4/2020 13:00	188.1	184.7	181.3	2196	65.04
12/4/2020 13:00	188.1	185.1	181.5	2199	64.96
12/4/2020 13:00	188	185.3	182.1	2197	64.88
12/4/2020 13:00	188	184.6	181.8	2194	64.92
12/4/2020 13:00	188	184.8	180.9	2194	65.01
12/4/2020 13:00	188	184.6	181.1	2194	64.92
12/4/2020 13:00	187.9	185.2	181.6	2191	65.04
12/4/2020 13:00	187.9	185.3	181.6	2195	65.12
12/4/2020 13:00	187.9	185.2	181.4	2193	65.04
12/4/2020 13:00	188	184.8	181.3	2196	65.04
12/4/2020 13:00	187.9	184.7	182	2196	64.96
12/4/2020 13:00	187.8	184.9	182.2	2194	65.04
12/4/2020 13:00	187.9	184.6	182	2194	65.12
12/4/2020 13:00	187.8	185.1	181.1	2197	64.93
12/4/2020 13:00	187.8	185.2	181.3	2198	65.04
12/4/2020 13:00	187.9	184.9	181.5	2194	64.96
12/4/2020 13:00	187.7	184.9	181.5	2195	65.09

12/4/2020 13:00	187.7	184.6	181.6	2197	64.92
12/4/2020 13:00	187.7	185.1	181.4	2192	64.96
12/4/2020 13:00	187.7	185.3	181.4	2191	65.04
12/4/2020 13:00	187.7	184.9	181.5	2196	64.96
12/4/2020 13:00	187.7	185.3	181.4	2195	64.96
12/4/2020 13:00	187.6	185.4	181.4	2198	65.05
12/4/2020 13:00	187.6	185	181.4	2194	64.92
12/4/2020 13:00	187.5	185.3	181.5	2194	64.96
12/4/2020 13:00	187.5	185.1	181.6	2194	65.09
12/4/2020 13:00	187.5	184.7	181.6	2192	64.96
12/4/2020 13:01	187.5	185.3	181.4	2194	65
12/4/2020 13:01	187.4	184.6	181.7	2196	65.04
12/4/2020 13:01	187.3	184.8	181.4	2192	64.8
12/4/2020 13:01	187.3	184.6	181.6	2191	64.96
12/4/2020 13:01	187.3	185	181.4	2187	65.12
12/4/2020 13:01	187.2	185.3	181.1	2185	65.04
12/4/2020 13:01	187.2	184.7	181	2184	65.04
12/4/2020 13:01	187.2	185	181.3	2189	64.97
12/4/2020 13:01	187.2	184.9	181.8	2188	64.95
12/4/2020 13:01	187.2	184.9	182.3	2187	64.96
12/4/2020 13:01	187.1	185.3	182.2	2190	65.16
12/4/2020 13:01	187.1	184.8	181.4	2191	64.83
12/4/2020 13:01	187	184.9	181.3	2195	64.92
12/4/2020 13:01	187	184.7	181.3	2196	65.16
12/4/2020 13:01	187	184.9	181.5	2196	64.99
12/4/2020 13:01	186.9	185.4	181.4	2192	64.99
12/4/2020 13:01	186.9	185.1	181.3	2195	65.08
12/4/2020 13:01	186.8	185.2	181.3	2195	64.92
12/4/2020 13:01	186.8	184.9	182	2193	65.04
12/4/2020 13:01	186.8	184.7	182.1	2191	65
12/4/2020 13:01	186.7	185.1	181.7	2194	64.92
12/4/2020 13:01	186.7	184.8	181.2	2197	65
12/4/2020 13:01	186.5	185.2	181.5	2195	65.04
12/4/2020 13:01	186.6	184.8	182	2188	64.99
12/4/2020 13:01	186.4	184.9	181.5	2192	64.96
12/4/2020 13:01	186.4	184.7	181.4	2190	65
12/4/2020 13:01	186.5	184.8	181	2188	64.92
12/4/2020 13:01	186.4	185.1	181.7	2188	64.96
12/4/2020 13:01	186.3	185.2	182.2	2194	65.09
12/4/2020 13:01	186.2	184.6	182.1	2194	64.92
12/4/2020 13:01	186.2	184.8	181.5	2193	64.96
12/4/2020 13:01	186.1	184.7	181.3	2191	65.08
12/4/2020 13:01	186	185	181.3	2190	64.92
12/4/2020 13:01	186.1	185.2	181.4	2192	64.95
12/4/2020 13:01	186	185.3	181.4	2195	65.08
12/4/2020 13:01	186	185.2	181.6	2196	64.88
12/4/2020 13:01	185.9	184.8	181.6	2194	65.08

12/4/2020 13:01	185.9	185.1	181.5	2195	64.92
12/4/2020 13:01	185.8	185.1	181.4	2194	64.99
12/4/2020 13:01	185.8	184.7	181.6	2192	64.92
12/4/2020 13:01	185.7	185.3	181.5	2189	64.92
12/4/2020 13:01	185.7	184.8	181.5	2189	64.8
12/4/2020 13:01	185.7	185.1	181.7	2190	64.88
12/4/2020 13:01	185.6	185.2	181.5	2190	65.08
12/4/2020 13:01	185.5	185.4	181.5	2193	65.04
12/4/2020 13:01	185.5	185.1	181.4	2189	65.08
12/4/2020 13:01	185.4	184.8	181.4	2195	64.99
12/4/2020 13:01	185.3	185	181.5	2193	64.92
12/4/2020 13:01	185.4	184.6	181.3	2189	65.08
12/4/2020 13:01	185.3	185.3	181.5	2186	64.92
12/4/2020 13:01	185.2	185.3	181.4	2187	64.88
12/4/2020 13:01	185.2	184.7	181.4	2196	64.96
12/4/2020 13:01	185.1	184.9	181.5	2189	64.96
12/4/2020 13:01	185.1	184.8	181.4	2192	65.04
12/4/2020 13:01	185	185.3	181.3	2192	64.96
12/4/2020 13:01	185	185.1	181.4	2189	64.92
12/4/2020 13:01	185	184.7	181.3	2184	65
12/4/2020 13:01	184.9	184.9	181.3	2181	65
12/4/2020 13:01	184.9	184.7	181.2	2184	64.96
12/4/2020 13:01	184.8	184.7	181.3	2181	64.96
12/4/2020 13:02	184.8	185.1	181.2	2185	65
12/4/2020 13:02	184.7	184.7	181.1	2185	65
12/4/2020 13:02	184.7	184.9	181	2185	64.92
12/4/2020 13:02	184.6	185.2	181.3	2185	65.04
12/4/2020 13:02	184.6	184.8	181.6	2185	64.99
12/4/2020 13:02	184.5	185	181.8	2186	64.96
12/4/2020 13:02	184.4	184.7	182.1	2185	65.12
12/4/2020 13:02	184.4	185	182.1	2189	64.88
12/4/2020 13:02	184.3	185.1	181.6	2189	64.92
12/4/2020 13:02	184.3	184.6	181.2	2189	64.96
12/4/2020 13:02	184.3	184.9	180.9	2190	65.12
12/4/2020 13:02	184.2	184.9	181.1	2186	64.99
12/4/2020 13:02	184.2	184.8	181.1	2192	65.08
12/4/2020 13:02	184.1	184.6	181.4	2193	64.99
12/4/2020 13:02	184.1	185	181.3	2193	65.09
12/4/2020 13:02	184	185.3	181	2194	64.96
12/4/2020 13:02	183.9	184.8	180.8	2190	64.83
12/4/2020 13:02	184	184.8	181.7	2195	65.04
12/4/2020 13:02	183.9	184.8	181.9	2198	65.09
12/4/2020 13:02	183.9	185	181.3	2199	64.96
12/4/2020 13:02	183.8	185.1	180.8	2198	65
12/4/2020 13:02	183.7	185.2	180.9	2196	65.05
12/4/2020 13:02	183.7	185.4	181.4	2197	65
12/4/2020 13:02	183.7	184.8	181.1	2193	65.04

12/4/2020 13:02	183.6	184.9	181.1	2189	65
12/4/2020 13:02	183.6	185.3	181.5	2188	64.87
12/4/2020 13:02	183.5	185.1	182.1	2187	65
12/4/2020 13:02	183.5	185.4	181.3	2189	65.05
12/4/2020 13:02	183.4	185.1	181	2192	64.99
12/4/2020 13:02	183.4	184.8	181.1	2192	64.91
12/4/2020 13:02	183.3	185.4	181.3	2192	65.12
12/4/2020 13:02	183.3	185	181.3	2193	64.95
12/4/2020 13:02	183.3	185.3	181	2194	64.95
12/4/2020 13:02	183.3	184.9	181.4	2193	65.04
12/4/2020 13:02	183.2	184.7	181.6	2191	64.99
12/4/2020 13:02	183.1	185.4	181.1	2192	65
12/4/2020 13:02	183.1	185.3	180.9	2194	64.88
12/4/2020 13:02	183.1	185.5	180.8	2192	64.96
12/4/2020 13:02	183	185	181.2	2195	64.84
12/4/2020 13:02	183	184.6	181.3	2196	65.16
12/4/2020 13:02	183	185.2	181.1	2192	64.8
12/4/2020 13:02	183	185.1	181	2184	64.84
12/4/2020 13:02	182.9	184.6	180.8	2179	64.96
12/4/2020 13:02	182.9	184.6	180.9	2184	65.04
12/4/2020 13:02	182.8	184.8	181.3	2185	64.96
12/4/2020 13:02	182.8	185.3	181.4	2182	65.04
12/4/2020 13:02	182.7	185	182	2181	64.8
12/4/2020 13:02	182.7	184.8	182	2185	64.99
12/4/2020 13:02	182.6	184.8	181.8	2188	65.01
12/4/2020 13:02	182.5	184	181.8	2185	64.84
12/4/2020 13:02	182.6	184.3	181.8	2181	64.84
12/4/2020 13:02	182.5	184.2	181.7	2183	64.96
12/4/2020 13:02	182.4	184.1	181.8	2181	64.88
12/4/2020 13:02	182.4	184.2	181.3	2180	65.04
12/4/2020 13:02	182.3	183.9	181	2181	65.09
12/4/2020 13:02	182.3	184.2	180.7	2183	64.92
12/4/2020 13:02	182.2	184.1	180.8	2187	65
12/4/2020 13:02	182.3	183.9	181.1	2185	65
12/4/2020 13:02	182.2	184.3	180.9	2191	64.96
12/4/2020 13:03	182.2	183.9	180.6	2189	65
12/4/2020 13:03	182	184.2	181.3	2189	64.96
12/4/2020 13:03	182.1	183.9	181.5	2184	64.96
12/4/2020 13:03	182.1	184	180.7	2186	65.09
12/4/2020 13:03	182	184.1	180.8	2185	64.83
12/4/2020 13:03	182	184.2	180.9	2184	64.99
12/4/2020 13:03	182	184.1	180.9	2183	65.04
12/4/2020 13:03	181.9	183.9	180.6	2184	65.04
12/4/2020 13:03	181.9	184.3	180.6	2182	64.92
12/4/2020 13:03	181.9	184.1	180.6	2185	65.09
12/4/2020 13:03	181.9	184.2	181.5	2177	64.88
12/4/2020 13:03	181.8	184	181.7	2180	64.96

12/4/2020 13:03	181.7	184.1	181.4	2181	65
12/4/2020 13:03	181.8	184.2	180.7	2178	64.99
12/4/2020 13:03	181.7	184.2	180.3	2174	65.04
12/4/2020 13:03	181.7	184.1	180.6	2176	64.96
12/4/2020 13:03	181.7	184.1	180.9	2174	64.8
12/4/2020 13:03	181.6	184	180.7	2178	64.88
12/4/2020 13:03	181.6	184.2	180.8	2184	65.08
12/4/2020 13:03	181.6	183.8	180.9	2180	64.95
12/4/2020 13:03	181.6	184.2	180.6	2178	64.92
12/4/2020 13:03	181.6	184.2	180.8	2182	65
12/4/2020 13:03	181.5	183.9	180.8	2185	64.99
12/4/2020 13:03	181.6	184	180.7	2188	64.96
12/4/2020 13:03	181.5	183.9	180.6	2189	65.03
12/4/2020 13:03	181.4	183.9	180.8	2186	64.95
12/4/2020 13:03	181.4	184.1	180.7	2184	64.88
12/4/2020 13:03	181.4	184	180.9	2185	64.92
12/4/2020 13:03	181.4	183.9	180.8	2187	64.99
12/4/2020 13:03	181.4	184	180.8	2185	64.99
12/4/2020 13:03	181.4	184	180.9	2185	65.12
12/4/2020 13:03	181.3	184.1	180.6	2180	64.99
12/4/2020 13:03	181.4	184.1	180.4	2183	64.99
12/4/2020 13:03	181.3	184.1	180.3	2182	64.92
12/4/2020 13:03	181.2	184	180.9	2182	64.99
12/4/2020 13:03	181.2	184.2	181.3	2180	64.92
12/4/2020 13:03	181.2	184.2	180.9	2182	65.04
12/4/2020 13:03	181.1	184.2	180.3	2180	64.99
12/4/2020 13:03	181.2	184.2	180.4	2178	64.99
12/4/2020 13:03	181.1	183.9	180.6	2178	65.01
12/4/2020 13:03	181.1	184	180.6	2176	64.99
12/4/2020 13:03	181.1	184.3	180.4	2178	65
12/4/2020 13:03	181.1	184.1	181.3	2180	65.08
12/4/2020 13:03	180.9	184.2	180.8	2178	64.87
12/4/2020 13:03	181	184.1	180.2	2175	64.92
12/4/2020 13:03	180.9	184.1	180.5	2174	65.04
12/4/2020 13:03	180.9	183.9	180.6	2173	64.92
12/4/2020 13:03	180.9	184.1	180.5	2176	65.04
12/4/2020 13:03	181	184.2	180.5	2176	64.87
12/4/2020 13:03	180.9	184.1	181.4	2180	64.96
12/4/2020 13:03	180.8	183.9	180.6	2176	65
12/4/2020 13:03	180.8	183.9	180.2	2174	65
12/4/2020 13:03	180.9	183.9	180.5	2174	64.96
12/4/2020 13:03	180.8	184.2	180.5	2176	64.96
12/4/2020 13:03	180.7	184.1	180.5	2174	64.96
12/4/2020 13:03	180.8	184.2	180.2	2174	65
12/4/2020 13:03	180.7	184.1	180.4	2173	65
12/4/2020 13:03	180.7	183.9	181.2	2176	64.92
12/4/2020 13:03	180.7	184.1	181.1	2181	64.96

12/4/2020 13:04	180.7	184.2	180.6	2178	64.96
12/4/2020 13:04	180.7	184.1	180.1	2178	65.04
12/4/2020 13:04	180.6	184.1	180.1	2173	64.95
12/4/2020 13:04	180.6	184	180.3	2178	64.88
12/4/2020 13:04	180.5	183	180.5	2174	65.04
12/4/2020 13:04	180.6	183.1	180.6	2176	64.99
12/4/2020 13:04	180.6	183.2	180.6	2176	64.96
12/4/2020 13:04	180.6	183	180.3	2175	64.92
12/4/2020 13:04	180.5	183.2	180.3	2178	64.92
12/4/2020 13:04	180.5	183	180.2	2182	65.12
12/4/2020 13:04	180.5	183	180.4	2180	64.92
12/4/2020 13:04	180.4	183.2	180.7	2178	64.88
12/4/2020 13:04	180.4	183	180.9	2183	64.92
12/4/2020 13:04	180.4	183	180.9	2180	65.04
12/4/2020 13:04	180.3	183	180.9	2180	64.83
12/4/2020 13:04	180.4	183.1	180.9	2177	64.92
12/4/2020 13:04	180.3	183.2	180.6	2180	64.99
12/4/2020 13:04	180.3	183	180.3	2178	65.08
12/4/2020 13:04	180.4	183	180.1	2174	64.96
12/4/2020 13:04	180.3	183	179.9	2174	64.88
12/4/2020 13:04	180.3	182.8	179.9	2172	64.88
12/4/2020 13:04	180.3	183.1	180.2	2174	65.08
12/4/2020 13:04	180.3	183	180.2	2176	64.92
12/4/2020 13:04	180.3	183	180.4	2174	65.08
12/4/2020 13:04	180.3	183	180.4	2172	64.88
12/4/2020 13:04	180.3	183.3	180.3	2174	64.92
12/4/2020 13:04	180.2	182.9	180.3	2174	65.03
12/4/2020 13:04	180.3	183	180.3	2176	64.92
12/4/2020 13:04	180.2	183	180.1	2180	65.04
12/4/2020 13:04	180.2	183.2	180.1	2182	64.92
12/4/2020 13:04	180.2	183	180	2180	64.88
12/4/2020 13:04	180.2	183.1	180.2	2182	64.92
12/4/2020 13:04	180.2	182.9	180.3	2180	64.88
12/4/2020 13:04	180.1	183	180.4	2181	64.92
12/4/2020 13:04	180.1	183	180.6	2181	64.92
12/4/2020 13:04	180.1	183.1	180.6	2178	64.99
12/4/2020 13:04	180.2	183	180.8	2180	65.08
12/4/2020 13:04	180.2	183	180.8	2178	64.88
12/4/2020 13:04	180.1	183.1	180.4	2180	65
12/4/2020 13:04	180.1	183.1	180.2	2179	65
12/4/2020 13:04	180.2	183	179.9	2182	64.92
12/4/2020 13:04	180.2	183.2	179.9	2180	64.92
12/4/2020 13:04	180.3	182.9	180.1	2181	65
12/4/2020 13:04	180.3	183	180.2	2178	64.92
12/4/2020 13:04	180.2	183.2	180.2	2180	64.96
12/4/2020 13:04	180.3	183	180.2	2178	64.96
12/4/2020 13:04	180.3	183	179.9	2181	65

12/4/2020 13:04	180.4	183	179.8	2181	65
12/4/2020 13:04	180.3	183	179.9	2181	65
12/4/2020 13:04	180.4	183	180.8	2181	64.87
12/4/2020 13:04	180.4	183	180.6	2181	64.96
12/4/2020 13:04	180.5	183.1	180	2180	64.96
12/4/2020 13:04	180.5	183	180.2	2184	64.92
12/4/2020 13:04	180.5	182.9	180.2	2188	64.92
12/4/2020 13:04	180.6	183.1	180.2	2190	64.92
12/4/2020 13:04	180.6	182.9	179.9	2189	64.92
12/4/2020 13:04	180.6	183	179.9	2185	64.99
12/4/2020 13:04	180.7	183.1	180.7	2183	65.08
12/4/2020 13:04	180.6	182.9	180.8	2186	65.08
12/4/2020 13:05	180.6	183.1	180.2	2188	65.04
12/4/2020 13:05	180.6	183	179.9	2188	64.92
12/4/2020 13:05	180.6	183.2	180.2	2186	65.08
12/4/2020 13:05	180.6	182.9	180.2	2188	64.92
12/4/2020 13:05	180.6	183.2	180.2	2190	65.04
12/4/2020 13:05	180.6	183	180.2	2190	64.92
12/4/2020 13:05	180.6	182.9	180	2184	64.99
12/4/2020 13:05	180.6	183	180	2188	65.04
12/4/2020 13:05	180.6	183.2	179.8	2189	64.88
12/4/2020 13:05	180.7	183.2	180	2184	64.99
12/4/2020 13:05	180.6	183.3	179.7	2185	64.83
12/4/2020 13:05	180.6	183	179.9	2189	64.92
12/4/2020 13:05	180.6	183	179.9	2192	64.99
12/4/2020 13:05	180.6	183.1	179.9	2189	64.99
12/4/2020 13:05	180.6	183	179.9	2189	65
12/4/2020 13:05	180.6	183.2	179.9	2187	64.99
12/4/2020 13:05	180.6	183	179.9	2185	64.96
12/4/2020 13:05	180.7	183.1	179.9	2181	65
12/4/2020 13:05	180.6	183.1	179.9	2185	64.87
12/4/2020 13:05	180.6	183	179.9	2189	65.04
12/4/2020 13:05	180.7	183.1	179.9	2189	65
12/4/2020 13:05	180.7	183	180.1	2184	64.92
12/4/2020 13:05	180.7	183.1	180.2	2181	65
12/4/2020 13:05	180.7	183	180.2	2184	65.04
12/4/2020 13:05	180.8	183.1	180.2	2181	64.87
12/4/2020 13:05	180.8	183.2	180.1	2183	64.92
12/4/2020 13:05	180.8	183	180	2187	65
12/4/2020 13:05	180.9	183.1	180.6	2187	64.92
12/4/2020 13:05	180.9	183.2	180.7	2188	64.96
12/4/2020 13:05	181	183.1	180.2	2185	65.05
12/4/2020 13:05	181	183.2	179.8	2181	64.91
12/4/2020 13:05	181.1	183	180.1	2181	64.92
12/4/2020 13:05	181.1	183	180.2	2184	65.12
12/4/2020 13:05	181.2	183	180.1	2188	64.95
12/4/2020 13:05	181.3	183	179.7	2189	64.88

12/4/2020 13:05	181.3	183	180.5	2189	65.04
12/4/2020 13:05	181.3	183.1	180.9	2187	64.88
12/4/2020 13:05	181.4	183	180.2	2186	65.04
12/4/2020 13:05	181.4	183.1	179.7	2185	65
12/4/2020 13:05	181.5	183	180.2	2184	64.99
12/4/2020 13:05	181.6	183	180.3	2181	64.99
12/4/2020 13:05	181.6	183.7	180.1	2185	65.01
12/4/2020 13:05	181.7	183	179.8	2183	64.99
12/4/2020 13:05	181.7	183	180.4	2180	64.95
12/4/2020 13:05	181.8	183	180.8	2182	65.08
12/4/2020 13:05	181.8	183.2	180.5	2185	64.95
12/4/2020 13:05	181.9	183.2	179.8	2185	65.08
12/4/2020 13:05	181.9	182.1	179.9	2192	65.16
12/4/2020 13:05	181.9	182.1	179.9	2191	64.95
12/4/2020 13:05	182	182.1	180.1	2188	64.99
12/4/2020 13:05	182	182.5	180.3	2192	65.01
12/4/2020 13:05	182	183.1	180.3	2190	64.99
12/4/2020 13:05	182	183	180	2190	64.88
12/4/2020 13:05	182	182.1	179.9	2189	65.04
12/4/2020 13:05	182.1	182.1	180.2	2185	64.84
12/4/2020 13:05	182.1	182.2	180.7	2185	65.04
12/4/2020 13:05	182.1	182	180.8	2182	64.92
12/4/2020 13:05	182.1	182.3	180.5	2178	64.83
12/4/2020 13:05	182.1	182.2	180	2183	64.88
12/4/2020 13:06	182.1	182.1	179.9	2183	65.04
12/4/2020 13:06	182.1	182.2	179.7	2182	64.99
12/4/2020 13:06	182.2	182.5	179.8	2181	64.96
12/4/2020 13:06	182.1	182.1	179.9	2181	65.04
12/4/2020 13:06	182.1	182.7	179.9	2181	64.96
12/4/2020 13:06	182.2	182	180	2181	65.04
12/4/2020 13:06	182.2	182.1	180.2	2181	65.09
12/4/2020 13:06	182.2	182.5	180.1	2187	64.92
12/4/2020 13:06	182.2	182.5	180.1	2185	64.92
12/4/2020 13:06	182.2	182.6	180.1	2185	65
12/4/2020 13:06	182.2	181.9	180.2	2185	64.96
12/4/2020 13:06	182.2	182.2	180.4	2181	64.96
12/4/2020 13:06	182.2	182.5	180.1	2182	65
12/4/2020 13:06	182.2	182.3	180.2	2178	64.92
12/4/2020 13:06	182.2	182.4	180.2	2180	64.92
12/4/2020 13:06	182.3	182	180.2	2184	64.96
12/4/2020 13:06	182.4	182.1	180.2	2185	64.92
12/4/2020 13:06	182.4	182.6	180.2	2187	64.92
12/4/2020 13:06	182.5	182.3	180.2	2187	65.01
12/4/2020 13:06	182.5	182.5	180.2	2182	64.96
12/4/2020 13:06	182.7	182	180.2	2183	64.96
12/4/2020 13:06	182.6	182.2	180.1	2183	65.04
12/4/2020 13:06	182.6	182	180.1	2183	65.04

12/4/2020 13:06	182.8	182	179.9	2182	65.01
12/4/2020 13:06	182.7	182.3	179.8	2183	64.88
12/4/2020 13:06	182.9	182.2	179.9	2183	65.08
12/4/2020 13:06	182.9	182.5	180.6	2181	64.96
12/4/2020 13:06	183	182	180.9	2184	64.92
12/4/2020 13:06	183	181.9	180.3	2180	64.88
12/4/2020 13:06	183.1	182.5	179.9	2181	64.92
12/4/2020 13:06	183.1	182.8	180.2	2182	65.04
12/4/2020 13:06	183.2	183	180.1	2181	65
12/4/2020 13:06	183.2	183.1	180.1	2180	65
12/4/2020 13:06	183.3	183.1	179.7	2181	65.04
12/4/2020 13:06	183.4	183	180.2	2178	65
12/4/2020 13:06	183.5	182.8	180.9	2180	64.96
12/4/2020 13:06	183.6	183	180.4	2181	65
12/4/2020 13:06	183.6	183.2	179.9	2179	64.96
12/4/2020 13:06	183.7	183	180.4	2184	64.92
12/4/2020 13:06	183.8	183.1	180.2	2186	64.96
12/4/2020 13:06	183.9	183	180.4	2182	64.92
12/4/2020 13:06	184	183	180	2185	64.96
12/4/2020 13:06	184	183.2	179.9	2185	65.12
12/4/2020 13:06	184.2	183	180.2	2184	65.08
12/4/2020 13:06	184.3	183	180.9	2181	65.12
12/4/2020 13:06	184.4	183.2	181.1	2180	65.08
12/4/2020 13:06	184.5	183.1	180.8	2182	65.08
12/4/2020 13:06	184.5	183.2	180.2	2183	65.12
12/4/2020 13:06	184.6	183.1	180	2184	65.08
12/4/2020 13:06	184.7	183.2	180.2	2180	65.04
12/4/2020 13:06	184.8	183.2	180.4	2181	65.04
12/4/2020 13:06	184.8	183	180.6	2181	64.96
12/4/2020 13:06	185	183.1	180.6	2176	65.08
12/4/2020 13:06	185.2	182.9	180.5	2176	65.09
12/4/2020 13:06	185.2	183	180.6	2178	64.96
12/4/2020 13:06	185.3	183.1	180.1	2173	65
12/4/2020 13:06	185.4	183.2	180.1	2174	65.04
12/4/2020 13:06	185.4	183.2	180.1	2175	64.96
12/4/2020 13:06	185.5	183	180.2	2177	64.96
12/4/2020 13:06	185.6	183	181	2175	64.92
12/4/2020 13:07	185.7	183.1	181.3	2175	65.04
12/4/2020 13:07	185.8	183	181.2	2180	65.04
12/4/2020 13:07	185.9	183	181	2181	65.08
12/4/2020 13:07	185.9	183	180.5	2180	65.01
12/4/2020 13:07	186.1	183	180.6	2183	64.96
12/4/2020 13:07	186	183.1	180.3	2181	65.04
12/4/2020 13:07	186.2	183.4	180.4	2187	64.92
12/4/2020 13:07	186.3	183.1	180.4	2188	64.96
12/4/2020 13:07	186.3	182.9	180.6	2189	65.04
12/4/2020 13:07	186.5	183	180.5	2190	65.05

12/4/2020 13:07	186.5	183.2	180.6	2187	65.04
12/4/2020 13:07	186.6	183.1	180.6	2188	64.96
12/4/2020 13:07	186.7	183.1	180.6	2190	65.05
12/4/2020 13:07	186.7	183.2	180.6	2192	65
12/4/2020 13:07	186.8	183	180.6	2192	64.96
12/4/2020 13:07	186.8	183.1	180.4	2192	65.13
12/4/2020 13:07	187	182.9	180.6	2192	64.99
12/4/2020 13:07	187	182.9	180.6	2190	65.04
12/4/2020 13:07	187	183.3	180.5	2194	65.17
12/4/2020 13:07	187.2	183	180.6	2192	65
12/4/2020 13:07	187.1	183	180.6	2192	65.03
12/4/2020 13:07	187.1	183	180.9	2192	65.12
12/4/2020 13:07	187.2	183	181.2	2189	65.03
12/4/2020 13:07	187.2	183	181.5	2189	65.04
12/4/2020 13:07	187.2	182.9	181.5	2189	65.08
12/4/2020 13:07	187.2	183.2	180.8	2187	65.03
12/4/2020 13:07	187.3	183.2	180.4	2189	64.92
12/4/2020 13:07	187.2	183.3	180.3	2190	65.01
12/4/2020 13:07	187.3	183	180.6	2189	64.92
12/4/2020 13:07	187.3	183	180.7	2190	65.04
12/4/2020 13:07	187.3	183	180.8	2189	64.96
12/4/2020 13:07	187.3	183.2	180.6	2187	65
12/4/2020 13:07	187.4	183.1	180.9	2190	65.08
12/4/2020 13:07	187.4	183.4	180.7	2192	65.09
12/4/2020 13:07	187.4	183	180.6	2197	65
12/4/2020 13:07	187.3	183.1	181	2194	64.92
12/4/2020 13:07	187.4	183	181.8	2193	65.05
12/4/2020 13:07	187.4	182.8	181.6	2192	64.92
12/4/2020 13:07	187.4	183.1	180.8	2192	65
12/4/2020 13:07	187.4	183	180.3	2193	65.12
12/4/2020 13:07	187.3	183	180.6	2190	65.04
12/4/2020 13:07	187.4	183	180.9	2193	65.04
12/4/2020 13:07	187.3	183	181	2192	64.96
12/4/2020 13:07	187.4	183.1	180.9	2191	64.92
12/4/2020 13:07	187.3	182.9	180.8	2192	65.08
12/4/2020 13:07	187.4	183	180.8	2194	65.01
12/4/2020 13:07	187.3	183.9	180.9	2193	65.04
12/4/2020 13:07	187.4	184.1	181.6	2193	65.04
12/4/2020 13:07	187.4	183.9	181.7	2192	65.05
12/4/2020 13:07	187.5	184.3	181.1	2191	65.04
12/4/2020 13:07	187.5	184.1	180.5	2192	64.99
12/4/2020 13:07	187.4	184.1	180.6	2189	64.96
12/4/2020 13:07	187.5	184.2	180.6	2186	64.99
12/4/2020 13:07	187.5	184.1	180.9	2189	64.92
12/4/2020 13:07	187.5	184.1	181.1	2192	65.01
12/4/2020 13:07	187.5	184.1	181.3	2193	64.96
12/4/2020 13:07	187.6	184.2	181	2189	64.96

12/4/2020 13:07	187.6	184.1	180.9	2192	65.05
12/4/2020 13:07	187.5	183.8	180.9	2192	64.92
12/4/2020 13:08	187.6	184.1	181.1	2194	64.92
12/4/2020 13:08	187.6	184.2	180.8	2195	65.01
12/4/2020 13:08	187.6	183.9	180.9	2195	65.04
12/4/2020 13:08	187.7	184	180.9	2189	64.96
12/4/2020 13:08	187.7	184	180.9	2195	65.08
12/4/2020 13:08	187.7	184.1	180.9	2195	65.03
12/4/2020 13:08	187.7	184.2	181.3	2198	64.88
12/4/2020 13:08	187.8	183.9	180.9	2202	64.96
12/4/2020 13:08	187.7	184.2	181.2	2203	64.96
12/4/2020 13:08	187.8	184.1	181	2203	64.96
12/4/2020 13:08	187.8	184.1	180.9	2200	65.08
12/4/2020 13:08	187.8	184.2	180.7	2204	65.04
12/4/2020 13:08	187.8	183.9	180.8	2200	64.96
12/4/2020 13:08	187.8	184.2	181.7	2196	65
12/4/2020 13:08	187.7	183.9	181.8	2197	64.96
12/4/2020 13:08	187.8	184.2	181.1	2197	65.04
12/4/2020 13:08	187.8	184.2	180.9	2199	65.04
12/4/2020 13:08	187.8	184.2	181.1	2197	64.96
12/4/2020 13:08	187.8	184	181.1	2196	64.96
12/4/2020 13:08	187.7	184.1	180.8	2196	65.04
12/4/2020 13:08	187.8	184.2	180.8	2193	64.92
12/4/2020 13:08	187.8	184.1	181.5	2195	64.92
12/4/2020 13:08	187.7	183.9	181.8	2196	64.97
12/4/2020 13:08	187.8	184.2	181.1	2195	64.88
12/4/2020 13:08	187.8	184.1	180.8	2197	65.04
12/4/2020 13:08	187.7	184	181.3	2195	64.88
12/4/2020 13:08	187.8	184.2	181.3	2194	64.92
12/4/2020 13:08	187.7	183.8	181.4	2197	65
12/4/2020 13:08	187.7	184.3	181	2195	64.88
12/4/2020 13:08	187.7	184.2	180.9	2196	64.96
12/4/2020 13:08	187.6	183.9	181.1	2192	65.04
12/4/2020 13:08	187.7	184.1	181.6	2193	65
12/4/2020 13:08	187.7	183.8	182	2192	65.08
12/4/2020 13:08	187.7	184.1	181.5	2195	64.99
12/4/2020 13:08	187.6	183.9	181.2	2197	64.96
12/4/2020 13:08	187.6	184	180.9	2196	64.88
12/4/2020 13:08	187.6	184.2	181.1	2196	65.04
12/4/2020 13:08	187.6	183.8	181.4	2201	64.88
12/4/2020 13:08	187.6	184.1	181.1	2198	64.96
12/4/2020 13:08	187.6	184.2	181.2	2192	65.04
12/4/2020 13:08	187.5	183.9	180.9	2193	64.96
12/4/2020 13:08	187.5	184.2	180.8	2192	64.99
12/4/2020 13:08	187.5	183.9	181.5	2193	65
12/4/2020 13:08	187.6	184.2	181.9	2193	65
12/4/2020 13:08	187.5	183.9	181.3	2191	64.96

12/4/2020 13:08	187.6	184	181.1	2192	65
12/4/2020 13:08	187.4	184.2	181.2	2191	65
12/4/2020 13:08	187.5	184.1	181.3	2196	64.96
12/4/2020 13:08	187.5	184.1	181.2	2198	65
12/4/2020 13:08	187.4	184.1	180.8	2196	64.96
12/4/2020 13:08	187.4	184	180.9	2193	64.96
12/4/2020 13:08	187.4	184.2	181.4	2191	65.08
12/4/2020 13:08	187.4	183.9	182.1	2197	65
12/4/2020 13:08	187.4	184.1	181.6	2196	65.03
12/4/2020 13:08	187.4	184.2	180.9	2192	65.04
12/4/2020 13:08	187.4	183.9	180.9	2197	64.92
12/4/2020 13:08	187.4	184.2	181.3	2191	64.87
12/4/2020 13:08	187.4	184.1	181.2	2192	65
12/4/2020 13:08	187.4	184.2	180.9	2194	64.92
12/4/2020 13:08	187.3	184	180.9	2193	65.08
12/4/2020 13:09	187.3	183.9	181.2	2191	64.96
12/4/2020 13:09	187.3	183.9	182	2189	64.96
12/4/2020 13:09	187.3	183.9	181.5	2193	65.03
12/4/2020 13:09	187.2	184.2	180.8	2194	64.93
12/4/2020 13:09	187.2	184.2	181.3	2193	65.08
12/4/2020 13:09	187.3	183.8	181.3	2192	65.04
12/4/2020 13:09	187.3	184.2	181.1	2189	64.96
12/4/2020 13:09	187.2	184	180.9	2189	64.97
12/4/2020 13:09	187.2	183.9	180.8	2192	64.84
12/4/2020 13:09	187.2	184.2	181.6	2189	64.96
12/4/2020 13:09	187.2	184.2	182	2189	64.96
12/4/2020 13:09	187.2	184.1	181.3	2189	64.88
12/4/2020 13:09	187.2	183.9	181.1	2191	65
12/4/2020 13:09	187.2	183.9	181.2	2189	65.12
12/4/2020 13:09	187.1	184.1	181.3	2185	64.91
12/4/2020 13:09	187.2	184.6	181.1	2185	64.96
12/4/2020 13:09	187.1	185.3	181	2187	65
12/4/2020 13:09	187.1	185.1	180.9	2187	64.92
12/4/2020 13:09	187.1	185.3	181.8	2189	64.96
12/4/2020 13:09	187	185.3	181.8	2190	65.04
12/4/2020 13:09	187.1	184.7	181.3	2190	64.96
12/4/2020 13:09	187	184.9	180.9	2191	64.96
12/4/2020 13:09	187	184.6	181.3	2198	64.96
12/4/2020 13:09	187	185.2	181.3	2192	64.95
12/4/2020 13:09	187	185.3	181.2	2188	65.04
12/4/2020 13:09	187	184.8	180.9	2191	64.96
12/4/2020 13:09	187	185.1	181.2	2190	64.92
12/4/2020 13:09	187	184.6	180.8	2189	64.99
12/4/2020 13:09	187	185.1	180.8	2196	65.04
12/4/2020 13:09	187	185.2	180.9	2194	64.84
12/4/2020 13:09	187	184.8	180.7	2192	65.08
12/4/2020 13:09	186.9	185.1	181.1	2194	65.16

12/4/2020 13:09	186.9	184.6	180.9	2189	64.99
12/4/2020 13:09	186.8	184.6	180.9	2191	64.99
12/4/2020 13:09	186.9	185.4	180.9	2188	65.08
12/4/2020 13:09	186.8	185	181	2188	64.84
12/4/2020 13:09	186.8	185.4	181.3	2189	64.88
12/4/2020 13:09	186.8	185.1	181.2	2185	64.93
12/4/2020 13:09	186.8	184.6	181.1	2184	64.91
12/4/2020 13:09	186.8	185.1	181.1	2185	64.92
12/4/2020 13:09	186.8	184.8	181.8	2185	65.17
12/4/2020 13:09	186.8	185.1	181.4	2188	64.96
12/4/2020 13:09	186.8	185.3	181	2185	64.87
12/4/2020 13:09	186.7	185.3	181.2	2192	65.09
12/4/2020 13:09	186.7	185	181.4	2191	64.99
12/4/2020 13:09	186.7	184.7	181	2193	64.96
12/4/2020 13:09	186.7	184.8	181.7	2192	65
12/4/2020 13:09	186.7	185.3	181.5	2190	64.96
12/4/2020 13:09	186.7	185.4	180.9	2190	64.99
12/4/2020 13:09	186.7	185.3	181.2	2193	65.04
12/4/2020 13:09	186.6	184.8	181.4	2191	64.96
12/4/2020 13:09	186.7	184.9	181.1	2194	64.92
12/4/2020 13:09	186.6	185	181.4	2197	65
12/4/2020 13:09	186.6	184.7	181.9	2194	64.87
12/4/2020 13:09	186.6	185.4	180.9	2196	64.92
12/4/2020 13:09	186.6	184.9	180.9	2199	65
12/4/2020 13:09	186.6	185	181.3	2199	64.91
12/4/2020 13:09	186.6	185.3	181	2199	64.96
12/4/2020 13:09	186.5	184.7	181.1	2201	65.04
12/4/2020 13:10	186.5	185.2	181.3	2203	65
12/4/2020 13:10	186.5	184.9	181.9	2203	65.04
12/4/2020 13:10	186.5	185.4	180.9	2202	64.96
12/4/2020 13:10	186.5	185.3	180.8	2202	64.91
12/4/2020 13:10	186.4	184.6	181.4	2203	65
12/4/2020 13:10	186.5	185.3	181.3	2207	65.12
12/4/2020 13:10	186.4	184.8	181	2202	64.88
12/4/2020 13:10	186.4	184.9	181.3	2199	64.92
12/4/2020 13:10	186.4	184.6	182	2197	65.08
12/4/2020 13:10	186.4	185.1	181.4	2194	64.83
12/4/2020 13:10	186.3	185.3	180.8	2196	64.84
12/4/2020 13:10	186.3	185.1	180.8	2197	65.08
12/4/2020 13:10	186.3	184.8	181.3	2193	64.99
12/4/2020 13:10	186.3	185.1	181.3	2192	64.88
12/4/2020 13:10	186.3	184.6	181	2195	64.96
12/4/2020 13:10	186.3	185.1	181.1	2192	64.83
12/4/2020 13:10	186.2	184.6	180.9	2194	64.99
12/4/2020 13:10	186.2	185.4	181.6	2194	64.92
12/4/2020 13:10	186.2	185.3	181.8	2192	64.95
12/4/2020 13:10	186.2	184.6	181.8	2192	65.04

12/4/2020 13:10	186.2	184.9	181.7	2192	65.08
12/4/2020 13:10	186.1	184.6	181.5	2191	64.79
12/4/2020 13:10	186.2	184.7	181.3	2195	64.92
12/4/2020 13:10	186	185.4	181.1	2195	65.08
12/4/2020 13:10	186.1	185	181	2194	65
12/4/2020 13:10	186.1	185.1	181.3	2192	64.83
12/4/2020 13:10	186	184.9	181.3	2199	64.96
12/4/2020 13:10	186	184.8	181.5	2199	65.04
12/4/2020 13:10	185.9	185.3	182	2195	65.03
12/4/2020 13:10	185.9	184.9	182.1	2192	65.08
12/4/2020 13:10	186	185.3	181.8	2193	64.95
12/4/2020 13:10	185.9	184.6	181.1	2193	65.04
12/4/2020 13:10	185.9	184.6	180.9	2193	65.08
12/4/2020 13:10	185.9	185.3	181	2194	64.87
12/4/2020 13:10	185.9	184.7	181.3	2192	64.83
12/4/2020 13:10	186.1	185.3	181	2196	65.12
12/4/2020 13:10	185.9	185	180.8	2196	64.92
12/4/2020 13:10	185.8	184.7	181.2	2194	64.92
12/4/2020 13:10	185.8	184.9	182	2196	65.04
12/4/2020 13:10	185.8	184.8	181.4	2198	64.87
12/4/2020 13:10	185.8	185.2	181.1	2199	64.99
12/4/2020 13:10	185.7	185	181.1	2199	65.04
12/4/2020 13:10	185.8	184.8	181.4	2199	64.87
12/4/2020 13:10	185.8	184.9	180.9	2200	64.96
12/4/2020 13:10	185.7	184.6	180.9	2202	65.09
12/4/2020 13:10	185.7	185.1	181.2	2199	64.96
12/4/2020 13:10	185.7	185.3	181.9	2199	64.92
12/4/2020 13:10	185.7	185.3	181.7	2197	65.09
12/4/2020 13:10	185.7	184.8	180.9	2193	64.87
12/4/2020 13:10	185.7	184.7	181	2197	65
12/4/2020 13:10	185.7	184.7	181.3	2197	64.96
12/4/2020 13:10	185.6	184.7	181.4	2198	64.87
12/4/2020 13:10	185.5	185.1	181.3	2194	65
12/4/2020 13:10	185.6	185.4	180.9	2194	65.04
12/4/2020 13:10	185.6	184.8	180.8	2192	64.96
12/4/2020 13:10	185.6	185.1	180.9	2188	64.92
12/4/2020 13:10	185.6	185.1	181.6	2193	65.09
12/4/2020 13:10	185.5	184.9	182	2193	64.91
12/4/2020 13:10	185.5	185	182	2193	64.96
12/4/2020 13:11	185.5	184.8	181.5	2193	65
12/4/2020 13:11	185.5	184.8	181.1	2194	64.91
12/4/2020 13:11	185.5	185.2	180.9	2192	64.99
12/4/2020 13:11	185.5	185.1	180.9	2198	65.12
12/4/2020 13:11	185.4	185.3	181.1	2193	64.95
12/4/2020 13:11	185.4	185.1	181.1	2192	64.92
12/4/2020 13:11	185.4	185.4	181.2	2189	65.04
12/4/2020 13:11	185.4	184.8	181.1	2189	64.83

12/4/2020 13:11	185.4	184.7	180.9	2189	64.95
12/4/2020 13:11	185.4	185.3	180.8	2189	65
12/4/2020 13:11	185.4	184.9	180.8	2189	64.88
12/4/2020 13:11	185.4	185.2	180.7	2189	65.04
12/4/2020 13:11	185.4	184.6	180.8	2194	65.04
12/4/2020 13:11	185.3	184.8	180.9	2195	64.95
12/4/2020 13:11	185.3	185.3	181.3	2201	64.92
12/4/2020 13:11	185.3	185.2	181.6	2201	64.96
12/4/2020 13:11	185.3	185.3	181.6	2198	64.8
12/4/2020 13:11	185.3	184.9	181.9	2196	64.99
12/4/2020 13:11	185.3	184.7	181.8	2201	64.92
12/4/2020 13:11	185.3	185.1	181.8	2197	64.99
12/4/2020 13:11	185.2	185.2	181.8	2196	64.92
12/4/2020 13:11	185.2	185.3	181.6	2195	65
12/4/2020 13:11	185.2	185.1	181.6	2193	65.03
12/4/2020 13:11	185.2	184.9	181.6	2193	64.92
12/4/2020 13:11	185.2	185	181.3	2191	64.88
12/4/2020 13:11	185.2	184.7	181.1	2194	64.99
12/4/2020 13:11	185.2	185.2	181	2192	64.99
12/4/2020 13:11	185.2	185.3	180.8	2195	64.88
12/4/2020 13:11	185.2	184.9	180.8	2198	64.8
12/4/2020 13:11	185.2	184.8	180.8	2197	64.92
12/4/2020 13:11	185.2	185.1	180.8	2201	64.92
12/4/2020 13:11	185.1	184.6	180.9	2202	64.99
12/4/2020 13:11	185.1	184.8	180.7	2204	64.92
12/4/2020 13:11	185.2	184.8	180.8	2207	65.03
12/4/2020 13:11	185	185.3	180.8	2202	65.04
12/4/2020 13:11	185.1	185.1	180.8	2197	65.08
12/4/2020 13:11	185.1	185.3	181.1	2195	65.04
12/4/2020 13:11	185	185.1	180.9	2194	64.83
12/4/2020 13:11	185	184.6	181.1	2192	64.99
12/4/2020 13:11	185	184.8	181.4	2191	64.84
12/4/2020 13:11	185	185.1	181.5	2191	65
12/4/2020 13:11	185	185.4	181.6	2195	64.92
12/4/2020 13:11	185	185.1	181.7	2193	65
12/4/2020 13:11	185	184.9	181.8	2193	64.92
12/4/2020 13:11	184.9	184.7	181.8	2193	64.96
12/4/2020 13:11	184.9	185.3	181.7	2196	64.99
12/4/2020 13:11	185	184.8	181.5	2195	65
12/4/2020 13:11	185	185.2	181.4	2195	64.99
12/4/2020 13:11	184.9	185	181	2195	65.04
12/4/2020 13:11	185	185.3	180.9	2196	65
12/4/2020 13:11	184.9	185.3	180.8	2195	65
12/4/2020 13:11	184.9	185	180.6	2200	65.09
12/4/2020 13:11	184.9	185.2	180.7	2200	64.96
12/4/2020 13:11	184.9	184.9	180.9	2197	64.96
12/4/2020 13:11	184.9	184.7	180.8	2199	65.04

12/4/2020 13:11	184.9	184.8	181.2	2203	64.96
12/4/2020 13:11	184.8	184.9	181	2199	65
12/4/2020 13:11	184.8	185.2	181.1	2202	65
12/4/2020 13:11	184.9	184.9	181.2	2202	64.96
12/4/2020 13:11	184.8	185.3	180.9	2200	64.96
12/4/2020 13:12	184.8	184.8	181	2196	65.04
12/4/2020 13:12	184.8	185.3	180.7	2203	64.88
12/4/2020 13:12	184.8	185.1	181.1	2201	65.04
12/4/2020 13:12	184.8	185.1	181.3	2201	65
12/4/2020 13:12	184.7	185.3	181.5	2201	64.92
12/4/2020 13:12	184.7	184.9	181.8	2199	64.92
12/4/2020 13:12	184.7	184.8	182	2199	64.92
12/4/2020 13:12	184.7	185.1	181.8	2196	64.96
12/4/2020 13:12	184.7	185.2	181.4	2196	64.92
12/4/2020 13:12	184.6	185.4	181.1	2193	64.92
12/4/2020 13:12	184.7	184.9	181.1	2191	65.04
12/4/2020 13:12	184.7	184.5	181.1	2190	65
12/4/2020 13:12	184.7	185.1	180.9	2196	65.08
12/4/2020 13:12	184.7	185.2	180.9	2199	65.08
12/4/2020 13:12	184.6	185.2	181	2198	64.96
12/4/2020 13:12	184.6	185.3	181.3	2200	65.08
12/4/2020 13:12	184.6	184.8	181.6	2199	64.96
12/4/2020 13:12	184.6	184.8	181.9	2199	64.92
12/4/2020 13:12	184.6	184.6	181.8	2196	65.05
12/4/2020 13:12	184.6	185.2	181.3	2196	64.96
12/4/2020 13:12	184.6	185.3	181	2197	64.96
12/4/2020 13:12	184.6	184.7	180.8	2196	65.12
12/4/2020 13:12	184.6	184.9	181.2	2197	64.96
12/4/2020 13:12	184.5	185.2	181.3	2200	65.04
12/4/2020 13:12	184.5	184.7	181.1	2199	65
12/4/2020 13:12	184.5	185.3	181.1	2200	64.92
12/4/2020 13:12	184.5	185.1	180.8	2205	64.96
12/4/2020 13:12	184.5	185.3	180.6	2203	65
12/4/2020 13:12	184.5	185.3	181.1	2202	64.95
12/4/2020 13:12	184.4	184.6	181.5	2201	65.04
12/4/2020 13:12	184.4	185.3	181.8	2198	65.05
12/4/2020 13:12	184.4	185	181.1	2196	64.92
12/4/2020 13:12	184.4	185.2	180.8	2198	65.04
12/4/2020 13:12	184.4	185.1	181	2203	64.96
12/4/2020 13:12	184.4	185.2	181	2199	64.99
12/4/2020 13:12	184.4	185.3	181	2199	64.96
12/4/2020 13:12	184.4	185	180.9	2197	64.92
12/4/2020 13:12	184.2	185.3	180.9	2196	64.88
12/4/2020 13:12	184.3	185.1	180.8	2203	64.88
12/4/2020 13:12	184.2	184.7	180.7	2201	64.88
12/4/2020 13:12	184.2	185.1	180.6	2203	64.88
12/4/2020 13:12	184.2	184.7	180.9	2202	65.03

12/4/2020 13:12	184.2	185.3	180.9	2200	65.12
12/4/2020 13:12	184.2	185.1	181.2	2195	65.04
12/4/2020 13:12	184.2	184.6	181.3	2197	65.08
12/4/2020 13:12	184.2	185.1	181.3	2194	65.05
12/4/2020 13:12	184.2	184.9	181.3	2193	64.87
12/4/2020 13:12	184.1	185.4	180.8	2201	65
12/4/2020 13:12	184.1	184.9	180.8	2200	65.04
12/4/2020 13:12	184.1	185	180.6	2196	64.92
12/4/2020 13:12	184.1	185.3	180.6	2196	64.99
12/4/2020 13:12	184.1	184.9	180.6	2195	65.04
12/4/2020 13:12	184.1	185.3	180.9	2195	64.99
12/4/2020 13:12	184	184	181.1	2196	65
12/4/2020 13:12	184	184.1	181.3	2196	65.08
12/4/2020 13:12	184	184.2	181.1	2192	65.04
12/4/2020 13:12	184	184	181	2197	65.08
12/4/2020 13:12	184	183.9	180.9	2198	64.96
12/4/2020 13:12	184	184.2	180.9	2206	64.93
12/4/2020 13:13	184	183.9	181.6	2207	64.92
12/4/2020 13:13	184	184.2	181.9	2206	65.12
12/4/2020 13:13	184	184.2	181	2206	64.88
12/4/2020 13:13	184	184.1	180.6	2203	65.03
12/4/2020 13:13	183.9	184	180.7	2203	65.08
12/4/2020 13:13	184	183.9	180.6	2204	64.99
12/4/2020 13:13	184	184.1	180.9	2203	65
12/4/2020 13:13	184	184.1	180.8	2204	65.12
12/4/2020 13:13	183.9	184.2	180.9	2203	64.96
12/4/2020 13:13	183.9	184.2	181.2	2202	65.04
12/4/2020 13:13	183.9	183.9	180.9	2205	65.04
12/4/2020 13:13	183.9	184.2	181.1	2202	64.96
12/4/2020 13:13	183.8	184	180.8	2196	64.96
12/4/2020 13:13	183.9	183.9	181	2197	64.96
12/4/2020 13:13	183.8	184.2	181.1	2196	65.05
12/4/2020 13:13	183.9	183.9	180.9	2196	65.04
12/4/2020 13:13	183.9	184.2	181	2194	64.92
12/4/2020 13:13	183.8	184.1	180.9	2198	65.04
12/4/2020 13:13	183.8	183.9	180.8	2198	65
12/4/2020 13:13	183.7	184	180.8	2201	64.96
12/4/2020 13:13	183.8	184	180.8	2206	64.93
12/4/2020 13:13	183.8	184.1	180.9	2206	64.92
12/4/2020 13:13	183.8	183.9	180.9	2202	65.04
12/4/2020 13:13	183.7	183.9	180.9	2197	65.04
12/4/2020 13:13	183.7	184	180.9	2198	64.84
12/4/2020 13:13	183.7	184.1	180.9	2199	64.92
12/4/2020 13:13	183.7	184.1	180.8	2199	65.04
12/4/2020 13:13	183.7	184	180.9	2199	65.04
12/4/2020 13:13	183.7	184	181.1	2203	64.84
12/4/2020 13:13	183.7	184.2	180.9	2200	65.08

12/4/2020 13:13	183.7	184.1	181	2202	65
12/4/2020 13:13	183.7	184.2	181.1	2200	64.88
12/4/2020 13:13	183.6	184.3	180.9	2203	65.05
12/4/2020 13:13	183.7	184.2	180.8	2203	65
12/4/2020 13:13	183.7	184.1	180.8	2203	65.04
12/4/2020 13:13	183.6	183.9	180.7	2200	65.09
12/4/2020 13:13	183.6	184.2	180.7	2200	65
12/4/2020 13:13	183.6	183.8	180.6	2202	64.96
12/4/2020 13:13	183.6	184.1	180.9	2207	65.01
12/4/2020 13:13	183.6	184.2	181.5	2206	64.88
12/4/2020 13:13	183.6	183.9	181.6	2207	64.92
12/4/2020 13:13	183.5	184.1	181.3	2205	65
12/4/2020 13:13	183.5	183.9	180.7	2205	64.88
12/4/2020 13:13	183.5	184.1	180.8	2207	64.92
12/4/2020 13:13	183.5	184.2	180.8	2205	65.08
12/4/2020 13:13	183.4	183.9	180.8	2207	64.99
12/4/2020 13:13	183.5	184.2	181.1	2203	65.08
12/4/2020 13:13	183.4	184.2	181.1	2206	65.12
12/4/2020 13:13	183.4	184.2	181	2200	64.92
12/4/2020 13:13	183.4	184.2	180.9	2204	65
12/4/2020 13:13	183.3	184.1	180.8	2201	65.12
12/4/2020 13:13	183.3	184.1	180.6	2203	64.92
12/4/2020 13:13	183.3	183.9	180.6	2199	64.96
12/4/2020 13:13	183.3	184.1	180.4	2198	65
12/4/2020 13:13	183.3	184.1	181.1	2195	64.92
12/4/2020 13:13	183.2	184	181.6	2198	64.92
12/4/2020 13:13	183.2	184.1	181.5	2203	65.04
12/4/2020 13:13	183.2	183.9	181.2	2204	64.92
12/4/2020 13:13	183.2	183.9	180.6	2205	65.04
12/4/2020 13:13	183.3	184.2	180.9	2206	65
12/4/2020 13:14	183.3	184.1	181.1	2204	65.04
12/4/2020 13:14	183.3	183.8	180.9	2204	64.96
12/4/2020 13:14	183.3	184.2	180.6	2207	65.12
12/4/2020 13:14	183.3	184.1	180.8	2203	65.08
12/4/2020 13:14	183.4	184.1	181.6	2205	64.96
12/4/2020 13:14	183.4	183.8	181.3	2207	65.12
12/4/2020 13:14	183.4	184.1	180.7	2207	64.88
12/4/2020 13:14	183.5	183.8	180.9	2210	65.08
12/4/2020 13:14	183.4	184.2	181	2207	65.05
12/4/2020 13:14	183.4	184.1	180.8	2207	65.04
12/4/2020 13:14	183.6	183.9	180.4	2210	64.92
12/4/2020 13:14	183.5	184	180.9	2210	65.12
12/4/2020 13:14	183.6	184	181.7	2207	64.99
12/4/2020 13:14	183.7	183.9	181.2	2209	65.08
12/4/2020 13:14	183.7	184.2	180.6	2210	65.12
12/4/2020 13:14	183.7	184.1	180.9	2203	65
12/4/2020 13:14	183.7	184.1	180.9	2201	65

12/4/2020 13:14	183.8	184	180.7	2205	65
12/4/2020 13:14	183.9	183.9	180.6	2203	64.92
12/4/2020 13:14	183.9	184.1	181	2202	65.04
12/4/2020 13:14	183.9	184.1	181.7	2198	65.08
12/4/2020 13:14	184	184.1	181.2	2202	65.04
12/4/2020 13:14	184	184.1	180.8	2202	64.92
12/4/2020 13:14	184.2	184.1	180.8	2202	65.16
12/4/2020 13:14	184.1	184.1	180.9	2198	64.93
12/4/2020 13:14	184.2	184.1	180.7	2196	65.04
12/4/2020 13:14	184.2	184.2	180.6	2203	65.05
12/4/2020 13:14	184.2	184.2	180.8	2202	64.92
12/4/2020 13:14	184.3	183.8	181.5	2204	64.92
12/4/2020 13:14	184.3	184.1	181.4	2203	65
12/4/2020 13:14	184.4	184.1	180.6	2203	64.88
12/4/2020 13:14	184.4	184	180.8	2203	64.96
12/4/2020 13:14	184.4	184.2	180.9	2210	65.17
12/4/2020 13:14	184.4	184	180.9	2207	65
12/4/2020 13:14	184.5	184.2	180.8	2203	64.92
12/4/2020 13:14	184.5	184	180.6	2199	65.09
12/4/2020 13:14	184.5	184.1	180.6	2198	65
12/4/2020 13:14	184.6	184.2	181.6	2199	65.04
12/4/2020 13:14	184.6	184	181.3	2200	65
12/4/2020 13:14	184.5	184.2	180.8	2202	64.96
12/4/2020 13:14	184.6	184.1	180.7	2199	65
12/4/2020 13:14	184.6	184.1	180.9	2202	65.09
12/4/2020 13:14	184.6	184.2	180.8	2199	64.92
12/4/2020 13:14	184.6	183.9	180.8	2203	65.04
12/4/2020 13:14	184.5	184.1	180.6	2203	65.09
12/4/2020 13:14	184.6	184.1	180.6	2201	64.92
12/4/2020 13:14	184.6	184	181.2	2198	64.88
12/4/2020 13:14	184.5	183.9	181.8	2202	65.08
12/4/2020 13:14	184.5	183.9	181.1	2205	64.92
12/4/2020 13:14	184.6	184.1	180.6	2201	64.92
12/4/2020 13:14	184.5	184	180.9	2199	64.96
12/4/2020 13:14	184.5	184	181.3	2199	64.83
12/4/2020 13:14	184.6	183.9	181	2201	65.08
12/4/2020 13:14	184.5	184	180.8	2198	64.96
12/4/2020 13:14	184.6	184.1	180.8	2197	65.08
12/4/2020 13:14	184.6	184.2	181.6	2196	64.88
12/4/2020 13:14	184.6	183.9	181.5	2200	65.08
12/4/2020 13:14	184.6	184.2	180.8	2198	64.96
12/4/2020 13:14	184.6	184	181.3	2196	65.12
12/4/2020 13:15	184.7	184.2	181.2	2195	65.08
12/4/2020 13:15	184.7	184.1	181.1	2191	64.92
12/4/2020 13:15	184.8	184.1	180.8	2196	65
12/4/2020 13:15	184.8	184.1	180.7	2197	65
12/4/2020 13:15	184.8	183.9	181.4	2197	65

12/4/2020 13:15	184.9	184.1	181.8	2195	64.96
12/4/2020 13:15	184.9	184.2	180.9	2192	64.99
12/4/2020 13:15	185	184	180.9	2194	65
12/4/2020 13:15	185	184.2	181.2	2195	64.91
12/4/2020 13:15	185.1	184.1	181	2199	65.12
12/4/2020 13:15	185.2	184.1	180.8	2201	65.04
12/4/2020 13:15	185.2	184	180.8	2199	65.04
12/4/2020 13:15	185.4	184	181.8	2197	65.04
12/4/2020 13:15	185.3	184.2	181.8	2196	64.84
12/4/2020 13:15	185.4	184	181.1	2198	64.92
12/4/2020 13:15	185.4	184	180.8	2198	64.88
12/4/2020 13:15	185.5	183.9	181	2203	64.96
12/4/2020 13:15	185.5	184	181	2201	64.88
12/4/2020 13:15	185.7	184.2	181.3	2199	65.08
12/4/2020 13:15	185.8	184.2	181.3	2198	64.99
12/4/2020 13:15	185.8	184.1	181.2	2197	65.08
12/4/2020 13:15	185.9	184	181.1	2196	64.88
12/4/2020 13:15	185.9	183.8	180.8	2198	64.92
12/4/2020 13:15	185.9	184.2	180.7	2195	64.92
12/4/2020 13:15	186.1	183.9	180.7	2197	64.92
12/4/2020 13:15	186.2	184.2	180.8	2196	64.96
12/4/2020 13:15	186.2	184.2	180.9	2193	64.92
12/4/2020 13:15	186.4	183.9	181	2194	64.99
12/4/2020 13:15	186.4	184.1	180.9	2197	65.12
12/4/2020 13:15	186.5	183.9	181.1	2196	64.95
12/4/2020 13:15	186.7	184.2	180.7	2196	65.08
12/4/2020 13:15	186.7	184	180.6	2194	65
12/4/2020 13:15	186.8	183.9	181.1	2194	64.91
12/4/2020 13:15	187	184.2	181.1	2196	64.96
12/4/2020 13:15	187.1	184.1	181.4	2198	65.09
12/4/2020 13:15	187.2	184	181.6	2197	64.92
12/4/2020 13:15	187.2	184.2	181.5	2196	64.92
12/4/2020 13:15	187.3	184.1	181.5	2197	65.04
12/4/2020 13:15	187.4	184.1	181.3	2195	65
12/4/2020 13:15	187.5	184.2	180.9	2196	65
12/4/2020 13:15	187.7	184.1	181.5	2199	65
12/4/2020 13:15	187.7	184.1	181.7	2195	64.91
12/4/2020 13:15	187.8	184	182.3	2198	64.92
12/4/2020 13:15	187.9	184.1	181.5	2194	65.05
12/4/2020 13:15	188	183.9	181.1	2195	64.88
12/4/2020 13:15	188.1	184.6	181.3	2195	64.92
12/4/2020 13:15	188.2	185.3	181.6	2196	64.96
12/4/2020 13:15	188.3	184.8	181.5	2201	64.92
12/4/2020 13:15	188.4	184.9	181.5	2203	65.04
12/4/2020 13:15	188.4	185.3	181.2	2205	64.92
12/4/2020 13:15	188.5	185.2	181.8	2207	64.8
12/4/2020 13:15	188.6	185.1	182.2	2207	64.96

12/4/2020 13:15	188.7	184.7	181.6	2207	64.92
12/4/2020 13:15	188.8	185.4	181.4	2205	64.93
12/4/2020 13:15	188.8	185.2	181.5	2204	64.92
12/4/2020 13:15	188.8	184.6	181.5	2205	64.96
12/4/2020 13:15	189	185.1	181.8	2209	65.03
12/4/2020 13:15	188.9	184.7	181.5	2207	64.84
12/4/2020 13:15	189.1	185.3	181.5	2208	65.12
12/4/2020 13:16	189.1	185.1	182.2	2203	64.92
12/4/2020 13:16	189.1	184.8	182.3	2208	64.96
12/4/2020 13:16	189.2	185.2	181.6	2209	65.04
12/4/2020 13:16	189.3	185.1	181.5	2207	64.99
12/4/2020 13:16	189.3	184.8	181.5	2208	64.92
12/4/2020 13:16	189.3	184.8	181.8	2207	64.92
12/4/2020 13:16	189.4	185	181.7	2209	64.92
12/4/2020 13:16	189.4	185.3	181.7	2205	64.95
12/4/2020 13:16	189.5	185.1	181.6	2208	64.92
12/4/2020 13:16	189.4	185.3	181.5	2203	65
12/4/2020 13:16	189.5	184.9	181.8	2209	65.04
12/4/2020 13:16	189.6	184.7	181.9	2210	65
12/4/2020 13:16	189.6	185.1	182.2	2209	64.96
12/4/2020 13:16	189.7	185.2	182.4	2210	64.96
12/4/2020 13:16	189.8	184.8	182.6	2209	64.96
12/4/2020 13:16	189.7	185.3	182.5	2212	64.96
12/4/2020 13:16	189.8	184.6	182.5	2210	64.92
12/4/2020 13:16	189.8	185.3	182.2	2211	64.91
12/4/2020 13:16	189.8	185.1	182.2	2210	64.96
12/4/2020 13:16	189.8	184.8	181.8	2207	64.96
12/4/2020 13:16	189.9	185.3	181.6	2209	65
12/4/2020 13:16	190	185.1	181.9	2209	65
12/4/2020 13:16	190	185.2	181.9	2207	64.88
12/4/2020 13:16	190.1	185.2	182	2207	64.92
12/4/2020 13:16	190.1	184.7	181.9	2212	65
12/4/2020 13:16	190	185.3	181.6	2214	64.83
12/4/2020 13:16	190.2	184.9	182.6	2214	64.99
12/4/2020 13:16	190.3	185.1	182.7	2209	65
12/4/2020 13:16	190.2	185.2	181.7	2207	64.88
12/4/2020 13:16	190.3	184.6	182.1	2209	64.96
12/4/2020 13:16	190.3	184.8	182.1	2207	65.08
12/4/2020 13:16	190.3	184.7	182.3	2207	64.84
12/4/2020 13:16	190.5	184.9	182	2207	64.84
12/4/2020 13:16	190.4	185.3	181.7	2212	64.88
12/4/2020 13:16	190.5	184.6	182.5	2211	65.04
12/4/2020 13:16	190.5	185.2	182.8	2214	65.04
12/4/2020 13:16	190.6	184.7	181.9	2216	65.04
12/4/2020 13:16	190.7	185.2	182	2219	64.88
12/4/2020 13:16	190.6	184.7	182.1	2216	64.92
12/4/2020 13:16	190.6	185	182.2	2214	65.12

12/4/2020 13:16	190.7	185.3	182.1	2210	64.88
12/4/2020 13:16	190.7	185.1	181.6	2213	64.88
12/4/2020 13:16	190.7	186.3	182	2212	64.96
12/4/2020 13:16	190.7	185.7	182.6	2216	64.88
12/4/2020 13:16	190.7	185.7	182.8	2218	65.08
12/4/2020 13:16	190.8	186.1	182.9	2214	65
12/4/2020 13:16	190.8	185.5	182.2	2216	64.92
12/4/2020 13:16	190.8	186.3	182.2	2214	64.92
12/4/2020 13:16	190.8	186.3	182.2	2218	65.09
12/4/2020 13:16	190.8	185.7	182.1	2217	64.96
12/4/2020 13:16	190.8	186.2	182.2	2220	64.92
12/4/2020 13:16	190.9	185.9	182.1	2221	65.05
12/4/2020 13:16	190.8	185.7	182.2	2222	64.92
12/4/2020 13:16	190.9	186.3	182.3	2218	65
12/4/2020 13:16	190.9	185.6	182.2	2218	65
12/4/2020 13:16	190.9	186.2	182.2	2217	64.84
12/4/2020 13:16	190.8	186.1	182.3	2214	64.99
12/4/2020 13:16	190.8	185.9	182.2	2220	65.01
12/4/2020 13:16	190.9	186.4	182.2	2216	64.99
12/4/2020 13:16	190.9	185.9	182.1	2219	64.96
12/4/2020 13:17	190.9	186.3	182.2	2224	64.96
12/4/2020 13:17	190.9	186	182.2	2225	64.88
12/4/2020 13:17	190.9	185.8	182.1	2219	64.96
12/4/2020 13:17	190.9	186.3	182	2218	64.96
12/4/2020 13:17	190.9	186.2	182	2216	64.84
12/4/2020 13:17	190.9	186.3	181.9	2214	65.08
12/4/2020 13:17	190.9	186.2	181.9	2222	64.96
12/4/2020 13:17	190.9	185.6	182.2	2221	65.04
12/4/2020 13:17	190.9	186	182.2	2215	64.84
12/4/2020 13:17	190.9	185.9	182.1	2214	64.93
12/4/2020 13:17	190.9	186.2	182	2214	64.99
12/4/2020 13:17	190.9	186.1	182	2211	64.92
12/4/2020 13:17	190.9	185.6	182.2	2211	65.01
12/4/2020 13:17	190.9	186.5	182.1	2212	64.88
12/4/2020 13:17	190.9	186.5	182.2	2215	64.96
12/4/2020 13:17	190.9	186.5	182.2	2220	65.12
12/4/2020 13:17	190.9	186.3	182.1	2219	64.92
12/4/2020 13:17	190.9	186.4	182.2	2221	65
12/4/2020 13:17	190.9	186.1	182	2217	64.96
12/4/2020 13:17	190.9	185.6	182.2	2213	64.87
12/4/2020 13:17	190.9	185.8	182.3	2214	64.96
12/4/2020 13:17	190.8	185.6	182.2	2218	65.04
12/4/2020 13:17	190.9	185.7	182.2	2218	64.96
12/4/2020 13:17	190.9	186.4	182.6	2220	65.04
12/4/2020 13:17	190.9	185.8	182.5	2218	65.04
12/4/2020 13:17	190.8	185.8	182.6	2216	64.96
12/4/2020 13:17	190.9	185.5	183	2210	65

12/4/2020 13:17	190.8	186.2	182.5	2217	65.04
12/4/2020 13:17	190.8	186.5	182.5	2220	64.99
12/4/2020 13:17	190.8	185.9	182.4	2221	64.99
12/4/2020 13:17	190.8	185.9	182.3	2222	65.04
12/4/2020 13:17	190.8	185.9	182.8	2221	64.91
12/4/2020 13:17	190.8	186.1	183.2	2220	65.04
12/4/2020 13:17	190.7	186.3	183.2	2218	64.92
12/4/2020 13:17	190.7	186.1	182.8	2220	65.04
12/4/2020 13:17	190.7	186.5	182.5	2218	64.88
12/4/2020 13:17	190.7	185.8	182.3	2220	65.04
12/4/2020 13:17	190.7	185.6	182.3	2224	64.88
12/4/2020 13:17	190.7	186.4	182.3	2219	64.88
12/4/2020 13:17	190.7	186.1	182.8	2219	64.99
12/4/2020 13:17	190.6	185.9	182.7	2220	65.08
12/4/2020 13:17	190.7	185.6	182.8	2216	64.83
12/4/2020 13:17	190.6	185.6	182.8	2219	64.92
12/4/2020 13:17	190.7	186.2	182.8	2220	65.08
12/4/2020 13:17	190.7	185.8	182.9	2216	64.99
12/4/2020 13:17	190.7	185.8	182.6	2219	64.88
12/4/2020 13:17	190.7	186.1	182.7	2219	65.16
12/4/2020 13:17	190.6	185.5	182.8	2219	64.95
12/4/2020 13:17	190.6	187.3	182.6	2223	64.96
12/4/2020 13:17	190.5	187.4	182.5	2222	65.01
12/4/2020 13:17	190.6	187.2	182.5	2218	64.87
12/4/2020 13:17	190.6	187.3	182.3	2221	64.96
12/4/2020 13:17	190.6	186.9	182.5	2224	65
12/4/2020 13:17	190.6	187.1	182.5	2224	64.92
12/4/2020 13:17	190.5	186.8	182.5	2221	65
12/4/2020 13:17	190.5	187.3	182.3	2219	65.09
12/4/2020 13:17	190.5	187.1	182.4	2216	64.96
12/4/2020 13:17	190.5	186.8	182.5	2216	64.96
12/4/2020 13:17	190.5	187	182.4	2219	65.09
12/4/2020 13:18	190.5	187.4	182.3	2221	64.96
12/4/2020 13:18	190.5	187.2	182.7	2221	64.96
12/4/2020 13:18	190.5	187.2	182.5	2222	65.08
12/4/2020 13:18	190.5	186.9	182.5	2221	65.04
12/4/2020 13:18	190.5	187.5	182.7	2218	64.96
12/4/2020 13:18	190.4	187	182.5	2216	65.04
12/4/2020 13:18	190.4	186.8	182.7	2218	64.95
12/4/2020 13:18	190.4	187.2	183	2220	64.88
12/4/2020 13:18	190.4	186.9	182.9	2221	65.09
12/4/2020 13:18	190.4	187.3	182.9	2221	64.99
12/4/2020 13:18	190.4	187.2	183	2217	64.88
12/4/2020 13:18	190.4	186.7	182.7	2220	64.96
12/4/2020 13:18	190.4	187	183	2221	65.04
12/4/2020 13:18	190.4	186.8	182.7	2221	64.92
12/4/2020 13:18	190.3	187.1	182.9	2225	65.01

12/4/2020 13:18	190.3	187.3	183	2221	64.99
12/4/2020 13:18	190.3	187.2	183	2219	65.04
12/4/2020 13:18	190.3	187.1	182.8	2217	64.92
12/4/2020 13:18	190.2	186.9	183	2216	64.88
12/4/2020 13:18	190.3	186.9	182.8	2216	64.99
12/4/2020 13:18	190.3	187.2	183.1	2223	65
12/4/2020 13:18	190.3	187.1	182.8	2225	64.92
12/4/2020 13:18	190.3	187.2	183	2221	64.92
12/4/2020 13:18	190.3	187	182.9	2221	64.92
12/4/2020 13:18	190.3	187.2	182.8	2214	65.08
12/4/2020 13:18	190.2	187.4	182.6	2215	64.92
12/4/2020 13:18	190.2	186.7	182.5	2220	64.96
12/4/2020 13:18	190.2	187.1	182.5	2223	64.96
12/4/2020 13:18	190.3	187	183.4	2221	64.96
12/4/2020 13:18	190.3	187.2	183.6	2220	65.04
12/4/2020 13:18	190.3	187	183.1	2218	64.96
12/4/2020 13:18	190.3	186.8	182.8	2221	64.92
12/4/2020 13:18	190.2	187	182.9	2221	64.96
12/4/2020 13:18	190.3	187	183	2219	65.08
12/4/2020 13:18	190.3	186.9	182.6	2219	65
12/4/2020 13:18	190.1	186.9	182.6	2224	64.99
12/4/2020 13:18	190.2	186.7	183.6	2224	64.92
12/4/2020 13:18	190.1	187.3	183.4	2229	64.92
12/4/2020 13:18	190.2	187	182.8	2223	65
12/4/2020 13:18	190.2	187.2	182.9	2225	65.03
12/4/2020 13:18	190.2	186.8	182.9	2224	65.08
12/4/2020 13:18	190.3	186.8	183	2223	65.08
12/4/2020 13:18	190.1	187.1	182.7	2225	65.08
12/4/2020 13:18	190.1	187.3	182.5	2219	65.04
12/4/2020 13:18	190.2	187.3	183.4	2221	65.04
12/4/2020 13:18	190.1	187.2	183.6	2228	64.92
12/4/2020 13:18	190.1	186.9	183	2225	65.04
12/4/2020 13:18	190.1	187	182.5	2223	65.04
12/4/2020 13:18	190.1	186.7	183	2219	65.04
12/4/2020 13:18	190.1	187.2	183.1	2219	65.08
12/4/2020 13:18	190.1	187.3	183.1	2221	65.08
12/4/2020 13:18	190.1	186.9	182.7	2224	64.92
12/4/2020 13:18	190.1	187.1	182.7	2225	64.83
12/4/2020 13:18	190.1	186.7	183	2225	64.96
12/4/2020 13:18	190.1	187.2	183.8	2224	65.04
12/4/2020 13:18	190.1	187.3	183.9	2227	64.92
12/4/2020 13:18	190.1	187.1	183.2	2224	65.04
12/4/2020 13:18	190	187	182.7	2227	65.04
12/4/2020 13:18	190	187.1	182.9	2227	65
12/4/2020 13:18	190.1	186.9	182.9	2226	65
12/4/2020 13:19	190.1	187.2	183	2226	64.91
12/4/2020 13:19	190	186.9	183	2229	65

12/4/2020 13:19	190.1	187.3	182.7	2227	65
12/4/2020 13:19	190	187	182.9	2225	65
12/4/2020 13:19	190	186.7	183.6	2225	65.04
12/4/2020 13:19	190	186.9	183.7	2220	64.96
12/4/2020 13:19	190	187	183	2218	65
12/4/2020 13:19	190	187.1	182.8	2216	64.92
12/4/2020 13:19	190	187	183.3	2216	64.96
12/4/2020 13:19	190	187	183	2220	65.04
12/4/2020 13:19	190	187.2	182.7	2220	64.92
12/4/2020 13:19	190	186.9	183	2220	64.92
12/4/2020 13:19	190	187	183.2	2220	64.96
12/4/2020 13:19	190	187.2	183.9	2223	64.88
12/4/2020 13:19	190	186.7	183.7	2218	64.83
12/4/2020 13:19	190	187.2	182.8	2221	65.08
12/4/2020 13:19	190	186.8	183	2224	64.92
12/4/2020 13:19	189.9	187	183.1	2223	64.92
12/4/2020 13:19	189.9	187.1	183.2	2224	65.08
12/4/2020 13:19	190	187.1	183.2	2226	64.99
12/4/2020 13:19	189.9	187	182.7	2225	65.04
12/4/2020 13:19	189.9	186.8	182.7	2230	64.84
12/4/2020 13:19	190	187	183	2225	65.04
12/4/2020 13:19	189.9	187.2	183.7	2227	64.95
12/4/2020 13:19	189.8	186.9	183.9	2228	65.05
12/4/2020 13:19	189.8	187.2	183.3	2227	64.87
12/4/2020 13:19	189.8	187	182.8	2226	64.96
12/4/2020 13:19	189.8	187	183	2230	65.04
12/4/2020 13:19	189.9	187.3	183.2	2227	64.92
12/4/2020 13:19	189.8	187.2	183.3	2229	65.04
12/4/2020 13:19	189.8	187.4	183.4	2229	65.04
12/4/2020 13:19	189.8	186.9	182.8	2224	64.96
12/4/2020 13:19	189.8	186.9	182.7	2225	65
12/4/2020 13:19	189.8	186.8	182.5	2224	65.09
12/4/2020 13:19	189.8	187.1	183	2222	64.96
12/4/2020 13:19	189.8	187	183.7	2224	64.92
12/4/2020 13:19	189.8	187	184	2224	64.96
12/4/2020 13:19	189.8	186.8	183.8	2225	64.92
12/4/2020 13:19	189.8	186.8	183.6	2227	65.08
12/4/2020 13:19	189.8	187.1	183.3	2228	64.96
12/4/2020 13:19	189.8	187.3	183.1	2225	64.83
12/4/2020 13:19	189.8	187.2	183	2224	65.08
12/4/2020 13:19	189.8	187.2	182.9	2225	64.92
12/4/2020 13:19	189.8	187	183.2	2225	64.84
12/4/2020 13:19	189.8	186.8	183.2	2224	64.83
12/4/2020 13:19	189.7	187	183	2223	64.92
12/4/2020 13:19	189.7	186.9	183.4	2221	64.92
12/4/2020 13:19	189.8	186.8	183.5	2229	64.99
12/4/2020 13:19	189.7	187	183.4	2225	64.88

12/4/2020 13:19	189.7	186.7	183.7	2224	64.88
12/4/2020 13:19	189.7	187.2	183	2223	64.84
12/4/2020 13:19	189.7	186.9	183.4	2218	65.08
12/4/2020 13:19	189.7	187.1	183.2	2217	64.92
12/4/2020 13:19	189.7	187.3	183.2	2217	64.83
12/4/2020 13:19	189.6	188	183.1	2219	65.03
12/4/2020 13:19	189.6	188.1	183.2	2221	64.92
12/4/2020 13:19	189.6	188.1	183.5	2223	64.88
12/4/2020 13:19	189.6	188.1	183.5	2225	64.92
12/4/2020 13:19	189.6	188.1	183	2227	64.93
12/4/2020 13:20	189.6	188	183.1	2228	64.96
12/4/2020 13:20	189.6	188.1	183	2228	64.92
12/4/2020 13:20	189.6	188.1	183.7	2224	65.08
12/4/2020 13:20	189.6	188.1	184.1	2227	64.99
12/4/2020 13:20	189.5	187.9	183.7	2227	64.96
12/4/2020 13:20	189.5	188	182.8	2229	65
12/4/2020 13:20	189.5	188.2	182.7	2232	64.88
12/4/2020 13:20	189.6	188.1	183.2	2230	64.96
12/4/2020 13:20	189.5	188.1	183.4	2231	65.04
12/4/2020 13:20	189.5	188.1	183.5	2228	64.96
12/4/2020 13:20	189.5	188.1	183	2231	65
12/4/2020 13:20	189.5	188.1	183.5	2224	65
12/4/2020 13:20	189.5	188.1	184.1	2228	64.92
12/4/2020 13:20	189.4	188	183.2	2223	64.92
12/4/2020 13:20	189.4	188.1	183	2225	65.13
12/4/2020 13:20	189.4	188	183.1	2224	64.87
12/4/2020 13:20	189.5	188.2	183.2	2223	64.92
12/4/2020 13:20	189.4	188	183.4	2227	65
12/4/2020 13:20	189.4	188.1	183	2225	64.92
12/4/2020 13:20	189.4	188	183.3	2228	64.96
12/4/2020 13:20	189.4	188.1	183.9	2227	65.04
12/4/2020 13:20	189.3	188.1	183.8	2218	64.96
12/4/2020 13:20	189.3	187.9	183.4	2214	64.91
12/4/2020 13:20	189.3	188	183	2212	65.04
12/4/2020 13:20	189.3	188.1	183.2	2216	64.96
12/4/2020 13:20	189.3	188.1	183.4	2225	64.83
12/4/2020 13:20	189.3	188.1	183.2	2230	65.12
12/4/2020 13:20	189.4	188.1	183.4	2227	64.96
12/4/2020 13:20	189.3	188.2	183.2	2231	64.99
12/4/2020 13:20	189.3	188.1	183.7	2234	64.92
12/4/2020 13:20	189.2	188.1	184.5	2232	64.88
12/4/2020 13:20	189.2	188.1	183.8	2232	64.92
12/4/2020 13:20	189.2	188.1	183.3	2232	64.92
12/4/2020 13:20	189.2	188.1	183	2229	64.88
12/4/2020 13:20	189.2	188.1	183.1	2232	65.03
12/4/2020 13:20	189.2	188.2	183.4	2232	64.96
12/4/2020 13:20	189.2	188.1	183.5	2232	65.08

12/4/2020 13:20	189.2	188.2	183.3	2233	64.96
12/4/2020 13:20	189.2	188.2	183.7	2234	65.12
12/4/2020 13:20	189.2	188.1	183.4	2234	64.96
12/4/2020 13:20	189.2	188	183.7	2231	64.99
12/4/2020 13:20	189.1	188.1	183.6	2235	64.88
12/4/2020 13:20	189.1	188.1	183.4	2231	65
12/4/2020 13:20	189.1	188.1	183.5	2233	64.95
12/4/2020 13:20	189.1	188.1	183.6	2231	64.83
12/4/2020 13:20	189.1	188	183.2	2232	65.04
12/4/2020 13:20	189.1	188.1	183.5	2227	64.99
12/4/2020 13:20	189.1	188.1	183.4	2228	64.96
12/4/2020 13:20	189	188.1	183.3	2231	65
12/4/2020 13:20	189.1	188.1	183.2	2235	65
12/4/2020 13:20	189	188	183.2	2235	64.92
12/4/2020 13:20	189	188.1	184	2235	65
12/4/2020 13:20	189	188.1	184.1	2232	65.04
12/4/2020 13:20	189	188.2	183	2232	64.96
12/4/2020 13:20	189.1	188.1	183.5	2235	65
12/4/2020 13:20	189	188	183.3	2228	64.96
12/4/2020 13:20	189	188	183.5	2227	64.96
12/4/2020 13:20	189	188.1	183.1	2231	65.04
12/4/2020 13:20	189	188.1	183.4	2225	64.92
12/4/2020 13:20	189	188.2	184.3	2230	64.95
12/4/2020 13:21	189	188.1	183.8	2232	65
12/4/2020 13:21	189	188.2	183	2230	64.96
12/4/2020 13:21	189	188.2	183.7	2229	65.04
12/4/2020 13:21	189	188.2	183.4	2232	65.04
12/4/2020 13:21	189	188	183.6	2230	65.04
12/4/2020 13:21	189	188.1	183.2	2231	64.88
12/4/2020 13:21	189	188.1	183	2235	65.12
12/4/2020 13:21	189	188.1	184.3	2232	64.88
12/4/2020 13:21	189	188.1	183.9	2230	64.88
12/4/2020 13:21	189	188.1	183.4	2228	65.12
12/4/2020 13:21	188.9	188.1	183.7	2231	64.99
12/4/2020 13:21	188.9	188	183.8	2236	65.08
12/4/2020 13:21	188.9	188.1	183.4	2231	64.92
12/4/2020 13:21	189	188.1	183.3	2231	64.8
12/4/2020 13:21	189	188.1	183.1	2231	64.92
12/4/2020 13:21	188.9	188.2	183.7	2234	64.96
12/4/2020 13:21	188.9	188.1	184.5	2230	64.88
12/4/2020 13:21	188.9	188.2	183.7	2225	64.99
12/4/2020 13:21	188.9	188.1	183.4	2228	65.09
12/4/2020 13:21	188.8	188.1	183.3	2228	64.99
12/4/2020 13:21	188.8	188	183.7	2230	65
12/4/2020 13:21	188.8	188.1	183.7	2229	64.96
12/4/2020 13:21	188.8	188	183.5	2230	64.96
12/4/2020 13:21	188.8	188	183.5	2232	64.96

12/4/2020 13:21	188.7	188.1	183.2	2231	64.92
12/4/2020 13:21	188.7	188.1	183.5	2232	64.96
12/4/2020 13:21	188.7	188	184.6	2231	64.92
12/4/2020 13:21	188.7	188.1	183.8	2232	65.05
12/4/2020 13:21	188.7	188.1	183.5	2232	64.92
12/4/2020 13:21	188.7	188.1	183.6	2231	64.92
12/4/2020 13:21	188.7	188.2	183.6	2235	65.09
12/4/2020 13:21	188.7	188	183.7	2232	64.96
12/4/2020 13:21	188.7	188	183.2	2229	65
12/4/2020 13:21	188.7	188.1	183	2228	65.08
12/4/2020 13:21	188.7	188	183.5	2229	64.87
12/4/2020 13:21	188.7	187.9	184.2	2227	65
12/4/2020 13:21	188.6	188.1	184	2230	64.92
12/4/2020 13:21	188.7	188.1	183.8	2228	65.04
12/4/2020 13:21	188.7	188.1	183.4	2225	64.92
12/4/2020 13:21	188.7	188.2	183.7	2230	65.08
12/4/2020 13:21	188.5	188	183.5	2231	64.96
12/4/2020 13:21	188.6	188.1	183.7	2227	65.04
12/4/2020 13:21	188.6	188.1	183.4	2228	65.01
12/4/2020 13:21	188.5	188.1	183.4	2226	64.88
12/4/2020 13:21	188.5	188	184.5	2227	65.04
12/4/2020 13:21	188.5	188.1	183.9	2227	65.04
12/4/2020 13:21	188.5	188.1	183.4	2228	64.88
12/4/2020 13:21	188.5	188.1	183.8	2232	64.84
12/4/2020 13:21	188.5	188.1	183.7	2233	64.92
12/4/2020 13:21	188.5	188.1	183.6	2235	65.08
12/4/2020 13:21	188.5	188	183.4	2232	64.99
12/4/2020 13:21	188.6	188	183	2233	65.04
12/4/2020 13:21	188.5	188.1	183.3	2233	65.04
12/4/2020 13:21	188.5	188.1	184.4	2233	64.92
12/4/2020 13:21	188.5	188.2	183.8	2232	64.88
12/4/2020 13:21	188.4	187.9	183.6	2233	65.09
12/4/2020 13:21	188.5	188	183.7	2232	64.88
12/4/2020 13:21	188.5	188.1	183.6	2236	65
12/4/2020 13:21	188.4	187.9	183.5	2240	65.08
12/4/2020 13:22	188.5	187.9	183.6	2236	64.92
12/4/2020 13:22	188.4	188.1	183.8	2240	64.96
12/4/2020 13:22	188.4	188.1	184.6	2239	65.12
12/4/2020 13:22	188.4	188	183.8	2239	64.92
12/4/2020 13:22	188.4	188.1	183.7	2234	64.96
12/4/2020 13:22	188.4	187.9	183.9	2232	65.04
12/4/2020 13:22	188.4	188.1	183.7	2230	64.96
12/4/2020 13:22	188.3	188	183.6	2229	64.91
12/4/2020 13:22	188.4	188	183.2	2230	65.13
12/4/2020 13:22	188.3	188	183.6	2229	64.91
12/4/2020 13:22	188.3	188.1	184.8	2227	64.92
12/4/2020 13:22	188.4	188.1	183.9	2233	65.05

12/4/2020 13:22	188.3	188	183.5	2230	64.92
12/4/2020 13:22	188.3	188.1	183.6	2231	65
12/4/2020 13:22	188.3	188.1	183.7	2236	65.08
12/4/2020 13:22	188.3	188	184	2235	64.83
12/4/2020 13:22	188.3	188	183.8	2236	65.04
12/4/2020 13:22	188.3	188.1	183.5	2236	65.12
12/4/2020 13:22	188.3	188.1	183.7	2238	65.03
12/4/2020 13:22	188.3	188.1	184.4	2235	64.99
12/4/2020 13:22	188.3	188.1	184.2	2236	65.01
12/4/2020 13:22	188.2	188.2	183.9	2236	65.04
12/4/2020 13:22	188.2	187.9	183.4	2235	64.96
12/4/2020 13:22	188.1	188	184	2236	64.96
12/4/2020 13:22	188.1	188	183.8	2236	64.93
12/4/2020 13:22	188.1	188	183.4	2232	65.08
12/4/2020 13:22	188	188.1	183.5	2235	65.08
12/4/2020 13:22	188.1	188.1	184.2	2234	64.88
12/4/2020 13:22	187.9	188.2	184.3	2230	64.84
12/4/2020 13:22	187.9	188.2	183.7	2231	65.04
12/4/2020 13:22	187.9	188.1	183.5	2232	64.96
12/4/2020 13:22	187.8	188.1	184.1	2232	64.92
12/4/2020 13:22	187.8	188.2	183.5	2237	65.04
12/4/2020 13:22	187.8	188.1	183.4	2237	65
12/4/2020 13:22	187.7	188.1	183.4	2236	65
12/4/2020 13:22	187.7	188.1	184.1	2239	64.92
12/4/2020 13:22	187.7	188.1	184.6	2239	65.04
12/4/2020 13:22	187.7	188.1	183.9	2238	64.91
12/4/2020 13:22	187.6	188.1	183.4	2238	64.96
12/4/2020 13:22	187.5	188.1	183.9	2236	64.96
12/4/2020 13:22	187.5	188.1	183.9	2236	64.92
12/4/2020 13:22	187.4	188.1	183.6	2232	64.96
12/4/2020 13:22	187.4	188.1	183.5	2232	64.96
12/4/2020 13:22	187.5	188	183.3	2230	64.92
12/4/2020 13:22	187.3	188	184.6	2231	64.96
12/4/2020 13:22	187.4	187.2	184.3	2225	65.04
12/4/2020 13:22	187.3	187.2	183.5	2224	64.91
12/4/2020 13:22	187.3	186.8	183.8	2227	65.04
12/4/2020 13:22	187.3	187	183.8	2227	65.08
12/4/2020 13:22	187.3	187.1	183.5	2229	64.88
12/4/2020 13:22	187.3	186.8	183.4	2235	64.88
12/4/2020 13:22	187.3	187	183.2	2231	65
12/4/2020 13:22	187.3	186.8	184.5	2230	64.88
12/4/2020 13:22	187.3	187.2	184.4	2236	64.92
12/4/2020 13:22	187.3	187.2	183.4	2240	65
12/4/2020 13:22	187.3	187.1	183.7	2237	64.99
12/4/2020 13:22	187.3	187.3	184.1	2232	65.04
12/4/2020 13:22	187.3	187	183.7	2232	65.12
12/4/2020 13:22	187.3	187	183.7	2231	64.99

12/4/2020 13:23	187.3	187.2	183.3	2229	64.92
12/4/2020 13:23	187.4	187	184.1	2230	64.96
12/4/2020 13:23	187.4	187	184.6	2232	65.03
12/4/2020 13:23	187.5	186.9	183.9	2227	65.03
12/4/2020 13:23	187.4	187.2	183.5	2227	65.04
12/4/2020 13:23	187.4	187	183.8	2229	64.83
12/4/2020 13:23	187.4	186.7	183.7	2228	64.92
12/4/2020 13:23	187.5	187.2	183.8	2228	65.08
12/4/2020 13:23	187.5	186.9	183.4	2228	65.04
12/4/2020 13:23	187.6	187	183.4	2227	64.88
12/4/2020 13:23	187.6	186.9	184.1	2227	65.09
12/4/2020 13:23	187.6	187	184.4	2228	64.96
12/4/2020 13:23	187.6	187.2	183.8	2228	64.96
12/4/2020 13:23	187.6	187	183.7	2233	65.04
12/4/2020 13:23	187.7	186.9	183.6	2232	64.96
12/4/2020 13:23	187.7	186.8	183.8	2230	64.92
12/4/2020 13:23	187.7	187	183.5	2232	65.09
12/4/2020 13:23	187.8	187.1	183.4	2232	65
12/4/2020 13:23	187.8	187.2	183.7	2222	65
12/4/2020 13:23	187.9	187.2	184.4	2232	65.08
12/4/2020 13:23	187.9	187	184.7	2228	65
12/4/2020 13:23	187.9	186.7	183.8	2227	64.92
12/4/2020 13:23	188	187	183.7	2231	65.05
12/4/2020 13:23	188.1	187.3	183.8	2228	64.91
12/4/2020 13:23	188	187.4	183.9	2227	65
12/4/2020 13:23	188.1	186.9	183.8	2227	65.04
12/4/2020 13:23	188.1	186.8	183.7	2228	64.92
12/4/2020 13:23	188.1	187	183.7	2231	65
12/4/2020 13:23	188.1	187.2	184.5	2228	64.88
12/4/2020 13:23	188.1	186.9	184.2	2225	65.04
12/4/2020 13:23	188.1	187.2	183.6	2224	64.92
12/4/2020 13:23	188.1	186.8	183.9	2225	64.96
12/4/2020 13:23	188.1	187	184.1	2228	64.84
12/4/2020 13:23	188.1	187.2	183.7	2224	64.99
12/4/2020 13:23	188.1	186.8	183.7	2227	65.04
12/4/2020 13:23	188.2	187.2	183.6	2227	64.84
12/4/2020 13:23	188.1	187	184.1	2228	64.92
12/4/2020 13:23	188.1	187	184.6	2225	65.12
12/4/2020 13:23	188.1	187.1	183.7	2224	64.99
12/4/2020 13:23	188.1	186.9	183.9	2225	64.88
12/4/2020 13:23	188.1	187	183.8	2225	65.04
12/4/2020 13:23	188.1	186.8	183.9	2226	64.96
12/4/2020 13:23	188.1	187.3	183.7	2225	64.88
12/4/2020 13:23	188.1	187	183.6	2225	65.03
12/4/2020 13:23	188.1	186.8	184.4	2224	64.92
12/4/2020 13:23	188.1	186.9	184.2	2225	65.03
12/4/2020 13:23	188.1	186.8	183.5	2221	65.04

12/4/2020 13:23	188.1	187.3	183.8	2224	64.96
12/4/2020 13:23	188.2	187.2	184	2224	64.96
12/4/2020 13:23	188.2	186.9	183.7	2224	64.92
12/4/2020 13:23	188.2	186.8	183.6	2227	65
12/4/2020 13:23	188.3	186.8	183.5	2224	64.92
12/4/2020 13:23	188.3	187.2	183.9	2227	64.99
12/4/2020 13:23	188.3	187.3	184.9	2228	65.08
12/4/2020 13:23	188.4	187.1	184.2	2226	64.96
12/4/2020 13:23	188.4	187	183.9	2228	64.96
12/4/2020 13:23	188.5	186.9	184.2	2229	65.04
12/4/2020 13:23	188.5	186.9	183.9	2227	64.92
12/4/2020 13:23	188.6	187.4	184.1	2225	65
12/4/2020 13:23	188.7	186.8	183.7	2224	65.04
12/4/2020 13:24	188.7	187.1	183.9	2224	64.96
12/4/2020 13:24	188.8	187.2	184.7	2225	64.92
12/4/2020 13:24	188.8	187.3	184.6	2226	65.05
12/4/2020 13:24	188.9	187.2	183.9	2224	64.88
12/4/2020 13:24	189	186.8	183.9	2221	65.04
12/4/2020 13:24	189	187.3	183.9	2219	65.09
12/4/2020 13:24	189	186.8	184.2	2220	64.84
12/4/2020 13:24	189.1	187	184.1	2217	65
12/4/2020 13:24	189.2	187.3	183.7	2218	65.04
12/4/2020 13:24	189.3	186.8	183.8	2215	64.99
12/4/2020 13:24	189.4	187	184.3	2216	64.84
12/4/2020 13:24	189.4	187.2	184.8	2220	65.01
12/4/2020 13:24	189.5	186.9	184.7	2219	64.88
12/4/2020 13:24	189.6	187.2	183.9	2221	64.96
12/4/2020 13:24	189.8	188.1	184.2	2224	65
12/4/2020 13:24	189.7	188.1	184.1	2221	64.99
12/4/2020 13:24	189.8	188.1	184.4	2224	64.88
12/4/2020 13:24	189.9	188.1	184.1	2223	64.93
12/4/2020 13:24	190	188.1	183.7	2225	64.88
12/4/2020 13:24	190.1	188.1	184.6	2223	64.99
12/4/2020 13:24	190.3	188.1	184.8	2221	64.92
12/4/2020 13:24	190.3	187.9	184.1	2224	64.84
12/4/2020 13:24	190.5	188.1	184.1	2220	65.04
12/4/2020 13:24	190.6	188.1	184.6	2221	64.88
12/4/2020 13:24	190.7	188.1	184.4	2220	65.04
12/4/2020 13:24	190.7	188.2	184.5	2217	64.88
12/4/2020 13:24	190.9	188.2	184	2219	65.04
12/4/2020 13:24	190.9	188	184	2221	64.92
12/4/2020 13:24	191	188	184.2	2223	64.99
12/4/2020 13:24	191.1	188.1	184.8	2228	64.88
12/4/2020 13:24	191.2	188.1	185.2	2226	65.04
12/4/2020 13:24	191.3	188	185.3	2225	64.88
12/4/2020 13:24	191.3	188.1	185	2232	64.88
12/4/2020 13:24	191.4	188.1	184.7	2232	65.05

12/4/2020 13:24	191.6	188.1	184.2	2232	64.92
12/4/2020 13:24	191.6	188.1	183.9	2232	64.96
12/4/2020 13:24	191.6	188.1	184.2	2232	64.96
12/4/2020 13:24	191.8	188	184.2	2229	64.96
12/4/2020 13:24	191.8	188.2	184.5	2232	64.96
12/4/2020 13:24	191.9	188.1	184.6	2231	65.05
12/4/2020 13:24	192	188.1	184.6	2228	65
12/4/2020 13:24	192	188.1	184.6	2227	64.92
12/4/2020 13:24	192.1	188.1	184.6	2230	65.09
12/4/2020 13:24	192.2	188	184.8	2224	65
12/4/2020 13:24	192.2	188	184.7	2225	65.04
12/4/2020 13:24	192.3	188	184.6	2225	64.96
12/4/2020 13:24	192.3	188.1	184.8	2226	64.96
12/4/2020 13:24	192.3	188.1	184.8	2225	65
12/4/2020 13:24	192.4	188.1	184.6	2229	65
12/4/2020 13:24	192.4	188	184.6	2231	65.08
12/4/2020 13:24	192.4	188.1	184.6	2232	65.04
12/4/2020 13:24	192.5	187.9	184.6	2232	65.08
12/4/2020 13:24	192.6	188	185	2230	65.08
12/4/2020 13:24	192.6	188	185.3	2228	64.96
12/4/2020 13:24	192.7	188.4	185.4	2234	65.05
12/4/2020 13:24	192.7	188.1	185.4	2233	65.04
12/4/2020 13:24	192.8	188.1	184.8	2231	65.08
12/4/2020 13:24	192.9	188.1	184.5	2235	65.17
12/4/2020 13:24	192.8	188	184.6	2234	65
12/4/2020 13:25	192.9	187.9	184.3	2236	64.96
12/4/2020 13:25	193	188.1	184.8	2236	64.96
12/4/2020 13:25	193	188.1	184.9	2238	64.92
12/4/2020 13:25	193	188.1	185.1	2239	65.04
12/4/2020 13:25	193.1	188.2	184.9	2240	65
12/4/2020 13:25	193.2	188.1	184.9	2236	64.96
12/4/2020 13:25	193.1	188.1	185	2232	64.96
12/4/2020 13:25	193.2	188.1	185.1	2236	65
12/4/2020 13:25	193.3	188.1	184.7	2233	65.08
12/4/2020 13:25	193.3	188.1	184.9	2232	65.04
12/4/2020 13:25	193.3	188.1	184.6	2233	65
12/4/2020 13:25	193.3	188	184.9	2232	64.92
12/4/2020 13:25	193.4	188.1	184.8	2231	64.99
12/4/2020 13:25	193.4	188.1	184.6	2227	64.93
12/4/2020 13:25	193.5	188	185.3	2232	64.92
12/4/2020 13:25	193.5	188.1	184.9	2234	64.92
12/4/2020 13:25	193.5	188	185	2233	65
12/4/2020 13:25	193.5	187.9	185.1	2235	65.04
12/4/2020 13:25	193.5	189.1	185.1	2234	64.92
12/4/2020 13:25	193.6	189.1	185	2238	64.99
12/4/2020 13:25	193.7	189.1	185	2239	65
12/4/2020 13:25	193.7	188.9	184.7	2239	65

12/4/2020 13:25	193.6	188.8	184.9	2238	65.04
12/4/2020 13:25	193.7	188.9	185.2	2234	64.96
12/4/2020 13:25	193.8	188.7	185.2	2232	64.96
12/4/2020 13:25	193.7	189.1	185.4	2236	64.96
12/4/2020 13:25	193.8	188.9	185.1	2239	65
12/4/2020 13:25	193.8	188.9	185.3	2236	64.92
12/4/2020 13:25	193.9	189	185.4	2239	64.96
12/4/2020 13:25	193.9	189.1	185.3	2236	65.12
12/4/2020 13:25	193.9	189.2	185.1	2238	64.88
12/4/2020 13:25	194	188.9	184.8	2239	64.92
12/4/2020 13:25	194	188.6	184.8	2241	64.96
12/4/2020 13:25	193.9	189	185.6	2236	64.92
12/4/2020 13:25	194	189.3	186.2	2233	65.04
12/4/2020 13:25	194.1	189.2	186	2234	65.01
12/4/2020 13:25	194.1	189.1	185.4	2237	64.99
12/4/2020 13:25	194.1	188.9	185.1	2237	65.08
12/4/2020 13:25	194.1	188.8	185.1	2239	65.12
12/4/2020 13:25	194.1	189.1	185.6	2239	64.83
12/4/2020 13:25	194.2	189.3	185.4	2242	64.88
12/4/2020 13:25	194.2	189.1	185.1	2243	65.08
12/4/2020 13:25	194.2	188.8	185.2	2241	64.95
12/4/2020 13:25	194.2	188.8	185.6	2242	65.08
12/4/2020 13:25	194.2	189.3	186	2244	65.12
12/4/2020 13:25	194.3	189.2	185.4	2243	64.99
12/4/2020 13:25	194.4	188.9	185.5	2240	64.92
12/4/2020 13:25	194.4	188.9	185.6	2241	65.09
12/4/2020 13:25	194.3	188.8	185.5	2241	64.83
12/4/2020 13:25	194.4	188.8	185.5	2245	65
12/4/2020 13:25	194.4	188.8	185.2	2243	65.05
12/4/2020 13:25	194.4	189.1	185	2242	64.92
12/4/2020 13:25	194.5	189.1	185.3	2241	65
12/4/2020 13:25	194.4	188.9	186	2243	65
12/4/2020 13:25	194.4	189.1	186.2	2245	65
12/4/2020 13:25	194.5	188.6	186.1	2243	65
12/4/2020 13:25	194.4	189.1	185.4	2245	65.08
12/4/2020 13:25	194.5	188.9	185.3	2245	64.96
12/4/2020 13:25	194.6	188.8	185.6	2241	64.92
12/4/2020 13:25	194.6	189.3	185.6	2242	65.12
12/4/2020 13:26	194.5	188.8	185.8	2239	65.04
12/4/2020 13:26	194.6	188.8	185.8	2243	64.93
12/4/2020 13:26	194.6	188.7	185.6	2242	65.08
12/4/2020 13:26	194.6	189.2	185.5	2243	64.92
12/4/2020 13:26	194.6	188.9	185.6	2244	64.92
12/4/2020 13:26	194.7	189.1	185.5	2243	65.04
12/4/2020 13:26	194.7	188.8	185.5	2242	64.84
12/4/2020 13:26	194.6	188.9	185.6	2246	64.92
12/4/2020 13:26	194.6	189.1	185.7	2250	64.96

12/4/2020 13:26	194.6	188.8	185.5	2247	65.08
12/4/2020 13:26	194.7	189.1	185.9	2245	65.04
12/4/2020 13:26	194.7	189.1	185.7	2245	65
12/4/2020 13:26	194.7	188.8	185.6	2246	65.04
12/4/2020 13:26	194.7	189.1	185.8	2241	64.96
12/4/2020 13:26	194.8	188.7	185.4	2239	65
12/4/2020 13:26	194.7	190.2	186	2238	65
12/4/2020 13:26	194.7	190.2	186.5	2237	65
12/4/2020 13:26	194.7	190	186	2239	64.96
12/4/2020 13:26	194.7	190	185.4	2234	64.99
12/4/2020 13:26	194.8	189.4	185.7	2237	65.04
12/4/2020 13:26	194.9	189.5	185.8	2240	65
12/4/2020 13:26	194.8	190.3	185.8	2239	64.92
12/4/2020 13:26	194.8	189.7	185.7	2242	64.88
12/4/2020 13:26	194.8	190.3	186.5	2242	65.12
12/4/2020 13:26	194.8	190.1	186.1	2244	64.84
12/4/2020 13:26	194.8	190	185.5	2241	64.88
12/4/2020 13:26	194.9	190.3	185.8	2246	64.96
12/4/2020 13:26	194.8	189.8	185.8	2243	64.88
12/4/2020 13:26	194.9	190.1	185.7	2241	64.84
12/4/2020 13:26	194.9	190.1	185.8	2244	65.08
12/4/2020 13:26	194.9	189.6	186.2	2244	64.99
12/4/2020 13:26	195	190.1	186.8	2247	64.96
12/4/2020 13:26	194.9	189.8	186	2250	64.96
12/4/2020 13:26	194.9	190.3	185.6	2246	64.99
12/4/2020 13:26	194.9	190.2	185.6	2246	64.92
12/4/2020 13:26	194.9	190.3	185.8	2249	65.09
12/4/2020 13:26	194.9	190.2	185.9	2245	64.88
12/4/2020 13:26	194.9	189.9	185.8	2242	64.96
12/4/2020 13:26	194.9	190	186	2245	65.05
12/4/2020 13:26	195	190.2	185.8	2243	65
12/4/2020 13:26	195	189.6	186.2	2244	65
12/4/2020 13:26	194.9	190.4	185.9	2250	65.04
12/4/2020 13:26	194.9	189.7	186	2249	64.96
12/4/2020 13:26	194.9	189.9	186.1	2243	65.04
12/4/2020 13:26	195	189.7	185.8	2245	65.12
12/4/2020 13:26	195	189.8	186.1	2243	64.99
12/4/2020 13:26	195	190.3	185.8	2242	64.92
12/4/2020 13:26	195	189.6	186.1	2243	65.12
12/4/2020 13:26	195	189.7	186.1	2243	64.96
12/4/2020 13:26	195	189.8	186	2245	64.96
12/4/2020 13:26	195.1	190.1	186.2	2246	65.08
12/4/2020 13:26	195	190.2	186.2	2246	65.08
12/4/2020 13:26	195	190	186.1	2243	64.88
12/4/2020 13:26	195	190.3	186.1	2247	65.04
12/4/2020 13:26	195	190.3	186	2250	64.96
12/4/2020 13:26	195	189.5	185.8	2250	64.95

12/4/2020 13:26	195	190.5	186.1	2252	65
12/4/2020 13:26	195.1	189.8	186.6	2254	65
12/4/2020 13:27	195	189.7	186.8	2255	64.96
12/4/2020 13:27	195.1	189.5	185.8	2253	65.04
12/4/2020 13:27	195.1	190.1	186	2253	64.96
12/4/2020 13:27	195.2	190.1	186.2	2253	64.96
12/4/2020 13:27	195.1	190	186.1	2253	65
12/4/2020 13:27	195	189.8	185.8	2250	65.05
12/4/2020 13:27	195.2	189.7	185.8	2250	64.96
12/4/2020 13:27	195.1	189.6	186.3	2247	65.04
12/4/2020 13:27	195.1	190.3	186.9	2246	65
12/4/2020 13:27	195.1	190.1	186.5	2241	65
12/4/2020 13:27	195.2	190.3	186	2243	64.99
12/4/2020 13:27	195.2	190.4	186.2	2247	65.12
12/4/2020 13:27	195.2	190	186.3	2245	64.84
12/4/2020 13:27	195.2	190.2	186.1	2246	65
12/4/2020 13:27	195.1	189.9	186.1	2245	64.88
12/4/2020 13:27	195.2	190.3	186.3	2245	65.08
12/4/2020 13:27	195.2	190.3	187	2248	65.04
12/4/2020 13:27	195.3	190	186.5	2248	64.96
12/4/2020 13:27	195.3	190.2	186.1	2251	64.99
12/4/2020 13:27	195.2	189.5	186.3	2247	65.08
12/4/2020 13:27	195.2	191.2	186.1	2247	64.96
12/4/2020 13:27	195.2	191.1	186.3	2246	64.96
12/4/2020 13:27	195.2	190.8	186.1	2250	65
12/4/2020 13:27	195.2	191	186	2250	65
12/4/2020 13:27	195.2	190.8	187	2247	64.96
12/4/2020 13:27	195.2	191.2	186.7	2246	64.96
12/4/2020 13:27	195.2	191.4	186.1	2248	65
12/4/2020 13:27	195.3	190.8	186.3	2249	64.96
12/4/2020 13:27	195.2	191.2	186.6	2251	64.96
12/4/2020 13:27	195.3	190.7	186.4	2254	65.04
12/4/2020 13:27	195.2	190.7	186.1	2250	64.91
12/4/2020 13:27	195.3	191.4	186.3	2252	65
12/4/2020 13:27	195.3	190.7	187	2253	65
12/4/2020 13:27	195.2	191.2	186.6	2252	64.92
12/4/2020 13:27	195.3	191	186.1	2250	64.88
12/4/2020 13:27	195.2	190.8	186.5	2250	65.16
12/4/2020 13:27	195.2	191.4	186.5	2248	64.88
12/4/2020 13:27	195.2	191	186.1	2248	64.93
12/4/2020 13:27	195.3	191.2	186.3	2253	65.01
12/4/2020 13:27	195.2	191.3	186.9	2254	65.04
12/4/2020 13:27	195.2	190.6	187	2256	64.92
12/4/2020 13:27	195.2	191.2	186.5	2258	65.16
12/4/2020 13:27	195.2	191	186.3	2257	65.08
12/4/2020 13:27	195.2	191.4	186.5	2256	64.92
12/4/2020 13:27	195.1	191.1	186.7	2256	64.96

12/4/2020 13:27	195.1	190.8	186.3	2252	64.99
12/4/2020 13:27	195.2	191.2	186.2	2252	65.04
12/4/2020 13:27	195.2	191	186.3	2252	65
12/4/2020 13:27	195.2	191.2	187	2253	64.88
12/4/2020 13:27	195.1	191.4	187	2253	65
12/4/2020 13:27	195	190.7	186.2	2252	65.09
12/4/2020 13:27	195	191.2	186.7	2246	64.92
12/4/2020 13:27	195	190.9	186.7	2245	64.92
12/4/2020 13:27	195	190.8	186.5	2247	65.04
12/4/2020 13:27	195	191.5	186.5	2246	64.92
12/4/2020 13:27	194.9	191.3	186.2	2247	64.96
12/4/2020 13:27	194.9	191.4	186.8	2249	65.04
12/4/2020 13:27	194.9	190.7	187.2	2249	64.91
12/4/2020 13:27	194.9	190.9	186.5	2247	65
12/4/2020 13:28	194.8	190.8	186.5	2249	64.96
12/4/2020 13:28	194.8	191	186.7	2248	64.92
12/4/2020 13:28	194.7	191.3	186.4	2246	64.96
12/4/2020 13:28	194.7	190.7	186.3	2252	65.12
12/4/2020 13:28	194.6	191	186.3	2256	64.96
12/4/2020 13:28	194.6	191.4	186.5	2254	64.96
12/4/2020 13:28	194.6	190.7	187.1	2252	64.92
12/4/2020 13:28	194.6	191.5	186.8	2254	65.08
12/4/2020 13:28	194.6	190.8	186.5	2246	64.88
12/4/2020 13:28	194.5	191	186.5	2245	64.92
12/4/2020 13:28	194.5	191.4	186.5	2250	64.88
12/4/2020 13:28	194.4	191	186.8	2252	65.08
12/4/2020 13:28	194.4	191.4	186.6	2253	65.04
12/4/2020 13:28	194.4	191.4	186.2	2255	65.12
12/4/2020 13:28	194.3	191.4	186.3	2256	65.04
12/4/2020 13:28	194.3	190.9	186.3	2254	65.08
12/4/2020 13:28	194.3	190.7	187.3	2256	64.96
12/4/2020 13:28	194.2	190.9	187.2	2256	65
12/4/2020 13:28	194.2	190.7	186.6	2254	64.96
12/4/2020 13:28	194.2	191.4	186.4	2253	65
12/4/2020 13:28	194.2	191.2	186.6	2250	64.96
12/4/2020 13:28	194.1	190.6	186.6	2249	64.96
12/4/2020 13:28	194.1	191.2	186.8	2253	65.09
12/4/2020 13:28	194	191.4	186.7	2253	64.99
12/4/2020 13:28	194.1	190.6	186.2	2254	65
12/4/2020 13:28	193.9	191.5	186.5	2253	65
12/4/2020 13:28	193.8	191	186.7	2253	64.99
12/4/2020 13:28	193.9	191.1	187.4	2251	64.92
12/4/2020 13:28	193.8	190.6	187	2258	65.08
12/4/2020 13:28	193.7	191.2	186.6	2259	64.92
12/4/2020 13:28	193.7	191.5	186.7	2257	64.99
12/4/2020 13:28	193.7	190.8	186.8	2258	65.09
12/4/2020 13:28	193.5	191.4	186.9	2256	64.88

12/4/2020 13:28	193.6	191.4	186.8	2256	64.99
12/4/2020 13:28	193.5	190.7	186.7	2261	64.96
12/4/2020 13:28	193.4	191.2	186.4	2257	64.91
12/4/2020 13:28	193.5	190.9	186.2	2254	64.92
12/4/2020 13:28	193.4	191.4	186.5	2260	65.08
12/4/2020 13:28	193.3	191.3	187.2	2255	65.03
12/4/2020 13:28	193.3	190.7	187.4	2254	64.96
12/4/2020 13:28	193.3	191.5	187	2259	65
12/4/2020 13:28	193.2	190.7	186.6	2259	64.92
12/4/2020 13:28	193.1	191.4	186.3	2255	64.92
12/4/2020 13:28	193.1	191.4	186.6	2253	65.13
12/4/2020 13:28	193.1	190.7	187	2248	64.96
12/4/2020 13:28	193	191.2	186.7	2254	65.04
12/4/2020 13:28	193	190.8	186.8	2255	64.96
12/4/2020 13:28	192.9	191.5	186.4	2253	64.92
12/4/2020 13:28	192.9	191.4	186.3	2250	64.96
12/4/2020 13:28	192.9	190.8	186.3	2254	65.04
12/4/2020 13:28	192.9	191.3	186.3	2253	65
12/4/2020 13:28	192.8	190.7	186.8	2253	64.92
12/4/2020 13:28	192.8	191.2	187.2	2256	65.04
12/4/2020 13:28	192.6	191.2	187.4	2253	65
12/4/2020 13:28	192.6	191	187	2255	64.92
12/4/2020 13:28	192.6	191.1	186.6	2257	65.04
12/4/2020 13:28	192.5	190.9	186.4	2261	65.04
12/4/2020 13:28	192.4	190.6	186.5	2261	64.92
12/4/2020 13:28	192.4	190.8	186.6	2263	64.92
12/4/2020 13:28	192.4	190.9	186.8	2261	64.88
12/4/2020 13:29	192.4	191.4	186.8	2265	64.99
12/4/2020 13:29	192.3	191.3	186.8	2260	64.84
12/4/2020 13:29	192.2	190.9	186.8	2257	64.92
12/4/2020 13:29	192.2	191.4	186.8	2255	64.88
12/4/2020 13:29	192.2	190.7	186.6	2253	64.92
12/4/2020 13:29	192.1	191.2	186.9	2253	65.04
12/4/2020 13:29	192	190.7	186.8	2252	64.99
12/4/2020 13:29	192	190.8	186.6	2253	64.92
12/4/2020 13:29	191.9	191.4	186.9	2254	65.08
12/4/2020 13:29	191.8	190.9	186.7	2254	64.95
12/4/2020 13:29	191.8	191.2	186.7	2253	64.84
12/4/2020 13:29	191.8	190.7	186.5	2256	64.88
12/4/2020 13:29	191.7	191.2	186.7	2254	65.04
12/4/2020 13:29	191.7	191.4	187.2	2253	65.04
12/4/2020 13:29	191.6	190.8	187.5	2253	64.96
12/4/2020 13:29	191.5	191.3	186.7	2253	64.88
12/4/2020 13:29	191.5	190.9	186.3	2257	65.08
12/4/2020 13:29	191.4	190.7	186.3	2258	65.08
12/4/2020 13:29	191.4	191.4	186.7	2254	65.04
12/4/2020 13:29	191.3	190.9	186.7	2255	64.92

12/4/2020 13:29	191.2	191.5	186.3	2253	64.92
12/4/2020 13:29	191.2	191.4	186.8	2253	64.96
12/4/2020 13:29	191.1	190.6	187.2	2254	64.96
12/4/2020 13:29	191.1	191	186.4	2256	65
12/4/2020 13:29	191	190.8	186.4	2253	64.87
12/4/2020 13:29	191	191	186.5	2252	64.92
12/4/2020 13:29	190.9	191.4	186.5	2254	65.04
12/4/2020 13:29	190.9	190.6	186.5	2254	64.92
12/4/2020 13:29	190.9	191	186.6	2253	65
12/4/2020 13:29	190.7	190.7	187.4	2254	65
12/4/2020 13:29	190.7	191.4	186.7	2253	64.88
12/4/2020 13:29	190.7	191.1	186.3	2252	64.96
12/4/2020 13:29	190.6	190.6	186.5	2250	65
12/4/2020 13:29	190.5	191	186.5	2248	64.92
12/4/2020 13:29	190.5	190.8	186.6	2253	65
12/4/2020 13:29	190.3	191.4	186.4	2254	65.01
12/4/2020 13:29	190.3	191.5	187	2254	64.96
12/4/2020 13:29	190.3	190.8	187	2252	64.96
12/4/2020 13:29	190.3	191	186.2	2251	65
12/4/2020 13:29	190.2	191	186.2	2251	64.95
12/4/2020 13:29	190.1	191	186.4	2250	65.04
12/4/2020 13:29	190.1	191.5	186.7	2255	64.96
12/4/2020 13:29	190	191.2	186.7	2255	64.99
12/4/2020 13:29	189.9	190.9	186.9	2252	64.88
12/4/2020 13:29	189.8	190.8	186.6	2253	65.08
12/4/2020 13:29	189.8	190.7	186.6	2254	64.91
12/4/2020 13:29	189.7	191	186.4	2252	64.92
12/4/2020 13:29	189.7	190.3	186.1	2253	64.88
12/4/2020 13:29	189.7	191.2	186.3	2252	64.99
12/4/2020 13:29	189.6	190.9	186.2	2248	65.04
12/4/2020 13:29	189.6	190.8	186.2	2250	64.92
12/4/2020 13:29	189.6	190.7	186.5	2249	64.96
12/4/2020 13:29	189.5	191	186.6	2250	64.88
12/4/2020 13:29	189.4	191.3	187.1	2249	65
12/4/2020 13:29	189.4	191.2	187.2	2250	64.92
12/4/2020 13:29	189.4	191.4	187	2248	65.04
12/4/2020 13:29	189.4	190.9	186.8	2253	65.08
12/4/2020 13:29	189.2	190.7	186.3	2253	64.88
12/4/2020 13:29	189.1	191	186.1	2251	64.88
12/4/2020 13:30	189.2	191.2	186.3	2253	64.96
12/4/2020 13:30	189.1	191.5	186.1	2252	64.91
12/4/2020 13:30	189.2	191.4	186.2	2254	64.92
12/4/2020 13:30	189.1	191	186.2	2258	65.08
12/4/2020 13:30	189.1	191.1	186.4	2257	64.92
12/4/2020 13:30	189.1	191.4	186.4	2254	64.96
12/4/2020 13:30	189.1	190.7	186.4	2254	64.88
12/4/2020 13:30	189.1	191.5	186.3	2252	64.96

12/4/2020 13:30	189	190.8	186.4	2250	64.92
12/4/2020 13:30	189	191.1	186.2	2253	65
12/4/2020 13:30	189.1	191.2	186.5	2254	64.92
12/4/2020 13:30	189.1	190.7	186.9	2253	65
12/4/2020 13:30	189	191.4	187	2254	64.96
12/4/2020 13:30	189.1	189.6	187.2	2253	64.96
12/4/2020 13:30	189	190.1	187.3	2249	64.92
12/4/2020 13:30	189	190	187.2	2253	64.96
12/4/2020 13:30	189	190.3	187	2256	64.96
12/4/2020 13:30	189.1	190.3	187	2257	64.96
12/4/2020 13:30	189.1	189.7	187	2260	65.04
12/4/2020 13:30	189.1	190.1	186.9	2262	65
12/4/2020 13:30	189.1	189.7	187	2254	65.04
12/4/2020 13:30	189.2	189.8	187.1	2254	65.04
12/4/2020 13:30	189.2	190.3	187.1	2255	65.04
12/4/2020 13:30	189.2	189.6	187	2253	65.04
12/4/2020 13:30	189.3	190.1	187.2	2250	64.92
12/4/2020 13:30	189.3	189.7	187	2252	64.96
12/4/2020 13:30	189.2	189.9	187	2251	64.99
12/4/2020 13:30	189.3	190.3	187.2	2250	64.99
12/4/2020 13:30	189.3	189.6	186.7	2259	65
12/4/2020 13:30	189.3	190.1	186.5	2255	65.04
12/4/2020 13:30	189.3	190	186.3	2259	64.99
12/4/2020 13:30	189.4	189.8	186.2	2257	64.96
12/4/2020 13:30	189.4	189.6	186.3	2252	64.88
12/4/2020 13:30	189.4	189.8	186.2	2253	64.99
12/4/2020 13:30	189.5	190.2	186.3	2253	64.96
12/4/2020 13:30	189.4	190.2	186.7	2253	65.03
12/4/2020 13:30	189.5	189.8	186.3	2252	65.04
12/4/2020 13:30	189.6	190.3	186.4	2252	65.08
12/4/2020 13:30	189.5	190	186.2	2253	64.88
12/4/2020 13:30	189.6	190.5	186.2	2247	65.08
12/4/2020 13:30	189.6	190.1	186.2	2247	65.12
12/4/2020 13:30	189.6	189.7	186.2	2249	64.95
12/4/2020 13:30	189.7	190.3	186.3	2246	64.96
12/4/2020 13:30	189.7	189.8	186.9	2246	64.92
12/4/2020 13:30	189.7	190.2	187	2246	65.03
12/4/2020 13:30	189.8	190.3	187	2244	65.04
12/4/2020 13:30	189.8	190.3	186.8	2246	64.96
12/4/2020 13:30	189.8	190.4	186.5	2247	64.88
12/4/2020 13:30	189.9	189.8	186.5	2248	65.04
12/4/2020 13:30	189.8	190.1	186.2	2252	65.08
12/4/2020 13:30	190	190.3	186.1	2258	64.83
12/4/2020 13:30	190	190.3	186.2	2257	65.04
12/4/2020 13:30	190	190.3	186.3	2259	64.92
12/4/2020 13:30	190	189.6	186.5	2256	65.04
12/4/2020 13:30	190.1	189.8	186.5	2259	64.99

12/4/2020 13:30	190.2	189.6	186.8	2256	65.04
12/4/2020 13:30	190.2	190.3	186.7	2258	64.99
12/4/2020 13:30	190.2	190.3	186.5	2259	64.88
12/4/2020 13:30	190.3	189.8	186.5	2261	64.99
12/4/2020 13:30	190.4	190.3	186.4	2259	64.96
12/4/2020 13:31	190.3	190.3	186.4	2257	64.96
12/4/2020 13:31	190.4	190.3	186.5	2255	64.99
12/4/2020 13:31	190.4	190.3	186.3	2257	65
12/4/2020 13:31	190.4	190.1	186.2	2255	64.92
12/4/2020 13:31	190.4	190.3	186.3	2256	64.91
12/4/2020 13:31	190.5	190.2	186.1	2253	65
12/4/2020 13:31	190.5	189.6	186.2	2254	64.91
12/4/2020 13:31	190.5	190.2	186.4	2252	64.96
12/4/2020 13:31	190.5	189.7	186.1	2253	64.96
12/4/2020 13:31	190.6	190.2	186.4	2250	64.92
12/4/2020 13:31	190.5	190.3	186.4	2250	64.96
12/4/2020 13:31	190.5	190.3	186.5	2248	65
12/4/2020 13:31	190.5	190.1	186.4	2248	64.96
12/4/2020 13:31	190.6	189.8	186.2	2249	64.91
12/4/2020 13:31	190.5	189.8	186.3	2249	65.05
12/4/2020 13:31	190.5	190.2	186.3	2247	64.87
12/4/2020 13:31	190.5	190.2	186.4	2252	65
12/4/2020 13:31	190.5	190.3	186.2	2250	65.04
12/4/2020 13:31	190.5	190.1	186.4	2250	64.88
12/4/2020 13:31	190.5	190	186.5	2253	64.92
12/4/2020 13:31	190.5	190.3	186.7	2255	65.04
12/4/2020 13:31	190.5	189.5	186.6	2255	64.92
12/4/2020 13:31	190.5	190.4	186.4	2253	64.92
12/4/2020 13:31	190.5	190	186.5	2255	64.99
12/4/2020 13:31	190.4	190.5	187	2259	64.92
12/4/2020 13:31	190.3	189.6	187.1	2256	64.95
12/4/2020 13:31	190.3	190.3	186.2	2259	64.92
12/4/2020 13:31	190.3	190.3	186.5	2259	64.92
12/4/2020 13:31	190.3	190.1	186.7	2259	64.92
12/4/2020 13:31	190.2	189.8	186.6	2258	65.16
12/4/2020 13:31	190.1	190.3	186.5	2256	64.99
12/4/2020 13:31	190.1	189.8	187.2	2254	65.03
12/4/2020 13:31	190	190	186.3	2254	65.04
12/4/2020 13:31	190	190.1	186.2	2250	64.88
12/4/2020 13:31	189.9	190.4	186.5	2252	64.92
12/4/2020 13:31	190	189.9	187.4	2255	65.04
12/4/2020 13:31	190	189.6	186.7	2249	64.95
12/4/2020 13:31	189.9	190	186.3	2246	65.03
12/4/2020 13:31	189.8	190.3	186.6	2246	65.08
12/4/2020 13:31	189.8	190.5	187.2	2246	64.83
12/4/2020 13:31	189.8	190	186.8	2246	64.88
12/4/2020 13:31	189.8	189.6	186.3	2249	65.04

12/4/2020 13:31	189.8	190.1	186.3	2248	64.88
12/4/2020 13:31	189.8	190.2	186.1	2249	64.99
12/4/2020 13:31	189.8	189.8	186.6	2242	65.04
12/4/2020 13:31	189.8	190.4	186.7	2240	64.83
12/4/2020 13:31	189.8	189.7	186.6	2239	64.88
12/4/2020 13:31	189.8	190.3	186.6	2250	65.04
12/4/2020 13:31	189.8	190.2	186.5	2249	64.96
12/4/2020 13:31	189.8	189.8	186.5	2244	64.96
12/4/2020 13:31	189.7	190.3	186.4	2246	65
12/4/2020 13:31	189.8	189.9	186.3	2239	65
12/4/2020 13:31	189.8	190.3	186.5	2241	64.96
12/4/2020 13:31	189.8	189.6	186.5	2237	64.96
12/4/2020 13:31	189.8	189.6	186.9	2241	64.87
12/4/2020 13:31	189.9	190.3	187.2	2245	65.04
12/4/2020 13:31	190	189.8	187.3	2246	65
12/4/2020 13:31	189.8	190	187.2	2244	64.92
12/4/2020 13:31	190	190	187.3	2246	64.96
12/4/2020 13:32	190	189.6	187.1	2250	64.96
12/4/2020 13:32	190.1	190	187.1	2244	64.96
12/4/2020 13:32	190.1	189.7	186.9	2248	64.92
12/4/2020 13:32	190.1	190.2	186.9	2249	65
12/4/2020 13:32	190.1	190.2	187	2250	65
12/4/2020 13:32	190.2	189.7	187	2250	65.04
12/4/2020 13:32	190.3	190.3	187	2246	64.92
12/4/2020 13:32	190.4	189.8	187.5	2245	65
12/4/2020 13:32	190.3	190.3	187.2	2241	64.88
12/4/2020 13:32	190.5	189.7	187.5	2245	64.88
12/4/2020 13:32	190.5	189.8	187.1	2246	64.96
12/4/2020 13:32	190.6	190.4	186.8	2243	65.04
12/4/2020 13:32	190.7	190	186.5	2243	64.99
12/4/2020 13:32	190.7	190	186.5	2244	65.08
12/4/2020 13:32	190.8	190	186.8	2241	64.88
12/4/2020 13:32	190.7	189.7	186.7	2245	65.03
12/4/2020 13:32	190.9	190	186.4	2247	64.96
12/4/2020 13:32	190.9	189.9	186.5	2248	65.08
12/4/2020 13:32	190.9	190.4	187.2	2247	65.03
12/4/2020 13:32	191	190.3	186.8	2250	65.08
12/4/2020 13:32	191.1	190.3	186.4	2248	65.03
12/4/2020 13:32	191.1	190.3	186.6	2244	64.92
12/4/2020 13:32	191.2	189.7	186.8	2245	64.96
12/4/2020 13:32	191.1	189.9	186.9	2242	64.87
12/4/2020 13:32	191.3	189.6	186.6	2245	64.92
12/4/2020 13:32	191.4	190.2	186.6	2241	65.08
12/4/2020 13:32	191.5	190.3	187.4	2246	64.88
12/4/2020 13:32	191.5	189.6	187.2	2246	65.04
12/4/2020 13:32	191.6	190	186.7	2247	65.12
12/4/2020 13:32	191.7	189.6	186.8	2245	65

12/4/2020 13:32	191.8	190.2	186.9	2240	65.04
12/4/2020 13:32	191.8	190.3	187	2244	65.04
12/4/2020 13:32	191.8	190	186.5	2239	64.92
12/4/2020 13:32	192	190.3	186.7	2237	64.87
12/4/2020 13:32	192	189.6	187	2238	65.09
12/4/2020 13:32	192.1	190.1	187.5	2240	64.92
12/4/2020 13:32	192.2	190.3	187.1	2240	64.96
12/4/2020 13:32	192.2	189.8	186.7	2238	65.09
12/4/2020 13:32	192.2	190.3	187	2240	64.96
12/4/2020 13:32	192.3	189.6	187.1	2242	65.04
12/4/2020 13:32	192.4	189.5	186.9	2246	65.01
12/4/2020 13:32	192.4	190.5	186.8	2243	64.92
12/4/2020 13:32	192.5	189.8	186.7	2245	65.08
12/4/2020 13:32	192.6	190.1	186.9	2250	65.05
12/4/2020 13:32	192.7	189.6	187.7	2250	64.99
12/4/2020 13:32	192.7	189.8	187.4	2254	65.04
12/4/2020 13:32	192.8	190.3	186.8	2258	65.08
12/4/2020 13:32	192.9	190	186.8	2258	65.08
12/4/2020 13:32	192.9	190.2	187	2258	64.88
12/4/2020 13:32	193	190.1	187	2259	64.92
12/4/2020 13:32	193	189.7	187.1	2259	64.88
12/4/2020 13:32	193.1	190.2	187	2257	65.08
12/4/2020 13:32	193.2	189.9	186.8	2259	65.08
12/4/2020 13:32	193.3	190.1	186.8	2260	65.08
12/4/2020 13:32	193.4	189.7	187.5	2259	65.04
12/4/2020 13:32	193.5	189.6	187.9	2262	65.03
12/4/2020 13:32	193.4	190.3	187.2	2265	64.96
12/4/2020 13:32	193.5	189.7	187	2262	64.88
12/4/2020 13:32	193.5	190.3	187	2263	64.88
12/4/2020 13:33	193.6	190.2	187.1	2264	64.96
12/4/2020 13:33	193.7	189.5	187.2	2262	64.83
12/4/2020 13:33	193.6	190.5	187.2	2261	64.96
12/4/2020 13:33	193.8	189.8	187.1	2260	65.04
12/4/2020 13:33	193.7	190.2	187.1	2257	64.96
12/4/2020 13:33	193.8	189.7	186.8	2259	64.92
12/4/2020 13:33	193.8	189.8	187	2259	64.96
12/4/2020 13:33	193.9	190.2	187	2262	64.96
12/4/2020 13:33	193.9	189.8	187	2261	64.92
12/4/2020 13:33	193.9	190.2	187	2265	65
12/4/2020 13:33	193.9	190.2	187	2263	64.96
12/4/2020 13:33	193.9	189.6	186.9	2261	64.96
12/4/2020 13:33	194	190.2	187	2263	65
12/4/2020 13:33	193.9	189.6	186.9	2257	64.88
12/4/2020 13:33	193.9	190	187	2253	65.04
12/4/2020 13:33	193.9	189.7	186.9	2259	64.92
12/4/2020 13:33	193.9	190.2	187.1	2259	65.04
12/4/2020 13:33	194	190.2	187.4	2256	64.88

12/4/2020 13:33	194	189.6	187.5	2255	64.92
12/4/2020 13:33	193.9	190.2	187.3	2258	64.88
12/4/2020 13:33	194	190.2	187.3	2257	64.99
12/4/2020 13:33	194	190.1	187.1	2258	65.08
12/4/2020 13:33	193.9	190.2	187.7	2256	65.04
12/4/2020 13:33	193.9	191.2	188.2	2253	65.12
12/4/2020 13:33	194	190.8	187.9	2254	65.04
12/4/2020 13:33	194	191.2	187.5	2254	64.99
12/4/2020 13:33	193.9	190.8	187.2	2256	64.92
12/4/2020 13:33	193.9	191.1	187.1	2259	65.04
12/4/2020 13:33	193.9	191.1	187.3	2258	64.84
12/4/2020 13:33	193.9	191.4	187.4	2255	65.08
12/4/2020 13:33	193.9	190.8	187.5	2255	64.97
12/4/2020 13:33	193.9	190.6	187.7	2256	64.92
12/4/2020 13:33	193.9	191	187.4	2253	65.08
12/4/2020 13:33	193.9	191.2	187.4	2261	65.01
12/4/2020 13:33	193.9	191.3	187.2	2264	65
12/4/2020 13:33	193.9	191	187.2	2262	65
12/4/2020 13:33	193.9	190.8	187.2	2263	65.04
12/4/2020 13:33	193.9	191.2	187.3	2263	64.87
12/4/2020 13:33	193.9	191.3	187.5	2265	64.96
12/4/2020 13:33	193.8	191.3	187.5	2265	65
12/4/2020 13:33	193.8	191.5	187.9	2266	64.96
12/4/2020 13:33	193.8	190.8	187.8	2265	65
12/4/2020 13:33	193.7	191.4	188.1	2267	65
12/4/2020 13:33	193.7	190.5	187.9	2263	64.92
12/4/2020 13:33	193.7	191.1	187.8	2263	64.88
12/4/2020 13:33	193.7	191.4	187.7	2262	65.08
12/4/2020 13:33	193.6	190.9	187.7	2264	64.92
12/4/2020 13:33	193.6	191.1	187.5	2269	65.12
12/4/2020 13:33	193.5	190.7	187.3	2264	64.92
12/4/2020 13:33	193.5	191.1	187.4	2261	64.88
12/4/2020 13:33	193.5	191.4	187.5	2258	65.04
12/4/2020 13:33	193.5	190.8	187.6	2257	64.88
12/4/2020 13:33	193.5	190.7	187.6	2261	65.04
12/4/2020 13:33	193.5	191.2	187.6	2261	64.88
12/4/2020 13:33	193.4	191.2	187.7	2264	64.92
12/4/2020 13:33	193.4	191.5	187.5	2265	64.88
12/4/2020 13:33	193.3	190.9	187.2	2262	64.93
12/4/2020 13:33	193.3	190.8	187.1	2263	64.88
12/4/2020 13:33	193.3	190.7	187	2264	65
12/4/2020 13:33	193.2	191	187.7	2264	64.96
12/4/2020 13:34	193.2	191.4	188.1	2265	64.92
12/4/2020 13:34	193.2	191	188.3	2263	65.01
12/4/2020 13:34	193.1	190.9	188.1	2261	65
12/4/2020 13:34	193.1	191.4	187.7	2257	64.83
12/4/2020 13:34	193.1	190.6	187.4	2261	65

12/4/2020 13:34	192.9	191.2	187.5	2259	64.96
12/4/2020 13:34	193	190.7	187.3	2261	64.96
12/4/2020 13:34	192.9	190.8	187.4	2261	65
12/4/2020 13:34	192.9	191.3	187.6	2262	65.04
12/4/2020 13:34	192.9	190.7	187.6	2261	64.96
12/4/2020 13:34	192.8	191.4	187.6	2267	65
12/4/2020 13:34	192.9	190.7	187.4	2266	65.08
12/4/2020 13:34	192.8	190.7	187.8	2265	65.04
12/4/2020 13:34	192.7	190.7	187.6	2268	65.12
12/4/2020 13:34	192.7	190.9	187.5	2261	64.99
12/4/2020 13:34	192.6	191.3	187.7	2259	65.08
12/4/2020 13:34	192.6	190.7	187.8	2261	65.01
12/4/2020 13:34	192.5	190.7	187.7	2261	64.92
12/4/2020 13:34	192.5	191.4	187.5	2264	65.08
12/4/2020 13:34	192.4	191	187.5	2261	65.05
12/4/2020 13:34	192.4	191.2	187.5	2263	64.99
12/4/2020 13:34	192.4	190.7	187.5	2263	64.92
12/4/2020 13:34	192.3	190.8	187.4	2263	65
12/4/2020 13:34	192.3	191.4	187.1	2263	65.08
12/4/2020 13:34	192.3	191	187.7	2264	64.92
12/4/2020 13:34	192.2	191.3	187.8	2265	65.17
12/4/2020 13:34	192.2	191.2	188.2	2263	65
12/4/2020 13:34	192.1	190.6	188.2	2261	65.04
12/4/2020 13:34	192.1	190.9	187.4	2256	65.04
12/4/2020 13:34	192	190.9	187	2254	64.96
12/4/2020 13:34	192	190.9	187.4	2254	65.04
12/4/2020 13:34	192	191.2	187.5	2261	65.04
12/4/2020 13:34	191.8	190.7	187.4	2256	65.04
12/4/2020 13:34	191.8	190.9	187.5	2259	64.92
12/4/2020 13:34	191.8	190.7	187.1	2260	64.96
12/4/2020 13:34	191.8	191	187.5	2257	65.04
12/4/2020 13:34	191.7	191.5	188.2	2260	64.92
12/4/2020 13:34	191.6	191.4	187.5	2263	65.12
12/4/2020 13:34	191.6	191.2	187	2263	64.92
12/4/2020 13:34	191.6	190.8	187.2	2260	64.93
12/4/2020 13:34	191.5	191.2	187.3	2261	64.96
12/4/2020 13:34	191.4	190.8	187.4	2264	65.04
12/4/2020 13:34	191.4	190.7	187.2	2265	64.92
12/4/2020 13:34	191.3	191.4	188.1	2262	65.04
12/4/2020 13:34	191.3	190.7	187.9	2264	64.92
12/4/2020 13:34	191.3	191	187	2260	64.96
12/4/2020 13:34	191.2	191.2	187	2259	64.96
12/4/2020 13:34	191.1	191.2	187.3	2257	65
12/4/2020 13:34	191	191.3	187.5	2257	64.96
12/4/2020 13:34	191.1	190.9	187.5	2258	64.96
12/4/2020 13:34	191	191.5	187.5	2259	64.96
12/4/2020 13:34	190.9	190.7	187.2	2259	65

12/4/2020 13:34	190.9	191	187	2260	65
12/4/2020 13:34	190.9	191.2	187.4	2263	65
12/4/2020 13:34	190.8	191	188.1	2265	64.96
12/4/2020 13:34	190.8	191.4	187.9	2262	64.96
12/4/2020 13:34	190.8	190.7	188.2	2263	65.09
12/4/2020 13:34	190.7	190.7	187.7	2260	64.99
12/4/2020 13:34	190.6	190.9	187.1	2260	65.05
12/4/2020 13:35	190.5	190.7	187.2	2257	64.93
12/4/2020 13:35	190.5	191.2	187	2257	65.04
12/4/2020 13:35	190.5	191.2	187	2255	64.88
12/4/2020 13:35	190.5	190.8	187	2253	64.97
12/4/2020 13:35	190.3	191.1	186.9	2254	64.99
12/4/2020 13:35	190.3	191	187	2253	64.88
12/4/2020 13:35	190.3	191.3	187	2252	65.05
12/4/2020 13:35	190.2	191.4	186.9	2254	65.04
12/4/2020 13:35	190.2	190.5	187	2258	65
12/4/2020 13:35	190.1	190.9	187	2262	64.92
12/4/2020 13:35	190.1	190.9	186.9	2262	64.96
12/4/2020 13:35	190	191.1	186.8	2264	64.99
12/4/2020 13:35	190	191.4	187	2266	65.08
12/4/2020 13:35	189.9	191	187.5	2263	65
12/4/2020 13:35	189.9	191	188.1	2258	64.96
12/4/2020 13:35	189.8	191.4	187.9	2257	65.09
12/4/2020 13:35	189.8	190.7	187.4	2257	64.96
12/4/2020 13:35	189.8	190	187	2256	65.05
12/4/2020 13:35	189.8	189.5	187	2263	65
12/4/2020 13:35	189.7	190.2	187.3	2257	64.83
12/4/2020 13:35	189.6	189.6	187.2	2257	65
12/4/2020 13:35	189.6	189.8	187	2254	65.04
12/4/2020 13:35	189.5	190.3	186.8	2253	65
12/4/2020 13:35	189.5	189.6	186.5	2256	64.92
12/4/2020 13:35	189.4	190.2	187.7	2257	65
12/4/2020 13:35	189.4	189.6	187.7	2258	65
12/4/2020 13:35	189.4	189.6	187	2259	64.96
12/4/2020 13:35	189.4	190.3	187	2256	64.96
12/4/2020 13:35	189.4	189.8	187.1	2259	65.04
12/4/2020 13:35	189.3	190.2	187	2258	65.04
12/4/2020 13:35	189.3	189.6	187	2261	64.93
12/4/2020 13:35	189.2	190.3	186.7	2261	64.93
12/4/2020 13:35	189.2	190.5	187	2261	64.99
12/4/2020 13:35	189.2	189.8	187.7	2257	64.96
12/4/2020 13:35	189.2	190.3	187.4	2257	65.08
12/4/2020 13:35	189.1	189.7	187	2254	64.92
12/4/2020 13:35	189.1	189.6	187	2259	64.88
12/4/2020 13:35	189.1	190.5	187.1	2261	64.88
12/4/2020 13:35	189.1	189.9	187.1	2260	64.92
12/4/2020 13:35	189.1	190.2	186.9	2255	64.96

12/4/2020 13:35	189	190	186.8	2255	65.04
12/4/2020 13:35	189	189.5	186.9	2250	65
12/4/2020 13:35	189	189.8	187	2249	65.04
12/4/2020 13:35	189	190.1	187.4	2249	64.96
12/4/2020 13:35	189	190	187.6	2248	64.96
12/4/2020 13:35	189.1	190.3	187.4	2251	65.09
12/4/2020 13:35	189	190.2	187.1	2255	64.96
12/4/2020 13:35	189	190.1	187	2255	64.96
12/4/2020 13:35	189	189.7	186.7	2254	65
12/4/2020 13:35	189	189.6	186.8	2257	64.96
12/4/2020 13:35	189	189.6	187	2256	64.96
12/4/2020 13:35	189.1	189.6	187	2259	65
12/4/2020 13:35	189.1	190.3	187	2258	65
12/4/2020 13:35	189.1	190.2	186.9	2260	65.08
12/4/2020 13:35	189.1	189.8	187.1	2258	65.04
12/4/2020 13:35	189.1	190.5	187	2258	64.96
12/4/2020 13:35	189.1	190	187	2256	64.88
12/4/2020 13:35	189.2	190.2	186.7	2249	65.04
12/4/2020 13:35	189.2	189.8	186.8	2249	65.04
12/4/2020 13:35	189.2	189.6	186.8	2249	64.88
12/4/2020 13:36	189.2	190.1	186.8	2245	65.12
12/4/2020 13:36	189.2	189.6	186.8	2244	65.08
12/4/2020 13:36	189.3	190.3	187	2243	65.04
12/4/2020 13:36	189.3	190.2	186.7	2245	64.88
12/4/2020 13:36	189.4	189.6	186.8	2245	64.96
12/4/2020 13:36	189.4	189.6	186.8	2250	64.88
12/4/2020 13:36	189.4	189.9	186.8	2252	64.99
12/4/2020 13:36	189.4	190.1	187	2254	65
12/4/2020 13:36	189.4	190.1	187	2254	64.99
12/4/2020 13:36	189.5	190.4	187	2254	64.99
12/4/2020 13:36	189.6	189.9	187	2258	65
12/4/2020 13:36	189.5	189.8	187	2259	65
12/4/2020 13:36	189.6	190.2	187	2262	65
12/4/2020 13:36	189.6	190.3	187.3	2264	65
12/4/2020 13:36	189.6	190.3	187	2263	64.96
12/4/2020 13:36	189.7	190.4	187	2262	64.92
12/4/2020 13:36	189.7	189.8	187	2266	65.09
12/4/2020 13:36	189.7	190	186.8	2263	65
12/4/2020 13:36	189.8	190.3	187	2261	64.96
12/4/2020 13:36	189.8	189.6	186.8	2262	65.12
12/4/2020 13:36	189.8	190.5	186.8	2260	65
12/4/2020 13:36	189.8	189.7	186.9	2258	65.04
12/4/2020 13:36	189.9	189.8	186.7	2259	65.04
12/4/2020 13:36	190	189.6	186.7	2259	64.95
12/4/2020 13:36	190	190.1	186.8	2263	65
12/4/2020 13:36	190.1	190.3	186.8	2261	65.04
12/4/2020 13:36	190.1	189.8	187	2260	65

12/4/2020 13:36	190.1	189.7	187.1	2255	64.96
12/4/2020 13:36	190.2	189.5	187.3	2261	64.88
12/4/2020 13:36	190.2	190	187.2	2265	65
12/4/2020 13:36	190.3	190.2	187.6	2266	64.92
12/4/2020 13:36	190.3	190.1	187.6	2263	65.08
12/4/2020 13:36	190.3	189.8	187.7	2264	64.99
12/4/2020 13:36	190.4	189.8	187.7	2259	65.04
12/4/2020 13:36	190.5	190.2	187.5	2257	65.04
12/4/2020 13:36	190.5	190.2	187.1	2256	64.96
12/4/2020 13:36	190.6	190.1	186.8	2254	64.92
12/4/2020 13:36	190.5	190.4	186.7	2255	64.96
12/4/2020 13:36	190.6	189.7	186.5	2259	65.04
12/4/2020 13:36	190.6	190	186.8	2259	64.96
12/4/2020 13:36	190.6	190.3	186.9	2259	65
12/4/2020 13:36	190.7	190.1	187	2259	65.04
12/4/2020 13:36	190.7	190.1	187.1	2258	64.92
12/4/2020 13:36	190.8	190.2	187	2259	65.04
12/4/2020 13:36	190.9	189.6	186.7	2263	64.96
12/4/2020 13:36	190.8	190.3	187	2267	65
12/4/2020 13:36	190.9	189.9	187.4	2266	64.96
12/4/2020 13:36	190.9	190.4	187.7	2265	65
12/4/2020 13:36	191	190.4	187	2265	64.96
12/4/2020 13:36	191	189.8	186.7	2263	65.04
12/4/2020 13:36	191.1	190.2	186.9	2267	65.09
12/4/2020 13:36	191	190.3	187.2	2265	65.04
12/4/2020 13:36	191.1	189.7	187.2	2267	65
12/4/2020 13:36	191.2	190	187.1	2268	64.88
12/4/2020 13:36	191.3	189.7	186.7	2263	64.84
12/4/2020 13:36	191.3	190	187.2	2264	64.84
12/4/2020 13:36	191.3	189.5	187.7	2265	64.96
12/4/2020 13:36	191.3	189.6	187.7	2268	64.84
12/4/2020 13:36	191.4	190.4	187.1	2267	65.04
12/4/2020 13:37	191.4	190	186.8	2268	65.01
12/4/2020 13:37	191.4	190	187	2270	65.08
12/4/2020 13:37	191.4	189.9	187.2	2271	64.96
12/4/2020 13:37	191.5	189.6	187	2272	65.08
12/4/2020 13:37	191.6	190	187.2	2269	64.92
12/4/2020 13:37	191.6	189.9	187	2269	64.96
12/4/2020 13:37	191.5	190.2	186.9	2268	65.04
12/4/2020 13:37	191.6	190.2	186.9	2270	65.04
12/4/2020 13:37	191.7	189.8	186.8	2271	64.92
12/4/2020 13:37	191.7	190.3	187.2	2272	64.96
12/4/2020 13:37	191.7	189.5	187.4	2267	64.96
12/4/2020 13:37	191.8	190	187.4	2270	65.04
12/4/2020 13:37	191.8	190.2	187.5	2269	65
12/4/2020 13:37	191.8	189.6	187.7	2272	65
12/4/2020 13:37	191.8	189.9	187.4	2268	64.96

12/4/2020 13:37	191.8	189.8	187.4	2264	65.04
12/4/2020 13:37	191.9	190.1	187.3	2258	64.96
12/4/2020 13:37	192	190.1	187	2256	65
12/4/2020 13:37	192	189.6	187	2259	64.99
12/4/2020 13:37	192	190.1	186.8	2259	64.92
12/4/2020 13:37	192.1	189.6	187	2259	64.99
12/4/2020 13:37	192	190.1	187	2261	64.99
12/4/2020 13:37	192.1	190.2	187	2259	65.08
12/4/2020 13:37	192.1	189.6	187.2	2260	65.04
12/4/2020 13:37	192.1	190.4	187.2	2263	64.84
12/4/2020 13:37	192	189.5	187	2268	65.08
12/4/2020 13:37	192.2	190.2	187	2269	64.88
12/4/2020 13:37	192.2	190.3	186.7	2272	64.99
12/4/2020 13:37	192.2	189.6	187.2	2271	64.96
12/4/2020 13:37	192.2	190	187.7	2273	64.92
12/4/2020 13:37	192.2	190	187.2	2271	65.04
12/4/2020 13:37	192.2	189.8	187.2	2271	64.92
12/4/2020 13:37	192.2	189.8	187.2	2271	64.87
12/4/2020 13:37	192.2	190.2	187.3	2270	64.96
12/4/2020 13:37	192.2	190	187	2263	65.09
12/4/2020 13:37	192.2	189.9	186.8	2260	64.96
12/4/2020 13:37	192.2	190	187.7	2258	64.91
12/4/2020 13:37	192.2	189.8	187.7	2261	65.04
12/4/2020 13:37	192.2	190	186.9	2257	64.96
12/4/2020 13:37	192.2	190.4	187.3	2254	65
12/4/2020 13:37	192.2	189.7	187.2	2255	65.04
12/4/2020 13:37	192.1	190.2	187.2	2255	64.96
12/4/2020 13:37	192.2	190.4	187.2	2253	64.96
12/4/2020 13:37	192.2	190.3	187	2259	65.01
12/4/2020 13:37	192.2	190.1	187.2	2259	64.88
12/4/2020 13:37	192.2	189.9	188	2262	64.88
12/4/2020 13:37	192.2	189.7	187.7	2261	65
12/4/2020 13:37	192.2	190.4	187.1	2257	65.04
12/4/2020 13:37	192.2	190.2	187	2255	64.92
12/4/2020 13:37	192.3	190.3	187.2	2259	64.96
12/4/2020 13:37	192.3	190	187.4	2259	64.92
12/4/2020 13:37	192.3	189.8	187	2257	65.04
12/4/2020 13:37	192.4	190.2	187	2256	65.01
12/4/2020 13:37	192.3	190.1	186.8	2253	64.88
12/4/2020 13:37	192.4	190.4	187.1	2253	64.92
12/4/2020 13:37	192.4	190	187.9	2256	65.12
12/4/2020 13:37	192.5	190.3	187.9	2254	64.96
12/4/2020 13:37	192.5	189.8	187.4	2255	64.96
12/4/2020 13:37	192.6	189.8	187	2259	64.96
12/4/2020 13:38	192.6	190.2	187	2251	64.92
12/4/2020 13:38	192.7	190.1	187.2	2248	65.04
12/4/2020 13:38	192.8	189.8	187.4	2243	64.88

12/4/2020 13:38	192.8	190.3	187.2	2242	64.96
12/4/2020 13:38	192.9	190.2	187.4	2241	64.92
12/4/2020 13:38	193	190.2	187.4	2246	65
12/4/2020 13:38	192.9	190.1	187.4	2250	65
12/4/2020 13:38	193.1	189.8	187.5	2252	65
12/4/2020 13:38	193.1	189.8	187.5	2250	64.96
12/4/2020 13:38	193.3	190	187.5	2255	65
12/4/2020 13:38	193.3	190.2	187.5	2256	64.96
12/4/2020 13:38	193.4	190.2	187.5	2256	65
12/4/2020 13:38	193.5	190.3	187.2	2253	65
12/4/2020 13:38	193.5	189.7	187.1	2258	64.88
12/4/2020 13:38	193.7	190.2	187.7	2261	65
12/4/2020 13:38	193.7	190.2	188.1	2261	65
12/4/2020 13:38	193.8	190.1	187.8	2258	64.92
12/4/2020 13:38	193.9	189.8	187	2261	65.08
12/4/2020 13:38	194	190.2	187.3	2262	65.04
12/4/2020 13:38	194.1	190.7	187.6	2263	64.96
12/4/2020 13:38	194.2	191.5	187.7	2258	64.99
12/4/2020 13:38	194.2	190.7	187.3	2261	64.96
12/4/2020 13:38	194.3	190.8	187.5	2264	64.92
12/4/2020 13:38	194.4	191.1	188.3	2264	64.99
12/4/2020 13:38	194.5	190.8	187.5	2263	64.96
12/4/2020 13:38	194.5	191	187.2	2261	64.99
12/4/2020 13:38	194.7	191.2	187.5	2263	64.96
12/4/2020 13:38	194.7	190.7	187.5	2261	65
12/4/2020 13:38	194.7	191.2	187.5	2259	64.96
12/4/2020 13:38	194.7	190.6	187.2	2265	64.88
12/4/2020 13:38	194.8	190.7	187.4	2266	65.01
12/4/2020 13:38	194.8	191.4	188.2	2265	64.96
12/4/2020 13:38	194.9	190.7	188.4	2267	64.88
12/4/2020 13:38	195	191.2	188.1	2270	65.04
12/4/2020 13:38	195	190.7	187.4	2269	64.87
12/4/2020 13:38	195.1	190.8	187.3	2268	65
12/4/2020 13:38	195.1	191.5	187.4	2270	65.04
12/4/2020 13:38	195.1	191	187.2	2267	64.92
12/4/2020 13:38	195.2	191.5	187.4	2266	64.96
12/4/2020 13:38	195.2	190.8	187.2	2268	65
12/4/2020 13:38	195.3	190.7	187.4	2268	64.88
12/4/2020 13:38	195.3	191.5	187.2	2272	64.96
12/4/2020 13:38	195.3	191	187.2	2270	65.04
12/4/2020 13:38	195.4	191.3	187.3	2267	65
12/4/2020 13:38	195.4	191.2	187.5	2269	64.96
12/4/2020 13:38	195.4	190.7	187.7	2272	65.05
12/4/2020 13:38	195.4	190.9	188.3	2272	65.04
12/4/2020 13:38	195.5	190.9	188.6	2269	64.92
12/4/2020 13:38	195.5	191.1	188.5	2271	64.92
12/4/2020 13:38	195.5	191.3	187.9	2274	65.03

12/4/2020 13:38	195.5	191.4	187.7	2274	65.08
12/4/2020 13:38	195.5	191.2	187.6	2271	64.96
12/4/2020 13:38	195.6	190.7	187.7	2272	64.96
12/4/2020 13:38	195.6	191	187.9	2273	64.84
12/4/2020 13:38	195.7	191.4	187.8	2275	64.96
12/4/2020 13:38	195.7	191.2	187.9	2270	64.92
12/4/2020 13:38	195.7	191.2	187.7	2269	65
12/4/2020 13:38	195.7	190.8	187.7	2268	64.96
12/4/2020 13:38	195.7	190.8	187.7	2268	64.84
12/4/2020 13:38	195.7	191.4	187.5	2267	65.08
12/4/2020 13:39	195.7	190.7	187.4	2265	65
12/4/2020 13:39	195.7	191.2	187.5	2265	65
12/4/2020 13:39	195.7	190.8	188.2	2271	64.92
12/4/2020 13:39	195.7	191.4	188.8	2267	64.87
12/4/2020 13:39	195.7	191.3	188.6	2263	64.96
12/4/2020 13:39	195.7	191.2	188.2	2263	65.04
12/4/2020 13:39	195.8	191.2	187.9	2266	65
12/4/2020 13:39	195.8	191	187.8	2269	64.96
12/4/2020 13:39	195.7	190.8	188.1	2268	64.96
12/4/2020 13:39	195.8	191.3	188.2	2268	64.96
12/4/2020 13:39	195.8	190.7	187.9	2271	65.05
12/4/2020 13:39	195.8	191.4	187.8	2274	64.92
12/4/2020 13:39	195.9	190.9	187.7	2270	64.92
12/4/2020 13:39	195.9	191	187.7	2271	65.09
12/4/2020 13:39	195.9	191.4	188.1	2271	64.88
12/4/2020 13:39	195.9	191.1	188.8	2269	64.88
12/4/2020 13:39	195.9	191	188.9	2269	64.96
12/4/2020 13:39	195.9	190.9	188.5	2270	64.88
12/4/2020 13:39	195.9	190.9	188.2	2269	64.92
12/4/2020 13:39	195.9	190.7	187.9	2271	65.05
12/4/2020 13:39	195.9	190.8	187.8	2275	64.88
12/4/2020 13:39	195.9	191.3	187.8	2274	64.88
12/4/2020 13:39	195.9	190.9	187.9	2275	65.12
12/4/2020 13:39	195.9	190.9	188.1	2270	64.95
12/4/2020 13:39	195.9	190.6	188.2	2269	64.92
12/4/2020 13:39	195.9	190.8	188.2	2271	65.04
12/4/2020 13:39	195.9	191.4	188.1	2271	64.88
12/4/2020 13:39	195.9	190.7	188.1	2269	64.88
12/4/2020 13:39	195.7	190.9	188.1	2266	64.88
12/4/2020 13:39	195.9	191.3	188.4	2263	64.92
12/4/2020 13:39	195.9	192.1	188.2	2266	64.88
12/4/2020 13:39	195.9	192.4	188.1	2263	64.93
12/4/2020 13:39	195.8	192.2	188.2	2259	64.88
12/4/2020 13:39	195.8	191.9	188.2	2268	64.96
12/4/2020 13:39	195.8	192.3	187.9	2275	65.05
12/4/2020 13:39	195.7	192	187.9	2278	64.96
12/4/2020 13:39	195.7	192.2	187.9	2275	64.96

12/4/2020 13:39	195.6	192.1	188.6	2275	65
12/4/2020 13:39	195.6	191.7	188.9	2275	65
12/4/2020 13:39	195.6	192.1	188.5	2275	65
12/4/2020 13:39	195.5	191.9	187.9	2277	65
12/4/2020 13:39	195.5	192.1	187.8	2277	64.96
12/4/2020 13:39	195.4	192.4	188.1	2275	64.92
12/4/2020 13:39	195.4	191.9	187.8	2278	65.12
12/4/2020 13:39	195.4	192.3	188	2275	65.05
12/4/2020 13:39	195.2	191.9	188	2278	65.08
12/4/2020 13:39	195.2	192.1	188.8	2277	64.96
12/4/2020 13:39	195.2	192.2	188.7	2276	65.08
12/4/2020 13:39	195.2	191.9	187.7	2275	65.08
12/4/2020 13:39	195.2	192.2	187.9	2278	64.93
12/4/2020 13:39	195.1	192	188.1	2278	64.88
12/4/2020 13:39	195.1	192.2	188.2	2283	65.08
12/4/2020 13:39	195	191.9	188	2284	65.12
12/4/2020 13:39	194.9	191.9	187.7	2282	65.04
12/4/2020 13:39	194.8	192.1	188.2	2279	65.04
12/4/2020 13:39	194.8	191.9	188.8	2280	64.96
12/4/2020 13:39	194.7	192	188.8	2282	65.04
12/4/2020 13:39	194.7	191.9	188.3	2281	64.88
12/4/2020 13:39	194.6	192.1	187.9	2282	65.05
12/4/2020 13:40	194.5	192.2	187.8	2279	64.96
12/4/2020 13:40	194.6	192.4	187.7	2281	64.96
12/4/2020 13:40	194.4	192	187.9	2280	64.96
12/4/2020 13:40	194.4	191.9	187.8	2279	64.92
12/4/2020 13:40	194.3	192.2	188.1	2282	65
12/4/2020 13:40	194.2	192.2	188.2	2281	64.96
12/4/2020 13:40	194.2	191.9	188.2	2279	64.96
12/4/2020 13:40	194	191.9	188.2	2276	65
12/4/2020 13:40	193.9	192.3	188.2	2271	65
12/4/2020 13:40	193.9	191.9	188.1	2272	65.08
12/4/2020 13:40	193.8	192.3	188.1	2274	64.92
12/4/2020 13:40	193.9	192.2	188.1	2275	64.92
12/4/2020 13:40	193.7	192	188.1	2276	64.88
12/4/2020 13:40	193.7	192.4	188.1	2278	65.04
12/4/2020 13:40	193.7	192.1	187.9	2276	65.04
12/4/2020 13:40	193.5	192.2	187.9	2279	64.92
12/4/2020 13:40	193.5	192.3	187.9	2275	64.8
12/4/2020 13:40	193.4	192.3	187.8	2279	65.03
12/4/2020 13:40	193.5	192.4	187.8	2279	64.96
12/4/2020 13:40	193.4	192.1	187.7	2278	64.91
12/4/2020 13:40	193.3	192.1	187.7	2279	65.08
12/4/2020 13:40	193.2	192.1	187.9	2278	65.04
12/4/2020 13:40	193.2	192.1	187.9	2276	64.92
12/4/2020 13:40	193.3	192.4	187.8	2274	64.96
12/4/2020 13:40	193.3	191.9	187.9	2275	65

12/4/2020 13:40	193.2	192.1	187.8	2273	65.04
12/4/2020 13:40	193.2	192.1	187.9	2271	64.96
12/4/2020 13:40	193.3	192	188.1	2272	65.04
12/4/2020 13:40	193.3	192.1	188.2	2275	64.99
12/4/2020 13:40	193.3	191.9	188.2	2274	64.96
12/4/2020 13:40	193.3	192.4	188.3	2272	64.96
12/4/2020 13:40	193.2	191.8	188.4	2272	64.92
12/4/2020 13:40	193.2	191.9	188.5	2274	65
12/4/2020 13:40	193.3	192	188.1	2275	65.04
12/4/2020 13:40	193.3	191.9	188.1	2275	64.96
12/4/2020 13:40	193.2	192.2	188.3	2274	65.04
12/4/2020 13:40	193.2	192.1	188.1	2276	65.04
12/4/2020 13:40	193.2	192.3	188.1	2279	64.92
12/4/2020 13:40	193.3	192.2	187.8	2279	64.96
12/4/2020 13:40	193.3	192	187.7	2278	65.12
12/4/2020 13:40	193.3	192.1	187.7	2275	64.83
12/4/2020 13:40	193.3	192.1	187.9	2272	65.04
12/4/2020 13:40	193.3	192.3	187.7	2271	65.04
12/4/2020 13:40	193.3	192.2	187.9	2269	65.03
12/4/2020 13:40	193.3	192	188.1	2270	64.99
12/4/2020 13:40	193.3	192.1	188.1	2274	65.04
12/4/2020 13:40	193.3	192.3	188.2	2274	64.88
12/4/2020 13:40	193.3	192	188.2	2275	64.99
12/4/2020 13:40	193.3	192.2	188.2	2275	65.12
12/4/2020 13:40	193.3	191.8	188.2	2277	64.99
12/4/2020 13:40	193.3	192.2	188.2	2281	65.08
12/4/2020 13:40	193.3	191.9	188.1	2280	64.96
12/4/2020 13:40	193.3	191.8	188	2279	64.88
12/4/2020 13:40	193.3	192.2	187.9	2279	64.96
12/4/2020 13:40	193.3	192.1	188.2	2275	65.03
12/4/2020 13:40	193.3	192.1	188.1	2270	65.08
12/4/2020 13:40	193.2	192.2	188.1	2268	64.99
12/4/2020 13:40	193.2	191.9	188.1	2275	65.08
12/4/2020 13:40	193.1	192.2	187.7	2272	64.99
12/4/2020 13:40	193.2	191.9	188	2276	65.04
12/4/2020 13:41	193.1	192.2	187.7	2278	65.08
12/4/2020 13:41	193	192.2	187.8	2279	65.04
12/4/2020 13:41	193	191.9	187.8	2277	65.08
12/4/2020 13:41	193	192.1	187.8	2278	64.99
12/4/2020 13:41	192.9	192	187.8	2275	65
12/4/2020 13:41	192.9	192.1	187.8	2273	64.96
12/4/2020 13:41	192.9	192.2	187.7	2272	65.04
12/4/2020 13:41	192.9	191.9	187.9	2272	65.04
12/4/2020 13:41	192.8	192.3	187.9	2270	64.96
12/4/2020 13:41	192.8	192.1	187.7	2267	64.92
12/4/2020 13:41	192.7	191.9	187.9	2270	64.96
12/4/2020 13:41	192.7	192.3	187.7	2267	65

12/4/2020 13:41	192.7	191.8	188.1	2265	64.96
12/4/2020 13:41	192.7	192.1	187.9	2263	65
12/4/2020 13:41	192.7	192	188	2265	65
12/4/2020 13:41	192.7	192	188.1	2264	64.96
12/4/2020 13:41	192.6	192.2	187.9	2265	65
12/4/2020 13:41	192.7	192.1	188	2266	64.92
12/4/2020 13:41	192.7	192.4	188	2261	64.88
12/4/2020 13:41	192.6	191.9	188	2265	64.96
12/4/2020 13:41	192.8	191.9	188.1	2270	64.96
12/4/2020 13:41	192.7	192.2	188.1	2265	65
12/4/2020 13:41	192.8	191.7	187.9	2264	65.04
12/4/2020 13:41	192.8	192.2	188	2261	64.88
12/4/2020 13:41	192.8	191.9	187.9	2265	64.88
12/4/2020 13:41	192.8	192	187.7	2269	64.96
12/4/2020 13:41	192.8	192.2	187.7	2265	64.88
12/4/2020 13:41	192.8	192.1	187.7	2266	64.84
12/4/2020 13:41	192.9	192.4	187.7	2266	64.96
12/4/2020 13:41	192.9	192	187.6	2261	64.99
12/4/2020 13:41	192.9	191.8	187.5	2261	64.88
12/4/2020 13:41	193	192.2	187.9	2259	65.12
12/4/2020 13:41	193.1	191.9	188.3	2263	64.83
12/4/2020 13:41	193	192.1	188.8	2265	65.04
12/4/2020 13:41	193.1	192.2	188.9	2264	65.04
12/4/2020 13:41	193.1	192.1	188.4	2262	64.96
12/4/2020 13:41	193.2	191.9	188	2259	65.08
12/4/2020 13:41	193.3	192	187.9	2260	65.04
12/4/2020 13:41	193.3	192.2	188.1	2261	64.88
12/4/2020 13:41	193.4	192.2	188.1	2258	65.04
12/4/2020 13:41	193.4	192.1	188.1	2256	64.96
12/4/2020 13:41	193.5	192.2	188.1	2254	64.84
12/4/2020 13:41	193.6	191.9	187.7	2251	64.88
12/4/2020 13:41	193.6	191.7	187.7	2254	64.92
12/4/2020 13:41	193.7	191.9	188	2257	64.92
12/4/2020 13:41	193.8	191.7	188.6	2262	64.92
12/4/2020 13:41	193.9	192.3	188.9	2268	65.03
12/4/2020 13:41	194	191.7	188.5	2267	65.08
12/4/2020 13:41	194.1	192.1	188	2268	65.03
12/4/2020 13:41	194.1	192.1	187.9	2271	64.8
12/4/2020 13:41	194.2	191.9	188.1	2276	65.08
12/4/2020 13:41	194.3	192.2	188.1	2278	64.8
12/4/2020 13:41	194.5	191.9	188.4	2278	64.83
12/4/2020 13:41	194.6	192	188.4	2275	64.92
12/4/2020 13:41	194.7	191.8	188.2	2272	64.83
12/4/2020 13:41	194.8	191.9	188.3	2272	64.99
12/4/2020 13:41	194.8	192.3	188.1	2270	65.04
12/4/2020 13:41	195	192.1	188	2272	64.83
12/4/2020 13:41	195	192.2	187.9	2276	64.88

12/4/2020 13:42	195.1	191.9	187.9	2272	64.92
12/4/2020 13:42	195.3	191.8	188.1	2267	65.03
12/4/2020 13:42	195.4	191.9	188.1	2266	64.88
12/4/2020 13:42	195.5	191.9	188	2271	65.08
12/4/2020 13:42	195.5	192	188.3	2272	64.96
12/4/2020 13:42	195.7	192.3	188.5	2268	64.92
12/4/2020 13:42	195.7	192.1	188.4	2270	64.99
12/4/2020 13:42	195.8	192.2	188.5	2271	64.96
12/4/2020 13:42	195.9	192.1	188.2	2274	64.92
12/4/2020 13:42	195.9	192.1	188.9	2274	65.08
12/4/2020 13:42	196	192.4	188.9	2279	64.83
12/4/2020 13:42	196.1	191.8	188	2280	64.96
12/4/2020 13:42	196.1	192.1	188.4	2286	65.08
12/4/2020 13:42	196.2	192.1	188.5	2286	64.88
12/4/2020 13:42	196.2	192.2	188.3	2283	64.92
12/4/2020 13:42	196.3	192.2	188.5	2285	65.04
12/4/2020 13:42	196.3	192.1	189.3	2284	64.92
12/4/2020 13:42	196.4	192.2	188.1	2283	64.88
12/4/2020 13:42	196.5	192	188.4	2281	64.96
12/4/2020 13:42	196.5	192.4	188.6	2278	64.92
12/4/2020 13:42	196.5	192.2	188.5	2281	64.92
12/4/2020 13:42	196.6	191.9	188.5	2278	65.04
12/4/2020 13:42	196.6	192.2	189.3	2275	65
12/4/2020 13:42	196.6	192.1	188.6	2272	64.92
12/4/2020 13:42	196.7	192	188.1	2274	65
12/4/2020 13:42	196.7	192.3	188.5	2274	64.92
12/4/2020 13:42	196.7	191.9	188.5	2272	65
12/4/2020 13:42	196.9	192.2	188.4	2268	65
12/4/2020 13:42	196.8	192.1	188.8	2272	64.96
12/4/2020 13:42	196.9	192	189.5	2272	64.92
12/4/2020 13:42	196.9	192.2	188.5	2268	64.92
12/4/2020 13:42	196.9	192	188.4	2270	64.92
12/4/2020 13:42	197	192.1	188.6	2272	64.96
12/4/2020 13:42	197	192.1	188.8	2272	65
12/4/2020 13:42	197	192.1	188.6	2276	64.96
12/4/2020 13:42	197	193.1	188.8	2279	64.96
12/4/2020 13:42	197	193.1	189.6	2280	64.99
12/4/2020 13:42	197	192.9	188.6	2282	64.96
12/4/2020 13:42	197	193.1	188.4	2282	64.96
12/4/2020 13:42	197.1	193.2	188.5	2282	64.88
12/4/2020 13:42	197.1	193.1	188.7	2284	64.92
12/4/2020 13:42	197.2	193.3	188.9	2284	65.04
12/4/2020 13:42	197.2	193	188.7	2279	64.96
12/4/2020 13:42	197.2	193	189.4	2279	65.12
12/4/2020 13:42	197.2	193.2	189.5	2279	64.99
12/4/2020 13:42	197.3	193.1	188.7	2272	64.92
12/4/2020 13:42	197.3	193.1	188.6	2271	64.99

12/4/2020 13:42	197.2	193.3	188.9	2271	64.95
12/4/2020 13:42	197.3	193.1	188.8	2270	64.96
12/4/2020 13:42	197.4	192.9	188.8	2275	64.96
12/4/2020 13:42	197.3	193.1	188.8	2279	64.95
12/4/2020 13:42	197.4	193.3	189.5	2281	65.04
12/4/2020 13:42	197.4	193.1	189.3	2282	64.88
12/4/2020 13:42	197.4	193.2	188.6	2281	65.08
12/4/2020 13:42	197.4	193.3	188.7	2279	64.92
12/4/2020 13:42	197.3	193.2	189.1	2279	64.88
12/4/2020 13:42	197.4	193	188.9	2283	64.83
12/4/2020 13:42	197.4	193.2	189.1	2286	64.99
12/4/2020 13:42	197.4	193.2	188.8	2285	65.08
12/4/2020 13:43	197.5	193.1	189.6	2282	64.88
12/4/2020 13:43	197.4	193.1	189.8	2280	64.88
12/4/2020 13:43	197.5	192.9	188.8	2283	65.08
12/4/2020 13:43	197.5	193.2	188.6	2280	64.88
12/4/2020 13:43	197.6	193	189.1	2279	64.88
12/4/2020 13:43	197.5	193	189.2	2279	65.04
12/4/2020 13:43	197.6	193.2	188.9	2281	64.99
12/4/2020 13:43	197.6	193.2	188.9	2279	64.96
12/4/2020 13:43	197.6	193.2	189.8	2278	64.92
12/4/2020 13:43	197.6	193	189.4	2276	65
12/4/2020 13:43	197.5	193.1	188.8	2278	64.92
12/4/2020 13:43	197.6	193	189.2	2282	64.92
12/4/2020 13:43	197.6	192.9	189.3	2279	64.88
12/4/2020 13:43	197.6	193.2	189	2280	64.92
12/4/2020 13:43	197.6	193.2	189.5	2281	65.09
12/4/2020 13:43	197.6	193.2	189.8	2278	64.92
12/4/2020 13:43	197.6	193.1	188.8	2280	64.96
12/4/2020 13:43	197.6	192.9	188.9	2279	64.96
12/4/2020 13:43	197.5	193.2	189.3	2278	64.92
12/4/2020 13:43	197.6	193	189.2	2278	64.88
12/4/2020 13:43	197.6	193.2	188.9	2279	65
12/4/2020 13:43	197.6	193.1	189.5	2279	65.04
12/4/2020 13:43	197.7	193.2	189.8	2281	65
12/4/2020 13:43	197.7	193.3	188.9	2288	65
12/4/2020 13:43	197.6	193.1	188.9	2287	65
12/4/2020 13:43	197.6	193.1	189.3	2290	65.04
12/4/2020 13:43	197.6	193.2	189.3	2291	64.88
12/4/2020 13:43	197.6	193.1	189.2	2289	64.96
12/4/2020 13:43	197.6	193.2	189.1	2292	64.88
12/4/2020 13:43	197.6	193.1	190	2295	65.04
12/4/2020 13:43	197.6	193	189.7	2299	64.96
12/4/2020 13:43	197.6	193	189.1	2298	64.88
12/4/2020 13:43	197.6	193.2	189	2294	64.92
12/4/2020 13:43	197.6	193.1	189.3	2292	65.12
12/4/2020 13:43	197.6	193.3	189.3	2286	64.88

12/4/2020 13:43	197.6	193.1	189.3	2287	64.92
12/4/2020 13:43	197.6	193	189.3	2291	65.16
12/4/2020 13:43	197.6	193.1	189.1	2288	65.04
12/4/2020 13:43	197.6	193.2	189.3	2289	64.92
12/4/2020 13:43	197.5	193.2	189.8	2291	65
12/4/2020 13:43	197.6	193.3	190	2287	64.87
12/4/2020 13:43	197.6	193.2	189.5	2283	65
12/4/2020 13:43	197.6	193	188.9	2283	65
12/4/2020 13:43	197.6	193.1	189.1	2282	65.04
12/4/2020 13:43	197.6	193.1	189.3	2281	65.04
12/4/2020 13:43	197.7	193.2	189.5	2280	65
12/4/2020 13:43	197.6	193.1	189.5	2278	64.92
12/4/2020 13:43	197.6	193	189.5	2274	65
12/4/2020 13:43	197.6	193.2	189.5	2274	65.04
12/4/2020 13:43	197.6	193.1	189.3	2276	64.92
12/4/2020 13:43	197.6	193	189.3	2279	64.92
12/4/2020 13:43	197.6	194.3	189.5	2280	65
12/4/2020 13:43	197.6	194.2	190.2	2286	64.92
12/4/2020 13:43	197.6	194.1	190.3	2285	64.92
12/4/2020 13:43	197.6	194	189.6	2288	64.88
12/4/2020 13:43	197.5	193.8	189.1	2285	65.04
12/4/2020 13:43	197.6	194	189.2	2286	64.88
12/4/2020 13:43	197.5	193.6	189.3	2285	65.08
12/4/2020 13:43	197.6	193.6	189.5	2286	64.96
12/4/2020 13:43	197.6	193.8	189.5	2292	64.99
12/4/2020 13:44	197.6	193.8	189.3	2293	64.88
12/4/2020 13:44	197.5	194.4	189.5	2292	64.97
12/4/2020 13:44	197.6	193.7	190.3	2289	64.99
12/4/2020 13:44	197.6	193.6	190.1	2287	64.96
12/4/2020 13:44	197.5	194.2	189.3	2294	64.96
12/4/2020 13:44	197.6	193.8	189.3	2293	64.96
12/4/2020 13:44	197.6	194	189.5	2291	65
12/4/2020 13:44	197.6	194	189.6	2288	64.96
12/4/2020 13:44	197.5	193.7	189.5	2288	65
12/4/2020 13:44	197.5	194.1	189.3	2283	64.96
12/4/2020 13:44	197.6	194	189.9	2285	64.92
12/4/2020 13:44	197.5	194	190.3	2287	64.96
12/4/2020 13:44	197.5	194.1	189.9	2288	65.09
12/4/2020 13:44	197.6	194.1	189.3	2286	64.96
12/4/2020 13:44	197.6	194.1	189.3	2288	64.96
12/4/2020 13:44	197.5	193.8	189.7	2288	64.88
12/4/2020 13:44	197.5	193.8	189.7	2288	64.92
12/4/2020 13:44	197.5	194.1	189.4	2286	65.04
12/4/2020 13:44	197.5	193.8	189.3	2287	64.96
12/4/2020 13:44	197.6	193.9	189.6	2286	65.04
12/4/2020 13:44	197.6	193.7	190.3	2287	64.92
12/4/2020 13:44	197.5	193.8	190.3	2288	65.04

12/4/2020 13:44	197.5	194.2	189.6	2286	65.08
12/4/2020 13:44	197.5	193.5	189.5	2283	64.96
12/4/2020 13:44	197.5	194.1	189.5	2285	65
12/4/2020 13:44	197.4	194.1	189.5	2289	65.08
12/4/2020 13:44	197.5	193.8	189.6	2288	64.96
12/4/2020 13:44	197.5	193.6	189.6	2288	64.96
12/4/2020 13:44	197.5	193.7	189.8	2287	65
12/4/2020 13:44	197.5	194.1	189.8	2289	65
12/4/2020 13:44	197.5	193.9	189.8	2285	65
12/4/2020 13:44	197.4	193.7	189.7	2285	65.09
12/4/2020 13:44	197.4	193.8	190	2283	64.96
12/4/2020 13:44	197.5	193.6	189.7	2285	64.96
12/4/2020 13:44	197.4	194.1	189.9	2284	65.05
12/4/2020 13:44	197.4	194.1	189.7	2283	64.88
12/4/2020 13:44	197.4	193.6	189.6	2286	64.92
12/4/2020 13:44	197.4	193.9	189.6	2281	65
12/4/2020 13:44	197.4	193.8	189.6	2280	64.99
12/4/2020 13:44	197.4	194	189.9	2279	65.08
12/4/2020 13:44	197.4	194.2	189.8	2281	64.88
12/4/2020 13:44	197.4	193.8	189.7	2283	64.84
12/4/2020 13:44	197.3	193.9	189.7	2287	64.92
12/4/2020 13:44	197.3	193.8	189.8	2286	65.12
12/4/2020 13:44	197.3	194.1	189.8	2289	65.04
12/4/2020 13:44	197.3	193.7	189.9	2285	65.04
12/4/2020 13:44	197.3	194.2	190	2285	64.96
12/4/2020 13:44	197.4	194	190.1	2290	64.92
12/4/2020 13:44	197.3	193.6	189.9	2293	65.04
12/4/2020 13:44	197.3	194	189.8	2291	64.92
12/4/2020 13:44	197.2	194.2	190	2289	64.96
12/4/2020 13:44	197.3	193.8	189.9	2290	65.04
12/4/2020 13:44	197.2	194	189.6	2290	65.08
12/4/2020 13:44	197.2	193.8	189.6	2286	65
12/4/2020 13:44	197.2	194.1	189.5	2281	65.04
12/4/2020 13:44	197.2	193.6	189.5	2288	64.92
12/4/2020 13:44	197.2	194.2	189.6	2287	65.08
12/4/2020 13:44	197.2	194.2	190.1	2288	64.99
12/4/2020 13:44	197.2	194.1	190.5	2285	64.92
12/4/2020 13:45	197.1	194.2	190.7	2288	65
12/4/2020 13:45	197.2	194.1	190.5	2290	65
12/4/2020 13:45	197.1	193.8	190.3	2289	64.92
12/4/2020 13:45	197	194.2	190.1	2287	64.96
12/4/2020 13:45	197.1	194	189.9	2288	64.96
12/4/2020 13:45	197.1	193.8	189.9	2285	65.04
12/4/2020 13:45	197.1	194.2	189.6	2291	65.04
12/4/2020 13:45	197	194.2	189.6	2286	64.92
12/4/2020 13:45	197	194.3	189.8	2286	64.96
12/4/2020 13:45	197	194	189.6	2286	65.01

12/4/2020 13:45	197	193.7	189.9	2283	64.99
12/4/2020 13:45	197	193.6	189.7	2282	65
12/4/2020 13:45	197	194	189.8	2280	64.92
12/4/2020 13:45	197	194.3	189.7	2284	64.88
12/4/2020 13:45	197	193.8	189.8	2283	64.92
12/4/2020 13:45	197	194.2	189.6	2288	65.01
12/4/2020 13:45	196.9	193.9	189.9	2289	64.8
12/4/2020 13:45	197	193.6	189.8	2286	64.92
12/4/2020 13:45	196.9	194.2	190	2285	64.92
12/4/2020 13:45	196.9	193.6	189.6	2285	64.96
12/4/2020 13:45	196.8	194.3	190	2286	65.04
12/4/2020 13:45	196.8	194.1	190.2	2286	65.04
12/4/2020 13:45	196.8	193.8	190.3	2283	64.96
12/4/2020 13:45	196.9	194.2	190.5	2286	64.96
12/4/2020 13:45	196.8	193.8	190.8	2289	65.08
12/4/2020 13:45	196.9	193.9	190.8	2286	64.99
12/4/2020 13:45	196.7	194.3	190.7	2286	64.96
12/4/2020 13:45	196.8	193.8	190.2	2290	65.09
12/4/2020 13:45	196.8	194	190	2287	64.96
12/4/2020 13:45	196.7	193.6	189.6	2289	64.96
12/4/2020 13:45	196.7	193.7	189.6	2292	65
12/4/2020 13:45	196.7	194.3	189.7	2289	64.92
12/4/2020 13:45	196.7	194	189.8	2289	65
12/4/2020 13:45	196.6	194.3	190.1	2287	64.96
12/4/2020 13:45	196.7	194	190.2	2292	65
12/4/2020 13:45	196.7	193.8	190.1	2292	64.92
12/4/2020 13:45	196.6	193.8	190.2	2291	64.96
12/4/2020 13:45	196.6	193.8	190.1	2292	64.96
12/4/2020 13:45	196.6	194.1	189.9	2291	65
12/4/2020 13:45	196.6	194.1	190	2288	64.92
12/4/2020 13:45	196.6	193.8	190.7	2291	64.96
12/4/2020 13:45	196.5	193.8	190.9	2296	64.88
12/4/2020 13:45	196.6	193.8	190.3	2296	64.92
12/4/2020 13:45	196.5	194.2	189.8	2295	65.08
12/4/2020 13:45	196.5	194	189.8	2293	64.92
12/4/2020 13:45	196.5	193.8	189.8	2293	64.99
12/4/2020 13:45	196.5	194.3	189.8	2290	65.12
12/4/2020 13:45	196.7	194.1	190.1	2288	64.88
12/4/2020 13:45	196.5	194.3	189.9	2289	65.08
12/4/2020 13:45	196.4	194.2	190	2290	64.96
12/4/2020 13:45	196.4	193.7	190.1	2288	64.99
12/4/2020 13:45	196.4	193.9	190	2285	65.04
12/4/2020 13:45	196.4	193.8	190.1	2287	65.04
12/4/2020 13:45	196.4	194.1	190.2	2285	64.96
12/4/2020 13:45	196.4	194.1	190.1	2286	65
12/4/2020 13:45	196.3	193.8	190.2	2289	65.09
12/4/2020 13:45	196.3	194.3	190	2291	64.96

12/4/2020 13:45	196.3	193.7	190	2291	65
12/4/2020 13:45	196.3	193.9	190	2293	65
12/4/2020 13:45	196.3	194.1	190.1	2289	64.91
12/4/2020 13:46	196.3	194.2	190.1	2286	64.96
12/4/2020 13:46	196.2	193.8	189.9	2285	64.96
12/4/2020 13:46	196.2	194	190	2285	65.08
12/4/2020 13:46	196.2	193.8	190	2285	64.92
12/4/2020 13:46	196.1	194.2	190.3	2282	65.08
12/4/2020 13:46	196.1	193.6	190	2284	64.92
12/4/2020 13:46	196.1	194.2	190.2	2285	65.04
12/4/2020 13:46	196.1	194.1	190.2	2286	64.96
12/4/2020 13:46	196.1	193.9	190.1	2287	64.92
12/4/2020 13:46	196.1	193.7	190.1	2288	64.88
12/4/2020 13:46	196.1	193.6	189.9	2291	64.96
12/4/2020 13:46	196	194.2	190.1	2297	64.99
12/4/2020 13:46	196	194	190	2297	65.08
12/4/2020 13:46	196	194.2	190	2297	65.04
12/4/2020 13:46	196	194	190.1	2295	64.92
12/4/2020 13:46	196	193.8	190.3	2292	64.97
12/4/2020 13:46	195.9	194.1	190.6	2288	65.08
12/4/2020 13:46	195.9	193.8	190.7	2286	64.88
12/4/2020 13:46	195.9	194.3	190.8	2283	65.04
12/4/2020 13:46	195.9	194.1	190.9	2287	65.01
12/4/2020 13:46	195.9	193.7	190.8	2282	64.92
12/4/2020 13:46	195.9	193.9	190.2	2285	65
12/4/2020 13:46	195.8	193.8	189.8	2282	64.96
12/4/2020 13:46	195.9	194	190	2283	64.92
12/4/2020 13:46	195.8	194	189.8	2284	65
12/4/2020 13:46	195.8	193.6	189.9	2283	65.05
12/4/2020 13:46	195.7	194.1	190	2283	64.88
12/4/2020 13:46	195.8	193.8	190.1	2285	65
12/4/2020 13:46	195.8	194	190.1	2284	64.92
12/4/2020 13:46	195.7	194.2	190.1	2283	65
12/4/2020 13:46	195.7	194	190	2283	64.92
12/4/2020 13:46	195.7	194	190	2287	65.21
12/4/2020 13:46	195.6	193.7	190.2	2287	64.88
12/4/2020 13:46	195.6	194.2	190.1	2285	64.88
12/4/2020 13:46	195.7	194.1	190	2292	64.88
12/4/2020 13:46	195.6	193.7	189.9	2289	64.96
12/4/2020 13:46	195.5	194	189.7	2287	64.93
12/4/2020 13:46	195.4	193.7	189.9	2288	64.93
12/4/2020 13:46	195.4	194.1	190	2288	65.08
12/4/2020 13:46	195.4	194.2	189.8	2283	65
12/4/2020 13:46	195.3	193.8	189.8	2284	64.88
12/4/2020 13:46	195.3	194.1	189.9	2289	65.09
12/4/2020 13:46	195.2	193.6	189.8	2292	65
12/4/2020 13:46	195.2	193.8	190.1	2290	65.05

12/4/2020 13:46	195.2	194	190.4	2288	64.96
12/4/2020 13:46	195.1	193.8	190.8	2284	64.96
12/4/2020 13:46	195.1	194.3	191	2288	64.96
12/4/2020 13:46	195	194.1	190.9	2282	65.09
12/4/2020 13:46	195	193.6	190.3	2279	64.96
12/4/2020 13:46	195	193.8	189.9	2275	65
12/4/2020 13:46	194.9	193.8	190	2277	65
12/4/2020 13:46	194.8	194.2	190.2	2280	64.88
12/4/2020 13:46	194.7	194.2	190.1	2279	65.08
12/4/2020 13:46	194.7	193.6	190.2	2280	64.93
12/4/2020 13:46	194.6	193.9	190.2	2282	64.96
12/4/2020 13:46	194.6	194	189.8	2282	64.83
12/4/2020 13:46	194.6	194.1	189.9	2285	65.2
12/4/2020 13:46	194.5	194.2	190.1	2290	64.88
12/4/2020 13:46	194.4	193.8	190.7	2287	65.08
12/4/2020 13:47	194.3	194.3	191	2288	65.12
12/4/2020 13:47	194.3	193.6	190.7	2284	65
12/4/2020 13:47	194.3	193.8	190.2	2285	65.04
12/4/2020 13:47	194.2	194.1	190	2289	65.08
12/4/2020 13:47	194.1	193.7	190.1	2286	64.99
12/4/2020 13:47	194.1	194.2	190	2292	65.04
12/4/2020 13:47	194	193.8	190.2	2287	65
12/4/2020 13:47	194	193.8	190.1	2283	64.92
12/4/2020 13:47	193.9	194	190.1	2282	65
12/4/2020 13:47	193.8	194.1	190	2288	65.09
12/4/2020 13:47	193.7	194.1	189.9	2289	64.96
12/4/2020 13:47	193.7	194	190	2291	64.96
12/4/2020 13:47	193.6	193.7	189.8	2289	65
12/4/2020 13:47	193.6	194.2	189.7	2288	65.04
12/4/2020 13:47	193.5	193.7	190.3	2284	65.04
12/4/2020 13:47	193.5	194.4	190.7	2282	64.96
12/4/2020 13:47	193.4	194	190.8	2278	64.95
12/4/2020 13:47	193.4	193.9	190.4	2279	65.04
12/4/2020 13:47	193.3	194.2	189.9	2283	65.12
12/4/2020 13:47	193.3	193.5	189.7	2283	65.04
12/4/2020 13:47	193.2	194.2	190.1	2283	64.99
12/4/2020 13:47	193.1	193.9	190.1	2285	65.12
12/4/2020 13:47	193.1	194.3	190.1	2283	65.08
12/4/2020 13:47	193	193.9	189.9	2286	64.97
12/4/2020 13:47	193	193.6	189.8	2288	64.93
12/4/2020 13:47	192.9	194.2	189.7	2283	64.96
12/4/2020 13:47	192.9	193.9	190.3	2286	65
12/4/2020 13:47	192.9	193.8	190.9	2289	64.96
12/4/2020 13:47	192.9	194.3	190.3	2289	65.08
12/4/2020 13:47	192.8	193.6	189.8	2281	65.04
12/4/2020 13:47	192.8	194.2	189.8	2286	64.96
12/4/2020 13:47	192.7	193.9	189.9	2286	65

12/4/2020 13:47	192.6	193.6	189.9	2282	64.92
12/4/2020 13:47	192.6	193.7	190	2286	65.08
12/4/2020 13:47	192.6	193.5	189.6	2284	64.96
12/4/2020 13:47	192.4	194.1	189.5	2289	65
12/4/2020 13:47	192.5	193.8	190	2292	65
12/4/2020 13:47	192.5	194.3	190.6	2286	65.08
12/4/2020 13:47	192.4	194.2	190.6	2290	65.04
12/4/2020 13:47	192.3	193.6	190.2	2290	64.92
12/4/2020 13:47	192.3	194.2	189.8	2286	65.08
12/4/2020 13:47	192.3	194.2	189.7	2279	64.83
12/4/2020 13:47	192.2	194	189.8	2283	64.92
12/4/2020 13:47	192.2	193.9	189.8	2283	64.97
12/4/2020 13:47	192.1	193.7	189.8	2277	64.84
12/4/2020 13:47	192.1	194.2	190	2275	64.84
12/4/2020 13:47	192.1	193.6	190	2276	64.96
12/4/2020 13:47	192	194.2	189.9	2281	64.88
12/4/2020 13:47	192	194.3	190	2281	64.92
12/4/2020 13:47	192	193.7	190	2288	64.96
12/4/2020 13:47	191.9	194.2	189.8	2293	64.92
12/4/2020 13:47	191.9	193.6	189.7	2291	64.92
12/4/2020 13:47	191.8	193.6	189.6	2293	65
12/4/2020 13:47	191.8	194.2	189.7	2294	64.88
12/4/2020 13:47	191.8	194	190.2	2289	64.96
12/4/2020 13:47	191.7	194.2	190.7	2292	65.05
12/4/2020 13:47	191.6	194	190.3	2287	64.96
12/4/2020 13:47	191.6	193.9	189.6	2283	64.96
12/4/2020 13:47	191.6	194.2	189.5	2288	65.05
12/4/2020 13:48	191.5	193.9	189.5	2292	65
12/4/2020 13:48	191.5	194.2	189.6	2289	65
12/4/2020 13:48	191.4	193.1	189.8	2290	65.08
12/4/2020 13:48	191.3	193.2	189.5	2289	64.88
12/4/2020 13:48	191.4	193.2	189.8	2289	65.08
12/4/2020 13:48	191.3	193.2	190.4	2290	64.96
12/4/2020 13:48	191.3	193.1	189.6	2289	65.04
12/4/2020 13:48	191.2	193.1	189.3	2289	64.92
12/4/2020 13:48	191.1	193.1	189.8	2292	64.92
12/4/2020 13:48	191.1	193.3	189.9	2292	65.04
12/4/2020 13:48	191.1	193.1	189.5	2291	65.12
12/4/2020 13:48	190.9	193.2	190.3	2293	65.08
12/4/2020 13:48	190.9	193	190.1	2281	64.92
12/4/2020 13:48	190.9	193	189.3	2270	65.08
12/4/2020 13:48	190.9	193.2	189.6	2260	65
12/4/2020 13:48	190.9	193.1	189.7	2253	64.99
12/4/2020 13:48	190.8	193.3	189.9	2250	65
12/4/2020 13:48	190.8	193.1	189.5	2250	65
12/4/2020 13:48	190.7	192.9	189.9	2254	64.87
12/4/2020 13:48	190.8	193.3	190.3	2268	65.04

12/4/2020 13:48	190.7	192.9	189.7	2268	65.04
12/4/2020 13:48	190.6	193.3	189.2	2272	64.91
12/4/2020 13:48	190.6	192.9	189.4	2274	65
12/4/2020 13:48	190.6	193.3	189.6	2274	65
12/4/2020 13:48	190.6	193.3	189.6	2276	64.92
12/4/2020 13:48	190.5	193.1	189.6	2277	64.96
12/4/2020 13:48	190.6	193.1	189.4	2276	64.96
12/4/2020 13:48	190.5	193	189.8	2278	64.96
12/4/2020 13:48	190.5	193.1	190.3	2275	65
12/4/2020 13:48	190.5	193.2	190.1	2274	65.04
12/4/2020 13:48	190.5	192.9	189.5	2274	64.92
12/4/2020 13:48	190.5	193.2	189.2	2277	64.92
12/4/2020 13:48	190.4	193	189.2	2279	64.92
12/4/2020 13:48	190.5	193	189.3	2282	65.04
12/4/2020 13:48	190.4	193.3	189.5	2280	64.84
12/4/2020 13:48	190.4	193.1	189.6	2284	64.92
12/4/2020 13:48	190.4	193.2	189.6	2289	65.12
12/4/2020 13:48	190.5	193.1	189.7	2294	64.88
12/4/2020 13:48	190.4	193.2	189.8	2292	65.04
12/4/2020 13:48	190.4	193.2	189.5	2293	65.08
12/4/2020 13:48	190.4	193	189.6	2292	64.95
12/4/2020 13:48	190.4	193.3	189.6	2292	64.99
12/4/2020 13:48	190.4	193	189.6	2288	64.96
12/4/2020 13:48	190.4	193.1	189.7	2283	65.03
12/4/2020 13:48	190.4	193.2	189.6	2279	64.84
12/4/2020 13:48	190.3	193.2	189.8	2276	65.12
12/4/2020 13:48	190.4	193.2	189.7	2279	64.92
12/4/2020 13:48	190.4	193.2	189.5	2274	64.88
12/4/2020 13:48	190.4	193.2	189.5	2275	65.04
12/4/2020 13:48	190.4	193.1	189.5	2271	64.92
12/4/2020 13:48	190.3	193	189.5	2268	64.88
12/4/2020 13:48	190.3	193.2	189.5	2271	64.92
12/4/2020 13:48	190.3	193.2	189.5	2274	64.92
12/4/2020 13:48	190.3	193.3	189.4	2274	64.92
12/4/2020 13:48	190.3	193.1	189.3	2275	65.04
12/4/2020 13:48	190.3	193.1	189.3	2274	64.92
12/4/2020 13:48	190.3	193.1	189.3	2274	64.92
12/4/2020 13:48	190.3	193.1	189.5	2278	65
12/4/2020 13:48	190.3	193	189.1	2272	64.96
12/4/2020 13:48	190.2	193.1	189.1	2273	64.96
12/4/2020 13:49	190.2	193.1	189.3	2278	65.04
12/4/2020 13:49	190.1	193.1	189.3	2279	65
12/4/2020 13:49	190.1	193.1	189.5	2275	64.92
12/4/2020 13:49	190	193	189.7	2275	64.96
12/4/2020 13:49	190.1	193.2	189.9	2272	64.96
12/4/2020 13:49	190	193.1	190	2268	64.92
12/4/2020 13:49	190	193.1	190.1	2270	65.08

12/4/2020 13:49	190	193.2	190.2	2269	64.96
12/4/2020 13:49	190	192.3	190.2	2266	65.08
12/4/2020 13:49	189.9	192.2	190.1	2267	64.92
12/4/2020 13:49	189.9	192.2	189.6	2267	64.92
12/4/2020 13:49	189.9	192.1	189.4	2269	64.96
12/4/2020 13:49	189.8	192.1	189.1	2266	65.08
12/4/2020 13:49	189.8	192.3	188.9	2270	64.88
12/4/2020 13:49	189.8	191.8	188.9	2269	64.99
12/4/2020 13:49	189.8	192.1	189.1	2271	65.04
12/4/2020 13:49	189.8	191.9	189.2	2275	64.92
12/4/2020 13:49	189.7	191.9	189.3	2271	64.99
12/4/2020 13:49	189.8	191.7	189.4	2270	65.04
12/4/2020 13:49	189.8	192.2	189.4	2271	64.88
12/4/2020 13:49	189.7	192.2	189.2	2273	65.03
12/4/2020 13:49	189.7	192.1	189.2	2272	65.04
12/4/2020 13:49	189.7	192.1	189	2274	64.88
12/4/2020 13:49	189.8	192.3	189.3	2278	64.91
12/4/2020 13:49	189.7	192.1	189.8	2275	64.88
12/4/2020 13:49	189.7	192.2	190	2275	65
12/4/2020 13:49	189.7	192	189.3	2273	64.99
12/4/2020 13:49	189.7	191.9	188.9	2270	64.88
12/4/2020 13:49	189.8	192.2	189.1	2273	65.08
12/4/2020 13:49	189.8	192.1	189.3	2268	64.87
12/4/2020 13:49	189.8	192	189.5	2267	64.92
12/4/2020 13:49	189.8	192	189.3	2267	64.96
12/4/2020 13:49	189.8	191.9	189.2	2271	64.88
12/4/2020 13:49	189.8	192.2	189.8	2268	64.84
12/4/2020 13:49	189.9	192.2	189.6	2269	65.08
12/4/2020 13:49	189.9	192.2	188.8	2268	65.03
12/4/2020 13:49	190	192	189.2	2269	64.92
12/4/2020 13:49	190	192.1	189.4	2269	64.88
12/4/2020 13:49	190	192.3	189.2	2268	64.95
12/4/2020 13:49	190.1	192.1	189.1	2269	65.04
12/4/2020 13:49	190.2	192.2	190	2268	65.08
12/4/2020 13:49	190.1	192.2	189.9	2270	65
12/4/2020 13:49	190.3	192	189.1	2271	64.83
12/4/2020 13:49	190.3	192	188.9	2271	64.96
12/4/2020 13:49	190.4	192	188.9	2272	65
12/4/2020 13:49	190.5	192.1	189.3	2267	64.96
12/4/2020 13:49	190.5	192.2	189.2	2263	64.88
12/4/2020 13:49	190.5	192.4	189.2	2268	64.96
12/4/2020 13:49	190.6	191.9	189.3	2268	64.96
12/4/2020 13:49	190.7	192	189.1	2267	64.87
12/4/2020 13:49	190.8	192.2	189.1	2265	64.96
12/4/2020 13:49	190.8	192	189	2264	64.83
12/4/2020 13:49	190.9	192.1	189.5	2267	64.83
12/4/2020 13:49	191	192.1	189.8	2276	64.96

12/4/2020 13:49	191	191.9	190.1	2274	64.92
12/4/2020 13:49	191.2	192.3	190.1	2274	65
12/4/2020 13:49	191.1	192.1	189.8	2276	64.96
12/4/2020 13:49	191.3	192.1	189.3	2275	64.87
12/4/2020 13:49	191.3	192.4	189.1	2272	64.99
12/4/2020 13:50	191.4	192.1	189.1	2273	65
12/4/2020 13:50	191.6	192	189.1	2271	64.96
12/4/2020 13:50	191.6	192.1	189.1	2271	64.87
12/4/2020 13:50	191.8	191.8	189.3	2273	64.96
12/4/2020 13:50	191.9	192.2	189.4	2271	65
12/4/2020 13:50	191.8	192.1	189.5	2272	64.92
12/4/2020 13:50	192	192.2	189.5	2271	65.04
12/4/2020 13:50	192.1	192.2	189.5	2275	64.96
12/4/2020 13:50	192.2	192.4	189.6	2270	65.04
12/4/2020 13:50	192.2	192.3	189.5	2270	64.96
12/4/2020 13:50	192.3	191.9	189.5	2269	64.96
12/4/2020 13:50	192.3	192.1	189.5	2269	64.92
12/4/2020 13:50	192.4	192.2	189.3	2271	64.96
12/4/2020 13:50	192.4	191.9	189.5	2271	64.96
12/4/2020 13:50	192.5	191.9	189.3	2276	64.96
12/4/2020 13:50	192.6	192.1	189.3	2274	64.96
12/4/2020 13:50	192.7	192	189.4	2271	64.96
12/4/2020 13:50	192.8	192.2	189.5	2272	64.87
12/4/2020 13:50	192.8	191.9	189.7	2268	64.91
12/4/2020 13:50	192.9	192.1	190	2270	65.04
12/4/2020 13:50	192.9	191.8	190.2	2268	64.83
12/4/2020 13:50	192.9	192.2	190.3	2269	64.91
12/4/2020 13:50	193	192.1	190.3	2278	64.96
12/4/2020 13:50	193.1	191.8	190.3	2276	64.87
12/4/2020 13:50	193.1	192.2	190.2	2275	64.99
12/4/2020 13:50	193.1	191.9	190.3	2277	65.04
12/4/2020 13:50	193.2	192.1	190.1	2271	64.87
12/4/2020 13:50	193.3	192.3	190.2	2272	64.87
12/4/2020 13:50	193.3	192.2	190	2271	64.88
12/4/2020 13:50	193.4	192.1	190.2	2264	64.91
12/4/2020 13:50	193.4	191.8	190.2	2262	64.92
12/4/2020 13:50	193.5	191.9	190.3	2268	64.92
12/4/2020 13:50	193.5	191.9	190.3	2270	64.88
12/4/2020 13:50	193.5	191.8	190.3	2270	64.96
12/4/2020 13:50	193.5	192.2	190.4	2271	64.96
12/4/2020 13:50	193.6	192	190.5	2267	64.92
12/4/2020 13:50	193.6	191.9	190.1	2269	65.04
12/4/2020 13:50	193.7	192.2	190.1	2270	64.93
12/4/2020 13:50	193.7	191.9	189.7	2272	64.79
12/4/2020 13:50	193.6	192.1	189.3	2274	64.99
12/4/2020 13:50	193.7	192.1	189.5	2275	64.88
12/4/2020 13:50	193.7	192.1	189.5	2277	64.88

12/4/2020 13:50	193.7	192.2	189.8	2272	65
12/4/2020 13:50	193.7	191.9	189.8	2275	65.08
12/4/2020 13:50	193.8	192.1	189.8	2275	64.92
12/4/2020 13:50	193.8	192.2	189.5	2275	65.04
12/4/2020 13:50	193.8	191.9	189.4	2279	64.92
12/4/2020 13:50	193.9	192.3	189.5	2275	64.99
12/4/2020 13:50	193.9	191.9	190.3	2275	65.04
12/4/2020 13:50	193.9	192.2	190.7	2277	65.08
12/4/2020 13:50	193.9	192.1	189.8	2276	65.04
12/4/2020 13:50	193.9	192	189.5	2275	64.92
12/4/2020 13:50	194	192.2	189.5	2276	65.04
12/4/2020 13:50	194	191.9	189.9	2275	65.04
12/4/2020 13:50	194.1	192.1	189.6	2279	64.96
12/4/2020 13:50	194	192.2	189.6	2282	64.88
12/4/2020 13:50	194.1	191.9	189.5	2279	64.92
12/4/2020 13:50	194.1	192.4	189.5	2279	64.88
12/4/2020 13:50	194.1	192.2	190.1	2275	65.04
12/4/2020 13:50	194.1	192.2	190.5	2277	65
12/4/2020 13:51	194.2	192.2	190.1	2277	65.04
12/4/2020 13:51	194.2	192	189.5	2279	65.08
12/4/2020 13:51	194.2	192.3	189.8	2276	64.92
12/4/2020 13:51	194.2	192.1	189.8	2274	64.92
12/4/2020 13:51	194.2	192.2	190	2275	64.92
12/4/2020 13:51	194.2	192.2	189.6	2278	64.96
12/4/2020 13:51	194.2	192.1	189.3	2275	64.96
12/4/2020 13:51	194.2	192.4	189.3	2270	64.96
12/4/2020 13:51	194.2	192	189.8	2278	65.05
12/4/2020 13:51	194.2	192.2	190.3	2281	64.96
12/4/2020 13:51	194.2	192.1	190.5	2280	64.92
12/4/2020 13:51	194.2	191.8	189.8	2282	65
12/4/2020 13:51	194.3	192.2	189.6	2285	64.87
12/4/2020 13:51	194.2	191.9	189.5	2285	64.96
12/4/2020 13:51	194.2	192.2	189.8	2289	65
12/4/2020 13:51	194.2	191.8	189.9	2291	64.96
12/4/2020 13:51	194.3	191.7	189.9	2292	64.99
12/4/2020 13:51	194.2	192.4	189.9	2290	65.08
12/4/2020 13:51	194.2	191.9	190	2289	64.95
12/4/2020 13:51	194.3	192.1	189.9	2288	64.92
12/4/2020 13:51	194.3	192	190	2287	65.08
12/4/2020 13:51	194.3	192	189.8	2285	64.92
12/4/2020 13:51	194.3	192.3	190	2283	65.04
12/4/2020 13:51	194.3	192.1	190	2278	64.96
12/4/2020 13:51	194.3	192.2	189.9	2276	64.8
12/4/2020 13:51	194.3	192	190	2276	64.92
12/4/2020 13:51	194.3	191.9	189.9	2283	64.93
12/4/2020 13:51	194.3	191.8	190	2285	65.12
12/4/2020 13:51	194.3	192	190	2288	64.88

12/4/2020 13:51	194.3	192.2	189.9	2287	64.96
12/4/2020 13:51	194.3	192.2	189.9	2289	65.08
12/4/2020 13:51	194.3	191.9	190	2286	64.93
12/4/2020 13:51	194.3	192	190	2286	65.08
12/4/2020 13:51	194.4	192	190	2286	64.88
12/4/2020 13:51	194.4	191.9	190.1	2283	65.04
12/4/2020 13:51	194.4	191.9	189.8	2284	65
12/4/2020 13:51	194.4	191.8	190.1	2288	65.04
12/4/2020 13:51	194.3	192.2	189.9	2285	64.92
12/4/2020 13:51	194.4	191.9	189.6	2285	64.96
12/4/2020 13:51	194.3	192	190.1	2284	65
12/4/2020 13:51	194.4	192	189.9	2283	64.96
12/4/2020 13:51	194.3	192.2	190.1	2283	64.96
12/4/2020 13:51	194.4	192.2	190	2286	64.96
12/4/2020 13:51	194.4	192	189.9	2286	64.92
12/4/2020 13:51	194.4	192.1	190	2286	64.99
12/4/2020 13:51	194.4	192.2	190	2286	65.05
12/4/2020 13:51	194.3	192	189.8	2286	64.99
12/4/2020 13:51	194.4	192.1	189.7	2285	64.96
12/4/2020 13:51	194.4	192.1	189.8	2287	65.08
12/4/2020 13:51	194.4	192.2	189.6	2288	65
12/4/2020 13:51	194.4	192.3	189.6	2286	65
12/4/2020 13:51	194.4	192	189.5	2289	64.92
12/4/2020 13:51	194.3	192.3	189.6	2281	64.88
12/4/2020 13:51	194.3	192.2	189.8	2283	64.92
12/4/2020 13:51	194.4	192.1	190.2	2283	65.12
12/4/2020 13:51	194.3	192.4	190.6	2285	65.08
12/4/2020 13:51	194.4	191.8	190.6	2283	64.96
12/4/2020 13:51	194.4	192.1	190.2	2288	65.12
12/4/2020 13:51	194.3	192	189.8	2288	65.04
12/4/2020 13:52	194.3	192.4	189.7	2290	65.08
12/4/2020 13:52	194.3	192.2	189.9	2287	64.96
12/4/2020 13:52	194.2	191.7	190	2287	65.04
12/4/2020 13:52	194.3	192.1	189.7	2286	65.04
12/4/2020 13:52	194.2	192.1	189.8	2289	64.96
12/4/2020 13:52	194.2	192.1	189.7	2290	64.96
12/4/2020 13:52	194.2	191.9	190.7	2291	64.83
12/4/2020 13:52	194.2	192.1	190.3	2293	64.92
12/4/2020 13:52	194.1	192.2	189.6	2289	65
12/4/2020 13:52	194.2	192.1	189.8	2288	64.96
12/4/2020 13:52	194.1	192.1	189.8	2288	64.96
12/4/2020 13:52	194.1	192	189.9	2283	65
12/4/2020 13:52	194.1	192.1	189.6	2286	65
12/4/2020 13:52	194.1	192.2	189.5	2289	64.88
12/4/2020 13:52	194	191.9	190.4	2291	65.09
12/4/2020 13:52	194.1	191.7	190.7	2283	64.96
12/4/2020 13:52	194	192.1	189.9	2282	65

12/4/2020 13:52	194	191.9	189.8	2278	64.96
12/4/2020 13:52	194	192.3	189.9	2278	65
12/4/2020 13:52	193.9	192.1	190	2274	64.96
12/4/2020 13:52	193.9	191.8	190	2274	64.88
12/4/2020 13:52	193.9	193.1	189.8	2274	64.84
12/4/2020 13:52	193.9	192.1	189.6	2282	64.92
12/4/2020 13:52	193.9	192.2	189.6	2285	65
12/4/2020 13:52	193.8	192.1	189.8	2285	65
12/4/2020 13:52	193.8	192.3	190.6	2284	64.88
12/4/2020 13:52	193.8	192	190.7	2289	65.04
12/4/2020 13:52	193.8	191.8	190.1	2289	64.96
12/4/2020 13:52	193.7	192.2	189.7	2291	64.92
12/4/2020 13:52	193.7	191.9	189.7	2296	65.12
12/4/2020 13:52	193.7	193.1	190	2289	64.99
12/4/2020 13:52	193.7	192.9	190.1	2292	65.08
12/4/2020 13:52	193.7	193	189.9	2293	65.12
12/4/2020 13:52	193.7	193.1	189.7	2289	64.91
12/4/2020 13:52	193.6	193	189.6	2288	64.92
12/4/2020 13:52	193.6	192.3	189.6	2286	65.04
12/4/2020 13:52	193.6	192	189.6	2286	64.96
12/4/2020 13:52	193.6	192	190.4	2285	65
12/4/2020 13:52	193.6	192	190.6	2287	65.09
12/4/2020 13:52	193.6	192.9	190.7	2289	65
12/4/2020 13:52	193.5	193.2	190.2	2289	64.96
12/4/2020 13:52	193.5	193.1	189.8	2290	65.08
12/4/2020 13:52	193.5	193.3	189.8	2290	64.88
12/4/2020 13:52	193.5	191.9	189.5	2292	65.04
12/4/2020 13:52	193.5	192.2	189.7	2292	65.08
12/4/2020 13:52	193.5	193	189.8	2286	65.04
12/4/2020 13:52	193.5	193.1	190.1	2287	65.08
12/4/2020 13:52	193.5	191.8	189.9	2284	64.96
12/4/2020 13:52	193.5	192.1	189.7	2285	65.04
12/4/2020 13:52	193.4	191.8	189.8	2282	65.04
12/4/2020 13:52	193.4	192.1	189.5	2284	65.04
12/4/2020 13:52	193.4	192.2	189.4	2287	65
12/4/2020 13:52	193.4	192	189.5	2285	65
12/4/2020 13:52	193.4	193.3	189.4	2286	64.93
12/4/2020 13:52	193.3	193.1	189.4	2288	65
12/4/2020 13:52	193.3	193.1	189.3	2288	64.84
12/4/2020 13:52	193.3	192.2	189.3	2288	64.99
12/4/2020 13:52	193.3	192.1	189.6	2289	65.04
12/4/2020 13:52	193.3	192.1	189.8	2287	64.96
12/4/2020 13:53	193.3	191.8	189.8	2290	65.04
12/4/2020 13:53	193.3	192.1	189.6	2290	65
12/4/2020 13:53	193.3	192.1	189.5	2285	64.92
12/4/2020 13:53	193.2	192	189.4	2286	64.96
12/4/2020 13:53	193.3	192	189.5	2289	65.04

12/4/2020 13:53	193.2	192.2	189.3	2289	65
12/4/2020 13:53	193.2	192	189.3	2286	65
12/4/2020 13:53	193.1	192.2	189.3	2282	64.88
12/4/2020 13:53	193.1	191.9	189.5	2286	64.88
12/4/2020 13:53	193.1	192.1	189.8	2287	65.04
12/4/2020 13:53	193.1	192.4	189.7	2286	65.01
12/4/2020 13:53	193.1	192.2	190.3	2286	64.99
12/4/2020 13:53	193	192.2	189.8	2288	65
12/4/2020 13:53	193.1	192.3	189.5	2292	64.96
12/4/2020 13:53	192.9	191.9	189.6	2293	65.04
12/4/2020 13:53	193	191.9	190.4	2292	65.01
12/4/2020 13:53	193	191.9	190.2	2292	64.96
12/4/2020 13:53	192.9	192.4	189.4	2288	64.88
12/4/2020 13:53	192.9	191.9	189.5	2289	65.08
12/4/2020 13:53	192.9	192	190	2286	65
12/4/2020 13:53	192.9	192.2	189.8	2281	65.04
12/4/2020 13:53	192.8	191.7	189.5	2283	64.99
12/4/2020 13:53	192.8	192.1	190.5	2283	65
12/4/2020 13:53	192.8	192.1	190	2280	64.96
12/4/2020 13:53	192.8	192.1	189.3	2275	64.96
12/4/2020 13:53	192.8	192.2	189.8	2279	65.04
12/4/2020 13:53	192.7	192.2	189.8	2280	64.91
12/4/2020 13:53	192.7	192.3	189.6	2282	65.09
12/4/2020 13:53	192.6	191.9	190.1	2286	65.04
12/4/2020 13:53	192.6	192.1	190.3	2282	64.91
12/4/2020 13:53	192.6	192.1	189.6	2280	64.96
12/4/2020 13:53	192.6	192.1	189.3	2282	64.96
12/4/2020 13:53	192.6	192.2	189.5	2283	65
12/4/2020 13:53	192.5	191.7	189.7	2286	64.92
12/4/2020 13:53	192.5	192.4	189.5	2288	65
12/4/2020 13:53	192.5	192	189.4	2288	65.08
12/4/2020 13:53	192.5	191.8	189.7	2286	64.96
12/4/2020 13:53	192.5	192.1	190.3	2287	65
12/4/2020 13:53	192.4	191.9	190.3	2289	65
12/4/2020 13:53	192.4	191.9	190	2289	65.04
12/4/2020 13:53	192.4	192.1	189.4	2286	64.92
12/4/2020 13:53	192.3	192	189.4	2284	64.96
12/4/2020 13:53	192.4	192.1	189.3	2286	64.88
12/4/2020 13:53	192.4	192.2	189.3	2286	65.04
12/4/2020 13:53	192.2	192.1	189.4	2284	65.08
12/4/2020 13:53	192.3	192.4	189.3	2284	64.92
12/4/2020 13:53	192.3	192	189.4	2283	65.04
12/4/2020 13:53	192.3	192.1	189.2	2285	64.96
12/4/2020 13:53	192.2	192	189.3	2281	64.88
12/4/2020 13:53	192.2	192.1	189.5	2283	65
12/4/2020 13:53	192.2	192.2	190	2288	65.09
12/4/2020 13:53	192.2	191.9	190.4	2282	64.96

12/4/2020 13:53	192.2	192	189.8	2282	64.96
12/4/2020 13:53	192.2	192.1	189.4	2288	65.09
12/4/2020 13:53	192.2	192.2	189.7	2283	65
12/4/2020 13:53	192.1	192.1	189.5	2284	65.09
12/4/2020 13:53	192.1	192	189.3	2286	65.08
12/4/2020 13:53	192.1	192.1	189.3	2285	64.95
12/4/2020 13:53	192.1	191.7	190.1	2281	64.96
12/4/2020 13:53	192	192	189.6	2282	65.16
12/4/2020 13:54	192.1	192.3	189.3	2281	64.92
12/4/2020 13:54	192	191.9	189.5	2279	64.88
12/4/2020 13:54	192	192.2	189.4	2279	65.04
12/4/2020 13:54	192	192.1	189.3	2283	64.88
12/4/2020 13:54	192	192.1	189	2285	64.88
12/4/2020 13:54	191.9	192.2	189.6	2286	65.08
12/4/2020 13:54	191.9	191.9	190.2	2283	64.96
12/4/2020 13:54	191.9	192.2	189.3	2285	64.99
12/4/2020 13:54	191.9	192.1	189.5	2282	65.04
12/4/2020 13:54	191.9	192.1	189.5	2280	65.01
12/4/2020 13:54	191.9	192.2	189.4	2282	64.99
12/4/2020 13:54	191.8	192	189.2	2280	64.92
12/4/2020 13:54	191.8	192.1	189.2	2282	65.05
12/4/2020 13:54	191.8	191.9	190.2	2283	64.87
12/4/2020 13:54	191.8	191.9	190	2283	64.96
12/4/2020 13:54	191.8	192	189.3	2285	65
12/4/2020 13:54	191.8	192	189.4	2283	65
12/4/2020 13:54	191.7	192.1	189.5	2283	64.92
12/4/2020 13:54	191.7	192.2	189.5	2283	65
12/4/2020 13:54	191.7	192.1	189	2282	65
12/4/2020 13:54	191.6	192.2	189.1	2284	64.96
12/4/2020 13:54	191.6	191.9	190.1	2289	65.05
12/4/2020 13:54	191.6	192.3	189.3	2289	64.96
12/4/2020 13:54	191.5	191.9	189	2288	65
12/4/2020 13:54	191.6	191.9	189.5	2286	64.96
12/4/2020 13:54	191.6	192.2	189.3	2283	64.8
12/4/2020 13:54	191.5	191.9	189.3	2282	65.04
12/4/2020 13:54	191.5	192.3	188.9	2282	65.04
12/4/2020 13:54	191.4	192.2	189.2	2283	64.99
12/4/2020 13:54	191.5	191.9	190	2283	64.88
12/4/2020 13:54	191.4	192.4	190	2286	65.04
12/4/2020 13:54	191.4	192.1	189.1	2282	64.88
12/4/2020 13:54	191.4	192.2	189.3	2281	65.12
12/4/2020 13:54	191.3	192.2	189.4	2284	65.08
12/4/2020 13:54	191.3	192.1	189.3	2287	64.92
12/4/2020 13:54	191.3	192.3	189.1	2286	65.04
12/4/2020 13:54	191.3	192	188.9	2284	65.12
12/4/2020 13:54	191.3	192.1	189.4	2284	64.88
12/4/2020 13:54	191.2	192.1	190.2	2278	65.12

12/4/2020 13:54	191.3	192	189.6	2279	64.96
12/4/2020 13:54	191.2	191.9	189.1	2277	64.91
12/4/2020 13:54	191.2	192	189.3	2276	64.92
12/4/2020 13:54	191.2	192.1	189.3	2272	65.04
12/4/2020 13:54	191.1	192.1	189.1	2272	65
12/4/2020 13:54	191.1	191.9	189	2275	65
12/4/2020 13:54	191.2	192.2	189.1	2279	65
12/4/2020 13:54	191.1	191.9	189.9	2279	64.92
12/4/2020 13:54	191.1	192.2	189.3	2280	64.96
12/4/2020 13:54	191.1	192.2	188.8	2281	65.05
12/4/2020 13:54	191.1	192.3	189.1	2286	64.96
12/4/2020 13:54	191.1	192	189.3	2286	65.04
12/4/2020 13:54	191.1	191.8	189.3	2288	64.96
12/4/2020 13:54	191	192.1	188.9	2288	65.04
12/4/2020 13:54	191.1	192.2	188.8	2283	64.92
12/4/2020 13:54	191.1	192.2	189.2	2282	65.04
12/4/2020 13:54	191	192.1	189.8	2284	65.12
12/4/2020 13:54	191	191.8	189.9	2278	64.92
12/4/2020 13:54	191	192.1	189.2	2278	65.04
12/4/2020 13:54	190.9	191.9	188.9	2279	64.88
12/4/2020 13:55	190.9	191.9	189.3	2281	65.08
12/4/2020 13:55	190.9	192.2	189.3	2278	65.04
12/4/2020 13:55	190.9	192.1	189.3	2278	64.93
12/4/2020 13:55	190.9	192.2	189.1	2280	65.04
12/4/2020 13:55	190.9	192.2	189	2282	64.92
12/4/2020 13:55	190.9	191.9	188.9	2281	65.01
12/4/2020 13:55	190.8	192.2	189.8	2275	64.88
12/4/2020 13:55	190.9	192	189.9	2273	65
12/4/2020 13:55	190.8	192.2	189	2272	65.09
12/4/2020 13:55	190.9	192	188.9	2271	64.96
12/4/2020 13:55	190.7	191.9	189	2268	64.96
12/4/2020 13:55	190.8	191.8	189.2	2271	65.04
12/4/2020 13:55	190.8	192.1	189.1	2272	64.92
12/4/2020 13:55	190.7	192	188.9	2274	64.96
12/4/2020 13:55	190.7	191.8	188.6	2276	65
12/4/2020 13:55	190.7	192.2	189.2	2276	64.92
12/4/2020 13:55	190.7	192.2	189.7	2280	64.96
12/4/2020 13:55	190.7	191.8	189.8	2283	64.96
12/4/2020 13:55	190.7	192.2	189	2282	64.99
12/4/2020 13:55	190.7	192.2	188.8	2281	65.04
12/4/2020 13:55	190.6	191.8	189	2279	65.08
12/4/2020 13:55	190.6	192.2	189.1	2281	64.84
12/4/2020 13:55	190.6	192.1	189.1	2279	65.08
12/4/2020 13:55	190.5	192.3	188.9	2283	65.08
12/4/2020 13:55	190.5	192.3	188.9	2284	65.12
12/4/2020 13:55	190.5	192	188.6	2282	65.03
12/4/2020 13:55	190.5	192.2	188.8	2282	65.08

12/4/2020 13:55	190.5	192.1	189.3	2285	65.04
12/4/2020 13:55	190.5	192.3	189.6	2281	65.08
12/4/2020 13:55	190.5	192.2	189.5	2283	64.92
12/4/2020 13:55	190.5	191.7	188.9	2288	64.84
12/4/2020 13:55	190.4	192	188.8	2288	65.08
12/4/2020 13:55	190.4	191.9	188.9	2289	65.04
12/4/2020 13:55	190.4	192.4	189	2289	64.83
12/4/2020 13:55	190.4	192.4	188.6	2288	64.92
12/4/2020 13:55	190.3	191.8	188.5	2287	65
12/4/2020 13:55	190.3	192.1	189.3	2288	65
12/4/2020 13:55	190.3	192.1	189.6	2285	64.92
12/4/2020 13:55	190.3	192	188.8	2284	64.96
12/4/2020 13:55	190.3	192.4	188.7	2283	65
12/4/2020 13:55	190.2	192.1	189	2281	64.96
12/4/2020 13:55	190.2	192.1	188.9	2283	65
12/4/2020 13:55	190.2	192	188.6	2282	64.96
12/4/2020 13:55	190.1	191.9	188.5	2281	65
12/4/2020 13:55	190.1	192.2	188.8	2278	64.92
12/4/2020 13:55	190.1	191.4	189.6	2279	65.04
12/4/2020 13:55	190.1	190.9	189.2	2281	64.88
12/4/2020 13:55	190.1	190.9	188.6	2283	64.96
12/4/2020 13:55	190.1	190.9	188.8	2285	64.96
12/4/2020 13:55	190	191.2	188.9	2283	64.99
12/4/2020 13:55	190.1	190.9	188.9	2281	65
12/4/2020 13:55	190	191.5	188.8	2283	65.04
12/4/2020 13:55	190	190.8	188.5	2281	65.03
12/4/2020 13:55	190	191	189	2279	65.03
12/4/2020 13:55	190	190.6	189.5	2282	65.16
12/4/2020 13:55	190	190.8	189.4	2280	65.04
12/4/2020 13:55	190	191.6	188.8	2280	65.04
12/4/2020 13:55	190	191	188.5	2279	65.12
12/4/2020 13:55	190	191.4	188.7	2281	64.99
12/4/2020 13:55	190	190.7	188.7	2283	64.92
12/4/2020 13:56	189.9	190.7	188.8	2289	65.04
12/4/2020 13:56	189.9	191.5	188.9	2288	64.83
12/4/2020 13:56	189.9	190.9	188.8	2283	64.96
12/4/2020 13:56	189.9	191.4	188.9	2286	65.08
12/4/2020 13:56	189.8	190.9	188.8	2286	64.92
12/4/2020 13:56	189.9	190.7	188.8	2286	65.04
12/4/2020 13:56	189.9	190.9	188.8	2288	64.96
12/4/2020 13:56	189.9	190.8	188.9	2286	64.91
12/4/2020 13:56	189.8	191.1	188.8	2282	64.96
12/4/2020 13:56	189.9	191.4	188.8	2281	65
12/4/2020 13:56	189.8	191.2	188.5	2281	64.87
12/4/2020 13:56	189.8	190.9	188.6	2279	64.96
12/4/2020 13:56	189.8	190.7	188.8	2278	64.96
12/4/2020 13:56	189.8	190.9	189.5	2282	65.04

12/4/2020 13:56	189.8	191.3	188.9	2286	64.92
12/4/2020 13:56	189.7	190.9	188.2	2287	64.96
12/4/2020 13:56	189.8	191.4	188.5	2285	65
12/4/2020 13:56	189.8	190.9	188.6	2281	64.96
12/4/2020 13:56	189.8	191.4	188.5	2284	64.96
12/4/2020 13:56	189.8	190.8	188.7	2284	65
12/4/2020 13:56	189.7	190.8	189.3	2282	64.96
12/4/2020 13:56	189.7	191.2	188.4	2282	64.99
12/4/2020 13:56	189.7	190.9	188.2	2278	65.13
12/4/2020 13:56	189.7	191.5	188.7	2279	64.83
12/4/2020 13:56	189.7	190.8	188.6	2277	64.88
12/4/2020 13:56	189.7	190.7	188.4	2277	65
12/4/2020 13:56	189.6	191.2	188.4	2277	64.92
12/4/2020 13:56	189.6	190.8	189	2278	64.88
12/4/2020 13:56	189.6	191.4	189.2	2279	65.08
12/4/2020 13:56	189.6	191.2	188.7	2279	64.99
12/4/2020 13:56	189.6	190.6	188.4	2280	64.92
12/4/2020 13:56	189.6	191.3	188.2	2286	65.12
12/4/2020 13:56	189.5	190.8	188.6	2284	64.88
12/4/2020 13:56	189.5	191.2	188.7	2285	65.04
12/4/2020 13:56	189.5	191.4	188.6	2289	64.92
12/4/2020 13:56	189.5	190.7	188.5	2286	64.88
12/4/2020 13:56	189.5	191.1	188.3	2288	64.92
12/4/2020 13:56	189.4	190.8	188.6	2292	64.88
12/4/2020 13:56	189.4	191.3	189.1	2290	64.99
12/4/2020 13:56	189.4	191.2	189.3	2288	64.92
12/4/2020 13:56	189.4	190.8	188.7	2286	65.12
12/4/2020 13:56	189.3	191.1	188.3	2285	64.88
12/4/2020 13:56	189.3	190.9	188.1	2284	65.03
12/4/2020 13:56	189.3	191.4	188.4	2287	64.96
12/4/2020 13:56	189.2	191.4	188.5	2285	64.96
12/4/2020 13:56	189.2	190.7	188.5	2283	64.99
12/4/2020 13:56	189.2	191.4	188.4	2287	65
12/4/2020 13:56	189.2	191	188.2	2286	65.04
12/4/2020 13:56	189.1	191	188.3	2280	64.88
12/4/2020 13:56	189.1	191.2	188.6	2284	65
12/4/2020 13:56	189.1	190.8	189.1	2281	64.96
12/4/2020 13:56	189.1	191.4	189.3	2279	65.04
12/4/2020 13:56	189.1	190.9	188.8	2279	65.08
12/4/2020 13:56	189.1	191.1	188.2	2278	64.96
12/4/2020 13:56	189.1	191.4	188.1	2279	65
12/4/2020 13:56	189.1	191.1	188.1	2281	64.96
12/4/2020 13:56	189.1	191.5	188.1	2279	64.96
12/4/2020 13:56	189.3	190.8	188.6	2282	64.92
12/4/2020 13:56	189	191.5	188.5	2285	64.96
12/4/2020 13:57	189.1	191.2	188.4	2288	64.92
12/4/2020 13:57	189	190.5	188.4	2284	64.96

12/4/2020 13:57	189.1	191.3	188.3	2282	64.96
12/4/2020 13:57	189	190.9	188.4	2280	65
12/4/2020 13:57	189	190.8	188.2	2280	64.88
12/4/2020 13:57	189	191.2	188.2	2282	64.88
12/4/2020 13:57	189	190.7	188.1	2287	64.96
12/4/2020 13:57	189.1	191.2	188.1	2283	64.92
12/4/2020 13:57	189	190.8	188.2	2285	64.88
12/4/2020 13:57	189.1	191.4	188	2285	64.92
12/4/2020 13:57	189.1	191.3	188.1	2280	64.87
12/4/2020 13:57	189.2	190.8	188	2282	64.83
12/4/2020 13:57	189.2	191.3	188	2286	64.88
12/4/2020 13:57	189.2	190.6	188.2	2286	64.92
12/4/2020 13:57	189.1	191	188.1	2289	64.96
12/4/2020 13:57	189.2	191.4	188.1	2286	65.04
12/4/2020 13:57	189.2	190.7	188.1	2283	64.96
12/4/2020 13:57	189.2	191.2	188	2279	64.92
12/4/2020 13:57	189.2	190.8	188.1	2277	65.04
12/4/2020 13:57	189.2	191.4	187.9	2275	64.95
12/4/2020 13:57	189.2	191.4	188.1	2278	65
12/4/2020 13:57	189.2	190.7	188.1	2283	65.08
12/4/2020 13:57	189.2	191.2	188.2	2281	64.83
12/4/2020 13:57	189.3	190.9	188.2	2280	64.88
12/4/2020 13:57	189.3	190.8	188.4	2279	65.08
12/4/2020 13:57	189.2	191.4	188.3	2280	64.95
12/4/2020 13:57	189.2	190.8	188.4	2285	65.04
12/4/2020 13:57	189.2	191.4	188.4	2286	64.96
12/4/2020 13:57	189.2	190.8	188.4	2285	65.04
12/4/2020 13:57	189.2	190.8	188.2	2282	64.99
12/4/2020 13:57	189.2	191.5	188.1	2284	64.96
12/4/2020 13:57	189.3	190.8	187.7	2284	64.92
12/4/2020 13:57	189.2	191.2	188.2	2281	65.08
12/4/2020 13:57	189.2	190.7	188.6	2278	65.09
12/4/2020 13:57	189.2	191	188.8	2276	64.92
12/4/2020 13:57	189.2	191.5	188	2276	65
12/4/2020 13:57	189.2	190.8	188.1	2269	65.04
12/4/2020 13:57	189.2	191.2	188.1	2271	64.92
12/4/2020 13:57	189.2	190.9	188	2272	65
12/4/2020 13:57	189.1	190.8	187.9	2273	65.09
12/4/2020 13:57	189.1	189.6	187.7	2274	65.08
12/4/2020 13:57	189.1	190.3	188.8	2272	64.91
12/4/2020 13:57	189.2	190.2	188.7	2274	64.96
12/4/2020 13:57	189.1	189.8	188	2276	65
12/4/2020 13:57	189.1	190.1	187.9	2276	64.92
12/4/2020 13:57	189.1	190.2	188.2	2276	65.12
12/4/2020 13:57	189.2	190.3	188.2	2277	64.96
12/4/2020 13:57	189.1	190.3	188	2277	64.96
12/4/2020 13:57	189.2	189.7	187.9	2280	64.96

12/4/2020 13:57	189.2	189.6	188.1	2283	65.08
12/4/2020 13:57	189.2	189.7	188.6	2276	64.88
12/4/2020 13:57	189.1	190	188.8	2274	64.84
12/4/2020 13:57	189.2	190.3	188.2	2273	64.92
12/4/2020 13:57	189.2	190	188	2274	64.88
12/4/2020 13:57	189.2	190.5	188.1	2270	64.95
12/4/2020 13:57	189.2	190	188.1	2272	65.08
12/4/2020 13:57	189.2	189.6	188.1	2271	64.88
12/4/2020 13:57	189.4	190.3	187.7	2270	64.88
12/4/2020 13:57	189.4	190	187.8	2272	65.08
12/4/2020 13:57	189.4	189.9	188.2	2266	64.99
12/4/2020 13:58	189.5	190.4	188.6	2267	64.99
12/4/2020 13:58	189.5	190	188.8	2268	64.93
12/4/2020 13:58	189.6	190.1	188.3	2271	64.84
12/4/2020 13:58	189.6	189.8	188	2268	65.04
12/4/2020 13:58	189.6	190.3	187.8	2271	65
12/4/2020 13:58	189.7	190.2	187.9	2269	64.8
12/4/2020 13:58	189.8	189.6	188.1	2268	64.88
12/4/2020 13:58	189.8	190.2	188.1	2271	65.01
12/4/2020 13:58	189.9	189.8	188.2	2272	64.92
12/4/2020 13:58	190	190.4	188.2	2275	64.95
12/4/2020 13:58	190	190.3	188.2	2278	65
12/4/2020 13:58	190.1	190.1	188.2	2272	65
12/4/2020 13:58	190.2	190	188.1	2270	65.04
12/4/2020 13:58	190.3	189.7	188.2	2273	64.92
12/4/2020 13:58	190.4	189.8	188.1	2279	64.92
12/4/2020 13:58	190.5	190.2	188.1	2282	65
12/4/2020 13:58	190.6	189.5	188.1	2282	64.96
12/4/2020 13:58	190.6	190	188.1	2278	65
12/4/2020 13:58	190.7	190	187.8	2274	64.92
12/4/2020 13:58	190.8	189.6	187.9	2272	64.92
12/4/2020 13:58	190.9	189.8	188.3	2274	65.04
12/4/2020 13:58	191.1	190.1	188.8	2274	64.91
12/4/2020 13:58	191.1	190.4	188.8	2277	65
12/4/2020 13:58	191.3	190	188.2	2281	64.92
12/4/2020 13:58	191.3	189.6	187.7	2281	64.92
12/4/2020 13:58	191.4	189.5	187.9	2283	65.04
12/4/2020 13:58	191.4	189.9	188.2	2283	65.08
12/4/2020 13:58	191.6	190.3	188.4	2282	64.91
12/4/2020 13:58	191.7	190	188.1	2278	64.92
12/4/2020 13:58	191.8	189.8	188.1	2275	65.08
12/4/2020 13:58	191.9	190.3	188.8	2275	64.99
12/4/2020 13:58	191.9	189.8	188	2281	65.04
12/4/2020 13:58	192	190.3	188	2280	65.09
12/4/2020 13:58	192	190.2	188.3	2275	64.91
12/4/2020 13:58	192.2	190.3	188.2	2275	64.92
12/4/2020 13:58	192.2	190	188.1	2279	65.04

12/4/2020 13:58	192.3	190.8	188.8	2280	65.04
12/4/2020 13:58	192.4	190.9	188.7	2284	65.04
12/4/2020 13:58	192.5	190.5	188	2283	64.92
12/4/2020 13:58	192.6	190.7	188.2	2282	65.08
12/4/2020 13:58	192.6	191.5	188.3	2279	64.92
12/4/2020 13:58	192.7	191	188.4	2278	64.88
12/4/2020 13:58	192.8	191	188.1	2280	65.04
12/4/2020 13:58	192.9	190.7	188.6	2282	64.92
12/4/2020 13:58	192.9	190.7	189.1	2283	65.03
12/4/2020 13:58	192.9	191.4	187.9	2286	65.08
12/4/2020 13:58	193	190.6	188	2282	64.99
12/4/2020 13:58	193.1	191	188.2	2283	64.92
12/4/2020 13:58	193.1	191.4	188.4	2282	64.92
12/4/2020 13:58	193.1	191.2	188.4	2284	65.04
12/4/2020 13:58	193.2	191.2	188.1	2284	64.92
12/4/2020 13:58	193.2	190.9	188.4	2284	64.88
12/4/2020 13:58	193.3	191	189	2281	64.91
12/4/2020 13:58	193.3	191.4	189.1	2283	65.04
12/4/2020 13:58	193.3	190.7	188.7	2285	64.96
12/4/2020 13:58	193.4	191.5	188	2288	65
12/4/2020 13:58	193.5	190.8	187.9	2291	65.09
12/4/2020 13:58	193.5	190.9	188.1	2287	65
12/4/2020 13:58	193.5	191.5	188.3	2290	64.96
12/4/2020 13:59	193.6	191.2	188.5	2288	64.96
12/4/2020 13:59	193.6	191.4	188.8	2286	65
12/4/2020 13:59	193.6	191.1	188.5	2285	65.08
12/4/2020 13:59	193.7	190.8	188.4	2281	65
12/4/2020 13:59	193.8	190.7	188	2278	64.96
12/4/2020 13:59	193.7	190.7	188.2	2278	64.88
12/4/2020 13:59	193.8	191.2	188.5	2278	65
12/4/2020 13:59	193.9	190.9	188.9	2279	64.92
12/4/2020 13:59	193.9	190.8	189.3	2282	64.92
12/4/2020 13:59	194	190.8	189.3	2285	64.99
12/4/2020 13:59	194	190.9	188.9	2287	64.88
12/4/2020 13:59	194	191.2	188.4	2282	64.8
12/4/2020 13:59	194.1	191.2	188.3	2286	64.92
12/4/2020 13:59	194.1	191.4	188.2	2288	65
12/4/2020 13:59	194.1	191.4	188.4	2285	65.03
12/4/2020 13:59	194.2	190.8	188.6	2284	64.99
12/4/2020 13:59	194.2	191.5	188.6	2288	65.12
12/4/2020 13:59	194.3	191.4	188.6	2279	64.79
12/4/2020 13:59	194.4	191.4	188.6	2278	64.96
12/4/2020 13:59	194.3	190.8	188.4	2285	65.08
12/4/2020 13:59	194.4	190.7	188.6	2284	64.92
12/4/2020 13:59	194.4	190.9	189.2	2285	64.92
12/4/2020 13:59	194.5	191.2	189.3	2288	65
12/4/2020 13:59	194.6	191.4	188.9	2292	64.87

12/4/2020 13:59	194.6	191	188.3	2290	65.08
12/4/2020 13:59	194.7	190.7	188.1	2293	65.08
12/4/2020 13:59	194.7	191.4	188.2	2294	64.99
12/4/2020 13:59	194.7	191	188.4	2294	64.92
12/4/2020 13:59	194.8	190.8	188.5	2291	65.08
12/4/2020 13:59	194.8	191.4	188.5	2293	64.92
12/4/2020 13:59	194.8	190.5	188.6	2292	64.92
12/4/2020 13:59	194.9	190.9	188.6	2294	65
12/4/2020 13:59	194.9	190.9	188.6	2295	64.96
12/4/2020 13:59	195	190.8	188.6	2290	65
12/4/2020 13:59	195	191.4	188.5	2288	65
12/4/2020 13:59	195	190.6	188.6	2285	64.96
12/4/2020 13:59	195.1	191	188.4	2287	65.04
12/4/2020 13:59	195.2	190.7	188.4	2290	65.04
12/4/2020 13:59	195.2	191.3	188.6	2295	64.96
12/4/2020 13:59	195.3	191.5	188.8	2297	64.96
12/4/2020 13:59	195.2	190.8	189.3	2296	64.96
12/4/2020 13:59	195.4	191	189.3	2292	65.04
12/4/2020 13:59	195.4	191.4	188.9	2289	64.88
12/4/2020 13:59	195.5	190.9	188.5	2289	65
12/4/2020 13:59	195.5	191.4	188.8	2292	64.96
12/4/2020 13:59	195.5	190.9	188.8	2289	65
12/4/2020 13:59	195.6	191	188.5	2290	65.04
12/4/2020 13:59	195.5	191.2	188.5	2288	65.04
12/4/2020 13:59	195.6	190.7	189.5	2289	64.92
12/4/2020 13:59	195.6	191.4	189.5	2293	64.96
12/4/2020 13:59	195.6	190.9	188.7	2296	65
12/4/2020 13:59	195.6	191.5	188.8	2295	64.88
12/4/2020 13:59	195.6	191.1	189	2294	64.99
12/4/2020 13:59	195.7	190.7	188.8	2297	64.92
12/4/2020 13:59	195.7	191.2	188.6	2294	64.88
12/4/2020 13:59	195.7	191.7	188.5	2293	64.88
12/4/2020 13:59	195.7	192	189.3	2294	64.92
12/4/2020 13:59	195.7	192.2	189.6	2297	64.88
12/4/2020 13:59	195.7	191.9	188.8	2297	64.92
12/4/2020 14:00	195.7	191.9	188.6	2297	65.08
12/4/2020 14:00	195.7	192.1	188.9	2292	64.99
12/4/2020 14:00	195.8	192	189	2292	64.99
12/4/2020 14:00	195.7	192.1	188.9	2289	65.01
12/4/2020 14:00	195.8	191.9	188.4	2291	64.96
12/4/2020 14:00	195.7	192.2	188.9	2288	64.92
12/4/2020 14:00	195.8	192.3	189.6	2291	65.04
12/4/2020 14:00	195.8	192.2	189.5	2290	64.96
12/4/2020 14:00	195.8	191.9	188.8	2287	64.96
12/4/2020 14:00	195.8	191.8	188.9	2292	65
12/4/2020 14:00	195.9	192.1	189	2291	64.96
12/4/2020 14:00	195.8	192.2	189.1	2293	64.92

12/4/2020 14:00	195.8	192.1	189.1	2297	65
12/4/2020 14:00	195.9	191.9	188.9	2297	64.92
12/4/2020 14:00	195.9	191.7	188.8	2297	64.92
12/4/2020 14:00	195.8	192.2	188.6	2294	65.04
12/4/2020 14:00	195.9	192.3	188.7	2292	65
12/4/2020 14:00	195.8	192.3	188.6	2290	64.96
12/4/2020 14:00	195.8	192.1	188.6	2292	65.04
12/4/2020 14:00	195.9	191.9	188.7	2293	64.92
12/4/2020 14:00	195.8	192.2	188.7	2293	64.92
12/4/2020 14:00	195.8	192.1	188.7	2295	65.04
12/4/2020 14:00	195.7	191.9	188.6	2292	65.08
12/4/2020 14:00	195.8	192	188.6	2292	64.99
12/4/2020 14:00	195.9	191.9	188.7	2299	64.99
12/4/2020 14:00	195.8	192.1	188.9	2297	65.03
12/4/2020 14:00	195.9	192	188.9	2295	64.99
12/4/2020 14:00	195.8	192.1	189.1	2296	65.08
12/4/2020 14:00	195.9	192.3	189.1	2296	64.92
12/4/2020 14:00	195.8	192.1	189.1	2295	65.08
12/4/2020 14:00	195.8	192.2	188.8	2296	64.88
12/4/2020 14:00	195.8	191.8	188.8	2296	64.96
12/4/2020 14:00	195.9	191.9	189.3	2297	65.03
12/4/2020 14:00	195.8	192.1	189.9	2293	65.04
12/4/2020 14:00	195.7	191.9	189.2	2295	65
12/4/2020 14:00	195.7	191.9	188.6	2292	65.08
12/4/2020 14:00	195.8	191.9	189.1	2289	64.99
12/4/2020 14:00	195.9	191.8	189.3	2289	64.92
12/4/2020 14:00	195.8	192.2	189.2	2289	64.91
12/4/2020 14:00	195.7	192.2	188.9	2293	65.08
12/4/2020 14:00	195.8	192.2	189.3	2294	65.05
12/4/2020 14:00	195.7	191.9	190	2294	65
12/4/2020 14:00	195.8	191.9	189.4	2291	64.96
12/4/2020 14:00	195.7	192.2	188.8	2294	65.04
12/4/2020 14:00	195.8	192	189	2292	64.96
12/4/2020 14:00	195.8	192.3	189.3	2288	65.04
12/4/2020 14:00	195.7	191.9	189.2	2289	65
12/4/2020 14:00	195.8	191.9	189.3	2286	64.92
12/4/2020 14:00	195.8	192.2	188.9	2286	64.96
12/4/2020 14:00	195.8	192	189.3	2291	64.96
12/4/2020 14:00	195.8	192.2	189.9	2289	65.08
12/4/2020 14:00	195.8	191.9	189.8	2288	65.04
12/4/2020 14:00	195.8	191.9	189.3	2288	64.92
12/4/2020 14:00	195.8	192.2	188.7	2286	65.04
12/4/2020 14:00	195.7	191.9	188.6	2288	64.92
12/4/2020 14:00	195.7	192.2	189	2288	65.04
12/4/2020 14:00	195.9	192.1	189.2	2285	64.92
12/4/2020 14:00	195.7	191.8	189.3	2286	64.96
12/4/2020 14:00	195.8	192.3	189.3	2287	64.92

12/4/2020 14:00	195.7	192	189.1	2286	64.99
12/4/2020 14:01	195.7	192.3	189.1	2288	64.96
12/4/2020 14:01	195.7	192.2	189.1	2291	65
12/4/2020 14:01	195.7	192	189.1	2285	64.99
12/4/2020 14:01	195.7	192.1	189	2287	65.04
12/4/2020 14:01	195.7	192.2	189.1	2290	65
12/4/2020 14:01	195.8	191.9	189.1	2290	65
12/4/2020 14:01	195.8	191.9	189.1	2289	64.96
12/4/2020 14:01	195.7	192.1	188.9	2293	65.04
12/4/2020 14:01	195.7	192.1	189.1	2291	65
12/4/2020 14:01	195.7	192.1	189.2	2291	65
12/4/2020 14:01	195.7	191.9	189.3	2288	65.05
12/4/2020 14:01	195.7	192.2	189.3	2292	64.96
12/4/2020 14:01	195.7	191.8	189.2	2290	65
12/4/2020 14:01	195.7	192.2	188.8	2295	64.92
12/4/2020 14:01	195.7	192	188.7	2294	65
12/4/2020 14:01	195.8	191.9	189.5	2293	64.92
12/4/2020 14:01	195.8	192.1	189.9	2294	65.04
12/4/2020 14:01	195.7	191.7	189.1	2296	65.03
12/4/2020 14:01	195.7	192.1	188.9	2292	65.04
12/4/2020 14:01	195.7	191.9	189.3	2292	65.12
12/4/2020 14:01	195.7	192.2	189.2	2294	64.93
12/4/2020 14:01	195.7	192.4	188.9	2291	65.08
12/4/2020 14:01	195.7	191.9	188.7	2286	64.88
12/4/2020 14:01	195.7	192.4	189.5	2289	64.88
12/4/2020 14:01	195.7	192.9	190	2286	64.92
12/4/2020 14:01	195.7	193	189.1	2286	64.92
12/4/2020 14:01	195.7	193	189.1	2289	64.92
12/4/2020 14:01	195.7	193.3	189.5	2289	64.92
12/4/2020 14:01	195.7	193.2	189.3	2287	64.92
12/4/2020 14:01	195.7	193	189.3	2290	64.92
12/4/2020 14:01	195.7	193.3	189.1	2290	65.04
12/4/2020 14:01	195.7	193	189.5	2291	64.96
12/4/2020 14:01	195.7	192.9	190.2	2294	65
12/4/2020 14:01	195.7	193.1	189.7	2293	65
12/4/2020 14:01	195.7	192.9	189.1	2291	64.96
12/4/2020 14:01	195.8	193.3	189.2	2295	65.05
12/4/2020 14:01	195.7	193.1	189.3	2292	64.96
12/4/2020 14:01	195.7	193.3	189.3	2289	64.96
12/4/2020 14:01	195.7	193.1	189.3	2289	65
12/4/2020 14:01	195.7	193	189	2288	64.96
12/4/2020 14:01	195.8	193.2	188.9	2290	64.88
12/4/2020 14:01	195.8	193.1	189.3	2289	64.96
12/4/2020 14:01	195.8	193.2	190	2287	64.99
12/4/2020 14:01	195.8	193.2	190	2286	65.08
12/4/2020 14:01	195.7	192.9	189.3	2290	65.04
12/4/2020 14:01	195.7	193.1	188.9	2288	64.92

12/4/2020 14:01	195.8	193.1	189.3	2283	64.88
12/4/2020 14:01	195.7	193.2	189.4	2291	64.97
12/4/2020 14:01	195.7	193.2	189.3	2292	64.84
12/4/2020 14:01	195.8	193.1	189.3	2294	64.93
12/4/2020 14:01	195.7	193.1	189.1	2300	64.97
12/4/2020 14:01	195.8	193	189.1	2301	64.88
12/4/2020 14:01	195.7	193.1	189.6	2300	64.88
12/4/2020 14:01	195.7	193.2	190	2297	64.96
12/4/2020 14:01	195.7	193	190.1	2293	64.84
12/4/2020 14:01	195.8	193.3	189.6	2291	64.92
12/4/2020 14:01	195.7	193.1	189.1	2293	65.05
12/4/2020 14:01	195.8	193.2	189.1	2293	64.92
12/4/2020 14:01	195.7	193.2	189.1	2293	64.88
12/4/2020 14:02	195.8	193.1	189.1	2296	65.04
12/4/2020 14:02	195.8	193.3	189.3	2294	64.92
12/4/2020 14:02	195.7	193	189.3	2293	64.96
12/4/2020 14:02	195.7	193.1	189.3	2293	65
12/4/2020 14:02	195.7	193.2	189.2	2299	65
12/4/2020 14:02	195.7	193.1	189.3	2293	65
12/4/2020 14:02	195.7	193.3	189.3	2289	64.96
12/4/2020 14:02	195.7	193.2	189.3	2292	65
12/4/2020 14:02	195.7	193.2	189.3	2293	65.04
12/4/2020 14:02	195.7	193.1	189.3	2293	65.04
12/4/2020 14:02	195.7	192.9	189.3	2297	64.92
12/4/2020 14:02	195.8	193.3	189.2	2297	65.04
12/4/2020 14:02	195.7	193.2	189.1	2300	64.96
12/4/2020 14:02	195.8	193	189.1	2297	65.08
12/4/2020 14:02	195.7	193.2	189.3	2296	65.04
12/4/2020 14:02	195.7	193.1	189.6	2292	65.04
12/4/2020 14:02	195.7	193.2	189.9	2294	64.88
12/4/2020 14:02	195.7	193	190.1	2292	64.99
12/4/2020 14:02	195.6	193	190	2296	65.04
12/4/2020 14:02	195.7	192.9	189.3	2297	65.16
12/4/2020 14:02	195.6	193	189.1	2295	64.99
12/4/2020 14:02	195.7	193.3	189.3	2293	64.92
12/4/2020 14:02	195.7	193	189.3	2292	64.96
12/4/2020 14:02	195.7	193	189.5	2292	64.92
12/4/2020 14:02	195.7	193.1	189.5	2300	64.92
12/4/2020 14:02	195.7	193	189.5	2300	65
12/4/2020 14:02	195.7	193.1	189.1	2303	64.96
12/4/2020 14:02	195.7	193.1	189.8	2303	64.88
12/4/2020 14:02	195.7	192.9	190.2	2301	65.04
12/4/2020 14:02	195.6	193.3	189.3	2296	65
12/4/2020 14:02	195.6	193.1	189.1	2296	65
12/4/2020 14:02	195.6	193.1	189.3	2301	65.09
12/4/2020 14:02	195.6	193.1	189.3	2301	64.96
12/4/2020 14:02	195.6	193.1	189.5	2297	65

12/4/2020 14:02	195.6	193.1	189.3	2297	64.96
12/4/2020 14:02	195.5	193.1	190.2	2299	65.08
12/4/2020 14:02	195.5	193.1	189.7	2299	64.92
12/4/2020 14:02	195.4	193.2	189	2301	64.96
12/4/2020 14:02	195.4	193	189.3	2301	64.92
12/4/2020 14:02	195.4	192.9	189.5	2300	64.92
12/4/2020 14:02	195.3	193.1	189.5	2304	64.96
12/4/2020 14:02	195.3	193.2	189.2	2301	65.04
12/4/2020 14:02	195.2	193.2	189.6	2299	64.92
12/4/2020 14:02	195.2	192.9	190.2	2296	64.96
12/4/2020 14:02	195.2	193.1	190	2297	65.04
12/4/2020 14:02	195.2	193	189.1	2297	65.08
12/4/2020 14:02	195.1	193.3	189	2304	65.04
12/4/2020 14:02	195.1	193.3	189.1	2304	64.92
12/4/2020 14:02	195	193.3	189.3	2306	64.88
12/4/2020 14:02	195	193.2	189.3	2310	65
12/4/2020 14:02	194.9	193	189.5	2306	65.08
12/4/2020 14:02	194.8	193.1	189.5	2306	64.96
12/4/2020 14:02	194.8	193.1	189.5	2308	64.96
12/4/2020 14:02	194.8	192.9	189.5	2308	65.05
12/4/2020 14:02	194.8	193.2	189.5	2306	64.96
12/4/2020 14:02	194.7	193	189.3	2306	65
12/4/2020 14:02	194.6	193.1	189.4	2303	65.09
12/4/2020 14:02	194.6	193	189.4	2304	64.95
12/4/2020 14:02	194.6	193.2	189.4	2306	65
12/4/2020 14:02	194.5	193.3	189.3	2308	65
12/4/2020 14:03	194.5	193.1	189.3	2303	64.92
12/4/2020 14:03	194.4	193	189.3	2307	65
12/4/2020 14:03	194.4	193.1	189.3	2305	64.96
12/4/2020 14:03	194.3	193.1	188.8	2302	64.99
12/4/2020 14:03	194.3	193.1	189	2304	64.92
12/4/2020 14:03	194.4	193	189.8	2300	64.96
12/4/2020 14:03	194.2	193.1	190.1	2300	64.92
12/4/2020 14:03	194.1	193.1	189.2	2301	64.93
12/4/2020 14:03	194.1	193.1	189.2	2304	64.88
12/4/2020 14:03	194.1	193.3	189.3	2301	64.96
12/4/2020 14:03	194.1	193	189.3	2306	64.92
12/4/2020 14:03	193.9	193.1	189.1	2308	64.96
12/4/2020 14:03	193.9	193.1	188.9	2304	64.88
12/4/2020 14:03	193.9	193	189.6	2304	64.99
12/4/2020 14:03	193.9	193.3	190.1	2308	65
12/4/2020 14:03	193.8	192.9	189.6	2305	64.96
12/4/2020 14:03	193.7	193.3	189.1	2303	64.96
12/4/2020 14:03	193.6	193.1	189.2	2301	65
12/4/2020 14:03	193.6	193.1	189.5	2299	65
12/4/2020 14:03	193.5	193.1	189.5	2308	64.96
12/4/2020 14:03	193.5	193	189.4	2306	65.04

12/4/2020 14:03	193.5	193.1	189.3	2304	64.92
12/4/2020 14:03	193.3	193.1	189.2	2300	64.88
12/4/2020 14:03	193.4	193.1	189.1	2303	65
12/4/2020 14:03	193.3	193.1	189.1	2303	64.96
12/4/2020 14:03	193.2	193.1	188.9	2301	65.04
12/4/2020 14:03	193.2	193.1	189	2302	65.08
12/4/2020 14:03	193.1	193.1	189	2310	64.96
12/4/2020 14:03	193.1	193	189.1	2307	64.92
12/4/2020 14:03	193	193.1	189.1	2312	64.96
12/4/2020 14:03	193	193	189.3	2310	64.88
12/4/2020 14:03	192.9	193.1	189.3	2306	65.03
12/4/2020 14:03	192.9	193.1	189.3	2306	64.97
12/4/2020 14:03	192.9	193.1	189.3	2302	64.84
12/4/2020 14:03	192.9	193.3	189.3	2303	64.88
12/4/2020 14:03	192.8	193.1	189.3	2304	65.01
12/4/2020 14:03	192.7	193.2	188.9	2300	64.92
12/4/2020 14:03	192.6	193.1	189.1	2300	65
12/4/2020 14:03	192.6	193.1	189.8	2296	64.92
12/4/2020 14:03	192.5	193.1	189.5	2298	64.92
12/4/2020 14:03	192.5	193	188.8	2299	65.04
12/4/2020 14:03	192.6	193.1	189.2	2299	65.09
12/4/2020 14:03	192.4	193.2	189.3	2297	64.96
12/4/2020 14:03	192.4	193.2	189.1	2299	64.96
12/4/2020 14:03	192.4	193.2	189.6	2303	65.09
12/4/2020 14:03	192.4	193	189.8	2303	64.96
12/4/2020 14:03	192.3	193.1	188.7	2307	65.04
12/4/2020 14:03	192.3	193.2	189	2306	65
12/4/2020 14:03	192.2	192.9	189.2	2301	64.96
12/4/2020 14:03	192.2	193.2	189.1	2308	64.92
12/4/2020 14:03	192.1	193.1	188.9	2305	64.96
12/4/2020 14:03	192.1	193.2	189.8	2304	64.92
12/4/2020 14:03	192	193.1	189.5	2303	65.08
12/4/2020 14:03	192	193.2	188.8	2305	65.08
12/4/2020 14:03	192	193.1	189.1	2305	65.04
12/4/2020 14:03	191.9	192.9	189.2	2304	64.99
12/4/2020 14:03	192	193.1	189.1	2303	65
12/4/2020 14:03	191.8	193.3	188.9	2305	64.92
12/4/2020 14:03	191.8	193.1	189.4	2304	65.12
12/4/2020 14:04	191.8	193.1	189.7	2303	65.08
12/4/2020 14:04	191.6	192.9	189	2303	65.05
12/4/2020 14:04	191.7	193.2	188.7	2305	64.92
12/4/2020 14:04	191.6	193.1	189	2304	64.96
12/4/2020 14:04	191.6	192.9	188.9	2305	65.04
12/4/2020 14:04	191.6	193.1	189.1	2305	64.92
12/4/2020 14:04	191.4	193.2	188.8	2306	64.96
12/4/2020 14:04	191.5	193.1	189.1	2309	64.96
12/4/2020 14:04	191.4	193.2	189.8	2309	64.96

12/4/2020 14:04	191.4	192.9	188.9	2308	64.92
12/4/2020 14:04	191.3	193.1	188.7	2308	65
12/4/2020 14:04	191.3	193.1	189.1	2306	64.92
12/4/2020 14:04	191.2	193.1	189.1	2308	65
12/4/2020 14:04	191.2	193.3	189.1	2308	65
12/4/2020 14:04	191.2	193.1	189	2303	64.87
12/4/2020 14:04	191.1	193.3	188.8	2302	65
12/4/2020 14:04	191.1	193.1	188.8	2304	65
12/4/2020 14:04	191.1	193.1	189.1	2306	64.96
12/4/2020 14:04	191	193.2	189.6	2304	65.08
12/4/2020 14:04	191	192.9	189.6	2304	64.96
12/4/2020 14:04	190.9	193.3	189.5	2303	64.96
12/4/2020 14:04	190.9	193.1	189.3	2297	64.88
12/4/2020 14:04	190.9	193.2	189	2299	65.08
12/4/2020 14:04	190.8	192.3	189	2299	64.99
12/4/2020 14:04	190.8	192.2	188.9	2299	64.93
12/4/2020 14:04	190.8	192.1	189.2	2303	65.08
12/4/2020 14:04	190.7	192.2	189.3	2304	64.84
12/4/2020 14:04	190.7	191.9	189.5	2303	64.88
12/4/2020 14:04	190.6	191.9	189.6	2304	65.01
12/4/2020 14:04	190.5	191.8	189.2	2305	64.88
12/4/2020 14:04	190.6	192.2	188.6	2305	64.88
12/4/2020 14:04	190.5	192.3	188.8	2301	65.01
12/4/2020 14:04	190.5	191.9	189.1	2297	64.88
12/4/2020 14:04	190.5	192	188.9	2292	64.96
12/4/2020 14:04	190.5	192.2	188.5	2297	65.09
12/4/2020 14:04	190.4	191.8	189.1	2294	65
12/4/2020 14:04	190.3	192.2	189.3	2297	64.96
12/4/2020 14:04	190.3	192.1	188.5	2301	64.96
12/4/2020 14:04	190.3	192.2	188.8	2303	65
12/4/2020 14:04	190.2	191.9	188.9	2304	64.96
12/4/2020 14:04	190.1	192	188.8	2305	64.96
12/4/2020 14:04	190	192.2	188.4	2306	65
12/4/2020 14:04	190	192.1	188.4	2306	64.83
12/4/2020 14:04	190	192.2	189.3	2304	64.96
12/4/2020 14:04	190	191.8	189.4	2303	65.12
12/4/2020 14:04	190	191.8	188.5	2301	64.88
12/4/2020 14:04	189.9	192	188.7	2299	65
12/4/2020 14:04	189.9	191.9	188.7	2296	64.96
12/4/2020 14:04	189.8	192	188.8	2297	64.92
12/4/2020 14:04	189.8	191.9	188.5	2297	64.88
12/4/2020 14:04	189.8	191.9	188.4	2296	65
12/4/2020 14:04	189.7	192.1	188.9	2300	64.83
12/4/2020 14:04	189.7	191.9	189.5	2301	64.88
12/4/2020 14:04	189.8	192.2	189.1	2301	64.92
12/4/2020 14:04	189.7	192.3	188.6	2298	65.04
12/4/2020 14:04	189.7	191.9	188.4	2298	65.04

12/4/2020 14:04	189.7	192.1	188.5	2301	65.08
12/4/2020 14:04	189.7	192.1	188.6	2299	64.84
12/4/2020 14:04	189.7	191.9	188.7	2296	64.99
12/4/2020 14:05	189.6	192.3	188.7	2298	65.12
12/4/2020 14:05	189.6	191.7	188.6	2299	65.03
12/4/2020 14:05	189.7	192.1	188.5	2301	65.04
12/4/2020 14:05	189.7	192.1	188.6	2303	65.12
12/4/2020 14:05	189.6	192.3	188.5	2301	65.04
12/4/2020 14:05	189.7	192.2	188.7	2297	65.04
12/4/2020 14:05	189.7	191.8	188.6	2297	65.04
12/4/2020 14:05	189.7	191.9	188.4	2296	64.88
12/4/2020 14:05	189.7	191.8	188.5	2296	65
12/4/2020 14:05	189.7	192.4	188.6	2296	65.12
12/4/2020 14:05	189.7	192.1	188.6	2301	65
12/4/2020 14:05	189.7	191.8	188.8	2299	64.88
12/4/2020 14:05	189.7	192.1	188.6	2301	65
12/4/2020 14:05	189.7	192.1	188.5	2295	64.87
12/4/2020 14:05	189.7	192.2	188.5	2297	64.92
12/4/2020 14:05	189.7	192.4	188.5	2301	65.04
12/4/2020 14:05	189.6	191.9	188.5	2303	64.92
12/4/2020 14:05	189.7	192.1	189.2	2302	64.96
12/4/2020 14:05	189.7	191.9	189.3	2303	65.04
12/4/2020 14:05	189.7	192.2	188.6	2304	64.99
12/4/2020 14:05	189.7	192.6	188.3	2301	64.92
12/4/2020 14:05	189.7	191.9	188.3	2298	64.92
12/4/2020 14:05	189.6	192.1	188.5	2297	64.88
12/4/2020 14:05	189.7	191.8	188.6	2300	64.92
12/4/2020 14:05	189.7	192.1	188.7	2301	64.97
12/4/2020 14:05	189.7	192.2	188.3	2299	64.84
12/4/2020 14:05	189.6	192.2	188.6	2300	64.88
12/4/2020 14:05	189.6	191.8	189.3	2300	64.96
12/4/2020 14:05	189.7	191.9	189.1	2299	64.99
12/4/2020 14:05	189.7	192.1	188.6	2300	65.08
12/4/2020 14:05	189.6	192.2	188.1	2304	65.12
12/4/2020 14:05	189.6	191.9	188.1	2301	64.99
12/4/2020 14:05	189.6	192.1	188.1	2300	65.08
12/4/2020 14:05	189.6	191.9	188.5	2300	65.04
12/4/2020 14:05	189.6	191.9	188.5	2296	65.04
12/4/2020 14:05	189.6	192.4	188.4	2298	65.04
12/4/2020 14:05	189.6	191.9	188.5	2297	65.04
12/4/2020 14:05	189.5	192.2	188.6	2297	64.92
12/4/2020 14:05	189.5	192.2	188.5	2300	64.88
12/4/2020 14:05	189.6	191.8	188.5	2299	64.96
12/4/2020 14:05	189.5	192.2	188.4	2298	64.96
12/4/2020 14:05	189.5	191.9	188.5	2295	65
12/4/2020 14:05	189.5	192.3	188.4	2301	65.04
12/4/2020 14:05	189.4	191.9	188.5	2300	64.96

12/4/2020 14:05	189.4	191.9	188.3	2304	64.96
12/4/2020 14:05	189.4	192.1	188	2304	65.04
12/4/2020 14:05	189.4	191.9	188.2	2303	64.96
12/4/2020 14:05	189.4	192.1	189.2	2301	65
12/4/2020 14:05	189.4	191	188.6	2302	64.92
12/4/2020 14:05	189.3	190.8	188.4	2300	65
12/4/2020 14:05	189.3	191.4	188.5	2297	65.04
12/4/2020 14:05	189.3	190.7	188.6	2300	65.04
12/4/2020 14:05	189.3	191.2	188.2	2297	64.92
12/4/2020 14:05	189.2	191.2	188.2	2297	64.99
12/4/2020 14:05	189.2	191.4	189	2296	64.88
12/4/2020 14:05	189.2	191.4	189.1	2298	65.01
12/4/2020 14:05	189.2	190.7	188.5	2297	64.83
12/4/2020 14:05	189.2	191.2	188.4	2293	64.99
12/4/2020 14:05	189.2	191	188.4	2293	64.96
12/4/2020 14:05	189.2	190.8	188.4	2292	64.99
12/4/2020 14:06	189.2	191	188.2	2289	65.04
12/4/2020 14:06	189.2	190.8	188	2290	65.04
12/4/2020 14:06	189.2	191.3	187.9	2288	64.99
12/4/2020 14:06	189.2	191	188.6	2286	65
12/4/2020 14:06	189.2	190.8	189.1	2290	64.93
12/4/2020 14:06	189.2	191.4	188.6	2289	65.04
12/4/2020 14:06	189.3	190.9	188.2	2290	64.84
12/4/2020 14:06	189.3	191.2	188.2	2292	64.93
12/4/2020 14:06	189.2	191.2	188.2	2289	64.96
12/4/2020 14:06	189.2	191.3	188.3	2289	64.92
12/4/2020 14:06	189.3	191	188.4	2290	65.08
12/4/2020 14:06	189.3	190.6	187.9	2290	64.92
12/4/2020 14:06	189.4	191	187.9	2286	64.92
12/4/2020 14:06	189.3	190.8	188.4	2290	65
12/4/2020 14:06	189.4	191.4	189.2	2290	64.87
12/4/2020 14:06	189.4	191.4	188.4	2293	64.96
12/4/2020 14:06	189.4	190.7	188	2296	65
12/4/2020 14:06	189.4	191.3	188.2	2296	64.96
12/4/2020 14:06	189.4	191.3	188.5	2296	64.92
12/4/2020 14:06	189.5	191.1	188.2	2295	64.96
12/4/2020 14:06	189.5	191.4	188	2293	65
12/4/2020 14:06	189.6	191.1	187.9	2290	65
12/4/2020 14:06	189.6	191.1	188.6	2288	64.96
12/4/2020 14:06	189.7	190.9	189	2289	64.88
12/4/2020 14:06	189.7	191.2	188.6	2292	65.04
12/4/2020 14:06	189.7	191.4	188	2291	64.92
12/4/2020 14:06	189.8	190.6	188.3	2290	64.92
12/4/2020 14:06	189.9	191.4	188.2	2289	64.88
12/4/2020 14:06	189.8	190.8	188.6	2288	64.92
12/4/2020 14:06	190	190.8	188.1	2286	64.92
12/4/2020 14:06	190	191.4	187.8	2289	64.88

12/4/2020 14:06	190	190.9	188.5	2292	65.04
12/4/2020 14:06	190.1	190.8	189.1	2290	65.08
12/4/2020 14:06	190.1	191	188.5	2293	65.03
12/4/2020 14:06	190.2	190.7	188	2295	64.88
12/4/2020 14:06	190.4	190.8	188.2	2297	65.01
12/4/2020 14:06	190.4	191	188.4	2292	65.03
12/4/2020 14:06	190.5	191.4	188.2	2291	64.99
12/4/2020 14:06	190.5	191.3	187.7	2292	65.08
12/4/2020 14:06	190.6	190.8	188.1	2291	64.99
12/4/2020 14:06	190.7	191.3	188.9	2291	64.99
12/4/2020 14:06	190.8	190.8	189.1	2289	65
12/4/2020 14:06	190.9	191.4	188.4	2288	64.8
12/4/2020 14:06	190.9	191.4	188.1	2290	64.92
12/4/2020 14:06	190.9	190.7	188.4	2292	64.96
12/4/2020 14:06	191	191.2	188.5	2290	64.88
12/4/2020 14:06	191.1	190.7	188.5	2290	64.79
12/4/2020 14:06	191.2	191	188.3	2295	64.96
12/4/2020 14:06	191.2	191.2	188.2	2293	65
12/4/2020 14:06	191.5	191.3	188	2289	64.96
12/4/2020 14:06	191.4	191.2	188.5	2297	64.96
12/4/2020 14:06	191.5	190.7	189.1	2292	64.96
12/4/2020 14:06	191.6	191	189.3	2294	64.96
12/4/2020 14:06	191.7	190.8	188.6	2292	64.96
12/4/2020 14:06	191.8	191	188.4	2290	65.04
12/4/2020 14:06	191.9	191.6	188.3	2291	64.91
12/4/2020 14:06	191.9	190.9	188.2	2300	64.96
12/4/2020 14:06	192	191.3	188.2	2299	65
12/4/2020 14:06	192.1	191.4	188.2	2302	64.92
12/4/2020 14:07	192.1	191.1	188.4	2301	65
12/4/2020 14:07	192.2	191.3	188.5	2301	65
12/4/2020 14:07	192.3	191	188.4	2296	64.88
12/4/2020 14:07	192.4	190.9	188.5	2296	64.92
12/4/2020 14:07	192.4	190.7	188.4	2296	65
12/4/2020 14:07	192.5	191.2	188.3	2296	64.99
12/4/2020 14:07	192.6	191.6	188.4	2292	65.04
12/4/2020 14:07	192.6	191.1	188.5	2296	65.08
12/4/2020 14:07	192.6	191.5	188.6	2299	64.99
12/4/2020 14:07	192.8	191.5	189.1	2297	64.88
12/4/2020 14:07	192.8	190.7	189.3	2290	65.12
12/4/2020 14:07	192.9	191.2	189.2	2296	64.99
12/4/2020 14:07	192.9	191	188.7	2297	65.04
12/4/2020 14:07	192.9	190.8	188.2	2296	64.93
12/4/2020 14:07	193	191.2	188.4	2301	64.99
12/4/2020 14:07	193	190.6	188.6	2301	64.92
12/4/2020 14:07	193	191.4	188.7	2301	64.96
12/4/2020 14:07	193.1	190.7	188.6	2304	64.88
12/4/2020 14:07	193.1	190.9	188.5	2306	64.92

12/4/2020 14:07	193	191.5	188.9	2304	65
12/4/2020 14:07	193.1	191.4	189.5	2298	64.92
12/4/2020 14:07	193.1	191	188.5	2301	64.92
12/4/2020 14:07	193.1	190.7	188.5	2297	65
12/4/2020 14:07	193.1	190.9	188.8	2296	64.87
12/4/2020 14:07	193.1	190.5	188.8	2300	65.04
12/4/2020 14:07	193.1	190.6	188.5	2303	65.04
12/4/2020 14:07	193	191.5	189.1	2297	64.96
12/4/2020 14:07	193	190.8	189.5	2294	65
12/4/2020 14:07	193.1	190.9	188.3	2299	65.04
12/4/2020 14:07	193	191.4	188.4	2300	64.87
12/4/2020 14:07	193	191	188.8	2305	65
12/4/2020 14:07	193	191.2	188.8	2304	65.09
12/4/2020 14:07	193	190.8	188.6	2301	64.87
12/4/2020 14:07	193	190.9	188.6	2300	65
12/4/2020 14:07	193	191.2	189.5	2300	65.08
12/4/2020 14:07	192.9	190.7	189.4	2297	64.83
12/4/2020 14:07	192.9	191.2	188.8	2298	64.96
12/4/2020 14:07	192.9	190.8	188.4	2301	65.12
12/4/2020 14:07	192.9	190.8	188.4	2303	65.04
12/4/2020 14:07	192.9	191.4	188.6	2303	65.04
12/4/2020 14:07	192.9	190.8	188.8	2301	64.96
12/4/2020 14:07	192.9	191.1	188.7	2299	64.99
12/4/2020 14:07	192.8	191	188.8	2301	64.92
12/4/2020 14:07	192.9	191.4	188.7	2304	65.16
12/4/2020 14:07	192.8	190.6	188.7	2306	65.08
12/4/2020 14:07	192.7	190.7	188.5	2302	64.92
12/4/2020 14:07	192.8	191.4	188.5	2300	65.08
12/4/2020 14:07	192.8	190.9	188.5	2303	64.99
12/4/2020 14:07	192.7	190.9	188.5	2301	64.99
12/4/2020 14:07	192.6	190.7	188.6	2304	64.92
12/4/2020 14:07	192.7	190.9	188.6	2307	65.08
12/4/2020 14:07	192.6	191.5	188.7	2299	64.96
12/4/2020 14:07	192.5	190.8	188.9	2303	64.96
12/4/2020 14:07	192.6	191.5	188.9	2306	65.08
12/4/2020 14:07	192.5	191.3	189.2	2301	64.92
12/4/2020 14:07	192.4	190.9	189.2	2304	65.04
12/4/2020 14:07	192.4	191.3	189.3	2306	64.96
12/4/2020 14:07	192.4	190.9	189.3	2305	64.92
12/4/2020 14:07	192.4	191.5	189.3	2306	64.96
12/4/2020 14:07	192.4	191	189.4	2305	65.04
12/4/2020 14:08	192.3	190.8	189.4	2305	64.91
12/4/2020 14:08	192.3	191.4	189.5	2304	64.96
12/4/2020 14:08	192.2	190.8	189.5	2305	65
12/4/2020 14:08	192.2	191.1	189.5	2306	64.96
12/4/2020 14:08	192.2	191.1	189.1	2305	64.96
12/4/2020 14:08	192.2	190.7	189	2301	65

12/4/2020 14:08	192.1	191	188.5	2303	64.99
12/4/2020 14:08	192.1	190.6	188.5	2303	64.92
12/4/2020 14:08	192	191	188.2	2306	65.05
12/4/2020 14:08	192.1	191.4	188.4	2304	64.88
12/4/2020 14:08	191.9	191.4	188.5	2303	64.88
12/4/2020 14:08	191.9	191	188.6	2301	64.92
12/4/2020 14:08	191.9	190.8	188.8	2300	64.83
12/4/2020 14:08	191.8	191	188.9	2298	64.92
12/4/2020 14:08	191.8	190.7	188.9	2299	64.96
12/4/2020 14:08	191.7	191.4	188.7	2301	64.99
12/4/2020 14:08	191.7	191.4	188.8	2303	64.93
12/4/2020 14:08	191.7	190.7	188.8	2305	65.08
12/4/2020 14:08	191.7	191	188.6	2304	64.8
12/4/2020 14:08	191.7	190.7	188.7	2306	65.08
12/4/2020 14:08	191.6	190.7	188.5	2304	64.92
12/4/2020 14:08	191.6	191.4	188.4	2300	64.84
12/4/2020 14:08	191.4	190.8	188.5	2297	64.92
12/4/2020 14:08	191.5	191.2	188.4	2297	65
12/4/2020 14:08	191.5	191.3	188.2	2304	64.96
12/4/2020 14:08	191.4	190.8	188.4	2302	64.92
12/4/2020 14:08	191.4	191.3	188.2	2303	64.96
12/4/2020 14:08	191.3	190.8	188.5	2306	64.88
12/4/2020 14:08	191.3	190.8	188.5	2305	64.96
12/4/2020 14:08	191.3	190.6	188.6	2306	64.96
12/4/2020 14:08	191.2	190.8	188.7	2309	65.09
12/4/2020 14:08	191.2	191.4	188.6	2304	64.92
12/4/2020 14:08	191.1	190.8	188.6	2301	64.96
12/4/2020 14:08	191	191.2	188.7	2305	65.04
12/4/2020 14:08	191.1	190.7	188.4	2301	64.92
12/4/2020 14:08	191	191.3	188.2	2299	64.96
12/4/2020 14:08	191	191.4	188.4	2306	64.92
12/4/2020 14:08	191	190.8	188.5	2306	65.04
12/4/2020 14:08	190.9	191.4	189.3	2304	64.99
12/4/2020 14:08	191	190.8	189.2	2305	65.08
12/4/2020 14:08	190.9	190.7	188.8	2302	64.88
12/4/2020 14:08	190.9	190.7	188.4	2303	64.92
12/4/2020 14:08	190.8	190.8	188.6	2303	65.08
12/4/2020 14:08	190.8	191	188.6	2300	64.92
12/4/2020 14:08	190.8	190.7	188.5	2300	65.04
12/4/2020 14:08	190.8	190.9	188.6	2300	64.92
12/4/2020 14:08	190.8	191	188.6	2301	64.95
12/4/2020 14:08	190.7	190.9	188.6	2300	64.92
12/4/2020 14:08	190.7	191.3	188.4	2301	65.05
12/4/2020 14:08	190.7	191.2	188.4	2302	64.88
12/4/2020 14:08	190.7	191.4	188.1	2301	65
12/4/2020 14:08	190.7	191.4	188.2	2308	65.04
12/4/2020 14:08	190.6	190.7	188.2	2305	65

12/4/2020 14:08	190.6	191	188.2	2303	65
12/4/2020 14:08	190.5	191.4	188.3	2306	64.96
12/4/2020 14:08	190.5	190.7	188.4	2306	64.92
12/4/2020 14:08	190.5	190.9	188.5	2306	64.96
12/4/2020 14:08	190.5	190.7	188.5	2306	65
12/4/2020 14:08	190.5	191.1	188.4	2304	65.04
12/4/2020 14:09	190.5	191.2	188.6	2303	64.88
12/4/2020 14:09	190.4	190.9	188.5	2301	64.88
12/4/2020 14:09	190.3	191.4	188.6	2303	64.96
12/4/2020 14:09	190.4	190.8	188.5	2301	65.04
12/4/2020 14:09	190.3	191.2	188.4	2300	64.92
12/4/2020 14:09	190.3	190.6	188.5	2298	65.08
12/4/2020 14:09	190.3	190.6	188.4	2299	64.88
12/4/2020 14:09	190.2	191.4	188.1	2303	64.99
12/4/2020 14:09	190.1	190.8	188.2	2302	65.01
12/4/2020 14:09	190.1	190.9	188.6	2304	64.99
12/4/2020 14:09	190.2	190.7	189.1	2300	64.99
12/4/2020 14:09	190.1	190.8	189.3	2300	65.08
12/4/2020 14:09	190.1	191.5	189.2	2303	65
12/4/2020 14:09	190	191.1	188.9	2303	65.04
12/4/2020 14:09	190	191.4	188.5	2304	64.96
12/4/2020 14:09	190	190.6	188	2303	64.92
12/4/2020 14:09	190	190.7	187.9	2304	65
12/4/2020 14:09	189.9	191.4	187.9	2304	65.09
12/4/2020 14:09	190	191	188.2	2302	65
12/4/2020 14:09	189.9	191.4	188.2	2304	64.96
12/4/2020 14:09	189.8	190.7	188.2	2309	65
12/4/2020 14:09	189.8	191	188.5	2308	64.92
12/4/2020 14:09	189.8	191.4	188.4	2303	65
12/4/2020 14:09	189.8	191.2	188.2	2306	65
12/4/2020 14:09	189.8	190.9	188	2304	64.92
12/4/2020 14:09	189.6	190.9	188.4	2305	64.96
12/4/2020 14:09	189.7	191.2	188.8	2307	65.08
12/4/2020 14:09	189.6	191.4	189.1	2304	64.84
12/4/2020 14:09	189.6	190.7	188.7	2302	64.96
12/4/2020 14:09	189.6	191.2	188.1	2304	65.08
12/4/2020 14:09	189.6	190.7	187.9	2301	64.95
12/4/2020 14:09	189.6	191.4	188.2	2304	65.12
12/4/2020 14:09	189.5	191.4	188.5	2304	65.12
12/4/2020 14:09	189.6	191	188.3	2304	65.08
12/4/2020 14:09	189.5	190.9	188.1	2300	64.99
12/4/2020 14:09	189.4	190.7	188.1	2301	65.12
12/4/2020 14:09	189.4	191.4	188.7	2306	65.04
12/4/2020 14:09	189.4	191.4	188.9	2304	64.96
12/4/2020 14:09	189.4	190.6	188.1	2303	65.08
12/4/2020 14:09	189.4	191.2	187.8	2302	64.96
12/4/2020 14:09	189.4	190.6	188	2301	65

12/4/2020 14:09	189.2	190.8	188.1	2300	65.04
12/4/2020 14:09	189.3	191.4	188.2	2303	65.04
12/4/2020 14:09	189.3	191.2	188.2	2297	64.96
12/4/2020 14:09	189.3	191.1	188.1	2301	65
12/4/2020 14:09	189.2	190.9	188.1	2301	64.96
12/4/2020 14:09	189.2	190.8	187.8	2300	64.92
12/4/2020 14:09	189.2	191.4	187.8	2298	64.96
12/4/2020 14:09	189.2	190.8	187.9	2300	64.96
12/4/2020 14:09	189.2	191.4	188.1	2299	65.04
12/4/2020 14:09	189.1	190.8	188.2	2297	64.96
12/4/2020 14:09	189.1	190.8	188.2	2294	64.92
12/4/2020 14:09	189	190.8	188.1	2293	64.96
12/4/2020 14:09	189.1	191.2	188.1	2295	65.04
12/4/2020 14:09	189	191.3	187.9	2296	64.96
12/4/2020 14:09	189	190.7	187.9	2295	64.83
12/4/2020 14:09	189	190.7	187.9	2296	64.84
12/4/2020 14:09	189	191.4	187.7	2297	65.08
12/4/2020 14:09	189	191.2	187.7	2297	64.92
12/4/2020 14:10	189	191.3	187.9	2295	64.84
12/4/2020 14:10	188.9	190.9	188.1	2296	64.93
12/4/2020 14:10	189	191	188	2299	64.92
12/4/2020 14:10	188.9	191.1	187.8	2298	65.08
12/4/2020 14:10	188.9	191.4	187.7	2297	65.04
12/4/2020 14:10	188.9	191	187.7	2297	64.84
12/4/2020 14:10	188.9	190.1	188.2	2299	65.04
12/4/2020 14:10	189	190.5	188.8	2297	64.88
12/4/2020 14:10	189	190.2	188.2	2299	64.96
12/4/2020 14:10	189	190.3	187.8	2295	64.96
12/4/2020 14:10	189	190	188	2297	64.88
12/4/2020 14:10	189	190.2	188.5	2297	64.96
12/4/2020 14:10	189	189.6	187.9	2299	65
12/4/2020 14:10	189	190.1	187.7	2301	64.96
12/4/2020 14:10	189	190.3	187.7	2300	65
12/4/2020 14:10	189.1	189.8	188.5	2301	64.92
12/4/2020 14:10	189	190.1	188.5	2304	65.04
12/4/2020 14:10	189.1	189.6	187.9	2297	64.96
12/4/2020 14:10	189.1	190.2	187.6	2303	65
12/4/2020 14:10	189.1	190.3	187.8	2304	65
12/4/2020 14:10	189	189.7	188	2304	64.96
12/4/2020 14:10	189.1	190.3	187.9	2301	65.04
12/4/2020 14:10	189.2	189.6	187.7	2303	64.93
12/4/2020 14:10	189.1	189.6	187.7	2301	64.92
12/4/2020 14:10	189.1	190.2	187.5	2300	64.92
12/4/2020 14:10	189.2	190	188.2	2303	65.04
12/4/2020 14:10	189.2	190	188.6	2303	65.04
12/4/2020 14:10	189.1	189.6	188.2	2301	64.92
12/4/2020 14:10	189.2	189.6	187.8	2301	64.97

12/4/2020 14:10	189.2	190.3	187.7	2301	64.84
12/4/2020 14:10	189.2	190	187.9	2305	64.92
12/4/2020 14:10	189.3	190.2	187.8	2306	64.88
12/4/2020 14:10	189.2	189.8	187.9	2305	64.88
12/4/2020 14:10	189.2	189.7	187.8	2304	64.92
12/4/2020 14:10	189.2	190.3	187.7	2303	65.04
12/4/2020 14:10	189.3	189.9	187.6	2300	64.92
12/4/2020 14:10	189.3	189.8	187.7	2299	64.83
12/4/2020 14:10	189.3	190	188.4	2300	65
12/4/2020 14:10	189.3	189.7	188.6	2303	65.04
12/4/2020 14:10	189.3	190.4	188.4	2303	64.96
12/4/2020 14:10	189.4	189.8	187.7	2301	64.96
12/4/2020 14:10	189.4	189.7	187.7	2301	65.12
12/4/2020 14:10	189.3	189.8	187.8	2308	64.99
12/4/2020 14:10	189.4	189.8	187.8	2308	65.04
12/4/2020 14:10	189.4	190.6	188	2312	65
12/4/2020 14:10	189.4	189.6	187.7	2310	64.92
12/4/2020 14:10	189.4	189.7	187.6	2310	65.04
12/4/2020 14:10	189.4	189.5	187.5	2308	65.04
12/4/2020 14:10	189.5	190.1	187.5	2304	64.96
12/4/2020 14:10	189.4	190.3	187.9	2303	64.96
12/4/2020 14:10	189.4	190.1	188.5	2306	64.96
12/4/2020 14:10	189.5	190	188.5	2306	65
12/4/2020 14:10	189.5	190	188.2	2304	64.96
12/4/2020 14:10	189.5	189.7	187.8	2305	65
12/4/2020 14:10	189.5	190.3	187.5	2305	64.96
12/4/2020 14:10	189.6	189.6	187.4	2303	65.08
12/4/2020 14:10	189.6	190	187.5	2303	65.16
12/4/2020 14:10	189.6	190.3	187.7	2301	65.04
12/4/2020 14:10	189.5	189.5	187.8	2301	65.04
12/4/2020 14:11	189.6	190.3	187.7	2301	65.04
12/4/2020 14:11	189.6	190	187.7	2301	64.88
12/4/2020 14:11	189.6	190.4	187.7	2301	65.04
12/4/2020 14:11	189.6	189.7	187.7	2297	64.99
12/4/2020 14:11	189.6	190.2	187.7	2300	64.97
12/4/2020 14:11	189.6	190.3	187.7	2299	64.88
12/4/2020 14:11	189.7	190	187.7	2302	64.92
12/4/2020 14:11	189.6	190.3	187.7	2300	65.08
12/4/2020 14:11	189.7	189.8	187.8	2301	64.96
12/4/2020 14:11	189.7	189.8	187.7	2305	65.04
12/4/2020 14:11	189.6	190.3	187.7	2308	65
12/4/2020 14:11	189.7	190	187.7	2308	65
12/4/2020 14:11	189.8	189.8	187.8	2306	65
12/4/2020 14:11	189.8	190.3	187.7	2298	65.13
12/4/2020 14:11	189.8	189.7	187.9	2303	64.96
12/4/2020 14:11	189.8	190.3	187.7	2300	65
12/4/2020 14:11	189.8	190	187.7	2299	65.04

12/4/2020 14:11	189.8	189.8	187.6	2297	64.96
12/4/2020 14:11	189.9	189.7	187.5	2300	65.08
12/4/2020 14:11	189.8	189.8	187.6	2303	65.08
12/4/2020 14:11	189.9	190.2	187.9	2301	64.92
12/4/2020 14:11	189.9	190	188.2	2303	64.96
12/4/2020 14:11	190	189.6	188.4	2304	64.92
12/4/2020 14:11	190	189.7	187.9	2301	64.92
12/4/2020 14:11	190	189.8	187.4	2303	64.92
12/4/2020 14:11	190	190.2	187.4	2300	65.08
12/4/2020 14:11	190	189.9	187.5	2300	64.92
12/4/2020 14:11	190.1	189.6	187.7	2299	64.92
12/4/2020 14:11	190	190	187.8	2303	64.96
12/4/2020 14:11	190.1	189.6	187.6	2306	65.08
12/4/2020 14:11	190.1	189.9	187.5	2308	65.04
12/4/2020 14:11	190.1	189.8	187.5	2305	64.92
12/4/2020 14:11	190.1	189.7	187.9	2301	65
12/4/2020 14:11	190.1	190.3	188.6	2299	64.96
12/4/2020 14:11	190.2	189.8	188.3	2297	64.96
12/4/2020 14:11	190.2	190.3	187.7	2300	65.05
12/4/2020 14:11	190.1	189.6	187.5	2298	64.91
12/4/2020 14:11	190.2	190.3	187.3	2299	65
12/4/2020 14:11	190.2	190.2	187.5	2300	65
12/4/2020 14:11	190.3	189.6	187.7	2294	64.92
12/4/2020 14:11	190.2	190.2	187.7	2297	65
12/4/2020 14:11	190.2	189.7	187.9	2300	65.08
12/4/2020 14:11	190.3	189.6	187.7	2301	64.92
12/4/2020 14:11	190.3	190.3	187.7	2300	64.88
12/4/2020 14:11	190.3	190	187.5	2301	64.96
12/4/2020 14:11	190.3	190.1	187.7	2303	64.83
12/4/2020 14:11	190.3	190	187.6	2297	65.04
12/4/2020 14:11	190.3	189.8	187.7	2304	65.04
12/4/2020 14:11	190.3	190	187.5	2303	64.99
12/4/2020 14:11	190.2	190	187.3	2301	64.99
12/4/2020 14:11	190.3	190.2	187.3	2298	65.01
12/4/2020 14:11	190.2	190	187.4	2291	64.99
12/4/2020 14:11	190.3	189.6	187.5	2295	65.04
12/4/2020 14:11	190.2	190.3	187.4	2296	64.92
12/4/2020 14:11	190.2	190	187.5	2292	64.95
12/4/2020 14:11	190.3	190.2	187.6	2292	64.92
12/4/2020 14:11	190.2	190.3	187.5	2292	65.08
12/4/2020 14:11	190.3	190.3	187.5	2293	65.04
12/4/2020 14:11	190.3	190.4	187.7	2292	64.88
12/4/2020 14:12	190.2	189.9	187.7	2291	65
12/4/2020 14:12	190.4	190.1	187.6	2292	65.03
12/4/2020 14:12	190.3	190.1	187.2	2292	64.95
12/4/2020 14:12	190.3	189.6	187.2	2296	65.12
12/4/2020 14:12	190.3	190.5	187.7	2296	64.88

12/4/2020 14:12	190.3	189.9	188.1	2294	65.03
12/4/2020 14:12	190.4	190.2	188.2	2289	64.83
12/4/2020 14:12	190.5	190.2	187.4	2288	65.08
12/4/2020 14:12	190.5	189.6	187.4	2292	65.04
12/4/2020 14:12	190.6	190.4	187.7	2289	64.99
12/4/2020 14:12	190.6	189.8	187.5	2292	65
12/4/2020 14:12	190.7	190	187.4	2288	64.84
12/4/2020 14:12	190.7	190.1	187.2	2286	64.88
12/4/2020 14:12	190.8	189.6	188.2	2287	65.12
12/4/2020 14:12	190.9	190.3	188.4	2285	64.95
12/4/2020 14:12	190.9	189.7	187.6	2282	65.04
12/4/2020 14:12	190.9	190	187.5	2280	64.92
12/4/2020 14:12	191	190.2	187.7	2285	64.92
12/4/2020 14:12	191.1	190.1	187.8	2283	64.88
12/4/2020 14:12	191.2	190.3	187.9	2288	64.96
12/4/2020 14:12	191.2	189.8	187.7	2290	64.96
12/4/2020 14:12	191.3	189.8	187.3	2291	64.96
12/4/2020 14:12	191.4	189.7	187.5	2293	64.96
12/4/2020 14:12	191.3	190.3	188.2	2287	64.92
12/4/2020 14:12	191.5	190.3	188.4	2292	65
12/4/2020 14:12	191.6	189.9	188.1	2291	65
12/4/2020 14:12	191.7	189.6	187.5	2291	64.96
12/4/2020 14:12	191.8	189.7	187.6	2293	65
12/4/2020 14:12	191.8	189.6	187.8	2292	65
12/4/2020 14:12	192	190.1	187.7	2294	65.05
12/4/2020 14:12	192.1	189.9	187.4	2292	64.92
12/4/2020 14:12	192.1	189.7	187.9	2297	64.88
12/4/2020 14:12	192.3	189.5	187.7	2294	64.88
12/4/2020 14:12	192.1	189.6	187.7	2294	64.92
12/4/2020 14:12	192.4	190.3	187.6	2292	64.96
12/4/2020 14:12	192.5	190	187.5	2296	64.96
12/4/2020 14:12	192.6	190	187.7	2297	65.04
12/4/2020 14:12	192.6	189.7	187.4	2297	64.92
12/4/2020 14:12	192.8	189.5	187.5	2294	65.04
12/4/2020 14:12	192.8	190.2	187.5	2297	64.84
12/4/2020 14:12	192.9	190	187.5	2294	64.99
12/4/2020 14:12	193	190.3	187.7	2297	64.96
12/4/2020 14:12	193	190.2	187.7	2294	64.92
12/4/2020 14:12	193.1	189.5	187.9	2297	64.92
12/4/2020 14:12	193.1	190.3	187.9	2299	64.96
12/4/2020 14:12	193.3	189.6	188.1	2303	64.8
12/4/2020 14:12	193.3	190.5	188.3	2307	64.96
12/4/2020 14:12	193.4	190.2	188.2	2306	65.08
12/4/2020 14:12	193.5	189.7	188.2	2304	65.04
12/4/2020 14:12	193.5	190.2	188.5	2309	64.99
12/4/2020 14:12	193.6	189.9	188.6	2309	64.99
12/4/2020 14:12	193.6	190.3	188.6	2310	64.92

12/4/2020 14:12	193.7	189.7	188.6	2308	64.88
12/4/2020 14:12	193.7	189.5	188.5	2308	65.08
12/4/2020 14:12	193.8	190.2	188.4	2306	64.93
12/4/2020 14:12	193.9	189.6	188.1	2304	64.84
12/4/2020 14:12	193.9	190.3	187.9	2299	64.84
12/4/2020 14:12	193.9	189.9	187.8	2300	65
12/4/2020 14:12	194	189.6	187.9	2301	64.92
12/4/2020 14:12	194	190.7	187.8	2301	64.83
12/4/2020 14:13	194	190.7	187.9	2304	65.08
12/4/2020 14:13	194	190.6	187.9	2299	65
12/4/2020 14:13	194.1	191	188.1	2301	64.92
12/4/2020 14:13	194.1	190.8	188.1	2301	65.04
12/4/2020 14:13	194.1	191	188	2300	65
12/4/2020 14:13	194.1	191.4	188.1	2297	64.96
12/4/2020 14:13	194.1	190.7	187.9	2297	64.96
12/4/2020 14:13	194.2	191.2	187.9	2304	64.92
12/4/2020 14:13	194.2	191	187.7	2304	64.92
12/4/2020 14:13	194.1	190.8	187.5	2308	65.04
12/4/2020 14:13	194.2	190.7	187.7	2309	64.92
12/4/2020 14:13	194.2	191.2	188.2	2309	64.96
12/4/2020 14:13	194.2	191.4	188.6	2313	65
12/4/2020 14:13	194.1	191	188.8	2310	64.96
12/4/2020 14:13	194.2	190.8	188.1	2308	64.96
12/4/2020 14:13	194.2	190.8	187.7	2306	64.92
12/4/2020 14:13	194.2	191.1	188.1	2305	65
12/4/2020 14:13	194.2	191.2	188.2	2301	64.96
12/4/2020 14:13	194.1	191.2	188.1	2303	64.92
12/4/2020 14:13	194.2	191.4	188	2305	65.04
12/4/2020 14:13	194.1	190.7	187.8	2304	64.87
12/4/2020 14:13	194.2	190.6	188.4	2303	64.99
12/4/2020 14:13	194.2	191.3	188.9	2306	65
12/4/2020 14:13	194.1	190.8	188.4	2310	64.83
12/4/2020 14:13	194.1	190.8	188	2305	64.92
12/4/2020 14:13	194.1	190.7	188.1	2310	65.12
12/4/2020 14:13	194.1	190.9	188.2	2311	64.99
12/4/2020 14:13	194.1	191.3	188.1	2308	65.04
12/4/2020 14:13	194	191	187.9	2309	65.01
12/4/2020 14:13	194.1	191.4	187.8	2305	64.88
12/4/2020 14:13	193.9	191.4	188.5	2301	64.88
12/4/2020 14:13	193.9	190.7	188.8	2307	65.04
12/4/2020 14:13	193.9	190.9	188.4	2309	65.04
12/4/2020 14:13	193.8	190.8	187.9	2311	64.88
12/4/2020 14:13	193.8	190.8	188.1	2317	64.88
12/4/2020 14:13	193.8	191.2	188.2	2312	65.04
12/4/2020 14:13	193.8	190.9	188.1	2310	64.95
12/4/2020 14:13	193.7	191.1	188	2309	64.99
12/4/2020 14:13	193.6	191.2	188.2	2308	64.88

12/4/2020 14:13	193.6	191.3	188	2309	64.96
12/4/2020 14:13	193.5	191.4	187.8	2304	64.83
12/4/2020 14:13	193.5	190.8	187.8	2313	65.08
12/4/2020 14:13	193.5	191.4	187.9	2311	64.95
12/4/2020 14:13	193.4	191.1	188.1	2309	64.84
12/4/2020 14:13	193.4	191.2	188.7	2310	64.88
12/4/2020 14:13	193.3	190.8	188.9	2312	64.96
12/4/2020 14:13	193.3	190.7	188.9	2310	64.92
12/4/2020 14:13	193.3	191.1	188.8	2309	65.04
12/4/2020 14:13	193.2	191.2	188.6	2309	64.92
12/4/2020 14:13	193.1	191.2	188.4	2308	64.92
12/4/2020 14:13	193.1	191	188.4	2316	65.05
12/4/2020 14:13	193	190.9	188.1	2311	64.87
12/4/2020 14:13	193	190.9	187.9	2310	64.92
12/4/2020 14:13	192.9	191.2	188	2312	65.04
12/4/2020 14:13	192.8	191.3	187.8	2310	64.96
12/4/2020 14:13	192.8	190.9	188	2310	65
12/4/2020 14:13	192.7	190.8	187.9	2308	65
12/4/2020 14:13	192.7	190.8	187.7	2305	64.88
12/4/2020 14:13	192.7	191.3	187.8	2304	64.96
12/4/2020 14:14	192.6	191.4	187.8	2303	65
12/4/2020 14:14	192.5	191	187.8	2310	64.96
12/4/2020 14:14	192.4	190.9	187.9	2310	65.04
12/4/2020 14:14	192.4	191.5	187.9	2310	64.92
12/4/2020 14:14	192.3	190.9	187.9	2314	64.96
12/4/2020 14:14	192.3	190.9	188	2310	64.96
12/4/2020 14:14	192.3	190.6	188.1	2305	64.92
12/4/2020 14:14	192.3	190.9	188	2303	65.04
12/4/2020 14:14	192.2	190.7	188	2303	65.04
12/4/2020 14:14	192.2	191.3	188	2304	64.88
12/4/2020 14:14	192.2	191.4	188	2306	64.88
12/4/2020 14:14	192.2	190.9	188.1	2306	65.04
12/4/2020 14:14	192.2	191.4	187.9	2311	64.96
12/4/2020 14:14	192.2	191.3	187.9	2312	64.95
12/4/2020 14:14	192.1	190.9	187.9	2307	64.88
12/4/2020 14:14	192.1	191.2	187.9	2308	65.09
12/4/2020 14:14	192.1	190.7	187.7	2305	64.88
12/4/2020 14:14	192.1	190.7	187.7	2303	64.88
12/4/2020 14:14	192.1	191.4	187.8	2309	64.92
12/4/2020 14:14	192	190.9	188.1	2307	64.92
12/4/2020 14:14	192	191.4	188.5	2308	64.84
12/4/2020 14:14	192	190.8	188.6	2308	64.96
12/4/2020 14:14	192	190.9	188.8	2306	64.88
12/4/2020 14:14	192	190.6	188.6	2304	64.88
12/4/2020 14:14	191.9	190.7	188.1	2301	65.12
12/4/2020 14:14	192	191.4	187.7	2302	65.04
12/4/2020 14:14	191.9	190.9	187.7	2300	65

12/4/2020 14:14	191.9	191.4	187.5	2304	65.04
12/4/2020 14:14	191.9	191	187.7	2304	65
12/4/2020 14:14	191.9	191	187.9	2303	65
12/4/2020 14:14	191.8	191.4	187.9	2301	65
12/4/2020 14:14	191.8	190.8	188	2302	65
12/4/2020 14:14	191.8	190.8	187.9	2301	65
12/4/2020 14:14	191.8	190.7	187.9	2300	65.04
12/4/2020 14:14	191.8	191.1	187.7	2302	64.96
12/4/2020 14:14	191.8	191.3	187.7	2301	64.87
12/4/2020 14:14	191.7	190.9	187.7	2306	65
12/4/2020 14:14	191.6	190.8	187.4	2304	65.05
12/4/2020 14:14	191.6	190.6	187.7	2304	64.92
12/4/2020 14:14	191.6	191.2	187.9	2301	64.92
12/4/2020 14:14	191.6	191.2	188.2	2303	65.08
12/4/2020 14:14	191.6	191.2	188.5	2306	65.03
12/4/2020 14:14	191.4	190.9	188.7	2301	64.92
12/4/2020 14:14	191.5	191.4	188.4	2303	65.12
12/4/2020 14:14	191.4	191.2	188.2	2301	65.03
12/4/2020 14:14	191.3	191.5	187.9	2299	64.88
12/4/2020 14:14	191.4	190.9	187.7	2304	64.96
12/4/2020 14:14	191.3	191	187.5	2299	64.84
12/4/2020 14:14	191.3	191.5	187.5	2299	65.08
12/4/2020 14:14	191.3	190.8	187.5	2301	64.92
12/4/2020 14:14	191.2	191.3	187.5	2301	65.08
12/4/2020 14:14	191.1	190.7	187.5	2299	64.88
12/4/2020 14:14	191.1	190.8	187.7	2300	64.96
12/4/2020 14:14	191.1	191.4	187.7	2303	64.84
12/4/2020 14:14	191	191	187.7	2298	64.96
12/4/2020 14:14	191	191.4	187.5	2300	64.92
12/4/2020 14:14	191	190.7	187.5	2299	65.04
12/4/2020 14:14	190.9	190.8	187.5	2297	64.99
12/4/2020 14:15	190.9	190.8	187.5	2293	64.99
12/4/2020 14:15	190.9	190.7	187.4	2293	65.04
12/4/2020 14:15	190.9	191.2	187.4	2293	64.83
12/4/2020 14:15	190.8	190.6	187.5	2297	64.99
12/4/2020 14:15	190.8	190.9	187.4	2295	65
12/4/2020 14:15	190.8	191.4	187.5	2293	64.87
12/4/2020 14:15	190.7	191.2	187.5	2291	64.96
12/4/2020 14:15	190.7	191.3	187.5	2294	65.09
12/4/2020 14:15	190.7	190.7	187.4	2294	64.91
12/4/2020 14:15	190.6	190.8	187.4	2293	65.04
12/4/2020 14:15	190.7	191.3	187.4	2293	65
12/4/2020 14:15	190.7	190.6	187.5	2291	64.96
12/4/2020 14:15	190.6	191.4	187.5	2292	64.96
12/4/2020 14:15	190.6	190.6	187.7	2297	64.96
12/4/2020 14:15	190.7	190.9	187.5	2293	64.91
12/4/2020 14:15	190.6	191.4	187.7	2289	65

12/4/2020 14:15	190.6	190.8	187.8	2289	65.08
12/4/2020 14:15	190.6	190.8	187.6	2290	64.99
12/4/2020 14:15	190.5	190.6	187.8	2289	64.96
12/4/2020 14:15	190.5	190.8	188	2292	64.92
12/4/2020 14:15	190.5	191.2	187.7	2289	64.84
12/4/2020 14:15	190.5	190.8	187.7	2288	65.03
12/4/2020 14:15	190.5	190.8	187.6	2293	65.08
12/4/2020 14:15	190.5	190.7	187.6	2292	64.96
12/4/2020 14:15	190.5	191.7	187.6	2292	64.83
12/4/2020 14:15	190.5	191.3	187.4	2292	64.99
12/4/2020 14:15	190.4	190.6	187.4	2295	64.88
12/4/2020 14:15	190.5	191.3	187.2	2292	64.99
12/4/2020 14:15	190.5	190.7	187.4	2291	64.88
12/4/2020 14:15	190.5	190.8	187.5	2289	65.08
12/4/2020 14:15	190.5	191.4	187.2	2294	64.84
12/4/2020 14:15	190.6	191	187.2	2291	65.08
12/4/2020 14:15	190.5	190.8	187.3	2289	65.09
12/4/2020 14:15	190.5	190.7	187.3	2293	64.8
12/4/2020 14:15	190.7	190.9	187.4	2291	64.84
12/4/2020 14:15	190.7	191.3	187.4	2297	64.96
12/4/2020 14:15	190.6	190.8	187.4	2297	64.88
12/4/2020 14:15	190.6	191	187.5	2297	64.96
12/4/2020 14:15	190.6	191.5	187.3	2297	65.04
12/4/2020 14:15	190.7	191.4	187.5	2293	64.83
12/4/2020 14:15	190.8	191.4	187.4	2292	64.99
12/4/2020 14:15	190.8	190.8	187.4	2290	65.08
12/4/2020 14:15	190.8	190.8	187.7	2290	65.08
12/4/2020 14:15	190.9	191.4	187.9	2289	64.99
12/4/2020 14:15	190.8	191.3	187.7	2288	65
12/4/2020 14:15	190.9	191.4	187.7	2292	65
12/4/2020 14:15	191	190.8	187.5	2289	65.04
12/4/2020 14:15	191	190.9	187.5	2288	64.92
12/4/2020 14:15	191	191.4	187.3	2290	64.96
12/4/2020 14:15	191.1	190.8	187	2293	65
12/4/2020 14:15	191.2	191.4	187.4	2293	64.96
12/4/2020 14:15	191.2	190.7	188.1	2297	64.96
12/4/2020 14:15	191.3	191.2	188.5	2296	65.04
12/4/2020 14:15	191.3	190.7	187.9	2293	64.96
12/4/2020 14:15	191.4	190.9	187.4	2297	64.96
12/4/2020 14:15	191.5	191.2	187.4	2295	64.92
12/4/2020 14:15	191.6	190.8	187.5	2292	64.96
12/4/2020 14:15	191.6	191.4	187.7	2297	65
12/4/2020 14:15	191.7	191.4	187.5	2299	64.87
12/4/2020 14:15	191.8	190.7	187.3	2299	64.99
12/4/2020 14:16	192	191.5	187.5	2298	65.08
12/4/2020 14:16	192	190.7	188.4	2301	64.99
12/4/2020 14:16	192.1	190.8	188.5	2304	65.04

12/4/2020 14:16	192.1	190.7	187.7	2306	64.96
12/4/2020 14:16	192.2	190.9	187.5	2303	64.99
12/4/2020 14:16	192.2	191	187.6	2304	64.84
12/4/2020 14:16	192.3	190.8	187.8	2307	65.12
12/4/2020 14:16	192.3	191.4	187.7	2304	64.99
12/4/2020 14:16	192.4	191.2	187.4	2299	64.92
12/4/2020 14:16	192.5	190.7	187.5	2301	65.12
12/4/2020 14:16	192.6	190.8	188.1	2304	64.88
12/4/2020 14:16	192.7	190.9	188.5	2304	65
12/4/2020 14:16	192.8	190.8	187.7	2304	65.04
12/4/2020 14:16	192.9	191.4	187.5	2303	64.84
12/4/2020 14:16	192.8	190.7	187.7	2301	65.04
12/4/2020 14:16	192.9	191	187.7	2303	65.08
12/4/2020 14:16	193	190.9	187.7	2304	64.96
12/4/2020 14:16	193	191.1	187.6	2304	65.04
12/4/2020 14:16	193.1	191.3	187.5	2305	64.88
12/4/2020 14:16	193.1	191.3	188.1	2304	64.96
12/4/2020 14:16	193.1	191	188.5	2301	64.88
12/4/2020 14:16	193.2	190.7	187.9	2301	64.92
12/4/2020 14:16	193.1	191.1	187.6	2299	64.92
12/4/2020 14:16	193.2	191.3	187.8	2300	64.88
12/4/2020 14:16	193.3	191.4	187.9	2303	64.83
12/4/2020 14:16	193.2	190.9	187.9	2306	64.96
12/4/2020 14:16	193.3	190.9	187.5	2308	64.95
12/4/2020 14:16	193.2	190.9	187.3	2310	64.88
12/4/2020 14:16	193.3	190.7	187.7	2314	65
12/4/2020 14:16	193.3	190.7	188.6	2308	65
12/4/2020 14:16	193.2	191.5	188.6	2308	65.04
12/4/2020 14:16	193.1	190.8	187.9	2308	65
12/4/2020 14:16	193.3	190.9	187.7	2303	64.96
12/4/2020 14:16	193.1	191.4	187.9	2305	65.04
12/4/2020 14:16	193.1	191.2	187.5	2301	65.04
12/4/2020 14:16	193.2	190.9	187.9	2301	64.95
12/4/2020 14:16	193.1	190.7	187.6	2300	64.96
12/4/2020 14:16	193.1	191	187.5	2301	65.04
12/4/2020 14:16	193	191.2	187.7	2303	64.83
12/4/2020 14:16	193	190.7	188.6	2306	64.92
12/4/2020 14:16	193	190.9	188.4	2309	65.01
12/4/2020 14:16	192.9	190.7	187.7	2314	65.08
12/4/2020 14:16	192.9	191	187.7	2312	65.08
12/4/2020 14:16	192.9	191.2	187.7	2314	65.08
12/4/2020 14:16	192.8	190.6	188.1	2314	64.99
12/4/2020 14:16	192.7	190.9	187.7	2314	65.04
12/4/2020 14:16	192.7	190.7	187.7	2312	65.04
12/4/2020 14:16	192.7	191.4	187.4	2311	65.04
12/4/2020 14:16	192.6	191.4	187.7	2307	64.99
12/4/2020 14:16	192.6	190.7	188.6	2308	64.84

12/4/2020 14:16	192.5	190.9	188.2	2313	64.93
12/4/2020 14:16	192.4	190.7	187.7	2312	64.88
12/4/2020 14:16	192.4	191.1	187.8	2309	65
12/4/2020 14:16	192.4	191.2	187.9	2308	65.04
12/4/2020 14:16	192.4	191.2	187.9	2307	64.92
12/4/2020 14:16	192.3	191.3	187.6	2313	64.92
12/4/2020 14:16	192.2	191	187.7	2315	65.05
12/4/2020 14:16	192.2	190.9	188.6	2310	65
12/4/2020 14:16	192.2	191.4	188.4	2311	64.96
12/4/2020 14:17	192.1	190.7	187.7	2312	65.05
12/4/2020 14:17	192.1	191	187.6	2312	64.96
12/4/2020 14:17	192.1	190.7	187.8	2308	65.05
12/4/2020 14:17	192	191.4	187.9	2308	65
12/4/2020 14:17	192	191.3	187.9	2310	64.88
12/4/2020 14:17	191.9	190.7	187.8	2310	64.92
12/4/2020 14:17	191.8	191.1	187.7	2310	64.96
12/4/2020 14:17	191.8	190.8	187.4	2312	65.04
12/4/2020 14:17	191.8	191	187.5	2314	64.92
12/4/2020 14:17	191.7	191.3	187.7	2313	64.96
12/4/2020 14:17	191.7	190.7	187.9	2315	65.08
12/4/2020 14:17	191.7	191	188.4	2319	65.08
12/4/2020 14:17	191.6	190.7	188.4	2317	65
12/4/2020 14:17	191.5	190.9	188	2314	64.96
12/4/2020 14:17	191.4	191.5	187.7	2308	65.04
12/4/2020 14:17	191.4	190.8	187.3	2309	65.04
12/4/2020 14:17	191.3	191.1	187.4	2307	65.08
12/4/2020 14:17	191.3	190.9	187.8	2304	65
12/4/2020 14:17	191.3	190.9	187.7	2305	64.96
12/4/2020 14:17	191.2	191.4	187.7	2308	64.96
12/4/2020 14:17	191.1	191.1	187.7	2305	64.96
12/4/2020 14:17	191.1	191.2	187.5	2308	64.96
12/4/2020 14:17	191.1	190.9	187.4	2308	64.96
12/4/2020 14:17	191	190.8	187.4	2308	64.92
12/4/2020 14:17	190.9	190.7	187.4	2308	64.96
12/4/2020 14:17	190.9	191	187.4	2312	65.01
12/4/2020 14:17	190.9	190.9	187.5	2315	64.92
12/4/2020 14:17	190.8	190.6	187.7	2312	64.88
12/4/2020 14:17	190.8	190.9	187.9	2312	64.96
12/4/2020 14:17	190.8	191.3	188.1	2309	64.8
12/4/2020 14:17	190.7	190.7	188.1	2310	64.96
12/4/2020 14:17	190.7	191	187.9	2310	65.12
12/4/2020 14:17	190.6	190.7	188	2311	64.88
12/4/2020 14:17	190.5	190.9	187.9	2313	65.12
12/4/2020 14:17	190.5	191.3	187.7	2314	65.08
12/4/2020 14:17	190.4	191.1	187.5	2310	64.92
12/4/2020 14:17	190.4	191	187.2	2309	65.04
12/4/2020 14:17	190.4	190.8	187.3	2311	65.01

12/4/2020 14:17	190.3	190.9	187.3	2312	65
12/4/2020 14:17	190.2	190.5	187.1	2310	65
12/4/2020 14:17	190.3	191	187.2	2312	65.08
12/4/2020 14:17	190.2	191.4	187.5	2312	65
12/4/2020 14:17	190.1	191	187.5	2314	64.96
12/4/2020 14:17	190	190.7	187.6	2310	64.96
12/4/2020 14:17	190.1	191.3	187.7	2309	65
12/4/2020 14:17	190	191.2	187.5	2307	64.96
12/4/2020 14:17	190	191.3	187.4	2315	65
12/4/2020 14:17	190	191.2	187.5	2310	65.08
12/4/2020 14:17	189.8	190.8	188.1	2308	64.99
12/4/2020 14:17	189.9	190.7	188.3	2310	64.96
12/4/2020 14:17	189.8	190.6	187.5	2310	65.08
12/4/2020 14:17	189.7	191.3	187.2	2309	64.95
12/4/2020 14:17	189.7	191.2	187.3	2310	65
12/4/2020 14:17	189.5	190.7	187.5	2310	65.12
12/4/2020 14:17	189.7	190.6	187.7	2311	64.96
12/4/2020 14:17	189.7	190.9	187.6	2312	64.92
12/4/2020 14:17	189.6	191.3	187.5	2314	65.16
12/4/2020 14:17	189.5	190.9	187.4	2318	64.99
12/4/2020 14:17	189.5	190.9	187.5	2314	65.16
12/4/2020 14:17	189.4	191.3	187.8	2312	64.93
12/4/2020 14:18	189.5	191	188.1	2312	64.88
12/4/2020 14:18	189.4	191.1	187.9	2306	64.88
12/4/2020 14:18	189.4	191.2	187.6	2308	64.92
12/4/2020 14:18	189.3	190.8	187.1	2309	64.88
12/4/2020 14:18	189.3	191.1	187	2311	64.92
12/4/2020 14:18	189.3	190.7	187.2	2310	65.01
12/4/2020 14:18	189.3	191.1	187.2	2312	64.92
12/4/2020 14:18	189.2	190.7	187.4	2310	64.96
12/4/2020 14:18	189.2	190.5	187.6	2312	65.09
12/4/2020 14:18	189.2	191.3	187.3	2309	64.96
12/4/2020 14:18	189.2	190.7	187.6	2309	65.04
12/4/2020 14:18	189.1	191.1	187.4	2309	65.13
12/4/2020 14:18	189.1	191.4	187.1	2307	64.96
12/4/2020 14:18	189	191.5	187	2310	64.92
12/4/2020 14:18	189	191.4	187.2	2317	65.05
12/4/2020 14:18	189	190.6	187.5	2315	65.03
12/4/2020 14:18	188.9	191	188.1	2312	65.04
12/4/2020 14:18	188.9	191.4	187.9	2312	64.96
12/4/2020 14:18	188.9	190.6	188	2313	65.03
12/4/2020 14:18	188.9	191.2	187.5	2313	65.08
12/4/2020 14:18	188.9	190.7	187	2311	65.12
12/4/2020 14:18	188.8	191	187	2315	64.88
12/4/2020 14:18	188.8	190.2	186.9	2314	64.99
12/4/2020 14:18	188.8	190.3	187.3	2313	65.16
12/4/2020 14:18	188.8	189.8	187.2	2311	64.88

12/4/2020 14:18	188.8	189.5	187.2	2310	65.08
12/4/2020 14:18	188.7	190.1	187.2	2311	65.04
12/4/2020 14:18	188.7	190	187	2312	64.91
12/4/2020 14:18	188.7	190.3	187.2	2313	64.96
12/4/2020 14:18	188.6	189.8	187.9	2312	64.92
12/4/2020 14:18	188.6	189.5	188	2312	64.92
12/4/2020 14:18	188.6	190.1	187.4	2312	65.04
12/4/2020 14:18	188.6	189.7	186.9	2311	64.92
12/4/2020 14:18	188.5	190.2	187	2310	65
12/4/2020 14:18	188.5	190	187.4	2310	65.04
12/4/2020 14:18	188.5	189.6	187.3	2311	65
12/4/2020 14:18	188.5	190.1	187.3	2310	64.96
12/4/2020 14:18	188.5	190.1	187	2308	65
12/4/2020 14:18	188.5	189.5	187.4	2313	65.04
12/4/2020 14:18	188.5	189.7	188.1	2312	64.92
12/4/2020 14:18	188.5	189.7	187	2312	65.04
12/4/2020 14:18	188.5	190.3	186.8	2312	65
12/4/2020 14:18	188.5	190.1	186.8	2312	65.12
12/4/2020 14:18	188.4	190.4	187	2306	64.88
12/4/2020 14:18	188.4	190.3	187.1	2308	64.92
12/4/2020 14:18	188.4	189.5	187.1	2308	64.88
12/4/2020 14:18	188.3	190.1	187.1	2311	64.88
12/4/2020 14:18	188.3	190.1	186.8	2310	64.88
12/4/2020 14:18	188.3	189.6	187	2313	65.08
12/4/2020 14:18	188.4	189.8	187.2	2315	64.88
12/4/2020 14:18	188.2	189.7	187.6	2315	65.08
12/4/2020 14:18	188.3	190.1	187.7	2316	65.12
12/4/2020 14:18	188.3	190.3	187.9	2310	64.91
12/4/2020 14:18	188.3	190.3	187.9	2310	64.92
12/4/2020 14:18	188.2	189.8	187.7	2311	64.97
12/4/2020 14:18	188.2	189.8	187.7	2313	64.88
12/4/2020 14:18	188.3	189.9	187.7	2312	64.92
12/4/2020 14:18	188.2	189.6	187.6	2311	65
12/4/2020 14:18	188.2	189.6	187.6	2308	64.91
12/4/2020 14:19	188.2	190.4	187.7	2312	64.92
12/4/2020 14:19	188.1	190.1	187.7	2315	64.96
12/4/2020 14:19	188.1	190	187.7	2310	64.96
12/4/2020 14:19	188.1	190.3	187.7	2311	64.96
12/4/2020 14:19	188.1	189.6	187.2	2310	65.09
12/4/2020 14:19	188.1	190.3	187	2305	64.96
12/4/2020 14:19	188	189.8	186.7	2308	65.04
12/4/2020 14:19	188.1	190.1	186.9	2307	65
12/4/2020 14:19	188	190.3	187	2306	64.87
12/4/2020 14:19	188	190	187.2	2308	64.96
12/4/2020 14:19	187.9	190.3	187.2	2306	65.08
12/4/2020 14:19	187.9	190.1	186.8	2304	65.08
12/4/2020 14:19	187.9	190.3	186.7	2304	64.88

12/4/2020 14:19	187.9	189.8	186.7	2304	64.96
12/4/2020 14:19	187.9	190	187.4	2301	64.96
12/4/2020 14:19	187.9	190.4	187.9	2301	64.88
12/4/2020 14:19	187.8	190	187.5	2301	64.99
12/4/2020 14:19	187.9	190.5	187	2304	64.93
12/4/2020 14:19	187.8	190.1	186.7	2304	65.08
12/4/2020 14:19	187.8	189.7	186.7	2305	64.84
12/4/2020 14:19	187.8	190.1	187	2309	65.12
12/4/2020 14:19	187.7	189.6	187	2306	64.88
12/4/2020 14:19	187.8	189.6	186.5	2306	64.92
12/4/2020 14:19	187.7	190.2	186.7	2305	65.12
12/4/2020 14:19	187.8	189.8	186.6	2303	64.99
12/4/2020 14:19	187.7	190.1	187.1	2300	64.88
12/4/2020 14:19	187.7	189.5	187.7	2305	64.96
12/4/2020 14:19	187.7	189.6	187.5	2308	64.83
12/4/2020 14:19	187.7	190.3	187	2312	64.92
12/4/2020 14:19	187.6	190.1	186.7	2313	65
12/4/2020 14:19	187.6	190.3	186.7	2311	64.96
12/4/2020 14:19	187.6	190.1	187	2308	64.96
12/4/2020 14:19	187.6	189.6	187	2308	65.04
12/4/2020 14:19	187.5	189.8	187	2306	64.87
12/4/2020 14:19	187.6	190	186.9	2307	64.96
12/4/2020 14:19	187.5	190.2	186.9	2310	65.04
12/4/2020 14:19	187.5	190.2	186.6	2309	65.04
12/4/2020 14:19	187.5	189.6	186.7	2311	65.04
12/4/2020 14:19	187.4	190.3	186.3	2313	65.09
12/4/2020 14:19	187.5	189.9	186.4	2308	65
12/4/2020 14:19	187.4	189.7	186.5	2308	64.92
12/4/2020 14:19	187.4	190.1	186.9	2310	65.04
12/4/2020 14:19	187.4	189.6	187.2	2306	65.12
12/4/2020 14:19	187.4	190.2	187.4	2310	64.95
12/4/2020 14:19	187.4	190.1	187.4	2310	65.04
12/4/2020 14:19	187.3	189.6	187.5	2310	64.93
12/4/2020 14:19	187.3	189.7	187.4	2309	64.84
12/4/2020 14:19	187.2	190.1	187.2	2308	64.99
12/4/2020 14:19	187.2	190.3	187	2310	64.92
12/4/2020 14:19	187.2	189.8	187	2308	64.99
12/4/2020 14:19	187.3	189.6	187.2	2308	64.92
12/4/2020 14:19	187.2	190.3	187.3	2308	65.08
12/4/2020 14:19	187.3	190	187.4	2310	64.87
12/4/2020 14:19	187.2	190.4	187.5	2306	64.88
12/4/2020 14:19	187.2	189.8	187.3	2306	64.97
12/4/2020 14:19	187.2	189.7	187.5	2310	64.88
12/4/2020 14:19	187.2	189.1	187	2310	65.04
12/4/2020 14:19	187.2	189.1	186.8	2310	64.92
12/4/2020 14:19	187.1	189.1	186.4	2311	64.92
12/4/2020 14:20	187.1	188.8	186.2	2311	64.88

12/4/2020 14:20	187.2	188.8	186.2	2314	64.96
12/4/2020 14:20	187.1	188.9	186.5	2311	64.92
12/4/2020 14:20	187.1	188.8	186.5	2312	65.04
12/4/2020 14:20	187.1	189.3	186.5	2309	65.08
12/4/2020 14:20	187.1	188.8	186.7	2308	64.96
12/4/2020 14:20	187.1	189.1	186.7	2305	64.95
12/4/2020 14:20	187	189.1	186.6	2306	65.04
12/4/2020 14:20	187	188.6	186.3	2308	64.99
12/4/2020 14:20	187	189	186.2	2307	64.92
12/4/2020 14:20	187	189	186.6	2306	65.08
12/4/2020 14:20	187	189.1	187	2306	64.87
12/4/2020 14:20	187	188.9	187.4	2308	64.96
12/4/2020 14:20	187	188.8	187.1	2310	65.04
12/4/2020 14:20	187	189.1	187	2310	65
12/4/2020 14:20	186.9	189	186.7	2308	64.96
12/4/2020 14:20	187	188.6	186.4	2308	65.04
12/4/2020 14:20	187	189.2	186.2	2309	64.96
12/4/2020 14:20	186.9	188.9	186.4	2306	64.92
12/4/2020 14:20	186.9	188.8	186.1	2305	65
12/4/2020 14:20	186.8	189.1	186.4	2306	65
12/4/2020 14:20	186.9	188.6	186.4	2306	64.96
12/4/2020 14:20	186.8	189.3	186.3	2311	64.96
12/4/2020 14:20	186.8	188.9	186.5	2310	64.96
12/4/2020 14:20	186.8	189	186.5	2311	64.88
12/4/2020 14:20	186.8	188.8	186.5	2310	64.92
12/4/2020 14:20	186.8	189.3	186.5	2312	64.88
12/4/2020 14:20	186.8	188.9	186.6	2309	65
12/4/2020 14:20	186.8	188.8	186.5	2309	64.97
12/4/2020 14:20	186.8	189	186.6	2310	65.04
12/4/2020 14:20	186.8	188.7	186.5	2308	64.92
12/4/2020 14:20	186.8	189	186.5	2306	64.88
12/4/2020 14:20	186.8	189.2	186.5	2313	65.12
12/4/2020 14:20	186.8	188.9	186.4	2310	64.83
12/4/2020 14:20	186.8	188.8	186.6	2307	65.03
12/4/2020 14:20	186.7	189.2	186.5	2310	65.08
12/4/2020 14:20	186.8	189.3	186.7	2305	64.83
12/4/2020 14:20	186.8	189.1	186.4	2306	64.96
12/4/2020 14:20	186.7	188.8	186.5	2308	64.97
12/4/2020 14:20	186.8	189.1	186.4	2308	64.88
12/4/2020 14:20	186.8	189.2	186.5	2308	64.88
12/4/2020 14:20	186.8	188.8	186.2	2308	65.12
12/4/2020 14:20	186.7	189.2	186.6	2306	64.83
12/4/2020 14:20	186.7	188.9	186.2	2304	64.99
12/4/2020 14:20	186.7	188.8	186.4	2306	65
12/4/2020 14:20	186.7	189.3	186.2	2305	65.04
12/4/2020 14:20	186.7	188.7	186.1	2305	64.92
12/4/2020 14:20	186.7	189.1	186.2	2307	65

12/4/2020 14:20	186.7	188.9	186	2304	64.99
12/4/2020 14:20	186.7	189.1	186.2	2303	64.96
12/4/2020 14:20	186.6	189.1	186.1	2302	65.04
12/4/2020 14:20	186.7	188.8	186.3	2300	65
12/4/2020 14:20	186.6	189.1	186.6	2302	65.04
12/4/2020 14:20	186.7	188.6	187.1	2303	64.92
12/4/2020 14:20	186.7	188.8	186.8	2308	65
12/4/2020 14:20	186.7	189.2	186.2	2306	64.96
12/4/2020 14:20	186.7	188.7	186.2	2303	65
12/4/2020 14:20	186.6	188.9	186.3	2298	64.96
12/4/2020 14:20	186.6	188.6	186.4	2297	64.96
12/4/2020 14:20	186.6	189.2	186.2	2297	65
12/4/2020 14:21	186.5	188.9	186.5	2297	65
12/4/2020 14:21	186.6	189	185.9	2294	65.04
12/4/2020 14:21	186.6	189	185.9	2292	65
12/4/2020 14:21	186.6	188.8	186.7	2294	65.09
12/4/2020 14:21	186.6	188.8	187	2289	64.83
12/4/2020 14:21	186.6	189	186.9	2297	65.08
12/4/2020 14:21	186.6	188.9	186.3	2300	65.08
12/4/2020 14:21	186.7	188.9	186.1	2294	64.83
12/4/2020 14:21	186.6	189.1	186.1	2296	65
12/4/2020 14:21	186.6	188.7	186.4	2293	64.93
12/4/2020 14:21	186.5	188.9	186.2	2292	64.76
12/4/2020 14:21	186.6	188.7	186.4	2290	65.08
12/4/2020 14:21	186.6	188.9	186.3	2294	65.08
12/4/2020 14:21	186.6	188.6	186.2	2289	64.99
12/4/2020 14:21	186.6	188.6	186.2	2290	64.84
12/4/2020 14:21	186.6	189.2	186	2292	65.12
12/4/2020 14:21	186.6	188.8	185.8	2292	64.99
12/4/2020 14:21	186.6	188.8	186.1	2290	64.96
12/4/2020 14:21	186.6	189.1	185.7	2290	65.01
12/4/2020 14:21	186.6	189	186	2292	64.88
12/4/2020 14:21	186.6	189.2	186.1	2292	65.04
12/4/2020 14:21	186.7	189	186	2292	65.04
12/4/2020 14:21	186.6	188.8	186	2294	64.99
12/4/2020 14:21	186.7	189.1	186.2	2293	64.96
12/4/2020 14:21	186.7	188.7	186	2293	65.01
12/4/2020 14:21	186.8	189.2	186.1	2291	64.92
12/4/2020 14:21	186.7	188.8	185.8	2293	64.99
12/4/2020 14:21	186.7	189.2	186	2294	65
12/4/2020 14:21	186.8	189.1	185.9	2297	64.96
12/4/2020 14:21	186.8	188.8	185.9	2291	64.99
12/4/2020 14:21	186.8	188.9	186.2	2290	64.92
12/4/2020 14:21	186.8	189.1	186.2	2288	65.08
12/4/2020 14:21	186.8	188.1	186.2	2292	64.88
12/4/2020 14:21	186.8	188.2	186.4	2289	64.96
12/4/2020 14:21	186.8	188.1	186.2	2293	65

12/4/2020 14:21	186.8	188.1	186.1	2293	64.92
12/4/2020 14:21	186.9	188.1	186.2	2294	65
12/4/2020 14:21	186.8	188.1	186.6	2294	65.04
12/4/2020 14:21	186.8	188	186.8	2291	64.91
12/4/2020 14:21	187	188.2	186	2289	64.96
12/4/2020 14:21	186.9	188	185.9	2296	65
12/4/2020 14:21	187	188.2	186.2	2296	64.83
12/4/2020 14:21	187	188.1	186.2	2293	64.96
12/4/2020 14:21	187	188.1	186.2	2293	65.04
12/4/2020 14:21	186.9	187.9	186.1	2293	64.96
12/4/2020 14:21	187	188.1	186.5	2291	64.99
12/4/2020 14:21	186.9	188.1	186.8	2295	64.92
12/4/2020 14:21	186.9	187.8	186	2304	64.99
12/4/2020 14:21	186.9	188.1	186	2304	65.04
12/4/2020 14:21	187	188.1	186.2	2304	64.93
12/4/2020 14:21	187	188.2	186.1	2305	65.04
12/4/2020 14:21	187	188.2	186	2308	65.08
12/4/2020 14:21	186.9	188.1	186.2	2314	65.08
12/4/2020 14:21	187	188	186.7	2311	65.04
12/4/2020 14:21	186.9	188	186	2308	64.99
12/4/2020 14:21	186.9	188.1	185.6	2304	64.92
12/4/2020 14:21	186.9	188	186.2	2297	65.04
12/4/2020 14:21	186.9	188.1	186.1	2299	64.83
12/4/2020 14:21	187	188	185.9	2301	64.92
12/4/2020 14:22	186.9	188.1	186.1	2301	65.08
12/4/2020 14:22	187	188.2	186.6	2303	64.83
12/4/2020 14:22	186.9	188	186.4	2303	64.99
12/4/2020 14:22	186.9	188.1	185.8	2305	65.04
12/4/2020 14:22	186.8	188.1	185.9	2307	64.95
12/4/2020 14:22	186.8	188.1	186	2308	64.96
12/4/2020 14:22	186.8	188.1	186.3	2304	65.01
12/4/2020 14:22	186.8	188.1	185.7	2305	64.95
12/4/2020 14:22	186.8	187.9	186.2	2308	64.96
12/4/2020 14:22	186.8	188	186.7	2308	65.08
12/4/2020 14:22	186.8	188	186.2	2304	64.91
12/4/2020 14:22	186.8	188	185.7	2307	65
12/4/2020 14:22	186.8	187.9	186.1	2307	65.04
12/4/2020 14:22	186.8	188.1	186.2	2309	64.91
12/4/2020 14:22	186.8	187.9	185.9	2308	64.92
12/4/2020 14:22	186.8	187.9	185.8	2310	64.96
12/4/2020 14:22	186.7	188	186.3	2311	65
12/4/2020 14:22	186.8	187.9	187	2312	65
12/4/2020 14:22	186.7	188.1	186.1	2312	64.96
12/4/2020 14:22	186.8	188.1	185.7	2312	64.96
12/4/2020 14:22	186.8	188	185.6	2312	64.91
12/4/2020 14:22	186.7	188.1	185.7	2311	65
12/4/2020 14:22	186.7	188.1	186	2308	65

12/4/2020 14:22	186.7	188.1	186	2312	64.92
12/4/2020 14:22	186.7	188	186	2312	64.92
12/4/2020 14:22	186.7	188.1	185.8	2315	64.84
12/4/2020 14:22	186.7	187.9	186.2	2314	65.04
12/4/2020 14:22	186.7	188.1	186.5	2312	64.92
12/4/2020 14:22	186.6	188.1	186.3	2312	65.08
12/4/2020 14:22	186.6	188.1	186	2305	64.88
12/4/2020 14:22	186.7	187.9	185.6	2308	65.12
12/4/2020 14:22	186.6	187.9	185.7	2306	64.92
12/4/2020 14:22	186.6	188.1	185.9	2303	65.03
12/4/2020 14:22	186.6	188	186	2303	65.04
12/4/2020 14:22	186.6	188.1	185.8	2308	64.96
12/4/2020 14:22	186.7	188	186.1	2310	65.08
12/4/2020 14:22	186.6	188.1	186	2310	64.88
12/4/2020 14:22	186.6	188.1	185.7	2313	65.04
12/4/2020 14:22	186.6	188	185.8	2311	64.83
12/4/2020 14:22	186.6	187.9	185.9	2310	64.99
12/4/2020 14:22	186.6	188	186	2307	64.96
12/4/2020 14:22	186.7	188.2	186.5	2308	64.99
12/4/2020 14:22	186.6	188	186.4	2310	65.03
12/4/2020 14:22	186.6	188	186.7	2315	65.01
12/4/2020 14:22	186.5	188	186.7	2310	64.99
12/4/2020 14:22	186.6	188.1	186.5	2308	65
12/4/2020 14:22	186.5	188.1	186.8	2306	65.08
12/4/2020 14:22	186.6	188.1	186.6	2304	64.99
12/4/2020 14:22	186.5	188.1	186.5	2308	64.99
12/4/2020 14:22	186.5	188	186.3	2308	65.08
12/4/2020 14:22	186.6	188.1	186.2	2310	64.96
12/4/2020 14:22	186.5	187.9	186.1	2309	64.96
12/4/2020 14:22	186.5	187.9	186	2314	65
12/4/2020 14:22	186.6	188	185.8	2316	64.92
12/4/2020 14:22	186.5	188	185.6	2313	64.96
12/4/2020 14:22	186.5	187.9	185.5	2316	65
12/4/2020 14:22	186.5	188	185.5	2315	64.92
12/4/2020 14:22	186.5	188	185.3	2310	65
12/4/2020 14:22	186.5	188	185.5	2311	65
12/4/2020 14:22	186.5	187.9	185.7	2311	64.96
12/4/2020 14:23	186.4	188.1	185.8	2307	64.87
12/4/2020 14:23	186.5	188	186	2306	64.92
12/4/2020 14:23	186.5	188.1	185.8	2311	64.96
12/4/2020 14:23	186.5	188.2	185.8	2310	64.96
12/4/2020 14:23	186.4	188.1	185.8	2313	64.92
12/4/2020 14:23	186.4	188.1	185.9	2313	65.12
12/4/2020 14:23	186.4	188.2	185.8	2317	65
12/4/2020 14:23	186.4	188.1	186	2315	64.96
12/4/2020 14:23	186.4	187.9	185.7	2318	65.16
12/4/2020 14:23	186.4	187.9	185.5	2315	64.99

12/4/2020 14:23	186.4	188	185.8	2312	64.96
12/4/2020 14:23	186.4	187.9	185.4	2314	65.16
12/4/2020 14:23	186.3	188	185.4	2312	65.04
12/4/2020 14:23	186.3	187.9	185.4	2311	64.92
12/4/2020 14:23	186.4	188.1	185.3	2309	65.08
12/4/2020 14:23	186.3	187.9	185.4	2312	65.03
12/4/2020 14:23	186.3	188.1	185.4	2308	65.12
12/4/2020 14:23	186.4	188.1	185.6	2311	65.05
12/4/2020 14:23	186.4	188.1	185.6	2308	64.92
12/4/2020 14:23	186.4	188	185.5	2307	65.04
12/4/2020 14:23	186.3	188	185.3	2309	65.04
12/4/2020 14:23	186.3	188.1	185.3	2308	65
12/4/2020 14:23	186.3	188.1	185.3	2305	65.04
12/4/2020 14:23	186.3	188	185.4	2308	65.09
12/4/2020 14:23	186.3	187.9	185.3	2309	65
12/4/2020 14:23	186.3	188	185.4	2307	65.04
12/4/2020 14:23	186.4	188	185.5	2304	65.08
12/4/2020 14:23	186.3	188	185.5	2305	64.92
12/4/2020 14:23	186.3	188.1	185.7	2306	64.96
12/4/2020 14:23	186.3	188.2	185.7	2311	65.08
12/4/2020 14:23	186.3	188.1	185.4	2312	65.12
12/4/2020 14:23	186.3	188.1	185.8	2311	65.04
12/4/2020 14:23	186.3	187.9	185.5	2310	65.08
12/4/2020 14:23	186.3	187.9	185.3	2311	64.96
12/4/2020 14:23	186.3	188.1	185.7	2313	64.95
12/4/2020 14:23	186.3	188	186.1	2311	64.84
12/4/2020 14:23	186.3	188	186.2	2309	65.08
12/4/2020 14:23	186.3	188.2	185.7	2310	65.04
12/4/2020 14:23	186.3	188.1	185.2	2308	65
12/4/2020 14:23	186.3	187.9	185.3	2312	65.12
12/4/2020 14:23	186.3	187.9	185.2	2308	64.96
12/4/2020 14:23	186.3	188.1	185.6	2306	65.04
12/4/2020 14:23	186.3	188.2	185.7	2308	65.04
12/4/2020 14:23	186.3	188	185.4	2306	65
12/4/2020 14:23	186.3	188.1	185.4	2306	65.05
12/4/2020 14:23	186.3	188.1	185.8	2307	64.96
12/4/2020 14:23	186.3	188.1	186.1	2308	64.92
12/4/2020 14:23	186.3	188.1	185.7	2308	64.96
12/4/2020 14:23	186.3	188	185.1	2309	65
12/4/2020 14:23	186.3	188.1	185.2	2308	65.04
12/4/2020 14:23	186.3	188.1	185.6	2306	65
12/4/2020 14:23	186.3	188.2	185.4	2306	64.96
12/4/2020 14:23	186.3	187.9	185.4	2308	65.08
12/4/2020 14:23	186.3	188.1	185.6	2306	64.92
12/4/2020 14:23	186.3	188.1	186.3	2306	65.12
12/4/2020 14:23	186.3	188.1	186	2304	64.92
12/4/2020 14:23	186.3	188.1	185	2305	65.08

12/4/2020 14:23	186.3	188.1	185.5	2306	64.88
12/4/2020 14:23	186.3	188.1	185.8	2306	65.01
12/4/2020 14:24	186.3	188.2	185.3	2310	64.8
12/4/2020 14:24	186.3	188.1	185.3	2308	65.03
12/4/2020 14:24	186.3	188	185.8	2307	65.08
12/4/2020 14:24	186.3	186.9	186.2	2301	64.92
12/4/2020 14:24	186.3	187	185.5	2297	65.04
12/4/2020 14:24	186.2	187.4	184.9	2304	65.08
12/4/2020 14:24	186.2	186.8	185.1	2303	65
12/4/2020 14:24	186.3	187.2	185.3	2300	65.04
12/4/2020 14:24	186.2	187	185.4	2301	65.04
12/4/2020 14:24	186.2	186.9	185.4	2300	64.96
12/4/2020 14:24	186.3	187.2	185.2	2301	64.96
12/4/2020 14:24	186.2	187	185.1	2310	65.13
12/4/2020 14:24	186.3	187	185.6	2308	65
12/4/2020 14:24	186.3	187	186	2306	64.96
12/4/2020 14:24	186.2	186.9	186.1	2306	65.04
12/4/2020 14:24	186.2	187.4	186	2303	64.88
12/4/2020 14:24	186.2	186.8	185.7	2301	65.08
12/4/2020 14:24	186.2	187.2	185.9	2299	65.12
12/4/2020 14:24	186.2	187	185.6	2294	64.99
12/4/2020 14:24	186.3	186.8	185.7	2295	64.96
12/4/2020 14:24	186.2	187.2	185.7	2293	65.01
12/4/2020 14:24	186.2	187	185.8	2294	64.99
12/4/2020 14:24	186.2	187.2	186	2297	65.08
12/4/2020 14:24	186.2	187.2	186.1	2299	64.96
12/4/2020 14:24	186.3	186.8	185.9	2296	64.99
12/4/2020 14:24	186.3	187.2	186	2298	65.08
12/4/2020 14:24	186.3	187	185.8	2301	65.12
12/4/2020 14:24	186.2	187.2	185.8	2297	65.04
12/4/2020 14:24	186.3	187.2	185.8	2299	65.04
12/4/2020 14:24	186.3	186.7	185.3	2297	65.04
12/4/2020 14:24	186.3	186.8	185.5	2299	65
12/4/2020 14:24	186.3	186.7	185.3	2299	64.92
12/4/2020 14:24	186.3	187.1	185.2	2301	64.96
12/4/2020 14:24	186.3	187.2	185.1	2302	65.09
12/4/2020 14:24	186.3	187	185	2294	64.96
12/4/2020 14:24	186.3	187	184.8	2297	65
12/4/2020 14:24	186.3	186.8	185.1	2299	65.01
12/4/2020 14:24	186.3	187	184.9	2293	64.99
12/4/2020 14:24	186.4	187.2	185.1	2295	65
12/4/2020 14:24	186.3	186.7	185.1	2300	65.05
12/4/2020 14:24	186.3	187.2	185.1	2297	64.84
12/4/2020 14:24	186.4	187.1	185.4	2293	65.08
12/4/2020 14:24	186.4	187	185.1	2297	65.12
12/4/2020 14:24	186.5	187.2	185.1	2299	64.84
12/4/2020 14:24	186.5	187	184.8	2296	64.92

12/4/2020 14:24	186.4	187.2	184.7	2297	65.04
12/4/2020 14:24	186.5	187	185.3	2301	64.93
12/4/2020 14:24	186.6	186.9	186.1	2303	64.92
12/4/2020 14:24	186.5	187.2	185.3	2299	64.88
12/4/2020 14:24	186.5	187	185	2300	65.08
12/4/2020 14:24	186.5	187.2	185.1	2299	64.88
12/4/2020 14:24	186.6	187	185.2	2299	64.87
12/4/2020 14:24	186.6	187.2	184.9	2306	65.17
12/4/2020 14:24	186.6	187	184.8	2303	64.83
12/4/2020 14:24	186.6	186.7	185	2296	65.04
12/4/2020 14:24	186.6	187.1	185.8	2297	65.05
12/4/2020 14:24	186.6	187.1	185.6	2301	64.92
12/4/2020 14:24	186.6	187.2	185.2	2297	64.96
12/4/2020 14:24	186.7	187	184.9	2299	65.09
12/4/2020 14:25	186.7	186.8	185.2	2296	65
12/4/2020 14:25	186.7	187	185.4	2298	65
12/4/2020 14:25	186.7	187.2	185.1	2301	65
12/4/2020 14:25	186.7	187.2	185.1	2304	65
12/4/2020 14:25	186.7	187.4	184.9	2301	65
12/4/2020 14:25	186.7	186.9	185.2	2301	65.08
12/4/2020 14:25	186.7	187	186.1	2304	64.84
12/4/2020 14:25	186.7	187.2	186	2303	64.88
12/4/2020 14:25	186.7	186.9	185.4	2302	64.96
12/4/2020 14:25	186.7	187	184.9	2300	65.08
12/4/2020 14:25	186.7	186.7	184.8	2300	64.93
12/4/2020 14:25	186.7	187	185.1	2303	64.92
12/4/2020 14:25	186.8	187.3	185.3	2301	64.93
12/4/2020 14:25	186.7	187.2	185.3	2299	65.08
12/4/2020 14:25	186.7	187	185.2	2298	64.88
12/4/2020 14:25	186.7	186.8	185.1	2299	64.93
12/4/2020 14:25	186.7	187.2	185.3	2297	64.83
12/4/2020 14:25	186.7	187.4	185.3	2297	64.88
12/4/2020 14:25	186.7	186.9	184.9	2301	65.09
12/4/2020 14:25	186.7	187.2	185.3	2306	65.04
12/4/2020 14:25	186.7	186.8	185.2	2303	65
12/4/2020 14:25	186.7	187	184.9	2304	64.96
12/4/2020 14:25	186.7	186.7	185	2301	65
12/4/2020 14:25	186.7	187.1	184.9	2303	65.04
12/4/2020 14:25	186.6	187.2	184.9	2305	65.04
12/4/2020 14:25	186.7	186.8	184.9	2302	65
12/4/2020 14:25	186.7	187.3	184.9	2297	64.96
12/4/2020 14:25	186.6	186.7	185.5	2299	65.04
12/4/2020 14:25	186.7	186.8	185.6	2302	64.96
12/4/2020 14:25	186.6	187.2	185.8	2305	64.96
12/4/2020 14:25	186.6	187	185.3	2304	65.05
12/4/2020 14:25	186.6	186.9	184.8	2308	65
12/4/2020 14:25	186.5	186.9	184.8	2308	64.96

12/4/2020 14:25	186.6	186.9	184.9	2306	64.92
12/4/2020 14:25	186.6	187	184.9	2306	65.08
12/4/2020 14:25	186.6	186.9	185.2	2302	64.92
12/4/2020 14:25	186.5	187.3	185	2306	64.92
12/4/2020 14:25	186.5	187.2	184.8	2310	65.12
12/4/2020 14:25	186.5	186.7	185.5	2311	64.88
12/4/2020 14:25	186.5	187	185.6	2312	64.92
12/4/2020 14:25	186.5	187	184.8	2310	65.12
12/4/2020 14:25	186.5	187.2	184.6	2314	64.84
12/4/2020 14:25	186.4	186.9	184.9	2310	64.96
12/4/2020 14:25	186.5	186.9	185.1	2314	65.08
12/4/2020 14:25	186.5	187	184.6	2318	65
12/4/2020 14:25	186.5	187	185.4	2313	64.92
12/4/2020 14:25	186.5	187	185.2	2311	65.04
12/4/2020 14:25	186.5	187.2	184.4	2312	65
12/4/2020 14:25	186.4	186.7	184.8	2310	65
12/4/2020 14:25	186.4	187	184.9	2314	65.05
12/4/2020 14:25	186.4	187	184.9	2310	65
12/4/2020 14:25	186.4	186.7	185	2310	65
12/4/2020 14:25	186.4	187.3	185.6	2310	65.04
12/4/2020 14:25	186.4	186.8	185.1	2311	64.96
12/4/2020 14:25	186.4	186.9	184.4	2308	65
12/4/2020 14:25	186.4	187	184.6	2308	64.88
12/4/2020 14:25	186.4	186.9	184.9	2310	65.01
12/4/2020 14:25	186.4	187.4	184.9	2306	64.84
12/4/2020 14:25	186.3	186.8	184.6	2308	64.96
12/4/2020 14:25	186.3	187	185.1	2314	65.08
12/4/2020 14:26	186.3	186.9	185.5	2312	65.07
12/4/2020 14:26	186.3	187.2	185.7	2313	64.96
12/4/2020 14:26	186.3	186.8	184.9	2317	65.16
12/4/2020 14:26	186.4	186.7	184.4	2310	65.08
12/4/2020 14:26	186.4	187	184.6	2309	65.08
12/4/2020 14:26	186.3	186.7	184.6	2315	65.04
12/4/2020 14:26	186.4	187.3	185.1	2315	65.04
12/4/2020 14:26	186.4	187	185	2312	65.04
12/4/2020 14:26	186.3	186.7	184.9	2309	65.08
12/4/2020 14:26	186.3	187	184.8	2310	65.04
12/4/2020 14:26	186.3	186.7	184.6	2311	65
12/4/2020 14:26	186.3	187.3	184.8	2315	65
12/4/2020 14:26	186.3	187.2	184.9	2314	65.05
12/4/2020 14:26	186.2	186.8	184.9	2311	64.96
12/4/2020 14:26	186.3	187	184.6	2309	64.92
12/4/2020 14:26	186.3	187.2	184.9	2311	65.05
12/4/2020 14:26	186.3	187.2	184.7	2313	64.88
12/4/2020 14:26	186.3	187.2	184.9	2310	65
12/4/2020 14:26	186.3	186.7	184.9	2312	65.01
12/4/2020 14:26	186.2	186.9	184.7	2313	64.84

12/4/2020 14:26	186.2	187.2	184.9	2311	64.92
12/4/2020 14:26	186.3	186.8	184.6	2314	64.96
12/4/2020 14:26	186.2	187.2	184.3	2312	65.12
12/4/2020 14:26	186.3	187	184.5	2311	64.88
12/4/2020 14:26	186.3	186.8	185.1	2312	65.04
12/4/2020 14:26	186.3	186.7	185.6	2315	64.92
12/4/2020 14:26	186.3	187.1	184.9	2312	65.08
12/4/2020 14:26	186.2	187.1	184.4	2313	65.09
12/4/2020 14:26	186.2	187	184.8	2311	64.99
12/4/2020 14:26	186.2	187	184.9	2308	65.04
12/4/2020 14:26	186.2	187	184.6	2312	65.12
12/4/2020 14:26	186.2	186.8	184.6	2312	64.96
12/4/2020 14:26	186.2	186.9	184.4	2310	65.04
12/4/2020 14:26	186.2	186.8	185.4	2315	65.04
12/4/2020 14:26	186.2	187.2	185.2	2317	65.04
12/4/2020 14:26	186.2	186.8	184.4	2313	64.92
12/4/2020 14:26	186.2	187	184.6	2313	64.92
12/4/2020 14:26	186.2	187.1	184.6	2315	65.08
12/4/2020 14:26	186.2	187	184.6	2308	64.96
12/4/2020 14:26	186.2	187.3	184.5	2304	65.04
12/4/2020 14:26	186.2	186.9	184.2	2308	65.01
12/4/2020 14:26	186.2	186.8	184.3	2306	64.99
12/4/2020 14:26	186.2	187	185.2	2304	65.08
12/4/2020 14:26	186.2	186.7	185.4	2308	64.93
12/4/2020 14:26	186.2	186.7	185.2	2308	64.83
12/4/2020 14:26	186.2	187.2	184.6	2311	64.92
12/4/2020 14:26	186.2	186.8	184.3	2312	65.08
12/4/2020 14:26	186.2	187.1	184.3	2309	65.04
12/4/2020 14:26	186.2	186.8	184.5	2308	64.83
12/4/2020 14:26	186.2	187.2	184.7	2314	65.12
12/4/2020 14:26	186.2	187.3	184.8	2316	64.92
12/4/2020 14:26	186.2	187	184.6	2314	64.96
12/4/2020 14:26	186.1	186.8	184.8	2315	65.12
12/4/2020 14:26	186.2	187	184.6	2313	64.92
12/4/2020 14:26	186.2	187.2	184.8	2310	65.04
12/4/2020 14:26	186.2	187.2	184.8	2308	65.04
12/4/2020 14:26	186.2	187	184.6	2308	64.96
12/4/2020 14:26	186.2	187.4	184.9	2309	64.96
12/4/2020 14:26	186.2	186.8	184.6	2311	64.96
12/4/2020 14:27	186.1	186.9	184.6	2310	65
12/4/2020 14:27	186.2	187.1	184.8	2312	64.96
12/4/2020 14:27	186.2	187	184.7	2314	64.96
12/4/2020 14:27	186.2	186.8	184.6	2316	65
12/4/2020 14:27	186.2	186.9	184.4	2316	64.96
12/4/2020 14:27	186.2	186.8	184.4	2318	65
12/4/2020 14:27	186.1	187.3	184.4	2319	65
12/4/2020 14:27	186.2	187	184.4	2319	64.91

12/4/2020 14:27	186.2	187	184.2	2318	64.92
12/4/2020 14:27	186.2	187.2	184.6	2317	65.04
12/4/2020 14:27	186.2	186.7	184.4	2318	64.92
12/4/2020 14:27	186.2	187	184.6	2312	64.88
12/4/2020 14:27	186.2	187.2	185	2316	65.09
12/4/2020 14:27	186.2	187.1	185.1	2318	64.99
12/4/2020 14:27	186.2	187.2	185.5	2320	65.04
12/4/2020 14:27	186.2	186.8	185.3	2321	65.08
12/4/2020 14:27	186.2	187.3	185.4	2317	64.99
12/4/2020 14:27	186.2	187.2	185.3	2312	64.92
12/4/2020 14:27	186.2	187.1	184.8	2309	64.88
12/4/2020 14:27	186.2	187.2	184.5	2314	64.93
12/4/2020 14:27	186.3	186.8	184.3	2314	64.83
12/4/2020 14:27	186.2	187.2	184.2	2316	64.95
12/4/2020 14:27	186.3	186.8	184.1	2313	65.12
12/4/2020 14:27	186.3	187	184.1	2312	65.04
12/4/2020 14:27	186.3	187.2	184	2308	64.88
12/4/2020 14:27	186.3	186.8	184.6	2311	65.08
12/4/2020 14:27	186.3	187	184.4	2315	65
12/4/2020 14:27	186.3	186.8	184.5	2315	64.92
12/4/2020 14:27	186.3	187	184.6	2315	65.08
12/4/2020 14:27	186.3	187.1	184.5	2312	64.88
12/4/2020 14:27	186.3	186.8	184.6	2318	64.91
12/4/2020 14:27	186.3	187	184.6	2322	65
12/4/2020 14:27	186.2	187.2	184.4	2318	64.87
12/4/2020 14:27	186.3	187	184.4	2315	64.92
12/4/2020 14:27	186.3	187	184.2	2317	65.08
12/4/2020 14:27	186.3	186.9	184.4	2318	64.92
12/4/2020 14:27	186.3	187.1	184.3	2317	65
12/4/2020 14:27	186.4	187	184.1	2317	65
12/4/2020 14:27	186.3	186.8	184.9	2320	64.96
12/4/2020 14:27	186.3	187.2	185.3	2320	64.92
12/4/2020 14:27	186.4	186.6	185.2	2322	64.96
12/4/2020 14:27	186.4	187.1	185.1	2325	64.96
12/4/2020 14:27	186.3	187.2	184.1	2320	64.96
12/4/2020 14:27	186.4	186.9	184.1	2316	64.92
12/4/2020 14:27	186.4	187.1	184.2	2320	65.04
12/4/2020 14:27	186.4	187	183.9	2317	64.96
12/4/2020 14:27	186.4	186.9	184.5	2315	64.92
12/4/2020 14:27	186.4	186.8	184.4	2315	65.08
12/4/2020 14:27	186.4	186.7	184.2	2318	64.99
12/4/2020 14:27	186.4	187.2	184.2	2319	64.92
12/4/2020 14:27	186.5	186.8	184.8	2320	65.04
12/4/2020 14:27	186.4	186.8	185.2	2321	64.99
12/4/2020 14:27	186.4	187.4	184.2	2320	65.04
12/4/2020 14:27	186.5	186.7	184	2324	65.01
12/4/2020 14:27	186.5	187.2	184.4	2321	65.08

12/4/2020 14:27	186.5	187	184.4	2320	65
12/4/2020 14:27	186.5	187.4	184.4	2316	65.08
12/4/2020 14:27	186.6	187	184.1	2318	64.88
12/4/2020 14:27	186.5	186.7	184.8	2317	64.88
12/4/2020 14:27	186.6	187.1	184.9	2313	65.04
12/4/2020 14:28	186.5	187	184	2313	64.92
12/4/2020 14:28	186.6	187.3	184.2	2310	64.83
12/4/2020 14:28	186.5	187.2	184.6	2310	64.93
12/4/2020 14:28	186.5	187	184.1	2315	65.12
12/4/2020 14:28	186.5	187.1	184.2	2318	64.99
12/4/2020 14:28	186.5	187	185.3	2317	65.08
12/4/2020 14:28	186.5	186.7	184.3	2315	65.08
12/4/2020 14:28	186.5	186.8	183.9	2317	64.88
12/4/2020 14:28	186.6	186.8	184.5	2318	65.08
12/4/2020 14:28	186.5	187.2	184.6	2320	65.04
12/4/2020 14:28	186.6	187	184.4	2315	64.96
12/4/2020 14:28	186.5	186.9	184.1	2312	64.99
12/4/2020 14:28	186.5	187	184.9	2310	65
12/4/2020 14:28	186.5	186.8	184.4	2312	65
12/4/2020 14:28	186.6	187.2	184	2310	64.92
12/4/2020 14:28	186.5	187.2	184.4	2314	64.96
12/4/2020 14:28	186.6	186.8	184.3	2314	65.04
12/4/2020 14:28	186.6	187	184.1	2314	64.92
12/4/2020 14:28	186.6	186.8	184.2	2315	65
12/4/2020 14:28	186.6	187	184.9	2315	65.04
12/4/2020 14:28	186.6	187.1	184.8	2317	64.96
12/4/2020 14:28	186.6	187	183.8	2311	65
12/4/2020 14:28	186.6	187	184.2	2312	65
12/4/2020 14:28	186.6	186.9	184.4	2317	64.87
12/4/2020 14:28	186.6	186.8	184.3	2315	64.96
12/4/2020 14:28	186.6	187.2	183.9	2312	65
12/4/2020 14:28	186.6	186.7	184.2	2312	64.96
12/4/2020 14:28	186.6	187.2	184.8	2311	64.91
12/4/2020 14:28	186.6	187	184.4	2313	64.88
12/4/2020 14:28	186.6	187.4	183.8	2311	64.92
12/4/2020 14:28	186.6	187.4	183.7	2311	64.87
12/4/2020 14:28	186.6	186.9	184.2	2315	64.88
12/4/2020 14:28	186.6	187.1	184.3	2314	65.04
12/4/2020 14:28	186.5	186.8	184.3	2312	64.92
12/4/2020 14:28	186.6	186.7	184.3	2313	64.83
12/4/2020 14:28	186.5	187.2	183.9	2310	65.08
12/4/2020 14:28	186.6	186.8	184.1	2310	65.04
12/4/2020 14:28	186.5	187	184.1	2309	64.88
12/4/2020 14:28	186.6	186.6	184.3	2310	65.04
12/4/2020 14:28	186.5	187.1	184.8	2310	64.91
12/4/2020 14:28	186.5	187.4	184.6	2308	64.96
12/4/2020 14:28	186.6	187	184.8	2311	64.96

12/4/2020 14:28	186.6	186.9	185.1	2312	64.95
12/4/2020 14:28	186.6	187.2	184.7	2312	65.08
12/4/2020 14:28	186.6	187	184.8	2314	65
12/4/2020 14:28	186.6	187.3	184.7	2315	64.76
12/4/2020 14:28	186.6	187	184.4	2314	64.96
12/4/2020 14:28	186.5	187.2	184.1	2313	64.96
12/4/2020 14:28	186.5	186.8	183.9	2315	64.8
12/4/2020 14:28	186.5	186.7	184	2313	64.76
12/4/2020 14:28	186.5	187.2	184.4	2313	64.97
12/4/2020 14:28	186.4	186.8	184.1	2315	64.88
12/4/2020 14:28	186.5	186.8	184.1	2312	64.84
12/4/2020 14:28	186.5	187.3	183.8	2315	64.96
12/4/2020 14:28	186.5	187	184.3	2313	64.96
12/4/2020 14:28	186.5	187.2	185.1	2313	65.04
12/4/2020 14:28	186.5	186.9	184	2311	65.04
12/4/2020 14:28	186.5	186.8	183.9	2312	64.92
12/4/2020 14:28	186.5	186.7	184.1	2311	64.92
12/4/2020 14:29	186.5	186.7	184.1	2309	64.87
12/4/2020 14:29	186.5	187	183.8	2310	64.96
12/4/2020 14:29	186.5	187	183.9	2306	64.91
12/4/2020 14:29	186.5	186.8	184.5	2307	64.88
12/4/2020 14:29	186.5	186.7	184.8	2310	65
12/4/2020 14:29	186.5	186.8	183.9	2309	64.87
12/4/2020 14:29	186.6	187	183.9	2306	64.96
12/4/2020 14:29	186.5	186.7	184.1	2308	64.96
12/4/2020 14:29	186.5	187.2	184.2	2310	64.87
12/4/2020 14:29	186.5	187.1	183.9	2306	64.92
12/4/2020 14:29	186.5	186.8	183.7	2315	65
12/4/2020 14:29	186.5	187.2	184.6	2315	64.96
12/4/2020 14:29	186.5	187.2	184.8	2315	64.96
12/4/2020 14:29	186.4	187.2	184.1	2316	65
12/4/2020 14:29	186.5	187	184.1	2316	64.92
12/4/2020 14:29	186.4	186.6	184.2	2315	65.04
12/4/2020 14:29	186.4	187.1	184	2314	65.04
12/4/2020 14:29	186.4	187.2	183.9	2312	64.92
12/4/2020 14:29	186.4	186.7	183.5	2308	65.04
12/4/2020 14:29	186.5	187	184.6	2308	64.92
12/4/2020 14:29	186.4	187	184.7	2315	65.04
12/4/2020 14:29	186.4	187.2	183.8	2317	65.04
12/4/2020 14:29	186.4	187.2	183.8	2314	65.04
12/4/2020 14:29	186.4	186.6	184.2	2312	65.04
12/4/2020 14:29	186.4	187.1	183.8	2308	64.91
12/4/2020 14:29	186.4	187	183.7	2312	65.04
12/4/2020 14:29	186.3	186.9	183.6	2313	64.93
12/4/2020 14:29	186.3	187.1	184	2308	64.88
12/4/2020 14:29	186.4	186.8	184.8	2312	65.04
12/4/2020 14:29	186.4	187.1	184.7	2315	65.04

12/4/2020 14:29	186.4	186.9	184.6	2314	64.84
12/4/2020 14:29	186.4	187.3	184	2314	65.04
12/4/2020 14:29	186.3	187.2	183.7	2318	65.08
12/4/2020 14:29	186.4	186.7	183.9	2320	64.99
12/4/2020 14:29	186.4	187.2	183.8	2321	64.92
12/4/2020 14:29	186.3	186.8	184.2	2321	65.05
12/4/2020 14:29	186.3	187.2	184	2318	64.92
12/4/2020 14:29	186.4	187.2	183.9	2317	65.12
12/4/2020 14:29	186.3	186.7	184.3	2322	65.05
12/4/2020 14:29	186.4	187	184.1	2319	64.96
12/4/2020 14:29	186.3	186.7	184.1	2320	65
12/4/2020 14:29	186.3	187.1	183.8	2321	64.96
12/4/2020 14:29	186.4	187.4	183.8	2317	65
12/4/2020 14:29	186.3	186.9	184.3	2315	64.96
12/4/2020 14:29	186.3	187	184.9	2318	65
12/4/2020 14:29	186.4	187.1	183.9	2317	65.09
12/4/2020 14:29	186.3	186.8	183.8	2313	64.99
12/4/2020 14:29	186.3	186.9	183.7	2312	64.96
12/4/2020 14:29	186.3	186.7	183.8	2315	65
12/4/2020 14:29	186.4	187	183.8	2315	64.99
12/4/2020 14:29	186.3	187	184.2	2315	64.88
12/4/2020 14:29	186.3	186.7	184.6	2318	65.08
12/4/2020 14:29	186.3	187.2	183.5	2318	65.12
12/4/2020 14:29	186.3	186.8	183.5	2318	64.92
12/4/2020 14:29	186.4	186.9	184	2317	64.99
12/4/2020 14:29	186.3	187	183.9	2318	65
12/4/2020 14:29	186.3	187.2	183.7	2312	64.99
12/4/2020 14:29	186.3	187.2	184.5	2318	64.97
12/4/2020 14:29	186.3	187	184.4	2317	64.88
12/4/2020 14:29	186.3	186.8	183.4	2310	65.04
12/4/2020 14:30	186.3	186.8	183.4	2310	65.08
12/4/2020 14:30	186.3	186.8	183.8	2308	64.99
12/4/2020 14:30	186.3	187.2	184.1	2310	64.96
12/4/2020 14:30	186.3	187	183.5	2310	64.92
12/4/2020 14:30	186.4	187	184.7	2311	64.96
12/4/2020 14:30	186.3	187.2	184.5	2310	64.87
12/4/2020 14:30	186.3	187.2	183.6	2312	65
12/4/2020 14:30	186.3	187.1	183.9	2312	65
12/4/2020 14:30	186.4	186.9	183.8	2312	64.88
12/4/2020 14:30	186.3	186.8	183.7	2309	65
12/4/2020 14:30	186.3	187.2	184.1	2311	65
12/4/2020 14:30	186.3	187	184.6	2309	65
12/4/2020 14:30	186.3	187.1	183.8	2308	64.96
12/4/2020 14:30	186.3	187	183.6	2312	65
12/4/2020 14:30	186.3	186	183.7	2311	64.92
12/4/2020 14:30	186.3	186.3	183.9	2306	65
12/4/2020 14:30	186.3	186.8	183.7	2310	64.96

12/4/2020 14:30	186.3	187.4	184.4	2310	64.96
12/4/2020 14:30	186.3	185.8	184.8	2308	64.99
12/4/2020 14:30	186.3	185.8	183.5	2311	64.88
12/4/2020 14:30	186.3	185.6	183.9	2309	64.96
12/4/2020 14:30	186.3	185.6	184.2	2304	64.92
12/4/2020 14:30	186.3	186.4	183.7	2304	64.99
12/4/2020 14:30	186.3	185.9	183.8	2304	65
12/4/2020 14:30	186.3	186.1	183.9	2305	64.99
12/4/2020 14:30	186.3	185.5	184.9	2306	64.92
12/4/2020 14:30	186.3	185.9	184.2	2305	65.08
12/4/2020 14:30	186.3	186.2	183.3	2304	64.83
12/4/2020 14:30	186.3	186.2	183.6	2300	65.08
12/4/2020 14:30	186.3	186.3	183.7	2304	65.04
12/4/2020 14:30	186.3	185.6	184	2301	65.04
12/4/2020 14:30	186.3	186.1	183.9	2304	64.88
12/4/2020 14:30	186.3	186.4	183.6	2304	65.04
12/4/2020 14:30	186.3	186.2	184.3	2304	65.04
12/4/2020 14:30	186.4	185.6	184.5	2300	65.08
12/4/2020 14:30	186.4	185.6	183.4	2303	64.92
12/4/2020 14:30	186.3	185.6	183.5	2300	65.08
12/4/2020 14:30	186.4	186.3	183.4	2302	65
12/4/2020 14:30	186.3	186	183.7	2304	65.08
12/4/2020 14:30	186.4	185.8	183.9	2304	65.08
12/4/2020 14:30	186.3	186.3	183.7	2305	64.88
12/4/2020 14:30	186.3	185.8	183.5	2303	64.88
12/4/2020 14:30	186.4	186.1	183.5	2302	64.88
12/4/2020 14:30	186.4	185.5	183.5	2304	64.92
12/4/2020 14:30	186.4	185.9	183.4	2306	65.04
12/4/2020 14:30	186.3	186.3	183.5	2310	64.92
12/4/2020 14:30	186.4	185.7	183.4	2310	64.92
12/4/2020 14:30	186.3	186.2	183.6	2306	65.04
12/4/2020 14:30	186.4	185.5	183.5	2306	64.96
12/4/2020 14:30	186.4	185.8	183.7	2302	65.04
12/4/2020 14:30	186.3	186.4	183.9	2304	64.96
12/4/2020 14:30	186.4	186	183.7	2307	65
12/4/2020 14:30	186.3	186	183.8	2305	64.96
12/4/2020 14:30	186.4	186.1	183.7	2304	64.96
12/4/2020 14:30	186.4	185.5	183.3	2306	65
12/4/2020 14:30	186.4	186.4	183.9	2304	64.92
12/4/2020 14:30	186.4	185.8	184.4	2306	65
12/4/2020 14:30	186.4	186.1	183.9	2306	65
12/4/2020 14:30	186.5	186.2	183.7	2306	64.92
12/4/2020 14:31	186.5	186.2	183.8	2304	65
12/4/2020 14:31	186.4	186.2	184	2305	65.04
12/4/2020 14:31	186.5	185.9	183.4	2303	64.95
12/4/2020 14:31	186.4	186	183.3	2303	65.04
12/4/2020 14:31	186.5	186.2	184.2	2304	65.09

12/4/2020 14:31	186.5	185.6	184.4	2302	64.92
12/4/2020 14:31	186.5	186.2	183.8	2300	64.83
12/4/2020 14:31	186.6	185.7	183.6	2306	65.08
12/4/2020 14:31	186.5	186	183.7	2309	64.96
12/4/2020 14:31	186.6	186.3	183.9	2308	64.93
12/4/2020 14:31	186.5	185.6	183.8	2308	64.96
12/4/2020 14:31	186.6	186.2	183.7	2310	64.99
12/4/2020 14:31	186.6	185.8	183.5	2307	64.88
12/4/2020 14:31	186.6	185.6	183.8	2308	65.08
12/4/2020 14:31	186.6	186.3	184.5	2310	64.88
12/4/2020 14:31	186.7	185.5	184.4	2310	64.92
12/4/2020 14:31	186.6	186.3	183.8	2313	64.96
12/4/2020 14:31	186.7	186.1	183.7	2313	64.96
12/4/2020 14:31	186.6	185.5	183.7	2312	64.99
12/4/2020 14:31	186.7	185.8	183.9	2312	64.92
12/4/2020 14:31	186.6	185.8	183.7	2314	65.04
12/4/2020 14:31	186.7	186.2	183.5	2315	65.03
12/4/2020 14:31	186.7	186.2	183.7	2316	64.99
12/4/2020 14:31	186.8	186.4	183.8	2319	65.12
12/4/2020 14:31	186.7	186.2	183.8	2318	64.95
12/4/2020 14:31	186.7	185.7	183.9	2313	64.88
12/4/2020 14:31	186.6	185.9	183.7	2312	65.08
12/4/2020 14:31	186.7	186.3	183.5	2313	64.83
12/4/2020 14:31	186.7	186	183.4	2314	64.83
12/4/2020 14:31	186.7	186.2	183.4	2317	64.92
12/4/2020 14:31	186.7	185.8	183.5	2322	64.92
12/4/2020 14:31	186.7	185.7	183.5	2321	64.96
12/4/2020 14:31	186.7	186.2	183.3	2320	65
12/4/2020 14:31	186.7	185.9	183.4	2322	64.99
12/4/2020 14:31	186.7	186.2	183.6	2319	64.95
12/4/2020 14:31	186.7	186	184.1	2318	65.04
12/4/2020 14:31	186.7	185.7	184.5	2320	64.96
12/4/2020 14:31	186.7	185.6	184.8	2322	64.96
12/4/2020 14:31	186.7	185.7	184.5	2322	64.96
12/4/2020 14:31	186.7	186.3	184.2	2325	64.96
12/4/2020 14:31	186.7	186.3	183.7	2322	64.96
12/4/2020 14:31	186.6	186.3	183.5	2322	65.04
12/4/2020 14:31	186.7	186.3	183.4	2324	64.96
12/4/2020 14:31	186.7	185.6	183.8	2323	64.96
12/4/2020 14:31	186.7	185.7	183.9	2322	64.92
12/4/2020 14:31	186.7	186.3	183.7	2321	64.95
12/4/2020 14:31	186.7	186.2	183.8	2322	64.96
12/4/2020 14:31	186.7	186.1	183.7	2321	65.08
12/4/2020 14:31	186.7	185.6	183.8	2322	64.92
12/4/2020 14:31	186.6	185.8	184.1	2324	64.91
12/4/2020 14:31	186.7	186.4	183.7	2321	64.92
12/4/2020 14:31	186.7	186.1	183.9	2329	65

12/4/2020 14:31	186.8	186.2	183.7	2323	64.92
12/4/2020 14:31	186.7	185.6	183.7	2318	64.99
12/4/2020 14:31	186.8	185.8	183.8	2321	64.96
12/4/2020 14:31	186.8	186.5	183.7	2322	64.84
12/4/2020 14:31	186.8	185.5	183.6	2320	64.88
12/4/2020 14:31	186.7	186.3	183.6	2326	65.05
12/4/2020 14:31	186.8	186.1	183.5	2326	64.83
12/4/2020 14:32	186.8	185.9	183.8	2322	65.03
12/4/2020 14:32	186.8	186.3	183.6	2321	64.96
12/4/2020 14:32	186.8	185.8	183.6	2320	64.83
12/4/2020 14:32	186.7	186.3	183.9	2321	65.04
12/4/2020 14:32	186.8	186.2	183.7	2325	65.08
12/4/2020 14:32	186.7	185.6	183.7	2324	65.04
12/4/2020 14:32	186.7	185.8	183.6	2321	65.08
12/4/2020 14:32	186.8	185.8	183.7	2322	65.04
12/4/2020 14:32	186.8	186.2	183.9	2322	65.04
12/4/2020 14:32	186.7	186.3	184	2318	64.99
12/4/2020 14:32	186.8	186.3	183.9	2318	65.04
12/4/2020 14:32	186.7	186	183.6	2322	65.04
12/4/2020 14:32	186.7	185.8	183.5	2321	65
12/4/2020 14:32	186.7	185.6	184.2	2318	64.88
12/4/2020 14:32	186.8	186.4	184.6	2322	65.04
12/4/2020 14:32	186.8	186.3	184.1	2322	64.88
12/4/2020 14:32	186.7	186.1	183.5	2321	64.99
12/4/2020 14:32	186.7	185.8	183.5	2327	64.96
12/4/2020 14:32	186.7	186	183.7	2324	64.87
12/4/2020 14:32	186.7	186.2	183.9	2327	65
12/4/2020 14:32	186.7	185.6	183.5	2326	65.09
12/4/2020 14:32	186.7	186	183.7	2325	64.87
12/4/2020 14:32	186.7	185.6	184.5	2322	64.96
12/4/2020 14:32	186.7	185.8	183.9	2323	65.04
12/4/2020 14:32	186.7	186.3	183.5	2321	64.92
12/4/2020 14:32	186.8	185.6	183.8	2319	64.96
12/4/2020 14:32	186.7	186.3	183.9	2320	65
12/4/2020 14:32	186.7	186	183.8	2320	65
12/4/2020 14:32	186.7	185.7	183.7	2320	64.96
12/4/2020 14:32	186.7	185.8	184.6	2326	65.09
12/4/2020 14:32	186.7	185.8	184.1	2325	65
12/4/2020 14:32	186.7	186.1	183.4	2326	64.91
12/4/2020 14:32	186.7	185.6	183.5	2326	65.08
12/4/2020 14:32	186.7	185.6	183.7	2325	65.01
12/4/2020 14:32	186.7	186.3	184	2323	64.88
12/4/2020 14:32	186.7	185.6	183.8	2324	65.08
12/4/2020 14:32	186.7	186.4	183.5	2320	64.96
12/4/2020 14:32	186.7	185.6	184	2322	64.87
12/4/2020 14:32	186.7	186.2	184.6	2322	64.88
12/4/2020 14:32	186.7	186.2	183.9	2322	65.05

12/4/2020 14:32	186.7	186	183.6	2318	64.95
12/4/2020 14:32	186.7	185.8	183.3	2321	65.04
12/4/2020 14:32	186.8	186.2	183.3	2328	64.93
12/4/2020 14:32	186.7	185.5	183.6	2328	64.99
12/4/2020 14:32	186.7	186.1	183.7	2329	65.04
12/4/2020 14:32	186.6	185.7	183.6	2333	64.96
12/4/2020 14:32	186.7	185.7	183.6	2328	64.88
12/4/2020 14:32	186.7	186.4	183.7	2329	65
12/4/2020 14:32	186.7	186.3	183.6	2328	65.04
12/4/2020 14:32	186.7	186.1	183.7	2324	65.05
12/4/2020 14:32	186.7	186.1	183.8	2324	64.96
12/4/2020 14:32	186.6	185.6	183.8	2328	65.09
12/4/2020 14:32	186.7	185.7	183.7	2325	64.92
12/4/2020 14:32	186.7	186	183.5	2322	64.92
12/4/2020 14:32	186.7	186	183.4	2319	64.96
12/4/2020 14:32	186.7	186.2	183.3	2317	64.99
12/4/2020 14:32	186.7	185.5	183.4	2320	65.04
12/4/2020 14:32	186.7	185.9	183.6	2317	65.08
12/4/2020 14:32	186.6	185.8	184.1	2315	64.88
12/4/2020 14:32	186.7	186.1	184.4	2312	64.99
12/4/2020 14:33	186.8	186.3	183.8	2312	65.16
12/4/2020 14:33	186.7	185.6	183.6	2310	64.99
12/4/2020 14:33	186.7	186.1	183.7	2310	64.99
12/4/2020 14:33	186.7	185.5	183.8	2314	65.05
12/4/2020 14:33	186.7	185.8	183.8	2309	65
12/4/2020 14:33	186.7	186.2	183.2	2308	65
12/4/2020 14:33	186.7	185.6	183.2	2313	64.96
12/4/2020 14:33	186.7	186.4	184.2	2314	64.96
12/4/2020 14:33	186.7	185.6	184.6	2312	64.77
12/4/2020 14:33	186.7	186.1	183.5	2315	64.73
12/4/2020 14:33	186.7	186.3	183.4	2316	65
12/4/2020 14:33	186.7	186.2	183.9	2308	64.91
12/4/2020 14:33	186.7	186.2	183.6	2308	65
12/4/2020 14:33	186.7	185.8	183.2	2308	64.96
12/4/2020 14:33	186.7	185.6	183.2	2309	64.73
12/4/2020 14:33	186.7	186.2	183.2	2310	64.76
12/4/2020 14:33	186.6	185.7	183.5	2314	64.69
12/4/2020 14:33	186.7	186.2	184.6	2314	64.69
12/4/2020 14:33	186.7	186	184.1	2309	65.03
12/4/2020 14:33	186.7	186.2	183.8	2311	65.12
12/4/2020 14:33	186.7	186.3	183.5	2314	64.96
12/4/2020 14:33	186.7	185.7	183.5	2310	65.15
12/4/2020 14:33	186.8	185.8	183.7	2310	65.12
12/4/2020 14:33	186.8	186.3	183.7	2309	64.99
12/4/2020 14:33	186.7	185.6	183.5	2308	65.04
12/4/2020 14:33	186.8	186	183.5	2305	65.79
12/4/2020 14:33	186.7	185.7	183.7	2304	65.03

12/4/2020 14:33	186.8	186.1	183.6	2305	64.84
12/4/2020 14:33	186.8	186.3	183.8	2304	64.96
12/4/2020 14:33	186.8	186	183.7	2308	64.92
12/4/2020 14:33	186.8	186.2	183.7	2304	64.92
12/4/2020 14:33	186.8	185.6	183.2	2311	65.05
12/4/2020 14:33	186.8	185.7	183.3	2312	64.84
12/4/2020 14:33	186.9	185.5	183.1	2315	64.88
12/4/2020 14:33	186.8	186	183.8	2318	65.04
12/4/2020 14:33	186.9	186.3	184.4	2316	64.96
12/4/2020 14:33	186.8	186	184.6	2313	64.93
12/4/2020 14:33	186.9	186.3	184.1	2313	64.96
12/4/2020 14:33	187	186.3	183.4	2313	64.96
12/4/2020 14:33	187	185.5	183.3	2312	64.92
12/4/2020 14:33	187	186	183.3	2315	65
12/4/2020 14:33	187	186	183.7	2317	65
12/4/2020 14:33	187	185.6	183.7	2320	64.96
12/4/2020 14:33	187	186.3	183.5	2325	65
12/4/2020 14:33	187	185.7	183.4	2325	65.05
12/4/2020 14:33	187	186	183.9	2318	64.96
12/4/2020 14:33	187	186.4	184.4	2317	64.96
12/4/2020 14:33	187	185.6	183.4	2313	64.96
12/4/2020 14:33	187	186.2	183.4	2315	64.96
12/4/2020 14:33	187	185.6	183.8	2315	64.88
12/4/2020 14:33	187	185.9	183.5	2318	65.08
12/4/2020 14:33	187.1	185.5	183.5	2321	64.83
12/4/2020 14:33	187	186.1	184	2322	64.92
12/4/2020 14:33	187.1	186.3	184	2318	64.92
12/4/2020 14:33	187	185.8	183.3	2321	64.87
12/4/2020 14:33	187	185.9	183.4	2322	64.92
12/4/2020 14:33	187.1	186.2	183.7	2326	65.12
12/4/2020 14:33	187.1	185.6	183.8	2326	64.96
12/4/2020 14:33	187	186	183.7	2322	65.12
12/4/2020 14:34	187	185.6	183.5	2327	64.93
12/4/2020 14:34	187.1	186	184.1	2327	65.12
12/4/2020 14:34	187.1	185.9	184.2	2324	64.92
12/4/2020 14:34	187.1	185.8	183.2	2326	64.96
12/4/2020 14:34	187.1	186	183.5	2322	64.92
12/4/2020 14:34	187.1	185.7	183.8	2322	65.04
12/4/2020 14:34	187.1	185.7	183.8	2323	65.08
12/4/2020 14:34	187.1	186.3	183.4	2325	65
12/4/2020 14:34	187.1	186	183.5	2325	65.08
12/4/2020 14:34	187.1	186	184.2	2321	65
12/4/2020 14:34	187.1	186.5	184.1	2321	64.96
12/4/2020 14:34	187.1	185.6	183.5	2321	65
12/4/2020 14:34	187.1	186.3	183	2322	65.04
12/4/2020 14:34	187.1	185.7	183.6	2324	65
12/4/2020 14:34	187.1	186.4	183.4	2322	64.96

12/4/2020 14:34	187.1	186.3	183.5	2321	64.96
12/4/2020 14:34	187.1	185.6	183.8	2325	65.04
12/4/2020 14:34	187.1	187	183.7	2321	64.96
12/4/2020 14:34	187.1	187	183.4	2318	64.96
12/4/2020 14:34	187.1	186.8	183.7	2321	65
12/4/2020 14:34	187.1	187.2	183.5	2324	64.88
12/4/2020 14:34	187.1	186.9	183.7	2322	65
12/4/2020 14:34	187.1	187.2	183.9	2323	65.09
12/4/2020 14:34	187.1	187.1	184.2	2324	64.99
12/4/2020 14:34	187.1	186.8	184.6	2322	64.93
12/4/2020 14:34	187.1	187	184.2	2325	65.08
12/4/2020 14:34	187.1	186.8	184.6	2325	64.88
12/4/2020 14:34	187.1	187	184.6	2322	64.96
12/4/2020 14:34	187	187.2	184.2	2324	64.93
12/4/2020 14:34	187	186.8	184.4	2320	65.08
12/4/2020 14:34	187	187	184.2	2318	64.92
12/4/2020 14:34	187	186.8	184.1	2321	65.12
12/4/2020 14:34	187	186.8	183.8	2320	65.08
12/4/2020 14:34	187	187.2	183.5	2322	64.88
12/4/2020 14:34	187	187	183.6	2324	65
12/4/2020 14:34	187	187.2	183.7	2323	65.09
12/4/2020 14:34	187.1	186.7	183.5	2324	64.92
12/4/2020 14:34	187	186.8	183.9	2321	64.96
12/4/2020 14:34	187.1	187.2	183.5	2324	65.04
12/4/2020 14:34	187	187	183.5	2326	64.96
12/4/2020 14:34	187	187.4	183.5	2323	64.92
12/4/2020 14:34	187	187.2	184.3	2323	65.09
12/4/2020 14:34	187	186.8	183.7	2319	64.96
12/4/2020 14:34	187	187.2	183.7	2320	65
12/4/2020 14:34	187	186.9	183.7	2321	65.04
12/4/2020 14:34	187	187	183.7	2317	64.92
12/4/2020 14:34	187	187	183.5	2318	65
12/4/2020 14:34	187	186.8	183.1	2322	65
12/4/2020 14:34	187	187.2	183.8	2319	64.88
12/4/2020 14:34	187	186.8	184.5	2320	65.08
12/4/2020 14:34	187	186.8	183.8	2325	65.04
12/4/2020 14:34	187	187	183.5	2325	65.08
12/4/2020 14:34	187	186.8	183.4	2326	65.08
12/4/2020 14:34	186.9	187	183.8	2326	65.01
12/4/2020 14:34	187	186.8	183.4	2320	64.88
12/4/2020 14:34	187	186.9	183.3	2321	64.92
12/4/2020 14:34	187	187.2	183.7	2326	64.99
12/4/2020 14:34	187	186.9	184.2	2325	65.04
12/4/2020 14:34	187	187.1	184.2	2328	64.93
12/4/2020 14:34	187	186.7	183.4	2330	64.96
12/4/2020 14:35	187	186.9	183.5	2329	64.83
12/4/2020 14:35	187	187.2	183.7	2327	65.04

12/4/2020 14:35	187	186.8	183.6	2322	65.04
12/4/2020 14:35	187	187	183.3	2325	65.04
12/4/2020 14:35	187	186.8	183.2	2324	64.92
12/4/2020 14:35	187	186.8	183.2	2324	65
12/4/2020 14:35	187	187	184.1	2326	64.96
12/4/2020 14:35	186.9	186.8	184.4	2330	64.96
12/4/2020 14:35	187	187	184.1	2326	64.96
12/4/2020 14:35	187	186.8	183.3	2327	65.04
12/4/2020 14:35	187	187	183.4	2326	64.96
12/4/2020 14:35	186.9	187.1	183.6	2318	64.96
12/4/2020 14:35	187	187	183.9	2321	65.09
12/4/2020 14:35	187	187.1	183.9	2320	64.92
12/4/2020 14:35	187	186.7	183.8	2320	65
12/4/2020 14:35	187	187	183.2	2321	65.09
12/4/2020 14:35	186.9	187.3	183.5	2322	65.04
12/4/2020 14:35	187	187	183.5	2327	65
12/4/2020 14:35	186.9	187.1	183.7	2328	64.93
12/4/2020 14:35	187	186.8	184.7	2325	64.92
12/4/2020 14:35	186.9	186.6	184.4	2324	65.08
12/4/2020 14:35	187	187	183.4	2326	65.08
12/4/2020 14:35	186.9	187	183.7	2328	64.96
12/4/2020 14:35	187	187.2	183.4	2329	64.92
12/4/2020 14:35	187	187.2	183.7	2326	64.92
12/4/2020 14:35	186.9	186.7	183.9	2328	64.96
12/4/2020 14:35	187	187	183.7	2329	64.92
12/4/2020 14:35	186.9	186.6	183.5	2326	65.04
12/4/2020 14:35	187	186.8	183.4	2329	64.96
12/4/2020 14:35	186.9	187.2	183.1	2329	64.88
12/4/2020 14:35	186.9	187	183.8	2326	64.96
12/4/2020 14:35	187	187.1	184.3	2328	65
12/4/2020 14:35	186.9	187.1	184.2	2329	64.96
12/4/2020 14:35	186.9	187.2	183.5	2332	64.92
12/4/2020 14:35	186.9	187.4	183.2	2335	65.05
12/4/2020 14:35	187	187	183.5	2330	64.87
12/4/2020 14:35	187	187	183.7	2329	64.92
12/4/2020 14:35	186.9	187.2	183.7	2328	65.09
12/4/2020 14:35	187	186.9	183.7	2329	64.92
12/4/2020 14:35	187	187.1	183.6	2328	64.96
12/4/2020 14:35	187	186.7	183.7	2329	65.04
12/4/2020 14:35	187	187	183.4	2326	64.88
12/4/2020 14:35	187	187.2	183.1	2326	64.96
12/4/2020 14:35	187	187	183.3	2331	65
12/4/2020 14:35	187	187.2	183.2	2335	64.96
12/4/2020 14:35	187	187	183.4	2326	65
12/4/2020 14:35	187	186.8	183.5	2327	64.92
12/4/2020 14:35	187	186.7	183.8	2322	65.04
12/4/2020 14:35	187	187	184.1	2325	65.03

12/4/2020 14:35	187	187.2	184.1	2326	64.93
12/4/2020 14:35	187	186.7	184.2	2326	65.16
12/4/2020 14:35	187	186.9	184.5	2326	65.04
12/4/2020 14:35	187	187.2	184.4	2326	64.88
12/4/2020 14:35	187	186.8	184.2	2326	65.05
12/4/2020 14:35	187	187	184.6	2327	64.92
12/4/2020 14:35	187.1	187.1	184.2	2325	64.88
12/4/2020 14:35	187	186.8	184.3	2324	65.04
12/4/2020 14:35	187	187.2	184.2	2324	64.92
12/4/2020 14:35	187.1	186.9	183.9	2330	64.96
12/4/2020 14:36	187	187	183.9	2327	65.04
12/4/2020 14:36	187	187.2	183.7	2325	64.96
12/4/2020 14:36	187	186.8	183.8	2326	64.96
12/4/2020 14:36	187	187	183.7	2332	64.96
12/4/2020 14:36	187	186.9	184	2333	64.96
12/4/2020 14:36	187.1	187	184.1	2330	64.96
12/4/2020 14:36	187	187.2	184.3	2326	65.04
12/4/2020 14:36	187.1	186.8	184.2	2333	64.92
12/4/2020 14:36	187.1	187	184.2	2333	64.96
12/4/2020 14:36	187.1	187	184.3	2329	64.96
12/4/2020 14:36	187.1	186.8	184.4	2333	65.08
12/4/2020 14:36	187.1	187.2	184.6	2329	64.88
12/4/2020 14:36	187.1	187	184.5	2325	64.92
12/4/2020 14:36	187.1	186.8	184.4	2330	65.08
12/4/2020 14:36	187.1	187	184.1	2329	64.88
12/4/2020 14:36	187.1	186.7	184.1	2324	64.88
12/4/2020 14:36	187.1	187	183.9	2330	64.97
12/4/2020 14:36	187.2	187	183.7	2333	64.84
12/4/2020 14:36	187.2	187.2	183.5	2332	64.92
12/4/2020 14:36	187.2	187.1	183.2	2335	64.97
12/4/2020 14:36	187.2	186.8	183.2	2336	64.96
12/4/2020 14:36	187.2	187	183.2	2334	65.08
12/4/2020 14:36	187.3	186.9	183.3	2331	64.96
12/4/2020 14:36	187.2	187	183.6	2332	64.96
12/4/2020 14:36	187.2	187.2	183.7	2335	64.96
12/4/2020 14:36	187.2	187	183.6	2331	65.08
12/4/2020 14:36	187.2	187.2	183.8	2336	65
12/4/2020 14:36	187.2	187	183.8	2331	64.96
12/4/2020 14:36	187.2	186.8	183.4	2329	65
12/4/2020 14:36	187.2	186.8	183.5	2328	65.04
12/4/2020 14:36	187.3	186.9	183.2	2331	64.96
12/4/2020 14:36	187.2	187	183.5	2328	65
12/4/2020 14:36	187.3	187.3	184.1	2327	65.08
12/4/2020 14:36	187.2	187.2	184.2	2321	64.92
12/4/2020 14:36	187.3	187.1	184.4	2328	64.96
12/4/2020 14:36	187.3	186.7	184.2	2330	64.92
12/4/2020 14:36	187.2	187	183.8	2330	65.04

12/4/2020 14:36	187.3	187.2	183.8	2328	64.99
12/4/2020 14:36	187.2	186.8	183.2	2333	65.01
12/4/2020 14:36	187.3	187.4	183.4	2331	64.96
12/4/2020 14:36	187.3	187	183.4	2332	64.88
12/4/2020 14:36	187.3	187	183.1	2332	64.92
12/4/2020 14:36	187.3	187.2	183.4	2333	65.16
12/4/2020 14:36	187.3	186.7	183.3	2332	64.84
12/4/2020 14:36	187.3	187.1	183.3	2331	65.08
12/4/2020 14:36	187.3	186.8	183.2	2331	65.08
12/4/2020 14:36	187.3	187	183.3	2330	64.99
12/4/2020 14:36	187.3	187.2	183.4	2326	65.12
12/4/2020 14:36	187.3	186.8	183.2	2326	65.16
12/4/2020 14:36	187.3	187.2	183	2325	64.96
12/4/2020 14:36	187.3	187.2	183.5	2329	65
12/4/2020 14:36	187.3	186.8	183.1	2330	65.08
12/4/2020 14:36	187.3	187.4	183.2	2330	65
12/4/2020 14:36	187.2	186.8	183.4	2329	64.92
12/4/2020 14:36	187.3	186.9	183.1	2332	64.96
12/4/2020 14:36	187.3	187.2	183.3	2330	65
12/4/2020 14:36	187.2	187	183.2	2329	64.92
12/4/2020 14:36	187.3	187.2	183.3	2329	65.04
12/4/2020 14:36	187.2	187	183.2	2326	65.04
12/4/2020 14:37	187.3	187.4	183.1	2322	64.92
12/4/2020 14:37	187.3	186.8	183.6	2327	64.96
12/4/2020 14:37	187.3	186.7	184.1	2329	65.09
12/4/2020 14:37	187.2	187.2	184.1	2326	64.88
12/4/2020 14:37	187.2	186.8	184.3	2325	64.96
12/4/2020 14:37	187.2	186.8	183.8	2327	64.96
12/4/2020 14:37	187.3	187	183.5	2330	64.99
12/4/2020 14:37	187.3	186.8	183.8	2332	64.92
12/4/2020 14:37	187.3	187.1	183.4	2333	65.12
12/4/2020 14:37	187.3	187	183.4	2328	64.8
12/4/2020 14:37	187.2	187.3	183.1	2332	64.88
12/4/2020 14:37	187.2	187.4	183.1	2330	65.08
12/4/2020 14:37	187.2	186.7	184.2	2329	64.99
12/4/2020 14:37	187.2	187	184.2	2329	64.92
12/4/2020 14:37	187.2	186.8	183.9	2326	64.96
12/4/2020 14:37	187.2	186.9	183.5	2327	64.83
12/4/2020 14:37	187.2	187.3	183.8	2329	64.84
12/4/2020 14:37	187.2	187	183.6	2329	64.92
12/4/2020 14:37	187.2	187.2	183.6	2333	65.04
12/4/2020 14:37	187.2	187	183.3	2335	64.96
12/4/2020 14:37	187.2	186.8	183.5	2333	64.92
12/4/2020 14:37	187.2	187	184	2333	64.92
12/4/2020 14:37	187.2	186.8	184.5	2333	64.99
12/4/2020 14:37	187.2	187	184.2	2334	64.92
12/4/2020 14:37	187.2	187	183.2	2333	65.04

12/4/2020 14:37	187.2	187.2	183.6	2333	64.83
12/4/2020 14:37	187.2	187.1	183.6	2333	65.04
12/4/2020 14:37	187.2	186.8	183.7	2337	65.05
12/4/2020 14:37	187.2	187.2	183.6	2336	64.96
12/4/2020 14:37	187.2	187.2	183.2	2335	64.96
12/4/2020 14:37	187.2	187.2	183.5	2334	65.05
12/4/2020 14:37	187.2	187.1	184.3	2330	64.91
12/4/2020 14:37	187.2	186.8	184.4	2328	64.92
12/4/2020 14:37	187.2	187	183.9	2332	65.12
12/4/2020 14:37	187.3	187.4	183.4	2330	64.88
12/4/2020 14:37	187.2	186.8	183.4	2329	65.04
12/4/2020 14:37	187.2	187	183.8	2326	64.92
12/4/2020 14:37	187.2	187	183.5	2328	64.99
12/4/2020 14:37	187.3	186.9	183.5	2329	65.04
12/4/2020 14:37	187.2	187.2	183.4	2329	65.08
12/4/2020 14:37	187.2	187	183.2	2331	65.08
12/4/2020 14:37	187.2	187.4	183.2	2333	64.84
12/4/2020 14:37	187.2	187.2	183.2	2333	64.99
12/4/2020 14:37	187.2	186.8	183.3	2332	64.93
12/4/2020 14:37	187.2	187.3	184	2334	64.88
12/4/2020 14:37	187.2	186.8	184.1	2333	64.92
12/4/2020 14:37	187.2	187.2	184.5	2335	65.12
12/4/2020 14:37	187.2	187.2	184.4	2337	64.88
12/4/2020 14:37	187.2	186.8	184.2	2337	65.08
12/4/2020 14:37	187.2	187	184.2	2337	65.16
12/4/2020 14:37	187.2	186.8	183.9	2333	64.96
12/4/2020 14:37	187.2	187	183.7	2333	65.04
12/4/2020 14:37	187.2	186.8	183.6	2330	65.08
12/4/2020 14:37	187.2	186.9	183.5	2331	65.04
12/4/2020 14:37	187.2	187.3	183.8	2328	64.96
12/4/2020 14:37	187.2	187	183.7	2329	65
12/4/2020 14:37	187.2	186.9	183.7	2327	65.04
12/4/2020 14:37	187.2	187.2	183.8	2329	65
12/4/2020 14:37	187.2	187	183.5	2330	64.96
12/4/2020 14:37	187.2	187.3	184.1	2333	65
12/4/2020 14:38	187.2	187.1	184.2	2332	65
12/4/2020 14:38	187.2	186.6	184.1	2333	65
12/4/2020 14:38	187.1	187	184.4	2335	65
12/4/2020 14:38	187.2	186.9	183.9	2336	64.88
12/4/2020 14:38	187.2	187	183.8	2330	65.04
12/4/2020 14:38	187.1	187.3	183.4	2332	65.01
12/4/2020 14:38	187.2	186.8	183	2328	64.99
12/4/2020 14:38	187.2	187.3	183.2	2330	64.84
12/4/2020 14:38	187.2	186.8	183.4	2332	64.96
12/4/2020 14:38	187.1	187	183.2	2331	64.93
12/4/2020 14:38	187.2	187.3	183.6	2327	65.08
12/4/2020 14:38	187.1	186.7	183.5	2326	64.99

12/4/2020 14:38	187.1	187	183.5	2330	65.16
12/4/2020 14:38	187.1	186.8	183.3	2329	64.99
12/4/2020 14:38	187.1	187	183.3	2326	64.88
12/4/2020 14:38	187.1	186.8	184.2	2330	65.04
12/4/2020 14:38	187.2	187	183.9	2331	64.8
12/4/2020 14:38	187.1	187.2	183.6	2331	64.92
12/4/2020 14:38	187.2	187	183.2	2328	65.09
12/4/2020 14:38	187.2	187	183	2329	65
12/4/2020 14:38	187.2	186.7	183.5	2323	65.04
12/4/2020 14:38	187.2	187	183.3	2320	64.92
12/4/2020 14:38	187.2	187.2	183.6	2326	64.96
12/4/2020 14:38	187.2	187	183.5	2326	64.92
12/4/2020 14:38	187.2	186.8	183.4	2322	65
12/4/2020 14:38	187.2	186.9	183.3	2328	65.05
12/4/2020 14:38	187.2	187	183.6	2326	64.96
12/4/2020 14:38	187.2	187	184.1	2328	64.96
12/4/2020 14:38	187.2	187.2	184.2	2334	65.05
12/4/2020 14:38	187.1	187.2	183.9	2329	64.96
12/4/2020 14:38	187.2	187	183.7	2326	64.99
12/4/2020 14:38	187.2	187	183.4	2325	65.08
12/4/2020 14:38	187.2	187	183.1	2324	64.96
12/4/2020 14:38	187.2	186.8	183.2	2326	64.88
12/4/2020 14:38	187.2	186.8	183.2	2329	64.92
12/4/2020 14:38	187.2	187.4	183.1	2329	65
12/4/2020 14:38	187.2	186.9	183.3	2326	65.08
12/4/2020 14:38	187.2	187.3	183.1	2325	64.99
12/4/2020 14:38	187.2	187.4	183.2	2323	65.01
12/4/2020 14:38	187.2	186.7	183.7	2329	64.8
12/4/2020 14:38	187.4	187.1	183.4	2325	64.99
12/4/2020 14:38	187.3	187	183.4	2322	65.12
12/4/2020 14:38	187.3	186.8	183.5	2321	65.04
12/4/2020 14:38	187.2	187.3	183.4	2322	64.88
12/4/2020 14:38	187.2	186.8	183.4	2322	65.08
12/4/2020 14:38	187.2	187	183.3	2319	64.88
12/4/2020 14:38	187.2	186.8	183.3	2318	64.96
12/4/2020 14:38	187.2	186.9	183.3	2321	65.04
12/4/2020 14:38	187.3	187.4	183	2323	65
12/4/2020 14:38	187.2	187	183.2	2320	64.96
12/4/2020 14:38	187.3	187	183.9	2318	64.96
12/4/2020 14:38	187.3	187	184.1	2318	65.04
12/4/2020 14:38	187.3	187.2	184.4	2315	64.92
12/4/2020 14:38	187.2	187.2	183.6	2317	64.92
12/4/2020 14:38	187.3	187	183.6	2319	65
12/4/2020 14:38	187.4	187.2	183.8	2321	64.96
12/4/2020 14:38	187.3	187.2	183.4	2319	65
12/4/2020 14:38	187.4	186.8	183.7	2322	64.92
12/4/2020 14:38	187.4	187	183.5	2324	65

12/4/2020 14:39	187.4	186.9	183.4	2328	64.92
12/4/2020 14:39	187.4	187	183.4	2328	65.04
12/4/2020 14:39	187.4	187.1	184.2	2328	65.08
12/4/2020 14:39	187.5	187	184.4	2330	64.84
12/4/2020 14:39	187.5	187	184.1	2328	64.92
12/4/2020 14:39	187.5	186.9	183.4	2328	65.12
12/4/2020 14:39	187.5	186.8	183.4	2327	65.03
12/4/2020 14:39	187.5	186.8	183.7	2329	64.88
12/4/2020 14:39	187.7	186.7	183.6	2329	64.96
12/4/2020 14:39	187.5	187	183.8	2326	64.84
12/4/2020 14:39	187.6	186.8	183.5	2330	65.04
12/4/2020 14:39	187.7	187.2	183.4	2329	64.92
12/4/2020 14:39	187.6	187	183.3	2332	65
12/4/2020 14:39	187.6	186.8	183	2331	65.04
12/4/2020 14:39	187.7	187	183.6	2328	64.96
12/4/2020 14:39	187.7	186.7	184.1	2331	65
12/4/2020 14:39	187.7	186.8	184.2	2333	64.88
12/4/2020 14:39	187.7	187.2	184.2	2334	64.96
12/4/2020 14:39	187.7	187	183.8	2337	65
12/4/2020 14:39	187.7	186.8	183.3	2339	64.92
12/4/2020 14:39	187.7	186.9	183.4	2336	64.96
12/4/2020 14:39	187.7	186.7	183.5	2337	65.04
12/4/2020 14:39	187.7	186.9	183.6	2334	64.92
12/4/2020 14:39	187.7	186.8	183.5	2333	64.92
12/4/2020 14:39	187.7	187.3	183.7	2335	65
12/4/2020 14:39	187.8	187.2	183.7	2333	64.83
12/4/2020 14:39	187.7	186.8	183.8	2336	65
12/4/2020 14:39	187.5	187	183.6	2337	65.12
12/4/2020 14:39	187.8	186.8	183.4	2341	64.92
12/4/2020 14:39	187.7	187.2	183.4	2339	65.04
12/4/2020 14:39	187.7	187.3	183.5	2341	65.04
12/4/2020 14:39	187.7	187	183.6	2343	65.04
12/4/2020 14:39	187.8	187	183.2	2340	64.88
12/4/2020 14:39	187.7	186.8	183.4	2342	64.92
12/4/2020 14:39	187.8	187	183.2	2344	64.96
12/4/2020 14:39	187.8	187.1	183.5	2340	64.83
12/4/2020 14:39	187.8	187	183.7	2341	64.92
12/4/2020 14:39	187.7	187.2	183.7	2344	64.96
12/4/2020 14:39	187.8	186.7	183.8	2344	64.88
12/4/2020 14:39	187.8	186.8	183.9	2344	65.08
12/4/2020 14:39	187.8	187.1	183.5	2344	65.08
12/4/2020 14:39	187.8	187	183.5	2344	64.99
12/4/2020 14:39	187.8	187.2	183.6	2341	64.92
12/4/2020 14:39	187.8	186.8	183.8	2340	64.92
12/4/2020 14:39	187.8	186.7	183.7	2344	65
12/4/2020 14:39	187.8	187	183.5	2344	64.99
12/4/2020 14:39	187.8	186.8	183.4	2344	64.96

12/4/2020 14:39	187.8	187.1	183.3	2348	65.08
12/4/2020 14:39	187.8	187	184.1	2349	64.92
12/4/2020 14:39	187.8	186.7	184.4	2348	64.96
12/4/2020 14:39	187.8	187.2	184	2347	65
12/4/2020 14:39	187.9	187.1	183.4	2344	64.92
12/4/2020 14:39	187.8	186.9	183.4	2341	65
12/4/2020 14:39	187.8	187.3	183.3	2344	65.05
12/4/2020 14:39	187.8	186.8	183.9	2342	65.04
12/4/2020 14:39	187.9	187.1	183.7	2339	64.96
12/4/2020 14:39	187.8	187.2	183.4	2339	65.04
12/4/2020 14:39	187.8	187	183.5	2344	65
12/4/2020 14:39	187.9	187.3	184.1	2342	64.92
12/4/2020 14:39	187.8	187	184.2	2340	65
12/4/2020 14:40	187.9	187	183.4	2345	64.96
12/4/2020 14:40	187.9	187.4	183.3	2344	64.88
12/4/2020 14:40	187.8	186.7	183.7	2344	64.92
12/4/2020 14:40	187.8	187.2	183.7	2342	65.08
12/4/2020 14:40	187.9	187	183.4	2341	65.04
12/4/2020 14:40	187.9	187.2	184.3	2343	64.88
12/4/2020 14:40	187.9	187	183.7	2343	64.96
12/4/2020 14:40	187.9	187	183.5	2339	64.92
12/4/2020 14:40	187.9	187.2	183.7	2338	64.92
12/4/2020 14:40	187.9	187	183.5	2342	65.01
12/4/2020 14:40	187.8	187.2	183.5	2343	65.08
12/4/2020 14:40	187.9	186.7	183.6	2343	64.84
12/4/2020 14:40	187.9	186.9	184.6	2344	64.99
12/4/2020 14:40	187.9	187.1	183.6	2344	65
12/4/2020 14:40	187.8	187.2	183.2	2344	64.91
12/4/2020 14:40	187.9	187.2	183.8	2349	64.92
12/4/2020 14:40	187.9	187.2	183.8	2344	65.09
12/4/2020 14:40	187.9	186.7	183.6	2347	64.96
12/4/2020 14:40	187.9	187.2	183.9	2346	64.96
12/4/2020 14:40	187.9	186.8	184.5	2342	65.09
12/4/2020 14:40	187.8	186.7	184.2	2348	65
12/4/2020 14:40	187.9	187.2	183.5	2347	65
12/4/2020 14:40	187.9	187	183.4	2343	64.92
12/4/2020 14:40	187.9	187.2	183.6	2344	65
12/4/2020 14:40	187.9	187.2	183.9	2339	64.88
12/4/2020 14:40	187.9	186.8	183.5	2338	64.92
12/4/2020 14:40	187.9	187.2	183.9	2341	65
12/4/2020 14:40	187.9	186.9	183.5	2337	64.92
12/4/2020 14:40	187.9	187.2	183.8	2337	65.01
12/4/2020 14:40	187.8	186.8	183.7	2339	64.96
12/4/2020 14:40	187.9	186.8	183.6	2340	64.92
12/4/2020 14:40	187.9	187.3	183.9	2343	64.92
12/4/2020 14:40	187.8	186.8	184.1	2343	64.96
12/4/2020 14:40	187.9	187.4	184.3	2342	64.96

12/4/2020 14:40	187.9	186.9	183.9	2343	64.83
12/4/2020 14:40	187.8	187	183.9	2341	65.04
12/4/2020 14:40	187.9	187.1	183.6	2337	64.96
12/4/2020 14:40	187.9	187.2	183.6	2337	64.88
12/4/2020 14:40	187.9	187.4	183.7	2337	64.93
12/4/2020 14:40	187.8	187.2	183.6	2338	65.08
12/4/2020 14:40	187.8	186.8	183.7	2335	64.91
12/4/2020 14:40	187.8	187	183.7	2337	64.96
12/4/2020 14:40	187.8	187	183.9	2339	65
12/4/2020 14:40	187.8	187	183.7	2339	64.96
12/4/2020 14:40	187.9	187.3	183.9	2338	65
12/4/2020 14:40	187.8	186.8	183.7	2334	64.92
12/4/2020 14:40	187.8	187.2	183.1	2332	65
12/4/2020 14:40	187.9	186.9	183.5	2333	64.96
12/4/2020 14:40	187.9	186.7	184.2	2333	65.04
12/4/2020 14:40	187.8	187.3	184.4	2337	65
12/4/2020 14:40	187.9	187	184	2334	65
12/4/2020 14:40	187.8	187.1	183.3	2337	64.96
12/4/2020 14:40	187.8	187.2	183.9	2339	64.96
12/4/2020 14:40	187.9	186.7	183.9	2337	65.04
12/4/2020 14:40	187.9	187.3	183.5	2339	64.92
12/4/2020 14:40	187.9	186.9	183.4	2340	64.96
12/4/2020 14:40	187.9	187.3	184.2	2338	65.08
12/4/2020 14:40	187.9	187.2	184.4	2340	64.99
12/4/2020 14:40	187.9	186.8	183.9	2339	64.96
12/4/2020 14:41	188	187	183.6	2342	65.16
12/4/2020 14:41	187.9	187.2	183.7	2337	64.92
12/4/2020 14:41	187.9	187.2	183.5	2341	65.12
12/4/2020 14:41	187.9	187	183.5	2344	65.04
12/4/2020 14:41	187.9	187.1	183.7	2343	64.96
12/4/2020 14:41	187.9	187	184.6	2341	65.08
12/4/2020 14:41	188	187	184.5	2337	65.08
12/4/2020 14:41	188	187.2	183.5	2336	64.92
12/4/2020 14:41	187.9	186.8	183.8	2339	64.92
12/4/2020 14:41	188	187.3	183.8	2343	65.04
12/4/2020 14:41	188	187.2	183.7	2342	64.91
12/4/2020 14:41	187.9	187.2	183.6	2342	64.88
12/4/2020 14:41	187.9	187	183.2	2344	65.04
12/4/2020 14:41	187.9	186.8	183.4	2347	64.91
12/4/2020 14:41	187.9	187	184.4	2343	65
12/4/2020 14:41	187.9	187.4	184.4	2341	65
12/4/2020 14:41	187.9	186.8	184.3	2339	64.92
12/4/2020 14:41	187.9	187.3	183.7	2338	64.96
12/4/2020 14:41	187.9	187	183.3	2338	64.96
12/4/2020 14:41	188	187.4	183.5	2340	64.91
12/4/2020 14:41	187.9	187.2	183.7	2337	65
12/4/2020 14:41	187.9	187.2	183.9	2337	65.04

12/4/2020 14:41	187.9	187.1	183.7	2337	64.92
12/4/2020 14:41	187.9	187	183.6	2338	65
12/4/2020 14:41	187.9	186.8	183.8	2339	65
12/4/2020 14:41	187.9	186.8	183.7	2337	64.92
12/4/2020 14:41	187.9	187.2	184.1	2337	64.88
12/4/2020 14:41	188	187.2	183.8	2333	64.96
12/4/2020 14:41	187.9	187.2	183.7	2330	64.96
12/4/2020 14:41	187.9	187	183.8	2329	64.99
12/4/2020 14:41	187.9	187.3	183.9	2326	64.92
12/4/2020 14:41	187.9	186.8	183.7	2328	64.96
12/4/2020 14:41	187.9	187.1	183.9	2325	64.92
12/4/2020 14:41	188	187.1	183.8	2325	65.04
12/4/2020 14:41	188	187	183.7	2323	64.99
12/4/2020 14:41	188	187.2	183.9	2328	64.88
12/4/2020 14:41	187.9	186.7	183.6	2322	64.99
12/4/2020 14:41	188	187.2	183.9	2324	64.96
12/4/2020 14:41	188	187	183.7	2329	64.96
12/4/2020 14:41	188	187.1	183.9	2329	64.99
12/4/2020 14:41	188	187	183.7	2326	64.92
12/4/2020 14:41	188	186.8	183.4	2326	64.92
12/4/2020 14:41	188	187	183.6	2324	64.83
12/4/2020 14:41	188.1	186.8	183.9	2328	64.84
12/4/2020 14:41	188.1	187.2	184.5	2331	64.92
12/4/2020 14:41	188.1	187.4	184.5	2334	64.99
12/4/2020 14:41	188.2	186.7	184.2	2330	64.88
12/4/2020 14:41	188.2	187	183.3	2329	64.88
12/4/2020 14:41	188.1	186.7	183.4	2331	64.88
12/4/2020 14:41	188.1	187	183.3	2330	64.83
12/4/2020 14:41	188.2	187.4	183.5	2328	64.88
12/4/2020 14:41	188.3	187	183.7	2329	65.08
12/4/2020 14:41	188.2	187.2	183.8	2328	64.88
12/4/2020 14:41	188.3	186.8	184.6	2329	64.99
12/4/2020 14:41	188.3	186.8	183.7	2329	65.08
12/4/2020 14:41	188.3	187.1	183.7	2329	64.99
12/4/2020 14:41	188.3	186.7	183.9	2330	64.92
12/4/2020 14:41	188.3	187	183.5	2337	64.88
12/4/2020 14:42	188.3	187.2	183.5	2337	65.05
12/4/2020 14:42	188.4	186.7	183.7	2334	64.92
12/4/2020 14:42	188.3	187.4	183.4	2335	65.04
12/4/2020 14:42	188.4	186.9	183.8	2337	65.05
12/4/2020 14:42	188.3	187	183.7	2340	64.92
12/4/2020 14:42	188.4	187.2	183.8	2337	64.92
12/4/2020 14:42	188.4	187	184	2336	65.04
12/4/2020 14:42	188.5	187.3	183.8	2339	64.96
12/4/2020 14:42	188.4	187.3	183.9	2337	64.99
12/4/2020 14:42	188.4	187	184	2335	65
12/4/2020 14:42	188.5	186.8	184.2	2336	65

12/4/2020 14:42	188.5	187	184.1	2331	64.92
12/4/2020 14:42	188.5	187.1	183.8	2327	64.96
12/4/2020 14:42	188.5	186.8	184.1	2333	65
12/4/2020 14:42	188.5	186.9	183.9	2330	64.92
12/4/2020 14:42	188.5	187.2	183.6	2337	64.96
12/4/2020 14:42	188.5	186.8	183.7	2339	65
12/4/2020 14:42	188.5	187.2	183.8	2343	65
12/4/2020 14:42	188.5	187.2	183.8	2340	65
12/4/2020 14:42	188.5	186.9	184	2346	65
12/4/2020 14:42	188.4	187	183.7	2343	65
12/4/2020 14:42	188.5	186.8	183.8	2340	64.88
12/4/2020 14:42	188.5	187.1	183.6	2342	64.92
12/4/2020 14:42	188.5	186.8	183.7	2341	65.08
12/4/2020 14:42	188.5	187.2	184.1	2338	64.96
12/4/2020 14:42	188.5	187	184.4	2339	64.92
12/4/2020 14:42	188.5	186.8	184.7	2339	65.12
12/4/2020 14:42	188.5	187	184.4	2339	65.04
12/4/2020 14:42	188.5	186.8	183.9	2340	65.08
12/4/2020 14:42	188.5	187.1	183.9	2342	64.96
12/4/2020 14:42	188.5	187	183.5	2346	65.04
12/4/2020 14:42	188.5	186.8	183.6	2340	65.03
12/4/2020 14:42	188.5	187.2	183.9	2340	65.08
12/4/2020 14:42	188.5	187.1	183.8	2343	64.96
12/4/2020 14:42	188.4	187	184	2342	64.84
12/4/2020 14:42	188.5	187.2	184.2	2342	65.03
12/4/2020 14:42	188.4	186.9	183.8	2343	65.12
12/4/2020 14:42	188.4	187	183.9	2344	65.08
12/4/2020 14:42	188.5	186.8	183.8	2342	64.99
12/4/2020 14:42	188.4	186.8	183.8	2346	65.08
12/4/2020 14:42	188.4	187.2	184.1	2343	64.88
12/4/2020 14:42	188.4	186.8	183.8	2346	64.99
12/4/2020 14:42	188.4	187	184.1	2348	65
12/4/2020 14:42	188.4	186.7	183.9	2346	64.96
12/4/2020 14:42	188.4	186.7	183.8	2346	65
12/4/2020 14:42	188.4	187.1	183.8	2346	64.96
12/4/2020 14:42	188.3	187	183.9	2348	65.04
12/4/2020 14:42	188.4	186.7	183.7	2349	64.96
12/4/2020 14:42	188.3	186.9	183.9	2348	65
12/4/2020 14:42	188.4	186.8	183.7	2354	65.04
12/4/2020 14:42	188.3	187.2	183.8	2353	65.04
12/4/2020 14:42	188.3	186.8	183.4	2349	65
12/4/2020 14:42	188.3	186.9	183.5	2347	64.88
12/4/2020 14:42	188.3	187.3	183.8	2341	65.08
12/4/2020 14:42	188.3	186.8	184.1	2340	64.96
12/4/2020 14:42	188.3	187.3	184.2	2344	64.96
12/4/2020 14:42	188.3	187.2	184.7	2342	65.08
12/4/2020 14:42	188.3	187	184.6	2344	64.99

12/4/2020 14:42	188.3	187.2	183.7	2339	65.04
12/4/2020 14:42	188.3	186.7	183.8	2341	64.97
12/4/2020 14:43	188.3	187.2	183.7	2340	65.04
12/4/2020 14:43	188.3	187.3	183.9	2346	65.08
12/4/2020 14:43	188.3	187	183.9	2346	65.12
12/4/2020 14:43	188.3	187.2	183.4	2348	64.99
12/4/2020 14:43	188.4	187.1	184.1	2346	65.04
12/4/2020 14:43	188.3	187.4	184.5	2347	64.88
12/4/2020 14:43	188.3	187	183.5	2345	65.08
12/4/2020 14:43	188.3	186.9	184	2346	64.91
12/4/2020 14:43	188.3	187.2	183.8	2347	64.95
12/4/2020 14:43	188.3	186.9	183.9	2348	65.04
12/4/2020 14:43	188.3	187	183.8	2347	65
12/4/2020 14:43	188.3	187.3	184.6	2344	65.04
12/4/2020 14:43	188.3	186.8	184.5	2346	64.92
12/4/2020 14:43	188.3	187	183.5	2342	64.96
12/4/2020 14:43	188.4	186.8	183.8	2343	64.96
12/4/2020 14:43	188.3	187.1	184.1	2348	64.96
12/4/2020 14:43	188.3	187	184	2348	64.96
12/4/2020 14:43	188.3	186.7	183.9	2347	64.91
12/4/2020 14:43	188.3	186.8	184	2344	64.96
12/4/2020 14:43	188.3	186.8	184.9	2343	65.04
12/4/2020 14:43	188.3	187	184.7	2340	64.96
12/4/2020 14:43	188.3	187.2	183.6	2343	64.92
12/4/2020 14:43	188.4	187	183.8	2344	65
12/4/2020 14:43	188.4	187.3	183.8	2347	64.92
12/4/2020 14:43	188.3	186.8	184.1	2347	64.96
12/4/2020 14:43	188.3	186.8	184.2	2347	65
12/4/2020 14:43	188.3	187.2	183.8	2348	64.92
12/4/2020 14:43	188.3	187.1	184.1	2347	64.96
12/4/2020 14:43	188.3	187.4	184.7	2348	64.96
12/4/2020 14:43	188.3	186.8	184.4	2344	64.92
12/4/2020 14:43	188.3	187	183.9	2340	65.04
12/4/2020 14:43	188.3	187.2	183.8	2344	65.16
12/4/2020 14:43	188.3	187.2	184.1	2344	64.88
12/4/2020 14:43	188.3	187.1	184.1	2344	64.99
12/4/2020 14:43	188.3	186.8	184.1	2344	65.08
12/4/2020 14:43	188.3	187	183.8	2341	65
12/4/2020 14:43	188.3	186.8	183.7	2342	64.95
12/4/2020 14:43	188.3	186.9	184.7	2340	65.04
12/4/2020 14:43	188.3	187.2	184.8	2341	65
12/4/2020 14:43	188.3	187	183.9	2343	64.96
12/4/2020 14:43	188.3	187	183.8	2338	64.99
12/4/2020 14:43	188.3	187.4	184.1	2344	64.96
12/4/2020 14:43	188.3	187	184.1	2347	65
12/4/2020 14:43	188.3	187.1	184.2	2346	64.96
12/4/2020 14:43	188.3	187.2	184.2	2346	64.88

12/4/2020 14:43	188.3	187.3	183.9	2346	65.04
12/4/2020 14:43	188.2	187	183.9	2344	64.96
12/4/2020 14:43	188.3	186.8	183.8	2346	65
12/4/2020 14:43	188.3	187.1	184.3	2346	65
12/4/2020 14:43	188.3	186.8	184.6	2343	64.96
12/4/2020 14:43	188.3	187	184.6	2344	64.87
12/4/2020 14:43	188.3	187.2	184.9	2347	65.04
12/4/2020 14:43	188.3	187	184.9	2346	64.87
12/4/2020 14:43	188.3	187	184.6	2345	64.96
12/4/2020 14:43	188.3	186.7	184.8	2346	65.04
12/4/2020 14:43	188.2	187.1	184.6	2348	64.96
12/4/2020 14:43	188.3	187.2	184.5	2346	65.04
12/4/2020 14:43	188.3	186.9	184.2	2346	65.04
12/4/2020 14:43	188.2	186.9	183.6	2348	64.92
12/4/2020 14:44	188.3	186.8	184	2350	64.96
12/4/2020 14:44	188.3	186.8	184.1	2346	64.92
12/4/2020 14:44	188.3	187.2	184	2346	65.01
12/4/2020 14:44	188.3	186.8	184.5	2344	64.92
12/4/2020 14:44	188.3	187	184.1	2347	65.04
12/4/2020 14:44	188.3	187.2	183.8	2348	65
12/4/2020 14:44	188.3	186.7	183.9	2351	65.03
12/4/2020 14:44	188.3	187.2	183.7	2350	65.04
12/4/2020 14:44	188.3	187	184.5	2350	65.08
12/4/2020 14:44	188.3	187.2	184.6	2351	64.88
12/4/2020 14:44	188.3	187.3	184.1	2351	65.04
12/4/2020 14:44	188.2	187.2	183.7	2348	65.08
12/4/2020 14:44	188.3	187.3	183.9	2352	64.92
12/4/2020 14:44	188.3	187	184.2	2351	65.03
12/4/2020 14:44	188.3	187.2	183.9	2351	64.92
12/4/2020 14:44	188.3	187.2	183.5	2347	65.08
12/4/2020 14:44	188.3	186.8	184	2346	64.99
12/4/2020 14:44	188.3	187	184.7	2351	64.96
12/4/2020 14:44	188.3	186.8	184.9	2348	65.04
12/4/2020 14:44	188.3	187.1	184	2351	64.96
12/4/2020 14:44	188.3	187.2	183.7	2352	64.91
12/4/2020 14:44	188.3	186.8	184.2	2346	65
12/4/2020 14:44	188.3	187	184.4	2347	64.88
12/4/2020 14:44	188.3	187.2	183.9	2343	65
12/4/2020 14:44	188.3	186.9	183.9	2344	65
12/4/2020 14:44	188.3	187.3	183.6	2348	65
12/4/2020 14:44	188.3	186.8	183.6	2349	64.96
12/4/2020 14:44	188.3	187.1	184.1	2348	64.96
12/4/2020 14:44	188.3	187	184.5	2351	65.04
12/4/2020 14:44	188.3	187.1	184.9	2352	64.92
12/4/2020 14:44	188.3	187.2	184.6	2349	64.96
12/4/2020 14:44	188.4	186.8	184	2351	65.04
12/4/2020 14:44	188.3	187	184.1	2351	64.92

12/4/2020 14:44	188.3	186.8	183.7	2354	64.88
12/4/2020 14:44	188.3	187	183.9	2353	65
12/4/2020 14:44	188.3	187.2	184.1	2351	64.96
12/4/2020 14:44	188.4	186.9	183.7	2350	64.92
12/4/2020 14:44	188.4	187.2	184	2348	64.92
12/4/2020 14:44	188.4	186.8	183.8	2352	64.96
12/4/2020 14:44	188.4	186.8	183.9	2350	64.95
12/4/2020 14:44	188.3	187.2	184	2347	64.96
12/4/2020 14:44	188.4	186.8	183.7	2348	65.01
12/4/2020 14:44	188.4	186.8	183.8	2349	65.04
12/4/2020 14:44	188.4	186.7	183.8	2351	64.84
12/4/2020 14:44	188.4	186.8	184.1	2356	65
12/4/2020 14:44	188.5	187.2	184.9	2352	64.99
12/4/2020 14:44	188.5	186.9	185	2350	64.96
12/4/2020 14:44	188.4	187.3	184.6	2348	64.96
12/4/2020 14:44	188.5	187	183.7	2350	64.84
12/4/2020 14:44	188.5	186.8	183.5	2348	64.84
12/4/2020 14:44	188.5	187.3	183.9	2350	64.88
12/4/2020 14:44	188.5	187.1	184.1	2350	64.93
12/4/2020 14:44	188.5	187.2	184.2	2347	64.88
12/4/2020 14:44	188.5	187	184.3	2346	64.88
12/4/2020 14:44	188.5	186.8	183.8	2347	65.09
12/4/2020 14:44	188.5	187.2	183.8	2342	65
12/4/2020 14:44	188.5	186.9	184.4	2342	65.04
12/4/2020 14:44	188.5	187.2	184.6	2349	64.96
12/4/2020 14:44	188.6	187.3	183.8	2349	64.96
12/4/2020 14:44	188.5	186.7	183.7	2347	65
12/4/2020 14:45	188.5	187	184.2	2347	65
12/4/2020 14:45	188.6	187	184.2	2349	65.04
12/4/2020 14:45	188.6	186.8	183.8	2344	64.96
12/4/2020 14:45	188.5	187.2	184.4	2346	64.92
12/4/2020 14:45	188.6	187	184.6	2350	65
12/4/2020 14:45	188.6	187	183.8	2343	64.96
12/4/2020 14:45	188.6	186.7	184.2	2343	64.96
12/4/2020 14:45	188.6	187	184.4	2340	64.96
12/4/2020 14:45	188.6	186.8	184.4	2344	64.91
12/4/2020 14:45	188.6	186.8	184.1	2341	64.92
12/4/2020 14:45	188.7	187	184.7	2344	64.96
12/4/2020 14:45	188.7	187	184.6	2348	65
12/4/2020 14:45	188.7	186.8	183.8	2348	64.91
12/4/2020 14:45	188.7	186.8	184.1	2354	64.96
12/4/2020 14:45	188.7	188.1	184.3	2357	65.04
12/4/2020 14:45	188.8	188.1	184.1	2355	64.91
12/4/2020 14:45	188.7	187.1	183.9	2352	65
12/4/2020 14:45	188.7	188.1	184.3	2353	64.92
12/4/2020 14:45	188.7	188	184.9	2355	64.92
12/4/2020 14:45	188.8	188.1	184.4	2356	64.99

12/4/2020 14:45	188.8	188.1	183.9	2355	64.92
12/4/2020 14:45	188.7	187.9	183.9	2351	64.96
12/4/2020 14:45	188.8	188.1	184.5	2354	64.99
12/4/2020 14:45	188.8	188.1	184.3	2348	64.92
12/4/2020 14:45	188.8	188	183.9	2348	64.96
12/4/2020 14:45	188.8	188	184.6	2347	64.96
12/4/2020 14:45	188.8	188.1	184.9	2347	65.08
12/4/2020 14:45	188.7	188.1	184.4	2350	64.95
12/4/2020 14:45	188.7	188	183.9	2352	65.08
12/4/2020 14:45	188.8	187.9	183.8	2347	64.92
12/4/2020 14:45	188.8	188	184.4	2350	65.08
12/4/2020 14:45	188.8	188.1	184.3	2351	64.92
12/4/2020 14:45	188.8	188.1	184.2	2349	64.99
12/4/2020 14:45	188.8	188.1	184.2	2349	65
12/4/2020 14:45	188.8	188.1	183.9	2353	64.96
12/4/2020 14:45	188.8	188	184.1	2346	64.79
12/4/2020 14:45	188.8	188	184.6	2348	64.88
12/4/2020 14:45	188.8	188	184.6	2347	65.04
12/4/2020 14:45	188.8	188.1	185.1	2347	64.84
12/4/2020 14:45	188.8	188.4	184.9	2344	65.03
12/4/2020 14:45	188.8	188.1	184.9	2346	65.04
12/4/2020 14:45	188.8	188.1	184.7	2348	64.83
12/4/2020 14:45	188.8	188	184.6	2348	64.88
12/4/2020 14:45	188.7	188.1	184.6	2347	65.12
12/4/2020 14:45	188.8	188.1	184.2	2347	64.92
12/4/2020 14:45	188.8	188.1	183.8	2341	64.88
12/4/2020 14:45	188.8	188	183.9	2339	64.96
12/4/2020 14:45	188.8	188.1	183.9	2340	65.08
12/4/2020 14:45	188.8	188	183.8	2340	64.96
12/4/2020 14:45	188.8	188.1	184.2	2335	64.99
12/4/2020 14:45	188.9	188	184.2	2340	64.92
12/4/2020 14:45	188.8	188.1	184.2	2340	64.92
12/4/2020 14:45	188.9	188.1	184.2	2341	64.92
12/4/2020 14:45	188.8	188.1	184.1	2344	65
12/4/2020 14:45	188.8	188.1	184.2	2344	64.96
12/4/2020 14:45	188.8	188.1	184.2	2343	64.92
12/4/2020 14:45	188.8	188.2	184.2	2345	65
12/4/2020 14:45	188.8	187.9	184.3	2348	65.04
12/4/2020 14:45	188.8	188	184	2344	64.92
12/4/2020 14:46	188.9	188.1	184.1	2347	64.92
12/4/2020 14:46	188.9	187.9	183.9	2346	65
12/4/2020 14:46	188.8	188	184.2	2352	64.96
12/4/2020 14:46	188.9	188	184	2351	65
12/4/2020 14:46	188.9	187.9	183.9	2353	65.09
12/4/2020 14:46	188.9	187.9	184.1	2355	64.92
12/4/2020 14:46	189	188	184.3	2350	64.96
12/4/2020 14:46	188.9	187.9	184	2355	65.04

12/4/2020 14:46	188.9	188	184.4	2357	64.96
12/4/2020 14:46	188.9	187.9	184.3	2359	65
12/4/2020 14:46	189	188.1	183.9	2358	64.92
12/4/2020 14:46	189	188.1	184.4	2354	64.92
12/4/2020 14:46	189	188.1	184.2	2356	64.88
12/4/2020 14:46	189	188.1	184.6	2353	64.83
12/4/2020 14:46	189	188.1	184.6	2352	65.01
12/4/2020 14:46	189	188.1	184.2	2348	64.99
12/4/2020 14:46	189	188	184.2	2347	65.04
12/4/2020 14:46	189	188	183.9	2347	65
12/4/2020 14:46	189	188.1	184.1	2350	64.83
12/4/2020 14:46	189	188	184.1	2350	64.96
12/4/2020 14:46	189	188.1	184.6	2351	64.96
12/4/2020 14:46	189	188	185.1	2351	64.96
12/4/2020 14:46	189.1	188.1	184.9	2348	64.88
12/4/2020 14:46	189.1	188	184.3	2348	64.96
12/4/2020 14:46	189.1	188.1	184.2	2354	64.93
12/4/2020 14:46	189.1	188.1	184.2	2350	65.03
12/4/2020 14:46	189.1	188	184.3	2348	64.92
12/4/2020 14:46	189.2	188.1	184.6	2351	65.08
12/4/2020 14:46	189.1	187.9	184.4	2350	64.88
12/4/2020 14:46	189.2	188	184.2	2353	64.88
12/4/2020 14:46	189.2	188.1	184.1	2355	65.08
12/4/2020 14:46	189.2	188	183.9	2358	64.88
12/4/2020 14:46	189.3	187.9	183.9	2360	64.92
12/4/2020 14:46	189.2	188	184.3	2359	65.04
12/4/2020 14:46	189.3	187.9	184.6	2362	65
12/4/2020 14:46	189.2	188	184.7	2359	65.04
12/4/2020 14:46	189.2	187.9	185	2356	65
12/4/2020 14:46	189.3	188.1	185.1	2355	65
12/4/2020 14:46	189.3	188.1	184.7	2354	64.96
12/4/2020 14:46	189.3	188.1	184.4	2350	64.96
12/4/2020 14:46	189.3	188	184.1	2352	65.05
12/4/2020 14:46	189.3	188	183.8	2347	65
12/4/2020 14:46	189.3	187.9	183.9	2342	64.96
12/4/2020 14:46	189.3	187.9	184.3	2344	65.08
12/4/2020 14:46	189.4	187.9	184.4	2351	65
12/4/2020 14:46	189.4	188.1	184.4	2347	65
12/4/2020 14:46	189.4	187.9	183.9	2346	65
12/4/2020 14:46	189.4	188.1	184.9	2346	64.96
12/4/2020 14:46	189.4	188	184.4	2346	64.96
12/4/2020 14:46	189.5	187.9	184.1	2350	65.04
12/4/2020 14:46	189.5	188.1	184.6	2355	65.12
12/4/2020 14:46	189.4	188.1	184.6	2355	65.03
12/4/2020 14:46	189.5	188	184	2354	65.04
12/4/2020 14:46	189.5	188	185.2	2355	64.96
12/4/2020 14:46	189.5	187.9	184.7	2355	64.92

12/4/2020 14:46	189.5	188.1	183.9	2355	64.96
12/4/2020 14:46	189.5	188.1	184.5	2352	64.88
12/4/2020 14:46	189.6	188.1	184.5	2352	64.96
12/4/2020 14:46	189.6	188.1	184.6	2348	64.95
12/4/2020 14:47	189.6	188.1	184.6	2347	65.03
12/4/2020 14:47	189.5	188	185.2	2351	65.12
12/4/2020 14:47	189.6	188.1	184.7	2350	64.96
12/4/2020 14:47	189.6	188.1	184.1	2348	65.08
12/4/2020 14:47	189.6	187.9	184.2	2347	65.08
12/4/2020 14:47	189.6	188.2	184.6	2346	65.04
12/4/2020 14:47	189.6	188.1	184.4	2348	64.99
12/4/2020 14:47	189.6	188.1	184.2	2347	64.88
12/4/2020 14:47	189.6	188	185.3	2352	65.08
12/4/2020 14:47	189.5	188.2	184.9	2352	64.99
12/4/2020 14:47	189.6	188	184.4	2351	64.99
12/4/2020 14:47	189.6	188.1	184.2	2350	65.08
12/4/2020 14:47	189.6	187.8	184.3	2353	64.87
12/4/2020 14:47	189.6	188.1	184.4	2350	65
12/4/2020 14:47	189.6	188	184.4	2356	65.04
12/4/2020 14:47	189.6	188.1	184.6	2355	65
12/4/2020 14:47	189.6	188.3	184.6	2353	64.87
12/4/2020 14:47	189.6	188.1	184.2	2354	64.96
12/4/2020 14:47	189.7	188.1	184.4	2355	65
12/4/2020 14:47	189.6	187.9	184.4	2357	64.96
12/4/2020 14:47	189.7	188.1	184.7	2356	65.04
12/4/2020 14:47	189.7	188	185.3	2352	65
12/4/2020 14:47	189.7	188.1	185.1	2350	64.96
12/4/2020 14:47	189.7	188	184.7	2345	64.92
12/4/2020 14:47	189.6	188	184.6	2347	64.92
12/4/2020 14:47	189.7	188	184	2346	65.08
12/4/2020 14:47	189.7	188.1	184.2	2344	64.99
12/4/2020 14:47	189.7	188.1	184.3	2346	64.96
12/4/2020 14:47	189.7	188.1	184.5	2344	64.96
12/4/2020 14:47	189.8	188	184.5	2346	65.03
12/4/2020 14:47	189.7	188	184.3	2345	65.03
12/4/2020 14:47	189.8	188.1	184.7	2346	64.92
12/4/2020 14:47	189.8	188.1	184.6	2346	64.99
12/4/2020 14:47	189.8	188.1	184.1	2346	65.03
12/4/2020 14:47	189.8	187.9	184.2	2346	65.12
12/4/2020 14:47	189.8	188.1	184.6	2347	64.92
12/4/2020 14:47	189.8	188.1	185.1	2350	65.08
12/4/2020 14:47	189.8	188.1	185.3	2354	64.96
12/4/2020 14:47	189.9	188	184.7	2352	64.92
12/4/2020 14:47	189.8	188.1	184.2	2350	64.96
12/4/2020 14:47	189.8	188.1	184.1	2349	65.05
12/4/2020 14:47	189.8	188.1	184.2	2349	65.04
12/4/2020 14:47	189.8	188.1	184.6	2348	64.99

12/4/2020 14:47	189.9	188.2	184.4	2344	65.09
12/4/2020 14:47	189.8	187.9	184.6	2343	65
12/4/2020 14:47	189.8	188	184.8	2338	65
12/4/2020 14:47	189.8	188	184.5	2338	65.04
12/4/2020 14:47	189.8	187.9	184.6	2337	64.96
12/4/2020 14:47	189.9	188.1	184.7	2337	64.96
12/4/2020 14:47	189.9	188	184.6	2337	64.96
12/4/2020 14:47	189.9	188.2	184.8	2340	65.05
12/4/2020 14:47	189.9	188.2	184.7	2343	64.88
12/4/2020 14:47	189.9	187.9	184.8	2341	64.96
12/4/2020 14:47	189.9	188.3	184.6	2350	65.12
12/4/2020 14:47	189.9	188	184.6	2353	64.88
12/4/2020 14:47	190.2	188	184.6	2354	65.04
12/4/2020 14:47	190	188	184.6	2352	64.88
12/4/2020 14:47	189.8	188.1	184.5	2354	65.04
12/4/2020 14:47	189.8	188.1	184.6	2358	64.96
12/4/2020 14:47	189.9	188.2	184.4	2357	65.04
12/4/2020 14:48	189.9	188.1	184.2	2355	65.04
12/4/2020 14:48	189.9	188	184.3	2351	64.99
12/4/2020 14:48	189.9	187.9	184.1	2353	65.04
12/4/2020 14:48	190	188	184.3	2356	65.08
12/4/2020 14:48	190	188	184.5	2351	64.88
12/4/2020 14:48	190	188.1	184.2	2352	64.96
12/4/2020 14:48	189.9	188.1	184.2	2351	65.05
12/4/2020 14:48	190	188	184.4	2354	64.91
12/4/2020 14:48	190	188	184.4	2354	65
12/4/2020 14:48	190	188.1	184.4	2351	65.08
12/4/2020 14:48	190	188.1	184.4	2352	65.04
12/4/2020 14:48	190	188.1	184.1	2350	65
12/4/2020 14:48	190	188	184.6	2348	64.92
12/4/2020 14:48	190	188.1	184.1	2350	65
12/4/2020 14:48	190	188.1	184.2	2353	64.92
12/4/2020 14:48	190	188	184.6	2353	64.96
12/4/2020 14:48	190	188	184.6	2355	65
12/4/2020 14:48	190	188.1	184.8	2353	64.92
12/4/2020 14:48	190.1	188.1	184.8	2355	64.88
12/4/2020 14:48	190.1	187.9	184.4	2356	65.05
12/4/2020 14:48	190.1	188	184.9	2354	64.93
12/4/2020 14:48	190	188.1	184.6	2348	64.99
12/4/2020 14:48	190.1	188	184.6	2350	64.99
12/4/2020 14:48	190.1	187.9	185.3	2355	65
12/4/2020 14:48	190.1	188	185.4	2351	64.88
12/4/2020 14:48	190.1	187.9	184.8	2354	64.88
12/4/2020 14:48	190.1	188.1	184.3	2356	65.08
12/4/2020 14:48	190	188	184.6	2355	64.99
12/4/2020 14:48	190.1	188	185	2352	65.03
12/4/2020 14:48	190.1	188	184.4	2350	64.96

12/4/2020 14:48	190	188	185.3	2350	64.84
12/4/2020 14:48	190.1	188.1	185.3	2350	64.88
12/4/2020 14:48	190.1	188.1	184.2	2350	64.88
12/4/2020 14:48	190.1	188	184.7	2355	64.99
12/4/2020 14:48	190	187.9	185.1	2357	65.04
12/4/2020 14:48	190.1	188.1	184.8	2358	65.12
12/4/2020 14:48	190.1	188.1	184.8	2357	65
12/4/2020 14:48	190.1	188.1	185.4	2357	65.04
12/4/2020 14:48	190.1	188.1	184.9	2357	64.96
12/4/2020 14:48	190.1	188.1	184.3	2356	65.13
12/4/2020 14:48	190.1	188	184.8	2355	64.92
12/4/2020 14:48	190.1	188.1	185.1	2356	64.96
12/4/2020 14:48	190	188.1	184.9	2355	65
12/4/2020 14:48	190	188.1	184.4	2355	64.96
12/4/2020 14:48	190.1	187.9	185.6	2357	64.96
12/4/2020 14:48	190.1	188.1	184.8	2362	65.05
12/4/2020 14:48	190	188.1	184.6	2359	64.92
12/4/2020 14:48	190	188	184.6	2359	64.88
12/4/2020 14:48	190	188.1	184.8	2357	65.04
12/4/2020 14:48	190.1	188.1	184.8	2357	64.93
12/4/2020 14:48	190	188	184.5	2357	64.83
12/4/2020 14:48	190	188	184.6	2358	64.99
12/4/2020 14:48	190	188.1	185.4	2359	65.08
12/4/2020 14:48	190	188	185.5	2359	64.88
12/4/2020 14:48	190.1	188.1	185.3	2358	65.08
12/4/2020 14:48	190	188.1	184.6	2359	65
12/4/2020 14:48	190	188.1	184.2	2359	64.99
12/4/2020 14:48	190.1	188.2	184.5	2354	64.92
12/4/2020 14:48	190	187.9	184.5	2355	65.08
12/4/2020 14:49	190.1	187.9	184.8	2357	64.96
12/4/2020 14:49	190.1	188.1	184.9	2356	64.88
12/4/2020 14:49	190.1	188	184.8	2356	64.96
12/4/2020 14:49	190	188.2	184.8	2354	65
12/4/2020 14:49	190	188.2	185	2355	64.99
12/4/2020 14:49	190	188.2	184.6	2357	65.04
12/4/2020 14:49	190	188.2	184.8	2355	65.04
12/4/2020 14:49	190.1	188.1	184.9	2357	64.91
12/4/2020 14:49	190	188.1	184.6	2353	65
12/4/2020 14:49	190.1	188.1	184.8	2358	65.05
12/4/2020 14:49	190	188.1	184.7	2356	65
12/4/2020 14:49	190	188.1	184.6	2355	65
12/4/2020 14:49	190	188.1	184.9	2355	64.92
12/4/2020 14:49	190	188	185.2	2359	65.12
12/4/2020 14:49	190	188	185.3	2357	64.96
12/4/2020 14:49	190	188.1	185.4	2358	65.04
12/4/2020 14:49	190	188.1	185.4	2358	65
12/4/2020 14:49	190	188.1	185.4	2352	65.04

12/4/2020 14:49	190	187.9	185.1	2350	65.04
12/4/2020 14:49	190	187.9	185	2357	64.96
12/4/2020 14:49	190	187.9	185.1	2360	64.88
12/4/2020 14:49	190	187.9	184.7	2358	64.83
12/4/2020 14:49	190	188.1	184.8	2360	65.08
12/4/2020 14:49	190	188.1	184.7	2358	64.92
12/4/2020 14:49	190	188.1	184.7	2355	65.03
12/4/2020 14:49	190	188.2	184.9	2357	64.92
12/4/2020 14:49	190	188.1	185.1	2358	64.92
12/4/2020 14:49	190	188.1	184.9	2355	64.99
12/4/2020 14:49	190	188.1	185.1	2355	64.99
12/4/2020 14:49	190	188	184.6	2355	64.93
12/4/2020 14:49	190	188	184.7	2351	64.83
12/4/2020 14:49	190	187.9	184.8	2348	65.03
12/4/2020 14:49	189.9	188.1	185.3	2351	65.17
12/4/2020 14:49	190	188.1	185.5	2351	65.08
12/4/2020 14:49	189.9	188.2	184.6	2350	64.99
12/4/2020 14:49	190	188.1	185	2353	65.12
12/4/2020 14:49	190	188.1	185.1	2351	65.04
12/4/2020 14:49	189.9	188.1	184.9	2348	65.04
12/4/2020 14:49	189.9	188.1	184.5	2351	64.99
12/4/2020 14:49	189.9	188	184.6	2351	65.04
12/4/2020 14:49	189.9	188	185.4	2353	64.96
12/4/2020 14:49	189.8	188	185.5	2354	64.92
12/4/2020 14:49	189.9	187.9	184.8	2354	65.09
12/4/2020 14:49	189.9	188.2	184.6	2353	64.92
12/4/2020 14:49	189.9	188	185.2	2351	64.96
12/4/2020 14:49	189.9	188	184.8	2355	65.05
12/4/2020 14:49	189.9	188	184.6	2358	64.96
12/4/2020 14:49	189.8	187.9	184.4	2352	64.92
12/4/2020 14:49	189.8	188	184.8	2355	65
12/4/2020 14:49	189.9	188	185.8	2357	64.92
12/4/2020 14:49	189.8	188.1	185.3	2356	65.04
12/4/2020 14:49	189.9	188.1	184.6	2359	64.96
12/4/2020 14:49	189.8	188.2	184.9	2359	65
12/4/2020 14:49	189.9	188.1	184.8	2354	64.88
12/4/2020 14:49	189.8	188.2	184.8	2352	64.92
12/4/2020 14:49	189.8	188.1	184.7	2349	65.08
12/4/2020 14:49	189.8	188.1	185	2348	65.03
12/4/2020 14:49	189.9	188	185.6	2345	64.84
12/4/2020 14:49	189.9	188.1	185.3	2348	65.01
12/4/2020 14:49	190	188	184.6	2346	64.95
12/4/2020 14:50	189.9	188.1	185.1	2344	64.96
12/4/2020 14:50	189.9	188.1	184.9	2348	64.96
12/4/2020 14:50	190	188.1	184.8	2347	65.08
12/4/2020 14:50	189.9	187.9	185	2346	64.96
12/4/2020 14:50	190	188.2	184.6	2347	65.08

12/4/2020 14:50	190	187.9	184.6	2348	65.04
12/4/2020 14:50	190	188.1	184.7	2344	64.99
12/4/2020 14:50	190	189.3	185.6	2339	65.04
12/4/2020 14:50	190	188.6	185.7	2340	65.08
12/4/2020 14:50	189.9	188.6	184.9	2338	64.96
12/4/2020 14:50	189.9	188.1	184.7	2337	64.88
12/4/2020 14:50	190	188.1	184.8	2340	64.96
12/4/2020 14:50	190	188.1	184.9	2341	64.88
12/4/2020 14:50	189.9	187.9	185	2340	64.96
12/4/2020 14:50	190	188.1	184.8	2341	65.05
12/4/2020 14:50	190	188	184.6	2341	64.96
12/4/2020 14:50	190	188.9	185.3	2338	64.96
12/4/2020 14:50	190	189.1	185.6	2341	65
12/4/2020 14:50	190	187.9	185.1	2342	64.96
12/4/2020 14:50	189.9	188.9	184.9	2337	64.96
12/4/2020 14:50	189.9	189.2	184.7	2335	64.92
12/4/2020 14:50	189.9	188.8	185.1	2340	65.04
12/4/2020 14:50	190	189	185.1	2340	64.88
12/4/2020 14:50	189.9	189.1	184.6	2340	64.92
12/4/2020 14:50	190	188.4	184.6	2343	65.01
12/4/2020 14:50	190	189	185.3	2344	64.99
12/4/2020 14:50	190	188.8	185.5	2344	65.04
12/4/2020 14:50	190	188.9	184.9	2351	65.17
12/4/2020 14:50	189.9	189.1	184.6	2350	64.92
12/4/2020 14:50	190	189.1	185.1	2347	65.04
12/4/2020 14:50	190	188.9	184.9	2344	65.04
12/4/2020 14:50	190	188.7	184.6	2344	65.08
12/4/2020 14:50	190	188.8	184.8	2344	64.99
12/4/2020 14:50	190	188.8	184.6	2344	64.84
12/4/2020 14:50	190	188.8	185.6	2342	65.04
12/4/2020 14:50	190	189.1	185.8	2340	64.83
12/4/2020 14:50	190	189	184.7	2340	64.96
12/4/2020 14:50	190	188.8	184.7	2345	65.12
12/4/2020 14:50	190	188.9	185.1	2347	64.88
12/4/2020 14:50	190	189	185.3	2351	65.04
12/4/2020 14:50	190.1	189.2	185.3	2348	65.08
12/4/2020 14:50	190.1	189	184.6	2348	64.96
12/4/2020 14:50	190.1	189.2	185.2	2349	65
12/4/2020 14:50	190.1	188.7	185.8	2351	65.04
12/4/2020 14:50	190.1	188.6	185.5	2355	65.09
12/4/2020 14:50	190.1	189	184.8	2351	64.96
12/4/2020 14:50	190.1	188.9	184.8	2346	65
12/4/2020 14:50	190.1	189.1	185.1	2348	65
12/4/2020 14:50	190.2	189	185.4	2347	64.96
12/4/2020 14:50	190.2	188.6	184.9	2348	65
12/4/2020 14:50	190.1	188.9	185.3	2351	65.05
12/4/2020 14:50	190.1	189.1	184.9	2351	64.96

12/4/2020 14:50	190.2	189.3	184.7	2350	65.08
12/4/2020 14:50	190.2	189.1	184.8	2353	64.96
12/4/2020 14:50	190.3	188.9	184.9	2354	65.08
12/4/2020 14:50	190.2	188.9	184.6	2355	64.88
12/4/2020 14:50	190.3	188.7	184.8	2355	65.12
12/4/2020 14:50	190.3	188.8	184.9	2354	64.93
12/4/2020 14:50	190.3	189.3	184.9	2351	64.88
12/4/2020 14:51	190.3	188.7	185.3	2354	64.92
12/4/2020 14:51	190.3	189.1	185.2	2357	65.08
12/4/2020 14:51	190.3	188.9	185.4	2356	64.96
12/4/2020 14:51	190.2	188.8	185.3	2352	65.04
12/4/2020 14:51	190.2	188.9	185.4	2350	64.96
12/4/2020 14:51	190.2	188.6	185.6	2350	65
12/4/2020 14:51	190.3	188.9	185.3	2350	64.88
12/4/2020 14:51	190.3	188.6	185.3	2350	64.96
12/4/2020 14:51	190.3	188.8	185.2	2354	65.04
12/4/2020 14:51	190.3	189.1	184.9	2354	64.92
12/4/2020 14:51	190.2	188.8	185.2	2354	65
12/4/2020 14:51	190.2	188.8	185.5	2354	65
12/4/2020 14:51	190.2	188.9	185.2	2351	64.92
12/4/2020 14:51	190.2	189.1	185.4	2353	65
12/4/2020 14:51	190.3	189.1	185.4	2348	65
12/4/2020 14:51	190.3	188.8	185.4	2350	65.04
12/4/2020 14:51	190.2	188.9	185.6	2349	64.83
12/4/2020 14:51	190.3	188.7	185.6	2348	64.92
12/4/2020 14:51	190.3	189.1	185.8	2348	65.01
12/4/2020 14:51	190.2	189	186	2352	64.95
12/4/2020 14:51	190.2	188.9	186	2351	65.04
12/4/2020 14:51	190.2	189	185.6	2353	64.96
12/4/2020 14:51	190.3	188.8	185.4	2348	65.04
12/4/2020 14:51	190.3	188.8	185.1	2348	65.08
12/4/2020 14:51	190.3	189.2	185.2	2350	64.96
12/4/2020 14:51	190.2	188.9	184.9	2350	65.04
12/4/2020 14:51	190.2	188.9	184.9	2352	65.04
12/4/2020 14:51	190.3	189.1	185.3	2349	64.92
12/4/2020 14:51	190.2	188.8	185.3	2348	65
12/4/2020 14:51	190.2	189.1	185.3	2351	64.99
12/4/2020 14:51	190.2	188.8	185.2	2351	64.99
12/4/2020 14:51	190.2	189.1	185	2349	65.12
12/4/2020 14:51	190.2	188.9	185.5	2346	64.92
12/4/2020 14:51	190.2	188.7	185.3	2347	64.96
12/4/2020 14:51	190.2	189.1	185.3	2348	65.05
12/4/2020 14:51	190.2	188.8	185.2	2347	64.92
12/4/2020 14:51	190.2	188.9	184.8	2347	65.04
12/4/2020 14:51	190.2	189.1	185.1	2351	65.05
12/4/2020 14:51	190.2	189.2	184.6	2350	65
12/4/2020 14:51	190.2	188.8	185.1	2351	64.83

12/4/2020 14:51	190.1	188.9	185.3	2352	65.08
12/4/2020 14:51	190.1	188.8	185.7	2355	65
12/4/2020 14:51	190.2	188.6	186.1	2355	65
12/4/2020 14:51	190.1	188.6	185.9	2353	65
12/4/2020 14:51	190.2	189.1	185.4	2357	65.12
12/4/2020 14:51	190.2	188.9	185	2355	64.83
12/4/2020 14:51	190.1	189.2	184.9	2351	64.99
12/4/2020 14:51	190.1	188.8	185.4	2353	65.13
12/4/2020 14:51	190.1	188.7	185.4	2350	64.84
12/4/2020 14:51	190.1	189.1	185.2	2349	64.92
12/4/2020 14:51	190.1	188.6	185.3	2355	65.16
12/4/2020 14:51	190.1	189.2	184.6	2355	64.88
12/4/2020 14:51	190.1	189.2	184.8	2352	65.08
12/4/2020 14:51	190.1	188.5	185.6	2354	65.12
12/4/2020 14:51	190.1	189.2	185.9	2355	65.08
12/4/2020 14:51	190.1	188.8	185.6	2355	65.04
12/4/2020 14:51	190.1	189.2	185	2350	65
12/4/2020 14:51	190.1	188.9	185.1	2355	64.96
12/4/2020 14:51	190.1	188.8	185.3	2354	64.99
12/4/2020 14:52	190.1	189.1	185.3	2356	64.92
12/4/2020 14:52	190.1	188.9	185.2	2355	64.96
12/4/2020 14:52	190.1	188.8	185.2	2356	64.96
12/4/2020 14:52	190.1	189.6	184.8	2355	65.04
12/4/2020 14:52	190.1	188.8	184.7	2357	65.04
12/4/2020 14:52	190	189.1	185.1	2358	64.96
12/4/2020 14:52	190.1	189	185.6	2358	65
12/4/2020 14:52	190.1	188.6	186.1	2357	65
12/4/2020 14:52	190.1	189.3	185.8	2354	65.04
12/4/2020 14:52	190	188.9	185.4	2351	64.92
12/4/2020 14:52	190	189.1	184.9	2351	64.92
12/4/2020 14:52	190	188.8	184.8	2352	65.04
12/4/2020 14:52	190	188.7	185.3	2354	64.88
12/4/2020 14:52	190	189.2	185.5	2354	64.92
12/4/2020 14:52	190	188.8	185.2	2355	65.08
12/4/2020 14:52	190	189.1	185.4	2353	65.04
12/4/2020 14:52	190.1	188.8	185.1	2357	65.08
12/4/2020 14:52	190.1	188.9	184.9	2358	64.93
12/4/2020 14:52	190	189.2	185.2	2355	64.88
12/4/2020 14:52	190.1	188.8	185.8	2353	64.88
12/4/2020 14:52	190	189.1	186	2355	65.16
12/4/2020 14:52	190.1	188.9	185	2355	64.99
12/4/2020 14:52	190.1	188.8	185.1	2353	64.88
12/4/2020 14:52	190	188.8	185.4	2355	65.12
12/4/2020 14:52	190.1	189.1	185.4	2357	65
12/4/2020 14:52	190	188.8	184.9	2357	65
12/4/2020 14:52	190.1	189.1	185	2358	64.92
12/4/2020 14:52	190	189.3	185.5	2362	65.08

12/4/2020 14:52	190	188.9	186	2362	64.96
12/4/2020 14:52	190	189.1	185.8	2358	65
12/4/2020 14:52	190.1	188.9	184.8	2360	65.09
12/4/2020 14:52	190	188.7	185.4	2357	64.96
12/4/2020 14:52	190	188.6	185.2	2358	65.04
12/4/2020 14:52	190.1	189.1	185.1	2359	65.09
12/4/2020 14:52	190	188.9	185.3	2358	64.92
12/4/2020 14:52	190.1	189.1	184.9	2357	64.96
12/4/2020 14:52	190.1	189.1	184.9	2353	64.99
12/4/2020 14:52	190.1	188.7	185.5	2355	64.96
12/4/2020 14:52	190.1	189.1	185.9	2358	65.04
12/4/2020 14:52	190.1	188.9	186.2	2361	64.88
12/4/2020 14:52	190.1	188.8	185.8	2359	65.08
12/4/2020 14:52	190.1	188.8	185.3	2357	65.08
12/4/2020 14:52	190	188.9	185.3	2357	65
12/4/2020 14:52	190.2	188.9	185.5	2359	65.01
12/4/2020 14:52	190.1	188.6	185.6	2361	64.96
12/4/2020 14:52	190.1	189.1	185.4	2360	65.04
12/4/2020 14:52	190.1	189.2	185.2	2361	65.12
12/4/2020 14:52	190.1	188.9	185.1	2359	64.99
12/4/2020 14:52	190.1	189	185.2	2358	65.04
12/4/2020 14:52	190.2	188.6	185.2	2359	65
12/4/2020 14:52	190.2	188.8	185.6	2358	65
12/4/2020 14:52	190.1	189.1	186	2355	64.88
12/4/2020 14:52	190.2	188.7	186.2	2356	65
12/4/2020 14:52	190.2	189.1	186	2357	65
12/4/2020 14:52	190.3	189.1	185.4	2358	64.96
12/4/2020 14:52	190.2	188.8	185.2	2362	64.92
12/4/2020 14:52	190.3	189.2	185.3	2362	65.04
12/4/2020 14:52	190.2	189.1	185	2359	65
12/4/2020 14:52	190.3	189.1	185.4	2358	65.04
12/4/2020 14:52	190.2	188.9	185.4	2361	65.01
12/4/2020 14:53	190.3	188.6	185.3	2359	64.96
12/4/2020 14:53	190.3	188.6	185.4	2359	65.01
12/4/2020 14:53	190.3	188.8	185.3	2362	65.12
12/4/2020 14:53	190.3	188.7	185.2	2358	64.96
12/4/2020 14:53	190.4	188.7	185.1	2359	64.99
12/4/2020 14:53	190.3	189.1	185.4	2359	64.96
12/4/2020 14:53	190.4	189.1	185.5	2358	65.03
12/4/2020 14:53	190.3	189.1	186.3	2358	64.96
12/4/2020 14:53	190.4	188.9	185.6	2358	65.01
12/4/2020 14:53	190.4	188.6	184.7	2359	64.96
12/4/2020 14:53	190.4	189.2	185.1	2355	64.99
12/4/2020 14:53	190.4	188.9	185.6	2356	65.12
12/4/2020 14:53	190.4	188.9	185.5	2352	64.92
12/4/2020 14:53	190.5	189	185.1	2354	65.12
12/4/2020 14:53	190.5	188.8	185.8	2358	65.08

12/4/2020 14:53	190.5	189.1	185.8	2362	64.96
12/4/2020 14:53	190.5	189.1	185.3	2364	65.04
12/4/2020 14:53	190.5	189.1	185.4	2358	65
12/4/2020 14:53	190.5	188.8	185.6	2358	65
12/4/2020 14:53	190.5	188.9	185.1	2362	64.92
12/4/2020 14:53	190.5	189.1	185.2	2359	65.04
12/4/2020 14:53	190.5	188.9	186.2	2362	65.09
12/4/2020 14:53	190.6	188.6	185.5	2359	64.92
12/4/2020 14:53	190.5	189.1	184.9	2356	65.04
12/4/2020 14:53	190.6	188.9	185.4	2361	65
12/4/2020 14:53	190.6	189	185.3	2358	64.92
12/4/2020 14:53	190.6	188.9	185.6	2358	64.99
12/4/2020 14:53	190.6	188.7	185.3	2358	64.96
12/4/2020 14:53	190.6	189.2	185.4	2356	65.01
12/4/2020 14:53	190.6	188.7	186.3	2355	64.92
12/4/2020 14:53	190.6	188.8	185.8	2356	64.99
12/4/2020 14:53	190.6	189	185.1	2358	65.01
12/4/2020 14:53	190.6	189.1	185.1	2357	64.95
12/4/2020 14:53	190.7	189.1	185.3	2359	64.92
12/4/2020 14:53	190.7	189	185.4	2362	64.96
12/4/2020 14:53	190.7	189.1	185.5	2360	65.08
12/4/2020 14:53	190.7	188.8	185.5	2358	64.83
12/4/2020 14:53	190.7	188.7	185.5	2358	64.92
12/4/2020 14:53	190.7	189.1	185.1	2359	64.99
12/4/2020 14:53	190.7	189	185.4	2360	64.99
12/4/2020 14:53	190.8	188.8	185.6	2358	64.92
12/4/2020 14:53	190.8	188.8	185.8	2357	65.16
12/4/2020 14:53	190.7	189	185.8	2357	65
12/4/2020 14:53	190.7	189.2	185.8	2360	65.04
12/4/2020 14:53	190.8	188.9	185.6	2362	65.08
12/4/2020 14:53	190.8	188.9	185.8	2361	65.04
12/4/2020 14:53	190.7	189.2	185.6	2359	64.96
12/4/2020 14:53	190.8	189.2	185.7	2362	65
12/4/2020 14:53	190.7	189	185.2	2361	64.92
12/4/2020 14:53	190.8	188.9	185.2	2364	65.04
12/4/2020 14:53	190.8	188.8	185.1	2364	65.05
12/4/2020 14:53	190.8	188.6	185.2	2362	64.96
12/4/2020 14:53	190.9	188.9	185.4	2357	64.92
12/4/2020 14:53	190.8	189.1	185.6	2358	64.96
12/4/2020 14:53	190.8	188.9	185.5	2358	65.04
12/4/2020 14:53	190.8	188.8	185.4	2359	64.92
12/4/2020 14:53	190.8	188.8	185.5	2358	65.08
12/4/2020 14:53	190.8	189.1	185.2	2360	65.12
12/4/2020 14:54	190.8	189.1	185.2	2359	64.88
12/4/2020 14:54	190.8	188.9	185.1	2357	64.88
12/4/2020 14:54	190.9	189.1	185.6	2362	65.04
12/4/2020 14:54	190.8	189.3	186.2	2360	65.08

12/4/2020 14:54	190.9	189.3	186.1	2359	65.03
12/4/2020 14:54	190.8	189.1	185.7	2361	64.88
12/4/2020 14:54	190.9	188.9	185.4	2360	65.04
12/4/2020 14:54	190.9	188.9	185.4	2359	64.96
12/4/2020 14:54	190.8	189.1	185.5	2363	65.12
12/4/2020 14:54	190.8	188.9	185.6	2364	64.95
12/4/2020 14:54	190.8	189	185.5	2364	65.08
12/4/2020 14:54	190.9	189	185.2	2368	65.08
12/4/2020 14:54	190.9	189.1	185.3	2371	64.92
12/4/2020 14:54	190.8	189	186.1	2367	64.88
12/4/2020 14:54	190.9	188.9	186.4	2366	64.92
12/4/2020 14:54	190.8	189.2	185.8	2365	64.96
12/4/2020 14:54	190.9	188.6	185.4	2366	64.92
12/4/2020 14:54	190.8	189	185.2	2363	64.96
12/4/2020 14:54	190.8	189.1	185.3	2364	65.04
12/4/2020 14:54	190.8	188.8	185.7	2364	64.91
12/4/2020 14:54	190.9	189.1	185.6	2365	64.96
12/4/2020 14:54	190.8	188.7	185.6	2364	65.04
12/4/2020 14:54	190.8	188.8	185.6	2369	64.96
12/4/2020 14:54	190.8	188.9	185.3	2368	65
12/4/2020 14:54	190.8	188.9	185.4	2365	65.04
12/4/2020 14:54	190.8	189.1	185.3	2361	64.96
12/4/2020 14:54	190.8	189	185.3	2361	64.96
12/4/2020 14:54	190.8	188.8	185.4	2364	64.99
12/4/2020 14:54	190.8	189.2	185.2	2365	65.09
12/4/2020 14:54	190.8	188.9	185.1	2362	64.95
12/4/2020 14:54	190.8	188.9	185.4	2359	65.04
12/4/2020 14:54	190.8	189	185.2	2364	65.08
12/4/2020 14:54	190.8	189.1	185.3	2365	64.92
12/4/2020 14:54	190.8	188.9	185.3	2368	64.92
12/4/2020 14:54	190.8	188.8	185.2	2370	64.99
12/4/2020 14:54	190.8	189	185.3	2365	65.08
12/4/2020 14:54	190.8	188.9	185.3	2366	64.99
12/4/2020 14:54	190.8	188.8	185.3	2364	65.04
12/4/2020 14:54	190.7	189.1	185.5	2365	64.92
12/4/2020 14:54	190.8	188.8	185.4	2368	64.84
12/4/2020 14:54	190.8	189.1	185.7	2368	64.92
12/4/2020 14:54	190.8	189.1	185.9	2368	65.01
12/4/2020 14:54	190.8	189.1	185.7	2368	65.08
12/4/2020 14:54	190.7	189	185.8	2368	65.04
12/4/2020 14:54	190.8	188.8	185.7	2369	64.96
12/4/2020 14:54	190.8	188.9	185.5	2367	64.92
12/4/2020 14:54	190.8	188.8	185.6	2365	64.96
12/4/2020 14:54	190.8	188.9	185.5	2365	65.12
12/4/2020 14:54	190.8	189.1	185.5	2367	65
12/4/2020 14:54	190.8	188.8	185.6	2369	65.08
12/4/2020 14:54	190.8	188.9	185.3	2367	65

12/4/2020 14:54	190.7	188.9	185.4	2363	65
12/4/2020 14:54	190.8	188.7	185.4	2364	64.96
12/4/2020 14:54	190.8	189.1	185.3	2365	64.96
12/4/2020 14:54	190.8	188.9	185.6	2369	65.09
12/4/2020 14:54	190.9	189.1	185.4	2370	64.91
12/4/2020 14:54	190.8	188.8	185.5	2369	65.04
12/4/2020 14:54	190.8	188.6	185.6	2373	65.17
12/4/2020 14:54	190.8	188.9	185.4	2369	64.96
12/4/2020 14:54	190.8	188.7	185.7	2367	64.96
12/4/2020 14:55	190.8	189.3	185.6	2364	65.08
12/4/2020 14:55	190.7	188.8	185.6	2365	65.08
12/4/2020 14:55	190.8	188.9	185.6	2361	64.84
12/4/2020 14:55	190.8	189.1	185.4	2361	65.04
12/4/2020 14:55	190.8	188.9	185.3	2363	64.88
12/4/2020 14:55	190.9	189.2	185.8	2363	64.99
12/4/2020 14:55	190.8	189.3	186.1	2366	64.92
12/4/2020 14:55	190.8	188.9	185.8	2368	64.96
12/4/2020 14:55	190.8	188.9	185.6	2365	64.88
12/4/2020 14:55	190.8	188.8	185.8	2368	64.92
12/4/2020 14:55	190.7	189.2	185.5	2369	65
12/4/2020 14:55	190.8	189.1	185.4	2368	64.99
12/4/2020 14:55	190.8	188.8	185.1	2366	65.08
12/4/2020 14:55	190.8	188.8	186.2	2368	64.99
12/4/2020 14:55	190.8	189	186.2	2365	65.04
12/4/2020 14:55	190.8	188.9	185.6	2363	64.96
12/4/2020 14:55	190.8	188.9	185.6	2363	65
12/4/2020 14:55	190.8	188.9	185.5	2362	65.05
12/4/2020 14:55	190.8	188.9	185.8	2362	64.96
12/4/2020 14:55	190.8	188.6	185.2	2361	65
12/4/2020 14:55	190.8	188.9	185.5	2361	65.04
12/4/2020 14:55	190.8	189	185.8	2362	65.04
12/4/2020 14:55	190.9	188.7	186.2	2364	64.92
12/4/2020 14:55	190.8	189.1	186.3	2363	65
12/4/2020 14:55	190.8	188.8	185.4	2361	64.92
12/4/2020 14:55	190.8	188.8	185.5	2362	65.08
12/4/2020 14:55	190.8	189	185.8	2361	64.92
12/4/2020 14:55	190.8	188.9	185.8	2362	65.09
12/4/2020 14:55	190.8	189.1	185.5	2361	64.88
12/4/2020 14:55	190.8	188.9	185.5	2359	64.92
12/4/2020 14:55	190.8	188.5	185.6	2358	64.96
12/4/2020 14:55	190.9	189.3	186.3	2355	65.04
12/4/2020 14:55	190.9	189.1	185.6	2357	64.88
12/4/2020 14:55	190.8	188.9	185.2	2356	64.92
12/4/2020 14:55	190.8	189.2	185.8	2355	65.03
12/4/2020 14:55	190.8	189.1	185.5	2358	64.99
12/4/2020 14:55	190.8	188.9	185.6	2359	64.92
12/4/2020 14:55	190.8	188.8	185.6	2358	65.04

12/4/2020 14:55	190.8	189	185.2	2358	64.92
12/4/2020 14:55	190.8	189	186	2353	65.04
12/4/2020 14:55	190.8	189.1	186.4	2351	65
12/4/2020 14:55	190.8	189.3	185.8	2347	64.96
12/4/2020 14:55	190.8	188.8	185.6	2345	64.96
12/4/2020 14:55	190.8	188.7	185.6	2345	65
12/4/2020 14:55	190.7	189.1	185.6	2346	64.96
12/4/2020 14:55	190.7	188.7	185.6	2346	65.04
12/4/2020 14:55	190.7	189	185.4	2347	65
12/4/2020 14:55	190.7	188.6	185.6	2347	64.96
12/4/2020 14:55	190.7	189	185.3	2346	65
12/4/2020 14:55	190.7	189	186.1	2351	65
12/4/2020 14:55	190.7	188.8	186.5	2352	64.96
12/4/2020 14:55	190.7	189.1	185.7	2354	64.92
12/4/2020 14:55	190.7	188.7	185.5	2352	64.92
12/4/2020 14:55	190.7	189	185.7	2348	65.01
12/4/2020 14:55	190.7	189	185.6	2351	64.99
12/4/2020 14:55	190.7	188.8	185.8	2348	65.12
12/4/2020 14:55	190.7	188.7	185.6	2350	65.01
12/4/2020 14:55	190.7	188.6	185.6	2348	65.04
12/4/2020 14:55	190.8	188.9	185.6	2346	65.12
12/4/2020 14:56	190.8	189.1	186.2	2346	65.03
12/4/2020 14:56	190.8	188.8	186.3	2349	65.03
12/4/2020 14:56	190.7	189	186.5	2348	65.04
12/4/2020 14:56	190.9	188.7	186.1	2351	64.92
12/4/2020 14:56	190.8	188.9	185.9	2354	65.04
12/4/2020 14:56	190.9	189.2	185.6	2350	64.99
12/4/2020 14:56	190.9	188.9	185.4	2352	65.04
12/4/2020 14:56	190.8	188.9	185.5	2352	65
12/4/2020 14:56	190.9	188.6	185.4	2355	64.95
12/4/2020 14:56	190.9	189	185.7	2351	64.96
12/4/2020 14:56	190.9	189.1	186	2350	65.08
12/4/2020 14:56	190.9	188.8	185.9	2353	64.96
12/4/2020 14:56	190.9	189	186.3	2350	65
12/4/2020 14:56	190.9	188.9	186.5	2348	65.04
12/4/2020 14:56	190.9	188.5	186	2347	64.92
12/4/2020 14:56	190.9	188.8	185.4	2347	65
12/4/2020 14:56	190.9	188.8	185.5	2348	64.96
12/4/2020 14:56	190.9	189	185.7	2350	64.92
12/4/2020 14:56	190.9	188.9	186.1	2348	64.92
12/4/2020 14:56	190.9	188.6	185.8	2345	64.96
12/4/2020 14:56	190.9	188.8	185.7	2352	65.04
12/4/2020 14:56	190.9	188.8	186.2	2354	64.96
12/4/2020 14:56	190.9	189.2	186.5	2357	65
12/4/2020 14:56	190.9	189.1	185.5	2358	65.01
12/4/2020 14:56	190.9	188.8	185.6	2358	65.04
12/4/2020 14:56	190.9	188.9	185.7	2358	64.92

12/4/2020 14:56	190.9	188.7	185.8	2358	64.96
12/4/2020 14:56	190.9	189.1	185.6	2358	64.84
12/4/2020 14:56	190.9	189.1	186.3	2352	64.8
12/4/2020 14:56	190.9	188.7	186.4	2353	65
12/4/2020 14:56	190.9	188.9	185.5	2357	65.08
12/4/2020 14:56	190.9	188.8	185.8	2357	64.88
12/4/2020 14:56	190.8	188.8	185.8	2357	65.04
12/4/2020 14:56	190.8	189.2	185.8	2358	64.92
12/4/2020 14:56	190.8	189	185.3	2358	64.92
12/4/2020 14:56	190.8	189	186.1	2354	65.08
12/4/2020 14:56	190.7	188.9	186.4	2358	65.12
12/4/2020 14:56	190.8	188.7	185.5	2359	64.88
12/4/2020 14:56	190.8	189.1	185.5	2362	65.04
12/4/2020 14:56	190.7	188.8	185.8	2362	65.12
12/4/2020 14:56	190.7	189.1	185.6	2362	64.88
12/4/2020 14:56	190.7	188.9	185.4	2361	65.04
12/4/2020 14:56	190.7	188.9	186.2	2366	65.08
12/4/2020 14:56	190.7	189.3	186.3	2364	64.92
12/4/2020 14:56	190.7	188.9	185.7	2367	65.04
12/4/2020 14:56	190.6	188.9	185.5	2365	64.96
12/4/2020 14:56	190.6	188.9	185.5	2366	64.96
12/4/2020 14:56	190.6	188.7	185.9	2363	64.87
12/4/2020 14:56	190.6	189.1	185.9	2364	64.92
12/4/2020 14:56	190.5	189.1	186	2368	65.04
12/4/2020 14:56	190.6	189.1	185.5	2368	64.92
12/4/2020 14:56	190.6	188.9	185.6	2365	65
12/4/2020 14:56	190.6	188.6	186.4	2366	64.96
12/4/2020 14:56	190.6	189.2	186.7	2361	64.96
12/4/2020 14:56	190.6	189.1	186.1	2365	64.92
12/4/2020 14:56	190.5	189.3	185.5	2369	65
12/4/2020 14:56	190.5	189.1	185.5	2369	64.99
12/4/2020 14:56	190.5	188.9	185.6	2367	64.92
12/4/2020 14:56	190.5	189.3	186	2366	64.96
12/4/2020 14:57	190.5	188.9	185.9	2371	65.08
12/4/2020 14:57	190.5	189.1	186.1	2361	64.95
12/4/2020 14:57	190.5	189.2	186.1	2359	64.99
12/4/2020 14:57	190.5	188.7	185.6	2362	65.08
12/4/2020 14:57	190.4	189.2	185.9	2361	65.03
12/4/2020 14:57	190.5	188.9	185.6	2362	65.04
12/4/2020 14:57	190.5	189.1	186	2365	65.08
12/4/2020 14:57	190.3	188.6	185.7	2363	65
12/4/2020 14:57	190.4	188.8	185.6	2362	64.99
12/4/2020 14:57	190.4	189	185.6	2366	64.92
12/4/2020 14:57	190.4	188.8	186.1	2368	64.92
12/4/2020 14:57	190.5	188.9	186.1	2366	65.03
12/4/2020 14:57	190.5	189.1	186	2365	65.04
12/4/2020 14:57	190.3	188.6	185.8	2368	64.96

12/4/2020 14:57	190.4	189.1	185.5	2367	64.84
12/4/2020 14:57	190.4	188.9	186.2	2366	64.99
12/4/2020 14:57	190.4	188.8	186.2	2369	64.96
12/4/2020 14:57	190.4	188.9	185.8	2370	65.03
12/4/2020 14:57	190.4	188.8	186	2371	65.08
12/4/2020 14:57	190.4	189.2	185.9	2371	64.96
12/4/2020 14:57	190.3	188.9	185.6	2372	64.96
12/4/2020 14:57	190.3	189.1	185.4	2369	65
12/4/2020 14:57	190.3	189.1	186.2	2368	64.83
12/4/2020 14:57	190.3	188.6	186.8	2371	65.05
12/4/2020 14:57	190.2	188.9	186.1	2369	64.87
12/4/2020 14:57	190.3	188.7	185.8	2366	65
12/4/2020 14:57	190.4	189.1	186	2372	65
12/4/2020 14:57	190.3	189.2	185.8	2369	64.96
12/4/2020 14:57	190.4	188.5	185.7	2369	64.92
12/4/2020 14:57	190.3	188.9	185.6	2370	64.92
12/4/2020 14:57	190.4	188.8	186.5	2370	64.92
12/4/2020 14:57	190.3	189.1	186.8	2373	64.92
12/4/2020 14:57	190.3	189.1	185.6	2368	64.96
12/4/2020 14:57	190.3	189.1	186.1	2373	65.04
12/4/2020 14:57	190.3	188.9	185.9	2369	64.88
12/4/2020 14:57	190.3	188.8	185.8	2370	64.96
12/4/2020 14:57	190.3	188.9	185.8	2371	65.08
12/4/2020 14:57	190.3	189.2	185.8	2373	65
12/4/2020 14:57	190.3	189.1	186.8	2373	65
12/4/2020 14:57	190.3	188.9	186	2373	64.92
12/4/2020 14:57	190.3	188.8	185.5	2372	65.04
12/4/2020 14:57	190.3	188.8	186	2368	64.88
12/4/2020 14:57	190.3	189.1	186.1	2368	64.92
12/4/2020 14:57	190.3	189	185.8	2368	65.12
12/4/2020 14:57	190.3	189.1	185.7	2365	64.99
12/4/2020 14:57	190.3	189.1	186.3	2365	65.04
12/4/2020 14:57	190.3	189	186.7	2367	64.89
12/4/2020 14:57	190.3	188.7	185.7	2365	64.92
12/4/2020 14:57	190.3	188.8	185.8	2364	65.03
12/4/2020 14:57	190.3	189.1	186	2363	64.88
12/4/2020 14:57	190.3	188.9	185.8	2362	65.08
12/4/2020 14:57	190.3	188.8	185.5	2359	64.92
12/4/2020 14:57	190.3	189.1	185.8	2359	65.04
12/4/2020 14:57	190.3	189.1	186.3	2364	65
12/4/2020 14:57	190.2	189.1	186.6	2366	64.92
12/4/2020 14:57	190.2	188.8	186.1	2367	65
12/4/2020 14:57	190.3	189.1	185.7	2369	65.05
12/4/2020 14:57	190.3	189.1	186	2369	65.04
12/4/2020 14:57	190.2	189.1	186	2366	65.04
12/4/2020 14:57	190.1	189.1	185.8	2368	65.13
12/4/2020 14:58	190.2	188.8	185.9	2365	64.96

12/4/2020 14:58	190.2	188.7	185.5	2362	65.04
12/4/2020 14:58	190.1	189	186	2361	65.12
12/4/2020 14:58	190.2	188.8	186.5	2367	65.04
12/4/2020 14:58	190.2	189.3	186.4	2365	64.99
12/4/2020 14:58	190.1	189.1	186.6	2366	64.95
12/4/2020 14:58	190.2	188.8	186.1	2368	64.96
12/4/2020 14:58	190.1	189.1	185.8	2368	64.88
12/4/2020 14:58	190.1	188.9	185.7	2365	64.88
12/4/2020 14:58	190.1	188.9	185.8	2367	65.08
12/4/2020 14:58	190.1	188.9	185.8	2365	64.96
12/4/2020 14:58	190.1	188.6	185.9	2365	64.96
12/4/2020 14:58	190.1	189.1	185.8	2370	65.01
12/4/2020 14:58	190.1	188.8	186	2368	64.88
12/4/2020 14:58	190.1	189.2	185.9	2368	64.92
12/4/2020 14:58	190.2	189	185.9	2366	65.04
12/4/2020 14:58	190.1	189.1	186.2	2367	64.96
12/4/2020 14:58	190.2	188.9	186.4	2365	65.08
12/4/2020 14:58	190.2	188.7	186.5	2366	64.96
12/4/2020 14:58	190.1	189.2	186.1	2366	65.04
12/4/2020 14:58	190.1	188.9	186.1	2364	64.92
12/4/2020 14:58	190.1	189	185.9	2365	64.96
12/4/2020 14:58	190.2	189.1	185.6	2364	64.96
12/4/2020 14:58	190.2	188.6	185.5	2360	64.91
12/4/2020 14:58	190.2	189.1	185.8	2358	65
12/4/2020 14:58	190.1	188.6	185.7	2363	65
12/4/2020 14:58	190.1	189.1	186	2364	64.87
12/4/2020 14:58	190.1	189.2	186.1	2363	65
12/4/2020 14:58	190.1	188.8	185.8	2365	64.96
12/4/2020 14:58	190.2	189	186.1	2364	64.96
12/4/2020 14:58	190.2	188.6	185.8	2363	64.96
12/4/2020 14:58	190.1	189.3	186.1	2362	64.92
12/4/2020 14:58	190.1	189	185.6	2360	64.96
12/4/2020 14:58	190.1	188.7	185.6	2359	65.04
12/4/2020 14:58	190.1	188.8	186.3	2358	64.99
12/4/2020 14:58	190.1	189.1	186.7	2360	65
12/4/2020 14:58	190.1	189.1	186.8	2359	64.88
12/4/2020 14:58	190.1	189	186.2	2361	64.96
12/4/2020 14:58	190.1	188.9	185.6	2361	65.04
12/4/2020 14:58	190.1	188.8	185.6	2359	64.88
12/4/2020 14:58	190	188.6	185.5	2358	65
12/4/2020 14:58	190	189.1	186	2358	65.03
12/4/2020 14:58	190	188.9	186.1	2356	65.04
12/4/2020 14:58	190	188.9	186	2354	64.92
12/4/2020 14:58	190	188.9	185.9	2353	64.84
12/4/2020 14:58	190	189.1	186	2355	64.88
12/4/2020 14:58	190	188.8	185.7	2350	64.83
12/4/2020 14:58	190	189.1	185.7	2351	65.04

12/4/2020 14:58	190	188.9	185.8	2353	64.88
12/4/2020 14:58	190	189.1	185.7	2354	64.92
12/4/2020 14:58	190	188.8	186.1	2353	64.88
12/4/2020 14:58	190	188.8	185.8	2353	64.92
12/4/2020 14:58	190	189	186	2355	65
12/4/2020 14:58	190	188.9	186.2	2356	65.04
12/4/2020 14:58	190	188.8	186	2354	65.08
12/4/2020 14:58	190	188.9	186.1	2355	65
12/4/2020 14:58	190	188.8	185.8	2357	64.96
12/4/2020 14:58	190	188.8	185.6	2357	64.92
12/4/2020 14:58	190	188.7	185.7	2360	64.92
12/4/2020 14:59	190	189.3	185.8	2359	64.92
12/4/2020 14:59	190	189.1	186	2358	64.92
12/4/2020 14:59	190	188.7	186	2355	65
12/4/2020 14:59	190	188.9	185.8	2355	64.96
12/4/2020 14:59	190	188.9	185.6	2353	64.96
12/4/2020 14:59	190	188.6	185.7	2354	65.04
12/4/2020 14:59	190	188.9	186.1	2355	64.92
12/4/2020 14:59	190	188.8	186.8	2355	65.08
12/4/2020 14:59	190	189.1	186.1	2358	65.08
12/4/2020 14:59	190	189.1	185.5	2359	64.92
12/4/2020 14:59	190	189.3	185.8	2361	64.92
12/4/2020 14:59	190	189	185.8	2361	64.96
12/4/2020 14:59	190	188.8	185.9	2359	65.08
12/4/2020 14:59	190	188.9	185.8	2357	64.99
12/4/2020 14:59	190	189.2	185.5	2359	64.95
12/4/2020 14:59	190	189.2	186.6	2362	65.12
12/4/2020 14:59	190	189.2	186.6	2362	64.99
12/4/2020 14:59	190	188.6	185.8	2359	64.92
12/4/2020 14:59	190	189	186	2360	65.04
12/4/2020 14:59	190.1	189.1	186	2355	64.99
12/4/2020 14:59	190.1	189.1	186	2357	65.04
12/4/2020 14:59	190	188.8	185.7	2357	64.96
12/4/2020 14:59	190	188.8	185.4	2356	65.08
12/4/2020 14:59	190	188.9	185.8	2358	64.99
12/4/2020 14:59	190	188.7	186.5	2359	64.8
12/4/2020 14:59	190	189.1	186.7	2361	65.12
12/4/2020 14:59	190	189	186	2361	64.95
12/4/2020 14:59	190	188.9	185.7	2362	64.88
12/4/2020 14:59	190	189.1	186.1	2368	65.04
12/4/2020 14:59	190	188.8	185.9	2366	64.88
12/4/2020 14:59	189.9	189.1	186	2368	65
12/4/2020 14:59	190	189.1	185.9	2365	65
12/4/2020 14:59	190	188.9	185.7	2367	65
12/4/2020 14:59	190	189.1	185.6	2365	64.92
12/4/2020 14:59	190	188.8	185.5	2365	64.92
12/4/2020 14:59	190	188.8	185.5	2362	65.04

12/4/2020 14:59	189.9	189.1	185.5	2365	64.96
12/4/2020 14:59	189.9	189	185.8	2365	65
12/4/2020 14:59	189.9	188.8	185.4	2365	65.04
12/4/2020 14:59	189.9	188.6	185.7	2368	64.88
12/4/2020 14:59	189.9	188.9	185.5	2368	65
12/4/2020 14:59	189.9	189.1	185.8	2363	65
12/4/2020 14:59	189.9	188.8	185.9	2363	64.88
12/4/2020 14:59	190	188.9	186	2362	64.96
12/4/2020 14:59	189.8	189	186	2363	65
12/4/2020 14:59	189.9	188.7	185.8	2363	65
12/4/2020 14:59	189.9	189.1	186.3	2360	64.99
12/4/2020 14:59	189.9	188.7	186.5	2359	64.92
12/4/2020 14:59	189.8	189.3	185.3	2361	64.96
12/4/2020 14:59	189.8	189	185.8	2359	65.04
12/4/2020 14:59	189.8	188.8	186.1	2357	64.88
12/4/2020 14:59	189.8	189.1	185.5	2360	65.05
12/4/2020 14:59	189.8	188.8	186.5	2366	64.99
12/4/2020 14:59	189.8	189.3	186.3	2364	65
12/4/2020 14:59	189.8	189.2	185.4	2363	65.12
12/4/2020 14:59	189.7	188.6	185.9	2361	64.92
12/4/2020 14:59	189.8	189.1	185.9	2360	64.96
12/4/2020 14:59	189.8	188.8	185.8	2358	65.05
12/4/2020 14:59	189.8	189.1	186.2	2361	64.92
12/4/2020 14:59	189.8	189.1	186.6	2360	64.96
12/4/2020 15:00	189.8	188.8	185.7	2359	64.92
12/4/2020 15:00	189.8	188.8	185.6	2362	65.09
12/4/2020 15:00	189.8	188.8	185.9	2360	64.96
12/4/2020 15:00	189.8	188.8	186	2360	64.96
12/4/2020 15:00	189.7	189.1	185.7	2358	65.09
12/4/2020 15:00	189.7	188.6	186.3	2359	64.96
12/4/2020 15:00	189.8	189.2	186.3	2362	65
12/4/2020 15:00	189.7	189	185.5	2363	64.96
12/4/2020 15:00	189.7	188.8	185.9	2365	65.04
12/4/2020 15:00	189.7	189.1	186.1	2362	64.96
12/4/2020 15:00	189.7	189	186	2361	65.04
12/4/2020 15:00	189.6	189.1	185.8	2362	64.96
12/4/2020 15:00	189.6	188.9	186.4	2360	65.03
12/4/2020 15:00	189.6	188.8	186.5	2358	65
12/4/2020 15:00	189.6	189.1	185.6	2359	65.08
12/4/2020 15:00	189.6	188.9	185.6	2359	64.84
12/4/2020 15:00	189.6	189.1	186.1	2363	65.08
12/4/2020 15:00	189.6	188.9	186.1	2365	64.93
12/4/2020 15:00	189.6	188.7	185.7	2362	64.99
12/4/2020 15:00	189.6	189.2	186	2365	64.88
12/4/2020 15:00	189.6	188.6	186.5	2370	65.08
12/4/2020 15:00	189.6	189.1	186	2367	64.92
12/4/2020 15:00	189.6	189	185.8	2365	64.88

12/4/2020 15:00	189.5	189.1	185.8	2367	64.92
12/4/2020 15:00	189.6	188.8	186.2	2368	65.08
12/4/2020 15:00	189.6	188.9	186.1	2362	65.08
12/4/2020 15:00	189.6	188.9	185.6	2359	65
12/4/2020 15:00	189.6	188.6	186.4	2357	65
12/4/2020 15:00	189.6	189.1	186.3	2359	64.96
12/4/2020 15:00	189.6	189.3	185.5	2359	64.92
12/4/2020 15:00	189.5	188.7	185.8	2361	65.04
12/4/2020 15:00	189.6	189.3	186	2364	64.87
12/4/2020 15:00	189.6	188.6	185.9	2364	65
12/4/2020 15:00	189.5	189	185.8	2367	65.04
12/4/2020 15:00	189.5	189.1	186.2	2366	64.92
12/4/2020 15:00	189.5	188.9	186.7	2360	64.96
12/4/2020 15:00	189.4	189.1	186.2	2366	65
12/4/2020 15:00	189.4	189.1	185.7	2368	65
12/4/2020 15:00	189.5	188.8	185.7	2364	64.96
12/4/2020 15:00	189.4	189.1	185.6	2364	64.96
12/4/2020 15:00	189.4	188.7	186	2368	65.08
12/4/2020 15:00	189.4	189.2	185.9	2363	64.99
12/4/2020 15:00	189.4	189.3	185.9	2363	65.08
12/4/2020 15:00	189.4	188.9	185.9	2360	64.96
12/4/2020 15:00	189.4	189.3	185.6	2360	64.83
12/4/2020 15:00	189.3	188.9	185.8	2360	65.12
12/4/2020 15:00	189.4	189.1	186.1	2363	64.96
12/4/2020 15:00	189.4	188.9	186.5	2359	64.99
12/4/2020 15:00	189.4	188.8	186.8	2361	65.03
12/4/2020 15:00	189.4	188.9	186.3	2361	65.08
12/4/2020 15:00	189.4	189.1	185.7	2363	65
12/4/2020 15:00	189.3	189.2	185.6	2362	65
12/4/2020 15:00	189.4	188.9	185.6	2362	65
12/4/2020 15:00	189.4	188.9	185.9	2362	64.92
12/4/2020 15:00	189.3	189.2	185.9	2362	64.88
12/4/2020 15:00	189.4	188.9	186.1	2362	64.96
12/4/2020 15:00	189.3	189.1	186	2361	65
12/4/2020 15:00	189.4	189	186	2359	64.92
12/4/2020 15:00	189.4	189.1	186.1	2358	64.92
12/4/2020 15:01	189.4	189.1	185.8	2359	65.04
12/4/2020 15:01	189.3	189.1	185.8	2359	64.96
12/4/2020 15:01	189.4	189.1	186	2358	64.96
12/4/2020 15:01	189.4	189	185.8	2358	65.04
12/4/2020 15:01	189.3	188.8	185.9	2362	65
12/4/2020 15:01	189.3	188.9	186.1	2359	64.91
12/4/2020 15:01	189.3	188.9	186.1	2359	64.92
12/4/2020 15:01	189.3	188.9	186.5	2358	64.96
12/4/2020 15:01	189.3	189	186.4	2360	64.91
12/4/2020 15:01	189.3	188.6	186.3	2361	64.92
12/4/2020 15:01	189.3	188.9	186.6	2362	65

12/4/2020 15:01	189.3	188.7	186.5	2362	64.92
12/4/2020 15:01	189.3	188.9	186.7	2362	64.92
12/4/2020 15:01	189.3	189	186.8	2360	64.96
12/4/2020 15:01	189.3	188.8	186.6	2358	64.99
12/4/2020 15:01	189.3	188.9	186.5	2358	64.96
12/4/2020 15:01	189.3	188.6	186.2	2357	64.92
12/4/2020 15:01	189.3	188.7	186.5	2358	64.92
12/4/2020 15:01	189.3	188.8	186.3	2359	64.92
12/4/2020 15:01	189.3	188.8	186.3	2358	64.83
12/4/2020 15:01	189.3	188.9	186.2	2362	65
12/4/2020 15:01	189.3	188.6	186	2365	64.92
12/4/2020 15:01	189.3	188.9	185.9	2363	64.92
12/4/2020 15:01	189.3	189.2	185.8	2364	64.92
12/4/2020 15:01	189.3	188.8	185.6	2363	64.88
12/4/2020 15:01	189.3	189.1	185.8	2360	65.04
12/4/2020 15:01	189.3	188.7	185.6	2364	65.04
12/4/2020 15:01	189.3	188.9	185.6	2363	64.99
12/4/2020 15:01	189.3	189.1	185.9	2362	64.99
12/4/2020 15:01	189.3	188.9	185.8	2362	64.92
12/4/2020 15:01	189.3	189	186	2364	65.08
12/4/2020 15:01	189.4	189	185.8	2365	64.83
12/4/2020 15:01	189.3	188.6	185.8	2365	65.04
12/4/2020 15:01	189.3	188.9	186	2366	64.96
12/4/2020 15:01	189.3	188.8	186	2365	64.96
12/4/2020 15:01	189.3	189.1	185.7	2363	64.92
12/4/2020 15:01	189.3	189.1	185.5	2365	65.08
12/4/2020 15:01	189.4	188.7	185.6	2363	64.84
12/4/2020 15:01	189.3	189	185.8	2361	64.88
12/4/2020 15:01	189.3	188.7	186.5	2362	64.92
12/4/2020 15:01	189.4	189.1	186.5	2366	65
12/4/2020 15:01	189.4	189.1	186	2366	64.99
12/4/2020 15:01	189.3	189.2	185.8	2366	64.88
12/4/2020 15:01	189.4	189	185.6	2368	64.96
12/4/2020 15:01	189.3	188.8	185.7	2363	65.04
12/4/2020 15:01	189.4	189.2	185.9	2362	64.88
12/4/2020 15:01	189.4	189	185.9	2364	65.08
12/4/2020 15:01	189.4	189.1	185.8	2368	64.88
12/4/2020 15:01	189.4	189.2	185.8	2365	64.92
12/4/2020 15:01	189.4	188.9	185.8	2360	64.96
12/4/2020 15:01	189.4	189	186	2360	64.96
12/4/2020 15:01	189.4	188.8	186	2359	64.99
12/4/2020 15:01	189.4	188.6	186.1	2358	64.96
12/4/2020 15:01	189.4	189.1	185.7	2357	65
12/4/2020 15:01	189.4	188.8	185.4	2355	64.96
12/4/2020 15:01	189.4	188.9	186	2357	64.92
12/4/2020 15:01	189.5	188.9	186.3	2364	65
12/4/2020 15:01	189.4	188.8	186.6	2362	64.96

12/4/2020 15:01	189.4	189.3	186.1	2364	65
12/4/2020 15:02	189.5	189.1	185.4	2362	65.04
12/4/2020 15:02	189.4	189.1	186	2361	64.99
12/4/2020 15:02	189.4	188.7	186.2	2364	65
12/4/2020 15:02	189.4	188.8	185.6	2365	65.04
12/4/2020 15:02	189.4	189.2	186	2362	65
12/4/2020 15:02	189.4	188.8	186.5	2365	65
12/4/2020 15:02	189.4	189.1	185.8	2367	64.88
12/4/2020 15:02	189.5	188.8	185.6	2370	65.04
12/4/2020 15:02	189.4	188.8	185.7	2369	64.83
12/4/2020 15:02	189.5	189.2	185.8	2369	64.88
12/4/2020 15:02	189.4	188.8	185.4	2370	64.93
12/4/2020 15:02	189.4	189.2	186.1	2372	65.03
12/4/2020 15:02	189.5	188.6	186.7	2370	65.08
12/4/2020 15:02	189.5	188.8	185.7	2368	65.12
12/4/2020 15:02	189.5	189.1	185.4	2365	64.99
12/4/2020 15:02	189.4	188.9	185.8	2364	64.92
12/4/2020 15:02	189.4	188.7	185.8	2365	64.92
12/4/2020 15:02	189.4	189.1	186	2366	64.92
12/4/2020 15:02	189.5	188.8	185.7	2365	64.88
12/4/2020 15:02	189.5	189.3	186.1	2364	65.04
12/4/2020 15:02	189.5	188.6	186.5	2367	65.12
12/4/2020 15:02	189.5	189.1	185.5	2364	64.83
12/4/2020 15:02	189.5	189.1	185.6	2366	65.12
12/4/2020 15:02	189.5	188.8	185.8	2368	65.08
12/4/2020 15:02	189.5	189.1	185.6	2366	65.04
12/4/2020 15:02	189.5	188.8	186.1	2369	65
12/4/2020 15:02	189.6	189	185.7	2369	64.96
12/4/2020 15:02	189.6	189	185.6	2370	65.05
12/4/2020 15:02	189.5	188.9	186.1	2370	64.96
12/4/2020 15:02	189.6	188.9	186.6	2368	64.92
12/4/2020 15:02	189.6	188.6	186.8	2368	65
12/4/2020 15:02	189.6	188.7	186.3	2369	64.96
12/4/2020 15:02	189.6	189.1	185.9	2366	65
12/4/2020 15:02	189.7	188.8	185.7	2364	65
12/4/2020 15:02	189.6	189.2	185.5	2361	64.92
12/4/2020 15:02	189.6	189.1	185.4	2360	65
12/4/2020 15:02	189.7	188.8	185.7	2362	65.09
12/4/2020 15:02	189.7	188.9	185.5	2368	64.96
12/4/2020 15:02	189.7	188.9	185.8	2369	65
12/4/2020 15:02	189.7	189.3	185.8	2368	65.04
12/4/2020 15:02	189.7	188.9	185.6	2369	64.99
12/4/2020 15:02	189.8	189.1	185.6	2372	65
12/4/2020 15:02	189.8	188.9	185.4	2374	64.96
12/4/2020 15:02	189.8	188.8	185.8	2376	64.96
12/4/2020 15:02	189.9	189.1	186.2	2366	64.88
12/4/2020 15:02	189.9	188.8	186.3	2369	64.99

12/4/2020 15:02	189.9	189	186.7	2373	64.96
12/4/2020 15:02	189.9	188.8	186.1	2375	64.88
12/4/2020 15:02	189.8	188.7	185.6	2369	64.92
12/4/2020 15:02	190	188.9	185.8	2367	65.08
12/4/2020 15:02	190	188.7	186	2366	64.96
12/4/2020 15:02	190	188.6	185.9	2361	64.99
12/4/2020 15:02	190	189.2	185.8	2362	64.96
12/4/2020 15:02	190	188.9	185.5	2366	64.99
12/4/2020 15:02	190	189	186.5	2368	64.92
12/4/2020 15:02	190	188.8	186.5	2367	64.93
12/4/2020 15:02	190.1	188.9	185.6	2373	64.92
12/4/2020 15:02	190	189.1	186	2373	64.84
12/4/2020 15:02	190.1	188.7	186	2372	64.92
12/4/2020 15:02	190.1	188.9	185.9	2374	65.01
12/4/2020 15:03	190.1	189.1	185.7	2375	64.99
12/4/2020 15:03	190.2	188.8	185.4	2373	64.96
12/4/2020 15:03	190.2	188.8	186.3	2379	65.12
12/4/2020 15:03	190.3	188.8	186.7	2376	65
12/4/2020 15:03	190.3	189.1	186	2376	64.96
12/4/2020 15:03	190.2	188.9	185.7	2377	65
12/4/2020 15:03	190.3	189.1	185.8	2376	64.92
12/4/2020 15:03	190.3	189	185.9	2373	64.96
12/4/2020 15:03	190.3	188.8	186.2	2373	65
12/4/2020 15:03	190.3	189.3	185.8	2369	64.96
12/4/2020 15:03	190.3	188.8	185.8	2370	64.83
12/4/2020 15:03	190.4	189.2	185.4	2374	64.96
12/4/2020 15:03	190.5	189.2	185.5	2373	65
12/4/2020 15:03	190.5	188.8	185.8	2367	64.96
12/4/2020 15:03	190.5	189	185.8	2367	64.92
12/4/2020 15:03	190.5	188.6	186.4	2365	65.04
12/4/2020 15:03	190.5	188.8	186.4	2368	64.91
12/4/2020 15:03	190.5	189.1	186.3	2368	64.92
12/4/2020 15:03	190.5	188.6	186.8	2371	65.04
12/4/2020 15:03	190.5	188.9	186.6	2372	65.04
12/4/2020 15:03	190.5	188.7	186.7	2373	64.92
12/4/2020 15:03	190.5	188.8	186.8	2371	65.04
12/4/2020 15:03	190.6	189.1	186.5	2370	64.92
12/4/2020 15:03	190.6	188.8	186.5	2365	64.99
12/4/2020 15:03	190.6	189.1	186.3	2363	64.84
12/4/2020 15:03	190.6	188.9	186.1	2368	64.92
12/4/2020 15:03	190.6	188.6	186.1	2369	64.99
12/4/2020 15:03	190.7	189.2	185.9	2371	65.08
12/4/2020 15:03	190.7	188.9	185.7	2374	65.08
12/4/2020 15:03	190.8	189.1	185.7	2373	65.08
12/4/2020 15:03	190.7	189.1	185.4	2373	64.84
12/4/2020 15:03	190.7	188.7	185.8	2373	64.92
12/4/2020 15:03	190.7	189.1	186.2	2373	64.88

12/4/2020 15:03	190.7	188.8	186.1	2370	64.99
12/4/2020 15:03	190.7	188.9	186.1	2372	64.88
12/4/2020 15:03	190.7	189.1	185.8	2369	65.12
12/4/2020 15:03	190.7	189.2	186.3	2370	64.99
12/4/2020 15:03	190.7	188.9	186.8	2373	64.92
12/4/2020 15:03	190.7	188.6	185.8	2373	65.04
12/4/2020 15:03	190.8	189.1	185.6	2376	65.04
12/4/2020 15:03	190.8	189	186.2	2372	64.99
12/4/2020 15:03	190.8	188.8	186.1	2372	65.03
12/4/2020 15:03	190.8	188.8	185.9	2373	65.04
12/4/2020 15:03	190.8	188.7	186	2368	64.99
12/4/2020 15:03	190.8	188.9	186.8	2370	64.88
12/4/2020 15:03	190.9	188.9	186.7	2373	64.96
12/4/2020 15:03	190.9	188.8	185.5	2373	65
12/4/2020 15:03	190.9	189.1	185.8	2371	65.04
12/4/2020 15:03	190.9	188.8	185.9	2373	65
12/4/2020 15:03	190.9	188.9	186	2376	64.92
12/4/2020 15:03	190.9	189.1	186.3	2379	64.92
12/4/2020 15:03	190.9	188.6	186.1	2380	64.96
12/4/2020 15:03	190.9	189.1	185.8	2377	64.92
12/4/2020 15:03	190.9	188.9	186	2374	65
12/4/2020 15:03	191	188.9	185.9	2373	65.04
12/4/2020 15:03	190.9	188.6	186.6	2373	64.96
12/4/2020 15:03	190.9	189.1	186.7	2374	64.96
12/4/2020 15:03	191	189.1	186.7	2378	65
12/4/2020 15:03	191	189	186.7	2376	65
12/4/2020 15:04	191	189.2	186.3	2376	64.91
12/4/2020 15:04	191	189.3	186	2374	64.96
12/4/2020 15:04	191	188.7	186	2380	65
12/4/2020 15:04	191	188.9	185.7	2377	64.95
12/4/2020 15:04	191	188.8	185.7	2378	64.92
12/4/2020 15:04	191	188.8	185.7	2380	65
12/4/2020 15:04	191	189.1	185.5	2380	64.88
12/4/2020 15:04	191	189.1	185.6	2380	65.12
12/4/2020 15:04	191.1	189	185.7	2378	65.04
12/4/2020 15:04	191.1	188.9	185.6	2375	65.04
12/4/2020 15:04	191.1	189.1	185.9	2372	65.03
12/4/2020 15:04	191.1	189.1	185.7	2376	65.08
12/4/2020 15:04	191.1	188.9	185.9	2374	65.04
12/4/2020 15:04	191.1	188.9	185.5	2370	64.88
12/4/2020 15:04	191.1	188.8	185.7	2370	65.04
12/4/2020 15:04	191.1	188.9	186	2373	65
12/4/2020 15:04	191.2	188.8	185.7	2373	64.95
12/4/2020 15:04	191.1	189.2	185.8	2375	64.92
12/4/2020 15:04	191.2	189.1	185.8	2372	64.92
12/4/2020 15:04	191.2	188.7	185.5	2371	64.88
12/4/2020 15:04	191.2	188.9	186	2372	64.92

12/4/2020 15:04	191.2	188.8	185.6	2372	64.99
12/4/2020 15:04	191.2	189	185.8	2377	64.96
12/4/2020 15:04	191.3	189.1	185.7	2378	64.88
12/4/2020 15:04	191.2	188.9	185.8	2379	64.92
12/4/2020 15:04	191.2	188.9	185.8	2380	65.08
12/4/2020 15:04	191.3	188.8	185.8	2382	64.83
12/4/2020 15:04	191.3	188.9	185.8	2377	64.92
12/4/2020 15:04	191.3	189.3	186.1	2382	65.04
12/4/2020 15:04	191.3	189	186.1	2378	65
12/4/2020 15:04	191.3	188.8	186.1	2384	65
12/4/2020 15:04	191.3	188.7	186.1	2384	65.04
12/4/2020 15:04	191.3	188.9	186.2	2387	65.04
12/4/2020 15:04	191.3	189.1	186.3	2384	64.92
12/4/2020 15:04	191.3	188.9	186.3	2381	64.96
12/4/2020 15:04	191.3	189	186.2	2382	64.99
12/4/2020 15:04	191.3	188.7	186.2	2377	64.96
12/4/2020 15:04	191.4	188.8	186.1	2379	64.96
12/4/2020 15:04	191.4	189.1	185.9	2378	64.96
12/4/2020 15:04	191.4	189.1	186.1	2378	64.92
12/4/2020 15:04	191.4	189.2	186.1	2380	65
12/4/2020 15:04	191.4	188.8	186.2	2380	65.04
12/4/2020 15:04	191.4	189	186.3	2381	64.96
12/4/2020 15:04	191.4	189.3	186.6	2379	64.92
12/4/2020 15:04	191.5	189.2	186.7	2382	65.04
12/4/2020 15:04	191.4	189.1	186.7	2384	65
12/4/2020 15:04	191.4	188.8	186.8	2385	64.87
12/4/2020 15:04	191.5	188.6	186.7	2382	64.96
12/4/2020 15:04	191.4	188.9	186.3	2384	64.96
12/4/2020 15:04	191.5	189	186.3	2382	64.92
12/4/2020 15:04	191.5	189	186.3	2384	64.99
12/4/2020 15:04	191.5	189.1	185.9	2386	65.08
12/4/2020 15:04	191.5	188.7	186	2383	64.99
12/4/2020 15:04	191.5	189.2	186.1	2382	64.96
12/4/2020 15:04	191.5	188.6	186.2	2386	65.08
12/4/2020 15:04	191.5	189.1	186.2	2385	64.88
12/4/2020 15:04	191.5	188.9	186.1	2384	64.95
12/4/2020 15:04	191.6	188.8	186.2	2388	64.96
12/4/2020 15:04	191.6	189.2	186.2	2391	65.08
12/4/2020 15:04	191.6	188.8	186.2	2388	65.04
12/4/2020 15:05	191.6	189.3	186	2386	65.12
12/4/2020 15:05	191.6	189	185.9	2389	64.92
12/4/2020 15:05	191.6	188.6	185.8	2384	64.88
12/4/2020 15:05	191.6	188.9	186.2	2387	64.99
12/4/2020 15:05	191.6	188.6	186.7	2387	65.08
12/4/2020 15:05	191.6	189.3	187.1	2384	64.95
12/4/2020 15:05	191.6	189.1	186.8	2380	64.88
12/4/2020 15:05	191.6	188.6	186.2	2386	65.12

12/4/2020 15:05	191.6	189.2	186.1	2384	65.08
12/4/2020 15:05	191.6	188.9	186	2385	65.04
12/4/2020 15:05	191.7	188.9	186.1	2385	65.04
12/4/2020 15:05	191.7	189	186.1	2387	64.96
12/4/2020 15:05	191.7	188.8	186.1	2390	64.88
12/4/2020 15:05	191.7	189.1	186.2	2391	64.96
12/4/2020 15:05	191.7	188.8	185.8	2393	64.92
12/4/2020 15:05	191.7	188.9	186	2386	64.91
12/4/2020 15:05	191.6	189.2	186.1	2387	64.96
12/4/2020 15:05	191.8	188.7	186.3	2386	65
12/4/2020 15:05	191.6	189.1	186.8	2386	64.87
12/4/2020 15:05	191.8	188.7	187	2386	64.96
12/4/2020 15:05	191.8	189.2	186.8	2384	64.96
12/4/2020 15:05	191.7	189.1	186.5	2384	64.87
12/4/2020 15:05	191.7	188.8	186.2	2387	64.96
12/4/2020 15:05	191.7	188.9	186.1	2385	65
12/4/2020 15:05	191.8	188.6	186.2	2388	64.96
12/4/2020 15:05	191.7	189.2	186.2	2390	65
12/4/2020 15:05	191.8	189.1	186.3	2386	65.04
12/4/2020 15:05	191.8	188.9	186.5	2385	65
12/4/2020 15:05	191.8	188.9	186.7	2387	64.92
12/4/2020 15:05	191.8	188.6	186.5	2385	65
12/4/2020 15:05	191.8	188.8	186.3	2386	64.96
12/4/2020 15:05	191.8	189.2	186.3	2381	64.92
12/4/2020 15:05	191.8	188.9	186.3	2377	65
12/4/2020 15:05	191.8	188.9	186.2	2380	64.96
12/4/2020 15:05	191.8	188.8	186.3	2380	64.92
12/4/2020 15:05	191.8	188.8	186.2	2384	64.95
12/4/2020 15:05	191.8	189.2	186.1	2387	65.04
12/4/2020 15:05	191.8	188.9	186.2	2387	65.08
12/4/2020 15:05	191.8	188.8	186	2384	64.88
12/4/2020 15:05	191.9	189.8	186	2383	65.08
12/4/2020 15:05	191.9	189.6	186.1	2390	64.96
12/4/2020 15:05	191.8	190.2	185.9	2389	65.04
12/4/2020 15:05	191.8	189.8	186.1	2386	64.99
12/4/2020 15:05	191.9	189.9	185.9	2393	64.96
12/4/2020 15:05	191.9	190.3	185.9	2396	64.92
12/4/2020 15:05	191.9	189.8	186.1	2395	65.04
12/4/2020 15:05	191.9	190	186.1	2391	65.05
12/4/2020 15:05	191.9	189.6	186.4	2386	65
12/4/2020 15:05	191.9	190.4	186.5	2384	65.04
12/4/2020 15:05	191.9	190	186.8	2387	64.96
12/4/2020 15:05	191.9	189.6	187	2388	64.96
12/4/2020 15:05	191.9	190.1	186.8	2390	64.96
12/4/2020 15:05	191.9	190	186.3	2386	64.96
12/4/2020 15:05	191.9	189.8	186.2	2384	65.09
12/4/2020 15:05	191.9	190.3	186.2	2386	64.87

12/4/2020 15:05	191.9	189.6	186.4	2386	65
12/4/2020 15:05	191.9	190	186.7	2386	65.09
12/4/2020 15:05	191.9	189.6	186.3	2381	64.96
12/4/2020 15:05	191.9	190.2	186.1	2381	64.96
12/4/2020 15:06	192	190.2	186	2384	65
12/4/2020 15:06	192	189.7	186.7	2383	64.88
12/4/2020 15:06	191.9	190.3	187	2388	65.12
12/4/2020 15:06	192	189.6	186.2	2384	65.08
12/4/2020 15:06	192	190.2	186.2	2384	64.96
12/4/2020 15:06	192	189.8	186.2	2385	64.88
12/4/2020 15:06	192	189.7	186.3	2386	64.96
12/4/2020 15:06	192	189.9	186.2	2389	65.08
12/4/2020 15:06	192	189.5	185.9	2387	64.88
12/4/2020 15:06	192.1	190.3	186.2	2384	65.08
12/4/2020 15:06	192	190.3	187	2385	65.05
12/4/2020 15:06	192.1	189.8	186.9	2384	64.96
12/4/2020 15:06	192.1	190.1	186.3	2380	64.96
12/4/2020 15:06	192	189.5	186.1	2379	65
12/4/2020 15:06	192	190.1	186.3	2377	65
12/4/2020 15:06	192	190.3	186.4	2375	64.96
12/4/2020 15:06	192	189.8	186.3	2377	64.96
12/4/2020 15:06	192.1	190	186	2377	65
12/4/2020 15:06	192.1	189.5	186.1	2378	64.96
12/4/2020 15:06	192.1	190.2	187	2381	65
12/4/2020 15:06	192.1	190.5	187.1	2380	64.96
12/4/2020 15:06	192.1	189.9	186.6	2379	64.92
12/4/2020 15:06	192	190.2	186.3	2380	64.92
12/4/2020 15:06	192.1	190.2	186.5	2382	64.96
12/4/2020 15:06	192.1	190	186.5	2382	65.04
12/4/2020 15:06	192.1	190.3	186.2	2384	65.08
12/4/2020 15:06	192.2	189.7	186.1	2386	64.92
12/4/2020 15:06	192.1	190	187	2385	64.96
12/4/2020 15:06	192.1	189.5	187	2384	65.04
12/4/2020 15:06	192.1	190.1	186.2	2386	65.04
12/4/2020 15:06	192.1	190.3	186.1	2382	64.96
12/4/2020 15:06	192.1	189.8	186.4	2381	65.04
12/4/2020 15:06	192.2	190.3	186.3	2375	64.96
12/4/2020 15:06	192.1	190	186.3	2379	65
12/4/2020 15:06	192.1	189.8	186.2	2378	64.96
12/4/2020 15:06	192.2	190.2	186.1	2372	64.92
12/4/2020 15:06	192.1	190.1	186.6	2374	65.05
12/4/2020 15:06	192.1	189.8	187.4	2376	65.04
12/4/2020 15:06	192.2	190.1	186.7	2374	64.96
12/4/2020 15:06	192.2	189.5	186.3	2374	64.96
12/4/2020 15:06	192.2	190.2	186.3	2378	64.96
12/4/2020 15:06	192.2	189.7	186.5	2375	64.92
12/4/2020 15:06	192.2	190	186.6	2376	65

12/4/2020 15:06	192.2	190.1	186.3	2379	65.01
12/4/2020 15:06	192.2	189.5	186.2	2380	65.04
12/4/2020 15:06	192.2	189.8	186	2376	65.04
12/4/2020 15:06	192.2	189.5	186.1	2378	65.01
12/4/2020 15:06	192.2	190.4	186.8	2376	64.88
12/4/2020 15:06	192.1	190.3	187.1	2373	64.92
12/4/2020 15:06	192.2	189.6	186.8	2372	65.01
12/4/2020 15:06	192.2	190.3	186.3	2373	64.96
12/4/2020 15:06	192.2	189.6	186.2	2375	64.93
12/4/2020 15:06	192.2	190.3	186.5	2377	65.01
12/4/2020 15:06	192.1	190.2	186.5	2374	64.92
12/4/2020 15:06	192.1	189.6	186.3	2373	65.12
12/4/2020 15:06	192.1	190.2	186.2	2369	64.92
12/4/2020 15:06	192.2	189.6	186	2374	65
12/4/2020 15:06	192.2	190	187.1	2373	64.96
12/4/2020 15:06	192.2	190.3	187	2371	64.92
12/4/2020 15:06	192.1	189.6	186.2	2376	65.04
12/4/2020 15:07	192.1	190	186.6	2373	64.96
12/4/2020 15:07	192.1	189.6	186.3	2372	65.04
12/4/2020 15:07	192.2	189.6	186.2	2374	64.96
12/4/2020 15:07	192.1	189.6	186.1	2377	65
12/4/2020 15:07	192.1	190.2	187	2378	65
12/4/2020 15:07	192.1	189.9	187.2	2383	64.96
12/4/2020 15:07	192.1	190.3	186.5	2384	64.96
12/4/2020 15:07	192.2	190.1	186.3	2385	64.88
12/4/2020 15:07	192.1	189.6	186.6	2388	64.92
12/4/2020 15:07	192.1	190.2	186.6	2384	64.99
12/4/2020 15:07	192	190.1	186.6	2382	64.92
12/4/2020 15:07	192	189.8	186.5	2388	65.01
12/4/2020 15:07	192.1	190.2	186.2	2377	64.84
12/4/2020 15:07	192.1	189.5	186.3	2371	64.88
12/4/2020 15:07	192	190.1	186.2	2369	65.12
12/4/2020 15:07	192	189.6	186.7	2373	64.92
12/4/2020 15:07	192	190	187.3	2372	65.12
12/4/2020 15:07	192	190.3	187.2	2373	65.04
12/4/2020 15:07	192	190.1	187.2	2380	65.08
12/4/2020 15:07	192	190.3	187	2382	64.99
12/4/2020 15:07	192	189.9	186.8	2380	64.99
12/4/2020 15:07	192	189.6	186.7	2383	65.08
12/4/2020 15:07	192	189.6	186.4	2380	65.04
12/4/2020 15:07	192	189.9	186.5	2380	64.88
12/4/2020 15:07	192	190.2	186.4	2381	65
12/4/2020 15:07	191.9	190.2	186.2	2383	64.87
12/4/2020 15:07	191.9	190.3	186.2	2381	64.96
12/4/2020 15:07	191.9	189.7	186.2	2379	65.04
12/4/2020 15:07	191.9	189.4	186.3	2384	65
12/4/2020 15:07	191.9	190.1	186.5	2381	64.96

12/4/2020 15:07	191.9	190.2	186.6	2380	64.96
12/4/2020 15:07	191.8	190.3	187.1	2384	65.04
12/4/2020 15:07	191.9	189.8	187.3	2380	64.92
12/4/2020 15:07	191.8	189.7	187.1	2382	64.92
12/4/2020 15:07	191.8	190.1	187.2	2382	65.05
12/4/2020 15:07	191.8	189.7	186.3	2379	64.99
12/4/2020 15:07	191.8	190.2	186.2	2380	64.96
12/4/2020 15:07	191.8	190.3	186.4	2382	64.96
12/4/2020 15:07	191.8	189.8	186.5	2383	64.96
12/4/2020 15:07	191.8	190.1	186.7	2384	64.92
12/4/2020 15:07	191.8	189.5	186.6	2383	64.84
12/4/2020 15:07	191.8	189.6	186.3	2387	65.08
12/4/2020 15:07	191.7	190.2	186.3	2391	64.88
12/4/2020 15:07	191.7	189.6	186.9	2390	64.84
12/4/2020 15:07	191.7	190.1	187	2389	64.96
12/4/2020 15:07	191.7	190	186.7	2384	64.84
12/4/2020 15:07	191.6	189.7	186.2	2376	65.04
12/4/2020 15:07	191.6	189.7	186.5	2382	64.96
12/4/2020 15:07	191.7	189.7	186.6	2379	65.04
12/4/2020 15:07	191.6	190.1	186.7	2380	64.99
12/4/2020 15:07	191.6	190.2	186.6	2379	65.12
12/4/2020 15:07	191.5	189.5	186.4	2381	64.92
12/4/2020 15:07	191.6	190.3	186.2	2379	64.99
12/4/2020 15:07	191.6	189.5	187.2	2382	65
12/4/2020 15:07	191.6	190	187.2	2385	65.05
12/4/2020 15:07	191.6	190	187	2386	64.96
12/4/2020 15:07	191.5	190.3	186.3	2382	64.96
12/4/2020 15:07	191.6	190.3	186.2	2384	65
12/4/2020 15:08	191.6	189.6	186.2	2384	64.96
12/4/2020 15:08	191.6	190.1	186.2	2381	64.92
12/4/2020 15:08	191.5	189.6	186.4	2384	65.05
12/4/2020 15:08	191.5	189.5	186.3	2384	65.04
12/4/2020 15:08	191.5	190.3	186.4	2384	64.96
12/4/2020 15:08	191.5	189.5	186.5	2386	65.04
12/4/2020 15:08	191.4	190	186.3	2387	65
12/4/2020 15:08	191.5	189.9	186.3	2383	64.88
12/4/2020 15:08	191.5	190.2	186.2	2382	64.92
12/4/2020 15:08	191.5	190.2	186.2	2382	65.12
12/4/2020 15:08	191.5	189.6	186.4	2382	65.04
12/4/2020 15:08	191.5	189.8	187.2	2374	64.99
12/4/2020 15:08	191.5	189.3	187.4	2378	65.12
12/4/2020 15:08	191.5	190.1	186.8	2379	65.08
12/4/2020 15:08	191.5	190.3	186.5	2376	65.08
12/4/2020 15:08	191.5	189.6	186.3	2381	65.12
12/4/2020 15:08	191.5	190.1	186.9	2384	64.92
12/4/2020 15:08	191.4	189.6	186.5	2386	64.92
12/4/2020 15:08	191.5	189.5	186.3	2385	65

12/4/2020 15:08	191.5	190.3	186.5	2383	64.96
12/4/2020 15:08	191.5	189.6	187.2	2375	64.92
12/4/2020 15:08	191.5	190.2	187.1	2375	64.96
12/4/2020 15:08	191.4	190.1	186.5	2382	65.05
12/4/2020 15:08	191.4	189.6	186.5	2385	65
12/4/2020 15:08	191.4	190.3	186.7	2382	65.04
12/4/2020 15:08	191.4	189.8	186.5	2382	65
12/4/2020 15:08	191.4	190.2	186.3	2384	64.92
12/4/2020 15:08	191.4	190.1	186.3	2383	64.96
12/4/2020 15:08	191.4	189.6	186.8	2384	64.99
12/4/2020 15:08	191.4	189.8	187.5	2387	65.05
12/4/2020 15:08	191.4	189.8	187	2388	64.91
12/4/2020 15:08	191.4	190	186.2	2386	64.92
12/4/2020 15:08	191.4	190.1	186.4	2384	65.08
12/4/2020 15:08	191.5	189.6	186.9	2377	65.04
12/4/2020 15:08	191.4	189.9	186.7	2376	65.08
12/4/2020 15:08	191.4	189.8	186.5	2378	64.93
12/4/2020 15:08	191.3	190.1	186.2	2379	65.04
12/4/2020 15:08	191.3	190.1	186.3	2379	65.04
12/4/2020 15:08	191.4	190.4	186.8	2377	64.88
12/4/2020 15:08	191.4	190.2	187.4	2377	65.08
12/4/2020 15:08	191.4	189.6	187.1	2374	64.8
12/4/2020 15:08	191.4	189.9	186.7	2380	64.92
12/4/2020 15:08	191.4	189.5	186.5	2382	64.93
12/4/2020 15:08	191.4	190.2	186.5	2382	64.88
12/4/2020 15:08	191.4	190	186.5	2384	65
12/4/2020 15:08	191.3	189.7	186.6	2384	64.96
12/4/2020 15:08	191.3	190.2	187.1	2382	64.92
12/4/2020 15:08	191.3	190.3	186.6	2382	65.04
12/4/2020 15:08	191.4	189.6	186.5	2380	64.88
12/4/2020 15:08	191.3	190.3	186.5	2377	65
12/4/2020 15:08	191.4	189.5	186.4	2381	64.96
12/4/2020 15:08	191.4	190.1	186.2	2384	65.04
12/4/2020 15:08	191.3	189.6	186.2	2382	65
12/4/2020 15:08	191.4	189.5	186.2	2382	64.92
12/4/2020 15:08	191.3	190.3	186.2	2378	64.96
12/4/2020 15:08	191.3	190	186.3	2382	65
12/4/2020 15:08	191.4	190.3	186.3	2384	64.96
12/4/2020 15:08	191.4	190.1	186.4	2385	65.04
12/4/2020 15:08	191.4	189.5	186.3	2390	65
12/4/2020 15:09	191.3	190.3	186.8	2389	64.92
12/4/2020 15:09	191.4	189.6	186.7	2389	65
12/4/2020 15:09	191.3	190.3	187.1	2389	64.96
12/4/2020 15:09	191.3	190.3	187.2	2388	64.96
12/4/2020 15:09	191.3	189.5	187.1	2386	64.92
12/4/2020 15:09	191.3	190.3	187.3	2385	64.99
12/4/2020 15:09	191.3	189.7	187.3	2390	64.92

12/4/2020 15:09	191.3	190.2	187.2	2388	64.88
12/4/2020 15:09	191.4	189.7	187.4	2386	64.96
12/4/2020 15:09	191.3	189.6	187.3	2390	64.96
12/4/2020 15:09	191.4	190.3	187.4	2387	65.12
12/4/2020 15:09	191.3	189.9	187.5	2388	65.08
12/4/2020 15:09	191.4	190.3	187.3	2387	65.01
12/4/2020 15:09	191.3	190	187.5	2382	65.03
12/4/2020 15:09	191.3	189.6	187.2	2381	65.03
12/4/2020 15:09	191.4	190.2	187.2	2381	64.96
12/4/2020 15:09	191.4	189.8	187.2	2385	65.08
12/4/2020 15:09	191.4	190.4	187.2	2385	64.96
12/4/2020 15:09	191.4	190	187.2	2391	65.12
12/4/2020 15:09	191.4	189.6	187	2391	64.96
12/4/2020 15:09	191.3	189.8	186.8	2390	65.04
12/4/2020 15:09	191.3	189.6	186.5	2384	65
12/4/2020 15:09	191.3	190	186.4	2384	64.96
12/4/2020 15:09	191.3	190.2	186.2	2382	64.92
12/4/2020 15:09	191.3	189.7	186.3	2386	64.96
12/4/2020 15:09	191.3	190.2	186.4	2387	65.04
12/4/2020 15:09	191.3	190	186.7	2385	64.92
12/4/2020 15:09	191.4	190.1	186.8	2382	64.96
12/4/2020 15:09	191.4	190.1	186.8	2380	65
12/4/2020 15:09	191.4	190.3	186.6	2380	64.92
12/4/2020 15:09	191.4	190	186.2	2383	64.96
12/4/2020 15:09	191.4	189.8	186.4	2384	64.96
12/4/2020 15:09	191.3	189.6	187.2	2386	65.04
12/4/2020 15:09	191.3	190.3	187.2	2389	65.04
12/4/2020 15:09	191.4	189.9	186.7	2387	65.04
12/4/2020 15:09	191.4	190.2	186.3	2386	65.08
12/4/2020 15:09	191.4	189.8	186.2	2386	64.84
12/4/2020 15:09	191.4	190.1	186.7	2386	64.96
12/4/2020 15:09	191.4	189.7	186.5	2388	65.05
12/4/2020 15:09	191.4	189.8	186.6	2386	64.88
12/4/2020 15:09	191.3	190.3	186.6	2389	65.04
12/4/2020 15:09	191.5	189.9	186.8	2388	65.12
12/4/2020 15:09	191.4	189.7	187.4	2385	64.88
12/4/2020 15:09	191.4	189.5	186.6	2385	64.92
12/4/2020 15:09	191.5	189.7	186.1	2388	64.96
12/4/2020 15:09	191.4	190.3	186.5	2390	65.04
12/4/2020 15:09	191.4	189.9	186.8	2391	65.04
12/4/2020 15:09	191.5	190.3	186.9	2388	64.92
12/4/2020 15:09	191.5	190.3	186.8	2393	64.96
12/4/2020 15:09	191.5	189.6	186.4	2393	65
12/4/2020 15:09	191.5	190.2	186.7	2390	65
12/4/2020 15:09	191.5	190	187.4	2392	65.05
12/4/2020 15:09	191.4	189.8	187.4	2393	65
12/4/2020 15:09	191.5	190	187.2	2391	64.96

12/4/2020 15:09	191.6	189.6	186.6	2391	65
12/4/2020 15:09	191.5	190.1	186.3	2389	64.88
12/4/2020 15:09	191.5	189.6	186.4	2391	65.04
12/4/2020 15:09	191.5	190.2	186.7	2393	65.12
12/4/2020 15:09	191.5	189.7	187	2391	64.92
12/4/2020 15:09	191.6	189.6	186.7	2388	64.88
12/4/2020 15:10	191.5	189.9	186.8	2386	64.88
12/4/2020 15:10	191.5	190.1	186.6	2388	64.88
12/4/2020 15:10	191.6	190.3	186.7	2389	64.84
12/4/2020 15:10	191.6	189.6	186.6	2386	64.97
12/4/2020 15:10	191.5	190.1	186.5	2389	64.88
12/4/2020 15:10	191.6	189.9	186.5	2388	64.92
12/4/2020 15:10	191.6	189.6	186.5	2393	65.08
12/4/2020 15:10	191.5	190.3	186.3	2394	64.88
12/4/2020 15:10	191.6	189.8	186.6	2394	65.04
12/4/2020 15:10	191.6	190.2	186.3	2398	65.08
12/4/2020 15:10	191.6	189.6	186.8	2400	65
12/4/2020 15:10	191.5	189.6	186.8	2399	64.96
12/4/2020 15:10	191.6	190.3	186.8	2399	65.04
12/4/2020 15:10	191.6	190.1	186.8	2397	64.99
12/4/2020 15:10	191.6	190	186.8	2394	65
12/4/2020 15:10	191.6	189.9	186.5	2394	65
12/4/2020 15:10	191.6	189.6	186.6	2394	65.09
12/4/2020 15:10	191.6	190.3	186.5	2395	64.96
12/4/2020 15:10	191.6	189.8	186.9	2395	65
12/4/2020 15:10	191.7	190.1	187.6	2397	65.04
12/4/2020 15:10	191.7	190	186.7	2393	64.92
12/4/2020 15:10	191.7	190.2	186.7	2393	65
12/4/2020 15:10	191.6	189.9	186.7	2397	65.08
12/4/2020 15:10	191.7	189.8	186.7	2394	64.92
12/4/2020 15:10	191.6	190	186.4	2391	65
12/4/2020 15:10	191.6	190.2	186.7	2391	65.12
12/4/2020 15:10	191.7	190.2	187.5	2393	64.92
12/4/2020 15:10	191.7	190.1	187.5	2394	65.08
12/4/2020 15:10	191.8	189.8	186.5	2395	64.92
12/4/2020 15:10	191.7	190.3	186.7	2397	64.92
12/4/2020 15:10	191.7	190.3	186.8	2398	64.92
12/4/2020 15:10	191.7	189.4	186.7	2401	65.04
12/4/2020 15:10	191.6	189.7	186.5	2401	64.96
12/4/2020 15:10	191.8	189.5	186.2	2399	64.88
12/4/2020 15:10	191.7	190.4	186.8	2402	65.04
12/4/2020 15:10	191.6	190.1	187.5	2399	65
12/4/2020 15:10	191.7	189.6	187.2	2398	64.92
12/4/2020 15:10	191.8	190.2	186.6	2394	64.96
12/4/2020 15:10	191.7	189.7	186.4	2397	65.04
12/4/2020 15:10	191.8	190.3	186.7	2394	65
12/4/2020 15:10	191.8	189.9	186.9	2391	65

12/4/2020 15:10	191.8	189.6	187	2394	65.04
12/4/2020 15:10	191.8	190.3	186.8	2395	64.96
12/4/2020 15:10	191.8	190	186.5	2394	65
12/4/2020 15:10	191.8	189.7	186.4	2400	65
12/4/2020 15:10	191.8	189.5	186.5	2402	64.96
12/4/2020 15:10	191.8	189.8	186.3	2402	64.96
12/4/2020 15:10	191.8	190.3	186.5	2399	65.04
12/4/2020 15:10	191.8	189.7	187	2402	65.04
12/4/2020 15:10	191.8	190.2	187.1	2399	64.99
12/4/2020 15:10	191.8	190	187.7	2402	65.04
12/4/2020 15:10	191.8	189.6	187.6	2401	65.01
12/4/2020 15:10	191.8	190.2	187	2401	64.92
12/4/2020 15:10	191.8	189.8	186.9	2398	64.92
12/4/2020 15:10	191.9	190.2	186.7	2399	65.08
12/4/2020 15:10	191.9	190	186.8	2400	64.99
12/4/2020 15:10	191.9	189.6	187	2401	64.92
12/4/2020 15:10	191.9	190	186.9	2404	64.92
12/4/2020 15:11	191.9	189.6	186.8	2402	64.92
12/4/2020 15:11	191.9	190.1	186.9	2403	65.04
12/4/2020 15:11	191.9	189.5	186.7	2404	65.08
12/4/2020 15:11	191.9	190.2	186.6	2401	64.92
12/4/2020 15:11	191.9	190.4	186.7	2401	65.04
12/4/2020 15:11	191.9	189.8	186.8	2398	65.05
12/4/2020 15:11	191.9	189.8	187.6	2402	65.04
12/4/2020 15:11	192	189.6	187.5	2395	65
12/4/2020 15:11	191.9	189.5	187.4	2396	65
12/4/2020 15:11	192	190.2	187.2	2397	65
12/4/2020 15:11	192	189.6	186.7	2398	64.92
12/4/2020 15:11	192	189.9	186.7	2396	65
12/4/2020 15:11	191.9	189.6	186.7	2395	65
12/4/2020 15:11	191.9	190.1	186.8	2397	65.04
12/4/2020 15:11	191.9	190.3	187.1	2398	64.96
12/4/2020 15:11	192	189.9	187	2401	65
12/4/2020 15:11	192	190.3	186.9	2398	64.99
12/4/2020 15:11	192	190.3	186.7	2397	64.83
12/4/2020 15:11	192.1	189.6	186.5	2395	65.08
12/4/2020 15:11	192	190.3	186.6	2400	64.92
12/4/2020 15:11	192	189.6	187.1	2402	64.92
12/4/2020 15:11	192	189.8	187.4	2399	65.12
12/4/2020 15:11	192	190.1	187.8	2397	64.84
12/4/2020 15:11	192	189.6	187.4	2398	65.04
12/4/2020 15:11	192	190.2	186.9	2398	65.04
12/4/2020 15:11	192.1	189.8	186.8	2400	65.08
12/4/2020 15:11	192.1	190	186.5	2397	64.99
12/4/2020 15:11	192.1	190.2	186.7	2391	65
12/4/2020 15:11	192	189.5	186.8	2393	65.08
12/4/2020 15:11	192.1	190.4	186.8	2388	64.99

12/4/2020 15:11	192.1	190.1	186.9	2387	64.96
12/4/2020 15:11	192	189.7	186.8	2388	65.04
12/4/2020 15:11	192.1	190.2	187	2387	65.03
12/4/2020 15:11	192.1	190.1	186.8	2385	65.04
12/4/2020 15:11	192.1	190.3	186.8	2387	65.08
12/4/2020 15:11	192	189.6	187	2390	65.04
12/4/2020 15:11	192	190	186.9	2394	65.04
12/4/2020 15:11	192.1	190.2	186.8	2393	65.12
12/4/2020 15:11	192.1	189.6	187	2394	64.96
12/4/2020 15:11	192.1	190	186.8	2394	64.92
12/4/2020 15:11	192.1	190	186.6	2395	65.04
12/4/2020 15:11	192.1	190.2	186.7	2391	64.96
12/4/2020 15:11	192.1	190.2	186.7	2397	65.04
12/4/2020 15:11	192.1	189.5	187.4	2394	65.04
12/4/2020 15:11	192.1	190.1	187.5	2395	64.87
12/4/2020 15:11	192	190.1	187.1	2397	64.96
12/4/2020 15:11	192	190	186.6	2397	65
12/4/2020 15:11	192.1	190.3	186.5	2399	65.04
12/4/2020 15:11	192.1	190.1	187	2397	64.92
12/4/2020 15:11	192.1	190.3	187	2393	64.88
12/4/2020 15:11	192.1	189.9	186.7	2394	64.88
12/4/2020 15:11	192.1	189.6	187.1	2392	64.96
12/4/2020 15:11	192.1	190.2	187.8	2393	65.04
12/4/2020 15:11	192.1	189.5	186.8	2390	65.04
12/4/2020 15:11	192.1	190	186.8	2391	64.99
12/4/2020 15:11	192.1	189.5	187	2389	64.92
12/4/2020 15:11	192.1	189.8	187	2385	65.08
12/4/2020 15:11	192.1	190.2	186.8	2390	64.88
12/4/2020 15:11	192.1	190.2	187.5	2387	64.83
12/4/2020 15:11	192.2	190.2	187.9	2387	64.92
12/4/2020 15:12	192.1	189.8	186.8	2386	64.96
12/4/2020 15:12	192.1	190.2	186.8	2384	64.92
12/4/2020 15:12	192.1	190.3	187	2389	64.92
12/4/2020 15:12	192.2	189.6	187.1	2390	64.99
12/4/2020 15:12	192.1	190.1	187.2	2391	65.03
12/4/2020 15:12	192.1	189.8	186.8	2393	64.92
12/4/2020 15:12	192.1	190.2	186.7	2392	65.08
12/4/2020 15:12	192.2	190.1	187.5	2390	64.88
12/4/2020 15:12	192.1	189.6	187.7	2391	64.84
12/4/2020 15:12	192.1	190.2	187.6	2390	64.96
12/4/2020 15:12	192.1	189.7	187	2393	64.95
12/4/2020 15:12	192.2	190.3	186.5	2390	65
12/4/2020 15:12	192.2	189.7	186.8	2391	64.92
12/4/2020 15:12	192.1	189.6	186.8	2390	64.99
12/4/2020 15:12	192.2	190.1	186.8	2388	65.04
12/4/2020 15:12	192.2	189.8	187	2387	65
12/4/2020 15:12	192.1	190	187	2386	65

12/4/2020 15:12	192.2	190.2	187	2387	64.88
12/4/2020 15:12	192.2	189.6	187.1	2391	64.96
12/4/2020 15:12	192.2	190.2	187.1	2391	65
12/4/2020 15:12	192.2	189.5	187	2391	65
12/4/2020 15:12	192.2	189.6	187	2395	64.96
12/4/2020 15:12	192.2	190.2	186.8	2393	65
12/4/2020 15:12	192.2	189.5	186.8	2394	64.92
12/4/2020 15:12	192.2	189.9	186.6	2391	64.92
12/4/2020 15:12	192.2	190	186.6	2393	65.04
12/4/2020 15:12	192.2	189.8	187	2394	65
12/4/2020 15:12	192.3	190.3	187.6	2391	64.92
12/4/2020 15:12	192.2	189.6	187.7	2391	65.04
12/4/2020 15:12	192.2	190.2	187	2393	64.92
12/4/2020 15:12	192.2	190.2	186.7	2388	65.04
12/4/2020 15:12	192.2	189.8	187	2389	65.05
12/4/2020 15:12	192.2	189.7	187	2391	65.04
12/4/2020 15:12	192.2	189.5	187	2391	64.84
12/4/2020 15:12	192.2	190.1	186.7	2388	65.04
12/4/2020 15:12	192.4	189.6	187.3	2388	64.92
12/4/2020 15:12	192.3	189.5	187.7	2390	64.92
12/4/2020 15:12	192.3	190	187.2	2391	64.99
12/4/2020 15:12	192.3	189.7	186.7	2388	65.08
12/4/2020 15:12	192.2	190.4	187	2384	64.83
12/4/2020 15:12	192.3	189.6	187	2386	64.84
12/4/2020 15:12	192.3	189.6	186.8	2391	64.93
12/4/2020 15:12	192.3	190.3	186.9	2390	64.8
12/4/2020 15:12	192.3	189.7	186.7	2388	65
12/4/2020 15:12	192.3	190	187	2386	65.04
12/4/2020 15:12	192.4	189.5	187.7	2385	64.88
12/4/2020 15:12	192.3	190.2	187.7	2384	64.99
12/4/2020 15:12	192.3	190.2	187.2	2389	64.92
12/4/2020 15:12	192.3	189.8	187	2385	64.92
12/4/2020 15:12	192.3	190.3	186.9	2384	64.99
12/4/2020 15:12	192.4	189.6	187	2384	64.88
12/4/2020 15:12	192.4	189.8	187	2391	64.93
12/4/2020 15:12	192.4	190.3	187	2390	64.99
12/4/2020 15:12	192.4	190	187	2391	64.92
12/4/2020 15:12	192.4	190	186.9	2393	65.04
12/4/2020 15:12	192.4	189.8	187	2393	64.96
12/4/2020 15:12	192.4	190.4	186.7	2389	64.88
12/4/2020 15:12	192.4	190.4	187.4	2391	65.12
12/4/2020 15:12	192.4	189.6	188	2391	64.88
12/4/2020 15:13	192.4	190.2	187.3	2389	64.92
12/4/2020 15:13	192.4	189.9	187.1	2394	64.87
12/4/2020 15:13	192.4	190.4	187	2394	65
12/4/2020 15:13	192.4	190	187.1	2390	65.05
12/4/2020 15:13	192.4	190.2	186.7	2385	64.96

12/4/2020 15:13	192.4	190.2	186.7	2382	64.96
12/4/2020 15:13	192.4	189.6	187.7	2383	65
12/4/2020 15:13	192.4	189.7	187.6	2382	64.96
12/4/2020 15:13	192.4	189.8	187	2385	64.96
12/4/2020 15:13	192.4	190.1	187.2	2391	64.96
12/4/2020 15:13	192.4	189.5	187.2	2396	65.08
12/4/2020 15:13	192.5	189.5	187.2	2391	64.92
12/4/2020 15:13	192.5	190.3	186.9	2394	64.96
12/4/2020 15:13	192.4	190.1	186.8	2391	65.08
12/4/2020 15:13	192.4	190.3	186.7	2387	64.88
12/4/2020 15:13	192.5	189.9	187.5	2388	64.88
12/4/2020 15:13	192.4	189.6	187.7	2391	64.96
12/4/2020 15:13	192.4	190.3	187.5	2390	64.96
12/4/2020 15:13	192.4	190	187	2387	64.99
12/4/2020 15:13	192.4	190.4	187.1	2388	65.12
12/4/2020 15:13	192.4	190.2	187.2	2391	64.88
12/4/2020 15:13	192.4	189.6	187	2390	64.88
12/4/2020 15:13	192.4	190.2	187.2	2396	64.97
12/4/2020 15:13	192.4	189.5	187	2390	64.88
12/4/2020 15:13	192.4	189.6	186.8	2391	65.04
12/4/2020 15:13	192.5	189.5	186.8	2394	64.96
12/4/2020 15:13	192.4	189.5	187.2	2394	64.83
12/4/2020 15:13	192.4	190	187.9	2394	64.92
12/4/2020 15:13	192.4	189.5	187.6	2395	64.92
12/4/2020 15:13	192.5	190	187	2392	64.96
12/4/2020 15:13	192.4	189.6	187	2389	64.96
12/4/2020 15:13	192.5	189.6	187.1	2388	64.92
12/4/2020 15:13	192.5	190.2	187.1	2391	64.92
12/4/2020 15:13	192.5	189.6	187.4	2395	64.91
12/4/2020 15:13	192.5	190.2	187	2395	64.96
12/4/2020 15:13	192.5	189.5	187	2399	64.96
12/4/2020 15:13	192.5	189.8	186.8	2398	64.88
12/4/2020 15:13	192.5	190.3	187.2	2396	65
12/4/2020 15:13	192.5	189.8	187.9	2395	65.09
12/4/2020 15:13	192.5	190	187.7	2393	64.96
12/4/2020 15:13	192.6	190.2	187.3	2391	65
12/4/2020 15:13	192.5	190.1	187	2391	65.09
12/4/2020 15:13	192.5	190.2	186.8	2391	65
12/4/2020 15:13	192.5	189.7	187	2389	64.96
12/4/2020 15:13	192.5	189.8	187	2394	64.96
12/4/2020 15:13	192.4	190.7	187.2	2397	64.96
12/4/2020 15:13	192.5	190.8	187	2391	64.99
12/4/2020 15:13	192.5	191.3	187	2388	64.92
12/4/2020 15:13	192.5	191	187	2389	64.99
12/4/2020 15:13	192.4	190.9	187	2390	65.08
12/4/2020 15:13	192.4	191.4	187	2391	65.04
12/4/2020 15:13	192.4	191	187.2	2388	64.96

12/4/2020 15:13	192.5	191.2	187.2	2388	64.83
12/4/2020 15:13	192.5	190.9	187.8	2391	64.88
12/4/2020 15:13	192.4	190.7	187.9	2391	65.08
12/4/2020 15:13	192.5	191.4	187.1	2390	64.8
12/4/2020 15:13	192.4	190.7	186.7	2390	65.08
12/4/2020 15:13	192.4	191.2	186.7	2391	64.96
12/4/2020 15:14	192.4	190.8	187.1	2390	64.92
12/4/2020 15:14	192.4	190.8	187.3	2388	65.04
12/4/2020 15:14	192.5	190.8	187.2	2384	64.96
12/4/2020 15:14	192.4	190.7	186.7	2387	65.04
12/4/2020 15:14	192.4	191.2	187.5	2388	64.99
12/4/2020 15:14	192.4	191	187.7	2388	65.01
12/4/2020 15:14	192.4	191.2	187	2389	65
12/4/2020 15:14	192.4	190.9	186.9	2393	64.96
12/4/2020 15:14	192.4	190.7	187	2391	64.92
12/4/2020 15:14	192.4	191.1	187.2	2390	64.96
12/4/2020 15:14	192.4	191.2	187.2	2388	64.87
12/4/2020 15:14	192.4	191.5	187	2389	64.92
12/4/2020 15:14	192.4	190.8	187	2395	65.04
12/4/2020 15:14	192.4	191	187.9	2393	64.87
12/4/2020 15:14	192.4	191.2	187.7	2391	64.92
12/4/2020 15:14	192.3	191.3	186.9	2390	65.13
12/4/2020 15:14	192.4	190.7	186.8	2389	64.92
12/4/2020 15:14	192.4	191.4	187.2	2390	64.96
12/4/2020 15:14	192.4	190.9	187.2	2390	65.09
12/4/2020 15:14	192.4	191.4	187	2388	64.92
12/4/2020 15:14	192.4	191.1	187.4	2389	65
12/4/2020 15:14	192.4	190.6	187.9	2392	65.12
12/4/2020 15:14	192.4	190.9	187.2	2392	64.88
12/4/2020 15:14	192.4	190.7	186.7	2394	64.96
12/4/2020 15:14	192.4	191	187	2398	65.04
12/4/2020 15:14	192.4	191.3	187.3	2398	64.92
12/4/2020 15:14	192.4	191.2	187.2	2398	64.99
12/4/2020 15:14	192.5	191.4	187	2393	64.88
12/4/2020 15:14	192.4	190.6	188	2390	65.08
12/4/2020 15:14	192.5	190.8	187.2	2385	64.88
12/4/2020 15:14	192.4	190.7	186.9	2386	64.96
12/4/2020 15:14	192.5	191.1	187	2389	65.16
12/4/2020 15:14	192.4	191.2	187	2387	64.99
12/4/2020 15:14	192.4	190.9	187	2388	65.04
12/4/2020 15:14	192.5	191.4	187.3	2388	65.08
12/4/2020 15:14	192.4	190.7	188	2383	64.92
12/4/2020 15:14	192.4	191	187	2381	64.92
12/4/2020 15:14	192.4	191.2	186.8	2384	64.96
12/4/2020 15:14	192.4	190.8	187.2	2377	64.99
12/4/2020 15:14	192.4	191.5	187	2375	65.08
12/4/2020 15:14	192.5	191.4	187	2376	65.04

12/4/2020 15:14	192.4	190.7	187.9	2379	65
12/4/2020 15:14	192.4	191.2	187.9	2385	64.96
12/4/2020 15:14	192.4	190.7	186.9	2388	64.92
12/4/2020 15:14	192.4	190.9	187	2392	65
12/4/2020 15:14	192.4	191.4	187.1	2396	65
12/4/2020 15:14	192.4	190.6	187.3	2391	65
12/4/2020 15:14	192.4	191.2	187.3	2386	65.04
12/4/2020 15:14	192.4	190.9	187.7	2390	64.87
12/4/2020 15:14	192.4	191.2	188.1	2387	64.96
12/4/2020 15:14	192.3	191.2	187.1	2385	65.05
12/4/2020 15:14	192.4	190.8	186.8	2387	64.96
12/4/2020 15:14	192.3	191	187.4	2385	64.92
12/4/2020 15:14	192.4	191	187.1	2384	65.01
12/4/2020 15:14	192.4	191.4	187.2	2381	64.96
12/4/2020 15:14	192.3	191.4	187	2380	65
12/4/2020 15:14	192.3	190.7	187.7	2382	64.92
12/4/2020 15:14	192.3	191.2	187.7	2378	64.92
12/4/2020 15:14	192.3	190.8	186.9	2376	65.04
12/4/2020 15:14	192.3	191	187.2	2372	64.99
12/4/2020 15:15	192.3	191.4	187.4	2373	65.08
12/4/2020 15:15	192.4	190.7	187.2	2373	65.04
12/4/2020 15:15	192.3	191.4	187	2376	64.99
12/4/2020 15:15	192.3	190.7	187.7	2382	65.08
12/4/2020 15:15	192.3	191.3	187.7	2386	65.04
12/4/2020 15:15	192.3	191.4	187	2385	64.92
12/4/2020 15:15	192.3	190.7	187.2	2384	65.12
12/4/2020 15:15	192.2	191.1	187.2	2381	64.92
12/4/2020 15:15	192.3	190.7	187.1	2384	65.08
12/4/2020 15:15	192.3	191.4	186.8	2387	65.04
12/4/2020 15:15	192.3	191.4	187.8	2386	65.04
12/4/2020 15:15	192.2	190.7	187.9	2389	64.99
12/4/2020 15:15	192.3	191.2	186.9	2390	65.08
12/4/2020 15:15	192.2	190.6	187	2389	64.99
12/4/2020 15:15	192.4	191.3	187.3	2384	64.99
12/4/2020 15:15	192.3	191.2	187.3	2383	65.01
12/4/2020 15:15	192.3	190.7	187.3	2379	64.83
12/4/2020 15:15	192.3	191.4	187.2	2385	65.04
12/4/2020 15:15	192.3	190.6	188.2	2386	65.12
12/4/2020 15:15	192.3	191.2	187.4	2386	64.96
12/4/2020 15:15	192.4	191.4	187	2387	64.96
12/4/2020 15:15	192.3	190.7	187.1	2384	64.92
12/4/2020 15:15	192.3	191	187.3	2384	65
12/4/2020 15:15	192.3	191.1	187.3	2384	64.96
12/4/2020 15:15	192.3	190.8	187.1	2384	65
12/4/2020 15:15	192.3	191.4	187.3	2384	65.04
12/4/2020 15:15	192.3	190.7	188.1	2384	64.96
12/4/2020 15:15	192.3	191.2	187.5	2386	64.96

12/4/2020 15:15	192.3	190.6	187	2384	65
12/4/2020 15:15	192.3	191.2	186.9	2380	65
12/4/2020 15:15	192.2	191.4	187.2	2376	64.96
12/4/2020 15:15	192.2	190.8	187.2	2376	65.08
12/4/2020 15:15	192.3	191.1	187.5	2380	65.04
12/4/2020 15:15	192.2	190.7	187.4	2382	64.96
12/4/2020 15:15	192.3	190.8	187.4	2386	65.12
12/4/2020 15:15	192.3	191.2	187.3	2385	64.95
12/4/2020 15:15	192.2	191	187.2	2384	64.96
12/4/2020 15:15	192.2	190.9	187.1	2386	65.12
12/4/2020 15:15	192.3	191.4	187.2	2383	64.84
12/4/2020 15:15	192.3	190.7	187.2	2382	64.92
12/4/2020 15:15	192.2	191.1	187.6	2384	64.92
12/4/2020 15:15	192.2	191	187.5	2385	64.95
12/4/2020 15:15	192.2	191.5	187.7	2386	65.12
12/4/2020 15:15	192.3	191.4	187.4	2386	65.04
12/4/2020 15:15	192.3	190.7	187.2	2382	65.04
12/4/2020 15:15	192.2	191.4	187.3	2384	65.08
12/4/2020 15:15	192.2	190.8	187	2380	64.88
12/4/2020 15:15	192.2	191	187.2	2381	64.96
12/4/2020 15:15	192.2	191.2	187.4	2383	64.88
12/4/2020 15:15	192.2	191	187.4	2382	64.92
12/4/2020 15:15	192.2	191.1	187.4	2381	64.88
12/4/2020 15:15	192.2	190.7	187	2380	64.99
12/4/2020 15:15	192.2	190.8	187	2384	64.96
12/4/2020 15:15	192.2	190.7	187.9	2386	64.92
12/4/2020 15:15	192.2	191.3	187.4	2382	64.99
12/4/2020 15:15	192.2	191.2	187	2382	64.88
12/4/2020 15:15	192.2	190.9	187.4	2378	64.96
12/4/2020 15:15	192.2	190.8	187.3	2376	64.87
12/4/2020 15:15	192.2	190.7	187	2377	65
12/4/2020 15:16	192.2	191	186.9	2377	65
12/4/2020 15:16	192.2	191.2	187.7	2380	64.91
12/4/2020 15:16	192.3	190.8	188	2385	65
12/4/2020 15:16	192.2	191.4	187	2385	64.96
12/4/2020 15:16	192.2	191.2	187.2	2382	64.92
12/4/2020 15:16	192.2	190.7	187.5	2377	64.96
12/4/2020 15:16	192.2	191.4	187.2	2380	65
12/4/2020 15:16	192.2	190.8	186.9	2379	64.96
12/4/2020 15:16	192.2	191.4	187	2380	64.96
12/4/2020 15:16	192.2	191.2	188	2386	65
12/4/2020 15:16	192.2	190.7	187.5	2382	65
12/4/2020 15:16	192.2	190.9	187	2382	65
12/4/2020 15:16	192.3	191	187.2	2379	64.96
12/4/2020 15:16	192.2	190.9	187.4	2382	64.96
12/4/2020 15:16	192.2	191.2	187.2	2382	64.96
12/4/2020 15:16	192.3	190.8	186.8	2384	65

12/4/2020 15:16	192.3	191.4	187.2	2391	65.04
12/4/2020 15:16	192.2	191.2	187.8	2386	64.88
12/4/2020 15:16	192.2	190.8	187.8	2388	64.88
12/4/2020 15:16	192.2	190.9	187	2390	65
12/4/2020 15:16	192.2	190.8	187.2	2386	65
12/4/2020 15:16	192.2	191.4	187.2	2386	65.08
12/4/2020 15:16	192.2	191.1	187.2	2387	65.12
12/4/2020 15:16	192.2	191.3	187	2386	64.95
12/4/2020 15:16	192.2	191.4	187	2386	64.84
12/4/2020 15:16	192.2	190.7	187	2386	65.08
12/4/2020 15:16	192.2	191.2	187.5	2385	65.08
12/4/2020 15:16	192.2	191.1	188.1	2385	64.96
12/4/2020 15:16	192.3	190.8	187.9	2386	64.95
12/4/2020 15:16	192.3	191	187.4	2387	65.08
12/4/2020 15:16	192.2	190.7	187.1	2384	64.99
12/4/2020 15:16	192.3	190.9	187	2388	65.08
12/4/2020 15:16	192.3	191.4	187.2	2391	65.08
12/4/2020 15:16	192.3	190.7	187	2387	65.03
12/4/2020 15:16	192.3	191	187.2	2389	64.99
12/4/2020 15:16	192.3	190.6	187.1	2395	65.12
12/4/2020 15:16	192.2	191	187.2	2394	64.84
12/4/2020 15:16	192.2	191.5	187.5	2392	64.99
12/4/2020 15:16	192.2	191.2	187.5	2391	65.04
12/4/2020 15:16	192.2	191	187.5	2386	65.04
12/4/2020 15:16	192.2	190.7	187.3	2386	65.04
12/4/2020 15:16	192.2	190.9	187.3	2385	65.04
12/4/2020 15:16	192.3	190.7	187.4	2386	64.91
12/4/2020 15:16	192.3	190.7	187.2	2389	65.08
12/4/2020 15:16	192.3	190.9	187.4	2387	65
12/4/2020 15:16	192.3	191	187.1	2386	64.88
12/4/2020 15:16	192.3	191	187	2384	64.96
12/4/2020 15:16	192.2	191.2	187.2	2391	64.96
12/4/2020 15:16	192.2	191.2	187.4	2394	65
12/4/2020 15:16	192.2	191.2	187.5	2390	64.96
12/4/2020 15:16	192.3	190.8	187.8	2385	64.92
12/4/2020 15:16	192.3	190.8	188.2	2387	65
12/4/2020 15:16	192.4	191.4	188.3	2380	64.87
12/4/2020 15:16	192.4	190.7	188.2	2384	64.96
12/4/2020 15:16	192.4	191.1	187.7	2385	65.05
12/4/2020 15:16	192.4	190.8	187.5	2381	64.88
12/4/2020 15:16	192.3	190.9	186.9	2381	64.88
12/4/2020 15:16	192.3	191.3	187	2382	65.04
12/4/2020 15:16	192.3	190.6	187.1	2384	64.92
12/4/2020 15:16	192.4	191.3	187.2	2390	65.04
12/4/2020 15:17	192.3	190.9	187.3	2394	64.88
12/4/2020 15:17	192.3	190.8	187.5	2393	64.96
12/4/2020 15:17	192.4	191.4	187.5	2391	64.92

12/4/2020 15:17	192.4	190.9	187.6	2391	64.88
12/4/2020 15:17	192.3	191.3	187.2	2392	64.92
12/4/2020 15:17	192.3	190.7	187.2	2387	64.99
12/4/2020 15:17	192.3	191.1	188	2388	64.88
12/4/2020 15:17	192.3	191.4	188.1	2390	65.05
12/4/2020 15:17	192.4	190.9	187.9	2386	64.99
12/4/2020 15:17	192.3	190.9	187.3	2390	65.08
12/4/2020 15:17	192.3	190.9	186.9	2389	65.08
12/4/2020 15:17	192.4	191.3	187.2	2386	64.8
12/4/2020 15:17	192.3	191.3	187.2	2384	64.88
12/4/2020 15:17	192.3	190.6	187.4	2391	65.08
12/4/2020 15:17	192.3	191.2	187.7	2391	64.99
12/4/2020 15:17	192.3	190.8	187.4	2391	64.92
12/4/2020 15:17	192.3	190.7	187.3	2391	65.03
12/4/2020 15:17	192.3	191.3	187.3	2393	64.88
12/4/2020 15:17	192.4	190.8	187.4	2397	65.04
12/4/2020 15:17	192.3	191.1	188.1	2392	64.99
12/4/2020 15:17	192.3	191.3	188.2	2391	65.04
12/4/2020 15:17	192.3	190.5	187	2390	64.95
12/4/2020 15:17	192.3	191.3	187.2	2390	64.99
12/4/2020 15:17	192.3	191.2	187.2	2391	64.99
12/4/2020 15:17	192.4	191.4	187.6	2398	65.04
12/4/2020 15:17	192.3	190.6	187.3	2395	64.92
12/4/2020 15:17	192.3	190.7	187.3	2393	65
12/4/2020 15:17	192.4	191.5	187.5	2390	64.96
12/4/2020 15:17	192.4	191	188.1	2387	65.08
12/4/2020 15:17	192.3	191.1	187.2	2389	65
12/4/2020 15:17	192.3	190.6	187.2	2389	64.96
12/4/2020 15:17	192.3	190.7	187.6	2390	65
12/4/2020 15:17	192.4	191.3	187.4	2390	65.04
12/4/2020 15:17	192.3	190.8	187.2	2391	65
12/4/2020 15:17	192.3	191.5	188	2390	65.04
12/4/2020 15:17	192.3	190.6	187.9	2390	65.09
12/4/2020 15:17	192.3	190.8	187	2388	64.96
12/4/2020 15:17	192.3	191.4	187.1	2390	64.92
12/4/2020 15:17	192.3	190.9	187.4	2394	65.05
12/4/2020 15:17	192.3	190.9	187.4	2395	64.88
12/4/2020 15:17	192.3	190.9	187.1	2395	64.88
12/4/2020 15:17	192.3	190.7	187.7	2394	65.12
12/4/2020 15:17	192.2	191.2	187.9	2393	64.99
12/4/2020 15:17	192.4	190.8	187	2393	64.92
12/4/2020 15:17	192.3	191.1	187.2	2397	65
12/4/2020 15:17	192.3	191.3	187.4	2391	65.04
12/4/2020 15:17	192.4	191.4	187.5	2393	64.92
12/4/2020 15:17	192.4	190.9	187.4	2396	65.08
12/4/2020 15:17	192.4	190.9	187.4	2397	64.93
12/4/2020 15:17	192.4	191.1	188	2395	64.99

12/4/2020 15:17	192.4	191.4	188.2	2390	64.84
12/4/2020 15:17	192.5	191.2	187.4	2395	64.96
12/4/2020 15:17	192.4	191.1	187	2395	65.04
12/4/2020 15:17	192.5	190.8	187.2	2394	65
12/4/2020 15:17	192.4	190.8	187.4	2399	64.96
12/4/2020 15:17	192.4	191.4	187.5	2397	65.04
12/4/2020 15:17	192.4	191.2	187.4	2393	65.04
12/4/2020 15:17	192.4	191.4	187.1	2395	65
12/4/2020 15:17	192.4	190.9	187.7	2399	65.04
12/4/2020 15:18	192.5	191	188.3	2395	64.92
12/4/2020 15:18	192.5	191.4	188	2395	65
12/4/2020 15:18	192.5	190.7	187	2392	64.87
12/4/2020 15:18	192.5	191.2	187	2394	64.96
12/4/2020 15:18	192.6	190.7	187.3	2395	65.05
12/4/2020 15:18	192.6	191.2	187.7	2395	65.04
12/4/2020 15:18	192.6	191.2	187.7	2393	65
12/4/2020 15:18	192.6	190.7	187.4	2394	64.92
12/4/2020 15:18	192.6	191.2	187.4	2398	64.99
12/4/2020 15:18	192.6	191.2	188.1	2398	64.99
12/4/2020 15:18	192.6	191.4	188.4	2401	64.92
12/4/2020 15:18	192.6	190.8	187.8	2404	65.08
12/4/2020 15:18	192.7	190.5	187.3	2397	64.84
12/4/2020 15:18	192.8	191	187.2	2395	64.92
12/4/2020 15:18	192.8	190.7	187.3	2394	65.16
12/4/2020 15:18	192.8	190.5	187.6	2394	64.88
12/4/2020 15:18	192.8	191.2	188.1	2396	65.08
12/4/2020 15:18	192.8	190.7	187.7	2398	64.93
12/4/2020 15:18	192.8	191.2	187.5	2400	64.88
12/4/2020 15:18	192.9	190.6	187.4	2395	64.96
12/4/2020 15:18	192.9	190.9	188.1	2397	65
12/4/2020 15:18	192.9	191.4	188.2	2396	64.92
12/4/2020 15:18	192.9	190.8	187.2	2396	64.88
12/4/2020 15:18	192.9	191.3	187.4	2397	65.17
12/4/2020 15:18	192.9	190.7	187.5	2399	64.96
12/4/2020 15:18	192.9	190.7	187.6	2397	65
12/4/2020 15:18	192.9	190.7	187.7	2402	65.04
12/4/2020 15:18	193	190.9	187.5	2400	64.96
12/4/2020 15:18	193	191.2	187.4	2398	65
12/4/2020 15:18	193	191.2	188.1	2398	65
12/4/2020 15:18	193	191.4	187.7	2395	65
12/4/2020 15:18	193.1	191.4	187.2	2393	64.92
12/4/2020 15:18	193	190.7	187.3	2394	64.88
12/4/2020 15:18	193	191.5	187.5	2396	65.08
12/4/2020 15:18	193.1	191.2	187.7	2397	64.92
12/4/2020 15:18	193.1	190.7	187.5	2397	64.92
12/4/2020 15:18	193.1	190.9	187.5	2402	64.96
12/4/2020 15:18	193.1	190.7	188.5	2399	64.99

12/4/2020 15:18	193.1	190.6	187.7	2399	65
12/4/2020 15:18	193.2	191.2	187.2	2398	65.16
12/4/2020 15:18	193.2	190.7	187.7	2394	64.99
12/4/2020 15:18	193.2	191.4	187.6	2397	64.92
12/4/2020 15:18	193.2	190.9	187.4	2396	64.92
12/4/2020 15:18	193.2	191.1	187.8	2398	65.04
12/4/2020 15:18	193.3	191.5	188.4	2395	65.08
12/4/2020 15:18	193.3	190.6	187.4	2395	65.08
12/4/2020 15:18	193.2	191.2	187.4	2394	64.84
12/4/2020 15:18	193.2	190.8	187.7	2392	65.08
12/4/2020 15:18	193.3	191.4	187.8	2392	65.04
12/4/2020 15:18	193.3	191.2	187.5	2394	64.96
12/4/2020 15:18	193.3	190.8	187.7	2397	64.83
12/4/2020 15:18	193.3	191.2	188.6	2402	64.92
12/4/2020 15:18	193.3	190.8	187.7	2398	65
12/4/2020 15:18	193.3	191.4	187.4	2398	64.92
12/4/2020 15:18	193.4	190.7	187.6	2402	65.04
12/4/2020 15:18	193.4	190.7	187.7	2406	64.96
12/4/2020 15:18	193.4	191.1	187.7	2406	64.92
12/4/2020 15:18	193.4	191	187.5	2407	64.96
12/4/2020 15:18	193.5	191.4	188.1	2407	65.08
12/4/2020 15:19	193.5	190.7	188.5	2404	65
12/4/2020 15:19	193.5	190.9	187.5	2402	64.92
12/4/2020 15:19	193.5	191	187.5	2406	64.96
12/4/2020 15:19	193.5	191	187.6	2405	65.04
12/4/2020 15:19	193.5	190.8	187.7	2405	65.04
12/4/2020 15:19	193.5	191.5	187.7	2403	65
12/4/2020 15:19	193.5	190.5	187.4	2405	64.96
12/4/2020 15:19	193.6	191.2	187.7	2403	64.99
12/4/2020 15:19	193.5	190.7	188.4	2402	64.96
12/4/2020 15:19	193.6	191.2	188.1	2405	65.05
12/4/2020 15:19	193.6	191.4	187.5	2406	64.88
12/4/2020 15:19	193.6	190.7	187.4	2405	64.88
12/4/2020 15:19	193.6	191.4	187.4	2406	65.05
12/4/2020 15:19	193.6	190.9	187.6	2405	64.84
12/4/2020 15:19	193.6	190.9	187.6	2404	64.92
12/4/2020 15:19	193.6	191.2	187.7	2407	65.01
12/4/2020 15:19	193.7	190.9	187.7	2407	64.96
12/4/2020 15:19	193.7	191.3	187.7	2407	65.08
12/4/2020 15:19	193.7	191	187.7	2408	65.08
12/4/2020 15:19	193.7	191.4	187.5	2410	64.96
12/4/2020 15:19	193.7	190.8	187.7	2405	64.88
12/4/2020 15:19	193.7	190.7	187.7	2406	64.88
12/4/2020 15:19	193.7	191	187.8	2405	64.96
12/4/2020 15:19	193.8	191	187.9	2401	64.92
12/4/2020 15:19	193.8	190.7	188	2402	64.99
12/4/2020 15:19	193.8	191.3	187.8	2401	64.96

12/4/2020 15:19	193.8	190.8	187.6	2403	64.99
12/4/2020 15:19	193.8	191	187.6	2401	64.96
12/4/2020 15:19	193.9	190.7	187.5	2402	64.92
12/4/2020 15:19	193.9	190.9	187.6	2402	64.92
12/4/2020 15:19	193.9	191.4	188.4	2402	64.96
12/4/2020 15:19	193.9	191.1	188.7	2401	65
12/4/2020 15:19	194	191	188.1	2402	64.92
12/4/2020 15:19	193.9	190.8	187.6	2402	64.96
12/4/2020 15:19	193.9	190.7	187.8	2404	65.04
12/4/2020 15:19	193.9	191.5	187.9	2404	64.92
12/4/2020 15:19	193.9	190.9	187.9	2406	65.04
12/4/2020 15:19	194	191.2	187.7	2403	64.92
12/4/2020 15:19	194.1	190.7	187.4	2403	64.99
12/4/2020 15:19	194	191.5	188.5	2405	65.04
12/4/2020 15:19	194	191.4	188.6	2402	65.04
12/4/2020 15:19	194.1	191	187.7	2402	64.99
12/4/2020 15:19	194	191.1	187.8	2410	65.12
12/4/2020 15:19	194.1	191.4	188.1	2412	64.93
12/4/2020 15:19	194	190.9	187.9	2408	64.99
12/4/2020 15:19	194.1	191.4	187.6	2408	65
12/4/2020 15:19	194	190.7	187.6	2412	65.08
12/4/2020 15:19	194.1	191.1	188.1	2409	64.88
12/4/2020 15:19	194.1	190.8	188.6	2405	65.08
12/4/2020 15:19	194.1	190.7	187.9	2406	65.08
12/4/2020 15:19	194.1	190.8	187.7	2407	64.99
12/4/2020 15:19	194.1	190.7	187.9	2412	64.88
12/4/2020 15:19	194.1	191	187.8	2409	64.88
12/4/2020 15:19	194.2	191	187.7	2412	65.04
12/4/2020 15:19	194.2	191.3	187.5	2409	64.87
12/4/2020 15:19	194.2	191.2	187.8	2408	64.92
12/4/2020 15:19	194.2	190.8	188.7	2412	65
12/4/2020 15:19	194.2	190.9	188.4	2409	64.91
12/4/2020 15:19	194.2	191.3	187.7	2409	65
12/4/2020 15:19	194.2	190.7	187.7	2411	65
12/4/2020 15:20	194.2	191.4	188.1	2408	64.96
12/4/2020 15:20	194.2	190.9	188	2410	64.92
12/4/2020 15:20	194.3	190.9	188.1	2408	65
12/4/2020 15:20	194.3	191.2	187.7	2409	64.92
12/4/2020 15:20	194.3	191	187.6	2413	65
12/4/2020 15:20	194.3	191.4	187.7	2413	65.04
12/4/2020 15:20	194.3	190.9	188.5	2411	65
12/4/2020 15:20	194.4	191.4	188.9	2407	64.92
12/4/2020 15:20	194.3	190.7	188.5	2410	65
12/4/2020 15:20	194.3	191	187.9	2408	64.96
12/4/2020 15:20	194.3	191.3	187.7	2411	64.96
12/4/2020 15:20	194.4	190.8	187.9	2411	64.96
12/4/2020 15:20	194.4	191.4	188.1	2409	65

12/4/2020 15:20	194.4	190.7	188.2	2407	64.96
12/4/2020 15:20	194.4	190.7	188.1	2411	64.88
12/4/2020 15:20	194.4	191.2	187.8	2410	64.97
12/4/2020 15:20	194.4	190.9	187.7	2409	64.84
12/4/2020 15:20	194.5	190.8	187.7	2410	64.92
12/4/2020 15:20	194.4	191.1	187.9	2413	64.96
12/4/2020 15:20	194.4	190.6	188.5	2413	64.88
12/4/2020 15:20	194.4	191.1	188.7	2407	64.92
12/4/2020 15:20	194.4	190.9	188.7	2411	64.96
12/4/2020 15:20	194.5	190.9	188.5	2410	64.92
12/4/2020 15:20	194.5	192.1	188.3	2411	65.04
12/4/2020 15:20	194.5	191.9	188	2411	65.04
12/4/2020 15:20	194.5	191.9	187.9	2412	65
12/4/2020 15:20	194.5	192.1	187.9	2411	65.04
12/4/2020 15:20	194.5	191.7	187.9	2408	65.04
12/4/2020 15:20	194.5	192.2	187.7	2409	65
12/4/2020 15:20	194.6	191.7	187.7	2404	65
12/4/2020 15:20	194.6	192.1	188.1	2405	65
12/4/2020 15:20	194.6	191.9	187.9	2409	64.96
12/4/2020 15:20	194.6	192	187.9	2410	64.91
12/4/2020 15:20	194.6	192.2	187.9	2415	65
12/4/2020 15:20	194.6	191.8	188	2420	65
12/4/2020 15:20	194.6	191.8	188	2415	64.96
12/4/2020 15:20	194.7	191.8	188	2411	64.96
12/4/2020 15:20	194.6	191.7	188.1	2413	65
12/4/2020 15:20	194.6	191.9	188.4	2414	64.96
12/4/2020 15:20	194.6	191.9	188.9	2416	65.01
12/4/2020 15:20	194.7	191.9	188.9	2416	65.04
12/4/2020 15:20	194.7	192.3	189	2415	64.99
12/4/2020 15:20	194.7	192.2	188.4	2416	65.04
12/4/2020 15:20	194.6	192.1	187.8	2416	65.04
12/4/2020 15:20	194.6	191.9	187.8	2415	64.92
12/4/2020 15:20	194.8	192	187.9	2416	64.92
12/4/2020 15:20	194.7	191.9	188.1	2413	64.88
12/4/2020 15:20	194.7	191.9	188.2	2415	64.96
12/4/2020 15:20	194.8	192.2	188.1	2413	64.84
12/4/2020 15:20	194.7	191.9	188.2	2411	64.88
12/4/2020 15:20	194.8	192.2	188	2415	64.96
12/4/2020 15:20	194.8	191.7	188.1	2418	64.99
12/4/2020 15:20	194.7	191.8	188.6	2414	65.04
12/4/2020 15:20	194.8	192.1	188.9	2413	65.16
12/4/2020 15:20	194.8	191.9	188.1	2411	64.91
12/4/2020 15:20	194.8	192	187.9	2406	64.88
12/4/2020 15:20	194.8	191.8	187.9	2408	65.01
12/4/2020 15:20	194.8	192.1	188.3	2409	65.04
12/4/2020 15:20	194.8	192.2	188.2	2408	65
12/4/2020 15:21	194.8	191.9	188	2411	65.04

12/4/2020 15:21	194.8	191.8	188.9	2414	64.96
12/4/2020 15:21	194.9	191.9	188.6	2414	64.96
12/4/2020 15:21	194.8	192.1	187.8	2413	65
12/4/2020 15:21	194.9	191.9	188.1	2414	64.87
12/4/2020 15:21	194.9	191.9	188.3	2409	64.96
12/4/2020 15:21	194.9	192.2	188.4	2409	65
12/4/2020 15:21	194.9	191.7	188	2410	64.96
12/4/2020 15:21	195	192	188.1	2412	64.96
12/4/2020 15:21	194.9	191.9	188.9	2411	65
12/4/2020 15:21	194.9	192	189	2410	64.88
12/4/2020 15:21	194.9	192.2	188.1	2410	64.92
12/4/2020 15:21	195	191.8	187.9	2415	65
12/4/2020 15:21	194.9	191.8	188.2	2416	64.99
12/4/2020 15:21	195	191.8	188.3	2415	64.96
12/4/2020 15:21	195	192.2	188.5	2416	65.08
12/4/2020 15:21	195	192.4	188.4	2416	64.96
12/4/2020 15:21	195	191.7	188.1	2416	65.04
12/4/2020 15:21	195	192.2	188.1	2415	65.03
12/4/2020 15:21	195	191.8	188.6	2418	64.93
12/4/2020 15:21	195	192.2	188.9	2416	65.03
12/4/2020 15:21	195	191.9	189.1	2420	64.99
12/4/2020 15:21	195.1	191.9	188.7	2419	64.96
12/4/2020 15:21	195	191.9	188.4	2417	64.99
12/4/2020 15:21	195.1	192.1	188.1	2420	64.99
12/4/2020 15:21	195	191.8	187.9	2421	65.09
12/4/2020 15:21	195.1	192.3	187.9	2416	64.83
12/4/2020 15:21	195.1	192.1	187.9	2417	65.04
12/4/2020 15:21	195	192.2	187.9	2417	65.04
12/4/2020 15:21	195	191.9	188.1	2417	64.88
12/4/2020 15:21	195.1	191.9	188.1	2415	64.92
12/4/2020 15:21	195.1	192.2	188.1	2419	65
12/4/2020 15:21	195.1	192	188.1	2419	64.96
12/4/2020 15:21	195.1	192.1	187.9	2415	64.91
12/4/2020 15:21	195.1	191.9	187.8	2422	65
12/4/2020 15:21	195.1	191.9	187.9	2422	65
12/4/2020 15:21	195.1	192.2	188.4	2419	64.96
12/4/2020 15:21	195.2	192	189.1	2416	65
12/4/2020 15:21	195.1	192.1	188.9	2416	64.96
12/4/2020 15:21	195.2	192.1	188.1	2420	65.04
12/4/2020 15:21	195.2	191.7	188.4	2422	65.04
12/4/2020 15:21	195.2	191.9	188.6	2423	64.92
12/4/2020 15:21	195.2	191.8	188.4	2421	65
12/4/2020 15:21	195.2	192	188.1	2419	65.08
12/4/2020 15:21	195.1	192.1	188.4	2417	64.99
12/4/2020 15:21	195.2	192.2	189.2	2421	65.03
12/4/2020 15:21	195.2	192	188.8	2418	64.92
12/4/2020 15:21	195.2	191.8	188.2	2420	65.04

12/4/2020 15:21	195.2	191.9	188.5	2420	64.83
12/4/2020 15:21	195.2	192.3	188.5	2417	65.12
12/4/2020 15:21	195.2	191.8	188.4	2422	65.16
12/4/2020 15:21	195.2	192.3	188.2	2420	65.08
12/4/2020 15:21	195.2	191.9	188.1	2422	64.92
12/4/2020 15:21	195.2	192.2	188.4	2423	65.08
12/4/2020 15:21	195.2	191.9	189.2	2422	64.88
12/4/2020 15:21	195.2	192.1	188.8	2424	65.08
12/4/2020 15:21	195.2	192.1	188.1	2423	65.04
12/4/2020 15:21	195.2	192	188.4	2423	64.92
12/4/2020 15:21	195.3	192.1	188.6	2420	64.88
12/4/2020 15:22	195.2	192	188.4	2419	64.96
12/4/2020 15:22	195.2	192.1	188.1	2418	64.96
12/4/2020 15:22	195.3	192.2	188.4	2414	65.04
12/4/2020 15:22	195.2	191.9	189.3	2419	64.96
12/4/2020 15:22	195.2	191.9	188.9	2416	65.04
12/4/2020 15:22	195.2	192.1	188.2	2419	65
12/4/2020 15:22	195.2	191.8	188.5	2415	64.96
12/4/2020 15:22	195.2	192.1	188.6	2416	65.09
12/4/2020 15:22	195.2	192.1	188.6	2417	64.83
12/4/2020 15:22	195.3	191.9	188.2	2420	65.04
12/4/2020 15:22	195.2	191.8	188.1	2421	65.08
12/4/2020 15:22	195.3	192.1	188.8	2419	64.99
12/4/2020 15:22	195.2	192.1	189.4	2419	65.12
12/4/2020 15:22	195.2	192.1	188.7	2423	65.12
12/4/2020 15:22	195.3	191.9	188.2	2419	64.99
12/4/2020 15:22	195.3	192.1	188.4	2416	65.08
12/4/2020 15:22	195.3	191.9	188.6	2416	65.12
12/4/2020 15:22	195.3	191.9	188.4	2420	64.96
12/4/2020 15:22	195.2	192	188.2	2416	64.88
12/4/2020 15:22	195.2	191.9	188.2	2418	65.08
12/4/2020 15:22	195.3	192.2	188.5	2415	64.96
12/4/2020 15:22	195.3	191.8	189.5	2415	65.04
12/4/2020 15:22	195.3	192	189.1	2415	64.92
12/4/2020 15:22	195.3	191.9	188.5	2418	65.08
12/4/2020 15:22	195.3	191.9	188.3	2417	65.04
12/4/2020 15:22	195.3	191.9	188.7	2413	64.92
12/4/2020 15:22	195.3	192	188.6	2414	65.17
12/4/2020 15:22	195.3	191.9	188.6	2418	64.92
12/4/2020 15:22	195.3	192.1	188.5	2419	65
12/4/2020 15:22	195.4	191.8	188.5	2412	65.04
12/4/2020 15:22	195.3	192.2	188.1	2417	64.92
12/4/2020 15:22	195.4	191.8	188.5	2416	65
12/4/2020 15:22	195.3	191.9	189.1	2417	65.05
12/4/2020 15:22	195.4	192.2	189.2	2414	65.04
12/4/2020 15:22	195.4	191.7	189.2	2414	65.04
12/4/2020 15:22	195.4	192	188.7	2412	65.08

12/4/2020 15:22	195.4	191.9	188.4	2414	65.04
12/4/2020 15:22	195.4	192.2	188.4	2415	64.96
12/4/2020 15:22	195.4	192.1	188.6	2415	65.04
12/4/2020 15:22	195.4	191.9	188.6	2415	64.93
12/4/2020 15:22	195.4	192.1	188.6	2413	64.92
12/4/2020 15:22	195.4	192.2	188.6	2416	65.08
12/4/2020 15:22	195.4	192.1	188.8	2417	65.08
12/4/2020 15:22	195.5	191.8	188.8	2418	64.96
12/4/2020 15:22	195.4	191.9	188.8	2416	65
12/4/2020 15:22	195.4	192.2	188.6	2415	65.04
12/4/2020 15:22	195.5	192	188.7	2417	64.96
12/4/2020 15:22	195.5	192.1	188.7	2419	65.04
12/4/2020 15:22	195.5	192.1	188.6	2422	65.04
12/4/2020 15:22	195.5	191.7	188.6	2421	65.04
12/4/2020 15:22	195.4	191.9	188.5	2413	64.96
12/4/2020 15:22	195.5	191.9	188.5	2409	65
12/4/2020 15:22	195.4	192.1	188.4	2409	65.05
12/4/2020 15:22	195.4	192.1	188.5	2416	64.92
12/4/2020 15:22	195.5	191.9	188.9	2418	65.04
12/4/2020 15:22	195.4	192.1	189.3	2418	65.08
12/4/2020 15:22	195.4	191.9	189.4	2416	64.92
12/4/2020 15:22	195.5	192.1	189	2416	64.92
12/4/2020 15:22	195.5	192.2	188.5	2422	65.08
12/4/2020 15:23	195.5	191.7	188.2	2419	65.03
12/4/2020 15:23	195.5	192.1	188.3	2415	64.99
12/4/2020 15:23	195.5	192	188.6	2416	65.21
12/4/2020 15:23	195.5	192	188.8	2416	65.08
12/4/2020 15:23	195.5	192.1	188.6	2416	65.12
12/4/2020 15:23	195.6	191.7	188.6	2415	65.04
12/4/2020 15:23	195.5	192	189.5	2415	64.96
12/4/2020 15:23	195.6	192	189.2	2412	65
12/4/2020 15:23	195.5	192.2	188.4	2416	64.96
12/4/2020 15:23	195.5	192.1	188.6	2410	65.04
12/4/2020 15:23	195.6	191.8	188.1	2413	64.92
12/4/2020 15:23	195.6	192.2	188.6	2411	64.96
12/4/2020 15:23	195.6	191.8	188.7	2411	65
12/4/2020 15:23	195.5	192.1	189.5	2406	64.96
12/4/2020 15:23	195.6	192.2	188.9	2408	65
12/4/2020 15:23	195.6	192.1	188.4	2408	65.12
12/4/2020 15:23	195.7	192.2	188.6	2409	64.92
12/4/2020 15:23	195.6	191.7	188.8	2409	64.93
12/4/2020 15:23	195.6	192.2	188.9	2411	65.01
12/4/2020 15:23	195.6	192.2	188.7	2406	64.84
12/4/2020 15:23	195.6	191.8	188.6	2407	65.01
12/4/2020 15:23	195.6	192	188.8	2408	64.97
12/4/2020 15:23	195.6	191.7	189.3	2406	64.92
12/4/2020 15:23	195.6	191.9	189.5	2407	65.04

12/4/2020 15:23	195.6	192.2	189.6	2407	64.96
12/4/2020 15:23	195.6	192.1	189.3	2408	64.99
12/4/2020 15:23	195.7	192.1	188.9	2406	65.04
12/4/2020 15:23	195.6	192	188.7	2410	64.92
12/4/2020 15:23	195.6	191.9	188.6	2411	65
12/4/2020 15:23	195.7	192.2	188.4	2408	64.91
12/4/2020 15:23	195.6	191.8	188.4	2405	64.96
12/4/2020 15:23	195.7	192.2	188.3	2404	65.04
12/4/2020 15:23	195.7	192.1	188.5	2402	64.96
12/4/2020 15:23	195.7	192	188.5	2404	64.92
12/4/2020 15:23	195.7	192.1	188.4	2405	65.05
12/4/2020 15:23	195.6	192.1	188.4	2405	65.04
12/4/2020 15:23	195.7	193.2	188.5	2406	64.92
12/4/2020 15:23	195.7	193.1	189	2408	64.96
12/4/2020 15:23	195.7	193.1	189.3	2404	65.04
12/4/2020 15:23	195.7	193.1	189.6	2405	65.04
12/4/2020 15:23	195.7	193	189.5	2410	64.93
12/4/2020 15:23	195.6	193	188.8	2408	64.99
12/4/2020 15:23	195.7	192.9	188.8	2408	65.08
12/4/2020 15:23	195.7	193.1	188.8	2408	65.08
12/4/2020 15:23	195.7	193	188.8	2405	64.84
12/4/2020 15:23	195.6	193.1	188.5	2410	65.08
12/4/2020 15:23	195.7	193.1	188.8	2411	64.92
12/4/2020 15:23	195.7	193.1	189.6	2410	65.03
12/4/2020 15:23	195.7	193.1	188.9	2410	64.92
12/4/2020 15:23	195.7	192.9	188.8	2412	65.08
12/4/2020 15:23	195.6	193.2	189.1	2409	64.99
12/4/2020 15:23	195.6	193	189	2409	65.08
12/4/2020 15:23	195.6	192.8	188.7	2406	64.96
12/4/2020 15:23	195.7	193.2	188.5	2409	64.91
12/4/2020 15:23	195.7	193.1	188.6	2408	65.04
12/4/2020 15:23	195.6	193.1	189.6	2411	65.04
12/4/2020 15:23	195.7	193	189.5	2417	65
12/4/2020 15:23	195.6	193	188.7	2418	65
12/4/2020 15:23	195.6	193.1	188.8	2416	64.96
12/4/2020 15:24	195.7	192.9	188.9	2420	65
12/4/2020 15:24	195.6	192.9	188.9	2420	64.96
12/4/2020 15:24	195.6	193.3	188.7	2420	64.92
12/4/2020 15:24	195.7	193.2	188.6	2419	65.04
12/4/2020 15:24	195.6	193.3	189.2	2417	64.87
12/4/2020 15:24	195.6	192.8	189.5	2417	65
12/4/2020 15:24	195.6	193.1	189.2	2416	64.92
12/4/2020 15:24	195.5	193.1	188.6	2417	65.04
12/4/2020 15:24	195.5	193	188.8	2418	64.92
12/4/2020 15:24	195.6	193.1	188.9	2422	65.08
12/4/2020 15:24	195.6	193	189	2420	64.84
12/4/2020 15:24	195.6	193.1	188.8	2416	65.04

12/4/2020 15:24	195.5	193.2	188.6	2420	64.96
12/4/2020 15:24	195.5	193.1	189	2419	65.04
12/4/2020 15:24	195.4	193.1	189.8	2416	64.96
12/4/2020 15:24	195.5	192.9	189.7	2410	65.08
12/4/2020 15:24	195.5	193.1	188.9	2407	64.93
12/4/2020 15:24	195.5	193.1	188.9	2406	64.99
12/4/2020 15:24	195.4	192.9	189.1	2409	64.92
12/4/2020 15:24	195.4	193.1	189.1	2411	65.08
12/4/2020 15:24	195.4	192.9	189.1	2409	65.04
12/4/2020 15:24	195.4	193.1	188.9	2410	64.84
12/4/2020 15:24	195.4	193.2	188.8	2412	64.96
12/4/2020 15:24	195.4	193	188.6	2415	64.96
12/4/2020 15:24	195.4	193.1	188.8	2414	65
12/4/2020 15:24	195.4	193.1	189.3	2418	65.09
12/4/2020 15:24	195.4	193.1	189.8	2411	64.96
12/4/2020 15:24	195.4	193.1	190	2411	64.96
12/4/2020 15:24	195.4	193.1	189.7	2412	65
12/4/2020 15:24	195.4	193.2	189.5	2411	64.96
12/4/2020 15:24	195.4	192.9	189.3	2416	65
12/4/2020 15:24	195.4	192.9	189.2	2418	64.96
12/4/2020 15:24	195.4	193.1	189.1	2416	65.04
12/4/2020 15:24	195.4	193	189.1	2416	65
12/4/2020 15:24	195.4	193.1	189.1	2415	65
12/4/2020 15:24	195.4	193.1	189.1	2416	64.96
12/4/2020 15:24	195.4	193	189	2412	64.99
12/4/2020 15:24	195.4	193.1	189.2	2412	65.08
12/4/2020 15:24	195.4	193.1	189.2	2413	64.96
12/4/2020 15:24	195.4	193.2	189.3	2412	64.88
12/4/2020 15:24	195.4	193.1	189.5	2416	64.92
12/4/2020 15:24	195.4	192.9	189.8	2418	65.12
12/4/2020 15:24	195.4	193.1	189.9	2419	64.92
12/4/2020 15:24	195.4	193	189.7	2415	65.08
12/4/2020 15:24	195.4	193.3	189.3	2416	64.96
12/4/2020 15:24	195.4	193.1	188.8	2416	65.08
12/4/2020 15:24	195.4	193.1	188.6	2411	64.88
12/4/2020 15:24	195.4	192.9	188.8	2412	65.12
12/4/2020 15:24	195.4	193	189.1	2415	65.08
12/4/2020 15:24	195.4	193.1	188.9	2417	64.92
12/4/2020 15:24	195.4	193.3	188.9	2420	65.04
12/4/2020 15:24	195.4	193.1	189	2418	65
12/4/2020 15:24	195.4	192.9	189.6	2416	64.96
12/4/2020 15:24	195.4	193.1	189.5	2416	65
12/4/2020 15:24	195.4	193.1	188.5	2417	65.09
12/4/2020 15:24	195.4	193.1	188.6	2411	64.87
12/4/2020 15:24	195.4	193	189	2412	64.96
12/4/2020 15:24	195.4	193.1	189.2	2416	65.04
12/4/2020 15:24	195.4	193.1	189.1	2416	64.92

12/4/2020 15:24	195.4	193.1	188.9	2415	65
12/4/2020 15:25	195.4	192.9	189.3	2420	65
12/4/2020 15:25	195.4	193	189.8	2420	65.04
12/4/2020 15:25	195.4	193	189.8	2419	64.99
12/4/2020 15:25	195.4	193	189.3	2418	65.04
12/4/2020 15:25	195.4	193.1	188.8	2419	65.04
12/4/2020 15:25	195.4	193.2	188.8	2419	65.04
12/4/2020 15:25	195.4	193.1	188.8	2411	64.99
12/4/2020 15:25	195.4	193.1	189	2415	65
12/4/2020 15:25	195.4	192.9	189.1	2419	64.92
12/4/2020 15:25	195.4	193.3	189.3	2420	64.96
12/4/2020 15:25	195.4	193.1	189.3	2424	65.08
12/4/2020 15:25	195.4	192.9	189.3	2423	64.99
12/4/2020 15:25	195.4	193.3	189.2	2420	64.88
12/4/2020 15:25	195.4	193.1	189.3	2423	65.01
12/4/2020 15:25	195.4	193.1	189.1	2422	64.99
12/4/2020 15:25	195.4	193.3	188.9	2424	64.99
12/4/2020 15:25	195.4	193	189	2424	65.08
12/4/2020 15:25	195.4	193.2	189.1	2421	65.04
12/4/2020 15:25	195.4	193	189.2	2415	65.08
12/4/2020 15:25	195.4	193.1	189.5	2417	64.96
12/4/2020 15:25	195.5	193.1	189.6	2416	65.04
12/4/2020 15:25	195.4	192.9	189.8	2419	64.91
12/4/2020 15:25	195.5	193.1	190	2422	64.96
12/4/2020 15:25	195.4	192.9	190	2422	65
12/4/2020 15:25	195.4	193.2	189.9	2425	64.95
12/4/2020 15:25	195.4	193.1	189.9	2424	65
12/4/2020 15:25	195.4	192.9	189.8	2423	65
12/4/2020 15:25	195.4	192.9	189.5	2423	64.96
12/4/2020 15:25	195.4	193.1	189.3	2423	64.96
12/4/2020 15:25	195.4	193.1	189.1	2423	65.04
12/4/2020 15:25	195.4	193.2	188.8	2422	64.96
12/4/2020 15:25	195.4	193.3	188.8	2419	64.92
12/4/2020 15:25	195.4	193.2	188.8	2418	65
12/4/2020 15:25	195.4	193.1	189.1	2420	65
12/4/2020 15:25	195.4	193.1	188.9	2420	65.04
12/4/2020 15:25	195.5	193.3	189.1	2425	64.96
12/4/2020 15:25	195.5	193.1	189.2	2422	65
12/4/2020 15:25	195.5	193.2	189.3	2423	65.04
12/4/2020 15:25	195.5	193.1	189.2	2423	64.92
12/4/2020 15:25	195.4	192.9	189.4	2423	64.96
12/4/2020 15:25	195.5	193.1	189.2	2422	64.83
12/4/2020 15:25	195.5	193.3	189.2	2420	64.88
12/4/2020 15:25	195.5	193.1	189.1	2422	64.97
12/4/2020 15:25	195.4	192.9	189.3	2422	64.84
12/4/2020 15:25	195.5	193.1	189.6	2420	64.96
12/4/2020 15:25	195.5	193.1	190.1	2424	64.96

12/4/2020 15:25	195.4	193.1	189.5	2422	64.88
12/4/2020 15:25	195.5	193.3	188.8	2420	65.04
12/4/2020 15:25	195.4	193.1	188.8	2418	65.01
12/4/2020 15:25	195.5	193	189.1	2415	65.04
12/4/2020 15:25	195.5	192.9	189.2	2415	64.88
12/4/2020 15:25	195.5	193.2	189.4	2426	65.04
12/4/2020 15:25	195.5	193.1	189.2	2422	64.99
12/4/2020 15:25	195.5	193.1	189.9	2423	65.04
12/4/2020 15:25	195.5	192.9	189.6	2426	64.92
12/4/2020 15:25	195.5	193.1	188.9	2427	64.92
12/4/2020 15:25	195.5	193	189.3	2428	64.92
12/4/2020 15:25	195.6	193	189.4	2429	65
12/4/2020 15:25	195.6	193.1	189.3	2427	64.91
12/4/2020 15:26	195.5	193	189.4	2428	65.04
12/4/2020 15:26	195.5	193.1	190.2	2424	65.04
12/4/2020 15:26	195.5	193	189.3	2424	64.96
12/4/2020 15:26	195.6	193.1	189.1	2419	65
12/4/2020 15:26	195.5	193.1	189.3	2416	64.96
12/4/2020 15:26	195.5	193.1	189.4	2413	64.96
12/4/2020 15:26	195.6	193.1	189.3	2417	65
12/4/2020 15:26	195.5	193.1	189.5	2423	65
12/4/2020 15:26	195.6	192.9	190.2	2428	65.08
12/4/2020 15:26	195.6	193.1	189.1	2427	64.83
12/4/2020 15:26	195.6	192.9	189.1	2427	64.88
12/4/2020 15:26	195.6	193.2	189.4	2428	65.08
12/4/2020 15:26	195.6	193	189.3	2426	64.8
12/4/2020 15:26	195.6	193.1	189.4	2423	64.99
12/4/2020 15:26	195.6	193.1	189.1	2423	65.16
12/4/2020 15:26	195.5	193	189.6	2425	64.93
12/4/2020 15:26	195.6	193.1	190.2	2424	64.88
12/4/2020 15:26	195.6	192.9	189.5	2426	64.92
12/4/2020 15:26	195.6	193.1	189	2426	64.88
12/4/2020 15:26	195.6	193.3	188.8	2424	64.92
12/4/2020 15:26	195.6	193.2	189.1	2429	65.16
12/4/2020 15:26	195.6	193.1	189.2	2432	64.84
12/4/2020 15:26	195.6	193	189.3	2429	65.04
12/4/2020 15:26	195.6	193.1	189.3	2427	65.12
12/4/2020 15:26	195.6	193.1	189.3	2426	64.99
12/4/2020 15:26	195.5	193.1	189.3	2427	65
12/4/2020 15:26	195.6	193	189.3	2431	64.96
12/4/2020 15:26	195.6	193.1	189.3	2432	65
12/4/2020 15:26	195.6	193	188.9	2428	64.96
12/4/2020 15:26	195.6	193.2	189.1	2426	64.96
12/4/2020 15:26	195.6	193	189.1	2425	65.04
12/4/2020 15:26	195.6	193.1	188.9	2422	65
12/4/2020 15:26	195.6	193	189.5	2418	64.96
12/4/2020 15:26	195.5	193.1	189.9	2418	65

12/4/2020 15:26	195.5	192.9	190.2	2413	64.88
12/4/2020 15:26	195.6	192.9	189.6	2416	64.96
12/4/2020 15:26	195.6	193.3	189.2	2419	65
12/4/2020 15:26	195.6	192.9	189.3	2420	64.92
12/4/2020 15:26	195.6	193.1	189.3	2422	64.96
12/4/2020 15:26	195.6	193.1	189.3	2425	65.08
12/4/2020 15:26	195.6	193.1	189.1	2419	64.96
12/4/2020 15:26	195.6	193.1	189.1	2419	64.96
12/4/2020 15:26	195.5	193.1	189.2	2423	65.08
12/4/2020 15:26	195.6	193	190	2429	64.96
12/4/2020 15:26	195.5	193.2	190.1	2426	64.99
12/4/2020 15:26	195.5	193	189.4	2424	65.04
12/4/2020 15:26	195.6	193.1	189.1	2425	64.93
12/4/2020 15:26	195.5	192.9	189.3	2424	64.88
12/4/2020 15:26	195.6	193.1	189.5	2424	64.92
12/4/2020 15:26	195.6	193.1	189.3	2420	65.12
12/4/2020 15:26	195.5	193	189.3	2420	64.99
12/4/2020 15:26	195.5	193.1	189	2420	65.04
12/4/2020 15:26	195.5	192.9	189.5	2422	64.96
12/4/2020 15:26	195.6	193	190.3	2420	64.99
12/4/2020 15:26	195.5	193.1	189.9	2417	65.08
12/4/2020 15:26	195.5	193.3	189.3	2416	64.84
12/4/2020 15:26	195.5	193.2	189.3	2416	64.92
12/4/2020 15:26	195.5	192.9	189.5	2416	64.92
12/4/2020 15:26	195.4	193.1	189.6	2420	65.08
12/4/2020 15:26	195.5	193.2	189.3	2416	64.88
12/4/2020 15:27	195.5	193.2	189.1	2419	64.96
12/4/2020 15:27	195.5	193	189	2418	65
12/4/2020 15:27	195.6	192.8	190	2421	64.96
12/4/2020 15:27	195.6	193	190.2	2419	64.96
12/4/2020 15:27	195.6	193.2	189.5	2416	65
12/4/2020 15:27	195.5	192.9	189.3	2416	65.04
12/4/2020 15:27	195.5	193.1	189.5	2415	64.96
12/4/2020 15:27	195.5	193.1	189.6	2419	64.96
12/4/2020 15:27	195.5	192.9	189.5	2427	65.09
12/4/2020 15:27	195.5	193.2	189.3	2424	64.96
12/4/2020 15:27	195.5	193.1	189.2	2424	64.96
12/4/2020 15:27	195.5	193.1	189.3	2427	65.05
12/4/2020 15:27	195.5	193	190	2427	64.96
12/4/2020 15:27	195.5	192.9	190.3	2427	65
12/4/2020 15:27	195.5	192.9	190	2427	64.96
12/4/2020 15:27	195.5	192.9	189.3	2426	64.96
12/4/2020 15:27	195.5	193.1	189.3	2429	65.08
12/4/2020 15:27	195.6	193.1	189.5	2432	64.99
12/4/2020 15:27	195.5	193.1	189.7	2435	65.04
12/4/2020 15:27	195.6	193.1	189.6	2431	64.88
12/4/2020 15:27	195.6	192.9	189.5	2427	64.92

12/4/2020 15:27	195.5	193.1	189.4	2426	65.04
12/4/2020 15:27	195.5	193	189.4	2429	64.92
12/4/2020 15:27	195.5	192.9	189.2	2424	65.04
12/4/2020 15:27	195.5	193.3	189.2	2423	65.08
12/4/2020 15:27	195.5	193	189.1	2423	64.88
12/4/2020 15:27	195.6	193	189.1	2427	64.88
12/4/2020 15:27	195.6	193.2	189.2	2427	65.04
12/4/2020 15:27	195.6	192.9	189.1	2424	64.95
12/4/2020 15:27	195.5	193.1	189.1	2422	65.04
12/4/2020 15:27	195.6	193.1	189.1	2423	65.12
12/4/2020 15:27	195.5	193.1	189.1	2420	64.99
12/4/2020 15:27	195.6	193	189.3	2423	64.99
12/4/2020 15:27	195.6	193.1	189.1	2427	64.84
12/4/2020 15:27	195.6	193.3	189.2	2429	64.96
12/4/2020 15:27	195.7	193.1	189.4	2429	64.84
12/4/2020 15:27	195.6	193.2	189.4	2428	64.88
12/4/2020 15:27	195.6	193.1	189.6	2432	64.92
12/4/2020 15:27	195.6	193	189.6	2429	64.99
12/4/2020 15:27	195.6	193.1	189.5	2428	65.04
12/4/2020 15:27	195.6	193.1	189.4	2426	64.96
12/4/2020 15:27	195.6	193.1	190	2423	64.8
12/4/2020 15:27	195.6	193	190.2	2422	64.88
12/4/2020 15:27	195.6	192.9	189.5	2427	65.08
12/4/2020 15:27	195.6	193.3	189.2	2429	65.04
12/4/2020 15:27	195.6	193.1	189.3	2431	64.99
12/4/2020 15:27	195.7	193.1	189.6	2429	64.99
12/4/2020 15:27	195.6	193	189.4	2428	64.96
12/4/2020 15:27	195.6	193	190.1	2431	65.04
12/4/2020 15:27	195.6	193.2	190.2	2431	64.92
12/4/2020 15:27	195.7	193.1	189.1	2430	64.92
12/4/2020 15:27	195.6	193.1	189.8	2429	64.96
12/4/2020 15:27	195.6	192.9	189.6	2429	64.96
12/4/2020 15:27	195.6	192.9	189.5	2428	64.96
12/4/2020 15:27	195.6	193	189.4	2423	64.92
12/4/2020 15:27	195.6	192.9	190.3	2424	64.96
12/4/2020 15:27	195.6	192.9	189.8	2425	65
12/4/2020 15:27	195.6	193.1	189.2	2428	64.96
12/4/2020 15:27	195.7	193.1	189.5	2429	65
12/4/2020 15:28	195.6	193	189.6	2429	65
12/4/2020 15:28	195.6	193.1	189.6	2424	64.92
12/4/2020 15:28	195.6	193.1	189.5	2427	65.04
12/4/2020 15:28	195.6	193.2	190.1	2427	65.04
12/4/2020 15:28	195.6	193	190	2425	64.99
12/4/2020 15:28	195.7	193.1	189.3	2427	64.99
12/4/2020 15:28	195.7	193	189.7	2430	64.96
12/4/2020 15:28	195.7	193.1	189.6	2431	64.88
12/4/2020 15:28	195.7	193	189.7	2433	64.99

12/4/2020 15:28	195.7	192.9	189.5	2432	65.16
12/4/2020 15:28	195.7	193.2	190.2	2433	65.04
12/4/2020 15:28	195.7	193.1	190.1	2431	64.91
12/4/2020 15:28	195.7	193.2	189.3	2428	64.88
12/4/2020 15:28	195.6	193.1	189.3	2432	64.92
12/4/2020 15:28	195.6	193.1	189.6	2431	65.08
12/4/2020 15:28	195.7	193.1	189.8	2431	64.92
12/4/2020 15:28	195.7	193.1	189.3	2430	65.08
12/4/2020 15:28	195.7	193.1	190.1	2429	64.88
12/4/2020 15:28	195.7	193.1	190.2	2429	64.88
12/4/2020 15:28	195.7	192.9	189.3	2431	64.96
12/4/2020 15:28	195.7	193.1	189.6	2433	64.83
12/4/2020 15:28	195.7	193.1	189.6	2432	64.99
12/4/2020 15:28	195.7	193	189.6	2439	64.96
12/4/2020 15:28	195.7	193	189.5	2436	64.88
12/4/2020 15:28	195.7	193.1	190.4	2434	64.96
12/4/2020 15:28	195.8	193.1	189.9	2434	65
12/4/2020 15:28	195.7	193	189.3	2431	64.88
12/4/2020 15:28	195.7	193.3	189.5	2431	64.96
12/4/2020 15:28	195.7	193.1	189.7	2433	64.92
12/4/2020 15:28	195.7	192.9	189.6	2435	64.84
12/4/2020 15:28	195.7	192.9	189.5	2434	64.92
12/4/2020 15:28	195.7	193.1	190.3	2434	64.92
12/4/2020 15:28	195.7	193.1	189.9	2431	64.96
12/4/2020 15:28	195.7	193.1	189.3	2429	64.96
12/4/2020 15:28	195.7	193.1	189.3	2435	65.04
12/4/2020 15:28	195.7	193.3	189.7	2431	64.96
12/4/2020 15:28	195.7	192.9	189.8	2431	64.92
12/4/2020 15:28	195.8	193	189.8	2428	64.92
12/4/2020 15:28	195.7	192.9	189.6	2427	64.96
12/4/2020 15:28	195.7	193.2	190.2	2429	65
12/4/2020 15:28	195.7	193	190.6	2429	64.96
12/4/2020 15:28	195.8	193	189.8	2431	65
12/4/2020 15:28	195.7	193	189.5	2431	64.96
12/4/2020 15:28	195.8	193.1	189.5	2428	64.96
12/4/2020 15:28	195.9	193	189.8	2428	65.04
12/4/2020 15:28	195.8	193.1	189.9	2431	64.83
12/4/2020 15:28	195.7	192.9	189.9	2432	64.96
12/4/2020 15:28	195.9	193.1	189.9	2431	64.96
12/4/2020 15:28	195.7	193.1	189.7	2429	64.96
12/4/2020 15:28	195.8	193	189.7	2433	65.04
12/4/2020 15:28	195.8	193.1	189.5	2429	64.92
12/4/2020 15:28	195.8	193.1	189.8	2433	65.04
12/4/2020 15:28	195.8	192.9	190.1	2433	64.84
12/4/2020 15:28	195.8	192.9	190.3	2435	65.08
12/4/2020 15:28	195.8	192.9	190.4	2435	64.88
12/4/2020 15:28	195.9	193	190.5	2435	64.99

12/4/2020 15:28	195.9	192.9	190.1	2439	64.96
12/4/2020 15:28	195.7	193	189.6	2435	64.88
12/4/2020 15:28	195.8	192.9	189.3	2436	65.04
12/4/2020 15:29	195.8	193.2	189.5	2434	64.88
12/4/2020 15:29	195.8	193.1	189.6	2435	65.12
12/4/2020 15:29	195.8	193	189.7	2433	65.04
12/4/2020 15:29	195.8	193	189.8	2428	64.88
12/4/2020 15:29	195.9	193	189.9	2424	65.16
12/4/2020 15:29	195.8	193.1	189.6	2424	64.99
12/4/2020 15:29	195.9	193.1	189.5	2429	65.04
12/4/2020 15:29	195.9	193	190.1	2432	64.96
12/4/2020 15:29	195.8	193.1	190.5	2434	64.88
12/4/2020 15:29	195.8	193	189.8	2433	64.96
12/4/2020 15:29	195.9	192.9	189.4	2434	65.08
12/4/2020 15:29	195.8	193.1	189.6	2434	64.92
12/4/2020 15:29	195.9	193	189.9	2435	64.83
12/4/2020 15:29	195.8	193.1	189.7	2432	64.88
12/4/2020 15:29	195.8	192.9	189.5	2436	64.92
12/4/2020 15:29	195.9	193.2	190.7	2437	64.92
12/4/2020 15:29	195.9	193	189.8	2435	64.99
12/4/2020 15:29	195.8	192.8	189.3	2437	65
12/4/2020 15:29	195.9	193	189.8	2436	64.87
12/4/2020 15:29	195.9	193	189.9	2439	64.92
12/4/2020 15:29	195.9	193.2	189.6	2437	65.04
12/4/2020 15:29	195.9	193.2	189.9	2436	64.92
12/4/2020 15:29	195.9	193	190.7	2433	64.92
12/4/2020 15:29	195.9	193.1	189.7	2436	65.04
12/4/2020 15:29	195.8	192.9	189.7	2433	65
12/4/2020 15:29	195.9	193.1	189.8	2431	65
12/4/2020 15:29	195.8	193.2	189.9	2433	64.96
12/4/2020 15:29	195.9	193.1	190.1	2432	64.92
12/4/2020 15:29	195.9	193.1	189.6	2433	64.96
12/4/2020 15:29	195.9	193.1	190	2434	65
12/4/2020 15:29	195.9	192.9	190.8	2437	65.08
12/4/2020 15:29	195.9	193.1	190	2439	64.92
12/4/2020 15:29	195.9	193.2	189.3	2438	65.04
12/4/2020 15:29	195.9	193.1	189.6	2439	65
12/4/2020 15:29	195.9	193.1	190.1	2438	64.88
12/4/2020 15:29	195.9	193	190	2438	64.99
12/4/2020 15:29	195.9	193.2	190	2439	64.93
12/4/2020 15:29	195.8	193	189.8	2440	65
12/4/2020 15:29	195.9	193.1	190.1	2441	64.99
12/4/2020 15:29	195.9	193.1	190.7	2444	65.12
12/4/2020 15:29	195.9	192.9	190.5	2440	65.04
12/4/2020 15:29	195.9	193.2	189.8	2436	64.88
12/4/2020 15:29	195.9	192.9	189.6	2433	65.04
12/4/2020 15:29	195.9	193.1	189.6	2433	64.99

12/4/2020 15:29	195.9	193.1	189.8	2432	64.92
12/4/2020 15:29	195.9	193.1	190.1	2435	65.04
12/4/2020 15:29	195.9	193.2	190	2435	64.84
12/4/2020 15:29	195.9	192.9	189.9	2430	64.99
12/4/2020 15:29	195.9	193.3	190	2433	64.99
12/4/2020 15:29	195.9	193	189.9	2434	65.04
12/4/2020 15:29	195.9	192.9	189.9	2429	64.91
12/4/2020 15:29	195.9	193.1	189.8	2431	65
12/4/2020 15:29	195.9	193	189.6	2435	64.97
12/4/2020 15:29	195.9	193	189.8	2432	64.96
12/4/2020 15:29	195.9	193.2	190	2432	64.92
12/4/2020 15:29	195.9	193	189.8	2432	65.04
12/4/2020 15:29	195.9	193.1	190.1	2431	64.88
12/4/2020 15:29	195.9	192.9	190.2	2434	64.88
12/4/2020 15:29	195.9	193.2	190.2	2438	64.96
12/4/2020 15:29	195.9	193.1	190.2	2440	65.04
12/4/2020 15:30	195.9	193	190	2440	64.99
12/4/2020 15:30	195.9	193.1	190.1	2440	64.92
12/4/2020 15:30	195.9	192.9	190	2437	64.99
12/4/2020 15:30	195.9	193.1	189.9	2431	64.83
12/4/2020 15:30	195.9	193.1	189.9	2432	64.92
12/4/2020 15:30	195.9	193.1	190	2433	65.09
12/4/2020 15:30	195.9	193.1	189.9	2433	64.87
12/4/2020 15:30	195.9	193.1	189.8	2435	64.96
12/4/2020 15:30	195.9	193	189.6	2436	65.13
12/4/2020 15:30	195.9	192.8	189.8	2439	65.04
12/4/2020 15:30	195.9	193	189.7	2440	64.92
12/4/2020 15:30	195.9	193.3	189.8	2440	65
12/4/2020 15:30	195.9	193	189.9	2439	64.92
12/4/2020 15:30	195.9	193.2	190	2438	64.87
12/4/2020 15:30	195.9	193.1	190.1	2442	65
12/4/2020 15:30	195.9	192.8	190.2	2443	64.91
12/4/2020 15:30	195.9	193.1	190.3	2439	64.96
12/4/2020 15:30	195.9	193.1	190.3	2439	65.09
12/4/2020 15:30	195.9	193.3	190.6	2438	64.92
12/4/2020 15:30	195.9	193	190.8	2437	64.96
12/4/2020 15:30	196	192.9	190.5	2435	65.04
12/4/2020 15:30	195.9	193.1	190.5	2437	64.99
12/4/2020 15:30	196	193	190.5	2439	64.92
12/4/2020 15:30	196	193.1	190.4	2440	65.04
12/4/2020 15:30	195.9	193.1	189.8	2442	65.04
12/4/2020 15:30	195.9	192.9	189.8	2438	64.84
12/4/2020 15:30	196	193.1	189.7	2435	64.99
12/4/2020 15:30	196.1	193	189.8	2439	64.96
12/4/2020 15:30	196	193.3	190	2437	64.99
12/4/2020 15:30	196	193.3	190	2431	64.84
12/4/2020 15:30	196.1	193.1	190.2	2432	65.16

12/4/2020 15:30	196.1	193.1	190.2	2429	64.84
12/4/2020 15:30	196.1	193.7	190.1	2431	64.92
12/4/2020 15:30	196	193.8	190.1	2433	64.93
12/4/2020 15:30	196.1	194.1	190.8	2433	64.88
12/4/2020 15:30	196.1	193.8	190.6	2431	64.96
12/4/2020 15:30	196.1	194	189.7	2436	65.04
12/4/2020 15:30	196.1	193.6	190	2437	64.88
12/4/2020 15:30	196.1	193.8	190.1	2435	64.88
12/4/2020 15:30	196.2	194.2	190.2	2439	65.04
12/4/2020 15:30	196.1	193.8	190	2441	64.96
12/4/2020 15:30	196.2	193.8	190.3	2438	65.04
12/4/2020 15:30	196.2	194	190.5	2438	65
12/4/2020 15:30	196.2	193.5	189.8	2442	65
12/4/2020 15:30	196.2	193.8	190.1	2445	64.91
12/4/2020 15:30	196.3	193.9	190.1	2444	65.04
12/4/2020 15:30	196.2	194	190.1	2441	65
12/4/2020 15:30	196.2	194	190	2440	64.87
12/4/2020 15:30	196.3	193.6	190.8	2442	64.96
12/4/2020 15:30	196.2	194	189.7	2444	65.04
12/4/2020 15:30	196.3	194	189.8	2438	64.96
12/4/2020 15:30	196.3	194.3	190.1	2441	65.04
12/4/2020 15:30	196.3	194.1	190.3	2437	65
12/4/2020 15:30	196.3	193.6	189.6	2440	65.04
12/4/2020 15:30	196.3	194.1	190.8	2435	64.99
12/4/2020 15:30	196.4	193.8	190.5	2440	65.04
12/4/2020 15:30	196.4	193.8	189.8	2438	65
12/4/2020 15:30	196.4	194.1	190.1	2437	64.96
12/4/2020 15:30	196.4	193.6	190.3	2438	64.92
12/4/2020 15:31	196.4	194.1	190.1	2436	65.08
12/4/2020 15:31	196.4	193.9	190.1	2439	64.88
12/4/2020 15:31	196.5	194	190.9	2438	65.04
12/4/2020 15:31	196.5	194.1	190.3	2437	65
12/4/2020 15:31	196.5	193.6	189.8	2430	65.08
12/4/2020 15:31	196.5	194	190.2	2433	64.99
12/4/2020 15:31	196.5	193.8	190.2	2436	65.12
12/4/2020 15:31	196.5	194.2	190.1	2438	64.96
12/4/2020 15:31	196.5	194	190.5	2435	65.08
12/4/2020 15:31	196.6	193.6	191	2434	65.12
12/4/2020 15:31	196.5	194.1	190.1	2435	64.96
12/4/2020 15:31	196.6	193.8	190	2438	65.08
12/4/2020 15:31	196.5	194.1	190.2	2441	64.99
12/4/2020 15:31	196.6	194.3	190.3	2438	64.88
12/4/2020 15:31	196.7	193.6	190.1	2438	64.92
12/4/2020 15:31	196.7	194.1	190.5	2441	65
12/4/2020 15:31	196.7	193.9	190.8	2448	65.08
12/4/2020 15:31	196.8	194.3	189.8	2446	64.83
12/4/2020 15:31	196.7	194.1	190	2442	64.88

12/4/2020 15:31	196.7	194.4	190.2	2444	65.08
12/4/2020 15:31	196.7	194.1	190.3	2440	64.96
12/4/2020 15:31	196.7	193.9	190.1	2441	65
12/4/2020 15:31	196.7	193.8	191	2441	64.96
12/4/2020 15:31	196.8	194.1	190.2	2439	65
12/4/2020 15:31	196.8	194.1	190	2434	65
12/4/2020 15:31	196.8	194.1	190	2440	65
12/4/2020 15:31	196.8	193.7	190	2441	64.96
12/4/2020 15:31	196.9	193.9	190.2	2442	64.96
12/4/2020 15:31	196.9	193.6	190.2	2439	64.96
12/4/2020 15:31	196.8	194	190.9	2438	65.05
12/4/2020 15:31	196.9	194.2	190.3	2436	64.88
12/4/2020 15:31	196.9	193.6	189.9	2437	64.96
12/4/2020 15:31	196.9	193.9	190.2	2440	64.96
12/4/2020 15:31	197	193.7	190.2	2444	64.92
12/4/2020 15:31	196.9	194	190.3	2441	65.08
12/4/2020 15:31	196.9	194.2	190.6	2444	65.08
12/4/2020 15:31	196.9	194	191	2448	64.8
12/4/2020 15:31	196.9	194.1	190	2443	64.88
12/4/2020 15:31	197	193.6	190	2442	65.17
12/4/2020 15:31	197	194.2	190.4	2440	65.08
12/4/2020 15:31	197	194.2	190.5	2436	64.92
12/4/2020 15:31	197	193.6	190.2	2439	64.92
12/4/2020 15:31	196.9	193.8	191	2440	64.99
12/4/2020 15:31	197	193.6	190.7	2438	64.92
12/4/2020 15:31	197	193.7	189.9	2436	64.92
12/4/2020 15:31	197	194.1	190.4	2437	65.04
12/4/2020 15:31	197	193.8	190.4	2438	64.88
12/4/2020 15:31	197	194.3	190.4	2438	65
12/4/2020 15:31	197	193.6	190.2	2438	65.09
12/4/2020 15:31	197	193.5	191	2441	64.92
12/4/2020 15:31	197	194.2	190.7	2438	64.96
12/4/2020 15:31	197	193.9	190	2440	65
12/4/2020 15:31	197	194	190.2	2440	64.92
12/4/2020 15:31	197	194.2	190.6	2436	65
12/4/2020 15:31	197	193.8	190.3	2439	64.96
12/4/2020 15:31	197	194.1	190.1	2440	65
12/4/2020 15:31	197	193.6	190.9	2439	65.08
12/4/2020 15:31	197	194.2	190.9	2439	65.04
12/4/2020 15:31	197	194.1	190	2436	64.92
12/4/2020 15:31	197	193.6	190	2434	65.08
12/4/2020 15:32	197	194	190.5	2434	64.88
12/4/2020 15:32	197	193.8	190.5	2434	65.09
12/4/2020 15:32	197	194.1	190.3	2433	64.88
12/4/2020 15:32	197	193.7	190.2	2434	65.04
12/4/2020 15:32	197	193.8	191.2	2437	65.12
12/4/2020 15:32	197	194.3	191	2437	64.99

12/4/2020 15:32	197	193.8	190.2	2436	65.08
12/4/2020 15:32	197	193.6	190.4	2440	64.96
12/4/2020 15:32	197	194.2	190.5	2437	64.99
12/4/2020 15:32	197	193.7	190.7	2435	64.96
12/4/2020 15:32	197	194.1	190.5	2435	65
12/4/2020 15:32	197	194	191.4	2431	64.92
12/4/2020 15:32	197	194	190.5	2430	65
12/4/2020 15:32	197	194.2	190.1	2433	65
12/4/2020 15:32	197	194.2	190.7	2436	64.96
12/4/2020 15:32	197	194	190.5	2439	65
12/4/2020 15:32	197	193.8	190.3	2441	65
12/4/2020 15:32	197	194.1	191	2441	65.04
12/4/2020 15:32	196.9	194.1	191.1	2439	65
12/4/2020 15:32	197	193.6	190.3	2442	65.04
12/4/2020 15:32	197	194.1	190.2	2445	64.92
12/4/2020 15:32	197	193.9	190.4	2444	64.88
12/4/2020 15:32	196.9	194.4	190.5	2442	65.08
12/4/2020 15:32	196.9	194.1	190.3	2440	64.92
12/4/2020 15:32	197	193.7	191.3	2435	64.92
12/4/2020 15:32	196.9	194.1	190.5	2438	64.92
12/4/2020 15:32	196.9	193.8	190	2446	65.08
12/4/2020 15:32	197	193.8	190.5	2447	65.04
12/4/2020 15:32	196.9	194.2	190.7	2448	64.99
12/4/2020 15:32	196.9	193.8	190.3	2445	65
12/4/2020 15:32	196.9	194.1	190.4	2443	64.92
12/4/2020 15:32	196.9	193.8	191	2440	64.96
12/4/2020 15:32	196.9	194	190.9	2440	65.05
12/4/2020 15:32	196.9	193.6	190.2	2442	65
12/4/2020 15:32	196.9	193.8	190.1	2442	65.04
12/4/2020 15:32	197	194.2	190.6	2441	64.96
12/4/2020 15:32	197	193.7	190.6	2444	65.04
12/4/2020 15:32	197	194.2	190.3	2445	65.04
12/4/2020 15:32	197	193.8	191	2443	65.04
12/4/2020 15:32	196.9	193.8	191.1	2443	64.96
12/4/2020 15:32	196.9	194.3	190.3	2440	64.92
12/4/2020 15:32	197	193.8	190.2	2440	65.04
12/4/2020 15:32	197	194.2	190.5	2441	64.92
12/4/2020 15:32	197	194.2	190.6	2439	64.99
12/4/2020 15:32	196.9	193.6	190.3	2438	64.92
12/4/2020 15:32	197	194	190.6	2441	65.08
12/4/2020 15:32	197	193.8	191.4	2441	64.84
12/4/2020 15:32	197	194.2	190.7	2442	65.04
12/4/2020 15:32	197	194.1	190.4	2443	64.88
12/4/2020 15:32	196.9	193.7	190.5	2442	64.99
12/4/2020 15:32	196.9	194.2	190.7	2444	64.84
12/4/2020 15:32	197	193.8	190.7	2445	64.96
12/4/2020 15:32	197	194.2	190.5	2444	65.04

12/4/2020 15:32	197	194.2	191.3	2445	65.08
12/4/2020 15:32	196.9	193.6	190.6	2449	65.04
12/4/2020 15:32	197	194	190.2	2447	64.96
12/4/2020 15:32	196.9	193.6	190.7	2445	65.04
12/4/2020 15:32	197	194.3	190.7	2443	64.96
12/4/2020 15:32	197	194.1	190.6	2444	65
12/4/2020 15:33	197	193.8	190.7	2444	64.87
12/4/2020 15:33	197	194	191.4	2444	64.96
12/4/2020 15:33	197	194.1	190.8	2444	65.09
12/4/2020 15:33	197	194.2	190.2	2441	64.92
12/4/2020 15:33	197	194.1	190.3	2443	65
12/4/2020 15:33	197	193.7	190.9	2446	65.05
12/4/2020 15:33	197	194.1	190.7	2442	64.96
12/4/2020 15:33	197	193.8	190.5	2446	64.96
12/4/2020 15:33	197	193.6	191	2446	65.08
12/4/2020 15:33	197	194.1	191.3	2445	64.96
12/4/2020 15:33	197	193.6	190.4	2440	65.08
12/4/2020 15:33	197	194.1	190.3	2441	65.03
12/4/2020 15:33	197	194	190.4	2446	64.92
12/4/2020 15:33	197	193.6	190.7	2444	65.08
12/4/2020 15:33	197	194.3	190.7	2444	64.99
12/4/2020 15:33	197	193.8	190.4	2444	65.04
12/4/2020 15:33	197	194	190.7	2449	64.88
12/4/2020 15:33	197	194.1	191.4	2449	64.99
12/4/2020 15:33	197	193.8	191	2449	65.12
12/4/2020 15:33	197	194.2	190.2	2449	65.04
12/4/2020 15:33	197	194	190.3	2442	64.92
12/4/2020 15:33	197	194	190.7	2444	64.96
12/4/2020 15:33	197	193.8	190.7	2441	64.92
12/4/2020 15:33	197	193.5	190.2	2442	65
12/4/2020 15:33	197	194.1	190.9	2445	65.13
12/4/2020 15:33	197	194	191.3	2446	64.96
12/4/2020 15:33	197	194.1	190.3	2444	65
12/4/2020 15:33	197	193.7	190.4	2445	64.96
12/4/2020 15:33	197	193.8	190.8	2446	64.96
12/4/2020 15:33	197.1	194.2	190.7	2446	64.83
12/4/2020 15:33	197.1	193.7	190.5	2447	64.84
12/4/2020 15:33	197	194.1	191.1	2448	65.08
12/4/2020 15:33	197.1	193.6	191.6	2442	64.88
12/4/2020 15:33	197	194.2	190.2	2439	65
12/4/2020 15:33	197	194.2	190.5	2447	64.97
12/4/2020 15:33	197	193.6	190.8	2449	65.03
12/4/2020 15:33	197	194.1	190.8	2448	64.92
12/4/2020 15:33	197.1	194	190.7	2447	64.96
12/4/2020 15:33	197.1	193.8	191	2445	65.12
12/4/2020 15:33	197.1	193.7	191.7	2446	65.08
12/4/2020 15:33	197	193.8	190.6	2449	64.88

12/4/2020 15:33	197.1	194.2	190.3	2449	65.08
12/4/2020 15:33	197	194.1	190.8	2451	64.96
12/4/2020 15:33	197.1	193.8	190.9	2453	64.96
12/4/2020 15:33	197.1	194.2	190.6	2452	65.08
12/4/2020 15:33	197.2	193.6	191	2452	64.88
12/4/2020 15:33	197.1	194.1	191.6	2450	65.08
12/4/2020 15:33	197.2	194	190.9	2449	65.08
12/4/2020 15:33	197.2	193.6	190.6	2452	64.96
12/4/2020 15:33	197.1	193.9	190.6	2452	65
12/4/2020 15:33	197.2	193.8	190.9	2455	65
12/4/2020 15:33	197.2	194.2	190.8	2456	64.96
12/4/2020 15:33	197.2	193.5	190.8	2456	64.96
12/4/2020 15:33	197.2	194.1	191.5	2456	65
12/4/2020 15:33	197.2	194.2	191.1	2451	65
12/4/2020 15:33	197.2	193.6	190.3	2448	65
12/4/2020 15:33	197.2	194.1	190.9	2449	65.04
12/4/2020 15:33	197.2	194	190.9	2445	65.08
12/4/2020 15:33	197.2	194.2	190.9	2445	65.03
12/4/2020 15:34	197.2	194.2	190.6	2445	65.04
12/4/2020 15:34	197.2	193.6	191.3	2445	65.21
12/4/2020 15:34	197.2	194.2	191.6	2447	64.92
12/4/2020 15:34	197.2	193.7	190.5	2445	64.92
12/4/2020 15:34	197.2	194.1	190.5	2448	64.96
12/4/2020 15:34	197.2	193.8	190.7	2444	64.99
12/4/2020 15:34	197.2	193.8	190.8	2446	64.92
12/4/2020 15:34	197.3	193.9	190.8	2446	65.08
12/4/2020 15:34	197.3	194.2	190.5	2449	64.96
12/4/2020 15:34	197.3	193.6	191.1	2445	64.99
12/4/2020 15:34	197.3	194.2	191.7	2446	64.96
12/4/2020 15:34	197.3	193.8	190.4	2452	65.04
12/4/2020 15:34	197.3	194	190.8	2450	64.88
12/4/2020 15:34	197.3	193.7	190.9	2451	65
12/4/2020 15:34	197.3	193.6	191	2447	65
12/4/2020 15:34	197.3	194.1	190.9	2442	65
12/4/2020 15:34	197.4	193.9	191.2	2442	65
12/4/2020 15:34	197.4	194.2	191.7	2444	65.05
12/4/2020 15:34	197.4	193.7	190.6	2442	64.96
12/4/2020 15:34	197.4	194.1	190.7	2442	65.04
12/4/2020 15:34	197.4	194.1	191.2	2446	64.96
12/4/2020 15:34	197.4	194	191.1	2449	64.99
12/4/2020 15:34	197.4	194	190.7	2445	64.92
12/4/2020 15:34	197.4	193.8	191.7	2447	65.01
12/4/2020 15:34	197.4	193.6	191.6	2443	64.99
12/4/2020 15:34	197.4	193.8	190.6	2442	64.92
12/4/2020 15:34	197.4	193.9	190.9	2444	64.93
12/4/2020 15:34	197.4	194.2	190.9	2450	64.92
12/4/2020 15:34	197.4	195.4	191.2	2452	65.12

12/4/2020 15:34	197.4	194.6	191	2453	65.08
12/4/2020 15:34	197.4	195.2	190.8	2453	64.96
12/4/2020 15:34	197.4	194.7	191.9	2453	64.96
12/4/2020 15:34	197.4	195.2	191.2	2449	65.09
12/4/2020 15:34	197.4	195.2	190.7	2451	65.04
12/4/2020 15:34	197.4	194.7	190.8	2445	64.96
12/4/2020 15:34	197.5	195	191	2445	64.96
12/4/2020 15:34	197.5	194.5	191.2	2449	65
12/4/2020 15:34	197.5	194.8	191.1	2452	64.96
12/4/2020 15:34	197.5	195.4	191	2448	65.04
12/4/2020 15:34	197.5	195.5	190.9	2450	65
12/4/2020 15:34	197.5	195.2	190.7	2452	64.88
12/4/2020 15:34	197.4	194.7	190.9	2453	65
12/4/2020 15:34	197.5	194.8	191	2448	65.08
12/4/2020 15:34	197.5	194.7	191.2	2446	64.92
12/4/2020 15:34	197.5	194.8	191.1	2445	64.92
12/4/2020 15:34	197.4	195.4	190.8	2449	65.2
12/4/2020 15:34	197.4	194.5	190.9	2445	64.88
12/4/2020 15:34	197.5	195.2	190.9	2445	65.03
12/4/2020 15:34	197.5	195.4	190.8	2442	64.92
12/4/2020 15:34	197.5	195.2	191	2447	64.96
12/4/2020 15:34	197.5	195.2	191	2442	65
12/4/2020 15:34	197.5	194.6	191.2	2443	64.88
12/4/2020 15:34	197.5	194.8	191.2	2444	65.12
12/4/2020 15:34	197.5	195.4	191	2441	64.95
12/4/2020 15:34	197.5	195.2	190.9	2445	64.96
12/4/2020 15:34	197.5	195.2	190.7	2447	65.05
12/4/2020 15:34	197.5	194.6	191.7	2444	64.99
12/4/2020 15:34	197.5	194.8	191.9	2440	64.92
12/4/2020 15:34	197.5	195.2	191.4	2442	65.09
12/4/2020 15:34	197.5	194.7	190.8	2442	64.92
12/4/2020 15:35	197.5	195.5	191	2442	64.96
12/4/2020 15:35	197.5	195.2	191.4	2442	65
12/4/2020 15:35	197.6	194.7	191.2	2445	64.96
12/4/2020 15:35	197.6	195.4	191.2	2442	64.92
12/4/2020 15:35	197.6	195.1	190.9	2440	64.92
12/4/2020 15:35	197.6	195.3	190.9	2445	65
12/4/2020 15:35	197.6	195	190.8	2445	64.87
12/4/2020 15:35	197.6	195.4	191.2	2449	64.96
12/4/2020 15:35	197.6	194.7	191.7	2456	64.96
12/4/2020 15:35	197.6	194.8	191.9	2450	64.92
12/4/2020 15:35	197.7	195.1	191.8	2449	65.04
12/4/2020 15:35	197.6	195.2	191.5	2452	65.04
12/4/2020 15:35	197.6	194.8	191	2447	64.92
12/4/2020 15:35	197.6	195.2	190.9	2444	64.88
12/4/2020 15:35	197.6	194.8	190.8	2444	65.08
12/4/2020 15:35	197.7	195.2	190.9	2445	65.12

12/4/2020 15:35	197.6	194.7	191.1	2444	64.95
12/4/2020 15:35	197.6	195.3	191.1	2444	64.91
12/4/2020 15:35	197.7	195.2	191.4	2448	65.04
12/4/2020 15:35	197.7	194.6	191.2	2447	64.84
12/4/2020 15:35	197.7	195.2	191.1	2449	65.04
12/4/2020 15:35	197.6	195	191.4	2449	65.04
12/4/2020 15:35	197.6	195.3	190.3	2445	64.99
12/4/2020 15:35	197.7	194.9	191.5	2444	64.88
12/4/2020 15:35	197.7	194.7	191.5	2444	65.04
12/4/2020 15:35	197.7	195.1	191.4	2445	64.88
12/4/2020 15:35	197.7	194.5	191.2	2444	64.88
12/4/2020 15:35	197.7	195.2	191	2446	65.01
12/4/2020 15:35	197.8	195	191.2	2443	64.92
12/4/2020 15:35	197.7	194.7	191.4	2442	64.92
12/4/2020 15:35	197.8	195	191.4	2442	65.04
12/4/2020 15:35	197.7	194.5	191.5	2441	65
12/4/2020 15:35	197.7	194.7	191.4	2442	64.92
12/4/2020 15:35	197.8	195.2	191.4	2441	64.88
12/4/2020 15:35	197.8	194.7	191.4	2436	64.96
12/4/2020 15:35	197.8	195.1	191.1	2436	64.91
12/4/2020 15:35	197.8	195.2	191.2	2438	64.96
12/4/2020 15:35	197.8	194.6	191.5	2443	64.96
12/4/2020 15:35	197.8	195.4	191.3	2440	64.96
12/4/2020 15:35	197.7	194.7	191.4	2441	65
12/4/2020 15:35	197.8	195.4	191.1	2442	65
12/4/2020 15:35	197.8	194.8	191.2	2442	64.92
12/4/2020 15:35	197.9	194.7	191.2	2444	64.92
12/4/2020 15:35	197.8	195.4	191	2444	64.96
12/4/2020 15:35	197.8	194.8	191.1	2444	64.96
12/4/2020 15:35	197.8	195.4	191	2449	64.87
12/4/2020 15:35	197.8	194.6	191	2445	64.92
12/4/2020 15:35	197.8	194.6	191.1	2442	64.96
12/4/2020 15:35	197.8	195.4	191.2	2441	64.96
12/4/2020 15:35	197.8	194.9	191	2441	64.96
12/4/2020 15:35	197.8	195.4	191.4	2445	65.04
12/4/2020 15:35	197.8	195.2	191.4	2442	64.96
12/4/2020 15:35	197.9	194.6	191.5	2445	64.96
12/4/2020 15:35	197.9	195.2	191.7	2445	64.96
12/4/2020 15:35	197.9	194.8	191.7	2442	65.04
12/4/2020 15:35	197.9	195.2	191.9	2441	64.96
12/4/2020 15:35	197.9	195.1	192.2	2444	65.08
12/4/2020 15:35	197.9	194.5	192.2	2445	65
12/4/2020 15:35	197.9	194.8	192	2448	64.92
12/4/2020 15:36	197.8	194.8	191.9	2448	64.84
12/4/2020 15:36	197.9	195.2	191.4	2445	65.04
12/4/2020 15:36	197.9	194.8	190.9	2444	64.95
12/4/2020 15:36	197.9	195.4	191	2442	65.08

12/4/2020 15:36	198	195	191.3	2448	65.12
12/4/2020 15:36	197.9	194.5	191.5	2447	64.92
12/4/2020 15:36	197.8	195	191.5	2447	65.04
12/4/2020 15:36	197.9	195.2	191.4	2450	65.08
12/4/2020 15:36	197.9	195.3	191.7	2449	64.96
12/4/2020 15:36	197.9	194.9	192.1	2449	64.92
12/4/2020 15:36	197.9	194.7	190.9	2449	64.88
12/4/2020 15:36	197.9	195	191.2	2448	65.08
12/4/2020 15:36	198	195.2	191.6	2444	64.99
12/4/2020 15:36	197.9	195.5	191	2443	65.04
12/4/2020 15:36	197.9	194.9	191.9	2444	64.93
12/4/2020 15:36	197.9	194.7	192.1	2445	64.92
12/4/2020 15:36	197.9	195	191	2448	64.88
12/4/2020 15:36	197.9	194.7	191.3	2449	65.08
12/4/2020 15:36	197.9	194.5	191.5	2450	64.95
12/4/2020 15:36	197.9	195.5	191.4	2454	65.08
12/4/2020 15:36	197.9	194.8	191.4	2459	65.04
12/4/2020 15:36	197.9	195.2	192.1	2454	65.04
12/4/2020 15:36	197.9	194.6	191.7	2455	64.92
12/4/2020 15:36	197.9	195.2	191	2455	65.12
12/4/2020 15:36	198	195.4	191	2449	64.96
12/4/2020 15:36	198	194.6	191.5	2444	64.92
12/4/2020 15:36	197.9	194.7	191.5	2448	64.96
12/4/2020 15:36	198	194.6	191.4	2447	64.92
12/4/2020 15:36	198	195	191.7	2446	64.91
12/4/2020 15:36	198	195	192.2	2448	65.04
12/4/2020 15:36	198	194.9	191.8	2449	64.87
12/4/2020 15:36	198	194.8	191	2449	65
12/4/2020 15:36	198	194.6	191.1	2455	65.04
12/4/2020 15:36	197.9	195	191.5	2451	64.96
12/4/2020 15:36	198	195	191.4	2450	65
12/4/2020 15:36	198	194.8	191	2452	65
12/4/2020 15:36	198	194.8	192.1	2450	65
12/4/2020 15:36	198	195.5	191.7	2446	64.91
12/4/2020 15:36	198	194.6	190.8	2449	64.96
12/4/2020 15:36	198	195.4	191.4	2452	64.96
12/4/2020 15:36	198	194.7	191.5	2453	64.99
12/4/2020 15:36	198	194.9	191.5	2451	64.96
12/4/2020 15:36	198	195.3	191.3	2453	65
12/4/2020 15:36	198	195.3	192.3	2453	64.87
12/4/2020 15:36	198	195.2	191.9	2456	65
12/4/2020 15:36	198	194.9	191	2455	65.04
12/4/2020 15:36	198	194.8	191.2	2456	64.92
12/4/2020 15:36	198	194.7	191.5	2453	64.96
12/4/2020 15:36	198	195	191.7	2449	65.04
12/4/2020 15:36	198	195.2	191.5	2450	64.92
12/4/2020 15:36	198	194.8	191.4	2451	64.88

12/4/2020 15:36	198.1	194.7	191.7	2456	65.08
12/4/2020 15:36	198.1	195.3	192.5	2455	64.92
12/4/2020 15:36	198	195	191.9	2455	65.04
12/4/2020 15:36	198	195.4	191.6	2454	64.84
12/4/2020 15:36	198	194.8	191.3	2454	64.88
12/4/2020 15:36	198	194.8	191.1	2453	64.92
12/4/2020 15:36	198.1	194.7	191.4	2448	64.88
12/4/2020 15:36	198	194.7	191.6	2449	65.04
12/4/2020 15:36	198	195.4	192.2	2453	64.83
12/4/2020 15:37	198	194.9	191.7	2452	65.04
12/4/2020 15:37	198	195.4	191.6	2453	64.93
12/4/2020 15:37	198	195.3	191.6	2455	64.92
12/4/2020 15:37	198	194.7	191.6	2454	65.12
12/4/2020 15:37	198.1	195.2	191.7	2457	64.99
12/4/2020 15:37	198.1	194.8	191.4	2456	64.95
12/4/2020 15:37	198.1	195.1	191.5	2455	64.92
12/4/2020 15:37	198	195.5	191.3	2450	64.96
12/4/2020 15:37	198	195.2	191.4	2452	64.84
12/4/2020 15:37	198	194.9	191.5	2450	64.83
12/4/2020 15:37	198	194.9	191.7	2452	64.99
12/4/2020 15:37	198.1	194.5	191.8	2453	65.04
12/4/2020 15:37	198.1	194.8	191.8	2451	64.8
12/4/2020 15:37	198.1	195	191.7	2451	64.92
12/4/2020 15:37	198	195.3	191.6	2452	64.92
12/4/2020 15:37	198.1	195.1	191.7	2452	64.92
12/4/2020 15:37	198.1	194.6	191.5	2454	64.99
12/4/2020 15:37	198	195.4	191	2452	64.96
12/4/2020 15:37	198.1	194.5	191.3	2452	64.99
12/4/2020 15:37	198	195.2	191.8	2447	64.99
12/4/2020 15:37	198	194.7	192.5	2449	65.04
12/4/2020 15:37	198.1	195.2	192.4	2448	64.96
12/4/2020 15:37	198.1	195.4	191.5	2449	64.96
12/4/2020 15:37	198.1	194.7	191.5	2452	65.04
12/4/2020 15:37	198.1	195.2	191.9	2455	64.87
12/4/2020 15:37	198.1	195.2	191.6	2455	64.96
12/4/2020 15:37	198.1	194.7	191.5	2456	64.96
12/4/2020 15:37	198.1	195	191.2	2456	65
12/4/2020 15:37	198.1	194.8	191.7	2452	64.96
12/4/2020 15:37	198.1	195	192.6	2450	64.92
12/4/2020 15:37	198.1	195.1	192	2453	65.04
12/4/2020 15:37	198.2	195.4	191.6	2452	64.92
12/4/2020 15:37	198.1	195.1	191.6	2449	64.96
12/4/2020 15:37	198.1	194.6	191.5	2455	64.96
12/4/2020 15:37	198.1	194.9	191.7	2452	64.92
12/4/2020 15:37	198.2	195.4	191.5	2456	64.96
12/4/2020 15:37	198.2	195.4	191.4	2457	64.96
12/4/2020 15:37	198.1	195.4	191.5	2459	65

12/4/2020 15:37	198.1	194.9	191.5	2460	64.92
12/4/2020 15:37	198.2	195.1	192.5	2457	64.83
12/4/2020 15:37	198.2	195.2	192.5	2460	64.99
12/4/2020 15:37	198.2	194.7	191.7	2458	64.88
12/4/2020 15:37	198.2	195.4	191.7	2459	65.04
12/4/2020 15:37	198.2	195	191.5	2460	65.04
12/4/2020 15:37	198.2	195	191.7	2451	65
12/4/2020 15:37	198.3	194.6	191.7	2452	65.03
12/4/2020 15:37	198.3	194.8	192	2455	64.92
12/4/2020 15:37	198.2	195.4	191.8	2452	64.88
12/4/2020 15:37	198.2	194.6	191.7	2453	64.95
12/4/2020 15:37	198.2	195.4	191.7	2453	65.12
12/4/2020 15:37	198.3	194.7	191.7	2456	65.04
12/4/2020 15:37	198.2	194.6	191.5	2456	64.84
12/4/2020 15:37	198.2	195.4	191.4	2458	64.92
12/4/2020 15:37	198.3	194.8	191.5	2464	64.96
12/4/2020 15:37	198.3	195.4	191.4	2457	65.04
12/4/2020 15:37	198.2	195	191.5	2455	65.03
12/4/2020 15:37	198.2	194.8	191.3	2454	65.08
12/4/2020 15:37	198.3	195.4	191.3	2456	64.95
12/4/2020 15:37	198.3	194.7	191.5	2457	64.84
12/4/2020 15:38	198.3	195.5	191.4	2460	65.12
12/4/2020 15:38	198.2	195.1	191.7	2459	64.95
12/4/2020 15:38	198.2	195.1	191.7	2457	64.88
12/4/2020 15:38	198.3	195.4	191.6	2462	64.92
12/4/2020 15:38	198.3	194.6	191.9	2464	64.84
12/4/2020 15:38	198.3	195	191.9	2460	64.92
12/4/2020 15:38	198.2	195.2	191.9	2461	65
12/4/2020 15:38	198.3	194.7	191.9	2463	64.92
12/4/2020 15:38	198.3	194.8	191.7	2461	65.04
12/4/2020 15:38	198.3	194.7	192	2457	64.92
12/4/2020 15:38	198.3	195.1	191.6	2459	64.92
12/4/2020 15:38	198.3	195.5	191.5	2458	64.95
12/4/2020 15:38	198.3	195.4	192	2460	64.88
12/4/2020 15:38	198.3	195	192.3	2456	65
12/4/2020 15:38	198.3	194.8	192.5	2457	64.92
12/4/2020 15:38	198.3	194.7	192.2	2454	64.96
12/4/2020 15:38	198.3	194.5	191.5	2459	65
12/4/2020 15:38	198.4	195.1	191.6	2458	65
12/4/2020 15:38	198.3	195.1	191.5	2454	65
12/4/2020 15:38	198.3	194.9	191.6	2458	65.09
12/4/2020 15:38	198.3	195	192.1	2457	65.04
12/4/2020 15:38	198.3	194.8	191.8	2459	65
12/4/2020 15:38	198.3	194.8	191.9	2463	65
12/4/2020 15:38	198.3	195.2	191.7	2459	65
12/4/2020 15:38	198.3	194.9	191.7	2462	65.04
12/4/2020 15:38	198.3	195.5	192.2	2468	65.08

12/4/2020 15:38	198.3	194.8	192.3	2467	64.92
12/4/2020 15:38	198.3	194.6	192.5	2467	65.04
12/4/2020 15:38	198.4	195	192.6	2462	64.96
12/4/2020 15:38	198.4	194.7	192.4	2463	65.04
12/4/2020 15:38	198.3	195	192.6	2461	64.99
12/4/2020 15:38	198.4	195.3	192.7	2459	64.99
12/4/2020 15:38	198.3	195.3	192.8	2460	65.04
12/4/2020 15:38	198.4	195.2	192.6	2464	65.04
12/4/2020 15:38	198.3	196.2	192.3	2458	65.03
12/4/2020 15:38	198.4	195.9	191.7	2463	65.04
12/4/2020 15:38	198.4	196.4	191.7	2463	64.92
12/4/2020 15:38	198.4	196	191.7	2465	65.04
12/4/2020 15:38	198.4	196.3	192	2464	64.93
12/4/2020 15:38	198.4	195.6	192.1	2467	65.04
12/4/2020 15:38	198.5	195.7	191.7	2467	64.88
12/4/2020 15:38	198.4	196.4	191.7	2471	65.08
12/4/2020 15:38	198.4	195.9	191.5	2466	64.88
12/4/2020 15:38	198.5	196.4	192.2	2464	64.99
12/4/2020 15:38	198.4	196.2	192.9	2464	65.05
12/4/2020 15:38	198.4	195.9	192.2	2461	65.08
12/4/2020 15:38	198.4	196.4	191.6	2463	64.92
12/4/2020 15:38	198.4	195.6	192	2458	65
12/4/2020 15:38	198.5	196.4	192.1	2463	64.96
12/4/2020 15:38	198.4	196.1	192.1	2460	65
12/4/2020 15:38	198.4	195.7	191.9	2459	65.04
12/4/2020 15:38	198.5	196	191.7	2459	65
12/4/2020 15:38	198.5	196	191.7	2458	64.91
12/4/2020 15:38	198.4	196	191.9	2460	64.96
12/4/2020 15:38	198.4	196.3	192.7	2460	65.09
12/4/2020 15:38	198.5	196.4	192.4	2462	64.96
12/4/2020 15:38	198.5	196.2	191.7	2461	64.88
12/4/2020 15:38	198.4	195.6	192.1	2467	64.92
12/4/2020 15:38	198.4	196	192.2	2467	64.88
12/4/2020 15:38	198.4	196.3	191.8	2467	64.92
12/4/2020 15:39	198.5	195.7	191.9	2468	64.96
12/4/2020 15:39	198.5	196.2	191.5	2470	64.84
12/4/2020 15:39	198.5	195.7	191.7	2471	64.96
12/4/2020 15:39	198.5	195.7	192.9	2467	65.08
12/4/2020 15:39	198.5	195.7	192.7	2467	64.88
12/4/2020 15:39	198.5	195.7	191.9	2462	64.99
12/4/2020 15:39	198.5	196.4	191.8	2463	64.97
12/4/2020 15:39	198.5	195.7	191.8	2463	65.08
12/4/2020 15:39	198.5	195.7	192.2	2464	64.96
12/4/2020 15:39	198.5	195.6	192.1	2463	64.99
12/4/2020 15:39	198.5	195.6	191.9	2466	65.12
12/4/2020 15:39	198.4	196.1	191.8	2465	65
12/4/2020 15:39	198.5	196	191.5	2460	65.04

12/4/2020 15:39	198.5	196.4	191.7	2459	64.96
12/4/2020 15:39	198.5	196.4	192.3	2459	64.99
12/4/2020 15:39	198.4	195.9	192.5	2458	65.04
12/4/2020 15:39	198.5	196.2	192.8	2466	65.08
12/4/2020 15:39	198.5	195.9	192.1	2467	65.03
12/4/2020 15:39	198.5	195.9	191.9	2474	65.04
12/4/2020 15:39	198.5	195.7	191.6	2474	65.12
12/4/2020 15:39	198.5	196	191.9	2473	65
12/4/2020 15:39	198.5	196.4	192	2469	65
12/4/2020 15:39	198.5	196	192.2	2469	65.09
12/4/2020 15:39	198.5	196.1	192.1	2467	64.96
12/4/2020 15:39	198.5	195.7	192.2	2471	65
12/4/2020 15:39	198.5	196	192.1	2469	65.09
12/4/2020 15:39	198.5	196.1	192.1	2467	65
12/4/2020 15:39	198.5	196.1	191.9	2464	65.04
12/4/2020 15:39	198.5	196.3	191.9	2470	64.92
12/4/2020 15:39	198.5	195.9	191.9	2466	65.04
12/4/2020 15:39	198.5	195.7	192.5	2464	65.04
12/4/2020 15:39	198.5	196.4	192.9	2462	65.12
12/4/2020 15:39	198.5	196.2	192.9	2467	64.92
12/4/2020 15:39	198.5	196.4	192.4	2464	64.99
12/4/2020 15:39	198.5	195.8	192.1	2467	65.04
12/4/2020 15:39	198.5	195.6	191.9	2471	65.01
12/4/2020 15:39	198.5	196.2	192	2471	64.88
12/4/2020 15:39	198.5	196.2	192.2	2475	65.04
12/4/2020 15:39	198.5	196.3	192.3	2474	64.99
12/4/2020 15:39	198.5	196	192.4	2471	64.88
12/4/2020 15:39	198.5	195.6	192.2	2471	65
12/4/2020 15:39	198.5	196.1	192.1	2476	64.92
12/4/2020 15:39	198.4	196.4	191.9	2477	65.04
12/4/2020 15:39	198.5	196.2	191.7	2473	64.92
12/4/2020 15:39	198.5	196.2	191.6	2468	64.96
12/4/2020 15:39	198.5	195.9	192	2467	64.96
12/4/2020 15:39	198.5	196.2	192.2	2467	64.96
12/4/2020 15:39	198.5	195.6	192.9	2467	65.04
12/4/2020 15:39	198.5	196.2	192.9	2467	65
12/4/2020 15:39	198.5	196.4	192.5	2466	64.96
12/4/2020 15:39	198.5	195.8	192.2	2464	65.04
12/4/2020 15:39	198.5	196.2	191.9	2467	65.04
12/4/2020 15:39	198.5	195.8	191.9	2471	64.96
12/4/2020 15:39	198.5	195.6	192.2	2473	64.92
12/4/2020 15:39	198.5	195.7	192.1	2471	65
12/4/2020 15:39	198.5	195.8	192.2	2470	64.99
12/4/2020 15:39	198.5	196	192.1	2469	65
12/4/2020 15:39	198.5	195.7	191.9	2470	64.92
12/4/2020 15:39	198.5	196.2	191.8	2467	64.99
12/4/2020 15:40	198.5	196.3	192.1	2466	64.96

12/4/2020 15:40	198.5	195.6	192.7	2470	65.01
12/4/2020 15:40	198.5	196	192.8	2470	65.04
12/4/2020 15:40	198.5	195.9	191.9	2471	64.8
12/4/2020 15:40	198.5	196	192.2	2476	65.08
12/4/2020 15:40	198.5	196.4	192.4	2478	64.93
12/4/2020 15:40	198.5	195.7	192.1	2476	64.88
12/4/2020 15:40	198.5	196.1	192.1	2473	65.04
12/4/2020 15:40	198.5	195.6	191.7	2470	65.04
12/4/2020 15:40	198.5	195.9	191.7	2468	64.92
12/4/2020 15:40	198.5	196.3	192.1	2465	64.92
12/4/2020 15:40	198.5	195.7	192.5	2463	65.04
12/4/2020 15:40	198.5	196	193	2466	64.92
12/4/2020 15:40	198.5	196	192.8	2465	64.96
12/4/2020 15:40	198.5	195.7	192.3	2463	65.04
12/4/2020 15:40	198.5	195.9	191.8	2461	64.92
12/4/2020 15:40	198.6	195.9	191.7	2456	65.04
12/4/2020 15:40	198.6	196.1	191.9	2459	65
12/4/2020 15:40	198.6	195.9	192.4	2463	64.92
12/4/2020 15:40	198.6	195.5	192.2	2462	65
12/4/2020 15:40	198.5	195.7	192.5	2459	65
12/4/2020 15:40	198.5	195.9	192.3	2458	64.92
12/4/2020 15:40	198.5	196.3	192.3	2458	64.88
12/4/2020 15:40	198.5	196.1	192.4	2459	65.08
12/4/2020 15:40	198.6	195.7	192.4	2454	64.96
12/4/2020 15:40	198.6	195.7	192.5	2454	65.08
12/4/2020 15:40	198.5	195.9	192.3	2458	65.08
12/4/2020 15:40	198.5	196.4	192.3	2459	64.96
12/4/2020 15:40	198.5	195.7	192.4	2462	64.88
12/4/2020 15:40	198.5	195.8	192.5	2462	65.04
12/4/2020 15:40	198.5	196.2	192.6	2460	65.12
12/4/2020 15:40	198.5	195.7	192.5	2460	64.92
12/4/2020 15:40	198.6	196.1	192.2	2463	64.92
12/4/2020 15:40	198.5	195.9	192.4	2467	65.08
12/4/2020 15:40	198.5	196.1	192.3	2466	65
12/4/2020 15:40	198.5	196.2	192.1	2468	65.04
12/4/2020 15:40	198.5	195.7	192.3	2467	65.04
12/4/2020 15:40	198.5	196.1	192.1	2462	64.92
12/4/2020 15:40	198.5	195.7	192.4	2459	65.04
12/4/2020 15:40	198.6	195.7	192.5	2462	65
12/4/2020 15:40	198.5	196.4	192.6	2463	65
12/4/2020 15:40	198.5	195.8	193	2457	65
12/4/2020 15:40	198.5	196.4	192.7	2458	65
12/4/2020 15:40	198.5	195.7	192.7	2458	64.96
12/4/2020 15:40	198.5	195.7	192.2	2458	65.04
12/4/2020 15:40	198.5	196.4	191.8	2453	65.08
12/4/2020 15:40	198.5	195.9	192.1	2449	65.04
12/4/2020 15:40	198.6	196.1	192.1	2453	64.92

12/4/2020 15:40	198.5	196.3	192.2	2456	65.12
12/4/2020 15:40	198.5	196.2	192.5	2457	65.04
12/4/2020 15:40	198.5	196.4	192.5	2457	65.12
12/4/2020 15:40	198.6	195.7	192.2	2457	64.92
12/4/2020 15:40	198.5	196.1	192.4	2462	65.08
12/4/2020 15:40	198.6	196.2	192.3	2465	64.96
12/4/2020 15:40	198.5	195.9	193.1	2464	65.04
12/4/2020 15:40	198.5	196.4	193.1	2464	65.08
12/4/2020 15:40	198.6	195.9	192	2465	64.92
12/4/2020 15:40	198.6	196.3	191.9	2466	65
12/4/2020 15:40	198.6	196.2	192.1	2469	65.05
12/4/2020 15:41	198.6	195.9	192.3	2463	64.96
12/4/2020 15:41	198.6	196.3	192.7	2461	64.96
12/4/2020 15:41	198.6	195.7	192.4	2464	65
12/4/2020 15:41	198.6	196.2	192.5	2459	65
12/4/2020 15:41	198.6	196.2	192.5	2464	65
12/4/2020 15:41	198.6	195.6	192.2	2464	65.04
12/4/2020 15:41	198.6	196.4	192.6	2467	65
12/4/2020 15:41	198.6	196	193.1	2465	65.04
12/4/2020 15:41	198.6	196.4	193.3	2465	65.01
12/4/2020 15:41	198.6	195.7	193.1	2465	64.99
12/4/2020 15:41	198.7	195.7	192.9	2462	65
12/4/2020 15:41	198.6	196.2	192.6	2461	65.08
12/4/2020 15:41	198.6	196.1	193.1	2459	65.12
12/4/2020 15:41	198.6	195.9	192.5	2460	65.08
12/4/2020 15:41	198.7	196.2	192.8	2459	64.92
12/4/2020 15:41	198.7	195.6	192.9	2461	64.97
12/4/2020 15:41	198.7	196.2	192.8	2460	65.08
12/4/2020 15:41	198.7	195.6	193	2461	64.99
12/4/2020 15:41	198.6	196.2	192.9	2464	65.05
12/4/2020 15:41	198.7	196.2	193.1	2462	64.96
12/4/2020 15:41	198.7	196.4	193	2466	64.96
12/4/2020 15:41	198.7	196.4	192.9	2472	65
12/4/2020 15:41	198.6	195.6	192.7	2466	64.95
12/4/2020 15:41	198.7	196.1	192.4	2466	64.96
12/4/2020 15:41	198.7	196.2	192.1	2466	65.08
12/4/2020 15:41	198.7	195.6	192.3	2467	65
12/4/2020 15:41	198.6	195.9	192.5	2464	64.96
12/4/2020 15:41	198.7	195.8	192.8	2464	65.13
12/4/2020 15:41	198.7	196.4	192.4	2464	64.96
12/4/2020 15:41	198.6	196.2	192.4	2463	64.96
12/4/2020 15:41	198.7	195.7	192.5	2459	65
12/4/2020 15:41	198.7	196.1	192.2	2466	65
12/4/2020 15:41	198.7	195.8	192.1	2459	65.04
12/4/2020 15:41	198.7	196.3	192.1	2466	64.96
12/4/2020 15:41	198.7	195.7	193.3	2468	64.88
12/4/2020 15:41	198.7	195.6	192.8	2467	64.92

12/4/2020 15:41	198.7	196.1	192.2	2469	64.88
12/4/2020 15:41	198.7	195.6	192.6	2469	65.01
12/4/2020 15:41	198.7	196.2	192.6	2463	64.88
12/4/2020 15:41	198.7	196.4	192.2	2459	64.92
12/4/2020 15:41	198.7	195.8	192.2	2465	64.96
12/4/2020 15:41	198.7	196.2	192.9	2462	64.88
12/4/2020 15:41	198.7	195.7	193.4	2462	64.93
12/4/2020 15:41	198.7	196.4	192.6	2466	65.04
12/4/2020 15:41	198.7	196.2	192.6	2467	64.99
12/4/2020 15:41	198.7	195.7	192.6	2467	65.01
12/4/2020 15:41	198.7	196.2	192.7	2470	65
12/4/2020 15:41	198.7	195.9	192.2	2469	65.04
12/4/2020 15:41	198.8	195.7	192.4	2467	64.92
12/4/2020 15:41	198.8	195.7	192.9	2463	64.96
12/4/2020 15:41	198.7	196.1	193.3	2466	64.96
12/4/2020 15:41	198.7	196.4	192.7	2463	64.96
12/4/2020 15:41	198.7	196	192.4	2460	64.96
12/4/2020 15:41	198.7	196.4	192.9	2459	65
12/4/2020 15:41	198.7	196.4	192.8	2463	64.96
12/4/2020 15:41	198.8	195.6	192.4	2467	64.96
12/4/2020 15:41	198.7	196	192.2	2470	65.08
12/4/2020 15:41	198.7	195.9	192.2	2473	64.96
12/4/2020 15:41	198.7	196.2	193	2470	65
12/4/2020 15:41	198.7	196.3	193.5	2469	64.96
12/4/2020 15:42	198.7	195.8	193.1	2465	64.88
12/4/2020 15:42	198.7	196.1	192.6	2462	65.16
12/4/2020 15:42	198.7	196.1	192.5	2465	64.99
12/4/2020 15:42	198.7	196.3	192.6	2466	65.04
12/4/2020 15:42	198.7	196.1	192.8	2463	65.08
12/4/2020 15:42	198.7	195.8	192.6	2463	65.03
12/4/2020 15:42	198.8	196.1	192.1	2469	65.04
12/4/2020 15:42	198.8	196.2	192.3	2467	65.03
12/4/2020 15:42	198.8	196.4	193.1	2467	65.08
12/4/2020 15:42	198.7	196.4	193.2	2470	65.08
12/4/2020 15:42	198.7	195.6	192.5	2466	64.87
12/4/2020 15:42	198.7	195.9	192.4	2471	64.96
12/4/2020 15:42	198.7	195.7	192.8	2469	65.08
12/4/2020 15:42	198.8	196.2	192.5	2468	65
12/4/2020 15:42	198.7	196.3	192.3	2468	65.04
12/4/2020 15:42	198.8	195.6	192.5	2470	65.04
12/4/2020 15:42	198.7	195.9	192.1	2470	64.92
12/4/2020 15:42	198.7	195.8	192.8	2470	64.92
12/4/2020 15:42	198.7	195.7	193.1	2469	65
12/4/2020 15:42	198.8	196.3	193.1	2466	65.04
12/4/2020 15:42	198.7	195.7	193.5	2461	64.96
12/4/2020 15:42	198.7	196	193.3	2465	64.99
12/4/2020 15:42	198.8	195.7	193.6	2470	65.05

12/4/2020 15:42	198.8	196.4	193.3	2467	64.99
12/4/2020 15:42	198.8	196.2	193.3	2462	64.88
12/4/2020 15:42	198.7	195.8	193.4	2461	64.93
12/4/2020 15:42	198.8	195.9	193.3	2459	64.92
12/4/2020 15:42	198.7	196.1	193	2464	64.99
12/4/2020 15:42	198.8	196.1	192.5	2469	64.96
12/4/2020 15:42	198.7	196.4	192.3	2467	65.04
12/4/2020 15:42	198.8	195.9	192.5	2467	65.08
12/4/2020 15:42	198.8	196.4	192.4	2473	65.12
12/4/2020 15:42	198.8	195.9	192.6	2471	65.16
12/4/2020 15:42	198.8	195.7	192.8	2471	64.92
12/4/2020 15:42	198.7	196.2	192.8	2469	64.96
12/4/2020 15:42	198.8	195.8	192.6	2473	65.04
12/4/2020 15:42	198.8	196.3	193.4	2470	64.88
12/4/2020 15:42	198.7	196.3	193.1	2473	64.92
12/4/2020 15:42	198.7	195.7	192.4	2469	65.04
12/4/2020 15:42	198.8	196.4	192.8	2467	64.92
12/4/2020 15:42	198.8	196	192.6	2463	65.04
12/4/2020 15:42	198.8	196.1	192.7	2467	65
12/4/2020 15:42	198.8	196.2	193.3	2463	64.92
12/4/2020 15:42	198.8	195.7	193.1	2462	65
12/4/2020 15:42	198.8	196.3	192.5	2467	65
12/4/2020 15:42	198.8	196	192.5	2466	64.99
12/4/2020 15:42	198.8	196.1	192.9	2463	65.04
12/4/2020 15:42	198.8	196.2	192.8	2467	65.04
12/4/2020 15:42	198.9	195.7	192.4	2468	64.88
12/4/2020 15:42	198.8	195.9	193.1	2473	64.92
12/4/2020 15:42	198.8	195.6	193	2476	64.96
12/4/2020 15:42	198.8	196.1	192.4	2473	65.04
12/4/2020 15:42	198.8	195.7	192.6	2474	65.04
12/4/2020 15:42	198.9	195.7	192.7	2478	65.08
12/4/2020 15:42	198.8	196.4	192.8	2473	64.88
12/4/2020 15:42	198.8	195.7	192.3	2469	64.96
12/4/2020 15:42	198.8	196.4	193.6	2469	65.04
12/4/2020 15:42	198.8	195.9	193	2471	64.92
12/4/2020 15:42	198.8	195.8	192.2	2475	65.04
12/4/2020 15:43	198.8	196.2	192.6	2476	65
12/4/2020 15:43	198.9	195.9	192.6	2475	65
12/4/2020 15:43	198.8	195.9	192.6	2469	65.04
12/4/2020 15:43	198.9	195.6	192.8	2467	64.96
12/4/2020 15:43	198.9	195.7	193.5	2468	64.96
12/4/2020 15:43	198.8	196.4	193.1	2470	64.92
12/4/2020 15:43	198.9	195.7	192.5	2470	64.92
12/4/2020 15:43	198.9	196.4	192.4	2473	65.04
12/4/2020 15:43	198.9	196.2	192.8	2469	64.96
12/4/2020 15:43	198.9	195.7	192.8	2469	65
12/4/2020 15:43	198.9	195.8	192.8	2466	65

12/4/2020 15:43	198.9	196	192.8	2464	64.99
12/4/2020 15:43	198.9	195.9	193.5	2466	64.96
12/4/2020 15:43	198.9	196.4	192.9	2470	64.96
12/4/2020 15:43	198.9	196.1	192.5	2470	65.08
12/4/2020 15:43	198.9	196.1	192.5	2470	64.96
12/4/2020 15:43	198.9	195.8	193.1	2465	64.93
12/4/2020 15:43	198.9	195.9	192.9	2469	65.04
12/4/2020 15:43	198.8	196.3	192.6	2471	65.04
12/4/2020 15:43	198.9	195.7	192.6	2468	64.88
12/4/2020 15:43	198.9	195.9	193	2470	65.08
12/4/2020 15:43	198.9	195.7	193.8	2470	65.04
12/4/2020 15:43	198.9	195.9	193.4	2467	64.88
12/4/2020 15:43	198.9	196.4	192.4	2473	65.12
12/4/2020 15:43	198.9	195.7	192.5	2469	64.99
12/4/2020 15:43	198.9	196.1	192.8	2473	65
12/4/2020 15:43	198.9	195.6	193.1	2479	64.96
12/4/2020 15:43	198.9	196.2	192.9	2477	64.96
12/4/2020 15:43	198.9	196.4	192.6	2480	65
12/4/2020 15:43	198.9	196.2	193.4	2479	65.04
12/4/2020 15:43	198.9	196.3	193.8	2477	65
12/4/2020 15:43	199	195.9	192.9	2473	65
12/4/2020 15:43	198.8	195.7	192.6	2471	64.96
12/4/2020 15:43	198.9	195.7	192.4	2473	64.96
12/4/2020 15:43	198.9	195.7	193.1	2468	64.92
12/4/2020 15:43	198.9	196.2	193.3	2466	65
12/4/2020 15:43	198.9	196.3	192.9	2466	65.04
12/4/2020 15:43	198.9	195.7	192.8	2463	64.92
12/4/2020 15:43	198.9	196.2	192.9	2464	64.96
12/4/2020 15:43	198.9	195.9	193.5	2467	65.04
12/4/2020 15:43	198.9	196.4	193.8	2465	64.88
12/4/2020 15:43	198.8	196.4	193.2	2466	64.99
12/4/2020 15:43	198.9	196.2	192.7	2469	64.92
12/4/2020 15:43	198.9	196	192.4	2463	64.83
12/4/2020 15:43	198.9	195.7	192.6	2461	64.99
12/4/2020 15:43	198.9	196.1	193.1	2466	65.12
12/4/2020 15:43	199	196.2	193	2469	64.92
12/4/2020 15:43	199	196.2	192.8	2467	64.88
12/4/2020 15:43	198.9	196.2	193.1	2475	64.92
12/4/2020 15:43	198.9	195.8	193.5	2475	64.92
12/4/2020 15:43	199	195.9	193	2472	64.84
12/4/2020 15:43	199	195.8	192.5	2466	65.08
12/4/2020 15:43	199	195.6	192.5	2467	65.08
12/4/2020 15:43	198.9	196.2	193.1	2466	64.84
12/4/2020 15:43	199	196	192.9	2463	65.08
12/4/2020 15:43	198.9	196.4	192.9	2464	64.96
12/4/2020 15:43	198.9	196	193.1	2466	64.92
12/4/2020 15:43	199	195.7	193.5	2470	64.96

12/4/2020 15:43	198.9	196.4	193.4	2470	65.04
12/4/2020 15:43	198.9	195.7	192.4	2469	64.92
12/4/2020 15:44	198.9	196.1	192.5	2467	64.96
12/4/2020 15:44	198.9	196.2	192.9	2466	65.04
12/4/2020 15:44	198.9	195.8	193	2469	64.96
12/4/2020 15:44	199	196.3	192.9	2463	64.92
12/4/2020 15:44	198.9	196	193.1	2464	65.04
12/4/2020 15:44	198.9	195.9	192.8	2464	65.04
12/4/2020 15:44	198.9	196.2	192.9	2463	64.83
12/4/2020 15:44	199	196.2	192.7	2463	65
12/4/2020 15:44	198.9	195.9	193.2	2464	64.96
12/4/2020 15:44	199	195.9	193.2	2467	64.96
12/4/2020 15:44	199	196	192.8	2467	64.92
12/4/2020 15:44	198.9	196.4	192.8	2467	65
12/4/2020 15:44	199	195.7	193	2467	64.96
12/4/2020 15:44	198.9	196.1	192.9	2471	64.96
12/4/2020 15:44	199	195.7	192.9	2471	65.04
12/4/2020 15:44	198.9	196	193.1	2467	65.04
12/4/2020 15:44	199	196.4	193	2471	64.92
12/4/2020 15:44	198.9	196.2	192.8	2470	64.87
12/4/2020 15:44	198.9	196.2	192.8	2470	65.04
12/4/2020 15:44	199	195.8	192.8	2470	64.95
12/4/2020 15:44	199	196	192.9	2470	64.99
12/4/2020 15:44	198.9	196.4	193.8	2471	65.12
12/4/2020 15:44	199	195.7	193.3	2470	65.03
12/4/2020 15:44	198.9	196.1	192.8	2469	64.88
12/4/2020 15:44	199	195.9	193.1	2473	65
12/4/2020 15:44	199	195.8	193.2	2474	64.88
12/4/2020 15:44	199	195.7	193.1	2474	65.08
12/4/2020 15:44	199	195.6	192.8	2470	64.88
12/4/2020 15:44	199	196.4	193	2470	65.08
12/4/2020 15:44	199.1	196.1	193.7	2469	64.95
12/4/2020 15:44	199	195.7	193.3	2467	65.08
12/4/2020 15:44	199	196.2	192.6	2472	64.93
12/4/2020 15:44	199	196	193.2	2474	64.8
12/4/2020 15:44	199	196.3	193.1	2471	64.8
12/4/2020 15:44	199	195.9	192.6	2471	65.08
12/4/2020 15:44	199	195.9	192.9	2469	64.99
12/4/2020 15:44	199.1	196.4	193.6	2466	65.04
12/4/2020 15:44	199.1	196.1	193.8	2469	64.93
12/4/2020 15:44	199.1	196.2	192.8	2465	64.92
12/4/2020 15:44	199.1	197.1	192.8	2466	64.92
12/4/2020 15:44	199.1	197.1	193.1	2468	65.04
12/4/2020 15:44	199.1	197.2	193.3	2470	64.92
12/4/2020 15:44	199	196.9	193	2470	64.91
12/4/2020 15:44	199.1	197.1	192.6	2469	64.96
12/4/2020 15:44	199	197.1	192.5	2467	65

12/4/2020 15:44	199.1	197	193.2	2467	64.91
12/4/2020 15:44	199	197.2	193.8	2467	64.96
12/4/2020 15:44	199	197.1	193.8	2467	65.04
12/4/2020 15:44	199.1	196.9	193.4	2464	64.96
12/4/2020 15:44	199.1	196.9	192.9	2467	64.96
12/4/2020 15:44	199.1	197	192.7	2467	65.04
12/4/2020 15:44	199.1	197.1	192.8	2471	64.96
12/4/2020 15:44	199.1	196.8	193.1	2473	64.92
12/4/2020 15:44	199.1	197.1	193.3	2473	65.04
12/4/2020 15:44	199.1	197.2	193.3	2469	65
12/4/2020 15:44	199.1	197.2	193.1	2467	65
12/4/2020 15:44	199.1	197.1	193.1	2476	65
12/4/2020 15:44	199.1	196.8	193.1	2473	65
12/4/2020 15:44	199.1	197	192.9	2472	64.92
12/4/2020 15:45	199.1	197	193.1	2467	65.04
12/4/2020 15:45	199.1	197	193	2467	64.83
12/4/2020 15:45	199.1	197.1	192.9	2466	65
12/4/2020 15:45	199.1	196.9	192.9	2472	65.04
12/4/2020 15:45	199.2	197.1	193	2469	65
12/4/2020 15:45	199.1	197.1	192.9	2466	64.99
12/4/2020 15:45	199.1	197	193.1	2464	64.92
12/4/2020 15:45	199.1	196.8	193.3	2466	64.96
12/4/2020 15:45	199.1	197.3	192.8	2467	64.99
12/4/2020 15:45	199.1	197.1	193	2470	64.99
12/4/2020 15:45	199.1	197.1	193.3	2470	65.08
12/4/2020 15:45	199.1	197.3	193.6	2470	64.99
12/4/2020 15:45	199.2	197	194.2	2470	64.99
12/4/2020 15:45	199.1	197	193.4	2470	64.96
12/4/2020 15:45	199.1	197.1	192.7	2471	64.99
12/4/2020 15:45	199.2	196.9	193.1	2474	64.84
12/4/2020 15:45	199.1	197.1	193.1	2475	64.92
12/4/2020 15:45	199.1	197.2	193.3	2470	64.83
12/4/2020 15:45	199.1	197	193.3	2468	64.88
12/4/2020 15:45	199.2	197.1	193.1	2471	65.08
12/4/2020 15:45	199.2	197.1	194	2476	64.88
12/4/2020 15:45	199.2	197.1	192.9	2477	65.08
12/4/2020 15:45	199.2	197.1	192.7	2475	64.99
12/4/2020 15:45	199.2	196.9	193.3	2478	64.92
12/4/2020 15:45	199.3	197.1	193.1	2476	64.88
12/4/2020 15:45	199.2	196.9	192.9	2473	64.84
12/4/2020 15:45	199.2	196.9	194.1	2470	64.88
12/4/2020 15:45	199.2	197.2	193.2	2467	64.95
12/4/2020 15:45	199.3	197.2	193	2470	64.99
12/4/2020 15:45	199.3	197.1	192.9	2474	64.88
12/4/2020 15:45	199.2	197	193.2	2473	64.88
12/4/2020 15:45	199.3	197	193.3	2469	64.88
12/4/2020 15:45	199.3	197.3	192.9	2471	64.96

12/4/2020 15:45	199.2	196.8	193.8	2471	64.96
12/4/2020 15:45	199.3	197.1	194	2471	64.87
12/4/2020 15:45	199.3	197.2	192.7	2471	64.99
12/4/2020 15:45	199.2	197	193	2469	64.92
12/4/2020 15:45	199.2	197.1	193.2	2469	64.99
12/4/2020 15:45	199.2	197.1	193.4	2474	64.92
12/4/2020 15:45	199.3	197.1	193.3	2473	65.04
12/4/2020 15:45	199.3	196.9	193.1	2476	64.83
12/4/2020 15:45	199.2	197.2	194.2	2479	64.88
12/4/2020 15:45	199.2	197.3	193.1	2480	64.92
12/4/2020 15:45	199.3	196.9	192.6	2474	64.88
12/4/2020 15:45	199.2	196.9	193.4	2470	64.99
12/4/2020 15:45	199.3	196.9	193.1	2470	65.04
12/4/2020 15:45	199.2	197	193.1	2471	64.83
12/4/2020 15:45	199.2	197.2	193.9	2469	64.92
12/4/2020 15:45	199.3	197	193.5	2473	65.04
12/4/2020 15:45	199.3	197.1	193	2477	65
12/4/2020 15:45	199.2	197	193.1	2477	65.04
12/4/2020 15:45	199.3	197.1	193.3	2478	64.96
12/4/2020 15:45	199.3	197.2	193.2	2476	64.96
12/4/2020 15:45	199.2	197.1	193.2	2476	65
12/4/2020 15:45	199.2	197.2	194.1	2476	64.96
12/4/2020 15:45	199.3	197.1	192.9	2473	64.96
12/4/2020 15:45	199.2	196.9	192.7	2472	64.87
12/4/2020 15:45	199.3	197.2	193.4	2469	64.92
12/4/2020 15:45	199.3	197	193.1	2471	65
12/4/2020 15:46	199.3	197	193	2467	64.87
12/4/2020 15:46	199.3	197.1	193.5	2467	64.96
12/4/2020 15:46	199.3	197.1	194.2	2471	64.96
12/4/2020 15:46	199.3	197.2	193.1	2471	64.92
12/4/2020 15:46	199.3	196.9	193.1	2475	64.88
12/4/2020 15:46	199.2	197.1	193.3	2476	65
12/4/2020 15:46	199.3	197.1	193.5	2481	64.91
12/4/2020 15:46	199.3	197.2	193.2	2481	64.96
12/4/2020 15:46	199.3	197.1	193.5	2478	65
12/4/2020 15:46	199.3	197	194.2	2476	64.96
12/4/2020 15:46	199.3	197.1	193	2478	64.99
12/4/2020 15:46	199.3	197.2	193	2480	64.95
12/4/2020 15:46	199.3	197.2	193	2478	65.04
12/4/2020 15:46	199.3	197.2	193.3	2476	64.92
12/4/2020 15:46	199.3	197.1	193.3	2476	64.96
12/4/2020 15:46	199.3	197	193.1	2479	65.12
12/4/2020 15:46	199.3	197.2	193.5	2481	64.83
12/4/2020 15:46	199.4	196.9	194.2	2477	64.88
12/4/2020 15:46	199.3	197.2	193.6	2476	65
12/4/2020 15:46	199.3	197	193.1	2473	64.88
12/4/2020 15:46	199.3	197	193	2475	64.96

12/4/2020 15:46	199.3	197.2	193	2480	64.88
12/4/2020 15:46	199.3	196.9	193.6	2480	65.12
12/4/2020 15:46	199.3	197.2	193.3	2477	64.99
12/4/2020 15:46	199.3	197	193.4	2474	64.88
12/4/2020 15:46	199.3	197	193.4	2473	64.88
12/4/2020 15:46	199.3	197.1	193	2474	65.03
12/4/2020 15:46	199.3	197	193.3	2474	64.88
12/4/2020 15:46	199.3	197.1	193.6	2477	64.96
12/4/2020 15:46	199.3	196.9	194	2474	64.92
12/4/2020 15:46	199.3	197.2	194.1	2474	65.04
12/4/2020 15:46	199.4	197	193.8	2473	64.92
12/4/2020 15:46	199.3	197.1	193.5	2474	64.99
12/4/2020 15:46	199.3	197.1	193.3	2478	65.08
12/4/2020 15:46	199.3	197.1	193.1	2484	65.12
12/4/2020 15:46	199.3	197.2	193.1	2488	64.88
12/4/2020 15:46	199.4	197.2	192.9	2490	64.88
12/4/2020 15:46	199.3	196.9	193	2490	65.12
12/4/2020 15:46	199.4	197	193.3	2488	65.04
12/4/2020 15:46	199.3	197.1	192.9	2489	64.92
12/4/2020 15:46	199.4	197.2	193.4	2488	65.04
12/4/2020 15:46	199.3	197.1	193.5	2491	64.92
12/4/2020 15:46	199.3	196.8	193.4	2484	65
12/4/2020 15:46	199.4	197.3	193.5	2478	64.92
12/4/2020 15:46	199.3	197.1	193.3	2478	65.04
12/4/2020 15:46	199.3	197.1	193.6	2485	64.87
12/4/2020 15:46	199.4	197.1	193.4	2485	64.96
12/4/2020 15:46	199.4	196.9	193.2	2487	64.96
12/4/2020 15:46	199.4	197.2	193.4	2484	64.92
12/4/2020 15:46	199.4	197.1	193.2	2478	65
12/4/2020 15:46	199.4	197.1	193.3	2484	65
12/4/2020 15:46	199.5	197.1	193.3	2483	64.92
12/4/2020 15:46	199.4	197	193.1	2478	65
12/4/2020 15:46	199.3	197.1	193.3	2481	65.04
12/4/2020 15:46	199.4	197.1	192.9	2480	65.04
12/4/2020 15:46	199.4	197.1	193.3	2483	64.99
12/4/2020 15:46	199.4	197.1	193.6	2482	64.88
12/4/2020 15:46	199.4	196.8	194	2482	65.08
12/4/2020 15:46	199.4	197.1	194.2	2483	64.88
12/4/2020 15:46	199.5	196.9	194.2	2481	64.92
12/4/2020 15:47	199.4	197	194.1	2481	65.12
12/4/2020 15:47	199.5	197.1	193.8	2482	64.92
12/4/2020 15:47	199.4	197.1	193.5	2483	65.04
12/4/2020 15:47	199.5	197.1	192.9	2485	65.12
12/4/2020 15:47	199.5	197.1	192.9	2482	64.92
12/4/2020 15:47	199.5	197.2	192.8	2484	64.93
12/4/2020 15:47	199.5	197.2	193.1	2482	64.92
12/4/2020 15:47	199.5	196.9	193.4	2481	64.92

12/4/2020 15:47	199.3	197.1	193.3	2481	64.92
12/4/2020 15:47	199.5	196.8	193.8	2482	64.92
12/4/2020 15:47	199.5	196.9	193.5	2484	64.92
12/4/2020 15:47	199.5	197.2	193.5	2480	64.96
12/4/2020 15:47	199.5	197.1	193.4	2483	65.08
12/4/2020 15:47	199.5	197.1	193.5	2484	64.88
12/4/2020 15:47	199.5	197	193.6	2481	65.04
12/4/2020 15:47	199.5	197	193.5	2480	65.04
12/4/2020 15:47	199.5	197	193.3	2479	64.92
12/4/2020 15:47	199.5	197	193.4	2478	65
12/4/2020 15:47	199.4	197.1	193.3	2481	65
12/4/2020 15:47	199.5	197.1	193.1	2482	65
12/4/2020 15:47	199.5	197	193.5	2482	65
12/4/2020 15:47	199.5	197	193.6	2485	64.96
12/4/2020 15:47	199.6	197.1	193.6	2488	65.04
12/4/2020 15:47	199.5	197.1	194.1	2488	64.96
12/4/2020 15:47	199.5	197.1	194	2491	65.08
12/4/2020 15:47	199.6	197	194.3	2491	65.08
12/4/2020 15:47	199.6	196.9	193.9	2488	64.99
12/4/2020 15:47	199.6	197	193.5	2487	65.04
12/4/2020 15:47	199.6	197	193.2	2486	65.05
12/4/2020 15:47	199.6	197.1	192.9	2485	64.95
12/4/2020 15:47	199.6	196.8	193.1	2483	65.03
12/4/2020 15:47	199.6	196.9	193.2	2480	64.93
12/4/2020 15:47	199.6	196.9	193.2	2481	65.03
12/4/2020 15:47	199.6	197.1	193.6	2478	64.99
12/4/2020 15:47	199.6	196.9	193.5	2486	65.12
12/4/2020 15:47	199.6	197.2	193.6	2488	64.99
12/4/2020 15:47	199.6	197.1	193.3	2488	65.08
12/4/2020 15:47	199.6	197	192.9	2489	64.93
12/4/2020 15:47	199.7	197.1	193.6	2488	65.04
12/4/2020 15:47	199.6	197.1	194.1	2491	64.88
12/4/2020 15:47	199.6	197.2	194.3	2489	65.08
12/4/2020 15:47	199.6	197.2	193.5	2491	64.96
12/4/2020 15:47	199.6	197.2	193.2	2494	64.96
12/4/2020 15:47	199.6	196.9	193.1	2489	65
12/4/2020 15:47	199.6	197.2	193.6	2488	65
12/4/2020 15:47	199.6	196.9	193.3	2489	65
12/4/2020 15:47	199.6	197.3	193.3	2484	65.04
12/4/2020 15:47	199.6	197.1	194.1	2490	65
12/4/2020 15:47	199.7	197.1	194.1	2489	65
12/4/2020 15:47	199.6	196.9	193.1	2483	64.96
12/4/2020 15:47	199.6	196.9	193.3	2484	65.04
12/4/2020 15:47	199.6	197.1	193.5	2488	64.95
12/4/2020 15:47	199.6	197.2	193.5	2495	65.04
12/4/2020 15:47	199.6	197	193.3	2501	65.01
12/4/2020 15:47	199.7	197.1	194.4	2498	64.88

12/4/2020 15:47	199.6	197.1	193.5	2497	64.92
12/4/2020 15:47	199.6	197.2	193.1	2496	65.04
12/4/2020 15:47	199.7	197.1	193.3	2489	65.08
12/4/2020 15:47	199.7	196.9	193.8	2492	65.08
12/4/2020 15:48	199.6	197.1	193.8	2491	65.12
12/4/2020 15:48	199.6	196.8	193.6	2495	65.03
12/4/2020 15:48	199.6	197.1	194.2	2496	65.04
12/4/2020 15:48	199.6	196.9	194	2490	65.12
12/4/2020 15:48	199.6	197.1	193.2	2489	65.08
12/4/2020 15:48	199.7	197.1	193.5	2490	65.08
12/4/2020 15:48	199.7	197	193.9	2489	65.04
12/4/2020 15:48	199.7	197.2	193.5	2493	65.08
12/4/2020 15:48	199.6	197.1	193.3	2490	65
12/4/2020 15:48	199.7	197.1	194.1	2490	64.96
12/4/2020 15:48	199.7	197.3	194.1	2488	65.04
12/4/2020 15:48	199.7	197	193.4	2491	65
12/4/2020 15:48	199.7	197.3	193.3	2489	64.96
12/4/2020 15:48	199.6	197.1	193.5	2488	64.96
12/4/2020 15:48	199.6	197.3	193.7	2488	64.92
12/4/2020 15:48	199.7	197	193.5	2490	65
12/4/2020 15:48	199.6	196.9	194	2489	65
12/4/2020 15:48	199.6	197.2	194.3	2487	65.04
12/4/2020 15:48	199.7	197.1	193.3	2486	65
12/4/2020 15:48	199.7	197.3	193.3	2482	64.96
12/4/2020 15:48	199.7	197.3	193.6	2481	64.96
12/4/2020 15:48	199.8	197.1	193.7	2482	64.99
12/4/2020 15:48	199.7	196.9	193.3	2480	65.04
12/4/2020 15:48	199.7	196.9	194	2480	64.99
12/4/2020 15:48	199.7	197	194.5	2484	65.08
12/4/2020 15:48	199.7	197.1	193.2	2486	64.92
12/4/2020 15:48	199.7	197	193	2478	65
12/4/2020 15:48	199.7	197	193.7	2478	64.84
12/4/2020 15:48	199.7	196.9	193.8	2479	64.96
12/4/2020 15:48	199.7	197.3	193.6	2484	65.08
12/4/2020 15:48	199.7	197.1	193.3	2485	64.84
12/4/2020 15:48	199.7	196.9	193.9	2487	65.04
12/4/2020 15:48	199.7	197.1	194.5	2489	64.92
12/4/2020 15:48	199.7	197.1	193.7	2482	64.92
12/4/2020 15:48	199.7	197.3	193.3	2484	64.92
12/4/2020 15:48	199.7	196.9	193.3	2486	64.84
12/4/2020 15:48	199.7	196.9	193.5	2484	64.83
12/4/2020 15:48	199.7	197.2	193.7	2483	65.03
12/4/2020 15:48	199.7	197	193.8	2486	65.12
12/4/2020 15:48	199.7	197	193.3	2482	64.88
12/4/2020 15:48	199.7	197.2	194	2484	65
12/4/2020 15:48	199.7	197	194.2	2491	65.08
12/4/2020 15:48	199.7	197.1	193.9	2487	64.92

12/4/2020 15:48	199.7	197.1	193.3	2488	65.04
12/4/2020 15:48	199.7	197.1	193.1	2485	65
12/4/2020 15:48	199.8	197.1	193.9	2487	64.96
12/4/2020 15:48	199.8	196.9	193.6	2484	64.96
12/4/2020 15:48	199.8	197.1	193.5	2480	65
12/4/2020 15:48	199.7	196.9	193.4	2482	64.96
12/4/2020 15:48	199.7	197.2	194.2	2482	64.96
12/4/2020 15:48	199.7	197.2	194.1	2482	64.92
12/4/2020 15:48	199.7	197.1	193.2	2483	65.04
12/4/2020 15:48	199.8	197.1	193.3	2479	64.91
12/4/2020 15:48	199.7	196.9	193.8	2479	65
12/4/2020 15:48	199.8	197.1	193.5	2487	65
12/4/2020 15:48	199.8	197	193.5	2482	64.95
12/4/2020 15:48	199.7	196.8	194.5	2476	65.04
12/4/2020 15:48	199.8	197.1	193.9	2476	65
12/4/2020 15:48	199.8	197.1	193.2	2480	64.92
12/4/2020 15:48	199.8	196.9	193.7	2475	64.92
12/4/2020 15:49	199.8	197.2	193.5	2476	65.08
12/4/2020 15:49	199.8	197.1	193.6	2479	64.96
12/4/2020 15:49	199.8	197.1	193.8	2475	64.96
12/4/2020 15:49	199.8	197	194.2	2478	65.16
12/4/2020 15:49	199.8	196.9	194.3	2476	64.92
12/4/2020 15:49	199.8	197.3	193.3	2479	65.08
12/4/2020 15:49	199.8	197.2	193.3	2476	65.04
12/4/2020 15:49	199.8	197	193.7	2474	65.04
12/4/2020 15:49	199.8	197	193.8	2476	64.99
12/4/2020 15:49	199.8	196.9	193.8	2473	65.03
12/4/2020 15:49	199.8	197.3	193.3	2478	64.92
12/4/2020 15:49	199.8	196.9	194	2478	65.08
12/4/2020 15:49	199.8	197.1	194.7	2476	64.88
12/4/2020 15:49	199.8	197.2	193.5	2478	64.88
12/4/2020 15:49	199.8	197.2	193.5	2478	64.96
12/4/2020 15:49	199.8	197.1	193.8	2476	65.04
12/4/2020 15:49	199.8	197	193.6	2478	64.96
12/4/2020 15:49	199.8	197	194	2474	64.96
12/4/2020 15:49	199.8	196.9	193.4	2473	64.92
12/4/2020 15:49	199.8	197.2	194	2473	65
12/4/2020 15:49	199.8	197.2	194.7	2471	64.96
12/4/2020 15:49	199.8	197	194	2472	64.96
12/4/2020 15:49	199.8	197.2	193.5	2482	65.05
12/4/2020 15:49	199.9	197.1	193.4	2480	64.92
12/4/2020 15:49	199.8	197.2	194	2479	65.04
12/4/2020 15:49	199.8	197.2	193.9	2480	65.04
12/4/2020 15:49	199.9	196.9	193.9	2478	64.92
12/4/2020 15:49	199.9	197.2	193.6	2480	65
12/4/2020 15:49	199.9	196.9	194.1	2484	65
12/4/2020 15:49	199.9	197.1	194.5	2479	64.83

12/4/2020 15:49	199.9	197.2	193.8	2477	65
12/4/2020 15:49	199.9	196.9	193.4	2478	64.96
12/4/2020 15:49	199.8	197.3	193.7	2478	64.96
12/4/2020 15:49	199.9	197.1	194	2478	64.92
12/4/2020 15:49	199.9	197	193.7	2475	65
12/4/2020 15:49	199.9	197.3	193.8	2476	64.92
12/4/2020 15:49	200	196.9	193.5	2476	64.83
12/4/2020 15:49	200	197.2	194.3	2479	65.04
12/4/2020 15:49	199.9	197	194.5	2480	64.92
12/4/2020 15:49	200	196.9	193.8	2482	64.83
12/4/2020 15:49	200	197.3	193.5	2483	64.88
12/4/2020 15:49	200	197.1	193.7	2482	65.04
12/4/2020 15:49	200	197.1	193.8	2481	64.8
12/4/2020 15:49	199.9	197.1	194	2478	64.83
12/4/2020 15:49	200	196.9	193.8	2485	65.12
12/4/2020 15:49	199.9	197.2	193.3	2484	64.84
12/4/2020 15:49	200	197	193.8	2479	64.96
12/4/2020 15:49	200	197.1	194.2	2474	64.96
12/4/2020 15:49	200	197.1	194.4	2472	64.84
12/4/2020 15:49	200	197	194.2	2473	64.99
12/4/2020 15:49	200	197.1	193.5	2474	64.92
12/4/2020 15:49	200	197	193.7	2474	64.92
12/4/2020 15:49	200	197.1	193.8	2475	64.88
12/4/2020 15:49	200	197	193.9	2474	64.96
12/4/2020 15:49	200	197.3	193.9	2473	64.83
12/4/2020 15:49	200	196.9	193.9	2475	64.88
12/4/2020 15:49	200.1	197	193.8	2477	65.01
12/4/2020 15:49	200	197.2	194	2482	64.96
12/4/2020 15:49	200	197.1	194.3	2481	64.92
12/4/2020 15:50	200	196.8	194.9	2484	65.08
12/4/2020 15:50	200	197.1	194.1	2485	64.96
12/4/2020 15:50	200	197	193.6	2482	64.88
12/4/2020 15:50	200	197.1	193.6	2482	65.04
12/4/2020 15:50	200	197.1	193.5	2482	65
12/4/2020 15:50	200	197.2	194	2480	64.83
12/4/2020 15:50	200	197.1	193.7	2480	65
12/4/2020 15:50	200.1	196.9	193.9	2480	65.04
12/4/2020 15:50	200	197.1	194.2	2479	64.96
12/4/2020 15:50	200	197.2	193.8	2475	65
12/4/2020 15:50	200	196.9	194	2477	65.04
12/4/2020 15:50	200	196.9	193.5	2477	65.04
12/4/2020 15:50	200	196.9	193.5	2475	64.96
12/4/2020 15:50	200	197.1	193.9	2476	65
12/4/2020 15:50	200	196.9	194	2482	64.92
12/4/2020 15:50	200.1	196.9	194.5	2478	64.96
12/4/2020 15:50	200.1	197.2	194.9	2478	64.96
12/4/2020 15:50	200.1	197	194.5	2479	64.92

12/4/2020 15:50	200.1	197.1	194.1	2478	65
12/4/2020 15:50	200	196.9	193.5	2482	64.96
12/4/2020 15:50	200.1	197.1	193.6	2483	64.92
12/4/2020 15:50	200	197.2	193.7	2482	64.88
12/4/2020 15:50	200.1	197	193.6	2481	64.96
12/4/2020 15:50	200	197.2	194	2486	65.04
12/4/2020 15:50	200.1	197.1	193.8	2483	64.88
12/4/2020 15:50	200.1	196.9	193.7	2483	64.92
12/4/2020 15:50	200	197.1	194.1	2485	64.96
12/4/2020 15:50	200	197	194.2	2482	64.84
12/4/2020 15:50	200	197.1	193.8	2482	65.04
12/4/2020 15:50	200	196.9	193.9	2483	65.08
12/4/2020 15:50	200.1	196.9	193.5	2484	64.92
12/4/2020 15:50	200	197.2	193.6	2484	65.04
12/4/2020 15:50	200.1	197	193.9	2485	64.96
12/4/2020 15:50	200	197.2	194	2481	65
12/4/2020 15:50	200.1	197.1	194.3	2478	64.96
12/4/2020 15:50	200	197.1	194.5	2482	64.96
12/4/2020 15:50	200	197.1	194.7	2479	65.08
12/4/2020 15:50	200.1	197.1	194.7	2474	64.96
12/4/2020 15:50	200.1	197.1	194	2478	65
12/4/2020 15:50	200.1	197.2	193.6	2479	65
12/4/2020 15:50	200.1	197.2	193.5	2476	65.04
12/4/2020 15:50	200	197	193.6	2482	65
12/4/2020 15:50	200	197.1	194	2482	65
12/4/2020 15:50	200.1	197.1	194	2484	64.92
12/4/2020 15:50	200	197.3	194	2485	65.04
12/4/2020 15:50	200.1	197.1	194.2	2487	65.05
12/4/2020 15:50	200.1	197.1	194.1	2485	64.88
12/4/2020 15:50	200	197.2	194.1	2484	65.08
12/4/2020 15:50	200.1	197.2	194.1	2483	64.96
12/4/2020 15:50	200	196.9	194.1	2482	65.03
12/4/2020 15:50	200.1	196.8	194	2484	65.04
12/4/2020 15:50	200.1	197.1	193.6	2485	65.12
12/4/2020 15:50	200.1	196.9	194.3	2488	64.96
12/4/2020 15:50	200.1	196.9	194.7	2487	65.08
12/4/2020 15:50	200.1	197.3	194.6	2491	65.04
12/4/2020 15:50	200.1	197.1	194.5	2490	64.96
12/4/2020 15:50	200.1	197.1	193.9	2490	65.04
12/4/2020 15:50	200.1	196.9	193.6	2489	65.08
12/4/2020 15:50	200.1	197.2	194	2491	65.08
12/4/2020 15:51	200.1	197.3	193.7	2491	64.96
12/4/2020 15:51	200.1	197.1	194.1	2491	65
12/4/2020 15:51	200	197	194.2	2486	65.05
12/4/2020 15:51	200.2	198	194.1	2487	64.96
12/4/2020 15:51	200.1	196.9	194.2	2488	65
12/4/2020 15:51	200.1	198.2	194.1	2488	65.05

12/4/2020 15:51	200.1	198.2	193.8	2484	64.88
12/4/2020 15:51	200.1	198	193.9	2482	65
12/4/2020 15:51	200.2	197.9	194	2489	64.96
12/4/2020 15:51	200.1	198	194.4	2489	64.92
12/4/2020 15:51	200.2	198	194.8	2489	65.08
12/4/2020 15:51	200.1	198.2	194.8	2489	65.05
12/4/2020 15:51	200.2	198.2	195	2489	65.12
12/4/2020 15:51	200.2	198	194.7	2487	64.93
12/4/2020 15:51	200.1	198.2	194.6	2486	64.84
12/4/2020 15:51	200.2	197.9	194.3	2488	65.12
12/4/2020 15:51	200.1	198.1	194	2489	65.04
12/4/2020 15:51	200.2	198	194	2488	65.08
12/4/2020 15:51	200.2	198.3	193.5	2493	64.96
12/4/2020 15:51	200.2	198	193.8	2489	65
12/4/2020 15:51	200.1	198	193.8	2490	65
12/4/2020 15:51	200.2	198.3	193.7	2496	65
12/4/2020 15:51	200.2	198	193.7	2494	64.96
12/4/2020 15:51	200.2	198.3	193.5	2488	65
12/4/2020 15:51	200.2	198.1	193.8	2488	65
12/4/2020 15:51	200.2	198	193.9	2488	65
12/4/2020 15:51	200.2	198.2	193.7	2490	64.96
12/4/2020 15:51	200.1	198.2	193.9	2492	65
12/4/2020 15:51	200.1	198.1	194	2484	64.99
12/4/2020 15:51	200.2	198.4	193.8	2484	64.96
12/4/2020 15:51	200.2	198	194	2484	65.04
12/4/2020 15:51	200.2	198.2	194	2491	65.04
12/4/2020 15:51	200.1	198.2	194	2487	64.96
12/4/2020 15:51	200.1	198.1	194.1	2484	65
12/4/2020 15:51	200.2	198.3	194.1	2488	64.92
12/4/2020 15:51	200.2	198.2	194.3	2484	64.92
12/4/2020 15:51	200.2	198.1	194.2	2485	65.12
12/4/2020 15:51	200.2	198	194.3	2487	64.88
12/4/2020 15:51	200.1	198	194.5	2484	64.92
12/4/2020 15:51	200.2	198.3	194.3	2484	65.12
12/4/2020 15:51	200.2	198.1	194.4	2485	65.04
12/4/2020 15:51	200.1	198	194.3	2488	64.96
12/4/2020 15:51	200.1	198	194.1	2488	65.05
12/4/2020 15:51	200.2	197.8	194.2	2488	65
12/4/2020 15:51	200.1	198	193.8	2482	65.04
12/4/2020 15:51	200.2	198.1	194.1	2481	65
12/4/2020 15:51	200.1	198.2	193.9	2485	65.05
12/4/2020 15:51	200.2	198.1	194	2486	64.96
12/4/2020 15:51	200.1	198	194.5	2488	64.96
12/4/2020 15:51	200.1	198	194.7	2487	65.04
12/4/2020 15:51	200.2	198.1	194.8	2485	65
12/4/2020 15:51	200.2	198.3	194.7	2482	64.92
12/4/2020 15:51	200.2	198.2	193.7	2482	64.96

12/4/2020 15:51	200.1	197.9	193.6	2485	64.99
12/4/2020 15:51	200.2	198.1	194.2	2483	65.04
12/4/2020 15:51	200.2	198.2	194.2	2485	64.93
12/4/2020 15:51	200.2	198.2	194.4	2482	65.04
12/4/2020 15:51	200.1	198.2	194.3	2487	64.92
12/4/2020 15:51	200.1	198	194.2	2488	65.01
12/4/2020 15:51	200.2	198	194.1	2488	64.88
12/4/2020 15:52	200.1	198.1	194.2	2488	64.96
12/4/2020 15:52	200.2	197.9	194.8	2485	65.05
12/4/2020 15:52	200.2	198.2	195.2	2485	65.04
12/4/2020 15:52	200.2	197.9	194.1	2480	65
12/4/2020 15:52	200.2	198.3	194	2478	64.96
12/4/2020 15:52	200.2	198.2	193.8	2486	65.04
12/4/2020 15:52	200.2	198.2	194.1	2484	65
12/4/2020 15:52	200.2	198.2	194.5	2484	65.09
12/4/2020 15:52	200.2	198	194.1	2484	65.05
12/4/2020 15:52	200.3	198	194.2	2480	65.04
12/4/2020 15:52	200.3	198.1	193.8	2479	64.96
12/4/2020 15:52	200.2	198	194.5	2480	65
12/4/2020 15:52	200.3	198.2	195.1	2484	64.92
12/4/2020 15:52	200.3	197.9	194.8	2484	65.04
12/4/2020 15:52	200.2	198	194.4	2484	64.96
12/4/2020 15:52	200.3	198.1	193.7	2487	64.84
12/4/2020 15:52	200.3	198.2	193.6	2484	64.93
12/4/2020 15:52	200.3	198.1	194	2487	64.96
12/4/2020 15:52	200.3	198.2	194.2	2488	64.92
12/4/2020 15:52	200.2	197.9	194.3	2486	65.08
12/4/2020 15:52	200.2	198	194.3	2490	64.96
12/4/2020 15:52	200.2	198.3	193.8	2490	65.16
12/4/2020 15:52	200.3	198	194.1	2489	65.12
12/4/2020 15:52	200.3	198.1	194.5	2488	65.01
12/4/2020 15:52	200.3	198.2	194.8	2486	65
12/4/2020 15:52	200.3	198	194.9	2487	64.96
12/4/2020 15:52	200.3	198	193.9	2488	64.96
12/4/2020 15:52	200.4	198.2	193.7	2492	65.05
12/4/2020 15:52	200.3	198	194.1	2486	64.92
12/4/2020 15:52	200.4	198.3	194	2487	65
12/4/2020 15:52	200.3	198.1	194.5	2492	65.04
12/4/2020 15:52	200.3	198.2	194.3	2490	65
12/4/2020 15:52	200.4	198.1	194.3	2487	64.92
12/4/2020 15:52	200.3	198	194.4	2491	65.04
12/4/2020 15:52	200.3	198.2	194.4	2490	64.92
12/4/2020 15:52	200.3	198	194.3	2491	65
12/4/2020 15:52	200.4	198.2	194.1	2490	65.08
12/4/2020 15:52	200.4	198.1	193.9	2489	64.96
12/4/2020 15:52	200.4	197.8	194.2	2493	64.88
12/4/2020 15:52	200.3	198	194.1	2490	64.97

12/4/2020 15:52	200.4	198	194.1	2484	64.92
12/4/2020 15:52	200.4	198.2	194.1	2485	65.08
12/4/2020 15:52	200.4	198	194.2	2485	65
12/4/2020 15:52	200.4	198	194.7	2485	64.96
12/4/2020 15:52	200.4	198.2	195.2	2486	65.04
12/4/2020 15:52	200.4	198.2	195.4	2483	65.04
12/4/2020 15:52	200.4	198.3	195.2	2484	65
12/4/2020 15:52	200.4	198.1	194.5	2482	65
12/4/2020 15:52	200.4	198	194.3	2483	65
12/4/2020 15:52	200.4	198	193.8	2485	64.96
12/4/2020 15:52	200.4	197.9	193.7	2484	64.96
12/4/2020 15:52	200.4	198.2	194.1	2485	64.96
12/4/2020 15:52	200.5	198.1	194.3	2488	65
12/4/2020 15:52	200.5	198	194.5	2487	64.99
12/4/2020 15:52	200.5	198	194.3	2485	65.08
12/4/2020 15:52	200.5	198	194.2	2488	65.12
12/4/2020 15:52	200.5	198	194.3	2489	64.96
12/4/2020 15:52	200.4	198	194.2	2488	64.92
12/4/2020 15:52	200.5	198	194.1	2492	64.92
12/4/2020 15:53	200.5	198.1	194.2	2490	65.04
12/4/2020 15:53	200.5	198	194.3	2488	64.92
12/4/2020 15:53	200.5	198.1	194.9	2491	64.96
12/4/2020 15:53	200.5	198	195	2488	65.08
12/4/2020 15:53	200.5	198	194.9	2490	64.99
12/4/2020 15:53	200.5	198.2	195.1	2489	64.99
12/4/2020 15:53	200.5	198	194.8	2494	65.12
12/4/2020 15:53	200.5	198.2	194.6	2492	64.96
12/4/2020 15:53	200.5	198.1	194.7	2487	65
12/4/2020 15:53	200.5	198	194.2	2486	65.04
12/4/2020 15:53	200.5	198.3	194.2	2484	64.96
12/4/2020 15:53	200.5	198.1	194	2484	65.04
12/4/2020 15:53	200.6	198.2	194.2	2488	65.09
12/4/2020 15:53	200.6	198.1	194.2	2491	64.96
12/4/2020 15:53	200.5	198	194	2486	65
12/4/2020 15:53	200.6	198.3	194	2492	64.96
12/4/2020 15:53	200.6	197.9	194.5	2487	64.96
12/4/2020 15:53	200.6	198.2	194.3	2487	64.88
12/4/2020 15:53	200.7	198.2	194.5	2491	64.92
12/4/2020 15:53	200.6	198	194.6	2487	65.04
12/4/2020 15:53	200.6	198	194.8	2489	65.04
12/4/2020 15:53	200.6	198.2	194.7	2491	65.08
12/4/2020 15:53	200.5	198.1	195.2	2485	65.04
12/4/2020 15:53	200.7	198.1	195.4	2485	64.96
12/4/2020 15:53	200.6	198	195.2	2487	65.03
12/4/2020 15:53	200.7	198	195.2	2490	64.88
12/4/2020 15:53	200.6	197.9	195.2	2488	64.99
12/4/2020 15:53	200.7	198.2	194.6	2488	65.08

12/4/2020 15:53	200.7	198	194.2	2489	64.88
12/4/2020 15:53	200.6	198.2	194.2	2488	65.04
12/4/2020 15:53	200.7	198	194.2	2492	64.99
12/4/2020 15:53	200.6	198	194.6	2490	65.08
12/4/2020 15:53	200.6	198.3	194.6	2491	64.96
12/4/2020 15:53	200.6	198.2	194.3	2490	64.96
12/4/2020 15:53	200.6	198.4	194.5	2492	65.04
12/4/2020 15:53	200.7	198	194	2488	64.96
12/4/2020 15:53	200.7	197.9	194.1	2484	64.96
12/4/2020 15:53	200.7	198.4	194.4	2489	65.04
12/4/2020 15:53	200.7	198.3	194.9	2491	64.96
12/4/2020 15:53	200.7	198.3	195.2	2492	64.96
12/4/2020 15:53	200.6	198.1	194.2	2491	65.04
12/4/2020 15:53	200.6	198	194.2	2487	64.92
12/4/2020 15:53	200.7	198.2	194.6	2487	64.92
12/4/2020 15:53	200.7	198	194.5	2489	65
12/4/2020 15:53	200.7	198	194.5	2489	64.96
12/4/2020 15:53	200.7	198.2	194.1	2492	65.08
12/4/2020 15:53	200.7	197.9	194.2	2494	65.01
12/4/2020 15:53	200.7	198.3	194.6	2494	65.04
12/4/2020 15:53	200.7	198	195.2	2493	65.04
12/4/2020 15:53	200.7	198.2	195.2	2496	64.92
12/4/2020 15:53	200.7	198.1	194.8	2491	64.84
12/4/2020 15:53	200.7	197.9	194.2	2491	64.99
12/4/2020 15:53	200.7	198.1	194.4	2490	65.04
12/4/2020 15:53	200.7	198	194.5	2492	65.08
12/4/2020 15:53	200.8	198.2	194.5	2495	65.03
12/4/2020 15:53	200.7	198.3	194.6	2495	65.08
12/4/2020 15:53	200.7	198.2	194.5	2495	65
12/4/2020 15:53	200.8	198.3	194.6	2496	65.08
12/4/2020 15:53	200.8	198	194.4	2496	65.08
12/4/2020 15:53	200.8	197.9	194.1	2493	64.96
12/4/2020 15:54	200.8	198.2	194.3	2491	65.08
12/4/2020 15:54	200.8	197.8	194.1	2490	64.99
12/4/2020 15:54	200.8	198.1	194.2	2489	64.99
12/4/2020 15:54	200.8	198	194.3	2485	65.04
12/4/2020 15:54	200.7	198	194	2490	64.92
12/4/2020 15:54	200.8	198.2	194.1	2489	65.09
12/4/2020 15:54	200.8	198.1	194.3	2490	64.96
12/4/2020 15:54	200.8	198.2	194.4	2490	65
12/4/2020 15:54	200.7	198.1	194.7	2492	65
12/4/2020 15:54	200.9	198	194.5	2496	64.96
12/4/2020 15:54	200.8	198.1	194.9	2495	64.96
12/4/2020 15:54	200.8	198.2	194.5	2489	64.96
12/4/2020 15:54	200.8	198.2	194.6	2491	64.96
12/4/2020 15:54	200.8	198	194.5	2485	64.91
12/4/2020 15:54	200.8	198.1	194.5	2488	64.96

12/4/2020 15:54	200.7	198	195.4	2487	65.04
12/4/2020 15:54	200.8	198.1	195.1	2489	64.92
12/4/2020 15:54	200.9	198.2	194.2	2490	64.92
12/4/2020 15:54	200.9	198	194.5	2491	65.08
12/4/2020 15:54	200.9	198	194.8	2488	64.88
12/4/2020 15:54	200.8	198	194.5	2487	64.96
12/4/2020 15:54	200.8	198	194.6	2490	65.08
12/4/2020 15:54	200.8	198.2	195.5	2488	65.04
12/4/2020 15:54	200.8	198	194.6	2491	64.88
12/4/2020 15:54	200.9	198	194.3	2495	65.04
12/4/2020 15:54	200.9	198.3	194.7	2494	64.96
12/4/2020 15:54	200.8	198	194.7	2495	65.04
12/4/2020 15:54	200.8	198	194.3	2496	65.04
12/4/2020 15:54	200.8	198	195	2496	64.93
12/4/2020 15:54	200.8	198.1	195.4	2488	64.88
12/4/2020 15:54	200.9	198.2	194.2	2485	64.99
12/4/2020 15:54	200.8	198	194.3	2487	65.16
12/4/2020 15:54	200.8	198.2	194.5	2481	64.83
12/4/2020 15:54	200.8	198	194.3	2487	64.92
12/4/2020 15:54	200.8	197.9	194.3	2491	65.01
12/4/2020 15:54	200.8	198.2	195.2	2492	64.96
12/4/2020 15:54	200.8	198	194.9	2491	65
12/4/2020 15:54	200.8	198.1	194.4	2492	65
12/4/2020 15:54	200.8	198.1	194.4	2496	64.96
12/4/2020 15:54	200.8	198	194.8	2496	65
12/4/2020 15:54	200.8	198	194.4	2493	64.96
12/4/2020 15:54	200.8	198	194.5	2496	65.04
12/4/2020 15:54	200.8	198.3	195.7	2492	65.05
12/4/2020 15:54	200.8	198.1	194.5	2492	64.96
12/4/2020 15:54	200.8	197.8	194.3	2490	65.08
12/4/2020 15:54	200.8	198.2	194.8	2487	64.88
12/4/2020 15:54	200.8	198	194.8	2489	64.88
12/4/2020 15:54	200.8	198.2	194.5	2492	65
12/4/2020 15:54	200.9	198.1	195.2	2491	64.99
12/4/2020 15:54	200.9	198	195.1	2488	64.92
12/4/2020 15:54	200.9	198.3	194.4	2488	65.16
12/4/2020 15:54	200.8	198	194.5	2485	65.04
12/4/2020 15:54	200.8	198.4	195.1	2485	65.04
12/4/2020 15:54	200.8	198.1	194.7	2491	65.12
12/4/2020 15:54	200.8	198.2	194.5	2486	64.88
12/4/2020 15:54	200.8	198.3	195.5	2484	64.99
12/4/2020 15:54	200.8	197.9	194.5	2486	65.08
12/4/2020 15:54	200.8	198.2	194.3	2488	65.04
12/4/2020 15:54	200.9	198.1	194.7	2487	64.88
12/4/2020 15:55	200.8	198.3	194.8	2486	64.88
12/4/2020 15:55	200.9	198	194.5	2485	65.08
12/4/2020 15:55	200.9	198	194.7	2485	65.04

12/4/2020 15:55	200.8	198.2	195.5	2486	64.88
12/4/2020 15:55	200.9	198.1	194.8	2488	64.96
12/4/2020 15:55	200.8	198	194.1	2488	64.87
12/4/2020 15:55	200.9	198	194.8	2486	64.96
12/4/2020 15:55	200.9	198.2	194.3	2487	64.96
12/4/2020 15:55	200.9	198.3	194.8	2485	64.92
12/4/2020 15:55	200.8	198	194.6	2489	65
12/4/2020 15:55	200.8	198.3	195.1	2489	65.09
12/4/2020 15:55	200.8	198	195.5	2486	65
12/4/2020 15:55	200.8	198	194.7	2484	64.92
12/4/2020 15:55	200.9	198.2	194.3	2487	65
12/4/2020 15:55	200.9	198.2	194.9	2485	64.92
12/4/2020 15:55	200.9	197.9	195	2482	65
12/4/2020 15:55	200.9	198.2	194.8	2480	65.08
12/4/2020 15:55	200.8	198	194.7	2484	64.84
12/4/2020 15:55	200.8	198	194.5	2487	65.08
12/4/2020 15:55	200.9	198.1	195.2	2487	64.95
12/4/2020 15:55	200.9	198.1	195.7	2488	65.08
12/4/2020 15:55	200.9	198.2	194.8	2487	64.92
12/4/2020 15:55	200.9	198	194.5	2488	64.92
12/4/2020 15:55	200.9	198.1	194.5	2488	65.12
12/4/2020 15:55	200.9	198	194.9	2486	65.07
12/4/2020 15:55	200.9	198	195	2486	64.96
12/4/2020 15:55	200.9	198.3	194.7	2485	64.96
12/4/2020 15:55	200.8	197.8	194.8	2488	64.88
12/4/2020 15:55	200.9	198	194.4	2484	65.08
12/4/2020 15:55	200.9	198	194.8	2487	65.04
12/4/2020 15:55	200.9	198	195.7	2488	65.04
12/4/2020 15:55	200.9	198.3	195.5	2487	65.12
12/4/2020 15:55	200.9	198.2	194.9	2486	64.96
12/4/2020 15:55	200.9	198.2	194.6	2482	65.04
12/4/2020 15:55	200.9	198	194.3	2479	64.88
12/4/2020 15:55	200.9	198	194.8	2478	64.92
12/4/2020 15:55	200.8	198.3	194.9	2479	64.95
12/4/2020 15:55	200.9	198	194.9	2481	64.99
12/4/2020 15:55	200.9	198.3	194.8	2478	65.01
12/4/2020 15:55	200.9	198	194.5	2481	64.92
12/4/2020 15:55	200.9	198	194.7	2481	65
12/4/2020 15:55	200.9	198.2	194.7	2480	64.88
12/4/2020 15:55	200.8	198.1	195.2	2478	64.96
12/4/2020 15:55	200.9	198.2	195.6	2477	64.92
12/4/2020 15:55	200.9	198	195.4	2476	64.92
12/4/2020 15:55	200.8	198	195	2479	65
12/4/2020 15:55	200.8	198.3	194.5	2478	64.87
12/4/2020 15:55	200.9	198.2	194.3	2479	64.92
12/4/2020 15:55	200.9	197.9	194.5	2479	65
12/4/2020 15:55	200.9	198	194.7	2475	64.96

12/4/2020 15:55	200.9	198	194.6	2476	65.04
12/4/2020 15:55	200.9	198.3	195	2480	65
12/4/2020 15:55	200.9	198.2	194.9	2480	64.91
12/4/2020 15:55	200.9	198.2	194.9	2479	65
12/4/2020 15:55	200.9	198.2	194.8	2479	64.92
12/4/2020 15:55	200.9	198	194.6	2479	65.08
12/4/2020 15:55	200.9	198	194.5	2480	64.96
12/4/2020 15:55	200.9	198	194.5	2479	64.95
12/4/2020 15:55	200.9	198.2	194.9	2480	64.93
12/4/2020 15:56	200.9	198	195.4	2482	64.8
12/4/2020 15:56	200.8	197.8	195.5	2480	64.96
12/4/2020 15:56	200.9	198.2	195.6	2481	65.08
12/4/2020 15:56	200.8	198.1	195.1	2481	64.96
12/4/2020 15:56	200.8	198.3	194.8	2483	65.03
12/4/2020 15:56	200.9	198.1	194.6	2485	64.93
12/4/2020 15:56	200.8	198	194.3	2485	64.84
12/4/2020 15:56	200.8	198	194.7	2487	64.84
12/4/2020 15:56	200.9	198	194.9	2485	65.12
12/4/2020 15:56	200.9	198.2	194.8	2486	64.92
12/4/2020 15:56	200.9	198.2	194.9	2487	64.88
12/4/2020 15:56	200.9	197.9	194.5	2489	64.92
12/4/2020 15:56	200.9	198.2	195.2	2488	64.92
12/4/2020 15:56	200.9	198.1	195.5	2488	64.93
12/4/2020 15:56	200.9	198.3	194.3	2487	64.92
12/4/2020 15:56	200.8	198.3	194.6	2492	64.92
12/4/2020 15:56	200.9	197.9	195	2489	64.88
12/4/2020 15:56	200.8	198.2	194.8	2490	64.96
12/4/2020 15:56	200.8	197.9	194.8	2490	64.96
12/4/2020 15:56	200.9	198.2	195.7	2492	64.92
12/4/2020 15:56	200.9	198.2	194.9	2491	64.99
12/4/2020 15:56	200.8	197.8	194.7	2493	64.96
12/4/2020 15:56	200.8	198	195	2491	64.92
12/4/2020 15:56	200.8	198.2	194.9	2489	64.92
12/4/2020 15:56	200.9	198.2	194.7	2493	65.04
12/4/2020 15:56	200.8	198	194.8	2494	64.88
12/4/2020 15:56	200.9	198	195.7	2491	64.92
12/4/2020 15:56	200.9	198.2	194.9	2490	65
12/4/2020 15:56	200.9	198.1	194.6	2491	64.96
12/4/2020 15:56	200.9	198	194.9	2492	65
12/4/2020 15:56	200.9	198.2	195.2	2494	65.04
12/4/2020 15:56	200.9	198.3	194.9	2496	64.96
12/4/2020 15:56	200.9	198	194.5	2494	65
12/4/2020 15:56	200.9	198	195.7	2496	65.04
12/4/2020 15:56	200.8	198	195.4	2494	65
12/4/2020 15:56	200.9	198.2	194.5	2493	64.96
12/4/2020 15:56	200.9	198.2	195	2496	64.88
12/4/2020 15:56	200.9	198.2	194.9	2496	65.12

12/4/2020 15:56	200.9	198	194.6	2493	64.99
12/4/2020 15:56	200.9	198.2	195.2	2493	65.08
12/4/2020 15:56	201	198.2	195.6	2493	65.12
12/4/2020 15:56	200.9	198	194.7	2494	64.95
12/4/2020 15:56	201	198.3	194.9	2493	65.04
12/4/2020 15:56	200.9	198	194.9	2496	65.04
12/4/2020 15:56	200.9	198.2	195	2492	65
12/4/2020 15:56	200.9	198.2	194.9	2493	64.92
12/4/2020 15:56	200.9	198	194.8	2493	65.12
12/4/2020 15:56	200.9	198.2	194.8	2493	64.92
12/4/2020 15:56	201	198	195.2	2491	64.99
12/4/2020 15:56	201	198.1	195.6	2491	65.04
12/4/2020 15:56	201	198.2	195.5	2491	65.08
12/4/2020 15:56	200.9	198	194.7	2489	65
12/4/2020 15:56	200.9	198.2	194.7	2489	64.99
12/4/2020 15:56	200.9	198	194.5	2492	65.04
12/4/2020 15:56	201	198.2	194.5	2488	64.96
12/4/2020 15:56	200.9	198.2	195	2491	64.96
12/4/2020 15:56	201	198.2	194.9	2491	65.04
12/4/2020 15:56	201	198.2	195.1	2489	64.92
12/4/2020 15:56	200.9	198	195.1	2496	64.96
12/4/2020 15:56	201	198	195	2492	65
12/4/2020 15:57	200.9	198	194.9	2484	64.96
12/4/2020 15:57	201	198	194.9	2488	65.04
12/4/2020 15:57	200.9	198.1	195.1	2488	65
12/4/2020 15:57	200.9	197.9	195	2488	65
12/4/2020 15:57	201	198.1	194.9	2488	65
12/4/2020 15:57	201	198.2	194.9	2489	64.92
12/4/2020 15:57	201	197.8	194.8	2490	65.01
12/4/2020 15:57	201	198.2	194.8	2489	64.84
12/4/2020 15:57	201	198	194.6	2487	64.88
12/4/2020 15:57	201	198	194.4	2486	65.04
12/4/2020 15:57	200.9	198.3	194.7	2484	64.88
12/4/2020 15:57	201	198	195.7	2484	64.84
12/4/2020 15:57	201	198	195.7	2492	64.92
12/4/2020 15:57	201	198	195.3	2490	64.8
12/4/2020 15:57	200.9	198	194.6	2487	65.04
12/4/2020 15:57	200.9	198.1	194.9	2488	64.93
12/4/2020 15:57	201	198	195.2	2488	64.88
12/4/2020 15:57	201	198	195	2485	64.99
12/4/2020 15:57	201	198.1	194.8	2489	65.08
12/4/2020 15:57	201	198.1	194.7	2486	64.92
12/4/2020 15:57	201.1	198.2	195.2	2486	64.92
12/4/2020 15:57	201	198	195.7	2487	65.04
12/4/2020 15:57	200.9	198.2	195	2485	64.92
12/4/2020 15:57	201	198	194.8	2481	64.96
12/4/2020 15:57	201	198.1	195	2483	64.96

12/4/2020 15:57	201	198.2	194.7	2487	64.96
12/4/2020 15:57	201	198	195	2484	64.92
12/4/2020 15:57	200.9	198.3	194.7	2483	64.92
12/4/2020 15:57	200.9	198.1	194.3	2479	65.09
12/4/2020 15:57	201	198.2	195.5	2476	65
12/4/2020 15:57	201	198.2	195.5	2479	65.04
12/4/2020 15:57	200.9	197.9	194.9	2478	65
12/4/2020 15:57	201	198.3	194.9	2480	64.96
12/4/2020 15:57	201	198.2	194.9	2479	64.96
12/4/2020 15:57	201	198.2	195	2482	65
12/4/2020 15:57	201	198.2	195	2479	65.04
12/4/2020 15:57	201	198.3	194.8	2482	64.92
12/4/2020 15:57	201	198.2	194.5	2485	64.92
12/4/2020 15:57	201	198	195.4	2488	65.08
12/4/2020 15:57	201	198	195.8	2488	64.99
12/4/2020 15:57	201	198.2	194.9	2485	65.04
12/4/2020 15:57	201	198.2	194.8	2487	64.88
12/4/2020 15:57	201	198	195.2	2487	65.04
12/4/2020 15:57	201	198	194.9	2485	65.08
12/4/2020 15:57	201	198	194.9	2486	64.88
12/4/2020 15:57	201	198.3	194.8	2483	64.88
12/4/2020 15:57	201	198.1	194.9	2482	64.92
12/4/2020 15:57	201	198.1	195.9	2484	64.96
12/4/2020 15:57	201.1	198	195.5	2485	64.95
12/4/2020 15:57	201	198	194.9	2483	64.96
12/4/2020 15:57	201.1	198.2	194.9	2480	65.08
12/4/2020 15:57	201.1	198.1	194.9	2480	64.88
12/4/2020 15:57	201.1	198.2	195.2	2479	64.92
12/4/2020 15:57	201.1	198.2	195	2480	65.12
12/4/2020 15:57	201	198	195	2480	65.04
12/4/2020 15:57	201.1	198.2	195.2	2480	64.96
12/4/2020 15:57	201.1	198.8	194.9	2483	64.96
12/4/2020 15:57	201	199	194.7	2481	64.96
12/4/2020 15:57	201.1	198.9	194.6	2480	64.92
12/4/2020 15:58	201.1	199.4	194.9	2480	64.96
12/4/2020 15:58	201.1	199.4	194.9	2478	65
12/4/2020 15:58	201.1	199.3	195.4	2478	64.96
12/4/2020 15:58	201.1	198.9	195	2478	64.92
12/4/2020 15:58	201.1	199.4	195.5	2483	65
12/4/2020 15:58	201.1	197.8	195	2479	64.92
12/4/2020 15:58	201.1	199.2	195	2483	65.04
12/4/2020 15:58	201.1	199.3	194.9	2480	65
12/4/2020 15:58	201	199.2	194.5	2476	64.96
12/4/2020 15:58	201.1	199.5	194.8	2477	65
12/4/2020 15:58	201.1	198.8	194.7	2480	64.96
12/4/2020 15:58	201.1	199.1	195.2	2482	65.04
12/4/2020 15:58	201.1	198.8	195	2482	65.03

12/4/2020 15:58	201.1	199.5	195.2	2483	65
12/4/2020 15:58	201.1	199.5	195	2488	64.88
12/4/2020 15:58	201.1	198.7	194.9	2487	64.88
12/4/2020 15:58	201.1	199.2	195.8	2482	64.92
12/4/2020 15:58	201.1	199	195.1	2486	64.88
12/4/2020 15:58	201.1	199.4	194.7	2480	64.88
12/4/2020 15:58	201.1	199	195.4	2462	64.88
12/4/2020 15:58	201.1	198.7	195.2	2451	65.08
12/4/2020 15:58	201.1	199.5	194.9	2444	64.99
12/4/2020 15:58	201.2	198.9	195.3	2441	64.84
12/4/2020 15:58	201.1	199.6	195.7	2445	64.96
12/4/2020 15:58	201.1	199.4	194.8	2441	64.88
12/4/2020 15:58	201.2	198.7	194.8	2438	65.08
12/4/2020 15:58	201.1	199.3	194.9	2441	65.08
12/4/2020 15:58	201.2	198.8	195.3	2437	64.8
12/4/2020 15:58	201.2	199.5	195	2437	65
12/4/2020 15:58	201.2	199.5	194.8	2436	64.96
12/4/2020 15:58	201.1	198.9	195.5	2441	65
12/4/2020 15:58	201.2	199.2	195.7	2440	64.92
12/4/2020 15:58	201.2	199.2	194.9	2440	64.92
12/4/2020 15:58	201.2	199.5	194.6	2445	65
12/4/2020 15:58	201.2	199.2	194.9	2443	64.96
12/4/2020 15:58	201.2	198.8	195.4	2444	64.96
12/4/2020 15:58	201.2	199.2	195.2	2447	65
12/4/2020 15:58	201.1	199.2	194.9	2441	64.92
12/4/2020 15:58	201.2	199.5	194.8	2440	64.96
12/4/2020 15:58	201.2	199.2	195.2	2441	65.04
12/4/2020 15:58	201.2	199	195.9	2442	65
12/4/2020 15:58	201.1	199	195.9	2444	65.04
12/4/2020 15:58	201.2	199.3	195.3	2445	64.92
12/4/2020 15:58	201.2	199.6	195	2445	65
12/4/2020 15:58	201.2	199.4	194.7	2443	64.92
12/4/2020 15:58	201.1	198.9	194.9	2441	65.04
12/4/2020 15:58	201.1	199.2	195.2	2441	65
12/4/2020 15:58	201.1	198.9	195.2	2444	64.88
12/4/2020 15:58	201.1	199.4	195.4	2442	65.03
12/4/2020 15:58	201.2	199.4	195.2	2444	65.12
12/4/2020 15:58	201.2	199	195.2	2444	64.99
12/4/2020 15:58	201.1	198.9	195.4	2447	64.99
12/4/2020 15:58	201.1	199.2	195.6	2448	64.96
12/4/2020 15:58	201.2	199.2	195.4	2448	64.88
12/4/2020 15:58	201.1	199.5	195.3	2445	65.04
12/4/2020 15:58	201.1	199.2	195.2	2446	65.12
12/4/2020 15:58	201.1	199.2	195.4	2447	65.04
12/4/2020 15:58	201.1	198.9	195	2445	64.88
12/4/2020 15:58	201.1	198.8	195.1	2446	64.92
12/4/2020 15:58	201.1	199.5	195	2449	64.92

12/4/2020 15:59	201.1	199	194.9	2446	64.99
12/4/2020 15:59	201.1	199.2	195.2	2445	65.04
12/4/2020 15:59	201.1	199.3	195.4	2450	64.92
12/4/2020 15:59	201.1	198.9	195.2	2449	64.99
12/4/2020 15:59	201.1	199.4	195.6	2450	64.92
12/4/2020 15:59	201.1	199.1	195.6	2445	65
12/4/2020 15:59	201.1	199	195.5	2445	64.96
12/4/2020 15:59	201.1	198.7	195.4	2444	65.04
12/4/2020 15:59	201.1	198.8	195	2441	65
12/4/2020 15:59	201.1	199.5	195.2	2440	64.92
12/4/2020 15:59	201.1	199.2	195.2	2440	64.92
12/4/2020 15:59	201	199.2	195	2444	65
12/4/2020 15:59	201	199.3	195.2	2442	65
12/4/2020 15:59	201.1	198.8	195.2	2442	64.92
12/4/2020 15:59	201.1	199.6	195.3	2441	64.92
12/4/2020 15:59	201.1	198.9	195.1	2444	65
12/4/2020 15:59	201.1	199.5	195.4	2444	64.96
12/4/2020 15:59	201.1	199.3	195.4	2444	64.96
12/4/2020 15:59	201.1	198.9	195.4	2445	65
12/4/2020 15:59	201.1	199	195.5	2444	65.04
12/4/2020 15:59	201.1	198.8	195.3	2446	64.99
12/4/2020 15:59	201.1	199.2	195.3	2445	64.99
12/4/2020 15:59	201.1	199.4	195.2	2445	64.99
12/4/2020 15:59	201.1	199.3	195	2444	64.92
12/4/2020 15:59	201	199.2	195.1	2447	65.04
12/4/2020 15:59	201	198.8	195.2	2444	65
12/4/2020 15:59	201	198.9	194.7	2442	64.83
12/4/2020 15:59	201	198.7	194.9	2440	64.99
12/4/2020 15:59	201	198.8	195	2441	65.01
12/4/2020 15:59	201	199.5	194.9	2442	64.88
12/4/2020 15:59	201.1	199.2	195.4	2444	64.88
12/4/2020 15:59	201.1	199.4	195.5	2446	64.93
12/4/2020 15:59	201	199.5	195.7	2448	65
12/4/2020 15:59	201	198.8	196	2441	65.08
12/4/2020 15:59	201.1	199.4	195.9	2445	65.08
12/4/2020 15:59	201.1	199	195.7	2441	64.99
12/4/2020 15:59	201	198.9	195.7	2441	65.03
12/4/2020 15:59	201	199.2	195	2443	64.96
12/4/2020 15:59	201	198.9	195.1	2446	64.96
12/4/2020 15:59	201	199.5	195	2444	64.95
12/4/2020 15:59	201	199	195.3	2445	64.88
12/4/2020 15:59	201	198.9	195.4	2448	64.96
12/4/2020 15:59	200.9	199.3	195.2	2444	64.96
12/4/2020 15:59	201	198.9	195.4	2444	65.04
12/4/2020 15:59	201	199.5	195.3	2442	64.92
12/4/2020 15:59	200.9	198.9	195.3	2446	64.96
12/4/2020 15:59	201	198.9	195.5	2449	65

12/4/2020 15:59	200.9	198.8	195.3	2447	65.05
12/4/2020 15:59	201	198.8	195.2	2444	64.92
12/4/2020 15:59	200.9	199.2	195.2	2442	65
12/4/2020 15:59	201	199	195.2	2442	65
12/4/2020 15:59	201	199.2	195.6	2444	64.96
12/4/2020 15:59	201	199.4	195.4	2442	64.96
12/4/2020 15:59	201	199.2	195.5	2446	64.96
12/4/2020 15:59	201	199.4	195.6	2447	65
12/4/2020 15:59	201	199.2	195.1	2448	64.92
12/4/2020 15:59	200.9	198.9	195.1	2455	64.96
12/4/2020 15:59	201	198.7	194.9	2454	65.04
12/4/2020 15:59	200.9	198.9	194.9	2452	64.88
12/4/2020 16:00	201	199.4	195.5	2450	64.99
12/4/2020 16:00	201	199.1	195.9	2450	65
12/4/2020 16:00	200.9	199.4	195.7	2452	64.99
12/4/2020 16:00	200.9	199.4	195.1	2450	64.99
12/4/2020 16:00	200.9	198.8	194.7	2447	64.96
12/4/2020 16:00	200.9	199.2	195	2445	64.92
12/4/2020 16:00	200.9	199.4	195.3	2441	64.88
12/4/2020 16:00	200.9	198.9	195.4	2444	65.04
12/4/2020 16:00	201	199.5	195.1	2446	65
12/4/2020 16:00	200.9	198.9	195	2446	64.83
12/4/2020 16:00	200.8	199.4	196.3	2447	65.03
12/4/2020 16:00	200.9	198.9	195.2	2448	65.08
12/4/2020 16:00	200.9	198.7	194.8	2445	64.88
12/4/2020 16:00	201	199.4	195.3	2445	64.99
12/4/2020 16:00	200.9	198.8	195.2	2445	65.12
12/4/2020 16:00	200.9	199.4	195.5	2448	64.91
12/4/2020 16:00	200.9	198.8	195	2447	65.03
12/4/2020 16:00	200.9	199	195.5	2448	64.96
12/4/2020 16:00	200.9	199.4	195.8	2444	65.08
12/4/2020 16:00	200.9	198.9	194.9	2444	65.08
12/4/2020 16:00	200.9	199.5	194.9	2441	64.88
12/4/2020 16:00	200.9	199	195.4	2442	64.99
12/4/2020 16:00	200.9	198.7	195.3	2448	64.88
12/4/2020 16:00	200.9	198.8	195.4	2445	64.99
12/4/2020 16:00	200.8	198.9	195.3	2451	64.96
12/4/2020 16:00	200.8	199.4	194.9	2447	64.88
12/4/2020 16:00	200.9	199.3	195.3	2449	64.99
12/4/2020 16:00	200.9	198.9	195.8	2451	65
12/4/2020 16:00	200.9	198.9	196.2	2450	64.92
12/4/2020 16:00	200.8	199	195.6	2452	65
12/4/2020 16:00	200.8	199.2	194.8	2453	64.96
12/4/2020 16:00	200.9	199.4	195	2448	64.87
12/4/2020 16:00	200.9	198.9	195.2	2448	64.96
12/4/2020 16:00	201	198.8	195.5	2447	64.96
12/4/2020 16:00	200.9	198.8	195.6	2445	64.96

12/4/2020 16:00	200.8	199.4	195.2	2445	64.92
12/4/2020 16:00	200.9	199.2	195.4	2445	64.96
12/4/2020 16:00	200.8	199	195.2	2445	64.96
12/4/2020 16:00	200.9	199.4	194.9	2444	64.92
12/4/2020 16:00	200.9	198.8	196	2441	64.92
12/4/2020 16:00	200.9	199.2	195.6	2449	64.96
12/4/2020 16:00	200.8	199.2	194.8	2448	64.91
12/4/2020 16:00	200.8	198.7	195	2449	64.96
12/4/2020 16:00	200.8	199.5	195.3	2449	65
12/4/2020 16:00	200.9	198.9	195.5	2448	64.99
12/4/2020 16:00	200.9	199.4	194.8	2451	65.04
12/4/2020 16:00	200.9	199.2	195.8	2453	65.04
12/4/2020 16:00	200.9	199.4	195.7	2450	65.04
12/4/2020 16:00	200.8	199.4	194.8	2449	64.99
12/4/2020 16:00	200.9	198.9	195.1	2446	64.99
12/4/2020 16:00	200.9	199	195.6	2446	65.04
12/4/2020 16:00	200.9	198.7	195.3	2446	64.99
12/4/2020 16:00	200.8	198.9	195.2	2451	64.96
12/4/2020 16:00	200.9	199.4	195.7	2453	64.96
12/4/2020 16:00	200.9	198.7	195.9	2453	64.95
12/4/2020 16:00	200.9	199	195.2	2458	64.92
12/4/2020 16:00	200.8	198.8	195.1	2456	64.92
12/4/2020 16:00	201	199.1	195.5	2455	65.12
12/4/2020 16:00	200.9	199.4	195.4	2452	64.95
12/4/2020 16:01	200.8	198.8	195.2	2455	64.99
12/4/2020 16:01	200.8	199.3	195.1	2454	65.04
12/4/2020 16:01	200.8	198.8	195.7	2452	65.04
12/4/2020 16:01	200.9	198.7	196.1	2449	65.03
12/4/2020 16:01	200.8	198.9	195.2	2449	64.96
12/4/2020 16:01	200.9	199	194.9	2446	64.99
12/4/2020 16:01	200.8	199	195.2	2444	64.99
12/4/2020 16:01	200.9	199.4	195.5	2448	65.04
12/4/2020 16:01	200.9	198.9	195.2	2452	64.92
12/4/2020 16:01	200.9	199	195.2	2450	64.92
12/4/2020 16:01	200.8	198.8	196.1	2450	64.96
12/4/2020 16:01	200.9	199.5	195.4	2454	64.96
12/4/2020 16:01	200.8	199.4	195.2	2451	65.04
12/4/2020 16:01	200.8	198.8	195.2	2455	64.92
12/4/2020 16:01	200.9	199.4	195.4	2456	65.08
12/4/2020 16:01	200.9	198.9	195.2	2456	64.91
12/4/2020 16:01	200.8	199.3	195.6	2456	65
12/4/2020 16:01	200.9	199.4	196.2	2460	65.05
12/4/2020 16:01	200.8	198.9	194.9	2454	65
12/4/2020 16:01	200.9	199.3	195.2	2456	65.04
12/4/2020 16:01	200.8	198.9	195.5	2456	65.04
12/4/2020 16:01	200.8	199	195.4	2449	64.96
12/4/2020 16:01	200.9	199.5	195.4	2448	64.96

12/4/2020 16:01	200.8	199.3	195.9	2449	64.96
12/4/2020 16:01	200.9	199.5	195.9	2453	64.92
12/4/2020 16:01	200.9	199.2	195.1	2454	64.92
12/4/2020 16:01	200.9	199.2	195.1	2452	64.88
12/4/2020 16:01	200.9	199.5	195.5	2450	64.99
12/4/2020 16:01	200.9	198.7	195.3	2452	64.88
12/4/2020 16:01	200.8	199.6	195.2	2449	64.96
12/4/2020 16:01	200.9	199	196.1	2452	64.96
12/4/2020 16:01	200.9	199	195.7	2450	64.88
12/4/2020 16:01	200.9	199.4	195	2449	65
12/4/2020 16:01	200.9	199.5	195.3	2453	65.08
12/4/2020 16:01	200.8	199.3	195.4	2455	64.84
12/4/2020 16:01	200.8	199	195.5	2452	64.99
12/4/2020 16:01	200.9	199.5	195.5	2457	64.93
12/4/2020 16:01	200.9	199.4	196.1	2453	65.04
12/4/2020 16:01	200.9	199.1	195.2	2452	64.92
12/4/2020 16:01	200.9	199.5	194.8	2453	64.99
12/4/2020 16:01	200.9	199	195.4	2452	64.92
12/4/2020 16:01	200.9	199.5	195.3	2457	64.88
12/4/2020 16:01	200.9	199.5	195.2	2455	65.04
12/4/2020 16:01	200.9	198.9	195.9	2455	64.96
12/4/2020 16:01	200.9	199.5	196.1	2455	64.95
12/4/2020 16:01	200.9	199.2	195	2457	64.92
12/4/2020 16:01	200.9	199.3	194.9	2452	64.96
12/4/2020 16:01	200.8	199.2	195.4	2452	64.83
12/4/2020 16:01	200.9	198.8	195.6	2449	64.92
12/4/2020 16:01	200.9	199.2	195.4	2456	64.96
12/4/2020 16:01	200.9	198.9	195.3	2452	64.99
12/4/2020 16:01	200.9	199.4	195.9	2455	65.03
12/4/2020 16:01	200.9	199.3	195.9	2458	65
12/4/2020 16:01	200.9	198.8	195.2	2454	64.91
12/4/2020 16:01	200.9	199.4	195.1	2452	65.04
12/4/2020 16:01	200.9	198.9	195.4	2450	65.04
12/4/2020 16:01	200.9	199.4	195.4	2449	65.04
12/4/2020 16:01	200.9	199.2	195.4	2449	64.92
12/4/2020 16:01	200.9	198.7	195.4	2449	64.92
12/4/2020 16:01	200.9	199	195.2	2452	64.96
12/4/2020 16:02	200.9	198.9	195.2	2449	64.91
12/4/2020 16:02	200.9	198.9	195.4	2447	64.96
12/4/2020 16:02	200.8	199.3	195.4	2449	65
12/4/2020 16:02	200.9	198.8	196.1	2447	64.96
12/4/2020 16:02	200.9	199.6	196.2	2451	64.92
12/4/2020 16:02	200.9	199.2	196	2450	64.96
12/4/2020 16:02	200.9	199	195.9	2449	64.96
12/4/2020 16:02	200.9	199.5	195.4	2449	64.96
12/4/2020 16:02	200.9	198.7	195.2	2449	65.04
12/4/2020 16:02	200.9	199.3	195	2449	64.87

12/4/2020 16:02	200.8	199.2	195	2446	64.96
12/4/2020 16:02	200.9	199.4	195.3	2451	65
12/4/2020 16:02	200.9	199.4	195.2	2449	64.88
12/4/2020 16:02	200.9	198.9	195.3	2449	64.96
12/4/2020 16:02	200.9	199.3	195.6	2449	65.08
12/4/2020 16:02	200.9	198.7	195.9	2450	65
12/4/2020 16:02	200.9	199.4	196.4	2450	64.96
12/4/2020 16:02	200.9	199.4	196.2	2451	64.99
12/4/2020 16:02	200.8	198.8	195.8	2456	65.01
12/4/2020 16:02	200.9	199.2	195.5	2456	64.99
12/4/2020 16:02	200.9	199.3	195.5	2453	64.96
12/4/2020 16:02	200.9	199.5	195.2	2456	65.08
12/4/2020 16:02	200.9	199	195.3	2454	64.88
12/4/2020 16:02	200.9	198.7	195.2	2454	65.04
12/4/2020 16:02	200.8	199.4	195.5	2451	65.08
12/4/2020 16:02	200.9	199.1	195.5	2449	64.92
12/4/2020 16:02	200.9	198.8	195.2	2450	65.03
12/4/2020 16:02	201	199.3	195.2	2450	64.96
12/4/2020 16:02	200.9	198.9	195	2453	64.92
12/4/2020 16:02	200.9	199.3	196.2	2450	65
12/4/2020 16:02	200.9	198.8	195.9	2451	65.04
12/4/2020 16:02	200.9	199.2	195.2	2451	64.92
12/4/2020 16:02	200.9	199.4	195.4	2452	65.03
12/4/2020 16:02	200.9	198.8	195.4	2453	64.88
12/4/2020 16:02	200.9	199.3	195.5	2453	65
12/4/2020 16:02	201	198.8	195.2	2452	65
12/4/2020 16:02	201	198.9	194.8	2452	65.04
12/4/2020 16:02	200.9	199.5	195.2	2449	65.04
12/4/2020 16:02	200.9	198.8	195.8	2449	64.96
12/4/2020 16:02	201	199.3	196	2449	64.99
12/4/2020 16:02	200.9	199.4	195.7	2453	65
12/4/2020 16:02	200.9	198.8	195.1	2453	64.96
12/4/2020 16:02	200.9	199.4	195.1	2453	64.96
12/4/2020 16:02	201	198.9	195.5	2454	65.04
12/4/2020 16:02	201	199.3	195.2	2452	65.08
12/4/2020 16:02	201	199.3	195.3	2455	64.92
12/4/2020 16:02	201	199.5	195.3	2455	64.91
12/4/2020 16:02	201	198.8	195.6	2454	65.05
12/4/2020 16:02	201	198.9	195.4	2453	64.96
12/4/2020 16:02	201	199.4	195.4	2451	64.96
12/4/2020 16:02	200.9	199.3	195.4	2450	65.05
12/4/2020 16:02	201	199.4	195.3	2453	64.96
12/4/2020 16:02	201	199.5	195.4	2451	65.04
12/4/2020 16:02	200.9	199	195.6	2453	65
12/4/2020 16:02	201	199.4	195.4	2459	64.92
12/4/2020 16:02	201	199.2	195.4	2459	64.92
12/4/2020 16:02	201	198.8	195.3	2458	64.92

12/4/2020 16:02	201	199.2	195.5	2455	65.08
12/4/2020 16:02	201	199	196.2	2453	64.99
12/4/2020 16:03	200.9	199.1	195.7	2449	64.84
12/4/2020 16:03	201	199.3	194.9	2446	64.92
12/4/2020 16:03	201.1	198.8	195.5	2452	64.88
12/4/2020 16:03	201	199.5	195.4	2454	64.88
12/4/2020 16:03	201	198.9	195.3	2454	65.08
12/4/2020 16:03	201	199.5	195.2	2448	64.84
12/4/2020 16:03	201	198.9	196.2	2451	64.99
12/4/2020 16:03	201.1	198.9	195.2	2449	65.08
12/4/2020 16:03	201	199.4	195	2447	65
12/4/2020 16:03	201	198.9	195.3	2451	65.04
12/4/2020 16:03	201.1	199.3	195.5	2452	64.96
12/4/2020 16:03	201	199.3	195.2	2455	64.99
12/4/2020 16:03	201	198.8	195.5	2448	64.92
12/4/2020 16:03	201	199	196.1	2453	65.04
12/4/2020 16:03	201	198.9	195.4	2453	65.04
12/4/2020 16:03	201.1	199.5	195	2452	64.96
12/4/2020 16:03	201	199.4	195.4	2448	64.96
12/4/2020 16:03	201.1	198.8	195.5	2452	65.04
12/4/2020 16:03	201	199	195	2452	64.96
12/4/2020 16:03	201	199.1	195.4	2450	64.96
12/4/2020 16:03	201	199.1	196.2	2459	65
12/4/2020 16:03	201	199.1	195.2	2453	64.92
12/4/2020 16:03	201.1	198.8	195	2457	64.96
12/4/2020 16:03	201.1	199.1	195.4	2459	65
12/4/2020 16:03	201.1	199	195.4	2460	64.88
12/4/2020 16:03	201.1	198.9	195.2	2457	64.92
12/4/2020 16:03	201.1	199.5	195.9	2459	65
12/4/2020 16:03	201	198.8	195.8	2456	65.08
12/4/2020 16:03	201.1	199.1	195	2450	64.88
12/4/2020 16:03	201.1	199	195.1	2448	64.84
12/4/2020 16:03	201.1	199.5	195.6	2453	64.92
12/4/2020 16:03	201.1	199.4	195.6	2449	64.84
12/4/2020 16:03	201.1	198.9	195	2449	64.92
12/4/2020 16:03	201.1	199.2	195.8	2453	64.96
12/4/2020 16:03	201	198.8	196.2	2450	65.04
12/4/2020 16:03	201.1	199.2	195.1	2447	64.96
12/4/2020 16:03	201.1	199.5	195.5	2443	65.08
12/4/2020 16:03	201	198.9	195.4	2447	64.96
12/4/2020 16:03	201.1	199.4	195.5	2447	64.92
12/4/2020 16:03	201.1	199.1	195.4	2449	64.92
12/4/2020 16:03	201.1	199.3	195.6	2452	64.96
12/4/2020 16:03	201	199.4	196.4	2452	64.92
12/4/2020 16:03	201.1	198.7	195.2	2451	64.96
12/4/2020 16:03	201.1	199.4	195.2	2451	65
12/4/2020 16:03	201.1	198.8	195.5	2450	64.92

12/4/2020 16:03	201.1	199.4	195.4	2454	65
12/4/2020 16:03	201.1	199.4	195.3	2456	65.09
12/4/2020 16:03	201.1	198.8	196.3	2451	64.96
12/4/2020 16:03	201.1	199.6	195.6	2449	64.96
12/4/2020 16:03	201.1	198.9	195.2	2453	64.96
12/4/2020 16:03	201.1	198.9	195.5	2452	65.04
12/4/2020 16:03	201.1	199.5	195.5	2451	65
12/4/2020 16:03	201.1	199.1	195.1	2449	64.96
12/4/2020 16:03	201.1	199.5	195.6	2453	65.04
12/4/2020 16:03	201.1	199.2	196.3	2452	64.92
12/4/2020 16:03	201.1	199.3	195.2	2451	64.99
12/4/2020 16:03	201.1	199.4	195.1	2455	65.08
12/4/2020 16:03	201.1	199.3	195.4	2454	64.99
12/4/2020 16:03	201.1	199.5	195.5	2452	64.99
12/4/2020 16:03	201	198.9	195.2	2456	65.08
12/4/2020 16:04	201.1	199.5	195.9	2452	64.96
12/4/2020 16:04	201.1	198.8	196.1	2451	64.88
12/4/2020 16:04	201.1	198.7	195.2	2450	65.01
12/4/2020 16:04	201.1	199.4	195.3	2453	65.04
12/4/2020 16:04	201.1	199	195.5	2458	65
12/4/2020 16:04	201.1	198.9	195.6	2455	65.12
12/4/2020 16:04	201.1	199.3	195.3	2457	64.96
12/4/2020 16:04	201.1	198.9	196.3	2453	65.04
12/4/2020 16:04	201.1	199.5	195.8	2454	65.05
12/4/2020 16:04	201.1	199	195.3	2452	65
12/4/2020 16:04	201.1	199	195.4	2451	64.96
12/4/2020 16:04	201.1	198.8	195.6	2451	64.92
12/4/2020 16:04	201.1	198.8	195.7	2452	65
12/4/2020 16:04	201.1	199.4	195.4	2448	64.92
12/4/2020 16:04	201.1	199	195	2452	64.92
12/4/2020 16:04	201.1	199.5	195.7	2454	65
12/4/2020 16:04	201.2	199.5	196.1	2450	64.96
12/4/2020 16:04	201.1	198.7	195.9	2450	65
12/4/2020 16:04	201.2	199.5	195.3	2450	65
12/4/2020 16:04	201.2	198.8	195.1	2451	64.88
12/4/2020 16:04	201.1	199.4	195.2	2452	64.96
12/4/2020 16:04	201.2	198.8	195.4	2456	65.04
12/4/2020 16:04	201.2	199.3	195.5	2459	65.04
12/4/2020 16:04	201.1	199.5	195.7	2458	64.99
12/4/2020 16:04	201.2	199	195.4	2459	65.04
12/4/2020 16:04	201.2	199.5	195.6	2462	64.96
12/4/2020 16:04	201.1	198.9	195.5	2458	64.92
12/4/2020 16:04	201.2	198.8	195.4	2456	64.84
12/4/2020 16:04	201.2	199.5	195.5	2456	65
12/4/2020 16:04	201.2	198.9	195.2	2456	64.84
12/4/2020 16:04	201.2	199.4	195.5	2460	64.92
12/4/2020 16:04	201.2	199.4	195.3	2466	64.93

12/4/2020 16:04	201.2	198.8	195.3	2463	65.03
12/4/2020 16:04	201.2	199.4	195.6	2465	64.88
12/4/2020 16:04	201.2	198.9	195.7	2459	65.01
12/4/2020 16:04	201.2	199	195.8	2459	65.04
12/4/2020 16:04	201.2	199.4	196.2	2458	64.92
12/4/2020 16:04	201.3	198.9	196.1	2465	65.01
12/4/2020 16:04	201.1	199.4	196.3	2461	65
12/4/2020 16:04	201.3	198.9	195.9	2456	64.96
12/4/2020 16:04	201.3	199.4	195.7	2454	64.96
12/4/2020 16:04	201.2	198.7	195.2	2458	64.92
12/4/2020 16:04	201.2	198.7	195.1	2458	64.92
12/4/2020 16:04	201.2	199.5	195.2	2457	64.96
12/4/2020 16:04	201.3	199.2	195.6	2460	65
12/4/2020 16:04	201.3	199.5	195.7	2457	64.92
12/4/2020 16:04	201.3	199.4	195.7	2457	65.01
12/4/2020 16:04	201.3	198.8	195.6	2463	65.08
12/4/2020 16:04	201.2	199.5	195.4	2464	64.96
12/4/2020 16:04	201.3	198.8	195.2	2465	64.92
12/4/2020 16:04	201.3	199.2	195.6	2462	65.12
12/4/2020 16:04	201.2	199.5	196.3	2463	64.96
12/4/2020 16:04	201.3	198.8	195.9	2461	64.92
12/4/2020 16:04	201.3	199.2	195.2	2459	64.99
12/4/2020 16:04	201.2	199	195.2	2457	65.04
12/4/2020 16:04	201.5	199	195.4	2458	65.04
12/4/2020 16:04	201.2	199.4	195.6	2459	65.08
12/4/2020 16:04	201.3	199.4	195.4	2458	65.08
12/4/2020 16:04	201.3	199.3	195.2	2456	64.96
12/4/2020 16:05	201.3	198.9	195.4	2459	65.08
12/4/2020 16:05	201.3	199.2	195.6	2458	65.01
12/4/2020 16:05	201.3	199	196.1	2456	65.04
12/4/2020 16:05	201.3	198.9	196.4	2459	65.04
12/4/2020 16:05	201.3	199.4	196	2457	65
12/4/2020 16:05	201.3	198.9	195.4	2458	65.04
12/4/2020 16:05	201.3	198.9	195.2	2459	65.04
12/4/2020 16:05	201.3	199.2	195.1	2460	64.96
12/4/2020 16:05	201.3	199.2	195.3	2462	65.04
12/4/2020 16:05	201.3	199.5	195.5	2463	64.96
12/4/2020 16:05	201.3	199.1	195.5	2461	64.92
12/4/2020 16:05	201.3	199.2	195.9	2459	64.96
12/4/2020 16:05	201.3	199.5	195.4	2459	64.88
12/4/2020 16:05	201.3	198.7	195.6	2459	64.92
12/4/2020 16:05	201.3	199.5	195.5	2457	65.09
12/4/2020 16:05	201.3	199	195.3	2456	64.92
12/4/2020 16:05	201.3	198.9	195.5	2459	64.92
12/4/2020 16:05	201.3	199.4	195.7	2461	64.97
12/4/2020 16:05	201.3	198.9	196.2	2462	65.04
12/4/2020 16:05	201.3	199.6	196.4	2465	64.96

12/4/2020 16:05	201.3	199.2	196.1	2462	65.08
12/4/2020 16:05	201.4	199.4	195.7	2460	64.88
12/4/2020 16:05	201.3	199.4	195.4	2460	64.92
12/4/2020 16:05	201.3	198.8	195.2	2459	65.08
12/4/2020 16:05	201.3	199.1	195	2462	64.88
12/4/2020 16:05	201.3	198.7	195.3	2456	64.88
12/4/2020 16:05	201.3	199.1	195.5	2456	64.88
12/4/2020 16:05	201.3	199.7	195.5	2459	64.93
12/4/2020 16:05	201.3	198.7	195.5	2458	64.99
12/4/2020 16:05	201.4	199.4	195.7	2458	65
12/4/2020 16:05	201.4	198.8	195.4	2460	65.08
12/4/2020 16:05	201.4	198.8	195.2	2455	64.96
12/4/2020 16:05	201.4	199.3	195.3	2452	65.04
12/4/2020 16:05	201.4	199.3	195.7	2452	65
12/4/2020 16:05	201.4	199.4	196.1	2451	65.04
12/4/2020 16:05	201.3	199	196.2	2449	64.96
12/4/2020 16:05	201.4	198.9	196	2452	64.96
12/4/2020 16:05	201.3	199.5	195.5	2453	65.04
12/4/2020 16:05	201.3	199.3	195.2	2450	65
12/4/2020 16:05	201.4	199.1	195.4	2453	65
12/4/2020 16:05	201.3	198.9	195.4	2457	65.04
12/4/2020 16:05	201.5	198.9	195.5	2460	65.04
12/4/2020 16:05	201.4	199.5	195.7	2458	64.99
12/4/2020 16:05	201.3	198.8	195.4	2454	64.99
12/4/2020 16:05	201.3	199.4	195.2	2453	64.92
12/4/2020 16:05	201.3	199.3	196.2	2455	65.04
12/4/2020 16:05	201.4	199	196.4	2452	65.04
12/4/2020 16:05	201.4	198.9	195.5	2451	65.12
12/4/2020 16:05	201.4	199.3	195	2456	64.92
12/4/2020 16:05	201.4	199.4	195.4	2456	64.99
12/4/2020 16:05	201.4	199	195.9	2461	64.96
12/4/2020 16:05	201.4	199.4	195.6	2460	64.99
12/4/2020 16:05	201.4	198.9	195.6	2454	64.99
12/4/2020 16:05	201.4	198.8	196.2	2452	64.96
12/4/2020 16:05	201.3	199.3	196	2453	64.88
12/4/2020 16:05	201.4	198.9	195.2	2454	65.04
12/4/2020 16:05	201.4	199.5	195.4	2453	65.08
12/4/2020 16:05	201.5	199.2	195.7	2449	64.92
12/4/2020 16:05	201.3	198.8	195.9	2450	65.04
12/4/2020 16:06	201.5	199.4	195.4	2451	64.99
12/4/2020 16:06	201.4	199	195.9	2452	65
12/4/2020 16:06	201.4	199.5	196.4	2454	65.04
12/4/2020 16:06	201.4	198.9	195.5	2449	64.88
12/4/2020 16:06	201.4	198.8	195.4	2449	65.04
12/4/2020 16:06	201.5	199.4	195.6	2449	64.99
12/4/2020 16:06	201.5	198.9	195.8	2452	64.92
12/4/2020 16:06	201.5	199.5	195.2	2453	65

12/4/2020 16:06	201.5	199.3	196.2	2450	64.92
12/4/2020 16:06	201.5	198.9	196.2	2453	64.96
12/4/2020 16:06	201.5	199.4	195.2	2458	65
12/4/2020 16:06	201.5	199.2	195.2	2453	64.92
12/4/2020 16:06	201.5	199.4	195.5	2454	64.96
12/4/2020 16:06	201.5	199.3	195.7	2453	65
12/4/2020 16:06	201.5	198.8	195.7	2451	64.96
12/4/2020 16:06	201.5	199.5	195.5	2449	64.92
12/4/2020 16:06	201.5	198.9	195.9	2449	65.04
12/4/2020 16:06	201.5	199.2	196.4	2449	64.87
12/4/2020 16:06	201.5	199.5	196.2	2448	64.96
12/4/2020 16:06	201.5	199.4	195.6	2448	64.96
12/4/2020 16:06	201.5	199.1	195.5	2447	64.96
12/4/2020 16:06	201.5	199	195.2	2444	64.88
12/4/2020 16:06	201.5	199	195.5	2445	64.96
12/4/2020 16:06	201.3	199.5	195.5	2448	65
12/4/2020 16:06	201.5	199.4	195.9	2445	64.96
12/4/2020 16:06	201.5	199.2	195.6	2444	64.96
12/4/2020 16:06	201.5	198.8	195.6	2445	65.04
12/4/2020 16:06	201.5	199	195.7	2448	64.88
12/4/2020 16:06	201.5	199.5	195.6	2444	64.83
12/4/2020 16:06	201.5	198.8	195.4	2444	65.09
12/4/2020 16:06	201.5	199.4	195.7	2449	65.04
12/4/2020 16:06	201.5	198.9	195.9	2449	64.88
12/4/2020 16:06	201.5	198.8	196.1	2449	64.93
12/4/2020 16:06	201.5	198.8	196.1	2450	64.88
12/4/2020 16:06	201.5	199.4	196.4	2445	65.04
12/4/2020 16:06	201.5	199.5	196.7	2448	64.83
12/4/2020 16:06	201.5	199.2	196.4	2452	64.97
12/4/2020 16:06	201.5	198.8	196.2	2450	64.83
12/4/2020 16:06	201.5	199.5	196.4	2451	65.03
12/4/2020 16:06	201.5	198.8	195.9	2450	65.01
12/4/2020 16:06	201.5	199.4	195.9	2451	64.88
12/4/2020 16:06	201.5	199	195.5	2452	64.96
12/4/2020 16:06	201.5	199.1	195.4	2453	64.96
12/4/2020 16:06	201.4	199.6	195.5	2452	64.83
12/4/2020 16:06	201.5	199	195.2	2452	64.88
12/4/2020 16:06	201.5	199.4	195.3	2452	65.08
12/4/2020 16:06	201.5	198.9	195.2	2453	65.04
12/4/2020 16:06	201.5	199.4	195.4	2450	65.04
12/4/2020 16:06	201.5	199.4	195.5	2450	65.04
12/4/2020 16:06	201.5	198.9	195.5	2449	64.88
12/4/2020 16:06	201.5	199	195.7	2447	64.99
12/4/2020 16:06	201.5	198.9	195.6	2446	65
12/4/2020 16:06	201.5	199.3	195.6	2445	65.08
12/4/2020 16:06	201.5	199.5	195.7	2448	64.88
12/4/2020 16:06	201.5	198.5	195.9	2453	64.88

12/4/2020 16:06	201.5	199.3	195.7	2455	64.96
12/4/2020 16:06	201.5	198.8	195.6	2459	64.88
12/4/2020 16:06	201.5	199	195.6	2457	64.96
12/4/2020 16:06	201.5	199	195.8	2458	64.96
12/4/2020 16:06	201.5	199.4	195.5	2459	64.96
12/4/2020 16:07	201.5	199.2	195.3	2457	64.92
12/4/2020 16:07	201.6	198.8	195.7	2453	65
12/4/2020 16:07	201.5	199.4	195.7	2454	65.04
12/4/2020 16:07	201.5	199.4	196.4	2453	64.96
12/4/2020 16:07	201.5	198.8	196.5	2449	65
12/4/2020 16:07	201.6	199.5	195.5	2446	65.04
12/4/2020 16:07	201.6	198.8	195.4	2448	64.91
12/4/2020 16:07	201.6	199.4	195.2	2449	64.99
12/4/2020 16:07	201.6	199.2	195.6	2451	65.05
12/4/2020 16:07	201.6	198.9	195.7	2451	64.88
12/4/2020 16:07	201.6	199.4	195.4	2452	65.04
12/4/2020 16:07	201.6	199	196	2453	65.08
12/4/2020 16:07	201.5	199.5	196.4	2452	64.88
12/4/2020 16:07	201.5	199.5	195.2	2450	64.84
12/4/2020 16:07	201.6	198.8	195.6	2451	64.92
12/4/2020 16:07	201.5	199	195.7	2449	65.12
12/4/2020 16:07	201.6	198.8	195.6	2452	64.88
12/4/2020 16:07	201.6	198.8	195.7	2450	64.93
12/4/2020 16:07	201.5	199.5	196.4	2452	65
12/4/2020 16:07	201.6	198.7	195.7	2449	64.88
12/4/2020 16:07	201.6	199	195.4	2449	64.96
12/4/2020 16:07	201.5	199	195.7	2453	65.08
12/4/2020 16:07	201.6	199	196.1	2449	64.99
12/4/2020 16:07	201.5	199.3	195.4	2447	64.92
12/4/2020 16:07	201.6	199.3	195.7	2450	65.04
12/4/2020 16:07	201.6	199.3	196.3	2451	64.83
12/4/2020 16:07	201.6	198.9	195.8	2453	64.96
12/4/2020 16:07	201.6	198.9	195.4	2452	64.96
12/4/2020 16:07	201.6	199.4	195.7	2455	64.96
12/4/2020 16:07	201.6	198.7	195.8	2449	64.92
12/4/2020 16:07	201.6	198.9	195.6	2450	64.96
12/4/2020 16:07	201.6	199.2	195.6	2452	65
12/4/2020 16:07	201.6	198.8	196	2449	64.87
12/4/2020 16:07	201.6	199.5	196.8	2452	64.96
12/4/2020 16:07	201.6	199.4	196.1	2452	65.04
12/4/2020 16:07	201.6	199.4	195.5	2453	65
12/4/2020 16:07	201.6	199	195.5	2453	64.88
12/4/2020 16:07	201.6	199	195.7	2452	64.92
12/4/2020 16:07	201.6	199.4	196	2453	64.99
12/4/2020 16:07	201.6	198.8	195.7	2456	64.91
12/4/2020 16:07	201.6	199.3	196	2457	65.09
12/4/2020 16:07	201.6	199.2	196	2458	64.96

12/4/2020 16:07	201.6	198.8	195.7	2452	64.92
12/4/2020 16:07	201.7	198.7	195.7	2453	64.99
12/4/2020 16:07	201.7	199.1	195.7	2459	65.08
12/4/2020 16:07	201.7	199.5	195.7	2461	64.99
12/4/2020 16:07	201.7	198.9	195.9	2461	64.84
12/4/2020 16:07	201.6	198.7	195.6	2464	65.08
12/4/2020 16:07	201.6	199.3	195.9	2463	65.04
12/4/2020 16:07	201.6	198.8	195.7	2462	65.04
12/4/2020 16:07	201.7	199.3	195.7	2458	65.08
12/4/2020 16:07	201.7	199.2	195.9	2455	64.96
12/4/2020 16:07	201.7	199.3	195.5	2456	65.03
12/4/2020 16:07	201.7	198.8	195.7	2455	64.88
12/4/2020 16:07	201.7	198.7	195.7	2453	65.08
12/4/2020 16:07	201.7	199.4	195.7	2454	64.99
12/4/2020 16:07	201.7	199.1	195.7	2449	64.99
12/4/2020 16:07	201.7	199	195.7	2453	65.01
12/4/2020 16:07	201.6	199.1	195.9	2453	64.88
12/4/2020 16:08	201.6	198.7	195.9	2451	64.96
12/4/2020 16:08	201.7	199.5	195.9	2454	65.04
12/4/2020 16:08	201.7	198.7	195.8	2455	64.92
12/4/2020 16:08	201.7	199.4	195.9	2452	64.96
12/4/2020 16:08	201.7	199.2	196.2	2453	65
12/4/2020 16:08	201.7	198.8	196.1	2453	65.04
12/4/2020 16:08	201.7	198.8	195.9	2454	64.92
12/4/2020 16:08	201.7	199	195.9	2451	64.88
12/4/2020 16:08	201.6	199.1	195.9	2453	65.05
12/4/2020 16:08	201.7	199.3	195.7	2451	64.88
12/4/2020 16:08	201.7	198.7	196	2446	64.96
12/4/2020 16:08	201.7	199.4	195.7	2445	65.08
12/4/2020 16:08	201.7	198.9	195.7	2444	64.88
12/4/2020 16:08	201.7	199	195.7	2452	64.96
12/4/2020 16:08	201.7	199.3	195.5	2453	65.04
12/4/2020 16:08	201.7	198.6	195.5	2449	64.88
12/4/2020 16:08	201.7	199.6	195.5	2446	65.04
12/4/2020 16:08	201.7	198.9	195.5	2448	65.12
12/4/2020 16:08	201.7	199.4	196.1	2449	64.84
12/4/2020 16:08	201.7	198.9	196.5	2449	65.08
12/4/2020 16:08	201.7	199	196.6	2447	64.96
12/4/2020 16:08	201.7	199.4	196.3	2449	65.04
12/4/2020 16:08	201.7	199.2	195.7	2445	64.84
12/4/2020 16:08	201.7	199.5	195.6	2450	64.96
12/4/2020 16:08	201.7	199.2	195.7	2455	65
12/4/2020 16:08	201.7	198.8	195.8	2454	64.96
12/4/2020 16:08	201.7	199	195.8	2453	64.96
12/4/2020 16:08	201.7	198.9	195.9	2453	65.04
12/4/2020 16:08	201.7	199.3	195.6	2452	65
12/4/2020 16:08	201.7	199.3	195.5	2451	64.96

12/4/2020 16:08	201.7	198.9	195.5	2453	65.04
12/4/2020 16:08	201.8	199.6	196.4	2452	64.91
12/4/2020 16:08	201.6	198.8	196.6	2453	64.92
12/4/2020 16:08	201.7	199.5	196.2	2457	65.01
12/4/2020 16:08	201.7	199.5	195.7	2452	65.08
12/4/2020 16:08	201.7	198.8	195.6	2450	64.92
12/4/2020 16:08	201.7	199.5	196	2451	64.88
12/4/2020 16:08	201.7	199.1	195.7	2453	64.88
12/4/2020 16:08	201.7	199.4	195.8	2450	64.96
12/4/2020 16:08	201.7	199.4	195.9	2453	65
12/4/2020 16:08	201.7	198.9	195.4	2454	64.93
12/4/2020 16:08	201.8	199.3	195.5	2453	64.99
12/4/2020 16:08	201.7	198.8	195.4	2449	64.84
12/4/2020 16:08	201.7	199.4	195.5	2449	64.96
12/4/2020 16:08	201.8	199.1	195.5	2452	64.99
12/4/2020 16:08	201.7	198.8	195.6	2451	64.84
12/4/2020 16:08	201.8	199.5	195.3	2456	65.04
12/4/2020 16:08	201.7	199.2	195.5	2451	64.99
12/4/2020 16:08	201.8	199.4	195.4	2448	64.92
12/4/2020 16:08	201.8	198.9	195.5	2449	65.01
12/4/2020 16:08	201.7	198.7	195.6	2453	64.92
12/4/2020 16:08	201.7	199.4	195.7	2452	65.08
12/4/2020 16:08	201.8	198.9	195.9	2452	65.04
12/4/2020 16:08	201.7	199.4	195.7	2449	64.99
12/4/2020 16:08	201.8	199	195.6	2449	65.04
12/4/2020 16:08	201.8	198.8	196.5	2449	65.08
12/4/2020 16:08	201.8	199	196.5	2449	65.04
12/4/2020 16:08	201.8	199	195.7	2453	64.96
12/4/2020 16:08	201.8	199.3	195.9	2452	64.96
12/4/2020 16:08	201.8	199.2	195.9	2452	65.09
12/4/2020 16:09	201.8	198.7	195.7	2453	64.92
12/4/2020 16:09	201.8	199.3	196.5	2453	64.92
12/4/2020 16:09	201.8	199	196.2	2452	65.04
12/4/2020 16:09	201.8	199.4	195.5	2455	64.96
12/4/2020 16:09	201.8	199	196	2452	65
12/4/2020 16:09	201.8	198.9	196.2	2453	65.12
12/4/2020 16:09	201.8	199.5	195.6	2452	64.92
12/4/2020 16:09	201.8	199	196.4	2448	64.96
12/4/2020 16:09	201.8	199.5	196.7	2449	64.92
12/4/2020 16:09	201.8	199.4	195.5	2453	64.96
12/4/2020 16:09	201.8	198.9	195.8	2453	64.92
12/4/2020 16:09	201.8	198.8	196	2453	65.04
12/4/2020 16:09	201.9	198.8	196.1	2458	65.2
12/4/2020 16:09	201.9	199.4	196	2447	64.88
12/4/2020 16:09	201.9	199.2	196.6	2447	65.08
12/4/2020 16:09	201.9	199.6	196.4	2449	65.08
12/4/2020 16:09	201.8	199.2	195.6	2450	64.99

12/4/2020 16:09	201.9	198.8	195.8	2455	65.12
12/4/2020 16:09	201.9	199.2	196.1	2451	65.08
12/4/2020 16:09	201.8	199.4	195.9	2452	64.88
12/4/2020 16:09	201.8	199.4	196.1	2452	64.88
12/4/2020 16:09	201.9	199	196.7	2456	64.96
12/4/2020 16:09	201.8	198.9	195.9	2456	64.92
12/4/2020 16:09	201.9	199.2	195.7	2459	64.99
12/4/2020 16:09	201.8	198.8	195.7	2456	64.96
12/4/2020 16:09	201.8	198.7	196	2449	65
12/4/2020 16:09	201.9	199.4	195.9	2449	64.96
12/4/2020 16:09	201.9	198.9	196.4	2453	64.92
12/4/2020 16:09	201.9	199.5	196.7	2453	65.04
12/4/2020 16:09	201.8	199	195.5	2456	65
12/4/2020 16:09	201.9	198.7	195.8	2452	64.96
12/4/2020 16:09	201.8	199.5	196.2	2453	65.09
12/4/2020 16:09	201.9	198.8	195.8	2452	65
12/4/2020 16:09	201.8	199.5	195.9	2449	65
12/4/2020 16:09	201.8	199.2	196.8	2449	65.04
12/4/2020 16:09	201.8	199.5	196.2	2455	65.05
12/4/2020 16:09	201.9	199.5	195.6	2457	64.92
12/4/2020 16:09	201.9	198.7	195.9	2458	64.96
12/4/2020 16:09	201.9	199.5	196.1	2458	65.04
12/4/2020 16:09	201.8	199.3	196	2456	64.99
12/4/2020 16:09	201.9	199.6	195.7	2457	65.04
12/4/2020 16:09	201.9	199.2	196.6	2456	65.12
12/4/2020 16:09	201.8	198.9	196.7	2454	64.99
12/4/2020 16:09	201.8	199.5	195.7	2455	65.08
12/4/2020 16:09	201.8	199.2	195.6	2456	65.09
12/4/2020 16:09	201.8	198.7	196	2454	64.92
12/4/2020 16:09	201.8	199.5	196.4	2455	64.96
12/4/2020 16:09	201.8	199	195.9	2455	64.96
12/4/2020 16:09	201.8	199.4	195.7	2453	65.04
12/4/2020 16:09	201.8	199	196.6	2451	64.92
12/4/2020 16:09	201.8	198.8	196.6	2453	64.88
12/4/2020 16:09	201.8	199.4	195.7	2458	64.96
12/4/2020 16:09	201.8	199	195.5	2456	64.99
12/4/2020 16:09	201.8	199.2	195.8	2452	65.04
12/4/2020 16:09	201.8	199.2	195.9	2458	65
12/4/2020 16:09	201.8	199.5	195.7	2457	64.96
12/4/2020 16:09	201.8	199.2	195.9	2458	64.96
12/4/2020 16:09	201.8	198.7	196.8	2458	65.09
12/4/2020 16:09	201.8	199.3	196.1	2458	65
12/4/2020 16:10	201.8	198.9	195.8	2455	64.96
12/4/2020 16:10	201.7	199.5	195.9	2455	64.96
12/4/2020 16:10	201.8	199.6	195.8	2457	64.96
12/4/2020 16:10	201.8	198.9	195.7	2453	64.92
12/4/2020 16:10	201.8	199	196.2	2456	64.88

12/4/2020 16:10	201.8	198.9	196.6	2454	65.05
12/4/2020 16:10	201.8	198.8	195.8	2453	64.88
12/4/2020 16:10	201.8	199.5	195.6	2449	64.92
12/4/2020 16:10	201.8	198.9	196.1	2451	65.01
12/4/2020 16:10	201.8	199.4	195.9	2452	64.96
12/4/2020 16:10	201.8	199.4	195.6	2449	64.99
12/4/2020 16:10	201.8	198.7	196.8	2451	65.04
12/4/2020 16:10	201.8	199.5	196	2457	64.93
12/4/2020 16:10	201.8	199.1	195.7	2451	64.92
12/4/2020 16:10	201.9	199	196.1	2453	64.92
12/4/2020 16:10	201.8	199.1	195.9	2453	65.04
12/4/2020 16:10	201.8	199.5	195.9	2452	65.04
12/4/2020 16:10	201.8	199	195.8	2454	65.12
12/4/2020 16:10	201.9	198.8	196.7	2456	64.96
12/4/2020 16:10	201.8	199.4	195.8	2458	64.92
12/4/2020 16:10	201.8	198.7	195.6	2452	64.99
12/4/2020 16:10	201.8	198.9	195.9	2454	65.04
12/4/2020 16:10	201.8	199	195.9	2456	64.92
12/4/2020 16:10	201.8	198.7	195.6	2454	64.92
12/4/2020 16:10	201.8	199.4	196.6	2455	65.09
12/4/2020 16:10	201.8	199	196.2	2458	65
12/4/2020 16:10	201.9	199	195.6	2460	65
12/4/2020 16:10	201.8	198.7	195.8	2460	65.04
12/4/2020 16:10	201.8	198.9	196	2458	64.96
12/4/2020 16:10	201.8	199.4	195.9	2457	65
12/4/2020 16:10	201.8	199.2	196.1	2457	65.08
12/4/2020 16:10	201.8	198.8	196.6	2457	64.88
12/4/2020 16:10	201.8	198.9	195.6	2458	65.08
12/4/2020 16:10	201.9	199.6	195.5	2463	65.04
12/4/2020 16:10	201.8	200.3	195.9	2459	64.92
12/4/2020 16:10	201.9	200.4	196.1	2454	64.92
12/4/2020 16:10	201.9	199.6	195.7	2456	64.92
12/4/2020 16:10	201.8	199.9	196.4	2458	65.04
12/4/2020 16:10	201.9	199.7	196.6	2453	64.99
12/4/2020 16:10	201.8	200.1	195.6	2454	65.04
12/4/2020 16:10	201.8	200.2	195.9	2462	65.01
12/4/2020 16:10	201.8	199.7	196	2462	65.04
12/4/2020 16:10	201.8	200.4	195.9	2458	65.04
12/4/2020 16:10	201.9	199.7	195.7	2457	65.08
12/4/2020 16:10	201.9	199.9	196.5	2462	65.04
12/4/2020 16:10	201.8	200.3	196.6	2462	64.96
12/4/2020 16:10	201.9	200.4	195.6	2460	64.88
12/4/2020 16:10	201.9	199.9	195.8	2456	65.01
12/4/2020 16:10	201.9	199.6	196.2	2456	64.96
12/4/2020 16:10	201.9	199.8	195.9	2457	64.96
12/4/2020 16:10	202	199.6	195.7	2461	65.04
12/4/2020 16:10	201.8	200	196.6	2462	65

12/4/2020 16:10	201.9	200.4	196.4	2460	64.96
12/4/2020 16:10	201.9	199.7	195.8	2462	65.04
12/4/2020 16:10	201.8	199.7	196.2	2463	65
12/4/2020 16:10	201.9	200.3	196.2	2462	65
12/4/2020 16:10	201.9	199.7	196	2459	65.04
12/4/2020 16:10	201.9	200.1	196.2	2462	64.96
12/4/2020 16:10	201.9	199.7	196.9	2461	65.08
12/4/2020 16:11	201.9	200	195.7	2458	64.96
12/4/2020 16:11	201.9	199.6	196.1	2462	65
12/4/2020 16:11	201.9	199.5	196.2	2461	64.99
12/4/2020 16:11	201.9	200.2	196.1	2462	65.04
12/4/2020 16:11	201.9	199.7	196	2461	64.96
12/4/2020 16:11	201.9	199.8	196.9	2459	64.96
12/4/2020 16:11	201.9	199.7	196	2460	64.99
12/4/2020 16:11	201.9	199.7	195.8	2465	65.12
12/4/2020 16:11	202	200	196.1	2460	65.04
12/4/2020 16:11	201.9	199.7	196.3	2459	64.88
12/4/2020 16:11	202	200.4	195.8	2460	65.04
12/4/2020 16:11	202	200.3	196.6	2460	64.96
12/4/2020 16:11	201.9	199.6	196.5	2464	64.88
12/4/2020 16:11	202	200.3	195.7	2464	64.96
12/4/2020 16:11	202	199.4	196.3	2466	64.96
12/4/2020 16:11	202	200.1	196.2	2462	64.92
12/4/2020 16:11	202	200.4	196.1	2456	64.96
12/4/2020 16:11	202	199.7	196.1	2457	65.04
12/4/2020 16:11	202	199.9	196.8	2454	64.92
12/4/2020 16:11	201.9	199.7	195.8	2453	64.96
12/4/2020 16:11	202	199.7	195.9	2459	65.04
12/4/2020 16:11	202	200.4	196.1	2460	65
12/4/2020 16:11	202	199.6	196	2459	65.04
12/4/2020 16:11	202	200.3	196.1	2457	64.96
12/4/2020 16:11	202	199.8	196.9	2457	64.96
12/4/2020 16:11	202	200	195.8	2458	64.96
12/4/2020 16:11	202	200.3	196.1	2459	64.83
12/4/2020 16:11	202	199.6	196.2	2462	65
12/4/2020 16:11	202	200.4	196.4	2463	64.92
12/4/2020 16:11	202	199.7	196.1	2460	65
12/4/2020 16:11	202	199.8	196.9	2461	65
12/4/2020 16:11	202	199.8	195.7	2456	64.92
12/4/2020 16:11	202	199.7	195.7	2454	64.96
12/4/2020 16:11	202	200.2	196.4	2456	64.96
12/4/2020 16:11	202	199.6	196.1	2458	64.99
12/4/2020 16:11	202	199.9	195.8	2456	64.92
12/4/2020 16:11	202	200.3	196.8	2456	64.92
12/4/2020 16:11	202	199.6	196.2	2462	64.92
12/4/2020 16:11	202	200.4	195.7	2461	64.88
12/4/2020 16:11	202.1	200	196.2	2456	64.88

12/4/2020 16:11	202	200.4	196.2	2462	65.16
12/4/2020 16:11	202	200.2	196.1	2459	64.96
12/4/2020 16:11	202	199.6	196.4	2455	64.92
12/4/2020 16:11	202	200.2	196.6	2459	64.96
12/4/2020 16:11	202	200.2	195.7	2457	64.92
12/4/2020 16:11	202	200	196	2457	65.04
12/4/2020 16:11	202	200.4	196.2	2457	65
12/4/2020 16:11	202	199.9	196.1	2458	64.96
12/4/2020 16:11	202	200.1	196.1	2456	64.92
12/4/2020 16:11	202	199.9	197	2459	65
12/4/2020 16:11	202	199.8	196	2459	65.05
12/4/2020 16:11	202	200.4	195.9	2454	64.96
12/4/2020 16:11	202	200.4	196	2452	64.92
12/4/2020 16:11	202	200.3	196.1	2452	65
12/4/2020 16:11	202.1	199.8	195.9	2453	65
12/4/2020 16:11	202	199.8	196.5	2457	64.92
12/4/2020 16:11	202.1	200.1	196.7	2459	64.92
12/4/2020 16:11	202	200.4	195.6	2459	65
12/4/2020 16:11	202	200.4	195.9	2459	64.96
12/4/2020 16:11	202	199.6	196.2	2461	65
12/4/2020 16:12	202	199.8	196.2	2463	64.96
12/4/2020 16:12	202.1	200.4	195.8	2464	64.96
12/4/2020 16:12	202	199.6	196.8	2463	64.96
12/4/2020 16:12	202	200.2	196.4	2458	65.04
12/4/2020 16:12	202	199.7	195.9	2460	65.04
12/4/2020 16:12	202.1	200.4	195.9	2459	64.99
12/4/2020 16:12	202	200.2	196.2	2464	64.92
12/4/2020 16:12	202.1	199.7	196.2	2461	65.04
12/4/2020 16:12	202	200.1	195.9	2458	64.83
12/4/2020 16:12	202	199.7	196.9	2459	65.04
12/4/2020 16:12	202	200.4	196.1	2458	65.04
12/4/2020 16:12	202	200.4	195.6	2461	64.88
12/4/2020 16:12	202	199.6	196.2	2464	64.96
12/4/2020 16:12	202	200.3	196.1	2458	65.17
12/4/2020 16:12	202.1	199.7	195.9	2463	64.83
12/4/2020 16:12	202	199.7	196.2	2461	65
12/4/2020 16:12	202.1	200.4	196.8	2466	65.12
12/4/2020 16:12	202.1	199.6	196.2	2462	65
12/4/2020 16:12	202	200.4	195.9	2463	65
12/4/2020 16:12	202.1	200.1	196	2461	65.04
12/4/2020 16:12	202	200.4	196.1	2463	64.96
12/4/2020 16:12	202.1	200.2	195.7	2465	64.92
12/4/2020 16:12	202	200.2	196.4	2463	65.04
12/4/2020 16:12	202	200.4	196.9	2462	64.96
12/4/2020 16:12	202.1	200.1	195.7	2463	64.96
12/4/2020 16:12	202	200	196.1	2464	65
12/4/2020 16:12	202.1	200.4	196.3	2463	65.05

12/4/2020 16:12	202.1	199.6	196	2465	65
12/4/2020 16:12	202.1	200.2	195.9	2463	64.96
12/4/2020 16:12	202.1	199.9	196.5	2467	65.05
12/4/2020 16:12	202.1	199.7	196.5	2462	64.88
12/4/2020 16:12	202.1	199.7	195.9	2465	65.04
12/4/2020 16:12	202.1	199.7	195.9	2463	65.08
12/4/2020 16:12	202.1	200.2	196.3	2465	65.04
12/4/2020 16:12	202.2	199.6	196.1	2461	65.04
12/4/2020 16:12	202.2	200.4	196.4	2459	65
12/4/2020 16:12	202.2	200.2	196.9	2459	64.92
12/4/2020 16:12	202.2	199.7	195.7	2463	65.04
12/4/2020 16:12	202.1	200.2	195.9	2460	64.88
12/4/2020 16:12	202.1	199.6	196.1	2467	65.04
12/4/2020 16:12	202.1	199.7	196.1	2465	65.03
12/4/2020 16:12	202.2	200.4	196.2	2464	64.84
12/4/2020 16:12	202.2	199.7	196.2	2469	65.08
12/4/2020 16:12	202.3	200	196.9	2467	64.99
12/4/2020 16:12	202.2	200.2	196.6	2462	65.04
12/4/2020 16:12	202.2	199.9	195.9	2461	64.84
12/4/2020 16:12	202.2	200.4	196.2	2455	64.83
12/4/2020 16:12	202.2	199.7	196.3	2456	65.03
12/4/2020 16:12	202.2	200	196.4	2456	64.92
12/4/2020 16:12	202.2	199.7	196	2456	64.96
12/4/2020 16:12	202.2	199.8	196.7	2455	65.03
12/4/2020 16:12	202.2	200.4	197	2456	64.96
12/4/2020 16:12	202.2	199.7	195.9	2462	65.04
12/4/2020 16:12	202.2	200.3	195.9	2462	64.96
12/4/2020 16:12	202.3	199.6	196.3	2458	64.99
12/4/2020 16:12	202.2	199.6	196.4	2459	64.96
12/4/2020 16:12	202.3	200.4	196.3	2459	64.96
12/4/2020 16:12	202.2	200.3	195.9	2465	64.96
12/4/2020 16:12	202.3	200	196.8	2466	65.04
12/4/2020 16:13	202.3	200	197.1	2464	65
12/4/2020 16:13	202.3	199.7	196	2461	64.96
12/4/2020 16:13	202.2	200.4	195.9	2463	64.96
12/4/2020 16:13	202.2	200.1	196.2	2464	65
12/4/2020 16:13	202.3	200.3	196.2	2463	65
12/4/2020 16:13	202.3	200.1	196.4	2462	65
12/4/2020 16:13	202.3	199.6	196.2	2463	65.04
12/4/2020 16:13	202.2	200.4	196.1	2463	64.88
12/4/2020 16:13	202.3	199.7	197	2463	65.04
12/4/2020 16:13	202.3	200.1	196.8	2463	65
12/4/2020 16:13	202.4	199.8	196.1	2464	65
12/4/2020 16:13	202.3	199.6	196.1	2463	65.04
12/4/2020 16:13	202.3	200.4	196.4	2463	64.96
12/4/2020 16:13	202.3	199.6	196.5	2470	65.04
12/4/2020 16:13	202.4	199.9	196	2467	64.96

12/4/2020 16:13	202.3	199.7	197	2469	64.96
12/4/2020 16:13	202.4	200.4	196.6	2471	65.08
12/4/2020 16:13	202.3	200.5	195.8	2471	64.99
12/4/2020 16:13	202.3	199.6	196.4	2469	64.99
12/4/2020 16:13	202.4	200.1	196.2	2470	65.12
12/4/2020 16:13	202.3	200.3	196.1	2472	64.99
12/4/2020 16:13	202.4	200.4	196.6	2473	64.99
12/4/2020 16:13	202.3	200	196.9	2476	65.08
12/4/2020 16:13	202.3	199.6	195.9	2473	64.88
12/4/2020 16:13	202.3	200.1	196.1	2471	65.04
12/4/2020 16:13	202.3	199.6	196.4	2474	65
12/4/2020 16:13	202.3	200.4	196.2	2471	64.99
12/4/2020 16:13	202.4	200.4	196.1	2469	64.96
12/4/2020 16:13	202.4	199.7	196.9	2469	64.99
12/4/2020 16:13	202.4	199.9	196.6	2463	64.96
12/4/2020 16:13	202.3	200	195.9	2465	64.95
12/4/2020 16:13	202.3	200.3	196.4	2467	64.96
12/4/2020 16:13	202.4	200.4	196.3	2467	65
12/4/2020 16:13	202.3	199.6	196.2	2466	64.87
12/4/2020 16:13	202.3	200.4	196.4	2469	64.96
12/4/2020 16:13	202.3	199.6	197.1	2470	65.04
12/4/2020 16:13	202.3	199.6	195.9	2473	64.92
12/4/2020 16:13	202.3	200.3	195.9	2471	64.96
12/4/2020 16:13	202.4	199.9	196.2	2471	65
12/4/2020 16:13	202.3	200.2	196.3	2473	65
12/4/2020 16:13	202.4	199.6	196.1	2470	65
12/4/2020 16:13	202.4	200.2	196.6	2471	64.96
12/4/2020 16:13	202.4	200.4	197.1	2471	65
12/4/2020 16:13	202.4	199.9	195.9	2471	64.96
12/4/2020 16:13	202.4	200.2	195.9	2471	65
12/4/2020 16:13	202.4	199.6	196.4	2473	65
12/4/2020 16:13	202.4	200.4	196.4	2469	65.08
12/4/2020 16:13	202.4	200.4	196.2	2470	64.88
12/4/2020 16:13	202.4	199.9	196.7	2472	65.08
12/4/2020 16:13	202.4	200.2	196.9	2471	64.99
12/4/2020 16:13	202.4	199.6	195.9	2470	64.84
12/4/2020 16:13	202.4	200.2	196.1	2473	65.08
12/4/2020 16:13	202.4	200.4	196.4	2466	64.88
12/4/2020 16:13	202.4	199.8	196.3	2464	64.99
12/4/2020 16:13	202.4	199.8	196.2	2467	65.12
12/4/2020 16:13	202.4	199.6	196.1	2469	64.88
12/4/2020 16:13	202.4	200.4	197	2470	65
12/4/2020 16:13	202.4	200.4	196.4	2473	64.92
12/4/2020 16:13	202.4	199.8	195.9	2477	65.05
12/4/2020 16:13	202.4	200.4	196.4	2473	64.96
12/4/2020 16:14	202.4	200.4	196.3	2474	64.96
12/4/2020 16:14	202.4	200.2	196.1	2473	65.08

12/4/2020 16:14	202.4	200.4	196.7	2469	64.92
12/4/2020 16:14	202.4	199.9	197	2469	65
12/4/2020 16:14	202.4	199.8	195.8	2471	65
12/4/2020 16:14	202.4	199.7	196.2	2471	64.92
12/4/2020 16:14	202.4	200.4	196.3	2469	65
12/4/2020 16:14	202.4	200.1	196.3	2468	64.96
12/4/2020 16:14	202.4	199.9	196.2	2464	64.96
12/4/2020 16:14	202.4	199.9	196.5	2464	64.96
12/4/2020 16:14	202.4	200.4	197.1	2465	64.96
12/4/2020 16:14	202.5	199.7	196	2464	65
12/4/2020 16:14	202.4	200.4	195.8	2462	64.96
12/4/2020 16:14	202.4	200	196.5	2463	64.92
12/4/2020 16:14	202.4	200.4	196.2	2467	64.96
12/4/2020 16:14	202.4	200.4	196.1	2467	64.96
12/4/2020 16:14	202.5	199.6	196.6	2467	64.96
12/4/2020 16:14	202.4	200.4	196.9	2469	64.96
12/4/2020 16:14	202.5	199.7	195.9	2466	64.96
12/4/2020 16:14	202.5	200.4	196	2462	64.99
12/4/2020 16:14	202.5	199.6	196.4	2466	64.96
12/4/2020 16:14	202.4	199.6	196.4	2466	65.04
12/4/2020 16:14	202.4	200.3	196	2462	65.04
12/4/2020 16:14	202.5	199.7	196.4	2463	65.04
12/4/2020 16:14	202.4	199.9	197.1	2466	64.96
12/4/2020 16:14	202.5	199.6	196.4	2469	65
12/4/2020 16:14	202.5	200.2	196.1	2466	65.04
12/4/2020 16:14	202.5	200.4	196.1	2463	64.96
12/4/2020 16:14	202.5	199.6	196.4	2463	65.08
12/4/2020 16:14	202.4	200.1	196.4	2462	64.99
12/4/2020 16:14	202.5	200.4	196.4	2463	64.92
12/4/2020 16:14	202.5	199.6	196.4	2463	64.99
12/4/2020 16:14	202.4	200.2	196.5	2467	65.04
12/4/2020 16:14	202.5	199.8	197	2463	64.92
12/4/2020 16:14	202.5	200.4	196.9	2458	64.83
12/4/2020 16:14	202.5	199.5	196.1	2461	64.92
12/4/2020 16:14	202.6	199.6	196.1	2463	65.04
12/4/2020 16:14	202.5	200.4	196.4	2460	64.83
12/4/2020 16:14	202.5	199.9	196.4	2462	65
12/4/2020 16:14	202.5	199.9	196.6	2462	65.12
12/4/2020 16:14	202.5	200.2	196.4	2461	64.92
12/4/2020 16:14	202.5	199.6	196.3	2462	64.99
12/4/2020 16:14	202.6	200.4	197.1	2464	65.12
12/4/2020 16:14	202.6	199.9	197.1	2466	64.99
12/4/2020 16:14	202.6	200.2	196.2	2467	64.99
12/4/2020 16:14	202.6	199.6	196.1	2467	64.96
12/4/2020 16:14	202.5	199.6	196	2467	64.96
12/4/2020 16:14	202.5	200.4	196.4	2465	65.04
12/4/2020 16:14	202.5	199.6	196.4	2465	65.09

12/4/2020 16:14	202.6	200.4	196.7	2463	64.96
12/4/2020 16:14	202.6	200.3	196.4	2459	65
12/4/2020 16:14	202.6	199.7	196.4	2459	65.04
12/4/2020 16:14	202.6	200.4	196.5	2460	64.92
12/4/2020 16:14	202.6	199.9	196.4	2456	64.91
12/4/2020 16:14	202.6	200	196.9	2458	64.96
12/4/2020 16:14	202.6	200.4	197.2	2458	64.96
12/4/2020 16:14	202.6	199.6	196.9	2452	64.92
12/4/2020 16:14	202.6	199.9	196.5	2452	64.96
12/4/2020 16:14	202.6	199.7	195.9	2456	65.04
12/4/2020 16:15	202.6	199.9	196.1	2457	64.84
12/4/2020 16:15	202.6	200.3	196.2	2458	64.92
12/4/2020 16:15	202.7	199.7	196.4	2459	64.96
12/4/2020 16:15	202.6	199.9	196.6	2463	64.92
12/4/2020 16:15	202.6	199.7	196.4	2461	64.92
12/4/2020 16:15	202.6	200.1	196.3	2463	64.99
12/4/2020 16:15	202.6	200.3	196.4	2465	65
12/4/2020 16:15	202.6	200.3	196.2	2462	65.08
12/4/2020 16:15	202.6	199.9	196.6	2461	64.96
12/4/2020 16:15	202.6	199.7	197.1	2461	65.08
12/4/2020 16:15	202.6	200	197.2	2460	65.04
12/4/2020 16:15	202.6	200.4	196.9	2463	64.88
12/4/2020 16:15	202.6	200	195.9	2462	65.08
12/4/2020 16:15	202.6	200.3	196.2	2456	64.92
12/4/2020 16:15	202.6	200.2	196.4	2457	64.92
12/4/2020 16:15	202.7	199.7	196.4	2460	65.08
12/4/2020 16:15	202.6	200.4	196.5	2460	64.88
12/4/2020 16:15	202.6	200.2	196.2	2460	64.92
12/4/2020 16:15	202.6	200.2	196.6	2461	65.04
12/4/2020 16:15	202.6	199.9	197.2	2458	64.83
12/4/2020 16:15	202.7	200.1	196	2458	64.96
12/4/2020 16:15	202.6	199.7	196.6	2457	64.92
12/4/2020 16:15	202.7	199.6	196.5	2459	65
12/4/2020 16:15	202.7	200.4	196.3	2460	64.96
12/4/2020 16:15	202.6	199.9	196.8	2458	65
12/4/2020 16:15	202.6	199.9	197.2	2458	64.96
12/4/2020 16:15	202.6	199.5	196.3	2456	64.96
12/4/2020 16:15	202.7	200.2	196.4	2459	64.92
12/4/2020 16:15	202.8	200.4	196.4	2464	65
12/4/2020 16:15	202.8	200	196.4	2462	64.92
12/4/2020 16:15	202.6	200.1	196.5	2465	64.96
12/4/2020 16:15	202.6	199.6	197.4	2465	64.92
12/4/2020 16:15	202.6	199.6	196.5	2466	65
12/4/2020 16:15	202.6	200.3	196.2	2463	64.88
12/4/2020 16:15	202.7	200.1	196.6	2464	65.12
12/4/2020 16:15	202.6	200.4	196.6	2459	64.88
12/4/2020 16:15	202.7	199.8	196.4	2460	64.88

12/4/2020 16:15	202.6	200.1	197.3	2467	64.96
12/4/2020 16:15	202.7	200.2	197.1	2469	64.88
12/4/2020 16:15	202.6	199.9	196	2470	65.04
12/4/2020 16:15	202.7	199.9	196.6	2470	64.92
12/4/2020 16:15	202.7	200.3	196.7	2466	64.84
12/4/2020 16:15	202.6	199.6	196.5	2467	64.92
12/4/2020 16:15	202.7	200.4	196.5	2466	64.92
12/4/2020 16:15	202.7	199.9	197.2	2465	64.88
12/4/2020 16:15	202.7	200	196.8	2463	64.88
12/4/2020 16:15	202.6	200.4	196.3	2463	64.92
12/4/2020 16:15	202.7	200.4	196.4	2464	64.96
12/4/2020 16:15	202.7	200.4	196.8	2465	65.03
12/4/2020 16:15	202.7	199.7	196.4	2466	64.92
12/4/2020 16:15	202.7	200	196.4	2467	65.08
12/4/2020 16:15	202.6	200.2	197.4	2464	64.88
12/4/2020 16:15	202.7	200.2	196.4	2460	65.04
12/4/2020 16:15	202.7	200.1	196.4	2461	64.96
12/4/2020 16:15	202.7	199.7	196.6	2461	64.99
12/4/2020 16:15	202.7	199.7	196.4	2462	64.96
12/4/2020 16:15	202.7	199.5	196.4	2462	65.08
12/4/2020 16:15	202.8	200.4	196.7	2460	65
12/4/2020 16:15	202.7	200.4	197.2	2459	65
12/4/2020 16:15	202.7	199.9	196.4	2460	65.04
12/4/2020 16:16	202.7	200.3	196.4	2459	64.96
12/4/2020 16:16	202.7	200.3	196.6	2460	65.04
12/4/2020 16:16	202.8	199.6	196.6	2461	64.96
12/4/2020 16:16	202.8	200.1	196.4	2467	65
12/4/2020 16:16	202.8	199.7	197.2	2467	64.96
12/4/2020 16:16	202.7	200.2	196.4	2465	64.92
12/4/2020 16:16	202.8	199.9	196.4	2461	65
12/4/2020 16:16	202.8	199.7	196.7	2462	64.88
12/4/2020 16:16	202.7	200.4	196.5	2457	64.99
12/4/2020 16:16	202.7	199.6	196.4	2459	65.08
12/4/2020 16:16	202.8	200.4	197.2	2459	64.96
12/4/2020 16:16	202.7	200.1	196.6	2455	65.08
12/4/2020 16:16	202.7	199.5	196.4	2456	65.08
12/4/2020 16:16	202.8	200.2	196.5	2462	65.04
12/4/2020 16:16	202.7	199.8	196.6	2460	64.92
12/4/2020 16:16	202.8	199.9	196.4	2460	65.12
12/4/2020 16:16	202.7	200.4	196.9	2457	64.92
12/4/2020 16:16	202.8	199.6	197.2	2455	65.08
12/4/2020 16:16	202.8	200.3	196.1	2459	64.88
12/4/2020 16:16	202.8	199.8	196.5	2462	64.88
12/4/2020 16:16	202.8	200.4	196.4	2460	65.08
12/4/2020 16:16	202.8	200.2	196.4	2462	65.08
12/4/2020 16:16	202.8	199.6	196.7	2460	65.08
12/4/2020 16:16	202.8	200.4	197.3	2460	65.03

12/4/2020 16:16	202.8	199.9	196.1	2466	64.95
12/4/2020 16:16	202.8	199.9	196.4	2466	64.96
12/4/2020 16:16	202.8	200.3	196.5	2464	64.88
12/4/2020 16:16	202.7	199.7	196.6	2462	64.96
12/4/2020 16:16	202.8	200.3	196.6	2460	65
12/4/2020 16:16	202.8	199.7	197.2	2459	65.04
12/4/2020 16:16	202.8	200	196.4	2457	64.96
12/4/2020 16:16	202.8	200.2	196.4	2458	65.04
12/4/2020 16:16	202.8	199.7	196.6	2456	64.92
12/4/2020 16:16	202.8	200.1	196.6	2457	64.96
12/4/2020 16:16	202.7	199.7	196.4	2461	65.04
12/4/2020 16:16	202.8	200.4	197.4	2461	65
12/4/2020 16:16	202.8	200.2	196.9	2463	64.96
12/4/2020 16:16	202.8	199.5	196.3	2466	65
12/4/2020 16:16	202.8	200.4	196.9	2466	65.04
12/4/2020 16:16	202.8	199.7	196.8	2464	64.96
12/4/2020 16:16	202.8	199.7	196.5	2465	65
12/4/2020 16:16	202.8	199.7	197.4	2461	64.96
12/4/2020 16:16	202.8	199.6	196.9	2461	64.96
12/4/2020 16:16	202.8	200.2	196.6	2466	64.84
12/4/2020 16:16	202.8	199.9	196.8	2464	65.08
12/4/2020 16:16	202.8	200.4	196.9	2465	64.88
12/4/2020 16:16	202.8	199.5	196.6	2463	64.96
12/4/2020 16:16	202.7	199.9	196.9	2467	64.92
12/4/2020 16:16	202.8	200.4	197.3	2465	64.88
12/4/2020 16:16	202.8	199.9	196.4	2459	65
12/4/2020 16:16	202.8	199.8	196.4	2457	65.04
12/4/2020 16:16	202.8	199.7	196.7	2457	64.99
12/4/2020 16:16	202.8	199.6	196.8	2455	64.92
12/4/2020 16:16	202.8	200.2	196.6	2458	65.08
12/4/2020 16:16	202.8	200	197.5	2459	65.03
12/4/2020 16:16	202.8	199.9	196.3	2457	64.92
12/4/2020 16:16	202.7	200.4	196.4	2457	64.96
12/4/2020 16:16	202.8	199.7	196.9	2460	64.88
12/4/2020 16:16	202.8	200.1	196.7	2462	64.99
12/4/2020 16:17	202.8	199.6	196.7	2464	65.08
12/4/2020 16:17	202.8	199.6	197.5	2466	65.04
12/4/2020 16:17	202.8	200.4	196.4	2463	64.96
12/4/2020 16:17	202.8	200.2	196.4	2463	64.96
12/4/2020 16:17	202.8	200.4	196.6	2463	64.96
12/4/2020 16:17	202.8	200.1	196.8	2464	65
12/4/2020 16:17	202.8	199.6	196.4	2463	64.96
12/4/2020 16:17	202.8	200.4	197.1	2467	65
12/4/2020 16:17	202.8	199.7	197.2	2463	64.96
12/4/2020 16:17	202.8	200.4	196.4	2461	64.87
12/4/2020 16:17	202.8	199.9	196.6	2466	65.04
12/4/2020 16:17	202.8	199.6	197	2462	64.92

12/4/2020 16:17	202.8	200.4	196.5	2459	65
12/4/2020 16:17	202.8	199.9	196.9	2459	65
12/4/2020 16:17	202.8	200.1	197.5	2463	64.99
12/4/2020 16:17	202.8	200.1	196.3	2461	64.99
12/4/2020 16:17	202.8	200.4	196.6	2463	65.05
12/4/2020 16:17	202.8	200.3	196.6	2460	64.99
12/4/2020 16:17	202.8	199.6	196.6	2462	65.08
12/4/2020 16:17	202.9	200.3	196.8	2466	65.04
12/4/2020 16:17	202.9	199.7	197.6	2462	64.99
12/4/2020 16:17	202.9	199.6	196.4	2460	65
12/4/2020 16:17	202.9	199.7	196.5	2462	65.04
12/4/2020 16:17	202.9	200	196.8	2465	64.92
12/4/2020 16:17	202.9	200.1	196.8	2467	65.08
12/4/2020 16:17	202.9	200.1	196.4	2466	64.99
12/4/2020 16:17	202.9	200.4	197.5	2470	65.08
12/4/2020 16:17	202.9	200.3	197.2	2468	65.04
12/4/2020 16:17	202.9	199.7	196.4	2466	65.03
12/4/2020 16:17	202.9	199.9	196.6	2468	65.04
12/4/2020 16:17	202.9	199.7	196.8	2468	65.03
12/4/2020 16:17	202.9	199.7	196.7	2465	65.04
12/4/2020 16:17	202.9	200.4	196.5	2461	64.92
12/4/2020 16:17	202.9	199.9	197.5	2462	64.83
12/4/2020 16:17	202.9	200.2	196.5	2464	64.99
12/4/2020 16:17	202.9	200.2	196.6	2463	65
12/4/2020 16:17	203	199.9	196.6	2463	65.08
12/4/2020 16:17	202.9	200.4	196.8	2462	65
12/4/2020 16:17	202.8	199.9	196.4	2463	64.96
12/4/2020 16:17	202.9	200	197.4	2462	64.92
12/4/2020 16:17	203	199.7	196.9	2466	64.99
12/4/2020 16:17	202.9	199.6	196.4	2463	64.99
12/4/2020 16:17	203	200.4	196.8	2466	65.04
12/4/2020 16:17	202.9	199.9	196.9	2465	64.96
12/4/2020 16:17	203	200.4	196.9	2464	64.92
12/4/2020 16:17	203	200.2	196.5	2466	64.96
12/4/2020 16:17	202.9	199.7	197.3	2459	64.96
12/4/2020 16:17	203	200.4	197.3	2459	64.91
12/4/2020 16:17	203	199.9	196.5	2463	65
12/4/2020 16:17	203	199.8	196.7	2462	64.91
12/4/2020 16:17	202.9	200.3	196.8	2459	64.96
12/4/2020 16:17	203	200.4	196.8	2462	65.04
12/4/2020 16:17	203	200.1	196.6	2465	64.96
12/4/2020 16:17	203	199.7	197.6	2466	64.96
12/4/2020 16:17	203	199.9	196.9	2464	65
12/4/2020 16:17	203	200.1	196.5	2463	64.96
12/4/2020 16:17	203	199.6	196.8	2460	64.92
12/4/2020 16:17	203	200.4	197.1	2462	64.96
12/4/2020 16:17	203	199.8	196.8	2463	65

12/4/2020 16:18	203	200.5	197.1	2459	64.87
12/4/2020 16:18	203	200.1	197.5	2460	64.96
12/4/2020 16:18	203	199.6	196.4	2459	65
12/4/2020 16:18	203	200.4	196.7	2461	64.91
12/4/2020 16:18	203	199.8	196.9	2460	64.96
12/4/2020 16:18	203	200.3	196.9	2463	65.04
12/4/2020 16:18	203	200.1	196.6	2466	64.88
12/4/2020 16:18	203	200.2	197.5	2467	65.08
12/4/2020 16:18	203	200.4	197.2	2469	65.08
12/4/2020 16:18	203.1	199.8	196.5	2471	64.96
12/4/2020 16:18	203.1	199.9	196.8	2469	65.04
12/4/2020 16:18	203	199.6	196.8	2466	64.96
12/4/2020 16:18	203	199.7	196.9	2470	65.08
12/4/2020 16:18	203	200.5	196.8	2467	64.92
12/4/2020 16:18	203	199.9	197.5	2470	64.92
12/4/2020 16:18	203	200.4	196.9	2471	65.12
12/4/2020 16:18	203	200	196.6	2469	64.92
12/4/2020 16:18	203	199.6	196.9	2471	64.96
12/4/2020 16:18	203	200.4	196.8	2471	65.05
12/4/2020 16:18	203	200	196.6	2469	64.96
12/4/2020 16:18	203	200.4	197.2	2467	65
12/4/2020 16:18	203	200.2	197.5	2468	65.13
12/4/2020 16:18	203.1	199.7	196.4	2464	65.08
12/4/2020 16:18	203	199.7	196.7	2466	65.04
12/4/2020 16:18	203	199.9	197.1	2467	65.04
12/4/2020 16:18	203.1	200.2	197.1	2468	65.12
12/4/2020 16:18	203.1	200.3	196.8	2470	65.04
12/4/2020 16:18	203	199.7	197.2	2470	64.92
12/4/2020 16:18	203.1	200.3	197.5	2471	65.21
12/4/2020 16:18	203.1	199.9	196.5	2469	65
12/4/2020 16:18	203.1	200.9	196.8	2471	65.08
12/4/2020 16:18	203.1	201.4	196.9	2469	65.04
12/4/2020 16:18	203.1	200.8	196.9	2467	64.99
12/4/2020 16:18	203	201.1	196.5	2470	64.96
12/4/2020 16:18	203.1	201.2	197.4	2471	65.09
12/4/2020 16:18	203	201.2	197.4	2474	65
12/4/2020 16:18	203.1	201.3	196.4	2470	65
12/4/2020 16:18	203	200.8	196.8	2468	65.09
12/4/2020 16:18	203.1	201.1	197.1	2470	64.96
12/4/2020 16:18	203.1	200.8	197.1	2478	64.92
12/4/2020 16:18	203.1	200.8	196.9	2482	65.08
12/4/2020 16:18	203.1	201.3	197.3	2481	65.05
12/4/2020 16:18	203.1	201.2	197.6	2482	65.04
12/4/2020 16:18	203.1	201.1	196.6	2479	65
12/4/2020 16:18	203.1	200.8	196.8	2476	65.01
12/4/2020 16:18	203.1	201.5	197.1	2475	64.96
12/4/2020 16:18	203.1	201.4	196.9	2476	64.96

12/4/2020 16:18	203.1	201.1	196.9	2476	65
12/4/2020 16:18	203.2	201.1	197.8	2474	65.04
12/4/2020 16:18	203.2	200.9	197.1	2474	65.04
12/4/2020 16:18	203.1	201.1	196.8	2478	65.09
12/4/2020 16:18	203.2	201.5	197.1	2478	64.96
12/4/2020 16:18	203.1	201.2	197.1	2477	64.92
12/4/2020 16:18	203.1	201.1	196.9	2474	65.09
12/4/2020 16:18	203.2	201.1	196.8	2471	64.96
12/4/2020 16:18	203.1	200.9	197.7	2473	65.01
12/4/2020 16:18	203.1	200.7	197.4	2472	65.17
12/4/2020 16:18	203.2	200.8	196.6	2470	65.08
12/4/2020 16:18	203.2	201.2	196.8	2476	65.05
12/4/2020 16:18	203.1	200.9	197	2475	64.92
12/4/2020 16:19	203.1	200.9	196.9	2478	65.04
12/4/2020 16:19	203.1	200.9	197.1	2474	65.08
12/4/2020 16:19	203.1	200.8	196.9	2475	64.92
12/4/2020 16:19	203.1	201.1	196.9	2478	64.96
12/4/2020 16:19	203.2	200.8	197.5	2480	65
12/4/2020 16:19	203.1	201	197.6	2478	64.96
12/4/2020 16:19	203.2	201.4	196.8	2481	65.13
12/4/2020 16:19	203.2	200.8	196.6	2482	64.92
12/4/2020 16:19	203.2	201.1	196.9	2482	64.96
12/4/2020 16:19	203.2	200.7	197.1	2485	65.09
12/4/2020 16:19	203.2	200.8	196.9	2482	65
12/4/2020 16:19	203.2	201.3	196.8	2478	64.92
12/4/2020 16:19	203.2	200.9	197.8	2480	65.17
12/4/2020 16:19	203.2	201.3	196.9	2478	65.08
12/4/2020 16:19	203.2	200.7	196.8	2479	64.96
12/4/2020 16:19	203.2	200.9	196.9	2481	65.17
12/4/2020 16:19	203.3	201.3	197.1	2480	65
12/4/2020 16:19	203.2	201.1	196.7	2476	64.96
12/4/2020 16:19	203.2	201.1	196.9	2478	65
12/4/2020 16:19	203.3	200.9	197.8	2477	65.05
12/4/2020 16:19	203.2	200.8	197.4	2474	64.96
12/4/2020 16:19	203.2	200.8	196.7	2474	65.04
12/4/2020 16:19	203.2	201.1	196.7	2476	65.09
12/4/2020 16:19	203.3	201.3	197.1	2473	65.04
12/4/2020 16:19	203.2	201.2	197	2470	65.04
12/4/2020 16:19	203.3	201.4	196.6	2475	65.12
12/4/2020 16:19	203.2	201.3	197	2475	64.92
12/4/2020 16:19	203.3	201.1	197.9	2475	65
12/4/2020 16:19	203.2	201.1	196.9	2478	65.08
12/4/2020 16:19	203.3	201	196.8	2480	64.92
12/4/2020 16:19	203.2	201.3	197	2478	64.92
12/4/2020 16:19	203.3	200.8	197	2478	65.16
12/4/2020 16:19	203.3	200.8	197.1	2478	64.96
12/4/2020 16:19	203.3	201.2	197.2	2476	65.04

12/4/2020 16:19	203.3	201.2	197.9	2478	64.92
12/4/2020 16:19	203.3	201.3	197	2476	65.04
12/4/2020 16:19	203.3	201	196.8	2472	65.04
12/4/2020 16:19	203.3	201	197.1	2471	65
12/4/2020 16:19	203.3	201.3	197.3	2470	64.96
12/4/2020 16:19	203.3	201.1	197	2465	64.92
12/4/2020 16:19	203.4	201.5	197.6	2464	64.92
12/4/2020 16:19	203.3	200.9	197.8	2471	65
12/4/2020 16:19	203.3	201	196.9	2472	64.96
12/4/2020 16:19	203.4	201.1	197	2473	64.92
12/4/2020 16:19	203.3	200.8	197.3	2471	65
12/4/2020 16:19	203.3	201.3	197.3	2470	65.04
12/4/2020 16:19	203.4	201.2	197.2	2470	65.04
12/4/2020 16:19	203.4	200.9	197.3	2471	65
12/4/2020 16:19	203.3	200.9	197.8	2466	65.08
12/4/2020 16:19	203.4	201.3	196.9	2467	65.03
12/4/2020 16:19	203.4	201	196.8	2463	65.03
12/4/2020 16:19	203.4	201.4	197.3	2470	64.93
12/4/2020 16:19	203.4	200.8	197.2	2474	64.92
12/4/2020 16:19	203.3	201.1	196.9	2476	64.88
12/4/2020 16:19	203.4	200.6	197.6	2473	65.05
12/4/2020 16:19	203.3	201.1	197.7	2472	64.99
12/4/2020 16:19	203.4	201.3	196.8	2473	65.08
12/4/2020 16:19	203.3	201.2	196.9	2473	65.16
12/4/2020 16:19	203.4	201.3	197.2	2479	65.03
12/4/2020 16:20	203.4	200.8	197.3	2478	65.04
12/4/2020 16:20	203.4	200.8	196.9	2476	65.04
12/4/2020 16:20	203.4	201.1	197.6	2473	65.04
12/4/2020 16:20	203.4	200.9	197.9	2475	65.04
12/4/2020 16:20	203.3	201.3	196.9	2472	64.96
12/4/2020 16:20	203.4	200.8	196.8	2478	64.96
12/4/2020 16:20	203.3	201.1	197.2	2474	64.92
12/4/2020 16:20	203.4	201.4	197.3	2473	64.96
12/4/2020 16:20	203.4	200.8	197.1	2480	65.09
12/4/2020 16:20	203.4	201.2	197.6	2477	65
12/4/2020 16:20	203.4	201.2	197.6	2473	65
12/4/2020 16:20	203.4	201.3	196.6	2473	65.09
12/4/2020 16:20	203.4	200.8	197	2478	64.96
12/4/2020 16:20	203.5	200.8	197.1	2478	65
12/4/2020 16:20	203.4	201.3	197.1	2476	65
12/4/2020 16:20	203.4	201.1	197.4	2477	65.08
12/4/2020 16:20	203.4	200.9	197.9	2474	64.92
12/4/2020 16:20	203.4	200.9	196.7	2474	65.04
12/4/2020 16:20	203.4	200.6	197.1	2478	65.01
12/4/2020 16:20	203.5	201.2	197.3	2478	64.99
12/4/2020 16:20	203.4	201.1	197.2	2478	64.88
12/4/2020 16:20	203.4	201.3	197.1	2477	65.12

12/4/2020 16:20	203.4	201.1	197.8	2476	65.04
12/4/2020 16:20	203.5	200.9	197.2	2476	64.88
12/4/2020 16:20	203.4	201.1	196.8	2476	64.93
12/4/2020 16:20	203.4	200.6	197	2473	65.12
12/4/2020 16:20	203.4	201.4	197.3	2474	64.88
12/4/2020 16:20	203.4	201.3	197.1	2476	64.84
12/4/2020 16:20	203.4	200.7	197.1	2475	64.84
12/4/2020 16:20	203.3	201.2	198	2477	64.92
12/4/2020 16:20	203.4	200.7	196.9	2477	64.84
12/4/2020 16:20	203.4	201.3	196.9	2481	65.05
12/4/2020 16:20	203.4	200.9	197.2	2481	64.83
12/4/2020 16:20	203.3	201.1	197.1	2478	64.96
12/4/2020 16:20	203.4	201.1	196.9	2477	65.09
12/4/2020 16:20	203.4	201.2	197.8	2475	65
12/4/2020 16:20	203.3	201.1	197.5	2477	65
12/4/2020 16:20	203.3	200.9	197.1	2481	64.96
12/4/2020 16:20	203.3	200.6	197.3	2487	64.92
12/4/2020 16:20	203.4	201.2	197.2	2483	64.96
12/4/2020 16:20	203.3	201.1	197.4	2479	64.92
12/4/2020 16:20	203.3	200.6	197.1	2481	65.04
12/4/2020 16:20	203.5	200.9	198	2476	64.96
12/4/2020 16:20	203.4	200.9	197.3	2478	64.92
12/4/2020 16:20	203.4	201.1	196.9	2482	65.05
12/4/2020 16:20	203.4	201.2	197.3	2478	64.99
12/4/2020 16:20	203.4	201.2	196.5	2478	65.08
12/4/2020 16:20	203.4	200.9	197.4	2482	65
12/4/2020 16:20	203.4	200.9	197.3	2482	64.99
12/4/2020 16:20	203.3	201	198	2483	65.04
12/4/2020 16:20	203.4	201.5	197.3	2485	64.96
12/4/2020 16:20	203.4	201.2	197	2488	64.8
12/4/2020 16:20	203.5	201.3	197.2	2485	64.92
12/4/2020 16:20	203.4	200.9	197.4	2482	64.96
12/4/2020 16:20	203.3	201.2	197.2	2483	65.04
12/4/2020 16:20	203.5	200.7	197.4	2484	65.03
12/4/2020 16:20	203.4	201.3	198.2	2484	65.04
12/4/2020 16:20	203.3	201.2	197.6	2486	65.04
12/4/2020 16:20	203.4	200.8	196.9	2488	64.84
12/4/2020 16:20	203.4	201	197.1	2487	65
12/4/2020 16:21	203.4	200.8	197.2	2485	64.96
12/4/2020 16:21	203.3	201.3	197.4	2482	64.92
12/4/2020 16:21	203.5	201.5	197.3	2482	64.92
12/4/2020 16:21	203.4	200.8	197	2485	64.96
12/4/2020 16:21	203.4	200.9	197.9	2479	64.96
12/4/2020 16:21	203.3	201.3	197.8	2480	64.96
12/4/2020 16:21	203.4	201.2	197.1	2480	65
12/4/2020 16:21	203.4	201.3	197.1	2480	64.96
12/4/2020 16:21	203.4	200.9	197.2	2481	64.96

12/4/2020 16:21	203.4	201.1	197.3	2481	65
12/4/2020 16:21	203.4	201	197.3	2480	65
12/4/2020 16:21	203.5	201.2	197.2	2477	64.92
12/4/2020 16:21	203.5	201.2	198	2478	65
12/4/2020 16:21	203.5	200.9	197.3	2480	64.96
12/4/2020 16:21	203.5	201.1	197.1	2480	64.88
12/4/2020 16:21	203.5	200.8	197.2	2480	65.04
12/4/2020 16:21	203.4	200.9	197.5	2483	65
12/4/2020 16:21	203.4	201.4	197.2	2480	64.92
12/4/2020 16:21	203.5	200.8	197.4	2476	65.04
12/4/2020 16:21	203.5	201.1	198	2483	64.96
12/4/2020 16:21	203.4	201.5	197.1	2488	64.96
12/4/2020 16:21	203.5	200.8	197	2494	65
12/4/2020 16:21	203.4	201.1	197.4	2496	64.88
12/4/2020 16:21	203.5	201	197.4	2489	65.08
12/4/2020 16:21	203.5	201.4	197.2	2488	65.04
12/4/2020 16:21	203.5	201.2	197.8	2488	65.08
12/4/2020 16:21	203.5	200.8	197.6	2487	64.96
12/4/2020 16:21	203.4	201.1	197	2486	64.88
12/4/2020 16:21	203.4	201.1	197.2	2488	64.8
12/4/2020 16:21	203.5	201.3	197.4	2485	65.04
12/4/2020 16:21	203.5	201.3	197.3	2488	64.84
12/4/2020 16:21	203.5	200.8	197.2	2490	65.03
12/4/2020 16:21	203.4	201.2	197.3	2489	64.93
12/4/2020 16:21	203.5	200.8	198.2	2487	64.92
12/4/2020 16:21	203.4	201.2	197.1	2487	65.08
12/4/2020 16:21	203.5	201.3	197.1	2486	65
12/4/2020 16:21	203.5	200.8	197.3	2491	64.88
12/4/2020 16:21	203.5	201.1	197.2	2490	64.99
12/4/2020 16:21	203.4	200.8	197.4	2487	64.92
12/4/2020 16:21	203.4	200.9	197.1	2490	65
12/4/2020 16:21	203.4	201.3	198.1	2492	64.92
12/4/2020 16:21	203.5	200.8	197.3	2493	65
12/4/2020 16:21	203.5	201.1	196.9	2493	65
12/4/2020 16:21	203.6	200.8	197.3	2486	64.92
12/4/2020 16:21	203.5	200.9	197.3	2485	64.96
12/4/2020 16:21	203.4	201.3	197.3	2490	65
12/4/2020 16:21	203.5	200.7	197.2	2487	64.92
12/4/2020 16:21	203.5	201.1	198.1	2487	64.96
12/4/2020 16:21	203.4	200.9	197.5	2489	65.05
12/4/2020 16:21	203.5	200.8	197	2493	65
12/4/2020 16:21	203.5	200.8	197.3	2492	64.96
12/4/2020 16:21	203.4	201	197.3	2492	65.09
12/4/2020 16:21	203.4	201.4	197.3	2490	65.04
12/4/2020 16:21	203.4	200.9	196.9	2486	65.04
12/4/2020 16:21	203.5	200.7	197.6	2487	65.04
12/4/2020 16:21	203.4	201.3	198	2491	65.04

12/4/2020 16:21	203.4	201.1	197.1	2492	64.95
12/4/2020 16:21	203.4	201.3	197.2	2486	65.04
12/4/2020 16:21	203.4	201	197.3	2487	65.08
12/4/2020 16:22	203.5	200.8	197.5	2488	64.95
12/4/2020 16:22	203.5	201.3	197.4	2487	64.84
12/4/2020 16:22	203.5	201.1	197.1	2488	65.12
12/4/2020 16:22	203.4	201.4	197.6	2491	64.8
12/4/2020 16:22	203.4	200.8	198.1	2493	65.04
12/4/2020 16:22	203.5	200.8	197.5	2495	65
12/4/2020 16:22	203.5	201	197	2495	65.04
12/4/2020 16:22	203.5	200.9	197.2	2493	65.08
12/4/2020 16:22	203.5	200.9	197.5	2495	65.04
12/4/2020 16:22	203.4	201.1	197.3	2494	64.93
12/4/2020 16:22	203.5	201.3	197.5	2496	64.99
12/4/2020 16:22	203.5	200.9	197.2	2493	64.88
12/4/2020 16:22	203.4	200.8	197.3	2492	65.04
12/4/2020 16:22	203.5	201.2	198	2491	64.92
12/4/2020 16:22	203.5	201.2	198.3	2491	65.08
12/4/2020 16:22	203.5	201.3	197.5	2496	65.04
12/4/2020 16:22	203.5	200.9	197.2	2499	65
12/4/2020 16:22	203.5	200.9	197.1	2498	64.88
12/4/2020 16:22	203.5	201.1	197.2	2502	64.84
12/4/2020 16:22	203.5	200.6	197.5	2499	64.92
12/4/2020 16:22	203.5	201.1	197.6	2496	64.99
12/4/2020 16:22	203.5	201.3	197.6	2494	64.88
12/4/2020 16:22	203.5	201.1	197.6	2494	65.04
12/4/2020 16:22	203.5	201.3	197.3	2496	65.03
12/4/2020 16:22	203.5	201.4	197.3	2492	65.04
12/4/2020 16:22	203.5	200.8	197.3	2495	65.09
12/4/2020 16:22	203.5	201.2	197.6	2492	64.96
12/4/2020 16:22	203.5	200.9	197.7	2492	64.87
12/4/2020 16:22	203.5	201.4	197.8	2490	65.04
12/4/2020 16:22	203.5	200.6	198.1	2486	64.96
12/4/2020 16:22	203.5	200.9	198	2488	65
12/4/2020 16:22	203.5	201.2	198	2489	65.09
12/4/2020 16:22	203.6	200.9	198	2487	64.87
12/4/2020 16:22	203.5	200.9	197.9	2487	64.92
12/4/2020 16:22	203.5	201.2	197.8	2485	64.87
12/4/2020 16:22	203.6	200.8	197.6	2480	64.96
12/4/2020 16:22	203.5	201.2	197.3	2478	64.87
12/4/2020 16:22	203.5	200.9	197.2	2477	64.96
12/4/2020 16:22	203.6	201.2	197.2	2480	65
12/4/2020 16:22	203.5	201.2	197.4	2475	64.96
12/4/2020 16:22	203.5	200.8	197.5	2473	64.92
12/4/2020 16:22	203.5	200.9	197.5	2473	65
12/4/2020 16:22	203.5	200.8	197.6	2475	64.87
12/4/2020 16:22	203.5	201.1	197.4	2478	64.96

12/4/2020 16:22	203.6	201.3	197.3	2480	64.96
12/4/2020 16:22	203.5	200.8	197.3	2482	65
12/4/2020 16:22	203.5	201.1	197	2488	64.79
12/4/2020 16:22	203.5	200.8	197.1	2490	64.88
12/4/2020 16:22	203.6	200.9	197.5	2492	65.04
12/4/2020 16:22	203.5	200.8	198	2491	64.88
12/4/2020 16:22	203.5	201.1	198.3	2489	65.04
12/4/2020 16:22	203.6	201.3	198.2	2491	64.96
12/4/2020 16:22	203.5	200.8	198	2487	64.99
12/4/2020 16:22	203.6	200.9	197.6	2485	64.92
12/4/2020 16:22	203.6	201.3	197.5	2485	64.88
12/4/2020 16:22	203.6	201.1	197.5	2484	64.91
12/4/2020 16:22	203.6	201.2	197.4	2480	64.99
12/4/2020 16:22	203.5	200.9	197.1	2478	64.95
12/4/2020 16:22	203.5	201	197.3	2484	65.04
12/4/2020 16:23	203.6	201.3	197.5	2484	64.84
12/4/2020 16:23	203.6	201.2	197.5	2482	64.99
12/4/2020 16:23	203.6	201.2	197.5	2482	64.88
12/4/2020 16:23	203.6	200.9	197.5	2479	65.03
12/4/2020 16:23	203.5	201.2	197.6	2480	65.08
12/4/2020 16:23	203.6	200.8	197.4	2480	64.96
12/4/2020 16:23	203.5	200.7	197.4	2480	64.8
12/4/2020 16:23	203.6	201.1	197.6	2478	64.88
12/4/2020 16:23	203.5	200.9	197.4	2476	64.96
12/4/2020 16:23	203.6	201.3	197.6	2477	65.04
12/4/2020 16:23	203.6	200.9	197.6	2475	64.88
12/4/2020 16:23	203.6	200.6	197.5	2474	64.92
12/4/2020 16:23	203.6	201.2	197.5	2473	64.99
12/4/2020 16:23	203.6	200.9	197.5	2473	65.03
12/4/2020 16:23	203.6	200.8	197.5	2474	64.92
12/4/2020 16:23	203.6	200.8	197.6	2481	65.08
12/4/2020 16:23	203.6	201.1	197.5	2479	64.96
12/4/2020 16:23	203.6	201.3	197.6	2480	64.88
12/4/2020 16:23	203.5	201.1	197.2	2485	65.08
12/4/2020 16:23	203.6	200.8	197.2	2485	64.88
12/4/2020 16:23	203.6	201.4	197.6	2485	64.84
12/4/2020 16:23	203.5	201.1	198.2	2487	65.12
12/4/2020 16:23	203.5	201.1	197.8	2488	64.84
12/4/2020 16:23	203.6	201.1	197.4	2488	64.88
12/4/2020 16:23	203.6	200.8	197.2	2484	65.04
12/4/2020 16:23	203.6	201.3	197.2	2485	65.04
12/4/2020 16:23	203.6	200.9	197.6	2486	65.04
12/4/2020 16:23	203.6	201.3	197.5	2485	64.83
12/4/2020 16:23	203.5	201.1	197.6	2484	64.96
12/4/2020 16:23	203.5	201.3	197.5	2477	64.95
12/4/2020 16:23	203.5	200.9	197.4	2476	64.83
12/4/2020 16:23	203.5	200.9	197.9	2476	65

12/4/2020 16:23	203.6	201.2	198.3	2474	64.99
12/4/2020 16:23	203.5	201.2	198.2	2478	64.96
12/4/2020 16:23	203.6	201.3	197.9	2478	65.09
12/4/2020 16:23	203.6	200.9	197.3	2478	65
12/4/2020 16:23	203.6	200.8	197.2	2480	64.96
12/4/2020 16:23	203.6	201.2	197.3	2480	64.92
12/4/2020 16:23	203.6	201	197.6	2477	64.92
12/4/2020 16:23	203.5	200.9	197.6	2476	64.96
12/4/2020 16:23	203.6	200.8	197.6	2474	65
12/4/2020 16:23	203.6	200.8	197.7	2476	65.04
12/4/2020 16:23	203.6	201.1	197.5	2478	64.92
12/4/2020 16:23	203.6	201.2	197.4	2478	64.92
12/4/2020 16:23	203.6	201.4	197.4	2479	65.04
12/4/2020 16:23	203.6	200.9	197.6	2476	64.96
12/4/2020 16:23	203.6	200.8	198	2476	65
12/4/2020 16:23	203.6	201.3	198.3	2477	64.92
12/4/2020 16:23	203.6	200.9	198.5	2477	64.88
12/4/2020 16:23	203.6	201.2	198.3	2475	64.88
12/4/2020 16:23	203.7	201.1	198.3	2478	64.99
12/4/2020 16:23	203.7	200.8	197.9	2483	64.92
12/4/2020 16:23	203.6	201	197.9	2484	64.91
12/4/2020 16:23	203.6	201.2	197.5	2483	65.04
12/4/2020 16:23	203.6	201.3	197.3	2483	64.92
12/4/2020 16:23	203.6	201.3	197.2	2480	64.88
12/4/2020 16:23	203.6	200.9	197.2	2481	65.03
12/4/2020 16:23	203.6	201.1	197.3	2482	64.96
12/4/2020 16:23	203.5	201.3	197.5	2483	64.83
12/4/2020 16:23	203.6	201.2	197.7	2481	64.92
12/4/2020 16:24	203.6	201.3	197.6	2479	65.08
12/4/2020 16:24	203.6	200.8	197.6	2480	64.92
12/4/2020 16:24	203.6	201	197.6	2483	65.03
12/4/2020 16:24	203.6	201.3	197.5	2488	65
12/4/2020 16:24	203.6	200.8	197.5	2484	64.96
12/4/2020 16:24	203.6	200.8	197.6	2482	64.99
12/4/2020 16:24	203.6	200.7	198	2478	64.88
12/4/2020 16:24	203.6	201.2	198.4	2479	65
12/4/2020 16:24	203.6	201.1	198.3	2482	64.83
12/4/2020 16:24	203.6	200.7	197.5	2480	65.04
12/4/2020 16:24	203.6	201.2	197.3	2482	65.08
12/4/2020 16:24	203.6	200.8	197.2	2481	64.99
12/4/2020 16:24	203.6	201.1	197.2	2482	64.88
12/4/2020 16:24	203.6	201.1	197.4	2485	64.92
12/4/2020 16:24	203.7	200.6	197.7	2484	64.96
12/4/2020 16:24	203.6	201.1	197.6	2482	65
12/4/2020 16:24	203.6	200.7	197.7	2482	64.96
12/4/2020 16:24	203.7	200.9	197.6	2484	65
12/4/2020 16:24	203.7	201.2	197.5	2480	64.87

12/4/2020 16:24	203.7	201.1	197.3	2484	64.91
12/4/2020 16:24	203.7	201.4	197.4	2485	65.09
12/4/2020 16:24	203.7	201.2	197.4	2484	64.91
12/4/2020 16:24	203.7	201	197.5	2487	64.96
12/4/2020 16:24	203.7	201.3	197.9	2489	65.04
12/4/2020 16:24	203.7	201.2	198	2487	65.04
12/4/2020 16:24	203.6	201.2	198.2	2483	65.04
12/4/2020 16:24	203.7	200.8	198.4	2487	64.96
12/4/2020 16:24	203.7	201.3	198.2	2490	65.04
12/4/2020 16:24	203.6	201.3	198.3	2491	65
12/4/2020 16:24	203.7	200.8	198.5	2490	64.96
12/4/2020 16:24	203.7	201.2	198.3	2489	65.08
12/4/2020 16:24	203.7	200.9	198	2487	64.83
12/4/2020 16:24	203.7	201	198	2486	64.88
12/4/2020 16:24	203.7	201.5	197.8	2487	65.01
12/4/2020 16:24	203.7	201.1	197.4	2482	64.88
12/4/2020 16:24	203.7	201.2	197.6	2486	64.84
12/4/2020 16:24	203.7	200.9	197.8	2487	65.04
12/4/2020 16:24	203.7	201.3	197.8	2486	64.99
12/4/2020 16:24	203.7	200.9	197.8	2484	64.88
12/4/2020 16:24	203.7	200.9	197.5	2481	64.99
12/4/2020 16:24	203.7	201.2	197.3	2482	64.92
12/4/2020 16:24	203.6	201.1	198	2485	64.99
12/4/2020 16:24	203.7	200.8	198.5	2491	65.08
12/4/2020 16:24	203.7	201.3	197.8	2486	65.12
12/4/2020 16:24	203.6	200.8	197.6	2483	64.88
12/4/2020 16:24	203.7	200.9	197.9	2481	65.03
12/4/2020 16:24	203.7	200.8	197.7	2488	65.08
12/4/2020 16:24	203.6	200.8	197.5	2491	64.88
12/4/2020 16:24	203.7	201.3	197.4	2485	64.88
12/4/2020 16:24	203.7	201.1	198	2484	64.92
12/4/2020 16:24	203.7	201.2	198.3	2486	64.83
12/4/2020 16:24	203.7	201.2	198	2485	65.04
12/4/2020 16:24	203.6	200.8	197.6	2488	65.04
12/4/2020 16:24	203.7	201.3	197.7	2486	65.09
12/4/2020 16:24	203.7	200.8	197.8	2488	64.91
12/4/2020 16:24	203.7	201.3	197.6	2488	65
12/4/2020 16:24	203.7	200.9	197.8	2489	65.04
12/4/2020 16:24	203.7	200.8	197.5	2489	64.92
12/4/2020 16:24	203.7	200.9	197.3	2491	64.96
12/4/2020 16:25	203.6	200.9	197.6	2496	65
12/4/2020 16:25	203.7	201.1	198.2	2490	64.92
12/4/2020 16:25	203.7	200.7	198.4	2486	64.92
12/4/2020 16:25	203.7	200.9	198.5	2492	65
12/4/2020 16:25	203.7	201.3	198	2487	64.92
12/4/2020 16:25	203.7	200.8	197.6	2487	64.91
12/4/2020 16:25	203.7	201.2	197.5	2487	65.05

12/4/2020 16:25	203.7	201.3	197.6	2484	64.99
12/4/2020 16:25	203.7	201.3	197.8	2485	64.96
12/4/2020 16:25	203.7	200.9	197.8	2480	65.04
12/4/2020 16:25	203.7	200.9	197.8	2483	65
12/4/2020 16:25	203.7	201.3	197.8	2486	64.99
12/4/2020 16:25	203.7	201.2	197.8	2486	65
12/4/2020 16:25	203.7	200.7	197.7	2485	65.04
12/4/2020 16:25	203.7	201.3	197.6	2482	64.99
12/4/2020 16:25	203.7	200.9	197.5	2485	65.04
12/4/2020 16:25	203.7	200.9	197.3	2492	65.04
12/4/2020 16:25	203.7	201.2	197.5	2492	64.99
12/4/2020 16:25	203.7	200.8	197.3	2496	65
12/4/2020 16:25	203.7	200.7	197.4	2497	65.01
12/4/2020 16:25	203.7	200.8	197.5	2493	64.91
12/4/2020 16:25	203.7	201.1	197.6	2493	64.92
12/4/2020 16:25	203.6	201.3	197.6	2495	65.08
12/4/2020 16:25	203.7	200.8	197.7	2496	65.04
12/4/2020 16:25	203.7	201.5	197.9	2493	64.96
12/4/2020 16:25	203.7	201	197.6	2494	64.96
12/4/2020 16:25	203.7	200.8	197.8	2492	64.96
12/4/2020 16:25	203.7	200.8	197.8	2488	65
12/4/2020 16:25	203.7	200.7	197.6	2493	64.96
12/4/2020 16:25	203.7	201.4	197.4	2496	65
12/4/2020 16:25	203.7	201	197.5	2492	65
12/4/2020 16:25	203.7	201.2	197.5	2487	64.96
12/4/2020 16:25	203.7	200.8	197.4	2487	65.04
12/4/2020 16:25	203.7	200.8	197.4	2492	65
12/4/2020 16:25	203.7	201	197.3	2492	65
12/4/2020 16:25	203.7	200.8	197.2	2496	65.01
12/4/2020 16:25	203.7	201.4	197.5	2497	64.99
12/4/2020 16:25	203.7	201.3	197.5	2496	64.88
12/4/2020 16:25	203.6	200.7	197.5	2493	65.01
12/4/2020 16:25	203.7	200.9	197.5	2496	65.12
12/4/2020 16:25	203.7	200.8	197.1	2496	64.96
12/4/2020 16:25	203.7	201.1	197.3	2499	65.08
12/4/2020 16:25	203.6	201.4	197.3	2497	64.88
12/4/2020 16:25	203.7	200.8	197.5	2493	64.92
12/4/2020 16:25	203.7	201.2	197.5	2493	65.12
12/4/2020 16:25	203.7	200.9	197.3	2492	65
12/4/2020 16:25	203.7	200.7	197.5	2492	65.03
12/4/2020 16:25	203.7	200.8	197.6	2493	64.88
12/4/2020 16:25	203.7	200.8	197.8	2492	64.96
12/4/2020 16:25	203.6	201.2	197.9	2489	65
12/4/2020 16:25	203.7	200.9	197.8	2492	64.88
12/4/2020 16:25	203.7	201.2	197.8	2495	65.04
12/4/2020 16:25	203.7	200.7	197.4	2491	65
12/4/2020 16:25	203.7	200.8	197.6	2494	65

12/4/2020 16:25	203.7	201.2	198.4	2496	65.04
12/4/2020 16:25	203.7	201	198.3	2492	64.96
12/4/2020 16:25	203.7	201.2	197.4	2492	64.96
12/4/2020 16:25	203.7	200.9	197.4	2494	65
12/4/2020 16:25	203.7	200.8	197.7	2498	64.96
12/4/2020 16:25	203.7	201.3	197.9	2494	64.96
12/4/2020 16:26	203.7	201	197.6	2491	65
12/4/2020 16:26	203.7	200.8	198.3	2495	65.04
12/4/2020 16:26	203.7	201.3	198.6	2490	65.04
12/4/2020 16:26	203.7	200.8	197.4	2495	65.04
12/4/2020 16:26	203.7	201.2	197.6	2500	65.04
12/4/2020 16:26	203.7	200.8	197.8	2496	64.99
12/4/2020 16:26	203.7	200.9	197.8	2493	64.99
12/4/2020 16:26	203.7	201.3	197.6	2496	64.97
12/4/2020 16:26	203.6	200.8	198.7	2494	65.08
12/4/2020 16:26	203.7	201	198.5	2495	64.92
12/4/2020 16:26	203.7	201.2	197.7	2496	65.01
12/4/2020 16:26	203.7	200.9	197.6	2499	64.92
12/4/2020 16:26	203.7	201.3	198	2500	64.92
12/4/2020 16:26	203.7	200.9	198	2498	64.8
12/4/2020 16:26	203.7	201.1	197.7	2500	65.04
12/4/2020 16:26	203.7	201.1	197.8	2496	64.88
12/4/2020 16:26	203.7	200.9	198.1	2491	64.99
12/4/2020 16:26	203.7	200.8	198.7	2493	64.96
12/4/2020 16:26	203.7	201.1	198.1	2492	64.88
12/4/2020 16:26	203.7	201.3	197.5	2495	64.96
12/4/2020 16:26	203.7	201.1	197.5	2497	65.05
12/4/2020 16:26	203.8	200.9	198	2496	64.91
12/4/2020 16:26	203.7	201.3	198	2496	64.92
12/4/2020 16:26	203.7	200.8	198	2494	65
12/4/2020 16:26	203.7	201.2	197.5	2494	65.04
12/4/2020 16:26	203.7	201.2	198.2	2495	65.04
12/4/2020 16:26	203.7	201.3	198.7	2498	64.96
12/4/2020 16:26	203.7	201.3	198	2500	65
12/4/2020 16:26	203.7	200.9	197.6	2500	64.91
12/4/2020 16:26	203.7	201.1	197.7	2500	64.96
12/4/2020 16:26	203.7	201.1	198	2502	64.96
12/4/2020 16:26	203.7	201.4	198	2496	64.92
12/4/2020 16:26	203.8	200.9	197.7	2493	64.96
12/4/2020 16:26	203.8	200.8	198.2	2494	65.12
12/4/2020 16:26	203.7	201.1	198.7	2493	64.92
12/4/2020 16:26	203.8	200.7	198	2491	64.99
12/4/2020 16:26	203.7	201.2	197.6	2493	65.09
12/4/2020 16:26	203.7	201.3	197.8	2492	64.92
12/4/2020 16:26	203.7	200.8	198	2491	64.92
12/4/2020 16:26	203.8	201.2	197.8	2495	64.92
12/4/2020 16:26	203.8	200.9	197.7	2496	65.08

12/4/2020 16:26	203.8	200.8	198.8	2493	65.04
12/4/2020 16:26	203.8	201.4	198	2488	64.88
12/4/2020 16:26	203.8	200.7	197.6	2488	65.05
12/4/2020 16:26	203.8	201.3	197.9	2494	65.03
12/4/2020 16:26	203.7	200.8	198	2495	64.88
12/4/2020 16:26	203.9	201.2	197.8	2494	64.96
12/4/2020 16:26	203.8	201.3	198.3	2494	65
12/4/2020 16:26	203.8	200.9	198.5	2493	65.04
12/4/2020 16:26	203.8	201.1	197.7	2490	64.99
12/4/2020 16:26	203.7	200.8	197.5	2490	64.88
12/4/2020 16:26	203.8	201.2	198	2492	64.96
12/4/2020 16:26	203.7	201.3	198	2492	64.92
12/4/2020 16:26	203.8	200.7	197.6	2492	65
12/4/2020 16:26	203.8	201.1	198.4	2495	64.96
12/4/2020 16:26	203.8	200.7	198.3	2495	64.96
12/4/2020 16:26	203.8	200.8	197.5	2496	65.04
12/4/2020 16:26	203.8	201.2	197.6	2493	64.92
12/4/2020 16:26	203.8	201.2	198	2496	64.96
12/4/2020 16:27	203.8	201.2	198	2496	65
12/4/2020 16:27	203.8	201.2	197.8	2496	64.92
12/4/2020 16:27	203.8	201.3	198.8	2499	65.04
12/4/2020 16:27	203.8	201.1	197.6	2503	64.92
12/4/2020 16:27	203.9	200.8	197.6	2502	65
12/4/2020 16:27	203.8	200.9	197.9	2498	64.99
12/4/2020 16:27	203.8	201.1	197.9	2499	64.92
12/4/2020 16:27	203.9	201.2	197.9	2496	65.08
12/4/2020 16:27	203.8	201.3	197.8	2492	65.03
12/4/2020 16:27	203.8	200.9	197.9	2490	65.04
12/4/2020 16:27	203.9	201.4	198.5	2490	64.97
12/4/2020 16:27	203.9	201.3	198.6	2495	64.84
12/4/2020 16:27	203.9	201.1	198	2492	64.99
12/4/2020 16:27	203.9	201.2	197.5	2492	65.08
12/4/2020 16:27	203.9	201.1	197.5	2495	64.88
12/4/2020 16:27	203.9	200.9	197.6	2493	64.92
12/4/2020 16:27	203.8	201.3	198	2499	64.96
12/4/2020 16:27	203.9	201.1	198	2497	64.96
12/4/2020 16:27	203.9	201.2	198	2497	64.92
12/4/2020 16:27	203.9	200.8	198	2496	65
12/4/2020 16:27	203.9	200.8	197.8	2497	64.88
12/4/2020 16:27	203.9	201.2	197.8	2494	65
12/4/2020 16:27	203.9	201.1	197.9	2496	65
12/4/2020 16:27	203.9	201.5	198.3	2494	64.96
12/4/2020 16:27	203.9	200.9	198.8	2495	64.92
12/4/2020 16:27	203.9	201	198.7	2489	64.87
12/4/2020 16:27	203.9	201.1	198.4	2491	65
12/4/2020 16:27	203.9	201.1	197.7	2487	64.96
12/4/2020 16:27	203.9	201.3	197.6	2487	64.92

12/4/2020 16:27	203.9	200.9	197.6	2490	65.12
12/4/2020 16:27	203.9	201.2	197.5	2488	64.96
12/4/2020 16:27	203.9	200.8	197.6	2485	65
12/4/2020 16:27	203.9	200.7	197.6	2488	65.12
12/4/2020 16:27	203.9	201.2	197.9	2488	64.99
12/4/2020 16:27	203.9	200.8	198	2488	65.04
12/4/2020 16:27	203.9	201.4	198.2	2487	65.04
12/4/2020 16:27	203.9	200.8	198	2495	64.88
12/4/2020 16:27	203.9	200.9	197.8	2499	64.84
12/4/2020 16:27	203.9	201.1	197.9	2503	65.03
12/4/2020 16:27	203.9	200.9	198	2505	64.88
12/4/2020 16:27	203.9	201.3	198	2499	64.88
12/4/2020 16:27	203.9	201.2	198.3	2496	65.08
12/4/2020 16:27	204	200.8	198.8	2496	65.12
12/4/2020 16:27	203.9	201	198.7	2496	64.99
12/4/2020 16:27	204	200.8	198.2	2490	64.88
12/4/2020 16:27	203.9	201	197.7	2490	65.08
12/4/2020 16:27	204	200.8	197.7	2491	64.99
12/4/2020 16:27	203.9	200.8	197.8	2495	64.96
12/4/2020 16:27	204	201.3	198	2495	65.04
12/4/2020 16:27	204.1	200.8	198	2496	64.96
12/4/2020 16:27	203.9	201.3	198	2496	64.92
12/4/2020 16:27	204	200.8	198	2498	64.96
12/4/2020 16:27	204	200.8	198	2501	65.09
12/4/2020 16:27	203.9	201.3	197.9	2496	64.96
12/4/2020 16:27	203.9	200.9	198.1	2494	65
12/4/2020 16:27	204	201.5	198.6	2500	64.96
12/4/2020 16:27	204	201.3	198.9	2499	65
12/4/2020 16:27	204	200.9	198.7	2498	64.96
12/4/2020 16:27	204	201.2	198.6	2493	65
12/4/2020 16:28	204.1	200.8	198.3	2496	64.96
12/4/2020 16:28	204	201.3	198.3	2499	64.91
12/4/2020 16:28	204	201.3	198	2496	65
12/4/2020 16:28	204	200.8	198	2496	65
12/4/2020 16:28	204	201.2	198	2496	64.91
12/4/2020 16:28	204.1	200.9	198.1	2495	65.39
12/4/2020 16:28	204	201.3	198.4	2495	66.59
12/4/2020 16:28	204	201.3	198.5	2490	66.83
12/4/2020 16:28	204	200.8	198.7	2480	65.62
12/4/2020 16:28	204	201.2	198.9	2482	64.34
12/4/2020 16:28	204.1	201	198.8	2478	64.73
12/4/2020 16:28	204	201.3	198.5	2475	64.84
12/4/2020 16:28	203.9	201.4	198.5	2476	65.2
12/4/2020 16:28	204.1	200.8	198.1	2480	65
12/4/2020 16:28	204	201.2	197.8	2476	64.96
12/4/2020 16:28	204	200.9	197.8	2478	65.44
12/4/2020 16:28	204.1	201.3	197.9	2473	65.82

12/4/2020 16:28	204	201.3	197.9	2469	66.05
12/4/2020 16:28	204	200.8	198	2464	66.02
12/4/2020 16:28	203.9	201.3	198	2462	65.6
12/4/2020 16:28	203.9	200.8	198.1	2458	65.82
12/4/2020 16:28	204	201.1	198	2449	65.83
12/4/2020 16:28	204	201.3	198.1	2444	65.56
12/4/2020 16:28	204.1	200.8	198.1	2434	65.56
12/4/2020 16:28	204	201.3	198.1	2433	65.33
12/4/2020 16:28	203.9	200.9	198	2433	63.58
12/4/2020 16:28	204	200.9	197.8	2438	63.1
12/4/2020 16:28	204	200.6	197.8	2441	63.53
12/4/2020 16:28	204	200.8	197.8	2451	63.8
12/4/2020 16:28	203.9	201.3	197.6	2465	64.11
12/4/2020 16:28	204	200.9	197.8	2474	64.46
12/4/2020 16:28	203.9	200.9	197.8	2480	64.49
12/4/2020 16:28	203.9	200.8	198.2	2486	64.77
12/4/2020 16:28	203.9	201.1	198.5	2484	64.65
12/4/2020 16:28	203.9	201.3	198.7	2487	64.84
12/4/2020 16:28	203.9	201	198.7	2493	65.09
12/4/2020 16:28	203.9	201.1	198.8	2491	64.83
12/4/2020 16:28	203.9	201.2	198.7	2493	64.65
12/4/2020 16:28	203.9	201	198.5	2496	65.12
12/4/2020 16:28	203.9	201.3	198.3	2497	65.08
12/4/2020 16:28	203.9	201.1	198.3	2500	65.31
12/4/2020 16:28	203.9	201.3	198.2	2502	65.15
12/4/2020 16:28	203.9	200.8	198.1	2496	64.96
12/4/2020 16:28	203.9	201	198	2496	64.99
12/4/2020 16:28	203.9	201.1	197.7	2492	65.12
12/4/2020 16:28	203.9	200.9	197.8	2490	65.23
12/4/2020 16:28	203.9	201.3	198	2484	65.03
12/4/2020 16:28	203.9	200.8	198	2487	65.11
12/4/2020 16:28	203.8	200.6	197.9	2487	65.36
12/4/2020 16:28	203.9	201.3	198	2485	65.15
12/4/2020 16:28	203.9	200.9	198.2	2482	65.12
12/4/2020 16:28	203.9	201.2	198.2	2486	65.17
12/4/2020 16:28	203.9	201.3	198	2487	65.2
12/4/2020 16:28	203.9	200.7	198	2482	65.2
12/4/2020 16:28	203.9	201.2	198	2483	64.96
12/4/2020 16:28	203.9	200.8	198.1	2482	64.92
12/4/2020 16:28	203.9	201.3	198	2479	65.43
12/4/2020 16:28	203.9	201.3	198.1	2482	65.97
12/4/2020 16:28	203.8	201.3	198	2474	66.83
12/4/2020 16:28	203.9	201.2	198	2467	64.41
12/4/2020 16:29	203.9	201.1	198.1	2469	64.81
12/4/2020 16:29	203.9	201	198.1	2471	65.4
12/4/2020 16:29	203.9	200.9	198.1	2467	64.96
12/4/2020 16:29	203.9	200.8	198	2465	65.67

12/4/2020 16:29	203.9	201.2	198.1	2463	65.32
12/4/2020 16:29	203.9	201	198	2460	65.2
12/4/2020 16:29	203.9	201.3	198	2459	64.69
12/4/2020 16:29	203.9	200.7	198	2461	63.77
12/4/2020 16:29	203.9	201	198.1	2463	63.99
12/4/2020 16:29	203.8	201.2	198.2	2465	64.25
12/4/2020 16:29	203.9	200.9	198.2	2471	64.38
12/4/2020 16:29	203.9	200.8	198	2475	64.61
12/4/2020 16:29	203.9	201.2	198.2	2481	64.96
12/4/2020 16:29	203.9	200.8	198.1	2486	64.96
12/4/2020 16:29	203.9	201.1	198.1	2487	65.08
12/4/2020 16:29	203.9	200.8	198	2487	65.03
12/4/2020 16:29	203.8	201.3	198	2487	64.96
12/4/2020 16:29	203.9	200.8	197.9	2486	65.04
12/4/2020 16:29	203.8	200.8	197.8	2482	65.18
12/4/2020 16:29	203.9	201.3	198.1	2483	64.96
12/4/2020 16:29	203.9	200.8	198.5	2480	64.84
12/4/2020 16:29	203.9	200.9	198.8	2478	64.96
12/4/2020 16:29	203.8	201.3	198.5	2482	64.65
12/4/2020 16:29	203.9	201	198	2479	64.26
12/4/2020 16:29	203.9	201.1	197.6	2481	64.84
12/4/2020 16:29	203.8	200.8	197.8	2484	64.96
12/4/2020 16:29	203.8	200.9	198.1	2488	64.93
12/4/2020 16:29	203.9	201.3	198	2488	65.07
12/4/2020 16:29	203.9	201.1	198	2490	65.38
12/4/2020 16:29	203.9	201.3	197.7	2491	66.24
12/4/2020 16:29	203.9	201	198.4	2486	65.46
12/4/2020 16:29	203.9	201	198.8	2478	64.77
12/4/2020 16:29	203.9	201.2	197.9	2481	64.74
12/4/2020 16:29	203.9	201.2	197.8	2480	64.61
12/4/2020 16:29	203.9	201.2	198.3	2480	64.76
12/4/2020 16:29	203.9	201	198.2	2482	64.89
12/4/2020 16:29	203.9	200.7	198	2480	64.93
12/4/2020 16:29	203.9	200.7	198.2	2481	64.88
12/4/2020 16:29	203.9	200.7	198.9	2481	64.96
12/4/2020 16:29	203.9	201.3	198	2482	65.04
12/4/2020 16:29	203.9	201.2	197.6	2480	64.83
12/4/2020 16:29	203.9	200.7	198.2	2478	64.95
12/4/2020 16:29	203.9	200.8	198.2	2484	65
12/4/2020 16:29	203.9	201.1	198	2480	64.88
12/4/2020 16:29	203.9	201.2	198	2480	64.96
12/4/2020 16:29	203.9	201.2	198.8	2482	65.09
12/4/2020 16:29	203.9	200.7	197.8	2476	64.96
12/4/2020 16:29	203.9	201.3	197.6	2482	64.96
12/4/2020 16:29	203.9	200.8	198.2	2487	65.15
12/4/2020 16:29	203.9	201.1	198.2	2483	65.31
12/4/2020 16:29	203.9	201.3	198	2479	65.23

12/4/2020 16:29	203.9	200.8	198.3	2477	64.81
12/4/2020 16:29	203.9	201.3	199	2479	64.96
12/4/2020 16:29	203.9	200.7	198.2	2476	64.77
12/4/2020 16:29	203.9	201.2	197.8	2476	65.12
12/4/2020 16:29	203.9	201.2	198.1	2478	64.89
12/4/2020 16:29	203.9	201.4	198.2	2476	64.84
12/4/2020 16:29	203.9	201.5	198.2	2477	65.08
12/4/2020 16:29	204	200.8	198	2481	64.89
12/4/2020 16:30	203.9	202	198.6	2479	64.72
12/4/2020 16:30	203.9	200.7	198.8	2479	64.77
12/4/2020 16:30	203.9	200.8	198	2484	65.05
12/4/2020 16:30	204	202	197.9	2488	65.08
12/4/2020 16:30	203.9	200.8	198.2	2485	64.96
12/4/2020 16:30	204	201.1	198.3	2482	65
12/4/2020 16:30	204	201	198	2486	65
12/4/2020 16:30	204	201.3	198.5	2481	65.04
12/4/2020 16:30	203.9	202.1	199	2484	64.81
12/4/2020 16:30	203.9	202.2	198	2485	65.16
12/4/2020 16:30	204	202	198	2487	65.07
12/4/2020 16:30	204	202	198.3	2483	64.88
12/4/2020 16:30	203.9	202.2	198.2	2485	64.81
12/4/2020 16:30	204	202.2	198.1	2487	65.03
12/4/2020 16:30	204	201.9	198.3	2487	64.99
12/4/2020 16:30	204	202.1	199	2491	65.08
12/4/2020 16:30	204	202	198.5	2492	64.99
12/4/2020 16:30	204	201.9	197.9	2490	64.99
12/4/2020 16:30	204	202.2	198	2492	65.12
12/4/2020 16:30	204	201.9	198.3	2488	65.12
12/4/2020 16:30	204	202.2	198.1	2486	65.08
12/4/2020 16:30	204	202.2	198.3	2482	65
12/4/2020 16:30	204.1	202	199	2483	64.99
12/4/2020 16:30	204	202	198	2479	65.15
12/4/2020 16:30	204.1	202	198	2477	65.15
12/4/2020 16:30	204.1	202	198.1	2476	64.99
12/4/2020 16:30	203.9	202	198.3	2478	65.3
12/4/2020 16:30	204	201.9	198	2480	65.12
12/4/2020 16:30	204	201.9	198.5	2480	65.2
12/4/2020 16:30	203.9	202.1	199	2478	65.15
12/4/2020 16:30	204	202	198.1	2476	65.23
12/4/2020 16:30	204	202.1	198	2474	65.08
12/4/2020 16:30	204	202	198.2	2470	65.15
12/4/2020 16:30	204	202	198.2	2474	64.96
12/4/2020 16:30	204.1	202	198	2473	65
12/4/2020 16:30	204	202	198.6	2467	65.3
12/4/2020 16:30	204	202.2	198.8	2470	65.89
12/4/2020 16:30	204.1	202	197.8	2467	66.68
12/4/2020 16:30	204	202.1	198	2457	66.46

12/4/2020 16:30	204	201.9	198.3	2452	66.28
12/4/2020 16:30	204	202	198.2	2442	63.18
12/4/2020 16:30	204	202.1	198	2442	63.84
12/4/2020 16:30	203.9	202	199	2442	63.84
12/4/2020 16:30	204	202.2	198.3	2446	64.18
12/4/2020 16:30	204.1	202	197.8	2458	64.27
12/4/2020 16:30	204	202	198.2	2462	64.33
12/4/2020 16:30	203.9	202.1	198.3	2465	64.53
12/4/2020 16:30	204	202	198.2	2473	64.7
12/4/2020 16:30	204.1	202	198.2	2476	64.72
12/4/2020 16:30	204	202	199.2	2475	64.93
12/4/2020 16:30	204	202.2	198.5	2475	64.88
12/4/2020 16:30	204	202.2	197.8	2477	64.76
12/4/2020 16:30	204.1	201.9	198	2480	64.92
12/4/2020 16:30	204	202.1	198.3	2479	64.84
12/4/2020 16:30	204	202.2	198.2	2484	64.96
12/4/2020 16:30	204	202.1	198	2482	64.87
12/4/2020 16:30	204	202	198.5	2482	64.92
12/4/2020 16:30	204	202	198.8	2484	65.08
12/4/2020 16:30	204	202.2	198	2484	64.8
12/4/2020 16:30	204.1	201.9	198	2482	64.92
12/4/2020 16:31	204.1	202	198.1	2487	65.03
12/4/2020 16:31	204.1	202.1	198.2	2485	64.92
12/4/2020 16:31	204.1	201.9	198.3	2489	64.99
12/4/2020 16:31	204	202.1	198	2486	64.92
12/4/2020 16:31	204	202.1	198.6	2484	65.03
12/4/2020 16:31	204.1	202	198.8	2485	64.99
12/4/2020 16:31	204	202.1	197.8	2488	64.99
12/4/2020 16:31	204	202	197.8	2489	64.99
12/4/2020 16:31	204	202.2	198.2	2487	64.88
12/4/2020 16:31	204.1	202.2	198.2	2487	64.88
12/4/2020 16:31	204	202.2	198.2	2491	65.04
12/4/2020 16:31	204.1	202.1	198.1	2484	65.03
12/4/2020 16:31	204.1	201.9	198.6	2486	64.92
12/4/2020 16:31	204.1	202	198.8	2484	64.88
12/4/2020 16:31	204	202	197.9	2483	64.92
12/4/2020 16:31	204	201.9	197.8	2484	64.88
12/4/2020 16:31	204.1	202.2	198	2488	65.03
12/4/2020 16:31	204.1	202	198.2	2487	65.04
12/4/2020 16:31	204	202	198.2	2485	64.83
12/4/2020 16:31	204.1	202	198.2	2482	64.92
12/4/2020 16:31	204.1	202	198	2478	65.08
12/4/2020 16:31	204.1	201.9	198.2	2479	64.95
12/4/2020 16:31	204.1	202	198.8	2479	64.88
12/4/2020 16:31	204.1	202.2	199	2485	65
12/4/2020 16:31	204.1	202.1	198.6	2484	64.88
12/4/2020 16:31	204.1	202.1	198.3	2480	64.92

12/4/2020 16:31	204.1	202.2	197.8	2488	64.92
12/4/2020 16:31	204.1	202	197.9	2484	64.92
12/4/2020 16:31	204.1	202	197.8	2484	64.96
12/4/2020 16:31	204.1	202.2	197.8	2486	65
12/4/2020 16:31	204.1	202	197.9	2485	64.91
12/4/2020 16:31	204.1	201.9	198	2478	64.96
12/4/2020 16:31	204.1	202	198	2478	64.96
12/4/2020 16:31	204.1	202.1	198.1	2478	64.87
12/4/2020 16:31	204.1	202.2	198.2	2478	64.88
12/4/2020 16:31	204.2	202	198.2	2478	64.88
12/4/2020 16:31	204.1	202.1	198.2	2481	64.92
12/4/2020 16:31	204.1	202	198	2478	64.83
12/4/2020 16:31	204.1	202	198.5	2477	64.88
12/4/2020 16:31	204.1	202	198.4	2480	65
12/4/2020 16:31	204.1	202	198.3	2479	64.92
12/4/2020 16:31	204.1	202	198.3	2481	64.96
12/4/2020 16:31	204.2	202.1	198.3	2484	64.92
12/4/2020 16:31	204.2	202.1	198.3	2480	64.92
12/4/2020 16:31	204.2	202	198.2	2479	64.96
12/4/2020 16:31	204.2	202.2	198.1	2480	64.96
12/4/2020 16:31	204.2	202	198.2	2478	64.87
12/4/2020 16:31	204.1	202	198.1	2480	64.96
12/4/2020 16:31	204.2	202	198	2476	64.96
12/4/2020 16:31	204.1	202	198	2478	64.96
12/4/2020 16:31	204.2	202.1	198	2481	64.96
12/4/2020 16:31	204.3	202.2	198.1	2482	64.96
12/4/2020 16:31	204.2	202.2	198.3	2478	64.96
12/4/2020 16:31	204.2	202	198.9	2479	64.92
12/4/2020 16:31	204.2	202	199.1	2479	64.92
12/4/2020 16:31	204.2	202	198.9	2483	64.96
12/4/2020 16:31	204.2	202	198.7	2480	64.92
12/4/2020 16:31	204.3	202	198.2	2477	64.96
12/4/2020 16:31	204.3	202.1	198	2478	64.92
12/4/2020 16:32	204.2	202.1	198.2	2480	64.92
12/4/2020 16:32	204.3	202	198.4	2474	65
12/4/2020 16:32	204.3	202.2	198.4	2467	64.92
12/4/2020 16:32	204.3	202	198.3	2465	64.96
12/4/2020 16:32	204.3	202.2	198.3	2461	64.91
12/4/2020 16:32	204.3	202	198.3	2458	64.92
12/4/2020 16:32	204.3	201.9	198.4	2462	64.96
12/4/2020 16:32	204.3	202	198.3	2462	64.92
12/4/2020 16:32	204.3	202	198.5	2462	64.96
12/4/2020 16:32	204.3	202	198.3	2461	64.92
12/4/2020 16:32	204.3	202	198.2	2463	64.96
12/4/2020 16:32	204.4	202	198.5	2462	64.96
12/4/2020 16:32	204.3	202.1	198.3	2460	65
12/4/2020 16:32	204.3	202	198.3	2464	65

12/4/2020 16:32	204.4	202	198.3	2467	64.96
12/4/2020 16:32	204.3	201.9	198.5	2470	64.91
12/4/2020 16:32	204.3	202.1	198.2	2476	65
12/4/2020 16:32	204.4	202	198.5	2477	64.91
12/4/2020 16:32	204.3	202.2	198.3	2478	64.91
12/4/2020 16:32	204.4	202	198.2	2475	64.96
12/4/2020 16:32	204.3	202	198.1	2474	64.88
12/4/2020 16:32	204.4	202	198.5	2474	64.92
12/4/2020 16:32	204.4	202	199	2470	65.04
12/4/2020 16:32	204.4	202	198.8	2471	64.96
12/4/2020 16:32	204.3	202.2	198.2	2467	64.92
12/4/2020 16:32	204.3	202.1	198.1	2465	64.91
12/4/2020 16:32	204.3	202	198.5	2465	64.96
12/4/2020 16:32	204.3	201.9	198.5	2466	64.87
12/4/2020 16:32	204.3	202	198.3	2462	64.92
12/4/2020 16:32	204.3	202	198.1	2466	64.96
12/4/2020 16:32	204.4	202.1	199.2	2464	64.99
12/4/2020 16:32	204.4	202.2	198.3	2465	65.04
12/4/2020 16:32	204.4	202	198	2467	65.08
12/4/2020 16:32	204.4	202	198.3	2463	64.88
12/4/2020 16:32	204.4	202	198.3	2466	65
12/4/2020 16:32	204.4	202	198.2	2457	64.88
12/4/2020 16:32	204.4	202.1	198.7	2460	64.96
12/4/2020 16:32	204.5	202	199.1	2453	64.88
12/4/2020 16:32	204.4	202.1	198.2	2453	64.92
12/4/2020 16:32	204.4	202	198.2	2455	64.96
12/4/2020 16:32	204.4	202	198.3	2452	64.92
12/4/2020 16:32	204.4	202	198.4	2451	65.04
12/4/2020 16:32	204.4	202.1	198.4	2452	65.12
12/4/2020 16:32	204.4	202	198.2	2451	64.84
12/4/2020 16:32	204.4	202	198.5	2453	65.04
12/4/2020 16:32	204.4	202	199.2	2454	64.92
12/4/2020 16:32	204.5	202	199	2455	65
12/4/2020 16:32	204.4	202.1	198.2	2452	64.99
12/4/2020 16:32	204.4	202.1	198.1	2460	64.88
12/4/2020 16:32	204.4	202	198.3	2468	65.08
12/4/2020 16:32	204.5	201.9	198.5	2469	64.99
12/4/2020 16:32	204.4	202.1	198.6	2472	64.96
12/4/2020 16:32	204.5	202.1	198.6	2478	64.99
12/4/2020 16:32	204.5	202	198.5	2476	64.88
12/4/2020 16:32	204.5	202.2	198.2	2466	65.04
12/4/2020 16:32	204.5	202	198.3	2458	65
12/4/2020 16:32	204.5	201.9	198.7	2453	65
12/4/2020 16:32	204.5	201.9	199	2449	64.96
12/4/2020 16:32	204.5	201.9	199.2	2445	64.92
12/4/2020 16:33	204.5	202.1	199.2	2449	65.04
12/4/2020 16:33	204.5	202	198.8	2450	64.92

12/4/2020 16:33	204.5	202.1	198.6	2452	64.96
12/4/2020 16:33	204.5	202.2	198.1	2456	65.04
12/4/2020 16:33	204.6	202	198.1	2460	64.96
12/4/2020 16:33	204.6	202.1	198.2	2459	65
12/4/2020 16:33	204.5	202	198.2	2458	65
12/4/2020 16:33	204.6	202	198.5	2456	65
12/4/2020 16:33	204.6	201.9	198.6	2457	64.92
12/4/2020 16:33	204.6	202	198.6	2458	64.96
12/4/2020 16:33	204.6	202	198.6	2460	65.04
12/4/2020 16:33	204.6	202.1	198.5	2457	64.92
12/4/2020 16:33	204.6	202.1	198.1	2451	64.96
12/4/2020 16:33	204.6	202.1	198.4	2455	65
12/4/2020 16:33	204.6	202	199	2454	64.92
12/4/2020 16:33	204.6	202.1	199.2	2453	65.04
12/4/2020 16:33	204.5	202.1	198.9	2459	64.88
12/4/2020 16:33	204.6	202	198.2	2458	64.88
12/4/2020 16:33	204.6	202.1	198	2461	65.04
12/4/2020 16:33	204.6	202	198.1	2463	64.99
12/4/2020 16:33	204.6	202	198.5	2462	65.08
12/4/2020 16:33	204.6	202	198.7	2460	65.03
12/4/2020 16:33	204.5	202	198.7	2459	65.08
12/4/2020 16:33	204.6	202	198.3	2464	64.96
12/4/2020 16:33	204.6	202.1	198.3	2466	65
12/4/2020 16:33	204.6	202.1	199	2469	64.96
12/4/2020 16:33	204.6	202	199.2	2473	65
12/4/2020 16:33	204.6	202.1	198.3	2478	65.08
12/4/2020 16:33	204.6	202.1	198.2	2478	64.96
12/4/2020 16:33	204.6	202	198.5	2482	65
12/4/2020 16:33	204.6	201.9	198.5	2482	65
12/4/2020 16:33	204.6	202	198.4	2482	64.91
12/4/2020 16:33	204.6	202	198.2	2484	64.92
12/4/2020 16:33	204.6	202.2	199.3	2484	65.04
12/4/2020 16:33	204.6	202.2	198.8	2485	65
12/4/2020 16:33	204.6	202.2	198.2	2485	64.96
12/4/2020 16:33	204.6	202.2	198.5	2487	65.04
12/4/2020 16:33	204.7	202	198.6	2483	65
12/4/2020 16:33	204.6	202.1	198.3	2485	65.04
12/4/2020 16:33	204.6	202.2	198.7	2490	65.08
12/4/2020 16:33	204.6	202	199.2	2490	64.96
12/4/2020 16:33	204.6	202	198.5	2489	64.96
12/4/2020 16:33	204.7	202	198.1	2488	64.96
12/4/2020 16:33	204.6	202	198.3	2489	64.99
12/4/2020 16:33	204.6	202	198.7	2486	64.99
12/4/2020 16:33	204.7	202	198.5	2484	65.12
12/4/2020 16:33	204.6	202	198.5	2487	65.08
12/4/2020 16:33	204.7	202.1	198.3	2485	64.99
12/4/2020 16:33	204.6	202	199	2482	65.04

12/4/2020 16:33	204.7	202.1	199.3	2482	65.12
12/4/2020 16:33	204.6	202.1	198.9	2481	65.04
12/4/2020 16:33	204.7	202.2	198.2	2479	64.88
12/4/2020 16:33	204.6	202	198.2	2476	65
12/4/2020 16:33	204.6	202.1	198.2	2480	64.96
12/4/2020 16:33	204.9	202.1	198.6	2486	65
12/4/2020 16:33	204.7	201.9	198.7	2489	65
12/4/2020 16:33	204.6	202	198.7	2489	65
12/4/2020 16:33	204.6	202.1	198.7	2489	64.96
12/4/2020 16:33	204.7	202.1	198.2	2488	64.92
12/4/2020 16:33	204.6	202	198.7	2492	65.04
12/4/2020 16:34	204.6	201.9	199.3	2496	65.04
12/4/2020 16:34	204.7	201.9	199.2	2497	64.96
12/4/2020 16:34	204.6	202	198.3	2494	65.09
12/4/2020 16:34	204.7	202.1	198.3	2492	65.04
12/4/2020 16:34	204.6	202.1	198.5	2491	64.92
12/4/2020 16:34	204.7	202	198.7	2489	65.12
12/4/2020 16:34	204.7	202	198.9	2484	65.04
12/4/2020 16:34	204.6	202.1	198.5	2484	64.92
12/4/2020 16:34	204.7	202.1	198.2	2482	65.04
12/4/2020 16:34	204.6	202	198.8	2485	64.92
12/4/2020 16:34	204.7	202	199.2	2485	65.04
12/4/2020 16:34	204.7	202	199.4	2488	64.92
12/4/2020 16:34	204.7	202.1	199	2488	65.05
12/4/2020 16:34	204.7	202.2	198.3	2488	64.96
12/4/2020 16:34	204.7	202.1	198.1	2488	65
12/4/2020 16:34	204.7	202.1	198.3	2490	65.09
12/4/2020 16:34	204.7	202	198.3	2489	65
12/4/2020 16:34	204.7	201.9	198.6	2485	65
12/4/2020 16:34	204.7	202	198.8	2484	65
12/4/2020 16:34	204.8	202.1	198.7	2487	65.04
12/4/2020 16:34	204.7	202.1	198.5	2493	65.04
12/4/2020 16:34	204.7	202	198.5	2491	64.92
12/4/2020 16:34	204.7	202	198.5	2492	65.17
12/4/2020 16:34	204.8	202	198.3	2488	64.88
12/4/2020 16:34	204.6	202.1	198.5	2488	65
12/4/2020 16:34	204.8	202	198.7	2479	65.12
12/4/2020 16:34	204.7	202.1	198.8	2469	64.92
12/4/2020 16:34	204.8	202	199	2467	64.96
12/4/2020 16:34	204.7	202	199.4	2462	65.12
12/4/2020 16:34	204.7	202.1	199.2	2464	64.99
12/4/2020 16:34	204.8	202	199.2	2467	65.08
12/4/2020 16:34	204.7	201.9	199.1	2470	64.96
12/4/2020 16:34	204.7	202	199.1	2466	64.96
12/4/2020 16:34	204.7	202	198.9	2460	64.92
12/4/2020 16:34	204.6	202.2	198.8	2460	65
12/4/2020 16:34	204.8	201.9	198.5	2462	65

12/4/2020 16:34	204.7	202.1	198.5	2460	64.96
12/4/2020 16:34	204.7	202.3	198.4	2459	64.96
12/4/2020 16:34	204.7	202.2	198.3	2463	65.04
12/4/2020 16:34	204.8	202.2	198.5	2464	64.96
12/4/2020 16:34	204.8	202	198.4	2463	65.04
12/4/2020 16:34	204.7	202	198.6	2464	65.04
12/4/2020 16:34	204.7	202.1	198.6	2466	65
12/4/2020 16:34	204.7	202	198.7	2464	65.04
12/4/2020 16:34	204.7	202	198.8	2464	65.04
12/4/2020 16:34	204.7	202	198.7	2459	65
12/4/2020 16:34	204.9	202.1	198.5	2460	64.92
12/4/2020 16:34	204.8	202.1	198.5	2462	65.08
12/4/2020 16:34	204.8	202.1	198.3	2465	64.93
12/4/2020 16:34	204.7	202.1	198.2	2474	65.08
12/4/2020 16:34	204.8	202	198.2	2481	65.12
12/4/2020 16:34	204.8	202.1	198.3	2483	65.08
12/4/2020 16:34	204.8	202.2	198.2	2488	64.92
12/4/2020 16:34	204.8	202	198.3	2495	64.92
12/4/2020 16:34	204.8	201.9	198.5	2488	64.92
12/4/2020 16:34	204.8	202	198.8	2488	65.17
12/4/2020 16:34	204.8	202	198.8	2488	65
12/4/2020 16:34	204.8	202	198.9	2488	65.04
12/4/2020 16:34	204.8	202	198.9	2490	65.09
12/4/2020 16:35	204.8	201.9	198.9	2488	65
12/4/2020 16:35	204.8	202.2	198.3	2489	65
12/4/2020 16:35	204.8	202.2	198.2	2494	65
12/4/2020 16:35	204.8	202.2	198.1	2497	64.92
12/4/2020 16:35	204.8	202.1	198.5	2496	64.92
12/4/2020 16:35	204.8	202	198.8	2495	65.08
12/4/2020 16:35	204.8	202	198.7	2499	65.05
12/4/2020 16:35	204.8	202.2	198.9	2501	65.08
12/4/2020 16:35	204.8	202.1	198.4	2496	65.08
12/4/2020 16:35	204.8	202	199	2490	64.96
12/4/2020 16:35	204.8	202.1	199.5	2487	65.12
12/4/2020 16:35	204.8	202.2	198.3	2490	65.04
12/4/2020 16:35	204.8	202.2	198.5	2495	64.96
12/4/2020 16:35	204.8	202	198.8	2496	65.04
12/4/2020 16:35	204.8	202.1	198.7	2492	65.04
12/4/2020 16:35	204.8	202.2	198.4	2493	64.96
12/4/2020 16:35	204.8	202.1	199.5	2496	65.04
12/4/2020 16:35	204.8	202	198.8	2496	65
12/4/2020 16:35	204.9	202.1	198.3	2496	64.96
12/4/2020 16:35	204.8	202	198.7	2497	65.05
12/4/2020 16:35	204.9	202.1	198.8	2496	64.87
12/4/2020 16:35	204.9	202.1	198.6	2494	64.92
12/4/2020 16:35	204.8	201.9	198.9	2496	65.04
12/4/2020 16:35	204.9	202	199.6	2494	64.96

12/4/2020 16:35	204.8	202	198.7	2492	65
12/4/2020 16:35	204.9	202.2	198.3	2496	65
12/4/2020 16:35	204.9	201.9	198.8	2494	65.08
12/4/2020 16:35	204.9	202.2	198.8	2494	64.99
12/4/2020 16:35	204.9	202.2	198.8	2494	64.92
12/4/2020 16:35	205	202	198.7	2492	65.01
12/4/2020 16:35	204.9	202.2	199.5	2491	64.88
12/4/2020 16:35	204.9	201.9	199.2	2487	64.92
12/4/2020 16:35	204.9	202.2	198.3	2485	65.05
12/4/2020 16:35	205	202.1	198.6	2484	65.04
12/4/2020 16:35	205	202.1	198.9	2484	65.12
12/4/2020 16:35	204.9	202	198.7	2490	64.96
12/4/2020 16:35	204.9	202	198.7	2488	64.96
12/4/2020 16:35	204.9	202	199.6	2487	64.96
12/4/2020 16:35	205	201.9	198.9	2490	64.96
12/4/2020 16:35	205	202.1	198.5	2489	65.04
12/4/2020 16:35	204.9	202.1	198.6	2493	64.92
12/4/2020 16:35	204.9	202	198.8	2493	65
12/4/2020 16:35	205	202.1	198.7	2493	65.04
12/4/2020 16:35	205	202	198.8	2491	64.96
12/4/2020 16:35	205	201.9	199.5	2496	64.92
12/4/2020 16:35	205	202.1	198.9	2499	65.01
12/4/2020 16:35	205	202.2	198.3	2495	64.95
12/4/2020 16:35	204.9	202.1	198.6	2496	64.93
12/4/2020 16:35	205	202.1	198.7	2492	65
12/4/2020 16:35	205	202	198.9	2490	64.88
12/4/2020 16:35	205	202	198.5	2488	65.04
12/4/2020 16:35	205	202.1	198.6	2493	65.08
12/4/2020 16:35	205.1	202.2	199.4	2496	64.88
12/4/2020 16:35	205	202	199.5	2494	65.12
12/4/2020 16:35	205	202.1	198.5	2479	65.16
12/4/2020 16:35	205	202.2	198.4	2474	64.96
12/4/2020 16:35	204.8	202.1	198.7	2472	64.95
12/4/2020 16:35	205	202	198.8	2469	65.04
12/4/2020 16:35	205.1	202.2	198.7	2476	65.16
12/4/2020 16:35	205.1	202	198.6	2470	65.08
12/4/2020 16:36	205.1	202.1	199.5	2471	65.12
12/4/2020 16:36	205	202	199.5	2473	65.04
12/4/2020 16:36	205	202	198.3	2470	65
12/4/2020 16:36	205.1	202.1	198.5	2469	65
12/4/2020 16:36	205	202	198.7	2470	65
12/4/2020 16:36	205.1	202	198.7	2471	65
12/4/2020 16:36	205.1	202.2	198.9	2471	64.96
12/4/2020 16:36	205.1	202	198.8	2470	65
12/4/2020 16:36	205.1	202	199.6	2470	65.09
12/4/2020 16:36	205	202	198.9	2470	64.96
12/4/2020 16:36	205.1	202.2	198.6	2470	65

12/4/2020 16:36	205	202.1	198.9	2474	65.04
12/4/2020 16:36	205	202	199	2471	64.91
12/4/2020 16:36	205	202.1	198.8	2469	65.04
12/4/2020 16:36	205.1	202.2	198.8	2466	65
12/4/2020 16:36	205.1	202	199.7	2465	64.88
12/4/2020 16:36	205.1	202	199.2	2465	64.92
12/4/2020 16:36	205.2	202.1	198.4	2462	65.04
12/4/2020 16:36	205	202.2	198.8	2467	65.04
12/4/2020 16:36	205.1	202	199	2469	64.88
12/4/2020 16:36	205.1	202.1	198.9	2463	64.92
12/4/2020 16:36	205.1	202	198.7	2463	65.12
12/4/2020 16:36	205.1	202	199.5	2466	64.84
12/4/2020 16:36	205.2	202.1	199.5	2467	65.08
12/4/2020 16:36	205.1	202	198.6	2470	65.08
12/4/2020 16:36	205.1	202.2	198.6	2476	65.04
12/4/2020 16:36	205.2	201.9	199	2472	64.92
12/4/2020 16:36	205.1	201.9	198.9	2473	64.96
12/4/2020 16:36	205.2	202.1	198.7	2470	65.08
12/4/2020 16:36	205.1	202	199.5	2472	64.96
12/4/2020 16:36	205.1	202.2	199.2	2471	64.88
12/4/2020 16:36	205.2	202.2	198.7	2461	65.12
12/4/2020 16:36	205.1	202	198.9	2450	64.88
12/4/2020 16:36	205.1	202	199	2444	64.96
12/4/2020 16:36	205.2	202	198.8	2444	64.92
12/4/2020 16:36	205.2	202.1	198.9	2448	64.92
12/4/2020 16:36	205.2	202	199.6	2448	64.92
12/4/2020 16:36	205.2	202	198.9	2449	65
12/4/2020 16:36	205.2	202	198.6	2448	64.96
12/4/2020 16:36	205.1	202	198.9	2447	65
12/4/2020 16:36	205.2	202.1	199	2447	64.96
12/4/2020 16:36	205.2	201.9	198.7	2449	65
12/4/2020 16:36	205.2	202	199.2	2451	64.96
12/4/2020 16:36	205.2	202.2	199.6	2449	65.03
12/4/2020 16:36	205.2	201.9	198.6	2447	65.16
12/4/2020 16:36	205.2	202.1	198.6	2442	64.92
12/4/2020 16:36	205.3	202	198.9	2442	64.92
12/4/2020 16:36	205.2	202	198.9	2446	64.96
12/4/2020 16:36	205.2	202.1	198.8	2445	64.87
12/4/2020 16:36	205.2	202.1	199.1	2444	64.88
12/4/2020 16:36	205.2	202	199.6	2448	65.01
12/4/2020 16:36	205.3	202.2	198.6	2450	64.92
12/4/2020 16:36	205.3	202.2	198.7	2447	64.96
12/4/2020 16:36	205.2	202	199	2441	64.88
12/4/2020 16:36	205.2	202.1	198.9	2444	64.96
12/4/2020 16:36	205.3	202	198.7	2444	64.99
12/4/2020 16:36	205.3	202	199.5	2442	64.88
12/4/2020 16:36	205.2	201.9	199.5	2461	65.04

12/4/2020 16:36	205.2	201.9	198.5	2473	64.99
12/4/2020 16:37	205.3	202.2	198.7	2482	64.88
12/4/2020 16:37	205.3	201.9	198.8	2485	64.96
12/4/2020 16:37	205.2	202	199	2486	64.99
12/4/2020 16:37	205.3	202	198.7	2491	64.99
12/4/2020 16:37	205.3	201.9	198.8	2490	64.99
12/4/2020 16:37	205.3	202	199.5	2494	64.96
12/4/2020 16:37	205.3	202	199.6	2496	64.95
12/4/2020 16:37	205.3	202	199.1	2496	64.84
12/4/2020 16:37	205.3	201.9	198.5	2496	64.96
12/4/2020 16:37	205.3	202.1	198.7	2496	64.92
12/4/2020 16:37	205.3	202.2	198.8	2494	64.96
12/4/2020 16:37	205.3	202	198.9	2493	65.04
12/4/2020 16:37	205.3	202	199	2495	64.88
12/4/2020 16:37	205.3	202	199	2496	65.08
12/4/2020 16:37	205.4	202.1	198.8	2499	64.99
12/4/2020 16:37	205.4	202	198.7	2495	65.04
12/4/2020 16:37	205.3	202	198.8	2490	64.95
12/4/2020 16:37	205.4	202.1	199.3	2492	65.04
12/4/2020 16:37	205.4	202	199.6	2492	65.08
12/4/2020 16:37	205.4	202.1	199.6	2487	64.92
12/4/2020 16:37	205.4	202	199.5	2487	64.92
12/4/2020 16:37	205.4	202.1	199	2487	65.04
12/4/2020 16:37	205.4	202	198.7	2491	64.88
12/4/2020 16:37	205.4	202	198.7	2496	65.04
12/4/2020 16:37	205.3	202.1	198.7	2493	64.91
12/4/2020 16:37	205.4	202	198.8	2496	64.99
12/4/2020 16:37	205.3	202.2	198.9	2498	64.92
12/4/2020 16:37	205.2	202	199	2496	64.84
12/4/2020 16:37	205.4	201.9	199.2	2496	64.96
12/4/2020 16:37	205.4	202.1	199.2	2501	64.83
12/4/2020 16:37	205.4	202	199	2497	64.96
12/4/2020 16:37	205.4	202	199.1	2497	64.99
12/4/2020 16:37	205.4	201.9	199.1	2498	64.96
12/4/2020 16:37	205.4	202	199.1	2498	64.96
12/4/2020 16:37	205.4	202.2	199.2	2496	64.88
12/4/2020 16:37	205.4	202	199	2495	65
12/4/2020 16:37	205.4	202	199.1	2490	64.92
12/4/2020 16:37	205.4	202	198.9	2488	65
12/4/2020 16:37	205.4	202	198.9	2490	65.09
12/4/2020 16:37	205.4	202.2	198.8	2492	64.83
12/4/2020 16:37	205.4	202	198.8	2491	64.96
12/4/2020 16:37	205.4	202.2	198.9	2488	65
12/4/2020 16:37	205.4	202.1	198.8	2489	64.96
12/4/2020 16:37	205.4	202	198.8	2494	65
12/4/2020 16:37	205.4	201.9	199.1	2496	64.96
12/4/2020 16:37	205.4	202.1	199.3	2499	65

12/4/2020 16:37	205.4	202	199.5	2500	64.92
12/4/2020 16:37	205.4	202.2	199.7	2496	65
12/4/2020 16:37	205.4	202	199.9	2496	65.04
12/4/2020 16:37	205.4	202	199.7	2496	64.91
12/4/2020 16:37	205.4	202	199.5	2497	65
12/4/2020 16:37	205.4	202.2	198.9	2496	65
12/4/2020 16:37	205.4	202	198.6	2499	64.87
12/4/2020 16:37	205.4	202	198.7	2500	64.99
12/4/2020 16:37	205.4	202.2	198.7	2509	65.08
12/4/2020 16:37	205.4	202.1	198.9	2502	64.95
12/4/2020 16:37	205.4	202	199	2501	64.92
12/4/2020 16:37	205.4	202	199.2	2501	65.04
12/4/2020 16:37	205.4	202	198.9	2503	65
12/4/2020 16:38	205.4	202	198.9	2502	64.99
12/4/2020 16:38	205.4	202.1	199.7	2497	64.99
12/4/2020 16:38	205.4	202	199.6	2496	64.96
12/4/2020 16:38	205.4	202.2	198.7	2496	65.04
12/4/2020 16:38	205.4	202	198.7	2494	64.92
12/4/2020 16:38	205.4	202.2	199	2495	65.04
12/4/2020 16:38	205.4	201.9	199	2496	64.95
12/4/2020 16:38	205.4	202	198.9	2496	64.88
12/4/2020 16:38	205.4	202	199	2494	65.04
12/4/2020 16:38	205.4	201.9	199.7	2497	65.08
12/4/2020 16:38	205.4	201.9	199.2	2497	64.91
12/4/2020 16:38	205.5	201.9	198.6	2497	64.83
12/4/2020 16:38	205.5	202	199	2500	65.04
12/4/2020 16:38	205.4	202	199.2	2496	64.8
12/4/2020 16:38	205.5	202	199	2497	64.99
12/4/2020 16:38	205.4	202.1	198.9	2499	65
12/4/2020 16:38	205.4	202.1	199.6	2496	64.96
12/4/2020 16:38	205.5	202	199.2	2501	64.92
12/4/2020 16:38	205.4	202.2	198.7	2501	65.08
12/4/2020 16:38	205.5	202.1	199	2505	64.88
12/4/2020 16:38	205.5	202	199	2504	65.08
12/4/2020 16:38	205.5	202.1	199	2503	65.08
12/4/2020 16:38	205.4	202.1	198.8	2500	64.99
12/4/2020 16:38	205.4	202.9	199.6	2500	64.96
12/4/2020 16:38	205.5	203.1	199.7	2501	64.96
12/4/2020 16:38	205.4	203.2	198.9	2503	65.04
12/4/2020 16:38	205.4	203.2	198.6	2499	64.91
12/4/2020 16:38	205.5	203	199.1	2498	64.92
12/4/2020 16:38	205.5	203	199.4	2499	65.04
12/4/2020 16:38	205.4	202.9	199.4	2502	65
12/4/2020 16:38	205.4	203.1	199.2	2500	64.99
12/4/2020 16:38	205.4	203	199	2499	65.04
12/4/2020 16:38	205.4	203.2	199.4	2504	65
12/4/2020 16:38	205.5	203	199.8	2503	64.95

12/4/2020 16:38	205.5	203.3	200.1	2508	64.96
12/4/2020 16:38	205.5	203.2	199.5	2509	65.04
12/4/2020 16:38	205.4	203.4	199.4	2505	64.92
12/4/2020 16:38	205.5	203	198.9	2503	65.04
12/4/2020 16:38	205.5	202.7	198.9	2502	65
12/4/2020 16:38	205.5	202.9	198.9	2505	64.96
12/4/2020 16:38	205.4	203.2	198.8	2503	65
12/4/2020 16:38	205.5	203	198.8	2502	65
12/4/2020 16:38	205.5	203	198.9	2509	65
12/4/2020 16:38	205.4	202.7	199	2506	64.96
12/4/2020 16:38	205.5	203.1	198.9	2500	64.88
12/4/2020 16:38	205.5	203.2	198.8	2507	64.96
12/4/2020 16:38	205.5	202.9	198.8	2503	64.99
12/4/2020 16:38	205.5	203.2	199.2	2505	65
12/4/2020 16:38	205.5	202.7	199.6	2507	64.88
12/4/2020 16:38	205.5	203.4	199.9	2504	64.99
12/4/2020 16:38	205.5	203.2	199.7	2505	65.12
12/4/2020 16:38	205.6	202.9	199.2	2505	64.93
12/4/2020 16:38	205.5	203.1	199	2500	64.88
12/4/2020 16:38	205.6	202.9	199.2	2498	64.96
12/4/2020 16:38	205.5	203.1	199.2	2502	65.04
12/4/2020 16:38	205.5	203.4	199	2504	65
12/4/2020 16:38	205.6	203.1	199	2506	64.84
12/4/2020 16:38	205.6	203.2	199.5	2505	65.04
12/4/2020 16:38	205.6	202.9	199.7	2506	64.93
12/4/2020 16:38	205.6	203.1	199.2	2507	64.88
12/4/2020 16:39	205.5	202.7	199	2510	65
12/4/2020 16:39	205.6	202.9	199.2	2509	65.04
12/4/2020 16:39	205.5	203.4	199.2	2503	64.84
12/4/2020 16:39	205.6	203	198.9	2502	64.88
12/4/2020 16:39	205.5	203.2	198.8	2509	65
12/4/2020 16:39	205.6	203.2	199.2	2510	65
12/4/2020 16:39	205.6	203	200	2507	64.88
12/4/2020 16:39	205.6	203.3	199.5	2504	64.96
12/4/2020 16:39	205.6	203.2	199.1	2506	65.09
12/4/2020 16:39	205.6	202.9	199	2507	64.96
12/4/2020 16:39	205.5	203	199.4	2509	65
12/4/2020 16:39	205.6	203.1	199.2	2505	65
12/4/2020 16:39	205.6	203.2	199	2507	65.04
12/4/2020 16:39	205.6	203.2	199	2504	64.92
12/4/2020 16:39	205.6	203.3	198.8	2507	64.96
12/4/2020 16:39	205.6	203.3	198.9	2509	65
12/4/2020 16:39	205.5	203	199.5	2510	64.99
12/4/2020 16:39	205.6	203.1	199.8	2510	64.96
12/4/2020 16:39	205.6	202.9	200	2509	64.96
12/4/2020 16:39	205.6	203	199.7	2512	64.96
12/4/2020 16:39	205.5	203.2	199.2	2514	65.04

12/4/2020 16:39	205.6	203	198.9	2515	65.04
12/4/2020 16:39	205.6	203.1	199	2512	65.04
12/4/2020 16:39	205.6	202.8	199.2	2512	65.04
12/4/2020 16:39	205.6	202.9	199.3	2510	64.92
12/4/2020 16:39	205.6	203.3	199.2	2509	64.96
12/4/2020 16:39	205.6	202.9	199.3	2507	64.88
12/4/2020 16:39	205.6	203.2	199.1	2508	65.08
12/4/2020 16:39	205.6	202.8	199.4	2511	64.96
12/4/2020 16:39	205.6	203	199.4	2511	64.83
12/4/2020 16:39	205.6	203.4	199.2	2511	64.88
12/4/2020 16:39	205.6	203.2	199.2	2514	65.12
12/4/2020 16:39	205.6	203.1	199.1	2514	64.96
12/4/2020 16:39	205.6	203.1	198.9	2512	64.92
12/4/2020 16:39	205.6	202.9	199.4	2509	64.92
12/4/2020 16:39	205.6	203.4	199.7	2508	64.96
12/4/2020 16:39	205.6	202.9	199.7	2508	64.91
12/4/2020 16:39	205.6	203.2	199.1	2507	64.92
12/4/2020 16:39	205.6	202.7	198.9	2508	65.04
12/4/2020 16:39	205.6	203.3	199.2	2504	64.92
12/4/2020 16:39	205.6	203.4	199.3	2505	64.96
12/4/2020 16:39	205.6	203	199.2	2505	65.04
12/4/2020 16:39	205.6	203	199	2504	65
12/4/2020 16:39	205.6	203.2	199.9	2504	65
12/4/2020 16:39	205.6	202.9	199.4	2506	64.96
12/4/2020 16:39	205.6	203.3	198.8	2507	65
12/4/2020 16:39	205.9	203.2	199	2507	64.96
12/4/2020 16:39	205.7	203.2	199.2	2510	65
12/4/2020 16:39	205.6	203	199	2514	65.04
12/4/2020 16:39	205.6	203.2	199.1	2511	64.96
12/4/2020 16:39	205.7	203.2	200.1	2514	64.96
12/4/2020 16:39	205.7	203	199.6	2514	64.96
12/4/2020 16:39	205.6	203.4	199	2516	65.04
12/4/2020 16:39	205.6	203	199.1	2509	65.04
12/4/2020 16:39	205.7	202.9	199.5	2507	65.04
12/4/2020 16:39	205.7	203.3	199.5	2505	64.96
12/4/2020 16:39	205.7	203	199	2504	64.88
12/4/2020 16:39	205.7	203.2	199.7	2500	64.88
12/4/2020 16:39	205.6	203.2	200	2499	65.04
12/4/2020 16:40	205.7	202.8	199	2497	64.84
12/4/2020 16:40	205.7	202.9	199	2498	65.04
12/4/2020 16:40	205.7	203	199.4	2503	65.04
12/4/2020 16:40	205.6	203	199.4	2505	64.92
12/4/2020 16:40	205.6	202.8	199.2	2502	64.92
12/4/2020 16:40	205.6	203.2	199.8	2498	65
12/4/2020 16:40	205.7	203.3	199.8	2497	64.96
12/4/2020 16:40	205.6	203.1	199.2	2495	65
12/4/2020 16:40	205.7	203.1	199	2496	64.92

12/4/2020 16:40	205.7	202.9	199.5	2498	65.04
12/4/2020 16:40	205.7	202.9	199.4	2499	64.88
12/4/2020 16:40	205.7	203.3	199	2500	64.91
12/4/2020 16:40	205.7	203	199.9	2499	64.96
12/4/2020 16:40	205.7	203.2	199.8	2497	64.96
12/4/2020 16:40	205.7	203	199	2496	64.92
12/4/2020 16:40	205.7	203	199.2	2498	65
12/4/2020 16:40	205.7	203.3	199.4	2499	64.92
12/4/2020 16:40	205.7	203	199.4	2499	65
12/4/2020 16:40	205.7	203.2	199.1	2503	65
12/4/2020 16:40	205.8	203.2	199.6	2502	65.09
12/4/2020 16:40	205.7	203	200	2499	65
12/4/2020 16:40	205.7	203.4	199	2500	64.92
12/4/2020 16:40	205.8	202.9	199.1	2498	65.04
12/4/2020 16:40	205.7	203.4	199.5	2496	64.88
12/4/2020 16:40	205.6	202.9	199.3	2492	65
12/4/2020 16:40	205.7	202.7	198.9	2492	64.92
12/4/2020 16:40	205.7	203.1	199.7	2496	64.83
12/4/2020 16:40	205.8	203	199.7	2497	64.99
12/4/2020 16:40	205.7	203	198.9	2497	64.96
12/4/2020 16:40	205.7	203.2	199	2498	65.08
12/4/2020 16:40	205.7	203.4	199.4	2497	65.04
12/4/2020 16:40	205.7	203	199.4	2494	64.99
12/4/2020 16:40	205.7	202.7	199	2498	65.08
12/4/2020 16:40	205.7	203.1	199.8	2501	64.92
12/4/2020 16:40	205.7	202.9	199.8	2496	64.88
12/4/2020 16:40	205.7	203.2	198.9	2499	64.89
12/4/2020 16:40	205.7	203.3	199.3	2500	64.83
12/4/2020 16:40	205.7	203.1	199.4	2496	64.92
12/4/2020 16:40	205.7	203	199.4	2495	65.08
12/4/2020 16:40	205.8	203.2	199.2	2498	65
12/4/2020 16:40	205.7	203.3	199.4	2502	64.88
12/4/2020 16:40	205.7	203	200.1	2497	64.88
12/4/2020 16:40	205.7	202.9	199.5	2497	65.08
12/4/2020 16:40	205.7	202.9	198.9	2496	64.92
12/4/2020 16:40	205.8	202.7	199.2	2499	64.96
12/4/2020 16:40	205.7	203.2	199.4	2499	65.08
12/4/2020 16:40	205.7	203.3	199.4	2500	64.92
12/4/2020 16:40	205.7	203.1	199.1	2498	64.92
12/4/2020 16:40	205.7	203.2	199.7	2500	65.08
12/4/2020 16:40	205.7	203.3	200	2501	65
12/4/2020 16:40	205.7	202.7	199.2	2499	65
12/4/2020 16:40	205.8	203.3	199	2502	64.99
12/4/2020 16:40	205.7	203.1	199.6	2500	65
12/4/2020 16:40	205.7	203.4	199.5	2498	64.96
12/4/2020 16:40	205.7	203.2	199	2500	65
12/4/2020 16:40	205.8	202.7	199.9	2499	65.04

12/4/2020 16:40	205.7	203.4	200.1	2495	65
12/4/2020 16:40	205.8	203	199.1	2493	64.96
12/4/2020 16:40	205.8	203.2	199.3	2493	65.04
12/4/2020 16:40	205.8	203.4	199.5	2496	64.92
12/4/2020 16:41	205.7	203.3	199.5	2500	64.92
12/4/2020 16:41	205.8	203	199.2	2496	64.92
12/4/2020 16:41	205.7	203	199.7	2502	64.96
12/4/2020 16:41	205.8	203.2	200.3	2498	64.88
12/4/2020 16:41	205.7	203.4	199.2	2499	65.04
12/4/2020 16:41	205.8	202.9	199.2	2500	64.96
12/4/2020 16:41	205.7	203.4	199.6	2499	64.99
12/4/2020 16:41	205.7	203.1	199.5	2493	64.88
12/4/2020 16:41	205.8	203	199.3	2495	65.16
12/4/2020 16:41	205.8	202.8	199.7	2497	65.04
12/4/2020 16:41	205.7	203.1	200.3	2502	64.88
12/4/2020 16:41	205.8	203.2	199	2503	64.99
12/4/2020 16:41	205.8	202.7	199.1	2500	64.96
12/4/2020 16:41	205.8	203	199.5	2502	64.88
12/4/2020 16:41	205.8	202.9	199.5	2498	65.04
12/4/2020 16:41	205.7	202.9	199.1	2499	64.96
12/4/2020 16:41	205.8	203.2	199.9	2499	64.96
12/4/2020 16:41	205.8	203	200.1	2497	64.96
12/4/2020 16:41	205.8	203.4	199.2	2500	65.08
12/4/2020 16:41	205.8	202.7	199	2503	65
12/4/2020 16:41	205.8	202.8	199.4	2501	64.92
12/4/2020 16:41	205.7	203.4	199.5	2503	64.96
12/4/2020 16:41	205.8	203	199.2	2503	65
12/4/2020 16:41	205.7	202.8	199.9	2503	64.96
12/4/2020 16:41	205.7	202.9	199.9	2500	64.92
12/4/2020 16:41	205.8	203.1	199	2501	65
12/4/2020 16:41	205.7	203.1	199.4	2498	64.91
12/4/2020 16:41	205.7	203.1	199.4	2497	65
12/4/2020 16:41	205.7	203	199.4	2500	65
12/4/2020 16:41	205.7	202.8	199.5	2500	64.96
12/4/2020 16:41	205.8	203	200.1	2502	65
12/4/2020 16:41	205.8	203.4	199.4	2500	64.92
12/4/2020 16:41	205.7	203.2	199.2	2503	64.96
12/4/2020 16:41	205.8	202.8	199.4	2503	64.96
12/4/2020 16:41	205.7	203.3	199.6	2499	64.91
12/4/2020 16:41	205.8	202.7	199.4	2504	65.04
12/4/2020 16:41	205.7	203.3	199.5	2502	64.8
12/4/2020 16:41	205.8	203.2	200.4	2503	65.04
12/4/2020 16:41	205.7	203.3	199	2503	64.93
12/4/2020 16:41	205.8	203	199.2	2504	65.04
12/4/2020 16:41	205.8	202.8	199.6	2500	64.93
12/4/2020 16:41	205.8	203.3	199.4	2499	64.92
12/4/2020 16:41	205.8	202.7	199.4	2496	65.08

12/4/2020 16:41	205.8	203.2	200.2	2500	65.03
12/4/2020 16:41	205.8	203.3	199.7	2505	64.92
12/4/2020 16:41	205.7	202.9	199.2	2512	64.96
12/4/2020 16:41	205.8	203.2	199.4	2511	64.88
12/4/2020 16:41	205.8	202.9	199.6	2507	64.84
12/4/2020 16:41	205.7	202.9	199.5	2507	64.96
12/4/2020 16:41	205.8	203.2	199.1	2503	64.99
12/4/2020 16:41	205.8	203.2	200.1	2500	65.04
12/4/2020 16:41	205.7	203.4	200.1	2503	65.04
12/4/2020 16:41	205.8	203	199.3	2501	64.92
12/4/2020 16:41	205.8	202.8	199.2	2497	64.88
12/4/2020 16:41	205.8	203.4	199.5	2496	64.92
12/4/2020 16:41	205.8	203.1	199.5	2497	65
12/4/2020 16:41	205.8	203.3	199.5	2496	64.96
12/4/2020 16:41	205.8	203.1	199.1	2496	64.99
12/4/2020 16:41	205.8	202.8	199.6	2498	65.04
12/4/2020 16:42	205.8	202.9	200.2	2500	64.96
12/4/2020 16:42	205.8	203	200.1	2502	65
12/4/2020 16:42	205.8	203.2	199.5	2503	65.04
12/4/2020 16:42	205.8	202.7	199.1	2502	65
12/4/2020 16:42	205.8	203.2	199.7	2502	64.96
12/4/2020 16:42	205.8	203.4	199.7	2503	64.96
12/4/2020 16:42	205.8	203	199.5	2498	65
12/4/2020 16:42	205.8	203.1	199.5	2498	64.92
12/4/2020 16:42	205.8	202.9	199.4	2499	65.04
12/4/2020 16:42	205.8	203.3	200.2	2496	65.04
12/4/2020 16:42	205.7	203.4	200.2	2498	64.92
12/4/2020 16:42	205.8	202.9	199.4	2496	64.96
12/4/2020 16:42	205.9	203.2	199.5	2498	65.08
12/4/2020 16:42	205.8	203	199.5	2496	65.04
12/4/2020 16:42	205.8	202.9	199.5	2498	65.08
12/4/2020 16:42	205.8	203.4	199.6	2500	64.93
12/4/2020 16:42	205.8	203.1	199.4	2500	65.04
12/4/2020 16:42	205.8	203.2	200.2	2497	64.88
12/4/2020 16:42	205.8	203	200.4	2498	65.04
12/4/2020 16:42	205.8	202.9	199.5	2502	65.04
12/4/2020 16:42	205.8	203.4	199.4	2502	65
12/4/2020 16:42	205.8	203.2	199.5	2504	65.01
12/4/2020 16:42	205.8	203.4	199.6	2500	65.04
12/4/2020 16:42	205.8	203	199.6	2504	64.96
12/4/2020 16:42	205.8	203.2	199.4	2501	65
12/4/2020 16:42	205.8	203.3	199.4	2501	65
12/4/2020 16:42	205.8	203	200.2	2505	65.04
12/4/2020 16:42	205.8	203.3	200.2	2507	64.92
12/4/2020 16:42	205.8	203	199.4	2508	65
12/4/2020 16:42	205.8	202.8	199.4	2505	64.92
12/4/2020 16:42	205.9	203.3	199.4	2505	65

12/4/2020 16:42	205.8	203.1	199.7	2507	65
12/4/2020 16:42	205.8	203.4	199.6	2507	65.08
12/4/2020 16:42	205.8	203.3	199.5	2508	64.96
12/4/2020 16:42	205.8	203.2	199.4	2509	65
12/4/2020 16:42	205.9	203.3	199.5	2507	64.92
12/4/2020 16:42	205.8	203	199.8	2508	65.08
12/4/2020 16:42	205.8	202.9	200.4	2513	64.96
12/4/2020 16:42	205.8	203.4	200.2	2509	65.08
12/4/2020 16:42	205.8	203.1	199.8	2506	64.84
12/4/2020 16:42	205.8	203.1	199.2	2505	64.84
12/4/2020 16:42	205.8	202.9	199.4	2503	64.96
12/4/2020 16:42	205.9	202.7	199.5	2504	64.8
12/4/2020 16:42	205.9	202.9	199.5	2505	65.04
12/4/2020 16:42	205.8	203.1	199.6	2505	64.96
12/4/2020 16:42	205.9	203.4	199.5	2504	64.84
12/4/2020 16:42	205.8	203.1	199.6	2503	64.92
12/4/2020 16:42	205.9	203	199.6	2507	64.96
12/4/2020 16:42	205.9	203.4	199.4	2511	65.08
12/4/2020 16:42	205.9	202.9	199.4	2511	64.88
12/4/2020 16:42	205.8	203.1	199.6	2506	65
12/4/2020 16:42	205.8	202.9	199.9	2513	64.96
12/4/2020 16:42	205.9	203	200.3	2511	65
12/4/2020 16:42	205.8	203.3	200.5	2507	64.92
12/4/2020 16:42	205.9	203.2	200.3	2507	64.96
12/4/2020 16:42	205.9	203.2	199.6	2506	64.96
12/4/2020 16:42	205.9	203.1	199.2	2507	64.96
12/4/2020 16:42	205.8	202.9	199.1	2507	65
12/4/2020 16:42	205.8	203.3	199.3	2508	65.05
12/4/2020 16:43	205.8	203	199.5	2502	64.96
12/4/2020 16:43	205.9	203.2	199.7	2500	64.96
12/4/2020 16:43	205.9	203.2	199.5	2500	64.96
12/4/2020 16:43	205.8	202.9	199.5	2504	64.92
12/4/2020 16:43	205.9	203.3	199.7	2503	65.04
12/4/2020 16:43	205.9	203	199.5	2499	65.05
12/4/2020 16:43	205.8	203.3	199.5	2502	64.88
12/4/2020 16:43	205.8	202.8	199.2	2499	64.92
12/4/2020 16:43	205.9	203.2	199.4	2503	65
12/4/2020 16:43	205.9	203.3	199.5	2502	64.92
12/4/2020 16:43	205.9	202.9	199.5	2501	64.88
12/4/2020 16:43	205.9	203.1	199.5	2500	65.01
12/4/2020 16:43	205.8	203.1	199.5	2504	64.99
12/4/2020 16:43	205.9	203.2	199.6	2508	64.96
12/4/2020 16:43	205.8	203.3	199.6	2513	65.12
12/4/2020 16:43	205.9	203.2	199.7	2514	64.92
12/4/2020 16:43	205.9	203.1	199.7	2509	64.92
12/4/2020 16:43	205.8	203.2	199.7	2505	64.92
12/4/2020 16:43	205.9	203	199.7	2509	65.04

12/4/2020 16:43	205.8	203.3	199.8	2509	64.88
12/4/2020 16:43	205.8	203.1	199.6	2507	64.92
12/4/2020 16:43	205.8	203.3	199.5	2509	65
12/4/2020 16:43	205.8	203.2	199.4	2507	65
12/4/2020 16:43	205.8	202.8	199.5	2507	65
12/4/2020 16:43	205.9	203.4	200.1	2508	64.96
12/4/2020 16:43	205.9	202.7	200.6	2507	64.91
12/4/2020 16:43	205.9	203.3	200.3	2505	64.92
12/4/2020 16:43	205.9	203.1	199.8	2505	65
12/4/2020 16:43	205.9	202.9	199.5	2504	64.96
12/4/2020 16:43	205.8	203.1	199.6	2503	64.92
12/4/2020 16:43	205.9	202.9	199.6	2505	64.96
12/4/2020 16:43	205.9	203.2	199.7	2507	65.05
12/4/2020 16:43	205.9	202.8	199.8	2508	64.96
12/4/2020 16:43	205.9	202.7	199.8	2505	64.92
12/4/2020 16:43	205.9	203.3	199.6	2509	65.12
12/4/2020 16:43	205.8	202.9	199.6	2509	65
12/4/2020 16:43	205.9	203.2	199.4	2509	64.88
12/4/2020 16:43	205.9	203.3	199.4	2509	64.92
12/4/2020 16:43	205.9	202.8	199.4	2507	64.92
12/4/2020 16:43	205.9	203.3	199.3	2505	64.99
12/4/2020 16:43	205.9	203	199.5	2507	64.99
12/4/2020 16:43	205.9	203.1	199.6	2504	64.92
12/4/2020 16:43	205.9	203.2	199.4	2504	65.03
12/4/2020 16:43	205.8	202.8	199.3	2503	64.99
12/4/2020 16:43	205.9	203.4	199.4	2500	65.08
12/4/2020 16:43	205.9	203	199.4	2502	65.04
12/4/2020 16:43	205.9	203.4	199.5	2502	65.08
12/4/2020 16:43	205.9	202.9	199.6	2504	65.03
12/4/2020 16:43	205.9	202.9	199.8	2504	65.04
12/4/2020 16:43	205.9	203.3	200.1	2505	65.04
12/4/2020 16:43	205.8	203.1	199.7	2501	64.96
12/4/2020 16:43	205.9	203.3	199.6	2504	65.08
12/4/2020 16:43	205.9	202.9	199.6	2503	65.04
12/4/2020 16:43	205.9	202.9	200.5	2501	65.04
12/4/2020 16:43	205.8	203.3	200.5	2503	65
12/4/2020 16:43	205.9	203.2	199.6	2507	65
12/4/2020 16:43	205.9	203.1	199.5	2505	65
12/4/2020 16:43	205.8	202.7	199.7	2507	65
12/4/2020 16:43	205.9	202.8	199.6	2507	65
12/4/2020 16:43	205.9	203.2	199.7	2510	64.96
12/4/2020 16:44	205.8	203	199.6	2512	65
12/4/2020 16:44	205.8	203.1	200.4	2510	64.99
12/4/2020 16:44	205.8	203.3	199.7	2505	65.04
12/4/2020 16:44	205.8	203	199.5	2502	65.04
12/4/2020 16:44	205.9	203.2	199.7	2507	65.08
12/4/2020 16:44	205.9	203	199.9	2505	64.88

12/4/2020 16:44	205.9	203.5	199.7	2505	64.84
12/4/2020 16:44	205.9	203.3	199.6	2503	64.96
12/4/2020 16:44	205.9	202.9	200.4	2499	64.88
12/4/2020 16:44	205.9	203.2	200.4	2502	64.88
12/4/2020 16:44	205.8	203	199.7	2507	65.08
12/4/2020 16:44	205.9	203.4	199.3	2507	64.88
12/4/2020 16:44	205.9	203.2	199.5	2505	64.92
12/4/2020 16:44	205.8	202.8	199.8	2504	65.09
12/4/2020 16:44	205.9	203	199.8	2505	64.83
12/4/2020 16:44	205.9	203	199.9	2501	65.04
12/4/2020 16:44	205.9	203	199.7	2504	65.04
12/4/2020 16:44	205.8	203.4	199.4	2507	65.04
12/4/2020 16:44	205.8	202.9	199.9	2507	64.96
12/4/2020 16:44	205.9	203.1	200.6	2511	65.04
12/4/2020 16:44	205.9	203	200.1	2509	64.92
12/4/2020 16:44	205.9	203	199.5	2509	64.92
12/4/2020 16:44	205.8	203.3	199.4	2508	64.99
12/4/2020 16:44	205.8	202.7	199.8	2503	65
12/4/2020 16:44	205.9	203.1	199.8	2503	64.99
12/4/2020 16:44	205.8	203.2	199.7	2503	64.96
12/4/2020 16:44	205.8	203.3	199.5	2506	64.96
12/4/2020 16:44	205.9	203.2	200.2	2505	64.96
12/4/2020 16:44	205.8	203	200.5	2504	64.96
12/4/2020 16:44	205.8	203.2	199.7	2508	65.04
12/4/2020 16:44	205.8	202.9	199.5	2505	64.96
12/4/2020 16:44	205.9	202.9	199.7	2509	65
12/4/2020 16:44	205.9	203.4	199.7	2509	65.04
12/4/2020 16:44	205.9	202.9	200	2508	64.96
12/4/2020 16:44	205.8	203.4	199.7	2506	64.91
12/4/2020 16:44	205.8	203	199.5	2504	64.96
12/4/2020 16:44	205.9	202.8	199.6	2505	64.96
12/4/2020 16:44	205.9	203.4	200.4	2504	64.96
12/4/2020 16:44	205.9	203	200.4	2506	65
12/4/2020 16:44	205.9	203	200.1	2510	65.08
12/4/2020 16:44	205.8	203.2	199.4	2510	64.99
12/4/2020 16:44	205.9	203.4	199.6	2505	64.99
12/4/2020 16:44	205.9	203	199.6	2506	65.04
12/4/2020 16:44	205.9	202.9	199.9	2510	64.88
12/4/2020 16:44	205.9	203.2	200.1	2511	64.99
12/4/2020 16:44	205.9	203.2	199.9	2514	64.96
12/4/2020 16:44	205.9	203.1	199.7	2513	65.08
12/4/2020 16:44	205.9	203.3	200.1	2513	64.96
12/4/2020 16:44	205.9	203	200.7	2515	65.03
12/4/2020 16:44	205.9	203.3	200.3	2512	64.93
12/4/2020 16:44	205.9	202.9	199.5	2511	65.03
12/4/2020 16:44	205.9	203.2	199.7	2504	64.99
12/4/2020 16:44	205.9	203.3	199.7	2507	65.16

12/4/2020 16:44	205.9	203.1	200.1	2507	65.03
12/4/2020 16:44	205.9	203.3	200.1	2511	65.04
12/4/2020 16:44	206	202.9	199.9	2510	65.09
12/4/2020 16:44	206	203	199.6	2514	64.96
12/4/2020 16:44	205.9	203.4	199.7	2514	64.96
12/4/2020 16:44	205.9	203	200.2	2514	65.05
12/4/2020 16:45	206	203.2	200.8	2511	64.96
12/4/2020 16:45	205.9	202.9	200.6	2508	64.96
12/4/2020 16:45	206	203.2	199.8	2507	64.92
12/4/2020 16:45	206	203.3	199.5	2509	65
12/4/2020 16:45	206	202.9	199.5	2511	64.96
12/4/2020 16:45	205.9	203.1	199.6	2508	64.96
12/4/2020 16:45	206	203.2	199.8	2506	65
12/4/2020 16:45	205.9	203.2	199.8	2508	64.92
12/4/2020 16:45	206	203	199.9	2509	65.08
12/4/2020 16:45	205.9	203.2	199.9	2507	64.96
12/4/2020 16:45	206	203.1	199.9	2505	65
12/4/2020 16:45	206	203	199.6	2503	64.92
12/4/2020 16:45	205.9	203.3	199.5	2505	65
12/4/2020 16:45	206	203.4	199.7	2510	65
12/4/2020 16:45	206.1	202.9	200.1	2509	65.04
12/4/2020 16:45	206	202.7	200.3	2510	64.99
12/4/2020 16:45	206.1	203.4	200.4	2512	65
12/4/2020 16:45	206	202.9	200.6	2516	64.88
12/4/2020 16:45	206	203.2	200.6	2514	65.03
12/4/2020 16:45	206	203	200.6	2518	64.88
12/4/2020 16:45	206.1	203.2	200.8	2517	64.83
12/4/2020 16:45	206.1	203	200.8	2514	64.88
12/4/2020 16:45	206.1	202.9	200.6	2517	64.96
12/4/2020 16:45	206.1	203.2	200.7	2518	64.88
12/4/2020 16:45	206.1	202.9	200.8	2518	64.99
12/4/2020 16:45	206.1	203.1	200.8	2517	64.88
12/4/2020 16:45	206.1	203	200.4	2516	65.12
12/4/2020 16:45	206.1	203	200.4	2511	64.96
12/4/2020 16:45	206.1	203.3	200.1	2510	65
12/4/2020 16:45	206.1	203.1	199.6	2510	64.96
12/4/2020 16:45	206.1	203	199.6	2507	64.99
12/4/2020 16:45	206.1	203.4	199.8	2504	64.96
12/4/2020 16:45	206	203	199.9	2505	65
12/4/2020 16:45	206.1	203.2	200.3	2512	64.92
12/4/2020 16:45	206.1	203.2	199.8	2510	64.96
12/4/2020 16:45	206.1	203.4	199.8	2510	64.96
12/4/2020 16:45	206.1	203	199.5	2510	65.04
12/4/2020 16:45	206	203	199.7	2510	65
12/4/2020 16:45	206.1	203	200.8	2507	65
12/4/2020 16:45	206.1	202.9	200.4	2508	64.96
12/4/2020 16:45	206.1	202.9	199.7	2510	64.96

12/4/2020 16:45	206.1	203.5	199.8	2514	64.96
12/4/2020 16:45	206.1	202.9	200.1	2516	65.09
12/4/2020 16:45	206.1	203.2	200.2	2516	64.96
12/4/2020 16:45	206.2	203.2	199.9	2514	64.92
12/4/2020 16:45	206.1	202.9	199.6	2511	65.05
12/4/2020 16:45	206.1	203.2	199.5	2512	64.92
12/4/2020 16:45	206.1	203	199.5	2509	64.92
12/4/2020 16:45	206.1	203.3	200.1	2516	65.03
12/4/2020 16:45	206.1	203.1	200.2	2514	64.92
12/4/2020 16:45	206.1	202.8	200.4	2513	65.07
12/4/2020 16:45	206.1	203.2	200.5	2514	64.87
12/4/2020 16:45	206.1	202.9	200.6	2514	64.96
12/4/2020 16:45	206.1	203.4	200.7	2515	64.95
12/4/2020 16:45	206.1	203.1	200.6	2513	65
12/4/2020 16:45	206.1	202.9	200.4	2514	64.97
12/4/2020 16:45	206.1	203.3	200.2	2509	65.08
12/4/2020 16:45	206.1	203	199.6	2510	65.12
12/4/2020 16:45	206.1	203.3	199.8	2511	64.96
12/4/2020 16:45	206.1	203.1	199.9	2512	64.96
12/4/2020 16:46	206.1	202.9	199.9	2514	65
12/4/2020 16:46	206.2	202.7	200.1	2516	65.04
12/4/2020 16:46	206.2	202.7	200	2516	64.96
12/4/2020 16:46	206.1	203	199.8	2520	64.92
12/4/2020 16:46	206.1	203.3	199.8	2521	64.92
12/4/2020 16:46	206.1	202.9	200.6	2516	65.04
12/4/2020 16:46	206.2	203.1	200.9	2516	64.92
12/4/2020 16:46	206.1	202.9	199.6	2517	65.04
12/4/2020 16:46	206.1	203	199.8	2516	65.04
12/4/2020 16:46	206.1	203.3	200.1	2517	64.96
12/4/2020 16:46	206.2	202.9	200.2	2511	64.96
12/4/2020 16:46	206.2	203.2	199.7	2514	65.12
12/4/2020 16:46	206.2	202.9	200.6	2512	64.92
12/4/2020 16:46	206.2	203	200.6	2512	64.92
12/4/2020 16:46	206.1	202.9	199.5	2514	65.04
12/4/2020 16:46	206.1	203.3	199.7	2516	64.92
12/4/2020 16:46	206.1	203.2	200.1	2512	64.88
12/4/2020 16:46	206.2	203	200.1	2510	64.84
12/4/2020 16:46	206.1	203	199.7	2512	64.93
12/4/2020 16:46	206.1	203.4	200.2	2514	65.08
12/4/2020 16:46	206.1	203.2	200.8	2514	65.04
12/4/2020 16:46	206.1	203.2	199.7	2514	65.08
12/4/2020 16:46	206.2	202.8	199.8	2517	64.96
12/4/2020 16:46	206.1	202.9	200.1	2520	64.96
12/4/2020 16:46	206.1	203.2	200.1	2522	64.97
12/4/2020 16:46	206.1	203.3	199.9	2518	64.96
12/4/2020 16:46	206.2	203.2	199.8	2518	65
12/4/2020 16:46	206.1	203	200.8	2516	65.05

12/4/2020 16:46	206.1	203.1	200.2	2513	65
12/4/2020 16:46	206.2	203.4	199.6	2511	65
12/4/2020 16:46	206.2	203.2	200.2	2511	65.04
12/4/2020 16:46	206.2	203.2	200.2	2512	65
12/4/2020 16:46	206.1	203.2	199.7	2513	64.96
12/4/2020 16:46	206.1	202.8	200.2	2509	64.87
12/4/2020 16:46	206.2	203.4	200.8	2510	65.04
12/4/2020 16:46	206.2	203.3	200	2510	65
12/4/2020 16:46	206.1	203.2	199.8	2510	64.96
12/4/2020 16:46	206.2	203	199.9	2514	65.04
12/4/2020 16:46	206.2	202.9	200.1	2514	64.96
12/4/2020 16:46	206.1	203.2	199.9	2516	64.99
12/4/2020 16:46	206.2	203.4	200.1	2513	65.12
12/4/2020 16:46	206.2	203.2	200.7	2517	65.08
12/4/2020 16:46	206.2	202.7	200.4	2516	65.08
12/4/2020 16:46	206.2	203	199.8	2516	64.92
12/4/2020 16:46	206.2	203.4	199.9	2513	64.93
12/4/2020 16:46	206.2	203	199.8	2514	64.99
12/4/2020 16:46	206.3	203.2	199.9	2516	64.96
12/4/2020 16:46	206.2	203	200.4	2514	65.04
12/4/2020 16:46	206.2	202.8	200	2514	64.99
12/4/2020 16:46	206.2	203.1	200.1	2513	65
12/4/2020 16:46	206.2	203	199.6	2511	65.12
12/4/2020 16:46	206.3	203.4	199.7	2509	64.96
12/4/2020 16:46	206.2	203.4	200.2	2509	64.96
12/4/2020 16:46	206.2	203	200.3	2510	64.96
12/4/2020 16:46	206.2	203.3	200.6	2512	65.04
12/4/2020 16:46	206.3	202.9	200.8	2513	64.92
12/4/2020 16:46	206.2	203.3	200.7	2513	64.96
12/4/2020 16:46	206.2	203.2	200.9	2514	65
12/4/2020 16:46	206.3	202.7	200.9	2508	64.96
12/4/2020 16:47	206.2	203.2	200.7	2510	65
12/4/2020 16:47	206.2	202.9	201	2515	65.04
12/4/2020 16:47	206.3	203.9	200.8	2513	64.96
12/4/2020 16:47	206.3	204.4	200.8	2514	64.96
12/4/2020 16:47	206.3	203.2	200.6	2514	65.09
12/4/2020 16:47	206.2	203.4	200.4	2510	64.96
12/4/2020 16:47	206.3	203	200.6	2510	64.96
12/4/2020 16:47	206.2	204.4	200.4	2513	64.88
12/4/2020 16:47	206.3	204	200.4	2513	64.92
12/4/2020 16:47	206.3	203.9	200.3	2514	64.83
12/4/2020 16:47	206.2	204.2	200	2509	64.88
12/4/2020 16:47	206.2	203.7	200	2511	64.92
12/4/2020 16:47	206.2	203.9	200	2507	64.88
12/4/2020 16:47	206.3	204.3	199.7	2509	64.92
12/4/2020 16:47	206.3	203.9	200.1	2511	65.12
12/4/2020 16:47	206.3	204.2	200.2	2511	65.03

12/4/2020 16:47	206.3	203.8	200.4	2510	65.04
12/4/2020 16:47	206.2	204.4	200.3	2510	65.12
12/4/2020 16:47	206.3	204.3	200.2	2514	65.12
12/4/2020 16:47	206.3	203.9	200.3	2517	65.04
12/4/2020 16:47	206.3	204	200.4	2518	65.08
12/4/2020 16:47	206.2	203.8	200.3	2517	65.08
12/4/2020 16:47	206.3	203.9	200.4	2514	64.91
12/4/2020 16:47	206.3	204.4	200.1	2513	64.99
12/4/2020 16:47	206.3	203.7	200.4	2515	65.04
12/4/2020 16:47	206.3	204.2	200.4	2520	64.96
12/4/2020 16:47	206.2	203.8	200.2	2517	64.96
12/4/2020 16:47	206.3	204.2	200.4	2521	65
12/4/2020 16:47	206.2	204	200.1	2517	64.92
12/4/2020 16:47	206.2	203.9	200.1	2517	64.96
12/4/2020 16:47	206.3	204.4	200.3	2517	65.04
12/4/2020 16:47	206.3	204.4	200.2	2519	65
12/4/2020 16:47	206.3	204.4	200.2	2518	64.96
12/4/2020 16:47	206.3	204.2	200.2	2516	65
12/4/2020 16:47	206.3	203.9	200.1	2518	65.09
12/4/2020 16:47	206.3	203.9	200.3	2517	64.88
12/4/2020 16:47	206.3	204.3	200.2	2517	64.96
12/4/2020 16:47	206.3	203.6	200.1	2518	65.05
12/4/2020 16:47	206.3	204.4	200.3	2518	64.88
12/4/2020 16:47	206.3	203.9	200.1	2516	65.04
12/4/2020 16:47	206.3	204.3	200.2	2519	64.96
12/4/2020 16:47	206.3	204.3	199.9	2517	65.03
12/4/2020 16:47	206.3	203.7	199.6	2520	65.08
12/4/2020 16:47	206.3	204.5	200.2	2517	64.92
12/4/2020 16:47	206.3	203.9	200.4	2520	65.08
12/4/2020 16:47	206.3	204.3	201.1	2518	65.04
12/4/2020 16:47	206.3	204.3	200.9	2514	64.88
12/4/2020 16:47	206.3	203.7	200	2517	65.12
12/4/2020 16:47	206.3	204.5	199.9	2517	64.88
12/4/2020 16:47	206.3	204.2	200.2	2517	64.96
12/4/2020 16:47	206.3	204.2	200.2	2520	64.96
12/4/2020 16:47	206.3	204	200.1	2520	65.04
12/4/2020 16:47	206.3	203.7	199.8	2524	64.92
12/4/2020 16:47	206.3	204.4	200.9	2523	65
12/4/2020 16:47	206.3	204.1	200.2	2524	64.96
12/4/2020 16:47	206.3	204.4	199.9	2524	64.92
12/4/2020 16:47	206.3	203.9	200.2	2521	65
12/4/2020 16:47	206.3	204.2	200.1	2522	65.09
12/4/2020 16:47	206.3	204.6	199.9	2518	64.92
12/4/2020 16:47	206.3	204	201.1	2518	65
12/4/2020 16:48	206.3	204.4	200.2	2521	65
12/4/2020 16:48	206.3	203.9	200	2518	65
12/4/2020 16:48	206.3	203.8	200.2	2518	64.96

12/4/2020 16:48	206.3	204.4	200.1	2521	65.08
12/4/2020 16:48	206.3	203.8	200.1	2520	64.88
12/4/2020 16:48	206.3	204.2	200.2	2518	64.99
12/4/2020 16:48	206.3	204.4	201	2520	64.99
12/4/2020 16:48	206.3	203.6	200.4	2525	65.12
12/4/2020 16:48	206.3	204.2	199.6	2517	64.96
12/4/2020 16:48	206.3	203.7	200.2	2521	64.96
12/4/2020 16:48	206.3	204	200.4	2514	64.93
12/4/2020 16:48	206.3	204.4	200.1	2514	65.04
12/4/2020 16:48	206.3	203.9	200	2514	65.04
12/4/2020 16:48	206.3	204.5	200.9	2520	64.99
12/4/2020 16:48	206.3	204.2	200.4	2524	64.95
12/4/2020 16:48	206.3	204.1	199.9	2518	64.88
12/4/2020 16:48	206.5	204.5	200	2518	64.88
12/4/2020 16:48	206.3	204.3	200.4	2519	65.08
12/4/2020 16:48	206.3	204.4	199.9	2513	64.8
12/4/2020 16:48	206.3	203.9	200.2	2511	65.04
12/4/2020 16:48	206.3	203.9	201	2513	64.96
12/4/2020 16:48	206.3	204.4	199.9	2511	64.88
12/4/2020 16:48	206.3	204.2	199.9	2512	65.08
12/4/2020 16:48	206.3	204.4	200.3	2512	64.99
12/4/2020 16:48	206.3	204.2	200.2	2514	64.96
12/4/2020 16:48	206.3	204.6	200.1	2511	65.04
12/4/2020 16:48	206.3	204.6	200.5	2514	64.92
12/4/2020 16:48	206.3	204.3	201.1	2516	65
12/4/2020 16:48	206.4	204.4	199.9	2517	64.96
12/4/2020 16:48	206.3	204.1	199.7	2515	64.96
12/4/2020 16:48	206.3	204.1	200.3	2511	65
12/4/2020 16:48	206.3	204.3	200.3	2511	64.87
12/4/2020 16:48	206.3	204.2	199.9	2507	64.96
12/4/2020 16:48	206.3	204.6	200.3	2513	65
12/4/2020 16:48	206.3	204.2	201.1	2513	64.96
12/4/2020 16:48	206.3	203.9	200.4	2513	65
12/4/2020 16:48	206.3	204.4	199.9	2511	64.96
12/4/2020 16:48	206.4	204	200.1	2514	64.92
12/4/2020 16:48	206.3	204.3	200.2	2516	65
12/4/2020 16:48	206.3	204.4	200.3	2518	64.92
12/4/2020 16:48	206.3	203.7	199.9	2520	64.92
12/4/2020 16:48	206.4	204.5	200.9	2522	64.91
12/4/2020 16:48	206.4	203.9	200.2	2522	65
12/4/2020 16:48	206.3	204.4	199.7	2529	64.96
12/4/2020 16:48	206.4	204.3	200.2	2529	64.92
12/4/2020 16:48	206.3	204.4	200.1	2526	65
12/4/2020 16:48	206.4	204.1	200.2	2521	65.04
12/4/2020 16:48	206.4	204.1	199.9	2514	64.95
12/4/2020 16:48	206.4	204	200.8	2512	64.92
12/4/2020 16:48	206.3	204.3	200.9	2508	64.88

12/4/2020 16:48	206.4	204.6	199.6	2509	64.92
12/4/2020 16:48	206.3	204.6	199.7	2510	64.83
12/4/2020 16:48	206.4	203.9	200.4	2513	65
12/4/2020 16:48	206.3	204	200.2	2514	65.16
12/4/2020 16:48	206.3	203.7	200.1	2510	64.84
12/4/2020 16:48	206.3	203.8	200.4	2509	64.89
12/4/2020 16:48	206.4	204.5	200.8	2507	64.93
12/4/2020 16:48	206.3	203.7	199.9	2507	65
12/4/2020 16:48	206.4	204	199.9	2510	64.88
12/4/2020 16:49	206.4	204.6	200.2	2510	64.84
12/4/2020 16:49	206.4	203.6	200.4	2514	64.93
12/4/2020 16:49	206.5	204.6	199.9	2510	64.88
12/4/2020 16:49	206.4	204.1	200.8	2509	64.8
12/4/2020 16:49	206.4	204.2	200.8	2510	64.96
12/4/2020 16:49	206.5	203.9	199.6	2509	64.92
12/4/2020 16:49	206.4	204.4	199.9	2510	64.92
12/4/2020 16:49	206.4	204.1	200.2	2515	65.04
12/4/2020 16:49	206.4	203.9	200.4	2514	65
12/4/2020 16:49	206.4	204.3	200.2	2513	64.88
12/4/2020 16:49	206.4	204.3	200.1	2507	64.92
12/4/2020 16:49	206.3	203.7	201.2	2509	65
12/4/2020 16:49	206.4	204	200.6	2503	64.96
12/4/2020 16:49	206.4	203.8	200	2504	65
12/4/2020 16:49	206.4	204.4	200.4	2509	64.96
12/4/2020 16:49	206.3	204.3	200.4	2510	64.92
12/4/2020 16:49	206.4	203.6	200.1	2508	65
12/4/2020 16:49	206.4	204.6	200.5	2509	65
12/4/2020 16:49	206.4	204.1	201.1	2506	65
12/4/2020 16:49	206.4	204.1	200.1	2503	65
12/4/2020 16:49	206.4	204.2	200.1	2505	65
12/4/2020 16:49	206.3	204.3	200.4	2509	65.01
12/4/2020 16:49	206.4	204.4	200.4	2509	64.99
12/4/2020 16:49	206.4	204	200.1	2508	64.84
12/4/2020 16:49	206.4	204.2	200.7	2508	65.08
12/4/2020 16:49	206.5	203.7	200.9	2506	64.99
12/4/2020 16:49	206.4	204.2	199.8	2504	65.08
12/4/2020 16:49	206.4	204.6	200.2	2507	64.96
12/4/2020 16:49	206.4	204.2	200.6	2503	64.95
12/4/2020 16:49	206.4	204.4	200.4	2504	64.92
12/4/2020 16:49	206.5	203.7	200.1	2503	64.92
12/4/2020 16:49	206.5	203.9	200.8	2509	65.08
12/4/2020 16:49	206.3	204.4	200.9	2508	64.83
12/4/2020 16:49	206.4	203.9	199.9	2507	64.99
12/4/2020 16:49	206.5	204.8	200.3	2509	64.96
12/4/2020 16:49	206.5	204.4	200.6	2508	65.04
12/4/2020 16:49	206.5	203.7	200.4	2509	64.96
12/4/2020 16:49	206.4	204.4	200.2	2512	64.92

12/4/2020 16:49	206.5	203.7	201.2	2508	65.04
12/4/2020 16:49	206.5	203.7	200.1	2509	65.04
12/4/2020 16:49	206.5	204.4	200.1	2509	64.96
12/4/2020 16:49	206.4	203.7	200.5	2511	65
12/4/2020 16:49	206.5	204.3	200.3	2511	64.92
12/4/2020 16:49	206.4	203.9	200.2	2511	64.92
12/4/2020 16:49	206.4	204.5	200.9	2514	65.05
12/4/2020 16:49	206.4	204.3	200.2	2508	65.04
12/4/2020 16:49	206.4	203.7	200.2	2510	65.04
12/4/2020 16:49	206.4	204.2	200.4	2512	65.04
12/4/2020 16:49	206.4	203.7	200.4	2509	65.04
12/4/2020 16:49	206.4	203.9	200.1	2513	64.96
12/4/2020 16:49	206.4	204.6	200.9	2512	65.04
12/4/2020 16:49	206.4	204.1	200.7	2511	65.04
12/4/2020 16:49	206.4	204.4	199.8	2509	64.92
12/4/2020 16:49	206.4	204.3	200.1	2509	64.88
12/4/2020 16:49	206.4	204.1	200.4	2508	65.08
12/4/2020 16:49	206.4	203.9	200.1	2507	65
12/4/2020 16:49	206.5	203.7	200.6	2505	64.88
12/4/2020 16:49	206.5	204.4	201.2	2510	64.88
12/4/2020 16:49	206.5	204.3	200.1	2504	65.08
12/4/2020 16:50	206.5	203.9	200.2	2505	64.88
12/4/2020 16:50	206.5	204.5	200.3	2507	65.08
12/4/2020 16:50	206.5	203.9	200.4	2508	64.96
12/4/2020 16:50	206.4	204.1	199.9	2508	64.92
12/4/2020 16:50	206.5	203.7	200.4	2507	64.99
12/4/2020 16:50	206.5	204.5	201.2	2511	65
12/4/2020 16:50	206.5	204.5	200.4	2511	64.99
12/4/2020 16:50	206.5	203.6	199.9	2509	65.08
12/4/2020 16:50	206.4	204.3	200.2	2510	65.04
12/4/2020 16:50	206.4	203.8	200.4	2510	64.87
12/4/2020 16:50	206.4	204.4	200.4	2510	65
12/4/2020 16:50	206.5	204.3	200.1	2514	65
12/4/2020 16:50	206.5	203.8	200.9	2517	65
12/4/2020 16:50	206.5	204.1	200.6	2513	64.96
12/4/2020 16:50	206.4	203.6	199.9	2514	64.96
12/4/2020 16:50	206.5	204.5	200.4	2514	65.04
12/4/2020 16:50	206.5	204.3	200.4	2510	64.92
12/4/2020 16:50	206.5	203.8	200.2	2511	65.12
12/4/2020 16:50	206.5	204.3	200.4	2513	65.04
12/4/2020 16:50	206.5	204.4	201	2515	65.04
12/4/2020 16:50	206.5	203.7	199.8	2509	64.88
12/4/2020 16:50	206.5	204.5	200.4	2507	65.08
12/4/2020 16:50	206.4	204	200.4	2509	64.96
12/4/2020 16:50	206.5	204.3	200.4	2506	65.03
12/4/2020 16:50	206.5	203.9	200.3	2506	65.08
12/4/2020 16:50	206.4	204.5	201.3	2509	65.16

12/4/2020 16:50	206.4	204.4	200.2	2509	64.96
12/4/2020 16:50	206.5	203.9	200.2	2513	65.04
12/4/2020 16:50	206.4	204.6	200.4	2511	64.92
12/4/2020 16:50	206.5	203.6	200.4	2512	65.08
12/4/2020 16:50	206.5	203.9	200.4	2514	64.92
12/4/2020 16:50	206.5	204.4	201.2	2514	64.99
12/4/2020 16:50	206.5	203.9	200.2	2514	65.04
12/4/2020 16:50	206.5	204.3	200.4	2512	65
12/4/2020 16:50	206.5	203.9	200.4	2515	65.04
12/4/2020 16:50	206.5	203.9	200.4	2518	65.08
12/4/2020 16:50	206.6	204.5	200.3	2518	65.04
12/4/2020 16:50	206.5	204	201.5	2516	64.92
12/4/2020 16:50	206.5	204.3	200.2	2517	65
12/4/2020 16:50	206.4	203.7	200.4	2516	64.96
12/4/2020 16:50	206.5	203.7	200.5	2517	64.96
12/4/2020 16:50	206.5	204.6	200.4	2516	64.96
12/4/2020 16:50	206.6	203.7	200.4	2510	64.96
12/4/2020 16:50	206.5	204.6	201.2	2510	64.96
12/4/2020 16:50	206.5	204.3	200.4	2507	64.96
12/4/2020 16:50	206.5	203.7	200.2	2511	64.92
12/4/2020 16:50	206.5	203.8	200.4	2510	64.99
12/4/2020 16:50	206.6	203.9	200.4	2510	65.08
12/4/2020 16:50	206.5	204.2	200.4	2514	65.08
12/4/2020 16:50	206.5	204.4	200.5	2513	65.04
12/4/2020 16:50	206.6	204.1	201.3	2512	64.88
12/4/2020 16:50	206.5	204.2	200.1	2517	64.93
12/4/2020 16:50	206.5	203.9	200.1	2514	64.99
12/4/2020 16:50	206.6	204.2	200.5	2514	64.92
12/4/2020 16:50	206.5	204.2	200.5	2517	64.96
12/4/2020 16:50	206.6	204.4	200.6	2518	65.08
12/4/2020 16:50	206.6	204.4	201.4	2513	64.88
12/4/2020 16:50	206.6	203.7	200.5	2509	65.03
12/4/2020 16:50	206.6	204.2	200.1	2516	65.08
12/4/2020 16:50	206.5	204.3	200.3	2517	64.96
12/4/2020 16:51	206.6	204.3	200.7	2516	64.96
12/4/2020 16:51	206.6	203.9	200.4	2518	65
12/4/2020 16:51	206.6	203.7	200.2	2518	64.92
12/4/2020 16:51	206.6	204.1	201.2	2520	65
12/4/2020 16:51	206.6	204.2	201.1	2520	65
12/4/2020 16:51	206.6	203.7	199.9	2520	65
12/4/2020 16:51	206.6	204.4	200.2	2520	65
12/4/2020 16:51	206.6	204	200.5	2518	64.96
12/4/2020 16:51	206.6	204.3	200.5	2521	65.09
12/4/2020 16:51	206.5	204.3	200.4	2521	64.99
12/4/2020 16:51	206.6	204.4	200.7	2524	65.04
12/4/2020 16:51	206.5	204.6	201.3	2521	65
12/4/2020 16:51	206.6	204	200.3	2519	65.04

12/4/2020 16:51	206.6	204.2	200.1	2519	64.88
12/4/2020 16:51	206.5	203.9	200.6	2517	64.92
12/4/2020 16:51	206.5	203.7	200.6	2520	64.96
12/4/2020 16:51	206.5	204.6	200.4	2517	64.99
12/4/2020 16:51	206.6	203.8	200.3	2513	64.88
12/4/2020 16:51	206.6	204.6	201.3	2517	65.04
12/4/2020 16:51	206.5	203.9	200.8	2525	65
12/4/2020 16:51	206.6	203.7	200.1	2521	65.04
12/4/2020 16:51	206.6	204.4	200.2	2525	65.09
12/4/2020 16:51	206.6	203.9	200.4	2524	64.84
12/4/2020 16:51	206.5	203.9	200.5	2522	64.92
12/4/2020 16:51	206.6	204.2	200.6	2519	65.12
12/4/2020 16:51	206.6	203.7	200.3	2520	64.96
12/4/2020 16:51	206.6	204.4	201.1	2517	65.08
12/4/2020 16:51	206.6	203.8	201	2518	64.95
12/4/2020 16:51	206.6	204.6	200	2520	65.08
12/4/2020 16:51	206.6	204.2	200.2	2519	64.96
12/4/2020 16:51	206.5	203.9	200.7	2516	65
12/4/2020 16:51	206.6	204.3	200.6	2518	65.04
12/4/2020 16:51	206.6	203.6	200.6	2518	64.92
12/4/2020 16:51	206.6	204.4	200.6	2518	65
12/4/2020 16:51	206.5	204.4	201.3	2522	64.96
12/4/2020 16:51	206.6	203.7	200.7	2527	65.09
12/4/2020 16:51	206.6	204.2	200.1	2525	65.04
12/4/2020 16:51	206.6	203.8	200.4	2525	64.96
12/4/2020 16:51	206.6	204.1	200.6	2531	65.04
12/4/2020 16:51	206.6	204.7	200.6	2528	65.04
12/4/2020 16:51	206.6	203.7	200.4	2530	64.92
12/4/2020 16:51	206.7	204.3	200.8	2529	65.04
12/4/2020 16:51	206.6	203.9	201.3	2527	65.04
12/4/2020 16:51	206.6	203.9	200.7	2527	65.04
12/4/2020 16:51	206.6	204.4	200.1	2528	64.96
12/4/2020 16:51	206.7	203.7	200.4	2527	65.12
12/4/2020 16:51	206.6	204.3	200.7	2527	64.99
12/4/2020 16:51	206.7	204.1	200.5	2528	64.92
12/4/2020 16:51	206.6	204.3	200.7	2530	65.12
12/4/2020 16:51	206.7	204.3	200.4	2532	64.92
12/4/2020 16:51	206.7	203.6	200.8	2532	65.12
12/4/2020 16:51	206.6	204.3	201.5	2530	64.92
12/4/2020 16:51	206.6	204.1	201.1	2529	64.92
12/4/2020 16:51	206.7	203.9	200.6	2529	65.04
12/4/2020 16:51	206.6	204.2	200.3	2528	64.96
12/4/2020 16:51	206.7	203.6	200.1	2531	65
12/4/2020 16:51	206.7	204.5	200.6	2530	64.96
12/4/2020 16:51	206.6	204.1	200.7	2526	65.04
12/4/2020 16:51	206.7	204	200.7	2529	65
12/4/2020 16:52	206.7	203.9	200.8	2528	64.96

12/4/2020 16:52	206.7	203.7	200.4	2529	64.96
12/4/2020 16:52	206.7	204.2	200.4	2531	64.96
12/4/2020 16:52	206.6	204.2	200.6	2533	64.92
12/4/2020 16:52	206.7	204	201.1	2532	64.92
12/4/2020 16:52	206.7	204.5	201.6	2530	65.12
12/4/2020 16:52	206.7	204.1	201.3	2527	64.93
12/4/2020 16:52	206.7	204.3	200.8	2527	64.92
12/4/2020 16:52	206.7	204.2	200.4	2530	64.88
12/4/2020 16:52	206.7	204.6	200.2	2532	65.04
12/4/2020 16:52	206.7	203.8	200.3	2527	64.92
12/4/2020 16:52	206.7	204	200.2	2531	64.96
12/4/2020 16:52	206.7	204.4	200.5	2529	65
12/4/2020 16:52	206.7	204.1	200.8	2529	64.95
12/4/2020 16:52	206.7	203.7	200.6	2527	64.93
12/4/2020 16:52	206.7	203.6	200.8	2525	64.93
12/4/2020 16:52	206.7	204.1	200.4	2525	64.92
12/4/2020 16:52	206.7	204.2	200.4	2527	64.88
12/4/2020 16:52	206.7	203.9	201.2	2525	64.92
12/4/2020 16:52	206.7	203.7	201.5	2530	65.09
12/4/2020 16:52	206.7	203.9	201.2	2529	64.92
12/4/2020 16:52	206.7	203.9	200.4	2527	65.04
12/4/2020 16:52	206.7	204.3	200.2	2529	64.92
12/4/2020 16:52	206.7	203.9	200.7	2523	65.04
12/4/2020 16:52	206.7	204.4	200.4	2524	64.92
12/4/2020 16:52	206.7	204.4	200.6	2527	65
12/4/2020 16:52	206.7	203.7	200.8	2527	64.96
12/4/2020 16:52	206.7	204.3	200.4	2528	64.96
12/4/2020 16:52	206.7	204.4	201.1	2529	65
12/4/2020 16:52	206.7	204.6	201.1	2532	65.04
12/4/2020 16:52	206.7	204.1	200.1	2534	64.96
12/4/2020 16:52	206.7	203.8	200.4	2535	65.04
12/4/2020 16:52	206.7	204	200.8	2534	65.04
12/4/2020 16:52	206.7	203.7	200.7	2530	65.08
12/4/2020 16:52	206.7	204.3	200.4	2529	65.04
12/4/2020 16:52	206.7	204.5	201.1	2534	64.99
12/4/2020 16:52	206.7	204	201.1	2531	65.08
12/4/2020 16:52	206.7	204.4	200.2	2531	64.88
12/4/2020 16:52	206.7	203.5	200.4	2527	64.92
12/4/2020 16:52	206.7	204.3	200.8	2531	65.04
12/4/2020 16:52	206.7	204.5	200.6	2527	64.99
12/4/2020 16:52	206.8	203.9	200.2	2526	64.92
12/4/2020 16:52	206.8	204.3	201.3	2529	65.01
12/4/2020 16:52	206.8	204.4	201.2	2525	65.04
12/4/2020 16:52	206.7	204.2	200.4	2529	64.92
12/4/2020 16:52	206.8	204.4	200.4	2529	64.92
12/4/2020 16:52	206.8	204.1	200.6	2528	64.99
12/4/2020 16:52	206.7	204.2	200.8	2525	64.92

12/4/2020 16:52	206.7	203.7	200.4	2533	64.92
12/4/2020 16:52	206.7	204.2	200.4	2537	65.04
12/4/2020 16:52	206.9	204.4	200.6	2532	64.96
12/4/2020 16:52	206.8	204	201.5	2527	65
12/4/2020 16:52	206.8	204.3	201.3	2529	65.05
12/4/2020 16:52	206.8	203.6	200.3	2525	64.92
12/4/2020 16:52	206.9	203.7	200.3	2527	64.96
12/4/2020 16:52	206.9	204.3	200.6	2528	65
12/4/2020 16:52	206.9	203.7	200.9	2530	65.04
12/4/2020 16:52	206.9	204	200.7	2528	65
12/4/2020 16:52	206.9	203.8	200.9	2529	64.96
12/4/2020 16:52	207	204.1	200.8	2527	65.04
12/4/2020 16:53	206.9	204.3	200.9	2524	65.04
12/4/2020 16:53	206.9	203.9	200.6	2527	64.92
12/4/2020 16:53	206.9	204.5	200.5	2524	65.08
12/4/2020 16:53	206.9	204.4	200.5	2524	65.04
12/4/2020 16:53	206.9	203.8	200.6	2524	64.92
12/4/2020 16:53	206.9	204.1	200.6	2527	64.89
12/4/2020 16:53	206.9	204.2	200.6	2531	64.88
12/4/2020 16:53	207	204.5	200.4	2532	64.92
12/4/2020 16:53	206.9	204.1	200.5	2529	64.93
12/4/2020 16:53	207	203.6	200.6	2530	65.04
12/4/2020 16:53	206.9	204.2	200.9	2529	65.08
12/4/2020 16:53	206.9	203.9	200.9	2528	65.03
12/4/2020 16:53	207	203.9	200.8	2531	64.96
12/4/2020 16:53	206.9	204.3	200.8	2527	64.99
12/4/2020 16:53	207	203.9	200.8	2526	64.96
12/4/2020 16:53	206.9	204.2	200.6	2531	64.96
12/4/2020 16:53	207	204	200.6	2526	64.92
12/4/2020 16:53	207	204.1	200.5	2529	65.04
12/4/2020 16:53	207	204.6	200.3	2529	64.96
12/4/2020 16:53	207	204.3	200.9	2528	64.96
12/4/2020 16:53	207.1	204.2	201.3	2528	64.92
12/4/2020 16:53	206.9	203.8	201.8	2530	64.92
12/4/2020 16:53	207	203.9	201.3	2525	65
12/4/2020 16:53	207	204.3	200.8	2529	64.92
12/4/2020 16:53	207	203.6	200.5	2526	65
12/4/2020 16:53	207	204.4	200.6	2526	65
12/4/2020 16:53	207	204.2	200.6	2529	64.96
12/4/2020 16:53	207	204.2	200.8	2528	65
12/4/2020 16:53	207.1	204.6	200.8	2525	65
12/4/2020 16:53	207	204.4	200.8	2525	65.04
12/4/2020 16:53	207.1	204.3	200.8	2524	65
12/4/2020 16:53	207.1	204.1	200.6	2527	64.96
12/4/2020 16:53	207.1	203.9	200.9	2534	64.96
12/4/2020 16:53	207.1	204.6	201	2537	65.04
12/4/2020 16:53	207.1	203.9	200.8	2535	64.92

12/4/2020 16:53	207.1	204.2	200.9	2536	64.96
12/4/2020 16:53	207.1	204.3	200.9	2534	64.83
12/4/2020 16:53	207.1	204.4	200.9	2532	65.12
12/4/2020 16:53	207.1	204.2	200.9	2530	65.08
12/4/2020 16:53	207.1	203.9	200.8	2532	64.88
12/4/2020 16:53	207.1	204.2	200.9	2534	64.79
12/4/2020 16:53	207.1	204.2	200.8	2532	64.99
12/4/2020 16:53	207.1	204.3	200.8	2531	65.08
12/4/2020 16:53	207.2	204.4	200.6	2532	64.92
12/4/2020 16:53	207.1	203.9	200.4	2531	65.08
12/4/2020 16:53	207.1	204.4	200.8	2532	65.03
12/4/2020 16:53	207.2	203.8	200.9	2528	65.03
12/4/2020 16:53	207.1	203.9	201.4	2526	64.99
12/4/2020 16:53	207.1	204.5	201.6	2523	64.99
12/4/2020 16:53	207.1	204.1	201.3	2524	64.96
12/4/2020 16:53	207.1	203.9	200.3	2522	64.8
12/4/2020 16:53	207.1	203.8	200.4	2521	65.03
12/4/2020 16:53	207.1	203.8	200.4	2527	64.93
12/4/2020 16:53	207.2	204.4	200.9	2525	65.08
12/4/2020 16:53	207.2	203.9	200.9	2526	64.93
12/4/2020 16:53	207.2	204.4	201	2531	64.99
12/4/2020 16:53	207.1	203.7	200.8	2530	65.04
12/4/2020 16:53	207.1	203.7	200.4	2528	64.88
12/4/2020 16:53	207.1	204.3	200.9	2529	64.99
12/4/2020 16:54	207.2	204.1	201.3	2535	65
12/4/2020 16:54	207.1	204.2	201.6	2535	64.96
12/4/2020 16:54	207.1	204.5	201.3	2537	64.92
12/4/2020 16:54	207.2	204.4	200.4	2539	64.96
12/4/2020 16:54	207.1	204.4	200.4	2538	65
12/4/2020 16:54	207.2	203.7	200.7	2543	64.92
12/4/2020 16:54	207.1	204.4	200.6	2542	65.04
12/4/2020 16:54	207.2	204.4	201	2541	65.09
12/4/2020 16:54	207.2	204.3	200.9	2540	65
12/4/2020 16:54	207.2	204.2	201.1	2543	64.96
12/4/2020 16:54	207.2	203.8	201	2538	64.92
12/4/2020 16:54	207.2	204.3	200.6	2538	65
12/4/2020 16:54	207.2	204.4	200.6	2538	64.92
12/4/2020 16:54	207.2	204.3	201	2533	64.92
12/4/2020 16:54	207.2	204.1	201.2	2533	65.04
12/4/2020 16:54	207.2	204	201.7	2534	65
12/4/2020 16:54	207.2	204.3	201.8	2532	64.96
12/4/2020 16:54	207.1	204.3	201.6	2535	64.88
12/4/2020 16:54	207.2	204.1	201.6	2536	65
12/4/2020 16:54	207.2	204.5	201.6	2535	65.04
12/4/2020 16:54	207.2	203.9	201.7	2534	64.95
12/4/2020 16:54	207.2	203.9	201.8	2536	64.92
12/4/2020 16:54	207.2	203.7	201.8	2539	64.95

12/4/2020 16:54	207.2	204.3	201.8	2535	64.88
12/4/2020 16:54	207.1	204.4	201.6	2536	64.88
12/4/2020 16:54	207.2	203.9	201.3	2534	64.88
12/4/2020 16:54	207.2	204.1	201.2	2532	65.04
12/4/2020 16:54	207.2	204.4	200.9	2534	65.08
12/4/2020 16:54	207.1	203.7	200.7	2533	65.03
12/4/2020 16:54	207.2	204.6	200.8	2534	64.88
12/4/2020 16:54	207.3	203.7	200.7	2533	65.04
12/4/2020 16:54	207.2	204.5	201	2535	64.92
12/4/2020 16:54	207.2	204.3	201.1	2535	64.99
12/4/2020 16:54	207.2	203.7	200.7	2538	64.88
12/4/2020 16:54	207.2	204.2	201	2539	64.92
12/4/2020 16:54	207.2	204	200.5	2540	64.88
12/4/2020 16:54	207.2	204.3	200.6	2540	65.08
12/4/2020 16:54	207.2	203.8	200.4	2539	65.08
12/4/2020 16:54	207.2	203.8	200.9	2539	65.04
12/4/2020 16:54	207.2	204.6	201.8	2537	64.87
12/4/2020 16:54	207.2	203.8	201.5	2535	64.88
12/4/2020 16:54	207.2	204.4	201.2	2535	64.96
12/4/2020 16:54	207.2	204.4	200.8	2529	64.99
12/4/2020 16:54	207.2	203.7	200.6	2531	65.04
12/4/2020 16:54	207.2	204.1	201.1	2533	64.92
12/4/2020 16:54	207.2	203.9	200.8	2535	64.95
12/4/2020 16:54	207.2	203.8	200.7	2535	64.83
12/4/2020 16:54	207.2	204.4	201	2534	64.88
12/4/2020 16:54	207.2	204.4	200.4	2537	64.99
12/4/2020 16:54	207.2	204.4	200.4	2532	64.96
12/4/2020 16:54	207.2	204.1	200.5	2530	64.99
12/4/2020 16:54	207.2	204.1	201.2	2532	64.92
12/4/2020 16:54	207.3	204.6	201.7	2533	64.99
12/4/2020 16:54	207.2	204.3	201.6	2533	64.92
12/4/2020 16:54	207.2	204.4	201.3	2534	64.92
12/4/2020 16:54	207.3	203.7	200.7	2529	64.92
12/4/2020 16:54	207.2	203.9	200.6	2532	65
12/4/2020 16:54	207.2	204.6	200.8	2534	64.92
12/4/2020 16:54	207.2	204.2	200.8	2532	64.88
12/4/2020 16:55	207.2	204.3	200.9	2530	64.96
12/4/2020 16:55	207.3	204.3	201.2	2529	65
12/4/2020 16:55	207.2	203.7	201.1	2527	65.04
12/4/2020 16:55	207.2	204.4	200.9	2531	64.92
12/4/2020 16:55	207.2	203.9	201.3	2525	64.92
12/4/2020 16:55	207.3	204.1	201.1	2523	65
12/4/2020 16:55	207.3	204.3	201.1	2527	65
12/4/2020 16:55	207.3	204.6	201	2529	64.92
12/4/2020 16:55	207.4	204.1	201.1	2532	65
12/4/2020 16:55	207.3	203.7	201.2	2530	64.92
12/4/2020 16:55	207.3	204.3	201.1	2530	65

12/4/2020 16:55	207.2	205.1	201.1	2529	64.88
12/4/2020 16:55	207.2	205.3	201.2	2535	64.96
12/4/2020 16:55	207.3	205.5	201	2531	64.92
12/4/2020 16:55	207.3	205.1	201.1	2535	64.95
12/4/2020 16:55	207.3	204.3	201	2535	64.99
12/4/2020 16:55	207.3	204.3	200.7	2534	64.83
12/4/2020 16:55	207.3	204.8	200.9	2535	65.04
12/4/2020 16:55	207.3	205.3	200.8	2540	65.08
12/4/2020 16:55	207.3	204.9	201.3	2539	64.84
12/4/2020 16:55	207.3	205.5	201.5	2535	65.04
12/4/2020 16:55	207.2	204.8	201.8	2537	64.88
12/4/2020 16:55	207.3	205.1	201.8	2539	64.88
12/4/2020 16:55	207.2	205.3	200.9	2538	64.92
12/4/2020 16:55	207.4	205.3	200.4	2536	65.04
12/4/2020 16:55	207.3	205.1	200.8	2539	65.01
12/4/2020 16:55	207.3	205.4	201	2535	64.84
12/4/2020 16:55	207.4	204.9	201.1	2539	64.92
12/4/2020 16:55	207.4	205.1	201	2541	65
12/4/2020 16:55	207.3	205.3	201	2541	65
12/4/2020 16:55	207.2	205.1	200.8	2537	64.92
12/4/2020 16:55	207.4	205.6	201.3	2532	65.03
12/4/2020 16:55	207.4	205.2	201.9	2533	64.96
12/4/2020 16:55	207.3	205.4	201.7	2533	64.92
12/4/2020 16:55	207.3	205.1	200.7	2531	64.92
12/4/2020 16:55	207.4	204.9	200.6	2536	64.96
12/4/2020 16:55	207.3	205.5	201.2	2539	65
12/4/2020 16:55	207.3	204.7	201.1	2539	65
12/4/2020 16:55	207.4	205.5	201.1	2541	65.08
12/4/2020 16:55	207.3	205.2	200.9	2542	65
12/4/2020 16:55	207.4	204.9	201.7	2538	65
12/4/2020 16:55	207.3	204.9	201.5	2539	65
12/4/2020 16:55	207.4	205	200.6	2541	65
12/4/2020 16:55	207.4	205.3	201	2540	64.92
12/4/2020 16:55	207.4	205.2	201.2	2536	64.92
12/4/2020 16:55	207.4	204.9	201	2535	65
12/4/2020 16:55	207.4	205.6	200.9	2539	65
12/4/2020 16:55	207.3	205.1	201.6	2536	64.96
12/4/2020 16:55	207.4	205.3	201.2	2534	64.96
12/4/2020 16:55	207.4	205.5	200.8	2534	65.04
12/4/2020 16:55	207.4	205.4	201	2535	64.95
12/4/2020 16:55	207.4	204.9	201.2	2536	64.92
12/4/2020 16:55	207.4	204.7	201.2	2539	65
12/4/2020 16:55	207.4	205.3	200.6	2543	65.04
12/4/2020 16:55	207.4	205.5	201.3	2539	64.92
12/4/2020 16:55	207.4	205.1	201.6	2536	64.91
12/4/2020 16:55	207.4	205.6	201.3	2537	65.08
12/4/2020 16:55	207.4	205	200.9	2537	64.95

12/4/2020 16:55	207.4	205.6	200.7	2541	65.03
12/4/2020 16:55	207.4	205.1	200.9	2543	64.88
12/4/2020 16:56	207.4	205.4	201.1	2540	64.88
12/4/2020 16:56	207.4	205.3	201.2	2536	65.04
12/4/2020 16:56	207.4	204.9	201.1	2535	64.92
12/4/2020 16:56	207.4	205.3	200.8	2539	64.93
12/4/2020 16:56	207.4	205.3	201.3	2538	65.07
12/4/2020 16:56	207.4	204.8	201.9	2539	64.88
12/4/2020 16:56	207.4	205.1	201.1	2543	65.08
12/4/2020 16:56	207.4	205.1	200.8	2546	64.88
12/4/2020 16:56	207.4	205.3	201	2546	64.96
12/4/2020 16:56	207.4	205.4	201.1	2547	65.04
12/4/2020 16:56	207.4	205.5	201.1	2543	64.88
12/4/2020 16:56	207.4	205	200.9	2547	64.99
12/4/2020 16:56	207.4	204.8	200.6	2545	64.99
12/4/2020 16:56	207.5	205.1	201.5	2543	64.92
12/4/2020 16:56	207.4	205.3	201.9	2543	64.91
12/4/2020 16:56	207.5	205	201.8	2536	64.92
12/4/2020 16:56	207.4	205.2	200.8	2538	64.88
12/4/2020 16:56	207.5	204.9	200.6	2543	64.88
12/4/2020 16:56	207.5	205.1	201.2	2543	65
12/4/2020 16:56	207.5	205.5	201.2	2543	65.04
12/4/2020 16:56	207.4	205.4	201.4	2545	64.92
12/4/2020 16:56	207.5	205.1	201.1	2544	65
12/4/2020 16:56	207.4	205	201.2	2541	64.96
12/4/2020 16:56	207.5	205.2	201.2	2543	64.92
12/4/2020 16:56	207.4	204.8	200.8	2546	64.87
12/4/2020 16:56	207.5	205.3	201	2546	64.92
12/4/2020 16:56	207.5	205.4	201.1	2543	65.04
12/4/2020 16:56	207.5	204.9	200.8	2543	64.96
12/4/2020 16:56	207.6	205.1	200.8	2543	65
12/4/2020 16:56	207.5	204.8	200.8	2546	65
12/4/2020 16:56	207.5	205.3	200.8	2545	64.96
12/4/2020 16:56	207.5	205.6	201.1	2546	65
12/4/2020 16:56	207.5	205.1	201.2	2545	64.96
12/4/2020 16:56	207.5	205.5	201.3	2544	65
12/4/2020 16:56	207.5	204.8	201.2	2546	64.88
12/4/2020 16:56	207.5	205.3	201.2	2546	64.92
12/4/2020 16:56	207.5	205.5	201.2	2546	64.96
12/4/2020 16:56	207.5	205.1	201.1	2545	64.87
12/4/2020 16:56	207.6	205.1	200.7	2546	64.96
12/4/2020 16:56	207.5	205.5	201.1	2552	64.88
12/4/2020 16:56	207.5	205.2	201.5	2550	64.88
12/4/2020 16:56	207.5	205.5	201.8	2546	64.88
12/4/2020 16:56	207.5	205.1	201.5	2546	65.04
12/4/2020 16:56	207.5	205.1	200.9	2549	64.92
12/4/2020 16:56	207.5	204.9	201.3	2547	65.03

12/4/2020 16:56	207.5	204.9	201.2	2549	65.08
12/4/2020 16:56	207.5	205.4	200.9	2547	65.08
12/4/2020 16:56	207.5	205.1	200.9	2544	64.84
12/4/2020 16:56	207.5	205	201.5	2554	64.88
12/4/2020 16:56	207.6	204.8	201.9	2554	64.92
12/4/2020 16:56	207.6	205.3	201.2	2551	65.08
12/4/2020 16:56	207.6	205.6	201	2547	64.99
12/4/2020 16:56	207.6	205.1	201.3	2543	64.88
12/4/2020 16:56	207.5	205	201.2	2546	65
12/4/2020 16:56	207.6	204.8	201	2545	64.99
12/4/2020 16:56	207.6	204.9	200.9	2543	64.96
12/4/2020 16:56	207.6	205.4	201.8	2543	65.08
12/4/2020 16:56	207.6	204.9	201.9	2542	64.96
12/4/2020 16:56	207.6	205	201.2	2543	64.8
12/4/2020 16:57	207.6	204.8	201.1	2545	65.04
12/4/2020 16:57	207.6	205.3	201.3	2543	64.96
12/4/2020 16:57	207.6	205.5	201.2	2541	65
12/4/2020 16:57	207.6	205.2	200.8	2542	65
12/4/2020 16:57	207.6	205.2	201.1	2538	65.04
12/4/2020 16:57	207.6	205.5	201.8	2537	65
12/4/2020 16:57	207.5	205.3	202.1	2538	64.96
12/4/2020 16:57	207.6	205.5	201.2	2539	64.92
12/4/2020 16:57	207.6	205.1	201	2538	64.96
12/4/2020 16:57	207.6	204.9	201.3	2540	64.96
12/4/2020 16:57	207.6	205.4	201.2	2541	65
12/4/2020 16:57	207.6	205.1	201.1	2539	64.96
12/4/2020 16:57	207.6	205.5	200.9	2541	65
12/4/2020 16:57	207.6	205	201.6	2539	65
12/4/2020 16:57	207.6	204.9	201.9	2539	64.96
12/4/2020 16:57	207.6	205.6	201.5	2544	64.96
12/4/2020 16:57	207.6	204.9	200.8	2546	65
12/4/2020 16:57	207.6	205.4	201.1	2547	64.88
12/4/2020 16:57	207.5	205.1	201.5	2543	64.99
12/4/2020 16:57	207.6	204.8	201	2543	65.04
12/4/2020 16:57	207.6	205.1	201.1	2545	65.08
12/4/2020 16:57	207.6	204.9	200.8	2548	64.88
12/4/2020 16:57	207.6	205.3	201.2	2545	65.08
12/4/2020 16:57	207.6	204.9	202.1	2547	65.12
12/4/2020 16:57	207.6	205.6	201.5	2547	64.84
12/4/2020 16:57	207.6	205	201.1	2550	64.96
12/4/2020 16:57	207.6	204.9	201.2	2551	64.88
12/4/2020 16:57	207.6	205.3	201.2	2547	65.08
12/4/2020 16:57	207.5	205.2	201.3	2543	64.92
12/4/2020 16:57	207.6	205.5	200.6	2546	65.08
12/4/2020 16:57	207.6	205.1	201.2	2547	65.04
12/4/2020 16:57	207.6	204.7	202.3	2547	65
12/4/2020 16:57	207.6	205.6	201.6	2543	65.09

12/4/2020 16:57	207.6	205.1	201.2	2542	64.96
12/4/2020 16:57	207.6	205.5	201.3	2544	64.96
12/4/2020 16:57	207.6	205.5	201.4	2538	65.04
12/4/2020 16:57	207.7	204.8	201.5	2529	64.83
12/4/2020 16:57	207.6	205.5	201.1	2534	65
12/4/2020 16:57	207.6	205	201.1	2536	64.92
12/4/2020 16:57	207.6	205	201.3	2536	65.08
12/4/2020 16:57	207.6	205.5	201.9	2538	64.92
12/4/2020 16:57	207.7	204.9	202.3	2538	64.88
12/4/2020 16:57	207.7	205.2	201.5	2540	65.04
12/4/2020 16:57	207.6	205.3	201.1	2543	65.12
12/4/2020 16:57	207.7	205.1	201.5	2541	65.08
12/4/2020 16:57	207.6	205.5	201.5	2538	64.96
12/4/2020 16:57	207.6	204.9	201.3	2543	65.08
12/4/2020 16:57	207.6	205.2	200.9	2543	65.05
12/4/2020 16:57	207.7	204.9	201.1	2542	64.92
12/4/2020 16:57	207.6	205	201.8	2537	64.88
12/4/2020 16:57	207.6	205.5	202.1	2539	65
12/4/2020 16:57	207.7	205.2	201.6	2540	65
12/4/2020 16:57	207.7	205.6	201	2539	65.04
12/4/2020 16:57	207.8	205.1	201.2	2542	65
12/4/2020 16:57	207.7	204.9	201.6	2540	65
12/4/2020 16:57	207.7	205.5	201.5	2541	65
12/4/2020 16:57	207.8	205.1	201.2	2534	64.96
12/4/2020 16:57	207.6	205.5	200.9	2537	65.04
12/4/2020 16:57	207.7	205.2	200.9	2538	64.92
12/4/2020 16:57	207.7	204.9	201.8	2540	64.92
12/4/2020 16:58	207.8	205.5	202.2	2537	65.01
12/4/2020 16:58	207.7	205.1	201.8	2536	64.92
12/4/2020 16:58	207.7	205.4	201.2	2534	65.04
12/4/2020 16:58	207.7	205.4	201.3	2535	65.04
12/4/2020 16:58	207.7	204.9	201.3	2536	65.03
12/4/2020 16:58	207.8	205.6	201.6	2532	64.92
12/4/2020 16:58	207.8	205.1	201.2	2533	64.96
12/4/2020 16:58	207.8	205.4	201.2	2533	64.93
12/4/2020 16:58	207.7	205.5	201.6	2533	64.83
12/4/2020 16:58	207.7	204.8	202.1	2537	64.92
12/4/2020 16:58	207.7	205.6	201.5	2539	65.12
12/4/2020 16:58	207.7	205.1	201.1	2536	64.92
12/4/2020 16:58	207.8	205.3	201.5	2536	65.08
12/4/2020 16:58	207.7	205.1	201.4	2541	64.96
12/4/2020 16:58	207.8	204.8	201.1	2538	65
12/4/2020 16:58	207.8	204.9	201	2539	64.96
12/4/2020 16:58	207.8	204.9	201.2	2538	65
12/4/2020 16:58	207.7	205	202	2539	65.04
12/4/2020 16:58	207.7	205.4	201.8	2538	64.96
12/4/2020 16:58	207.8	204.8	201.1	2534	65.04

12/4/2020 16:58	207.8	205.5	201.4	2535	65.09
12/4/2020 16:58	207.8	204.9	201.6	2535	64.96
12/4/2020 16:58	207.8	205.1	201.2	2538	65.04
12/4/2020 16:58	207.8	204.9	201	2540	65.08
12/4/2020 16:58	207.8	205.3	200.9	2538	65.04
12/4/2020 16:58	207.9	205.3	202	2535	64.96
12/4/2020 16:58	207.8	205.1	202	2535	64.88
12/4/2020 16:58	207.8	205.5	201.3	2531	65.12
12/4/2020 16:58	207.8	205.5	201.5	2528	64.92
12/4/2020 16:58	207.8	204.6	201.8	2527	65.12
12/4/2020 16:58	207.8	205.4	201.4	2531	64.96
12/4/2020 16:58	207.8	205	201.5	2531	65.08
12/4/2020 16:58	207.8	205.1	201.2	2531	64.96
12/4/2020 16:58	207.8	205.4	201.1	2537	65.12
12/4/2020 16:58	207.8	205.4	201.2	2535	64.96
12/4/2020 16:58	207.8	205.3	202.2	2533	65.04
12/4/2020 16:58	207.8	204.9	202.4	2528	65.04
12/4/2020 16:58	207.8	205.5	201.8	2532	65
12/4/2020 16:58	207.8	205.7	201.2	2538	64.92
12/4/2020 16:58	207.8	205.3	201.4	2537	65
12/4/2020 16:58	207.8	205.2	201.6	2539	65.09
12/4/2020 16:58	207.8	204.9	201.6	2542	65.04
12/4/2020 16:58	207.8	204.9	201.6	2543	65.04
12/4/2020 16:58	207.8	205.6	200.9	2543	65.04
12/4/2020 16:58	207.8	205.2	201.1	2542	65
12/4/2020 16:58	207.8	205.2	201.9	2541	65
12/4/2020 16:58	207.9	204.8	202.5	2546	64.96
12/4/2020 16:58	207.8	204.7	201.8	2544	65.08
12/4/2020 16:58	207.8	204.7	201.3	2544	64.88
12/4/2020 16:58	207.8	205.1	201.7	2540	65.04
12/4/2020 16:58	207.9	205.5	201.5	2541	65.12
12/4/2020 16:58	207.8	205.2	201.2	2539	65.04
12/4/2020 16:58	207.8	204.8	201.1	2539	64.96
12/4/2020 16:58	207.8	205.5	201.6	2544	65.08
12/4/2020 16:58	207.8	204.8	202.4	2543	64.92
12/4/2020 16:58	207.9	205.4	201.7	2543	65
12/4/2020 16:58	207.8	205.2	201.2	2546	64.96
12/4/2020 16:58	207.9	204.8	201.5	2542	65.04
12/4/2020 16:58	207.8	204.8	201.5	2544	64.96
12/4/2020 16:59	207.9	204.9	201.2	2548	65.04
12/4/2020 16:59	207.8	205	201	2554	65.05
12/4/2020 16:59	207.9	204.6	201.4	2553	64.99
12/4/2020 16:59	207.9	204.9	202.2	2553	64.96
12/4/2020 16:59	207.9	205.6	201.9	2550	65.17
12/4/2020 16:59	208	205	201.2	2551	64.96
12/4/2020 16:59	207.9	205.3	201.5	2549	65.12
12/4/2020 16:59	207.9	205.2	201.6	2554	65

12/4/2020 16:59	207.9	204.8	201.5	2553	65
12/4/2020 16:59	208	204.9	201.3	2552	65.08
12/4/2020 16:59	207.9	205	201.1	2548	65.04
12/4/2020 16:59	208	205.4	201.2	2549	65.13
12/4/2020 16:59	207.9	205.4	201.9	2547	64.96
12/4/2020 16:59	207.9	204.9	202.3	2549	65.09
12/4/2020 16:59	208	205.6	202.2	2549	65.05
12/4/2020 16:59	208	204.8	201.5	2546	65.04
12/4/2020 16:59	208	205.2	201.2	2543	65
12/4/2020 16:59	208	204.8	201.5	2546	65.04
12/4/2020 16:59	208	205.3	201.6	2546	65.08
12/4/2020 16:59	208	205.2	201.8	2544	65
12/4/2020 16:59	208	204.9	201.6	2548	65.04
12/4/2020 16:59	208	205.1	201.4	2547	65.12
12/4/2020 16:59	208	205.3	201.3	2544	64.88
12/4/2020 16:59	207.9	204.8	201.3	2552	65.04
12/4/2020 16:59	208	205.6	202	2554	65.12
12/4/2020 16:59	208	205.1	202.6	2547	64.84
12/4/2020 16:59	208	205.1	201.8	2547	64.93
12/4/2020 16:59	208	204.8	201.4	2546	64.96
12/4/2020 16:59	208	205.2	201.5	2552	65
12/4/2020 16:59	208	205.6	201.5	2553	64.96
12/4/2020 16:59	208	205.2	201.8	2553	65
12/4/2020 16:59	208	204.9	201.3	2553	65
12/4/2020 16:59	208	204.9	201.3	2547	64.92
12/4/2020 16:59	208.1	204.8	202.3	2544	65.04
12/4/2020 16:59	207.9	205.5	202.3	2547	65.05
12/4/2020 16:59	208	205.3	201.8	2545	64.96
12/4/2020 16:59	208	205.5	201.1	2544	65.04
12/4/2020 16:59	208	205.1	201.7	2545	64.96
12/4/2020 16:59	208.1	204.7	201.9	2546	64.92
12/4/2020 16:59	208	205.3	201.8	2543	65.08
12/4/2020 16:59	208	205	201.8	2542	65.04
12/4/2020 16:59	208	205.5	201.5	2543	65.04
12/4/2020 16:59	207.9	205.6	201.3	2541	64.96
12/4/2020 16:59	208	205.5	201.3	2536	64.92
12/4/2020 16:59	208	205.5	201.5	2536	65.16
12/4/2020 16:59	208	204.8	201.4	2538	64.88
12/4/2020 16:59	208.1	205.6	201.3	2538	65.04
12/4/2020 16:59	208	205.3	201.7	2540	64.96
12/4/2020 16:59	208.1	204.9	202.2	2545	64.99
12/4/2020 16:59	208	205.2	202.7	2550	65.08
12/4/2020 16:59	208.1	205.3	202	2550	64.88
12/4/2020 16:59	208	205	201.9	2548	64.92
12/4/2020 16:59	208	205.5	201.3	2546	64.92
12/4/2020 16:59	208	204.9	201.3	2546	64.92
12/4/2020 16:59	208	205.3	201.5	2552	64.96

12/4/2020 16:59	208	205	201.6	2549	64.92
12/4/2020 16:59	208	205.1	201.8	2548	64.96
12/4/2020 16:59	208	205.7	201.4	2550	64.96
12/4/2020 16:59	208.1	205.1	201.4	2553	64.92
12/4/2020 17:00	208.1	205.6	201.8	2554	65.04
12/4/2020 17:00	208	204.9	202.3	2552	65
12/4/2020 17:00	208.1	205.4	202.2	2552	65
12/4/2020 17:00	208	205.6	201.3	2547	64.96
12/4/2020 17:00	208.1	204.8	201.3	2547	64.92
12/4/2020 17:00	208.1	205.3	201.8	2547	64.96
12/4/2020 17:00	208	205.2	201.8	2546	64.92
12/4/2020 17:00	208.1	205	201.4	2550	65.04
12/4/2020 17:00	208	205.5	201.4	2549	65.08
12/4/2020 17:00	208	205.3	202	2544	65
12/4/2020 17:00	208	205.5	202.7	2546	65.04
12/4/2020 17:00	208.1	204.7	201.9	2551	64.96
12/4/2020 17:00	208	204.8	201.4	2553	65.04
12/4/2020 17:00	208	205.5	201.9	2549	64.84
12/4/2020 17:00	208.1	205	201.9	2549	64.96
12/4/2020 17:00	208.1	205.5	201.9	2546	64.96
12/4/2020 17:00	208	205.3	201.6	2546	65
12/4/2020 17:00	208	204.8	201.3	2548	65.04
12/4/2020 17:00	208.1	205.3	202.3	2544	65.03
12/4/2020 17:00	208.1	205.1	202.5	2544	65.04
12/4/2020 17:00	208.1	205.5	202	2548	64.96
12/4/2020 17:00	208.1	205.3	201.7	2545	65.04
12/4/2020 17:00	208.1	204.8	201.6	2546	64.88
12/4/2020 17:00	208.1	205.5	201.8	2545	65.04
12/4/2020 17:00	208.1	205.1	202	2546	64.99
12/4/2020 17:00	208.1	205.7	201.9	2548	65
12/4/2020 17:00	208.2	205.2	201.8	2544	64.92
12/4/2020 17:00	208.1	204.8	201.5	2549	65
12/4/2020 17:00	208.2	205.1	201.6	2552	65
12/4/2020 17:00	208.1	204.9	202.3	2549	65
12/4/2020 17:00	208.1	205.3	202.5	2548	64.96
12/4/2020 17:00	208.1	205.6	201.9	2550	64.96
12/4/2020 17:00	208.1	205.4	201.5	2550	64.96
12/4/2020 17:00	208.1	205.1	201.9	2548	64.96
12/4/2020 17:00	208.2	205	202	2552	64.96
12/4/2020 17:00	208.2	205.1	201.7	2552	65.12
12/4/2020 17:00	208.1	205.5	201.3	2547	64.96
12/4/2020 17:00	208.2	204.7	201.7	2549	65.08
12/4/2020 17:00	208.2	205.1	202.5	2548	65.08
12/4/2020 17:00	208.1	205	202.7	2549	64.99
12/4/2020 17:00	208.2	205	201.9	2552	65.04
12/4/2020 17:00	208.2	204.7	201.6	2551	64.92
12/4/2020 17:00	208.2	204.8	201.8	2549	65.04

12/4/2020 17:00	208.2	205.4	201.8	2550	64.88
12/4/2020 17:00	208.2	204.9	201.8	2547	65.03
12/4/2020 17:00	208.2	205.1	201.8	2551	64.96
12/4/2020 17:00	208.2	204.9	201.6	2550	65.04
12/4/2020 17:00	208.3	205.1	201.5	2549	64.96
12/4/2020 17:00	208.2	205.3	201.9	2548	64.96
12/4/2020 17:00	208.2	205.1	202.3	2548	64.87
12/4/2020 17:00	208.2	205.4	202.5	2545	65.04
12/4/2020 17:00	208.2	205.6	202.1	2547	64.92
12/4/2020 17:00	208.2	204.8	201.6	2547	65
12/4/2020 17:00	208.2	205.6	201.6	2552	65
12/4/2020 17:00	208.3	205.1	201.7	2551	64.92
12/4/2020 17:00	208.2	205.1	201.9	2548	65.09
12/4/2020 17:00	208.2	205	201.6	2549	64.96
12/4/2020 17:00	208.2	205	201.6	2549	65.04
12/4/2020 17:00	208.2	205.3	201.3	2550	65
12/4/2020 17:00	208.2	204.9	201.3	2551	64.96
12/4/2020 17:01	208.3	204.9	202	2546	65
12/4/2020 17:01	208.3	205.6	202.8	2545	64.92
12/4/2020 17:01	208.3	204.8	202.1	2549	64.96
12/4/2020 17:01	208.2	205.2	201.8	2549	64.96
12/4/2020 17:01	208.3	205	201.8	2550	64.88
12/4/2020 17:01	208.2	205.6	202.2	2550	65.08
12/4/2020 17:01	208.2	205.6	202	2547	65.04
12/4/2020 17:01	208.3	204.8	201.5	2546	64.92
12/4/2020 17:01	208.3	205.1	201.8	2547	64.96
12/4/2020 17:01	208.2	205.2	202.7	2550	64.99
12/4/2020 17:01	208.2	205.1	202.3	2552	65.08
12/4/2020 17:01	208.3	205.3	201.9	2556	65.04
12/4/2020 17:01	208.2	205	201.6	2558	64.88
12/4/2020 17:01	208.2	205.1	202	2556	64.92
12/4/2020 17:01	208.2	204.9	202	2554	64.92
12/4/2020 17:01	208.2	205.5	201.8	2554	65.04
12/4/2020 17:01	208.2	205.1	201.8	2556	64.83
12/4/2020 17:01	208.2	204.8	201.6	2554	64.99
12/4/2020 17:01	208.3	205.3	202.3	2552	64.96
12/4/2020 17:01	208.3	204.9	202.4	2549	64.96
12/4/2020 17:01	208.2	205.4	201.7	2547	64.96
12/4/2020 17:01	208.2	205.1	201.9	2549	64.96
12/4/2020 17:01	208.2	204.8	202.2	2551	64.92
12/4/2020 17:01	208.3	205.4	201.8	2552	65.04
12/4/2020 17:01	208.3	205.3	201.8	2555	65
12/4/2020 17:01	208.2	205.5	201.5	2556	65.04
12/4/2020 17:01	208.3	205.5	202	2552	64.87
12/4/2020 17:01	208.3	205.1	203	2551	65
12/4/2020 17:01	208.2	205.5	202.3	2553	64.96
12/4/2020 17:01	208.3	205.5	201.6	2553	64.92

12/4/2020 17:01	208.4	205.3	201.6	2552	65
12/4/2020 17:01	208.3	205.5	201.8	2549	65
12/4/2020 17:01	208.2	204.8	202.2	2548	64.96
12/4/2020 17:01	208.2	205.2	202	2549	64.88
12/4/2020 17:01	208.2	205	201.8	2550	64.96
12/4/2020 17:01	208.3	205.5	201.6	2553	65.04
12/4/2020 17:01	208.3	205.6	201.5	2552	64.99
12/4/2020 17:01	208.2	204.9	202.1	2553	65.03
12/4/2020 17:01	208.3	205.5	202.4	2552	65.08
12/4/2020 17:01	208.3	204.9	203	2556	64.92
12/4/2020 17:01	208.3	205.3	202.7	2559	64.92
12/4/2020 17:01	208.3	205.5	202.5	2559	65.04
12/4/2020 17:01	208.3	205.1	202	2555	64.92
12/4/2020 17:01	208.3	205.1	201.9	2560	65.08
12/4/2020 17:01	208.3	205.3	201.6	2558	64.92
12/4/2020 17:01	208.3	204.8	201.8	2560	64.92
12/4/2020 17:01	208.3	205.5	201.7	2557	64.88
12/4/2020 17:01	208.3	205.1	201.5	2558	65.03
12/4/2020 17:01	208.3	205.5	201.5	2560	65.04
12/4/2020 17:01	208.3	204.9	201.6	2558	64.99
12/4/2020 17:01	208.3	205.1	201.6	2556	65.04
12/4/2020 17:01	208.4	205.6	201.9	2558	65.08
12/4/2020 17:01	208.4	205.1	201.8	2553	64.99
12/4/2020 17:01	208.4	205.5	201.8	2549	64.92
12/4/2020 17:01	208.3	205.3	201.8	2547	64.96
12/4/2020 17:01	208.4	205.1	201.6	2554	65.04
12/4/2020 17:01	208.4	205.6	201.9	2555	64.92
12/4/2020 17:01	208.3	205.1	201.7	2554	65.04
12/4/2020 17:01	208.3	205.5	201.9	2549	65
12/4/2020 17:02	208.3	205.2	201.9	2547	64.96
12/4/2020 17:02	208.4	205	201.8	2553	65.04
12/4/2020 17:02	208.4	205.3	202	2555	64.96
12/4/2020 17:02	208.4	205.1	202	2558	64.96
12/4/2020 17:02	208.4	205.5	202	2556	64.92
12/4/2020 17:02	208.4	204.9	202	2556	64.92
12/4/2020 17:02	208.3	204.9	201.8	2558	64.96
12/4/2020 17:02	208.4	205.4	202	2554	64.92
12/4/2020 17:02	208.4	205.1	202.2	2558	64.96
12/4/2020 17:02	208.4	205.1	201.8	2555	65
12/4/2020 17:02	208.4	205	202	2554	64.87
12/4/2020 17:02	208.4	204.8	201.9	2557	64.96
12/4/2020 17:02	208.4	205.5	201.9	2558	65
12/4/2020 17:02	208.3	205	202.2	2558	65
12/4/2020 17:02	208.4	205.1	201.9	2554	65
12/4/2020 17:02	208.4	204.8	202	2551	64.92
12/4/2020 17:02	208.4	204.8	201.9	2551	65.08
12/4/2020 17:02	208.4	205.5	201.9	2551	64.83

12/4/2020 17:02	208.4	205.3	202.2	2547	64.92
12/4/2020 17:02	208.4	204.9	201.9	2550	65.04
12/4/2020 17:02	208.4	205.4	201.9	2554	64.91
12/4/2020 17:02	208.4	204.9	201.9	2556	65.04
12/4/2020 17:02	208.4	205.2	201.7	2563	64.97
12/4/2020 17:02	208.4	204.9	202.3	2563	64.88
12/4/2020 17:02	208.4	204.7	202.7	2560	65.04
12/4/2020 17:02	208.4	205.6	202.9	2556	65.04
12/4/2020 17:02	208.4	204.8	202.7	2556	64.84
12/4/2020 17:02	208.4	205.4	201.6	2558	65.03
12/4/2020 17:02	208.4	205.4	201.5	2562	65.04
12/4/2020 17:02	208.4	205.5	201.9	2558	64.92
12/4/2020 17:02	208.4	205.3	202.2	2552	64.99
12/4/2020 17:02	208.4	204.7	202.3	2554	64.92
12/4/2020 17:02	208.5	205.3	202	2558	65.04
12/4/2020 17:02	208.4	205.3	202.9	2560	64.99
12/4/2020 17:02	208.4	205.5	201.5	2560	65.04
12/4/2020 17:02	208.5	205.1	201.8	2553	65.04
12/4/2020 17:02	208.5	205	202.3	2556	64.92
12/4/2020 17:02	208.4	205.4	201.9	2552	65
12/4/2020 17:02	208.5	205.1	201.9	2556	64.96
12/4/2020 17:02	208.4	205.7	202.7	2552	65
12/4/2020 17:02	208.5	205.1	202.3	2552	64.96
12/4/2020 17:02	208.4	204.9	201.8	2554	65
12/4/2020 17:02	208.4	205.5	202.1	2557	64.92
12/4/2020 17:02	208.4	205.6	202	2561	64.96
12/4/2020 17:02	208.4	205.5	202.1	2561	65
12/4/2020 17:02	208.5	205.1	201.8	2559	64.96
12/4/2020 17:02	208.4	204.8	202.8	2561	64.96
12/4/2020 17:02	208.4	205.1	202.6	2563	64.92
12/4/2020 17:02	208.4	205.2	201.6	2564	64.96
12/4/2020 17:02	208.4	204.8	201.9	2560	65
12/4/2020 17:02	208.4	205.6	202.2	2556	64.92
12/4/2020 17:02	208.5	205	201.9	2557	65
12/4/2020 17:02	208.4	205.2	202.3	2557	65.04
12/4/2020 17:02	208.4	205.5	202.9	2556	64.99
12/4/2020 17:02	208.4	205.5	202	2556	64.92
12/4/2020 17:02	208.4	205.2	201.7	2557	65.04
12/4/2020 17:02	208.4	204.8	201.9	2555	65.04
12/4/2020 17:02	208.4	205.2	202.2	2552	64.83
12/4/2020 17:02	208.4	204.9	202	2552	64.84
12/4/2020 17:02	208.4	205.4	201.9	2557	65.08
12/4/2020 17:03	208.4	205.5	202.2	2556	65.08
12/4/2020 17:03	208.4	205.1	202.9	2557	64.88
12/4/2020 17:03	208.4	205.6	202.3	2558	64.92
12/4/2020 17:03	208.4	205.5	201.8	2556	65
12/4/2020 17:03	208.4	204.9	201.8	2556	64.84

12/4/2020 17:03	208.4	205.4	202	2554	64.84
12/4/2020 17:03	208.4	204.9	202.2	2556	65.12
12/4/2020 17:03	208.4	205.6	202	2556	65
12/4/2020 17:03	208.4	205.2	202.3	2552	64.88
12/4/2020 17:03	208.5	204.9	202.9	2557	65
12/4/2020 17:03	208.4	205.1	202.6	2554	65
12/4/2020 17:03	208.4	205.1	201.5	2552	65.08
12/4/2020 17:03	208.4	205.3	201.7	2552	64.88
12/4/2020 17:03	208.4	205.5	202.2	2549	64.88
12/4/2020 17:03	208.5	205.5	201.9	2551	65.04
12/4/2020 17:03	208.5	205.3	201.8	2552	64.99
12/4/2020 17:03	208.5	204.8	202.2	2550	64.91
12/4/2020 17:03	208.4	205.1	202.6	2550	65.08
12/4/2020 17:03	208.4	205.3	202.7	2552	64.92
12/4/2020 17:03	208.4	205.5	201.8	2552	65.04
12/4/2020 17:03	208.5	205.3	201.9	2552	64.87
12/4/2020 17:03	208.4	205	201.9	2551	64.96
12/4/2020 17:03	208.5	205.1	201.9	2559	65.09
12/4/2020 17:03	208.4	205.5	202.1	2558	65
12/4/2020 17:03	208.5	205.5	202.1	2556	64.96
12/4/2020 17:03	208.5	205.5	202.2	2556	64.96
12/4/2020 17:03	208.5	205.1	202	2555	64.96
12/4/2020 17:03	208.5	205.6	201.6	2553	64.96
12/4/2020 17:03	208.5	205.4	201.7	2550	64.96
12/4/2020 17:03	208.4	204.8	202	2553	64.92
12/4/2020 17:03	208.4	205.4	202	2550	64.95
12/4/2020 17:03	208.4	205.1	202	2548	65.04
12/4/2020 17:03	208.5	204.9	202	2553	65.08
12/4/2020 17:03	208.4	205.5	201.6	2555	64.88
12/4/2020 17:03	208.4	205.9	202	2552	65.04
12/4/2020 17:03	208.4	206	202.2	2547	64.92
12/4/2020 17:03	208.5	206.3	202	2550	64.92
12/4/2020 17:03	208.5	205.9	202.3	2549	64.88
12/4/2020 17:03	208.5	206.4	202.3	2547	64.99
12/4/2020 17:03	208.5	206	202.2	2546	65
12/4/2020 17:03	208.5	205.9	202	2547	64.99
12/4/2020 17:03	208.5	205.9	202	2551	64.88
12/4/2020 17:03	208.5	206.2	202.5	2552	64.92
12/4/2020 17:03	208.5	206.3	202.7	2552	64.92
12/4/2020 17:03	208.5	205.9	202.2	2550	65.03
12/4/2020 17:03	208.5	205.9	202.3	2551	65.04
12/4/2020 17:03	208.5	205.9	202.3	2550	65.08
12/4/2020 17:03	208.5	206.1	202.3	2549	65.04
12/4/2020 17:03	208.5	206.2	202	2551	64.92
12/4/2020 17:03	208.5	205.9	201.8	2554	64.96
12/4/2020 17:03	208.5	206.2	202.5	2555	64.88
12/4/2020 17:03	208.5	206.2	202.9	2551	65.04

12/4/2020 17:03	208.5	206	202.8	2547	64.88
12/4/2020 17:03	208.5	206.1	202.2	2546	65
12/4/2020 17:03	208.6	205.9	202.1	2545	64.88
12/4/2020 17:03	208.5	206.1	202.3	2548	64.96
12/4/2020 17:03	208.5	206.5	202.4	2549	64.96
12/4/2020 17:03	208.5	206	202.4	2550	64.92
12/4/2020 17:03	208.5	206.2	202.3	2550	64.92
12/4/2020 17:04	208.5	206	201.9	2549	65
12/4/2020 17:04	208.5	205.8	201.8	2550	64.96
12/4/2020 17:04	208.6	206.2	202	2545	64.96
12/4/2020 17:04	208.5	206.2	202.2	2543	64.87
12/4/2020 17:04	208.6	206.2	202.6	2543	65
12/4/2020 17:04	208.6	205.9	202.7	2543	64.96
12/4/2020 17:04	208.5	206	202.7	2546	65.04
12/4/2020 17:04	208.5	206.2	203	2545	64.96
12/4/2020 17:04	208.5	206	202.9	2545	65
12/4/2020 17:04	208.5	206.2	203.1	2543	64.96
12/4/2020 17:04	208.5	206	202.8	2544	64.92
12/4/2020 17:04	208.6	206.4	202.3	2545	65.04
12/4/2020 17:04	208.6	205.8	201.8	2543	64.96
12/4/2020 17:04	208.5	205.9	201.8	2546	64.88
12/4/2020 17:04	208.6	206.1	202	2547	65.04
12/4/2020 17:04	208.5	205.9	202.4	2545	64.96
12/4/2020 17:04	208.5	205.9	202.2	2543	65
12/4/2020 17:04	208.5	206.3	201.8	2542	64.92
12/4/2020 17:04	208.5	205.8	202.8	2542	64.88
12/4/2020 17:04	208.5	206.2	202.5	2542	64.92
12/4/2020 17:04	208.5	206	201.9	2546	64.99
12/4/2020 17:04	208.6	206	202.2	2547	64.88
12/4/2020 17:04	208.5	206.3	202.5	2546	64.88
12/4/2020 17:04	208.5	205.8	202.2	2544	64.99
12/4/2020 17:04	208.5	206.3	202.3	2543	64.96
12/4/2020 17:04	208.6	206.1	203	2541	64.99
12/4/2020 17:04	208.5	206	202	2543	64.88
12/4/2020 17:04	208.5	206	202.1	2543	65.08
12/4/2020 17:04	208.5	205.8	202.4	2546	64.88
12/4/2020 17:04	208.5	206.3	202.2	2545	65.12
12/4/2020 17:04	208.5	205.9	202.1	2547	64.92
12/4/2020 17:04	208.5	206.3	202.2	2548	65.04
12/4/2020 17:04	208.5	206.3	202.9	2546	64.99
12/4/2020 17:04	208.6	206	202.4	2546	64.99
12/4/2020 17:04	208.5	206	201.9	2549	64.93
12/4/2020 17:04	208.5	205.9	202.5	2551	64.88
12/4/2020 17:04	208.6	206.5	202.3	2556	64.92
12/4/2020 17:04	208.6	206.3	201.9	2556	64.99
12/4/2020 17:04	208.6	205.9	202.2	2554	64.92
12/4/2020 17:04	208.5	206.2	203	2553	65.03

12/4/2020 17:04	208.6	206.2	202.3	2547	65.04
12/4/2020 17:04	208.6	206.3	202.2	2548	65.04
12/4/2020 17:04	208.5	206	202.1	2546	64.83
12/4/2020 17:04	208.6	205.9	202.5	2543	64.88
12/4/2020 17:04	208.5	206.1	202.5	2545	65.08
12/4/2020 17:04	208.5	205.9	202.2	2543	64.88
12/4/2020 17:04	208.6	205.9	203	2548	64.92
12/4/2020 17:04	208.5	206.2	203	2550	64.96
12/4/2020 17:04	208.5	205.9	202.1	2550	64.88
12/4/2020 17:04	208.5	205.9	202.3	2552	64.88
12/4/2020 17:04	208.5	205.9	202.3	2553	64.88
12/4/2020 17:04	208.6	206.1	202.5	2556	64.92
12/4/2020 17:04	208.5	206.4	202.2	2556	64.95
12/4/2020 17:04	208.5	205.8	202.2	2555	65.04
12/4/2020 17:04	208.5	206.3	203.2	2556	64.96
12/4/2020 17:04	208.6	205.9	202.4	2550	64.99
12/4/2020 17:04	208.6	205.8	201.9	2551	65
12/4/2020 17:04	208.6	206.2	202.3	2552	64.99
12/4/2020 17:04	208.6	205.9	202.3	2550	65
12/4/2020 17:05	208.6	206.3	202.4	2554	64.92
12/4/2020 17:05	208.5	205.9	202	2553	64.99
12/4/2020 17:05	208.6	205.8	202.1	2558	65.04
12/4/2020 17:05	208.6	206.2	202.9	2558	64.92
12/4/2020 17:05	208.5	205.9	203	2554	64.96
12/4/2020 17:05	208.5	206.3	202.9	2554	65.04
12/4/2020 17:05	208.6	206.1	202	2554	64.92
12/4/2020 17:05	208.6	205.8	202	2554	64.92
12/4/2020 17:05	208.5	206.1	201.9	2550	64.92
12/4/2020 17:05	208.6	205.8	202.2	2548	64.96
12/4/2020 17:05	208.7	206	202.3	2547	64.96
12/4/2020 17:05	208.6	206.3	202.7	2547	64.87
12/4/2020 17:05	208.6	206.2	202.5	2554	65
12/4/2020 17:05	208.6	206.4	202.5	2556	64.96
12/4/2020 17:05	208.6	206	202.4	2554	64.96
12/4/2020 17:05	208.6	206	202.2	2554	65
12/4/2020 17:05	208.6	206.3	202.5	2555	64.96
12/4/2020 17:05	208.6	205.9	202.5	2559	65
12/4/2020 17:05	208.6	206.3	202.3	2560	64.96
12/4/2020 17:05	208.6	206.2	202.7	2561	65.04
12/4/2020 17:05	208.6	206.1	202.2	2564	64.95
12/4/2020 17:05	208.6	206.2	202.5	2563	64.88
12/4/2020 17:05	208.7	205.8	202.5	2564	64.96
12/4/2020 17:05	208.6	206.3	202.2	2563	64.88
12/4/2020 17:05	208.7	205.9	202.3	2564	64.92
12/4/2020 17:05	208.6	206	202.2	2565	64.96
12/4/2020 17:05	208.6	206.3	202	2559	64.83
12/4/2020 17:05	208.6	205.8	202	2558	64.92

12/4/2020 17:05	208.6	206	201.9	2561	64.96
12/4/2020 17:05	208.7	205.9	202.7	2561	64.88
12/4/2020 17:05	208.6	206.2	203	2560	65.04
12/4/2020 17:05	208.7	206.3	203	2553	65
12/4/2020 17:05	208.6	205.8	202.6	2552	64.99
12/4/2020 17:05	208.7	206.1	202.1	2552	64.99
12/4/2020 17:05	208.6	206.2	202.3	2556	64.84
12/4/2020 17:05	208.7	206.3	202.3	2559	64.92
12/4/2020 17:05	208.7	206	202.4	2558	64.95
12/4/2020 17:05	208.7	205.9	202.6	2560	64.83
12/4/2020 17:05	208.6	206.1	202.2	2556	64.92
12/4/2020 17:05	208.6	205.9	202.3	2558	64.99
12/4/2020 17:05	208.7	206.3	202.2	2558	64.88
12/4/2020 17:05	208.7	206	202.2	2558	65.04
12/4/2020 17:05	208.7	205.9	202.9	2560	65.04
12/4/2020 17:05	208.7	206	203	2563	64.92
12/4/2020 17:05	208.7	205.9	203.4	2565	65.04
12/4/2020 17:05	208.7	206.2	203.4	2563	65.12
12/4/2020 17:05	208.7	206.3	203.4	2559	64.99
12/4/2020 17:05	208.7	206	203.2	2558	65.04
12/4/2020 17:05	208.7	206.2	203.5	2561	64.92
12/4/2020 17:05	208.7	206.1	203.3	2559	64.96
12/4/2020 17:05	208.7	206	203.4	2559	64.88
12/4/2020 17:05	208.7	206.2	203.3	2560	65.04
12/4/2020 17:05	208.7	206	203	2558	65.03
12/4/2020 17:05	208.7	206.3	202.2	2560	65.04
12/4/2020 17:05	208.7	205.9	202	2560	65.04
12/4/2020 17:05	208.7	205.9	202.2	2565	65.08
12/4/2020 17:05	208.7	206.3	202.7	2560	64.92
12/4/2020 17:05	208.7	206.1	202.7	2561	64.96
12/4/2020 17:05	208.7	206	202.4	2563	65.08
12/4/2020 17:05	208.7	206.2	202.2	2559	64.92
12/4/2020 17:06	208.7	205.8	203.1	2555	65
12/4/2020 17:06	208.7	206	202.2	2557	65.04
12/4/2020 17:06	208.7	205.9	202.2	2560	64.96
12/4/2020 17:06	208.8	206.4	202.7	2558	65
12/4/2020 17:06	208.8	206.2	202.6	2553	65.17
12/4/2020 17:06	208.8	206.2	202.7	2554	65
12/4/2020 17:06	208.7	206.2	203.4	2551	65.04
12/4/2020 17:06	208.8	205.8	202.3	2554	64.88
12/4/2020 17:06	208.8	206.3	202.1	2556	64.96
12/4/2020 17:06	208.8	206.2	202.7	2558	65.04
12/4/2020 17:06	208.8	206.3	202.6	2553	64.99
12/4/2020 17:06	208.7	206.1	202.3	2553	65
12/4/2020 17:06	208.8	205.9	203.1	2555	64.84
12/4/2020 17:06	208.7	206	202.9	2552	64.88
12/4/2020 17:06	208.7	206.3	202.3	2554	65.08

12/4/2020 17:06	208.8	206.3	202.3	2559	65.04
12/4/2020 17:06	208.8	206.2	202.7	2556	64.88
12/4/2020 17:06	208.8	206.1	202.5	2556	64.84
12/4/2020 17:06	208.8	206	202.4	2556	65.04
12/4/2020 17:06	208.8	206.3	203.3	2558	65.04
12/4/2020 17:06	208.8	206.2	202.2	2559	65
12/4/2020 17:06	208.7	206.2	202	2560	65.04
12/4/2020 17:06	208.9	205.9	202.7	2553	64.92
12/4/2020 17:06	208.9	206.1	202.5	2552	64.92
12/4/2020 17:06	208.8	206.2	202.2	2554	65
12/4/2020 17:06	208.9	205.9	203.2	2551	64.92
12/4/2020 17:06	208.9	206.2	203.1	2554	65
12/4/2020 17:06	208.8	205.9	202.4	2551	65.04
12/4/2020 17:06	208.8	206	202.4	2556	65
12/4/2020 17:06	208.8	206.4	202.3	2556	65
12/4/2020 17:06	208.8	206	202.6	2554	64.92
12/4/2020 17:06	208.8	206.2	202.3	2558	65.04
12/4/2020 17:06	208.8	206	203.3	2557	65
12/4/2020 17:06	208.9	205.9	202.7	2557	65
12/4/2020 17:06	208.9	206.4	202	2553	65.12
12/4/2020 17:06	208.9	205.8	202.7	2554	64.92
12/4/2020 17:06	208.8	206.2	202.7	2553	65.08
12/4/2020 17:06	208.9	206.2	202	2549	64.92
12/4/2020 17:06	208.9	205.8	203.3	2552	65.04
12/4/2020 17:06	208.9	205.9	203.1	2551	65.04
12/4/2020 17:06	208.9	206	202.2	2549	65
12/4/2020 17:06	208.9	206.1	202.5	2554	64.96
12/4/2020 17:06	208.9	206.2	202.7	2553	64.96
12/4/2020 17:06	208.9	206	202.7	2551	64.92
12/4/2020 17:06	208.9	206.2	202.5	2552	65.01
12/4/2020 17:06	208.9	205.9	203.2	2552	64.96
12/4/2020 17:06	208.9	206.2	202.9	2552	65
12/4/2020 17:06	208.9	206.1	202.1	2554	65.05
12/4/2020 17:06	208.9	205.9	202.6	2553	65.05
12/4/2020 17:06	208.9	206.2	202.7	2551	65.04
12/4/2020 17:06	208.9	206	202.6	2551	65.12
12/4/2020 17:06	208.9	206.3	202.9	2551	64.96
12/4/2020 17:06	209	206	203.4	2550	64.96
12/4/2020 17:06	208.9	206	202.3	2549	64.96
12/4/2020 17:06	208.9	205.9	202.2	2552	65.09
12/4/2020 17:06	208.9	205.9	202.7	2548	64.99
12/4/2020 17:06	208.9	206.2	202.7	2546	64.88
12/4/2020 17:06	208.9	206.2	202.3	2547	65.08
12/4/2020 17:06	208.9	205.8	203.2	2547	64.92
12/4/2020 17:07	208.9	206	203.3	2549	64.92
12/4/2020 17:07	209	206.2	202.3	2544	65.04
12/4/2020 17:07	209	206.1	202.5	2545	64.88

12/4/2020 17:07	208.9	205.9	202.5	2547	64.92
12/4/2020 17:07	208.9	206	202.5	2546	64.88
12/4/2020 17:07	209	206.2	202.5	2549	65.01
12/4/2020 17:07	209	206.2	203.5	2548	65
12/4/2020 17:07	209	206.3	202.9	2548	64.96
12/4/2020 17:07	209	206.5	202.4	2548	65.09
12/4/2020 17:07	209	205.9	202.5	2548	65
12/4/2020 17:07	208.9	206.1	202.7	2552	64.96
12/4/2020 17:07	209	206.1	202.9	2552	65
12/4/2020 17:07	209	206.4	202.5	2552	65
12/4/2020 17:07	209.1	205.9	203.2	2552	64.96
12/4/2020 17:07	209	206	203.2	2549	64.92
12/4/2020 17:07	209.1	206.2	202.2	2549	64.88
12/4/2020 17:07	209	206.2	202.7	2549	64.88
12/4/2020 17:07	209	206.3	202.6	2550	64.96
12/4/2020 17:07	209	206	202.7	2554	65.01
12/4/2020 17:07	209.1	205.8	202.7	2554	64.93
12/4/2020 17:07	209	206.1	203.2	2554	64.88
12/4/2020 17:07	209	205.8	202.7	2561	64.93
12/4/2020 17:07	209.1	206.2	202	2561	65.04
12/4/2020 17:07	209.1	206.2	202.6	2556	64.92
12/4/2020 17:07	209.1	205.8	202.7	2558	65.04
12/4/2020 17:07	209	206.1	202.3	2556	64.97
12/4/2020 17:07	209	206.1	202.3	2555	64.88
12/4/2020 17:07	209.1	206.3	202.7	2551	64.96
12/4/2020 17:07	209.1	206.2	203.6	2555	65.04
12/4/2020 17:07	209.1	205.9	203.1	2553	65
12/4/2020 17:07	209	206	202.2	2555	65
12/4/2020 17:07	209	205.8	202.7	2556	65.05
12/4/2020 17:07	209.1	206.2	202.9	2554	64.92
12/4/2020 17:07	209.1	206.3	202.7	2555	65
12/4/2020 17:07	209	206.2	202.6	2551	64.96
12/4/2020 17:07	209.1	206	202.9	2554	65
12/4/2020 17:07	209	205.9	203.9	2559	65.04
12/4/2020 17:07	209	205.9	203.2	2557	64.88
12/4/2020 17:07	209.1	206.4	202.4	2556	65
12/4/2020 17:07	209.1	206.2	202.5	2552	64.84
12/4/2020 17:07	209.1	206.3	202.7	2552	64.92
12/4/2020 17:07	209.1	206.2	202.7	2556	64.96
12/4/2020 17:07	209.1	205.9	202.8	2549	64.88
12/4/2020 17:07	209.1	205.8	202.6	2548	64.93
12/4/2020 17:07	209.1	205.9	203.6	2552	64.93
12/4/2020 17:07	209.1	206	203.5	2554	64.88
12/4/2020 17:07	209.1	205.8	202.5	2555	65.04
12/4/2020 17:07	209.1	205.9	202.6	2560	64.92
12/4/2020 17:07	209.1	206.3	202.9	2561	64.93
12/4/2020 17:07	209.1	206	202.8	2559	64.92

12/4/2020 17:07	209.1	206.2	202.7	2558	64.84
12/4/2020 17:07	209.1	206.4	203.6	2556	65
12/4/2020 17:07	209.1	206.2	203.1	2555	64.92
12/4/2020 17:07	209.1	205.9	202.2	2556	64.96
12/4/2020 17:07	209	205.9	202.8	2559	65
12/4/2020 17:07	209.1	206.2	202.9	2556	64.92
12/4/2020 17:07	209.1	206.3	202.7	2558	64.96
12/4/2020 17:07	209.1	205.9	202.7	2556	65
12/4/2020 17:07	209.1	206.3	203.8	2550	65.04
12/4/2020 17:07	209.1	205.9	202.7	2550	64.92
12/4/2020 17:08	209.2	206.3	202.6	2552	64.87
12/4/2020 17:08	209.2	206.2	203	2552	65
12/4/2020 17:08	209.1	205.9	202.7	2554	64.92
12/4/2020 17:08	209.2	206.2	202.9	2556	65.04
12/4/2020 17:08	209.1	206	202.8	2557	65
12/4/2020 17:08	209.2	206	203.6	2558	64.99
12/4/2020 17:08	209.2	206.3	203.1	2556	65.04
12/4/2020 17:08	209.1	205.8	202.3	2553	64.88
12/4/2020 17:08	209.2	206.2	202.9	2556	65.12
12/4/2020 17:08	209.2	205.9	202.8	2557	65.08
12/4/2020 17:08	209.1	206	202.6	2555	64.88
12/4/2020 17:08	209.2	205.9	202.7	2556	65.12
12/4/2020 17:08	209.2	206	203.6	2560	65.08
12/4/2020 17:08	209.2	206.2	203	2556	65.04
12/4/2020 17:08	209.1	205.9	202.5	2563	65.05
12/4/2020 17:08	209.2	206	202.6	2562	64.96
12/4/2020 17:08	209.2	206.2	202.9	2559	64.92
12/4/2020 17:08	209.1	205.8	202.5	2556	64.92
12/4/2020 17:08	209.2	206.3	203.6	2560	65.09
12/4/2020 17:08	209.2	206	203.6	2560	65
12/4/2020 17:08	209.3	205.9	202.6	2558	64.92
12/4/2020 17:08	209.2	205.9	202.9	2561	65
12/4/2020 17:08	209.2	205.9	203.2	2562	65
12/4/2020 17:08	209.2	206.3	202.7	2560	64.96
12/4/2020 17:08	209.3	206.1	203.1	2560	64.96
12/4/2020 17:08	209.3	206.2	203.6	2559	64.96
12/4/2020 17:08	209.2	206.4	202.5	2561	65.04
12/4/2020 17:08	209.2	205.9	202.9	2563	64.96
12/4/2020 17:08	209.2	206.1	202.9	2567	65.08
12/4/2020 17:08	209.2	206.3	202.9	2564	64.92
12/4/2020 17:08	209.3	206.3	202.7	2564	64.84
12/4/2020 17:08	209.3	206	203.5	2565	65.08
12/4/2020 17:08	209.2	205.8	203.6	2559	65
12/4/2020 17:08	209.2	206.1	202.7	2558	64.84
12/4/2020 17:08	209.2	205.8	202.9	2561	65.04
12/4/2020 17:08	209.2	206.2	203	2561	64.88
12/4/2020 17:08	209.2	206.4	202.7	2563	65.08

12/4/2020 17:08	209.2	205.9	203.2	2558	64.92
12/4/2020 17:08	209.2	206	203.7	2556	65.08
12/4/2020 17:08	209.3	206.2	202.4	2556	64.96
12/4/2020 17:08	209.2	206.3	202.8	2557	65.04
12/4/2020 17:08	209.2	205.9	203.1	2561	64.92
12/4/2020 17:08	209.2	205.9	202.7	2558	64.96
12/4/2020 17:08	209.3	205.9	203	2559	64.96
12/4/2020 17:08	209.3	206.2	203.6	2563	65
12/4/2020 17:08	209.2	206.2	203.2	2559	64.92
12/4/2020 17:08	209.3	205.9	202.5	2563	65
12/4/2020 17:08	209.3	205.8	203.1	2561	65.05
12/4/2020 17:08	209.3	206	203.2	2560	65.04
12/4/2020 17:08	209.3	206.3	202.6	2560	64.96
12/4/2020 17:08	209.3	206.2	203.7	2561	64.96
12/4/2020 17:08	209.3	206.2	203.6	2561	64.96
12/4/2020 17:08	209.2	205.9	202.3	2561	64.96
12/4/2020 17:08	209.4	206	202.9	2557	64.92
12/4/2020 17:08	209.3	205.8	202.9	2558	64.96
12/4/2020 17:08	209.3	205.9	202.5	2556	64.88
12/4/2020 17:08	209.3	206.3	203.5	2556	64.88
12/4/2020 17:08	209.3	205.8	203.5	2558	64.96
12/4/2020 17:08	209.3	206	202.6	2564	65.08
12/4/2020 17:09	209.3	206.2	202.9	2565	64.84
12/4/2020 17:09	209.3	205.9	202.9	2565	64.88
12/4/2020 17:09	209.3	206.3	202.9	2563	65.08
12/4/2020 17:09	209.3	205.9	202.9	2564	64.88
12/4/2020 17:09	209.3	206	203.6	2563	65.04
12/4/2020 17:09	209.4	205.9	203	2560	65.08
12/4/2020 17:09	209.3	206.2	202.5	2556	64.88
12/4/2020 17:09	209.3	206.3	203.1	2556	65.08
12/4/2020 17:09	209.3	205.9	203.1	2556	65
12/4/2020 17:09	209.4	206.3	202.5	2556	65.04
12/4/2020 17:09	209.3	205.9	203.2	2554	64.96
12/4/2020 17:09	209.4	205.9	203.7	2559	65
12/4/2020 17:09	209.3	206.2	202.5	2561	65.05
12/4/2020 17:09	209.3	206	202.6	2561	64.92
12/4/2020 17:09	209.3	206.2	202.9	2564	65
12/4/2020 17:09	209.4	206.4	203	2567	65.04
12/4/2020 17:09	209.4	206.4	202.8	2564	65
12/4/2020 17:09	209.4	206.3	203.7	2566	65
12/4/2020 17:09	209.3	206	203.4	2568	65.04
12/4/2020 17:09	209.4	206	202.5	2567	64.99
12/4/2020 17:09	209.4	206.1	203	2563	64.99
12/4/2020 17:09	209.3	205.8	203.4	2560	65.04
12/4/2020 17:09	209.4	205.9	203.2	2567	65.16
12/4/2020 17:09	209.4	205.9	203.1	2561	65.04
12/4/2020 17:09	209.4	205.9	204.1	2561	65.08

12/4/2020 17:09	209.4	205.9	203.2	2561	65.08
12/4/2020 17:09	209.4	206.2	202.8	2561	64.88
12/4/2020 17:09	209.4	206.2	203	2564	65.08
12/4/2020 17:09	209.4	205.9	203.1	2565	65.04
12/4/2020 17:09	209.4	206	203	2561	65.08
12/4/2020 17:09	209.4	206	203.5	2563	65.03
12/4/2020 17:09	209.4	206.1	203.7	2563	65.04
12/4/2020 17:09	209.4	206.2	202.6	2563	65.08
12/4/2020 17:09	209.4	205.9	202.7	2565	65
12/4/2020 17:09	209.4	206.2	203.2	2561	64.96
12/4/2020 17:09	209.4	206	202.9	2564	65.08
12/4/2020 17:09	209.4	205.9	202.7	2563	64.96
12/4/2020 17:09	209.4	206.2	203.9	2559	65
12/4/2020 17:09	209.5	205.9	203.5	2556	64.96
12/4/2020 17:09	209.4	206.2	202.7	2561	65
12/4/2020 17:09	209.5	205.8	202.9	2562	65.04
12/4/2020 17:09	209.4	206	203.2	2558	64.96
12/4/2020 17:09	209.4	206.4	203	2561	64.92
12/4/2020 17:09	209.4	205.9	203.4	2560	64.92
12/4/2020 17:09	209.5	206.3	203.9	2560	65.04
12/4/2020 17:09	209.4	205.9	202.5	2564	64.96
12/4/2020 17:09	209.5	206	203	2556	65.04
12/4/2020 17:09	209.5	206.1	203.2	2556	64.92
12/4/2020 17:09	209.4	206.1	203	2560	64.88
12/4/2020 17:09	209.5	206.5	203.2	2560	64.88
12/4/2020 17:09	209.5	206	203.9	2561	64.96
12/4/2020 17:09	209.4	206.2	203.2	2564	64.93
12/4/2020 17:09	209.5	206.2	202.7	2566	65.12
12/4/2020 17:09	209.5	206	203.1	2567	64.88
12/4/2020 17:09	209.5	206	203	2565	65
12/4/2020 17:09	209.5	205.9	202.9	2565	65.04
12/4/2020 17:09	209.5	206.2	202.9	2567	65
12/4/2020 17:09	209.5	206.3	204.1	2565	65.04
12/4/2020 17:09	209.5	205.9	203.4	2567	65.04
12/4/2020 17:10	209.5	206.3	202.7	2564	64.92
12/4/2020 17:10	209.5	205.9	203.2	2563	65.04
12/4/2020 17:10	209.5	206.1	203	2563	65.05
12/4/2020 17:10	209.5	206.4	203	2561	65.09
12/4/2020 17:10	209.5	205.9	203.3	2559	64.99
12/4/2020 17:10	209.5	206	203.8	2557	64.88
12/4/2020 17:10	209.5	206	202.9	2559	65.05
12/4/2020 17:10	209.5	205.8	203	2559	64.84
12/4/2020 17:10	209.6	206.3	203	2557	64.96
12/4/2020 17:10	209.5	205.9	203.3	2558	64.96
12/4/2020 17:10	209.6	206	202.9	2559	64.99
12/4/2020 17:10	209.6	205.8	203.7	2560	65.08
12/4/2020 17:10	209.5	206	203.9	2563	64.96

12/4/2020 17:10	209.5	206.1	202.9	2566	64.84
12/4/2020 17:10	209.6	206	202.7	2568	64.88
12/4/2020 17:10	209.6	205.9	202.9	2570	64.92
12/4/2020 17:10	209.6	205.8	203.3	2569	64.88
12/4/2020 17:10	209.6	205.8	203.3	2571	64.92
12/4/2020 17:10	209.6	206.3	202.9	2570	64.96
12/4/2020 17:10	209.6	206.2	203.1	2570	65.08
12/4/2020 17:10	209.7	205.9	204.1	2565	65.04
12/4/2020 17:10	209.6	206	203.3	2561	64.92
12/4/2020 17:10	209.7	206.1	202.9	2559	65.03
12/4/2020 17:10	209.7	206	203.2	2559	64.96
12/4/2020 17:10	209.7	206	203.2	2558	65.03
12/4/2020 17:10	209.6	206.2	203.4	2558	64.91
12/4/2020 17:10	209.6	206.3	203	2560	64.96
12/4/2020 17:10	209.6	205.9	202.9	2561	65.04
12/4/2020 17:10	209.7	206.3	204	2559	64.96
12/4/2020 17:10	209.6	205.8	203.9	2559	65.08
12/4/2020 17:10	209.6	206.1	203.4	2556	65.04
12/4/2020 17:10	209.7	206.3	203	2555	64.96
12/4/2020 17:10	209.7	205.9	203	2560	64.96
12/4/2020 17:10	209.7	206.1	203.3	2558	64.96
12/4/2020 17:10	209.8	205.9	203.2	2563	64.92
12/4/2020 17:10	209.6	206.3	203.2	2563	64.96
12/4/2020 17:10	209.7	206.4	203.5	2563	65.04
12/4/2020 17:10	209.7	205.9	203.9	2559	64.92
12/4/2020 17:10	209.7	206.2	203.2	2559	64.92
12/4/2020 17:10	209.7	206.7	203	2564	65
12/4/2020 17:10	209.7	207.2	203.2	2564	64.92
12/4/2020 17:10	209.7	207.1	203.6	2560	65.04
12/4/2020 17:10	209.7	207.1	203	2561	65
12/4/2020 17:10	209.6	207.1	203	2560	64.99
12/4/2020 17:10	209.7	207.2	203.7	2558	64.96
12/4/2020 17:10	209.7	207	203.9	2554	64.99
12/4/2020 17:10	209.7	207.1	203	2556	64.92
12/4/2020 17:10	209.7	207.2	202.9	2558	64.95
12/4/2020 17:10	209.7	207.2	203.4	2561	64.96
12/4/2020 17:10	209.7	207.1	203.5	2563	65.01
12/4/2020 17:10	209.7	207.2	203.2	2566	64.99
12/4/2020 17:10	209.7	207	203	2564	65.04
12/4/2020 17:10	209.7	207	202.9	2564	64.88
12/4/2020 17:10	209.7	207.2	203.2	2564	64.84
12/4/2020 17:10	209.7	207.2	203.3	2565	64.96
12/4/2020 17:10	209.7	207.2	203	2560	64.84
12/4/2020 17:10	209.7	207	203.3	2563	65.04
12/4/2020 17:10	209.7	207	203.7	2561	64.83
12/4/2020 17:10	209.6	207.3	203.4	2565	64.88
12/4/2020 17:10	209.7	207	203.7	2564	65.12

12/4/2020 17:11	209.6	207.2	203.6	2564	64.99
12/4/2020 17:11	209.6	207.1	203.6	2567	65.04
12/4/2020 17:11	209.7	207.2	203.7	2565	64.88
12/4/2020 17:11	209.6	207.2	203.5	2563	64.96
12/4/2020 17:11	209.7	207.2	203.8	2559	65.04
12/4/2020 17:11	209.6	207.1	203.9	2563	64.96
12/4/2020 17:11	209.7	207.1	203.7	2564	65.08
12/4/2020 17:11	209.7	207.2	204.1	2566	64.96
12/4/2020 17:11	209.6	207.2	203.9	2567	65
12/4/2020 17:11	209.6	207.2	204.1	2565	65.04
12/4/2020 17:11	209.6	207	204.1	2565	64.92
12/4/2020 17:11	209.6	207	204	2563	65
12/4/2020 17:11	209.6	207	204.1	2561	64.96
12/4/2020 17:11	209.7	207.1	204.1	2563	64.96
12/4/2020 17:11	209.7	207	203.5	2566	64.96
12/4/2020 17:11	209.6	207.2	203.2	2567	65
12/4/2020 17:11	209.6	207.2	202.7	2566	65.05
12/4/2020 17:11	209.7	207	202.9	2565	65.08
12/4/2020 17:11	209.7	207	202.7	2567	64.96
12/4/2020 17:11	209.7	207	203.2	2570	65.08
12/4/2020 17:11	209.7	207.1	203.5	2571	64.99
12/4/2020 17:11	209.7	207	203.5	2567	65.04
12/4/2020 17:11	209.6	207	203.3	2565	64.92
12/4/2020 17:11	209.6	207	203.2	2562	65.08
12/4/2020 17:11	209.7	207.1	202.9	2558	64.95
12/4/2020 17:11	209.6	207.2	203.7	2562	65.08
12/4/2020 17:11	209.7	207	203.9	2564	64.93
12/4/2020 17:11	209.7	207.1	203	2562	64.88
12/4/2020 17:11	209.7	207	203	2563	64.92
12/4/2020 17:11	209.7	207	203.2	2565	64.92
12/4/2020 17:11	209.8	207.1	203.1	2565	64.99
12/4/2020 17:11	209.7	207.1	203.4	2564	64.88
12/4/2020 17:11	209.7	207.2	202.7	2570	64.8
12/4/2020 17:11	209.7	207.2	203.7	2567	65.08
12/4/2020 17:11	209.7	207	203.9	2564	64.8
12/4/2020 17:11	209.7	207	203.1	2560	65.04
12/4/2020 17:11	209.6	207.1	203.1	2561	65.01
12/4/2020 17:11	209.8	207.2	203.1	2560	64.92
12/4/2020 17:11	209.7	207	202.9	2563	64.92
12/4/2020 17:11	209.7	207.3	203	2561	65.05
12/4/2020 17:11	209.7	207.1	204	2562	64.96
12/4/2020 17:11	209.7	207	203.3	2558	65
12/4/2020 17:11	209.7	207.1	203.2	2560	64.96
12/4/2020 17:11	209.8	207.1	203.5	2559	65
12/4/2020 17:11	209.6	207	203.5	2557	65
12/4/2020 17:11	209.7	207.1	203	2561	64.96
12/4/2020 17:11	209.7	207.2	203.6	2564	64.92

12/4/2020 17:11	209.7	207.1	204	2560	64.96
12/4/2020 17:11	209.7	206.9	203.1	2556	64.92
12/4/2020 17:11	209.7	207.5	203.3	2564	65.04
12/4/2020 17:11	209.7	207.2	203.1	2563	64.96
12/4/2020 17:11	209.7	207	203.3	2563	64.96
12/4/2020 17:11	209.7	207.2	203	2561	64.96
12/4/2020 17:11	209.7	207.2	203.6	2560	64.88
12/4/2020 17:11	209.7	207.2	204.4	2559	64.96
12/4/2020 17:11	209.7	207.2	203	2560	65
12/4/2020 17:11	209.7	207	202.9	2561	64.96
12/4/2020 17:11	209.7	207.2	203.5	2556	64.99
12/4/2020 17:11	209.7	206.9	203.2	2553	64.92
12/4/2020 17:12	209.7	207.2	202.9	2556	64.96
12/4/2020 17:12	209.7	207.2	204.2	2558	65.08
12/4/2020 17:12	209.7	207.1	203.7	2558	64.92
12/4/2020 17:12	209.7	207.2	202.9	2555	64.96
12/4/2020 17:12	209.7	207	203	2556	64.83
12/4/2020 17:12	209.7	207.2	203.5	2557	65.08
12/4/2020 17:12	209.7	207	203.4	2559	64.92
12/4/2020 17:12	209.7	207.3	203.3	2560	64.92
12/4/2020 17:12	209.7	207.1	204.1	2561	64.84
12/4/2020 17:12	209.7	207.2	203.6	2558	64.92
12/4/2020 17:12	209.7	206.9	202.9	2560	64.92
12/4/2020 17:12	209.7	207.2	203.3	2556	64.99
12/4/2020 17:12	209.7	207	203.4	2555	64.96
12/4/2020 17:12	209.7	207	203.2	2552	65.04
12/4/2020 17:12	209.7	207.2	203.2	2550	64.96
12/4/2020 17:12	209.7	207.2	203.7	2553	64.99
12/4/2020 17:12	209.7	207.1	204.3	2556	64.92
12/4/2020 17:12	209.7	207	203.3	2559	64.96
12/4/2020 17:12	209.7	207	202.9	2560	64.79
12/4/2020 17:12	209.7	207.2	203.5	2561	64.96
12/4/2020 17:12	209.7	207.2	203.7	2561	65.04
12/4/2020 17:12	209.7	207.1	203.1	2561	64.91
12/4/2020 17:12	209.7	207.2	203.5	2556	65.04
12/4/2020 17:12	209.7	206.9	204.2	2556	64.92
12/4/2020 17:12	209.7	207.1	203.4	2553	65
12/4/2020 17:12	209.8	207.2	203	2554	65
12/4/2020 17:12	209.7	207.2	203.4	2556	65
12/4/2020 17:12	209.8	207.1	203.5	2558	65.04
12/4/2020 17:12	209.8	207	203.3	2556	64.96
12/4/2020 17:12	209.7	207.3	203.9	2554	64.99
12/4/2020 17:12	209.7	207.2	204	2552	65
12/4/2020 17:12	209.8	207.2	202.9	2553	64.99
12/4/2020 17:12	209.8	207.2	203.4	2553	65.04
12/4/2020 17:12	209.8	207.1	203.6	2554	65.04
12/4/2020 17:12	209.7	207	203.4	2551	64.84

12/4/2020 17:12	209.8	207.2	203.2	2550	64.92
12/4/2020 17:12	209.7	207.2	203.8	2552	64.92
12/4/2020 17:12	209.8	207	204.1	2551	64.92
12/4/2020 17:12	209.8	207.1	203.3	2549	64.88
12/4/2020 17:12	209.8	207	203	2550	64.88
12/4/2020 17:12	209.8	207	203.4	2551	64.96
12/4/2020 17:12	209.8	207.2	203.6	2553	64.88
12/4/2020 17:12	209.8	207.1	203	2554	65.03
12/4/2020 17:12	209.8	207.2	203.5	2558	65.12
12/4/2020 17:12	209.8	207.3	204.1	2558	64.99
12/4/2020 17:12	209.8	207	203.8	2559	64.93
12/4/2020 17:12	209.8	207.1	203.4	2559	64.92
12/4/2020 17:12	209.9	207.1	203.3	2556	65.04
12/4/2020 17:12	209.8	207.1	203.7	2556	64.99
12/4/2020 17:12	209.8	207	203.4	2559	64.95
12/4/2020 17:12	209.8	207.2	203.4	2562	64.84
12/4/2020 17:12	209.8	207	204	2563	64.8
12/4/2020 17:12	209.8	207.1	204.4	2564	64.93
12/4/2020 17:12	209.9	206.9	203.3	2562	64.97
12/4/2020 17:12	209.8	207.1	203.1	2563	64.83
12/4/2020 17:12	209.8	207.2	203.6	2559	64.92
12/4/2020 17:12	209.8	207.1	203.9	2560	64.92
12/4/2020 17:12	209.9	207.2	203.4	2560	64.88
12/4/2020 17:12	209.9	207.2	203.2	2558	64.87
12/4/2020 17:12	209.8	207.2	204.2	2558	64.92
12/4/2020 17:13	209.9	207.2	204.3	2560	65
12/4/2020 17:13	209.9	207.2	203.4	2560	64.99
12/4/2020 17:13	209.8	207.1	203.4	2556	65.04
12/4/2020 17:13	209.8	207	203.5	2559	65.12
12/4/2020 17:13	209.8	207.2	203.7	2560	64.88
12/4/2020 17:13	209.8	207	203.5	2560	64.92
12/4/2020 17:13	209.8	207	203.2	2563	64.92
12/4/2020 17:13	209.8	207.2	204.2	2567	65
12/4/2020 17:13	209.8	207	204.4	2570	64.87
12/4/2020 17:13	209.9	207	203.6	2571	65
12/4/2020 17:13	209.9	207	203.4	2572	65.04
12/4/2020 17:13	209.8	207	203.2	2569	65
12/4/2020 17:13	209.8	207.1	203.9	2570	65
12/4/2020 17:13	209.8	207	203.6	2571	65
12/4/2020 17:13	209.9	207	203.4	2568	64.96
12/4/2020 17:13	209.9	207	203.5	2568	64.92
12/4/2020 17:13	209.9	207.3	203.9	2565	64.92
12/4/2020 17:13	209.8	207.2	204.3	2567	65
12/4/2020 17:13	209.8	207	203.5	2567	64.92
12/4/2020 17:13	209.9	207	203	2567	64.96
12/4/2020 17:13	209.8	207.1	203.5	2567	64.96
12/4/2020 17:13	209.8	207	203.6	2562	65.04

12/4/2020 17:13	209.8	207.2	203.5	2561	65
12/4/2020 17:13	209.9	207	203.4	2564	65.08
12/4/2020 17:13	209.9	207.2	203.4	2567	64.84
12/4/2020 17:13	209.8	207	204.4	2571	65.04
12/4/2020 17:13	209.9	207.2	204.2	2574	64.99
12/4/2020 17:13	209.9	207	203.1	2574	65.08
12/4/2020 17:13	209.9	207	203.5	2581	64.91
12/4/2020 17:13	209.8	207.2	203.4	2576	64.92
12/4/2020 17:13	209.9	207.1	203.5	2578	65.12
12/4/2020 17:13	209.9	207.1	203.6	2578	64.99
12/4/2020 17:13	209.9	207.1	203.3	2578	65.04
12/4/2020 17:13	209.8	207	204.2	2581	64.92
12/4/2020 17:13	209.9	207.4	204.2	2576	65.04
12/4/2020 17:13	209.9	207.1	203.4	2576	64.99
12/4/2020 17:13	209.9	207.3	203.3	2576	64.84
12/4/2020 17:13	209.9	207.1	203.6	2578	64.88
12/4/2020 17:13	209.9	207	203.6	2572	64.96
12/4/2020 17:13	209.8	207.1	203.3	2574	65.04
12/4/2020 17:13	209.9	207.2	204.1	2574	65.08
12/4/2020 17:13	209.9	207.2	203.9	2569	64.99
12/4/2020 17:13	209.9	207	203.1	2571	65.04
12/4/2020 17:13	209.8	207.1	203.5	2570	64.83
12/4/2020 17:13	209.9	207.2	203.8	2570	65.04
12/4/2020 17:13	209.9	207	203.3	2572	64.99
12/4/2020 17:13	210	207.2	203.7	2572	64.88
12/4/2020 17:13	209.9	207.2	204.4	2567	65.04
12/4/2020 17:13	209.9	206.9	203.2	2568	64.88
12/4/2020 17:13	210	207.2	203.5	2570	64.96
12/4/2020 17:13	209.9	207	203.5	2575	65
12/4/2020 17:13	209.9	207.1	203.7	2569	64.91
12/4/2020 17:13	209.9	207	203.4	2570	64.96
12/4/2020 17:13	210	207.2	203.7	2576	64.96
12/4/2020 17:13	210	207	204.5	2577	64.96
12/4/2020 17:13	209.9	206.9	203.1	2578	64.96
12/4/2020 17:13	210	207.1	203.5	2581	65
12/4/2020 17:13	210	207.2	204.1	2583	64.96
12/4/2020 17:13	210	207.2	203.7	2580	64.92
12/4/2020 17:14	210	207.1	203.5	2576	64.96
12/4/2020 17:14	209.9	207.1	204.2	2578	65
12/4/2020 17:14	210	207.1	204.3	2574	64.92
12/4/2020 17:14	210	207	203.3	2576	64.91
12/4/2020 17:14	210	207.2	203.4	2579	65
12/4/2020 17:14	209.9	207	203.5	2578	64.96
12/4/2020 17:14	210	207	203.7	2577	65.04
12/4/2020 17:14	209.9	207.1	203.4	2575	65
12/4/2020 17:14	209.9	207.2	204.2	2574	64.87
12/4/2020 17:14	209.9	207.1	204.1	2576	64.92

12/4/2020 17:14	209.9	207.2	203.2	2578	64.96
12/4/2020 17:14	209.9	207.2	203.5	2582	64.96
12/4/2020 17:14	210	207.1	203.6	2578	64.92
12/4/2020 17:14	210	207.1	203.9	2578	64.96
12/4/2020 17:14	210	207.1	203.7	2581	65.04
12/4/2020 17:14	209.9	207.2	204.3	2581	64.99
12/4/2020 17:14	209.9	207.1	203.7	2581	64.88
12/4/2020 17:14	210	207	203.4	2576	65
12/4/2020 17:14	210	207.2	203.7	2575	64.88
12/4/2020 17:14	209.9	207	203.8	2574	64.92
12/4/2020 17:14	210	207.1	203.3	2572	64.99
12/4/2020 17:14	209.9	207.2	204.1	2577	64.96
12/4/2020 17:14	210	207	204.4	2578	64.88
12/4/2020 17:14	209.9	207.1	203.2	2577	64.92
12/4/2020 17:14	210	207.2	203.6	2579	65.12
12/4/2020 17:14	210	207.2	203.6	2581	64.88
12/4/2020 17:14	210	207	203.6	2581	64.92
12/4/2020 17:14	210	206.9	203.6	2579	64.92
12/4/2020 17:14	210	207.3	204.5	2578	64.8
12/4/2020 17:14	210	207.2	203.9	2579	64.92
12/4/2020 17:14	210	207.2	203.3	2578	64.92
12/4/2020 17:14	210	207.1	203.5	2576	65.08
12/4/2020 17:14	210	207	203.6	2573	64.99
12/4/2020 17:14	210	207	203.5	2567	64.83
12/4/2020 17:14	210	207	204.1	2573	64.96
12/4/2020 17:14	210	207.2	204.5	2573	65.03
12/4/2020 17:14	210	207	203.4	2572	64.96
12/4/2020 17:14	210	207	203.3	2572	64.96
12/4/2020 17:14	209.9	207.3	203.9	2573	64.88
12/4/2020 17:14	210	207	203.7	2567	64.99
12/4/2020 17:14	210	206.9	203.5	2565	64.96
12/4/2020 17:14	209.9	207.2	204.2	2567	65.08
12/4/2020 17:14	209.9	207	204.2	2561	64.92
12/4/2020 17:14	210	207	203.2	2561	64.92
12/4/2020 17:14	210	207.1	203.6	2565	65.04
12/4/2020 17:14	210	206.9	203.9	2561	64.96
12/4/2020 17:14	210	207	203.7	2565	64.96
12/4/2020 17:14	210	207	203.6	2568	64.96
12/4/2020 17:14	210	207.2	204.6	2567	64.92
12/4/2020 17:14	210	207.2	203.7	2565	65
12/4/2020 17:14	210	207.1	203.4	2567	65
12/4/2020 17:14	210	207.3	203.8	2571	64.96
12/4/2020 17:14	210	207	203.8	2572	65.04
12/4/2020 17:14	209.9	207.2	203.6	2570	64.96
12/4/2020 17:14	210	207	204.1	2567	64.92
12/4/2020 17:14	210	207	204.3	2567	64.88
12/4/2020 17:14	210	207.1	203.4	2560	64.88

12/4/2020 17:14	210	207.1	203.4	2564	64.96
12/4/2020 17:14	210	207.1	204.4	2565	64.76
12/4/2020 17:15	210	207.1	203.6	2571	65
12/4/2020 17:15	210	207.1	203.9	2570	65.08
12/4/2020 17:15	210	207.2	204.5	2569	64.96
12/4/2020 17:15	209.9	207	203.2	2567	64.96
12/4/2020 17:15	210	207.2	203.4	2563	64.88
12/4/2020 17:15	210	207	203.7	2564	65
12/4/2020 17:15	210	207.1	203.4	2564	65.04
12/4/2020 17:15	210	207.2	204.1	2564	65.04
12/4/2020 17:15	210	207.1	204.4	2566	64.92
12/4/2020 17:15	210	207	203.2	2561	64.88
12/4/2020 17:15	210	207.1	203.6	2560	64.96
12/4/2020 17:15	210	207	203.7	2563	65
12/4/2020 17:15	210	207	203.6	2561	65.08
12/4/2020 17:15	210	206.9	204	2561	65
12/4/2020 17:15	210	207.1	204.4	2563	64.96
12/4/2020 17:15	210	207	203.5	2564	64.96
12/4/2020 17:15	210	207.1	203.4	2561	64.96
12/4/2020 17:15	210	207.2	203.8	2563	65
12/4/2020 17:15	210	207	203.7	2566	65.04
12/4/2020 17:15	210	207	203.8	2567	64.96
12/4/2020 17:15	210	207.1	204.9	2569	64.96
12/4/2020 17:15	210	207	203.9	2570	65.04
12/4/2020 17:15	209.9	207.2	203.5	2565	64.92
12/4/2020 17:15	210	207	203.9	2566	65.04
12/4/2020 17:15	210	207	203.6	2567	64.96
12/4/2020 17:15	210	206.9	203.6	2567	65.04
12/4/2020 17:15	210	206.9	204.4	2561	65.08
12/4/2020 17:15	210	207.2	204.4	2564	65.08
12/4/2020 17:15	210	207	203.6	2566	64.92
12/4/2020 17:15	210	206.9	203.8	2562	64.99
12/4/2020 17:15	210	207.1	203.8	2568	64.84
12/4/2020 17:15	210	207	203.8	2569	64.96
12/4/2020 17:15	210	207.2	203.9	2570	64.92
12/4/2020 17:15	210	207.1	204.6	2572	65.04
12/4/2020 17:15	210	207	203.6	2573	64.96
12/4/2020 17:15	210	207.1	203.6	2571	64.92
12/4/2020 17:15	210	207	203.9	2573	64.96
12/4/2020 17:15	210	207.2	203.6	2577	64.96
12/4/2020 17:15	210	207.1	203.6	2575	65
12/4/2020 17:15	210	207	204.7	2572	65
12/4/2020 17:15	210	207	203.7	2570	64.96
12/4/2020 17:15	210	207	203.4	2569	65
12/4/2020 17:15	210	207.1	204.2	2572	64.96
12/4/2020 17:15	210	207.1	203.8	2574	64.96
12/4/2020 17:15	210	207.1	203.5	2573	65.09

12/4/2020 17:15	210	207.1	204.5	2571	64.96
12/4/2020 17:15	210	207.1	204.3	2569	65
12/4/2020 17:15	210	207.3	203.7	2574	65
12/4/2020 17:15	210	207	203.9	2574	64.96
12/4/2020 17:15	210	207	203.7	2575	64.92
12/4/2020 17:15	210	207.1	203.5	2576	64.88
12/4/2020 17:15	210	207.2	203.9	2575	65.12
12/4/2020 17:15	210	207.3	204.6	2570	64.99
12/4/2020 17:15	210	207.2	203.6	2568	64.92
12/4/2020 17:15	209.9	207	203.8	2570	64.96
12/4/2020 17:15	210	207	204.1	2568	64.92
12/4/2020 17:15	210	207	203.7	2567	64.96
12/4/2020 17:15	210	207	203.6	2572	64.96
12/4/2020 17:15	210	207.1	204.5	2571	64.95
12/4/2020 17:15	210	207.2	203.7	2571	65.04
12/4/2020 17:16	210	207.1	203.5	2569	65
12/4/2020 17:16	209.9	207	203.9	2568	64.88
12/4/2020 17:16	209.9	207.2	203.6	2572	64.92
12/4/2020 17:16	210	206.9	203.8	2574	64.92
12/4/2020 17:16	210	207.2	204.6	2573	64.92
12/4/2020 17:16	210	207.2	203.6	2572	64.88
12/4/2020 17:16	209.9	207.1	203.6	2571	65
12/4/2020 17:16	210	207.1	203.7	2572	64.96
12/4/2020 17:16	210	207.1	204.1	2574	64.99
12/4/2020 17:16	210	207	203.7	2576	64.96
12/4/2020 17:16	210	207.1	204.6	2575	65.04
12/4/2020 17:16	210	207.2	204	2573	64.88
12/4/2020 17:16	210	207	203.5	2573	64.92
12/4/2020 17:16	210	207.1	203.9	2577	65
12/4/2020 17:16	210	207.1	203.7	2575	64.96
12/4/2020 17:16	210	206.9	203.6	2577	65.04
12/4/2020 17:16	210	207.2	204.7	2574	65.04
12/4/2020 17:16	210	207.2	204.2	2575	64.96
12/4/2020 17:16	210	207	203.5	2569	64.96
12/4/2020 17:16	210	207	204.2	2572	65
12/4/2020 17:16	210	207.3	204	2568	64.96
12/4/2020 17:16	210	207	203.7	2564	64.88
12/4/2020 17:16	210	207.2	204.5	2563	64.92
12/4/2020 17:16	210	207.2	204.4	2568	64.96
12/4/2020 17:16	210	207	203.7	2564	64.84
12/4/2020 17:16	210	207	204	2566	64.92
12/4/2020 17:16	210	207.1	204.2	2571	64.93
12/4/2020 17:16	210	207.1	203.9	2571	64.92
12/4/2020 17:16	210	207	204.1	2571	64.92
12/4/2020 17:16	210	207.2	205	2570	64.92
12/4/2020 17:16	210	207.2	203.8	2571	64.92
12/4/2020 17:16	210	207	203.6	2568	65.12

12/4/2020 17:16	210	207.2	204.1	2570	64.92
12/4/2020 17:16	210	207.2	204.1	2573	65.04
12/4/2020 17:16	210	207.1	204	2572	64.99
12/4/2020 17:16	210	207.1	204.8	2571	65.08
12/4/2020 17:16	210	207.1	203.9	2568	65.08
12/4/2020 17:16	210	207.1	203.7	2570	64.96
12/4/2020 17:16	210	207.1	204.1	2567	64.96
12/4/2020 17:16	210	207.1	204.1	2568	65.09
12/4/2020 17:16	210	207.2	203.9	2572	65.04
12/4/2020 17:16	210	207	204.8	2575	65
12/4/2020 17:16	210	207.1	204.3	2576	65
12/4/2020 17:16	210	207	203.7	2576	64.96
12/4/2020 17:16	210	207.1	203.9	2577	65.04
12/4/2020 17:16	210	207	203.9	2578	65
12/4/2020 17:16	210	207	203.7	2575	64.92
12/4/2020 17:16	210	207	203.7	2572	64.96
12/4/2020 17:16	210	207.2	204.9	2568	64.88
12/4/2020 17:16	210	207.3	204.1	2574	64.96
12/4/2020 17:16	210	207.1	203.5	2572	64.88
12/4/2020 17:16	210	207.2	204.1	2570	64.96
12/4/2020 17:16	210	207	203.6	2571	65
12/4/2020 17:16	210	207	204.1	2570	65.04
12/4/2020 17:16	210	207	203.7	2568	65.08
12/4/2020 17:16	210.1	207.1	204.4	2571	64.84
12/4/2020 17:16	210	207.1	204.4	2573	64.93
12/4/2020 17:16	210	207.2	203.6	2571	64.88
12/4/2020 17:16	210	207	203.7	2569	64.88
12/4/2020 17:17	210	207	204.2	2566	64.92
12/4/2020 17:17	210	207.1	203.9	2567	65.04
12/4/2020 17:17	210	207	203.9	2568	64.88
12/4/2020 17:17	210	207.1	204.2	2572	64.96
12/4/2020 17:17	210.1	207.1	204.8	2575	64.99
12/4/2020 17:17	210	207.2	204	2576	64.99
12/4/2020 17:17	210	207	203.6	2580	64.93
12/4/2020 17:17	210	207	203.9	2575	64.88
12/4/2020 17:17	210	207.2	204.1	2569	64.96
12/4/2020 17:17	210	207.1	203.8	2573	64.88
12/4/2020 17:17	210	207	204	2574	64.96
12/4/2020 17:17	210	207.1	204.6	2575	64.96
12/4/2020 17:17	210.1	207.2	204.4	2572	65.08
12/4/2020 17:17	210.1	207	203.8	2576	64.92
12/4/2020 17:17	210	207	203.9	2575	64.99
12/4/2020 17:17	210.1	207	203.6	2575	64.92
12/4/2020 17:17	210	207.2	203.7	2571	65.04
12/4/2020 17:17	210.1	207	203.9	2570	64.91
12/4/2020 17:17	210	207.1	204.9	2570	64.91
12/4/2020 17:17	210	207.2	203.7	2576	65

12/4/2020 17:17	210	207	203.8	2583	64.96
12/4/2020 17:17	210.1	207.1	204.2	2584	65
12/4/2020 17:17	210.1	207.2	203.9	2584	65.04
12/4/2020 17:17	210	207.2	203.7	2583	64.88
12/4/2020 17:17	210.1	207	204.1	2580	64.95
12/4/2020 17:17	210.1	207.1	204.8	2575	64.88
12/4/2020 17:17	210	207.1	204.2	2575	65.04
12/4/2020 17:17	210.1	207.1	203.8	2575	64.88
12/4/2020 17:17	210.1	207	204.1	2576	64.83
12/4/2020 17:17	210.1	207.2	204.1	2578	65.08
12/4/2020 17:17	210.1	207.1	204	2572	64.88
12/4/2020 17:17	210.1	207.2	204.1	2576	65.08
12/4/2020 17:17	210.1	207	204.9	2571	64.88
12/4/2020 17:17	210.1	207	204.5	2575	64.83
12/4/2020 17:17	210.1	207.3	203.9	2576	65.03
12/4/2020 17:17	210.1	207	204	2579	64.88
12/4/2020 17:17	210.2	207.3	204.3	2579	64.88
12/4/2020 17:17	210.2	207.1	204.3	2582	64.88
12/4/2020 17:17	210.2	207	203.9	2576	64.99
12/4/2020 17:17	210.1	207	204.9	2575	64.92
12/4/2020 17:17	210.2	207.2	204.6	2575	64.99
12/4/2020 17:17	210.1	207.2	203.7	2577	64.88
12/4/2020 17:17	210.1	207	204.2	2583	65.04
12/4/2020 17:17	210.1	207	204.2	2581	65.04
12/4/2020 17:17	210.1	207.2	203.7	2585	64.92
12/4/2020 17:17	210.1	207.1	204.5	2585	65.04
12/4/2020 17:17	210.2	207.1	204.8	2585	65.04
12/4/2020 17:17	210.1	207.2	203.9	2584	64.99
12/4/2020 17:17	210.2	207	204.1	2588	64.99
12/4/2020 17:17	210.1	207.2	204.2	2583	64.96
12/4/2020 17:17	210.2	207.1	204.3	2584	64.99
12/4/2020 17:17	210.2	207.2	203.8	2579	64.84
12/4/2020 17:17	210.2	207	204.7	2580	65.03
12/4/2020 17:17	210.1	207.2	204.8	2578	65.04
12/4/2020 17:17	210.2	207.1	203.8	2576	64.99
12/4/2020 17:17	210.2	207.2	204.1	2576	64.99
12/4/2020 17:17	210.2	207.2	204.1	2575	64.92
12/4/2020 17:17	210.2	207.1	204.1	2574	64.99
12/4/2020 17:17	210.2	207	204.1	2576	65.04
12/4/2020 17:17	210.1	207.1	204.9	2581	65
12/4/2020 17:18	210.2	207.2	204.1	2581	64.92
12/4/2020 17:18	210.2	207.1	203.9	2583	65.04
12/4/2020 17:18	210.2	207.2	203.9	2584	64.92
12/4/2020 17:18	210.2	207.4	204.2	2577	64.87
12/4/2020 17:18	210.1	207.2	203.8	2579	64.91
12/4/2020 17:18	210.2	207.1	204.8	2581	64.96
12/4/2020 17:18	210.1	207	204.9	2581	65.04

12/4/2020 17:18	210.2	207.2	203.6	2579	64.96
12/4/2020 17:18	210.2	207	204.1	2581	65
12/4/2020 17:18	210.2	207.1	204.2	2584	64.96
12/4/2020 17:18	210.2	207	204.1	2574	64.87
12/4/2020 17:18	210.2	207	204	2576	64.96
12/4/2020 17:18	210.2	207.1	204.8	2578	64.96
12/4/2020 17:18	210.2	207.2	204.5	2581	65
12/4/2020 17:18	210.2	207.2	203.9	2579	65
12/4/2020 17:18	210.1	207	203.9	2579	64.96
12/4/2020 17:18	210.2	207	204.3	2575	64.96
12/4/2020 17:18	210.2	207.1	203.9	2574	64.88
12/4/2020 17:18	210.2	207.1	203.7	2576	65.01
12/4/2020 17:18	210.2	207	205.1	2581	64.96
12/4/2020 17:18	210.2	207.3	204.3	2582	64.88
12/4/2020 17:18	210.2	207.1	203.9	2583	64.96
12/4/2020 17:18	210.2	207	204.1	2585	64.93
12/4/2020 17:18	210.2	207	204.1	2581	65
12/4/2020 17:18	210.2	207.1	203.9	2582	64.88
12/4/2020 17:18	210.2	207	204.8	2585	65.08
12/4/2020 17:18	210.2	207.2	204.8	2583	64.83
12/4/2020 17:18	210.2	207.1	203.8	2581	64.99
12/4/2020 17:18	210.2	207	203.9	2584	64.93
12/4/2020 17:18	210.2	207	204.2	2586	64.96
12/4/2020 17:18	210.2	207.1	203.9	2583	64.92
12/4/2020 17:18	210.2	207.1	204.2	2578	65.08
12/4/2020 17:18	210.2	207.2	205	2577	64.96
12/4/2020 17:18	210.2	207.1	204.2	2575	65.04
12/4/2020 17:18	210.2	207.1	204	2576	65
12/4/2020 17:18	210.1	207.1	204.2	2579	65
12/4/2020 17:18	210.2	207	204.3	2578	65
12/4/2020 17:18	210.2	207.2	204.2	2576	64.96
12/4/2020 17:18	210.2	207.1	204	2575	65.04
12/4/2020 17:18	210.2	207.2	204.9	2575	64.96
12/4/2020 17:18	210.2	207.2	204.3	2569	64.96
12/4/2020 17:18	210.2	207.1	203.6	2567	65
12/4/2020 17:18	210.3	207	204.2	2570	64.96
12/4/2020 17:18	210.2	207.1	204.4	2571	65.04
12/4/2020 17:18	210.3	207.1	204	2572	65.08
12/4/2020 17:18	210.2	207	204.1	2573	65.04
12/4/2020 17:18	210.2	207.3	204.6	2576	64.96
12/4/2020 17:18	210.2	207.2	204.7	2577	64.92
12/4/2020 17:18	210.2	207.1	203.9	2579	65.08
12/4/2020 17:18	210.2	207	203.9	2575	64.99
12/4/2020 17:18	210.3	207	204.3	2575	65.03
12/4/2020 17:18	210.3	207	204.2	2584	64.97
12/4/2020 17:18	210.2	207.2	204.1	2585	64.99
12/4/2020 17:18	210.2	207.1	204	2582	64.89

12/4/2020 17:18	210.2	207.2	203.7	2582	65.04
12/4/2020 17:18	210.2	207.1	204.3	2581	64.99
12/4/2020 17:18	210.3	207.1	205.3	2578	64.88
12/4/2020 17:18	210.2	207.1	204.7	2578	65.04
12/4/2020 17:18	210.3	207.1	203.6	2581	64.88
12/4/2020 17:19	210.3	207	203.7	2575	64.88
12/4/2020 17:19	210.3	207.1	204.1	2574	65.08
12/4/2020 17:19	210.2	207	204.2	2575	64.96
12/4/2020 17:19	210.3	207	204.3	2578	64.88
12/4/2020 17:19	210.2	207.2	204.3	2575	64.88
12/4/2020 17:19	210.3	207.2	204.1	2580	65
12/4/2020 17:19	210.3	207.2	204.8	2581	64.92
[truncated: 378,731 more chars]
